# Systemic inflammation alters the neuroinflammatory response: a prospective clinical trial in traumatic brain injury

**DOI:** 10.1186/s12974-021-02264-2

**Published:** 2021-09-25

**Authors:** Philipp Lassarén, Caroline Lindblad, Arvid Frostell, Keri L. H. Carpenter, Mathew R. Guilfoyle, Peter J. A. Hutchinson, Adel Helmy, Eric Peter Thelin

**Affiliations:** 1 Department of Clinical Neuroscience, Karolinska Institutet, Stockholm, Sweden; 2 Department of Neurosurgery, Karolinska University Hospital, Stockholm, Sweden; 3 Division of Neurosurgery, Department of Clinical Neurosciences, University of Cambridge, Cambridge, UK; 4 Wolfson Brain Imaging Centre, Department of Clinical Neurosciences, University of Cambridge, Cambridge, UK; 5 Department of Neurology, Karolinska University Hospital, Stockholm, Sweden

**Keywords:** Traumatic brain injury, Inflammation, Cytokines, IL1-ra, Human, Neuroinflammation, Infection

## Abstract

**Background:**

Neuroinflammation following traumatic brain injury (TBI) has been shown to be associated with secondary injury development; however, how systemic inflammatory mediators affect this is not fully understood. The aim of this study was to see how systemic inflammation affects markers of neuroinflammation, if this inflammatory response had a temporal correlation between compartments and how different compartments differ in cytokine composition.

**Methods:**

TBI patients recruited to a previous randomised controlled trial studying the effects of the drug anakinra (Kineret®), a human recombinant interleukin-1 receptor antagonist (rhIL1ra), were used (*n* = 10 treatment arm, *n* = 10 control arm). Cytokine concentrations were measured in arterial and jugular venous samples twice a day, as well as in microdialysis-extracted brain extracellular fluid (ECF) following pooling every 6 h. C-reactive protein level (CRP), white blood cell count (WBC), temperature and confirmed systemic clinical infection were used as systemic markers of inflammation. Principal component analyses, linear mixed-effect models, cross-correlations and multiple factor analyses were used.

**Results:**

Jugular and arterial blood held similar cytokine information content, but brain-ECF was markedly different. No clear arterial to jugular gradient could be seen. No substantial delayed temporal associations between blood and brain compartments were detected. The development of a systemic clinical infection resulted in a significant decrease of IL1-ra, G-CSF, PDGF-ABBB, MIP-1b and RANTES (*p* < 0.05, respectively) in brain-ECF, even if adjusting for injury severity and demographic factors, while an increase in several cytokines could be seen in arterial blood.

**Conclusions:**

Systemic inflammation, and infection in particular, alters cytokine levels with different patterns seen in brain and in blood. Cerebral inflammatory monitoring provides independent information from arterial and jugular samples, which both demonstrate similar information content. These findings could present potential new treatment options in severe TBI patients, but novel prospective trials are warranted to confirm these associations.

**Graphical abstract:**

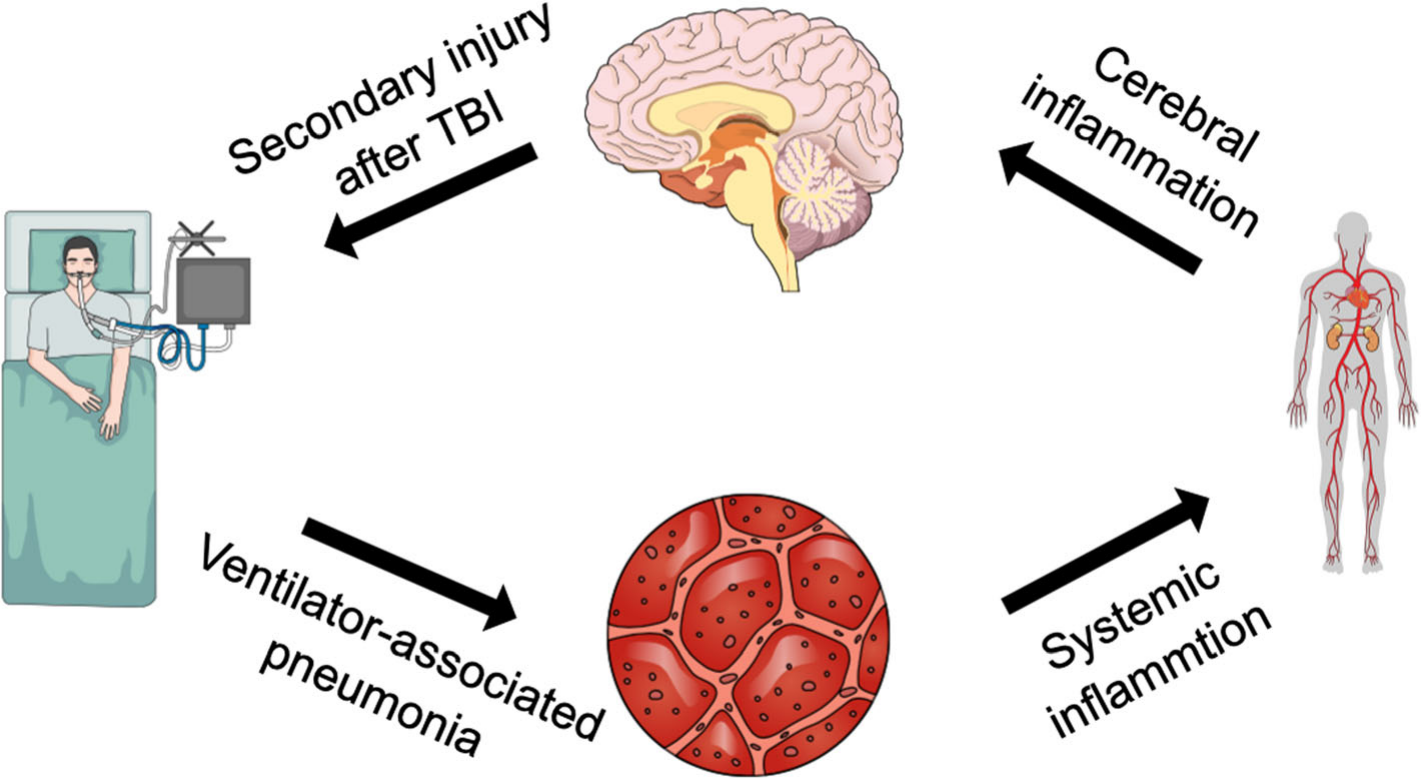

**Supplementary Information:**

The online version contains supplementary material available at 10.1186/s12974-021-02264-2.

## Introduction

Traumatic brain injury (TBI) is a devastating condition, with an increasing morbidity and mortality [1, 2]. Following the primary impact, secondary injury mechanisms play a major role in patient deterioration [3], and of these pathologies neuroinflammation is acknowledged as being one of the main drivers [4].

Patients with TBI have shown to present a peripheral shift in their immune capacity, sometimes referred to as a ‘TBI-induced peripheral immune suppression’, which is also supported by TBI animal models [5–8]. Up to 50% of severe TBI patients have been suggested to suffer from infections during their hospital stay [9]. A recent study in mice exposed to *Streptococcus pneumoniae* and to either TBI or sham surgery showed that brain-injured mice were unable to demonstrate an appropriate immune response due to impaired monocytic function in both acute and chronic stages of TBI which resulted in greater pulmonary bacterial loads [10]. In humans, ventilator-associated pneumonia (VAP) is present in up to 36% of TBI cases and is associated with a longer hospital stay and increased morbidity [11].

Conversely, a systemic-to-brain inflammatory interaction has also been observed. Mice systemically injected with endotoxin lipopolysaccharide (LPS) respond with subsequent neuroinflammation, primarily via an activated potent microglial response and increased concentrations of pro-inflammatory cytokines, specifically interleukin (IL)-1b, IL-6 and tumour necrosis factor alpha (TNFa) [12–16]. An increased glial activation has similarly been observed on positron emission tomography (PET) scans of LPS-injected non-human primates [17]. These systemic inflammatory stimuli have been shown to impair cognitive functions and behavioural changes [15], and are suggested to play an important role in the development in conditions such as long-term neuro-degeneration and psychiatric conditions following inflammatory exposure [18].

In humans, there are several studies describing the cytokine levels in central nervous system (CNS) infections, such as meningitis and encephalitis [19–21]. However, to the best of our knowledge, there are no studies specifically analysing the neuroinflammatory response to systemic inflammatory conditions, i.e. to infections originating from outside the CNS, or to common systemic inflammatory markers such as white blood cell count (WCC), C-reactive protein (CRP) levels and high temperature.

Due to the inaccessibility of the human CNS, there are obvious logistical and ethical caveats concerning longitudinal sampling and monitoring. Commonly, cerebrospinal fluid (CSF) cytokine levels are used as surrogates for measuring cerebral inflammatory activity, including in TBI [22, 23]. In addition, the jugular blood has been suggested as a surrogate locale to measure neuroinflammation due to its proximity to the brain, as compared to arterial blood [24]. Another method for measuring brain CNS cytokine levels is microdialysis (MD) [25], whereby a catheter with a semi-permeable membrane is implanted in brain tissue following conditions such as severe TBI where it enables monitoring of cerebral metabolism, assisting in guiding therapy and interventions [26].

Human recombinant IL-1 receptor antagonist (rhIL1ra), a potent immunomodulatory drug currently used for treating auto-immune conditions such as rheumatoid arthritis, has received a lot of interest in treating CNS conditions [27]. Our group has undertaken a phase II trial of rhIL1ra in TBI, where a panel of 42 cytokines were measured in brain microdialysate for 5 days following injury, noting that the subcutaneously administered drug reached the brain and shifted the cytokine response in the brain extracellular fluid [28]. A similar shift was also seen in the cytokine profile in arterial serum [29], with the CNS cytokine profile shift being time-dependent. Yet, it is still unknown to which extent the inflammatory state of the brain is affected following systemic inflammation by conditions such as severe infections. If it were established that a systemic inflammatory condition affected the brain, it could highlight a potential treatment avenue for improved management of acute CNS conditions, an area where there is a paucity of pharmaceutical options.

We aimed to use safety-data collected in a previously performed prospective trial of rhIL1ra in order to study if systemic cytokine levels, as well as other markers of inflammation and infection, are associated with cerebral cytokine levels (from MD-retrieved extracellular fluid (ECF)). As secondary aims, we wished to study the temporal interaction to see if systemic inflammation precedes brain inflammation, or vice-versa. Additionally, we studied the jugular cytokine levels to see how they compare to arterial and cerebral levels.

## Methods

### Patient population and treatment

This patient population has been described in detail in the previous studies [28, 29]. In short, *n* = 20 TBI patients (*n* = 10 drug, *n* = 10 no drug) with predominantly diffuse injury were included in the randomised clinical trial to study the efficacy of rhIL1ra on cytokine profiles (REC# 06/Q0108/64). Ethical assent was collected from next of kin. Patients randomised to the treatment arm were administered 100 mg rhIL1ra (Anakinra, brand name Kineret®, Sobi, Stockholm, Sweden) once daily subcutaneously for 5 days. Apart from this, all patients received standard neuro-critical care, as described [30], for their TBI.

### Microdialysis and blood sampling

A detailed description can be found in the original publication [28]. In short, brain ECF was acquired using a microdialysis catheter (CMA 71, 100 kDa molecular weight cutoff) perfused with 3.5% (w/v) Human Albumin Solution (Pharmacy Manufacturing Unit, Ipswich Hospital NHS Trust, Ipswich, UK) composed in central nervous system perfusion fluid. Microdialysate collection vials were changed hourly, and samples were pooled from a 6-h epoch to allow sufficient volume to assay.

Blood samples were taken concurrently into EDTA tubes, from arterial and jugular venous catheters at 1 h before and after rhIL1ra administration. The blood was immediately centrifuged at 15 min at 4000×*g*. at 4 °C and the supernatant extracted and stored in − 80 °C until analysis. Plasma samples had sufficient volume for analysis without requiring dilution or pooling.

### Clinical management and parameter definitions

C-reactive protein (CRP) and white blood cell count (WCC) were analysed simultaneously one hour before and one hour after the daily administration of anakinra or no drug using conventional hospital laboratory equipment at Addenbrooke’s Hospital, Cambridge, UK. Core body temperature was measured at the same time using a nasopharyngeal temperature probe. Confirmed clinical infection was defined as an aggregate of clinical, laboratory parameters and cultures, and/or initiation of treatment, and commenced when the condition was defined. VAP was defined as per guidelines laid forth, at the time, by the American Thoracic Society (ATS) and the Infectious Diseases Society of America (IDSA) [31]. No patient suffered from a confirmed infection originating from the CNS.

Acquired clinical and demographic data included age, biological sex, motor score component of the Glasgow Coma Scale (GCSm) [32] on admission, Injury Severity Score (ISS) [33] and computerized tomography (CT) severity scoring assessed as per the Stockholm CT-score [34]. Deterioration on follow-up CT scan was noted.

### Cytokine assay

Samples were analysed using the Milliplex Multi-Analyte Profiling Human Cytokine/Chemokine 42 analyte premixed kit (Millipore, St Charles, MI, USA) using the manufacturer’s instructions as described previously [28]; the 42 cytokines and chemokines assayed are detailed in Additional file 1. All samples were assayed in duplicate wells (25 μL per well) and the mean of the ensuing results was used. The plates were read using a Luminex 200 analyser (Luminex Corporation, Austin, TX, USA) using the STarStation software (Applied Cytometry Systems, Sheffield, UK). Cytokine concentrations were calculated by reference to an eight-point five-parameter logistic standard curve for each cytokine.

### Statistical analysis

Statistical analysis was performed using R, version 3.6.2 and RStudio, version 1.3.1073 for Macintosh. The methods included principal component analysis and multiple factor analysis, performed using the package ‘FactoMineR’ [35]; cross-correlation analysis, performed using ‘ccf’ from base R; and linear mixed-effects modelling, performed using the package ‘nlme’ [36]. Other packages used include ‘messageR’ [37], ‘ggplot2’ [38] and ‘gplots’ [39]. The full reproducible code is available in Additional file 2.

### Principal component analyses and multiple factor analysis of cytokine compartments

In order to investigate whether the cytokines recovered from brain-ECF, and arterial and jugular venous plasma demonstrate different information, we conducted principal component analysis (PCA) and multiple factor analysis (MFA). The analysis was performed using the FactoMineR package in R, applying default configurations of the PCA function, such as scaling the data to unit variance. Any timepoints with incomplete data, i.e. zero values, were omitted. Furthermore, potential cytokine gradients between jugular and arterial cytokines were studied, using the cytokine levels, grouped by patient. MFA is an extension to PCA, by which it is possible to analyse groups of variables [40]. In this case, an MFA was deployed on the three fluid compartments as groups.

### Cross-correlations of inter-compartmental dynamics of cytokines

In order to investigate the direction of cytokine level changes between the different compartments, we performed cross-correlation analyses on all cytokines between brain-ECF and arterial blood. Each time lag was approximately 6 h; the timings of the blood samples were matched to the appropriate time interval during which the brain-ECF samples were drawn. We used the ‘ccf’ function in R to find the cross-correlations and calculated the signed absolute maximum value for each cytokine and patient time series (plotted using the ‘ggplot2’ package). An exclusion algorithm was employed in order to select robust cross-correlation series (Additional file 3).

### Linear mixed-effect models

In order to investigate whether systemic inflammation was associated with cerebral inflammation (i.e. brain-ECF cytokine levels), we used linear mixed-effect models. As fixed effects, we entered the cytokine level in arterial blood, CRP, WCC, temperature, confirmed clinical infection, rhIL1ra treatment and time from TBI (without any interaction terms). For each model, *p* values were extracted; no correction for multiple testing was used as we had an unbiased approach and our scope was not to identify a particular cytokine of importance. Finally, the coefficients of the models were normalized, and visualized as heatmaps, using the ‘massageR’ and ‘gplots’ packages. A similar analysis was performed predicting arterial cytokine levels, but here replacing the parameter ‘arterial cytokine’ with the brain-ECF counterpart.

Blood and brain-ECF samples were not acquired simultaneously (Additional file 4). Since the time variable primarily serves as a predictor of the brain-ECF cytokines and as a parameter in the correlation structure of the linear mixed-effect model, we used the correct brain-ECF sampling times and subsequently matched the blood sampling times with these. As random effects, we had intercepts for patients, as well as by-patient random slope for the effect of time. Similar models were then used to predict brain-ECF and arterial cytokine levels, with clinical and admission parameters (age, sex, GCSm, ISS and Stockholm CT score) as independent variables. The strongest signals, based on the number of significant cytokines in the linear models of both compartments, were then combined with inflammatory markers into a final analysis.

Further details can be found in the supplementary statistical text (Additional file 3).

## Results

### Patient demographics and missing analytes

The patients included in this study have been previously described in detail [28]. In summary, the twenty recruited patients (10 females, 10 males) had predominantly diffuse injury, all with a post-resuscitation GCS of less than 8 (traditionally considered a ‘severe’ TBI). A relatively large variation can be observed between patients in trends of WCC, CRP and temperature during the first 5 days following the TBI (Additional file 1). Apart from a more substantial decrease in CRP in the intervention group compared to the control group, there were no significant differences between the groups in terms of inflammatory markers. Both treatment groups experienced four infections each requiring treatment, a majority of these were VAPs. Five of these were reported as SAEs as per the pre-specified definitions [28]. Median levels of CRP, WCC, temperature as well as admission CT scores and injury characteristics can be seen in Table 1.

**Table 1:** Patient characteristics

Patient ID	White cell count	C-reactive protein	Temperature	Infection type	Severe adverse event	Stockholm CT score	Injury severity score
C01	12.4 (8.7–15.3)	94 (63–114)	37.07 (36.82–37.5)	–	–	1.5	45
C02	1.5 (0.6–3.4)	172 (37–250)	37.3 (35.83–37.88)	–	Yes	0.5	38
C03	14.85 (13.5–20.2)	147 (52–250)	36.92 (35.65–37.93)	VAP	–	1	45
C04	10.4 (8.4–15.7)	182 (103–250)	36.17 (33.07–37.75)	VAP	Yes	3	33
C05	21.5 (16.5–26)	216 (26–250)	36.47 (35.45–38.1)	VAP	–	2.5	38
C06	8.1 (5.7–16.7)	136.5 (45–143)	36.03 (34.72–37.15)	–	–	1.9*	38
C07	9.1 (6.8–18.3)	187 (5–250)	35.42 (35.02–37.72)	–	–	2	29
C08	7.1 (6.4–10.4)	131.5 (107–250)	37.19 (36.63–37.4)	–	–	3	36
C09	9.55 (8.2–10.7)	136.5 (47–194)	36.36 (34.48–37.72)	–	–	0.5	38
C10	4.7 (0.9–13.5)	250 (92–250)	33.21 (32.92–35.42)	IAS	Yes	4	30
I01	7.8 (7.8–9.8)	104.5 (48–186)	36.1 (35.88–36.65)	–	–	3	27
I02	12.35 (7.8–22.1)	74 (18–250)	36.92 (36.32–38.3)	CI	Yes	3	43
I03	5.6 (4.4–8.1)	181 (152–211)	35.37 (34.3–35.67)	–	–	2.4*	50
I04	10.4 (7.2–16.1)	230 (162–250)	38.13 (37.17–39.68)	–	Yes	3	50
I05	14.3 (4.9–23.4)	238 (128–250)	35.48 (34.95–37.97)	VAP	Yes	1.5*	30
I06	11.45 (7.3–12.7)	102 (26–201)	37.28 (36.83–37.72)	VAP	Yes	3.8	27
I07	7.7 (5.8–14.2)	64 (38–150)	34.55 (33.12–37.02)	–	–	2*	45
I08	6.15 (4–8.3)	96.5 (31–204)	36.92 (36.57–37.17)	–	–	2	66
I09	11 (7–15.3)	113 (2–239)	37.47 (36.87–37.62)	VAP	–	1.5*	43
I10	7.15 (4.7–13.4)	76 (17–158)	36.58 (36.08–37.45)	–	–	3	27

The first ten patients are control (C) patients and the other ten patients are intervention (I) patients receiving rhIL1ra treatment. White cell count, C-reactive protein (CRP) and temperature are displayed as median values, with maximum and minimum values in parentheses; their respective units are number of 10^9^ cells per liter, μg/ml and °C. Infection types are ventilator-associated pneumonia (VAP), intraabdominal sepsis (IAS) and chest infection (CI). Stockholm CT score and injury severity score (ISS) are defined as in [34, 41], respectively. CT scores with an asterisk indicate a deterioration on subsequent scan

On average, cytokines measured in arterial and venous jugular plasma, and brain-ECF compartments, had missing rates of 18.5%, 30.9% and 26.3%, respectively (Additional file 1). There were no apparent differences in distribution of missing data between the two treatment groups, compartments or patients, but there was a difference in missing rate (frequency of absence) between different cytokines. This indicates that missingness might not be random, but presumably related to the Luminex assay’s capabilities to measure specific cytokines (such as IL-4, which is known from previous studies [42]). The missingness of IL-4 in microdialysates is unlikely to be due to poor relative recovery, as previous studies in vitro have shown IL-4 to be well-recovered (57%) using the same type of 100 kDa microdialysis catheter used in the present study [25]. Patient C04 had no cytokine measured in jugular blood due to clotting of the catheter.

### Arterial and jugular compartments provide similar information

For each cytokine, a variables factor map was plotted (Additional file 2: Figure 3A code) and visually analysed. Most cytokines exhibit a similar pattern: the axes corresponding to arterial and venous cytokine concentrations are adjacent to each other and near the first principal component axis, while the brain-ECF axis is approximately orthogonal to these and near the second principal component axis. Consequently, variations seen in arterial blood are generally corresponding to variations found in jugular venous blood, indicating that the two samples carry similar information and that this is different from the information carried by brain-ECF concentrations.

**Fig. 1:**
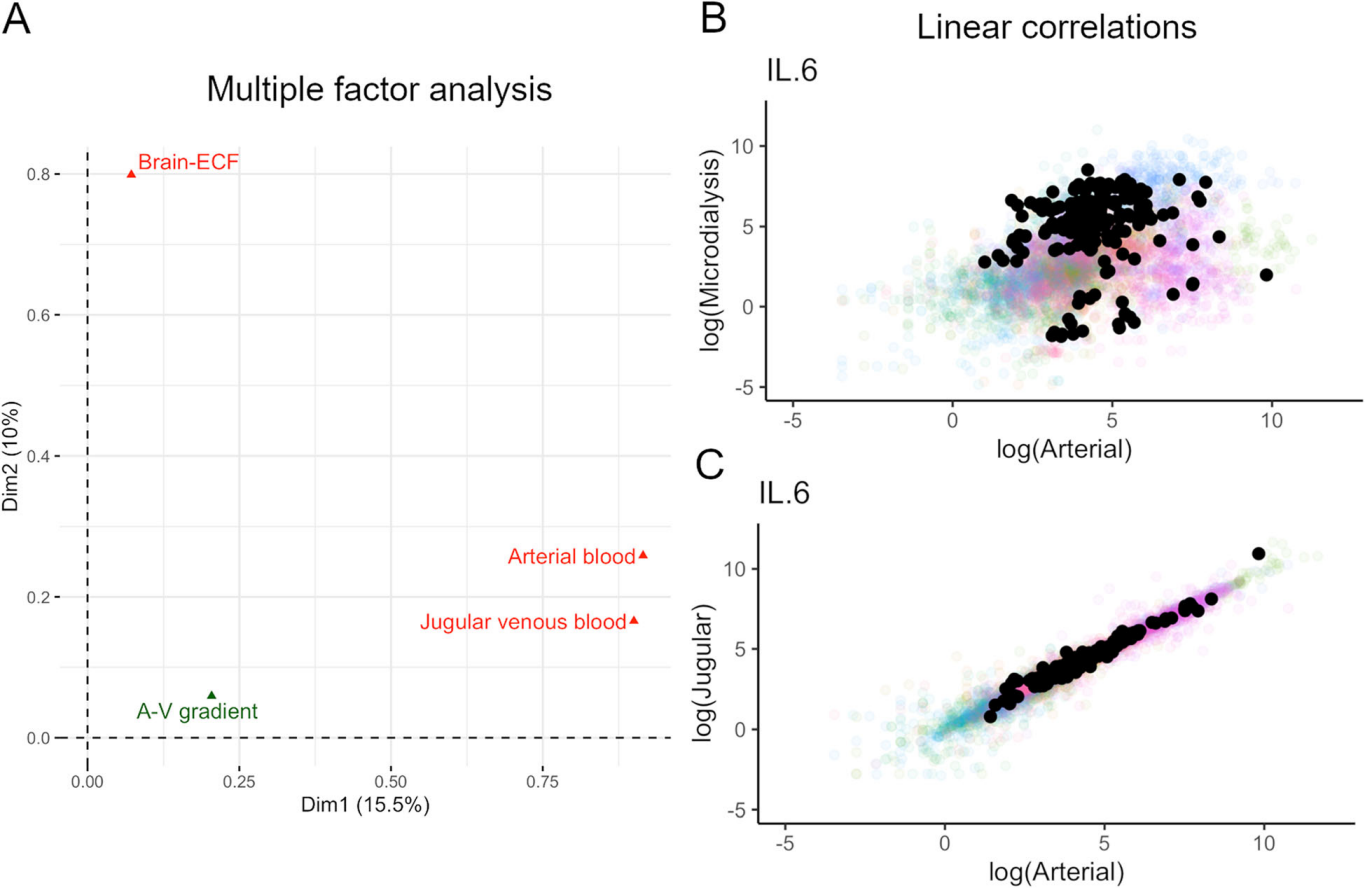
Relationship between brain extracellular fluid (ECF) and blood compartments. **A** Multiple factor analysis of compartments as groups. The arterio-jugular venous (A-V) gradient is plotted as a supplementary variable, while the other variables are active. Displayed on the right, there is an example of the log-log plot of **B** brain ECF vs arterial blood and of **C** jugular venous vs arterial blood concentration of IL-6—the cytokine with least missing data

Figure 1 shows the groups’ representation of the MFA (Fig. 1A), where the dichotomy between the brain-ECF and the blood plasma samples is evident, as well as the similarity of information in arterial and venous blood samples. This includes the arterial-venous gradient which was localized differently from the blood compartments, but more similar to them as compared to the cerebral compartment (Fig. 1A). As shown by linear correlations, the blood and brain compartments did not correlate (Fig. 1B), while there was a strong correlation between the arterial and jugular compartments for all cytokines with significant data (Fig. 1C). Due to these similarities, only arterial blood was used in future analyses due to lower levels of missingness.

The median arterial-jugular (AJ) gradient for each cytokine is shown in Table 2 (and in Additional file 2: Table 2 code). As can be seen, 28 cytokines had a positive AJ gradient, having higher concentrations in arterial blood than in jugular venous blood. Accordingly, 14 cytokines had higher concentrations in the venous compartment. Hence, these results oppose the idea that there is a one-way flow of cytokines from the injured brain to the blood, and that in general, the level of cytokines are in fact higher in arterial blood in NCCU treated TBI patients.

**Table 2:** Median difference between arterial (A) and jugular (J) venous cytokine concentrations

Cytokine	Abbreviation	A–J [pg/mL]
Epidermal growth factor	EGF	1.19
Eotaxin	Eotaxin	− 0.207
Basic fibroblast growth factor	FGF.2	0.641
Fms-related tyrosine kinase 3 ligand	FLT.3.ligand	− 2.053
Fractalkine/CX3CL	Fractalkine	− 0.662
Granulocyte colony-stimulating factor	G.CSF	− 3.413
Granulocyte-monocyte colony stimulating factor	GM.CSF	− 0.388
GRO/CXCL3	GRO	20.593
Interferon alpha-2	IFNa2	0.476
Interferon gamma	IFNg	− 0.172
Interleukin-1 alpha	IL.1a	1.654
Interleukin-1 beta	IL.1b	− 0.055
Interleukin-1 receptor antagonist	IL.1ra	2.157
Interleukin-2	IL.2	0.313
Interleukin-3	IL.3	− 0.004
Interleukin-4	IL.4	0
Interleukin-5	IL.5	0.084
Interleukin-6	IL.6	3.942
Interleukin-7	IL.7	0.139
Interleukin-8	IL.8	1.87
Interleukin-9	IL.9	− 1.634
Interleukin-10	IL.10	0.894
Interleukin-12 subunit beta	IL.12.p40	0
Interleukin-12	IL.12.p70	0.004
Interleukin-13	IL.13	0.232
Interleukin-15	IL.15	0.334
Interleukin-17	IL.17	0.061
Chemokine (C-X-C motif) ligand 10	IP.10	− 1.628
Monocyte chemotactic protein 1	MCP.1	− 0.658
Monocyte chemotactic protein 3	MCP.3	0
Macrophage-derived chemoattractant	MDC	19.1
Macrophage inflammatory protein-1 alpha	MIP.1a	1.333
Macrophage inflammatory protein-1 beta	MIP.1b	2.014
Platelet-derived growth factor AA	PDGF.AA	19.988
Platelet-derived growth factor AB/BB	PDGF.ABBB	201.807
RANTES	RANTES	88.263
Soluble CD40 Ligand	sCD40L	16.129
Soluble interleuking-2 receptor	sIL.2Ra	7.785
Transforming growth factor alpha	TGFa	0.47
Tumour necrosis factor alpha	TNFa	0.7
Tumour necrosis factor beta	TNFb	0.741
Vascular endothelial growth factor	VEGF	2.574

### No temporal trends between compartments could be visualized

**Fig. 2:**
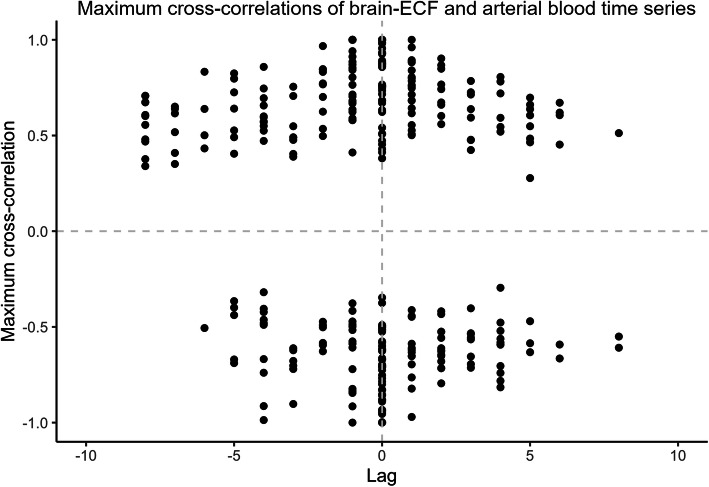
Signed absolute maximum cross-correlations of brain and blood cytokine time series. For every cytokine and patient, the signed absolute maximum of the cross-correlation series of cytokines in extracellular brain fluid and arterial blood was recorded as the *y*-value, and the lag at which this occurred was recorded as the *x*-value. Each lag represents 6 h

In order to investigate the direction of cytokine level changes between the different compartments, we performed cross-correlation analyses on all cytokines between their levels in brain-ECF and in arterial blood. Figure 2 depicts a fairly even distribution of cytokines in the four quadrants of the signed absolute maximum mean cross-correlation plot. The points are distributed such that there is a global symmetry around lag zero for both positively and negatively cross-correlated cytokine and patient series. This indicates that there is no observed global trend in neither directionality in movement between the blood and brain-ECF compartments nor in relative concentration changes between the two compartments.

Although there might not be an average global trend for all 42 cytokines in the 20 patients included in this study, individual cytokines or patients could have distinct patterns. In an attempt to address this hypothesis, we visually examined plots for every cytokine, as well as plots for every patient, annotated with the same parameters. Generally, we find a similar picture when visualizing individual cytokines as seen in Fig. 2 when all cytokines were combined (Additional file 2: Figure 2 code). While no individual cytokine showed any significant delayed temporal association, notable cytokines include eotaxin, IFN-γ, and MIP-1a which trended from brain to blood, while others showed a predominant delayed temporal association from blood to brain, such as IL-1α and IL-6 and the anticipated IL1ra which was given systemically (Additional file 2: Figure 2 code).

### Markers of systemic inflammation affect brain cytokine levels

**Fig. 3:**
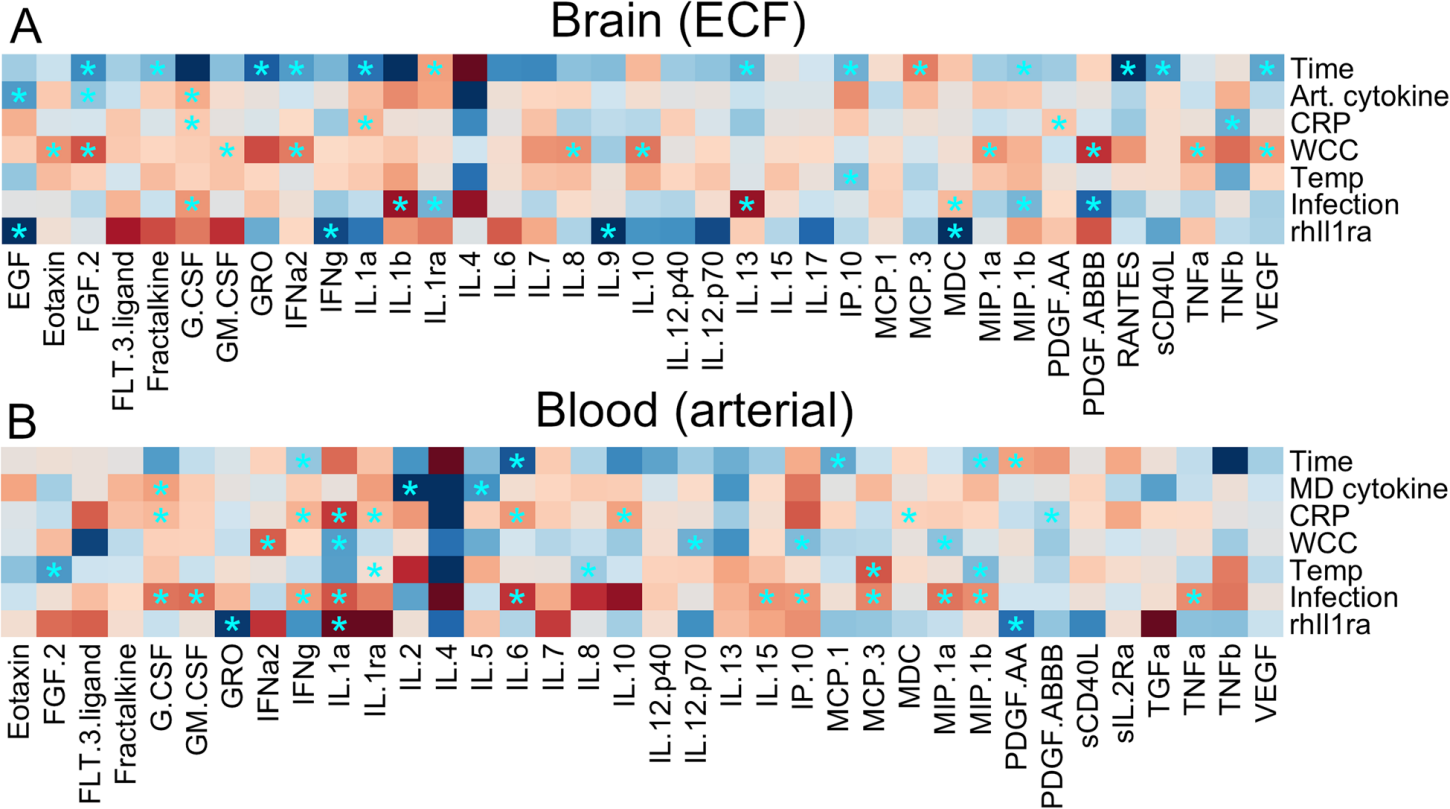
Coefficients of linear mixed effect models, displayed as heatmaps. The colours of the heatmaps are graded such that red represents positive coefficients and blue represents negative coefficients. All coefficients are normalized using the quotient of their standard deviation and that of the dependent variable. Significant coefficients are highlighted with an asterisk. Independent variables are along the y-axis and the dependent variable for each model is the cytokine of the respective row on the *x*-axis in either **A** the brain extracellular fluid or **B** arterial blood. Differences in cytokines displayed between the two subfigures are due to insufficient data to generate all coefficients from the model. *EGF* epidermal growth factor, *FGF*.*2* basic fibroblast growth factor, *FLT*.*3*.*ligand* Fms-related tyrosine kinase 3 ligand, *G*.*CSF* granulocyte colony stimulating factor, *GM*.*CSF* granulocyte-monocyte colony stimulating factor, *IFNa2* interferon alpha-2, *IFNg* interferon gamma, *IL* interleukin, *IL*-*1R* interleukin 1 receptor, *IL1ra* interleukin-1 receptor antagonist, *IL12p40* interleukin 12 subunit beta, *IL12p70* interleukin-12, *IP10* chemokine (C-X-C motif) ligand 10, *MCP* monocyte chemotactic protein, *MDC* macrophage-derived chemoattractant, *MIP1a* macrophage inflammatory protein-1alpha, *MIP1b* macrophage inflammatory protein-1beta, *PDGF* platelet-derived growth factor, *RANTES* chemokine (C-C motif) ligand 5, *sCD40L* soluble CD40 ligand, *sIL*.*2R* soluble interleuking-2 receptor, *TGFa* transforming growth factor alpha, *TNFa* tumour necrosis factor alpha, *TNFb* tumour necrosis factor beta, *VEGF* vascular endothelial growth factor

Among the systemic inflammatory markers, confirmed systemic clinical infections resulted in the largest relative effects on brain-ECF levels. Infections increased brain-ECF levels of, e.g. IL-1b and decreased levels of, e.g. IL-1ra (Fig. 3A and Additional file 5). rhIL1ra treatment resulted in significant alterations of both the systemic and neuroinflammatory responses (Fig. 3A). In brain-ECF, EGF, IFN-γ, IL-9 and MDC were statistically significant, all of which were associated with lower cerebral cytokine levels for the treatment group. High WCC and temperature generally showed associations to increased cerebral levels of cytokines, with stronger associations for WCC (Fig. 3A). Levels of CRP generally showed weaker normalized coefficients, and the clustering was most associated with arterial cytokine levels. Notably, increases in arterial cytokines were not necessarily associated with brain-ECF cytokine levels highlighting the difference between the two compartments.

Using a similar approach to see which parameters affect blood cytokine levels, confirmed systemic infections seemed to have the strongest positive association with several cytokines in blood, notably IL-6, IL-8 and IL-10 (Fig. 3B and Additional file 5). On a group level, CRP was more associated with increased blood cytokine levels than brain cytokine levels. Time from study inclusion also seemed to affect blood cytokine levels, but not as much as brain-ECF levels, and not as many cytokines decreased in blood over time as in the brain (Fig. 3B). Similar to the brain-ECF levels, rhIL1ra treatment resulted in a distinct separation between cytokines that were altered in blood, though these were not the same as those affected in the cerebral compartment. Altogether, these results highlight marked differences in the down-stream cytokine response to inflammatory stimuli in brain compared to its systemic counterparts.

### The systemic effect on brain-ECF levels remain following adjustment of baseline and injury severity parameters

Similar mixed-models were used to illustrate cytokine patterns using demographic and injury severity parameters. Interestingly, these quite strongly affected both brain-ECF and blood cytokine levels (Additional files 5-6). An increasing age was associated with increasing cytokine levels in brain-ECF, while lower levels in blood (Additional file 5). Similarly, female sex was associated with higher levels of cytokines in brain-ECF while the inverse was shown for blood cytokine levels where female sex was associated with a decrease for a majority of cytokines (Additional file 5). Stockholm CT score, ISS and GCSm showed relatively similar cytokine trends, clustering in proximity (Additional file 5).

**Fig. 4:**
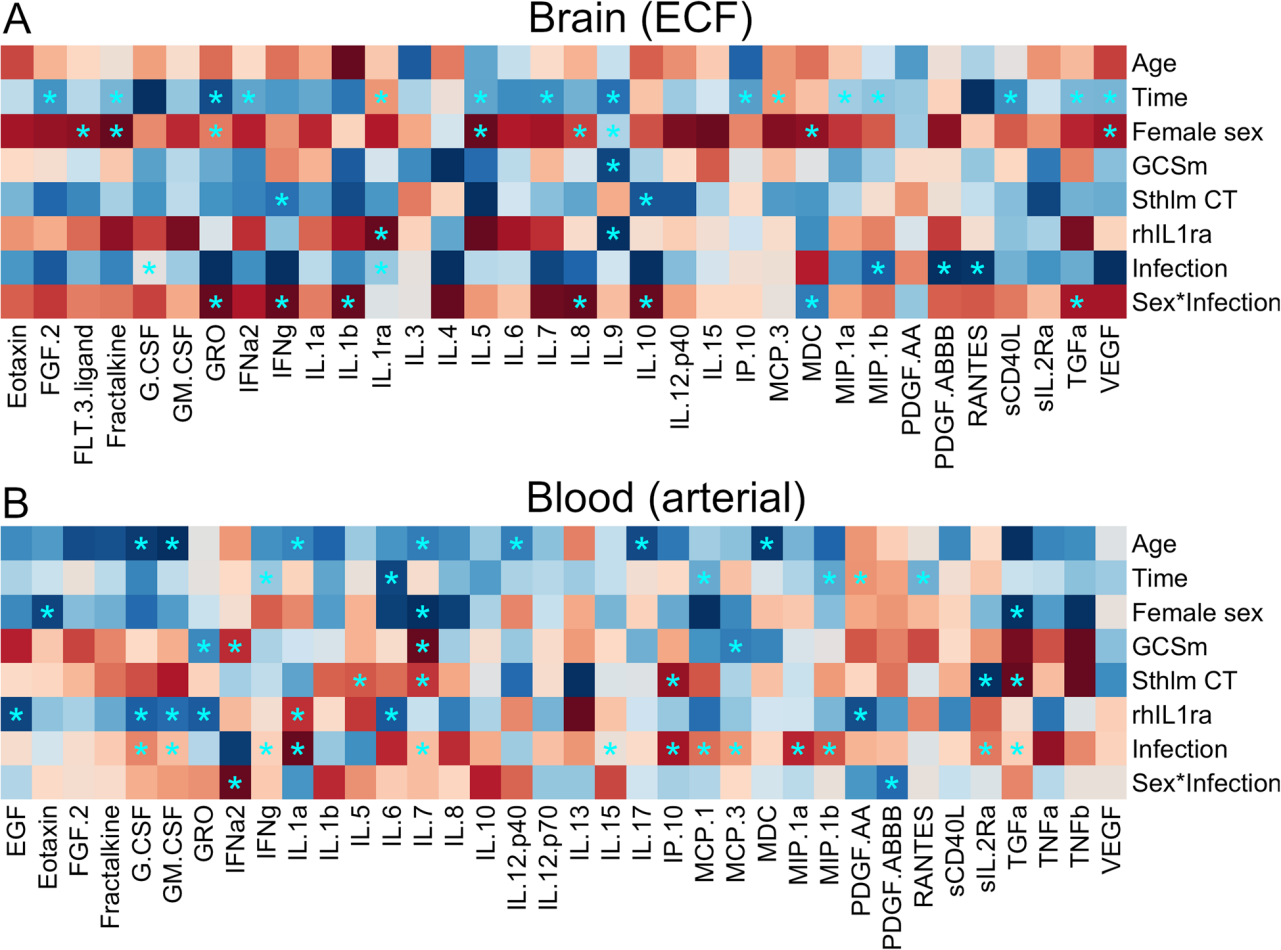
Coefficients of linear mixed effect models, displayed as heatmaps. The colours of the heatmaps are graded such that red represents positive coefficients and blue represents negative coefficients. All coefficients are normalized using the quotient of their standard deviation and that of the dependent variable. Significant coefficients are highlighted with an asterisk. An interaction term between female sex and infection. Independent variables are along the *y*-axis and the dependent variable for each model is the cytokine of the respective row on the *x*-axis in either **A** the brain extracellular fluid or **B** arterial blood. Differences in cytokines displayed between the two subfigures are due to insufficient data to generate all coefficients from the model

When combining demographic- and injury severity parameters with ‘clinical infection’ as our choice of infection marker, there were still brain-ECF cytokine levels independently associated with the development of a systemic infection (IL1-ra, G-CSF, PDGF-ABBB, MIP-1b and RANTES, *p* < 0.05 respectively) (Fig. 4A, B and Additional file 6). Interestingly, all these cytokines significantly decreased in brain-ECF following during. Since most patients with infection were female (7/8), an interaction term between sex and infection was added to this model. Interactions were not significant in any of the cytokines with significant coefficients for the impact of infection, and were significant only in three cytokines with significant coefficients for the impact of sex (Fig. 4A). In blood, clinical infections now showed a significant increase in fourteen cytokines (Fig. 4B), more than any other variable. rhIL1ra treatment showed similar trends following these types of adjustment for both brain and blood cytokines (Fig. 4A, B).

**Fig. 5:**
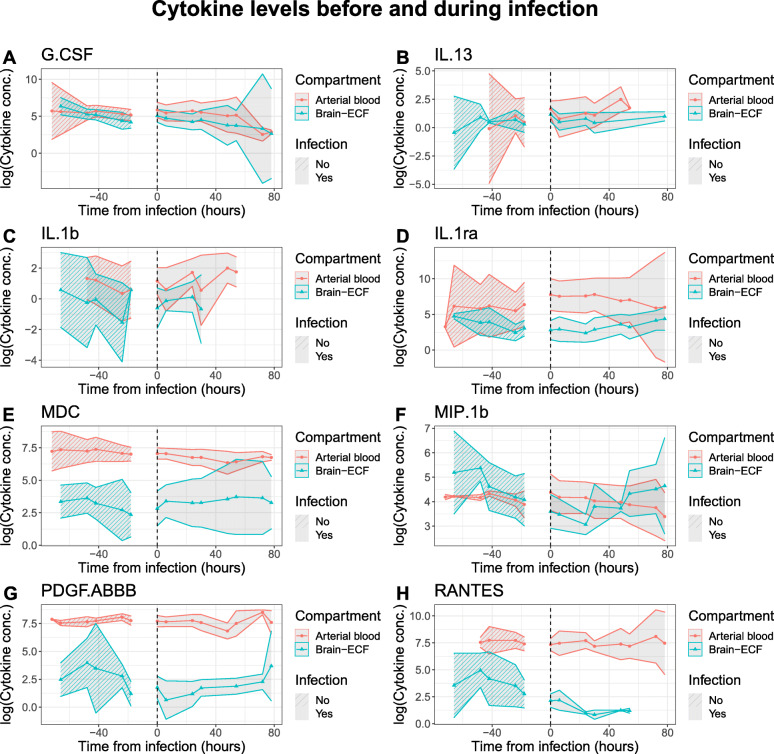
Cytokine levels before and after infection for patients with infection. Cytokines significant for infection in the heatmaps are shown as logarithms. The gap between data before infection and after infection is the time of uncertainty from last known non-infection to first known infection. The standard error of the mean was used to compute 95% confidence intervals

The different cytokine trends before and after the development of a clinical infection for brain-ECF and blood (of cytokines significant in Fig. 3A) are also visualized in Fig. 5. Here, it can be seen that arterial levels of cytokines are generally higher, and have an upwards trend while the opposite is shown for a majority of the brain-ECF cytokines.

## Discussion

We have demonstrated that following human TBI, there are distinct cytokine profiles that differ between blood and brain in response to systemic markers of inflammation, specifically infections, as well as an effect of anti-inflammatory treatment. To the best of our knowledge, this is the first study to study the interplay between peripheral/systemic markers of inflammation and cerebral cytokine levels in acute CNS conditions in humans.

### Peripheral inflammation modulates cerebral inflammation

The cytokine profile in brain-ECF was altered following the development of a systemic infection with a significant decrease seen in five cytokine concentrations (IL1-ra, G-CSF, PDGF-ABBB, MIP-1b and RANTES), while an increase in several cytokines were simultaneously seen in blood. Due to the inaccessibility of the human CNS, studies looking at simultaneous cerebral and systemic inflammation in living humans are scarce. In an autopsy study from *n* = 21 TBI patients, they noted an increase of IL-6, IL-8, TNFa and IL-1b mRNA levels which are significantly increased in tissue following injury [43]. They concluded that pro-inflammatory cytokines play a key role in the cerebral inflammatory response following TBI, but did not adjust for the influence of systemic infections/inflammation. Autopsy studies from patients that have died with sepsis reveal a distinct increase of microglia activation and astrocytosis as compared to non-sepsis controls [44, 45]. In an autopsy study by Warford et al, they noticed an increase of predominantly chemokines in sepsis patients, while TNFa was increased in all patients, IL-1b expression was upregulated in 2 out of 3 patients [45]. Unfortunately, neither the TBI or sepsis autopsy studies analysed the cytokines significantly decreasing in brain-ECF following systemic infections in our study. Patients with TBI and subarachnoid haemorrhages (SAH) that develop sepsis have been shown to exhibit higher levels of IL-6 in blood during the first week following ictus [46, 47], but not in CSF [48], showing similar differences between the blood and brain compartments seen in our study, and potential differences between studies analysing fluid vs tissue cytokine levels.

rhIL1ra treatment resulted in a strong alteration of brain and blood cytokine levels. Treatment with rhIL1ra in trials of human SAH have resulted in lower levels of IL-6 in both serum and CSF [49, 50], thus there is evidence that the drug will alter cytokine levels in several compartments following brain injury. Unfortunately, to our knowledge, there are no studies multiplexing cytokines following rhIL1ra treatment, making it difficult to compare our findings, though our new mixed-model in part validates the previous results found with different statistical approaches in the same material [28, 29]. While WCC, temperature and CRP levels were shown to be associated with cytokines, we believe clinical infection represent the best aggregate of inflammatory markers and a judgement call of the treating physician, why we used it throughout the manuscript to define systemic inflammation. Interestingly, WCC, temperature and CRP, commonly aggregated when defining infections, as well as almost all the cytokines, exhibit different information content (Additional file 7). Additional file 7 also demonstrates that, due to collinearity, the variable ‘clinical infection’ largely captures all these other inflammatory markers making it a suitable marker for systemic inflammation. While we specifically adjusted for time when analysing cytokine levels, we only implicitly used the timing of an infection by coding the infection parameter as 0 outside the time of infection and as 1 during an infection. Therefore, we did not draw any conclusions of differences between patients with early or late infections, respectively. However, this approach could at least to some extent account for timing of infection in the model.

### Patient demographics affect cytokine levels

The demographic and injury severity markers all showed some significant associations with alterations on cytokine levels in both brain-ECF and blood. Female sex was associated with higher cytokine levels in brain-ECF and lower levels in blood, as compared to males, after adjusting for interaction with infection, in which female patients were overrepresented. Sex differences concerning cytokine responses in humans have previously been shown [51, 52]. Mellergård and colleagues demonstrated in a cohort of SAH and TBI patients that IL-1b and IL-6, extracted from brain-ECF using MD, were higher and increased for a longer period in females compared to males [53]. Similarly, these cytokines were higher in females in our study as well, though several other cytokines exhibited even higher concentrations. In another study by Majetschak and co-workers, they could not see a sex difference in post-traumatic cytokine release in blood in a trauma cohort of *n* = 89 patients [54]. Older age was shown to increase levels of cytokine in brain-ECF, but decrease cytokine levels in blood. In the pre-clinical literature, TBI in the aged brain is often considered more detrimental due to a maladaptive neuroinflammatory response [55, 56], which could explain the increased cytokine response seen in brain-ECF in our study. In mild TBI, patients > 55 years old (compared to 21–54 years) levels of, e.g. IL-6, TNF and fractalkine were elevated in blood up to 6 months following injury [57]. TBI and polytrauma patients exhibit clinically relevant cytokine patterns in blood [58–60], CSF and brain-ECF [22], which was similar to what we noticed in our cohort, and demonstrates cytokine release from brain and other injured tissues.

### No clear delayed temporal association was seen between brain and blood cytokines

In general, we could not see any clear trends for cytokines moving from brain to blood, or vice versa. This could indicate that the movement between compartments occurs in a time frame much shorter than the sampling interval, and half-lives in vivo have been suggested to be short and thus cytokine concentrations very dynamic and could be difficult to assess [61]. However, the levels of some selected cytokines, like IL-6, IL-8 and TNFa, have been shown to be relatively stable for days following TBI in both serum and CSF, with selected cytokine concentrations higher in CSF than in serum [62, 63]. There are several studies that have serially sampled cytokines following human TBI in both serum and brain [42, 62, 64], but to the best of our knowledge we are the first to attempt a temporal cross correlation between brain-blood compartments. As is seen in these TBI studies, it is entirely possible that the gradient between compartments is such that the cytokine movement could follow a cerebral to systemic direction, as well as a systemic to brain as suggested by the preclinical studies using systemic LPS stimulation [13]. Further, a possible explanation for not being able to see any over-arching temporal associations could be that the 6h pooled epochs of brain microdialysates are too long to detect rapid cytokine fluxes. Moreover, our results could also reflect any inherent differences and circumstances concerning cytokine generation/degradation or consumption in brain-ECF and blood, respectively. This is supported by the cytokine concentration similarities found in both arterial and jugular blood vs brain-ECF. Some cytokines showed a predominant delayed temporal association from brain to blood and vice-versa, but altogether, while we cannot rule out that temporal correlations and fluxes are absent due to intrinsic compartmental differences, future techniques might make more frequent sampling possible.

### Both arterial and jugular compartments hold similar information content

There was no difference in cytokine levels between jugular- and arterial blood, as compared to the distinctly different brain-ECF compartment. A jugular-arterial gradient has been suggested with higher levels of cytokines and brain protein biomarkers in blood, indicative of a cerebral source of these markers [24, 65]. In fact, we noticed that more cytokines were higher in arterial blood as compared to jugular blood, which could indicate that a systemic inflammation masked any increase of cytokines in the jugular compartment. Jugular samples had a higher degree of missingness, but it is uncertain how this would affect the analyses. McKeating and colleagues looked at the jugular-arterial gradient in a mixed cohort of TBI and SAH patients, and noted a trans-cranial gradient of IL-6 with higher levels in jugular as compared to arterial blood the first 48 h after injury [24] (this could not be seen in our material (data not shown)). They could not see any difference for IL-1b, TNFa or IL-8, which could be due to a significant amount of missing data in their study (86%, 88% and 52%, respectively) [24]. Possibly, the effective half-life of any brain-released cytokines is longer than the time it takes to reach the arterial compartment. Additionally, any marker that can only be accessed in a jugular compartment is in all likelihood too erratic to act as a good marker of neuro-inflammation clinically.

### Clinical implications

It is currently unclear how a systemic inflammation impairs cerebral functions and causes an inflammatory response, though it has been shown that concomitant infections in TBI increase the risk of an unfavourable outcome [9]. Several theories suggest that cytokines in blood targets different cerebral receptors, resulting in, for example, drowsiness, effects on the circulation and respiration, hyperthermia and cognitive implications [66, 67]. However, we can demonstrate that systemic infections not only result in extensive increase of cytokines in blood, but also alter the cytokine levels in brain-ECF. This suggests that infections in TBI patients could benefit from more aggressive treatment, as their brain is presumably more susceptible to this potentially detrimental neuroinflammatory response [9] with potential impacts on both the immunological response to infection and a detrimental inflammatory response with respect to the TBI. However, due to the low sample size, we could not associate these cytokine dynamics with brain pathology or outcome, limiting the results to an exploratory and preliminary nature, and further research in larger cohorts is necessary. Interestingly, levels of IL-6 and IL-8 seen in patients suffering from Covid-19 have been shown to be similar to those of trauma patients [68]. As the notorious ‘cytokine-storm’ in this disease, as well as many other septicaemias, have been attributed to an unfavourable outcome, we believe that cytokine monitoring of both brain and blood compartments are crucial in order to better understand the pathophysiology and to device new anti-inflammatory treatment strategies for brain injured patients.

### Limitations

Due to ethical restraints, there is a lack of proper controls without a concomitant brain injury. We are limited to TBI patients where microdialysis catheters are inserted as part of clinical management (while usually harmless, insertion of these catheters may result in haemorrhages and infections [69]). Thus, it becomes difficult to assess exactly what cytokine signal that comes from the injured brain and what may be the result of a peripheral infection. However, we did attempt to adjust for both ISS as a marker of extracranial injury severity and Stockholm CT-score as a marker of intracranial injury severity (as well as other demographic factors) in order to filter out the exact contribution of the systemic inflammation. Furthermore, we tried to address these questions in relatively small sample size of only 20 patients, where we parametrized the inter-patient variability (Additional file 3). However, for each block seen in the heatmaps (Figs. 3 and 4), a total of 382 data points were used in generating the results. In short, our methods allow us to include all the acquired brain-ECF samples, adjusted for individual patients over time, in order to conduct our analyses. That being said, the low sample size in this study renders the results exploratory and requires validation in larger prospective trials.

As has been highlighted in previous microdialysis studies, the relative recovery (levels of cytokines extracted through the pores in the microdialysis catheter/actual level of cytokines in a fluid) will strongly influence the levels measured in brain-ECF [25, 70]. As can be seen in Additional file 1, there are a few cytokines with a lot of missing data from the brain-ECF compartment (e.g. IL-4), highlighting the limitations of the current method for these selected cytokines. However, there are no other methods available to access the brain-ECF in vivo than microdialysis, and the Luminex technology is still amongst the more sensitive methods for multiplexing small volumes while also retaining a standard curve for absolute concentrations. The technique to measure cytokines in brain-ECF is also largely unavailable outside of the research setting, and is currently associated with substantial costs, obstructing translation into main-stream clinical use.

Previous studies studying cytokines in TBI often select one or a few, and assign them attributes being ‘pro-’ or ‘anti-inflammatory’ [22]. The inflammatory pathways are often more complex than this, so we believe that this simplification could potentially skew our understanding by attributing biological function to cytokines that are easy to measure using a particular technique. By multiplexing > 40 cytokines from several compartments, we instead chose to focus on them all in a more unbiased fashion. Out of the seven SAEs reported in the study, five were infections. The other two were neutropenia of unknown etiology (in the control group), and right upper lobe lung collapse after bronchoscopy to remove traumatic clots (in the treatment group). While we believe that infections are the most likely cause of alterations in brain and blood cytokine levels, it is possible that these other two (non-infection) events resulted in cytokine changes as well.

## Conclusions

Systemic inflammation alters the cerebral inflammatory response with lower levels seen in brain-ECF, while higher levels were seen in blood, which remains after adjusting for relevant demographic and injury severity markers. rhIL1ra treatment affected systemic and cerebral cytokine levels, demonstrating the effect of anti-inflammatory treatment following TBI. Venous jugular and arterial compartments contain similar cytokine information following TBI, while the cerebral compartment has a unique profile. While these results should be considered exploratory due to the small sample size, improved monitoring of the neuroinflammatory response and better management of systemic infections could potentially improve outcome in acute brain injuries, although larger trials are needed to confirm these assumptions.

## Supplementary Information

**Additional file 1.** Title of data: Baseline data. Description of data: Columns for the first eight rows are for each patient (C, control; I, intervention); moreover, the first four rows have time along the horizontal axis. Columns for the final row are for each cytokine. Rows 1-3 display changes over time of white cell count (WCC), C-reactive protein (CRP) and temperature, with corresponding lines of best fit. Row 4 displays infection status of patients over time. Row 5 shows infection (VAP, ventilator-associated pneumonia; IAS intra-abdominal sepsis; CI, chest infection), severe adverse events (SAE), sex (M, male; F, female) and years of age for each patient. Row 6 shows Stockholm computed tomography (CT) score for each patient, coloured after whether a deterioration was observed during hospital stay. Row 7 shows injury severity score (ISS) for each patient. Row 8 shows fraction of missingness in each compartment and for each patient. Row 9 shows fraction of missing cytokines in each compartment. The chest infection was a result of mucous plugging resulting in a right lower lobe collapse, hence not fulfilling VAP criteria.

**Additional file 2.** Title of data: Code. Description of data: Code for generating all figures and tables provided. Order of appearance: Figures 1-5, Tables 1-2, Additional file 6-7. Additional file 5 is generated by code for Figures 3-4.

**Additional file 3.** Title of data: Mixed models details. Description of data: Statistical details.

**Additional file 4.** Title of data: Timing of blood samples and cytokine pooling. Description of data: Illustrating the timing between microdialysis samples, blood compartment samples and recombinant human Interleukin 1 receptor antagonist (rhIL1ra) treatment. Microdialysis sampling (green) were pooled during 6 h epochs throughout the study. The rhIL1ra treatment was administered once daily to patients in the treatment arm (blue). Blood samples were taken one hour before and one hour after administration of rhIL1ra (or equivalent timing, but no drug, in the control group) (red).

**Additional file 5. **Title of data: Coefficients and *p*-values of mixed models. Description of data: There are 12 sheets, with coefficients and *p*-values of mixed models corresponding to each of Figures 3-4 A-B and Additional file 6. In the 6 sheets containing coefficients, maximum and minimum coefficients for every variable are highlighted at the bottom. Theoretical equivalent increments required in other variables than infection to change their respective concentrations of cytokines with maximum or minimum coefficients by the same amount as a change from 0 to 1 in “infection” are shown. Furthermore, theoretical equivalent increments required in other variables than infection to change the concentrations of cytokines with maximum or minimum infection coefficients by the same amount as a change from 0 to 1 in “infection” are shown. The remaining 6 sheets contain *p*-values of coefficients of linear mixed effect models. Significant values at *p* < 0.05 were marked with an asterisk in Figures 3-4 A-B.

**Additional file 6. **Description of data: Coefficients of linear mixed effect models, displayed as heatmaps. The colours of the heatmaps are graded such that red represents positive coefficients and blue represents negative coefficients. All coefficients are normalized using the quotient of their standard deviation and that of the dependent variable. Significant coefficients are highlighted with an asterisk. Independent variables are along the *x*-axis and the dependent variable for each model is the cytokine of the respective row on the *y*-axis in either (A) the brain extracellular fluid or (B) arterial blood. Differences in cytokines displayed between the two subfigures is due to insufficient data to generate all coefficients from the model.

**Additional file 7.** Title of data: Principal component analysis of all cytokines, with additional inflammatory variables. Description of data: Unlabelled arrows are loadings of cytokines. Labelled and dashed arrows are post-analysis projections of continuous inflammatory markers. Infection and non-infection shaded areas show confidence intervals of scores labelled as infection or non-infection.

## Data Availability

The datasets generated and/or analysed during the current study are not publicly available due to local regulations, but are available from the corresponding author on reasonable request.

## Abbreviations

CNS: Central nervous system
CRP: C-reactive protein
CSF: Cerebrospinal fluid
CT: Computerized tomography
ECF: Extracellular fluid
EGF: Epidermal growth factor
G-CSF: Granulocyte colony-stimulating factor
GCSm: Glasgow coma scale, motor score
IFN: Interferon
IL: Interleukin
ISS: Injury severity score
LPS: Lipopolysaccharide
MD: Microdialysis
MDC: Macrophage-derived chemokine
MFA: Multiple factor analysis
MS: Multiple sclerosis
PCA: Principal component analysis
PDGF: Platelet-derived growth factor
PET: Positron emission tomography
PLS-DA: Partial least squares-discriminant analysis
rhIL1ra: Recombinant human interleukin-1 receptor antagonist
SAH: Subarachnoid haemorrhage
sICAM-1: Soluble intracellular adhesion molecule-1
TBI: Traumatic brain injury
TNF: Tumour necrosis factor
VAP: Ventilator-associated pneumonia
VIF: Variance inflation factor
WBC: White blood cell count
WCC: White cell count

## References

[1] Roozenbeek B, Maas AI, Menon DK (2013). Changing patterns in the epidemiology of traumatic brain injury. Nat Rev Neurol.

[2] Hyder AA, Wunderlich CA, Puvanachandra P, Gururaj G, Kobusingye OC (2007). The impact of traumatic brain injuries: a global perspective. NeuroRehabilitation.

[3] Werner C, Engelhard K (2007). Pathophysiology of traumatic brain injury. Br J Anaesth.

[4] Hinson HE, Rowell S, Schreiber M (2015). Clinical evidence of inflammation driving secondary brain injury: a systematic review. J Trauma Acute Care Surg.

[5] Schwulst SJ, Trahanas DM, Saber R, Perlman H (2013). Traumatic brain injury-induced alterations in peripheral immunity. J Trauma Acute Care Surg.

[6] Ritzel RM, Doran SJ, Barrett JP, Henry RJ, Ma EL, Faden AI, Loane DJ (2018). Chronic alterations in systemic immune function after traumatic brain injury. J Neurotrauma.

[7] Hazeldine J, Lord JM, Belli A (2015). Traumatic brain injury and peripheral immune suppression: primer and prospectus. Front Neurol.

[8] Rowe RK, Ellis GI, Harrison JL, Bachstetter AD, Corder GF, Van Eldik LJ, Taylor BK, Marti F, Lifshitz J (2016). Diffuse traumatic brain injury induces prolonged immune dysregulation and potentiates hyperalgesia following a peripheral immune challenge. Mol Pain.

[9] Sharma R, Shultz SR, Robinson MJ, Belli A, Hibbs ML, O'Brien TJ, Semple BD (2019). Infections after a traumatic brain injury: The complex interplay between the immune and neurological systems. Brain Behav Immun.

[10] Doran SJ, Henry RJ, Shirey KA, Barrett JP, Ritzel RM, Lai W, Blanco JC, Faden AI, Vogel SN, Loane DJ (2020). Early or late bacterial lung infection increases mortality after Traumatic brain injury in male mice and chronically impairs monocyte innate immune function. Crit Care Med.

[11] Li Y, Liu C, Xiao W, Song T, Wang S (2020). Incidence, risk factors, and outcomes of ventilator-associated pneumonia in traumatic brain injury: a meta-analysis. Neurocrit Care.

[12] Zhao J, Bi W, Xiao S, Lan X, Cheng X, Zhang J, Lu D, Wei W, Wang Y, Li H, Fu Y, Zhu L (2019). Neuroinflammation induced by lipopolysaccharide causes cognitive impairment in mice. Sci Rep.

[13] Henry CJ, Huang Y, Wynne AM, Godbout JP (2009). Peripheral lipopolysaccharide (LPS) challenge promotes microglial hyperactivity in aged mice that is associated with exaggerated induction of both pro-inflammatory IL-1beta and anti-inflammatory IL-10 cytokines. Brain Behav Immun.

[14] Chakravarty S, Herkenham M (2005). Toll-like receptor 4 on nonhematopoietic cells sustains CNS inflammation during endotoxemia, independent of systemic cytokines. J Neurosci.

[15] Cunningham C, Campion S, Lunnon K, Murray CL, Woods JF, Deacon RM, Rawlins JN, Perry VH (2009). Systemic inflammation induces acute behavioral and cognitive changes and accelerates neurodegenerative disease. Biol Psychiatry.

[16] Lynch JR, Tang W, Wang H, Vitek MP, Bennett ER, Sullivan PM, Warner DS, Laskowitz DT (2003). APOE genotype and an ApoE-mimetic peptide modify the systemic and central nervous system inflammatory response. J Biol Chem.

[17] Hannestad J, Gallezot JD, Schafbauer T, Lim K, Kloczynski T, Morris ED, Carson RE, Ding YS, Cosgrove KP (2012). Endotoxin-induced systemic inflammation activates microglia: [(1)(1)C]PBR28 positron emission tomography in nonhuman primates. Neuroimage.

[18] Perry VH (2010). Contribution of systemic inflammation to chronic neurodegeneration. Acta Neuropathol.

[19] Park SE, Song D, Shin K, Nam SO, Ko A, Kong J, Kim YM, Yeon GM, Lee YJ (2019). Prospective research of human parechovirus and cytokines in cerebrospinal fluid of young children less than one year with sepsis-like illness: Comparison with enterovirus. J Clin Virol.

[20] Coutinho LG, Grandgirard D, Leib SL, Agnez-Lima LF (2013). Cerebrospinal-fluid cytokine and chemokine profile in patients with pneumococcal and meningococcal meningitis. BMC Infect Dis.

[21] Winter PM, Dung NM, Loan HT, Kneen R, Wills B, Thu le T, House D, White NJ, Farrar JJ, Hart CA, Solomon T (2004). Proinflammatory cytokines and chemokines in humans with Japanese encephalitis. J Infect Dis.

[22] Zeiler FA, Thelin EP, Czosnyka M, Hutchinson PJ, Menon DK, Helmy A (2017). Cerebrospinal fluid and microdialysis cytokines in severe traumatic brain injury: a scoping Systematic Review. Front Neurol.

[23] Lindblad C, Pin E, Just D, Nimer FA, Nilsson P, Bellander B-M, Svensson M, Piehl F, Thelin EP (2020). Fluid proteomics of CSF and serum reveal important neuroinflammatory proteins in blood-brain barrier disruption and outcome prediction following severe traumatic Brain Injury: a Prospective, Observational Study. Crit Care.

[24] McKeating EG, Andrews PJ, Signorini DF, Mascia L (1997). Transcranial cytokine gradients in patients requiring intensive care after acute brain injury. Br J Anaesth.

[25] Helmy A, Carpenter KL, Skepper JN, Kirkpatrick PJ, Pickard JD, Hutchinson PJ (2009). Microdialysis of cytokines: methodological considerations, scanning electron microscopy, and determination of relative recovery. J Neurotrauma.

[26] Hutchinson PJ, Jalloh I, Helmy A, Carpenter KL, Rostami E, Bellander BM, Boutelle MG, Chen JW, Claassen J, Dahyot-Fizelier C (2015). Consensus statement from the 2014 International Microdialysis Forum. Intensive Care Med.

[27] Allan SM, Tyrrell PJ, Rothwell NJ (2005). Interleukin-1 and neuronal injury. Nat Rev Immunol.

[28] Helmy A, Guilfoyle MR, Carpenter KL, Pickard JD, Menon DK, Hutchinson PJ (2014). Recombinant human interleukin-1 receptor antagonist in severe traumatic brain injury: a phase II randomized control trial. J Cereb Blood Flow Metab.

[29] Helmy A, Guilfoyle MR, Carpenter KL, Pickard JD, Menon DK, Hutchinson PJ (2016). Recombinant human interleukin-1 receptor antagonist promotes M1 microglia biased cytokines and chemokines following human traumatic brain injury. J Cereb Blood Flow Metab.

[30] Helmy A, Vizcaychipi M, Gupta AK (2007). Traumatic brain injury: intensive care management. Br J Anaesth.

[31] American Thoracic S (2005). Infectious Diseases Society of A: Guidelines for the management of adults with hospital-acquired, ventilator-associated, and healthcare-associated pneumonia. Am J Respir Crit Care Med.

[32] Teasdale G, Jennett B (1976). Assessment and prognosis of coma after head injury. Acta Neurochir (Wien).

[33] Medicine AftAoA: Abbreviated Injury Scale (c) 2005 Update 2008. Chicago, Illinois; 2016.

[34] Nelson DW, Nystrom H, MacCallum RM, Thornquist B, Lilja A, Bellander BM, Rudehill A, Wanecek M, Weitzberg E (2010). Extended analysis of early computed tomography scans of traumatic brain injured patients and relations to outcome. J Neurotrauma.

[35] Lê S, Josse J, Husson F (2008). FactoMineR: A Package for Multivariate Analysis. J Stat Softw.

[36] Pinheiro J, Bates D, DebRoy S, Sarkar D, Team RC (2019). nlme: linear and nonlinear mixed effects models.

[37] Stanstrup J: massageR. 0.7.2 edition; 2017.

[38] Wickham H (2009). ggplot2: Elegant Graphics for Data Analysis.

[39] Gregory R, Warnes BB, Lodewijk B (2016). gplots: Various R programming tools for plotting data.

[40] Pagès J (2004). Multiple factor analysis: main features and application to sensory data. Rev Colomb Estad.

[41] Baker SP, O'Neill B, Haddon W, Long WB (1974). The injury severity score: a method for describing patients with multiple injuries and evaluating emergency care. J Trauma.

[42] Helmy A, Carpenter KL, Menon DK, Pickard JD, Hutchinson PJ (2011). The cytokine response to human traumatic brain injury: temporal profiles and evidence for cerebral parenchymal production. J Cereb Blood Flow Metab.

[43] Frugier T, Morganti-Kossmann MC, O'Reilly D, McLean CA (2010). In situ detection of inflammatory mediators in post mortem human brain tissue after traumatic injury. J Neurotrauma.

[44] Lemstra AW, Groen in't Woud JC, Hoozemans JJ, van Haastert ES, Rozemuller AJ, Eikelenboom P, van Gool WA (2007). Microglia activation in sepsis: a case-control study. J Neuroinflammation.

[45] Warford J, Lamport AC, Kennedy B, Easton AS (2017). Human brain chemokine and cytokine expression in sepsis: a report of three cases. Can J Neurol Sci.

[46] Chaudhry SR, Stoffel-Wagner B, Kinfe TM, Guresir E, Vatter H, Dietrich D, Lamprecht A, Muhammad S (2017). Elevated systemic IL-6 levels in patients with aneurysmal subarachnoid hemorrhage is an unspecific marker for post-SAH complications. Int J Mol Sci.

[47] Woiciechowsky C, Schoning B, Cobanov J, Lanksch WR, Volk HD, Docke WD (2002). Early IL-6 plasma concentrations correlate with severity of brain injury and pneumonia in brain-injured patients. J Trauma.

[48] Vlachogiannis P, Hillered L, Khalil F, Enblad P, Ronne-Engstrom E (2019). Interleukin-6 levels in Cerebrospinal fluid and plasma in patients with severe spontaneous subarachnoid hemorrhage. World Neurosurg.

[49] Galea J, Ogungbenro K, Hulme S, Patel H, Scarth S, Hoadley M, Illingworth K, McMahon CJ, Tzerakis N, King AT (2018). Reduction of inflammation after administration of interleukin-1 receptor antagonist following aneurysmal subarachnoid hemorrhage: results of the Subcutaneous Interleukin-1Ra in SAH (SCIL-SAH) study. J Neurosurg.

[50] Singh N, Hopkins SJ, Hulme S, Galea JP, Hoadley M, Vail A, Hutchinson PJ, Grainger S, Rothwell NJ, King AT, Tyrrell PJ (2014). The effect of intravenous interleukin-1 receptor antagonist on inflammatory mediators in cerebrospinal fluid after subarachnoid haemorrhage: a phase II randomised controlled trial. J Neuroinflammation.

[51] Wegner A, Benson S, Rebernik L, Spreitzer I, Jager M, Schedlowski M, Elsenbruch S, Engler H (2017). Sex differences in the pro-inflammatory cytokine response to endotoxin unfold in vivo but not ex vivo in healthy humans. Innate Immun.

[52] Asai K, Hiki N, Mimura Y, Ogawa T, Unou K, Kaminishi M (2001). Gender differences in cytokine secretion by human peripheral blood mononuclear cells: role of estrogen in modulating LPS-induced cytokine secretion in an ex vivo septic model. Shock.

[53] Mellergard P, Aneman O, Sjogren F, Saberg C, Hillman J (2011). Differences in cerebral extracellular response of interleukin-1beta, interleukin-6, and interleukin-10 after subarachnoid hemorrhage or severe head trauma in humans. Neurosurgery.

[54] Majetschak M, Christensen B, Obertacke U, Waydhas C, Schindler AE, Nast-Kolb D, Schade FU (2000). Sex differences in posttraumatic cytokine release of endotoxin-stimulated whole blood: relationship to the development of severe sepsis. J Trauma.

[55] Campuzano O, Castillo-Ruiz MM, Acarin L, Castellano B, Gonzalez B (2009). Increased levels of proinflammatory cytokines in the aged rat brain attenuate injury-induced cytokine response after excitotoxic damage. J Neurosci Res.

[56] Kumar A, Stoica BA, Sabirzhanov B, Burns MP, Faden AI, Loane DJ (2013). Traumatic brain injury in aged animals increases lesion size and chronically alters microglial/macrophage classical and alternative activation states. Neurobiol Aging.

[57] Thompson HJ, Martha SR, Wang J, Becker KJ (2020). Impact of age on plasma inflammatory biomarkers in the 6 months following mild traumatic brain injury. J Head Trauma Rehabil.

[58] Rowland B, Savarraj JPJ, Karri J, Zhang X, Cardenas J, Choi HA, Holcomb JB, Wade CE (2020). Acute inflammation in traumatic brain injury and polytrauma patients using network analysis. Shock.

[59] Ferreira LC, Regner A, Miotto KD, Moura S, Ikuta N, Vargas AE, Chies JA, Simon D (2014). Increased levels of interleukin-6, -8 and -10 are associated with fatal outcome following severe traumatic brain injury. Brain Inj.

[60] Buttram SD, Wisniewski SR, Jackson EK, Adelson PD, Feldman K, Bayir H, Berger RP, Clark RS, Kochanek PM (2007). Multiplex assessment of cytokine and chemokine levels in cerebrospinal fluid following severe pediatric traumatic brain injury: effects of moderate hypothermia. J Neurotrauma.

[61] Zhou X, Fragala MS, McElhaney JE, Kuchel GA (2010). Conceptual and methodological issues relevant to cytokine and inflammatory marker measurements in clinical research. Curr Opin Clin Nutr Metab Care.

[62] Csuka E, Morganti-Kossmann MC, Lenzlinger PM, Joller H, Trentz O, Kossmann T (1999). IL-10 levels in cerebrospinal fluid and serum of patients with severe traumatic brain injury: relationship to IL-6, TNF-alpha, TGF-beta1 and blood-brain barrier function. J Neuroimmunol.

[63] Woodcock T, Morganti-Kossmann MC (2013). The role of markers of inflammation in traumatic brain injury. Front Neurol.

[64] Yan EB, Satgunaseelan L, Paul E, Bye N, Nguyen P, Agyapomaa D, Kossmann T, Rosenfeld JV, Morganti-Kossmann MC (2014). Post-traumatic hypoxia is associated with prolonged cerebral cytokine production, higher serum biomarker levels, and poor outcome in patients with severe traumatic brain injury. J Neurotrauma.

[65] Raabe A, Menon DK, Gupta S, Czosnyka M, Pickard JD (1998). Jugular venous and arterial concentrations of serum S-100B protein in patients with severe head injury: a pilot study. J Neurol Neurosurg Psychiatry.

[66] Dantzer R, O'Connor JC, Freund GG, Johnson RW, Kelley KW (2008). From inflammation to sickness and depression: when the immune system subjugates the brain. Nat Rev Neurosci.

[67] Perry VH (2004). The influence of systemic inflammation on inflammation in the brain: implications for chronic neurodegenerative disease. Brain Behav Immun.

[68] Kox M, Waalders NJB, Kooistra EJ, Gerretsen J, Pickkers P (2020). Cytokine levels in critically ill patients with COVID-19 and other conditions. JAMA.

[69] Zeiler FA, Thelin EP, Helmy A, Czosnyka M, Hutchinson PJA, Menon DK (2017). A systematic review of cerebral microdialysis and outcomes in TBI: relationships to patient functional outcome, neurophysiologic measures, and tissue outcome. Acta Neurochir (Wien).

[70] Ao X, Stenken JA (2006). Microdialysis sampling of cytokines. Methods.

