# Supplementary material for: Systemic inflammation alters the neuroinflammatory response: a prospective clinical trial in traumatic brain injury
Source: J Neuroinflammation. 2021 Sep 25;18:221. doi: 10.1186/s12974-021-02264-2 (PMC8464153; doi:10.1186/s12974-021-02264-2)
Supplement: Supplementary file 2 — Additional file 2. Title of data: Code. Description of data: Code for generating all figures and tables provided. Order of appearance: Figures 1-5, Tables 1-2, Additional file 6-7. Additional file 5 is generated by code for Figures 3-4. [file 12974_2021_2264_MOESM2_ESM.pdf]

Code for Figure 1 (a)

Philipp

Revised: May 11, 2021

Contents

Load data 1

Run PCA for each interleukin 1

Test MFA 23

Load data

```
library(openxlsx)
library(dplyr)
```

```
##
## Attaching package: 'dplyr'
## The following objects are masked from 'package:stats':
##
##     filter, lag
## The following objects are masked from 'package:base':
##
##     intersect, setdiff, setequal, union
```

```
ip_data <- read.xlsx("../../Dataset/Interpolated_data4.1.xlsx")
na_rows <- ip_data %>% select(contains("_")) %>% is.na(.) %>% rowSums(.) == 126
ip_data <- ip_data[!na_rows, ]
```

Run PCA for each interleukin

```
library(FactoMineR)
library(factoextra)
```

```
## Loading required package: ggplot2
## Welcome! Want to learn more? See two factoextra-related books at https://goo.gl/ve3WBa
```

```
library(ggplot2)

for (i in 1:42) {
```

```
  il.pca <- PCA(na.omit(ip_data[, (5+3*i):(7+3*i)]), graph = FALSE)
  il.name <- substr(colnames(ip_data)[5+3*i], 4, 100)
  my_plot_hook <- function(x, options)
    paste("\n", knitr::hook_plot_tex(x, options), "\n")
    knitr::knit_hooks$set(plot = my_plot_hook)

  tryCatch(
    {
        print(fviz_pca_var(il.pca, col.var = "cos2", title = il.name,
                           gradient.cols = c("#00AFBB", "#E7B800", "#FC4E07"),
                           repel = TRUE))
    }, error = function(e)
      {
      print(paste("Error in", il.name))
      }
  )

}
```

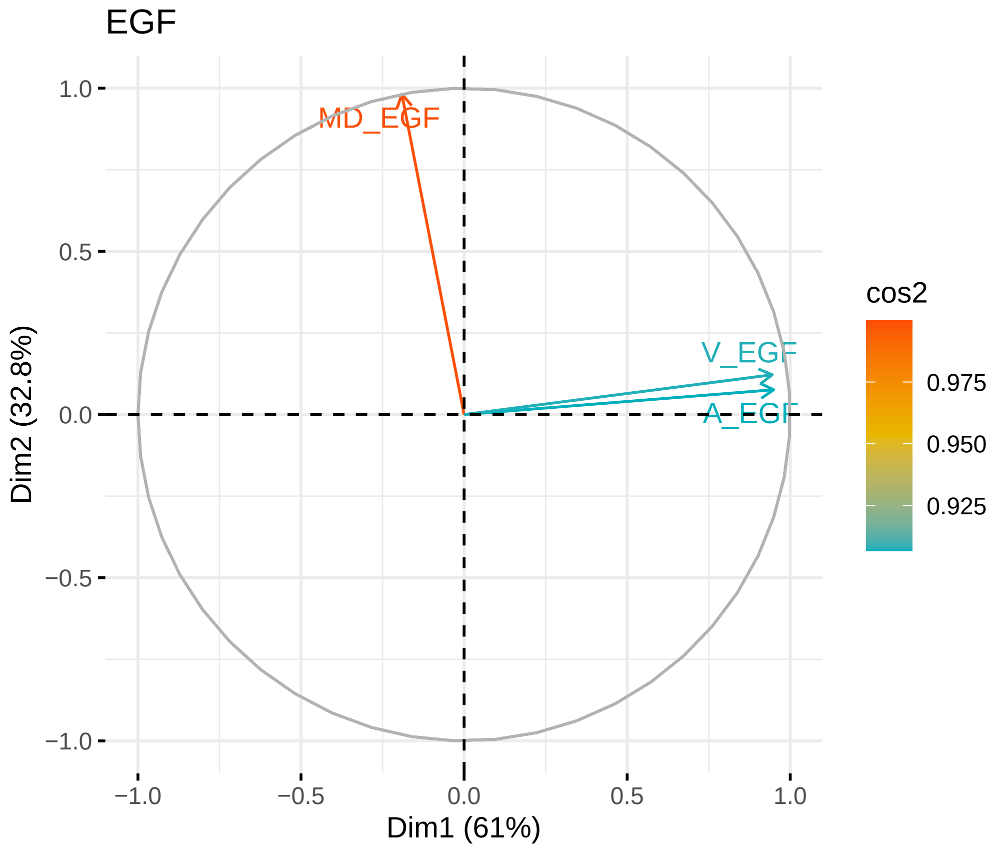

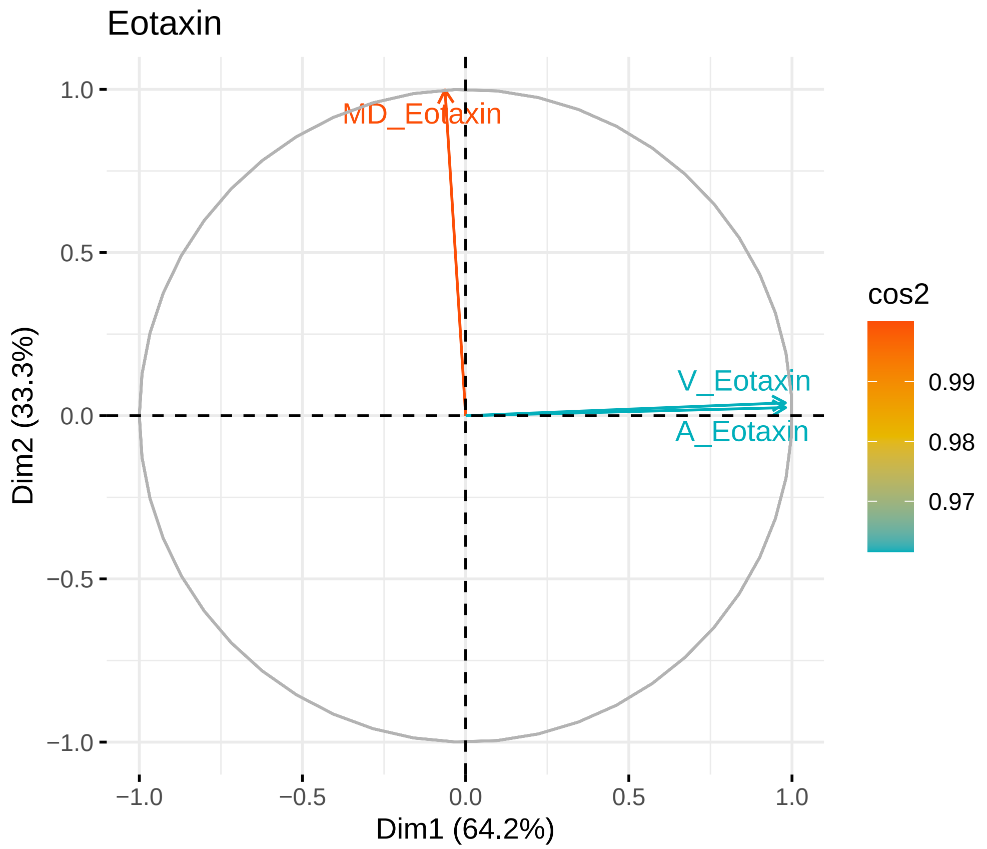
Eotaxin
MD_Eotaxin
V_Eotaxin
A_Eotaxin
Dim2 (33.3%)
Dim1 (64.2%)
1.0
0.5
0.0
−0.5
−1.0
−1.0
−0.5
0.0
0.5
1.0
cos2
0.99
0.98
0.97

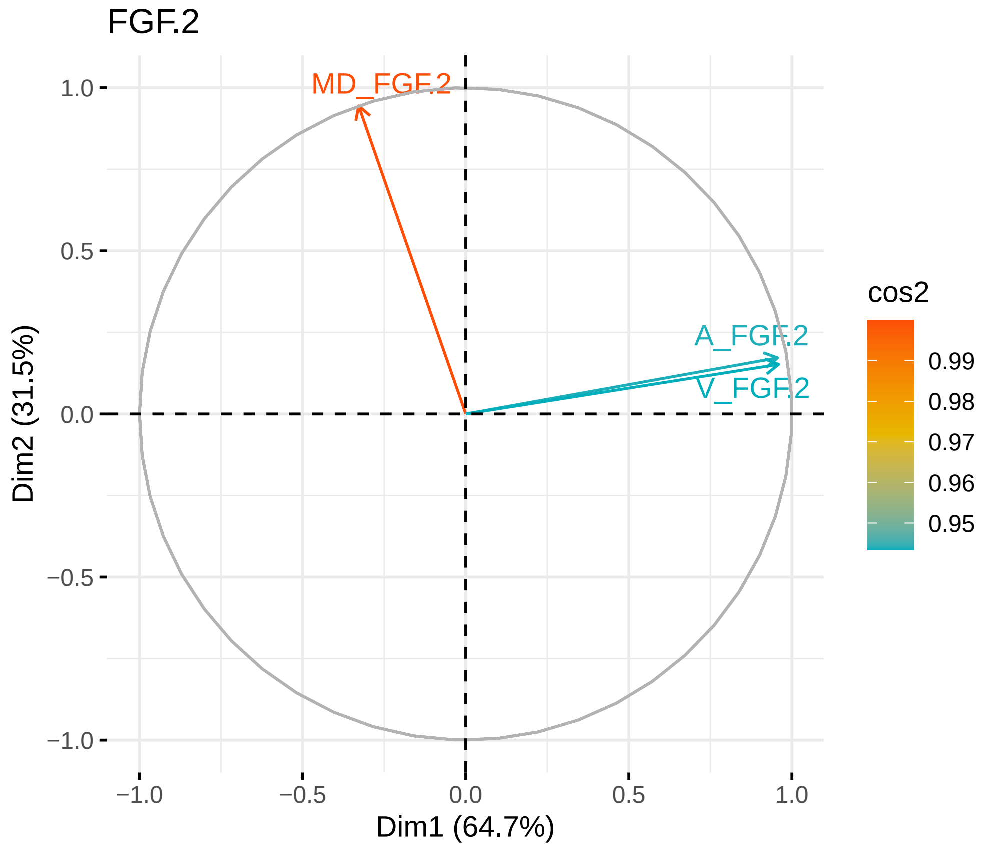
FGF.2
MD_FGF.2
A_FGF.2
V_FGF.2
Dim2 (31.5%)
Dim1 (64.7%)
1.0
0.5
0.0
−0.5
−1.0
−1.0
−0.5
0.0
0.5
1.0
cos2
0.99
0.98
0.97
0.96
0.95

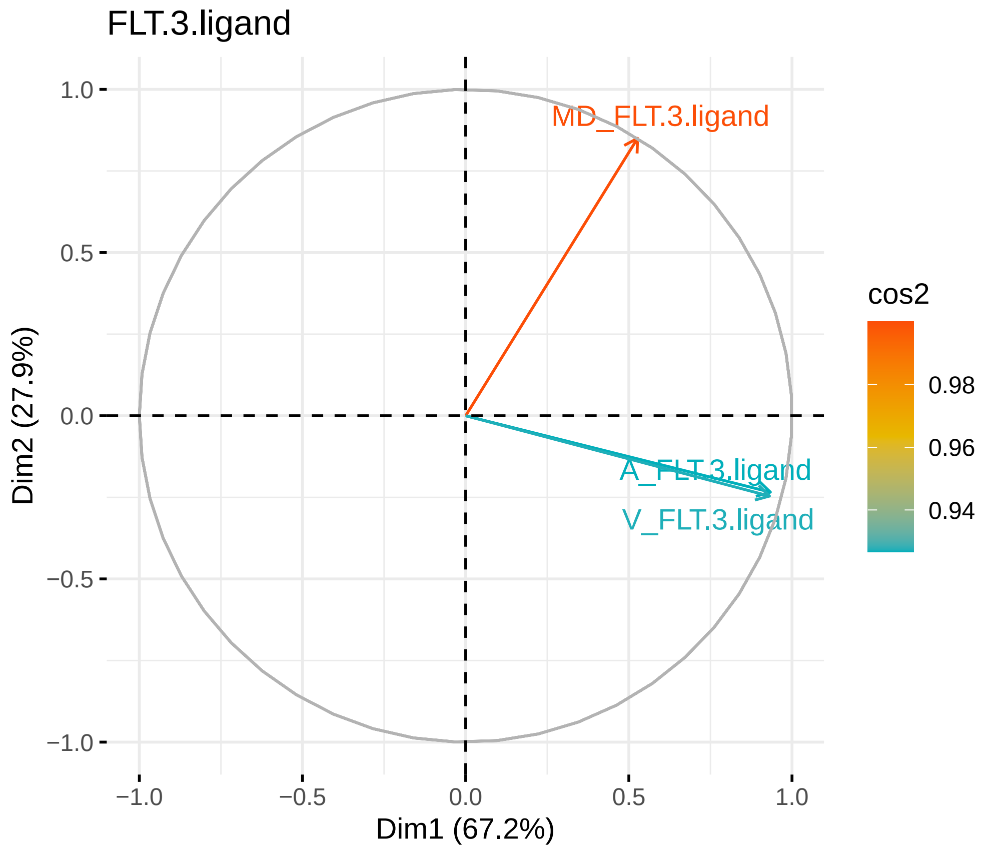
FLT.3.ligand
MD_FLT.3.ligand
A_FLT.3.ligand
V_FLT.3.ligand
cos2
0.98
0.96
0.94
1.0
0.5
0.0
−0.5
−1.0
Dim2 (27.9%)
−1.0
−0.5
0.0
0.5
1.0
Dim1 (67.2%)

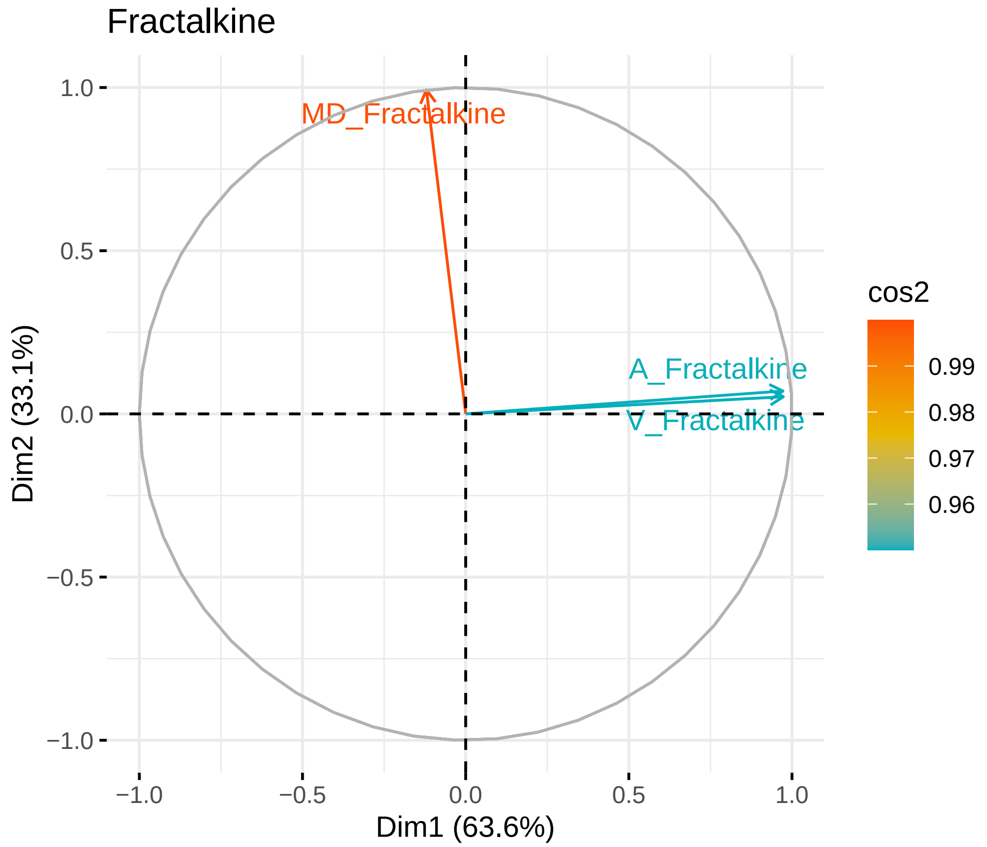
Fractalkine
MD_Fractalkine
A_Fractalkine
V_Fractalkine
cos2
0.99
0.98
0.97
0.96
1.0
0.5
0.0
−0.5
−1.0
Dim2 (33.1%)
−1.0
−0.5
0.0
0.5
1.0
Dim1 (63.6%)

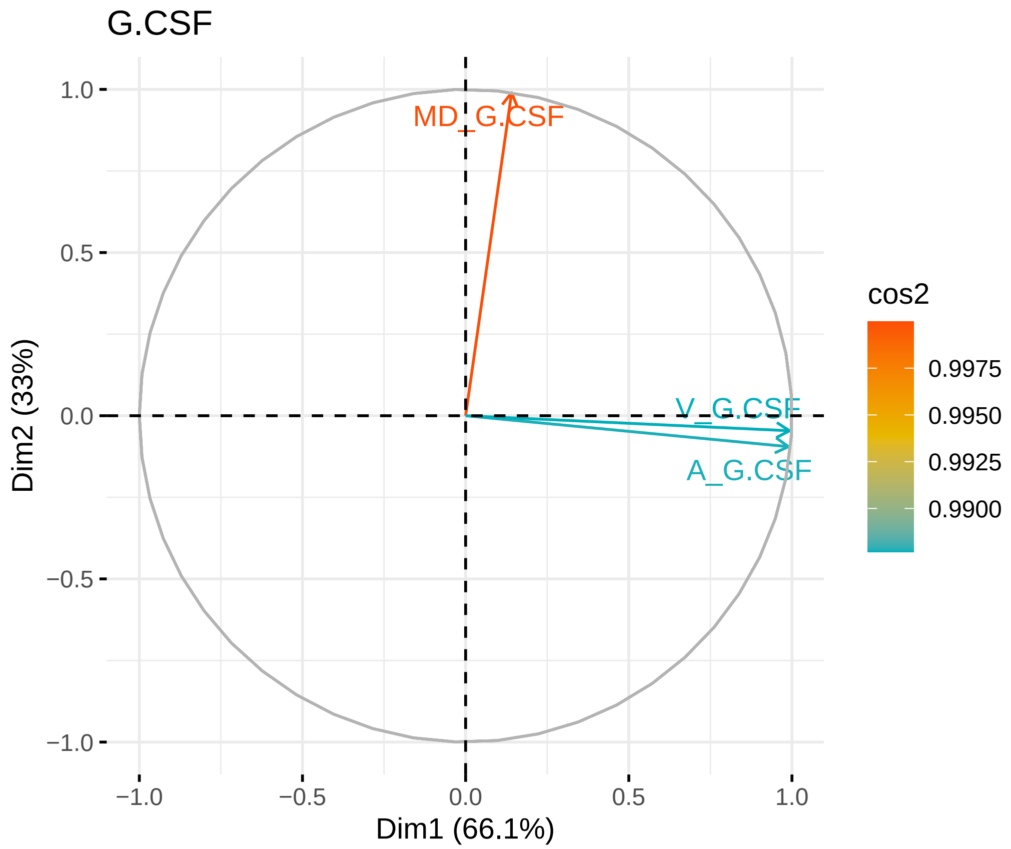
G.CSF
MD_G.CSF
V_G.CSF
A_G.CSF
Dim1 (66.1%)
Dim2 (33%)
cos2
0.9975
0.9950
0.9925
0.9900

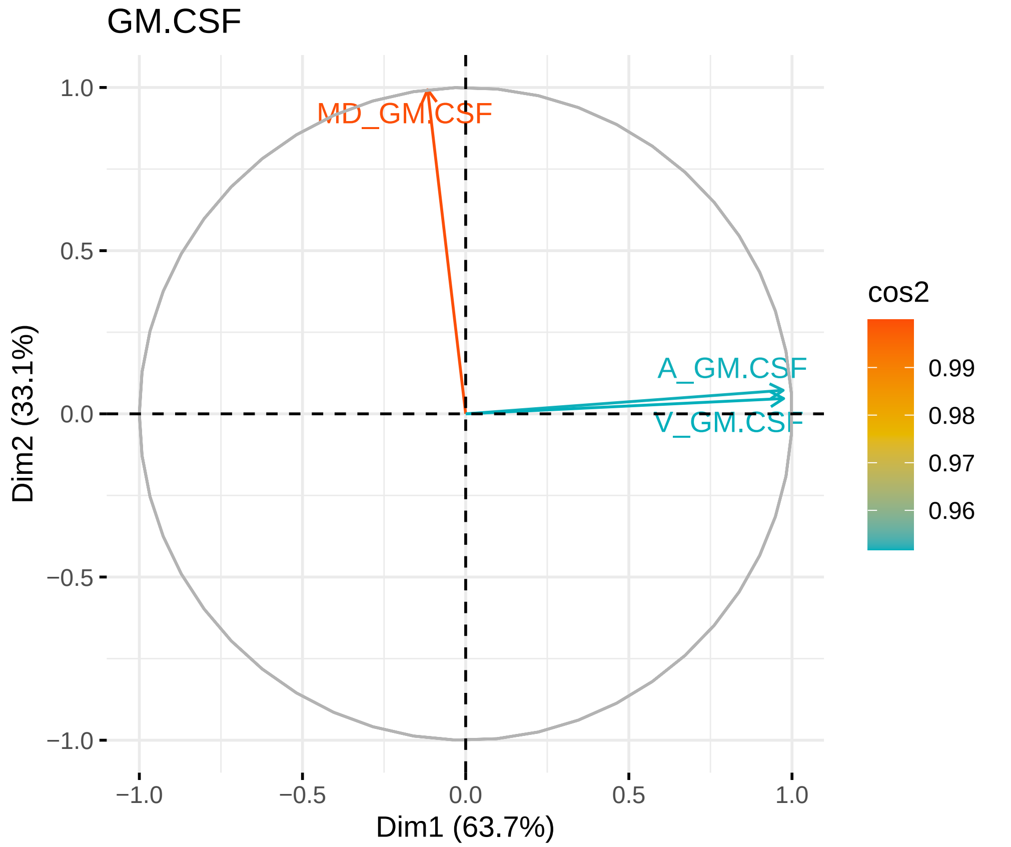
GM.CSF
MD_GM.CSF
A_GM.CSF
V_GM.CSF
Dim1 (63.7%)
Dim2 (33.1%)
cos2
0.99
0.98
0.97
0.96

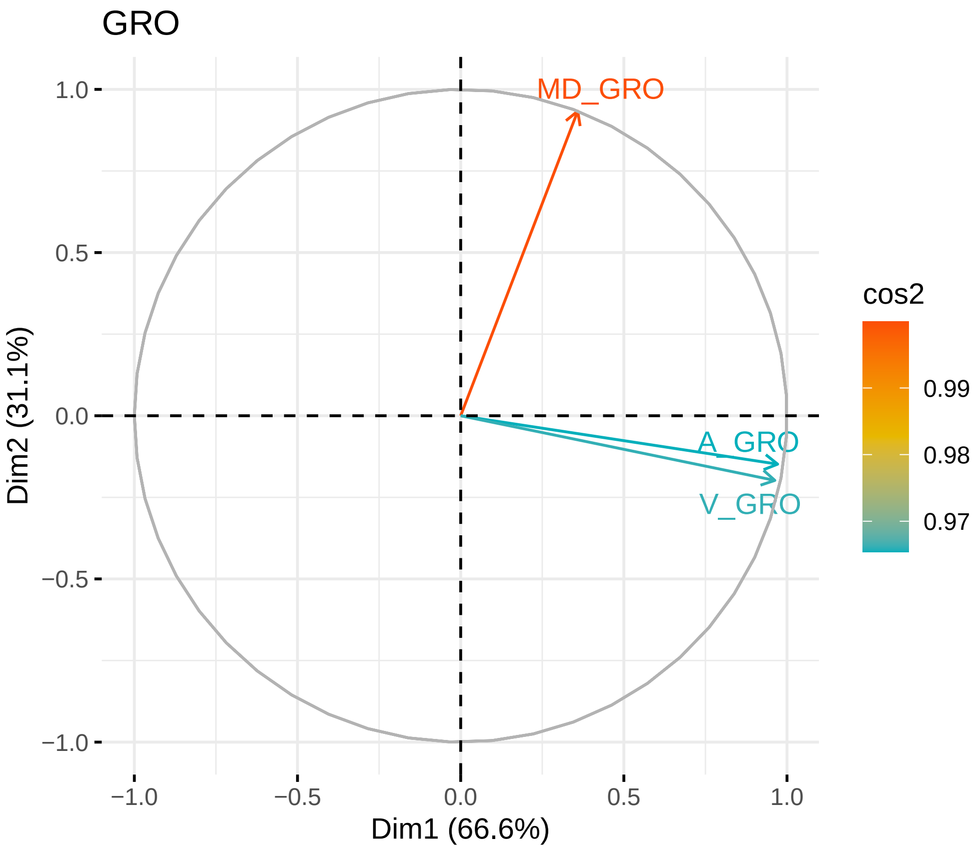
GRO
MD_GRO
A_GRO
V_GRO
Dim1 (66.6%)
Dim2 (31.1%)
cos2
0.99
0.98
0.97

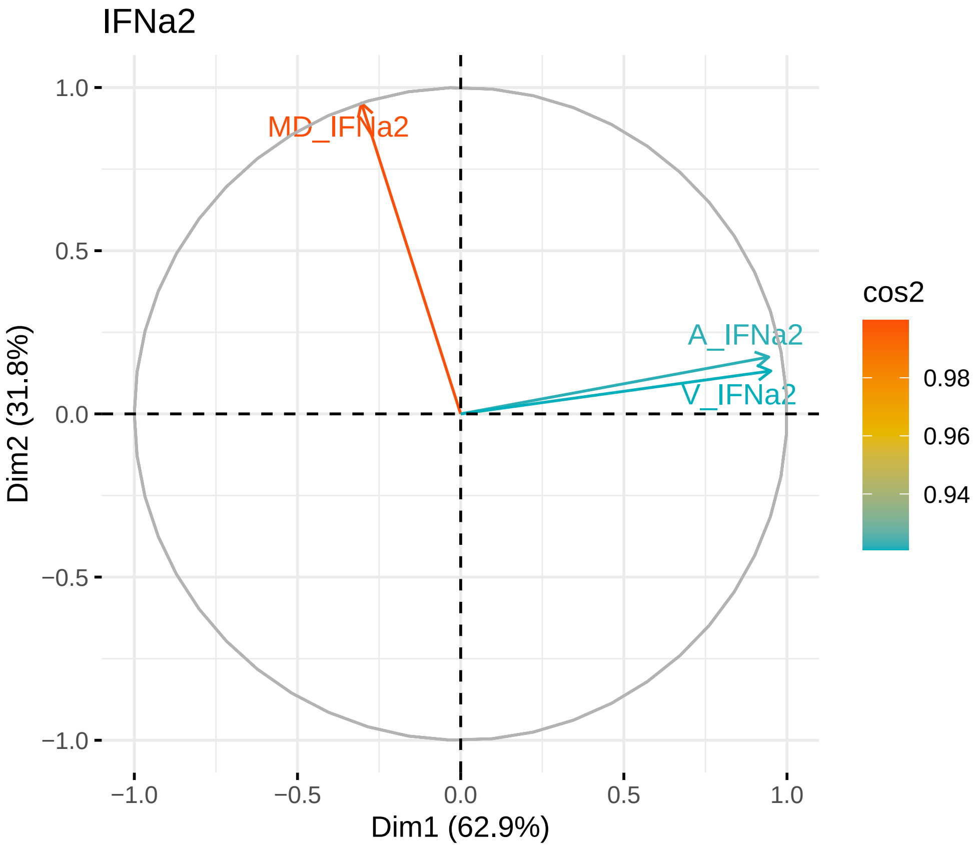
IFNa2
MD_IFNa2
A_IFNa2
V_IFNa2
Dim1 (62.9%)
Dim2 (31.8%)
cos2
0.98
0.96
0.94

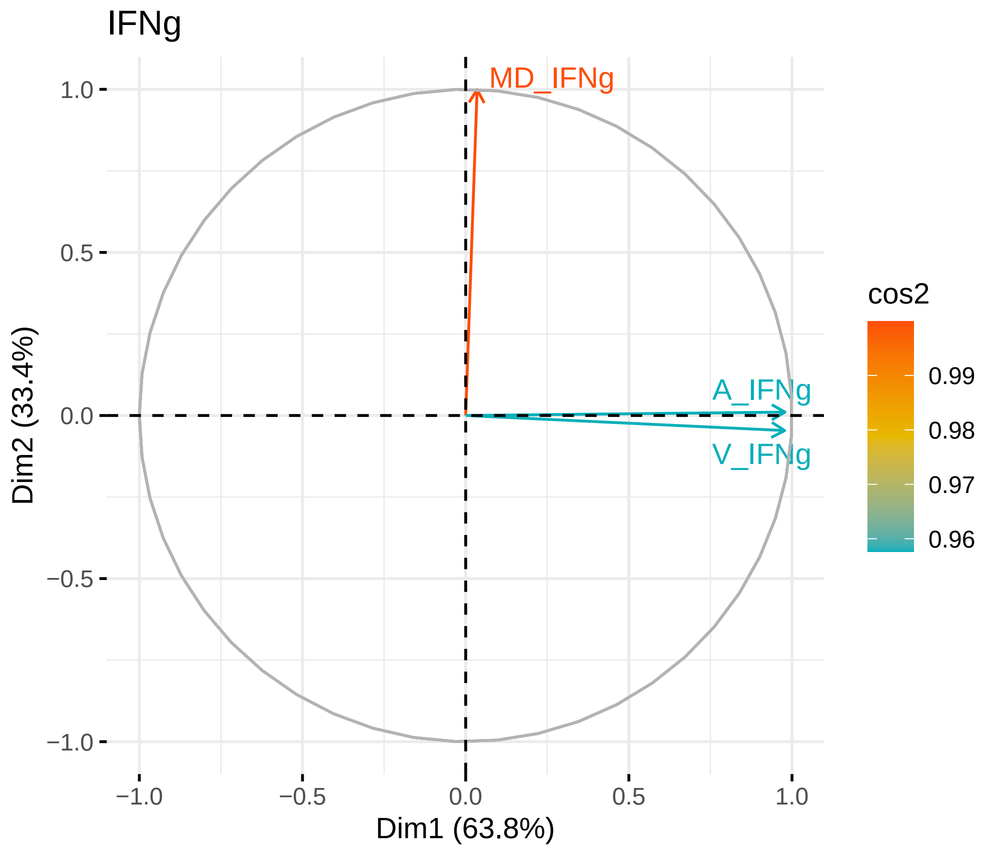

IFNg
MD_IFNg
A_IFNg
V_IFNg
cos2
0.99
0.98
0.97
0.96
1.0
0.5
0.0
−0.5
−1.0
Dim2 (33.4%)
−1.0
−0.5
0.0
0.5
1.0
Dim1 (63.8%)

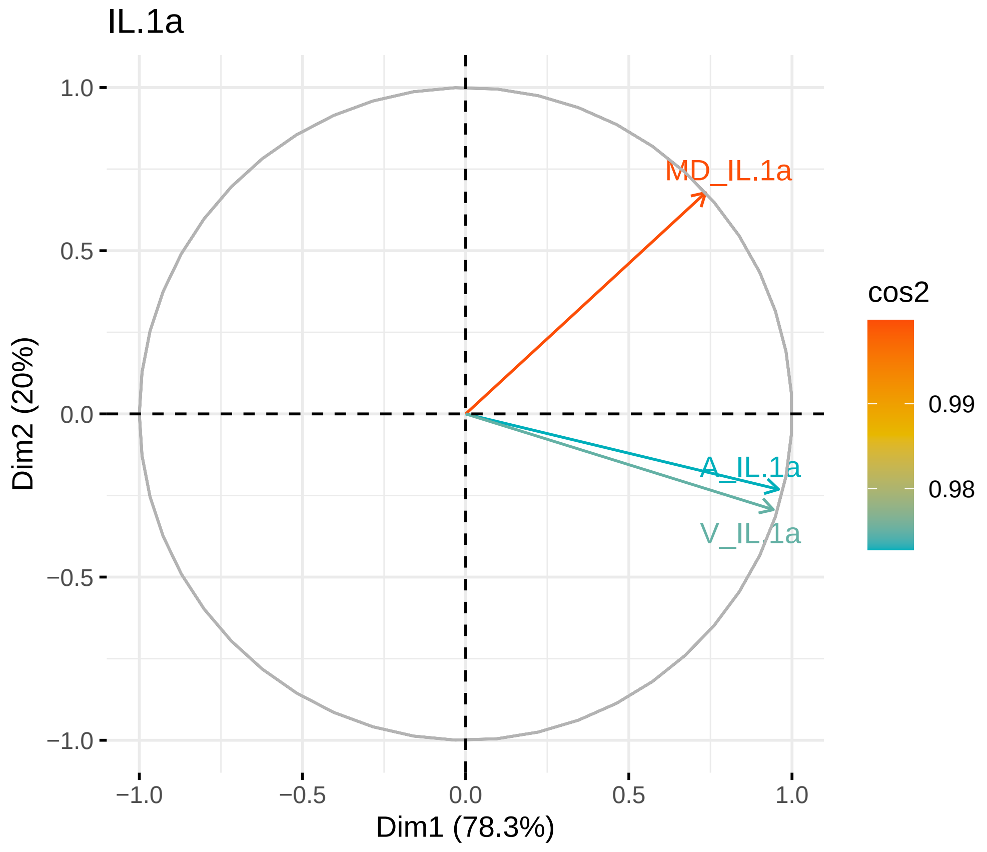

IL.1a
MD_IL.1a
A_IL.1a
V_IL.1a
cos2
0.99
0.98
1.0
0.5
0.0
−0.5
−1.0
Dim2 (20%)
−1.0
−0.5
0.0
0.5
1.0
Dim1 (78.3%)

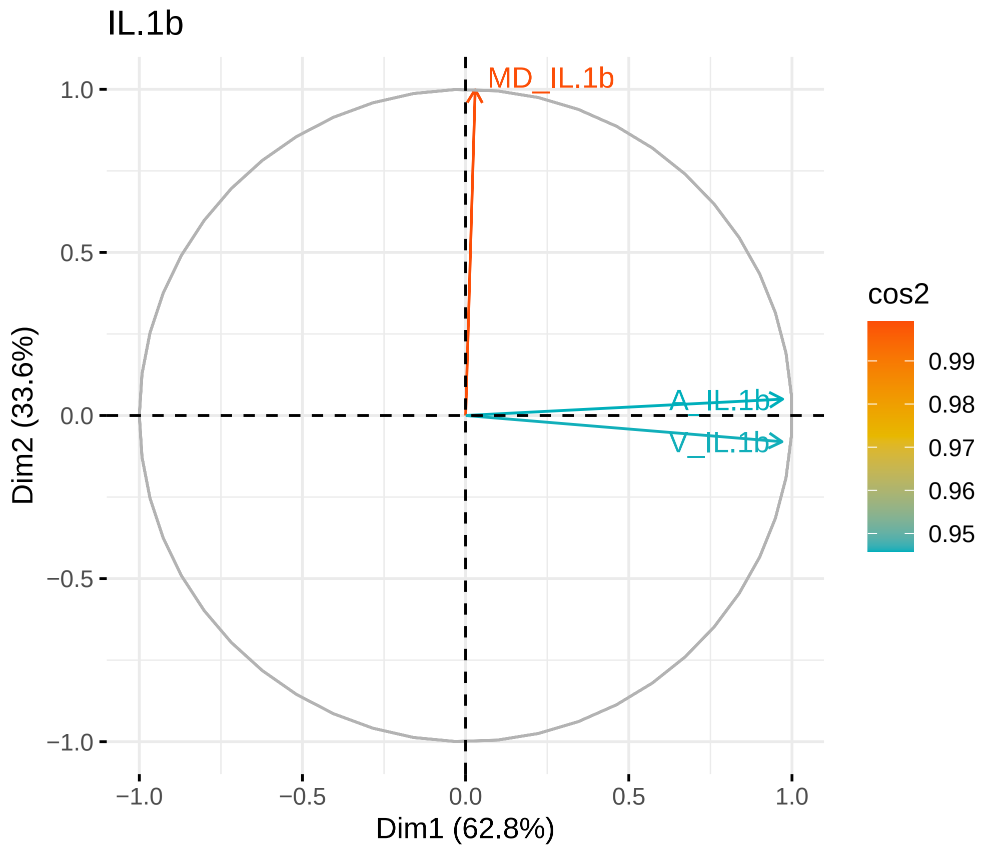
IL.1b
MD_IL.1b
A_IL.1b
V_IL.1b
cos2
0.99
0.98
0.97
0.96
0.95
1.0
0.5
0.0
−0.5
−1.0
Dim2 (33.6%)
Dim1 (62.8%)

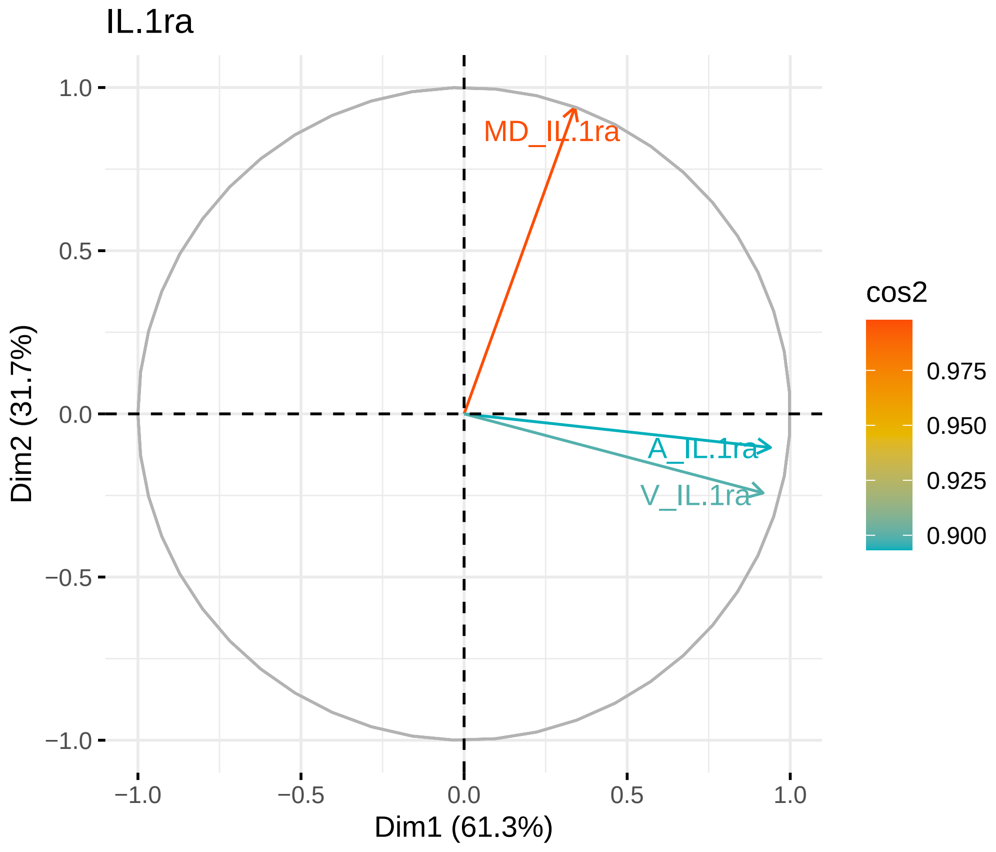
IL.1ra
MD_IL.1ra
A_IL.1ra
V_IL.1ra
cos2
0.975
0.950
0.925
0.900
1.0
0.5
0.0
−0.5
−1.0
Dim2 (31.7%)
Dim1 (61.3%)

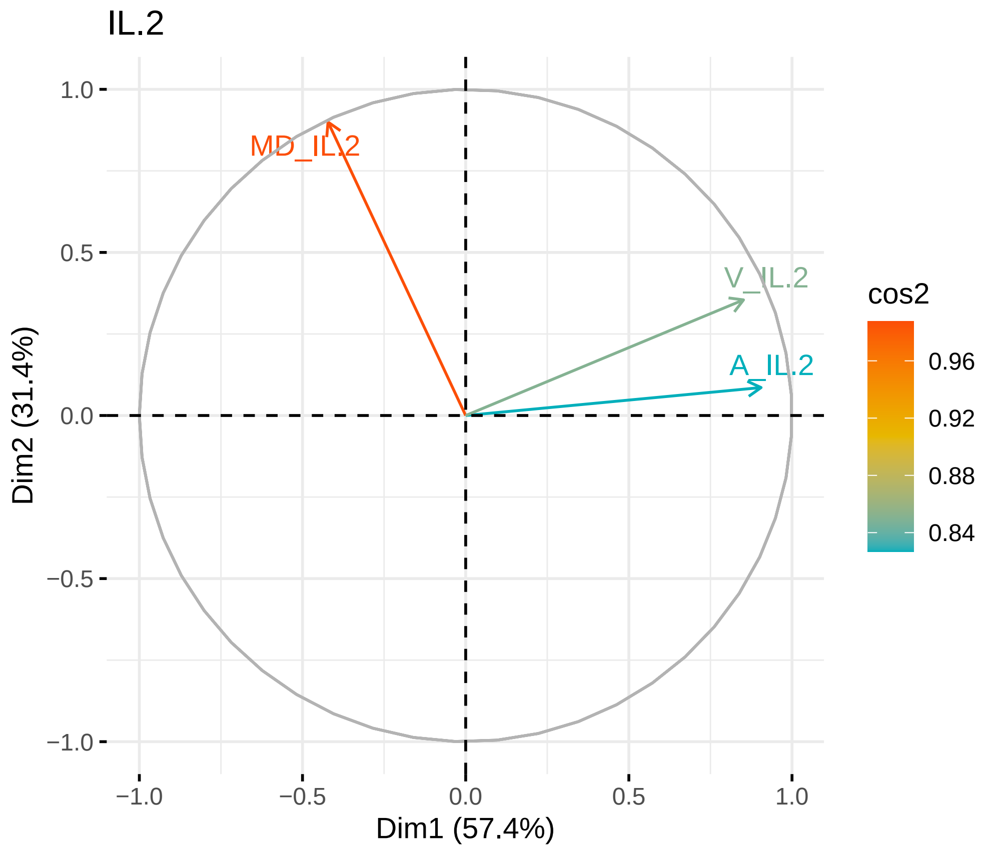
IL.2
MD_IL.2
V_IL.2
A_IL.2
cos2
0.96
0.92
0.88
0.84
1.0
0.5
0.0
−0.5
−1.0
Dim2 (31.4%)
−1.0
−0.5
0.0
0.5
1.0
Dim1 (57.4%)

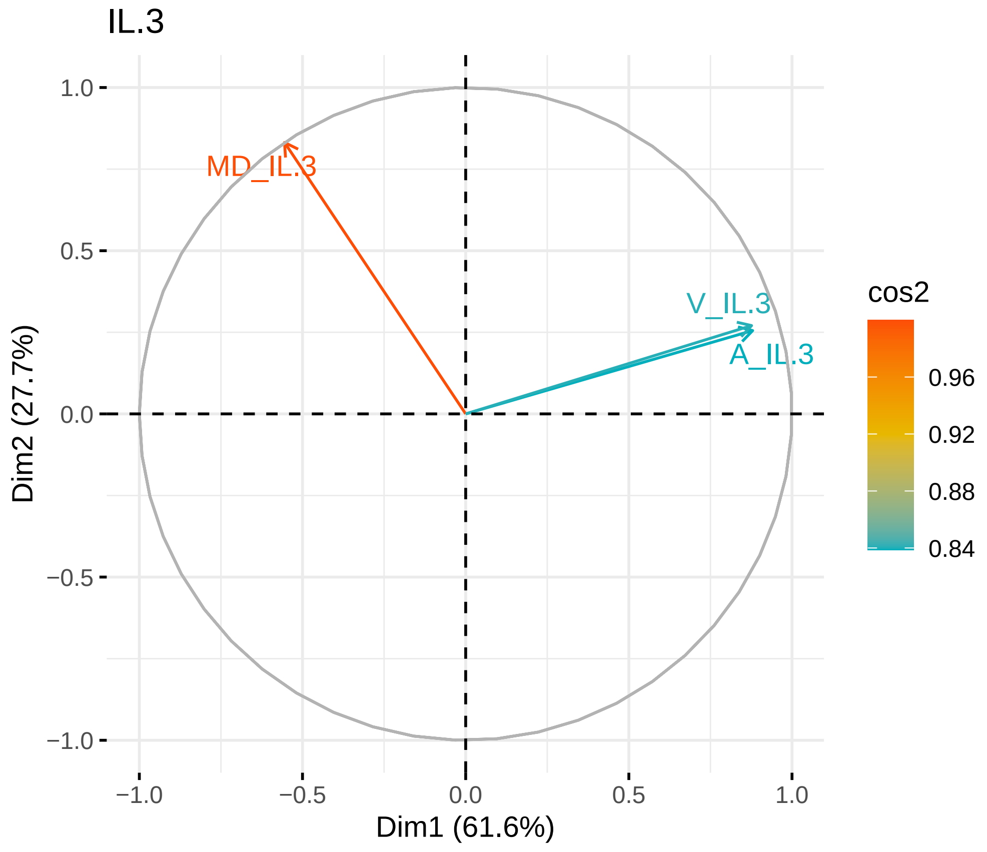
IL.3
MD_IL.3
V_IL.3
A_IL.3
cos2
0.96
0.92
0.88
0.84
1.0
0.5
0.0
−0.5
−1.0
Dim2 (27.7%)
−1.0
−0.5
0.0
0.5
1.0
Dim1 (61.6%)

```
## [1] "Error in IL.4"
```

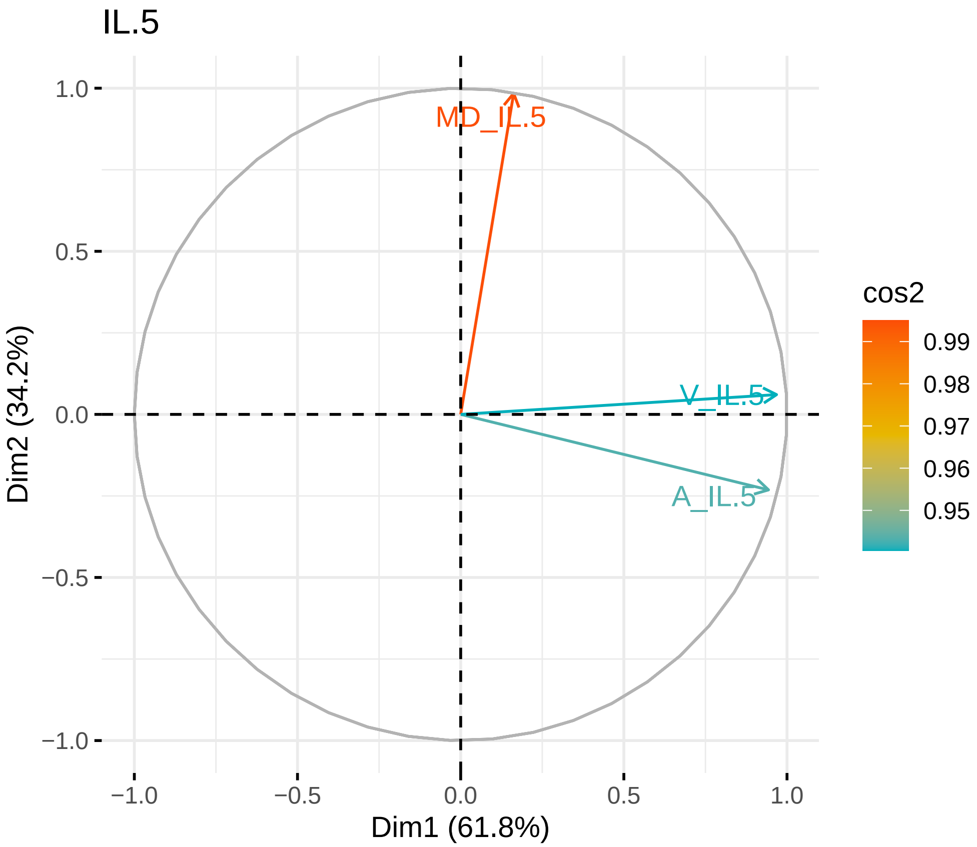

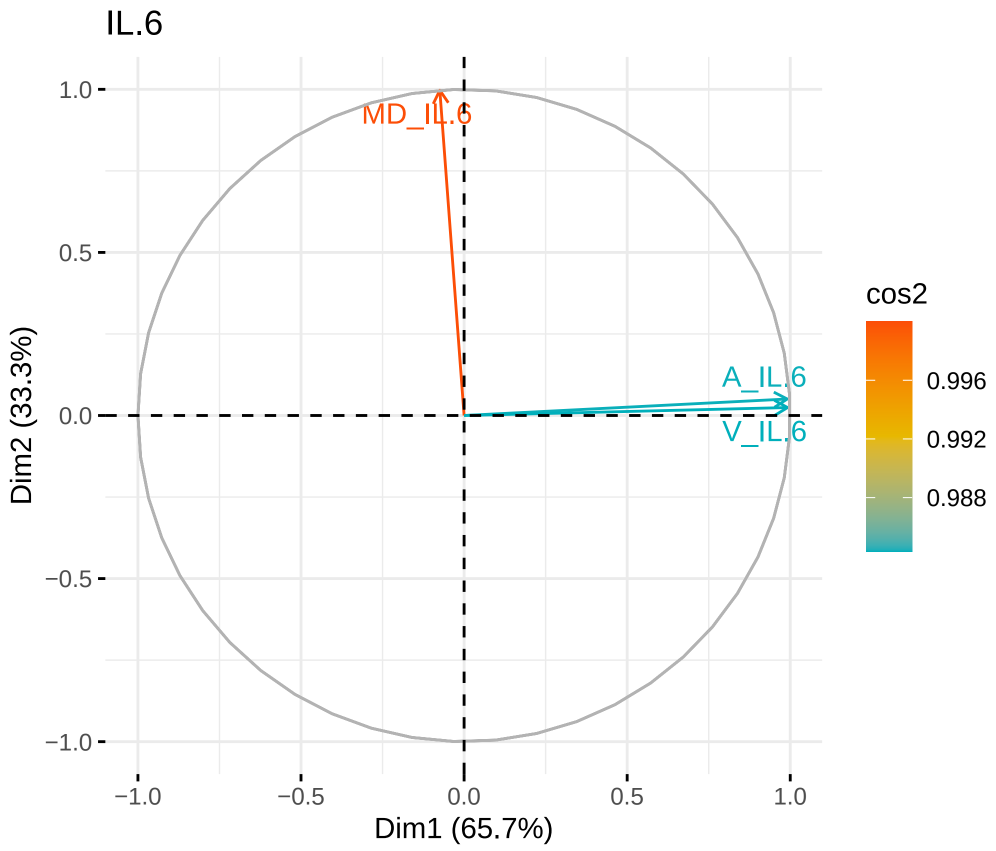
IL.6
MD_IL.6
A_IL.6
V_IL.6
cos2
0.996
0.992
0.988
Dim2 (33.3%)
Dim1 (65.7%)
1.0
0.5
0.0
−0.5
−1.0

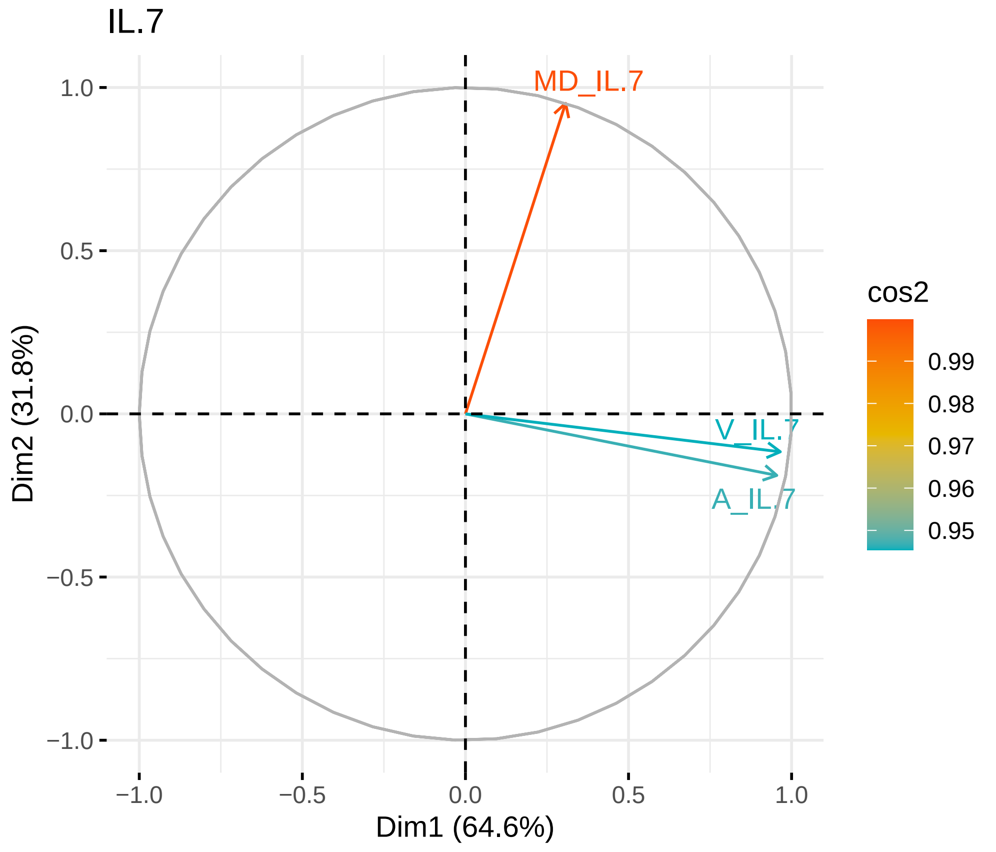
IL.7
MD_IL.7
V_IL.7
A_IL.7
cos2
0.99
0.98
0.97
0.96
0.95
Dim2 (31.8%)
Dim1 (64.6%)
1.0
0.5
0.0
−0.5
−1.0

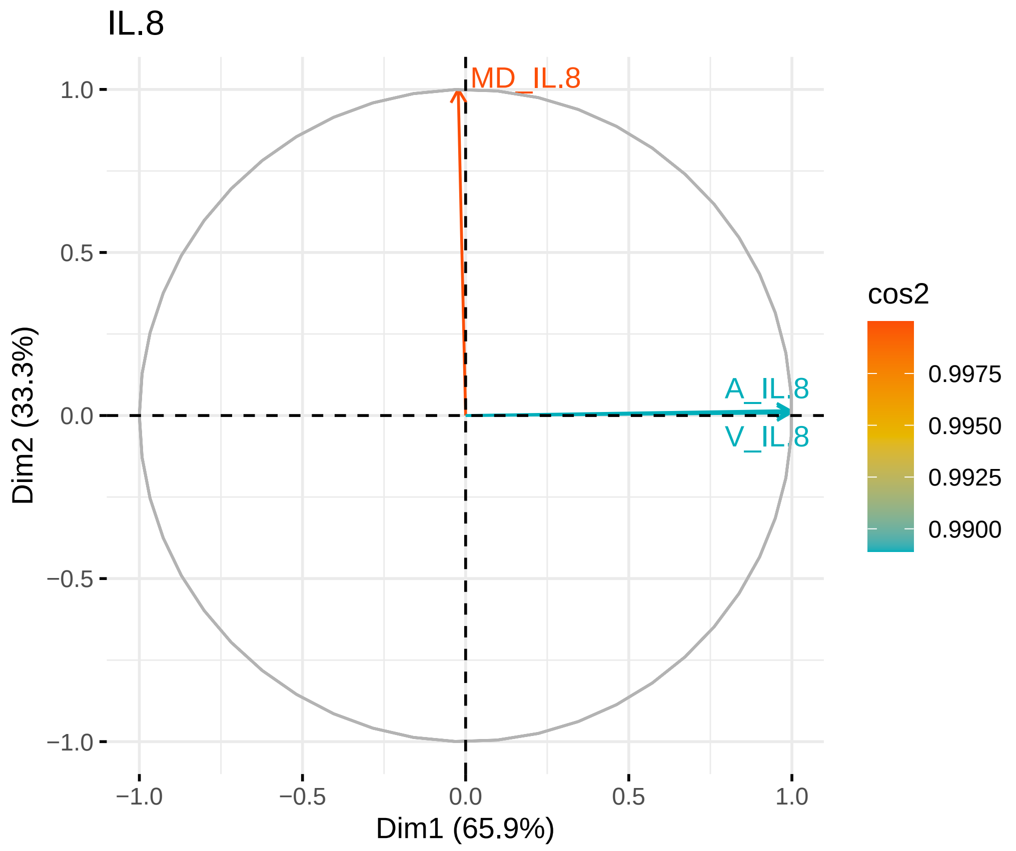
IL.8
MD_IL.8
A_IL.8
V_IL.8
Dim2 (33.3%)
Dim1 (65.9%)
1.0
0.5
0.0
−0.5
−1.0
−1.0
−0.5
0.0
0.5
1.0
cos2
0.9975
0.9950
0.9925
0.9900

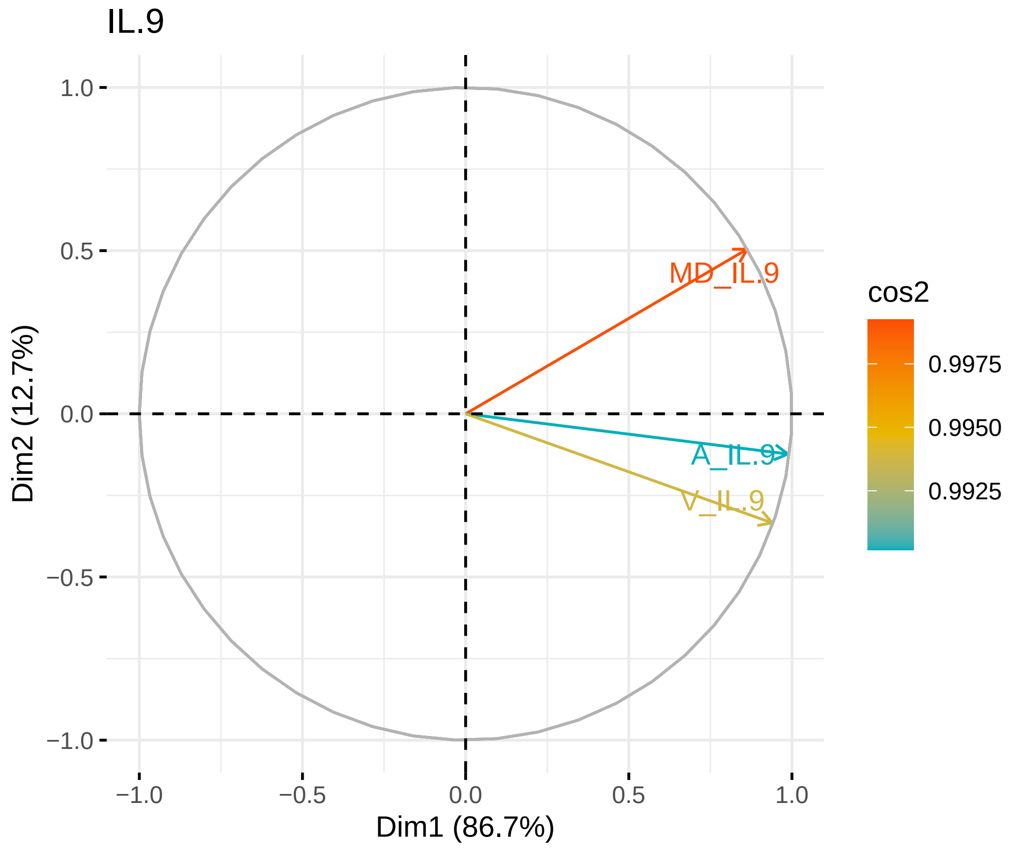
IL.9
MD_IL.9
A_IL.9
V_IL.9
Dim2 (12.7%)
Dim1 (86.7%)
1.0
0.5
0.0
−0.5
−1.0
−1.0
−0.5
0.0
0.5
1.0
cos2
0.9975
0.9950
0.9925

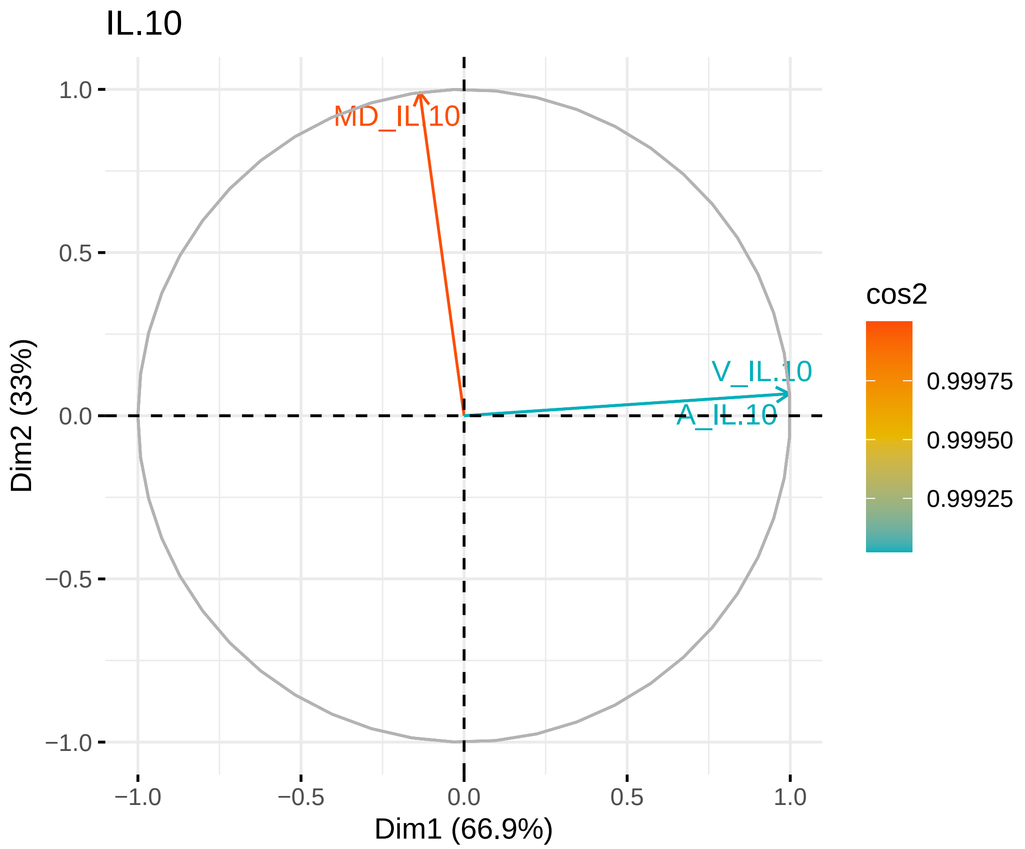
IL.10
MD_IL.10
V_IL.10
A_IL.10
Dim1 (66.9%)
Dim2 (33%)
cos2
0.99975
0.99950
0.99925
1.0
0.5
0.0
−0.5
−1.0

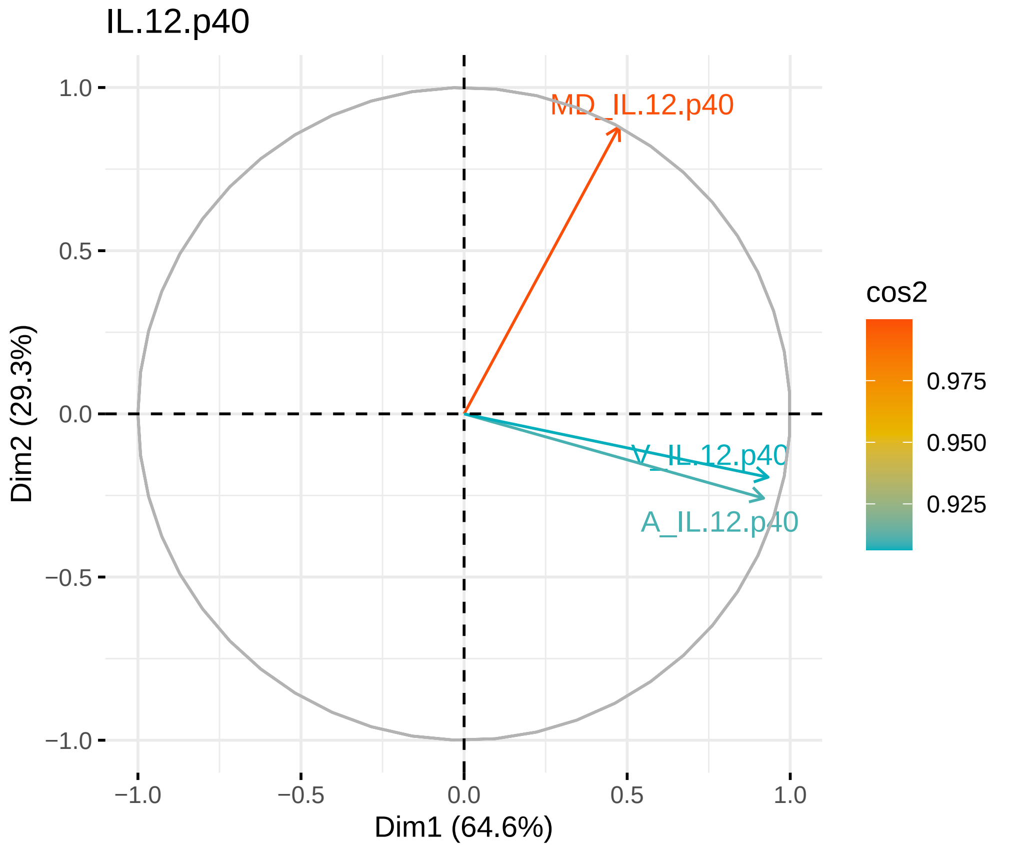
IL.12.p40
MD_IL.12.p40
V_IL.12.p40
A_IL.12.p40
Dim1 (64.6%)
Dim2 (29.3%)
cos2
0.975
0.950
0.925
1.0
0.5
0.0
−0.5
−1.0

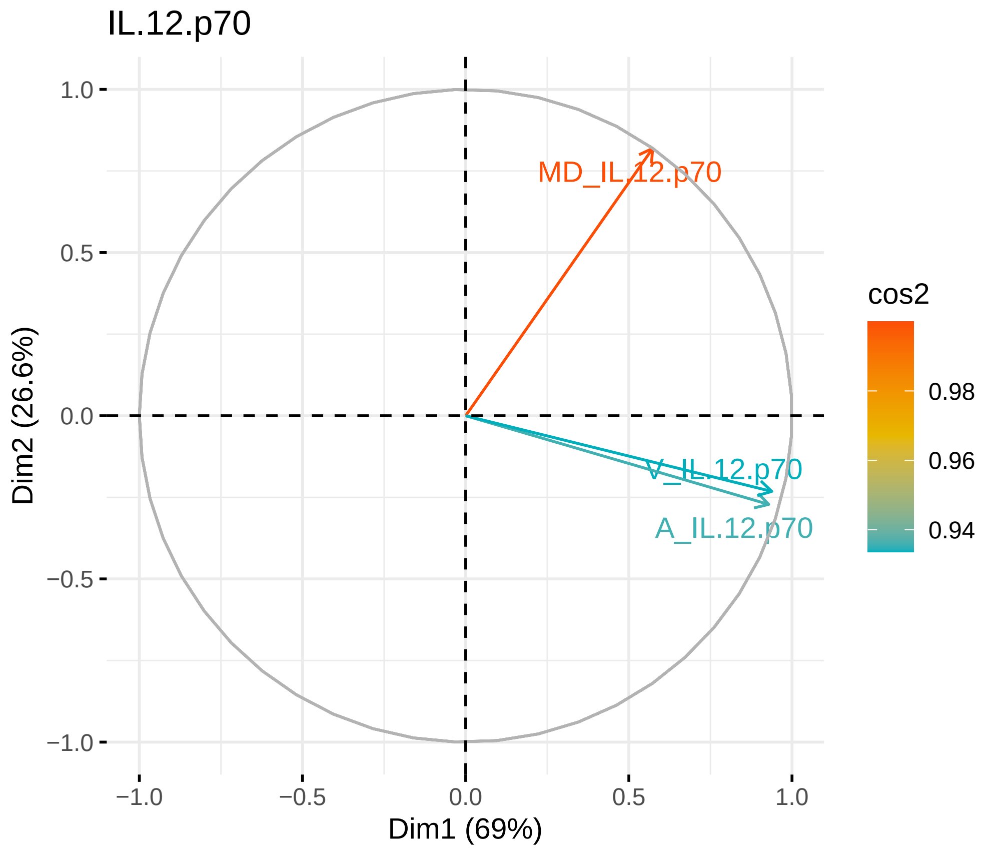
IL.12.p70
MD_IL.12.p70
V_IL.12.p70
A_IL.12.p70
cos2
0.98
0.96
0.94
1.0
0.5
0.0
−0.5
−1.0
Dim2 (26.6%)
−1.0
−0.5
0.0
0.5
1.0
Dim1 (69%)

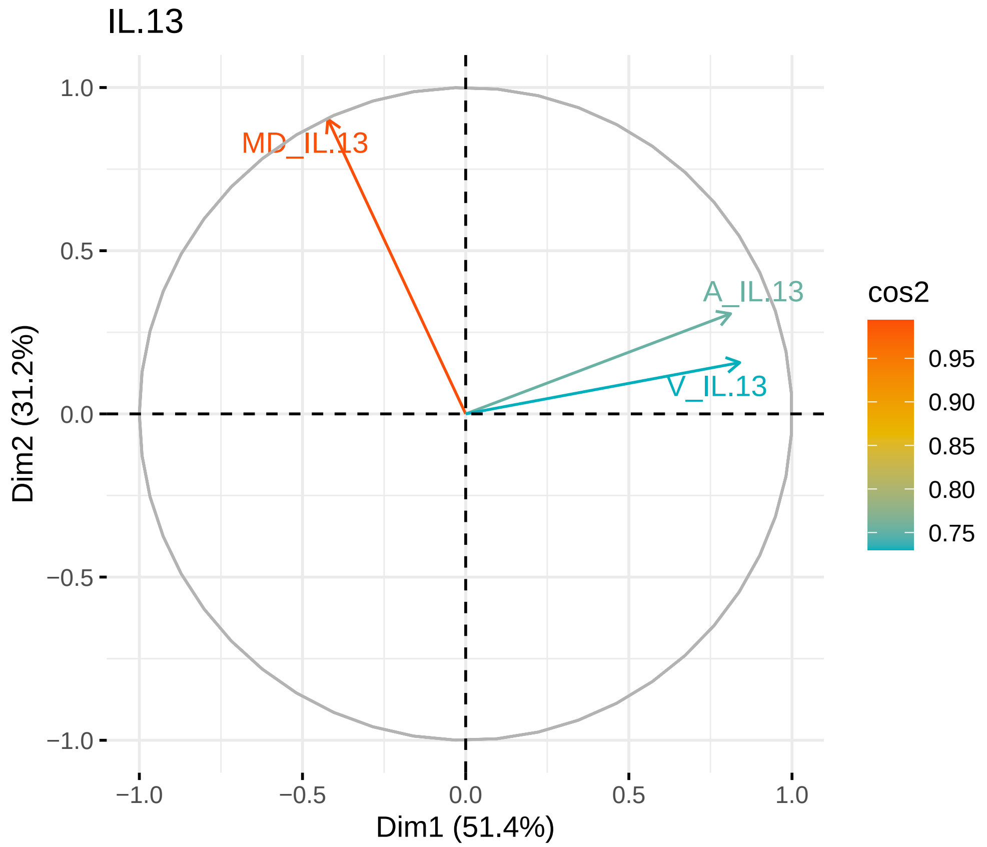
IL.13
MD_IL.13
A_IL.13
V_IL.13
cos2
0.95
0.90
0.85
0.80
0.75
1.0
0.5
0.0
−0.5
−1.0
Dim2 (31.2%)
−1.0
−0.5
0.0
0.5
1.0
Dim1 (51.4%)

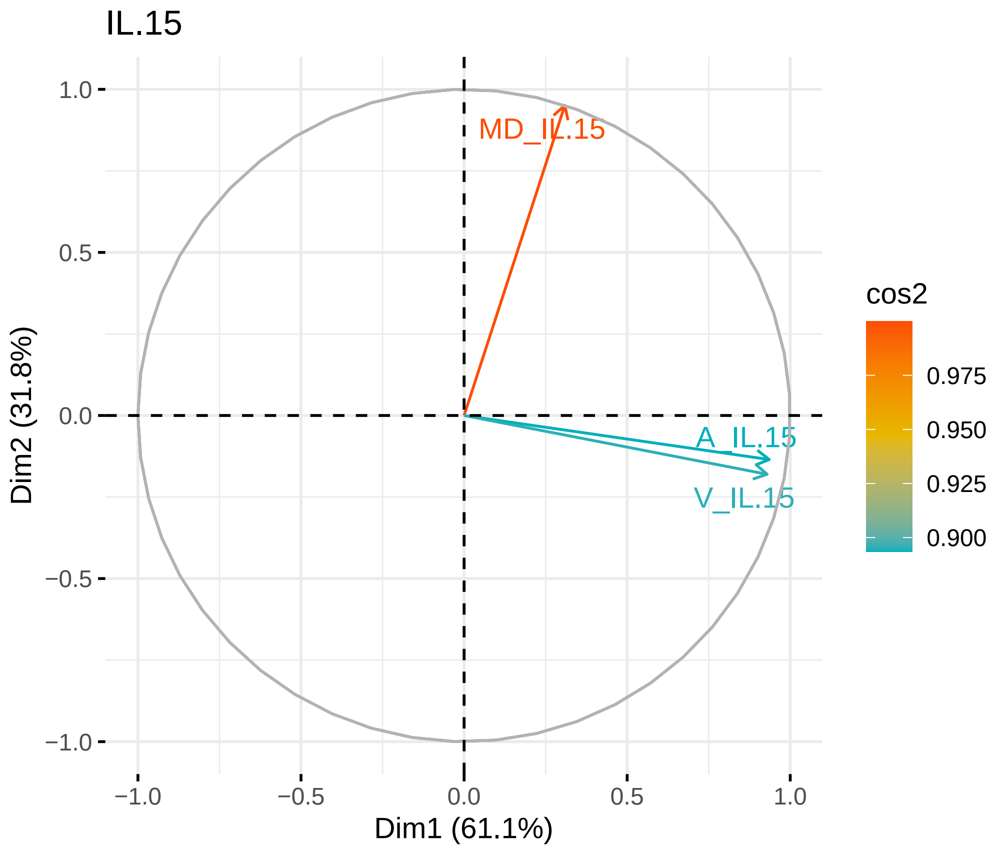
IL.15
MD_IL.15
A_IL.15
V_IL.15
1.0
0.5
0.0
−0.5
−1.0
−1.0
−0.5
0.0
0.5
1.0
Dim1 (61.1%)
Dim2 (31.8%)
cos2
0.975
0.950
0.925
0.900

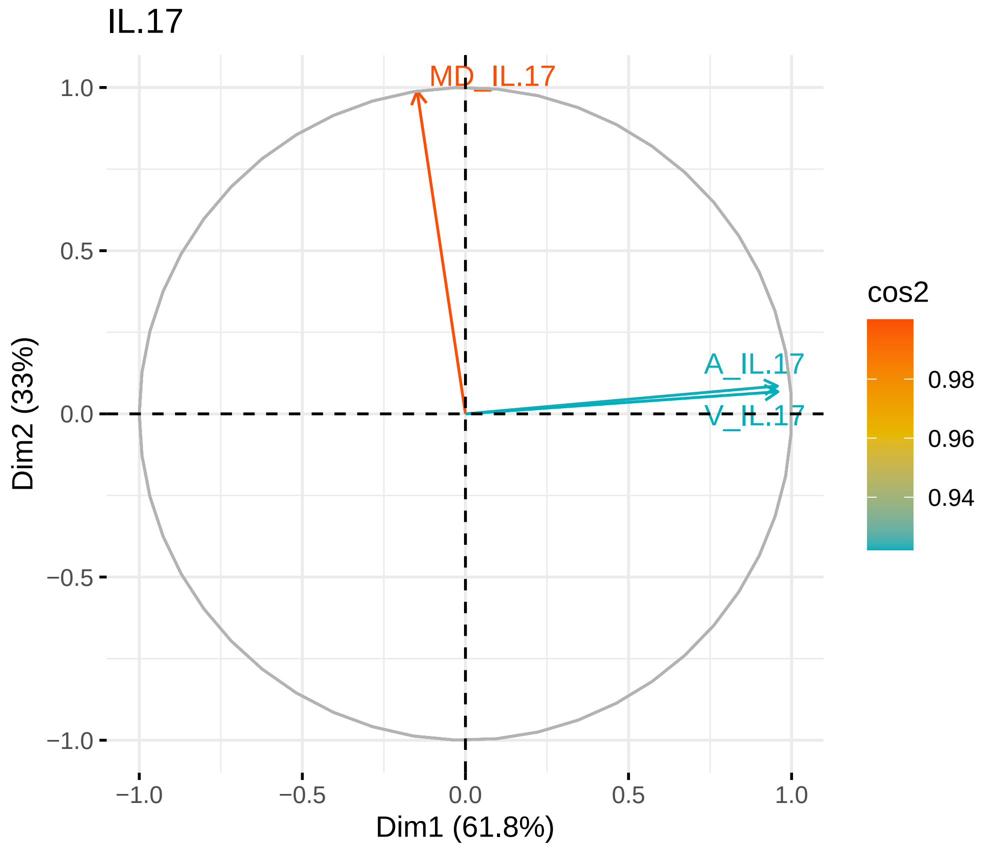
IL.17
MD_IL.17
A_IL.17
V_IL.17
1.0
0.5
0.0
−0.5
−1.0
−1.0
−0.5
0.0
0.5
1.0
Dim1 (61.8%)
Dim2 (33%)
cos2
0.98
0.96
0.94

IP.10

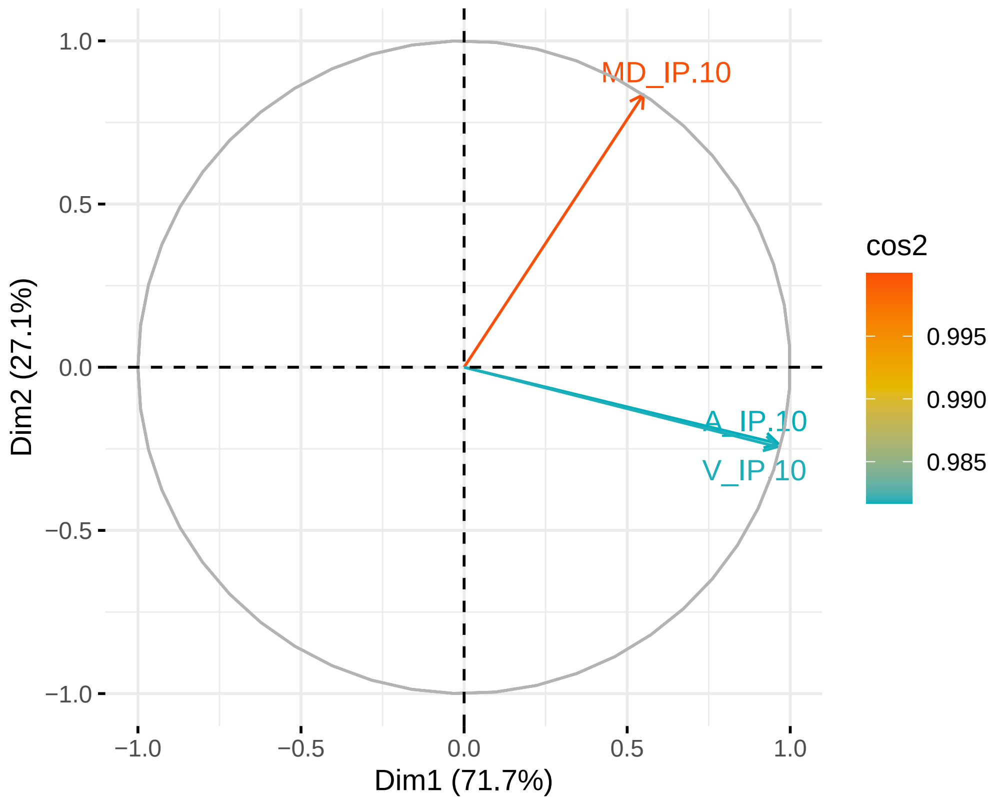
MD_IP.10
A_IP.10
V_IP.10
cos2
0.995
0.990
0.985
1.0
0.5
0.0
−0.5
−1.0
Dim2 (27.1%)
Dim1 (71.7%)

MCP.1

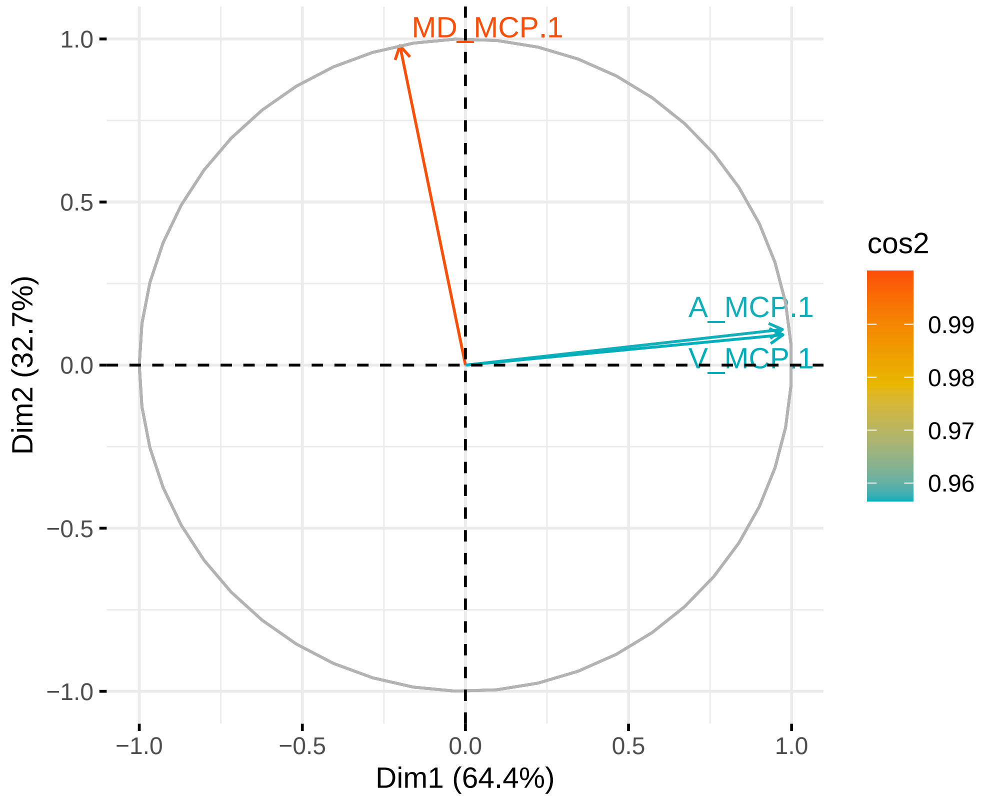
MD_MCP.1
A_MCP.1
V_MCP.1
cos2
0.99
0.98
0.97
0.96
1.0
0.5
0.0
−0.5
−1.0
Dim2 (32.7%)
Dim1 (64.4%)

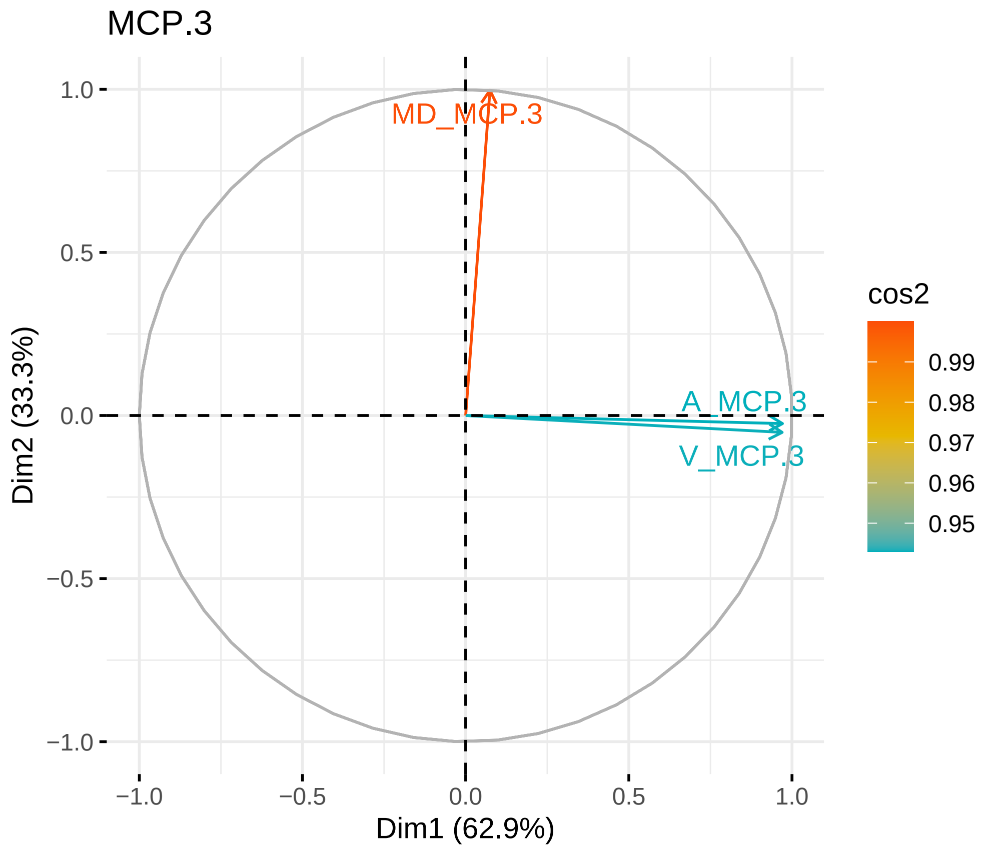
MCP.3
MD_MCP.3
A_MCP.3
V_MCP.3
Dim1 (62.9%)
Dim2 (33.3%)
cos2
0.99
0.98
0.97
0.96
0.95

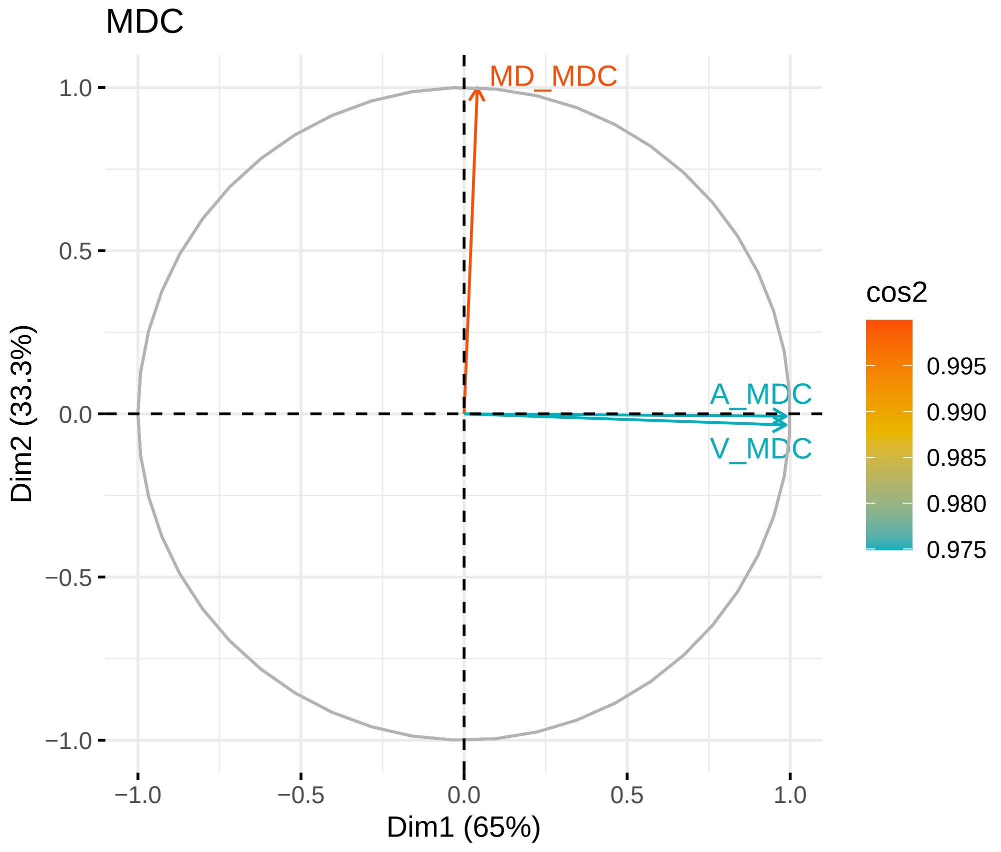
MDC
MD_MDC
A_MDC
V_MDC
Dim1 (65%)
Dim2 (33.3%)
cos2
0.995
0.990
0.985
0.980
0.975

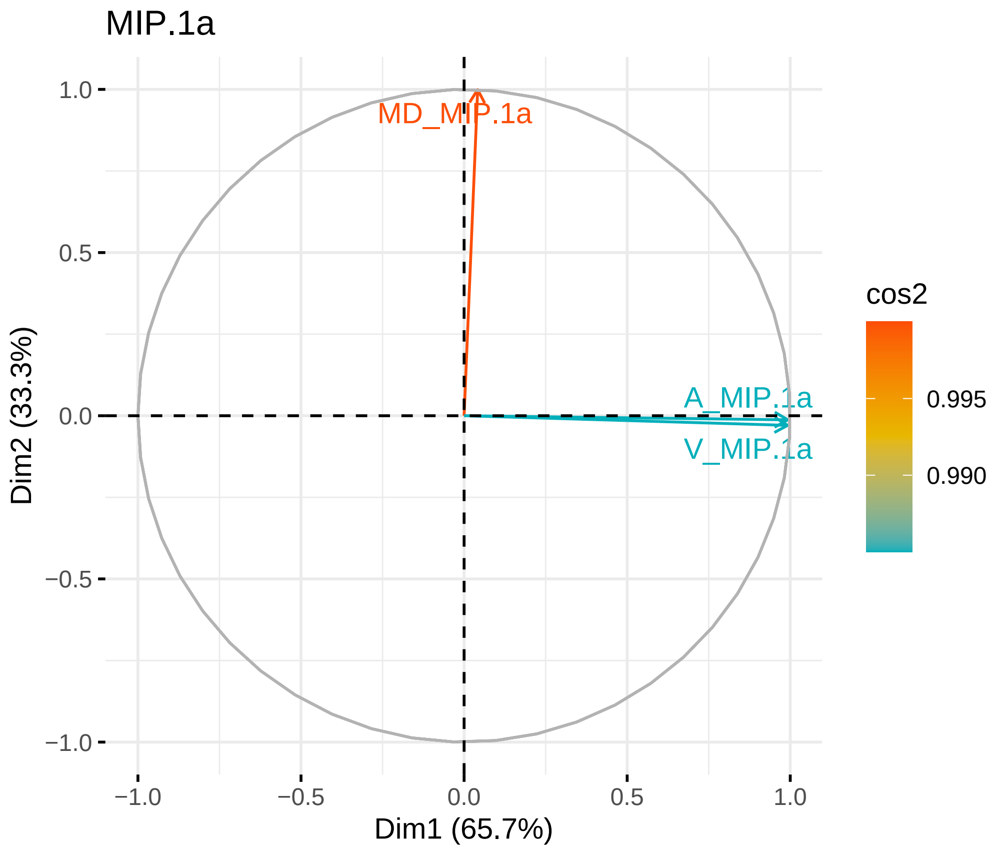
MIP.1a
MD_MIP.1a
A_MIP.1a
V_MIP.1a
cos2
0.995
0.990
1.0
0.5
0.0
−0.5
−1.0
−1.0
−0.5
0.0
0.5
1.0
Dim1 (65.7%)
Dim2 (33.3%)

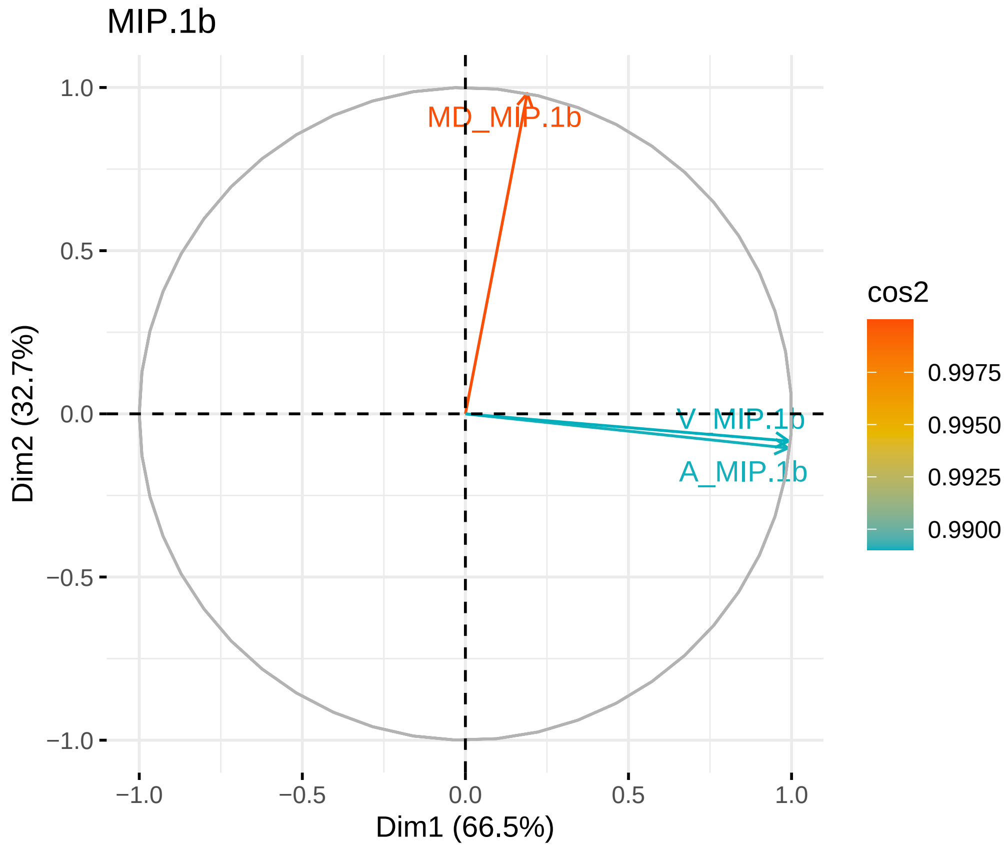
MIP.1b
MD_MIP.1b
V_MIP.1b
A_MIP.1b
cos2
0.9975
0.9950
0.9925
0.9900
1.0
0.5
0.0
−0.5
−1.0
−1.0
−0.5
0.0
0.5
1.0
Dim1 (66.5%)
Dim2 (32.7%)

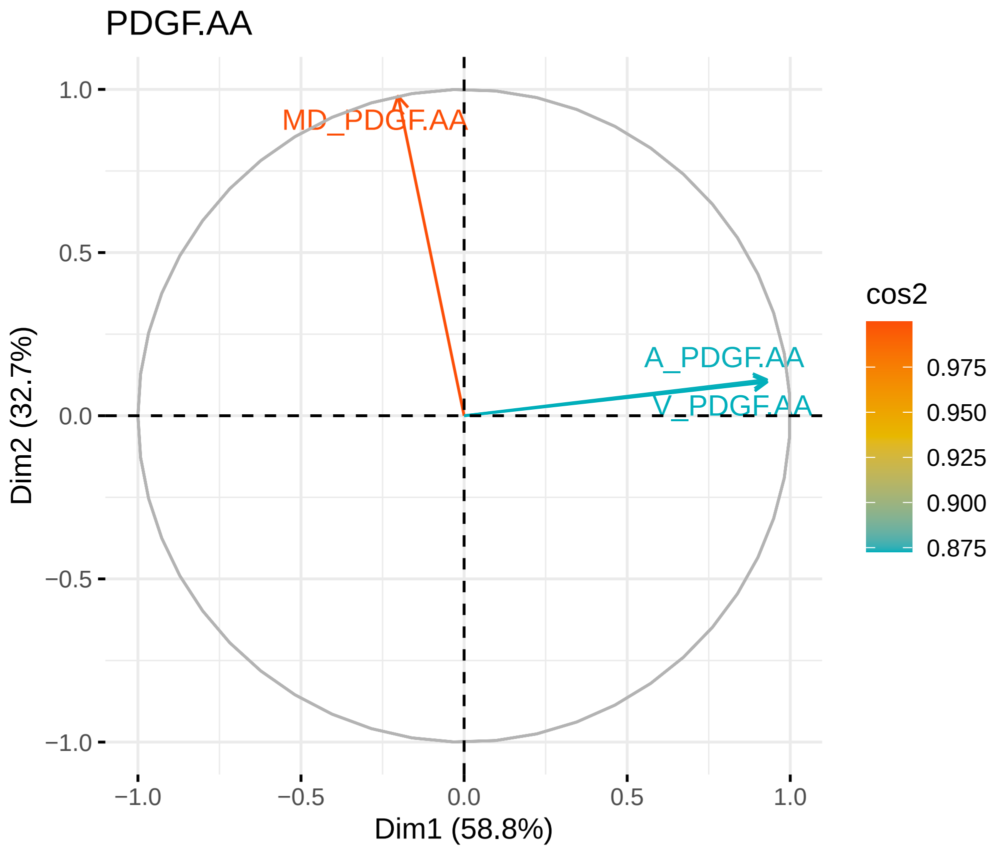
PDGF.AA
MD_PDGF.AA
A_PDGF.AA
V_PDGF.AA
Dim2 (32.7%)
Dim1 (58.8%)
1.0
0.5
0.0
−0.5
−1.0
cos2
0.975
0.950
0.925
0.900
0.875

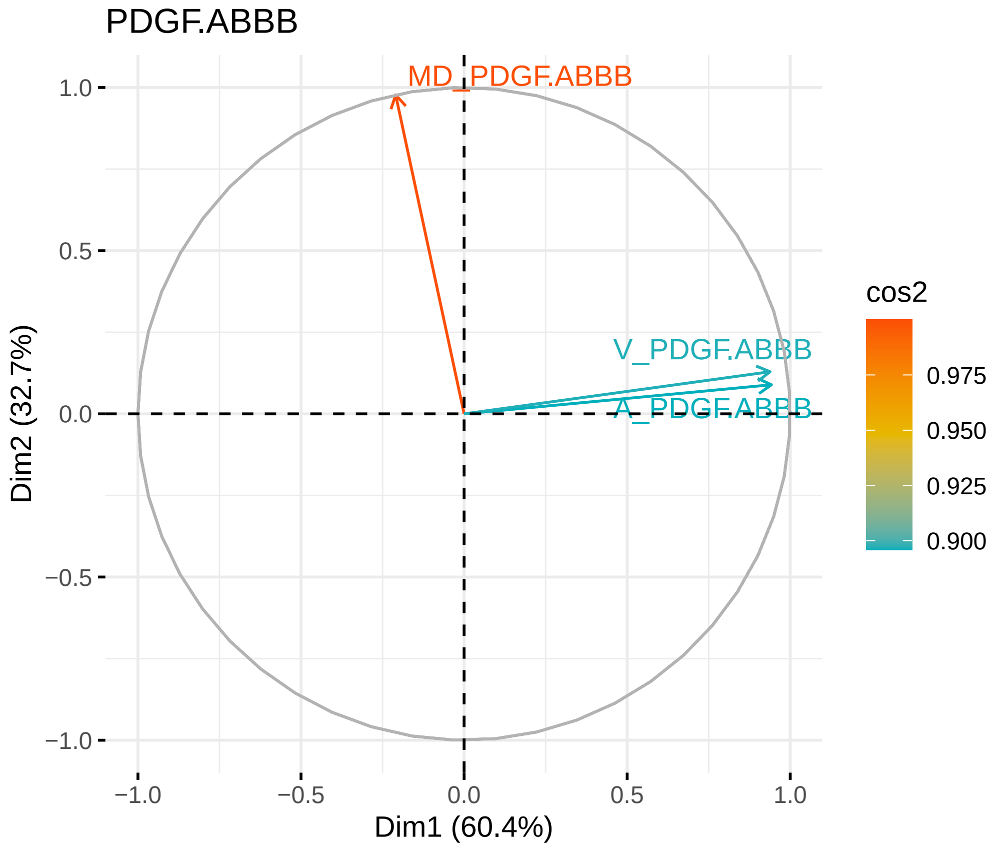
PDGF.ABBB
MD_PDGF.ABBB
V_PDGF.ABBB
A_PDGF.ABBB
Dim2 (32.7%)
Dim1 (60.4%)
1.0
0.5
0.0
−0.5
−1.0
cos2
0.975
0.950
0.925
0.900

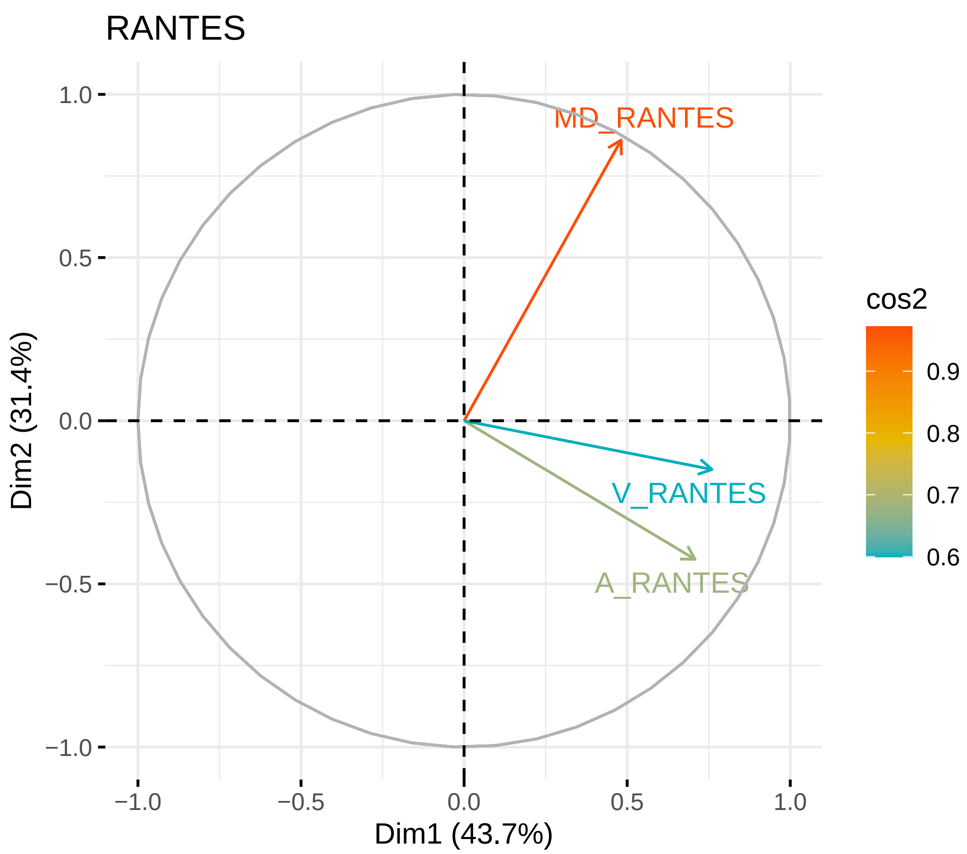
RANTES
MD_RANTES
V_RANTES
A_RANTES
cos2
0.9
0.8
0.7
0.6
1.0
0.5
0.0
−0.5
−1.0
Dim2 (31.4%)
Dim1 (43.7%)

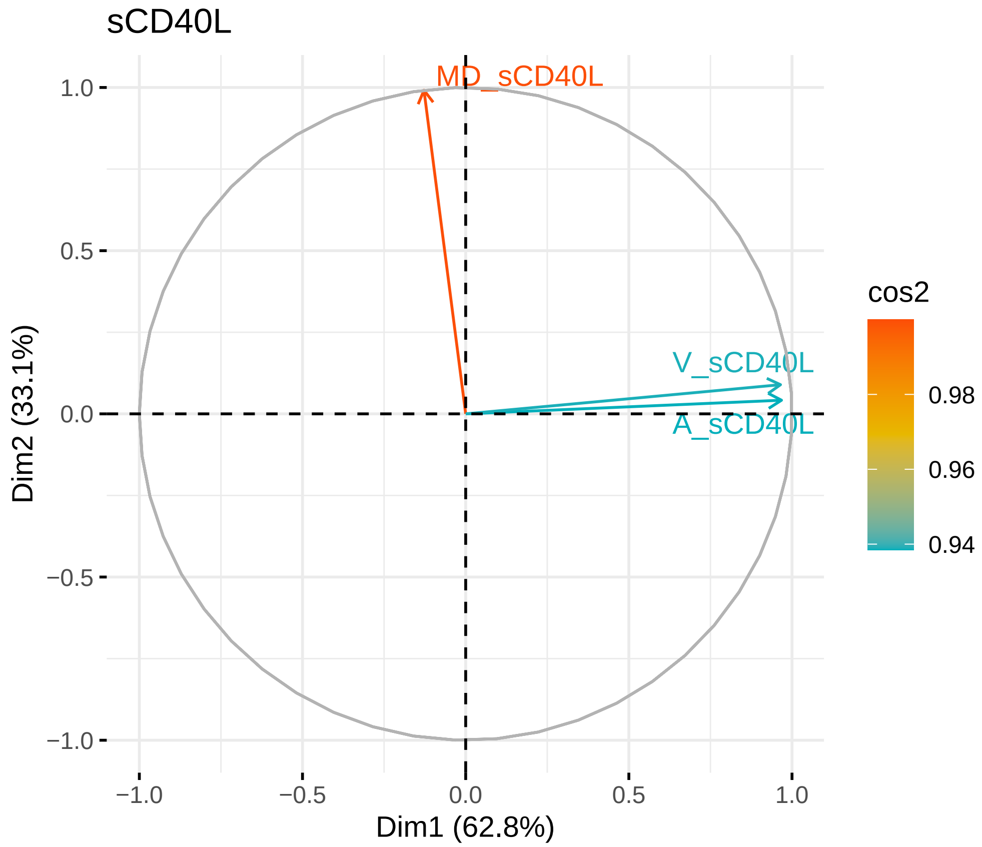
sCD40L
MD_sCD40L
V_sCD40L
A_sCD40L
cos2
0.98
0.96
0.94
1.0
0.5
0.0
−0.5
−1.0
Dim2 (33.1%)
Dim1 (62.8%)

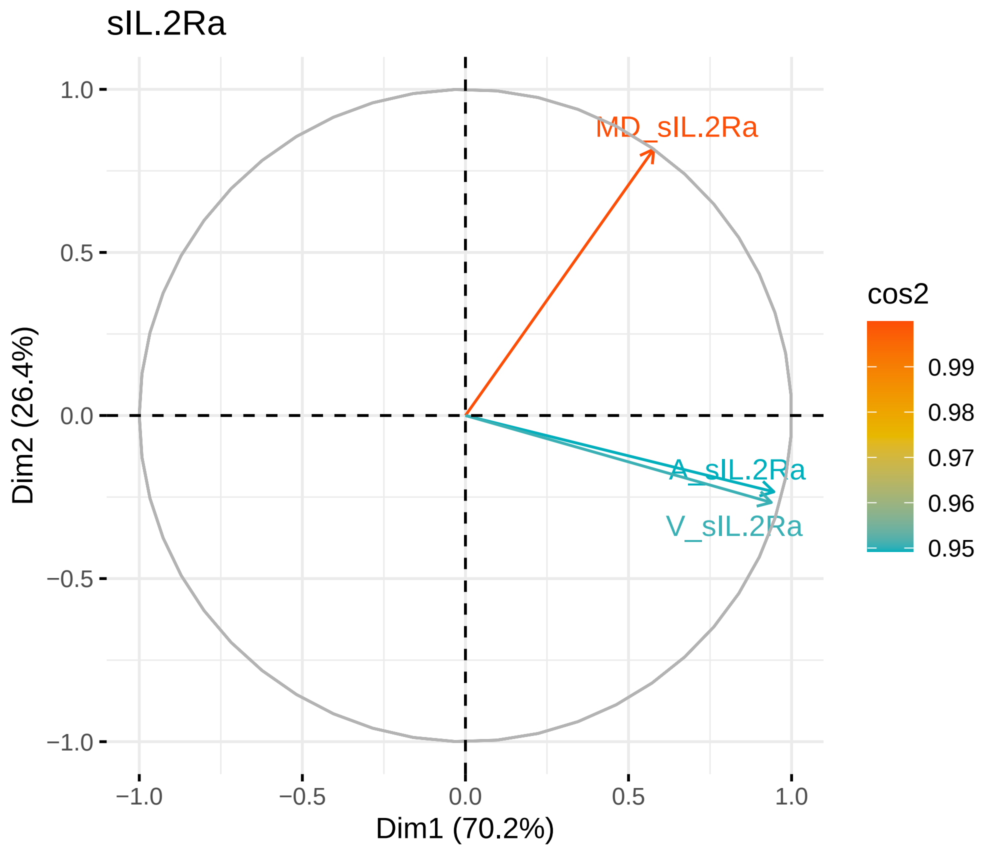
sIL.2Ra
MD_sIL.2Ra
A_sIL.2Ra
V_sIL.2Ra
Dim1 (70.2%)
Dim2 (26.4%)
cos2
0.99
0.98
0.97
0.96
0.95

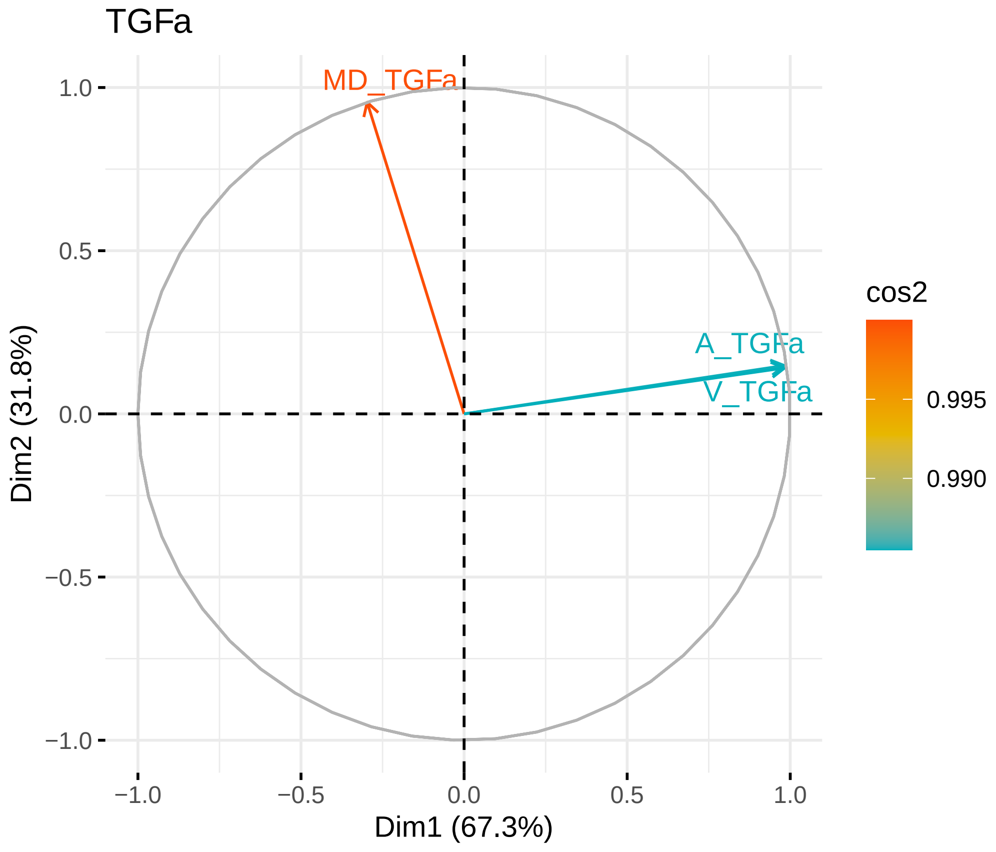
TGFa
MD_TGFa
A_TGFa
V_TGFa
Dim1 (67.3%)
Dim2 (31.8%)
cos2
0.995
0.990

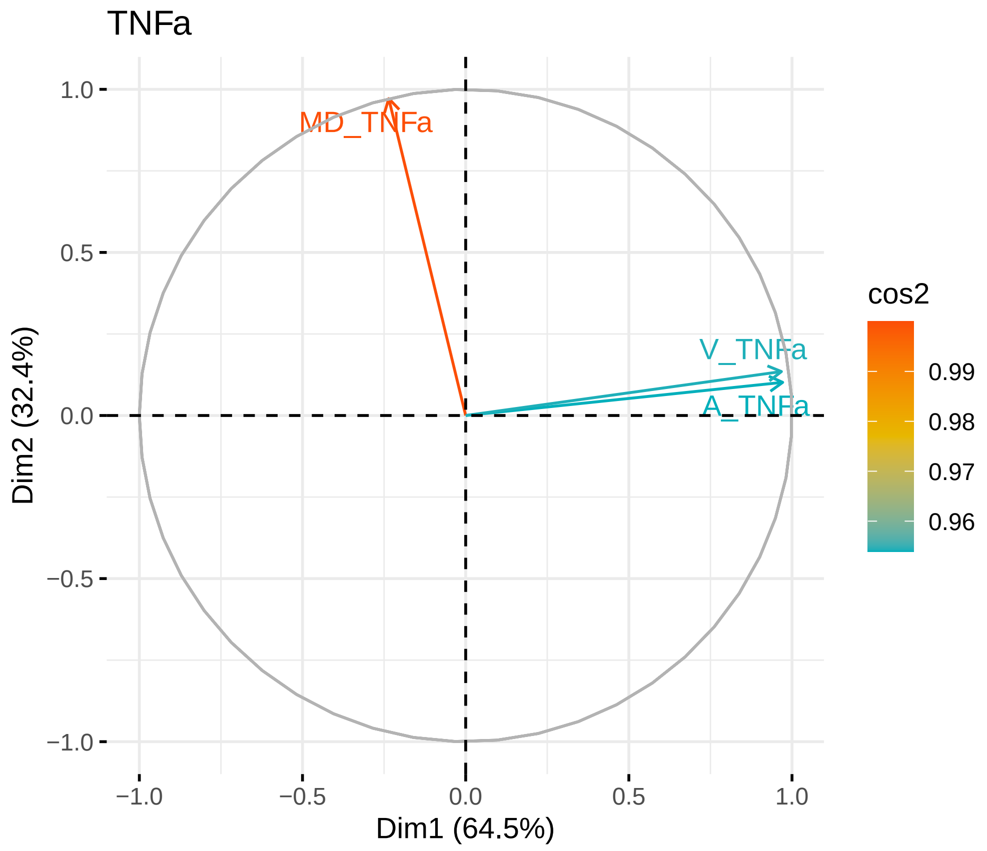
TNFa
MD_TNFa
V_TNFa
A_TNFa
Dim1 (64.5%)
Dim2 (32.4%)
cos2
0.99
0.98
0.97
0.96

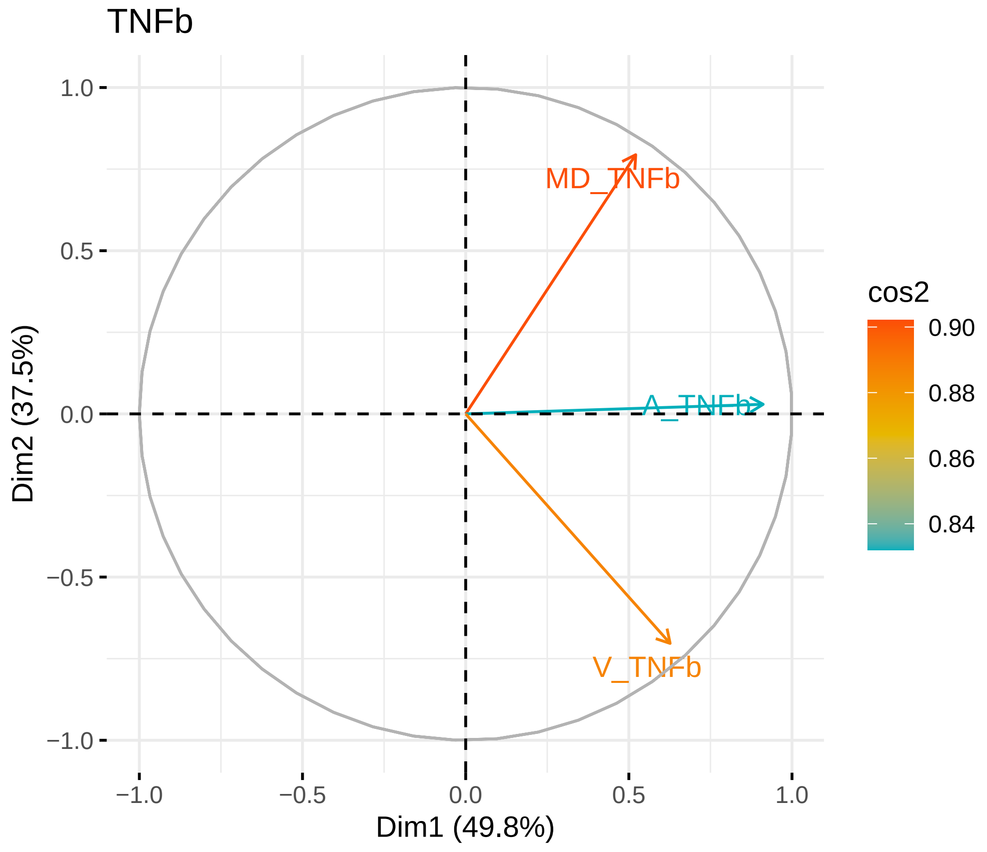
TNFb
MD_TNFb
A_TNFb
V_TNFb
Dim1 (49.8%)
Dim2 (37.5%)
cos2
0.90
0.88
0.86
0.84

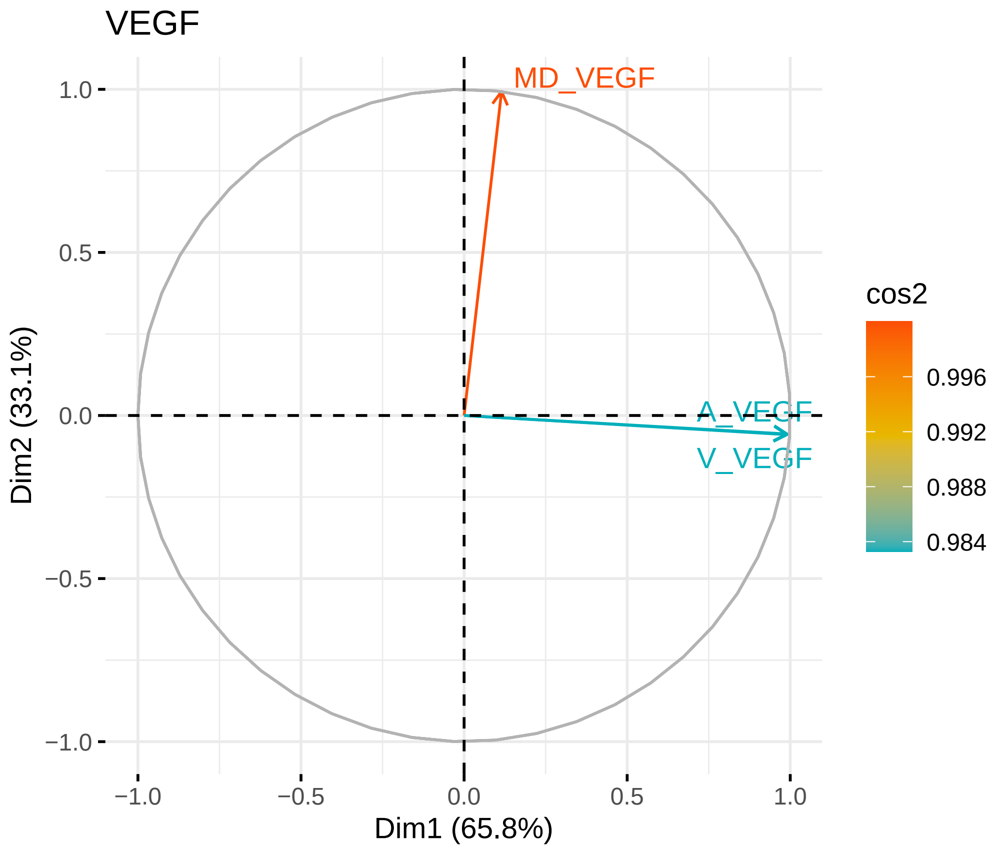

Test MFA

```
library(tidyr)
library(dplyr)
library(ggrepel)

patient_data.a <- ip_data[, seq(9, ncol(ip_data), 3)]
patient_data.v <- ip_data[, seq(10, ncol(ip_data), 3)]
patient_data.md <- ip_data[, seq(8, ncol(ip_data), 3)]
patient_data.a_v_md <- cbind(patient_data.a, patient_data.v, patient_data.md)
timeselect <-
  (6 == ip_data$Time) +
  (12 == ip_data$Time) +
  (30 == ip_data$Time) +
  (36 == ip_data$Time) +
  (54 == ip_data$Time) +
  (60 == ip_data$Time) +
  (78 == ip_data$Time) +
  (84 == ip_data$Time) +
  (102 == ip_data$Time) +
  (108 == ip_data$Time)
timeselect <- as.logical(timeselect)
patient_data.a_v_md <- patient_data.a_v_md[timeselect, ]
patient_data.a_v_md_av <- patient_data.a_v_md
```

```r
patient_data.a_v_md[is.na(patient_data.a_v_md)] <- 0
for (cyt in 1:42) {
  AV <- patient_data.a_v_md_av[ , cyt] - patient_data.a_v_md_av[ , cyt + 42]
  patient_data.a_v_md_av <- cbind(patient_data.a_v_md_av, AV)
  colnames(patient_data.a_v_md_av)[cyt + 42*3] <-
    paste0("AV", substr(colnames(patient_data.a_v_md_av)[cyt], 2, 100))
}
patient_data.a_v_md_av[is.na(patient_data.a_v_md_av)] <- 0

mfa.res0 <- MFA(patient_data.a_v_md, group = c(42, 42, 42),
                name.group = c("Arterial blood", "Jugular venous blood", "Brain-ECF")
                # , palette = c("red", "darkslateblue", "green4")
                )
```

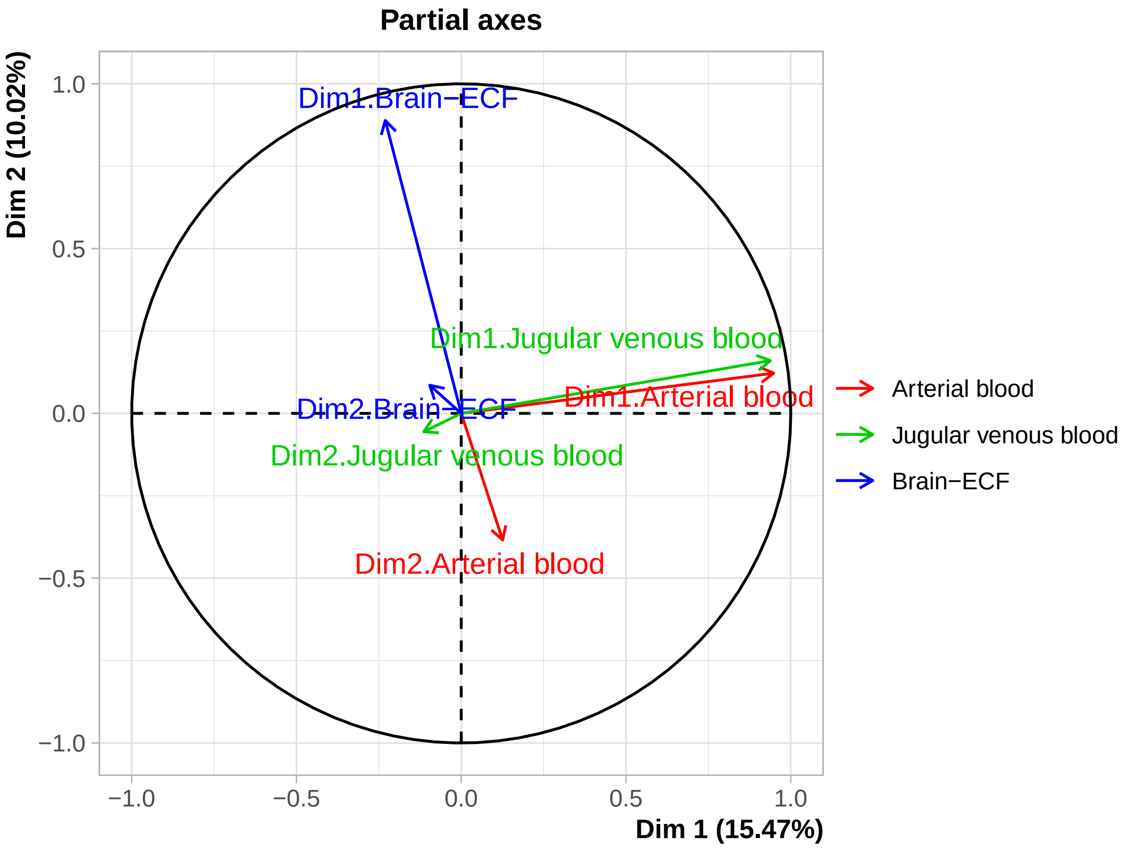


```
## Warning: ggrepel: 184 unlabeled data points (too many overlaps). Consider
## increasing max.overlaps
```

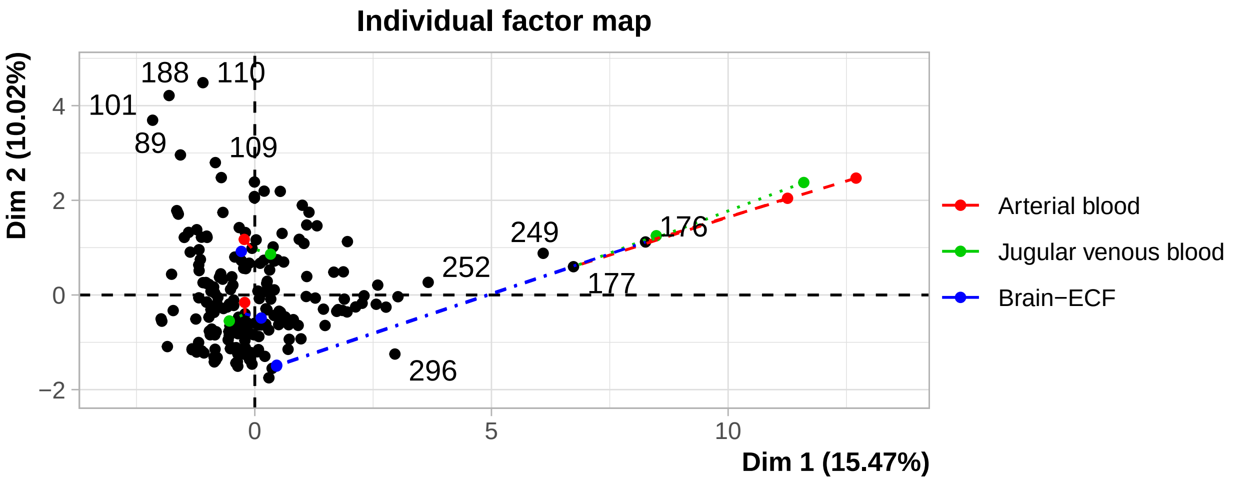

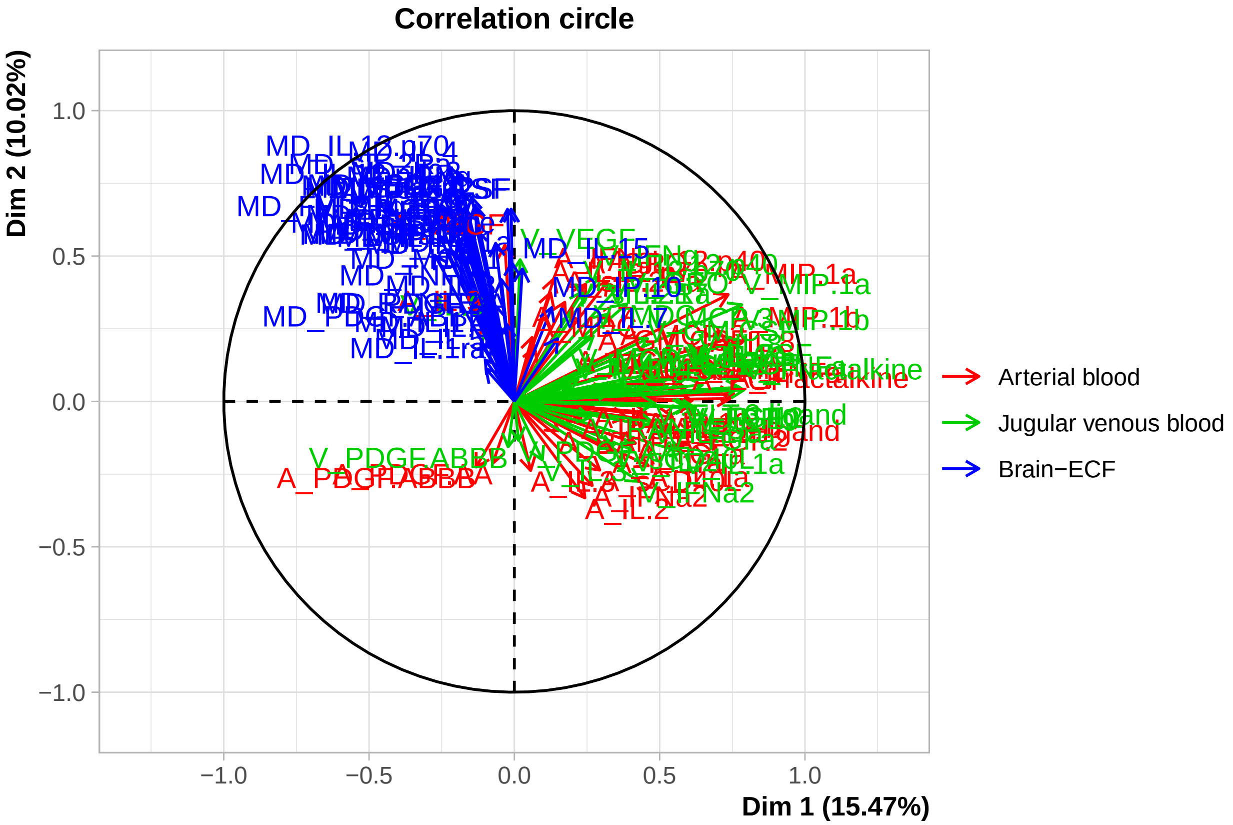


```
## Warning: ggrepel: 150 unlabeled data points (too many overlaps). Consider
## increasing max.overlaps
```

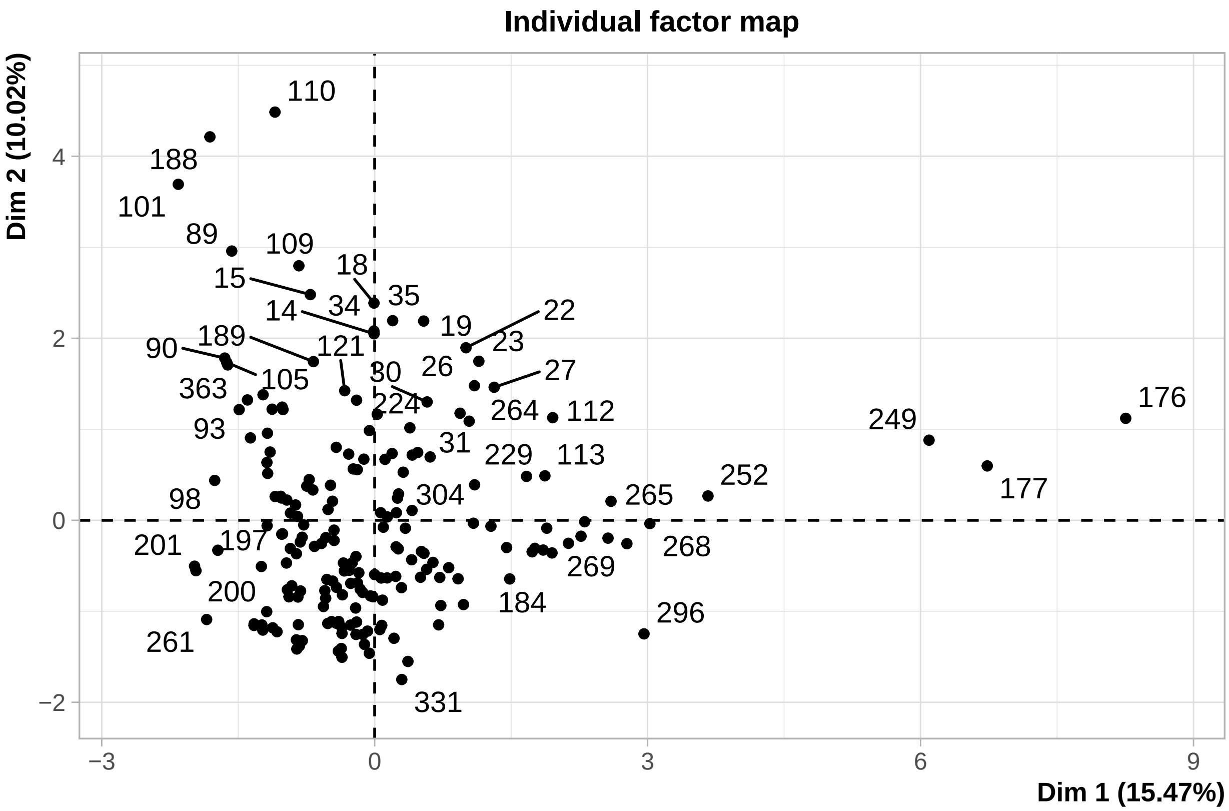
Individual factor map
Dim 2 (10.02%)
Dim 1 (15.47%)
110
188
101
89
109
18
15
35
34
14
22
19
23
189
121
90
26
27
30
105
363
224
264
112
176
249
93
31
229
113
252
177
98
304
265
268
201
197
269
200
184
296
261
331

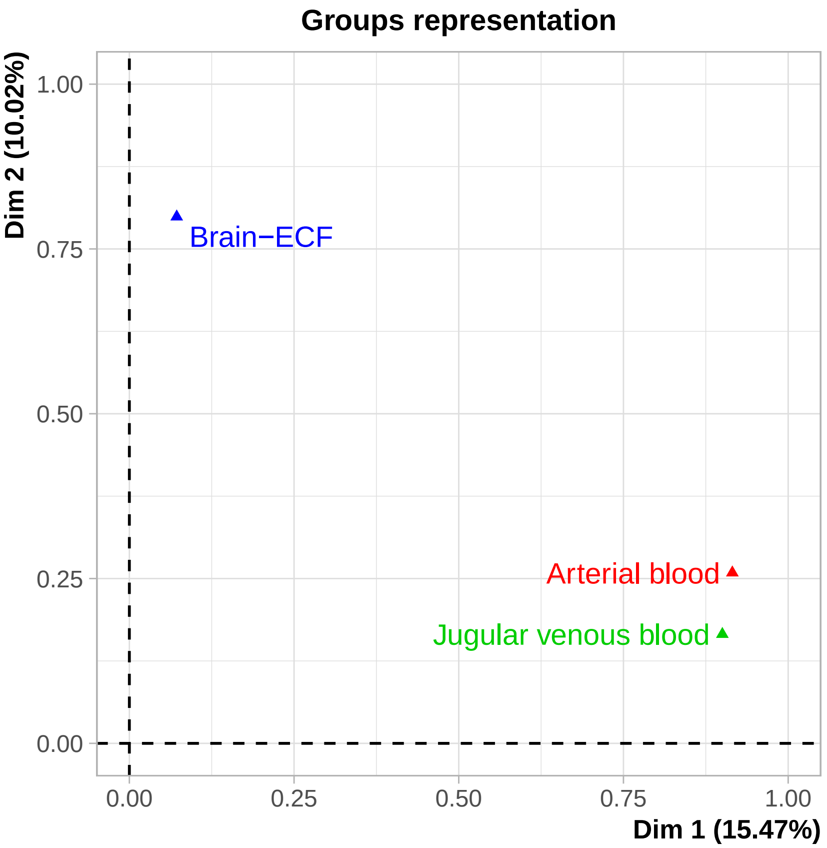
Groups representation
Dim 2 (10.02%)
Dim 1 (15.47%)
Brain−ECF
Arterial blood
Jugular venous blood

```r
pdf("MFA_0_200610.updated210111.pdf")
plot(mfa.res0, choix = "group")
dev.off()
```

```
## pdf
##   2
```

```r
tiff("MFA_0_200610.updated210111.tiff", width = 1000, height = 1000,
     pointsize = 100, compression = "lzw")
plot(mfa.res0, choix = "group", title = "",
     palette = palette(c("green4", "red", "blue")),
     cex = 3, cex.axis = 3, cex.lab = 3, cex.main = 3)
dev.off()
```

```
## pdf
##   2
```

```r
mfa.new <- MFA(patient_data.a_v_md_av, group = c(42, 42, 42, 42),
               name.group = c("Arterial blood", "Jugular venous blood", "Brain-ECF",
                              "A-V gradient"),
               num.group.sup = 4)
```

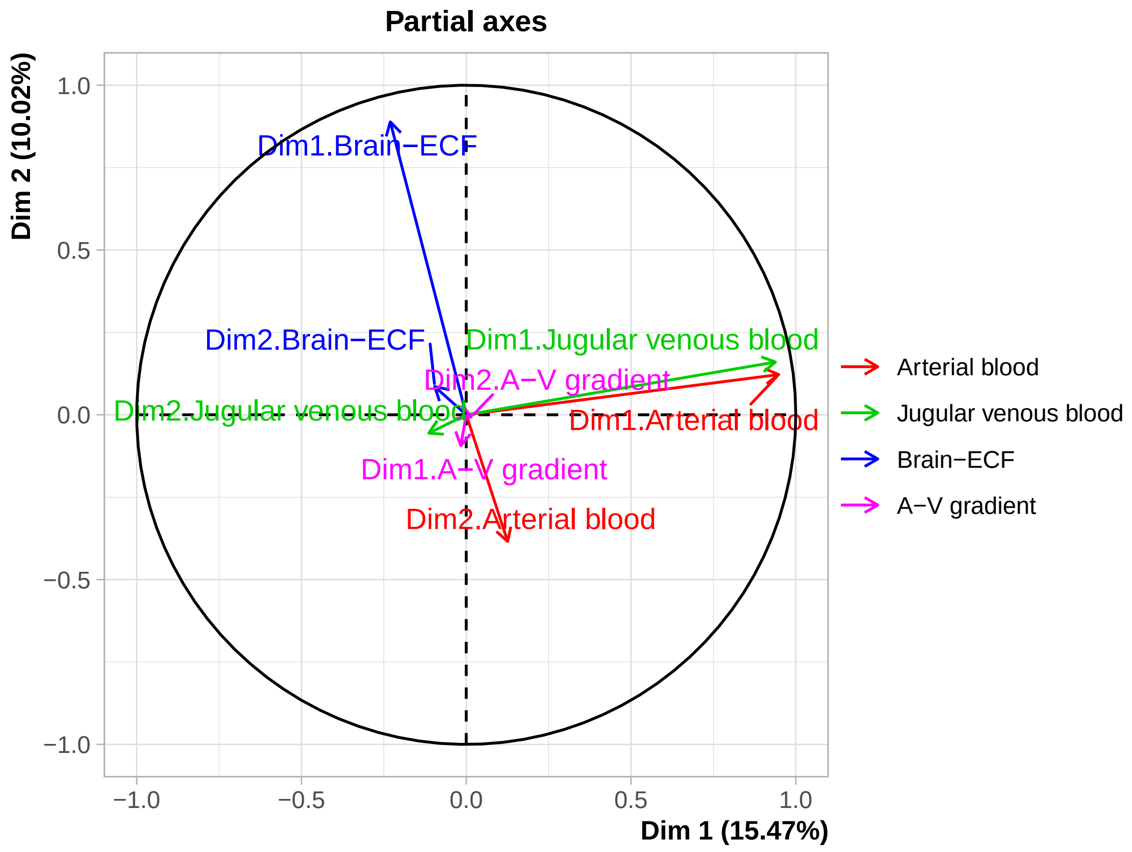


```
## Warning: ggrepel: 184 unlabeled data points (too many overlaps). Consider
## increasing max.overlaps
```

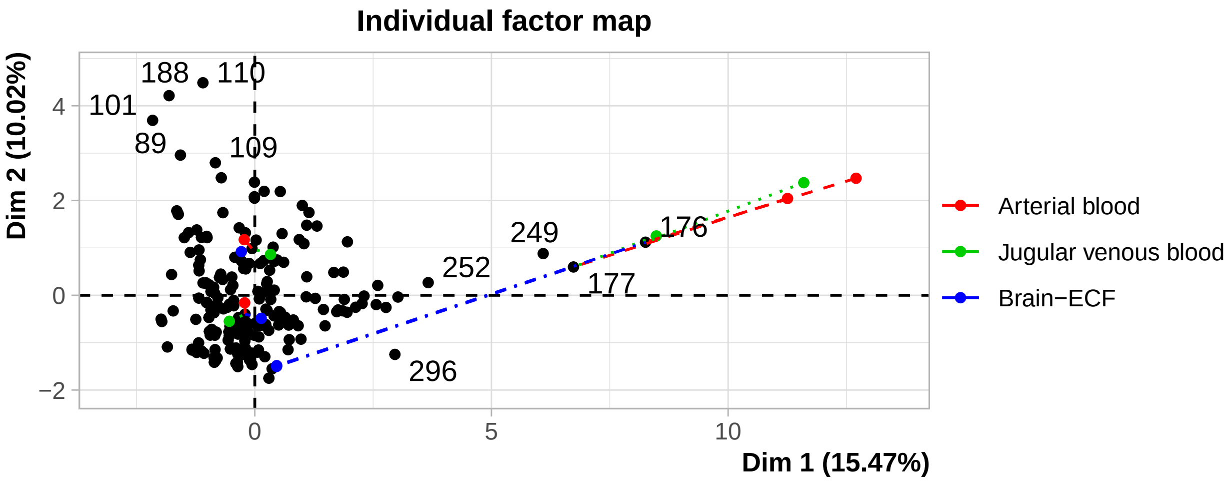

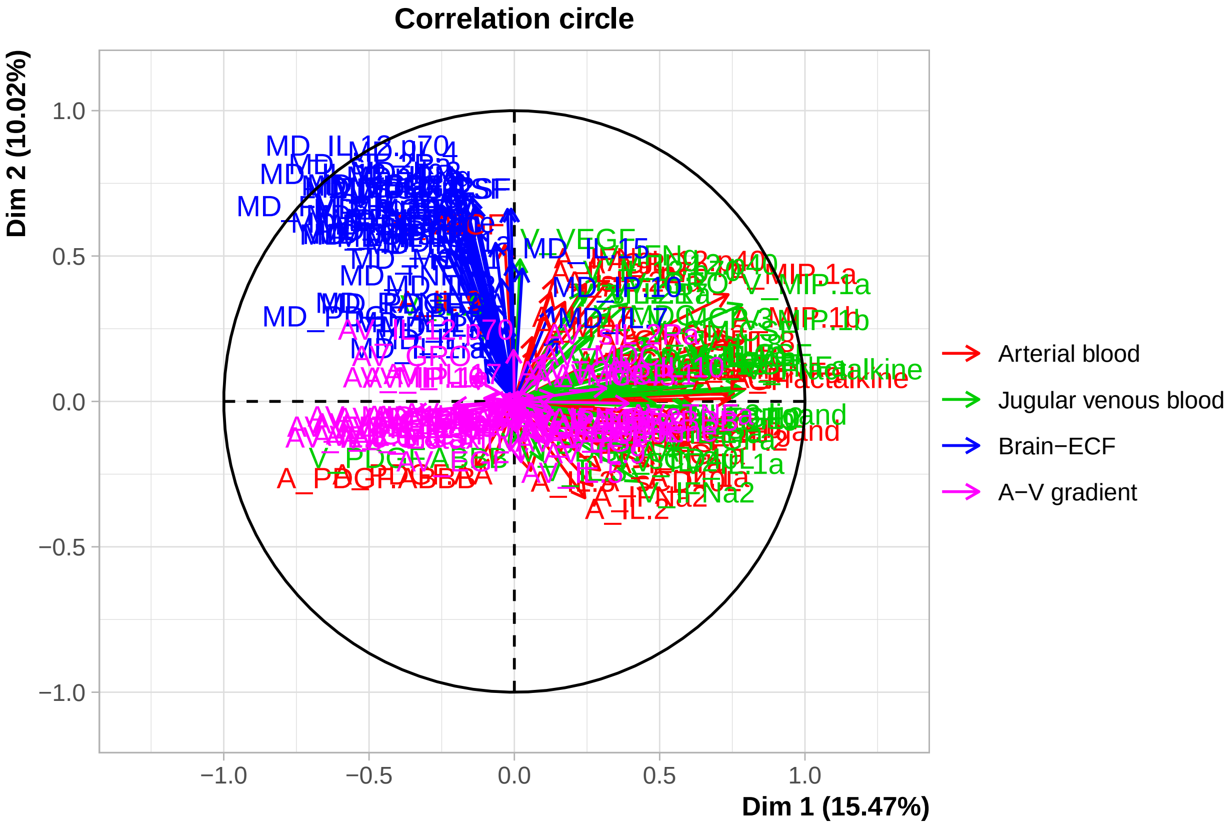


```
## Warning: ggrepel: 150 unlabeled data points (too many overlaps). Consider
## increasing max.overlaps
```

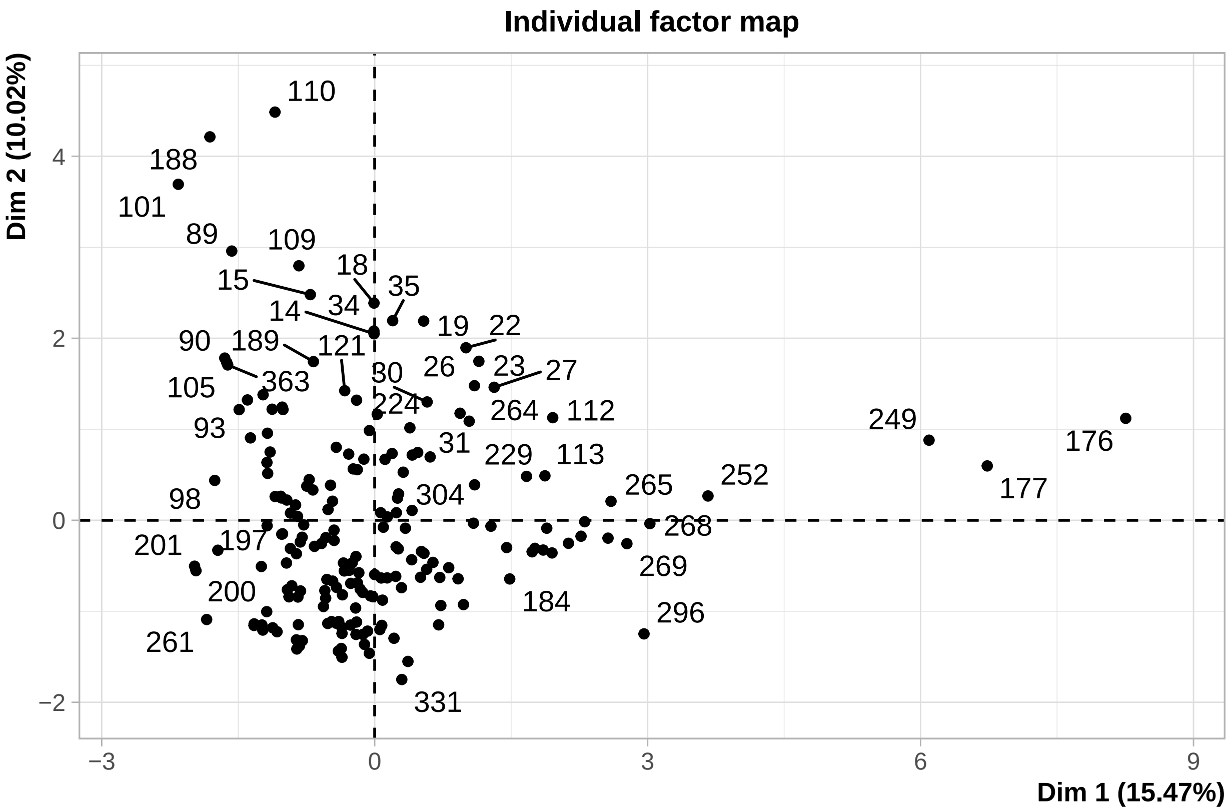
Individual factor map
Dim 2 (10.02%)
Dim 1 (15.47%)

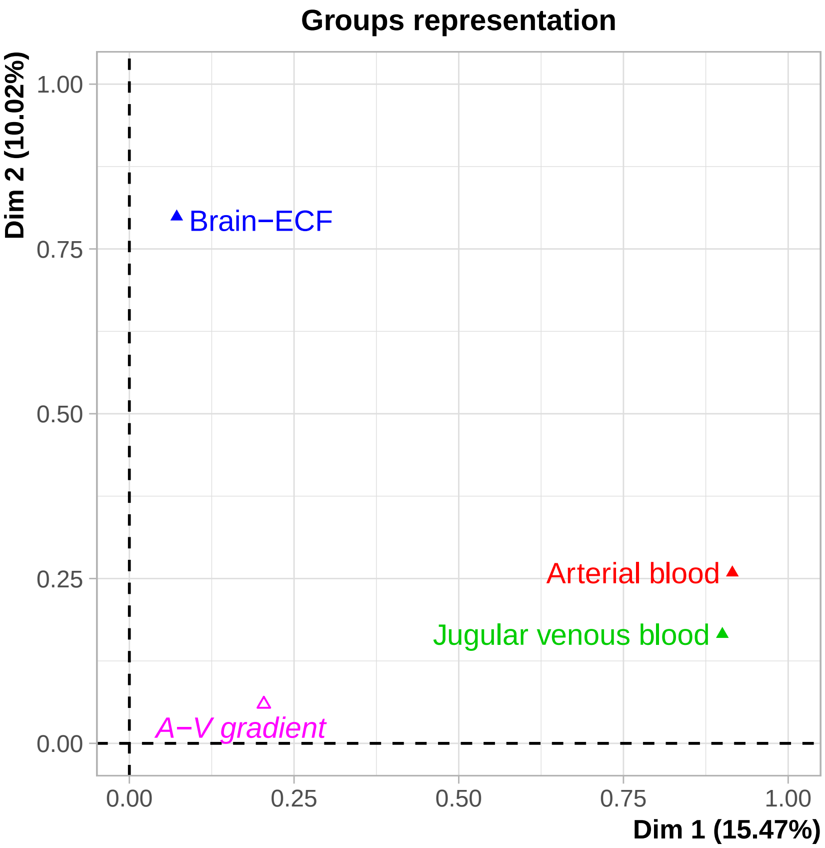
Groups representation
Dim 2 (10.02%)
Dim 1 (15.47%)
Brain−ECF
Arterial blood
Jugular venous blood
A−V gradient

```r
p1 <- fviz_mfa_var(mfa.new, choice = "group", repel = TRUE) +
  theme(plot.title = element_blank())

ggsave("MFA_new_200610.updated210111.pdf",
       plot = p1,
       device = "pdf",
       width = 5,
       height = 5,
       units = "in")

ggsave("MFA_new_200610.updated210111.tiff",
       plot = p1,
       device = "tiff",
       width = 5,
       height = 5,
       units = "in",
       dpi = "retina")
```

Code for Figure 1 (b, c)

Philipp

Revised: May 11, 2021

Contents

Load the data 1

For loop over cytokines to do the log plots 1

Load the data

```
library(openxlsx)
ip_data <- read.xlsx("../Dataset/Interpolated_data4.3.Adel201001.xlsx")
```

For loop over cytokines to do the log plots

```
library(reshape)
library(ggplot2)

data_MD <- log(ip_data[ , seq(from = 8, to = 133, by = 3)])
data_A <- log(ip_data[ , seq(from = 9, to = 133, by = 3)])
data_V <- log(ip_data[ , seq(from = 10, to = 133, by = 3)])
colnames(data_MD) <-
  substr(colnames(ip_data)[seq(from = 8, to = 133, by = 3)], 4, 100)
colnames(data_A) <-
  substr(colnames(ip_data)[seq(from = 8, to = 133, by = 3)], 4, 100)
colnames(data_V) <-
  substr(colnames(ip_data)[seq(from = 8, to = 133, by = 3)], 4, 100)
data_long_MD <- melt(data_MD)
```

```
## Using  as id variables
```

```
data_long_A <- melt(data_A)
```

```
## Using  as id variables
```

```
data_long_V <- melt(data_V)
```

```
## Using  as id variables
```

```
data_long_MD_A <- cbind(data.frame(Cytokine = data_long_MD[ , 1]),
                        Microdialysis = data_long_MD[ , 2],
```

```
                        Arterial = data_long_A[ , 2])
data_long_A_V <- cbind(data.frame(Cytokine = data_long_A[ , 1]),
                       Arterial = data_long_A[ , 2],
                       Venous = data_long_V[ , 2])

for (cytokine in 1:42) {
  cytname <- substr(colnames(ip_data)[5 + 3 * cytokine], 4, 100)

  p1 <- ggplot(data_long_MD_A, aes(x = Arterial, y = Microdialysis)) +
    geom_point(aes(color = Cytokine), alpha = 0.05) +
    annotate("point",
             data_long_MD_A[data_long_MD_A$Cytokine == cytname, "Arterial"],
             data_long_MD_A[data_long_MD_A$Cytokine == cytname, "Microdialysis"],
             size = 2) +
    scale_x_continuous(limits = c(-5,12)) +
    scale_y_continuous(limits = c(-5,12)) +
    ggtitle(cytname) +
    labs(x = "log(Arterial)", y = "log(Microdialysis)") +
    theme(panel.grid.major = element_blank(),
          panel.grid.minor = element_blank(),
          panel.background = element_blank(),
          axis.line = element_line(colour = "black"),
          legend.position = "none")
  print(p1)

  p2 <- ggplot(data_long_A_V, aes(x = Arterial, y = Venous)) +
    geom_point(aes(color = Cytokine), alpha = 0.05) +
    annotate("point",
             data_long_A_V[data_long_A_V$Cytokine == cytname, "Arterial"],
             data_long_A_V[data_long_A_V$Cytokine == cytname, "Venous"],
             size = 2) +
    scale_x_continuous(limits = c(-5,12)) +
    scale_y_continuous(limits = c(-5,12)) +
    ggtitle(cytname) +
    labs(x = "log(Arterial)", y = "log(Jugular)") +
    theme(panel.grid.major = element_blank(),
          panel.grid.minor = element_blank(),
          panel.background = element_blank(),
          axis.line = element_line(colour = "black"),
          legend.position = "none")
  print(p2)

  if (cytname == "IL.6") {
    ggsave("arvid.il6.md.210108.revised210304.tiff",
           plot = p1,
           device = "tiff",
           width = 4,
           height = 2.5,
           units = "in",
           dpi = "retina")
    ggsave("arvid.il6.a.210108.revised210304.tiff",
           plot = p2,
           device = "tiff",
```

```
           width = 4,
           height = 2.5,
           units = "in",
           dpi = "retina")
  }
}
```

```
## Warning: Removed 10923 rows containing missing values (geom_point).

## Warning: Removed 318 rows containing missing values (geom_point).
```

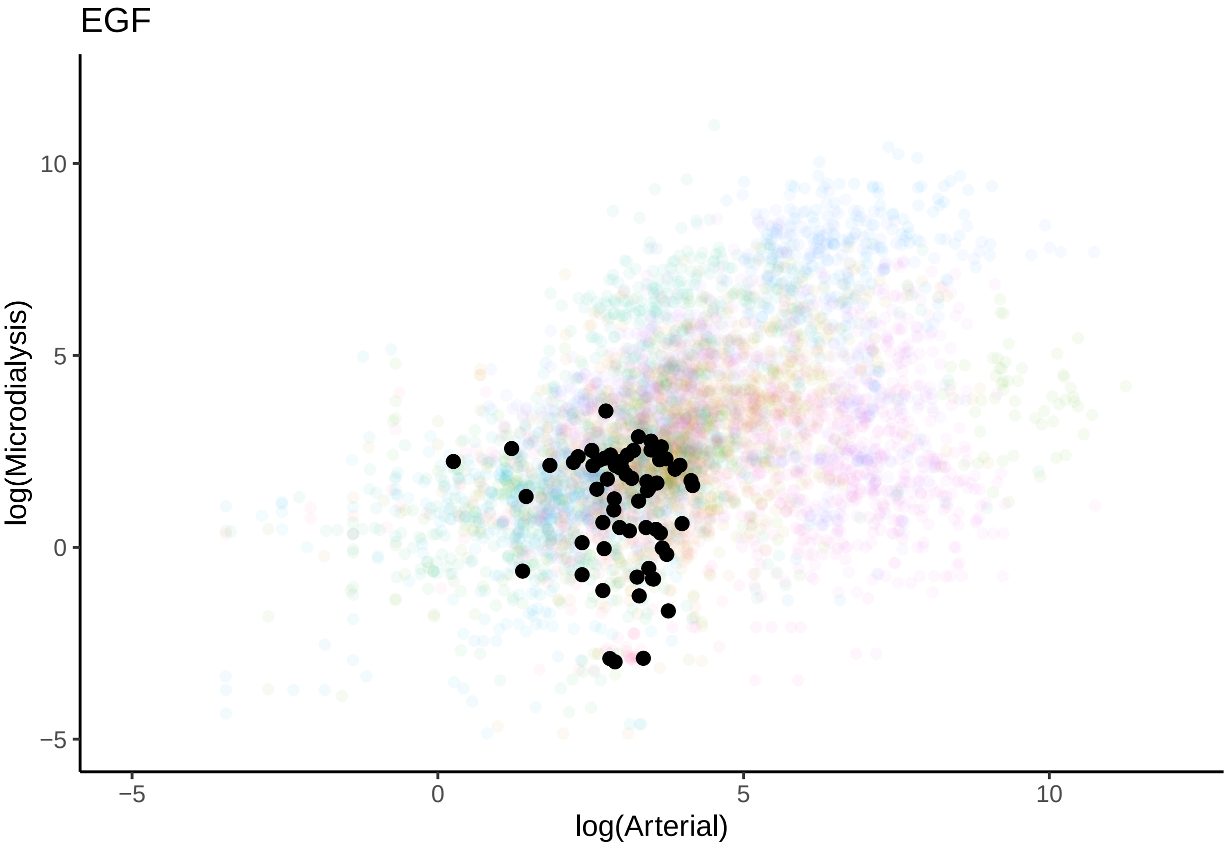


```
## Warning: Removed 10462 rows containing missing values (geom_point).

## Warning: Removed 264 rows containing missing values (geom_point).
```

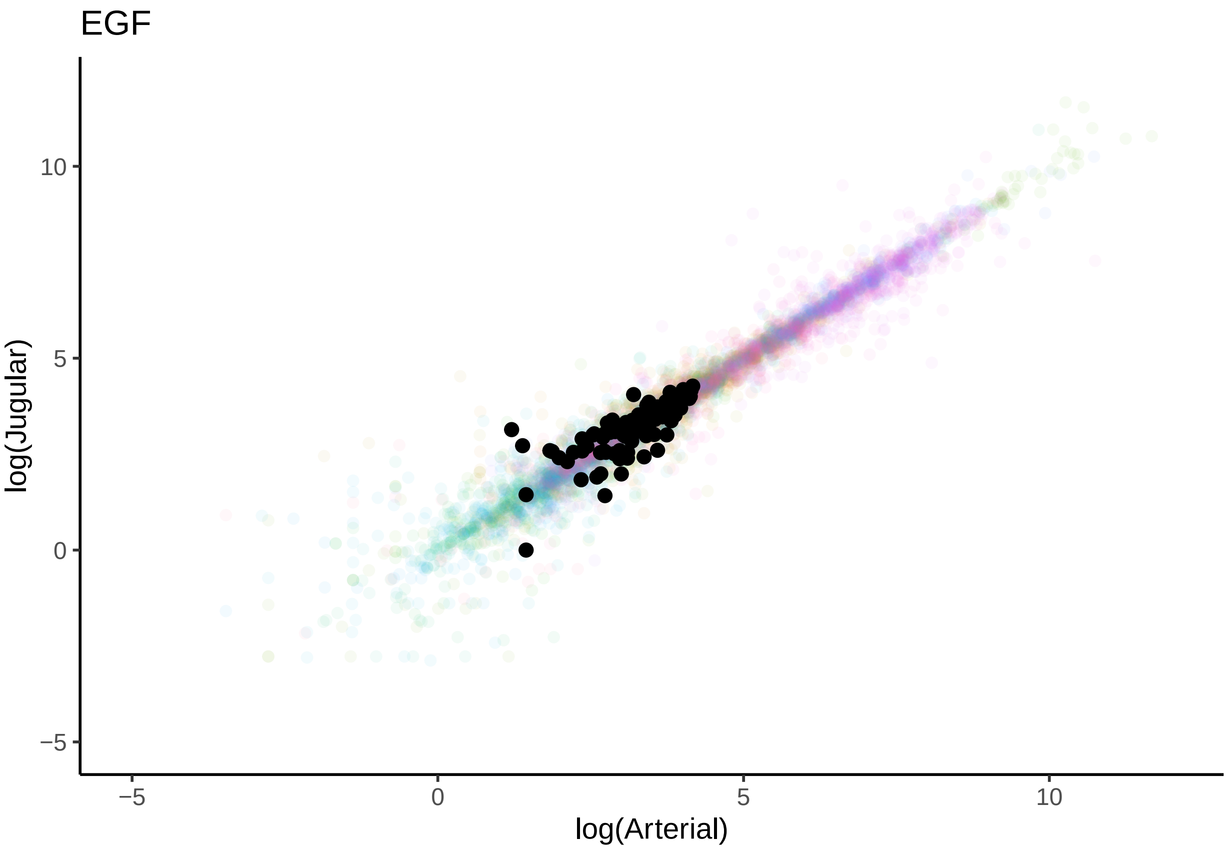


```
## Warning: Removed 10923 rows containing missing values (geom_point).

## Warning: Removed 202 rows containing missing values (geom_point).
```

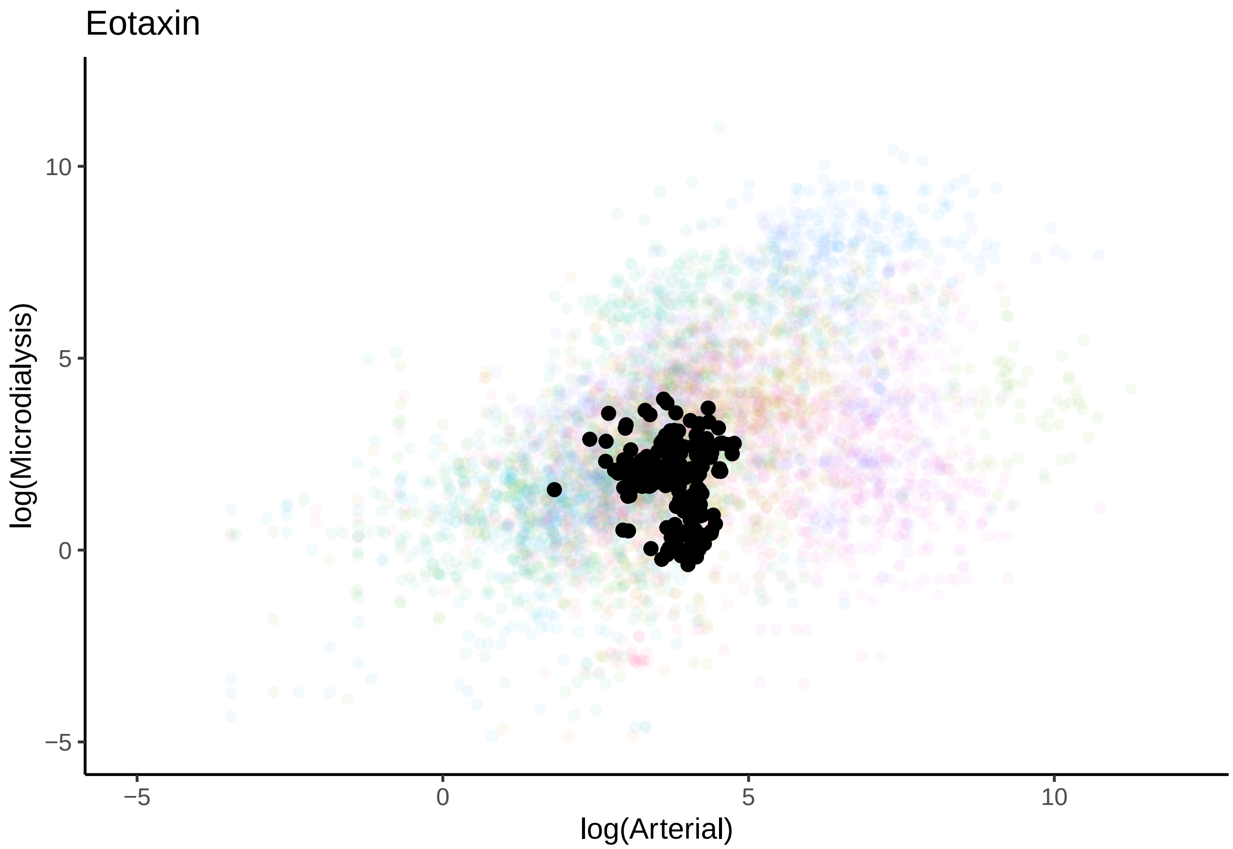


```
## Warning: Removed 10462 rows containing missing values (geom_point).
## Warning: Removed 213 rows containing missing values (geom_point).
```

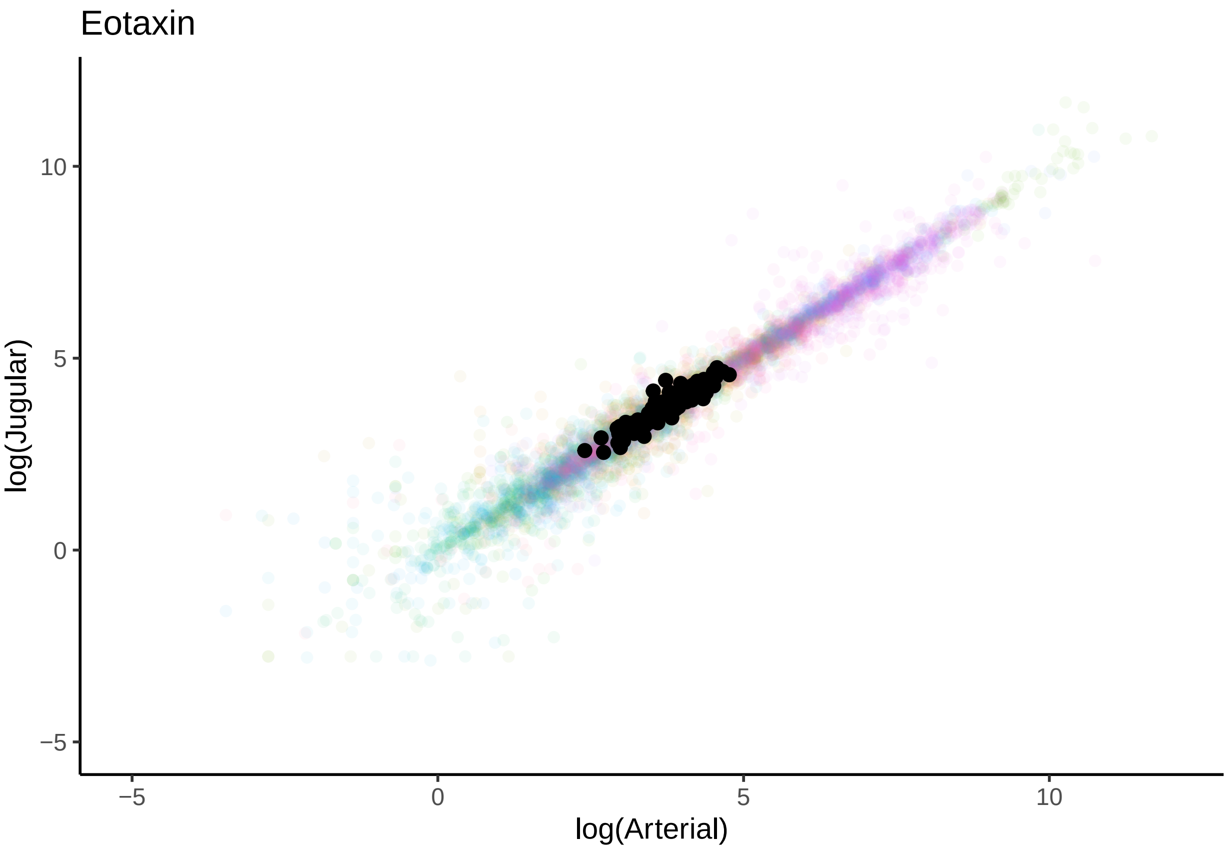


```
## Warning: Removed 10923 rows containing missing values (geom_point).
## Warning: Removed 218 rows containing missing values (geom_point).
```

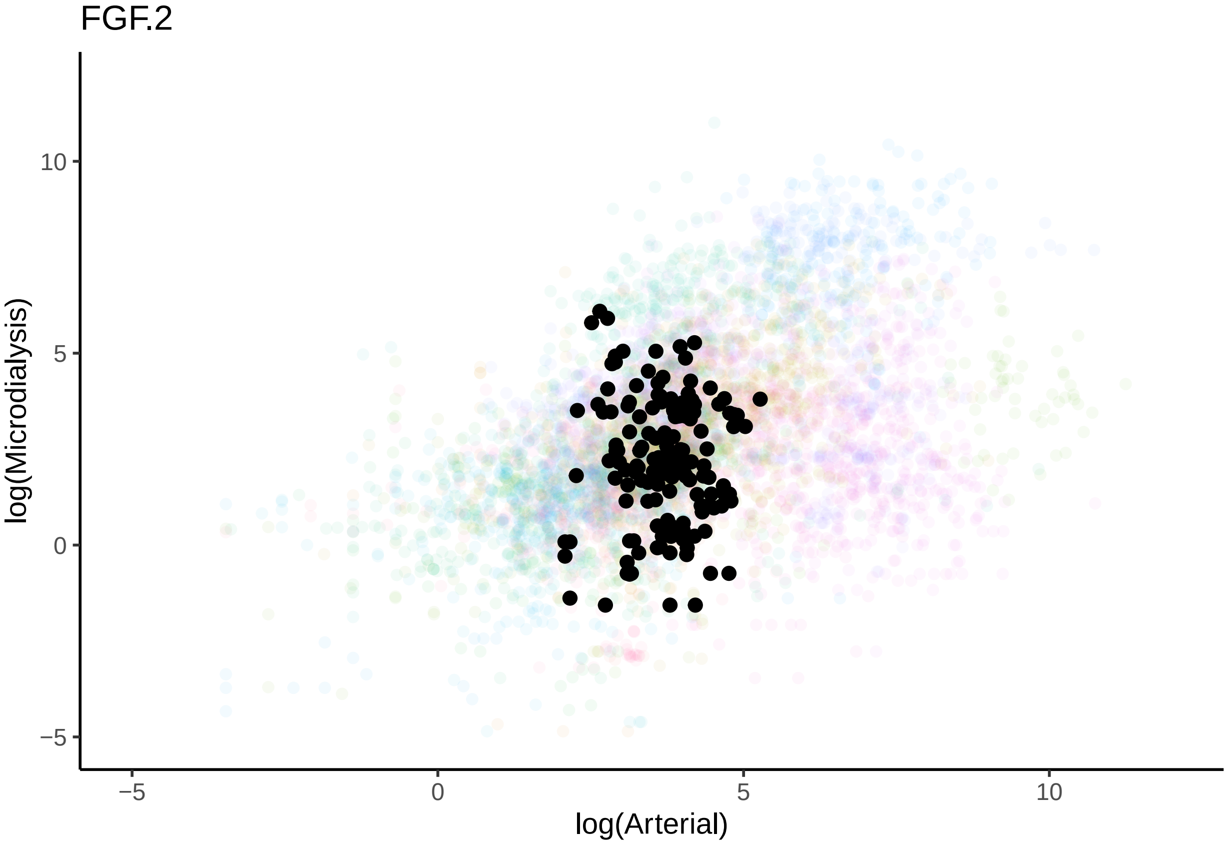


```
## Warning: Removed 10462 rows containing missing values (geom_point).
## Warning: Removed 216 rows containing missing values (geom_point).
```

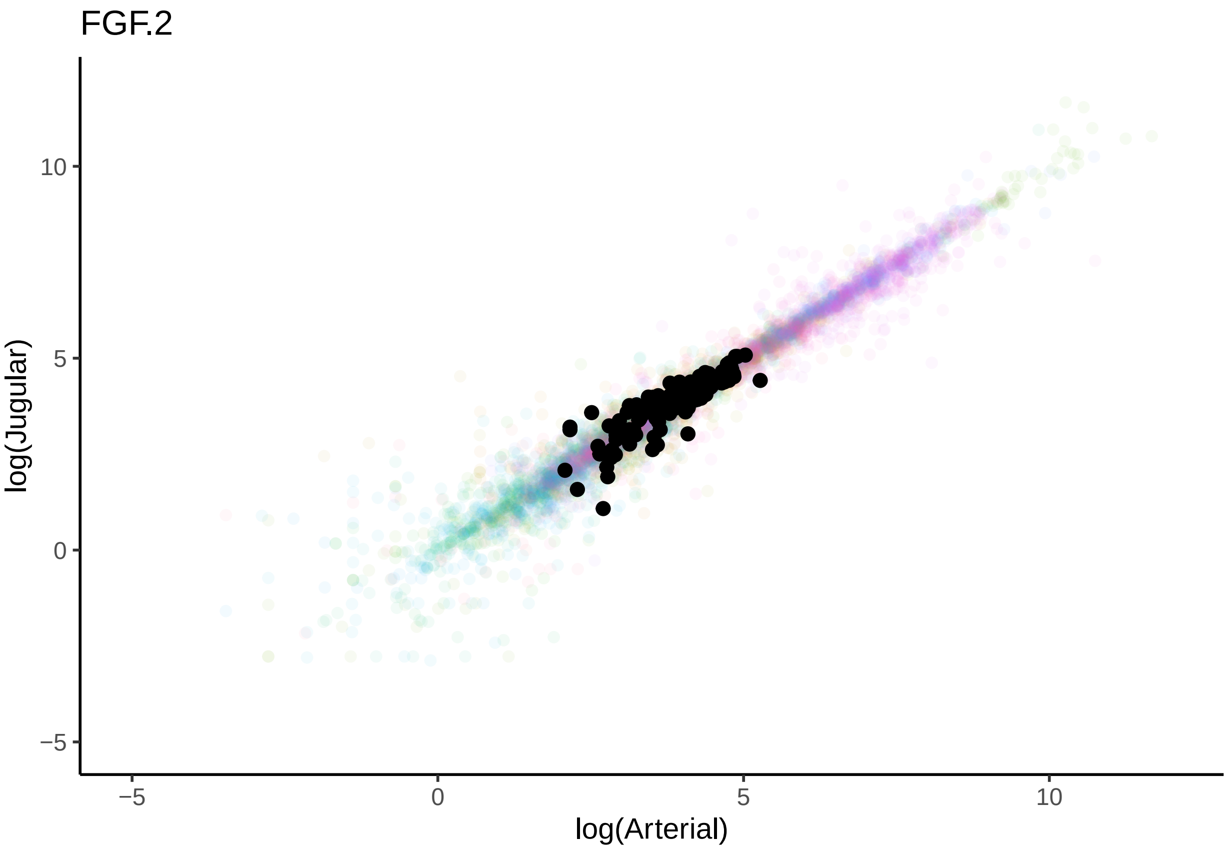


```
## Warning: Removed 10923 rows containing missing values (geom_point).
## Warning: Removed 323 rows containing missing values (geom_point).
```

FLT.3.ligand

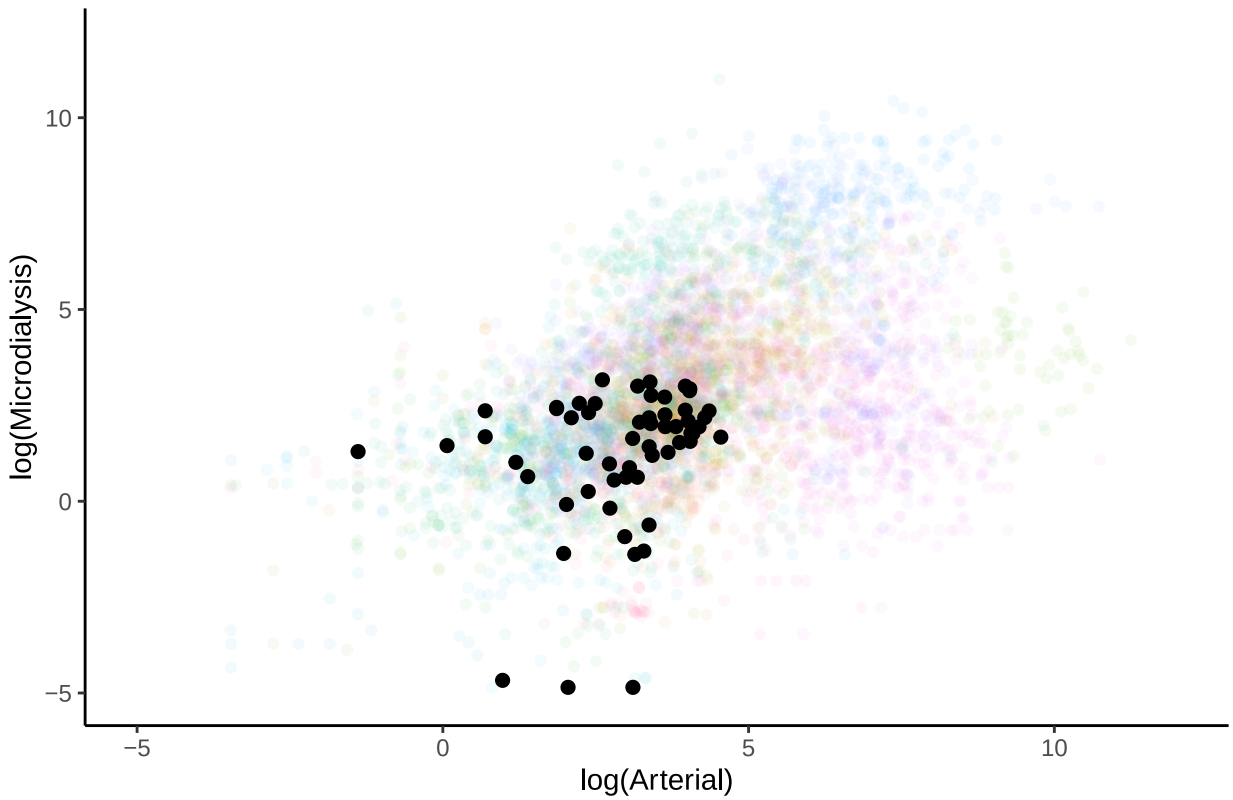


```
## Warning: Removed 10462 rows containing missing values (geom_point).

## Warning: Removed 337 rows containing missing values (geom_point).
```

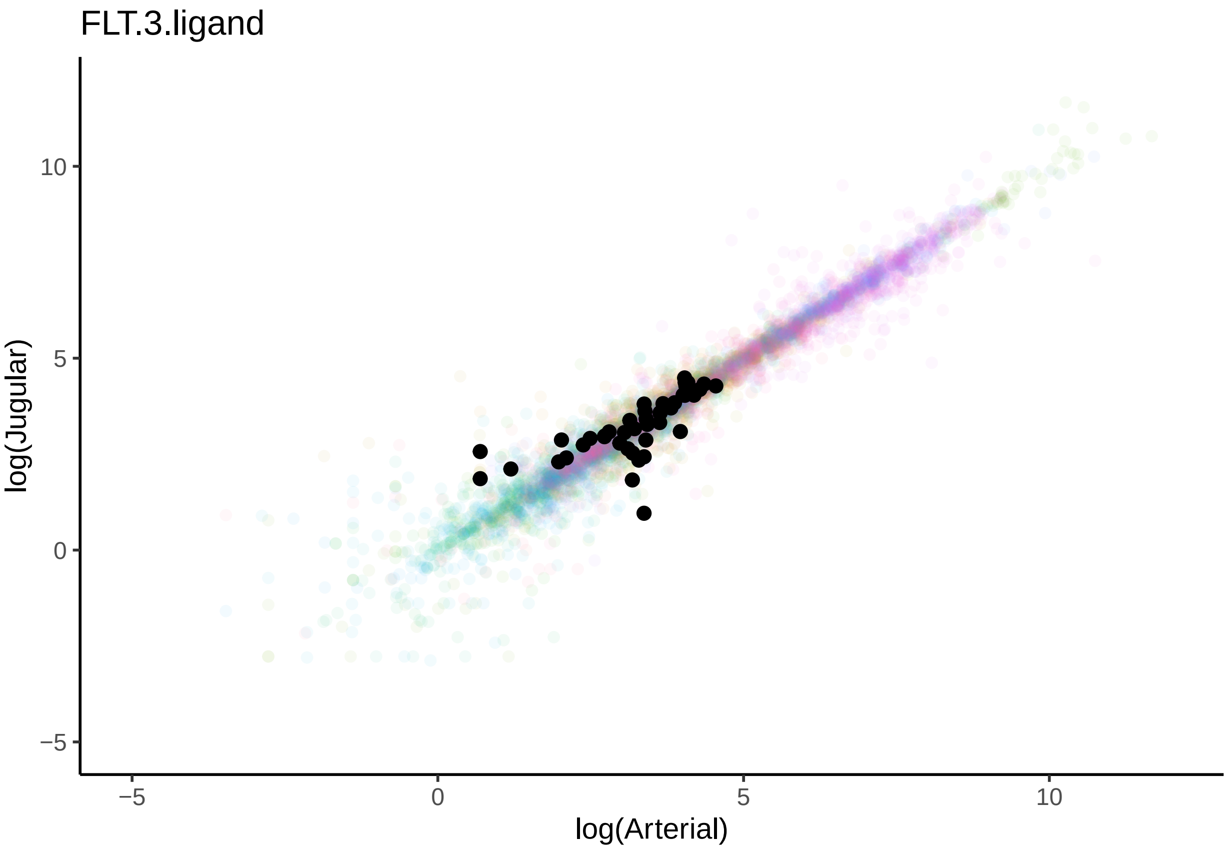


```
## Warning: Removed 10923 rows containing missing values (geom_point).

## Warning: Removed 215 rows containing missing values (geom_point).
```

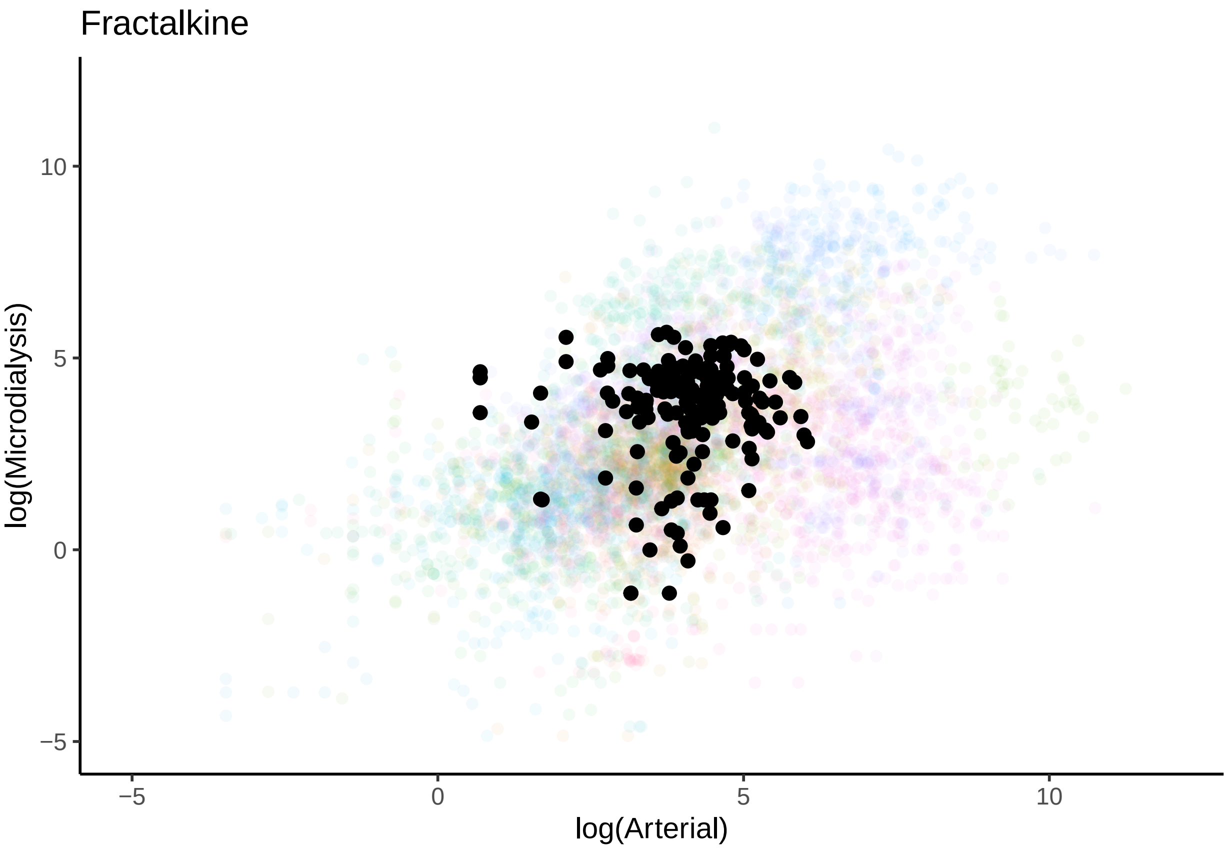


```
## Warning: Removed 10462 rows containing missing values (geom_point).

## Warning: Removed 230 rows containing missing values (geom_point).
```

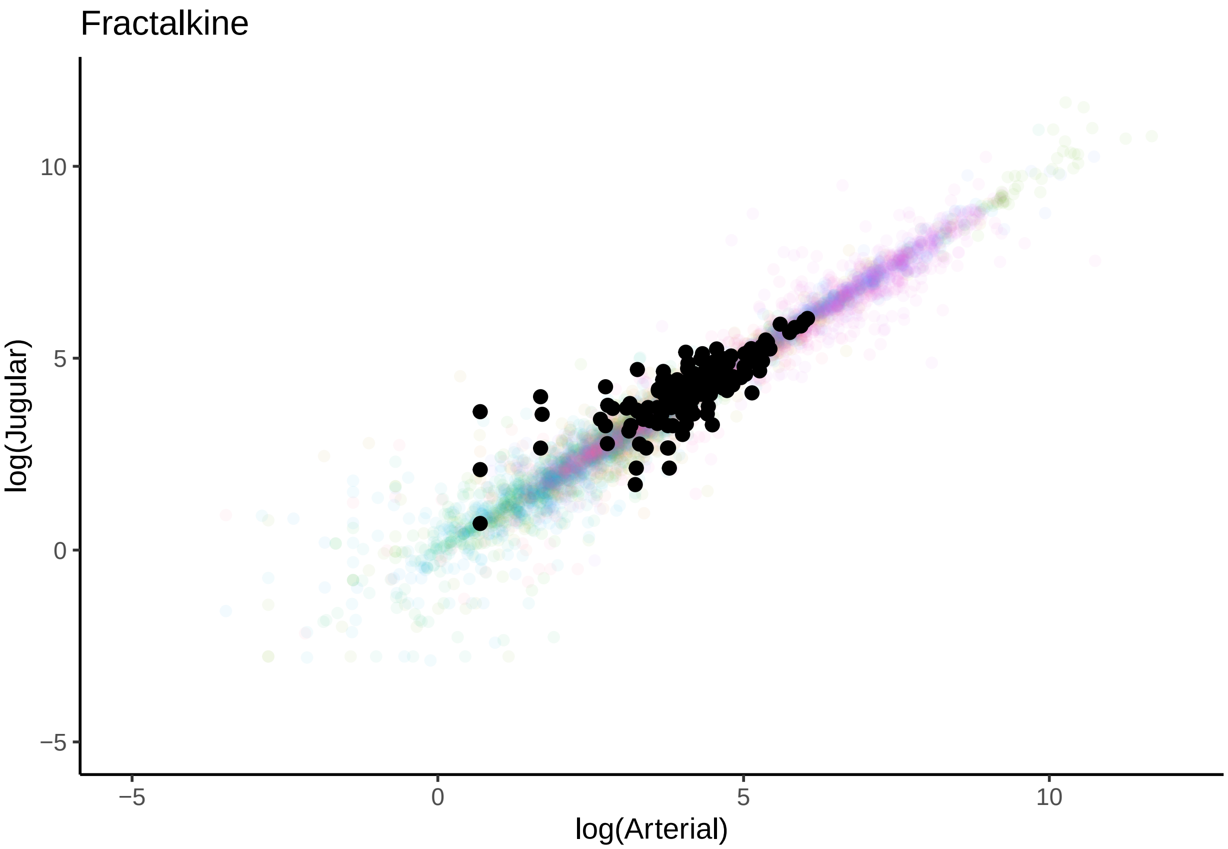


```
## Warning: Removed 10923 rows containing missing values (geom_point).

## Warning: Removed 214 rows containing missing values (geom_point).
```

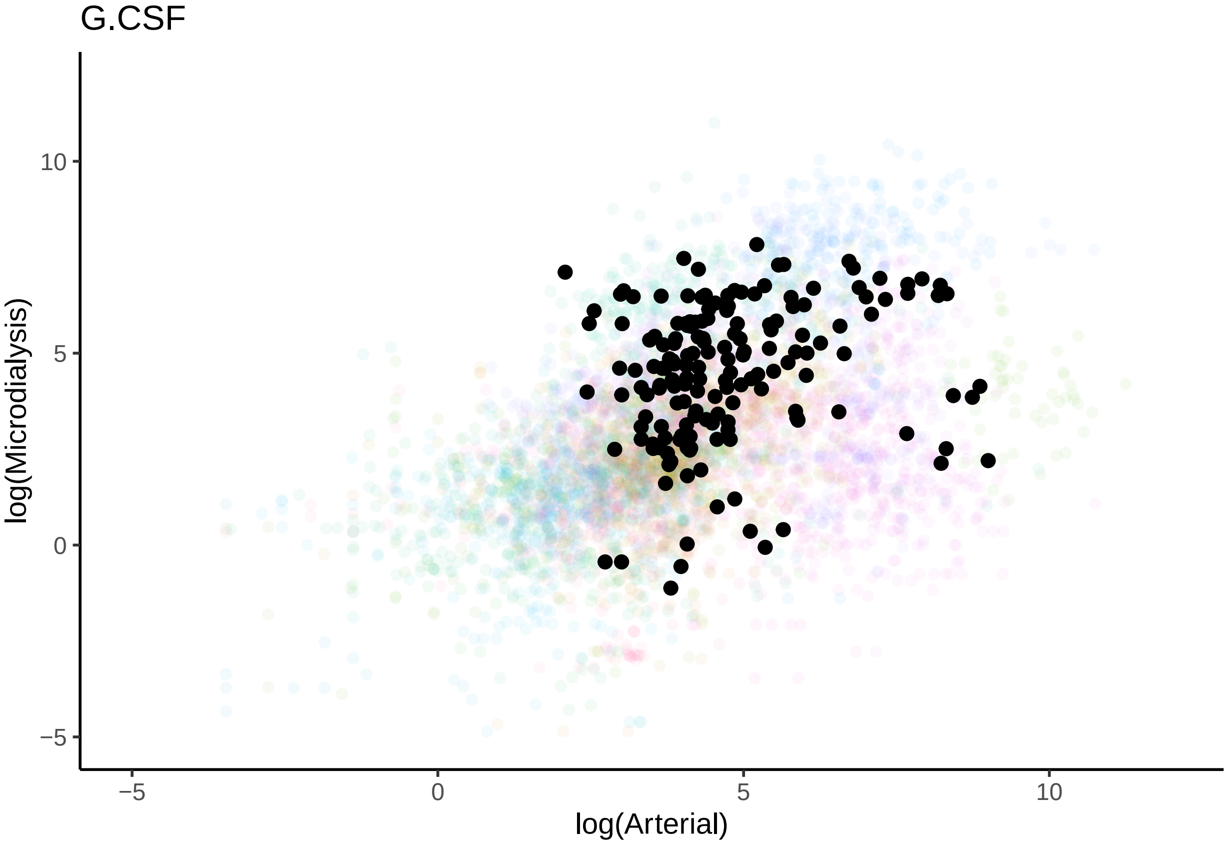


```
## Warning: Removed 10462 rows containing missing values (geom_point).
## Warning: Removed 215 rows containing missing values (geom_point).
```

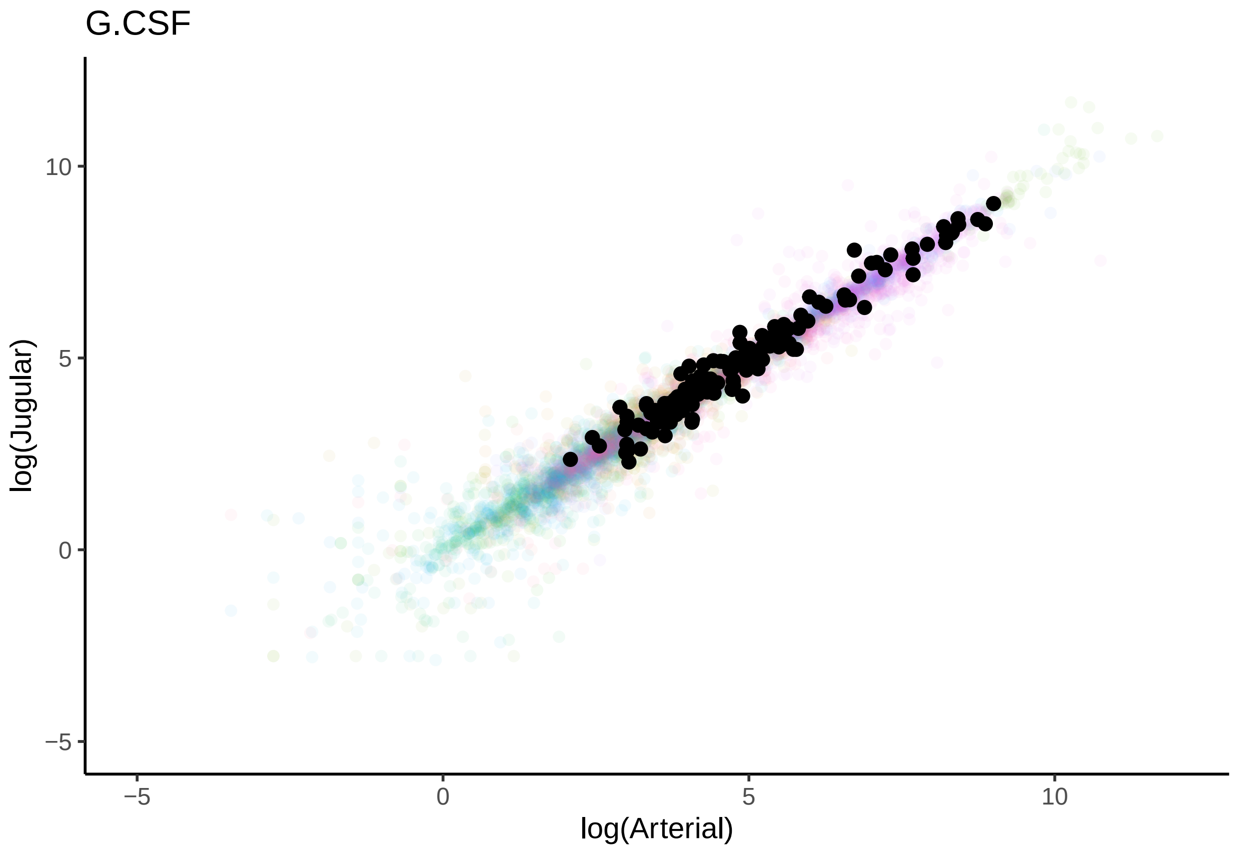


```
## Warning: Removed 10923 rows containing missing values (geom_point).
## Warning: Removed 209 rows containing missing values (geom_point).
```

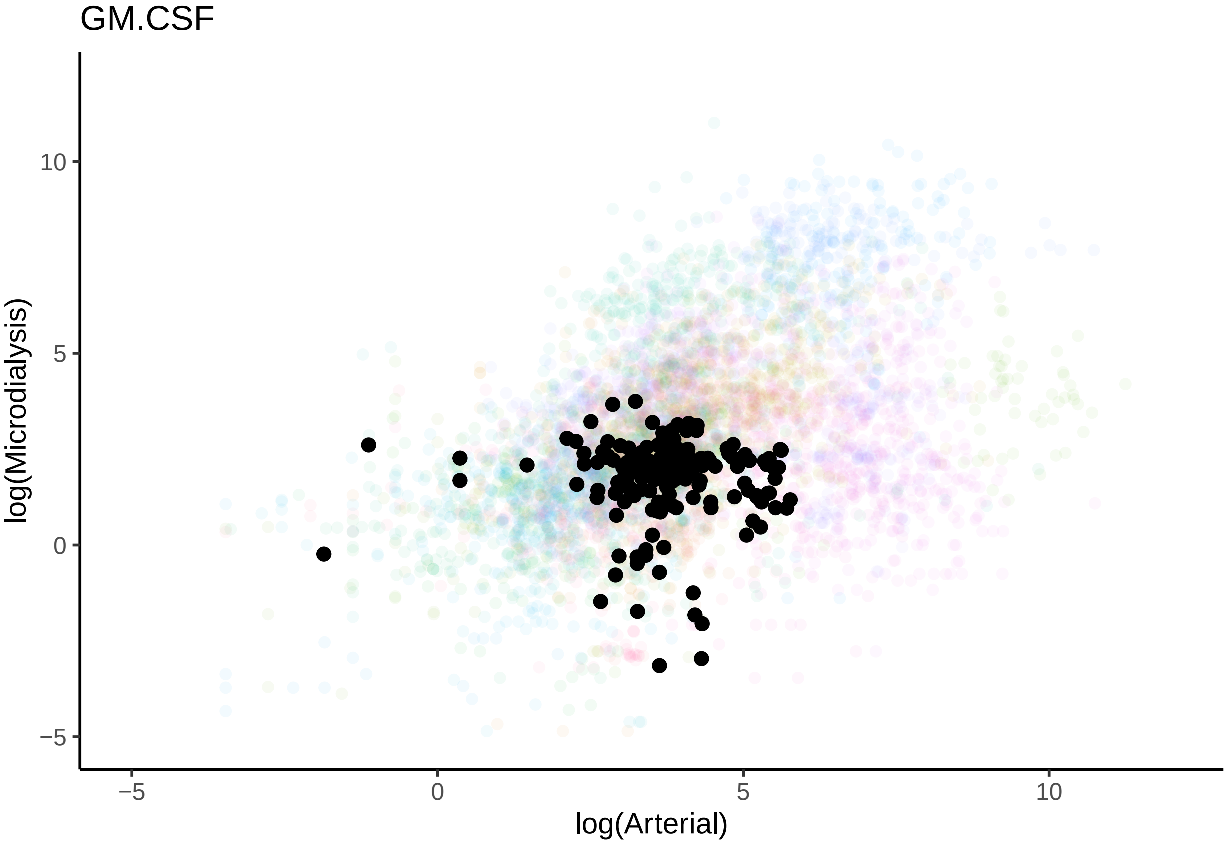


```
## Warning: Removed 10462 rows containing missing values (geom_point).
## Warning: Removed 221 rows containing missing values (geom_point).
```

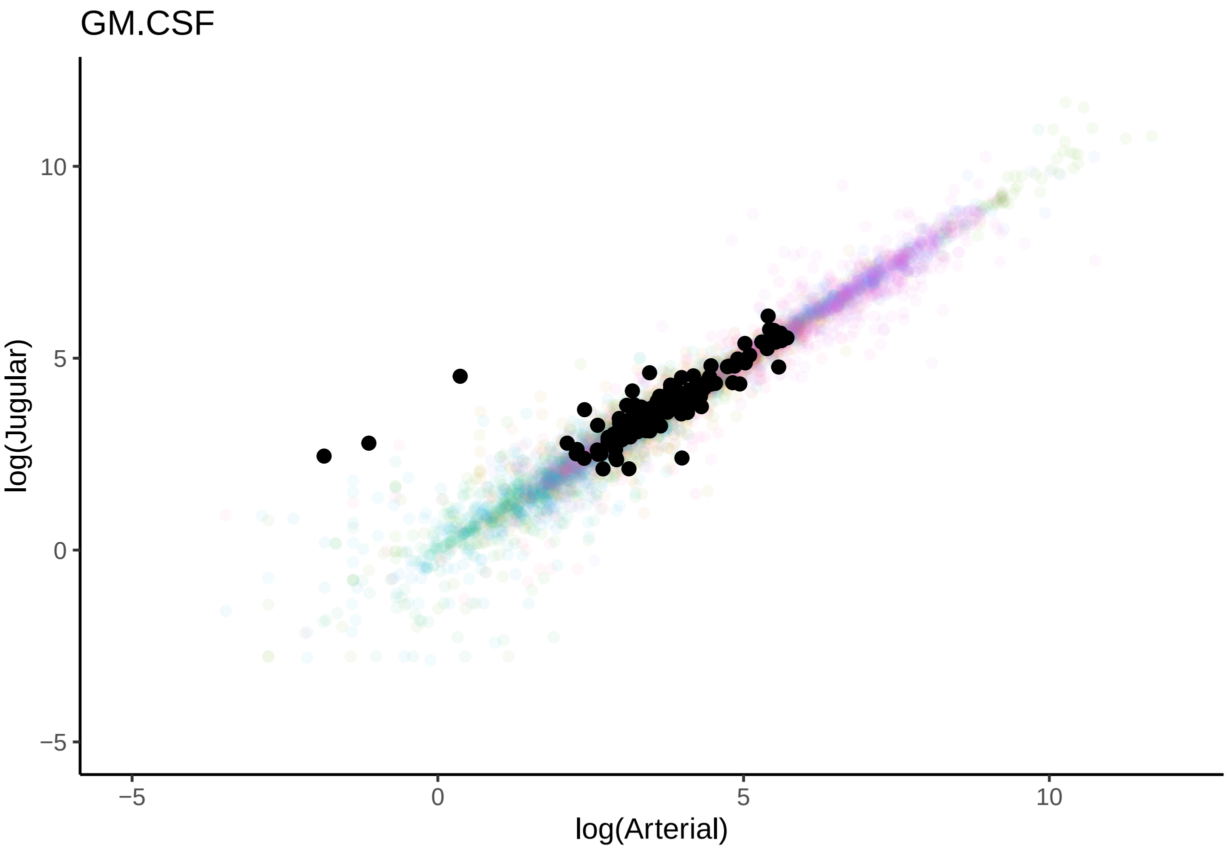


```
## Warning: Removed 10923 rows containing missing values (geom_point).

## Warning: Removed 204 rows containing missing values (geom_point).
```

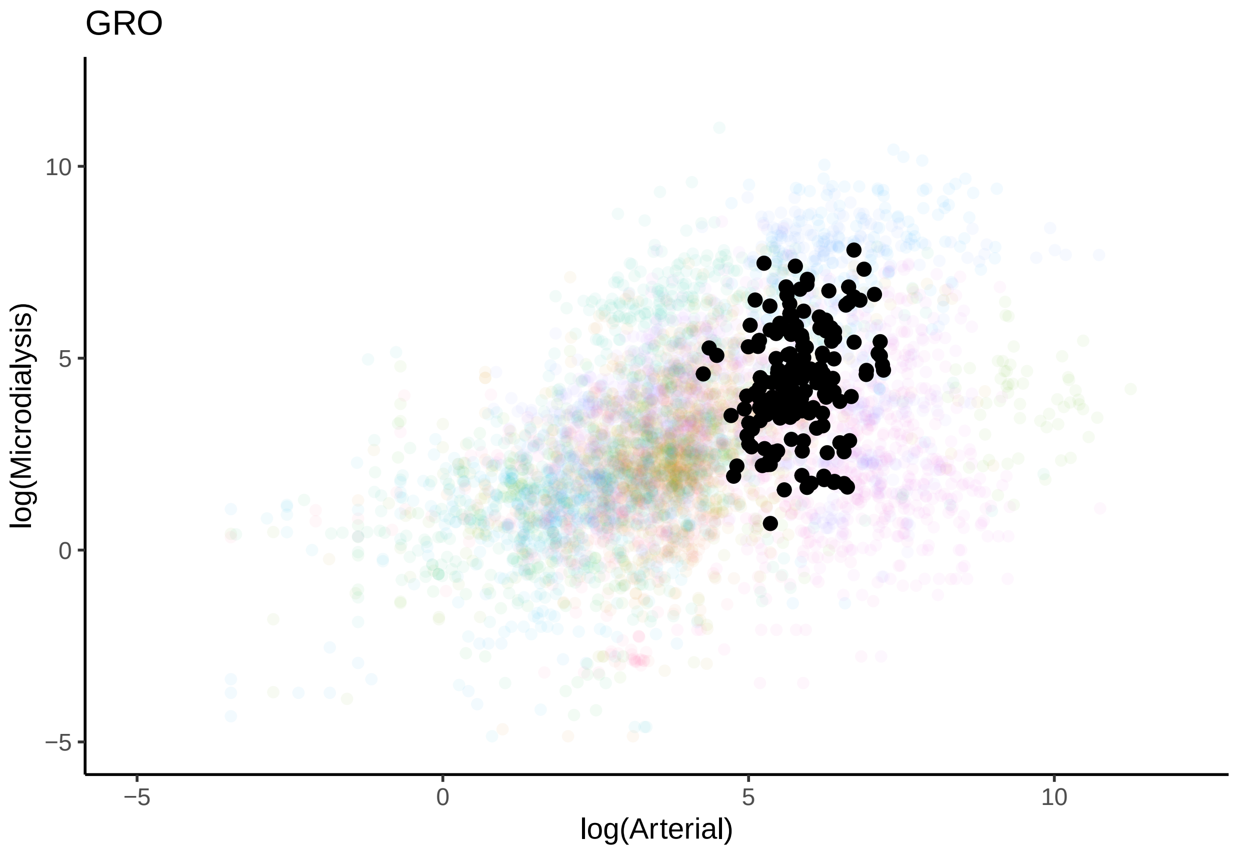


```
## Warning: Removed 10462 rows containing missing values (geom_point).
## Warning: Removed 213 rows containing missing values (geom_point).
```

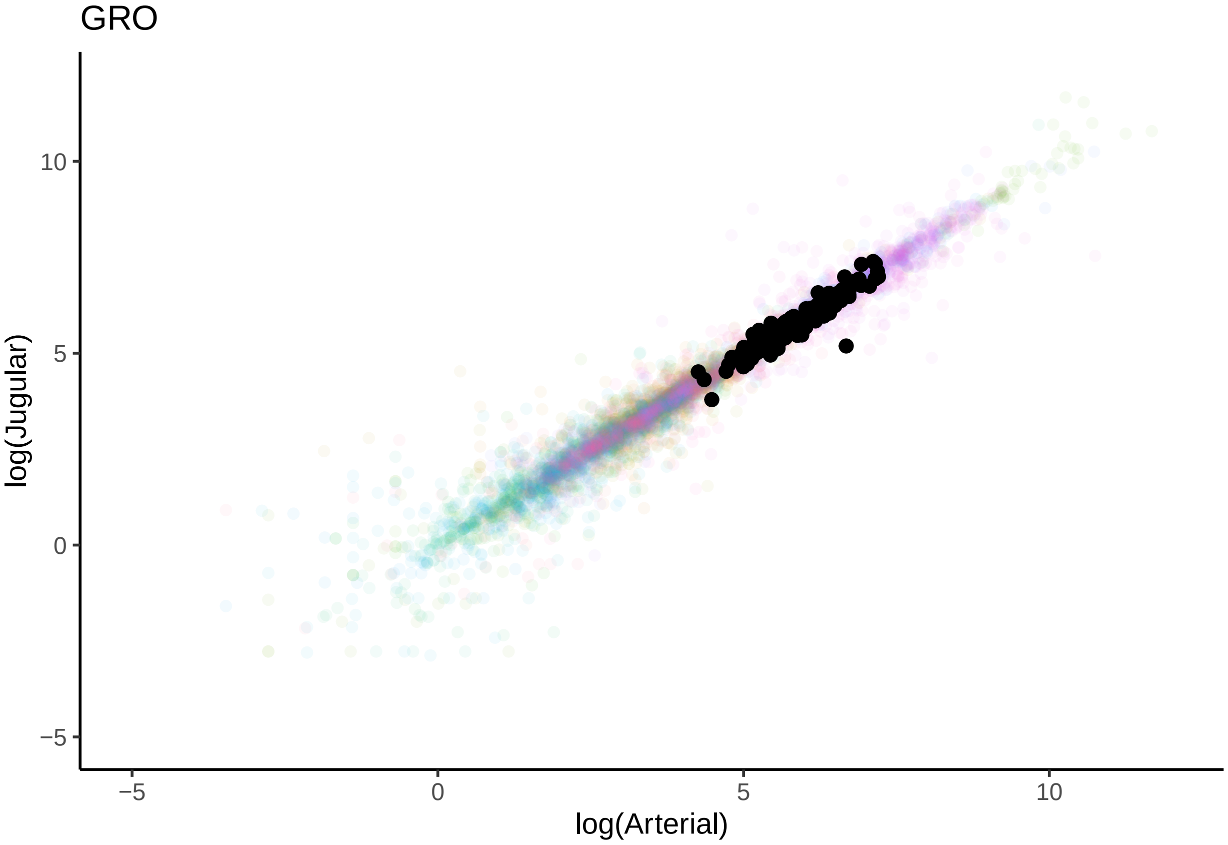


```
## Warning: Removed 10923 rows containing missing values (geom_point).
## Warning: Removed 261 rows containing missing values (geom_point).
```

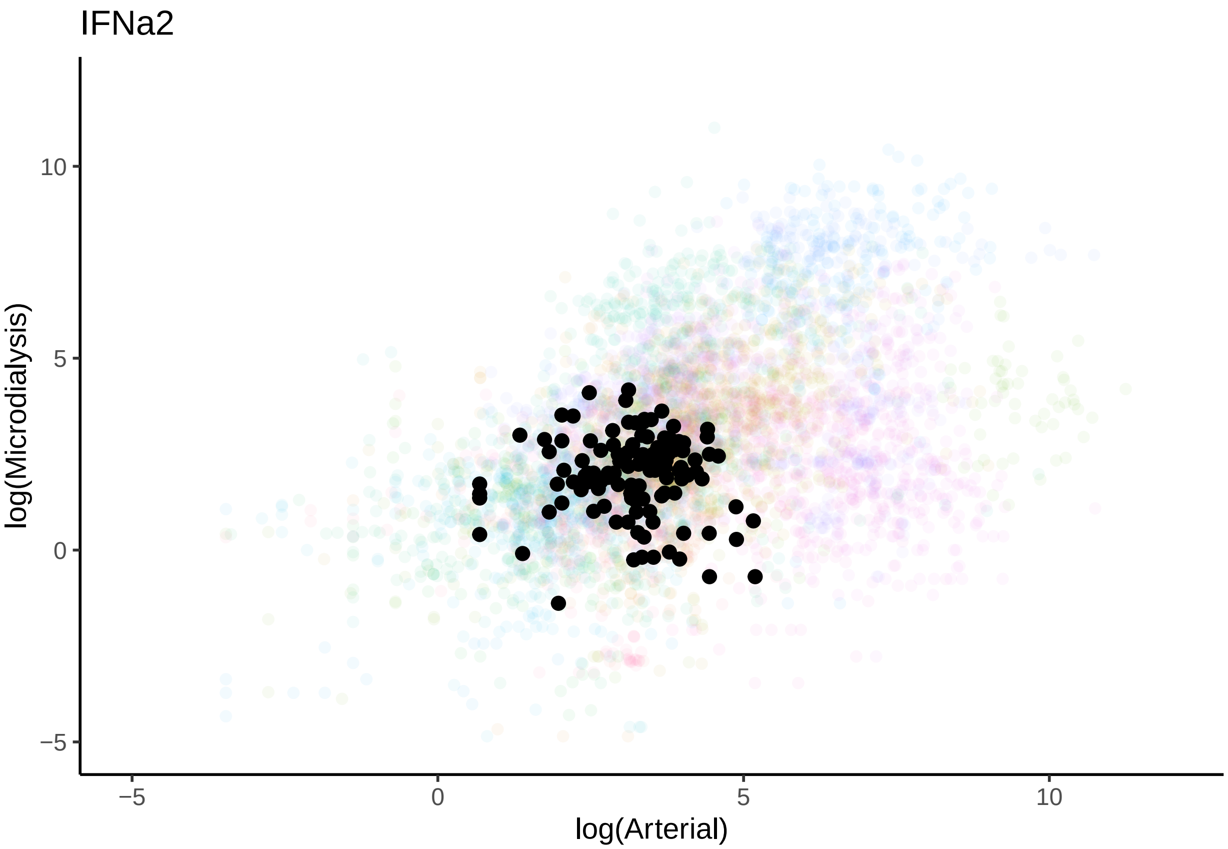


```
## Warning: Removed 10462 rows containing missing values (geom_point).
## Warning: Removed 238 rows containing missing values (geom_point).
```

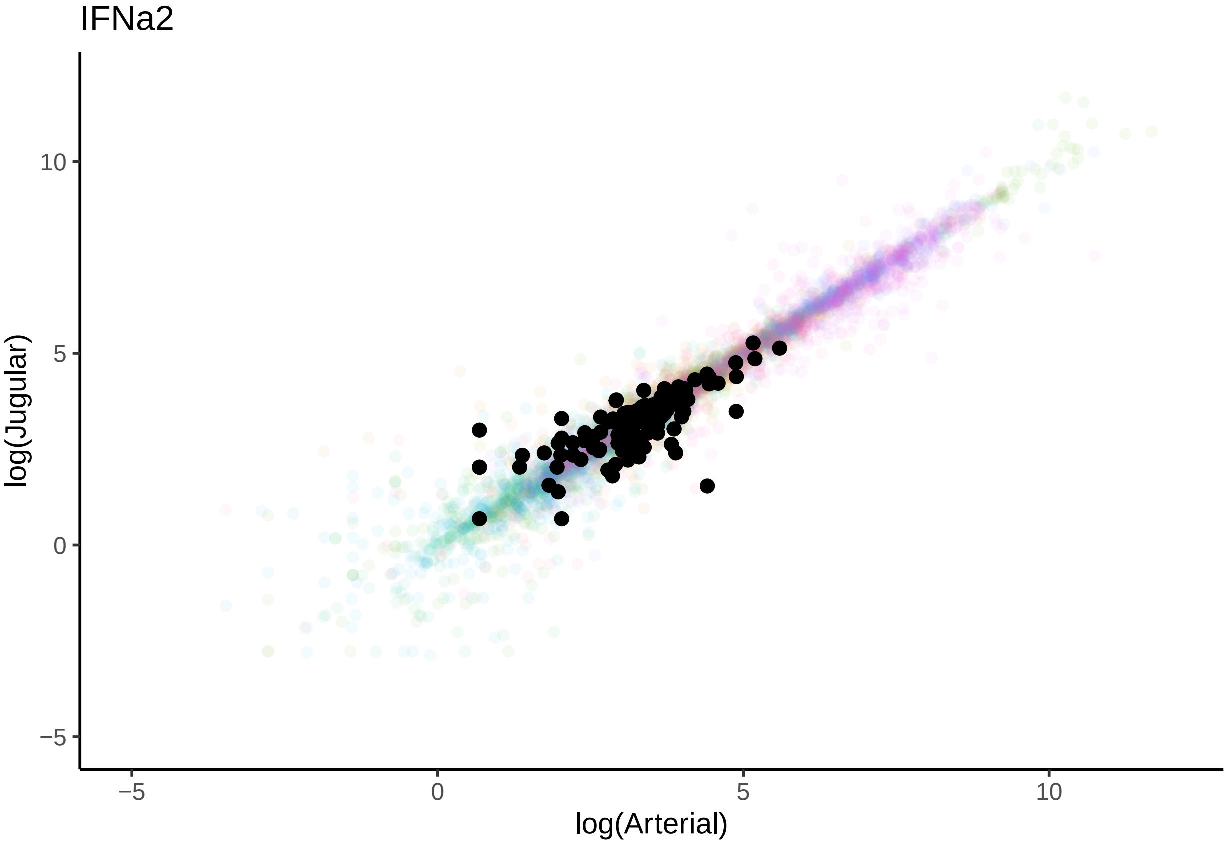


```
## Warning: Removed 10923 rows containing missing values (geom_point).
## Warning: Removed 251 rows containing missing values (geom_point).
```

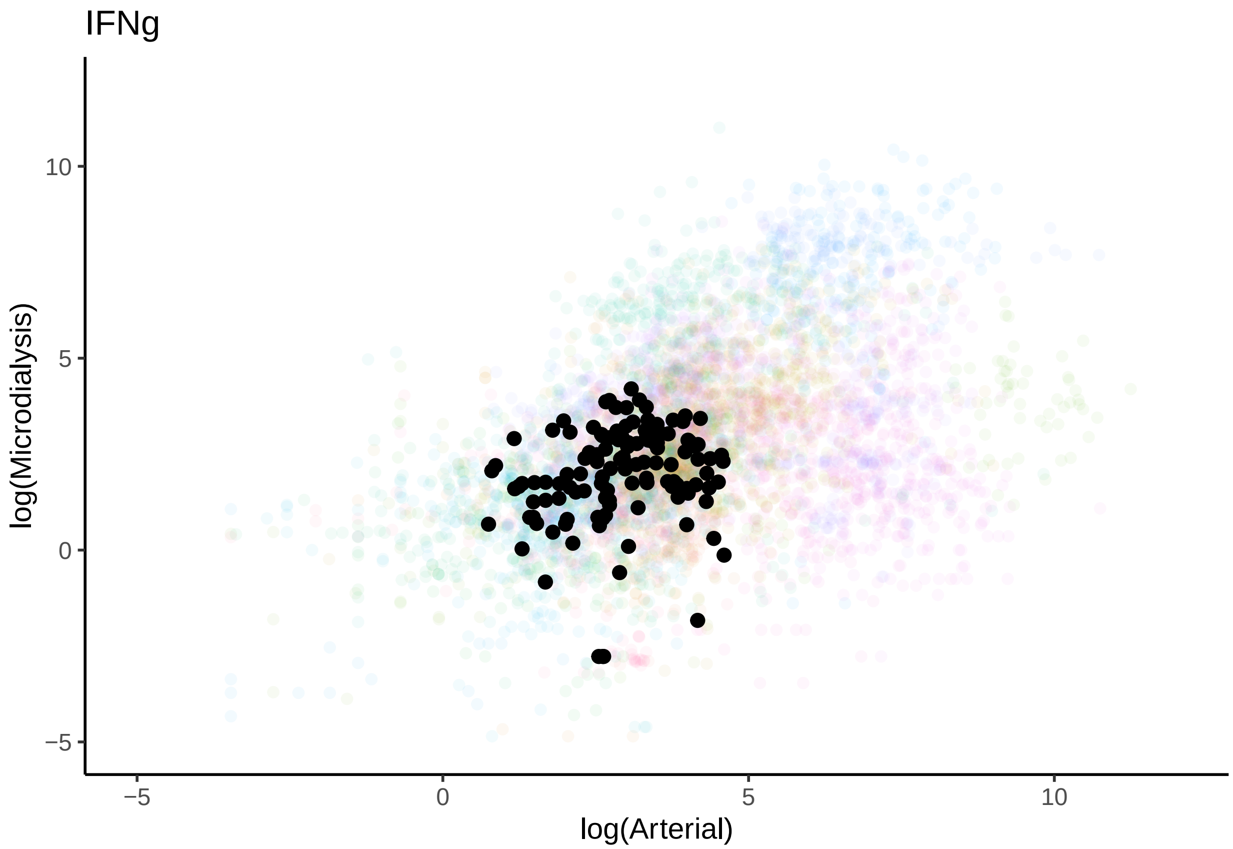


```
## Warning: Removed 10462 rows containing missing values (geom_point).
## Warning: Removed 221 rows containing missing values (geom_point).
```

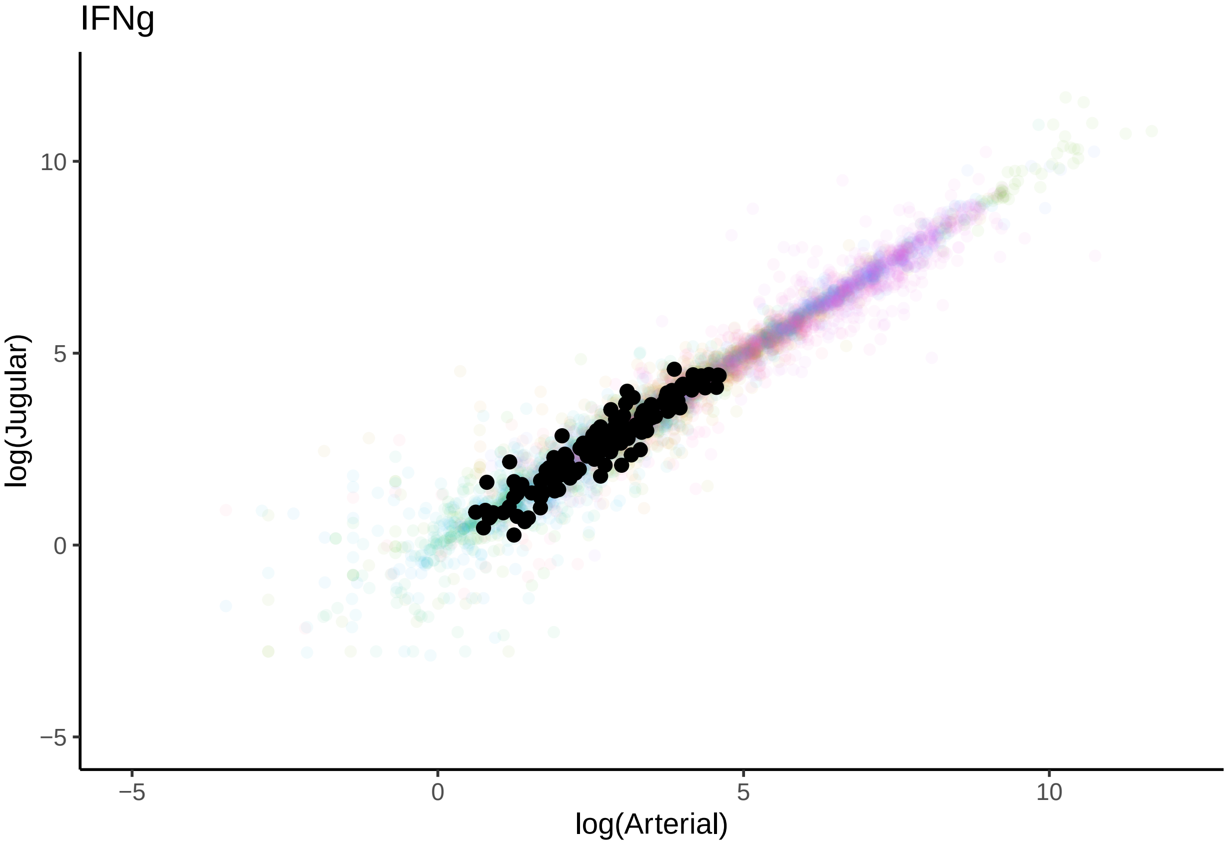


```
## Warning: Removed 10923 rows containing missing values (geom_point).
## Warning: Removed 273 rows containing missing values (geom_point).
```

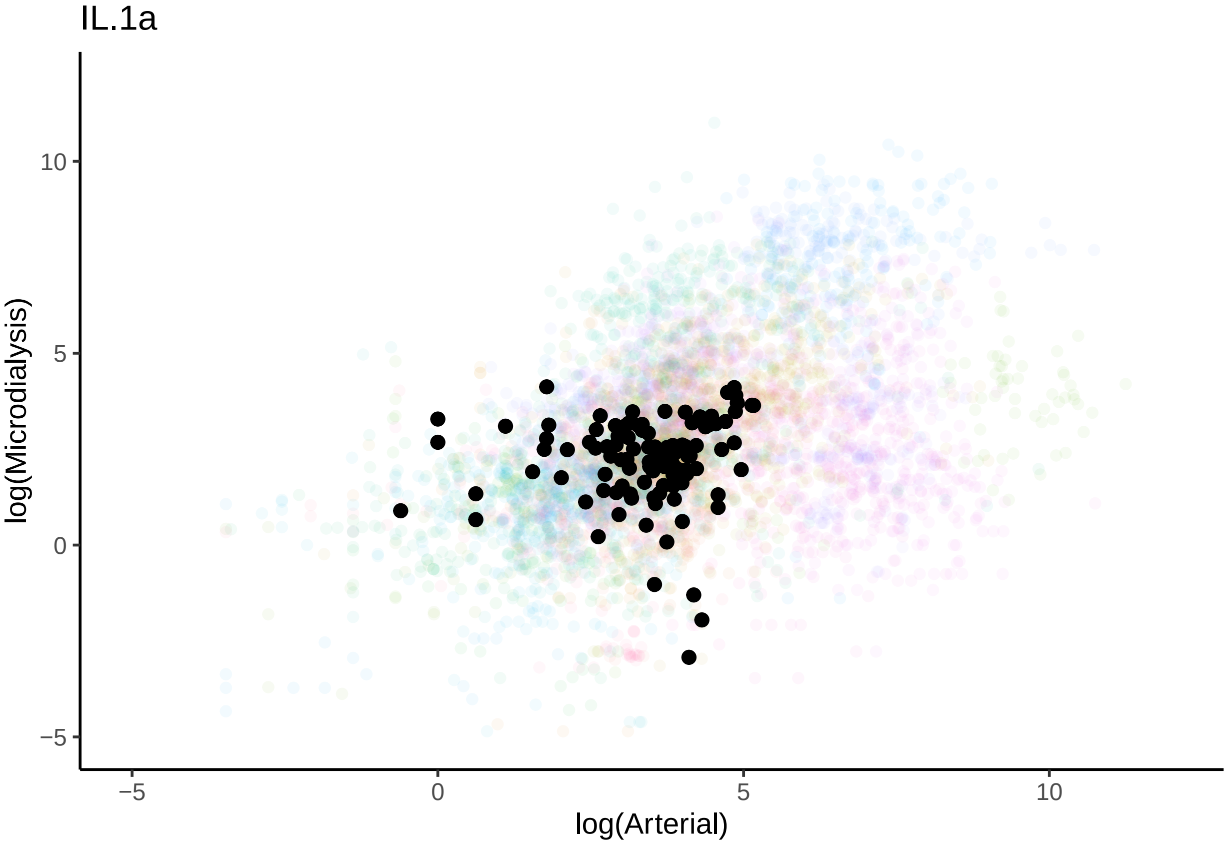


```
## Warning: Removed 10462 rows containing missing values (geom_point).
## Warning: Removed 266 rows containing missing values (geom_point).
```

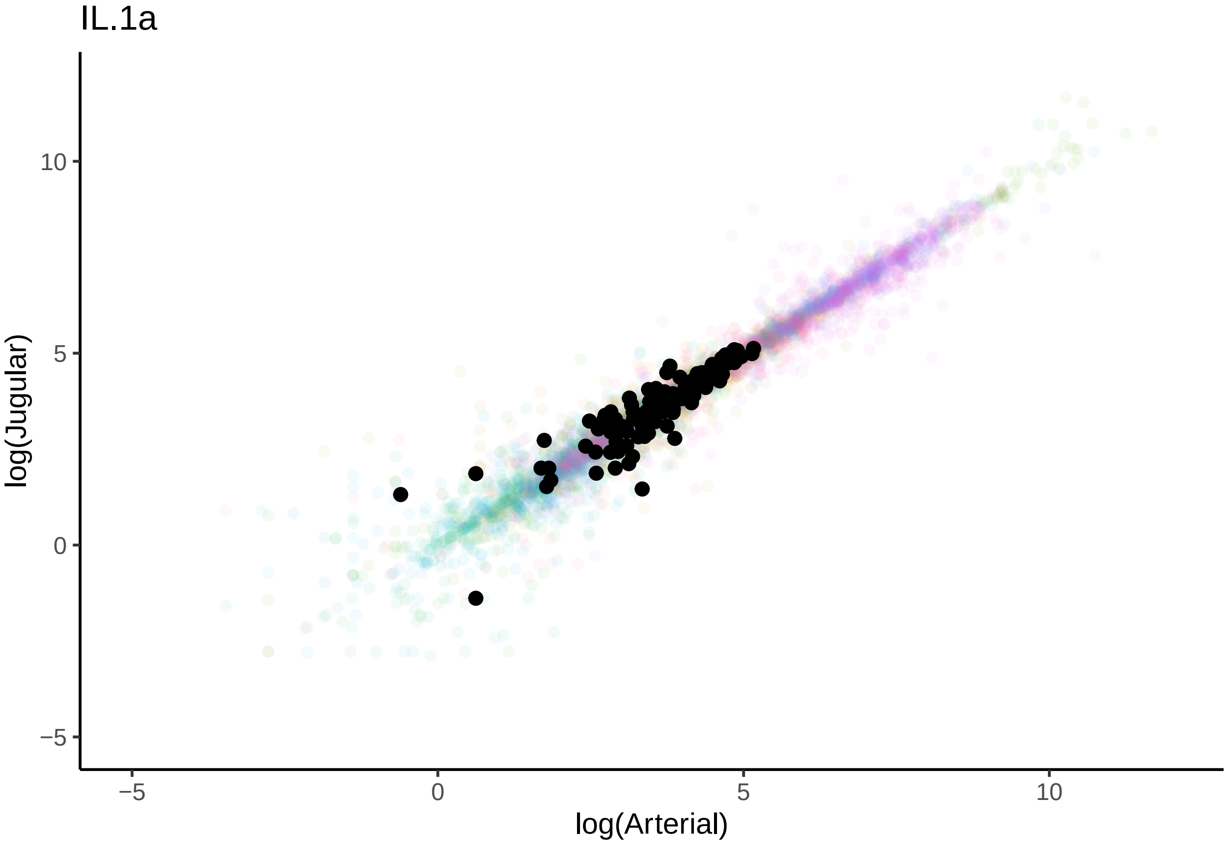


```
## Warning: Removed 10923 rows containing missing values (geom_point).
## Warning: Removed 337 rows containing missing values (geom_point).
```

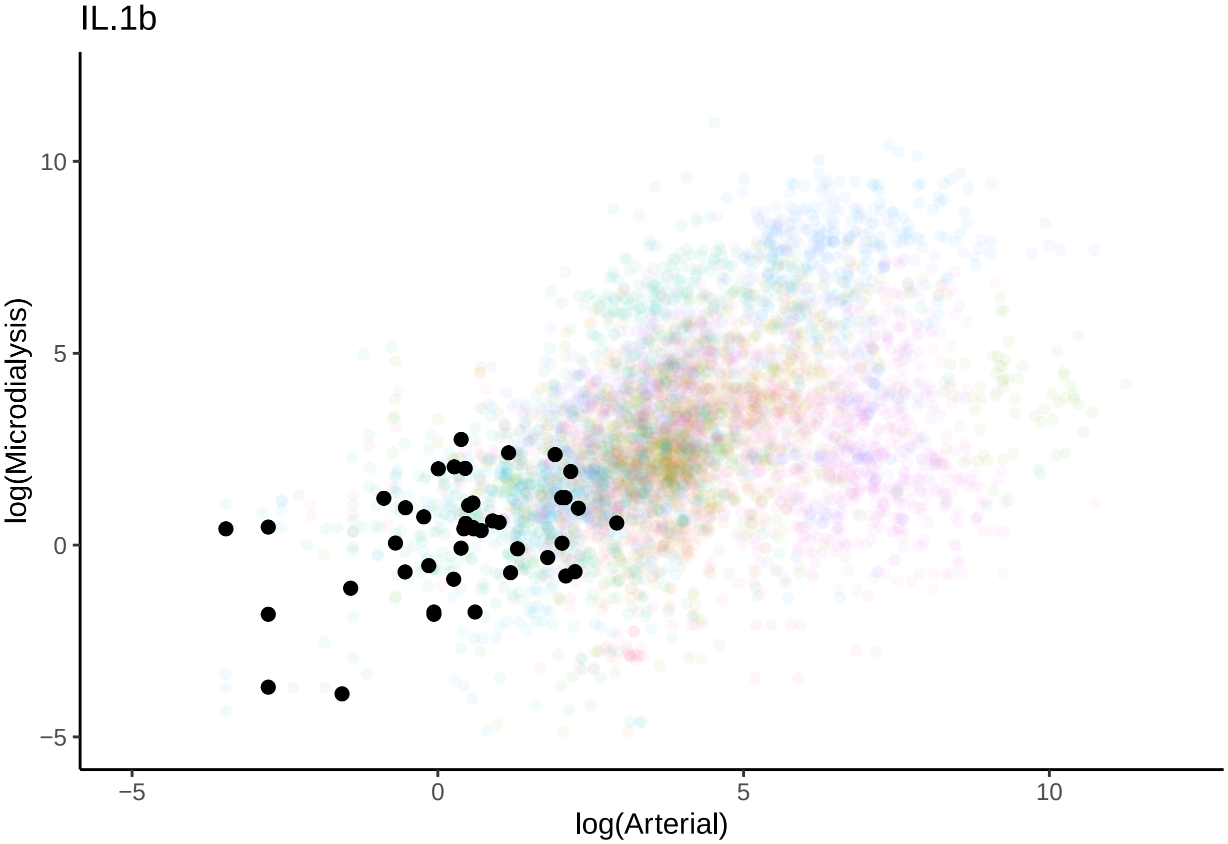


```
## Warning: Removed 10462 rows containing missing values (geom_point).
## Warning: Removed 304 rows containing missing values (geom_point).
```

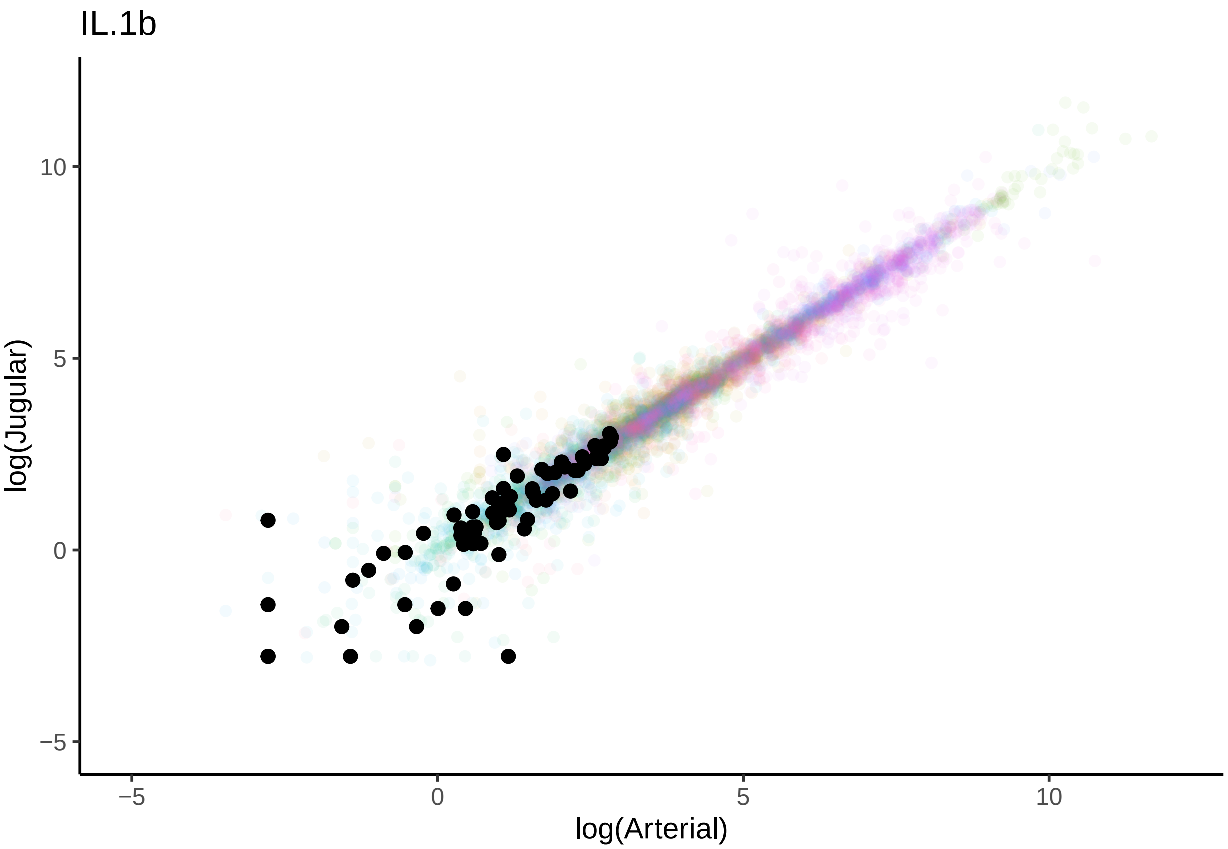


```
## Warning: Removed 10923 rows containing missing values (geom_point).
## Warning: Removed 228 rows containing missing values (geom_point).
```

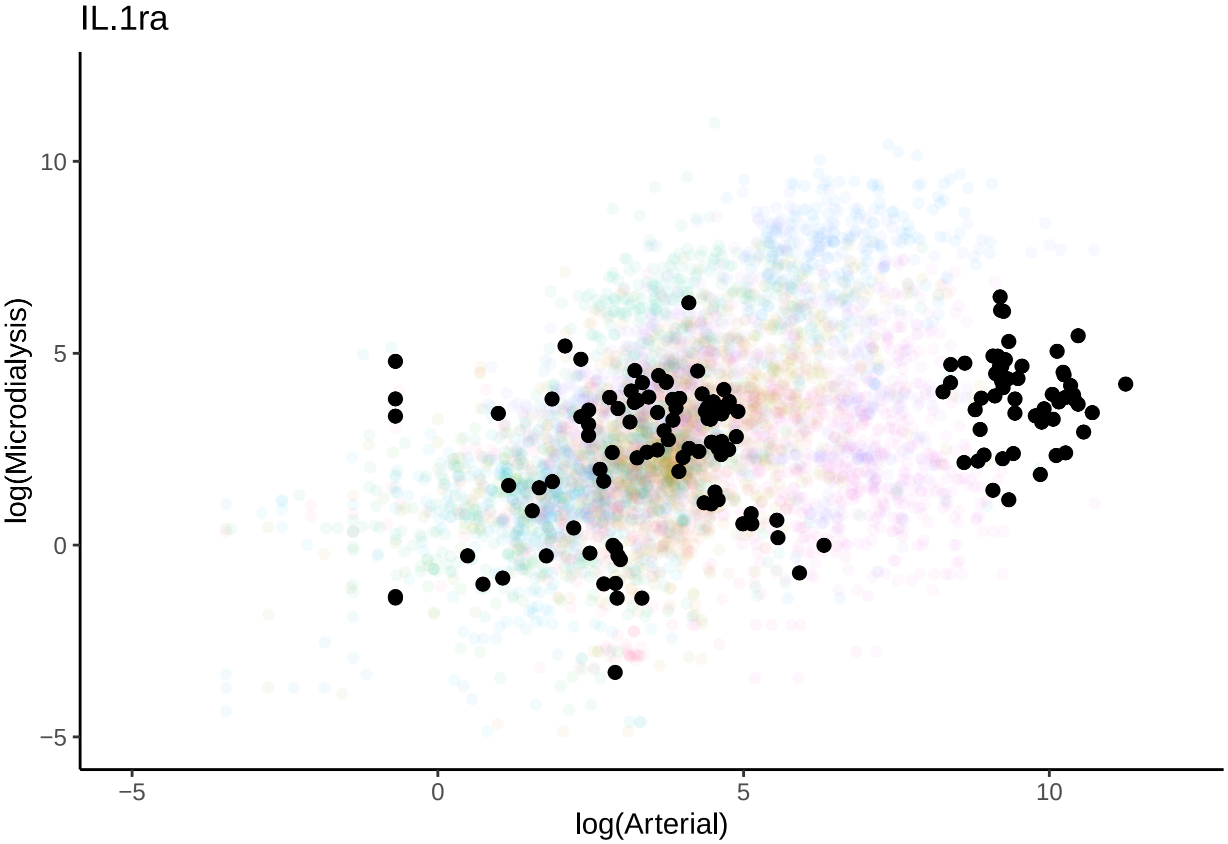


```
## Warning: Removed 10462 rows containing missing values (geom_point).
## Warning: Removed 246 rows containing missing values (geom_point).
```

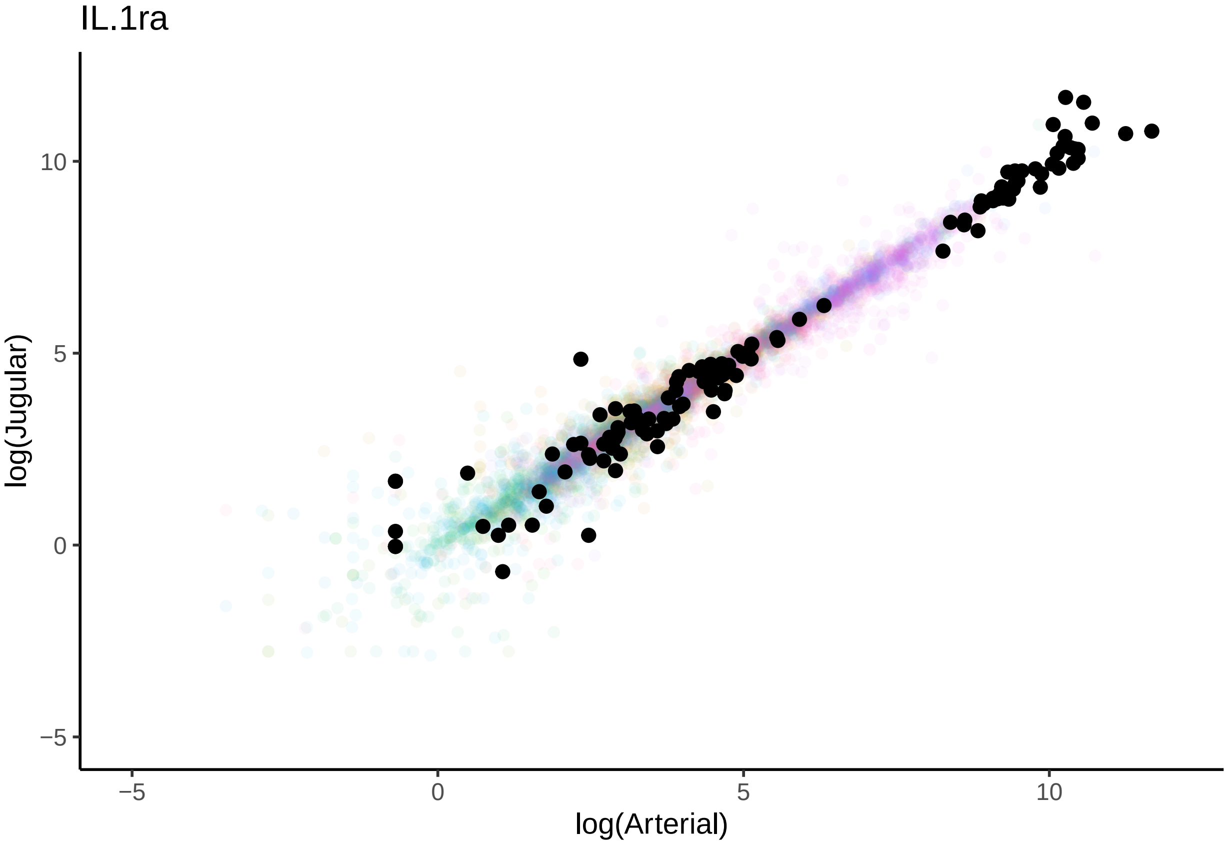


```
## Warning: Removed 10923 rows containing missing values (geom_point).
## Warning: Removed 332 rows containing missing values (geom_point).
```

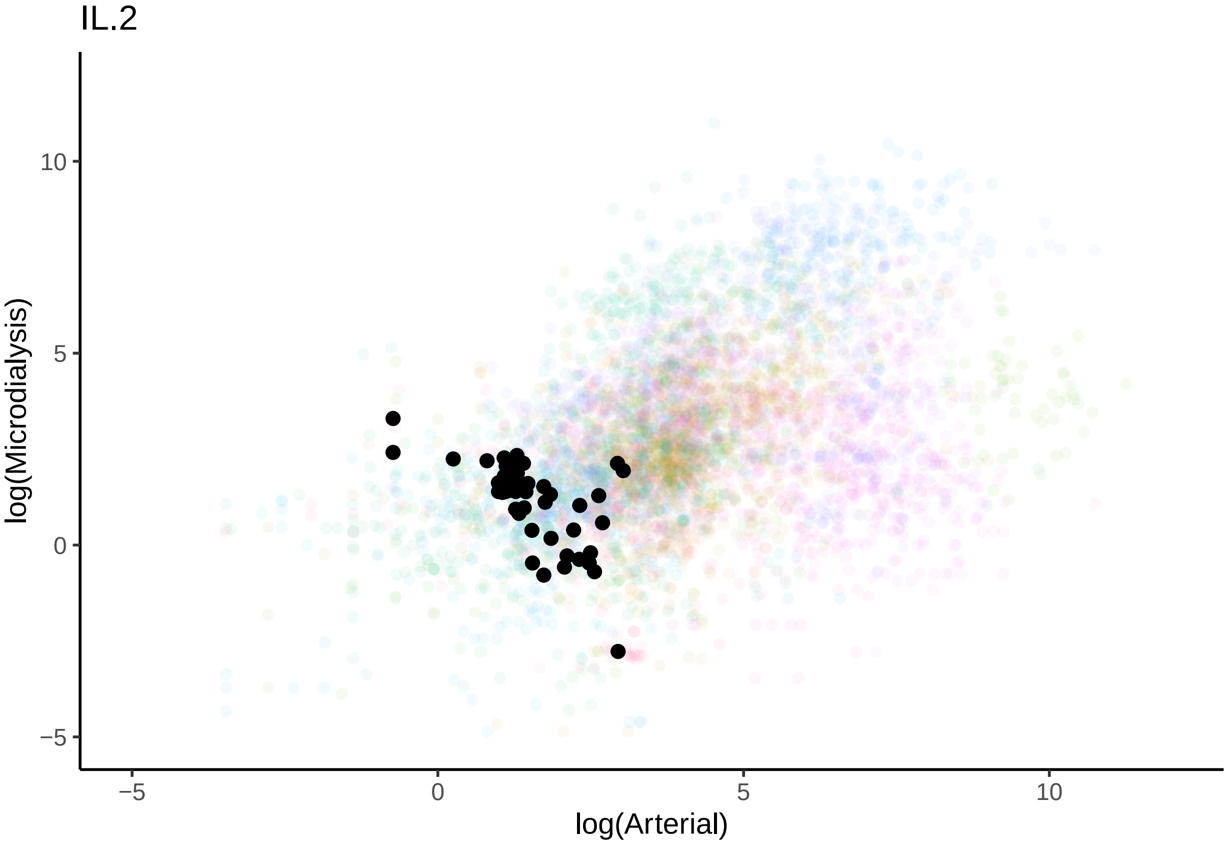


```
## Warning: Removed 10462 rows containing missing values (geom_point).

## Warning: Removed 252 rows containing missing values (geom_point).
```

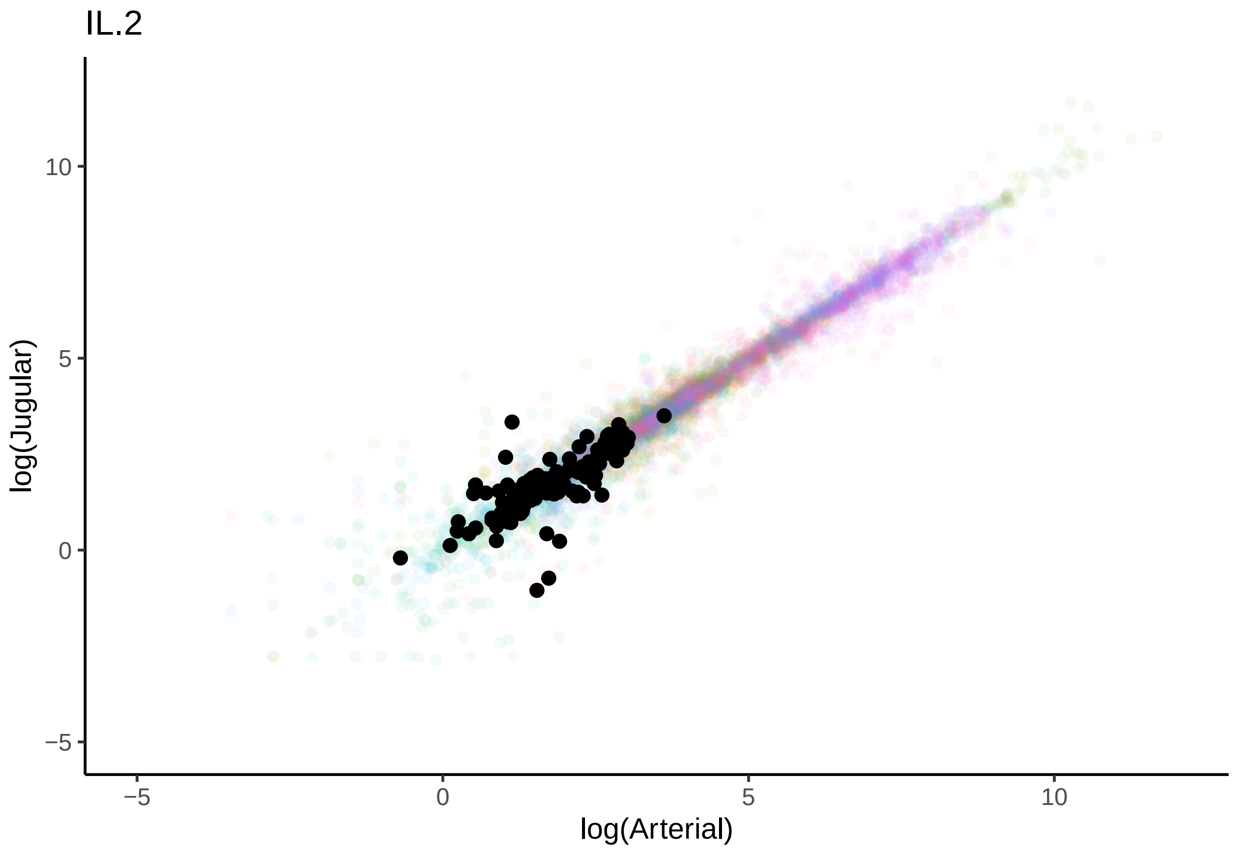


```
## Warning: Removed 10923 rows containing missing values (geom_point).
## Warning: Removed 341 rows containing missing values (geom_point).
```

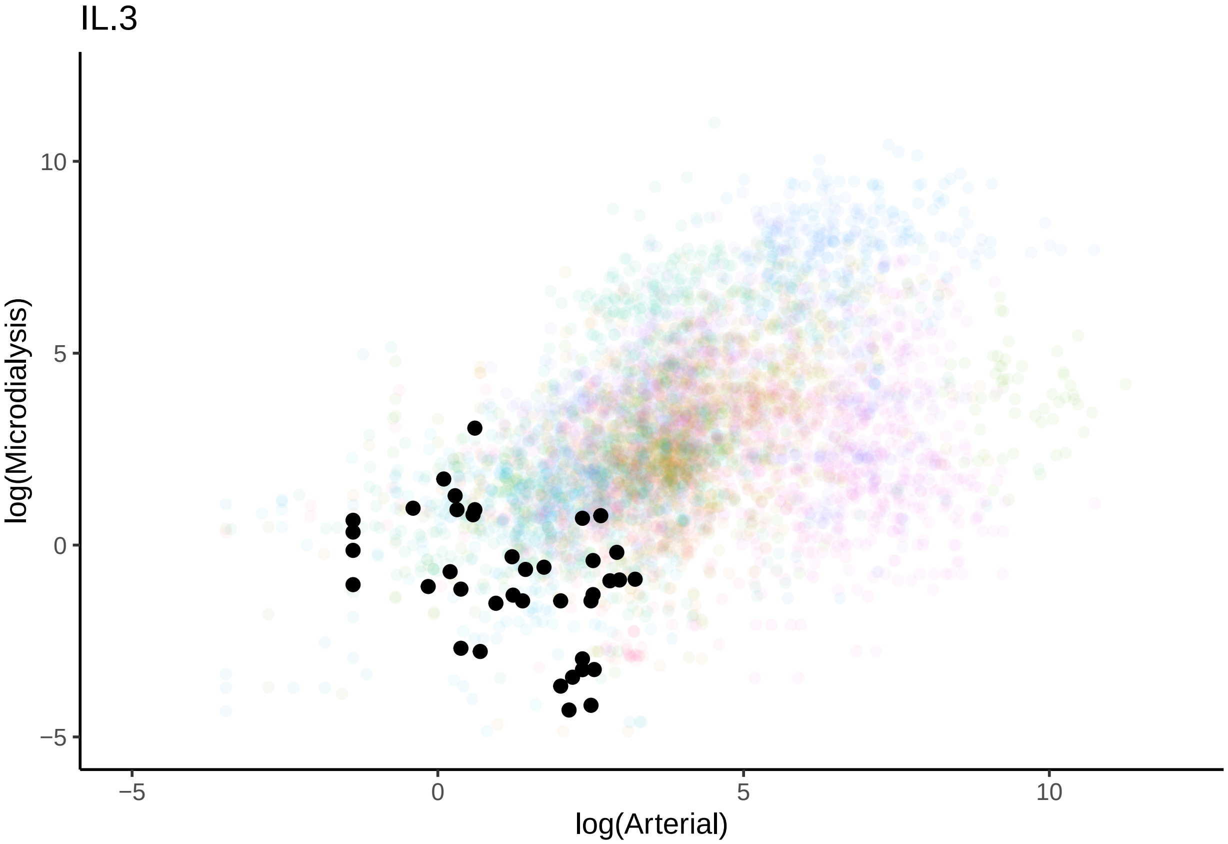


```
## Warning: Removed 10462 rows containing missing values (geom_point).
## Warning: Removed 320 rows containing missing values (geom_point).
```

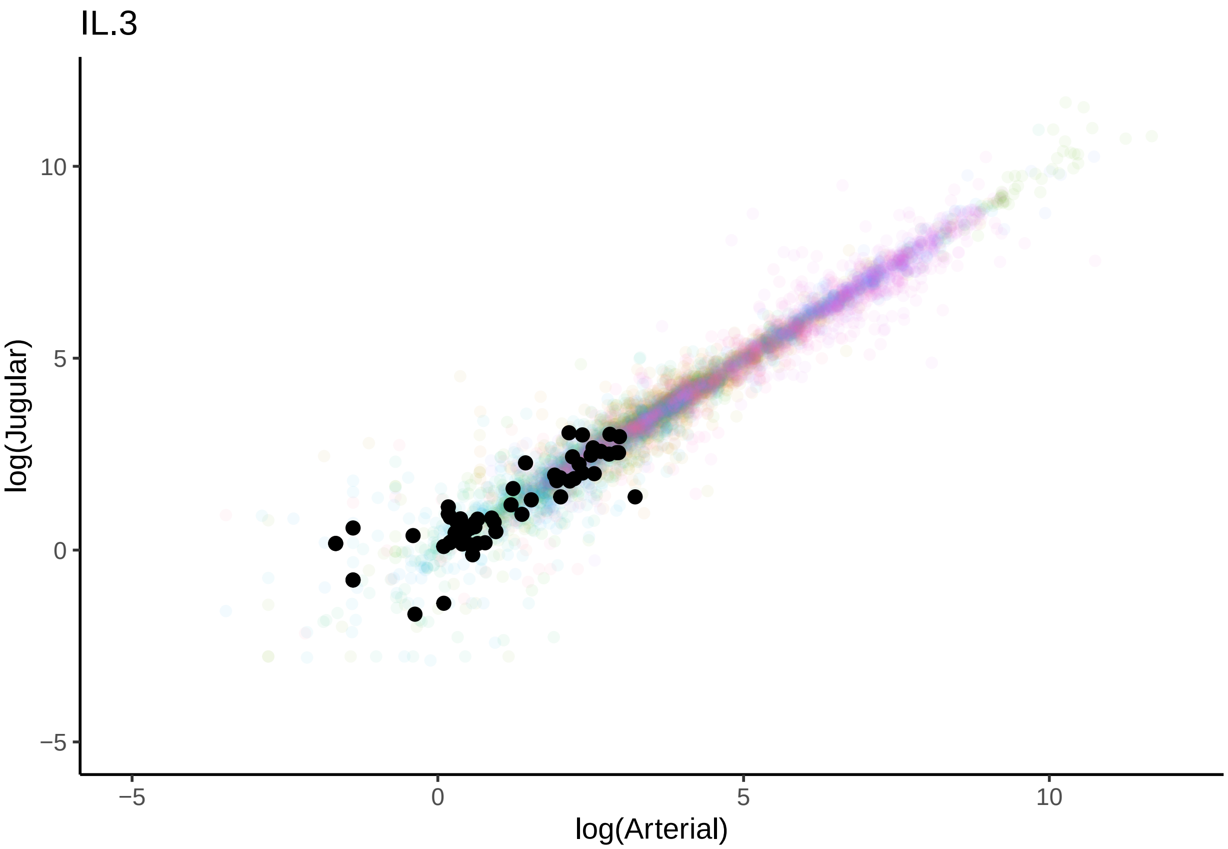


```
## Warning: Removed 10923 rows containing missing values (geom_point).

## Warning: Removed 365 rows containing missing values (geom_point).
```

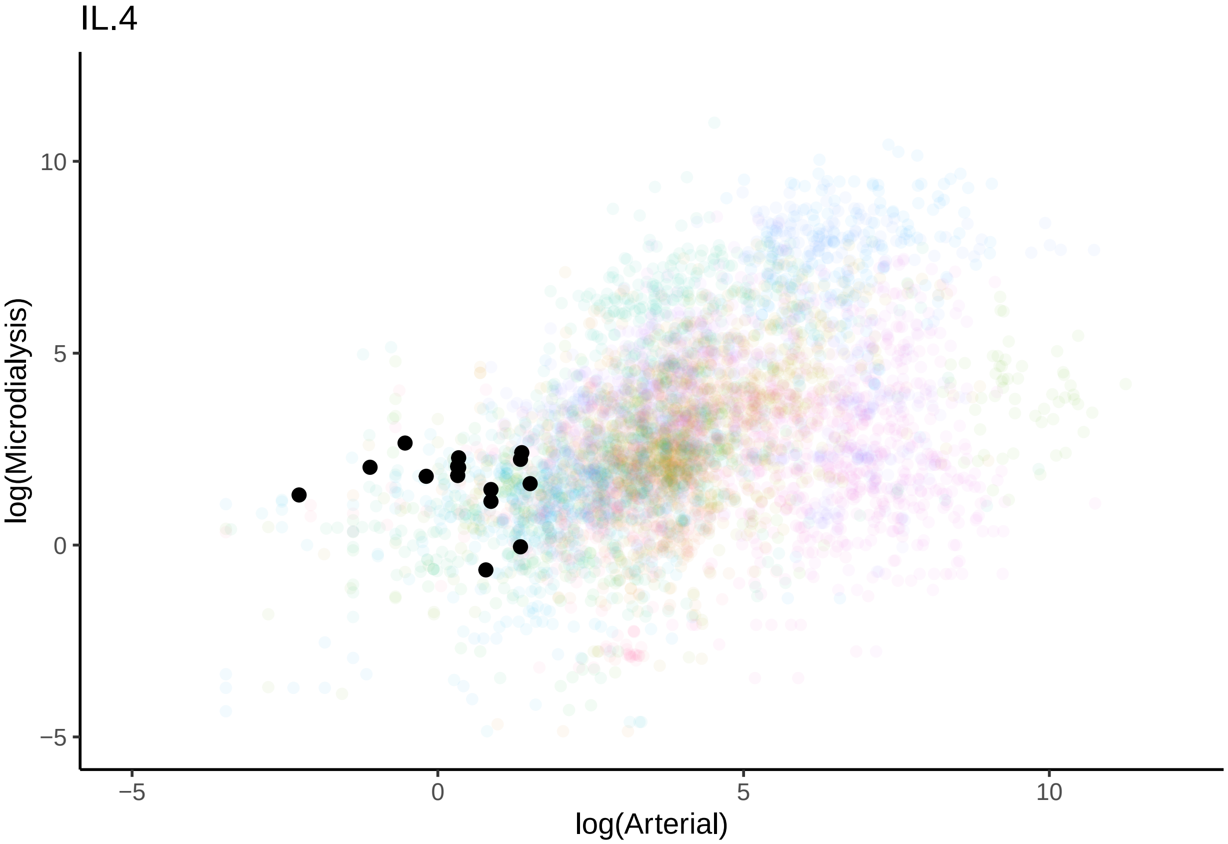


```
## Warning: Removed 10462 rows containing missing values (geom_point).

## Warning: Removed 368 rows containing missing values (geom_point).
```

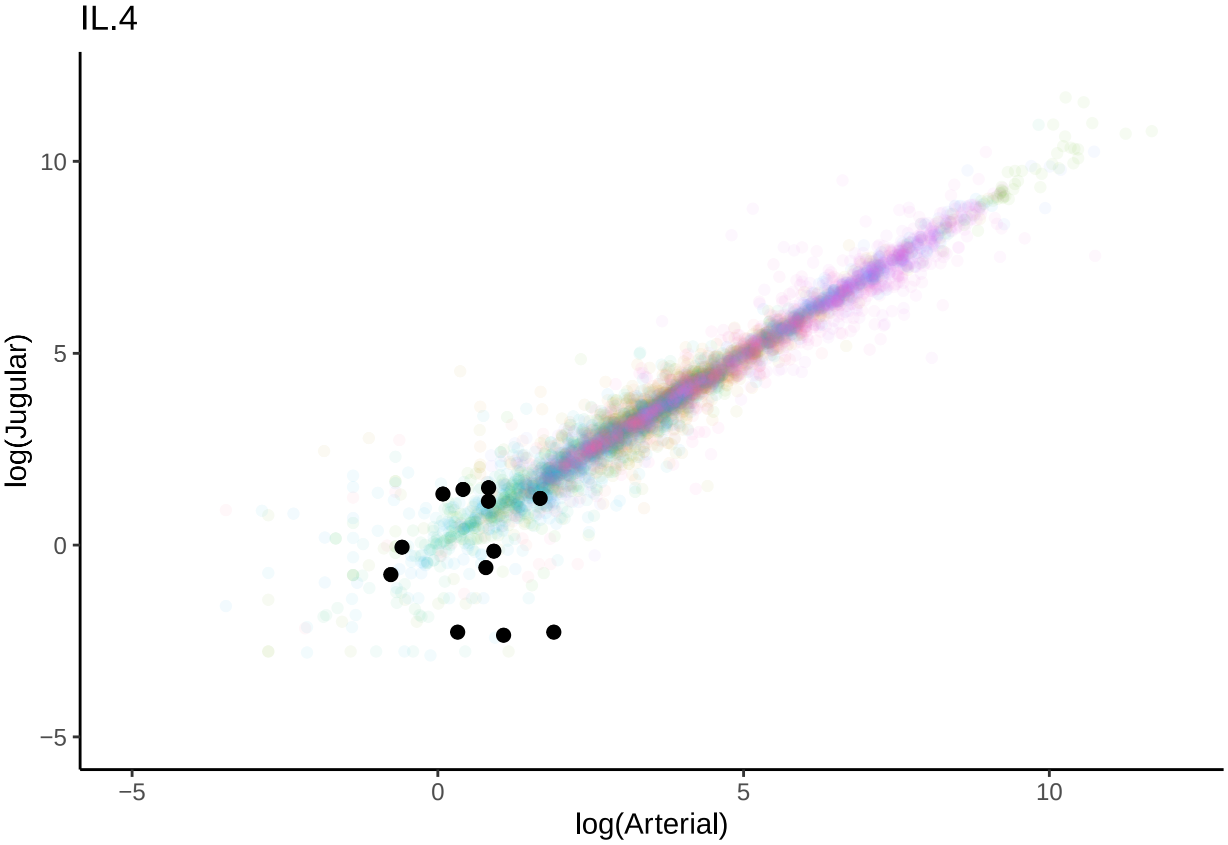


```
## Warning: Removed 10923 rows containing missing values (geom_point).

## Warning: Removed 308 rows containing missing values (geom_point).
```

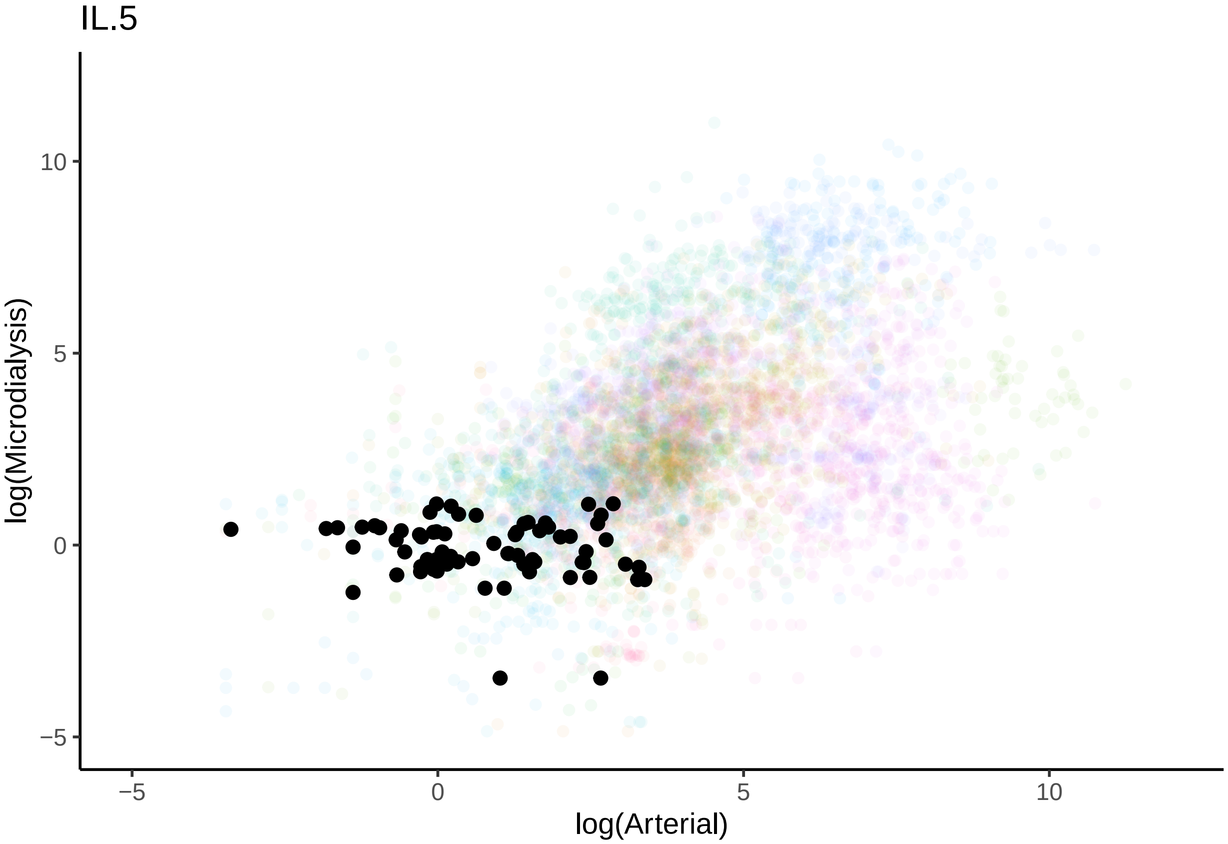


```
## Warning: Removed 10462 rows containing missing values (geom_point).
## Warning: Removed 286 rows containing missing values (geom_point).
```

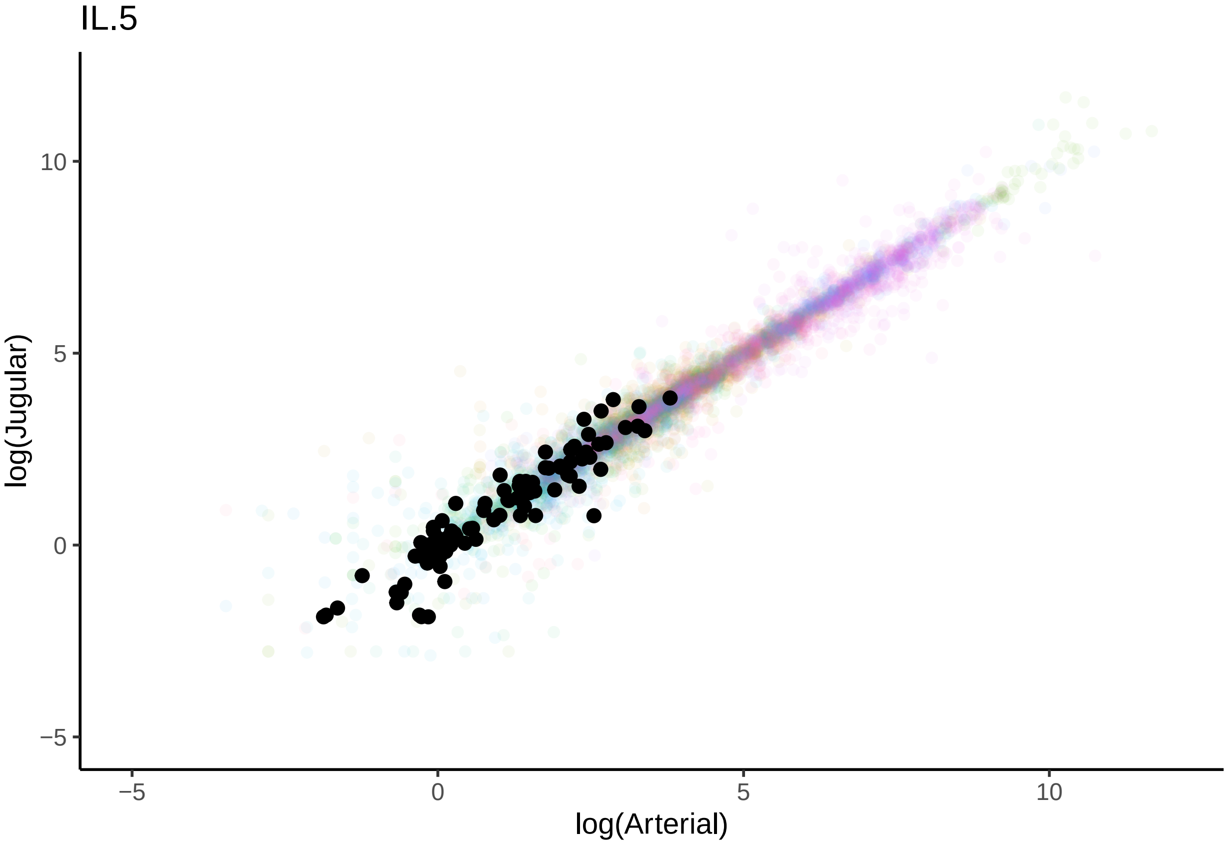


```
## Warning: Removed 10923 rows containing missing values (geom_point).
## Warning: Removed 200 rows containing missing values (geom_point).
```

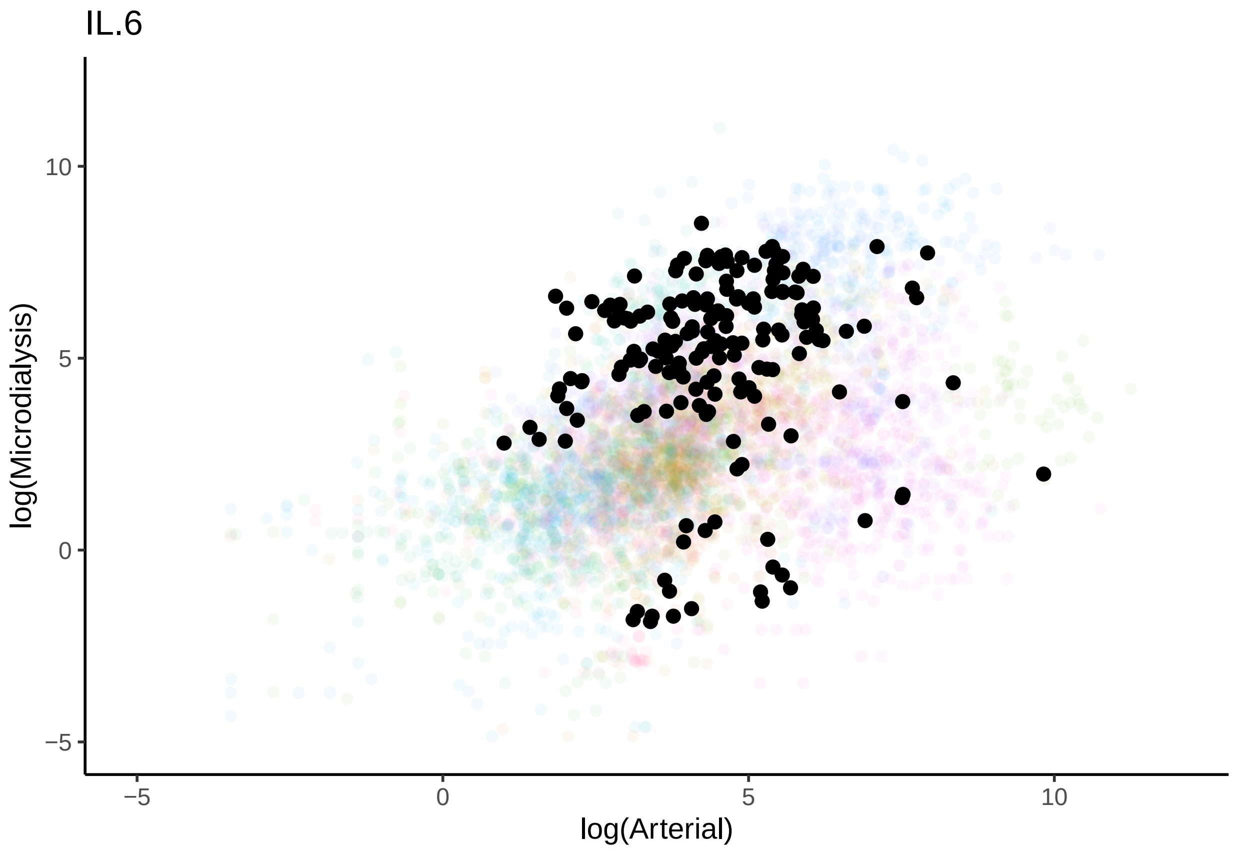


```
## Warning: Removed 10462 rows containing missing values (geom_point).
## Warning: Removed 214 rows containing missing values (geom_point).
## Warning: Removed 10923 rows containing missing values (geom_point).
## Warning: Removed 200 rows containing missing values (geom_point).
## Warning: Removed 10462 rows containing missing values (geom_point).
## Warning: Removed 214 rows containing missing values (geom_point).
```

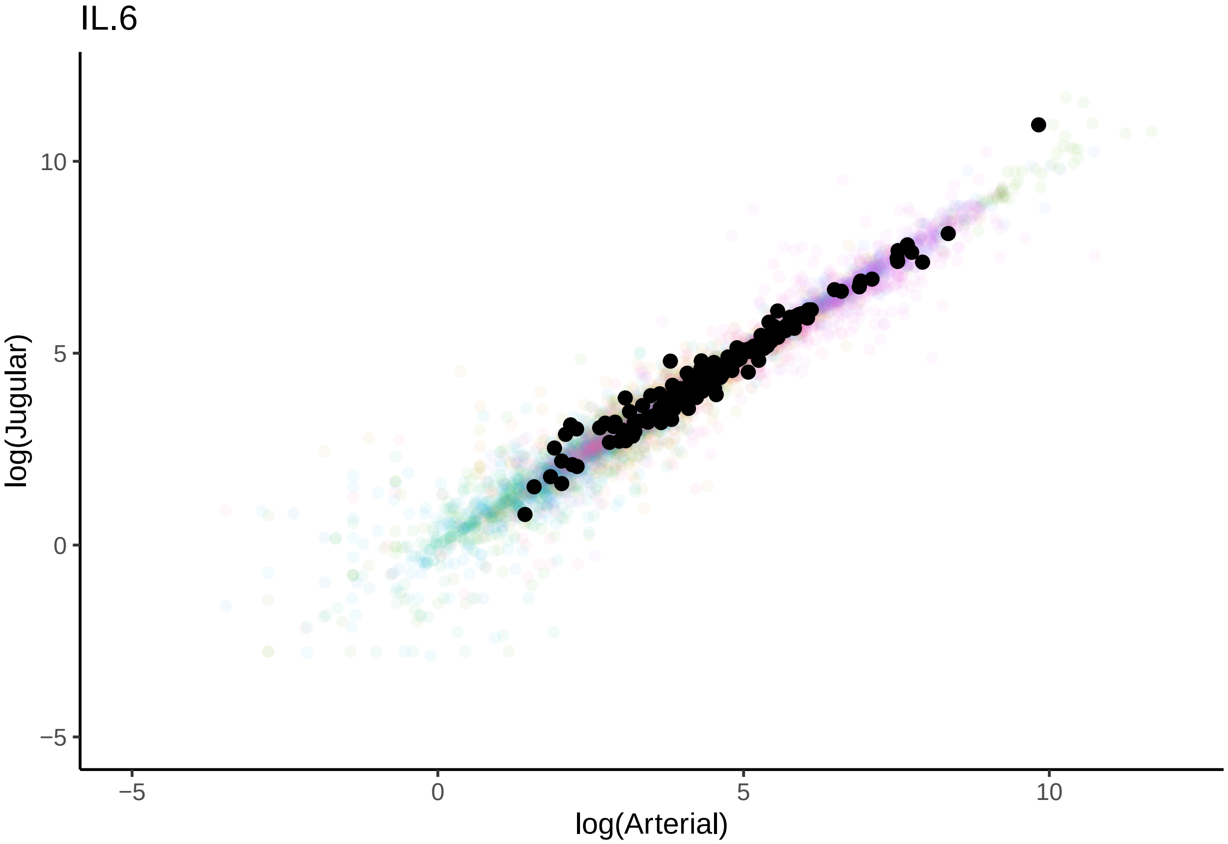


```
## Warning: Removed 10923 rows containing missing values (geom_point).
## Warning: Removed 304 rows containing missing values (geom_point).
```

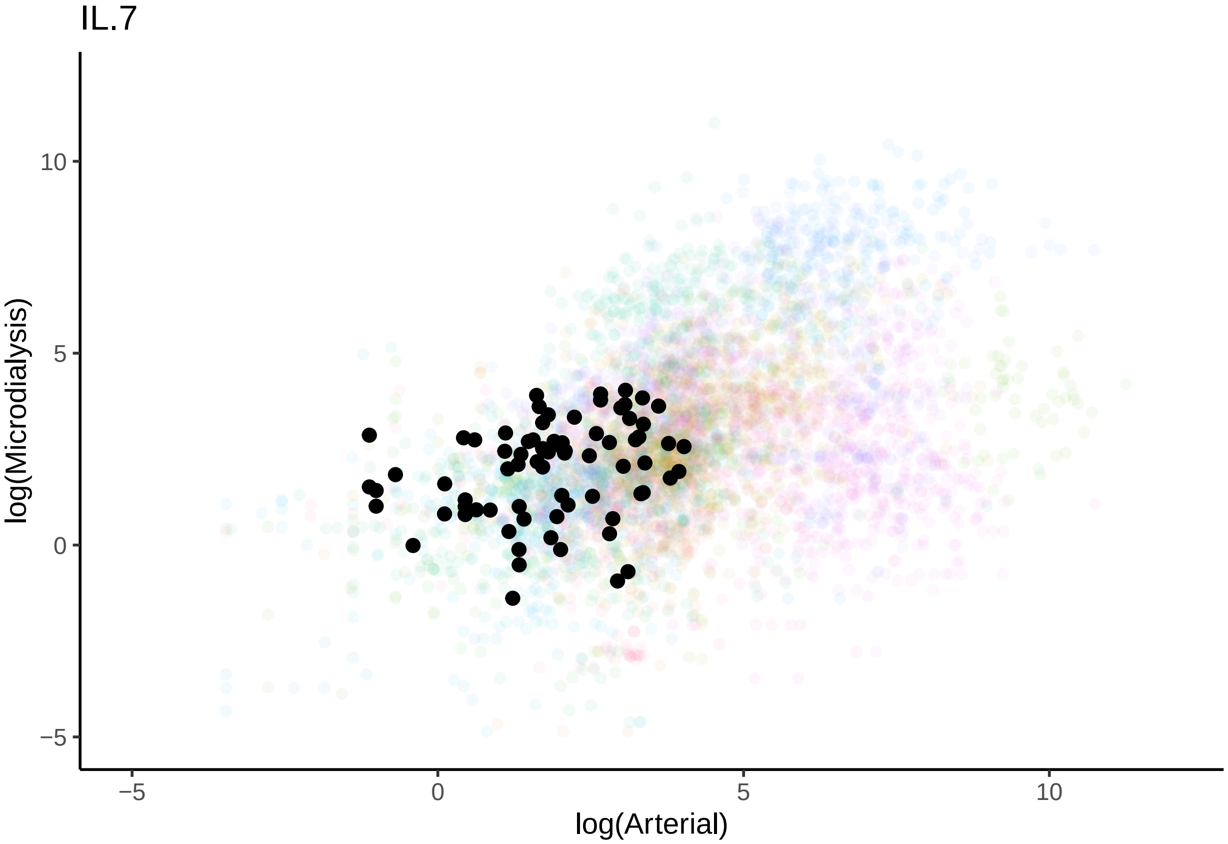


```
## Warning: Removed 10462 rows containing missing values (geom_point).

## Warning: Removed 304 rows containing missing values (geom_point).
```

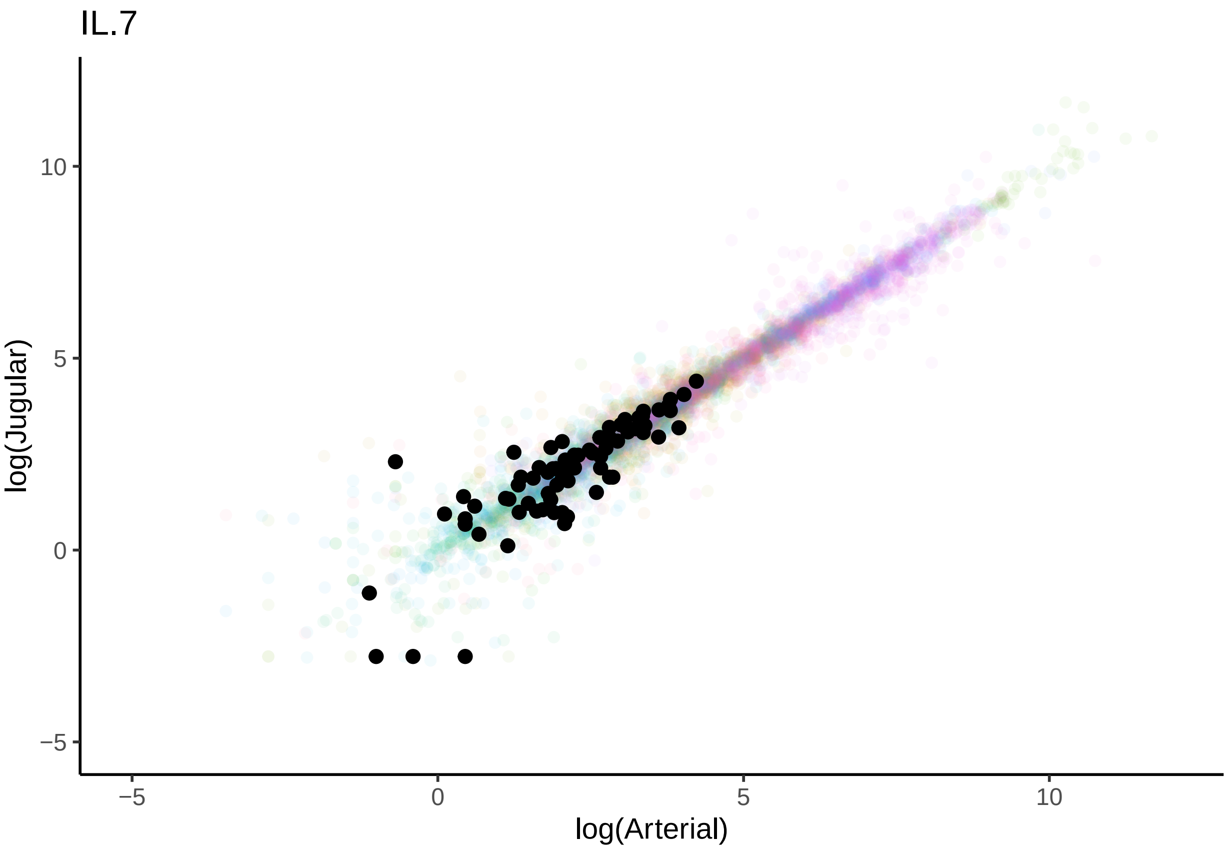


```
## Warning: Removed 10923 rows containing missing values (geom_point).
## Warning: Removed 202 rows containing missing values (geom_point).
```

IL.8

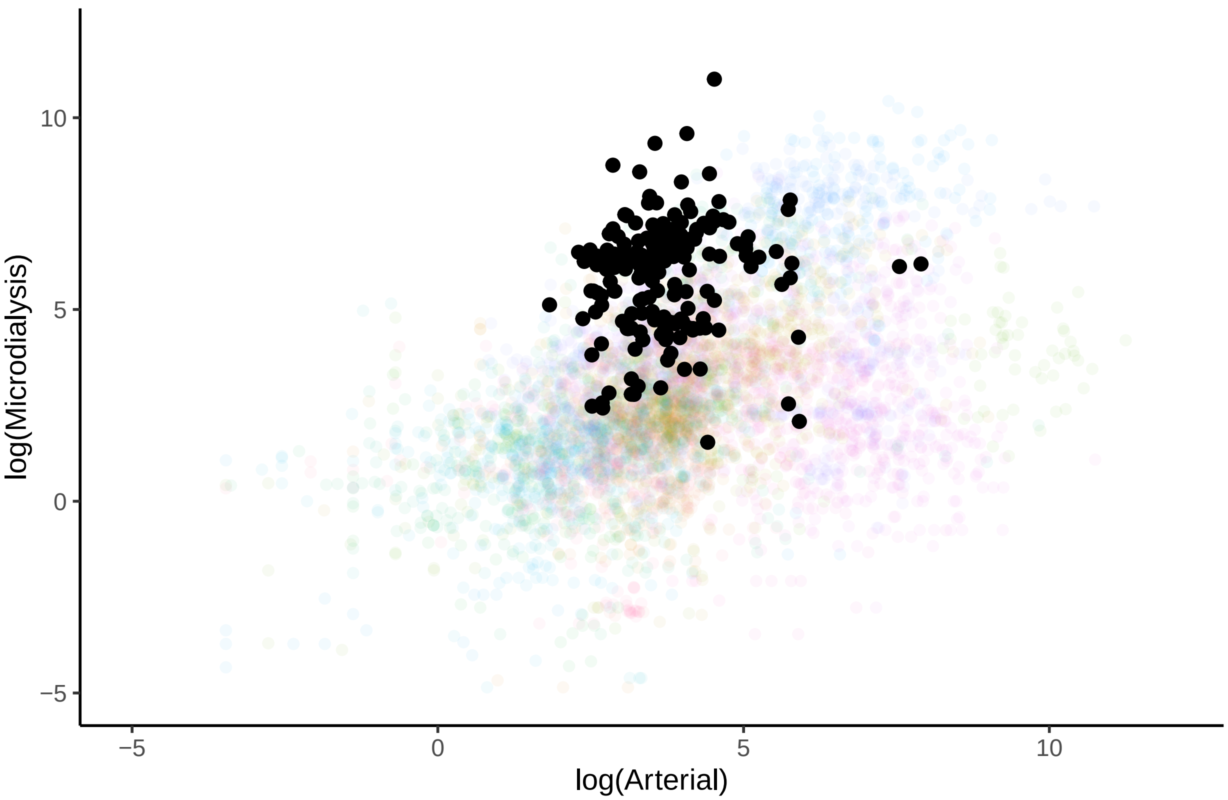


```
## Warning: Removed 10462 rows containing missing values (geom_point).
## Warning: Removed 213 rows containing missing values (geom_point).
```

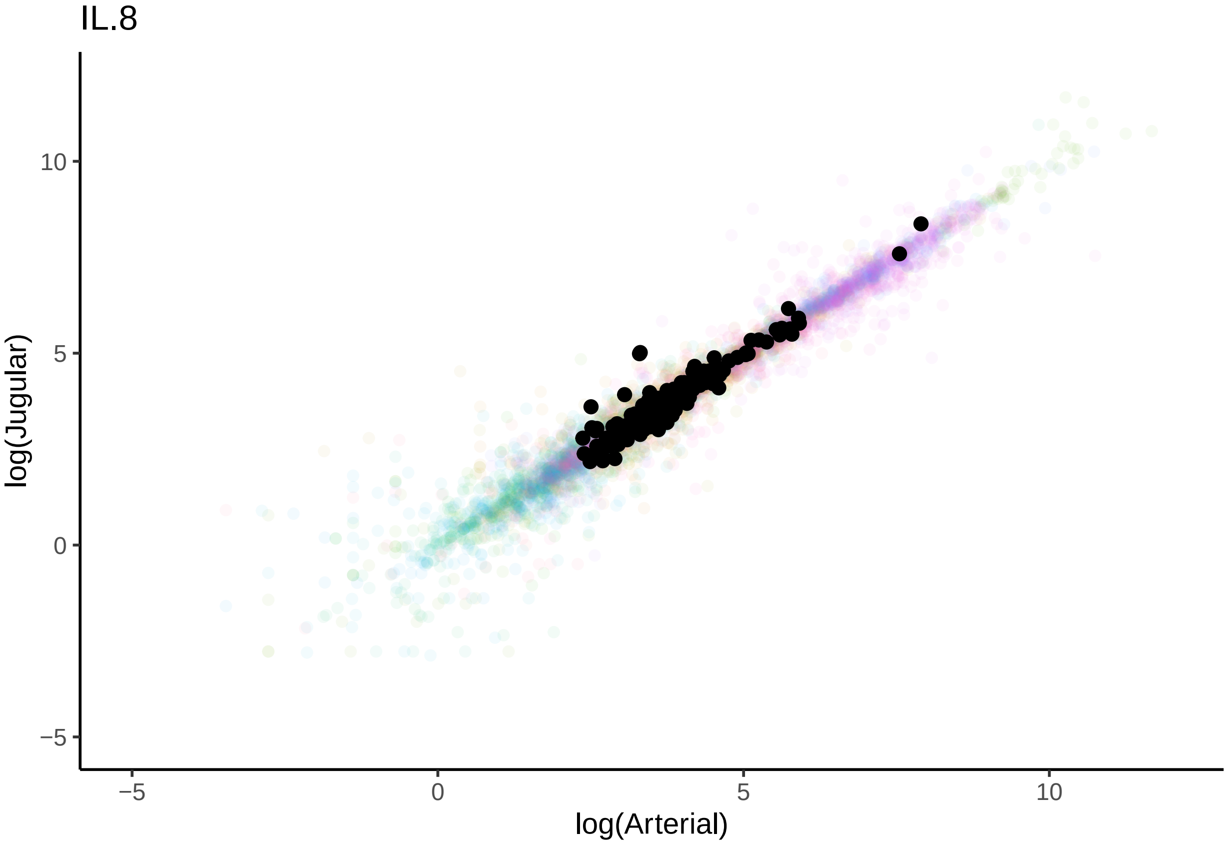


```
## Warning: Removed 10923 rows containing missing values (geom_point).

## Warning: Removed 345 rows containing missing values (geom_point).
```

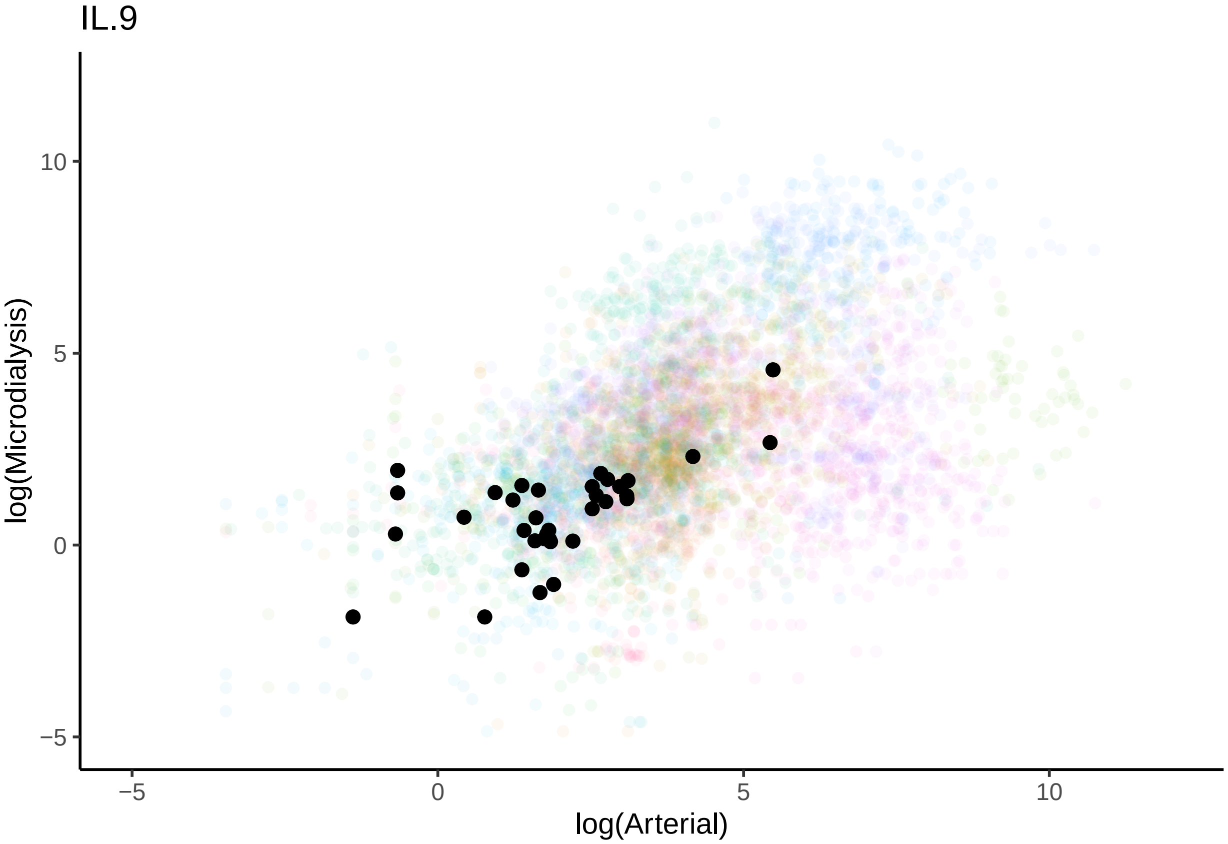


```
## Warning: Removed 10462 rows containing missing values (geom_point).

## Warning: Removed 340 rows containing missing values (geom_point).
```

IL.9

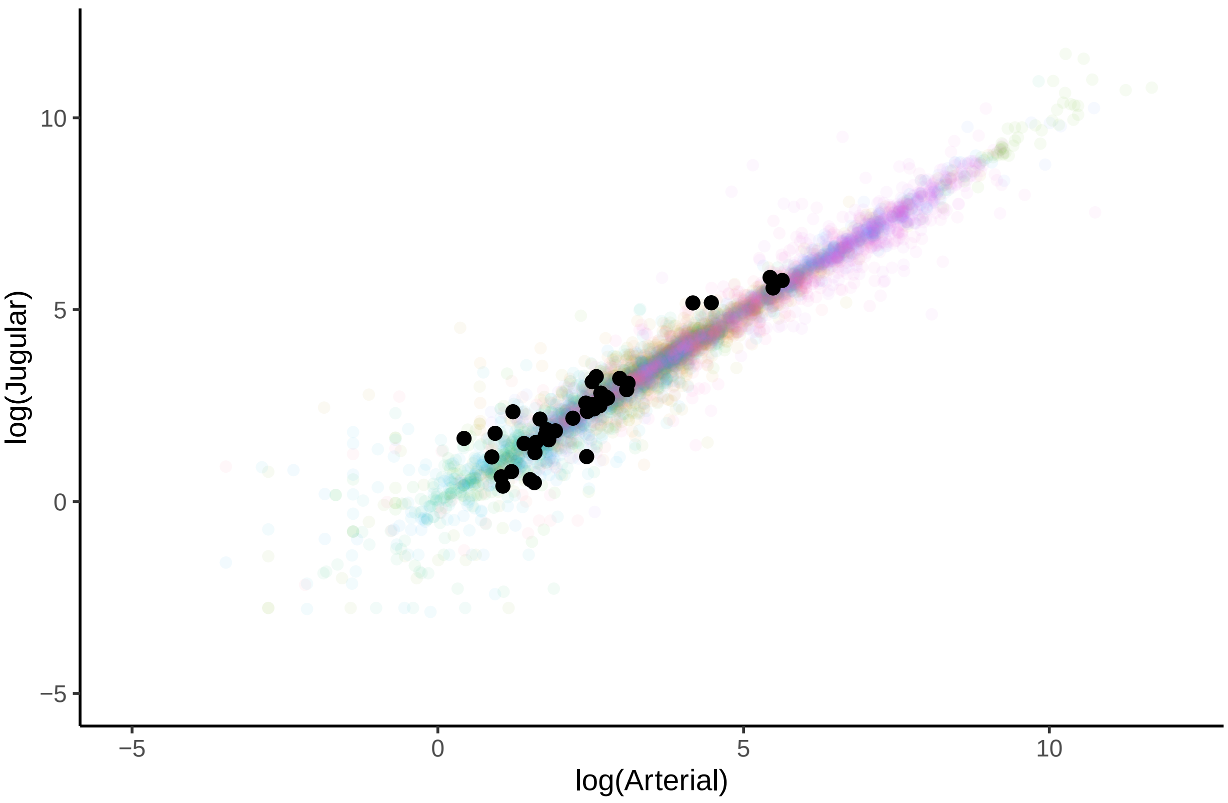


```
## Warning: Removed 10923 rows containing missing values (geom_point).
```

```
## Warning: Removed 225 rows containing missing values (geom_point).
```

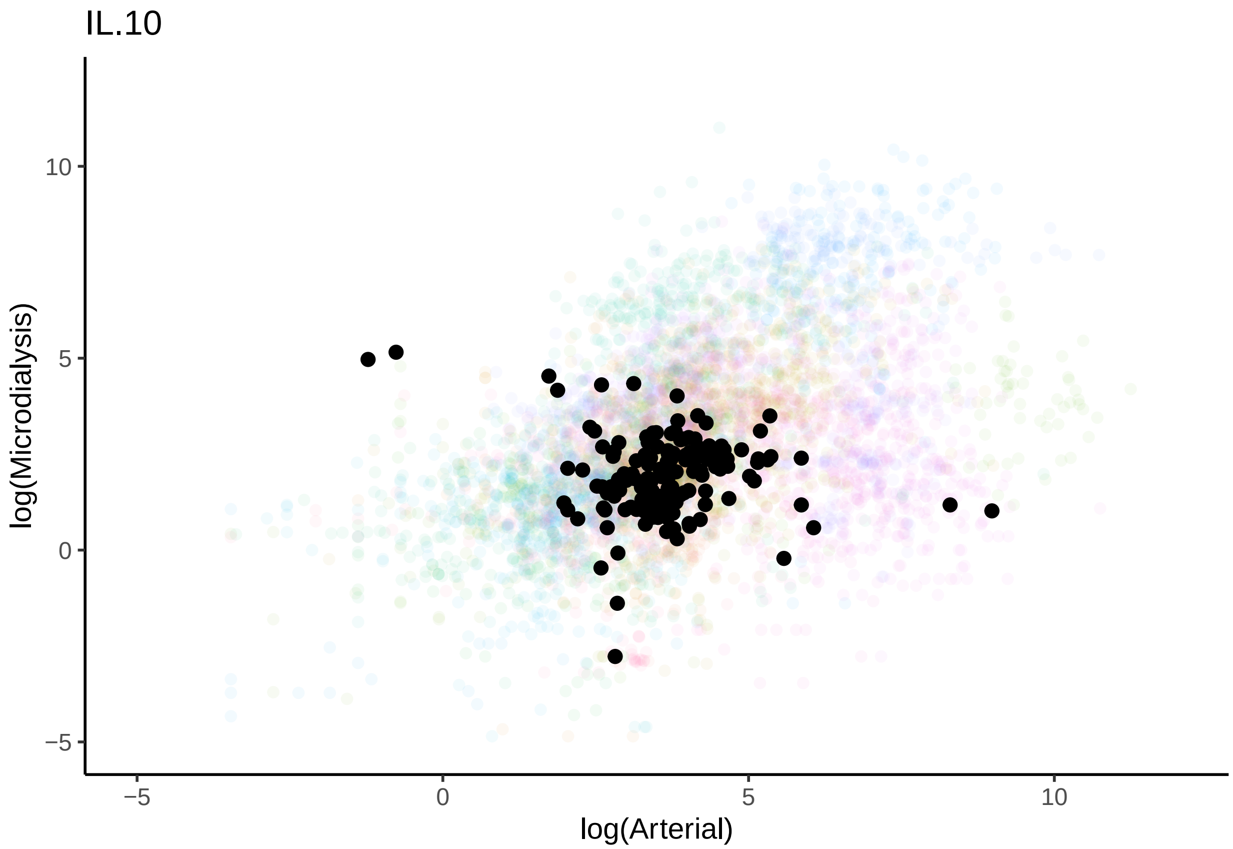


```
## Warning: Removed 10462 rows containing missing values (geom_point).
## Warning: Removed 216 rows containing missing values (geom_point).
```

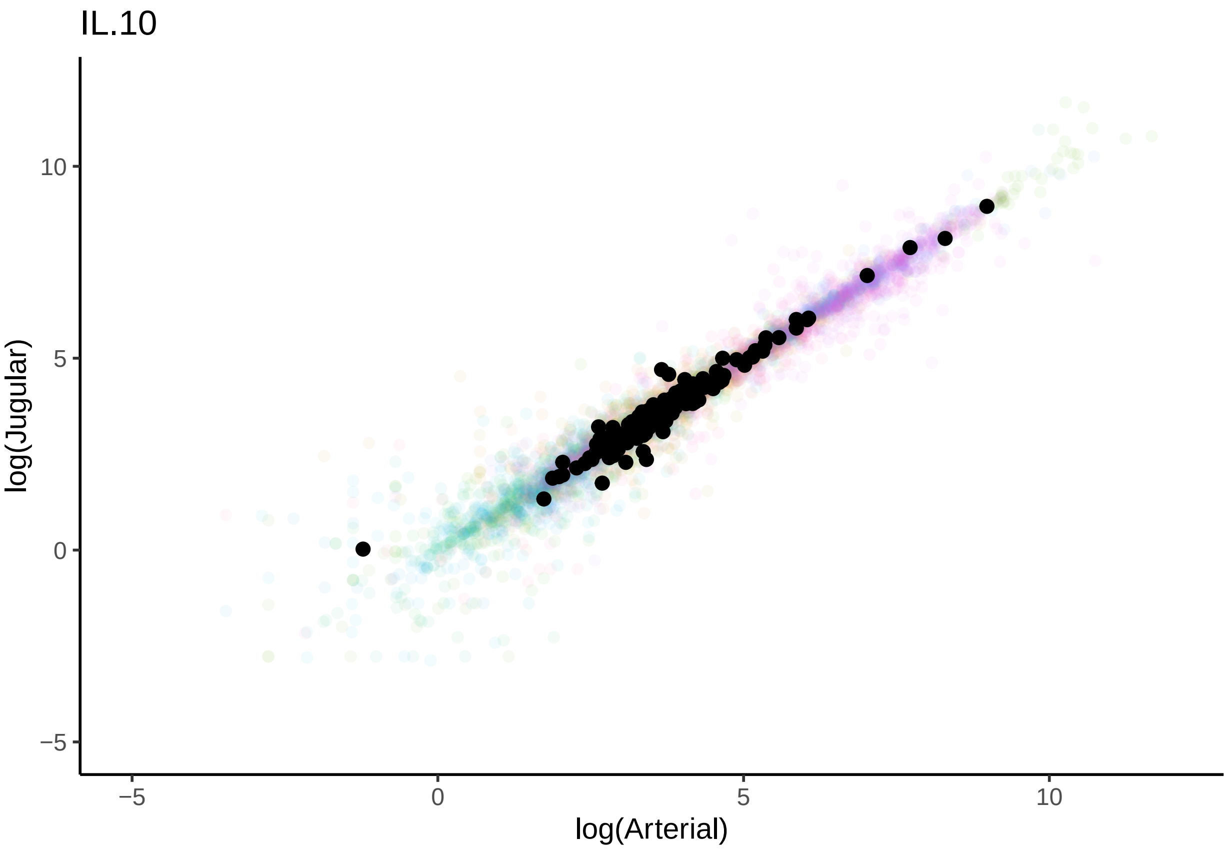


```
## Warning: Removed 10923 rows containing missing values (geom_point).
## Warning: Removed 280 rows containing missing values (geom_point).
```

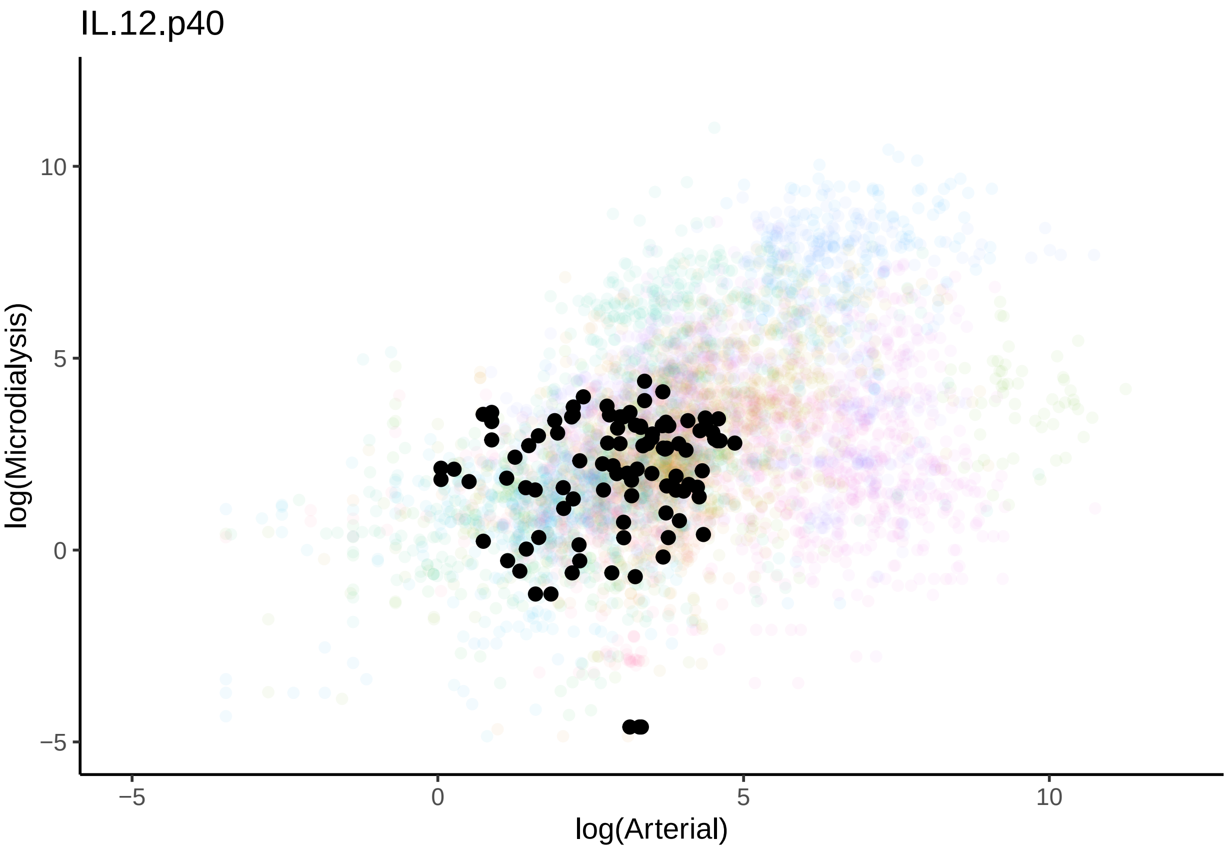


```
## Warning: Removed 10462 rows containing missing values (geom_point).
## Warning: Removed 285 rows containing missing values (geom_point).
```

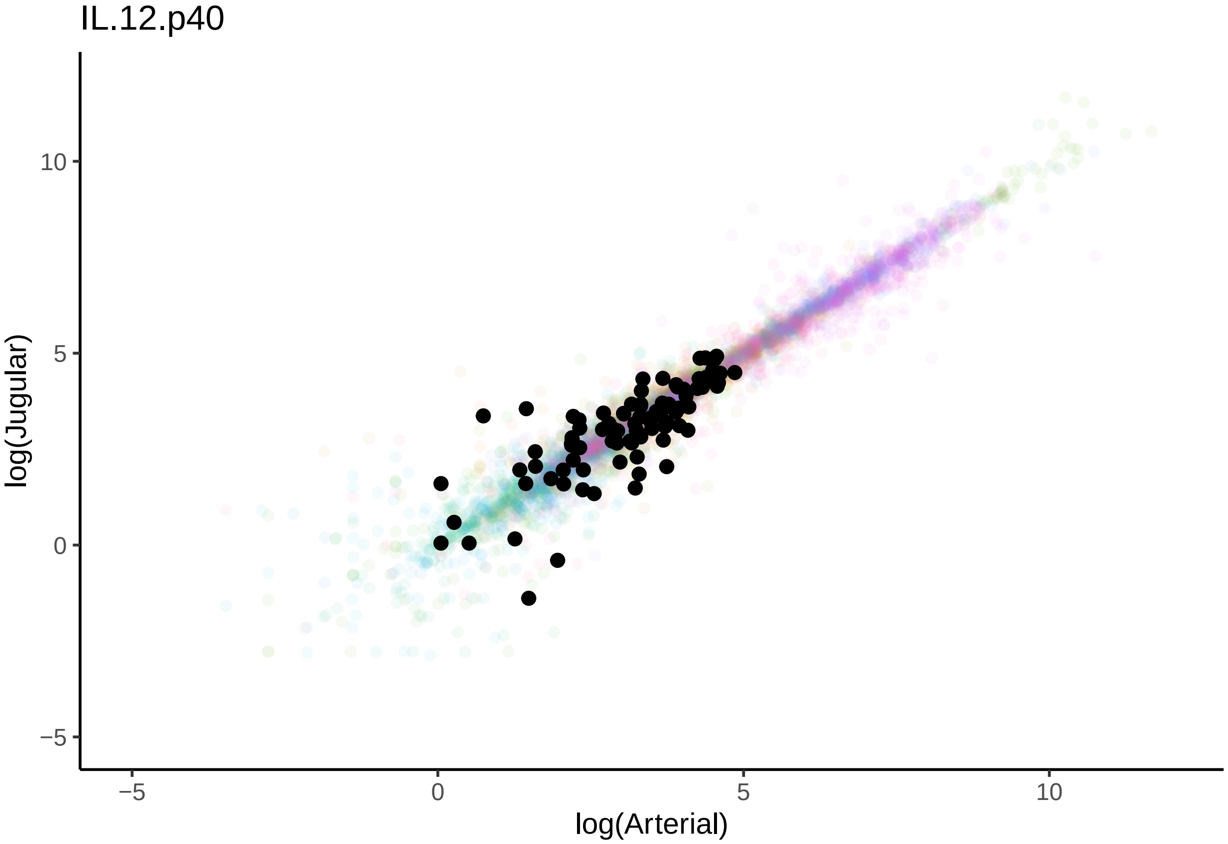


```
## Warning: Removed 10923 rows containing missing values (geom_point).
## Warning: Removed 236 rows containing missing values (geom_point).
```

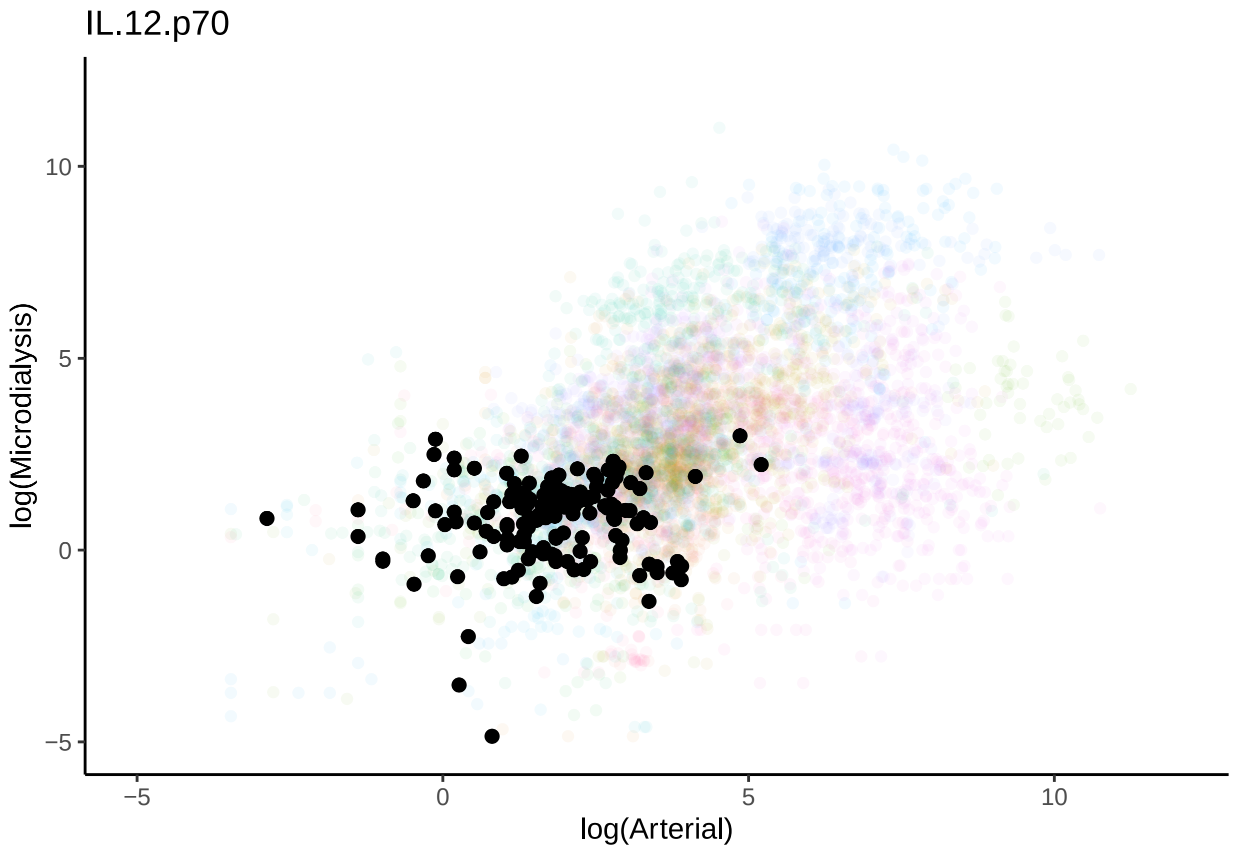


```
## Warning: Removed 10462 rows containing missing values (geom_point).
## Warning: Removed 222 rows containing missing values (geom_point).
```

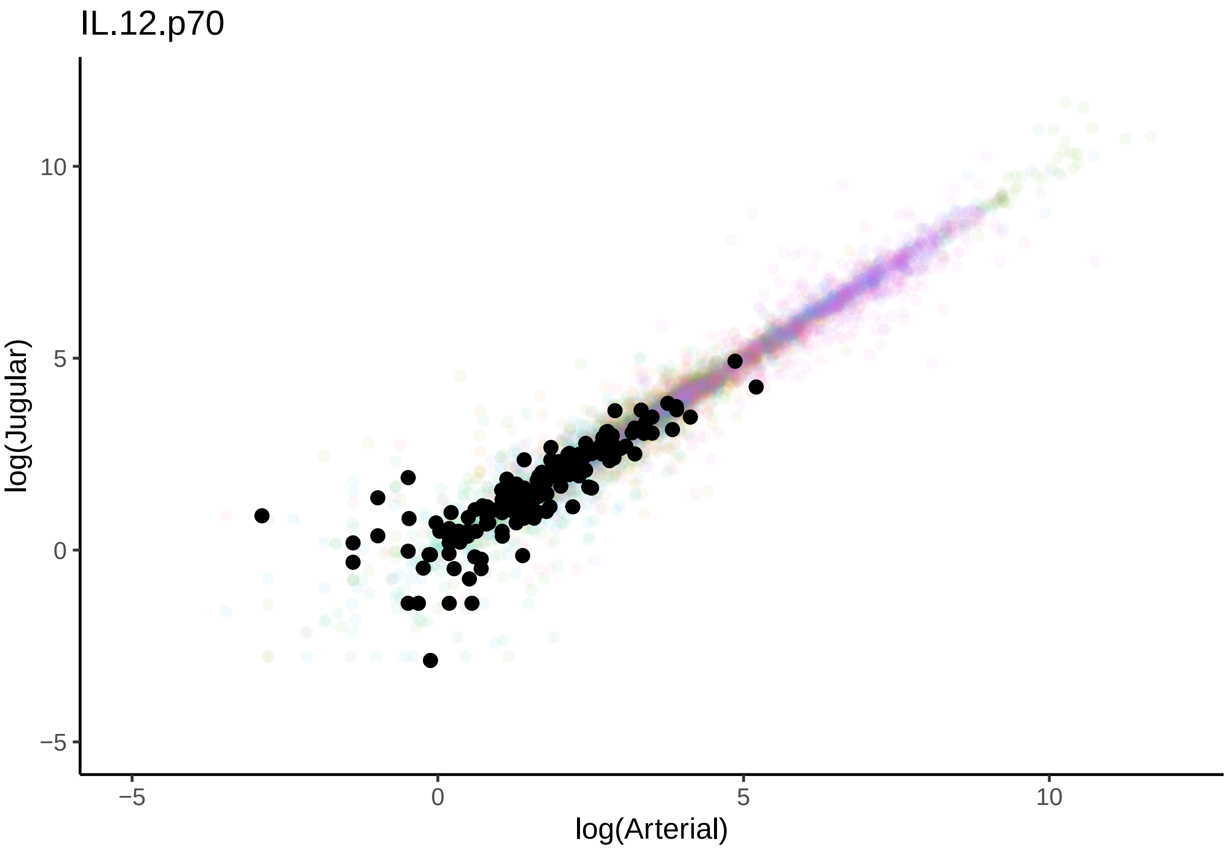


```
## Warning: Removed 10923 rows containing missing values (geom_point).

## Warning: Removed 339 rows containing missing values (geom_point).
```

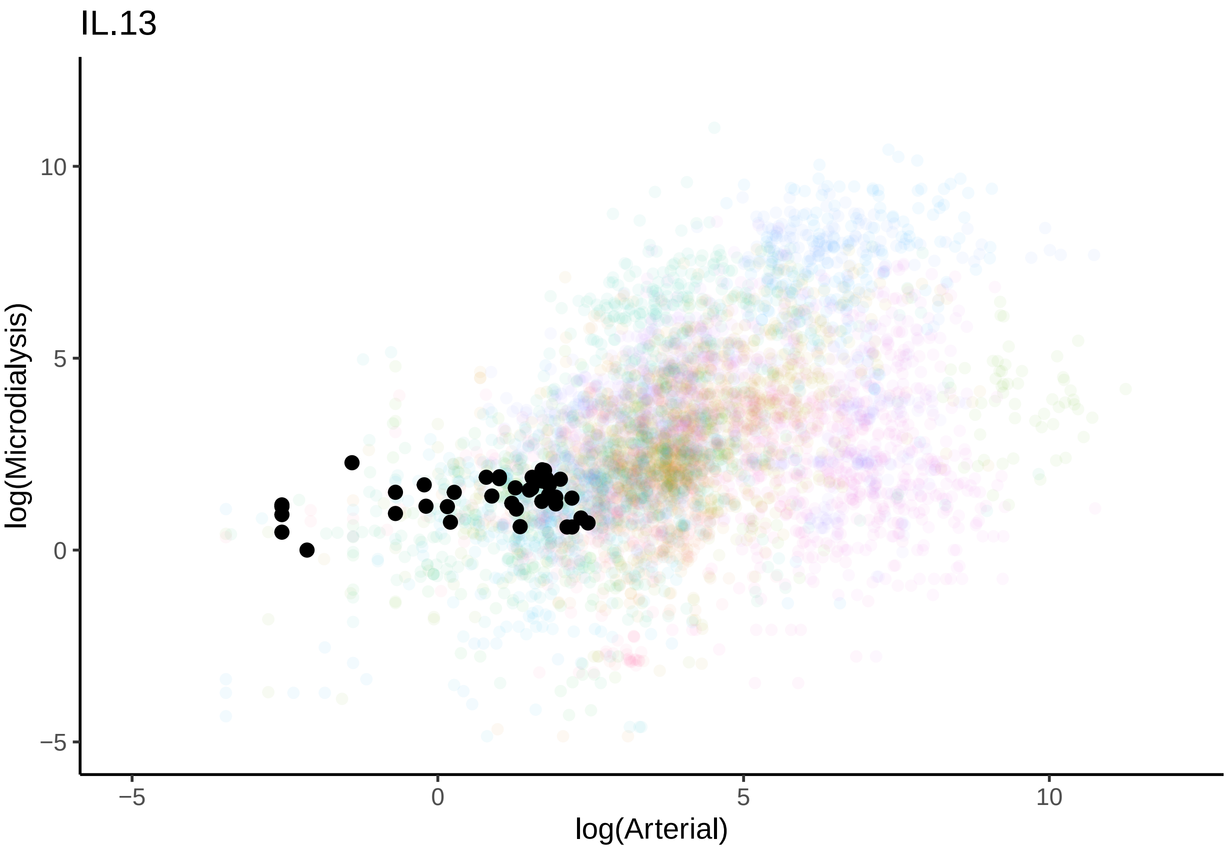


```
## Warning: Removed 10462 rows containing missing values (geom_point).

## Warning: Removed 324 rows containing missing values (geom_point).
```

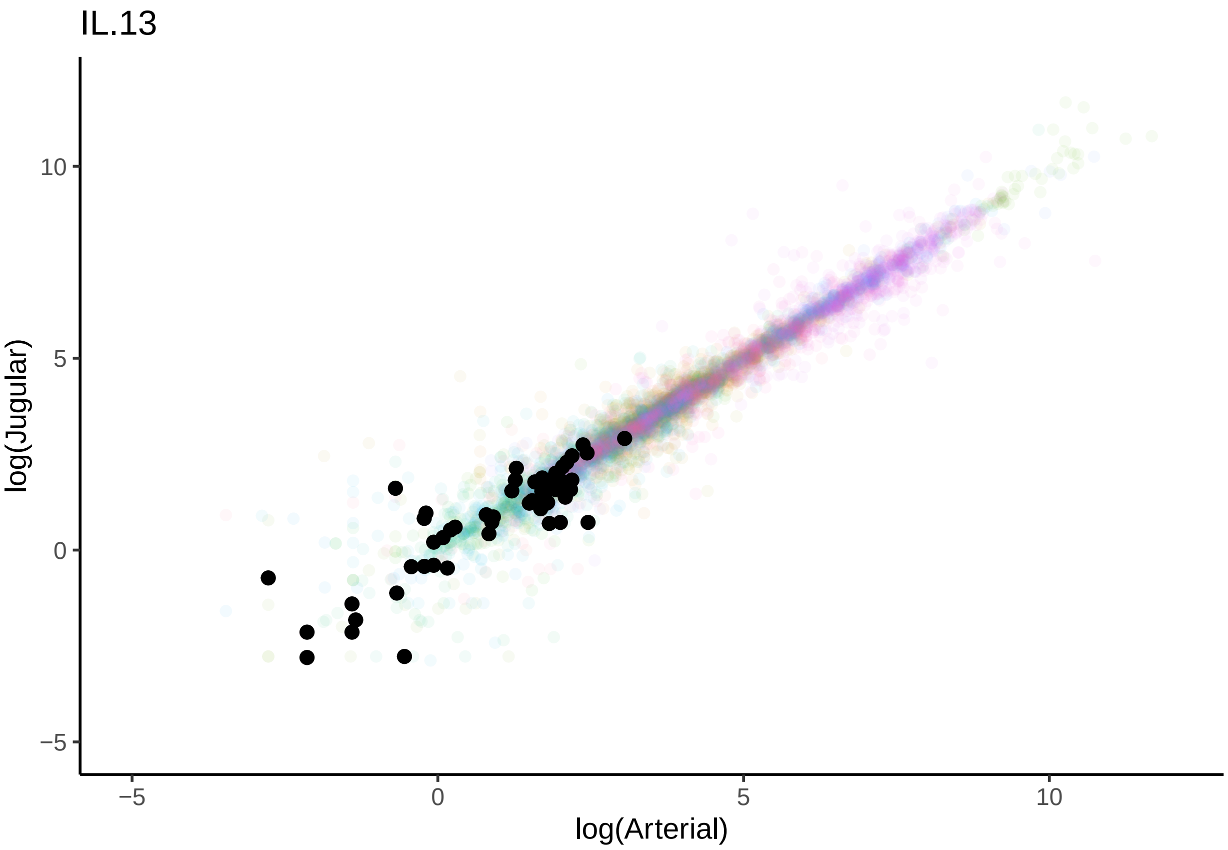


```
## Warning: Removed 10923 rows containing missing values (geom_point).
## Warning: Removed 248 rows containing missing values (geom_point).
```

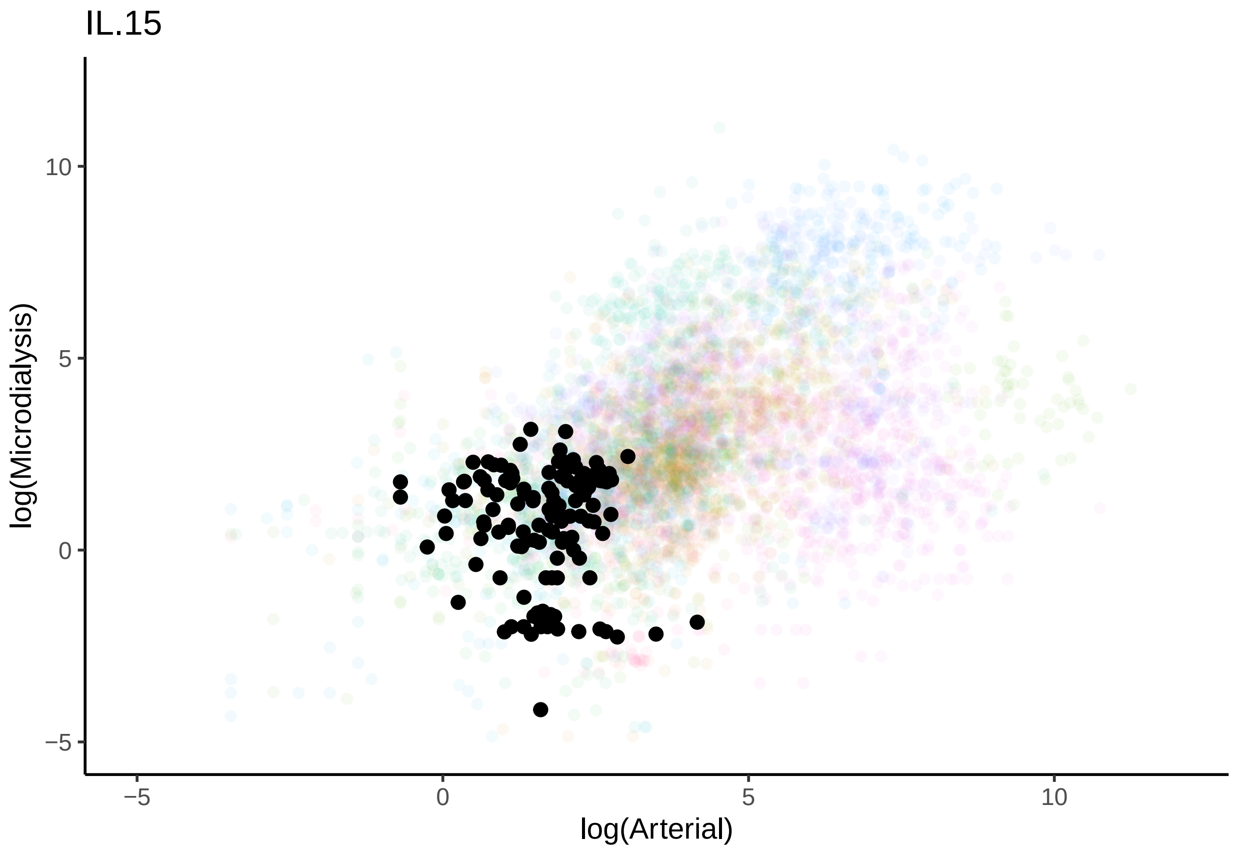


```
## Warning: Removed 10462 rows containing missing values (geom_point).
## Warning: Removed 221 rows containing missing values (geom_point).
```

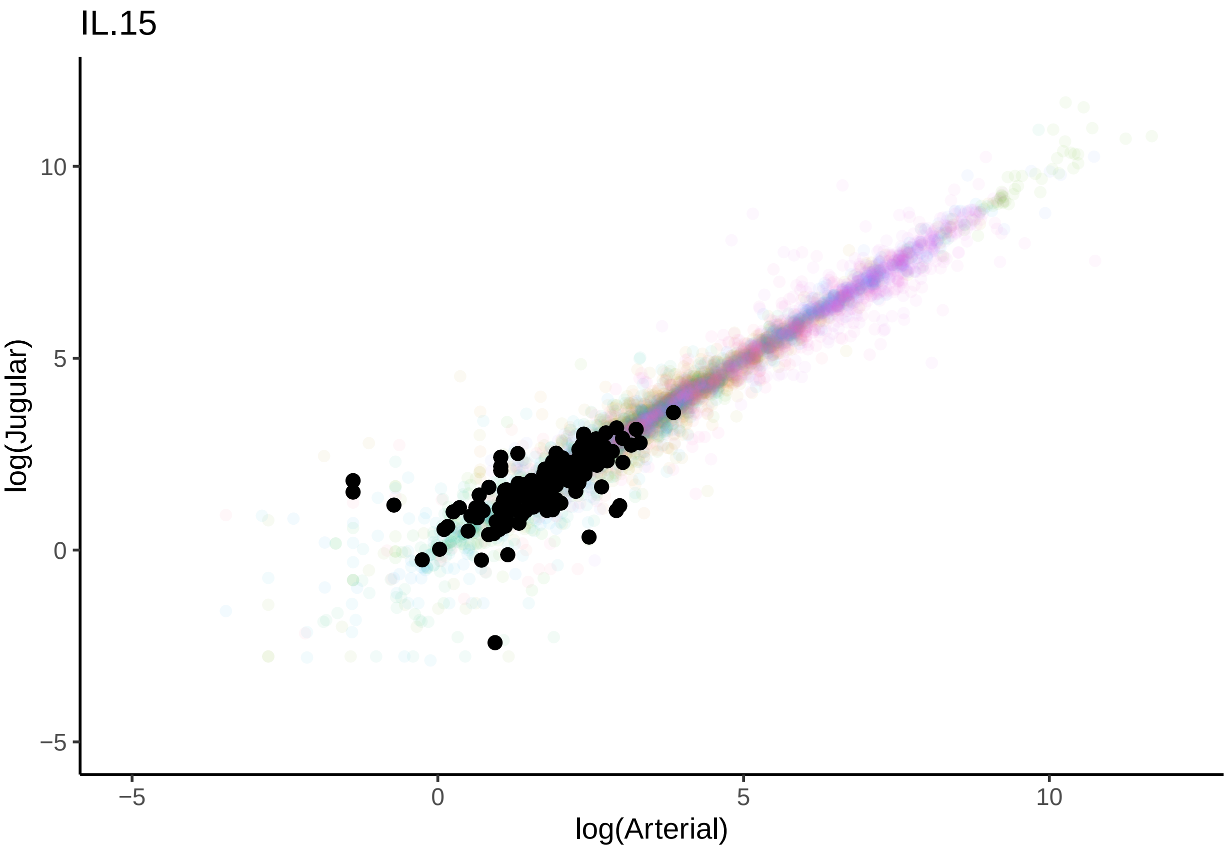


```
## Warning: Removed 10923 rows containing missing values (geom_point).
## Warning: Removed 313 rows containing missing values (geom_point).
```

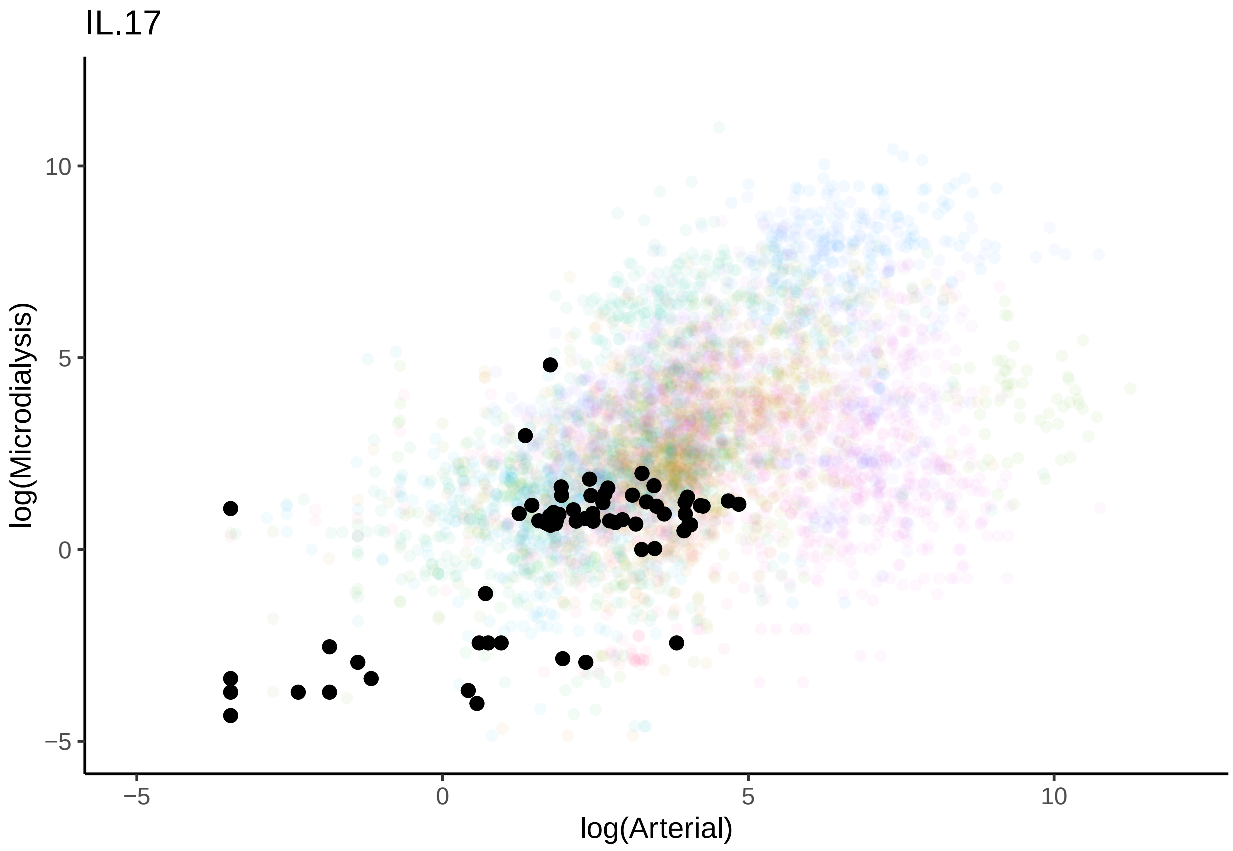


```
## Warning: Removed 10462 rows containing missing values (geom_point).

## Warning: Removed 237 rows containing missing values (geom_point).
```

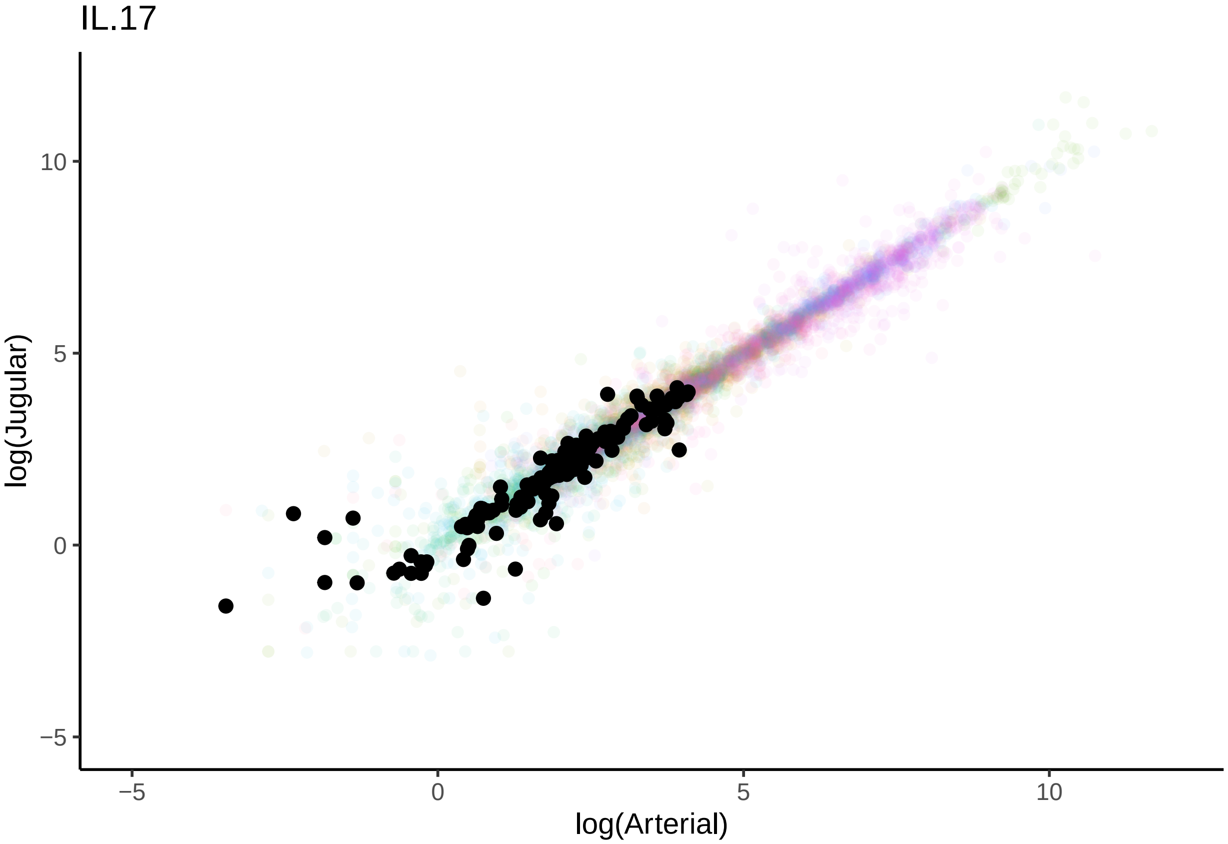


```
## Warning: Removed 10923 rows containing missing values (geom_point).
## Warning: Removed 202 rows containing missing values (geom_point).
```

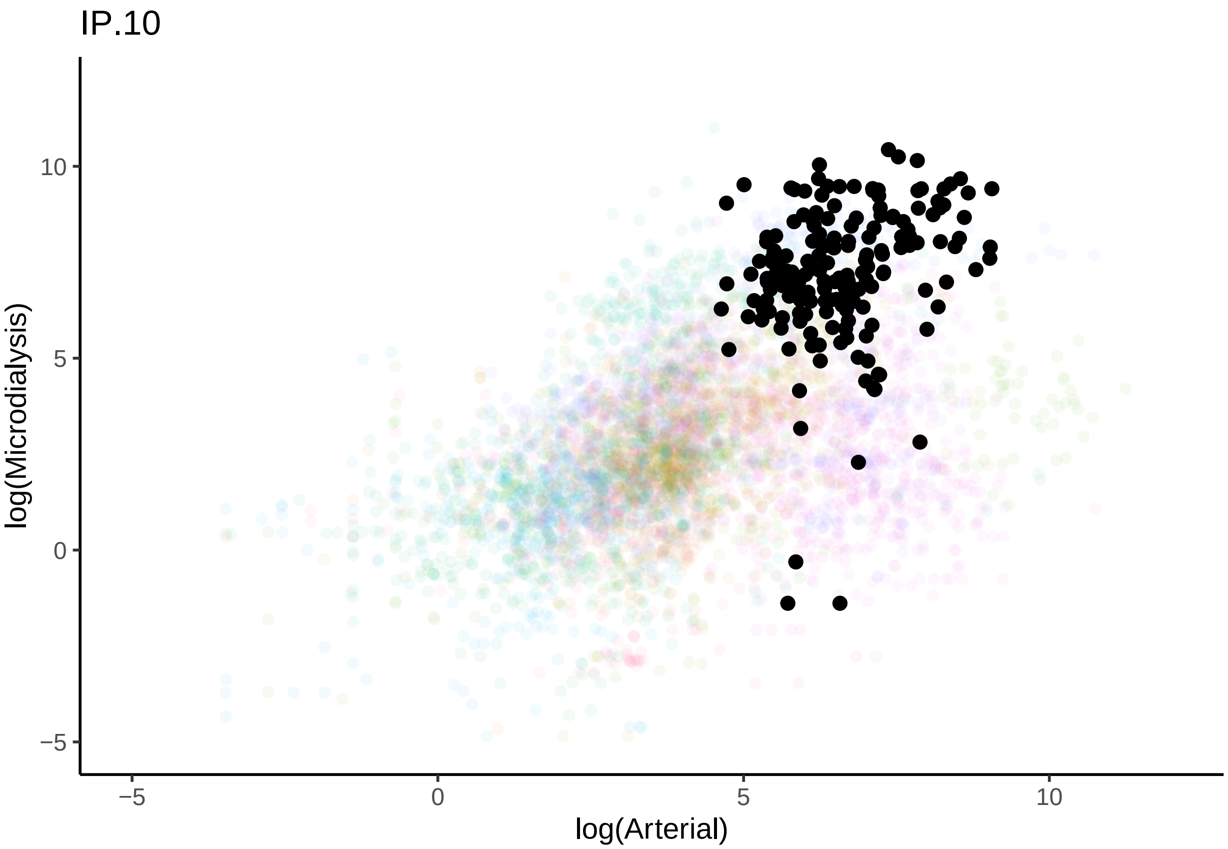


```
## Warning: Removed 10462 rows containing missing values (geom_point).
## Warning: Removed 213 rows containing missing values (geom_point).
```

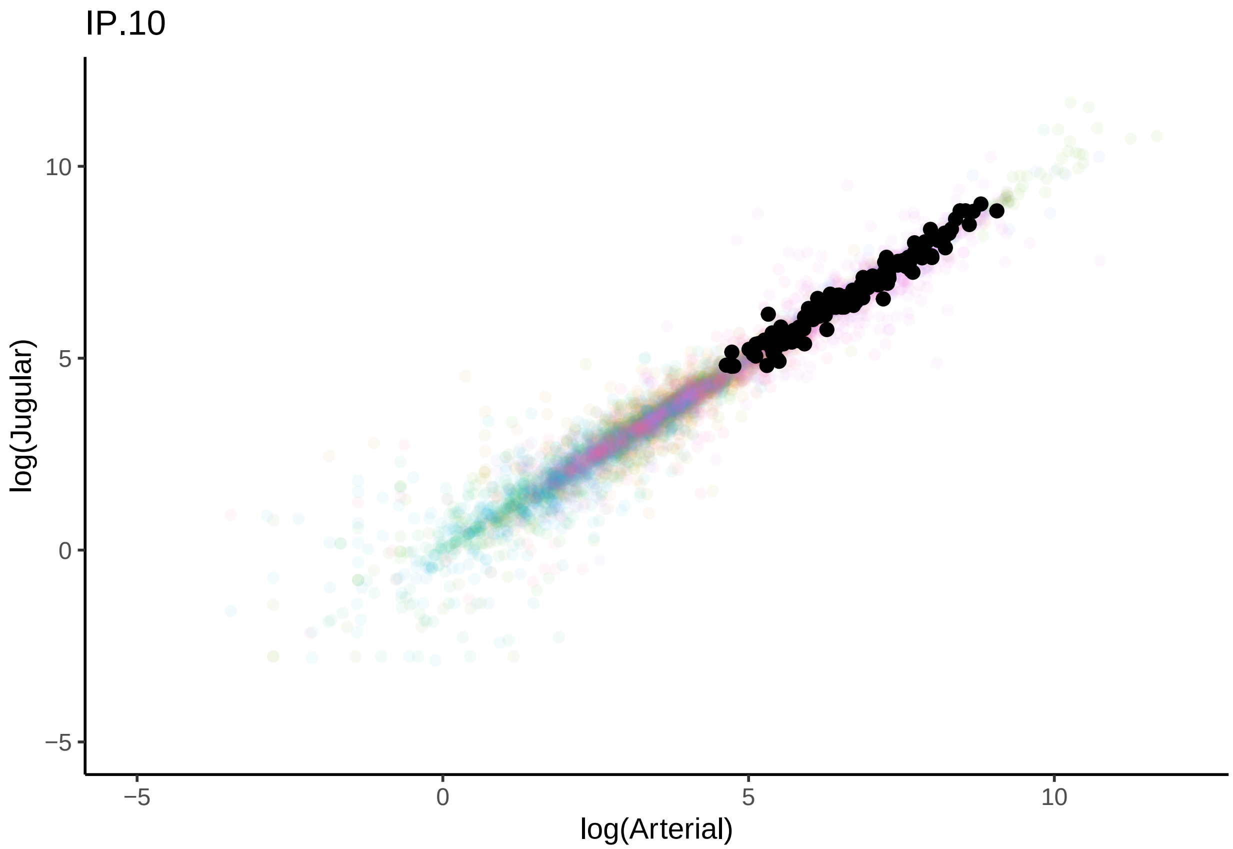


```
## Warning: Removed 10923 rows containing missing values (geom_point).

## Warning: Removed 240 rows containing missing values (geom_point).
```

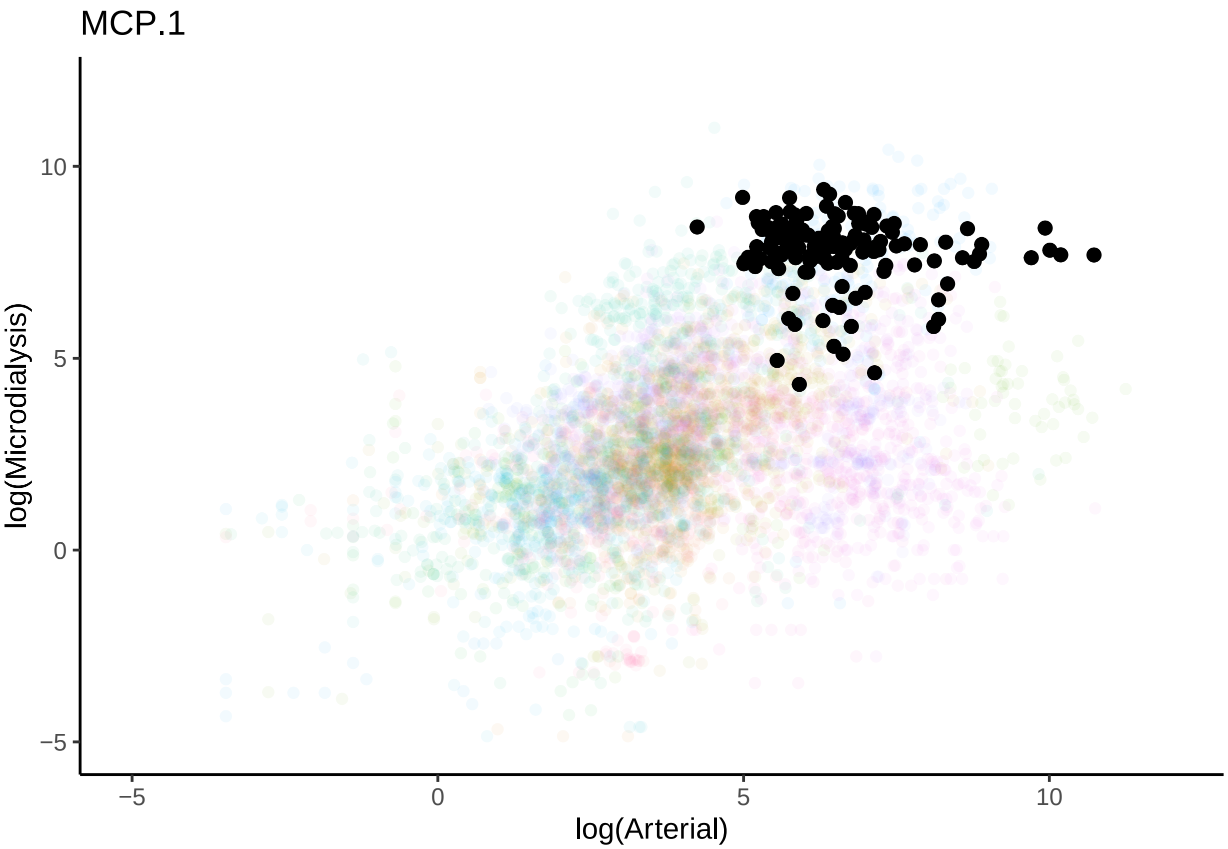


```
## Warning: Removed 10462 rows containing missing values (geom_point).
## Warning: Removed 213 rows containing missing values (geom_point).
```

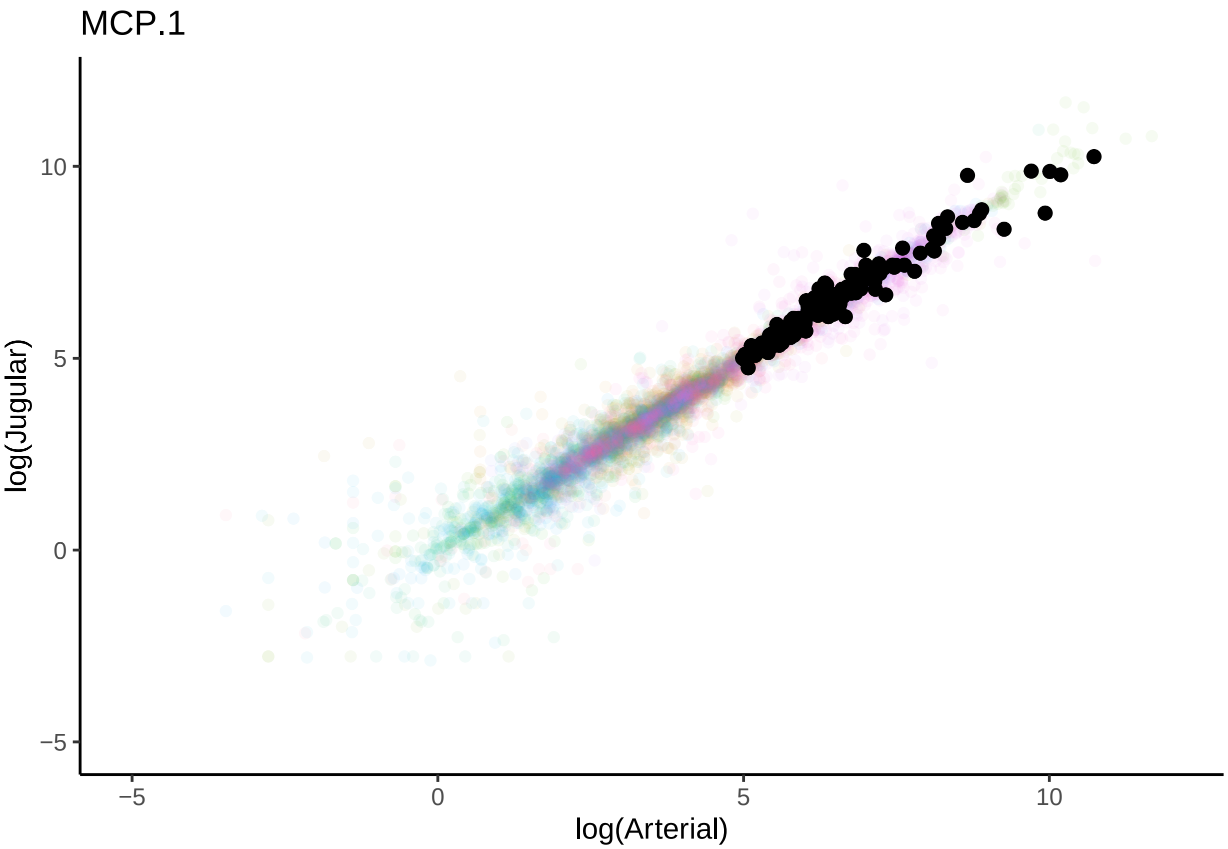


```
## Warning: Removed 10923 rows containing missing values (geom_point).
## Warning: Removed 239 rows containing missing values (geom_point).
```

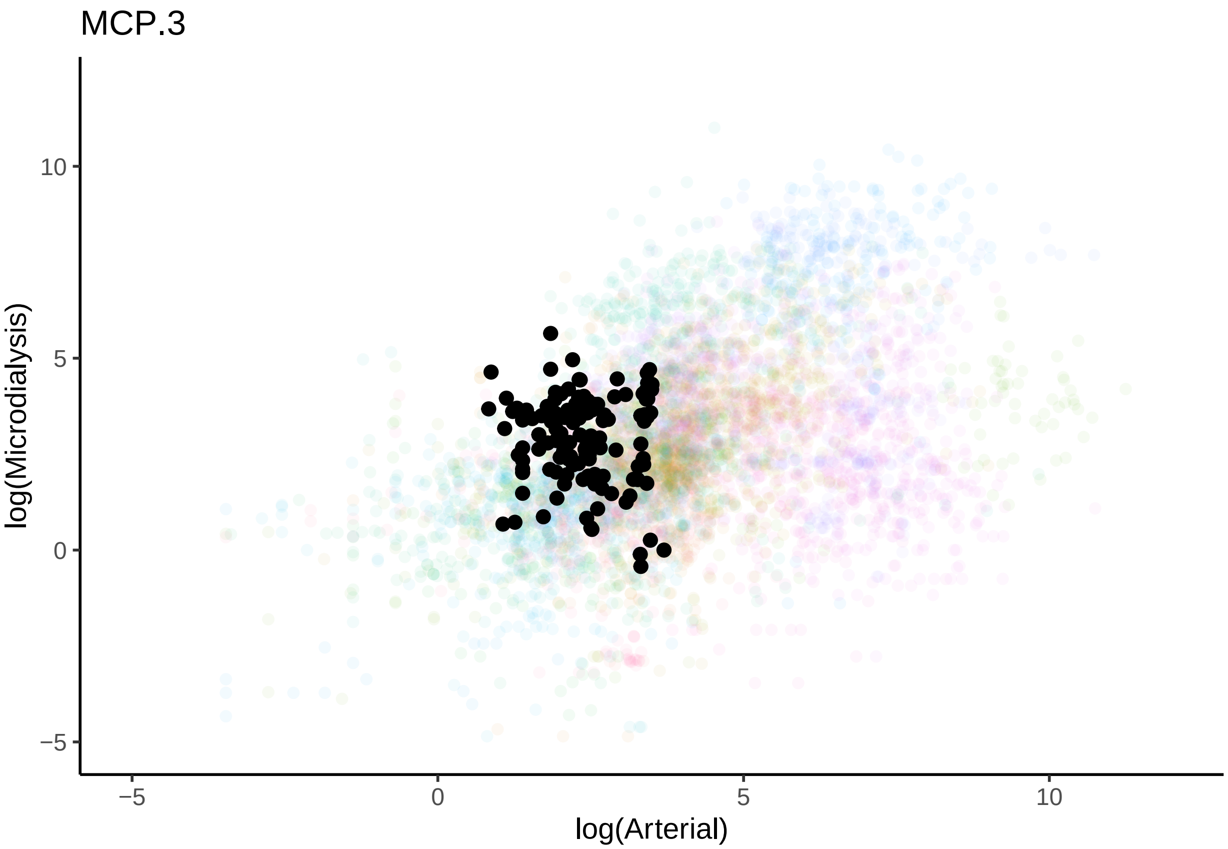


```
## Warning: Removed 10462 rows containing missing values (geom_point).
## Warning: Removed 248 rows containing missing values (geom_point).
```

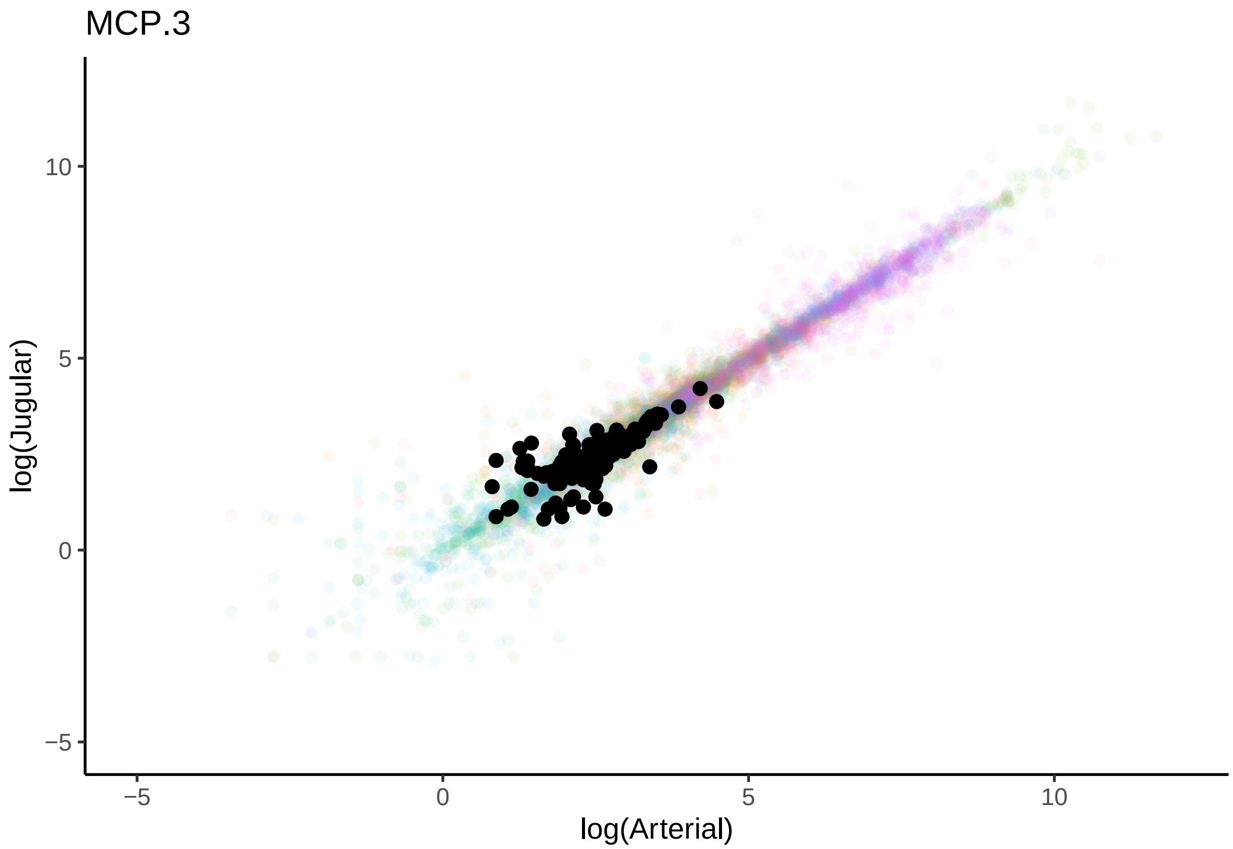


```
## Warning: Removed 10923 rows containing missing values (geom_point).

## Warning: Removed 225 rows containing missing values (geom_point).
```

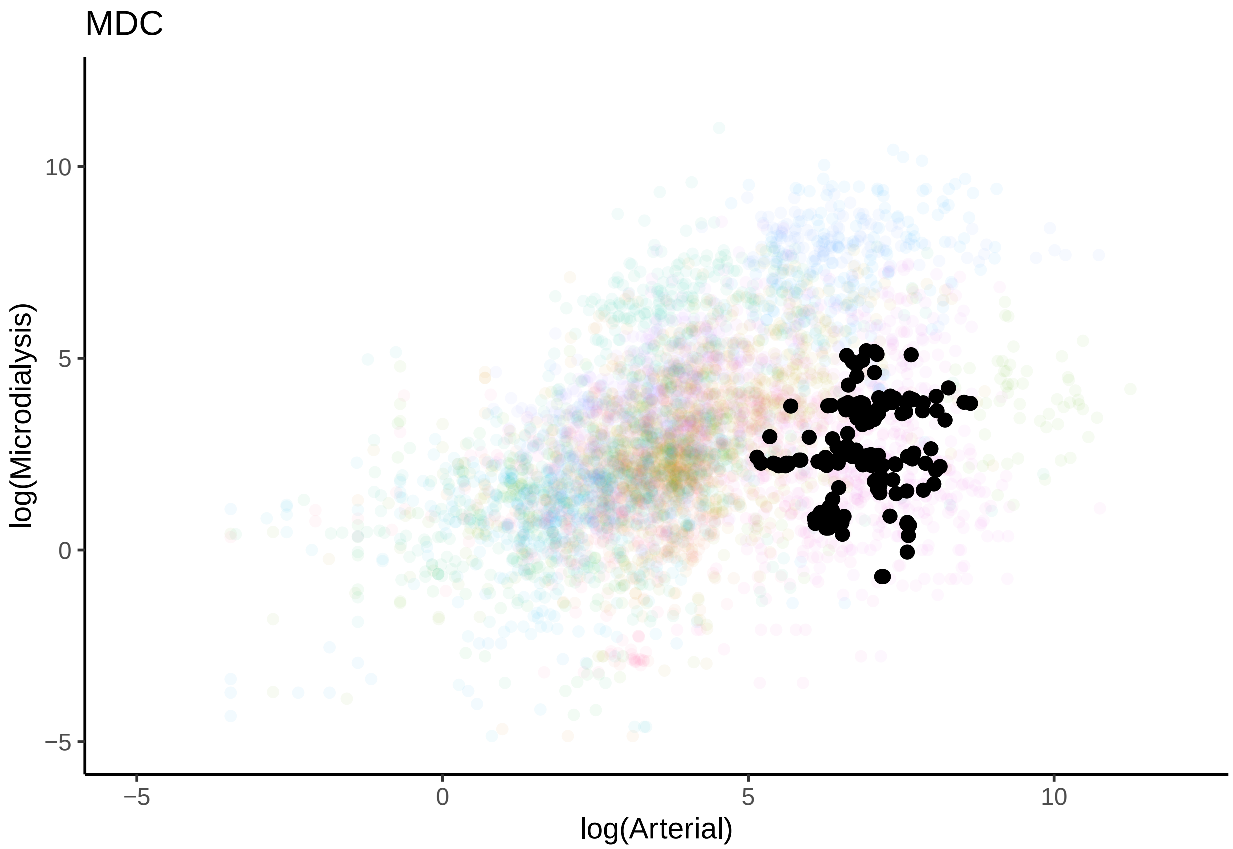


```
## Warning: Removed 10462 rows containing missing values (geom_point).

## Warning: Removed 211 rows containing missing values (geom_point).
```

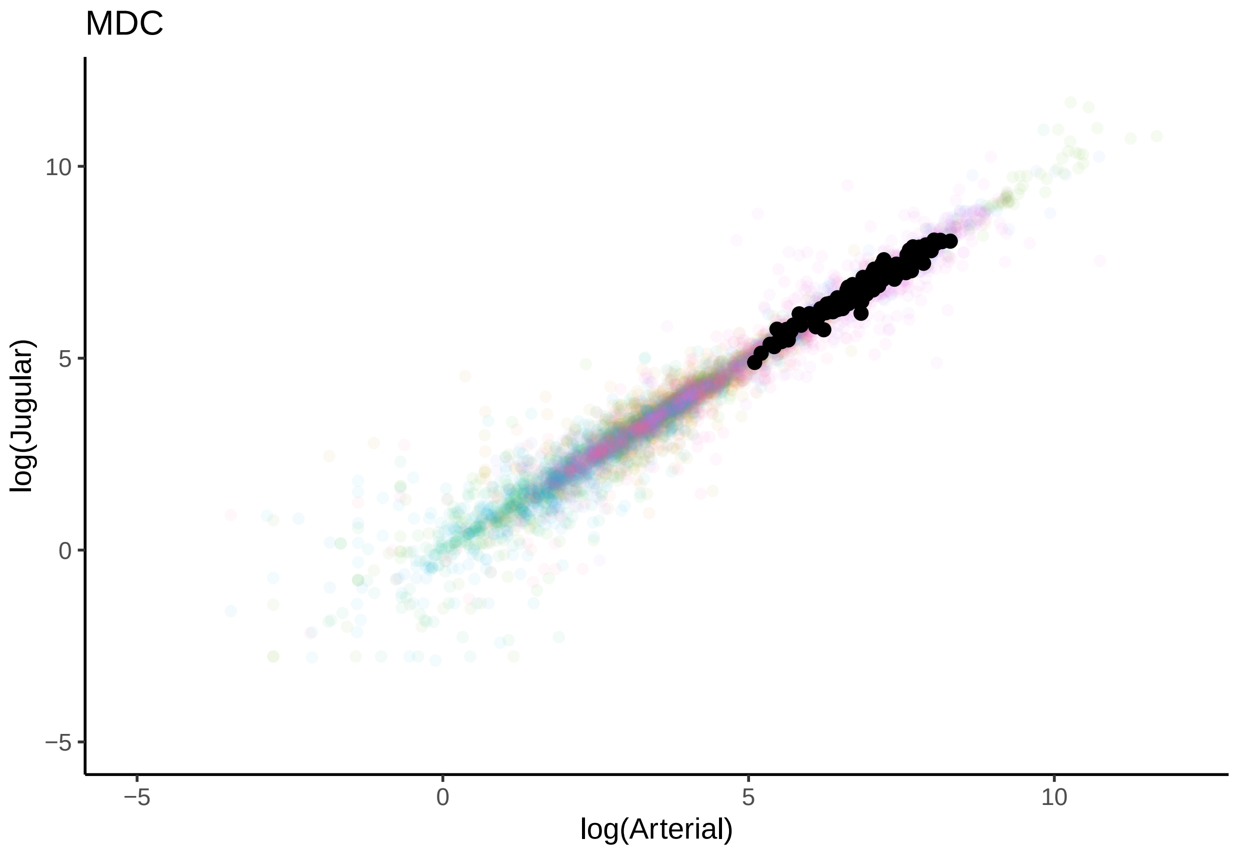


```
## Warning: Removed 10923 rows containing missing values (geom_point).

## Warning: Removed 212 rows containing missing values (geom_point).
```

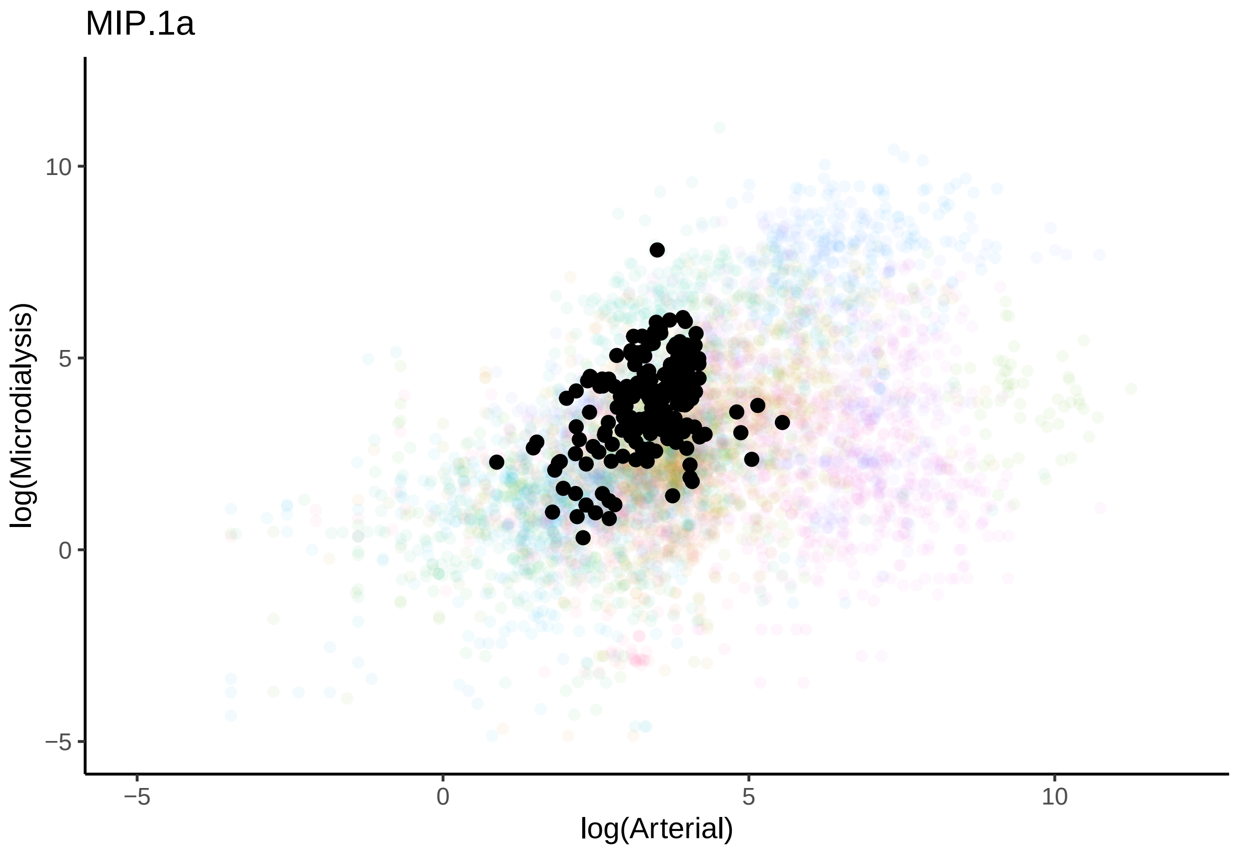


```
## Warning: Removed 10462 rows containing missing values (geom_point).
```

```
## Warning: Removed 218 rows containing missing values (geom_point).
```

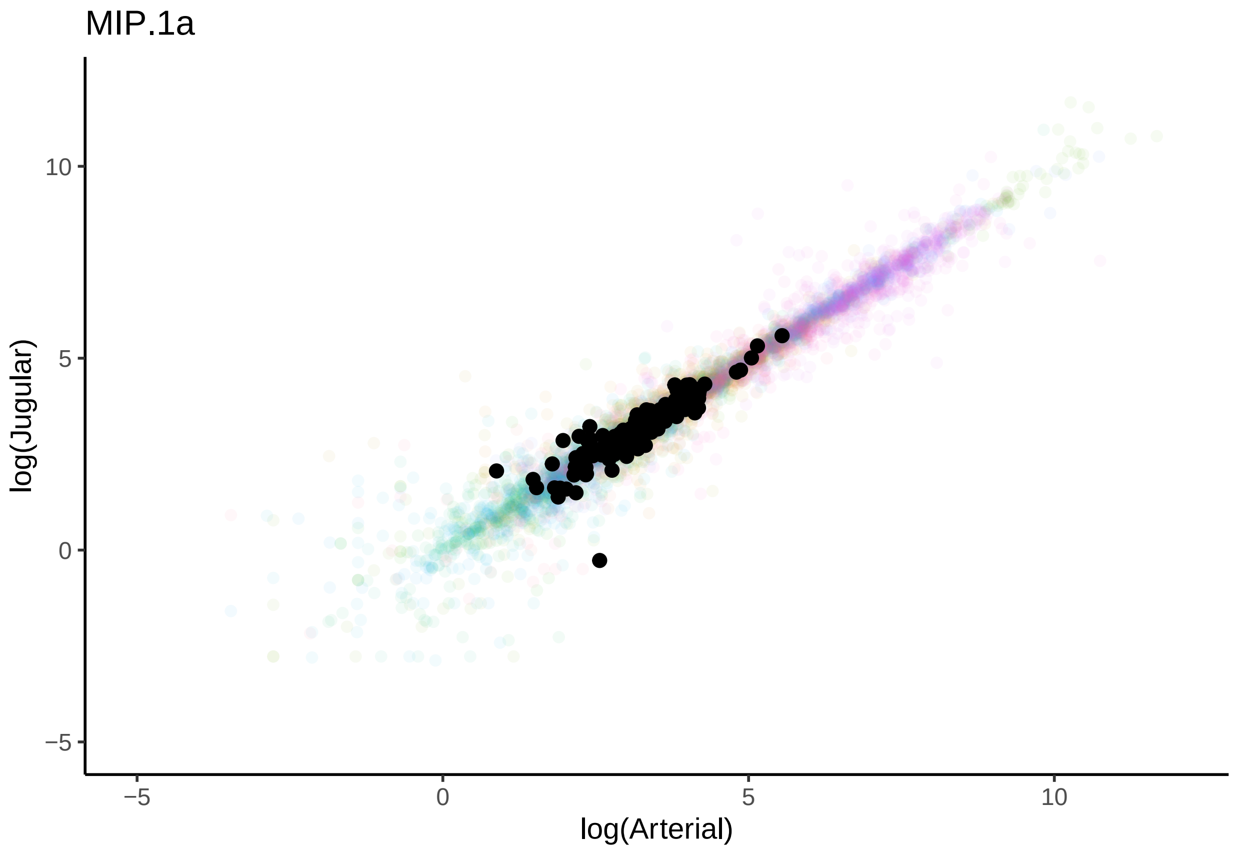


```
## Warning: Removed 10923 rows containing missing values (geom_point).
## Warning: Removed 214 rows containing missing values (geom_point).
```

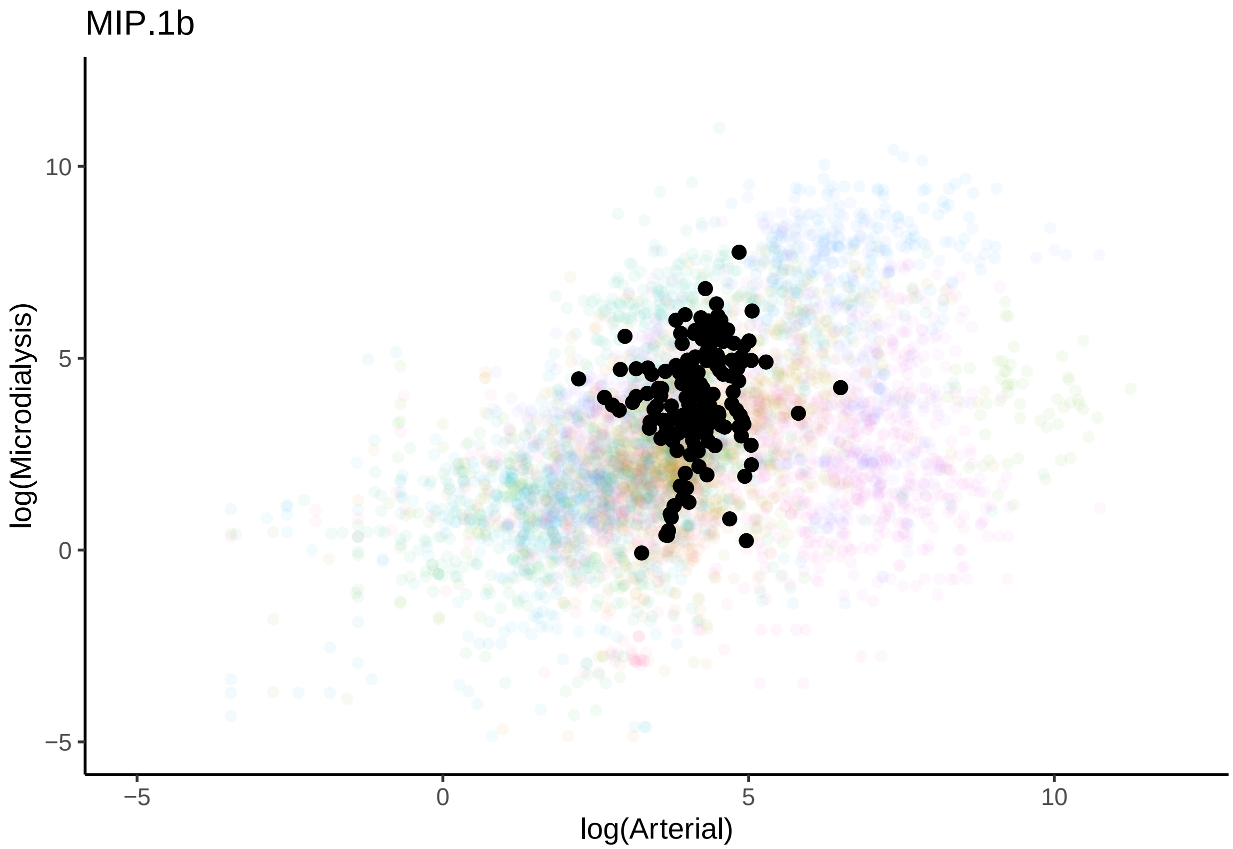


```
## Warning: Removed 10462 rows containing missing values (geom_point).

## Warning: Removed 214 rows containing missing values (geom_point).
```

MIP.1b

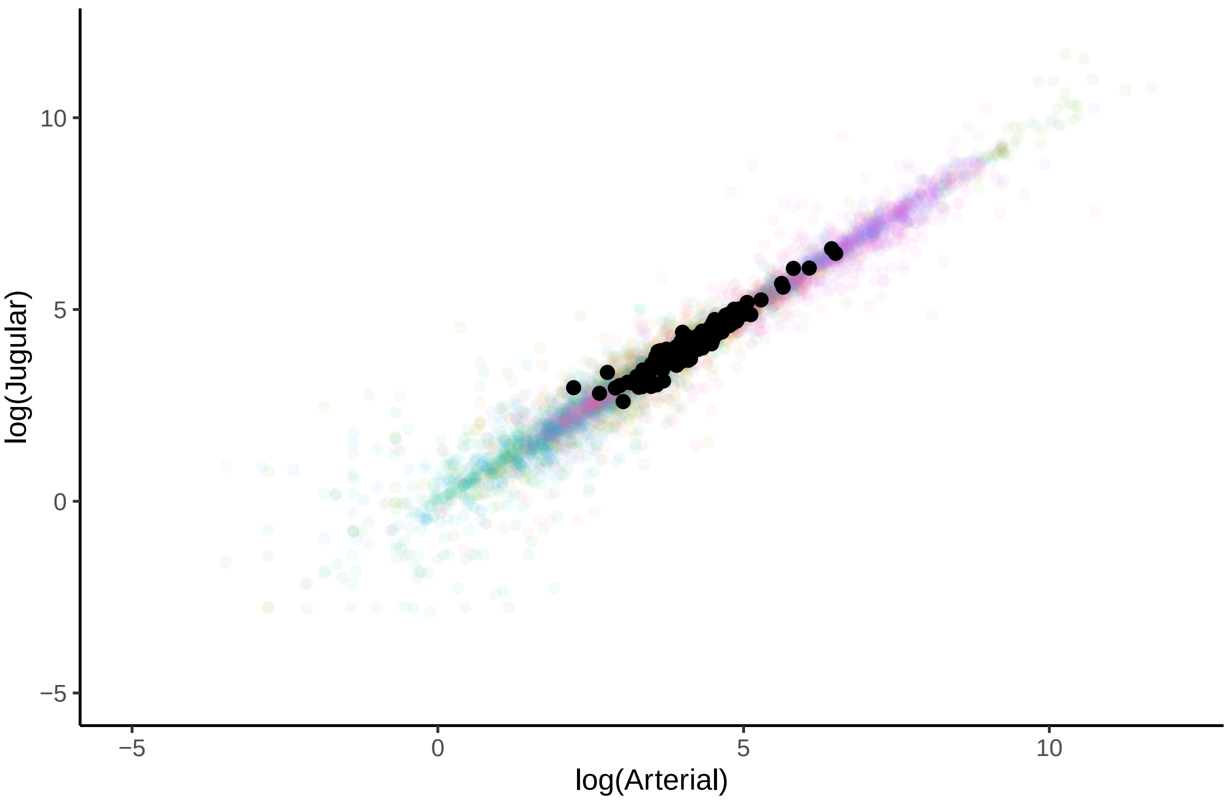


```
## Warning: Removed 10923 rows containing missing values (geom_point).
## Warning: Removed 208 rows containing missing values (geom_point).
```

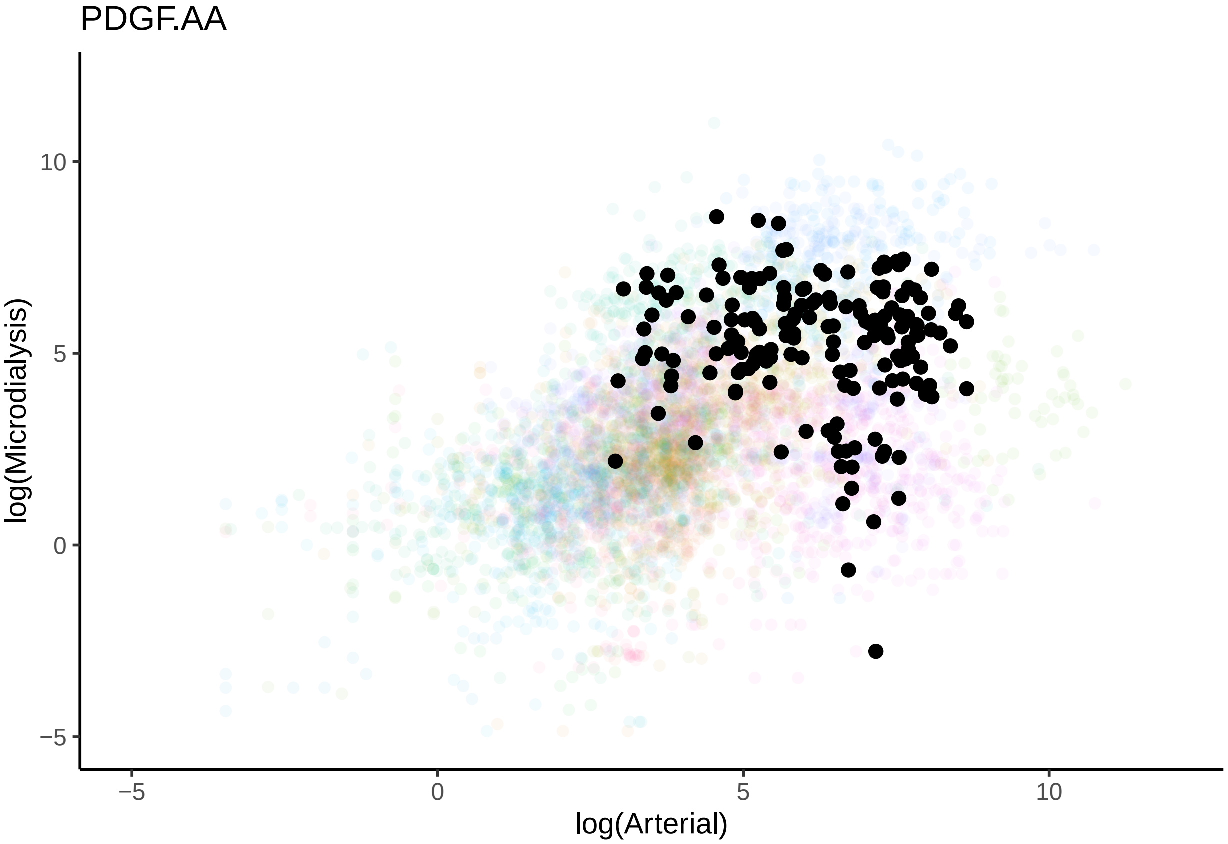


```
## Warning: Removed 10462 rows containing missing values (geom_point).
## Warning: Removed 214 rows containing missing values (geom_point).
```

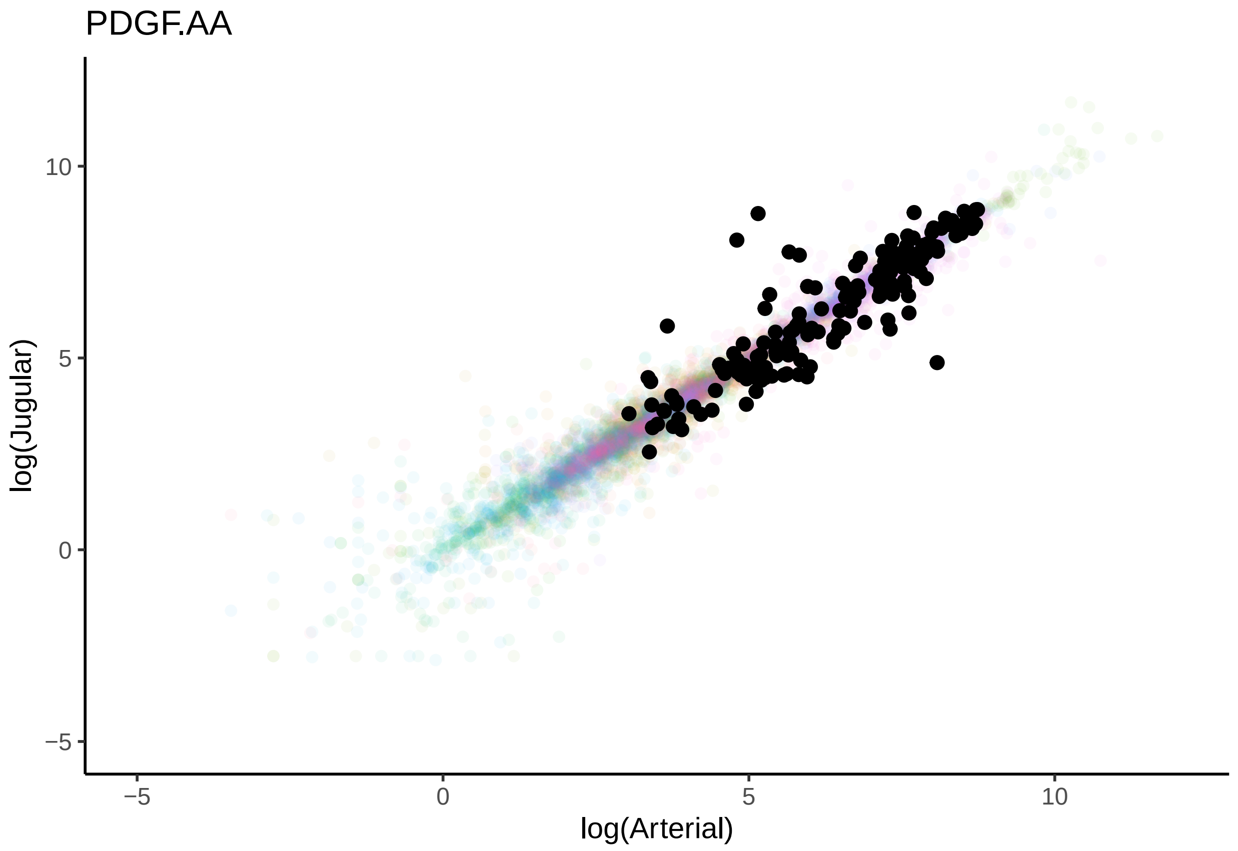


```
## Warning: Removed 10923 rows containing missing values (geom_point).
## Warning: Removed 249 rows containing missing values (geom_point).
```

PDGF.ABBB

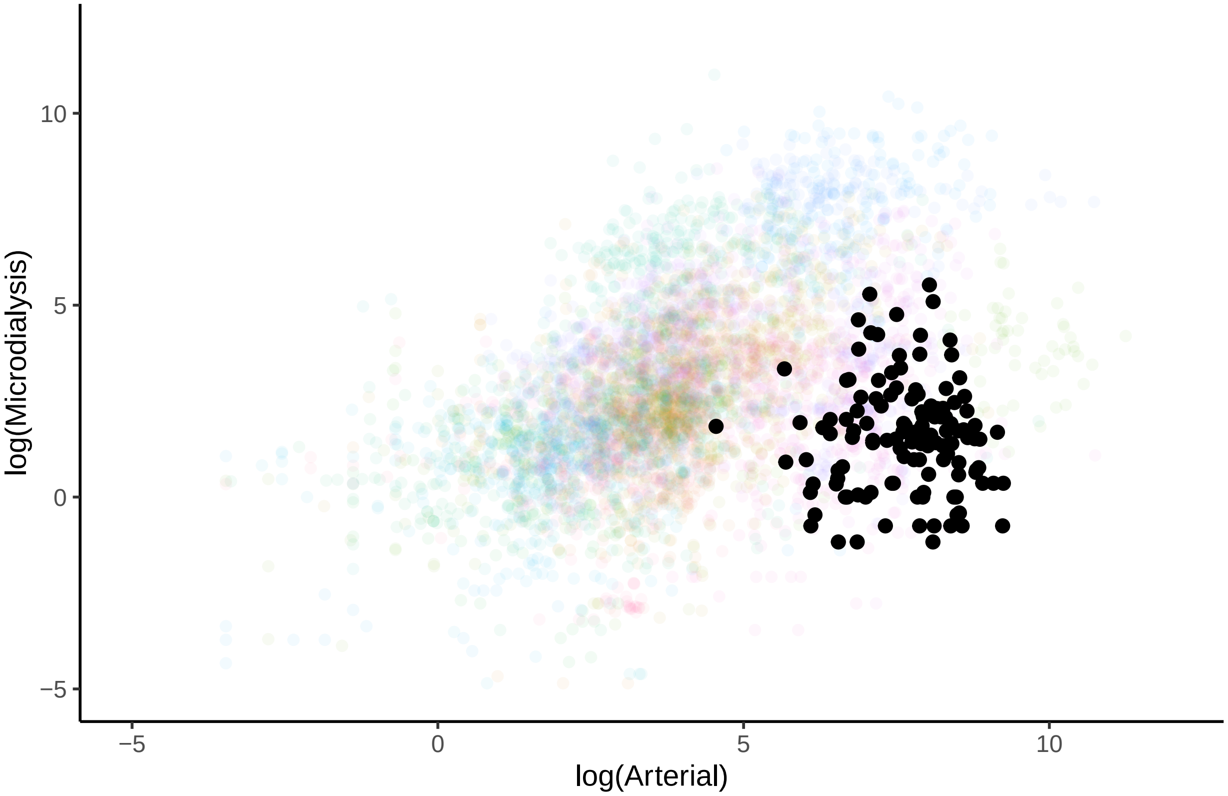


```
## Warning: Removed 10462 rows containing missing values (geom_point).

## Warning: Removed 215 rows containing missing values (geom_point).
```

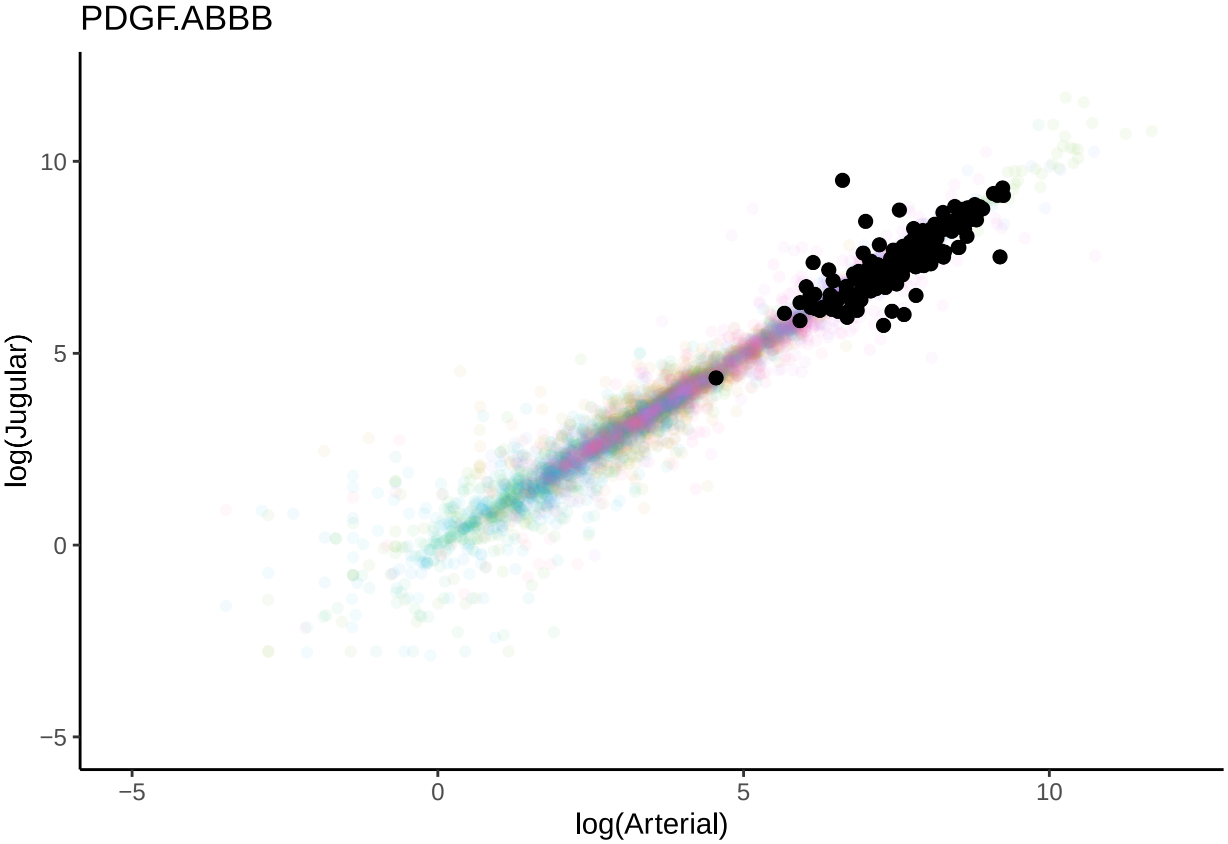


```
## Warning: Removed 10923 rows containing missing values (geom_point).
## Warning: Removed 231 rows containing missing values (geom_point).
```

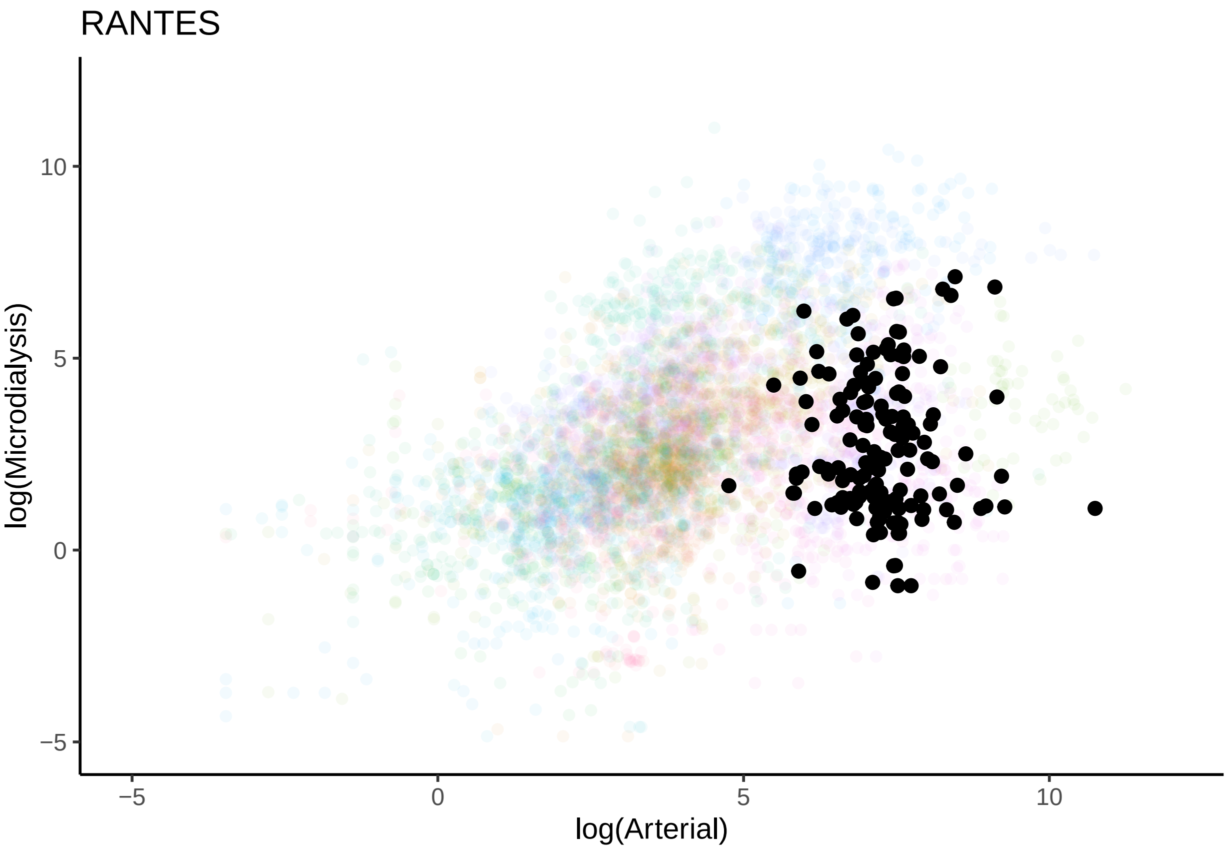


```
## Warning: Removed 10462 rows containing missing values (geom_point).
## Warning: Removed 228 rows containing missing values (geom_point).
```

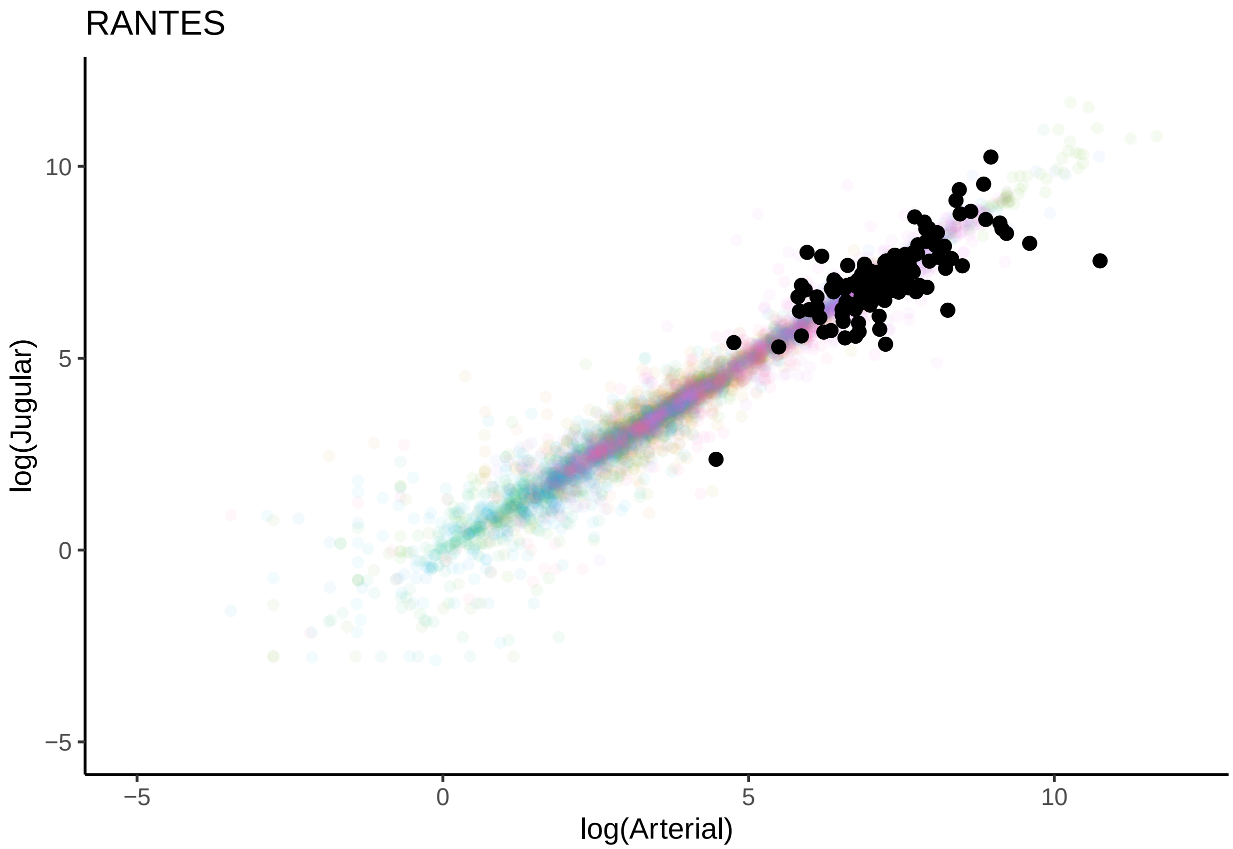


```
## Warning: Removed 10923 rows containing missing values (geom_point).

## Warning: Removed 228 rows containing missing values (geom_point).
```

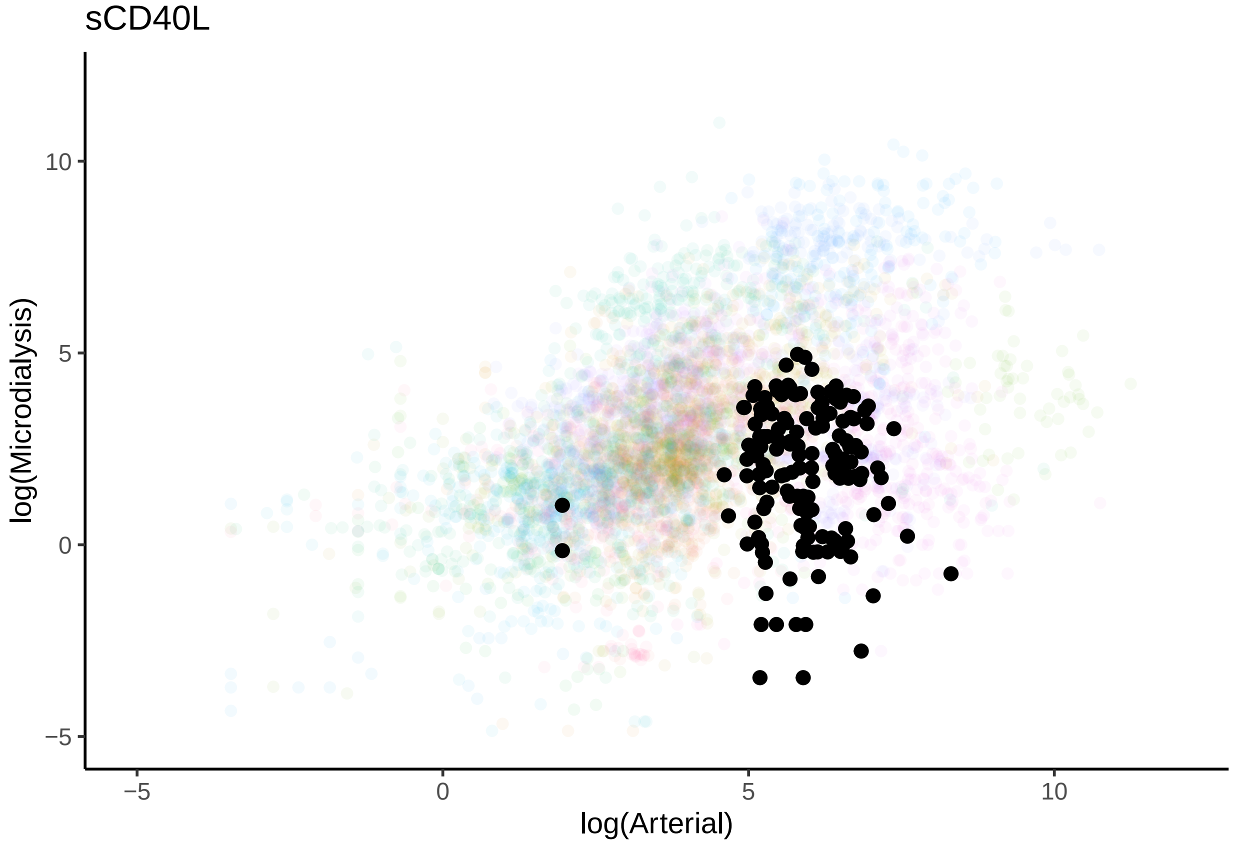


```
## Warning: Removed 10462 rows containing missing values (geom_point).
## Warning: Removed 222 rows containing missing values (geom_point).
```

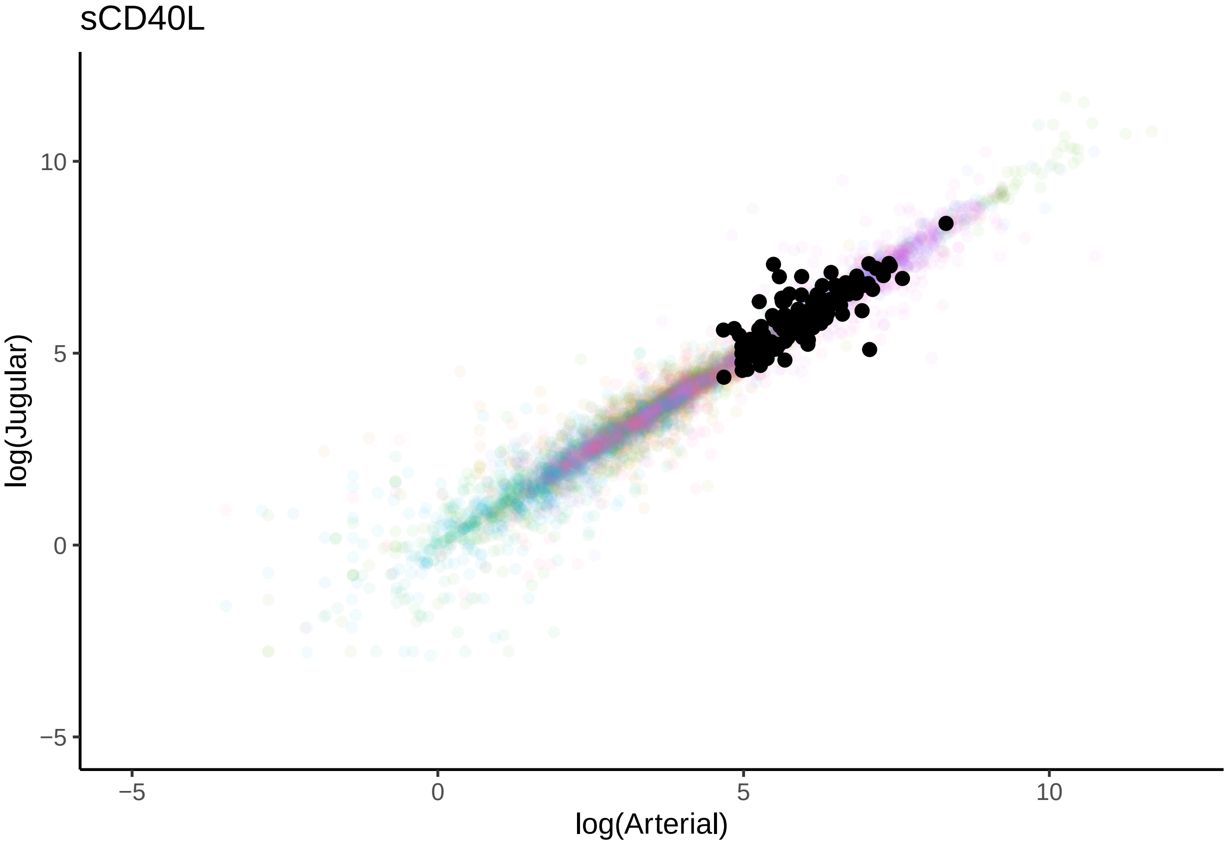


```
## Warning: Removed 10923 rows containing missing values (geom_point).

## Warning: Removed 277 rows containing missing values (geom_point).
```

sIL.2Ra

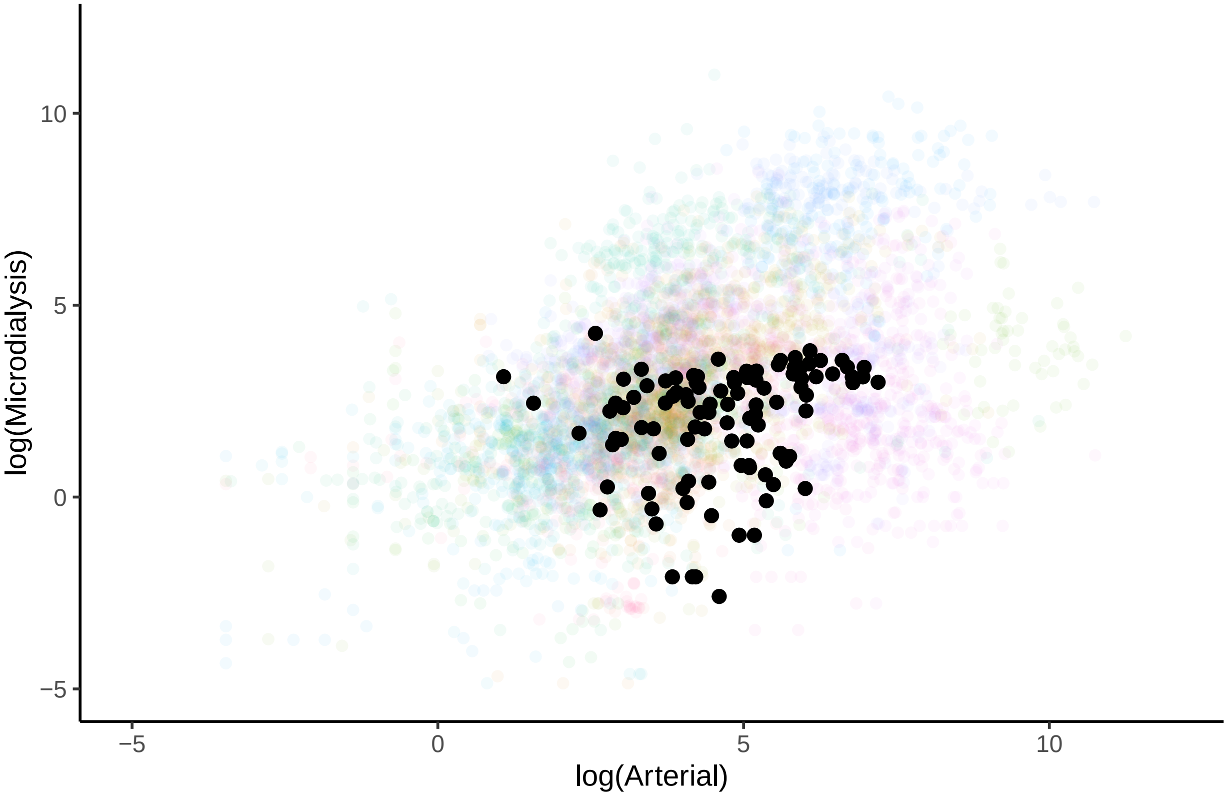


```
## Warning: Removed 10462 rows containing missing values (geom_point).

## Warning: Removed 234 rows containing missing values (geom_point).
```

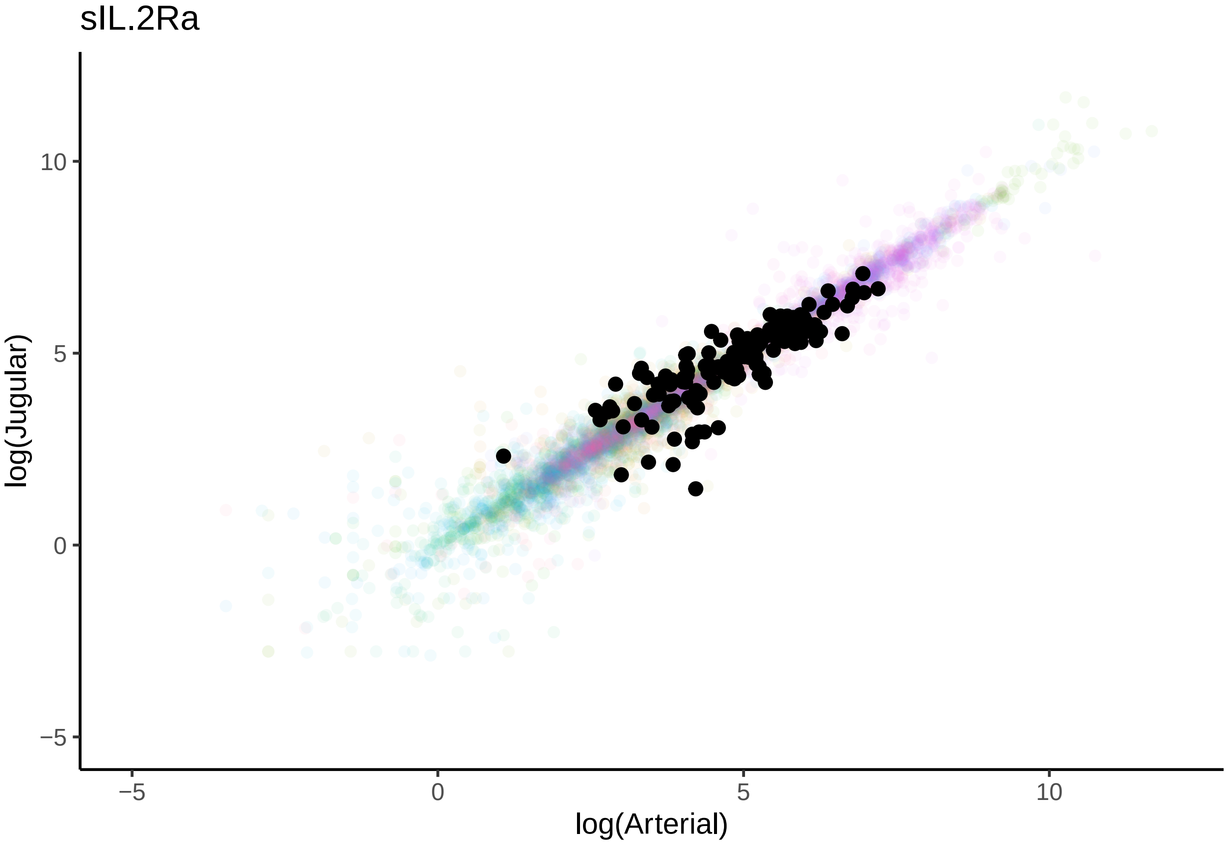


```
## Warning: Removed 10923 rows containing missing values (geom_point).
## Warning: Removed 305 rows containing missing values (geom_point).
```

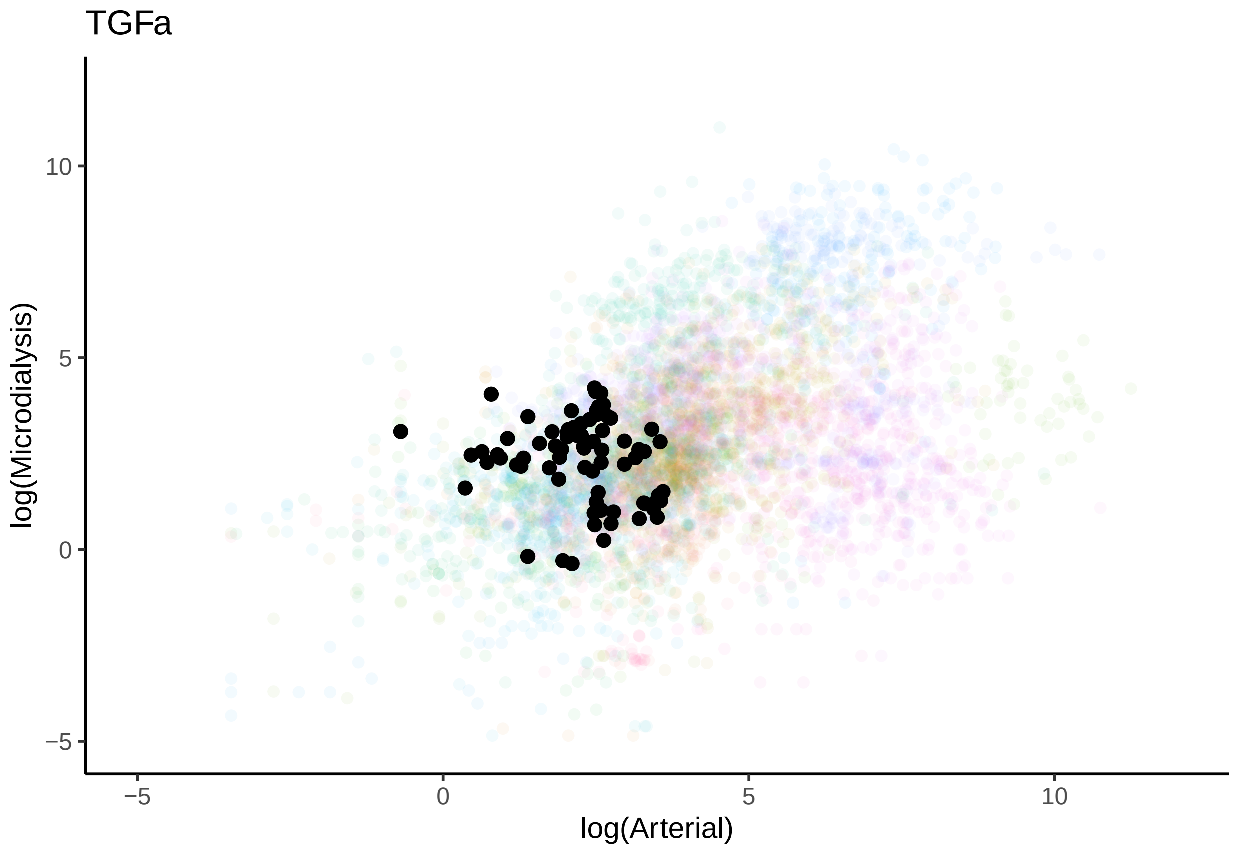


```
## Warning: Removed 10462 rows containing missing values (geom_point).

## Warning: Removed 290 rows containing missing values (geom_point).
```

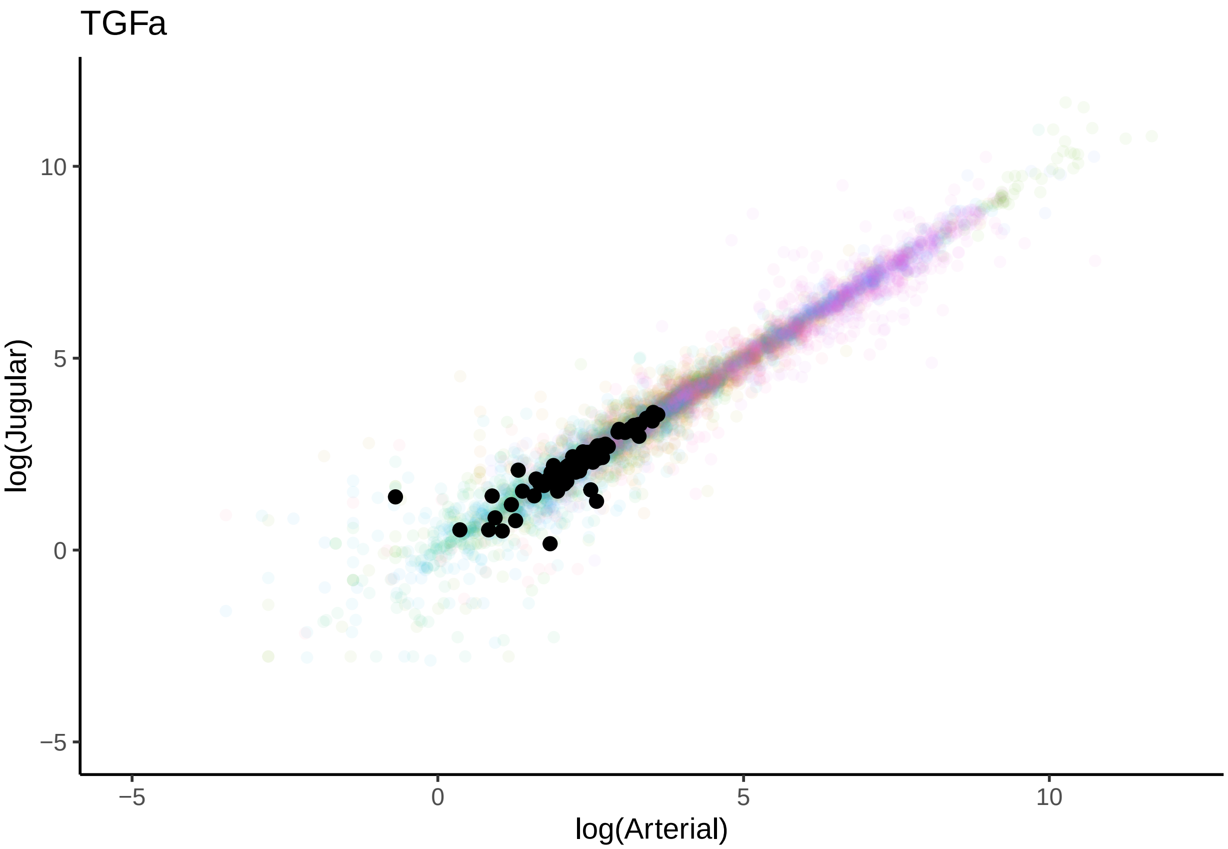


```
## Warning: Removed 10923 rows containing missing values (geom_point).
## Warning: Removed 232 rows containing missing values (geom_point).
```

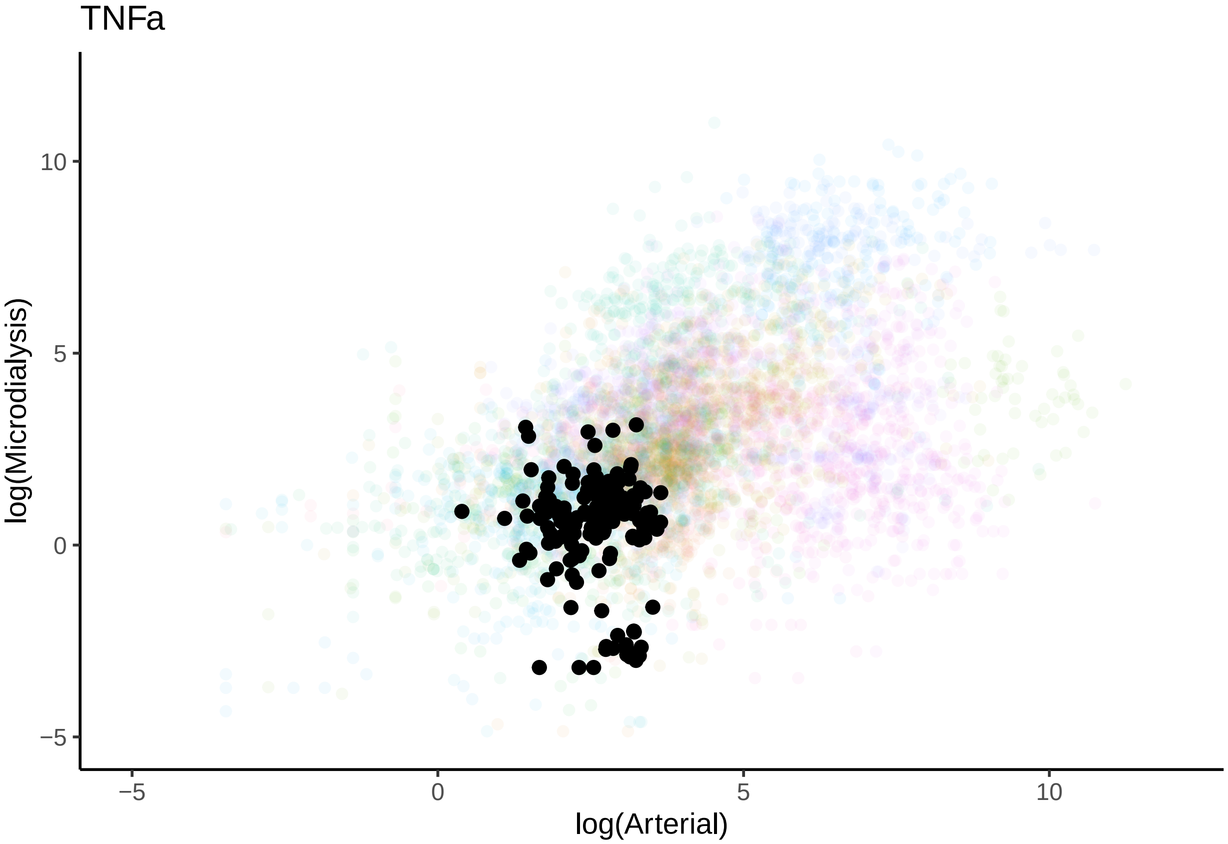


```
## Warning: Removed 10462 rows containing missing values (geom_point).
## Warning: Removed 213 rows containing missing values (geom_point).
```

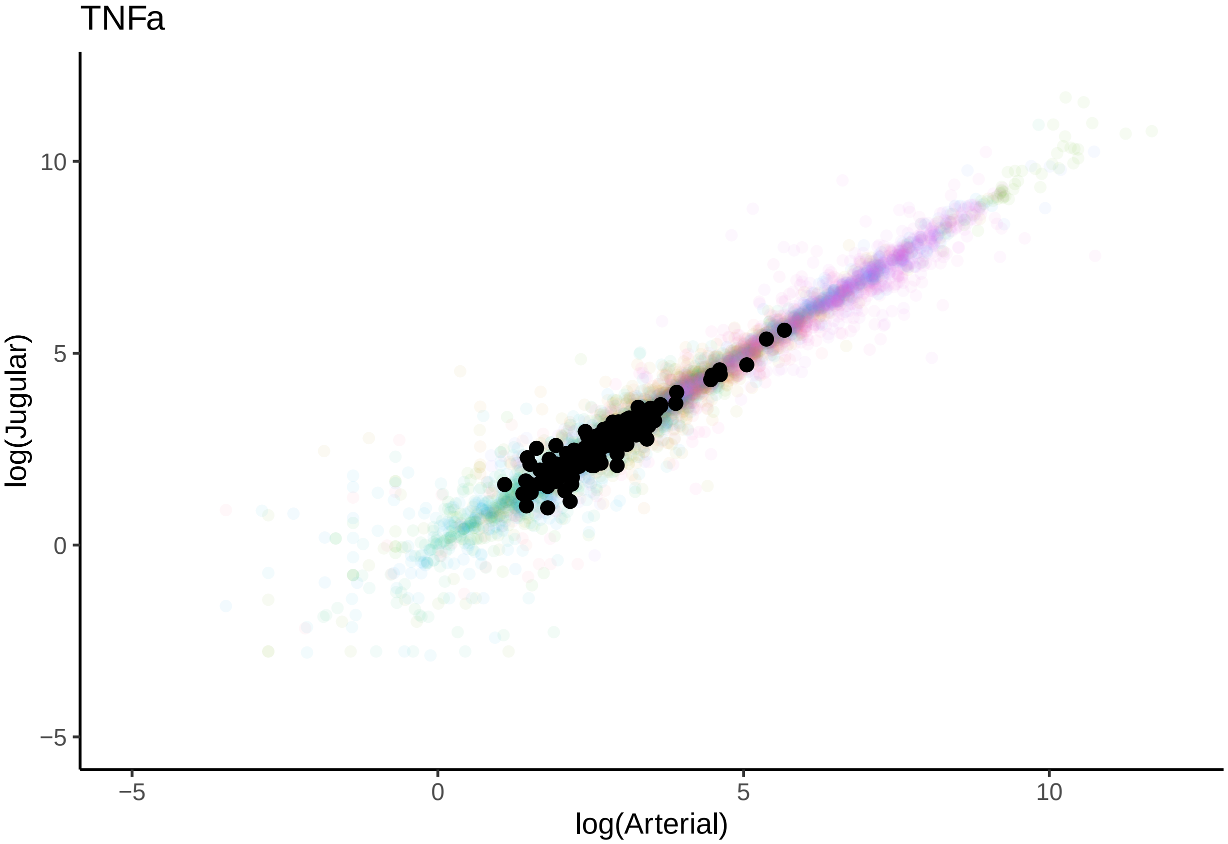


```
## Warning: Removed 10923 rows containing missing values (geom_point).

## Warning: Removed 346 rows containing missing values (geom_point).
```

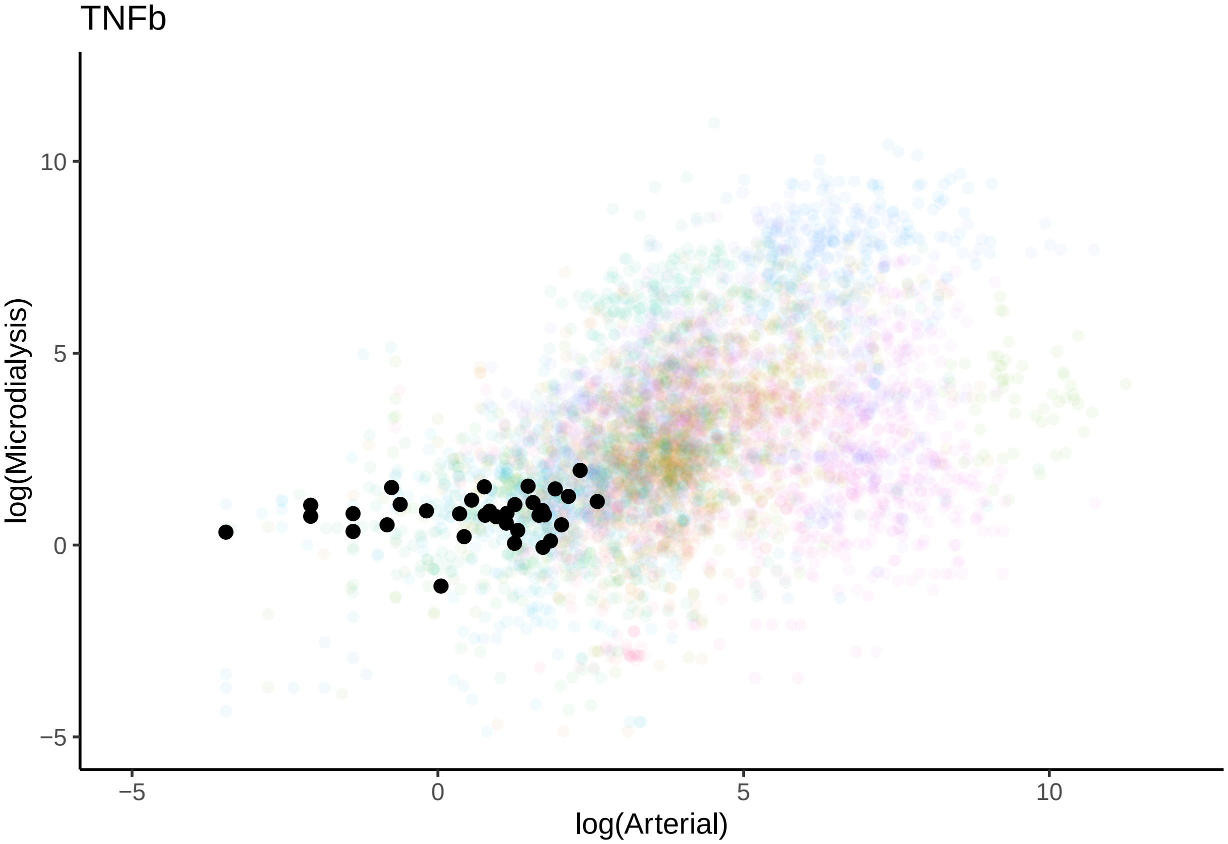


```
## Warning: Removed 10462 rows containing missing values (geom_point).

## Warning: Removed 324 rows containing missing values (geom_point).
```

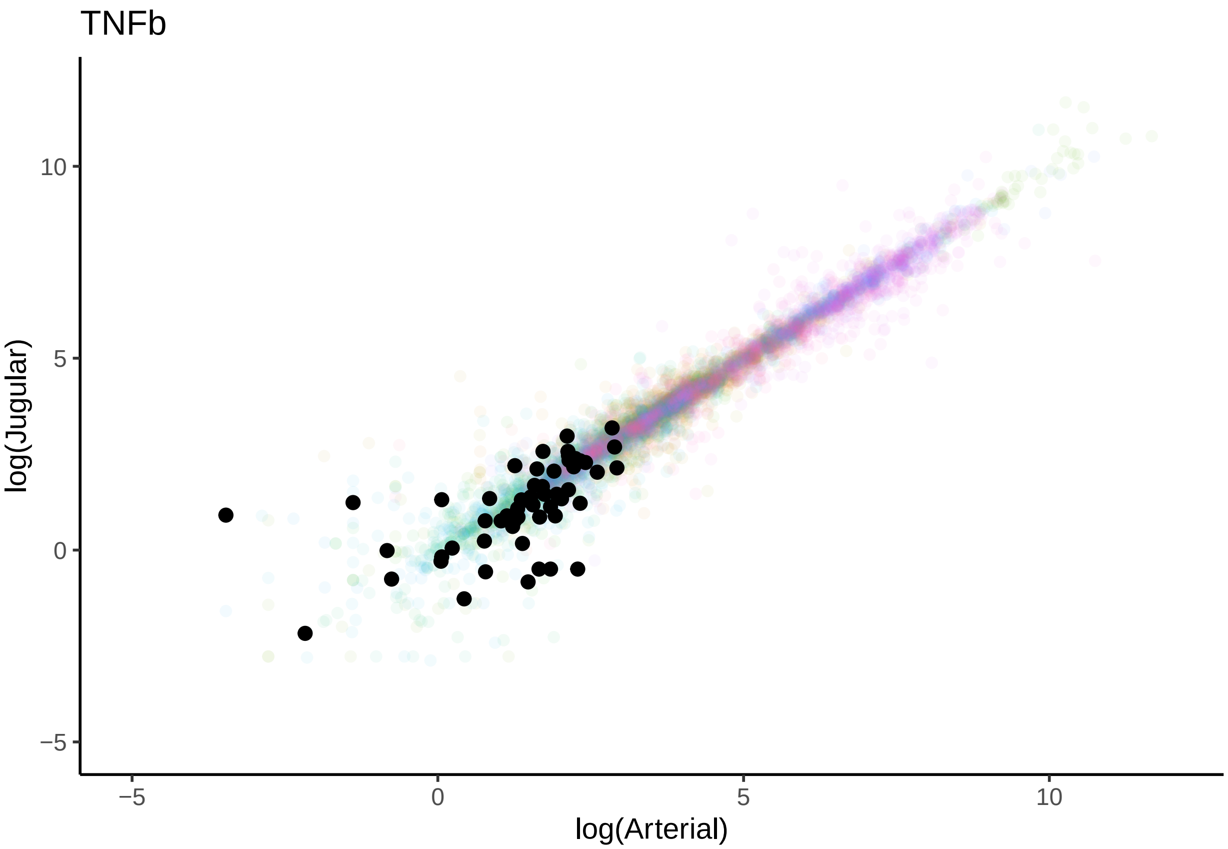


```
## Warning: Removed 10923 rows containing missing values (geom_point).
## Warning: Removed 224 rows containing missing values (geom_point).
```

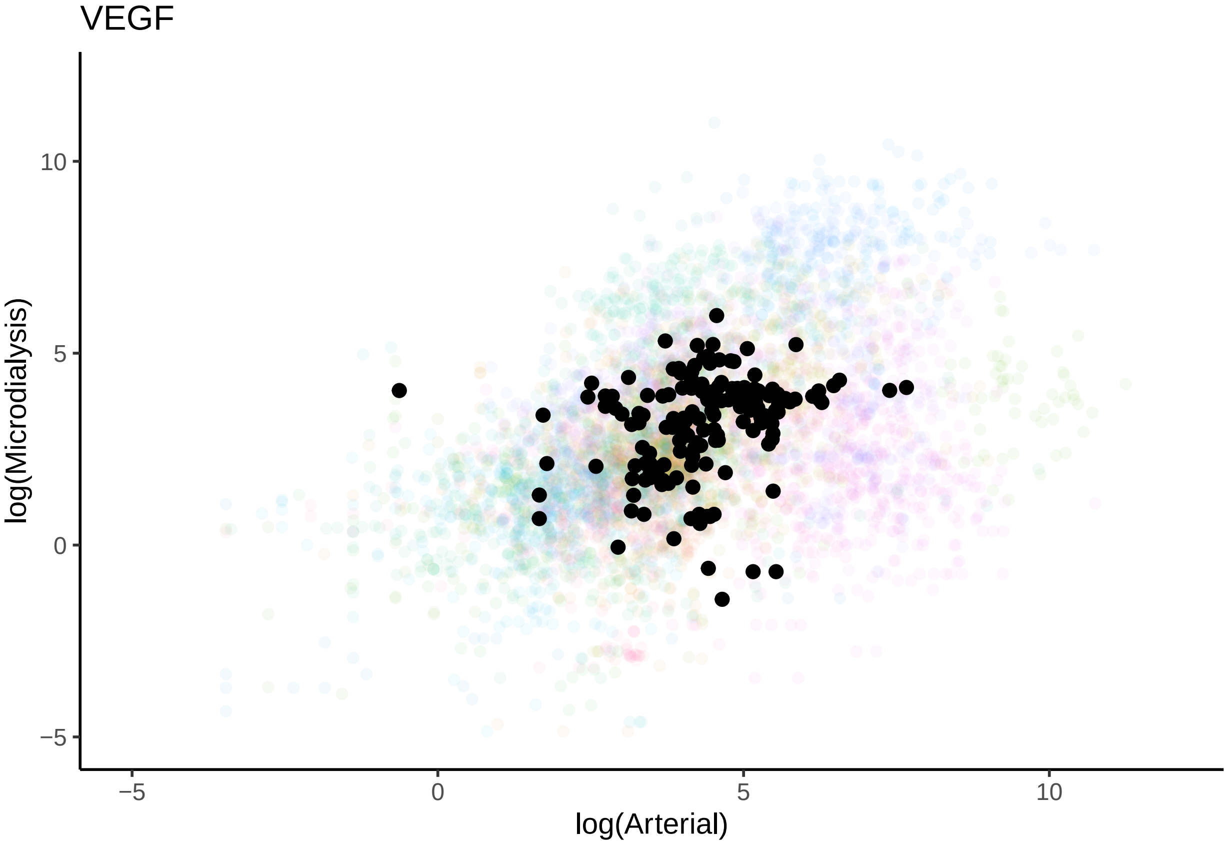


```
## Warning: Removed 10462 rows containing missing values (geom_point).

## Warning: Removed 219 rows containing missing values (geom_point).
```

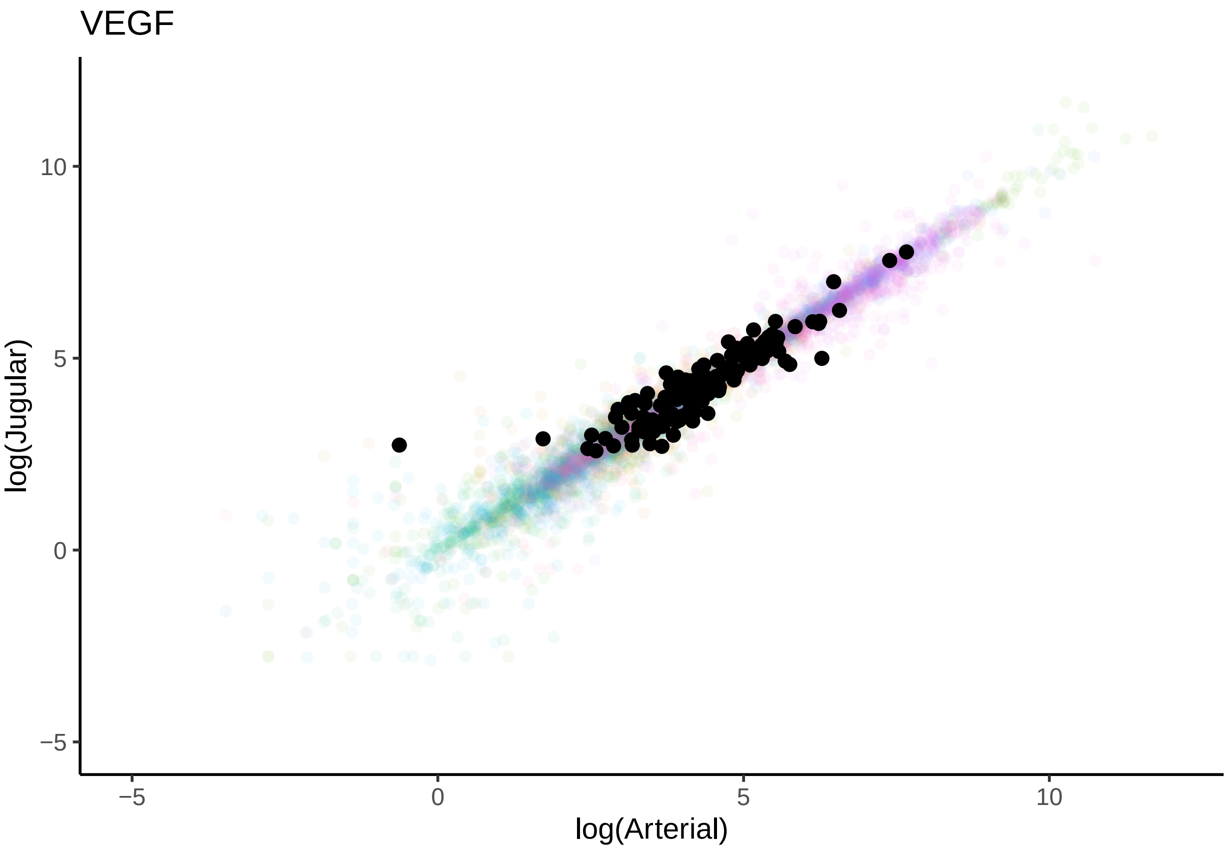
VEGF
log(Jugular)
10
5
0
−5
−5
0
5
10
log(Arterial)

Code for Figure 2

Philipp

Revised: May 11, 2021

Contents

Load the data **1**

Exclude inconclusive data **1**

Plot relevant ccfs **338**

Load the data

```
library(openxlsx)
ip_data <- read.xlsx("../Dataset/Interpolated_data4.2.xlsx")
```

Exclude inconclusive data

```
library(tidyr)
output <- matrix(NA, nrow = 20*42, ncol = 12)
colnames(output) <- c("Cytokine",
                      "Patient",
                      "Maximum_value",
                      "Lag",
                      "Included",
                      "Reason_for_exclusion",
                      "Treatment",
                      "Infection",
                      "Above_median_WCC",
                      "Above_median_CRP",
                      "Above_median_Temp",
                      "Above_median_ISS")
output <- data.frame(output)

median_WCC <- median(ip_data$WCC, na.rm = TRUE)
median_CRP <- median(ip_data$CRP, na.rm = TRUE)
median_Temp <- median(ip_data$Temp, na.rm = TRUE)
median_ISS <- median(ip_data$ISS, na.rm = TRUE)

reasons <- c("Included",
             "Surrounding of maximum not decreasing",
```

```
             "NAs surrounding maximum_value",
             "Maximum too close to edge",
             "Two equal maxima",
             "Only NAs")

output_compact <- matrix(NA, nrow = 42, ncol = length(reasons) + 1)
colnames(output_compact) <- c("Cytokine", reasons)
output_compact <- data.frame(output_compact)

library(ggplot2)
for (cytokine in 1:42) {
  cytname <- substr(colnames(ip_data)[cytokine * 3 + 5], start = 4, stop = 100)
  for (patient in 1:20) {
    rowcytpat <- (cytokine - 1) * 20 + patient
    output[rowcytpat, "Cytokine"] <- cytname
    output[rowcytpat, "Patient"] <- patient
    output[rowcytpat, "Treatment"] <- ifelse(patient > 10, "Yes", "No")
    output[rowcytpat, "Infection"] <-
      ifelse(any(na.omit(ip_data[ip_data$Patient == patient,
                                 "Infection"]) == 1), "Yes", "No")
    patmedian_WCC <- median(ip_data[ip_data$Patient == patient,
                                    "WCC"], na.rm = TRUE)
    output[rowcytpat, "Above_median_WCC"] <-
      ifelse(patmedian_WCC > median_WCC, "Yes", "No")
    patmedian_CRP <- median(ip_data[ip_data$Patient == patient,
                                    "CRP"], na.rm = TRUE)
    output[rowcytpat, "Above_median_CRP"] <-
      ifelse(patmedian_CRP > median_CRP, "Yes", "No")
    patmedian_Temp <- median(ip_data[ip_data$Patient == patient,
                                     "Temp"], na.rm = TRUE)
    output[rowcytpat, "Above_median_Temp"] <-
      ifelse(patmedian_Temp > median_Temp, "Yes", "No")
    patmedian_ISS <- median(ip_data[ip_data$Patient == patient,
                                    "ISS"], na.rm = TRUE)
    output[rowcytpat, "Above_median_ISS"] <-
      ifelse(patmedian_ISS > median_ISS, "Yes", "No")

    brain <- ip_data[ip_data$Patient == patient, 3 * cytokine + 5]
    blood <- ip_data[ip_data$Patient == patient, 3 * cytokine + 6]
    cross <- ccf(brain, blood, na.action = na.pass, plot = FALSE)
    cross.val <- cross$acf
    cross.lag <- cross$lag
    cross.both <- cbind(cross.lag, cross.val)
    colnames(cross.both) <- c("Lag", "Crosscorr")
    if (!all(is.na(cross.val))) {
      max_val <- max(abs(cross.val), na.rm = TRUE)
      which_max_val <- max_val == abs(cross.val)
      max_vec <- c(rep("No", dim(cross.both)[1]))
      max_vec[which_max_val] <- "Yes"
      cross.all <- data.frame(cross.both, Max = max_vec)
      max_num <- which(which_max_val)
      if (!length(max_num) > 1) {
        if (max_num >= 3 & max_num + 2 <= dim(cross.val)[1]) {
```

```
      if (!anyNA(cross.val[c((max_num-2):(max_num+2))])) {
        if ((cross.val[max_num] > cross.val[max_num-1] &
            cross.val[max_num-1] > cross.val[max_num-2] &
            cross.val[max_num] > cross.val[max_num+1] &
            cross.val[max_num+1] > cross.val[max_num+2]) ||
            (cross.val[max_num] < cross.val[max_num-1] &
            cross.val[max_num-1] < cross.val[max_num-2] &
            cross.val[max_num] < cross.val[max_num+1] &
            cross.val[max_num+1] < cross.val[max_num+2])) {
          output[rowcytpat, "Maximum_value"] <- cross.val[max_num]
          output[rowcytpat, "Lag"] <- cross.all[max_num, "Lag"]
          output[rowcytpat, "Included"] <- "Yes"
          output[rowcytpat, "Reason_for_exclusion"] <- reasons[1]

          p1 <- ggplot(data = cross.all, aes(x = Lag, y = Crosscorr)) +
            geom_point(aes(color = Max), show.legend = FALSE) +
            scale_color_manual(values = c("Yes" = "red",
                                          "No" = "darkslateblue")) +
            ggtitle(paste(substr(colnames(ip_data)[cytokine * 3 + 5],
                                 start = 4, stop = 100),
                          "for patient", patient)) +
            theme_classic() +
            geom_hline(yintercept = 0, linetype = "dashed", color = "grey80") +
            geom_vline(xintercept = 0, linetype = "dashed", color = "grey80") +
            xlim(-10, 10) +
            ylim(-1, 1)
          print(p1)

          a <- t.test(c(cross.val))
          print(paste(substr(colnames(ip_data)[cytokine * 3 + 5],
                             start = 4, stop = 100),
                      "for patient", patient,
                       "- p-value:", a$p.value))
        } else {
          output[rowcytpat, "Included"] <- "No"
          output[rowcytpat, "Reason_for_exclusion"] <- reasons[2]
        }
      } else {
        output[rowcytpat, "Included"] <- "No"
        output[rowcytpat, "Reason_for_exclusion"] <- reasons[3]
      }
    } else {
      output[rowcytpat, "Included"] <- "No"
      output[rowcytpat, "Reason_for_exclusion"] <- reasons[4]
    }
  } else {
    output[rowcytpat, "Included"] <- "No"
    output[rowcytpat, "Reason_for_exclusion"] <- reasons[5]
  }
} else {
  output[rowcytpat, "Included"] <- "No"
  output[rowcytpat, "Reason_for_exclusion"] <- reasons[6]
}
```

```
  }
  output_compact[cytokine, 1] <- cytname
  for (i in 1:length(reasons)) {
    output_compact[cytokine, i + 1] <-
      sum(output[((cytokine - 1) * 20 + 1):(cytokine * 20),
                 "Reason_for_exclusion"] == reasons[i])
  }
}
```

```
## Warning: Removed 1 rows containing missing values (geom_point).
```

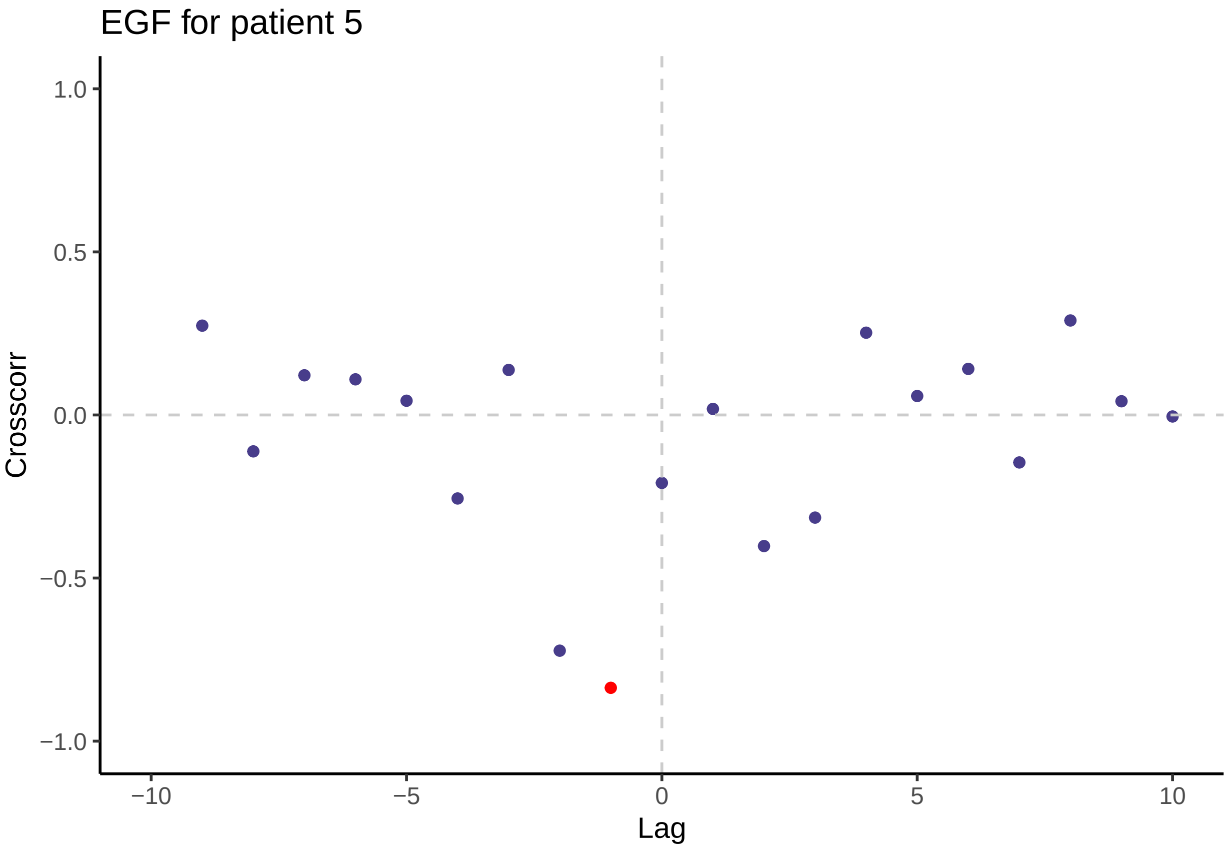


```
## [1] "EGF for patient 5 - p-value: 0.286068148149277"
```

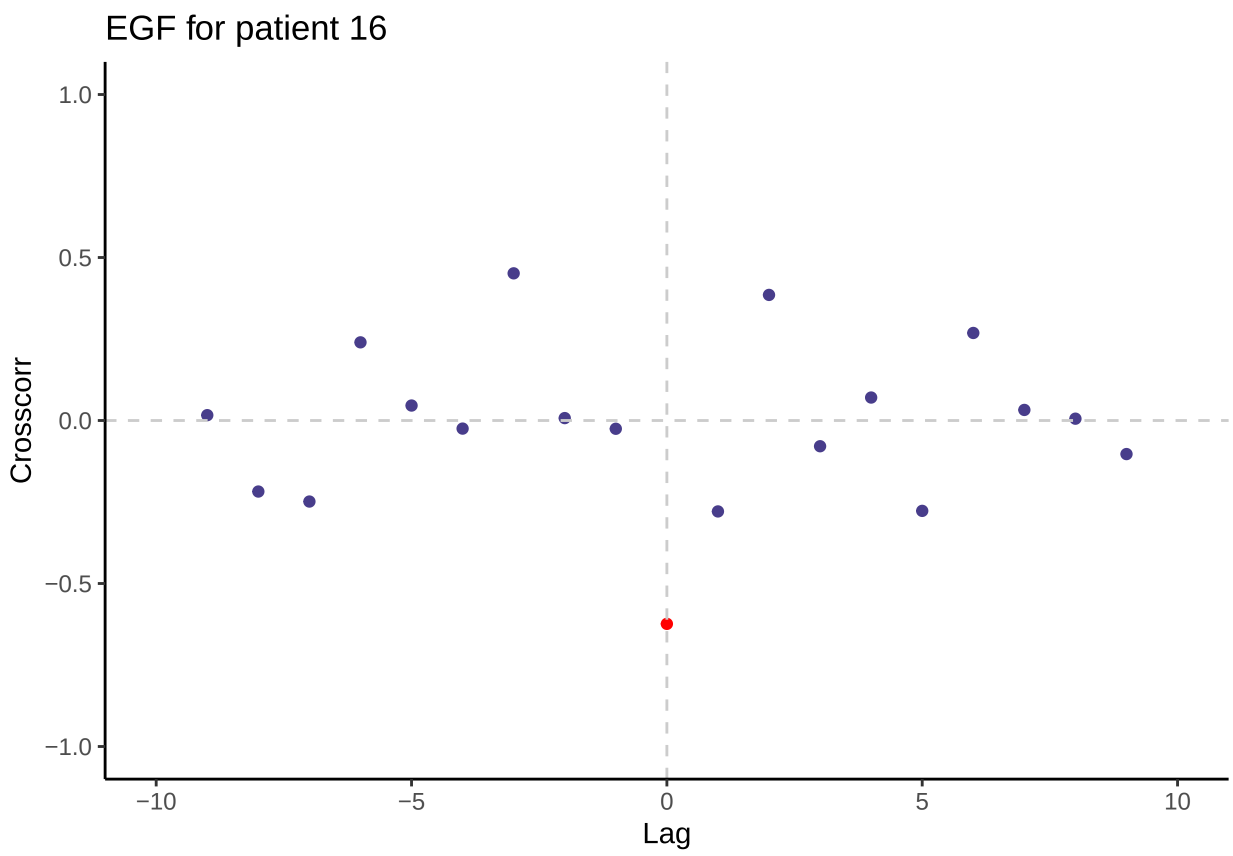


```
## [1] "EGF for patient 16 - p-value: 0.751469421418132"
```

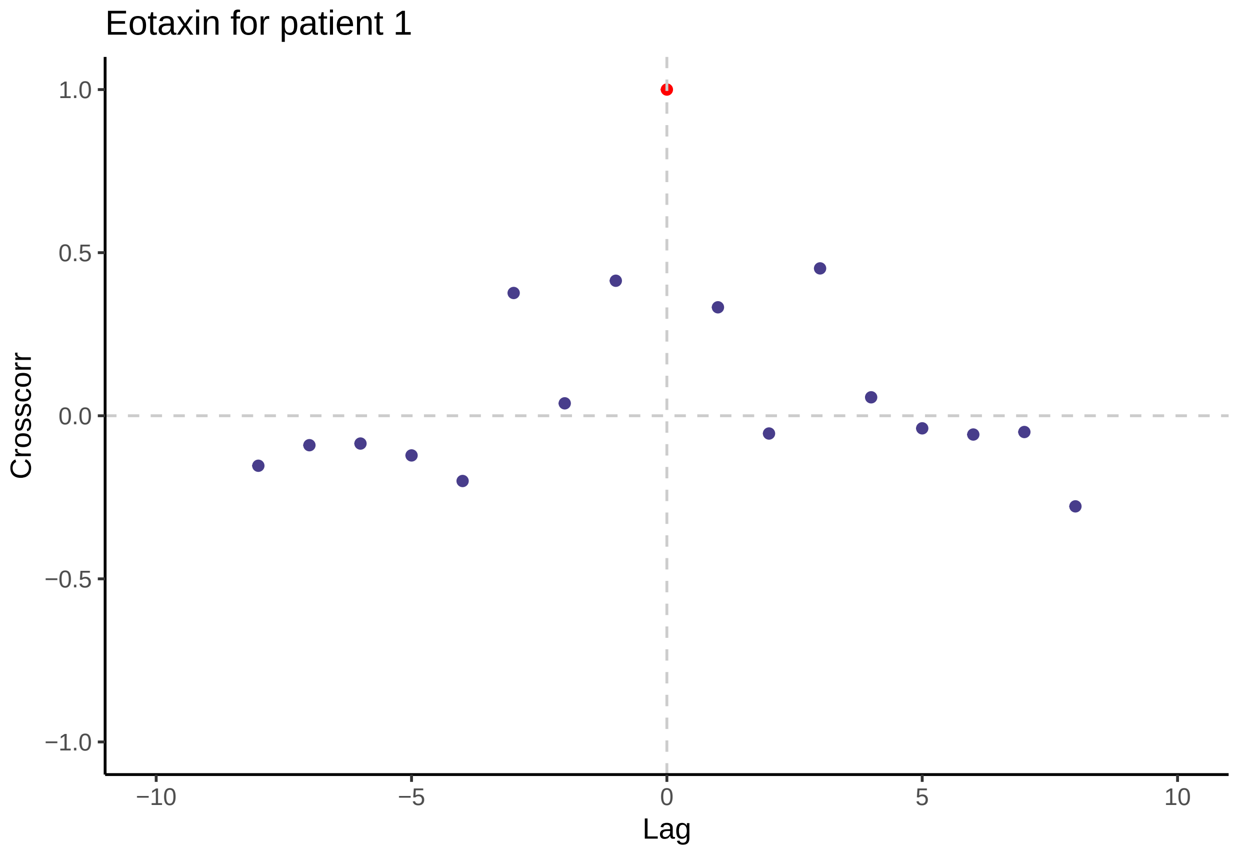


```
## [1] "Eotaxin for patient 1 - p-value: 0.264880571174586"
```

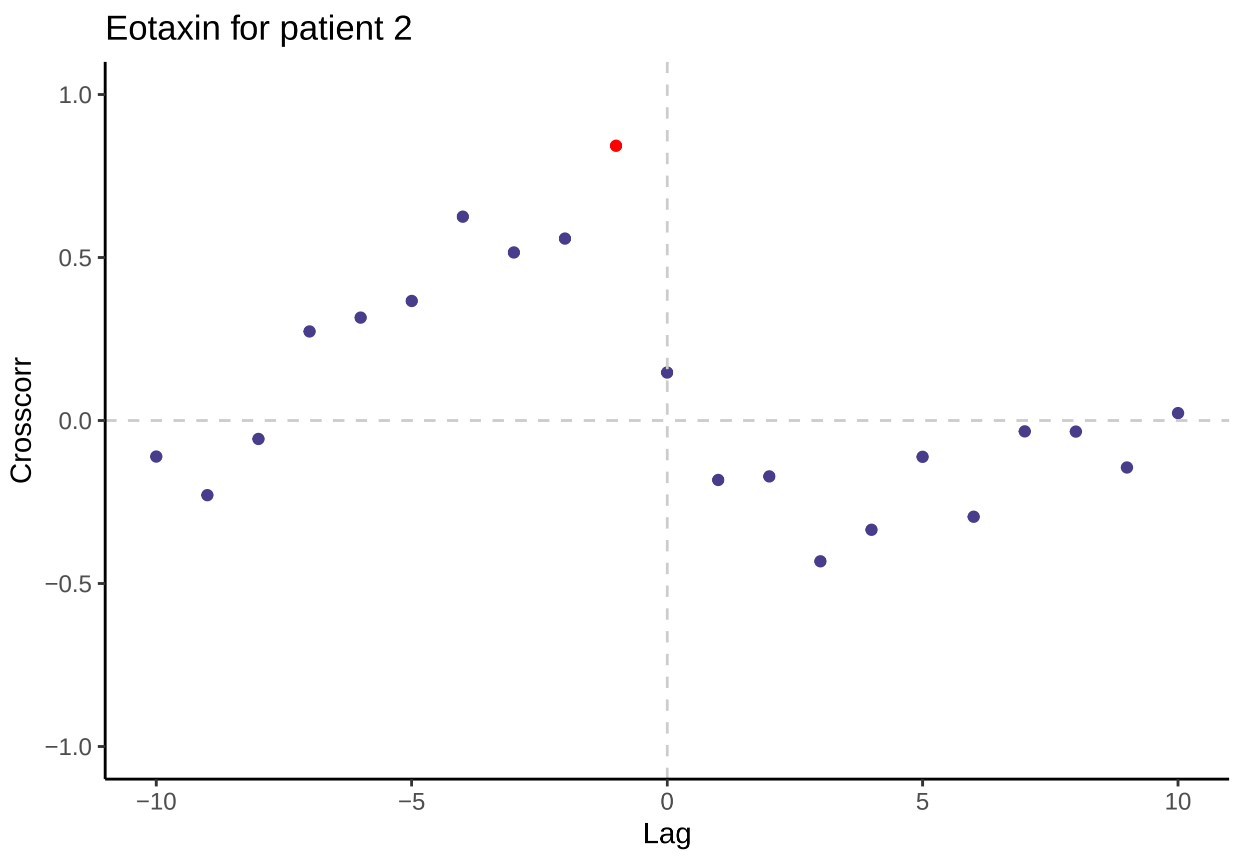


```
## [1] "Eotaxin for patient 2 - p-value: 0.35080322013721"
```

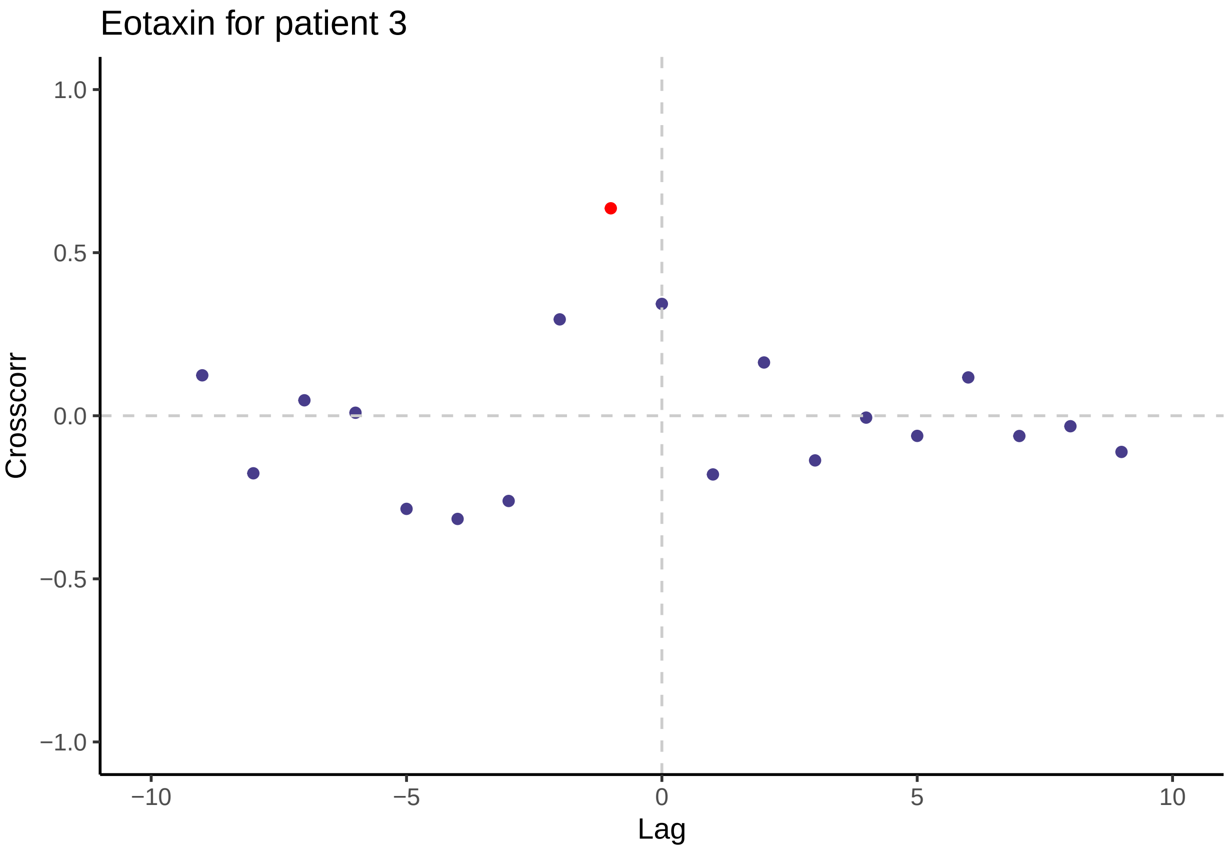


```
## [1] "Eotaxin for patient 3 - p-value: 0.91887280458073"
```

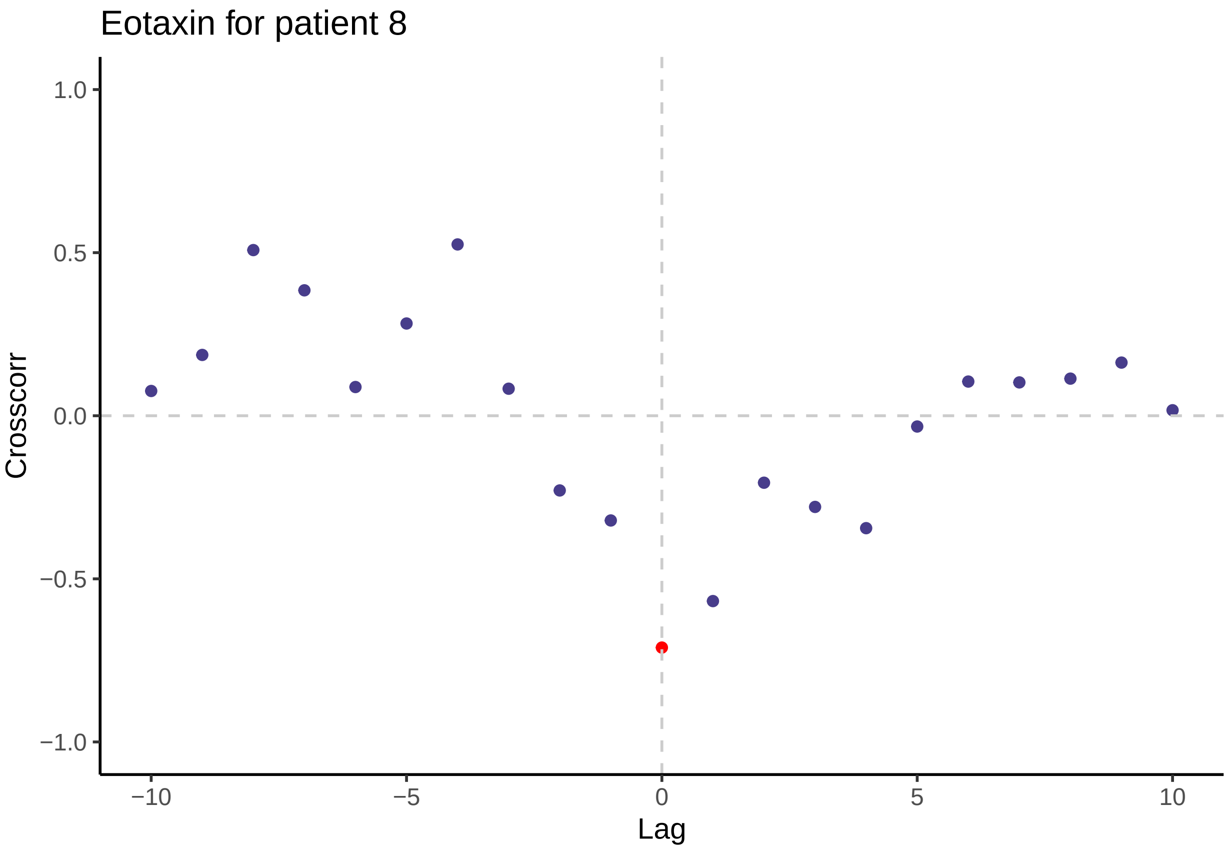


```
## [1] "Eotaxin for patient 8 - p-value: 0.970123481747339"
```

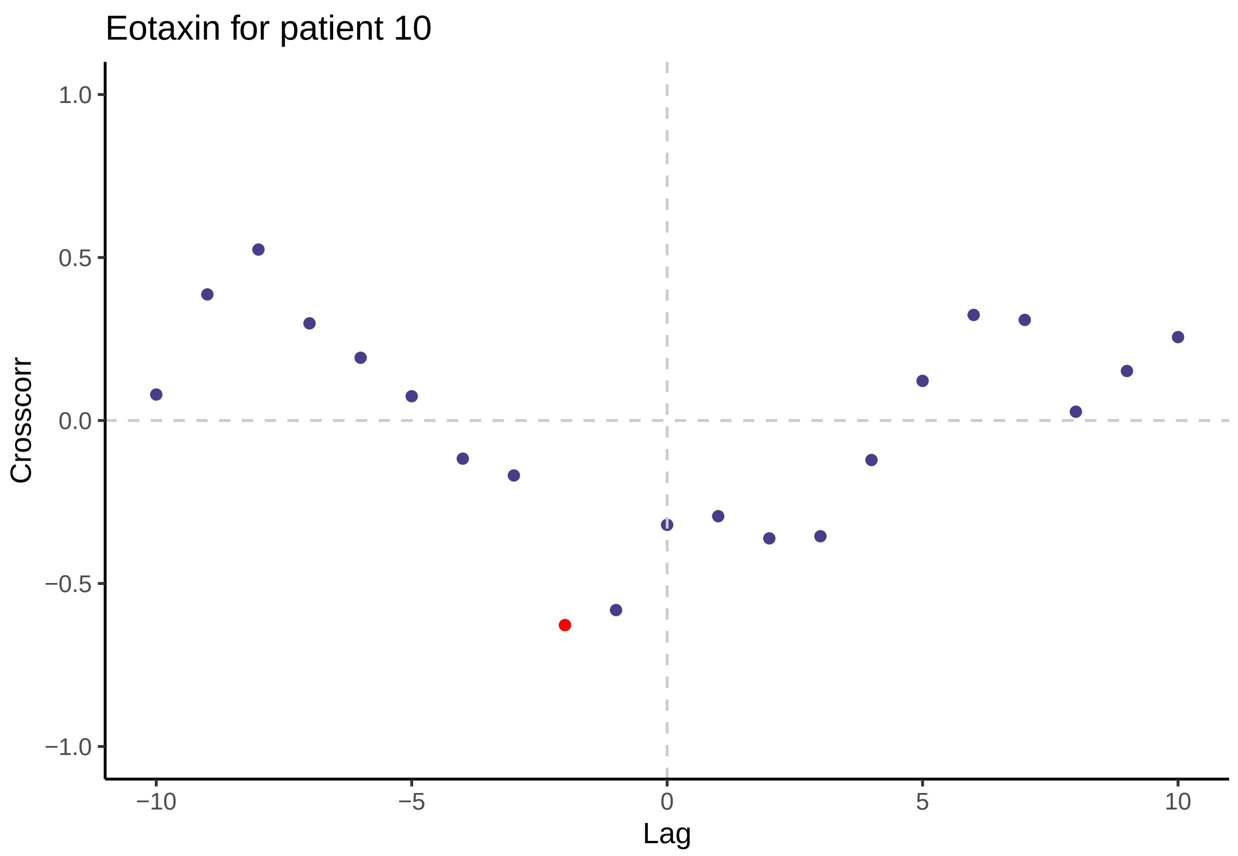


```
## [1] "Eotaxin for patient 10 - p-value: 0.893688430113908"
```

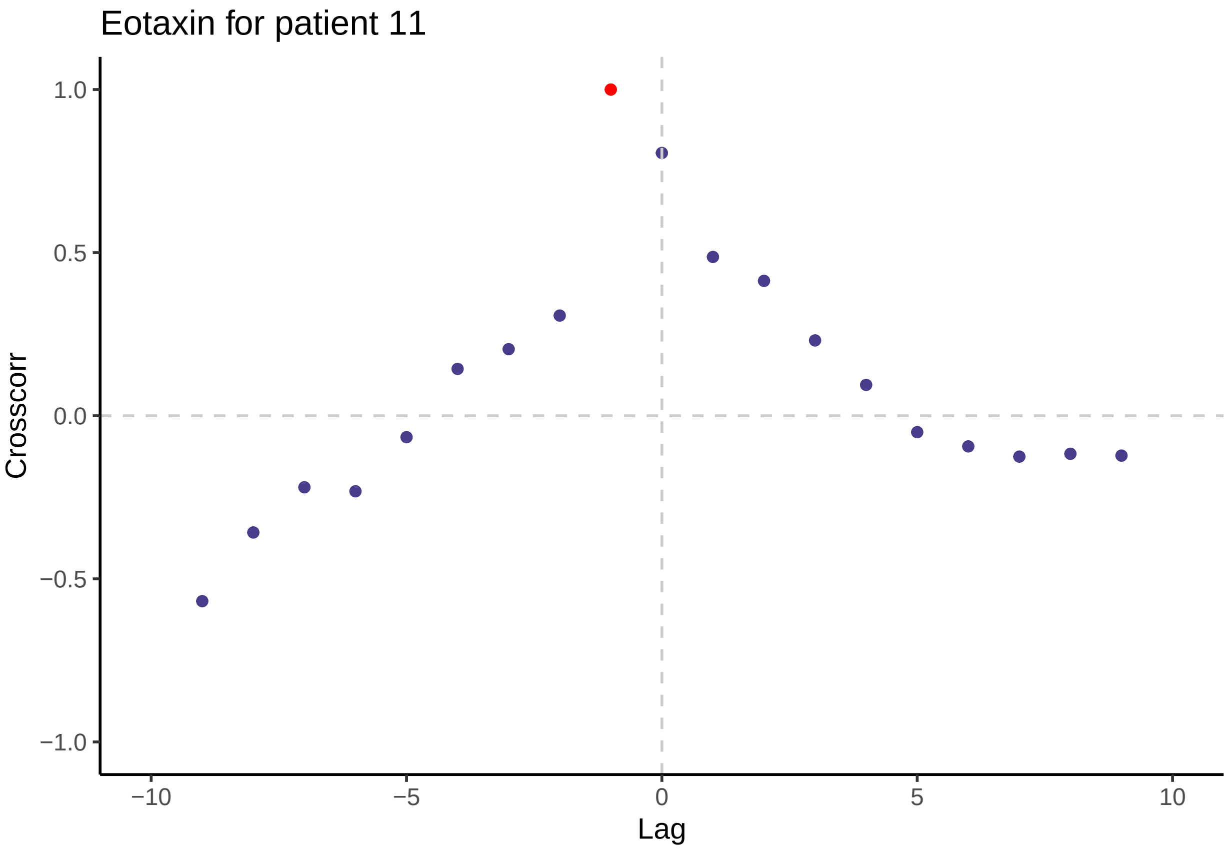


```
## [1] "Eotaxin for patient 11 - p-value: 0.320293960054486"
## Warning: Removed 1 rows containing missing values (geom_point).
```

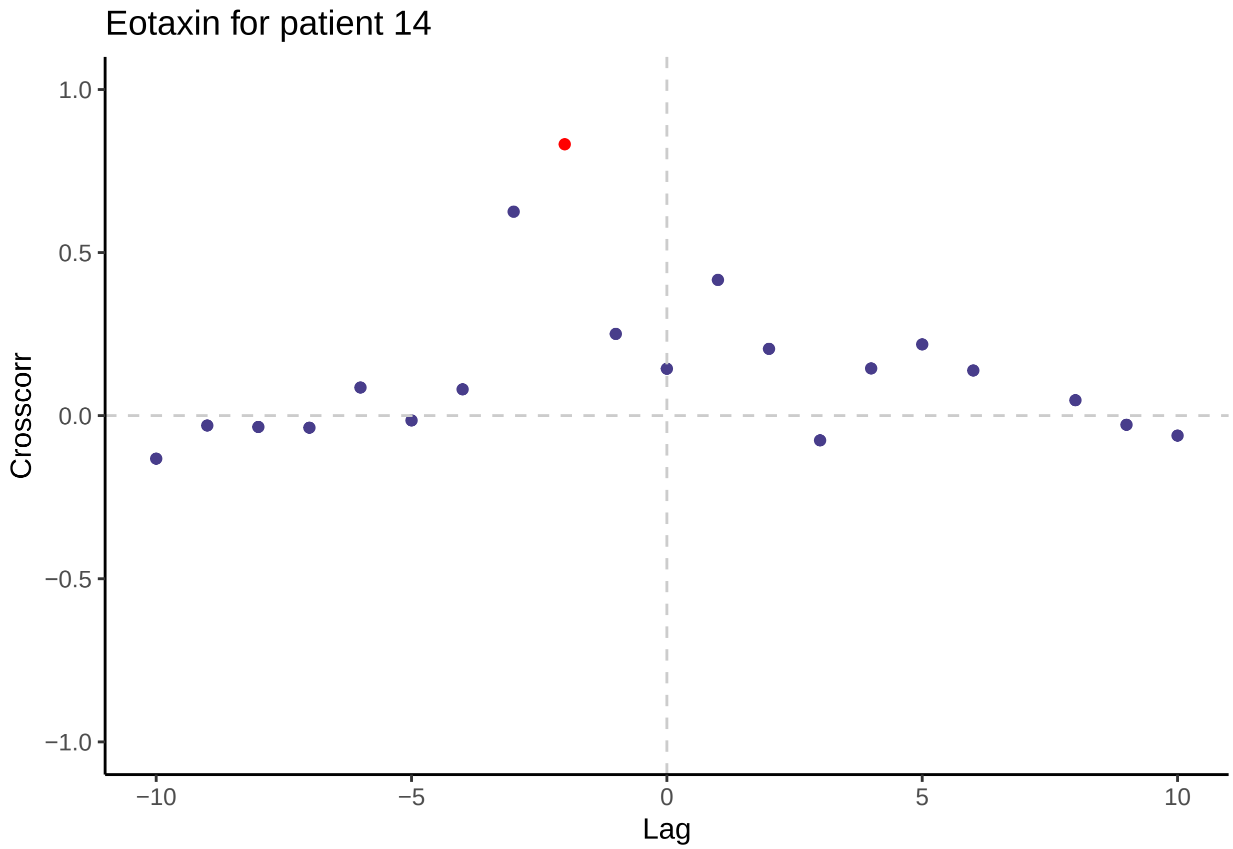


```
## [1] "Eotaxin for patient 14 - p-value: 0.0196106679654882"
```

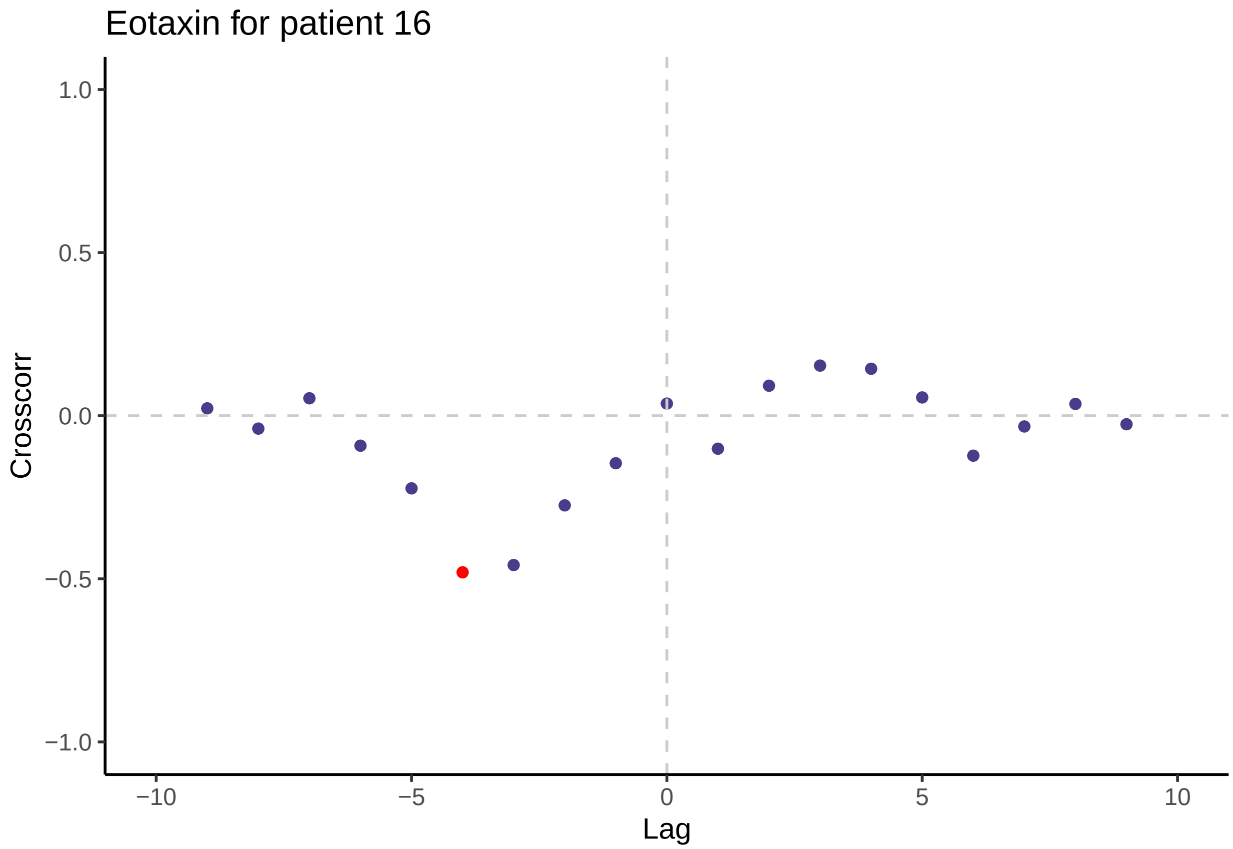


```
## [1] "Eotaxin for patient 16 - p-value: 0.0913875204884545"
```

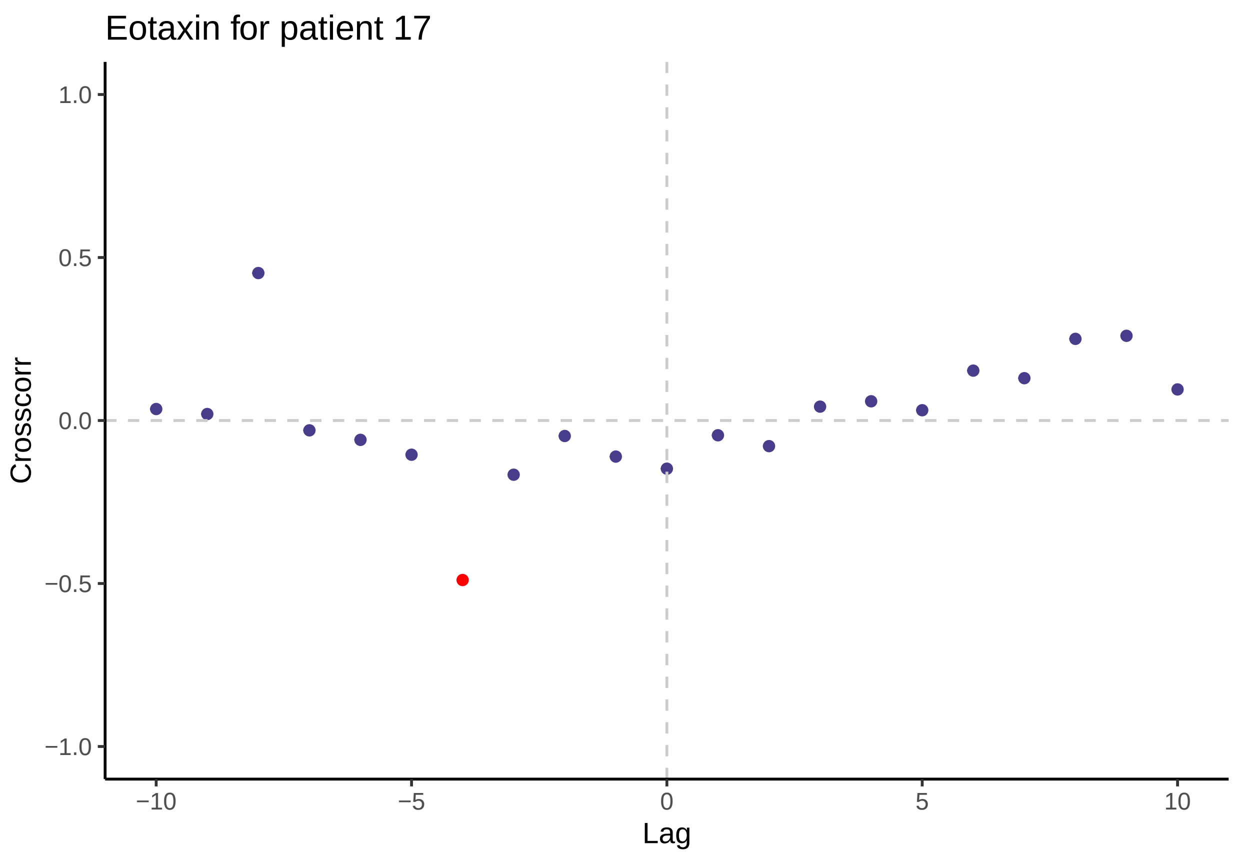


```
## [1] "Eotaxin for patient 17 - p-value: 0.776656465115091"
```

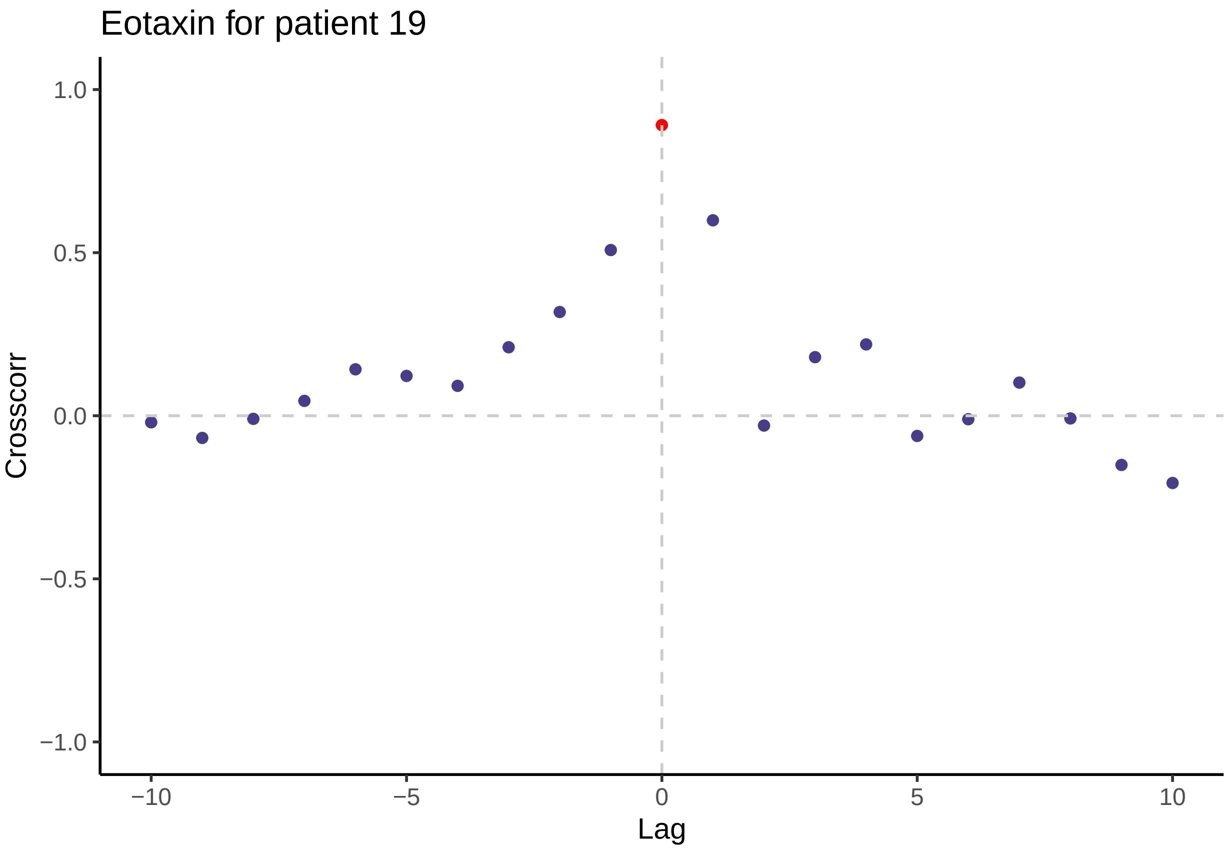


```
## [1] "Eotaxin for patient 19 - p-value: 0.0273606138836972"
```

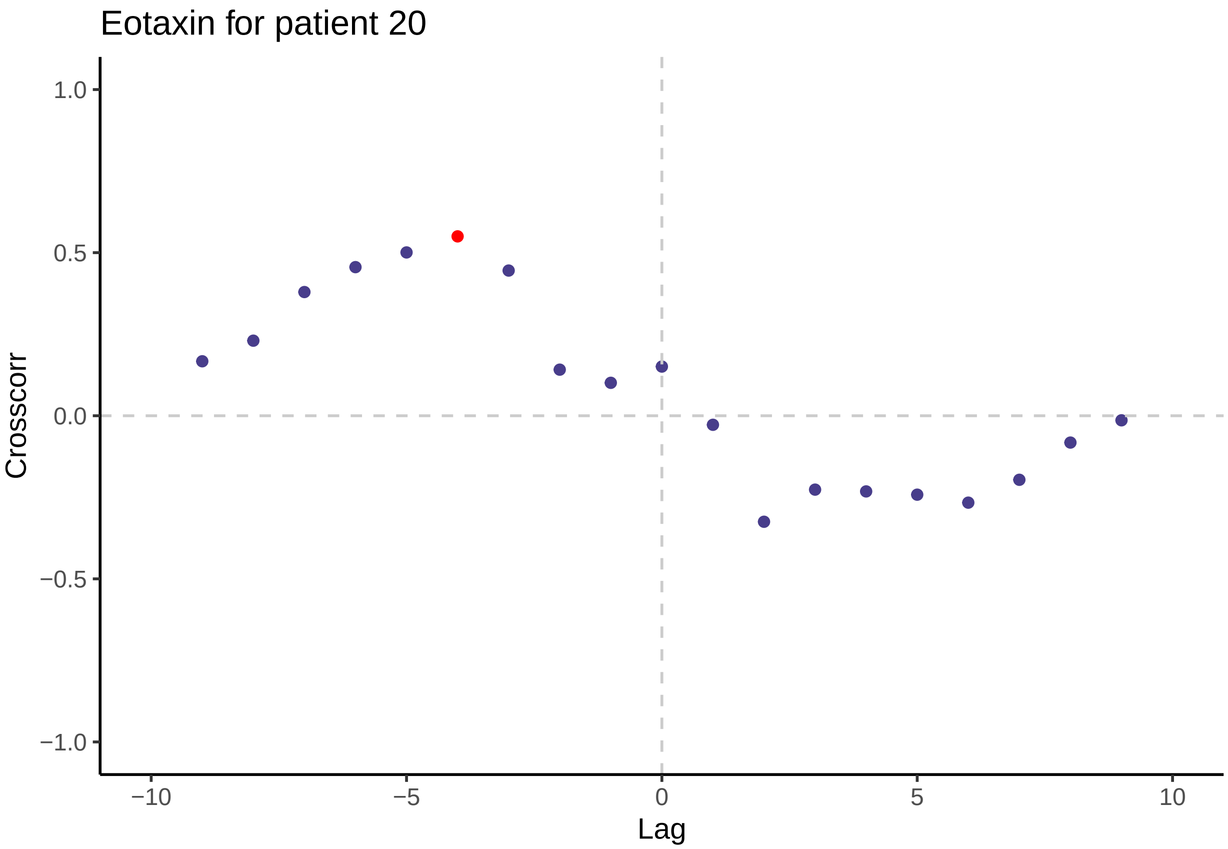


```
## [1] "Eotaxin for patient 20 - p-value: 0.246428463579744"
```

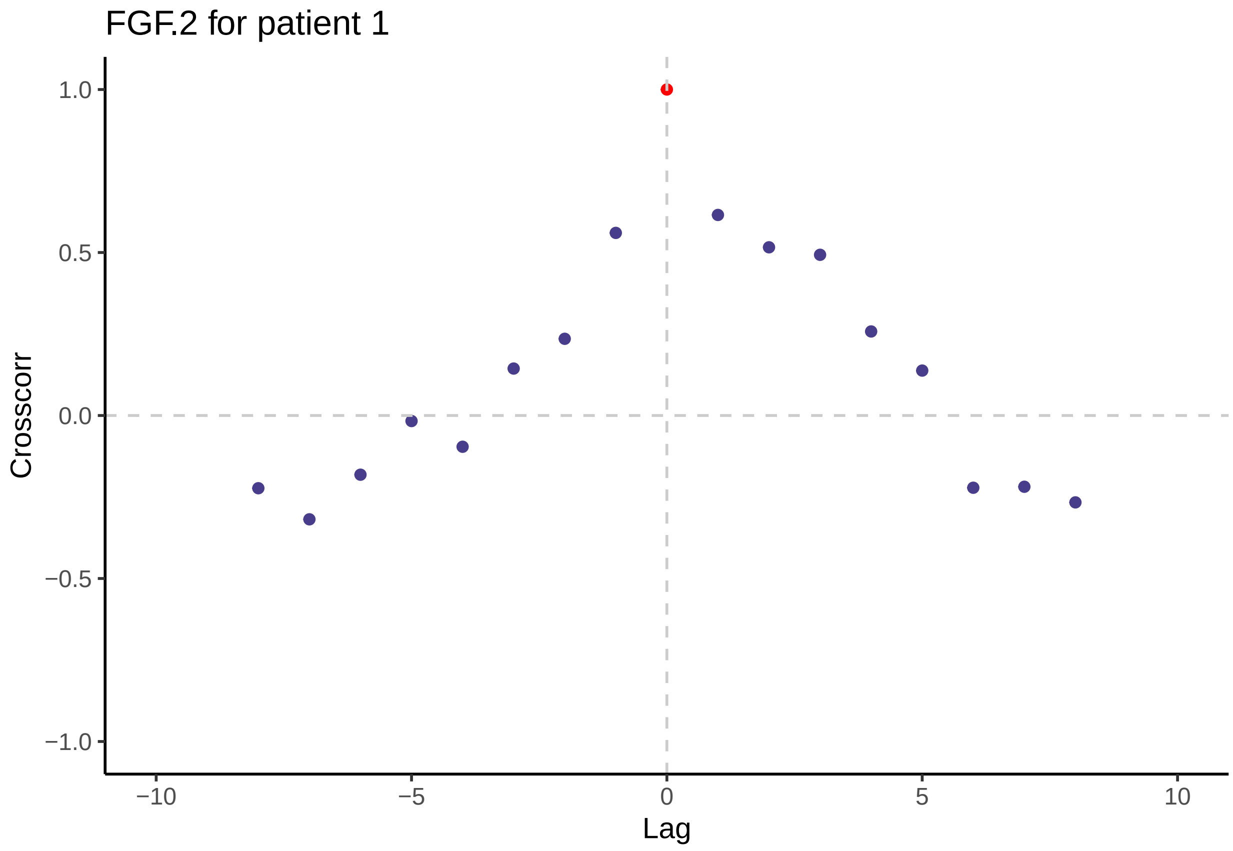


```
## [1] "FGF.2 for patient 1 - p-value: 0.148092534237168"
```

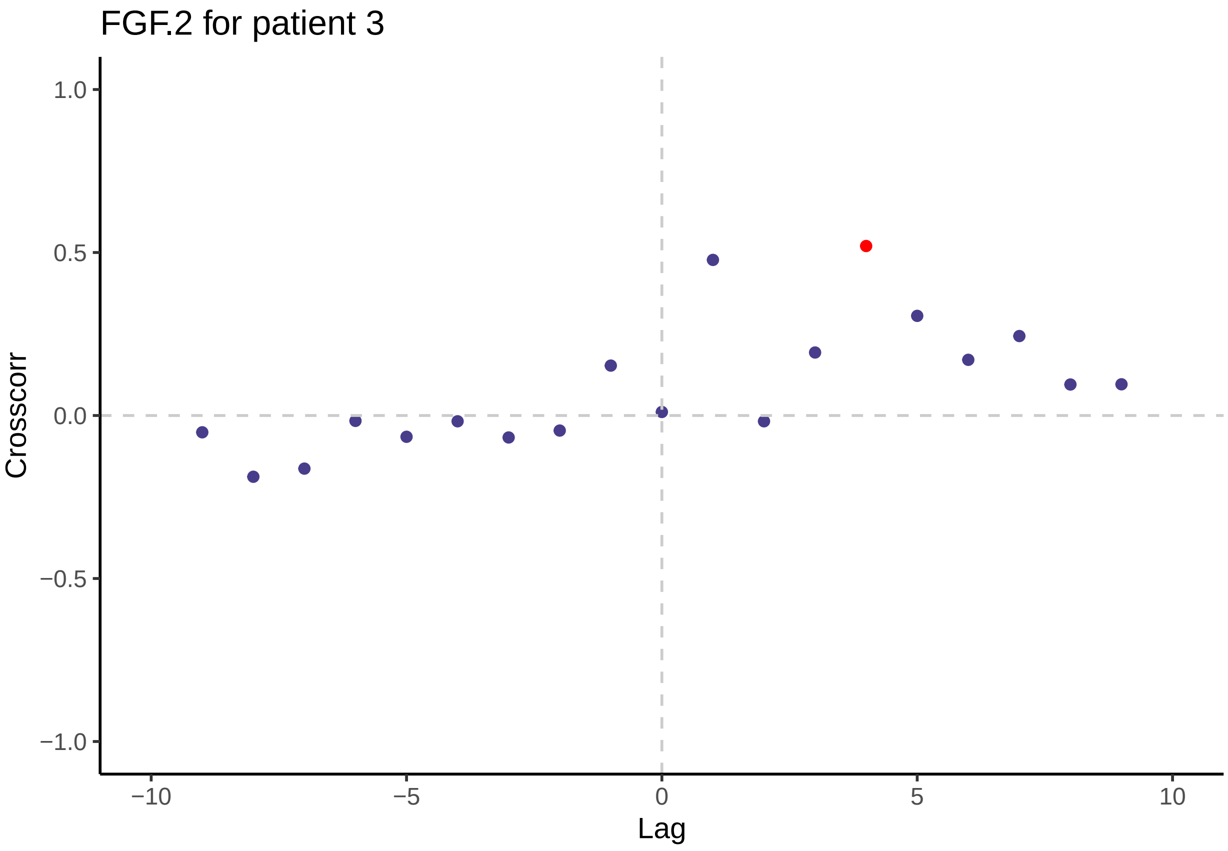


```
## [1] "FGF.2 for patient 3 - p-value: 0.0727420088737739"
```

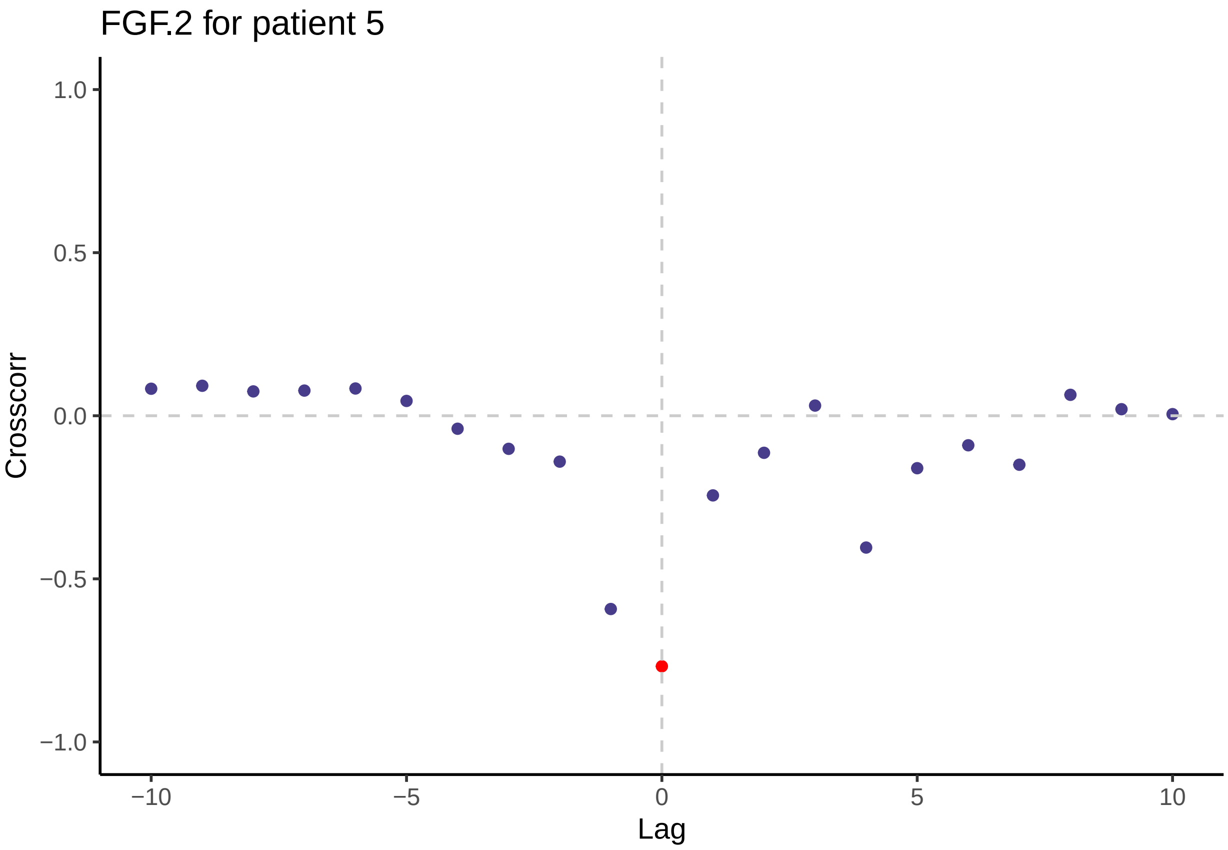


```
## [1] "FGF.2 for patient 5 - p-value: 0.0482695170699199"
```

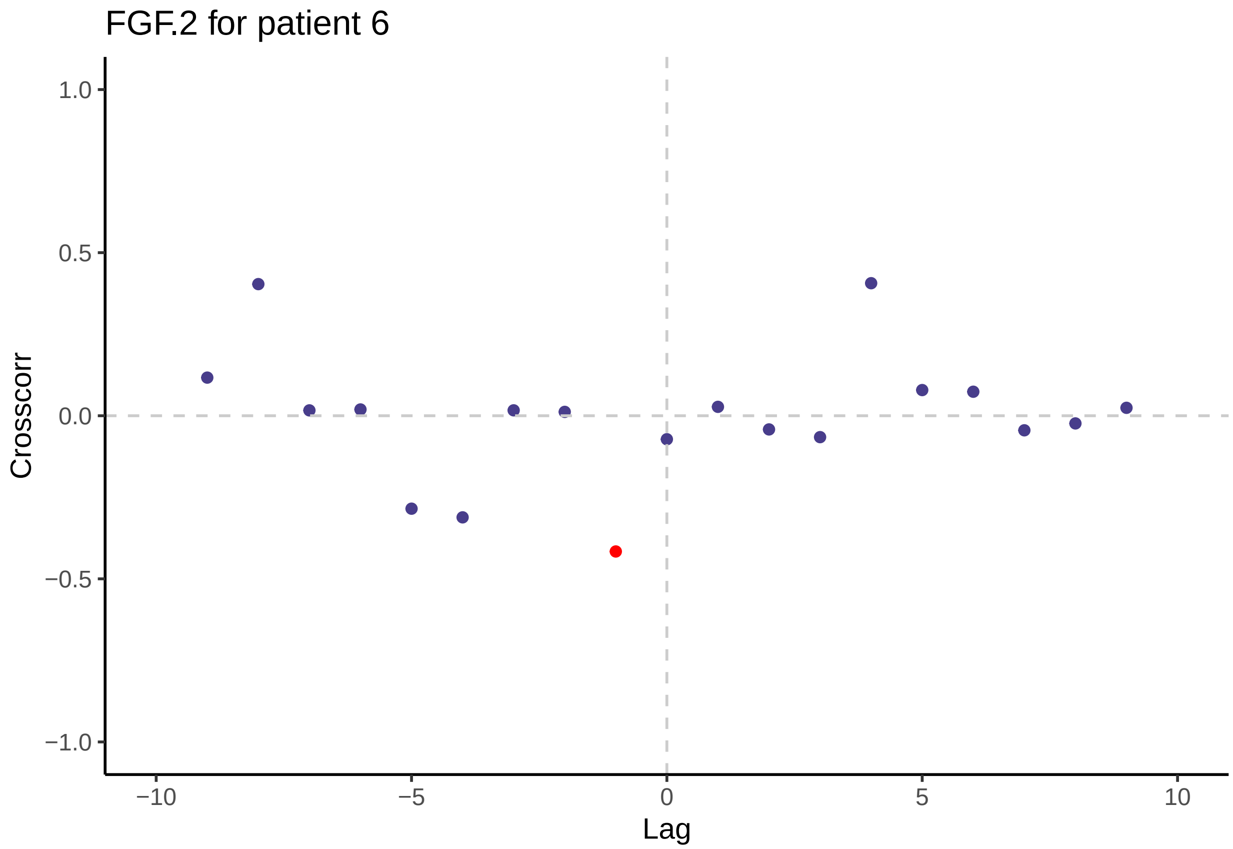


```
## [1] "FGF.2 for patient 6 - p-value: 0.941658706374537"
```

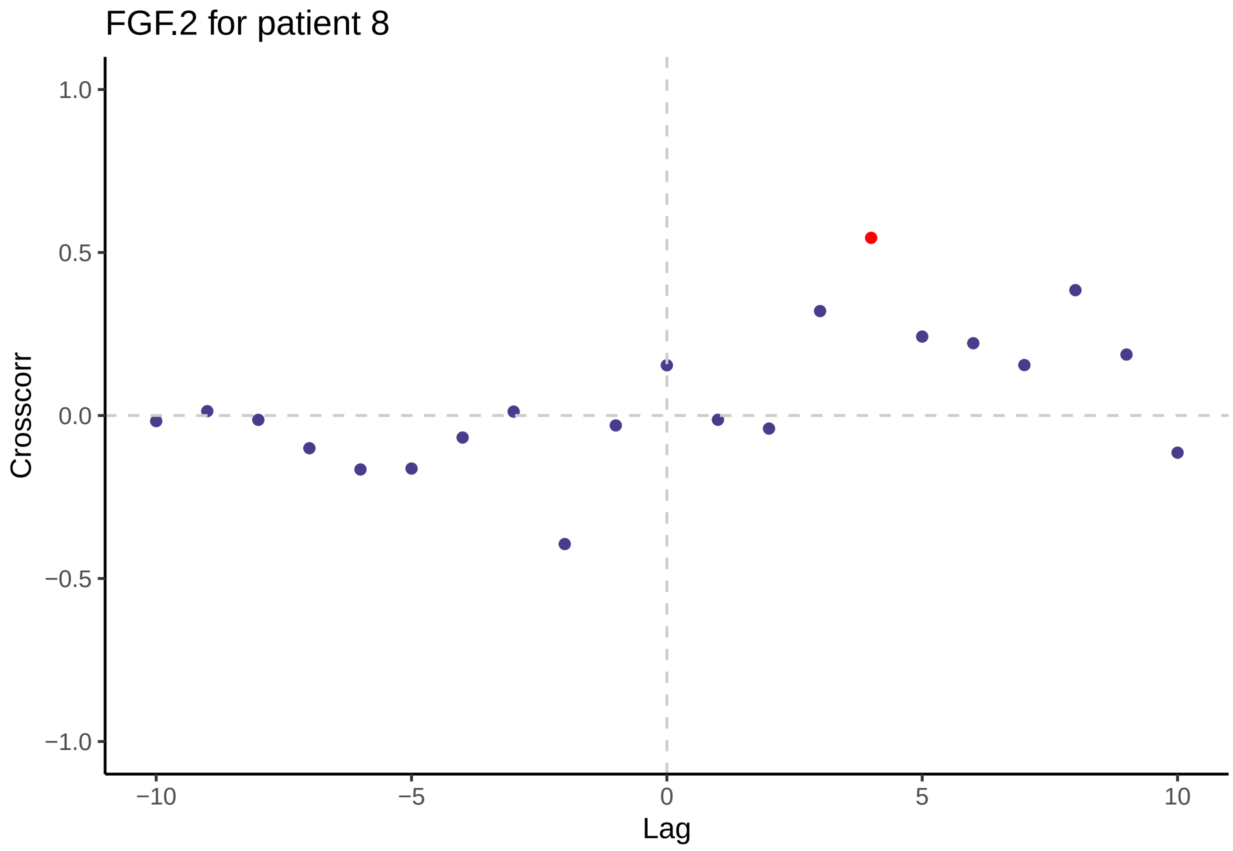


```
## [1] "FGF.2 for patient 8 - p-value: 0.269212989748638"
```

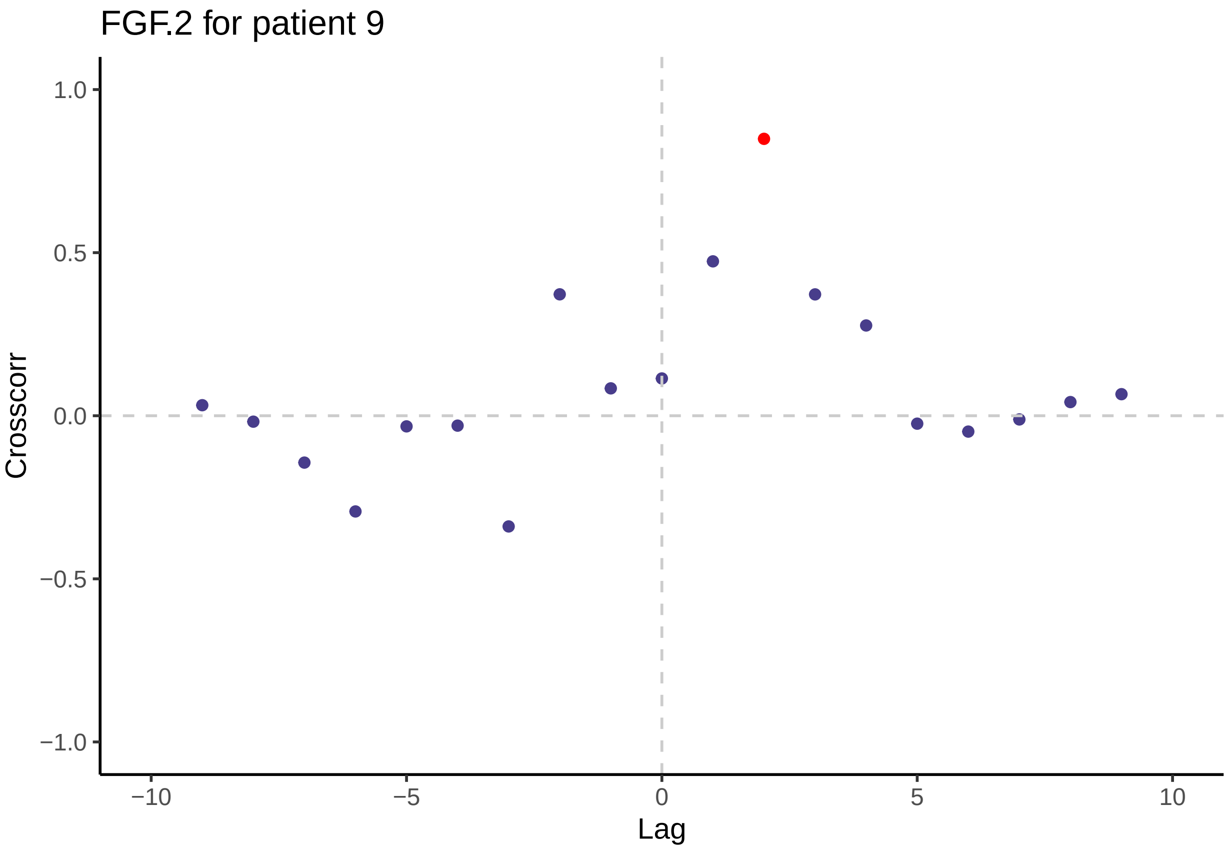


```
## [1] "FGF.2 for patient 9 - p-value: 0.168327250066343"
```

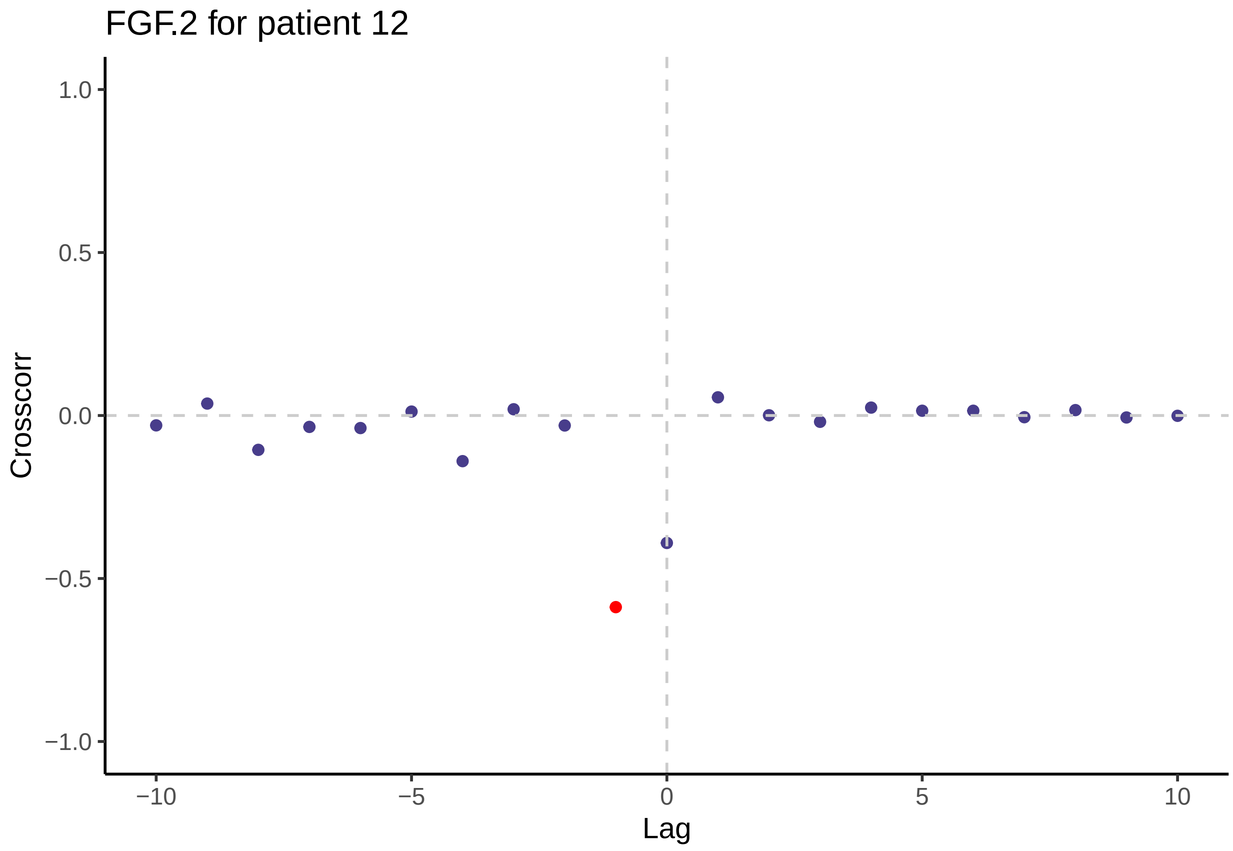


```
## [1] "FGF.2 for patient 12 - p-value: 0.105131716798449"
```

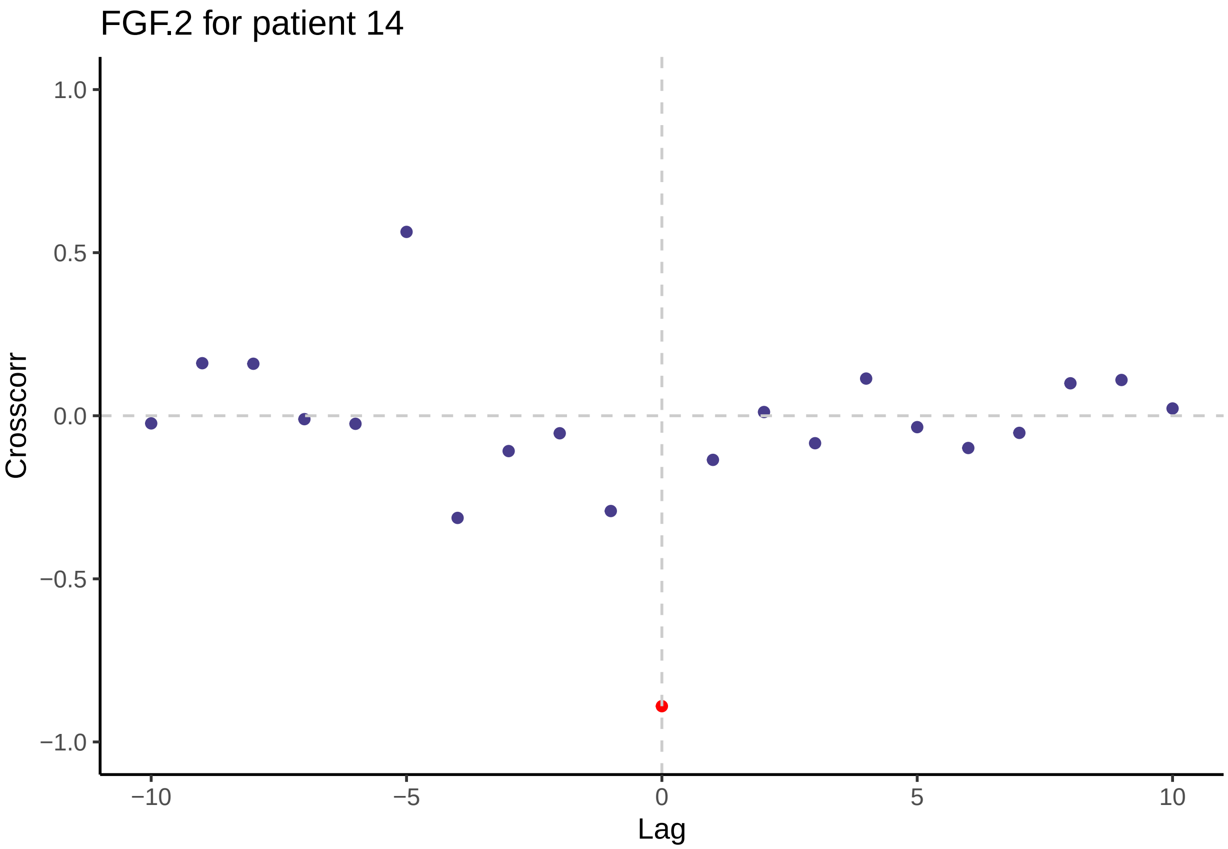


```
## [1] "FGF.2 for patient 14 - p-value: 0.475819597798307"
```

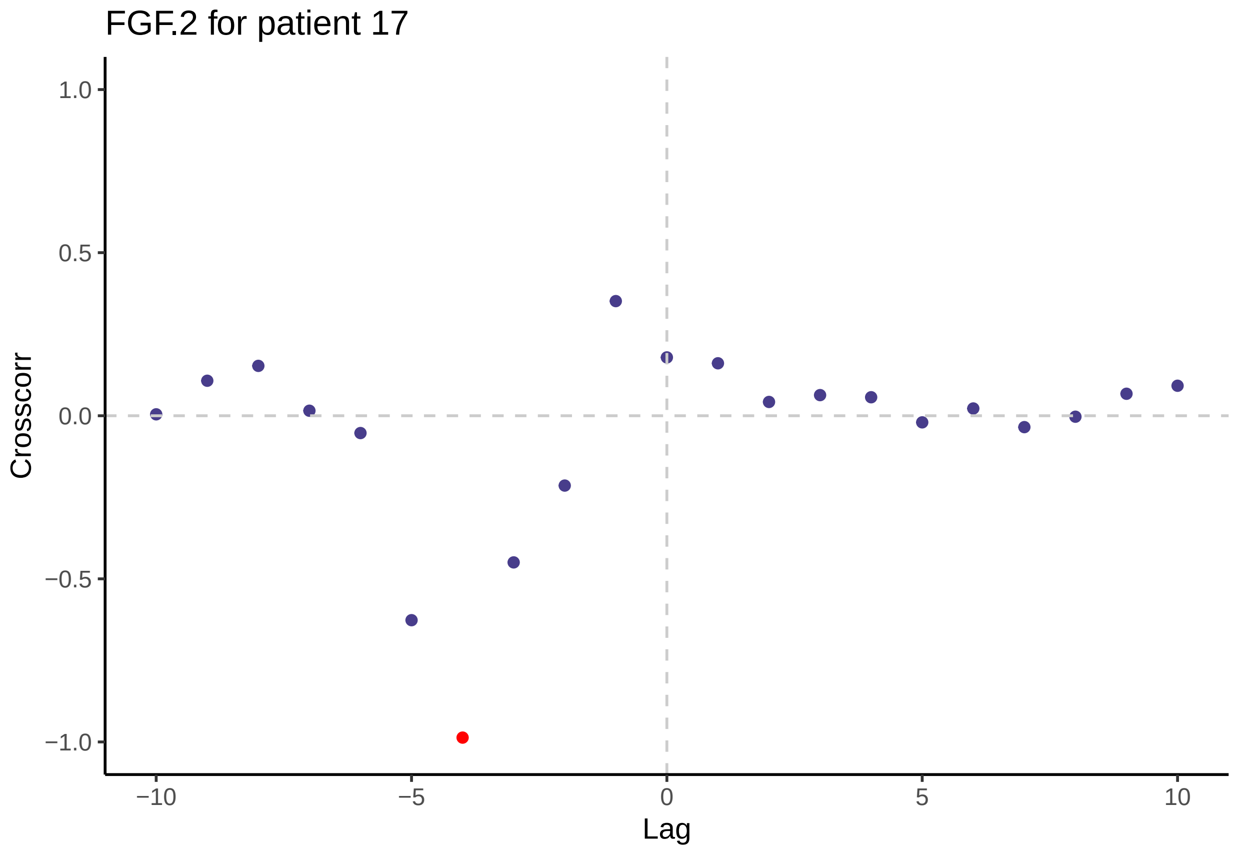


```
## [1] "FGF.2 for patient 17 - p-value: 0.444739365188214"
```

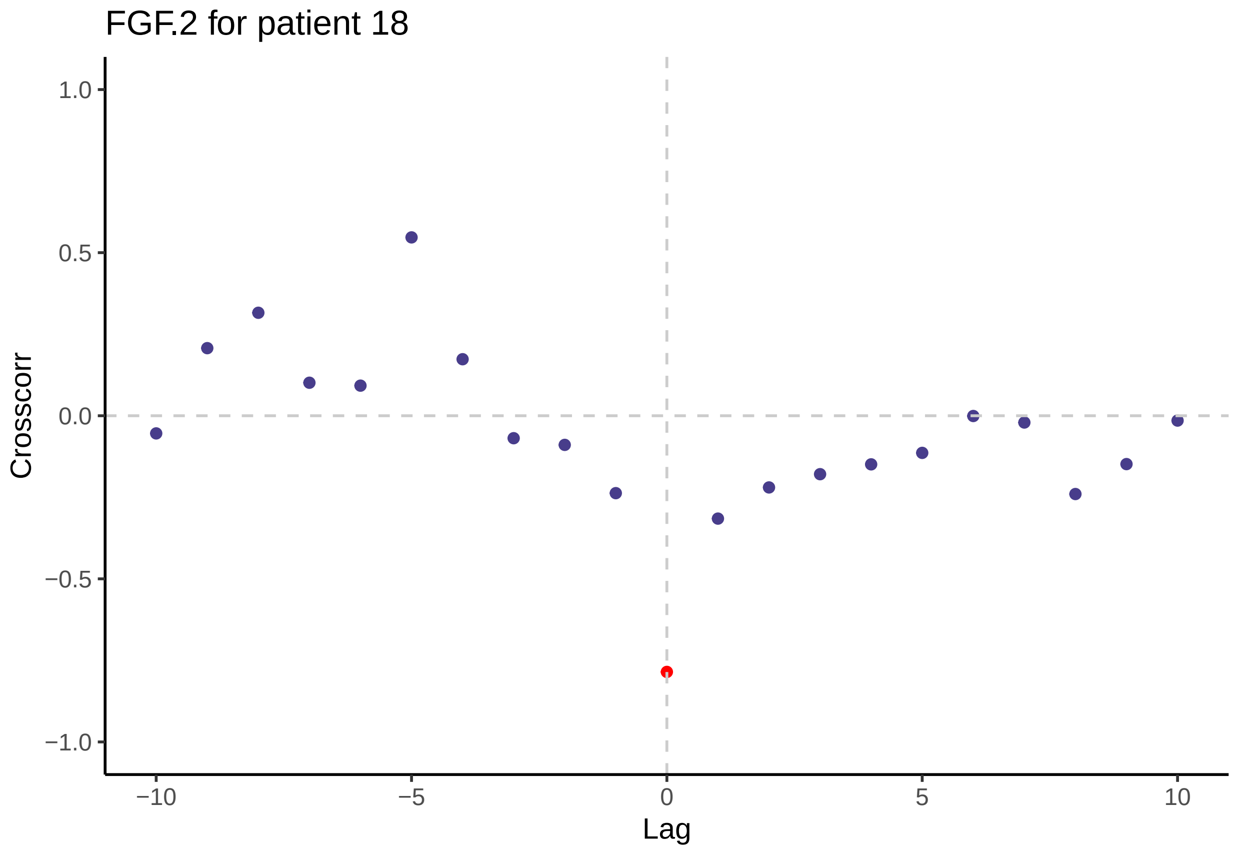


```
## [1] "FGF.2 for patient 18 - p-value: 0.335240910566316"
```

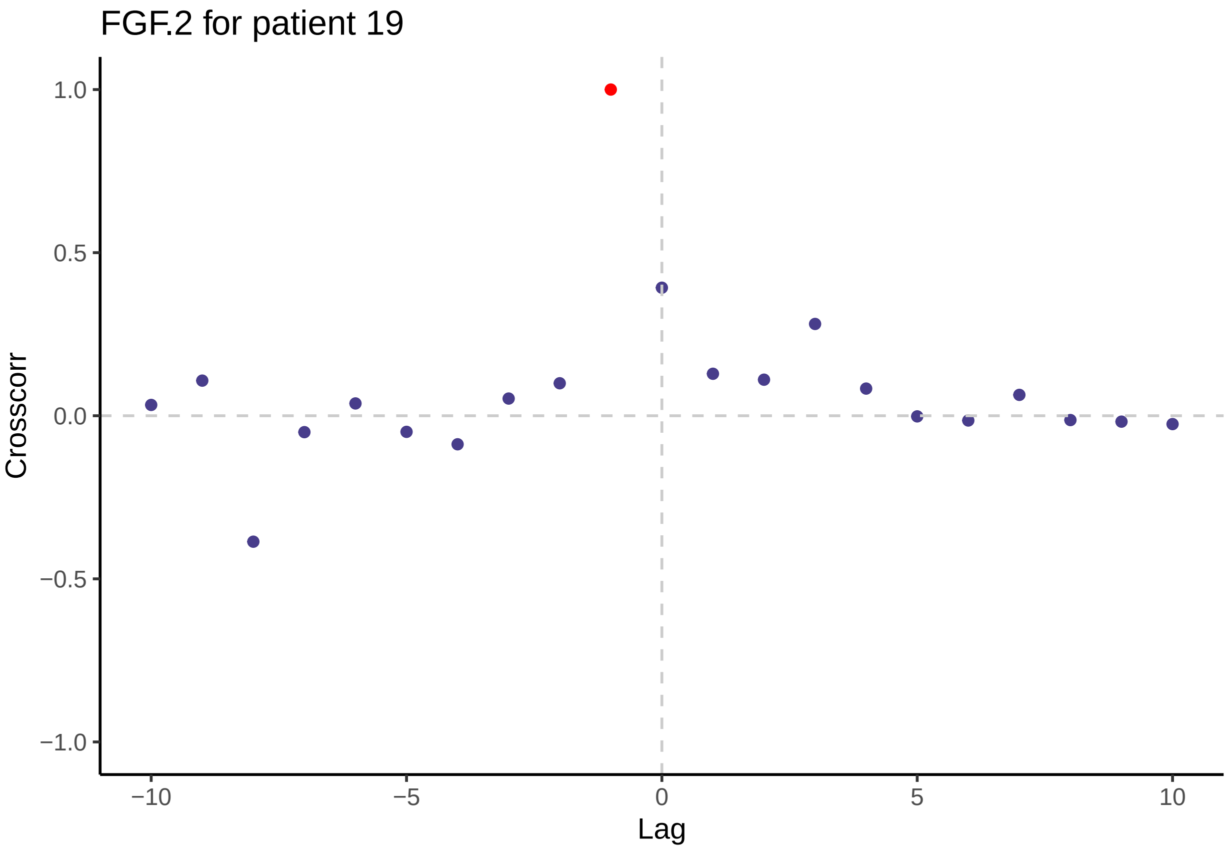


```
## [1] "FGF.2 for patient 19 - p-value: 0.153469144957594"
```

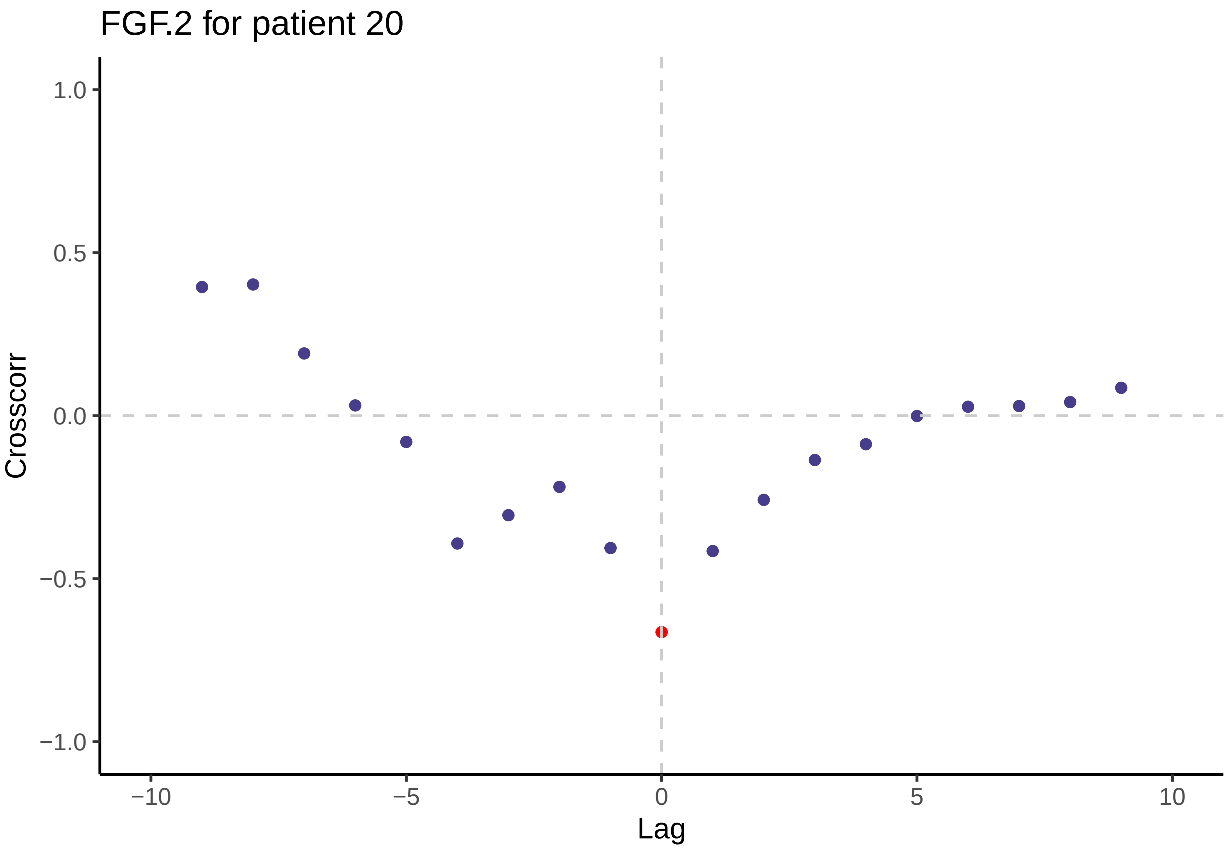


```
## [1] "FGF.2 for patient 20 - p-value: 0.162102992243067"
```

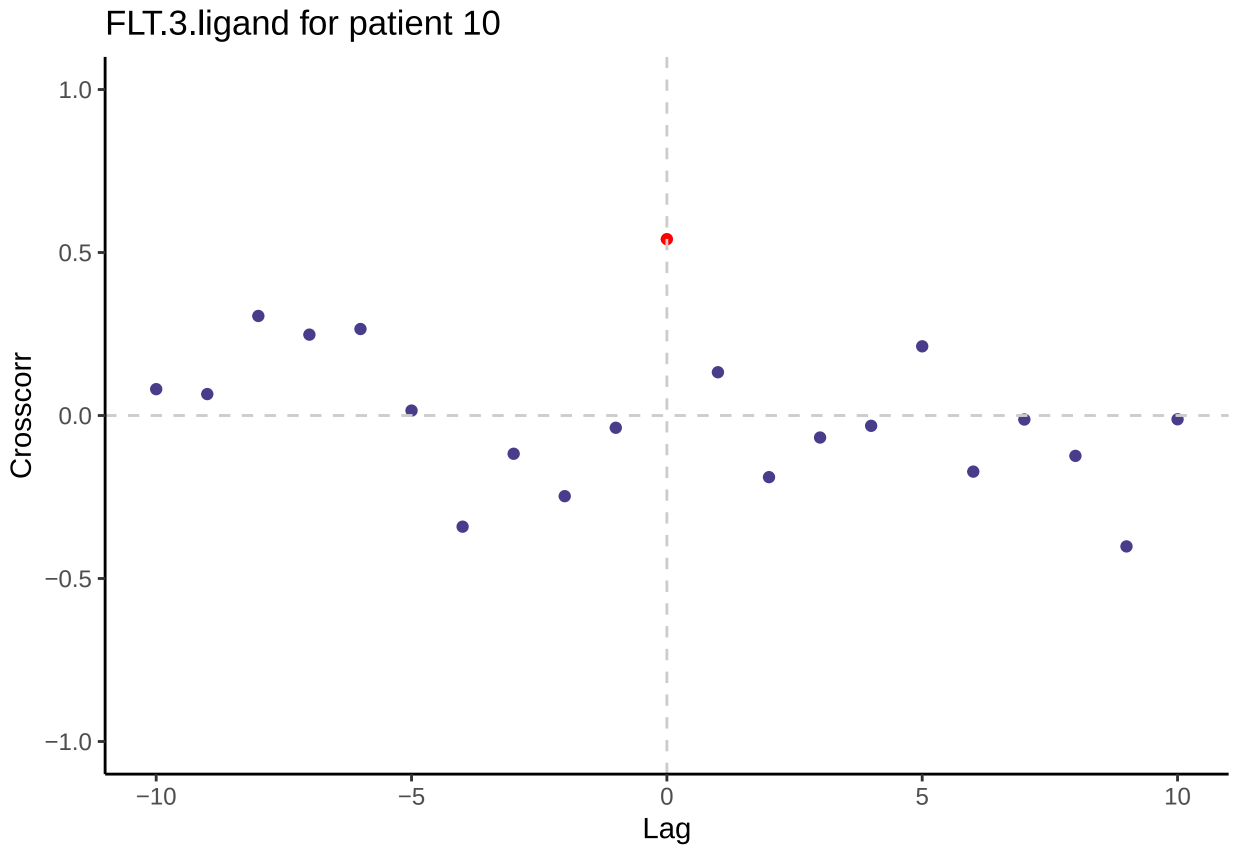


```
## [1] "FLT.3.ligand for patient 10 - p-value: 0.914338954256999"
```

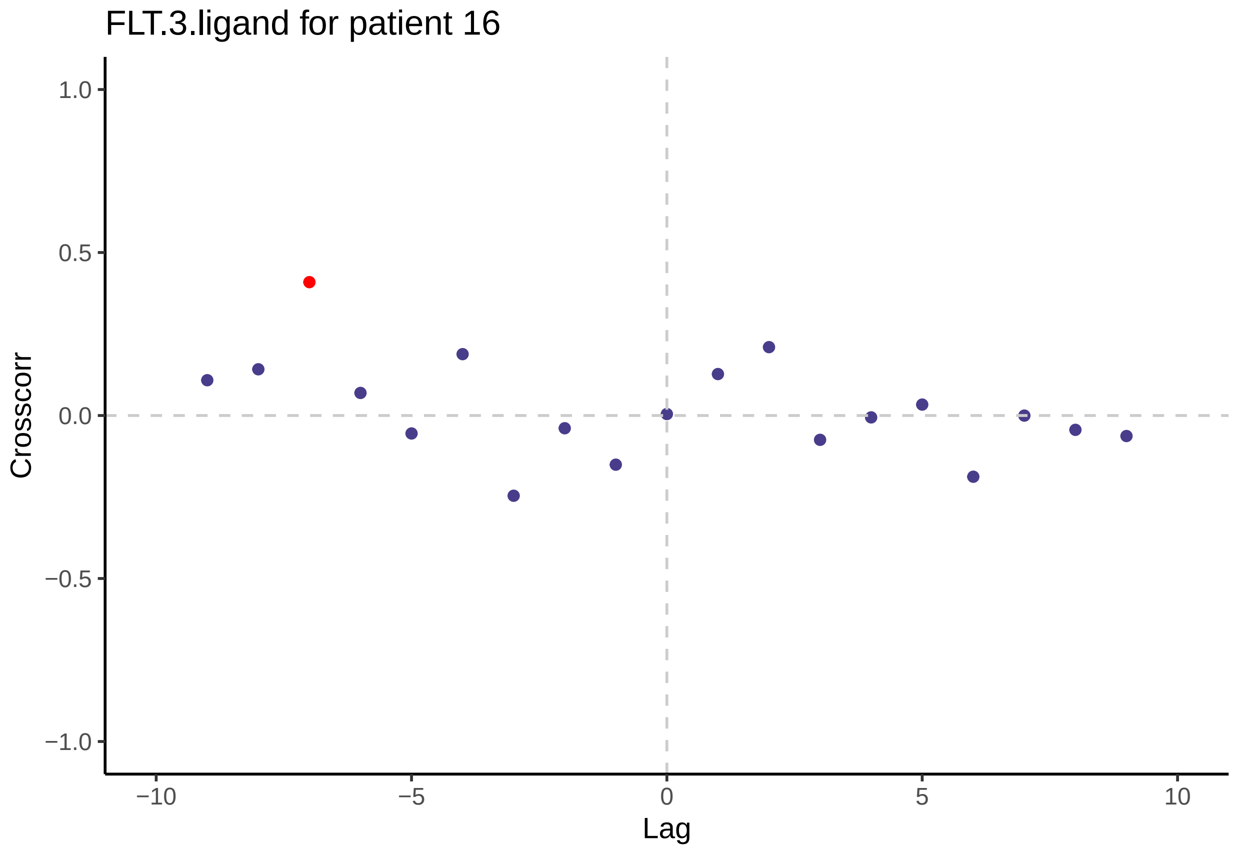


```
## [1] "FLT.3.ligand for patient 16 - p-value: 0.533770031652728"
## Warning: Removed 1 rows containing missing values (geom_point).
```

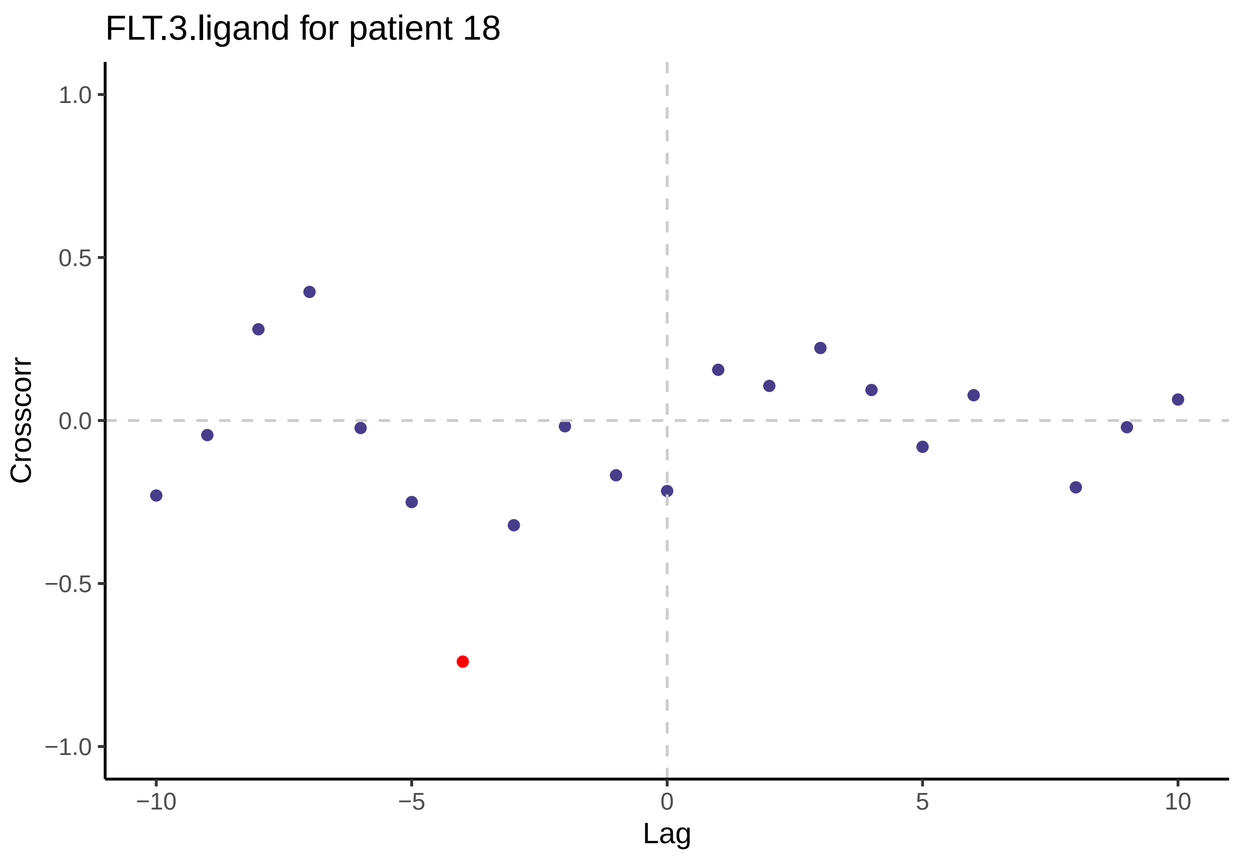


```
## [1] "FLT.3.ligand for patient 18 - p-value: 0.419299931800353"
```

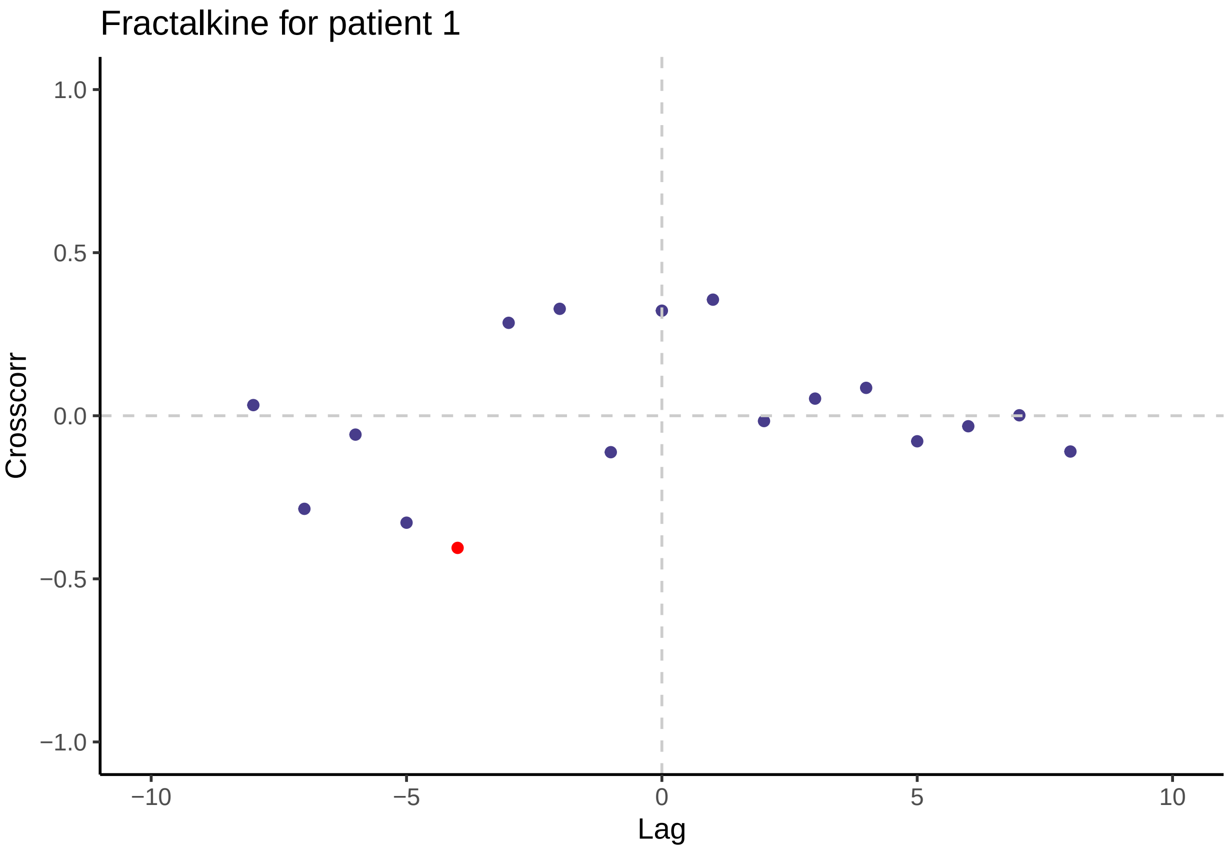


```
## [1] "Fractalkine for patient 1 - p-value: 0.967136531355666"
```

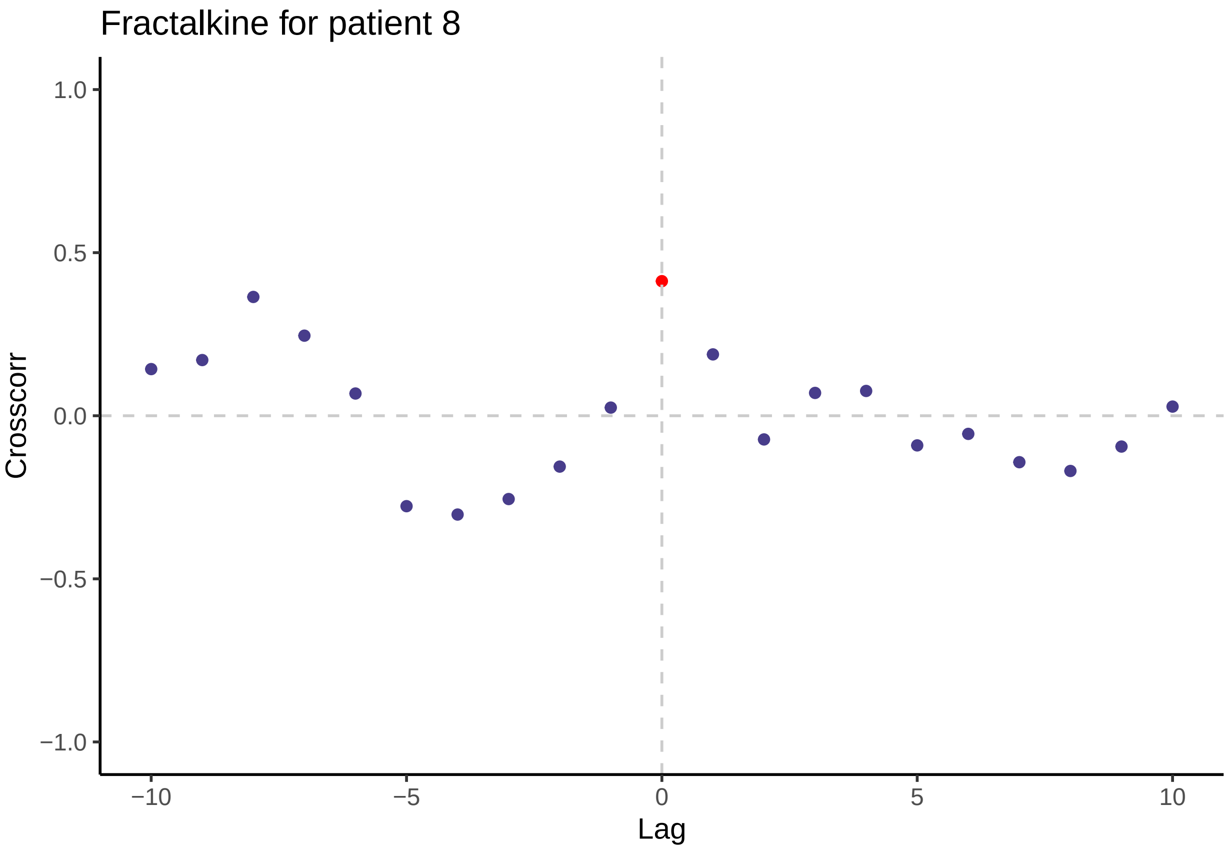


```
## [1] "Fractalkine for patient 8 - p-value: 0.849871308410551"
```

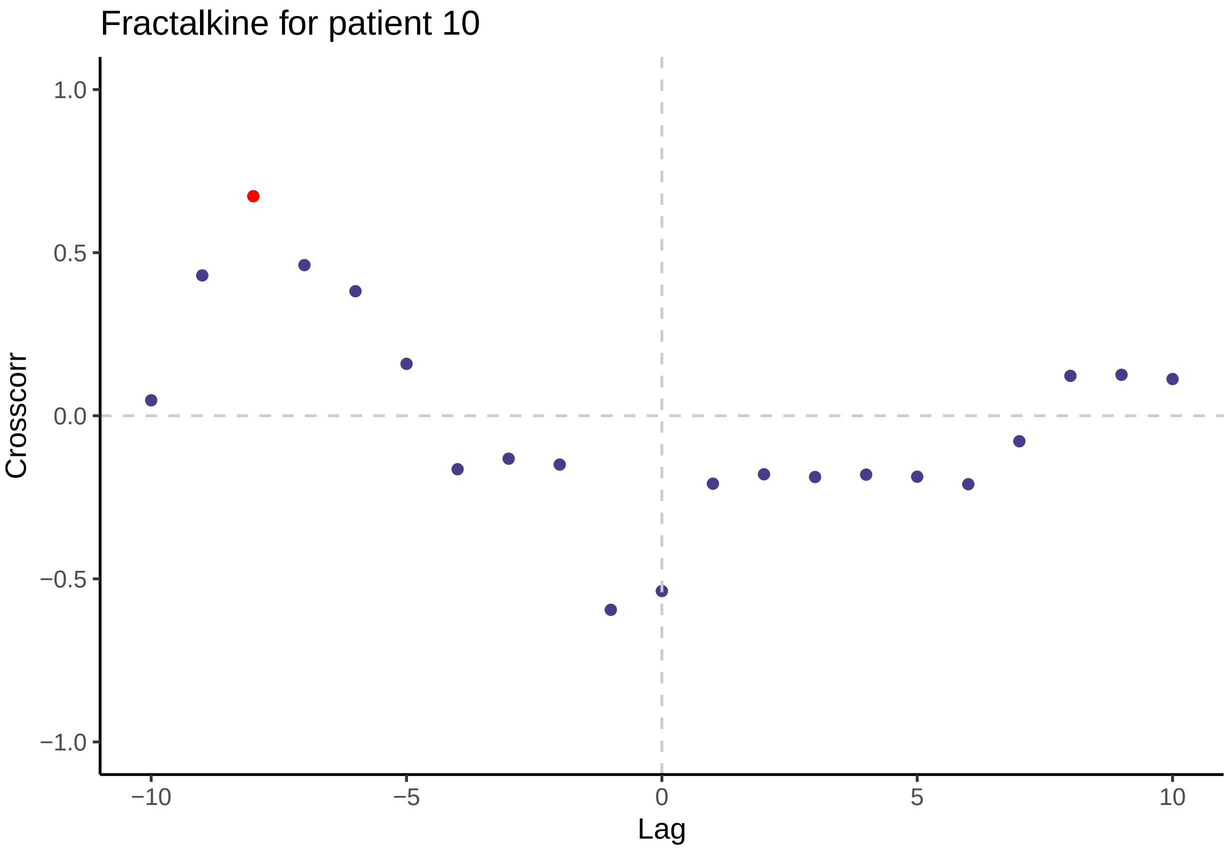


```
## [1] "Fractalkine for patient 10 - p-value: 0.841101296194634"
```

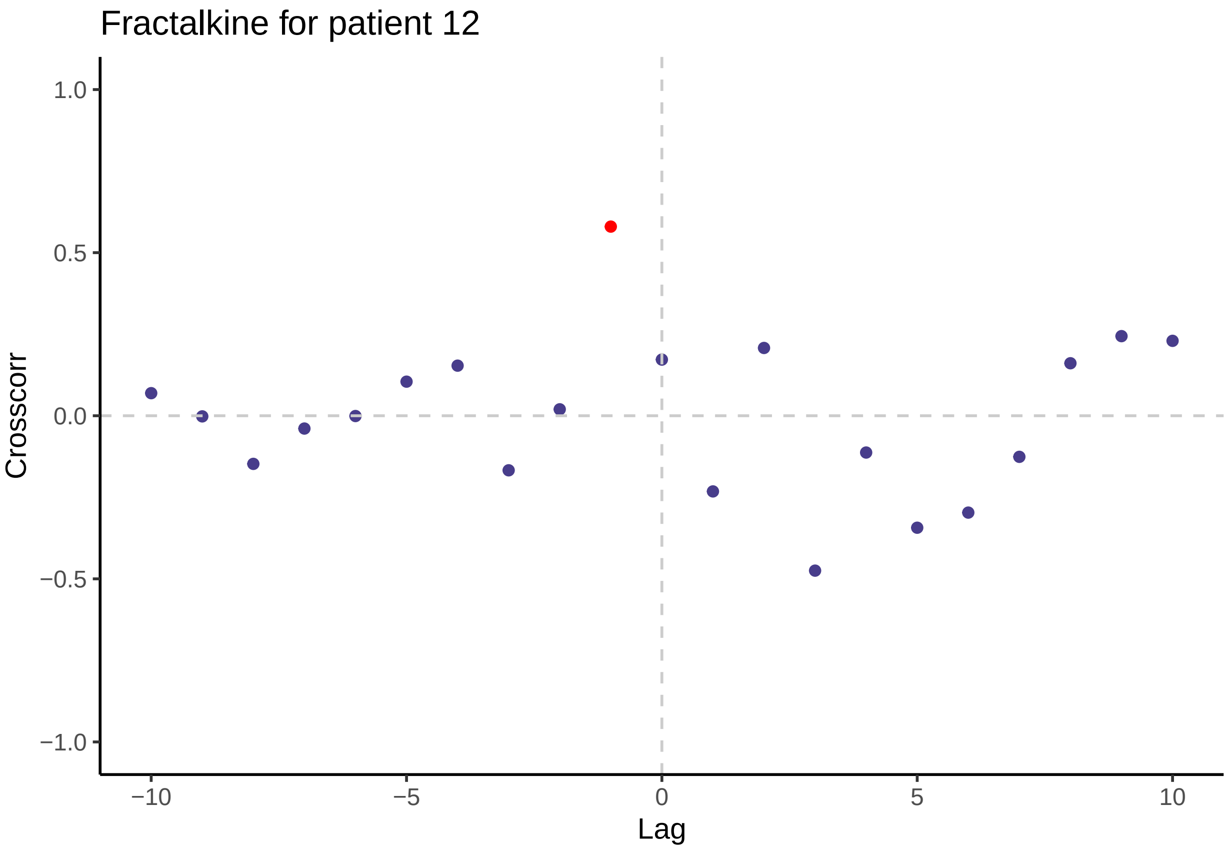


```
## [1] "Fractalkine for patient 12 - p-value: 0.999686080155155"
```

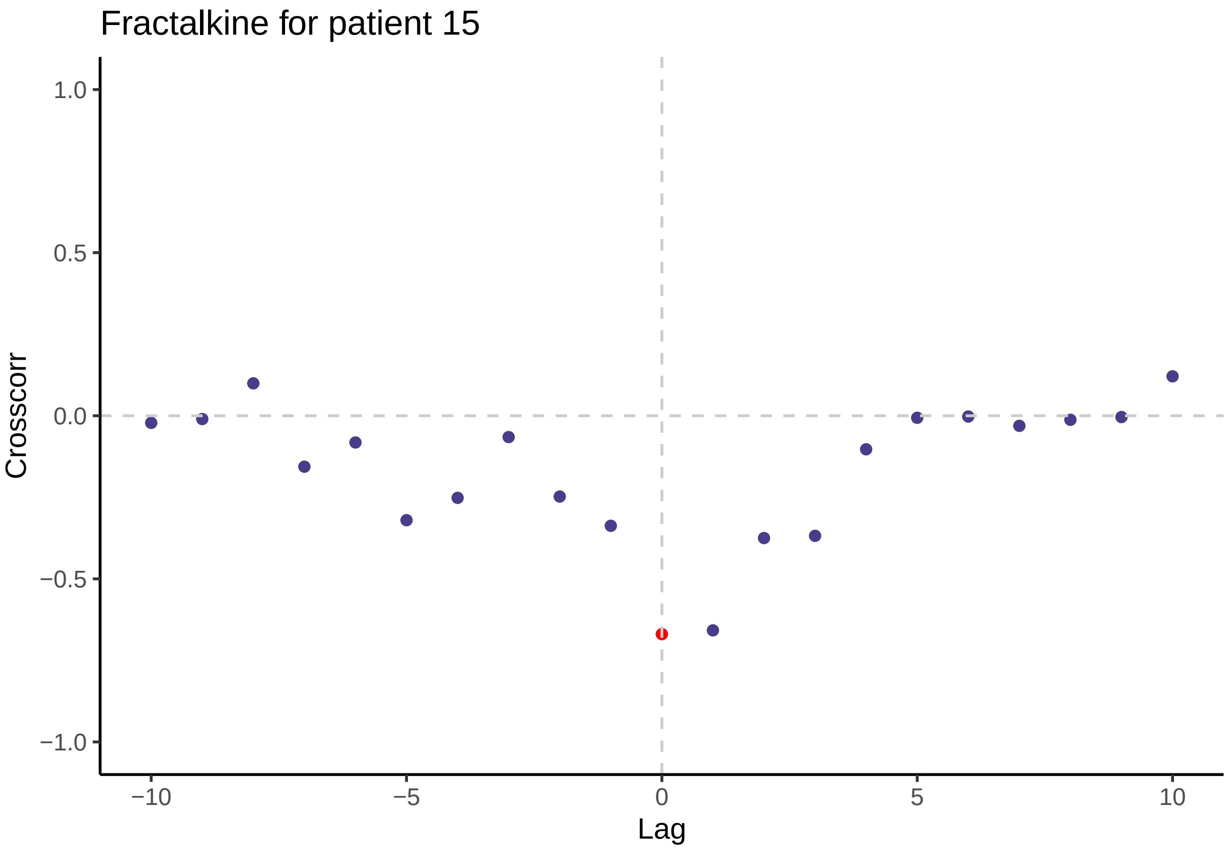


```
## [1] "Fractalkine for patient 15 - p-value: 0.00259112292383531"
```

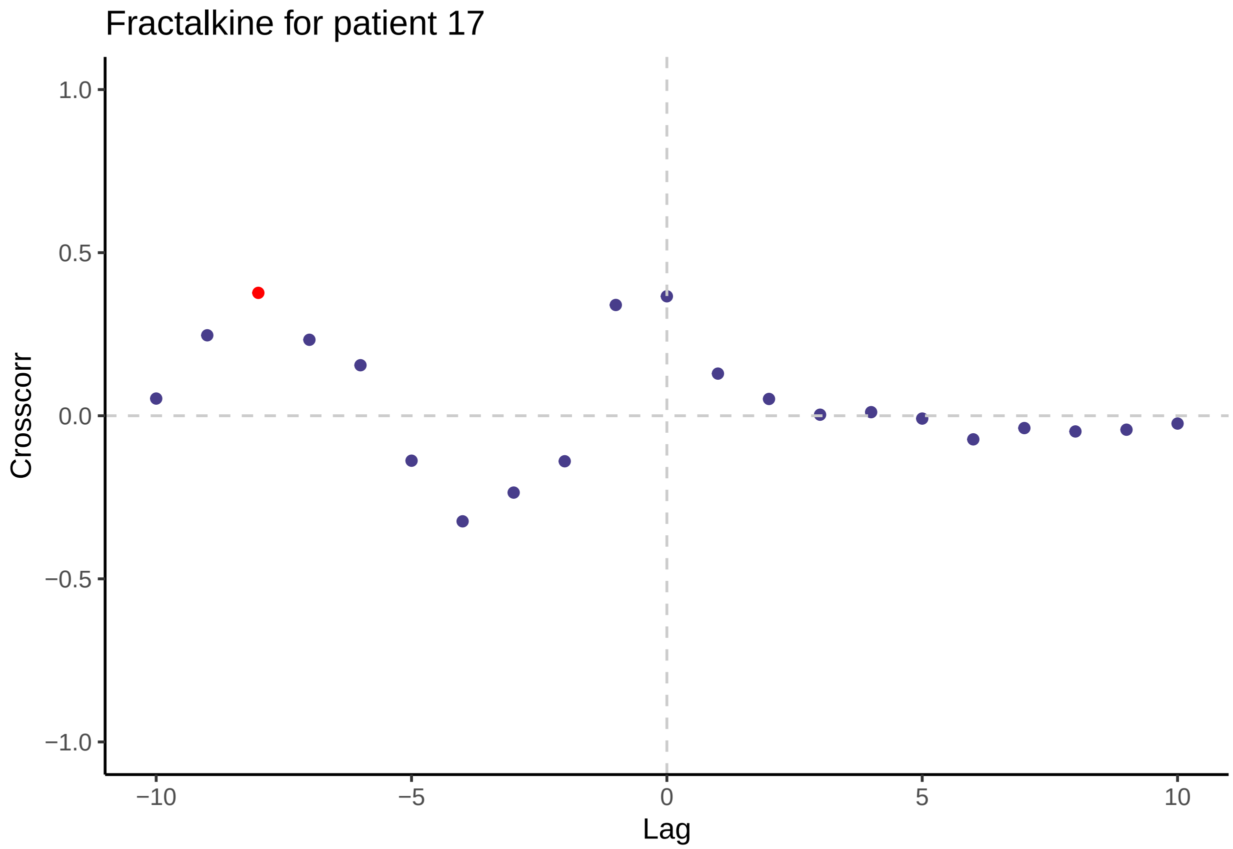


```
## [1] "Fractalkine for patient 17 - p-value: 0.317211885916919"
```

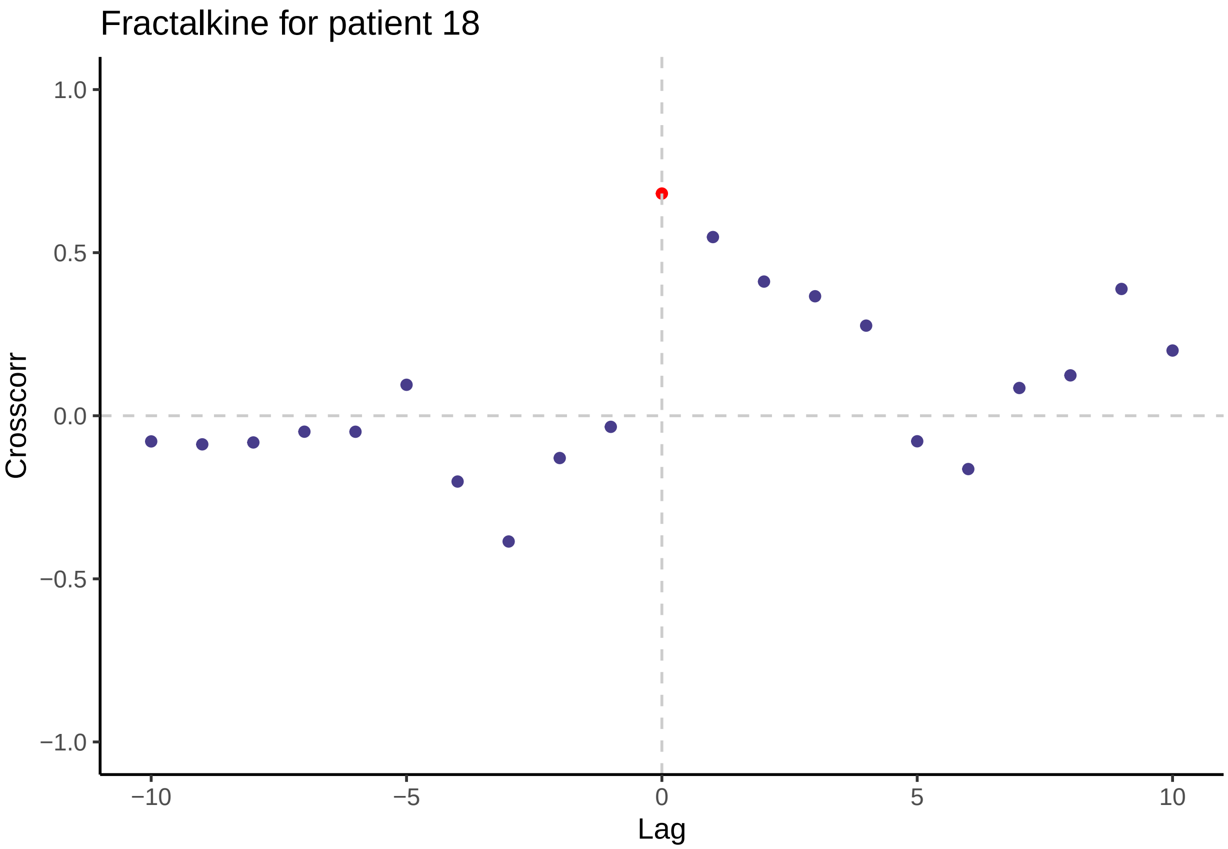


```
## [1] "Fractalkine for patient 18 - p-value: 0.154975923502484"
```

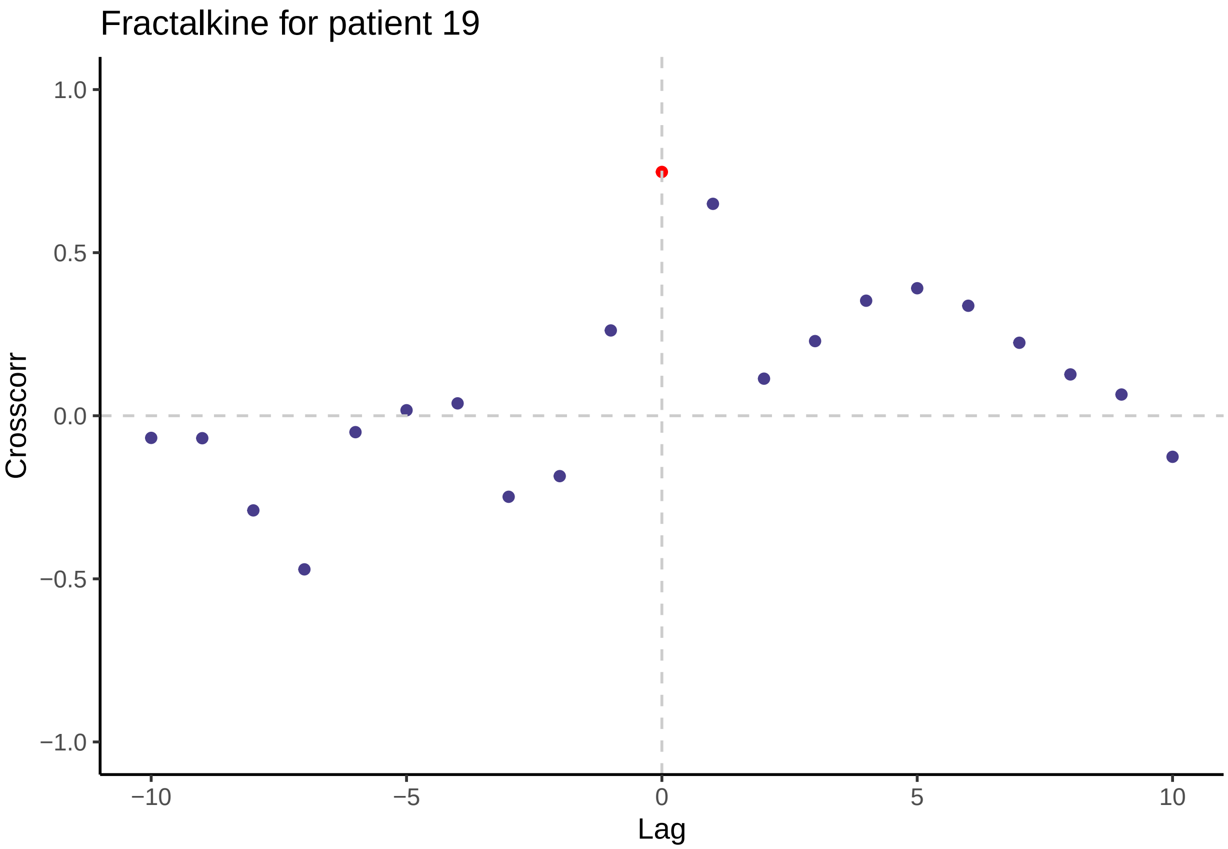


```
## [1] "Fractalkine for patient 19 - p-value: 0.153258024076272"
```

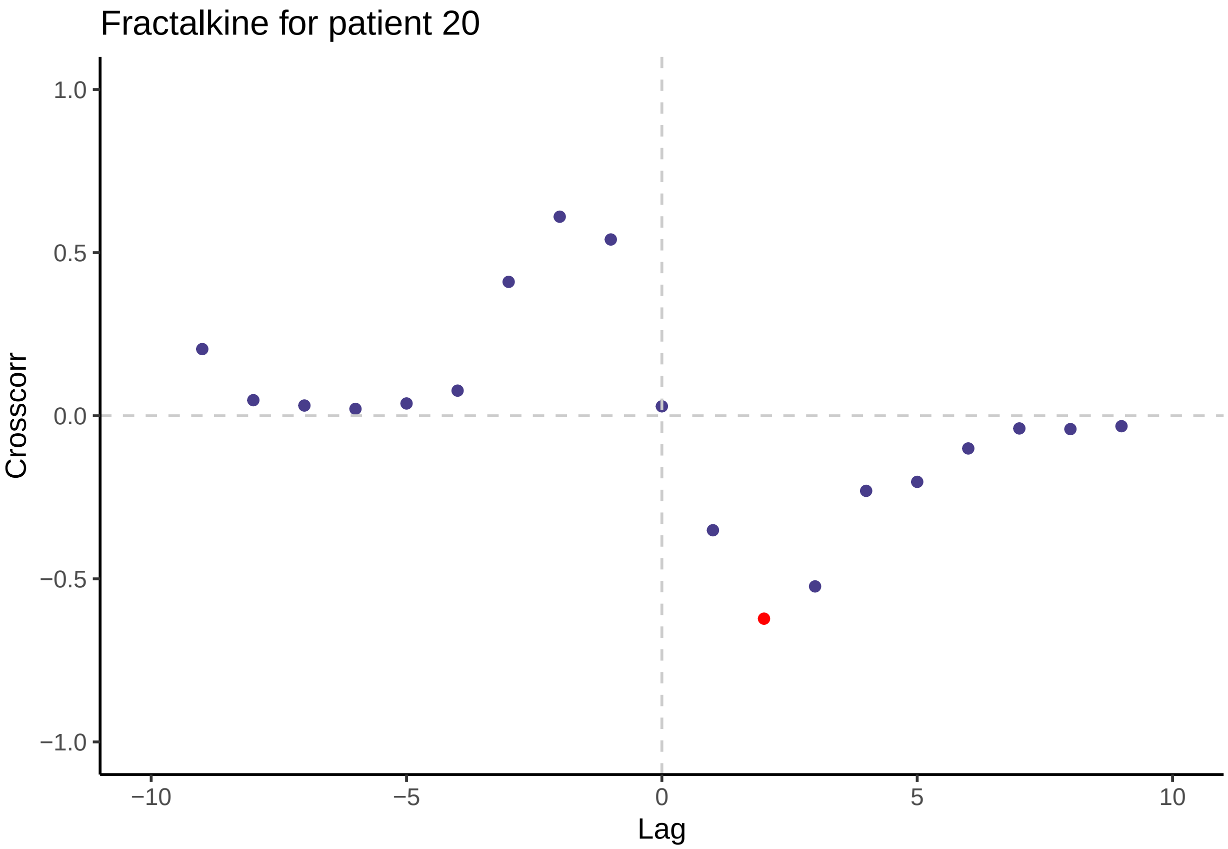


```
## [1] "Fractalkine for patient 20 - p-value: 0.924423538394642"
```

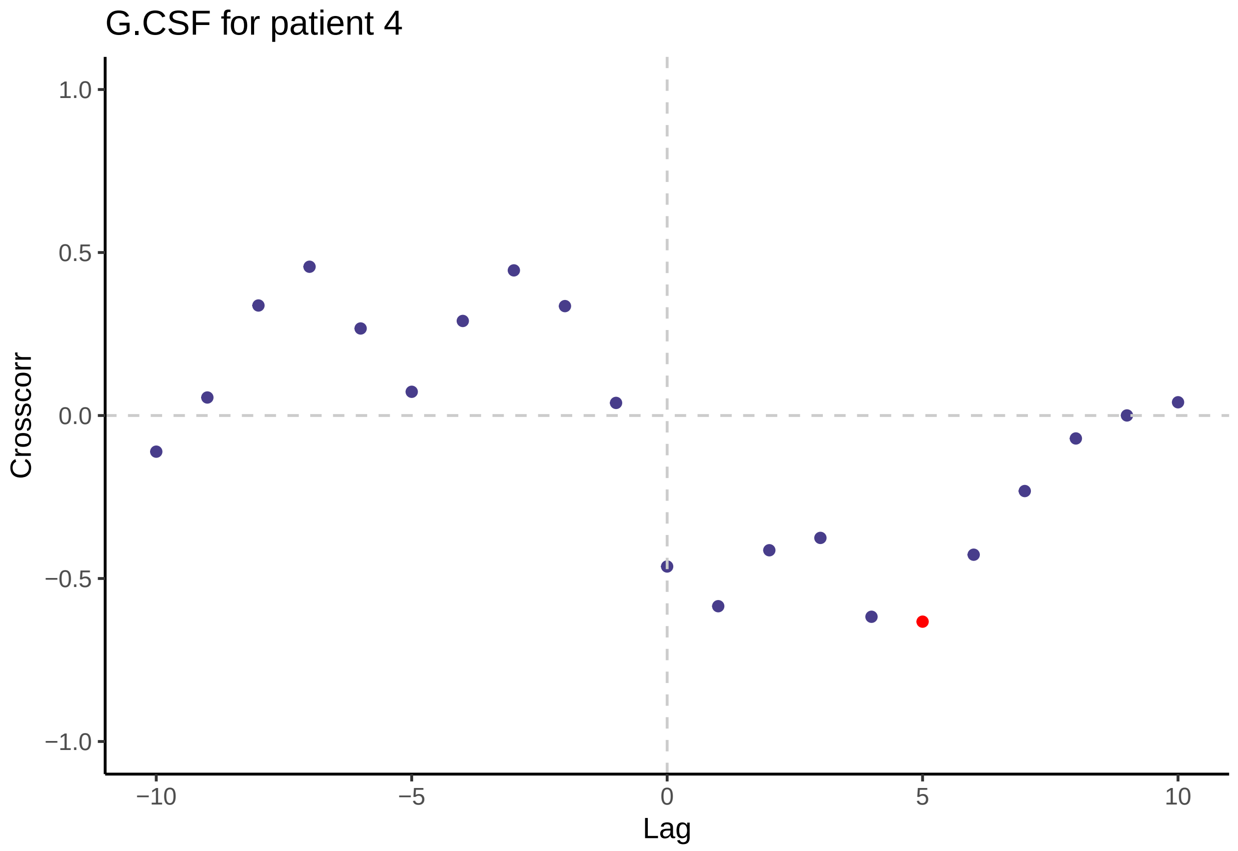


```
## [1] "G.CSF for patient 4 - p-value: 0.346675311348956"
```

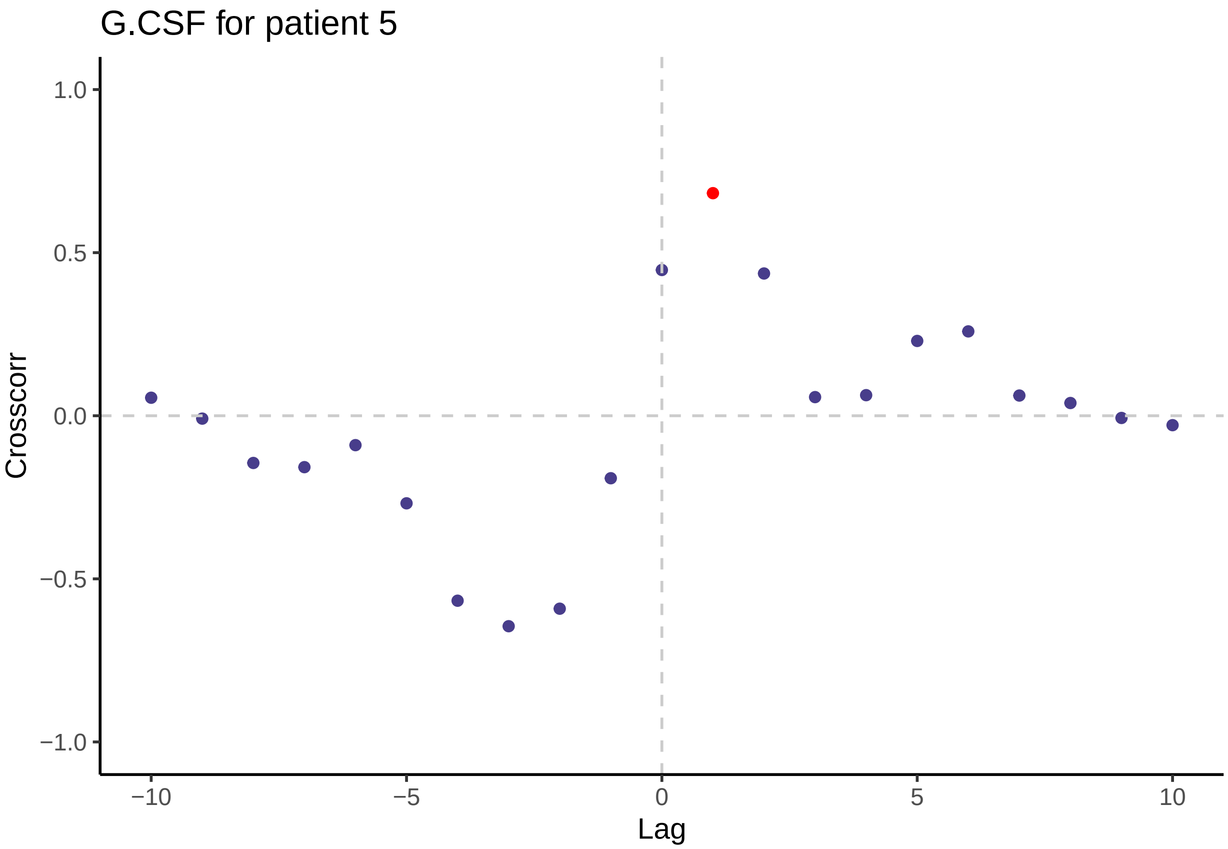


```
## [1] "G.CSF for patient 5 - p-value: 0.811379391250711"
```

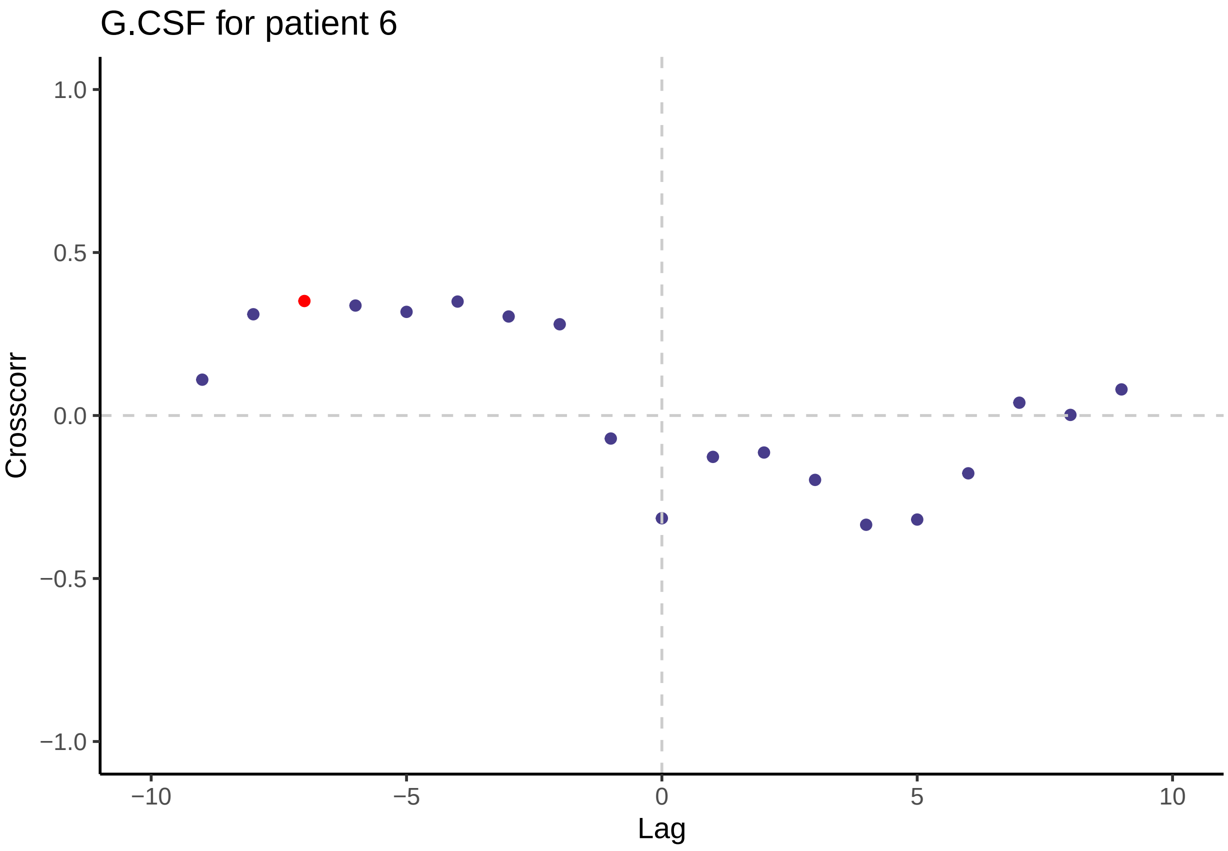


```
## [1] "G.CSF for patient 6 - p-value: 0.459132887534464"

## Warning: Removed 5 rows containing missing values (geom_point).
```

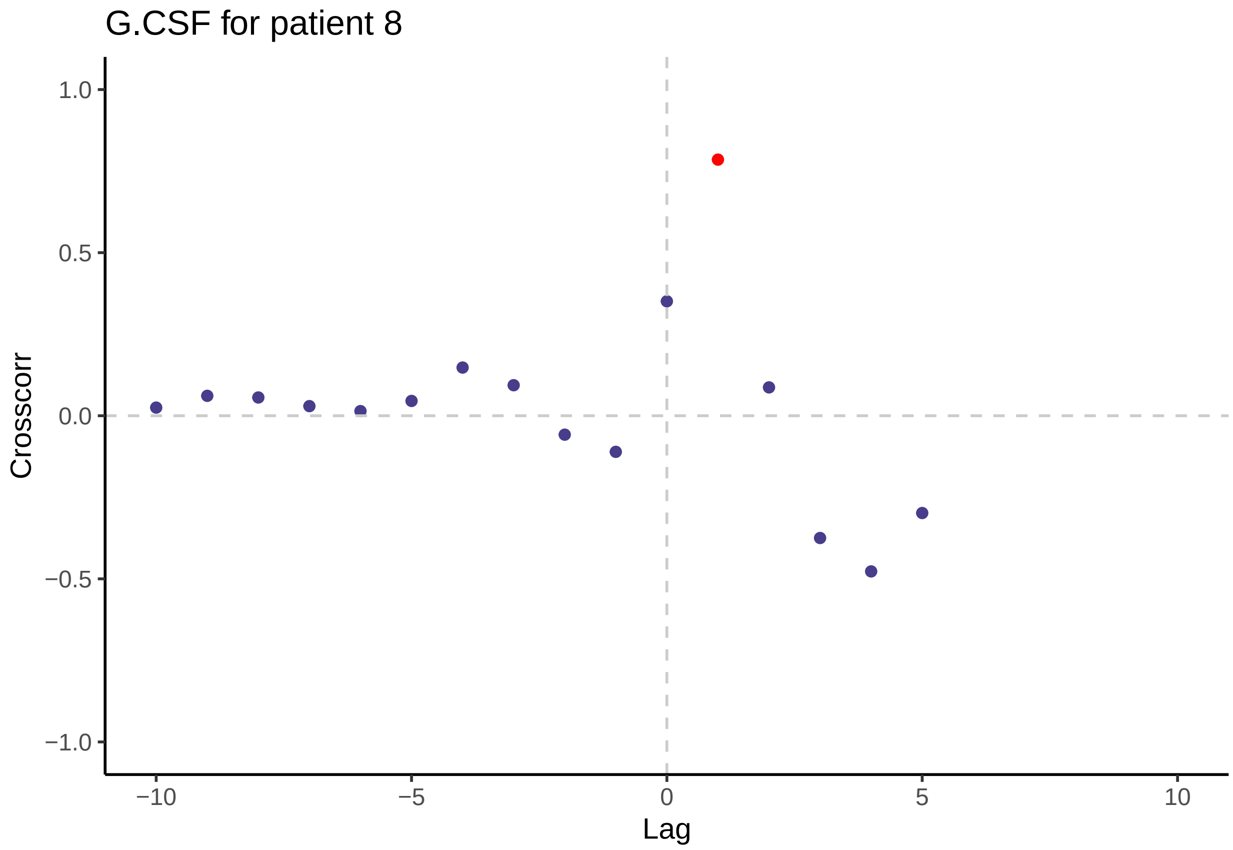


```
## [1] "G.CSF for patient 8 - p-value: 0.748634686759974"
```

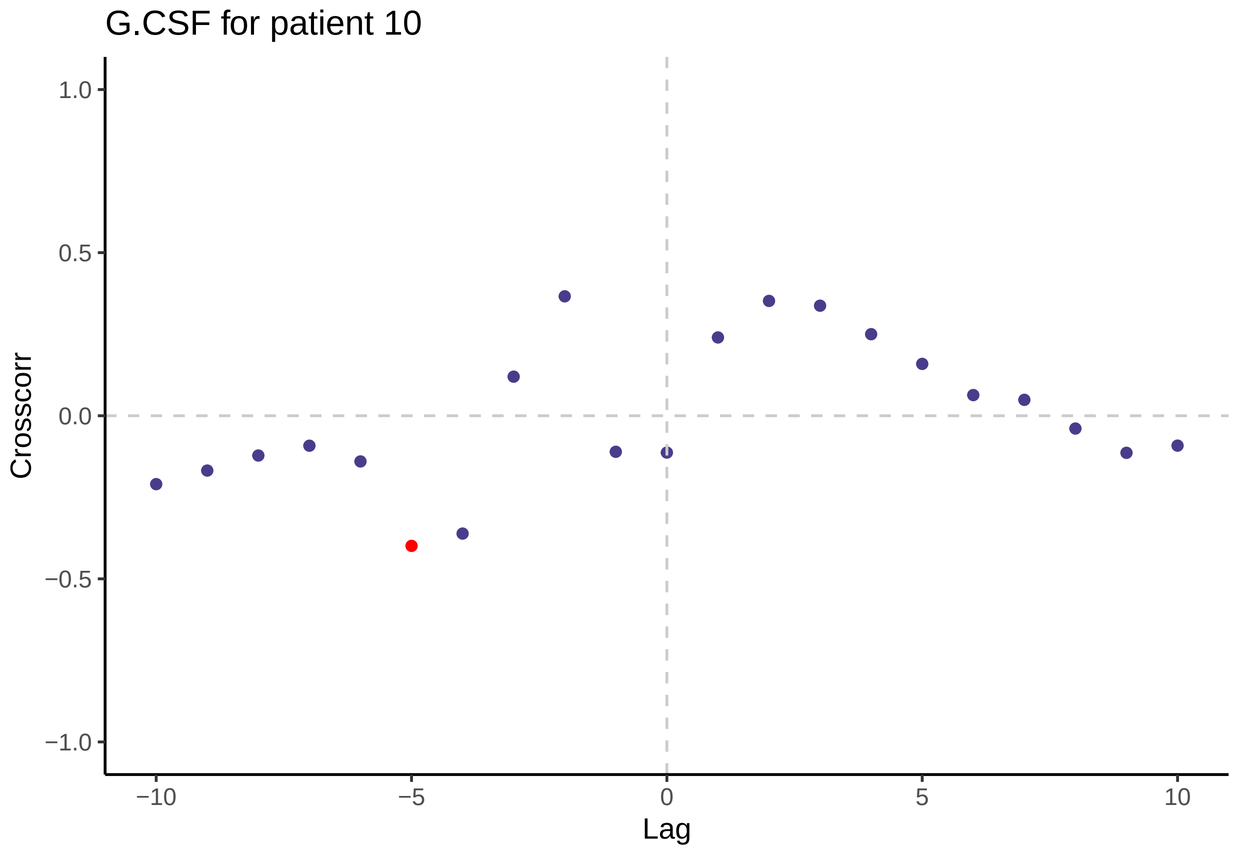


```
## [1] "G.CSF for patient 10 - p-value: 0.982420464298154"
```

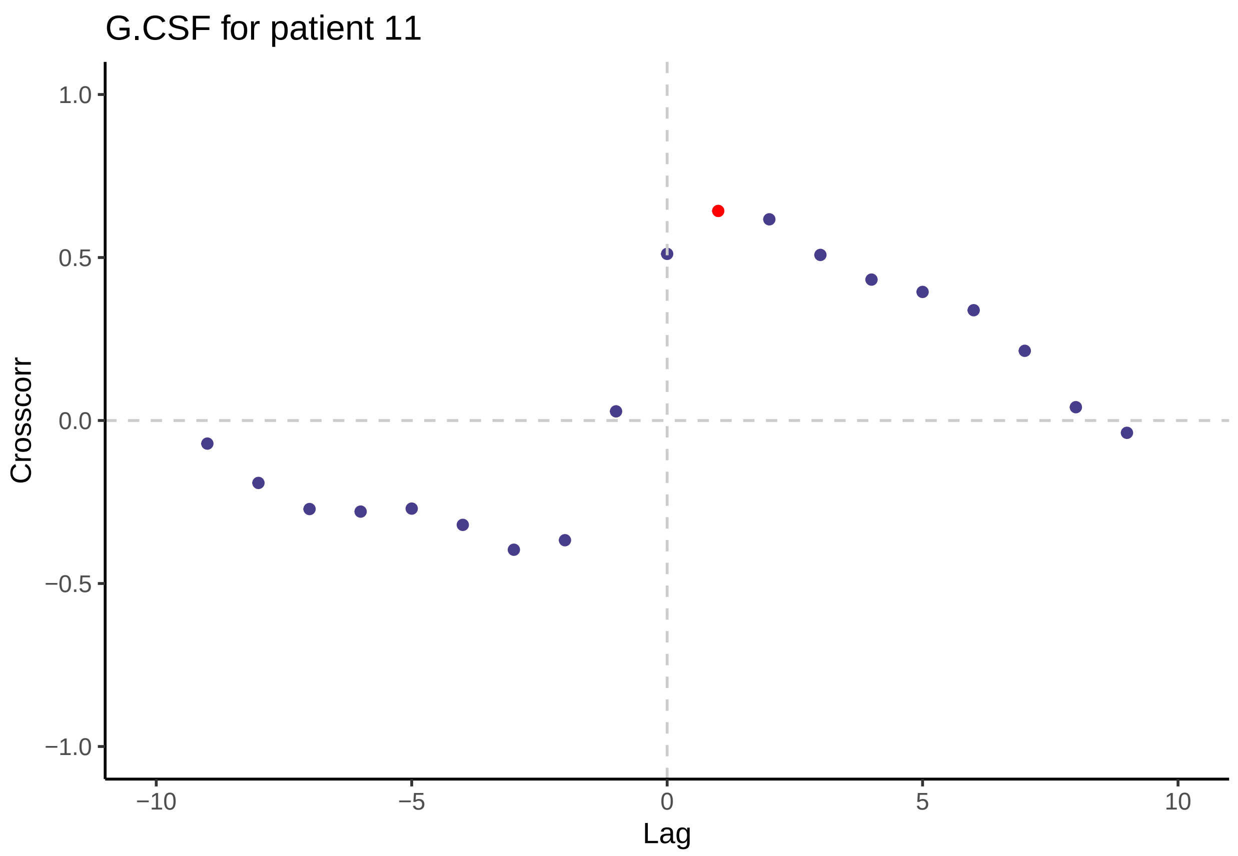


```
## [1] "G.CSF for patient 11 - p-value: 0.34756013303751"
```

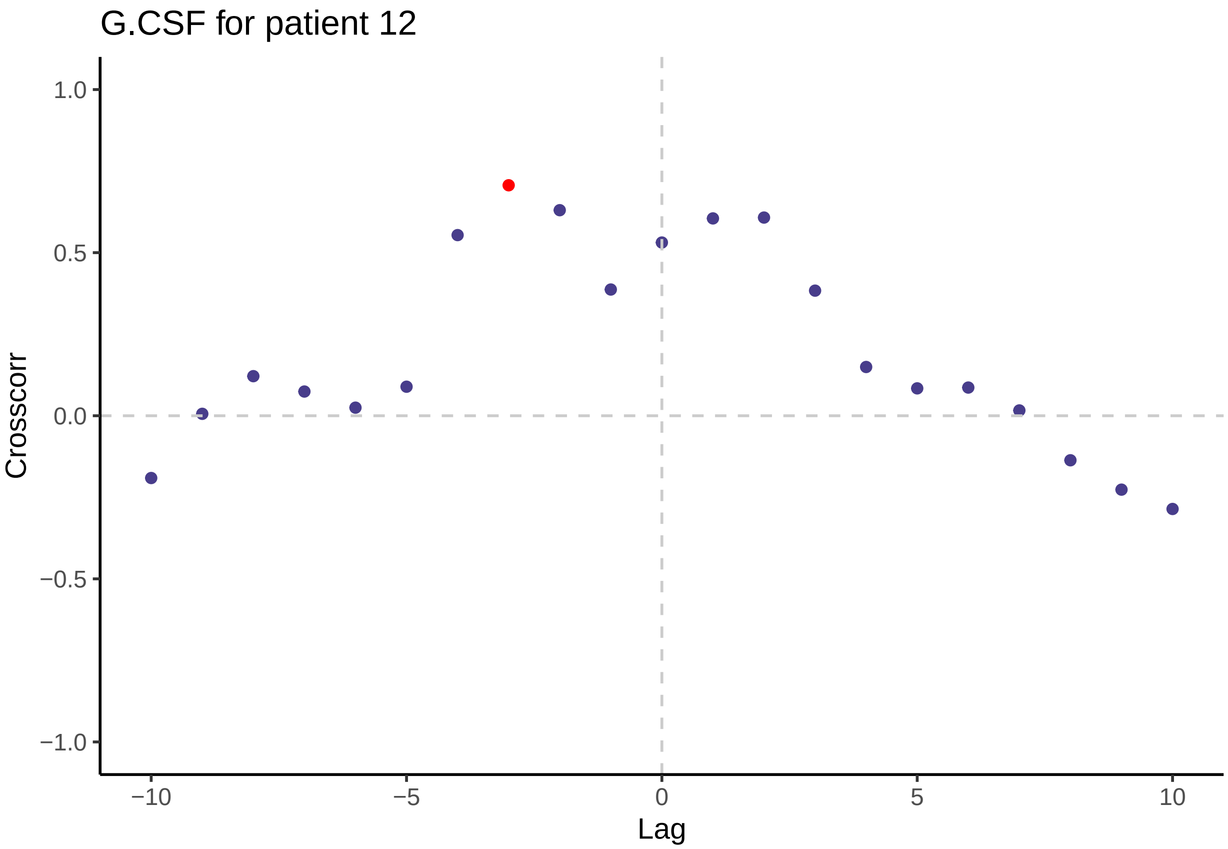


```
## [1] "G.CSF for patient 12 - p-value: 0.0076290209328954"
```

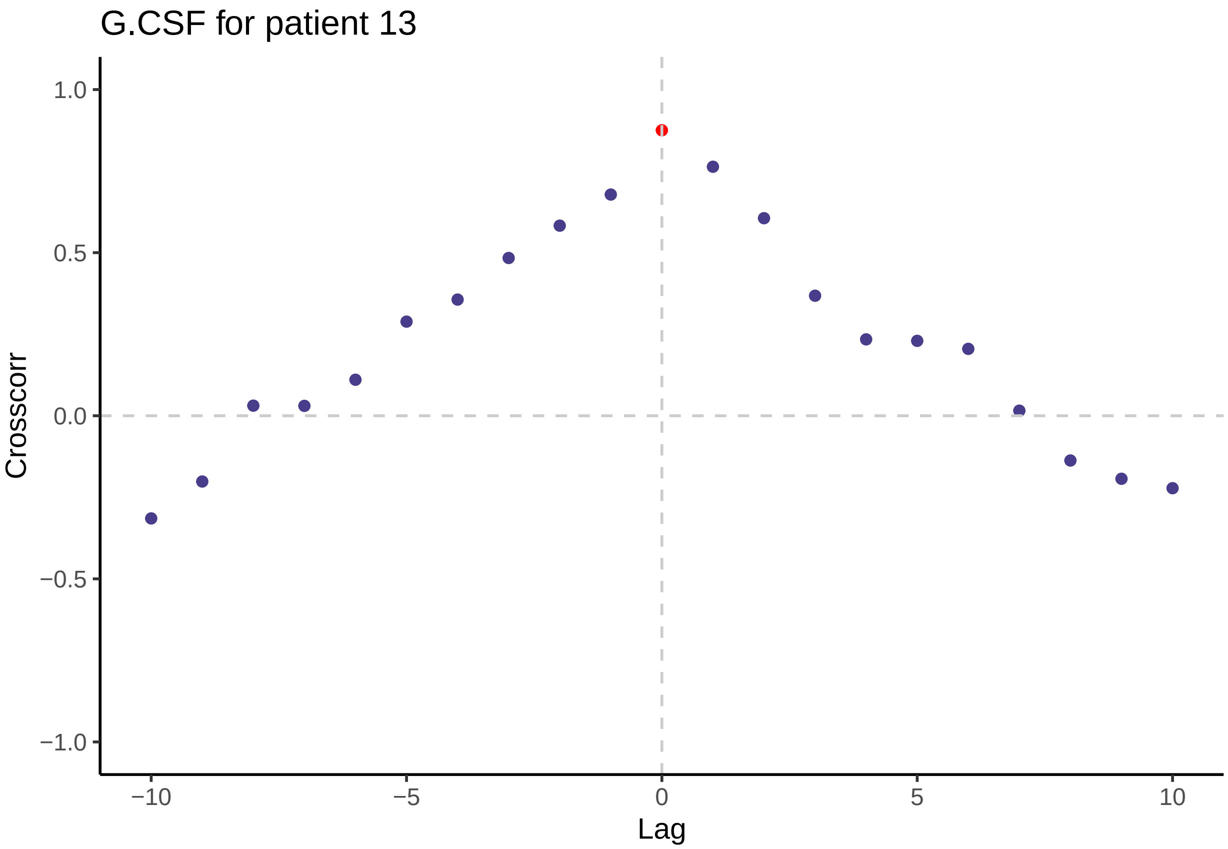


```
## [1] "G.CSF for patient 13 - p-value: 0.00692661993829351"
```

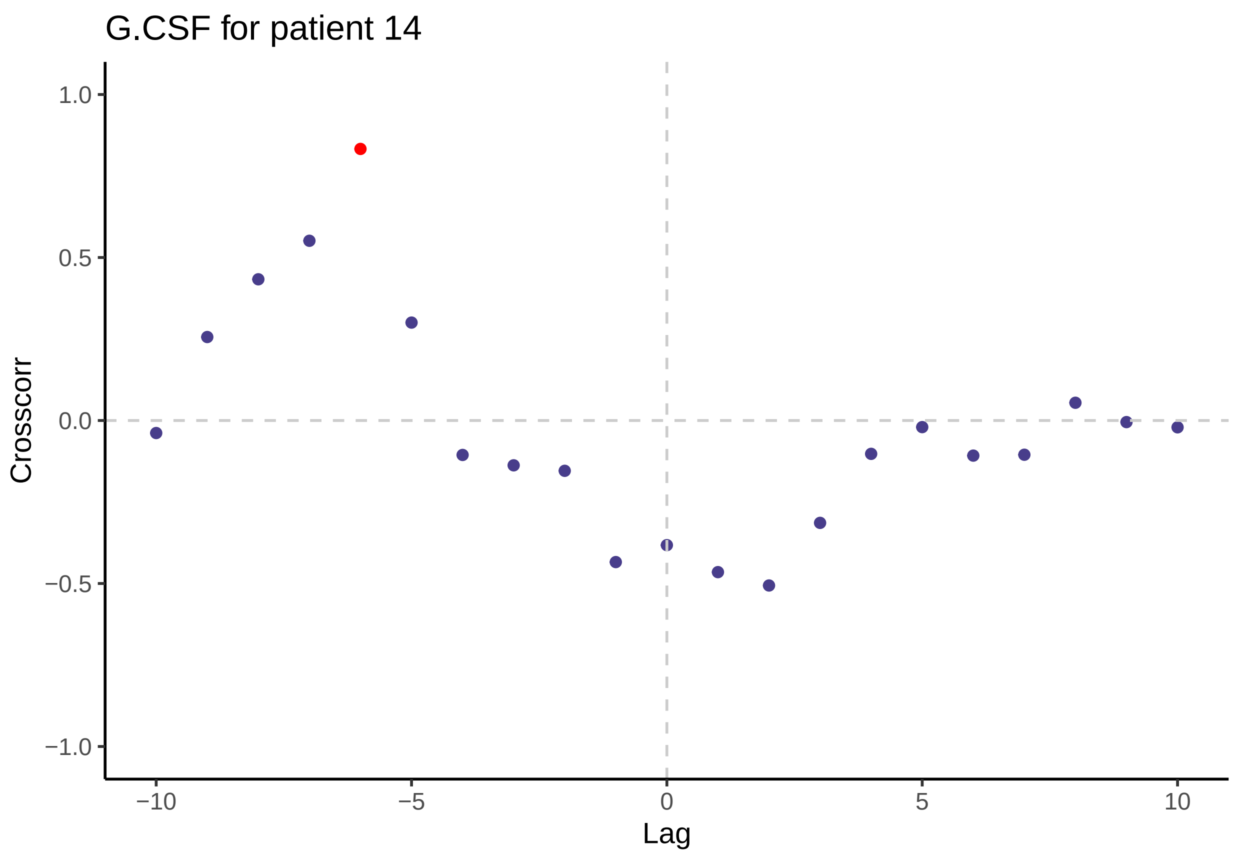


```
## [1] "G.CSF for patient 14 - p-value: 0.766870776789906"
```

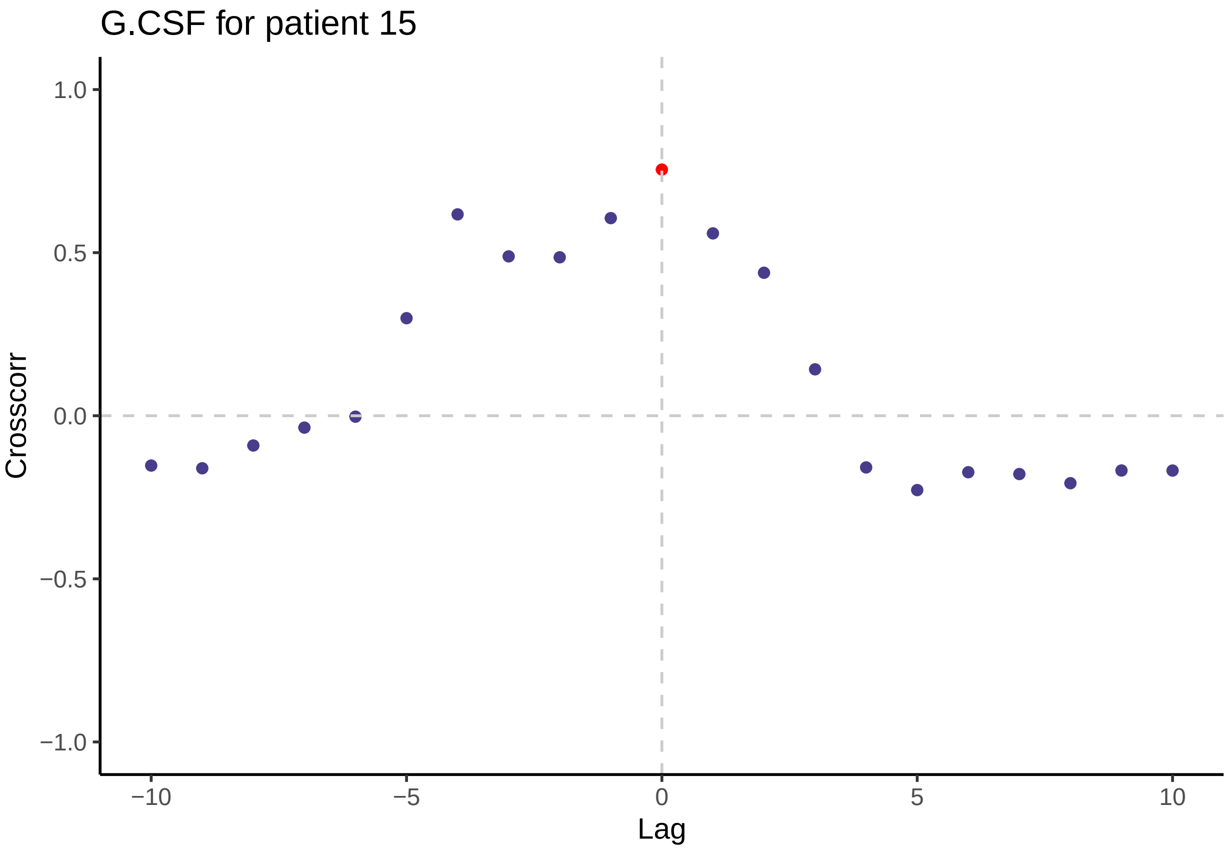


```
## [1] "G.CSF for patient 15 - p-value: 0.106134540657625"
```

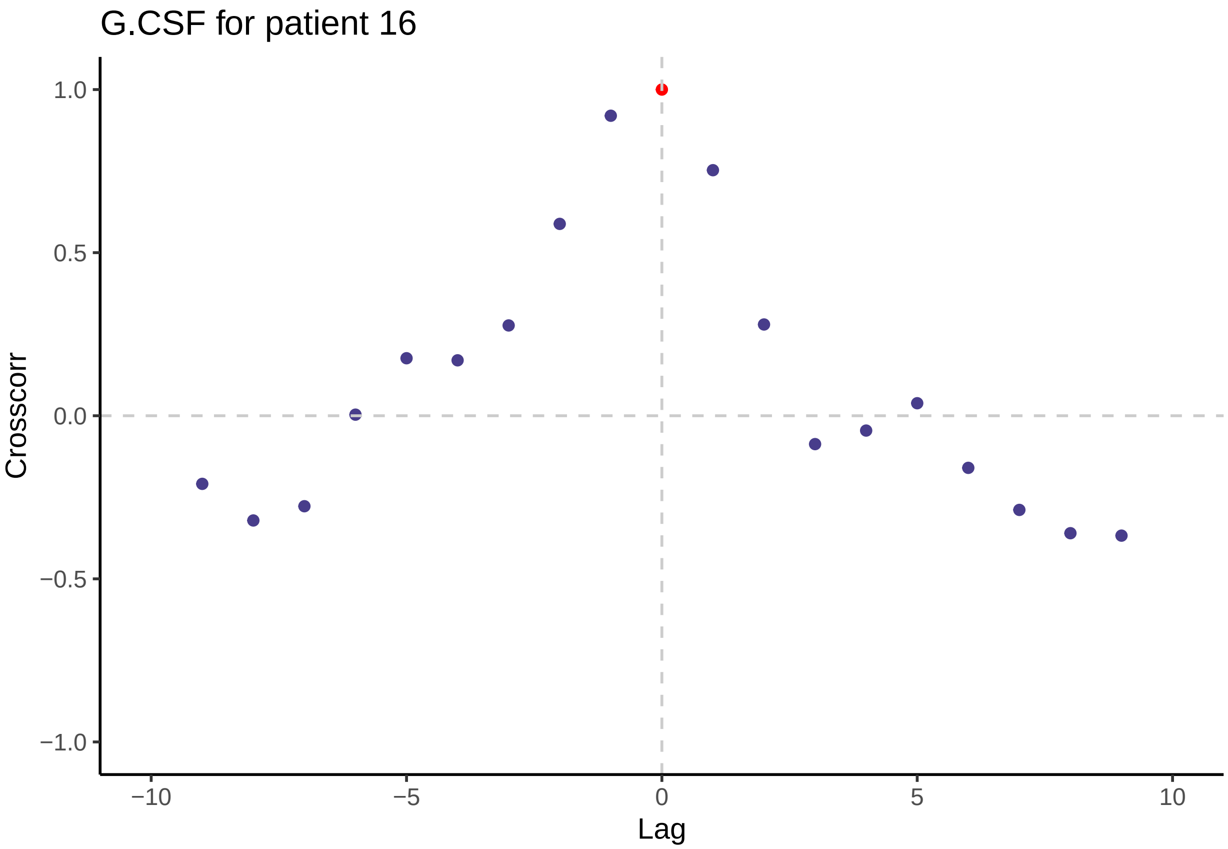


```
## [1] "G.CSF for patient 16 - p-value: 0.281120298876858"
```

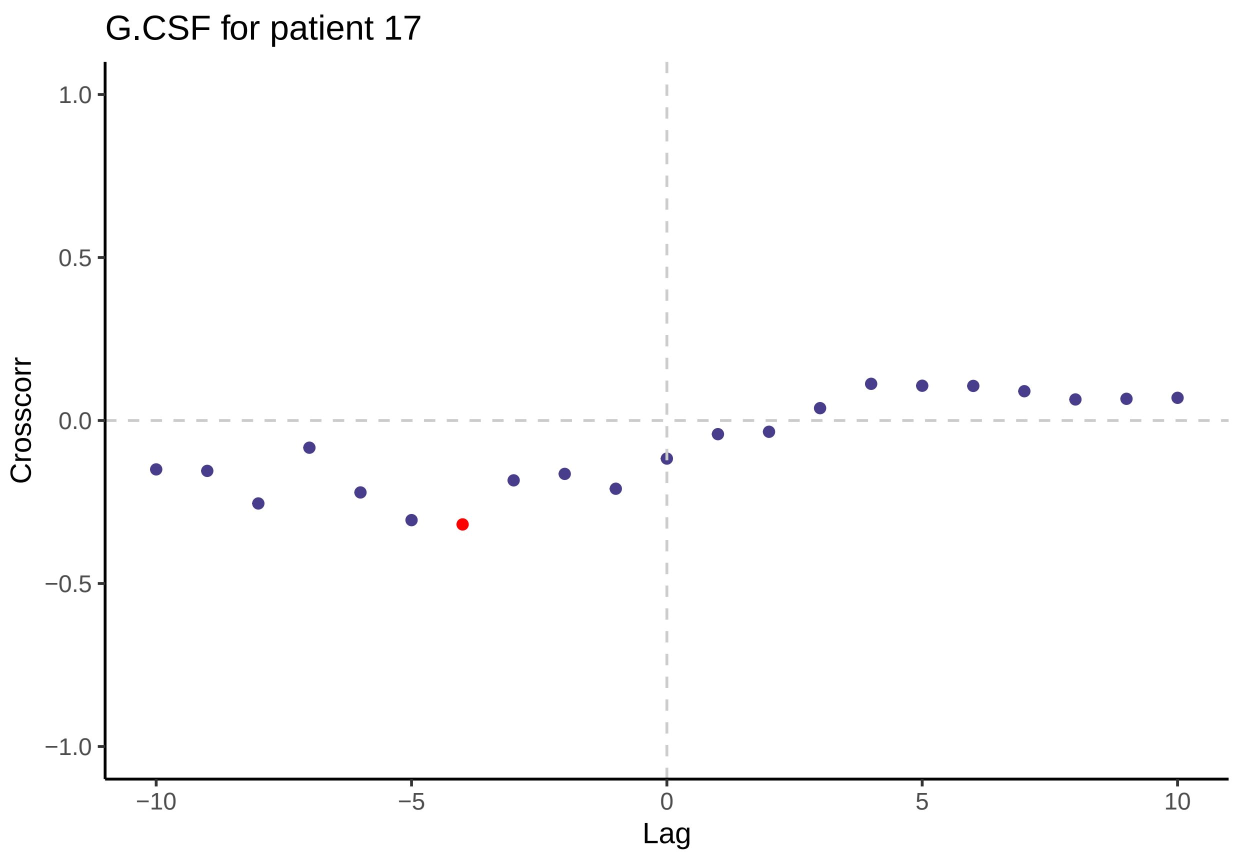


```
## [1] "G.CSF for patient 17 - p-value: 0.0273792879180031"
```

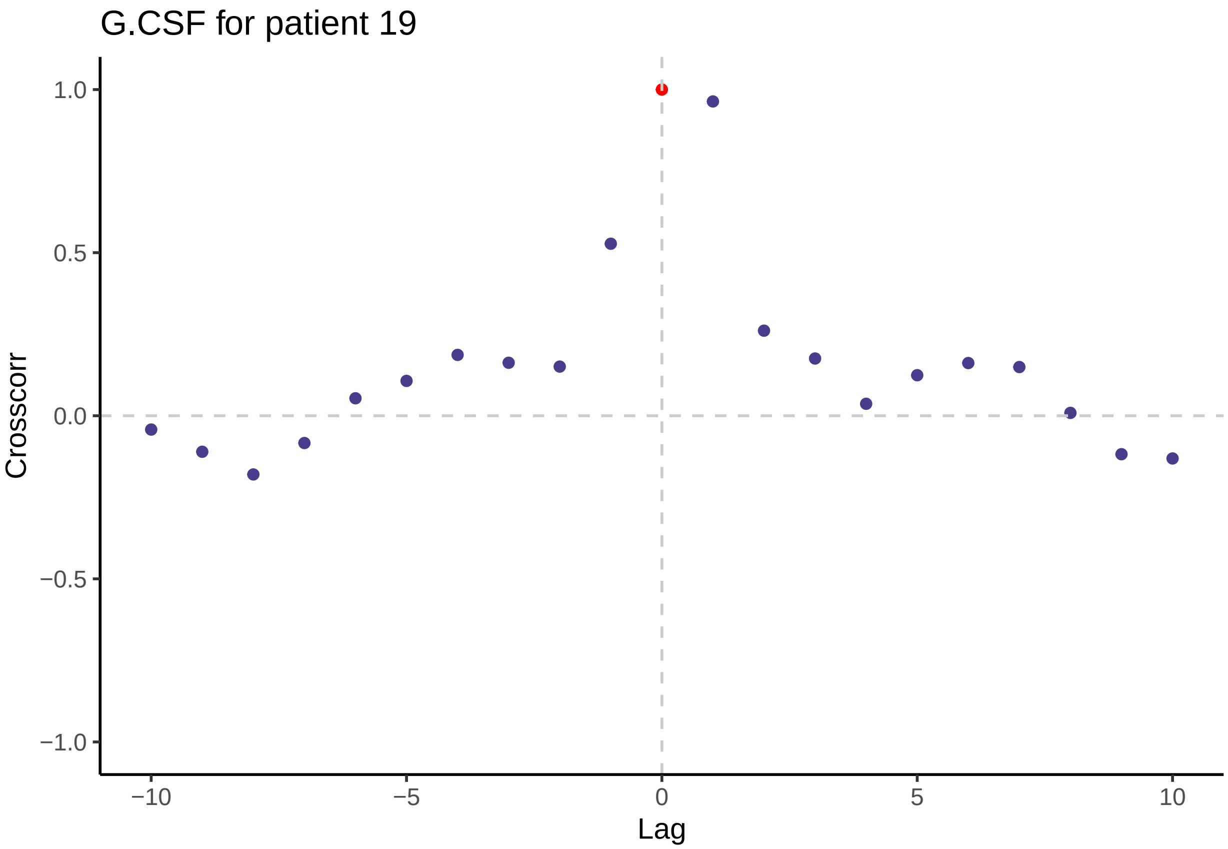


```
## [1] "G.CSF for patient 19 - p-value: 0.0293160953053918"
```

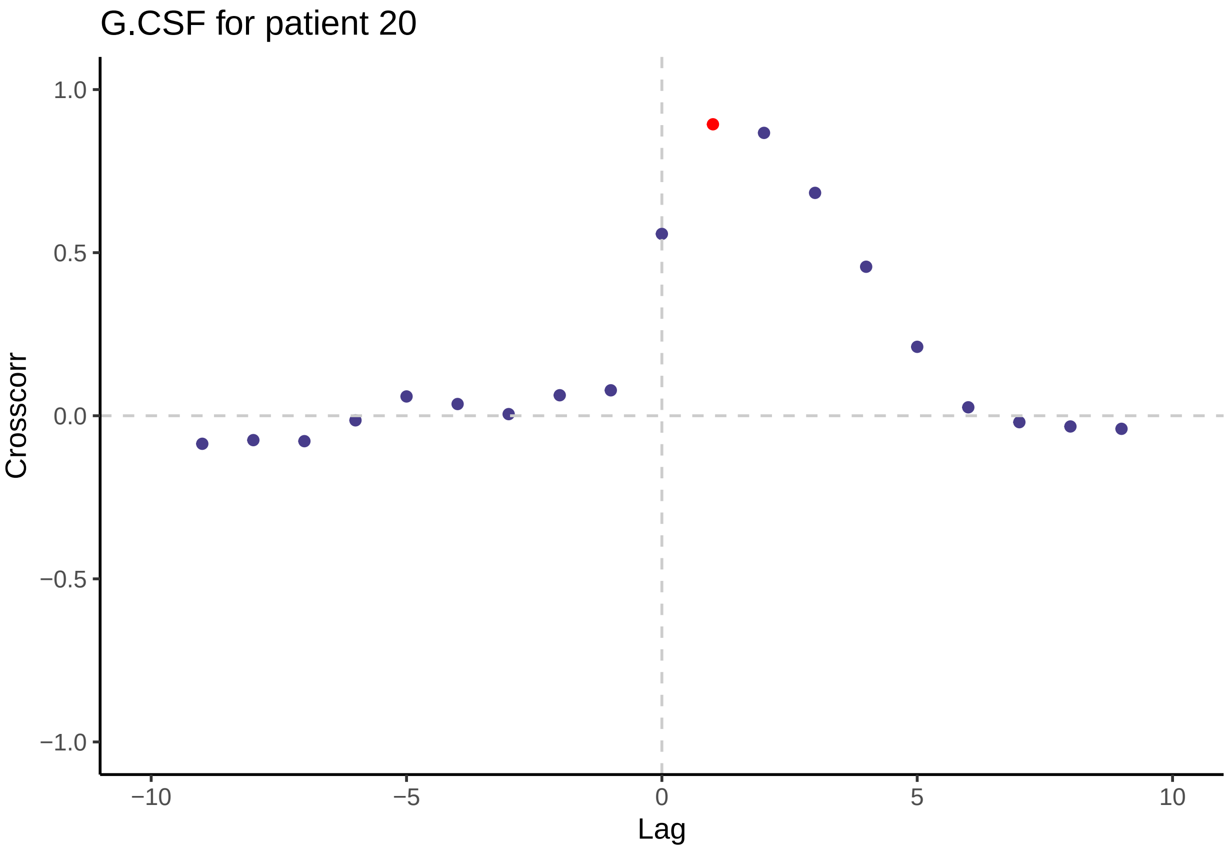


```
## [1] "G.CSF for patient 20 - p-value: 0.0218653116459317"
```

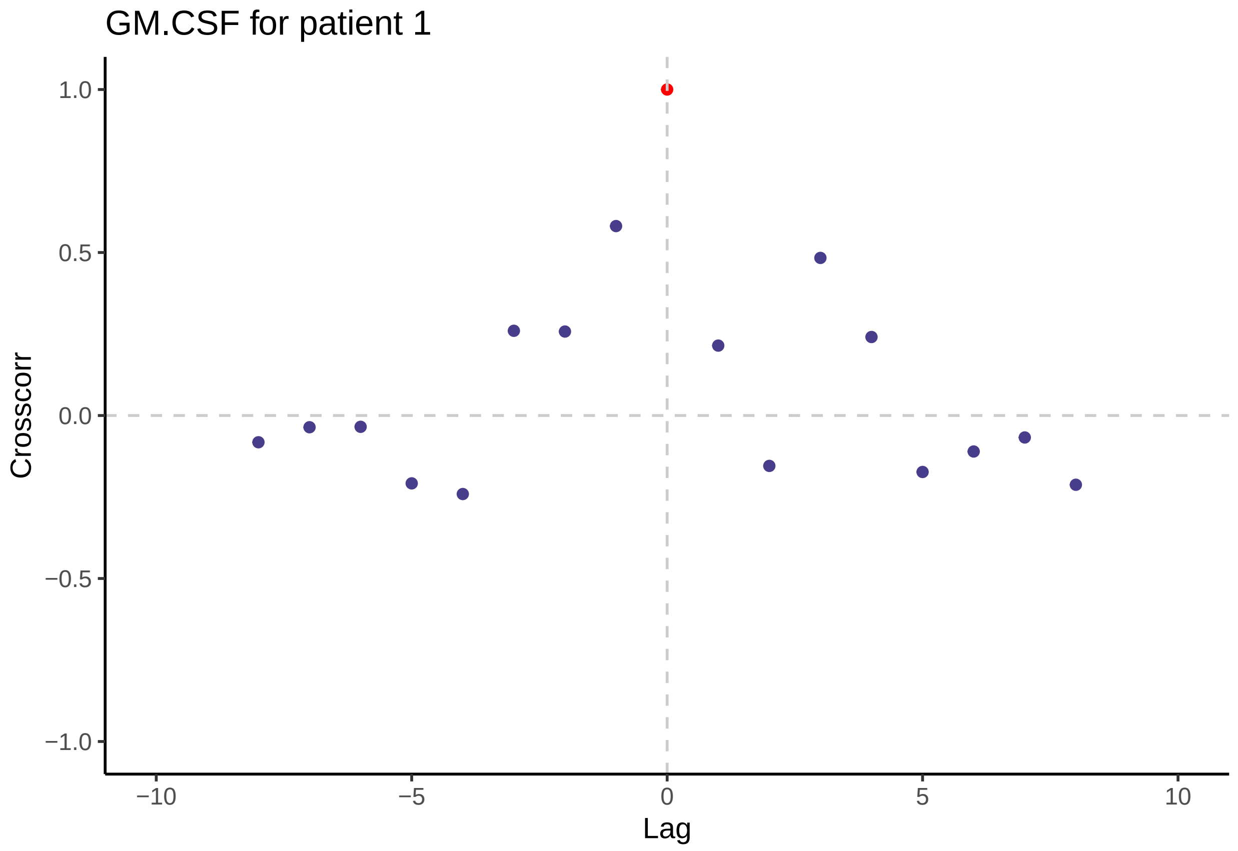


```
## [1] "GM.CSF for patient 1 - p-value: 0.239707858334268"
```

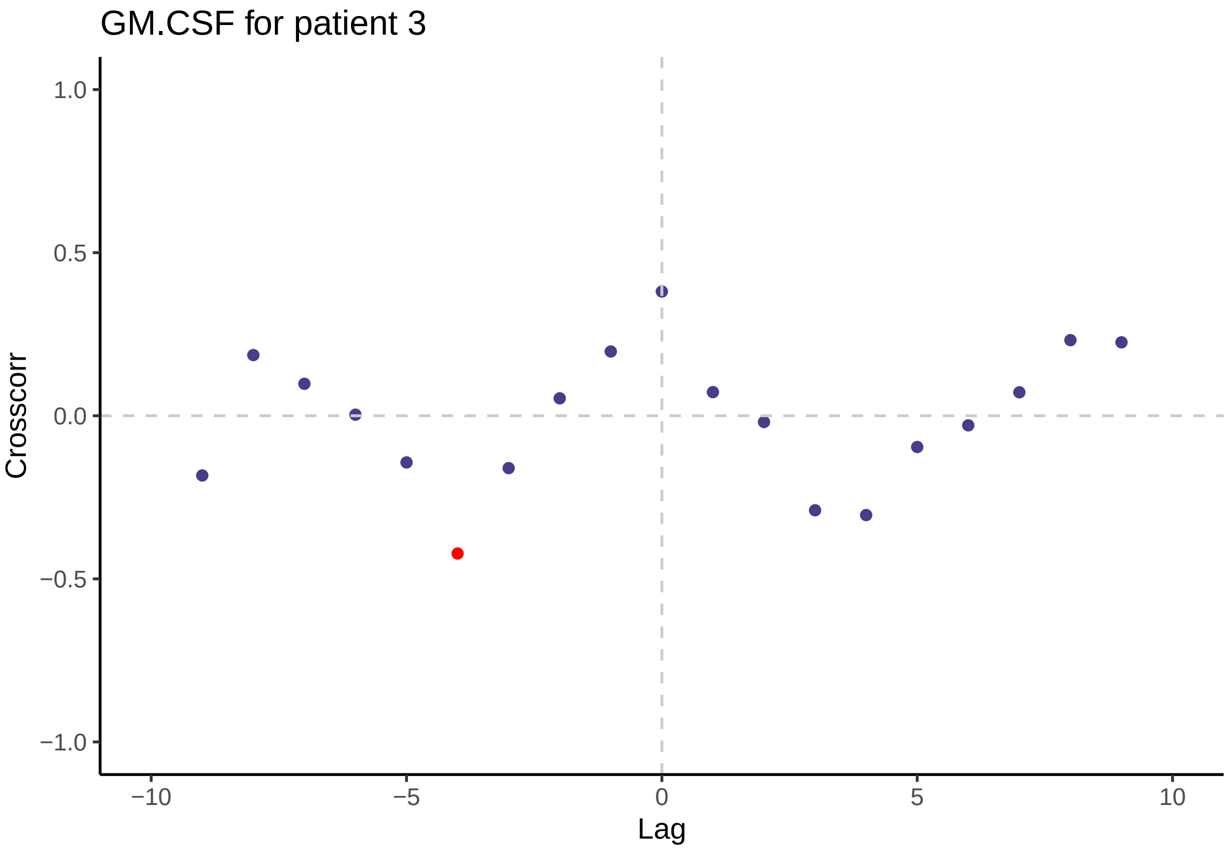


```
## [1] "GM.CSF for patient 3 - p-value: 0.890427020599969"
```

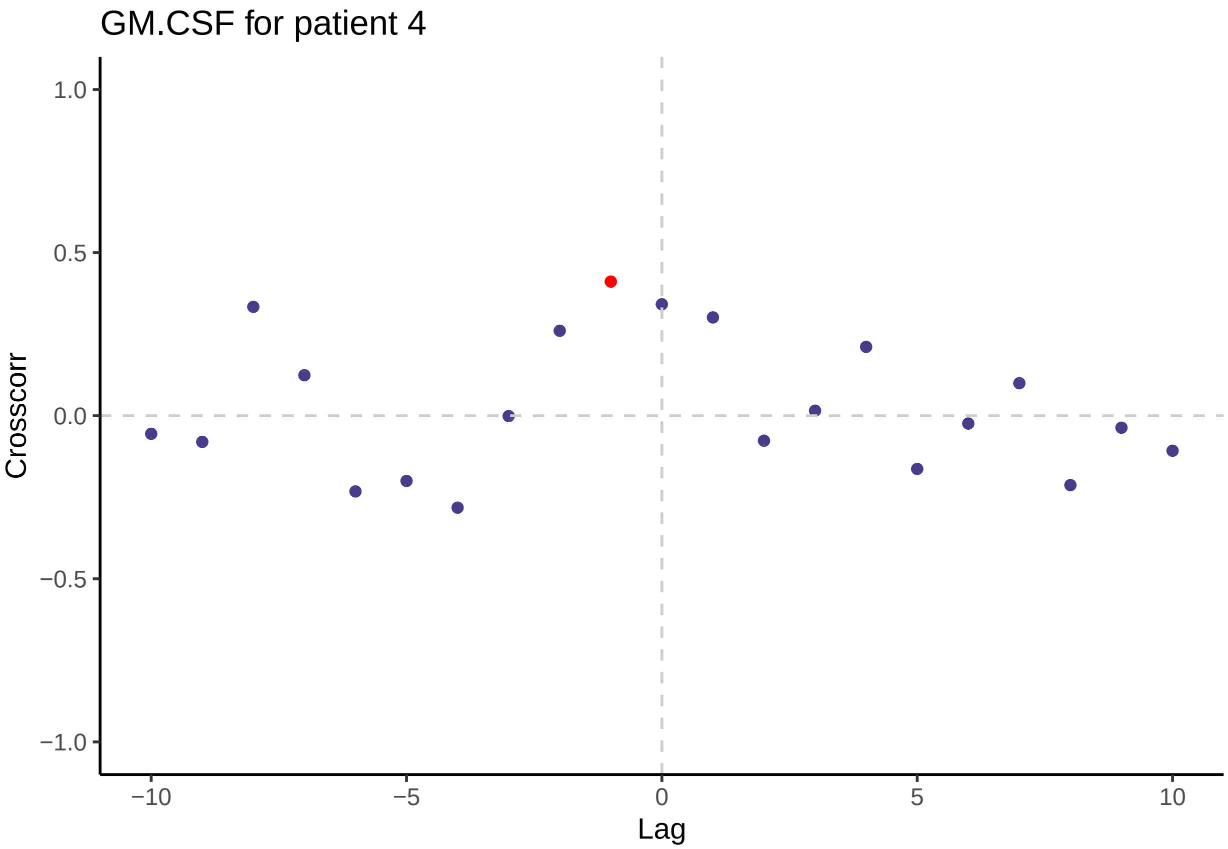


```
## [1] "GM.CSF for patient 4 - p-value: 0.520427181554808"
```

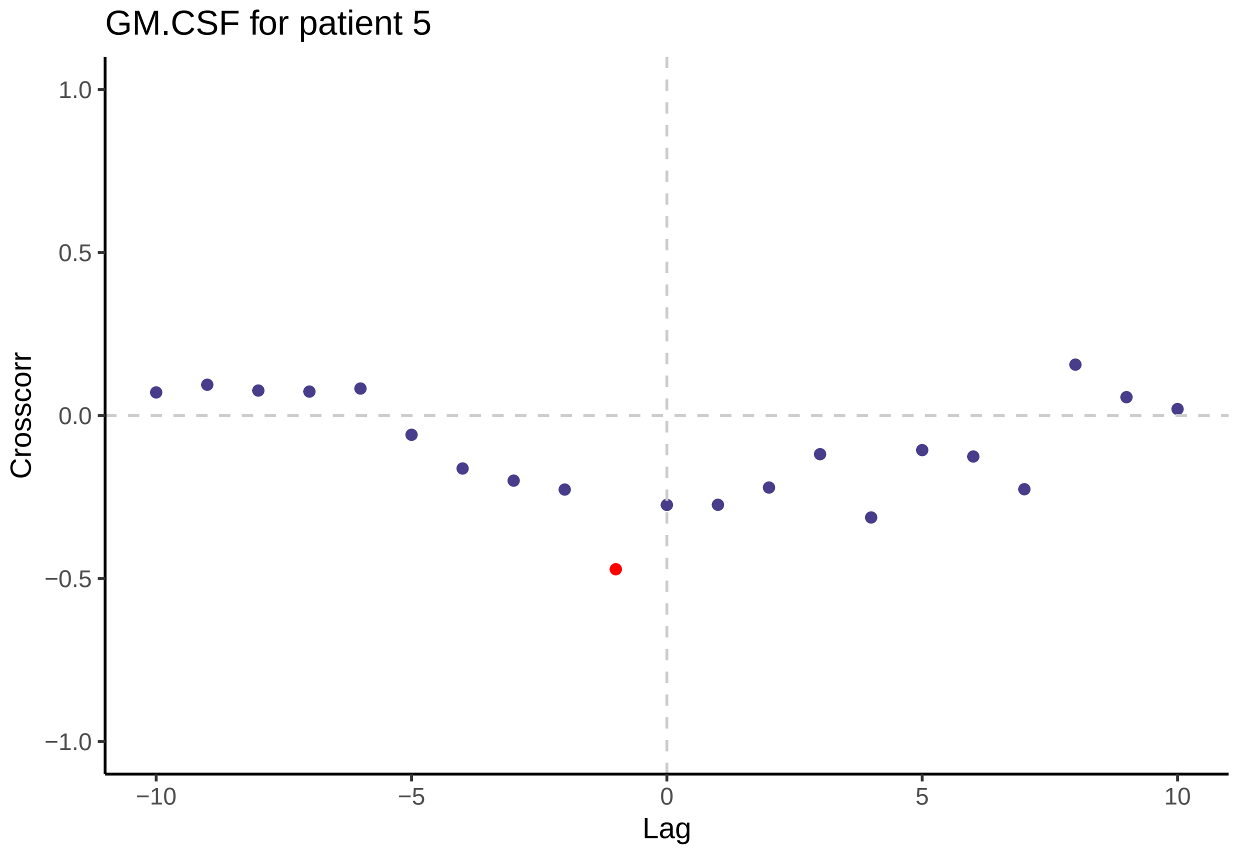


```
## [1] "GM.CSF for patient 5 - p-value: 0.0118038797801981"
```

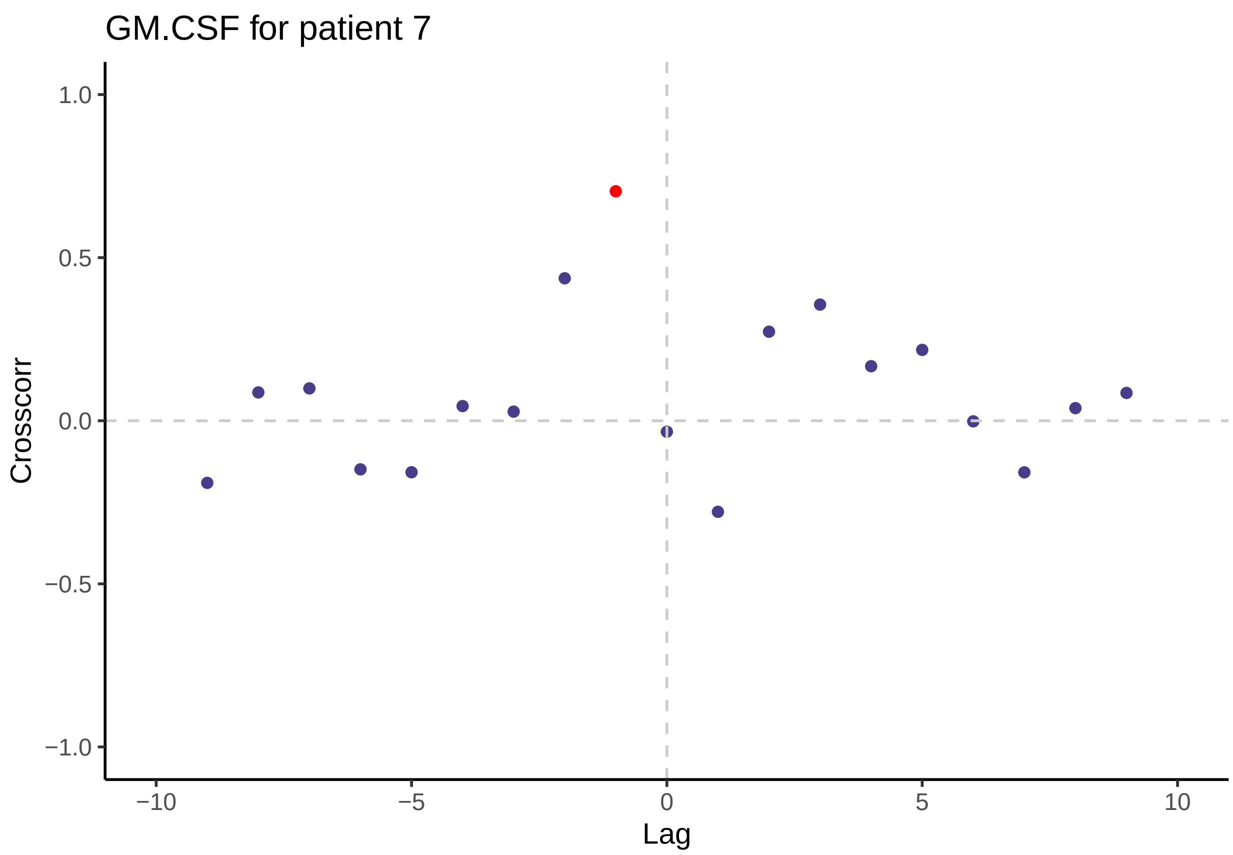


```
## [1] "GM.CSF for patient 7 - p-value: 0.153962924828532"
```

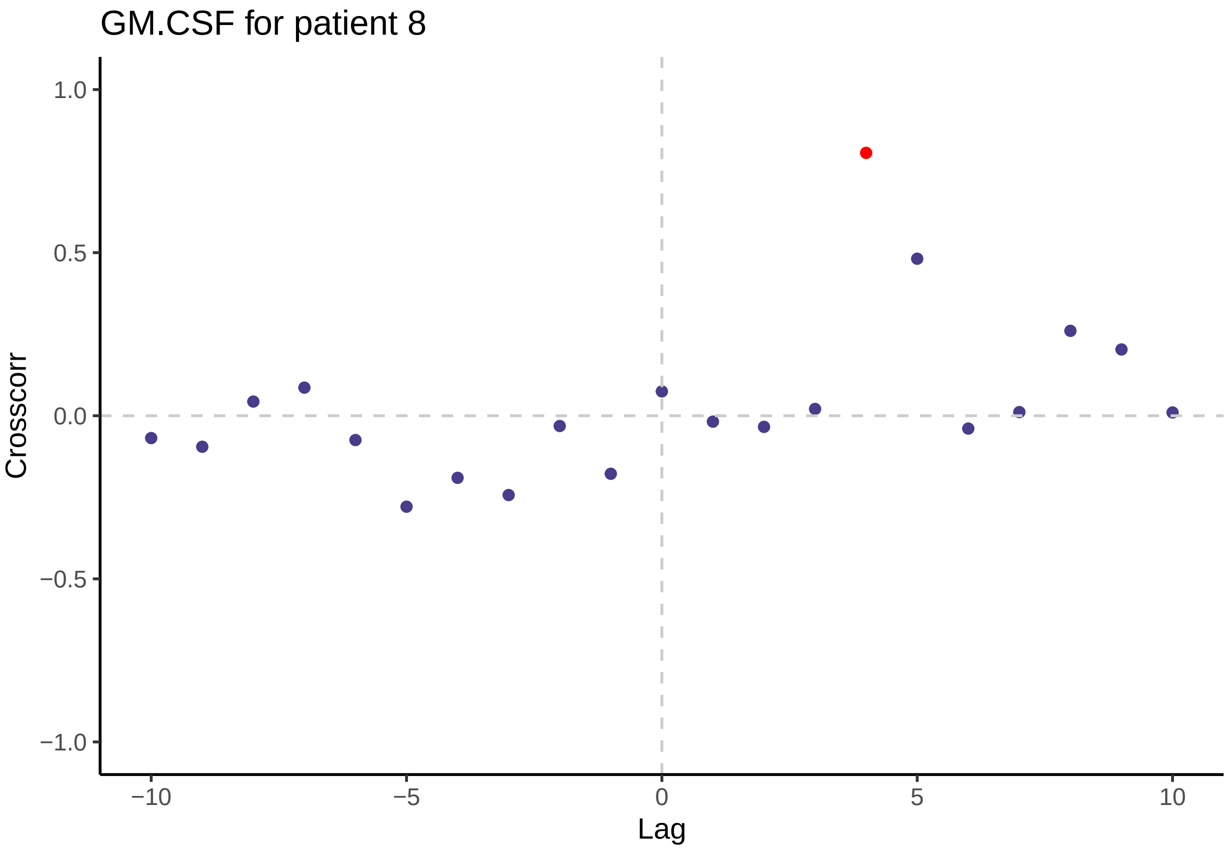


```
## [1] "GM.CSF for patient 8 - p-value: 0.514774698621221"
```

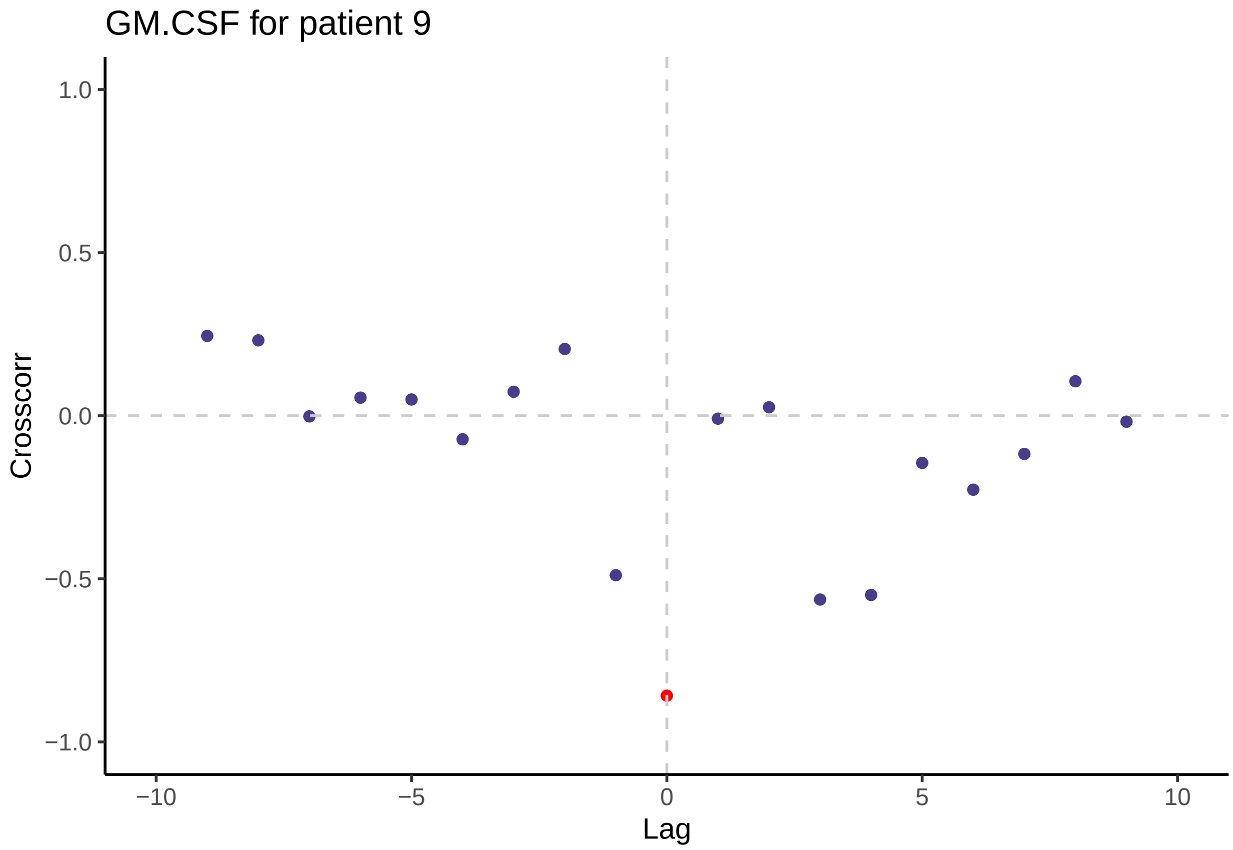


```
## [1] "GM.CSF for patient 9 - p-value: 0.135459023911363"
```

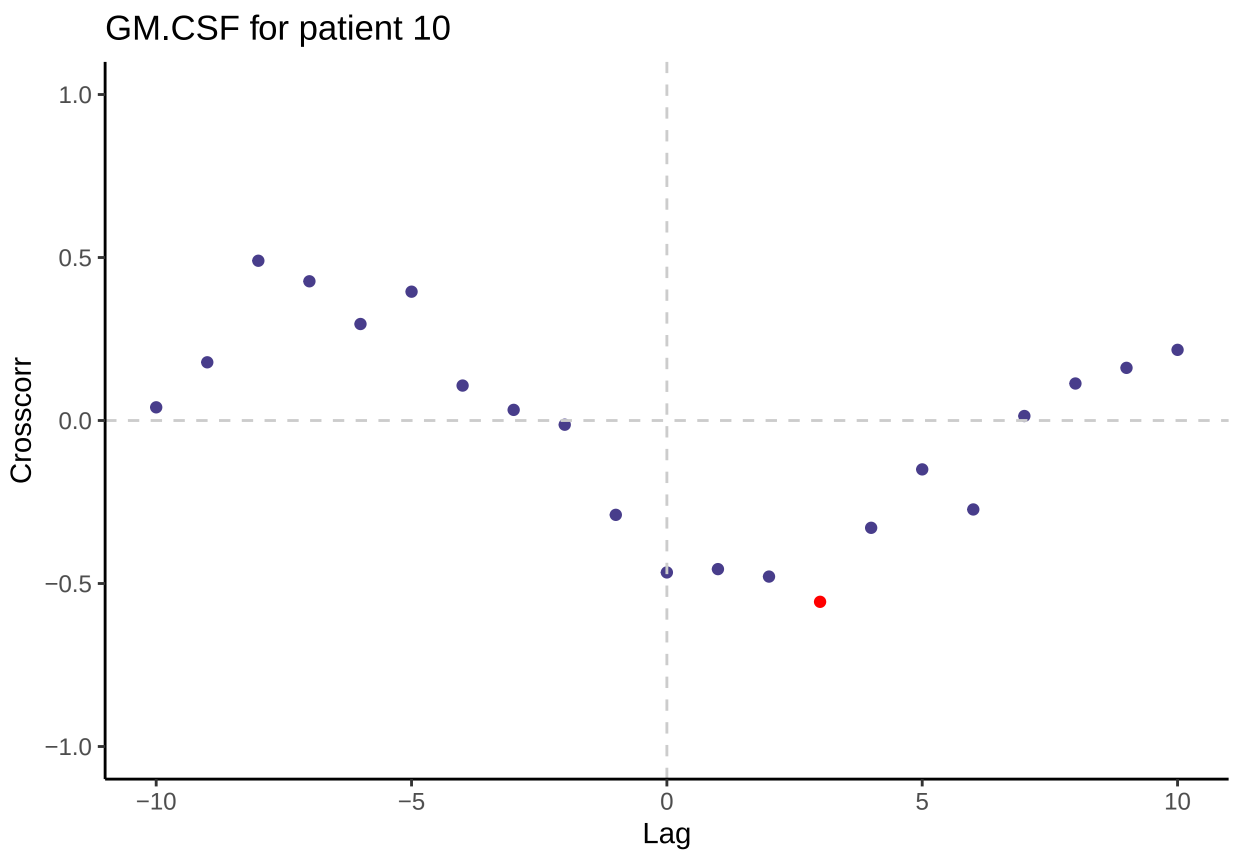


```
## [1] "GM.CSF for patient 10 - p-value: 0.71682338733926"
```

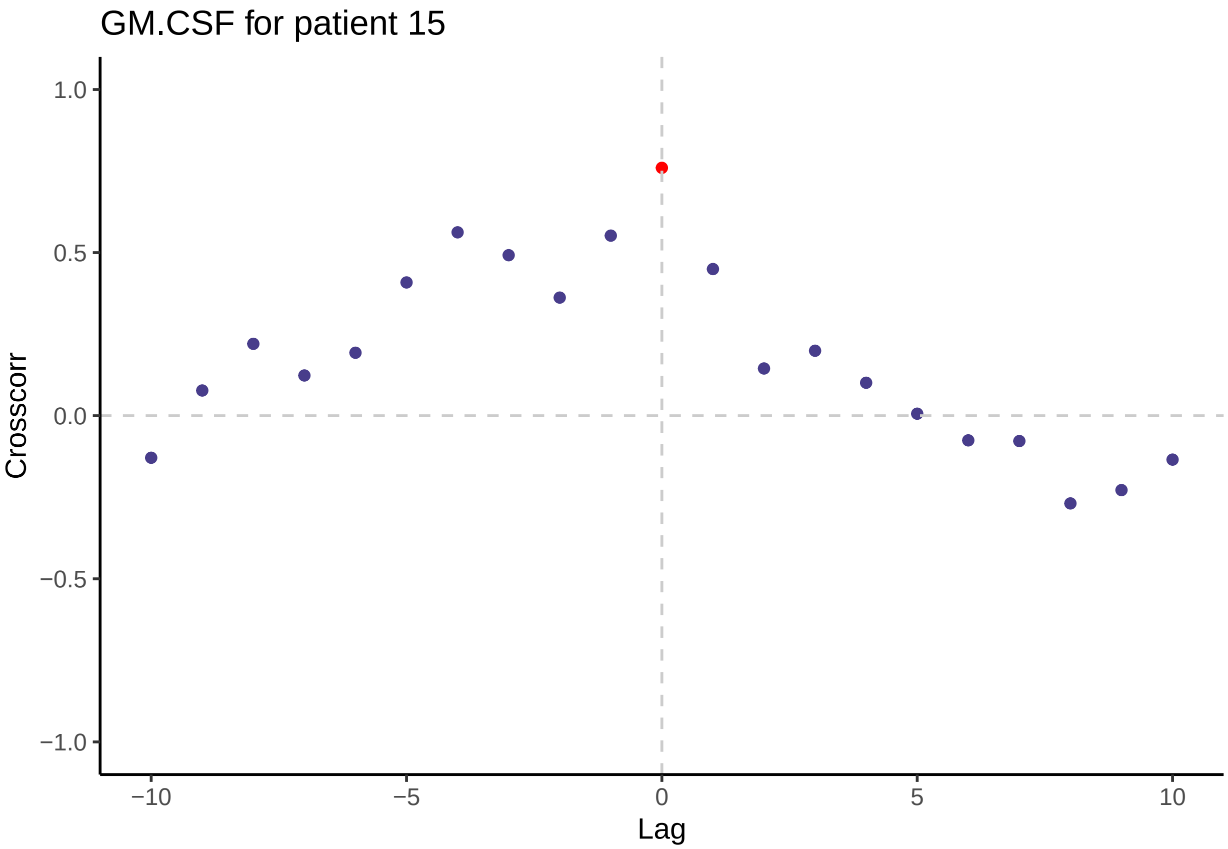


```
## [1] "GM.CSF for patient 15 - p-value: 0.00957471942065312"
```

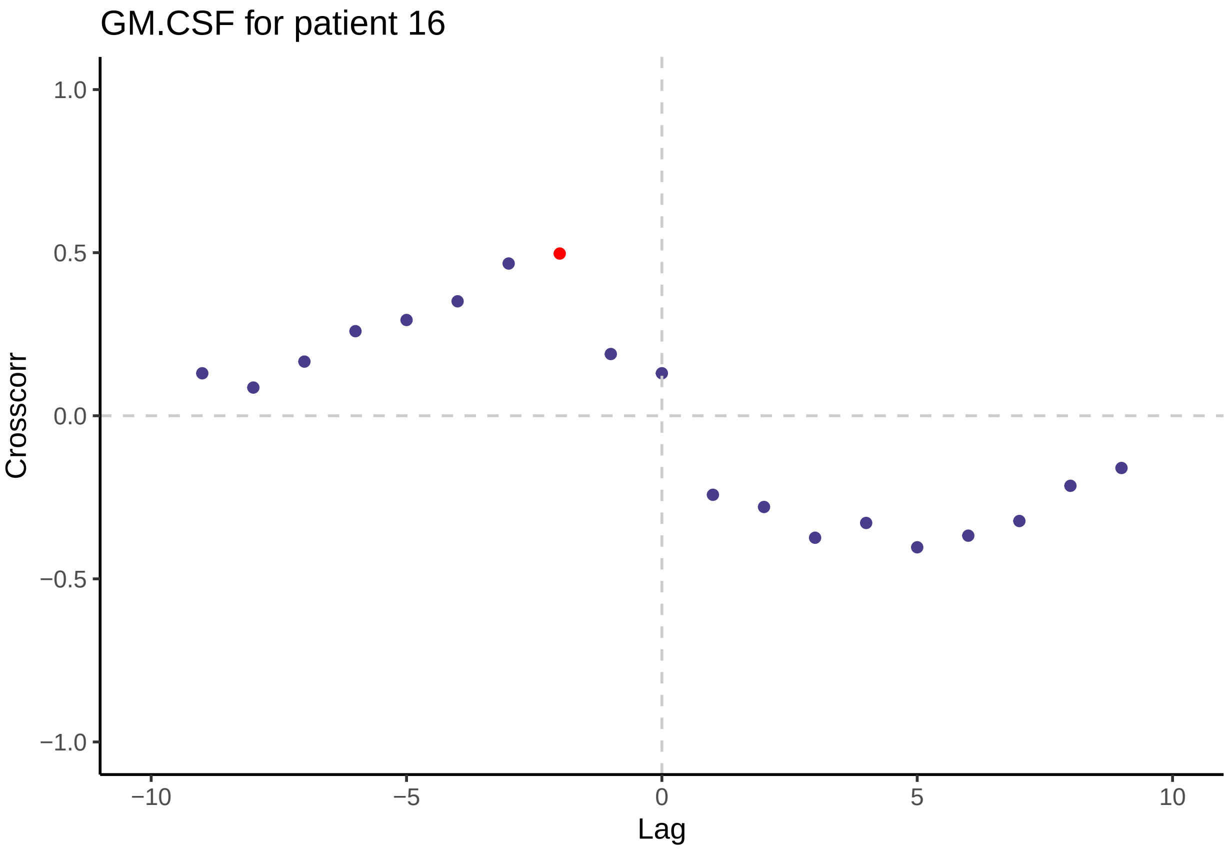


```
## [1] "GM.CSF for patient 16 - p-value: 0.928090245102229"
```

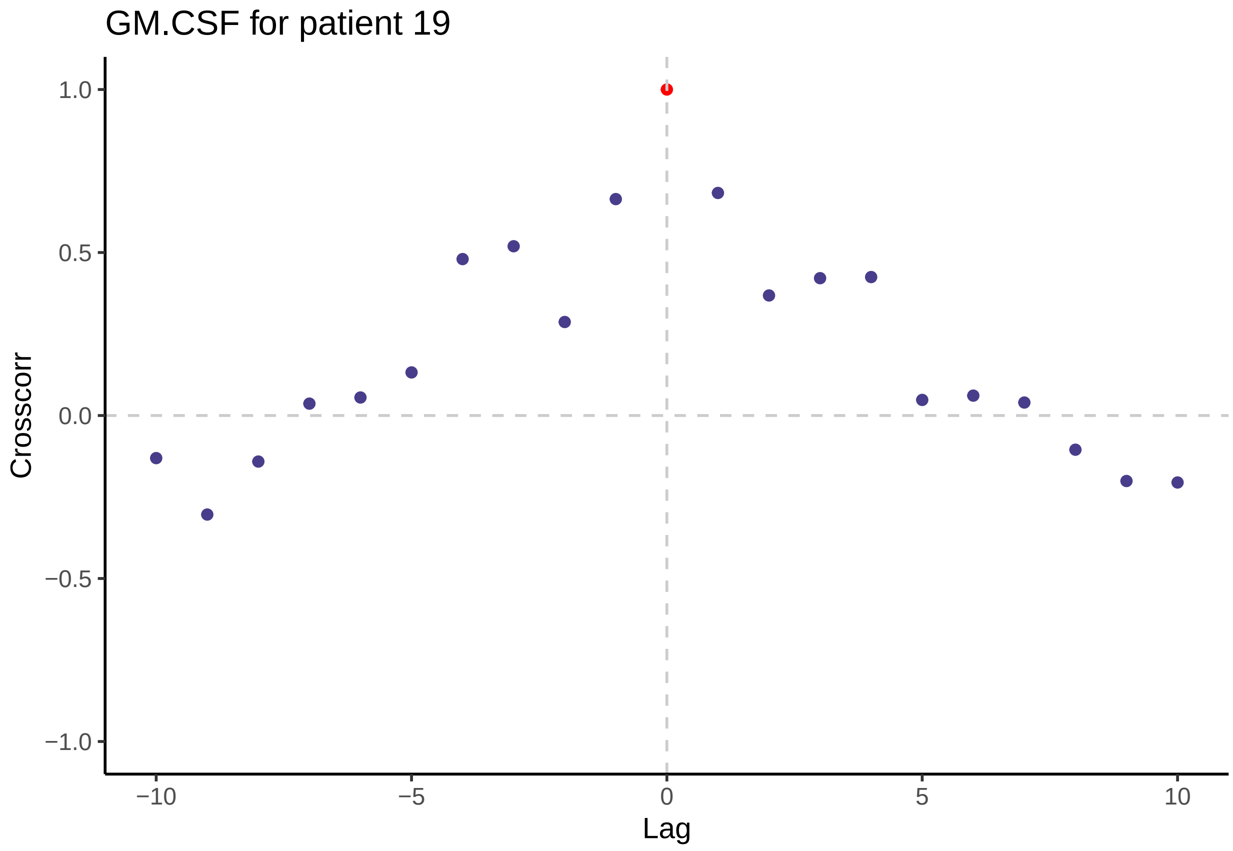


```
## [1] "GM.CSF for patient 19 - p-value: 0.0174426111115853"
```

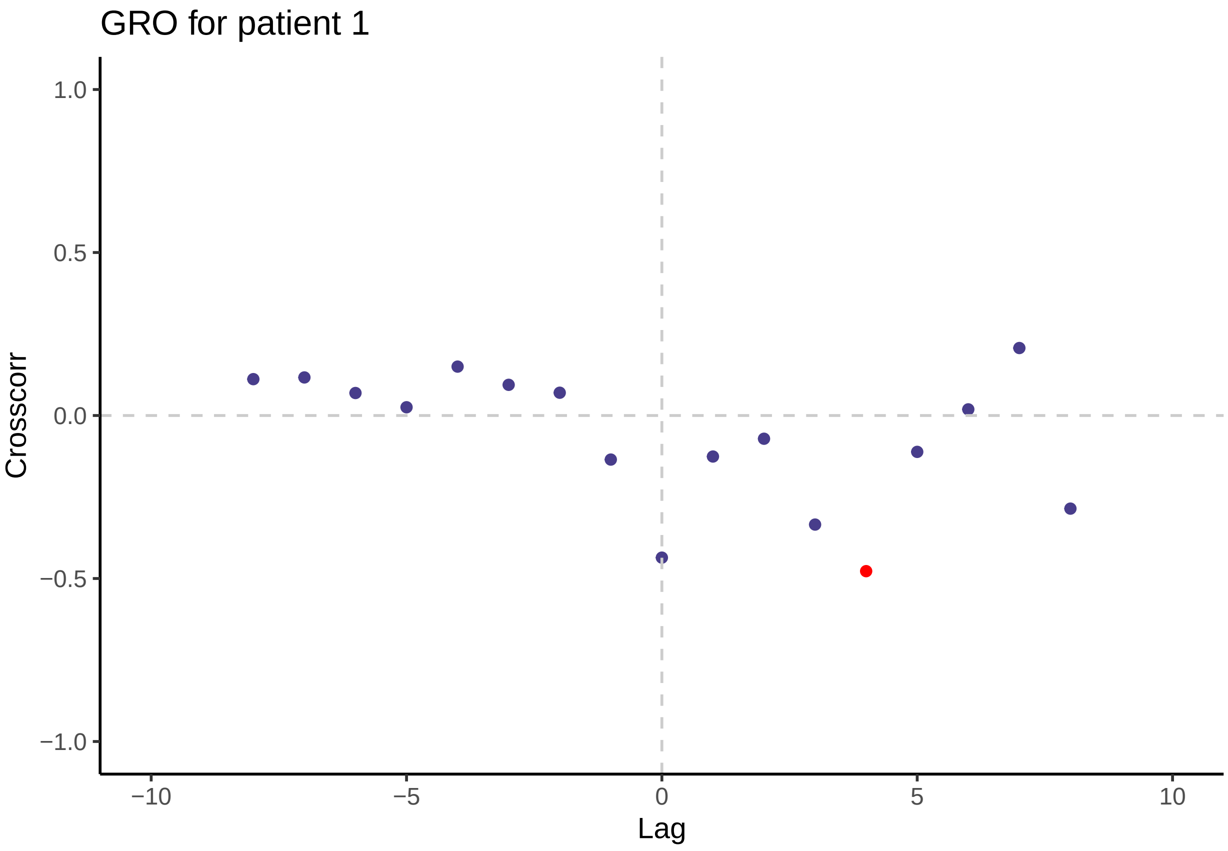


```
## [1] "GRO for patient 1 - p-value: 0.214781188252842"
```

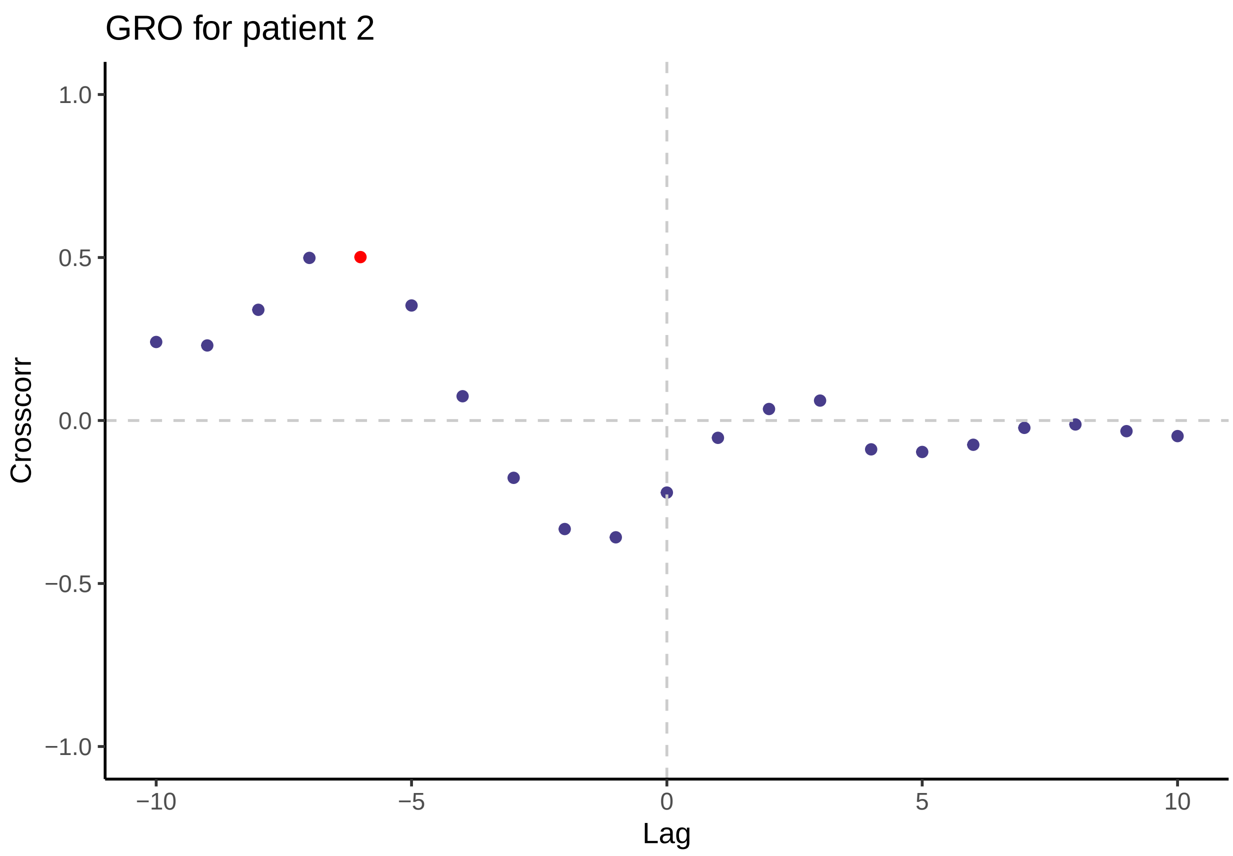


```
## [1] "GRO for patient 2 - p-value: 0.470038738570019"
```

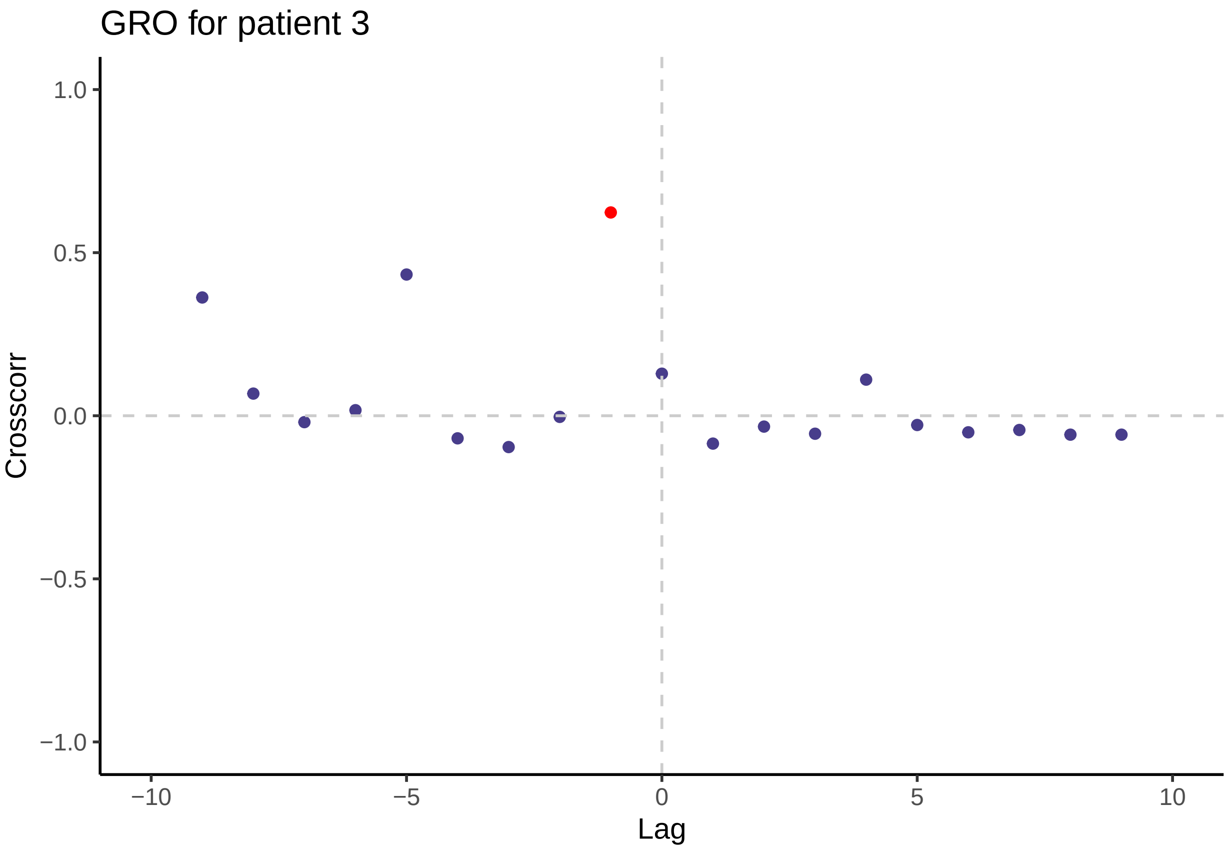


```
## [1] "GRO for patient 3 - p-value: 0.203368239414371"
```

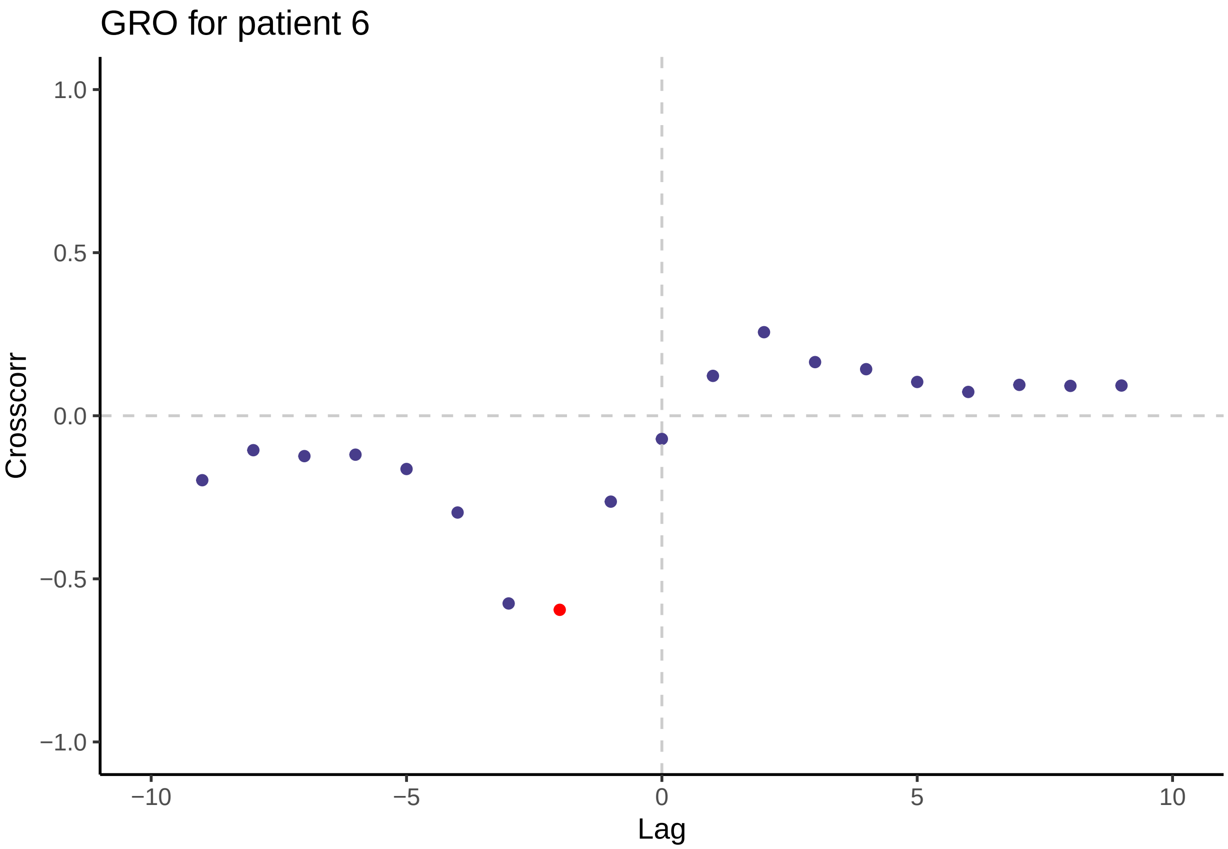


```
## [1] "GRO for patient 6 - p-value: 0.20451553773981"
```

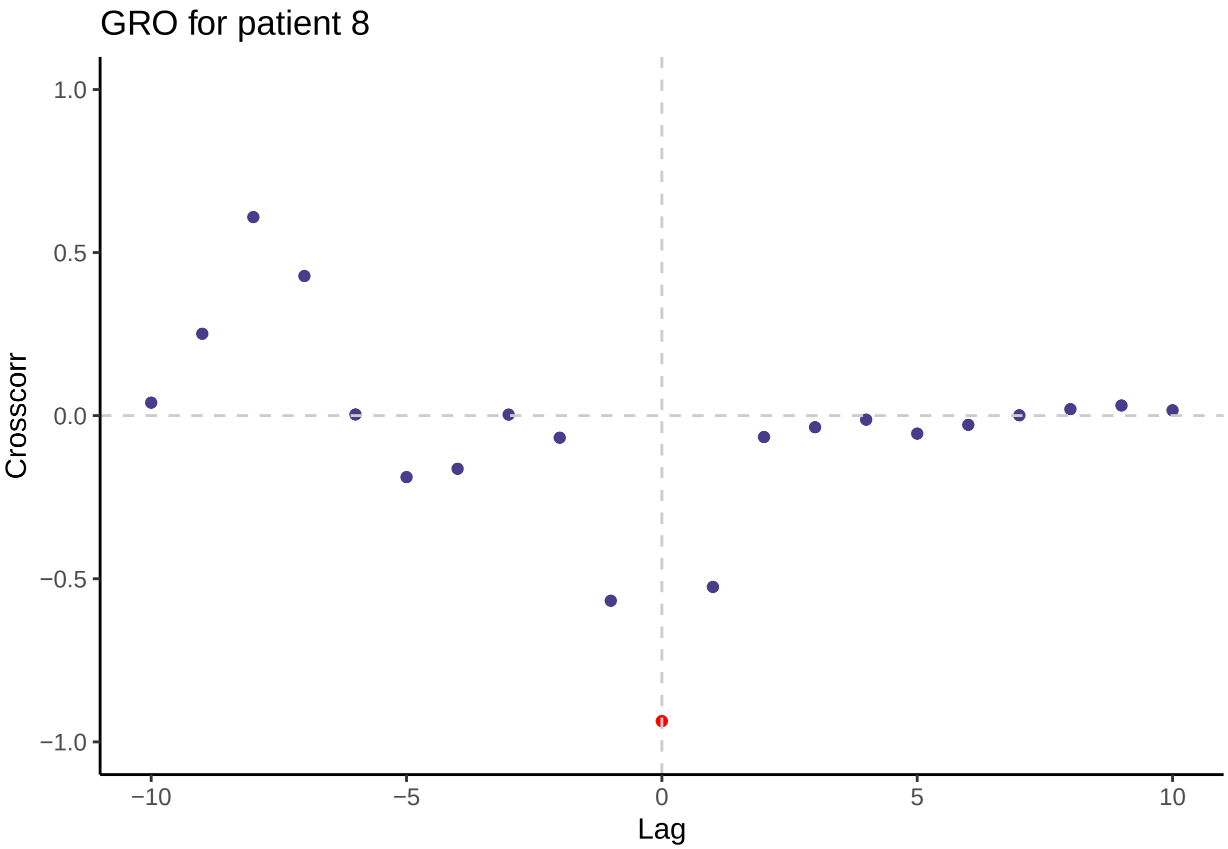


```
## [1] "GRO for patient 8 - p-value: 0.415411076450063"
```

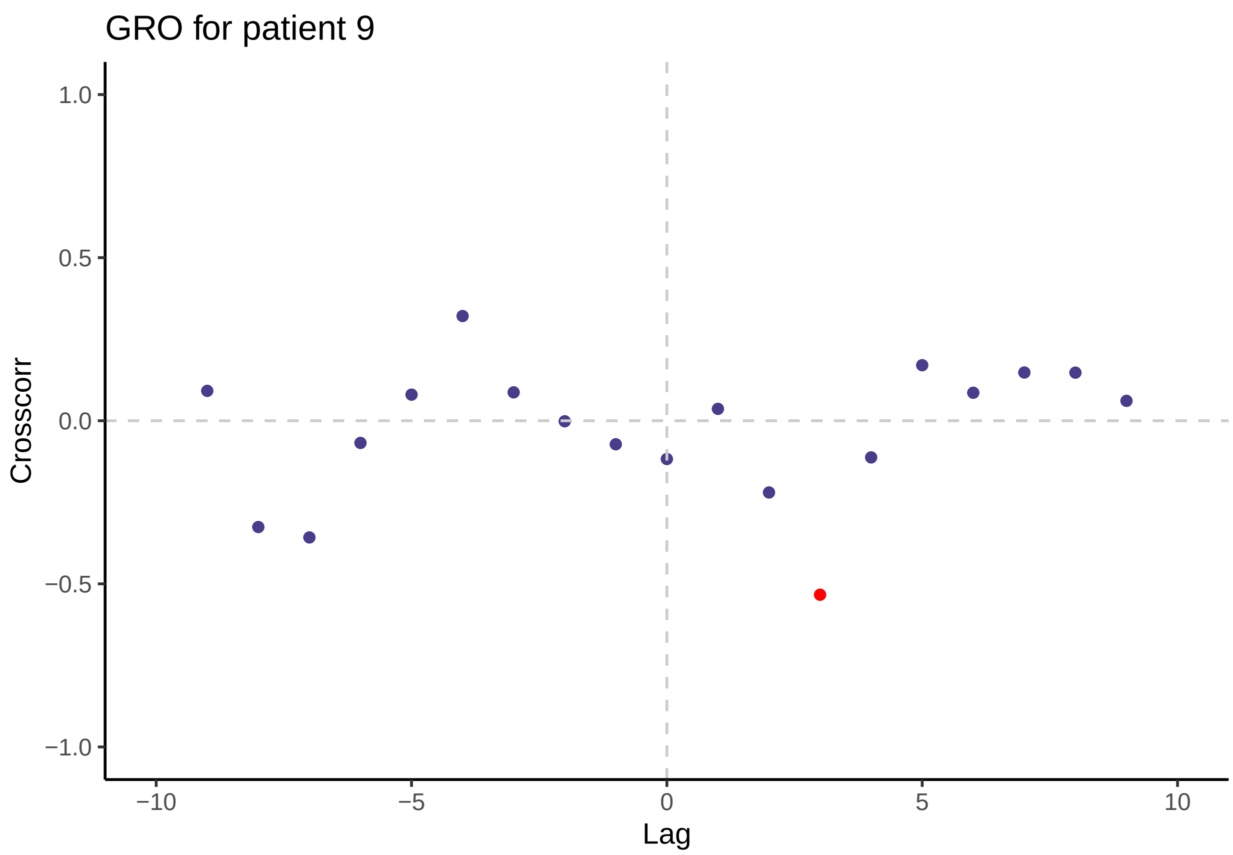


```
## [1] "GRO for patient 9 - p-value: 0.535636202017912"
```

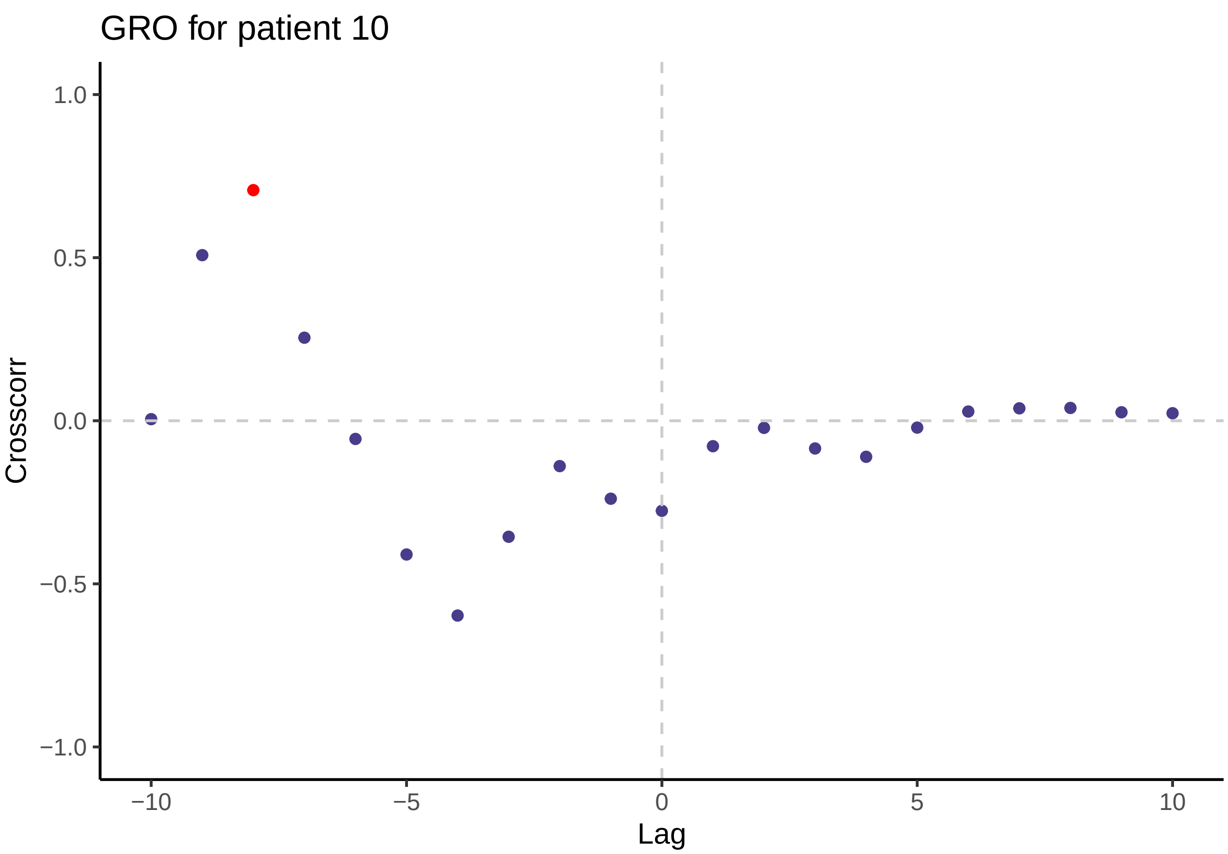


```
## [1] "GRO for patient 10 - p-value: 0.568259010926121"
```

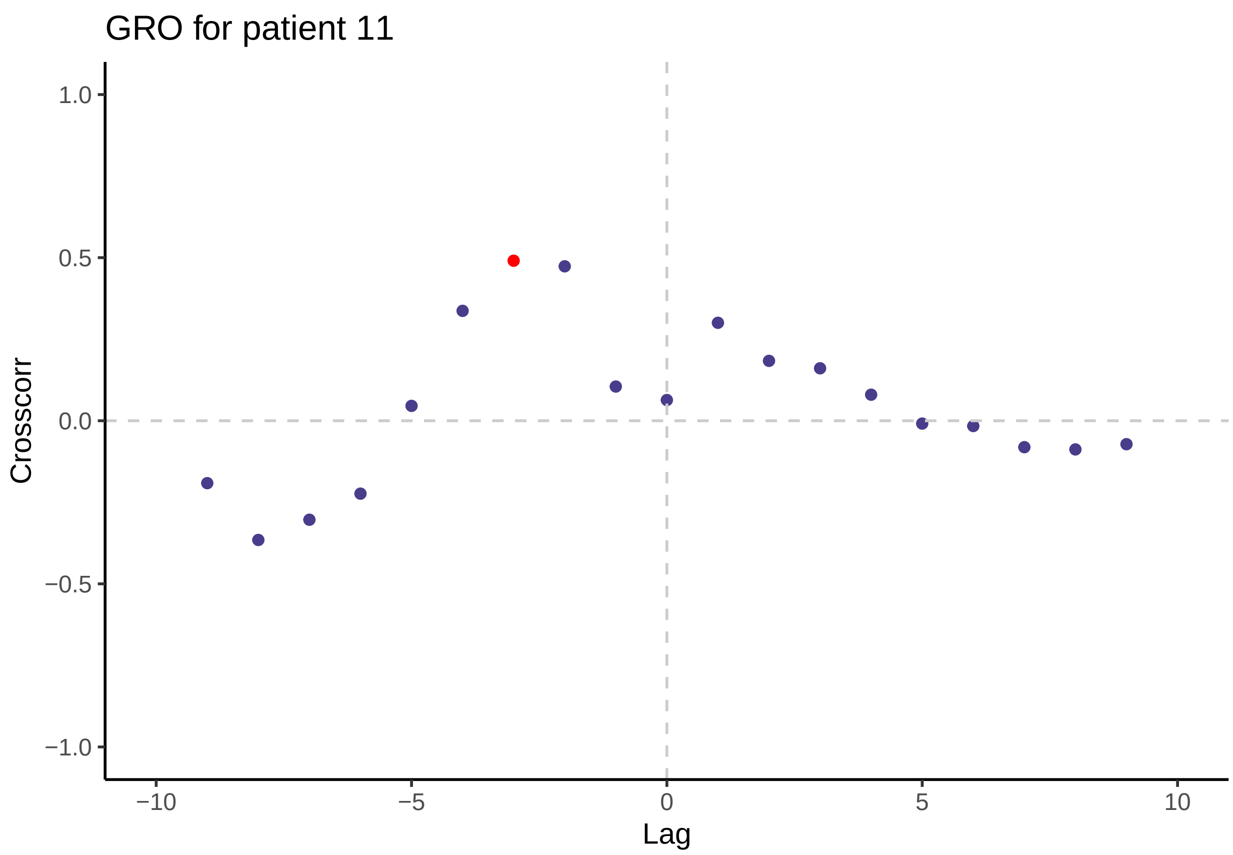


```
## [1] "GRO for patient 11 - p-value: 0.40684881388911"
```

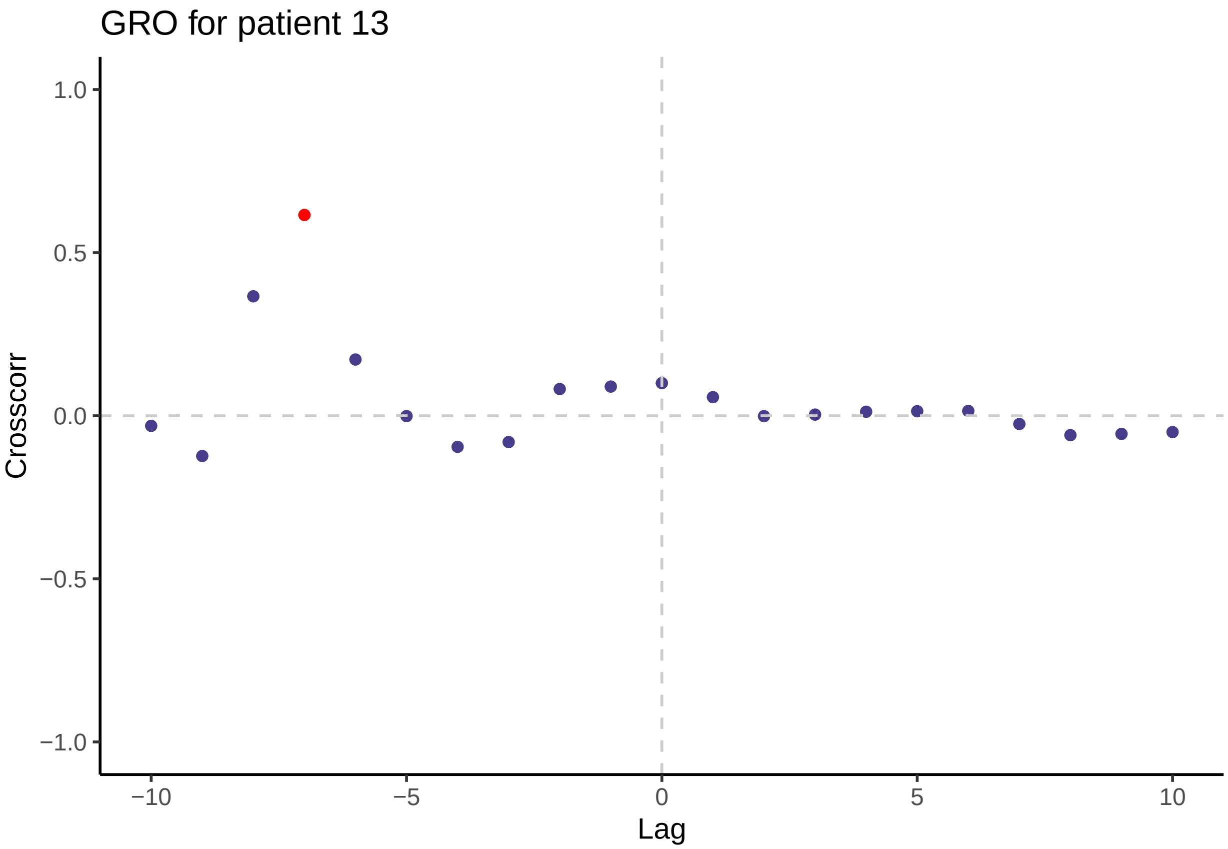


```
## [1] "GRO for patient 13 - p-value: 0.207187048246824"
```

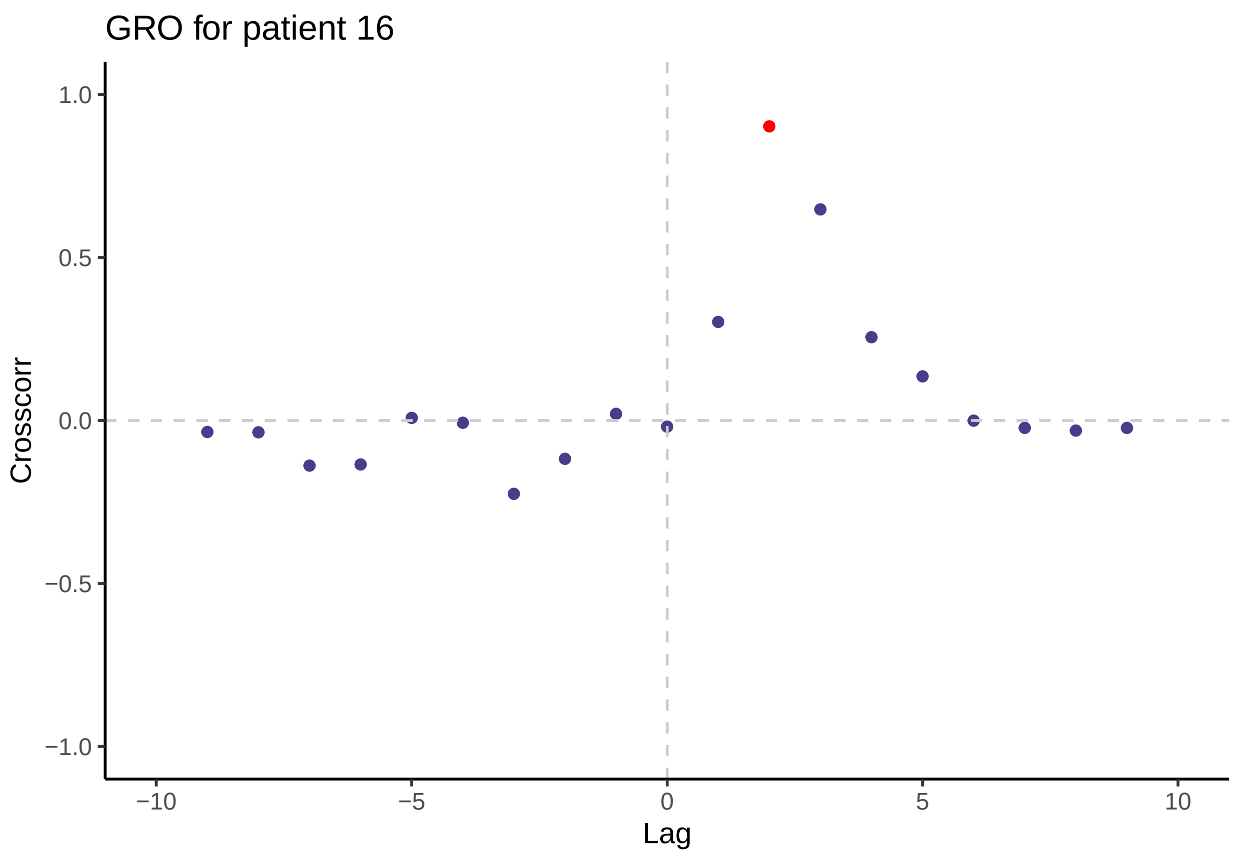


```
## [1] "GRO for patient 16 - p-value: 0.238495396281208"
```

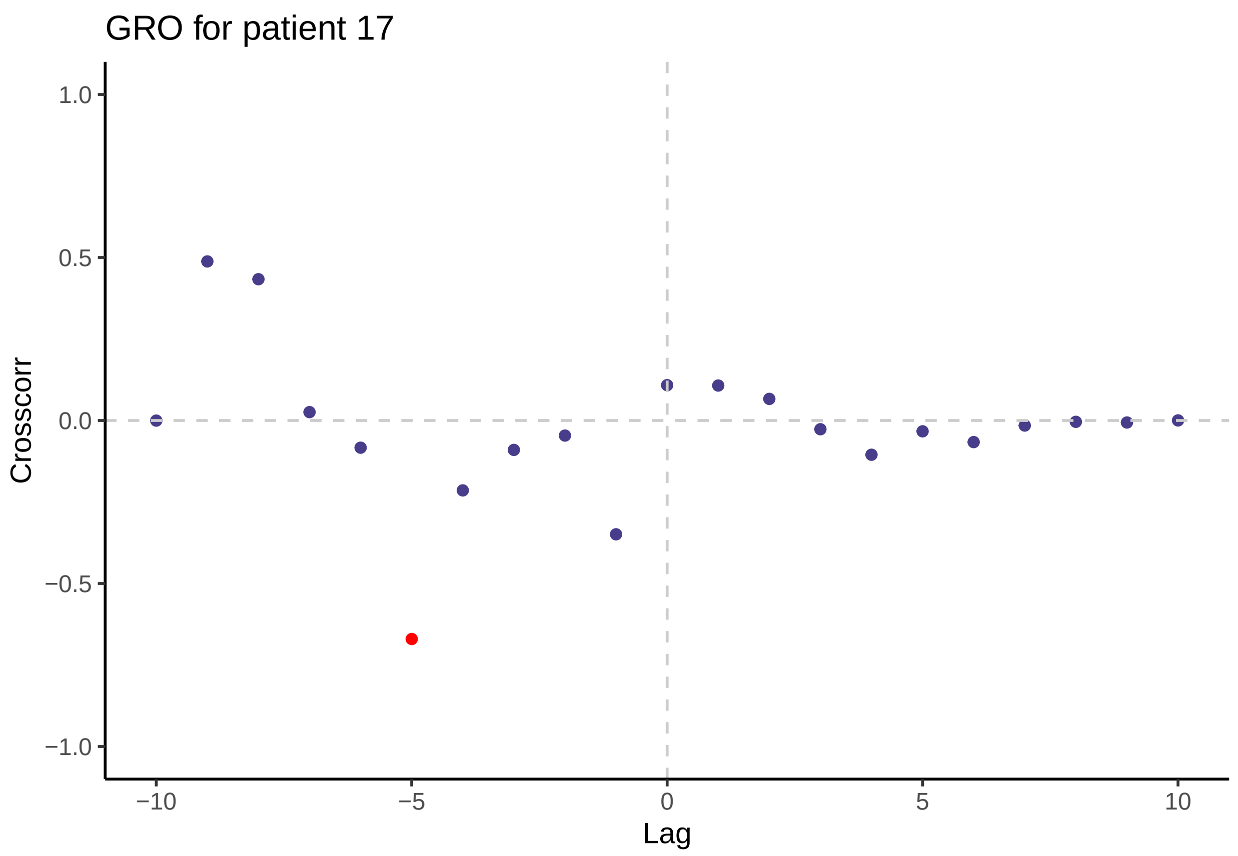


```
## [1] "GRO for patient 17 - p-value: 0.658671136435693"
```

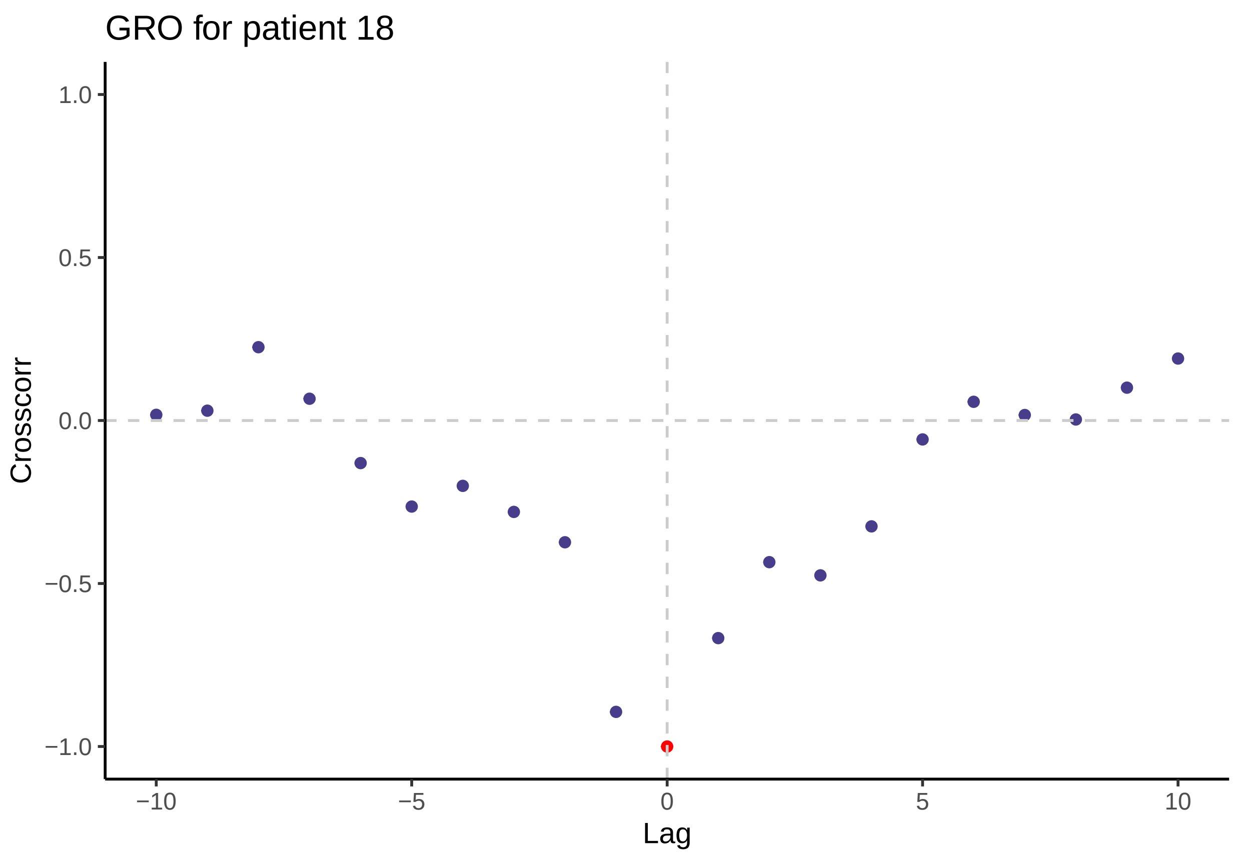


```
## [1] "GRO for patient 18 - p-value: 0.0103802173698875"
```

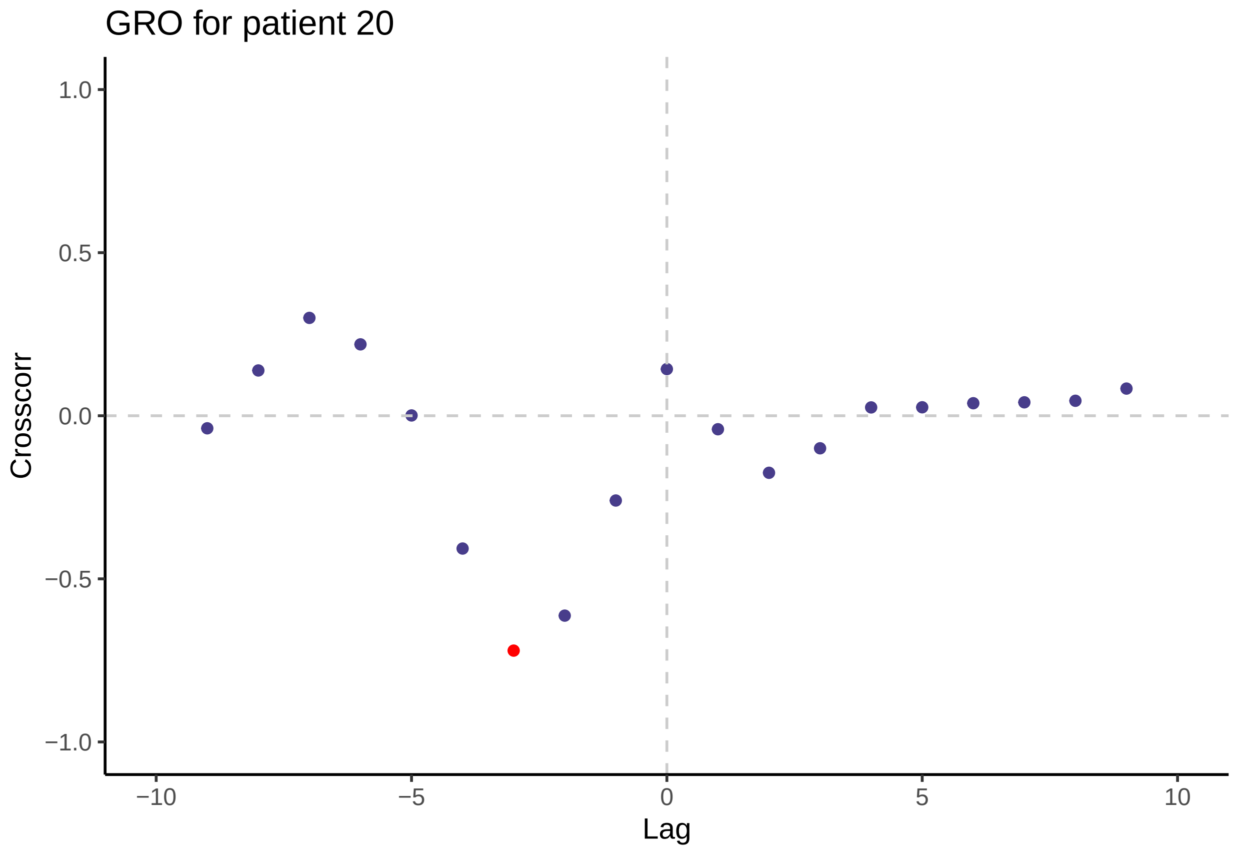


```
## [1] "GRO for patient 20 - p-value: 0.279938790393905"
## Warning: Removed 8 rows containing missing values (geom_point).
```

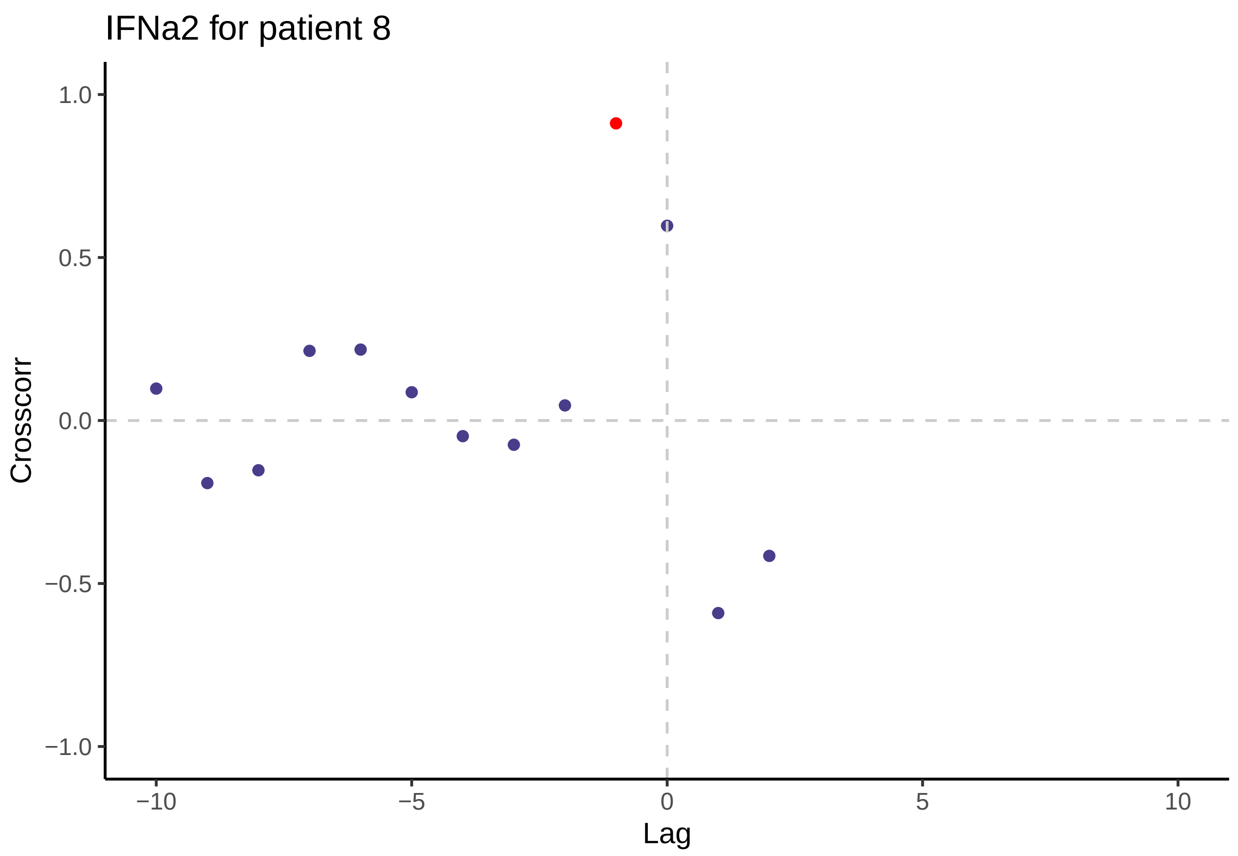


```
## [1] "IFNa2 for patient 8 - p-value: 0.630914293359589"

## Warning: Removed 1 rows containing missing values (geom_point).
```

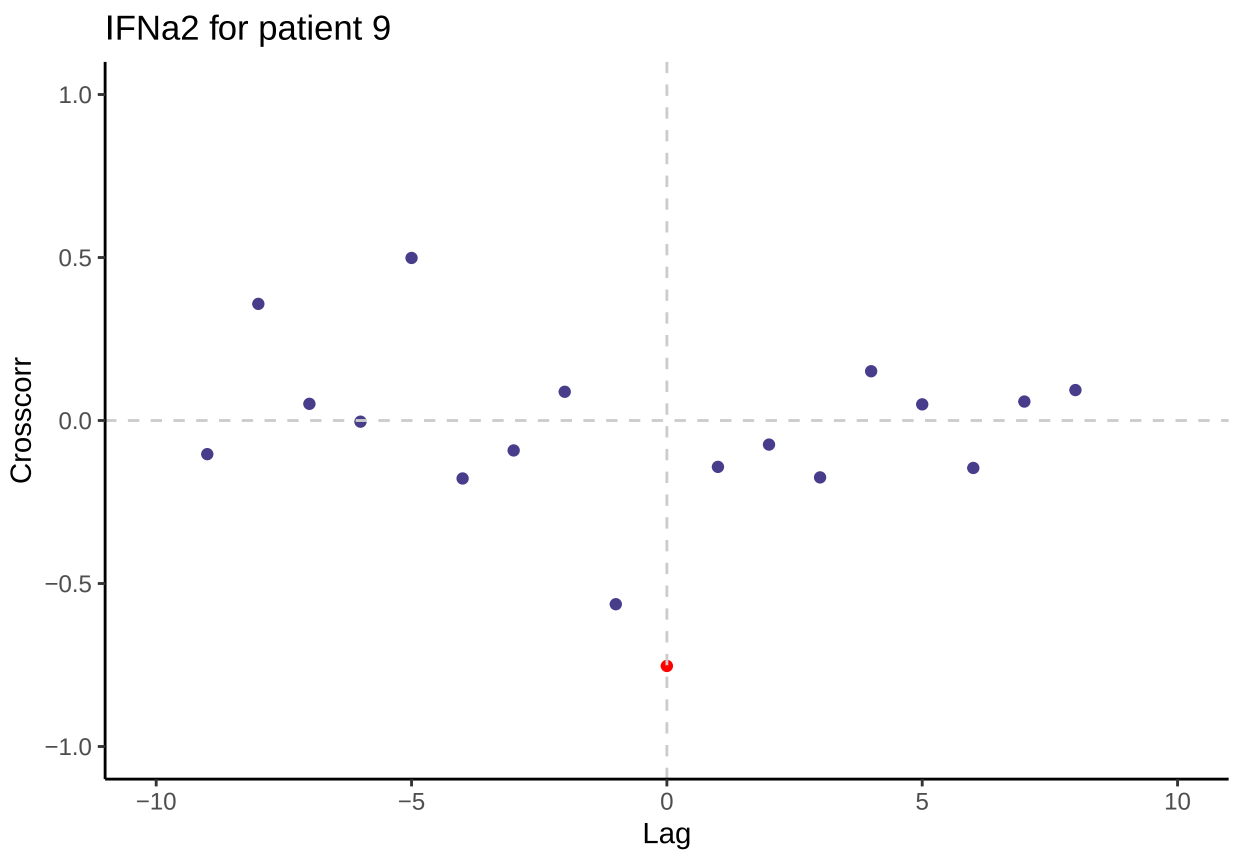


```
## [1] "IFNa2 for patient 9 - p-value: 0.47824312258887"
```

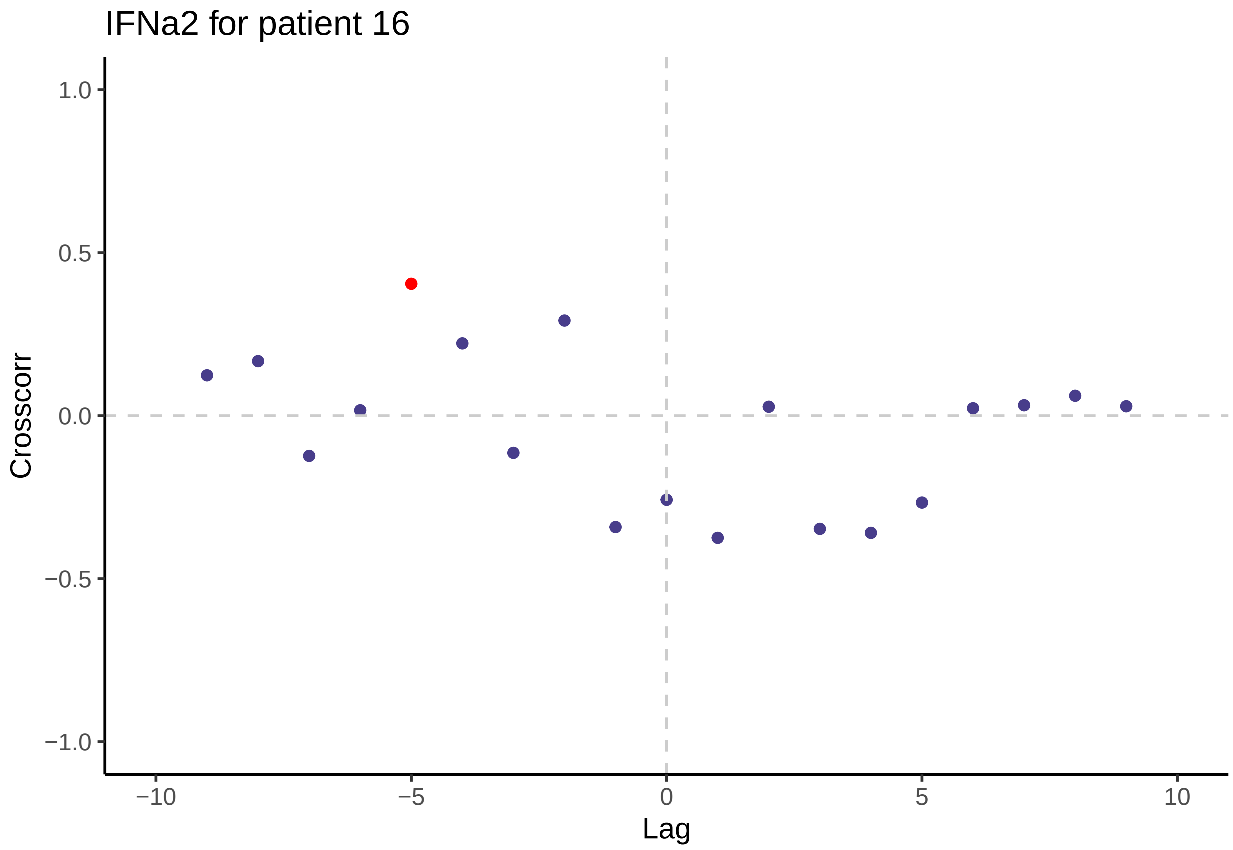


```
## [1] "IFNa2 for patient 16 - p-value: 0.453844600356184"
```

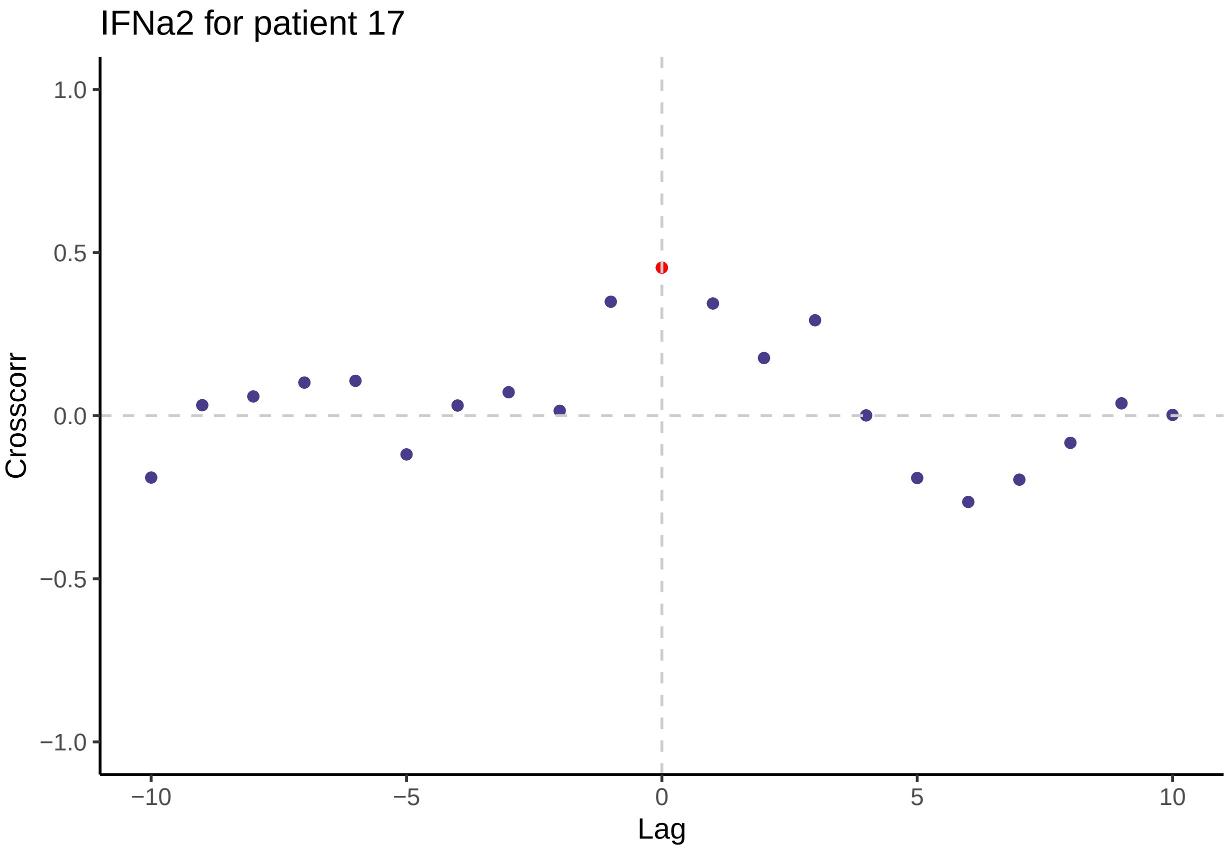


```
## [1] "IFNa2 for patient 17 - p-value: 0.256179076258858"
```

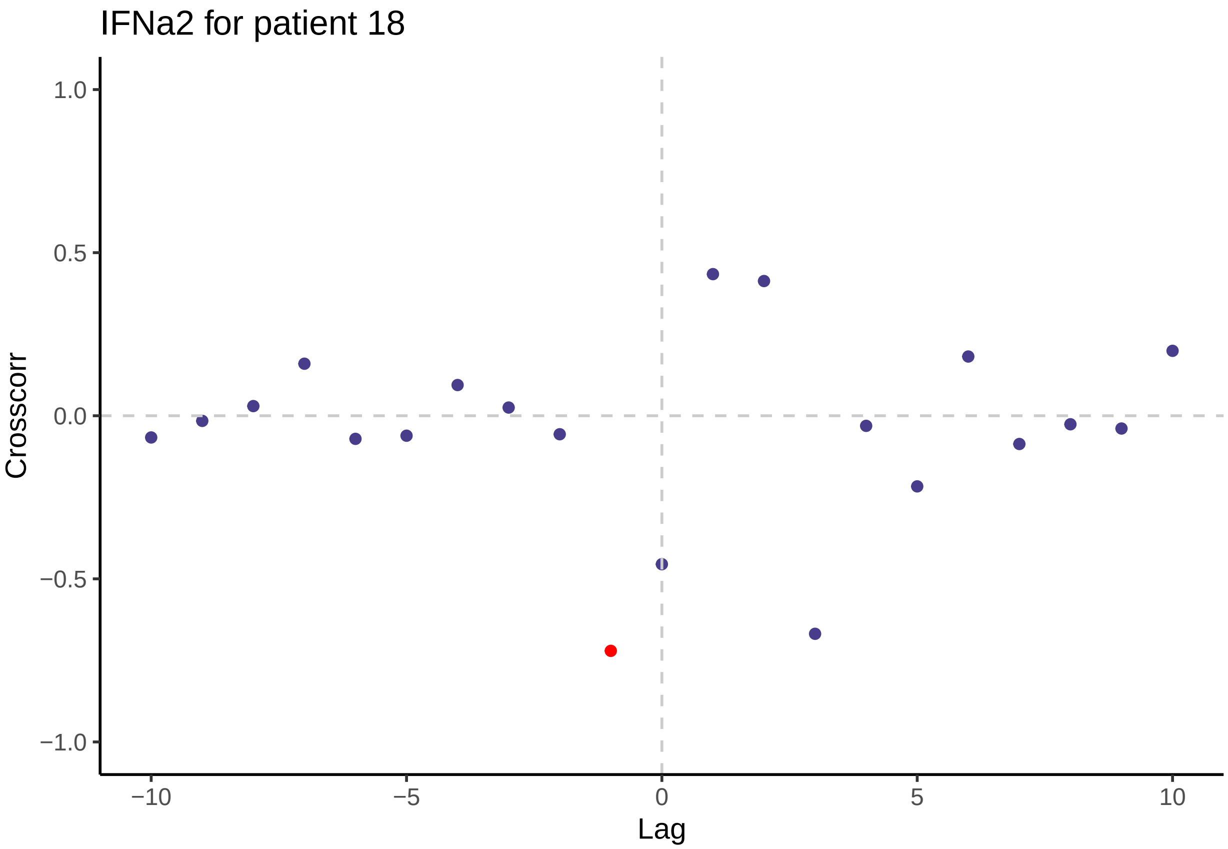


```
## [1] "IFNa2 for patient 18 - p-value: 0.468664696952532"
```

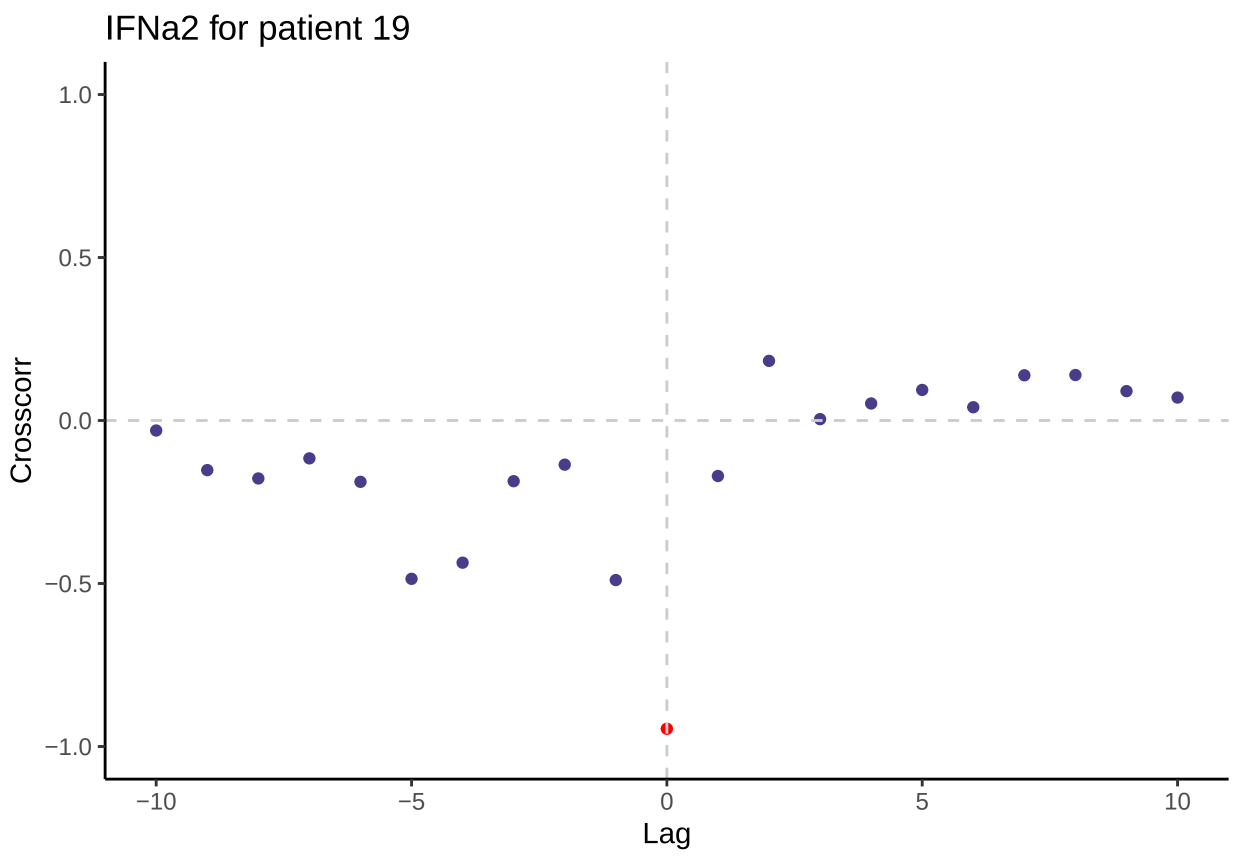


```
## [1] "IFNa2 for patient 19 - p-value: 0.0436448019707661"
```

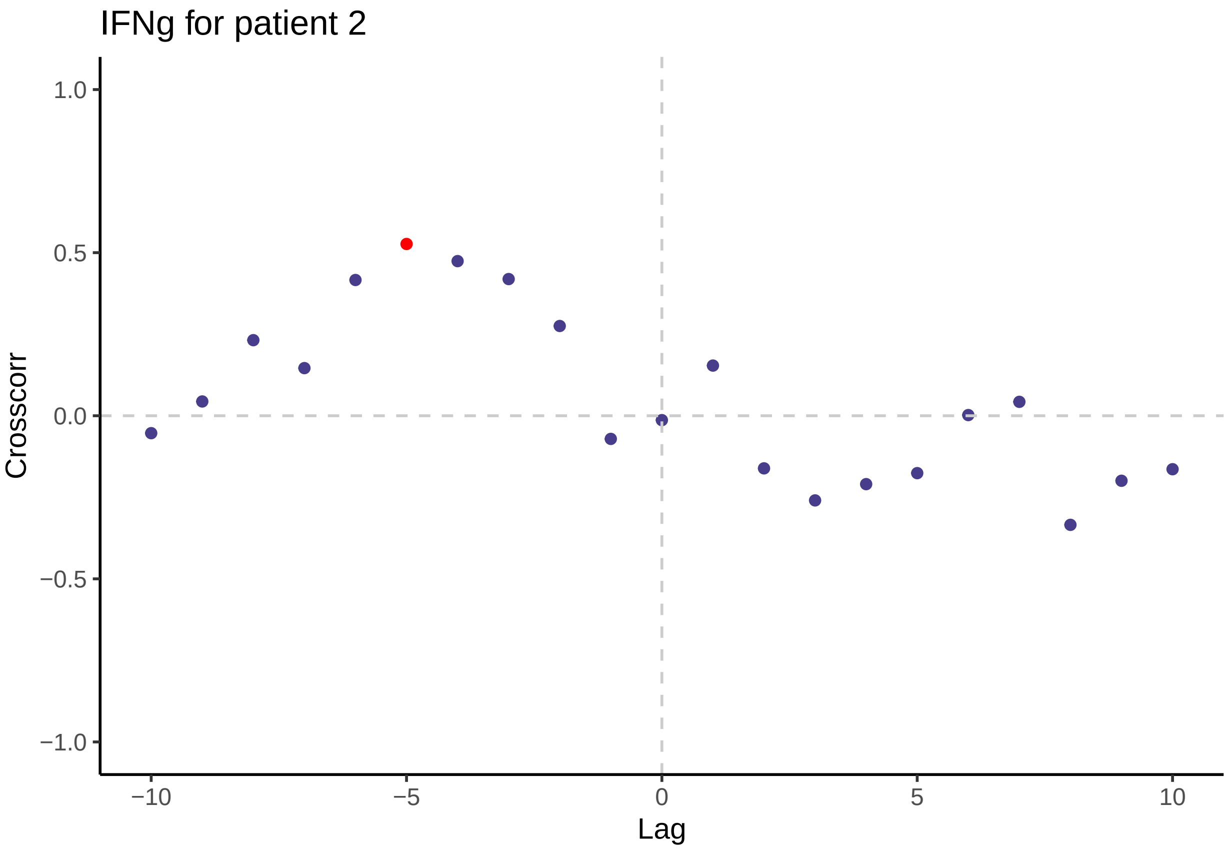


```
## [1] "IFNg for patient 2 - p-value: 0.366925084597668"
```

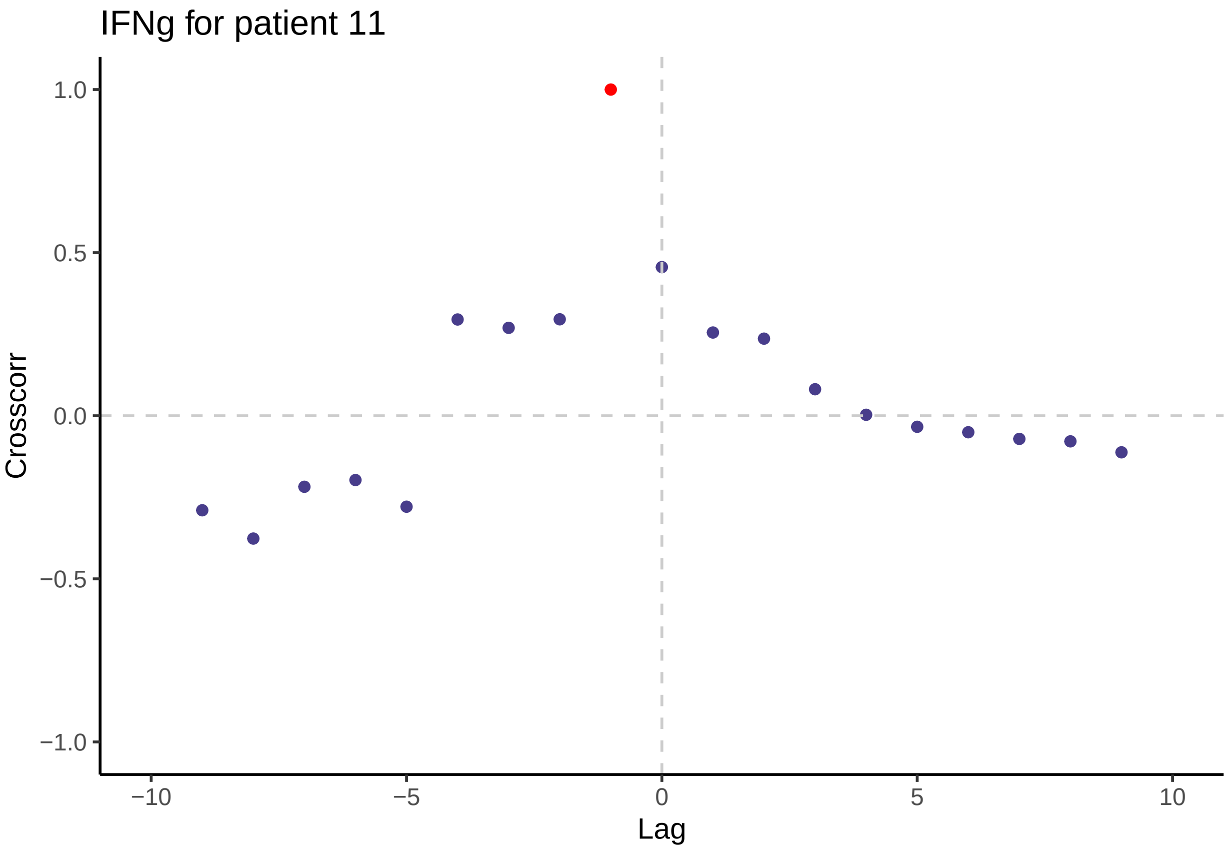


```
## [1] "IFNg for patient 11 - p-value: 0.416313912901634"
```

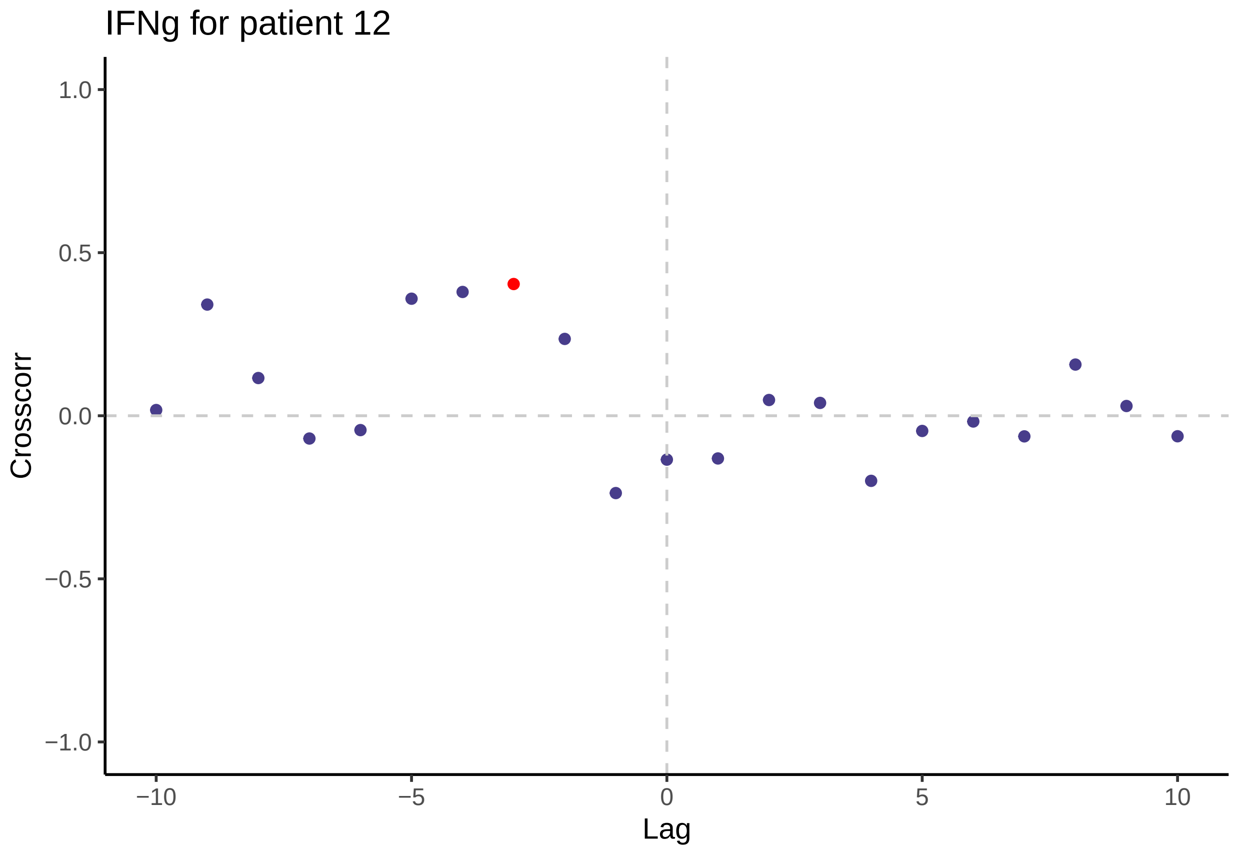


```
## [1] "IFNg for patient 12 - p-value: 0.218132892218271"
```

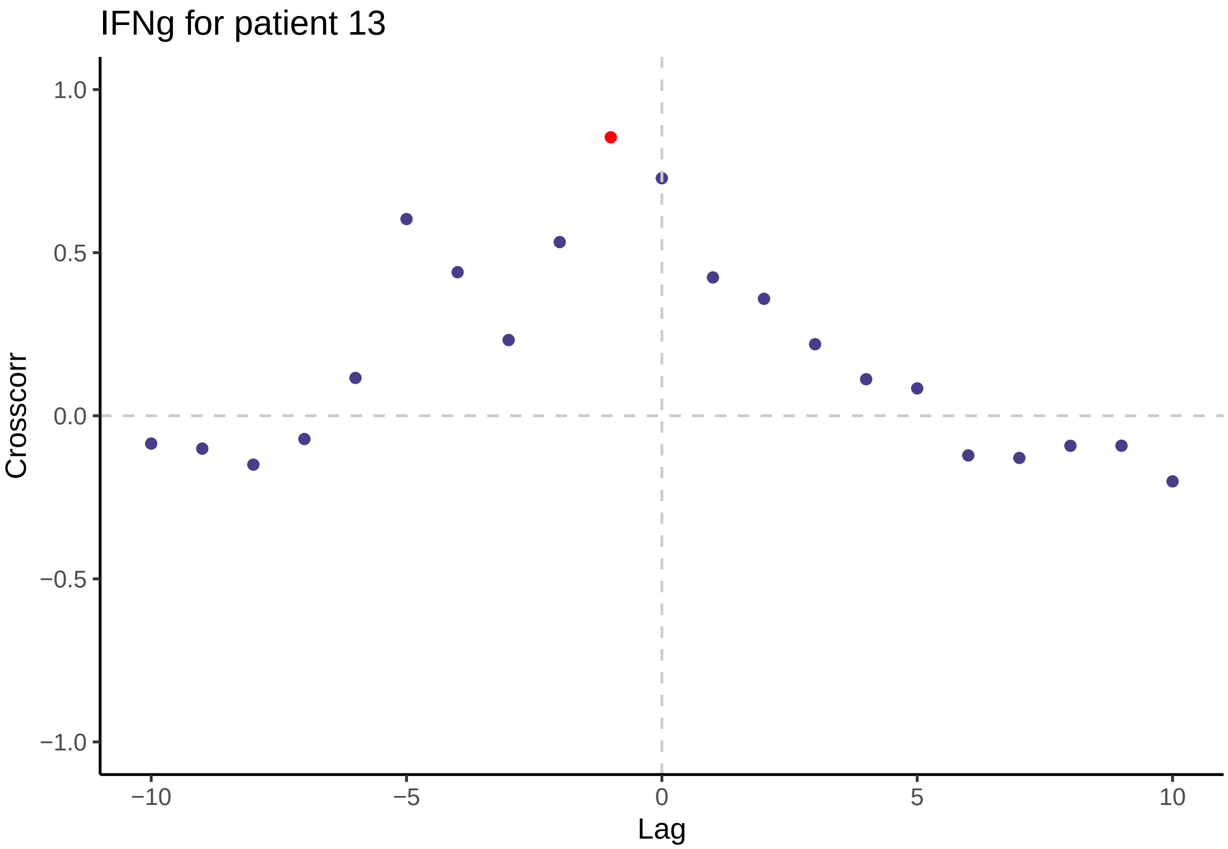


```
## [1] "IFNg for patient 13 - p-value: 0.0211734829988823"
```

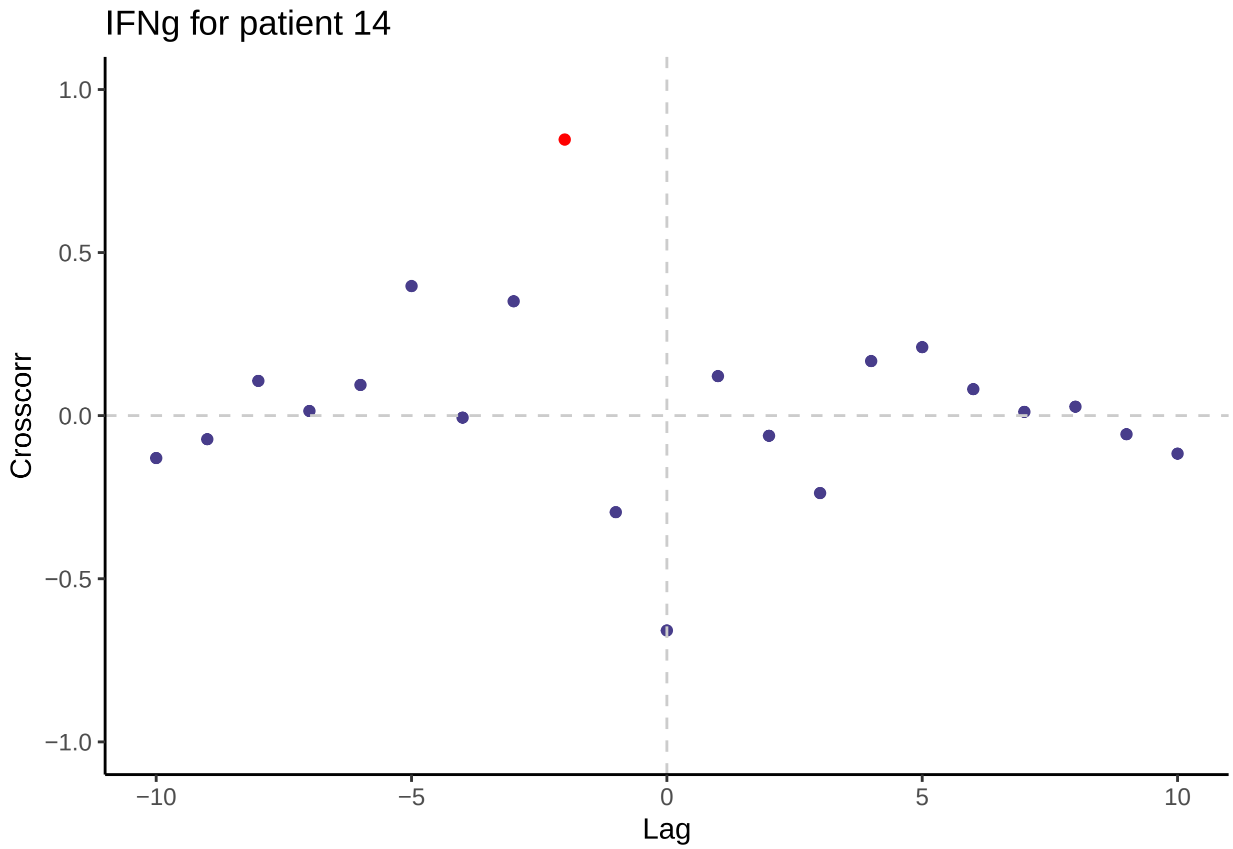


```
## [1] "IFNg for patient 14 - p-value: 0.556808366970071"
```

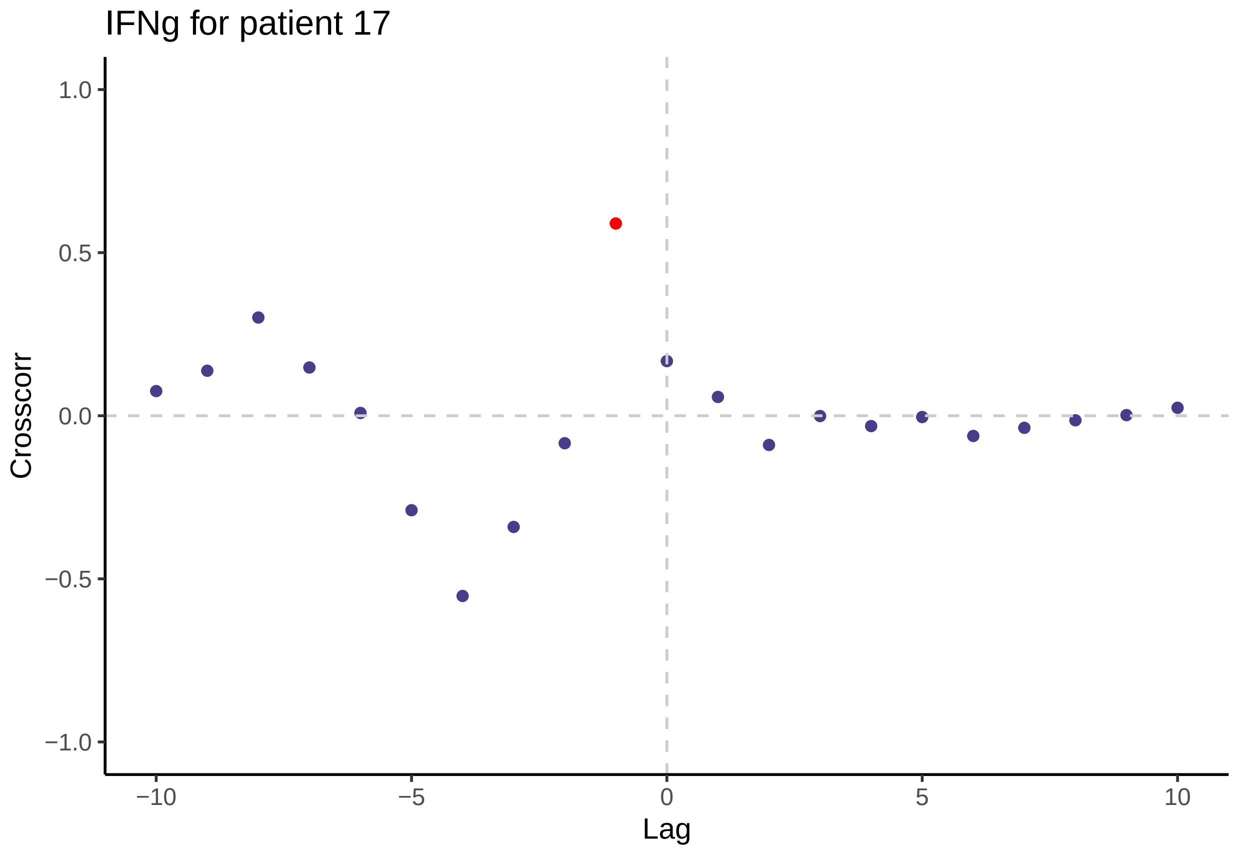


```
## [1] "IFNg for patient 17 - p-value: 0.995296591016922"
```

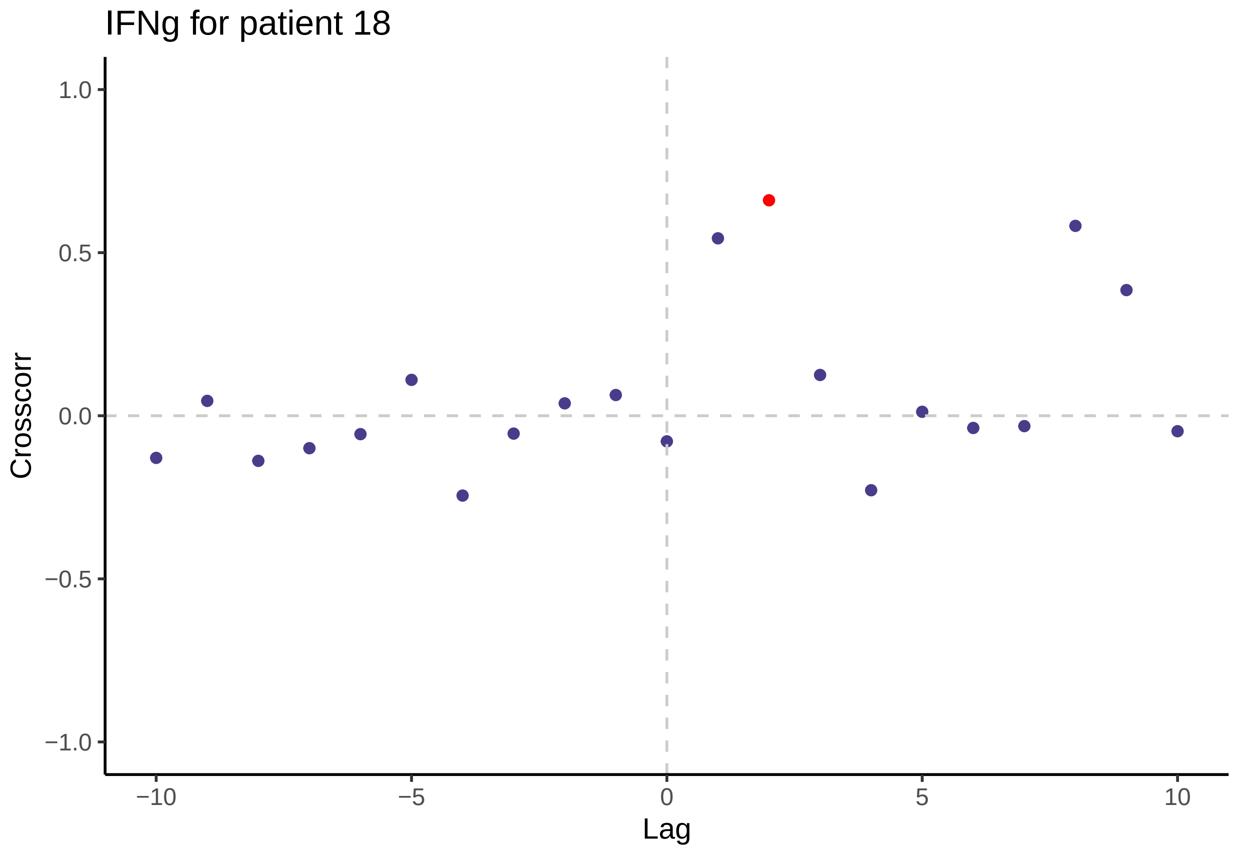


```
## [1] "IFNg for patient 18 - p-value: 0.244055318153031"
```

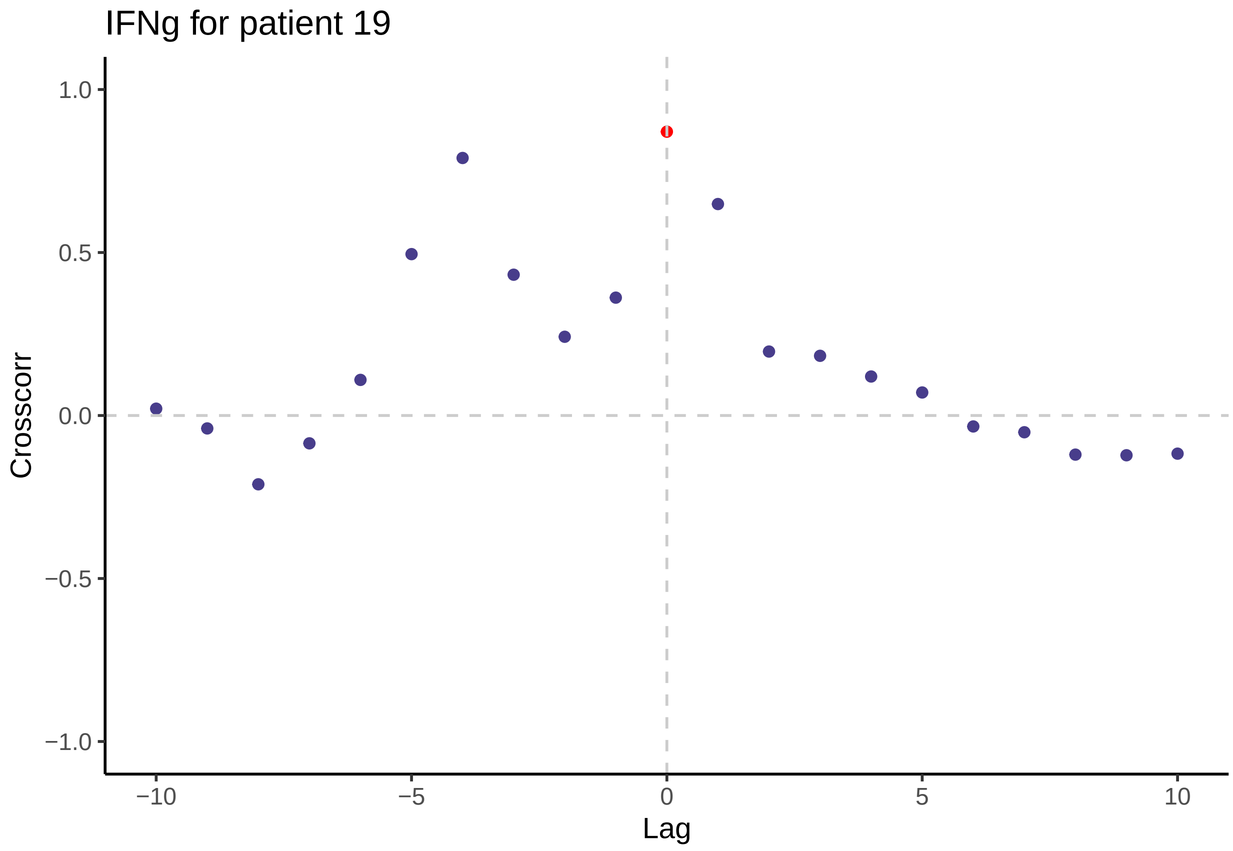


```
## [1] "IFNg for patient 19 - p-value: 0.0162440545108279"
## Warning: Removed 5 rows containing missing values (geom_point).
```

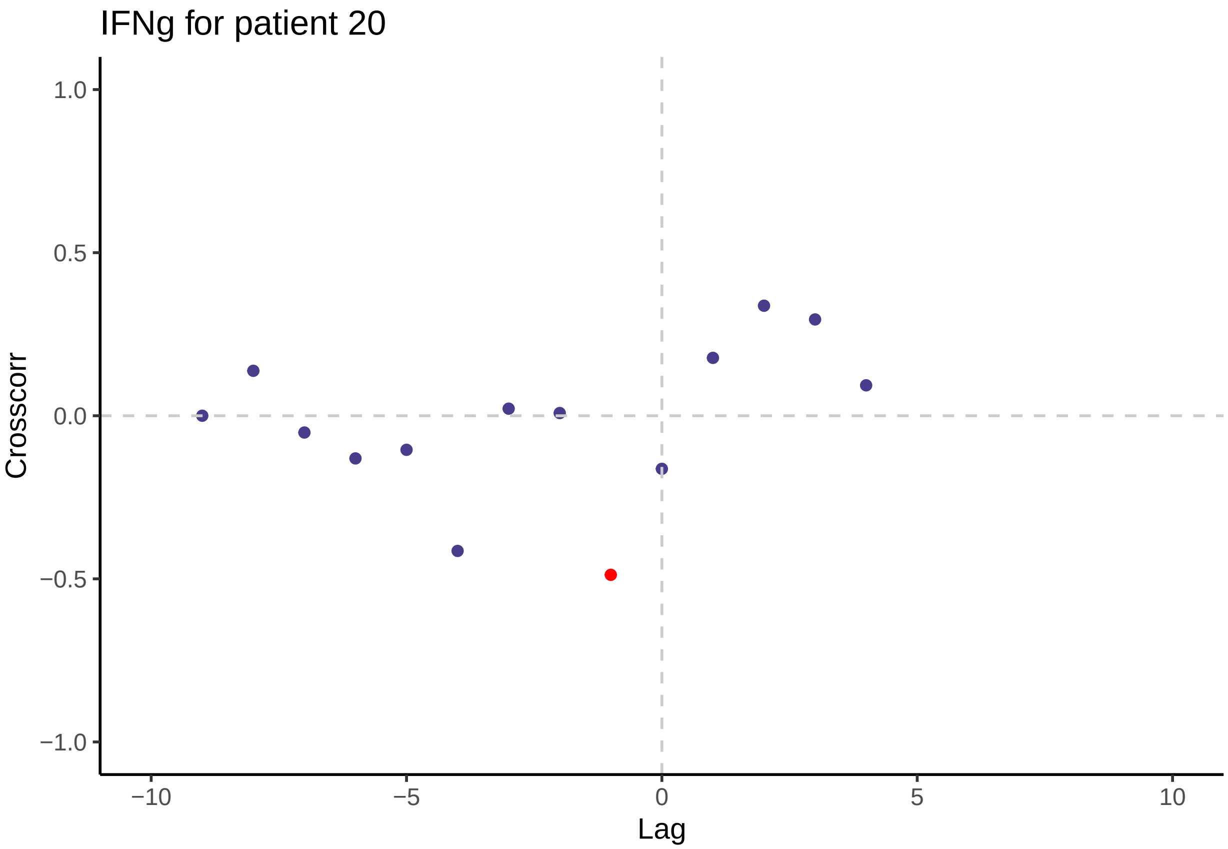


```
## [1] "IFNg for patient 20 - p-value: 0.755815625812887"
```

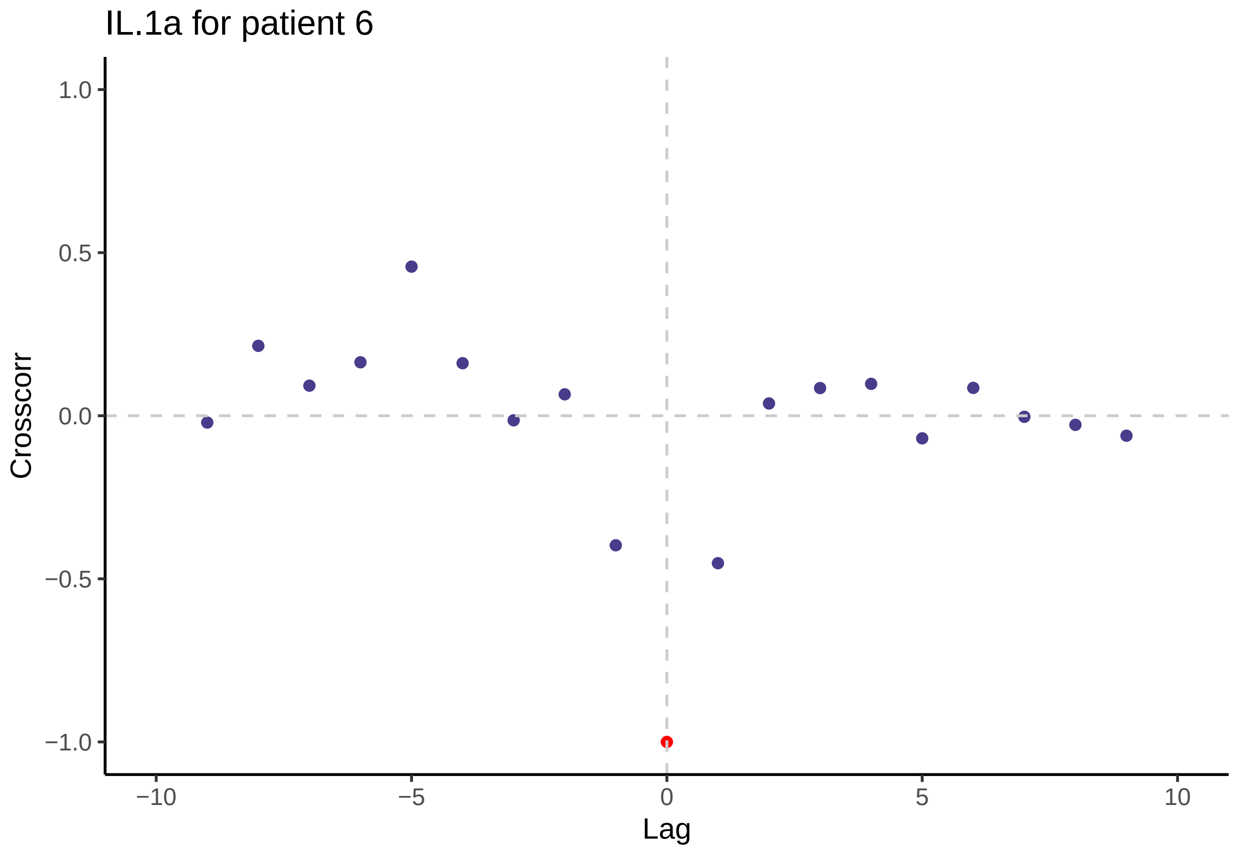


```
## [1] "IL.1a for patient 6 - p-value: 0.667443409074751"
```

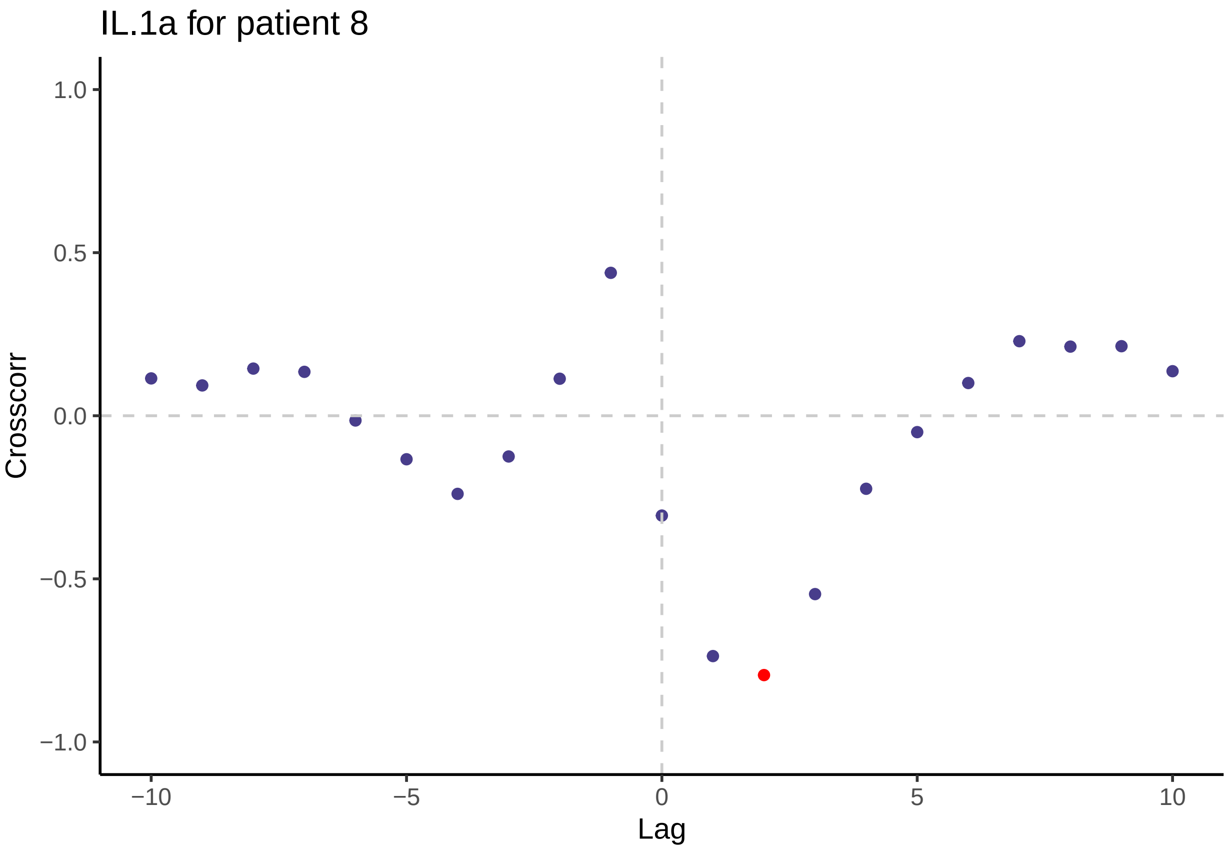


```
## [1] "IL.1a for patient 8 - p-value: 0.410160319917748"
```

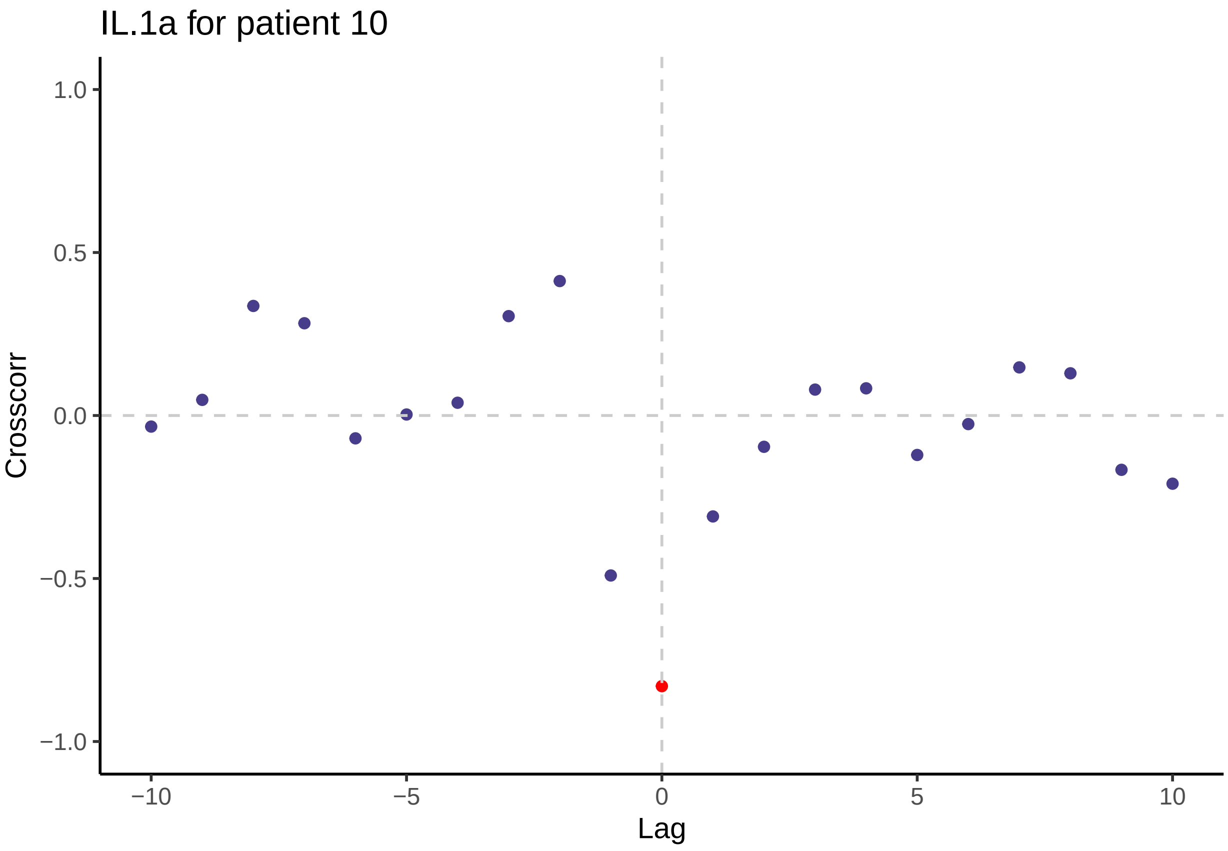


```
## [1] "IL.1a for patient 10 - p-value: 0.713611061100785"
```

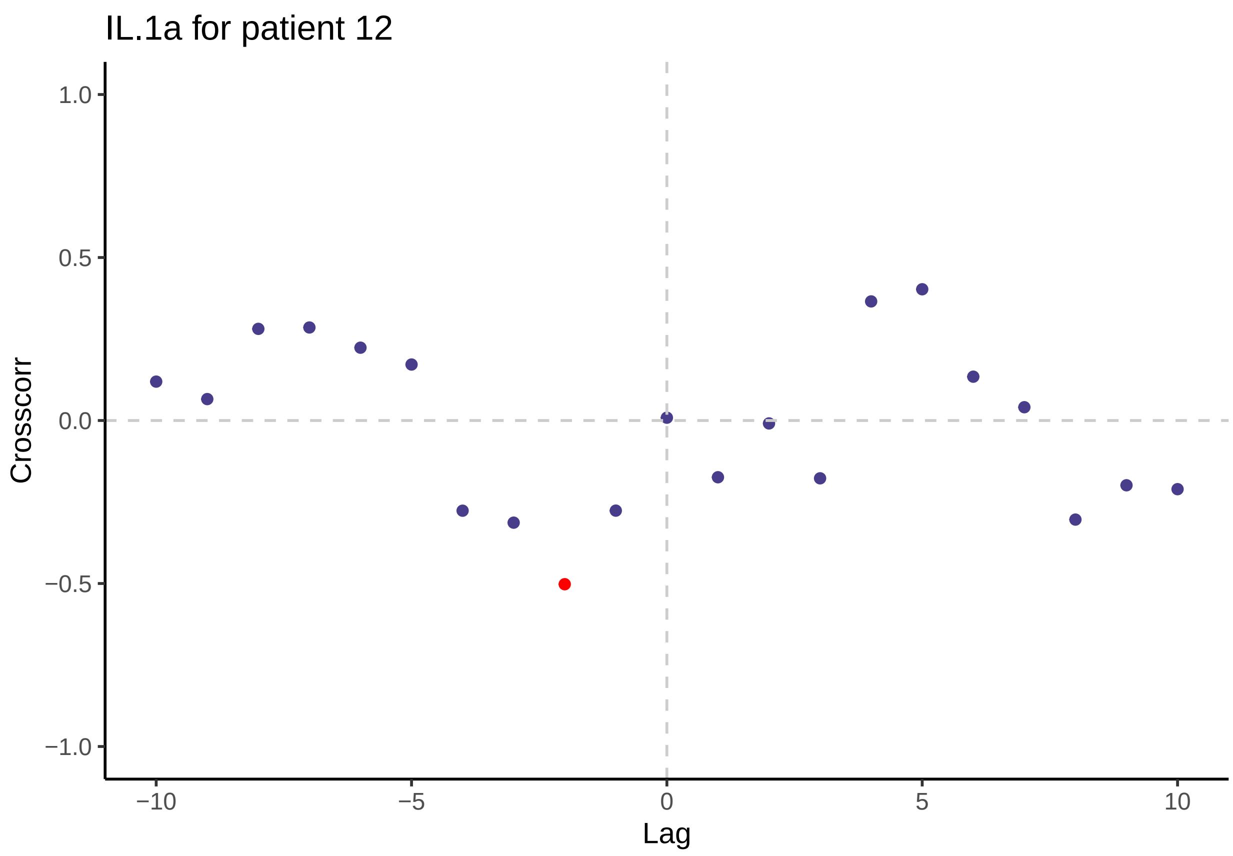


```
## [1] "IL.1a for patient 12 - p-value: 0.772455567689749"
```

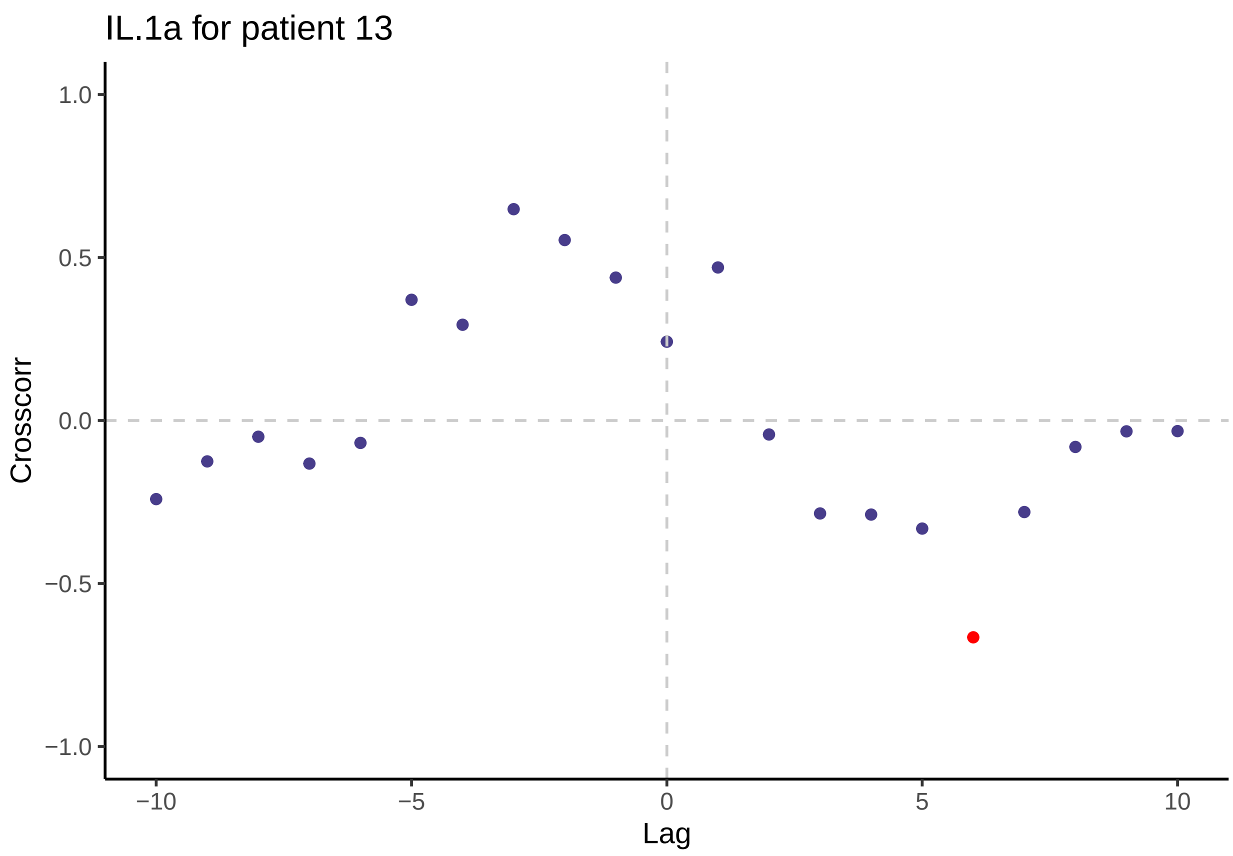


```
## [1] "IL.1a for patient 13 - p-value: 0.821266365469899"
```

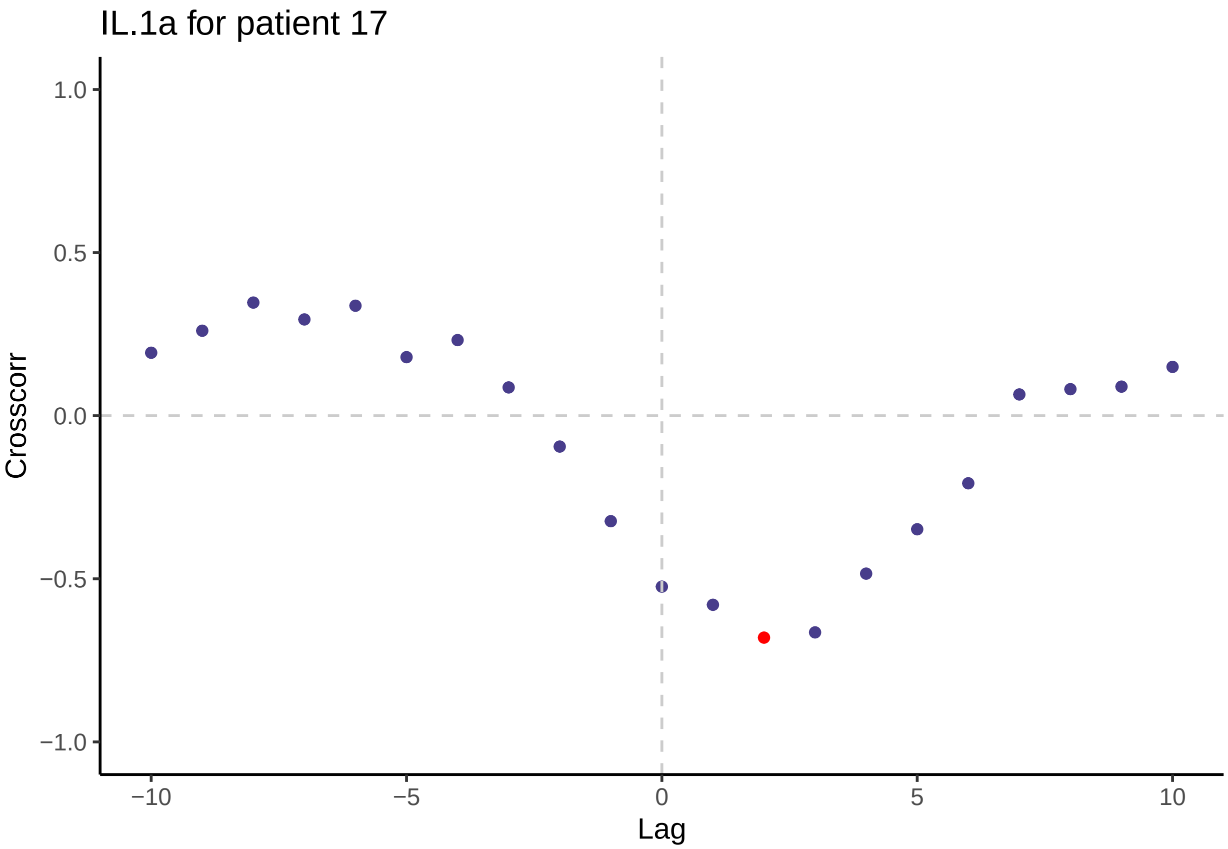


```
## [1] "IL.1a for patient 17 - p-value: 0.336056854960194"
```

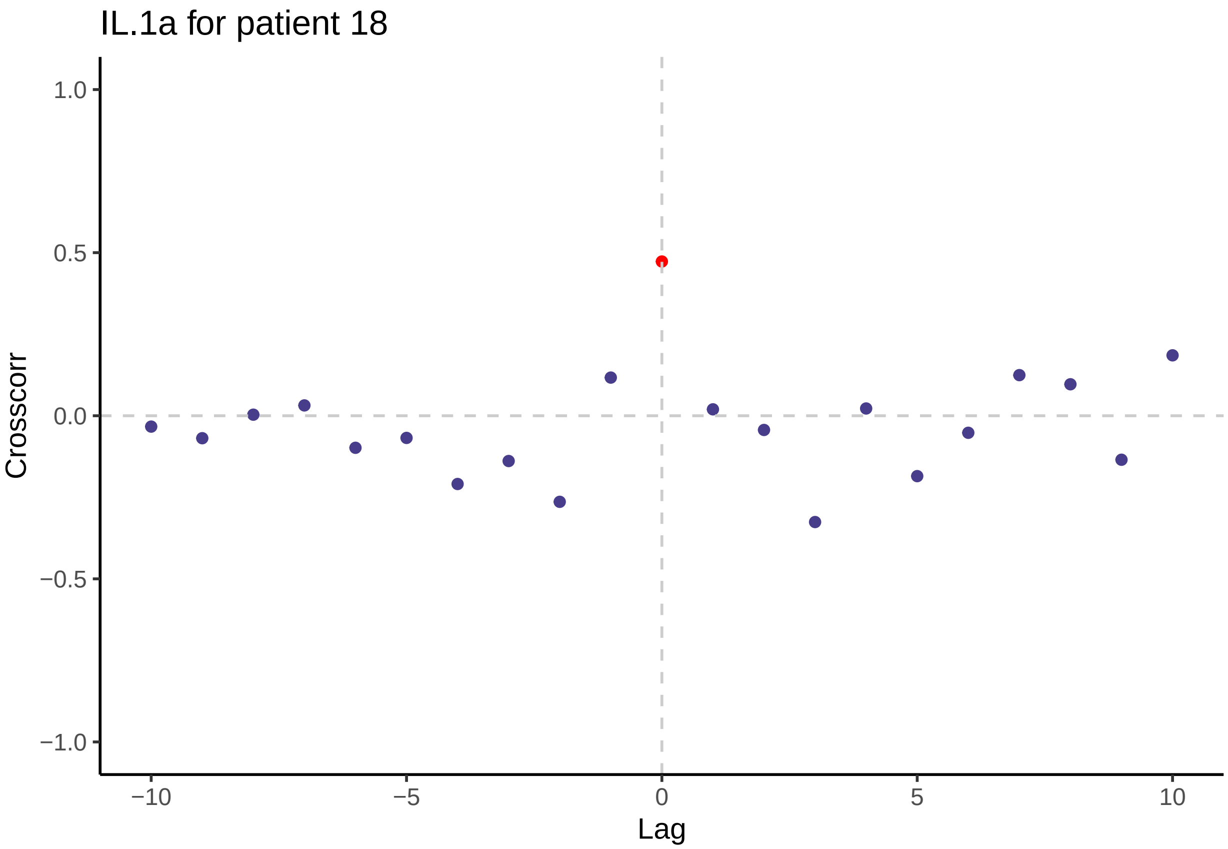


```
## [1] "IL.1a for patient 18 - p-value: 0.497796558040603"
```

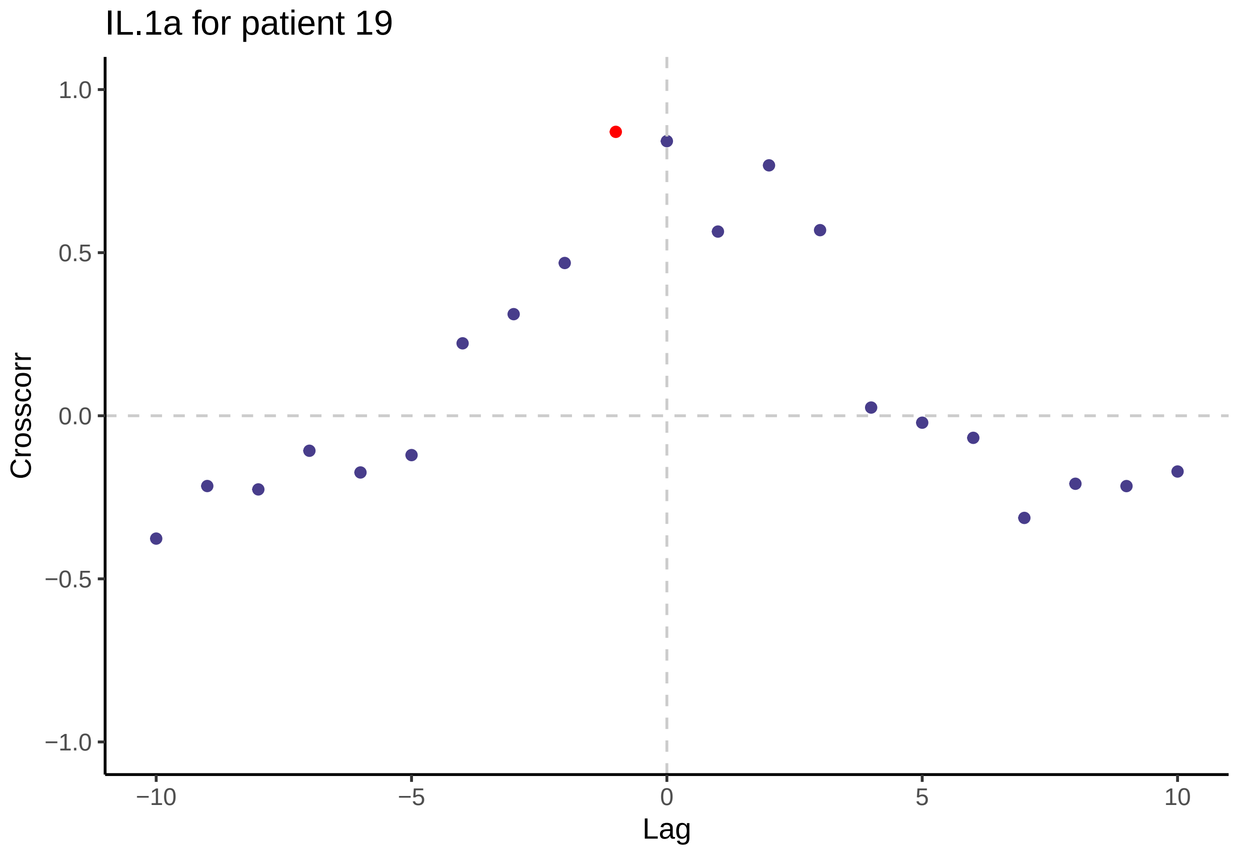


```
## [1] "IL.1a for patient 19 - p-value: 0.207788325972981"
```

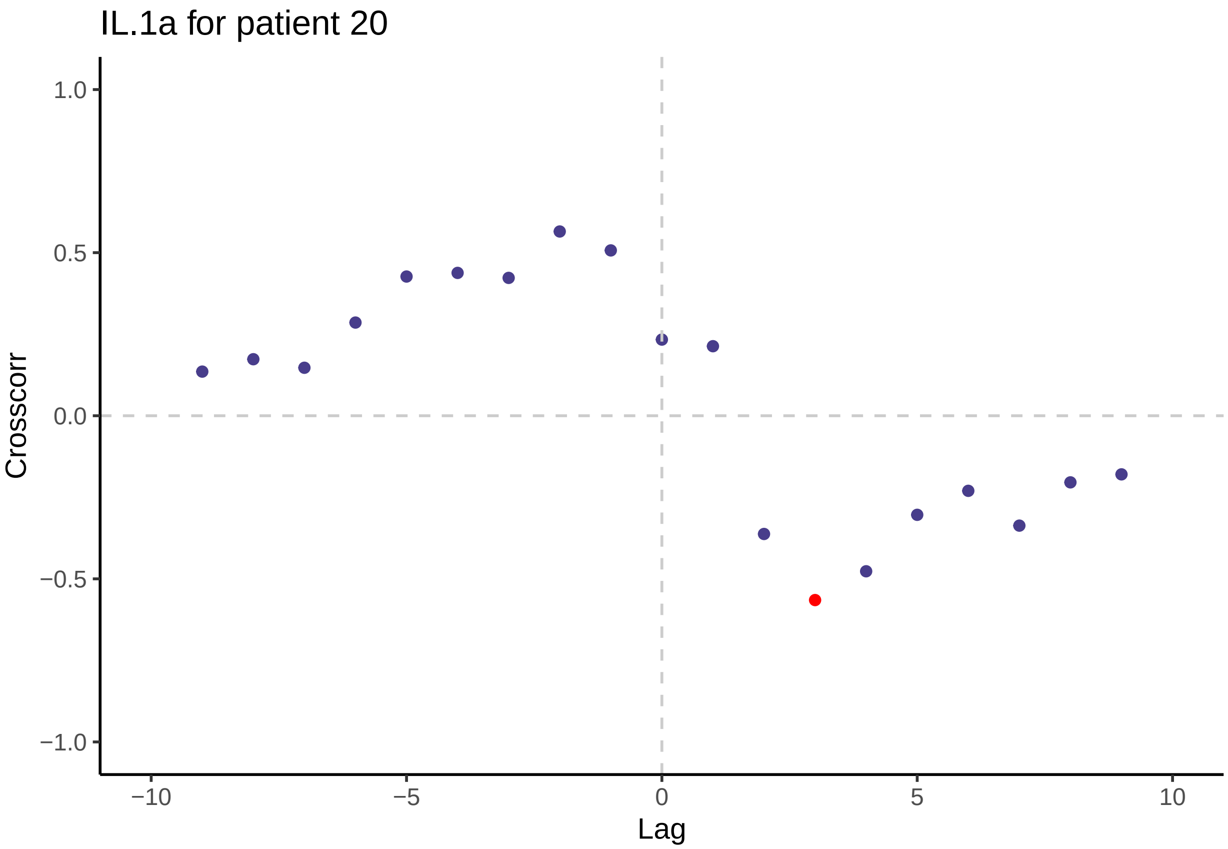


```
## [1] "IL.1a for patient 20 - p-value: 0.579476850788381"
## Warning: Removed 4 rows containing missing values (geom_point).
```

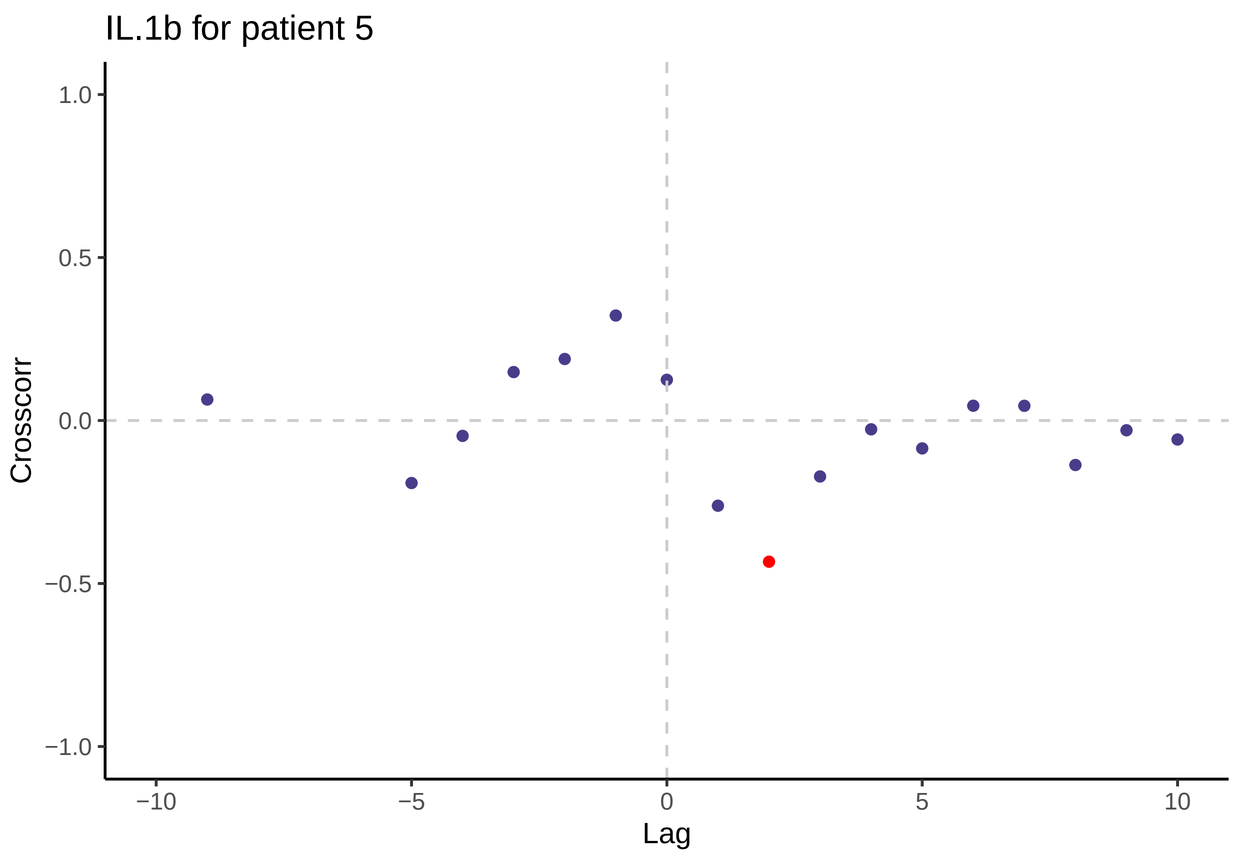


```
## [1] "IL.1b for patient 5 - p-value: 0.510188593098496"
```

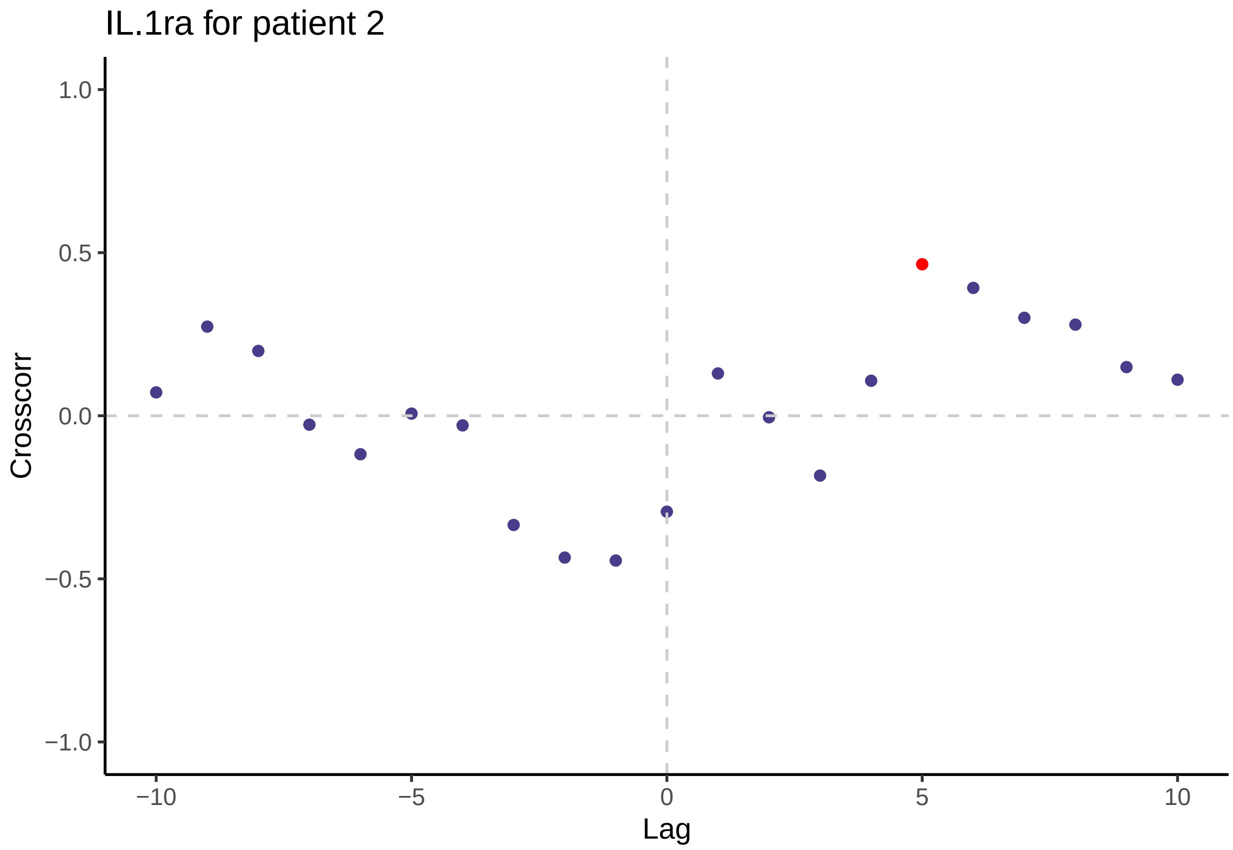


```
## [1] "IL.1ra for patient 2 - p-value: 0.610795270125041"
```

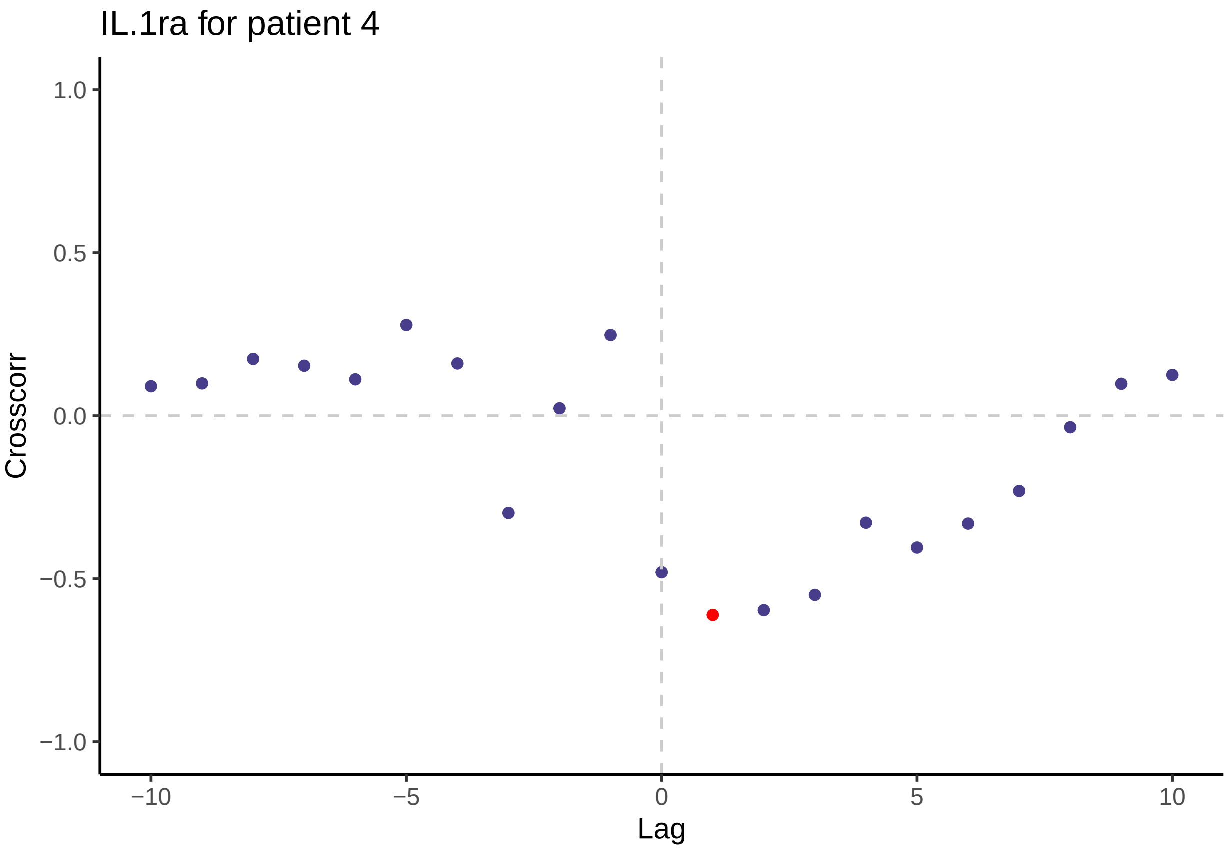


```
## [1] "IL.1ra for patient 4 - p-value: 0.110565872906454"
```

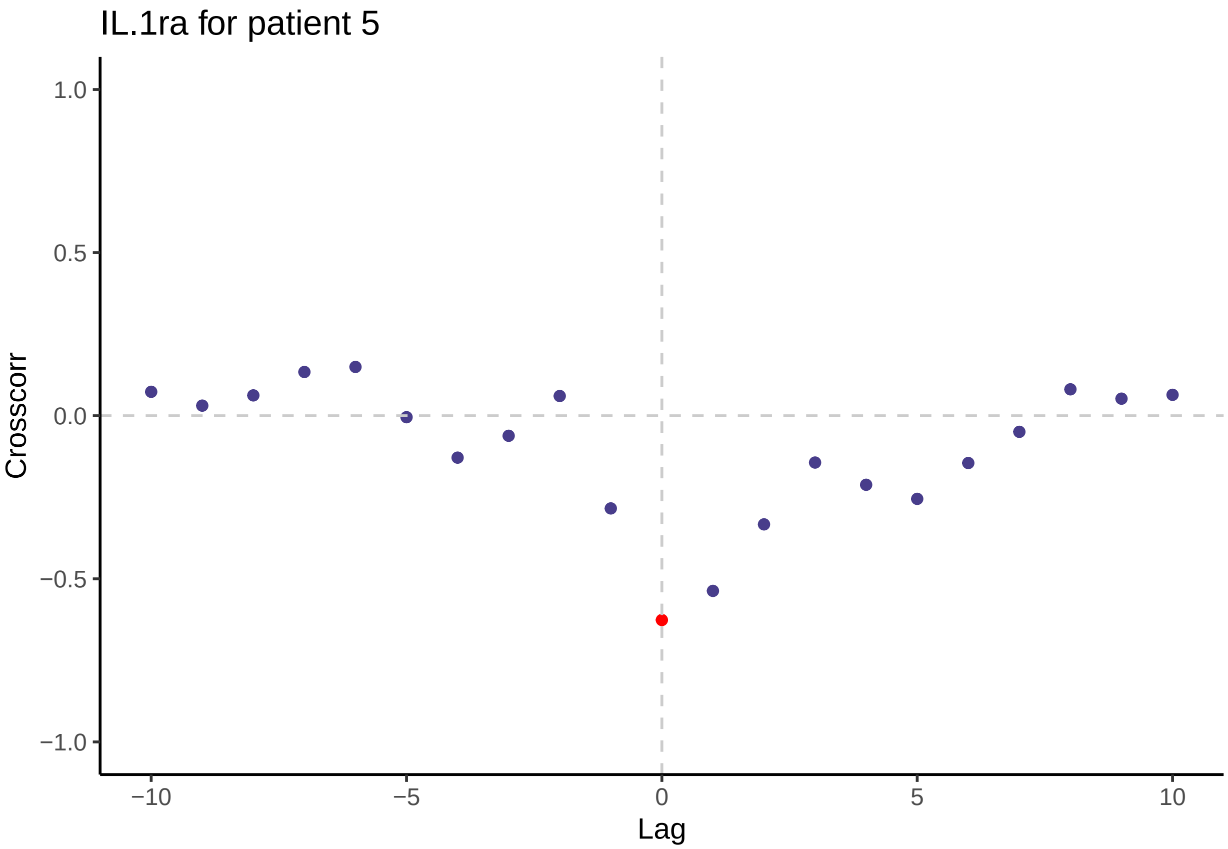


```
## [1] "IL.1ra for patient 5 - p-value: 0.0469472148143756"
```

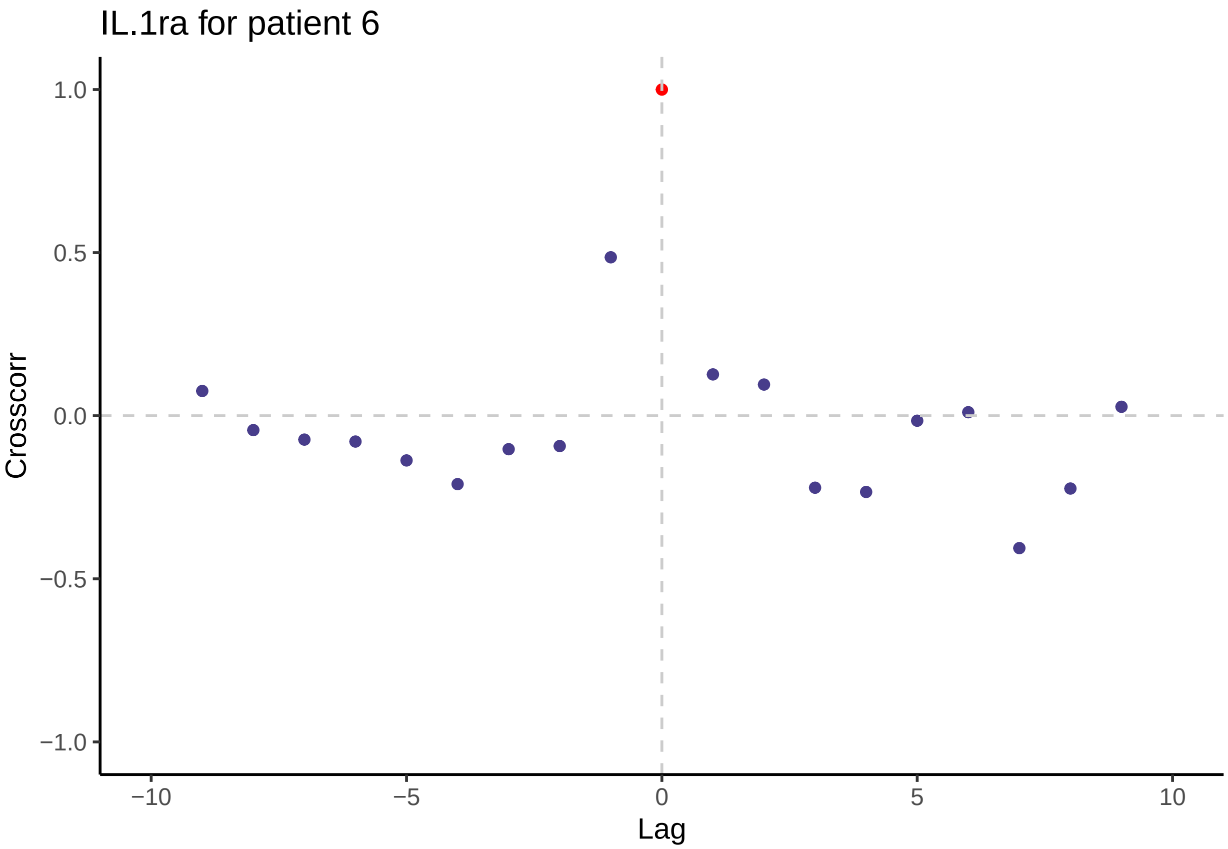


```
## [1] "IL.1ra for patient 6 - p-value: 0.991773127207814"
```

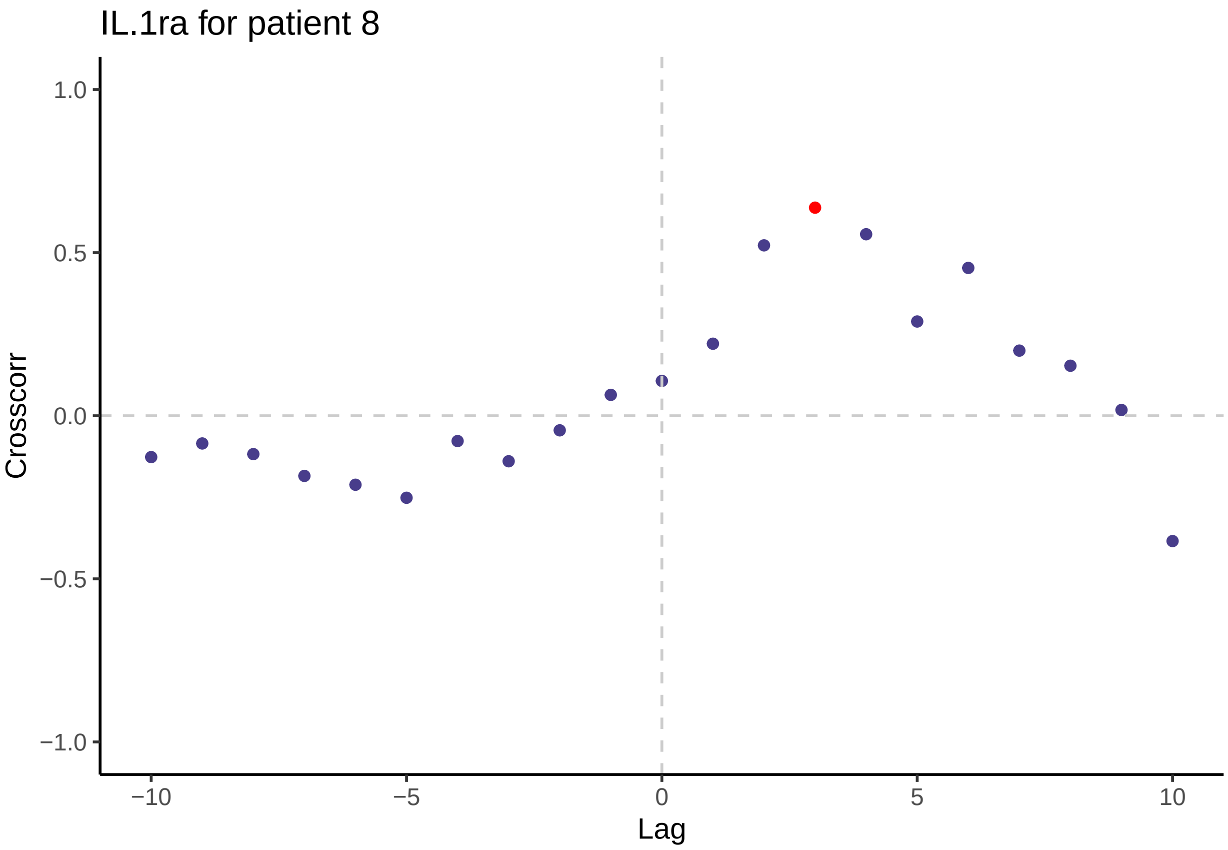


```
## [1] "IL.1ra for patient 8 - p-value: 0.237026849344455"
```

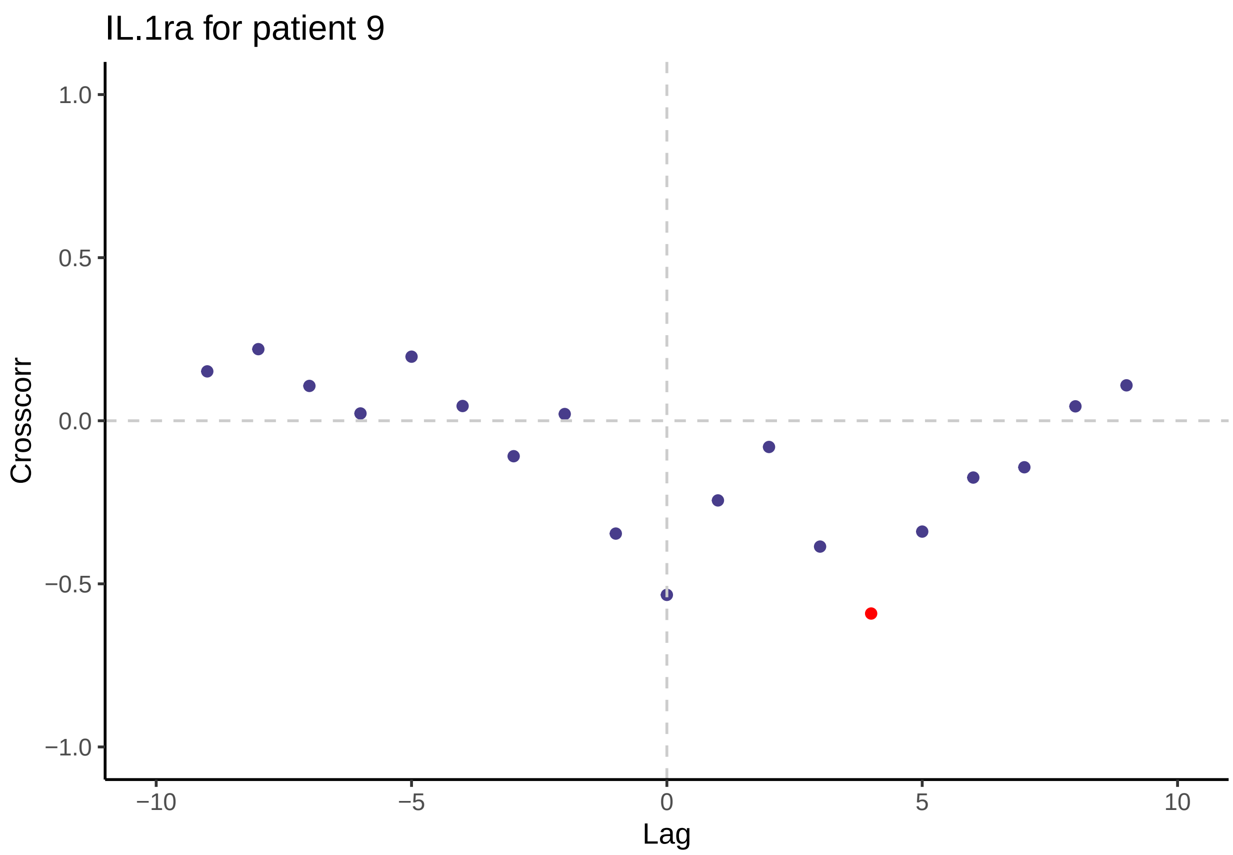


```
## [1] "IL.1ra for patient 9 - p-value: 0.0717173828315279"
```

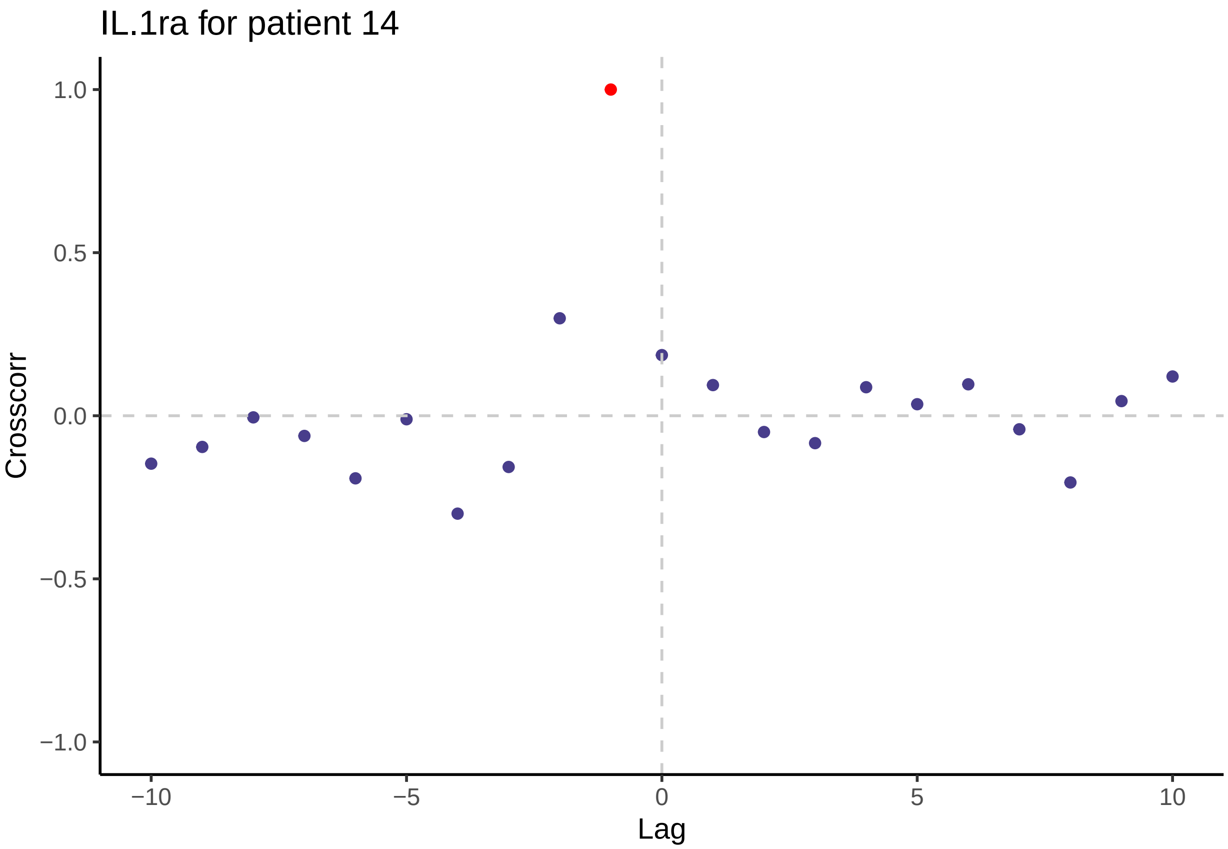


```
## [1] "IL.1ra for patient 14 - p-value: 0.616363286022408"
```

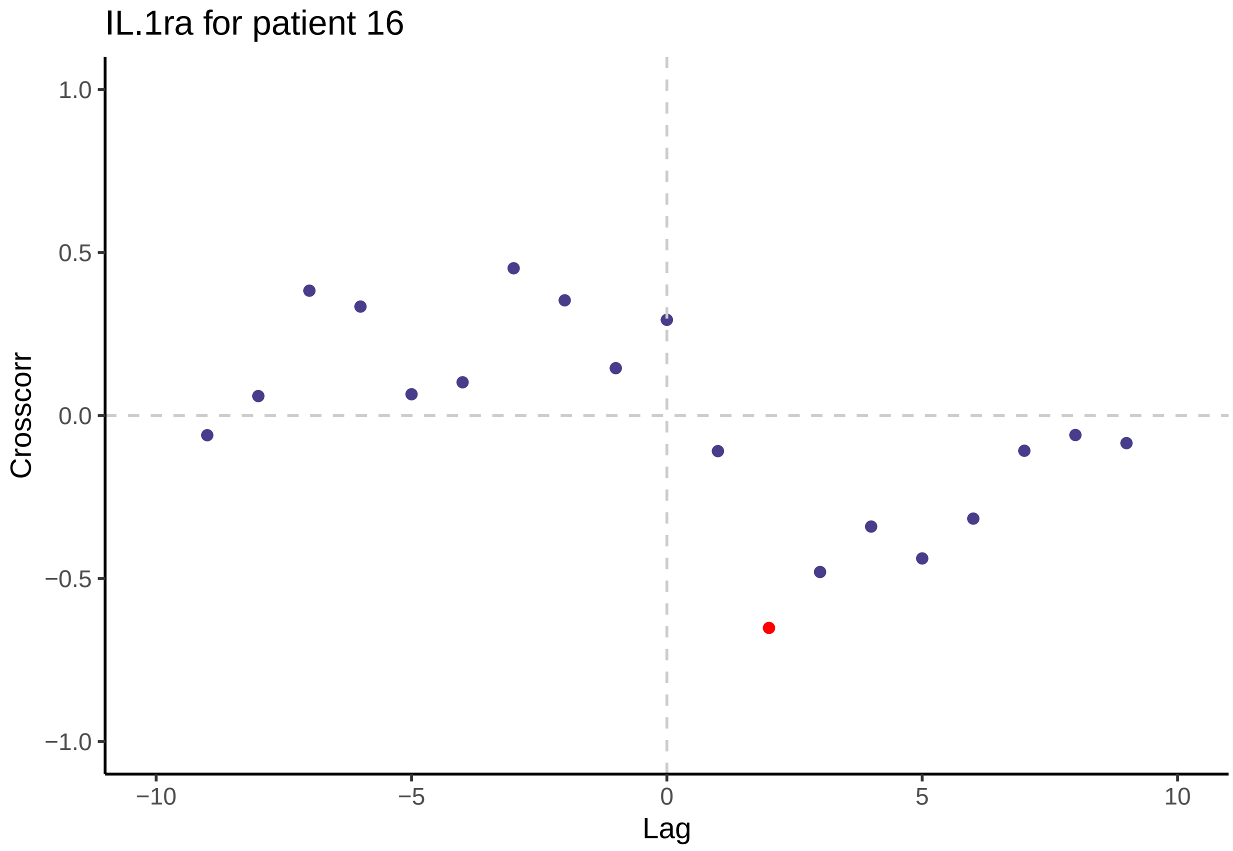


```
## [1] "IL.1ra for patient 16 - p-value: 0.741693366840727"
```

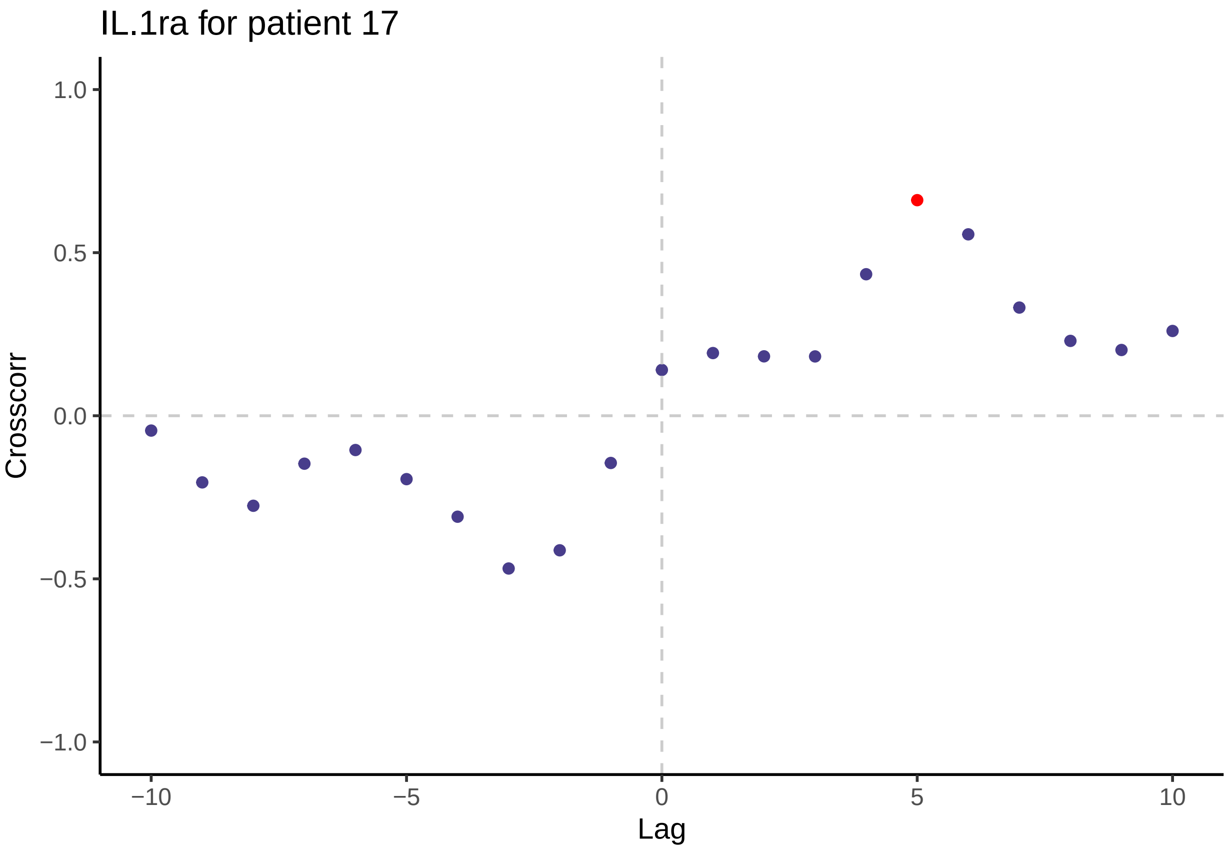


```
## [1] "IL.1ra for patient 17 - p-value: 0.46834556768805"
```

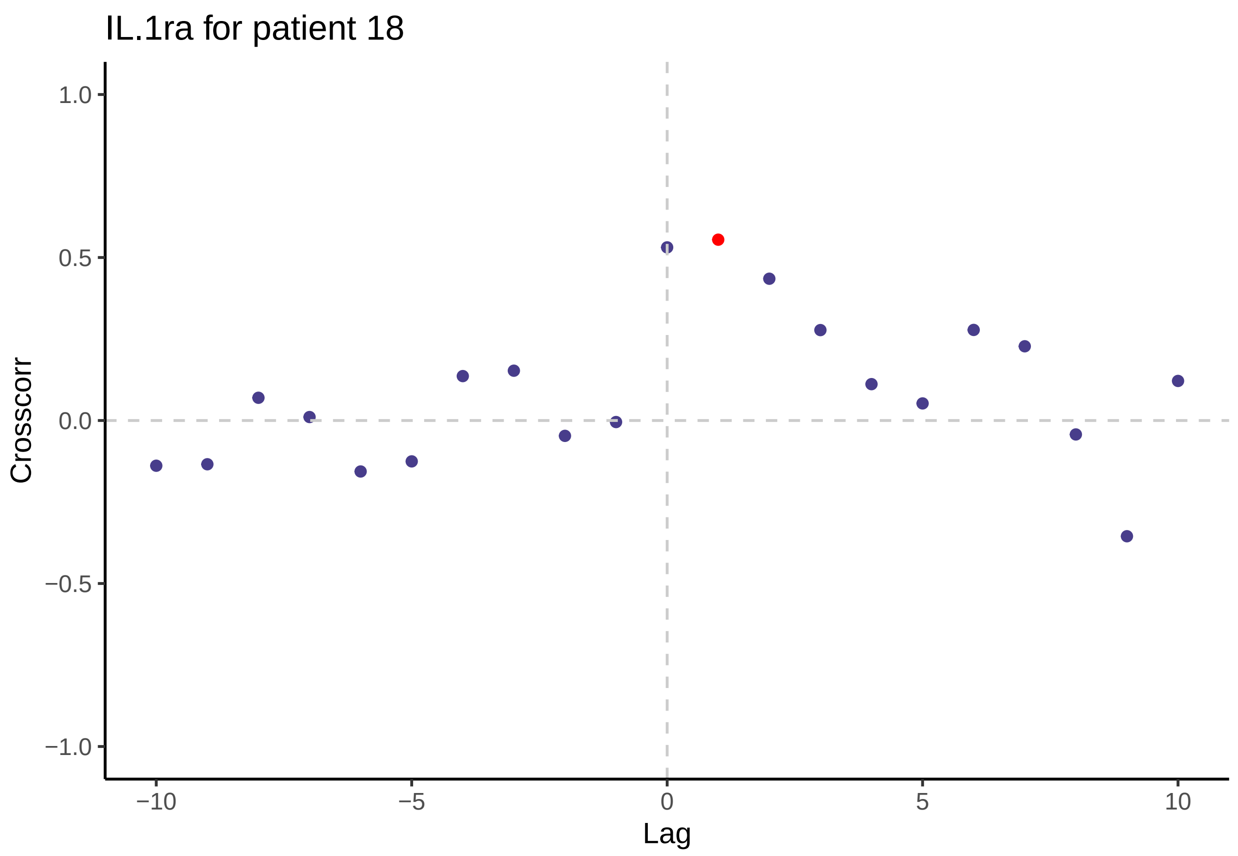


```
## [1] "IL.1ra for patient 18 - p-value: 0.082331804348165"
```

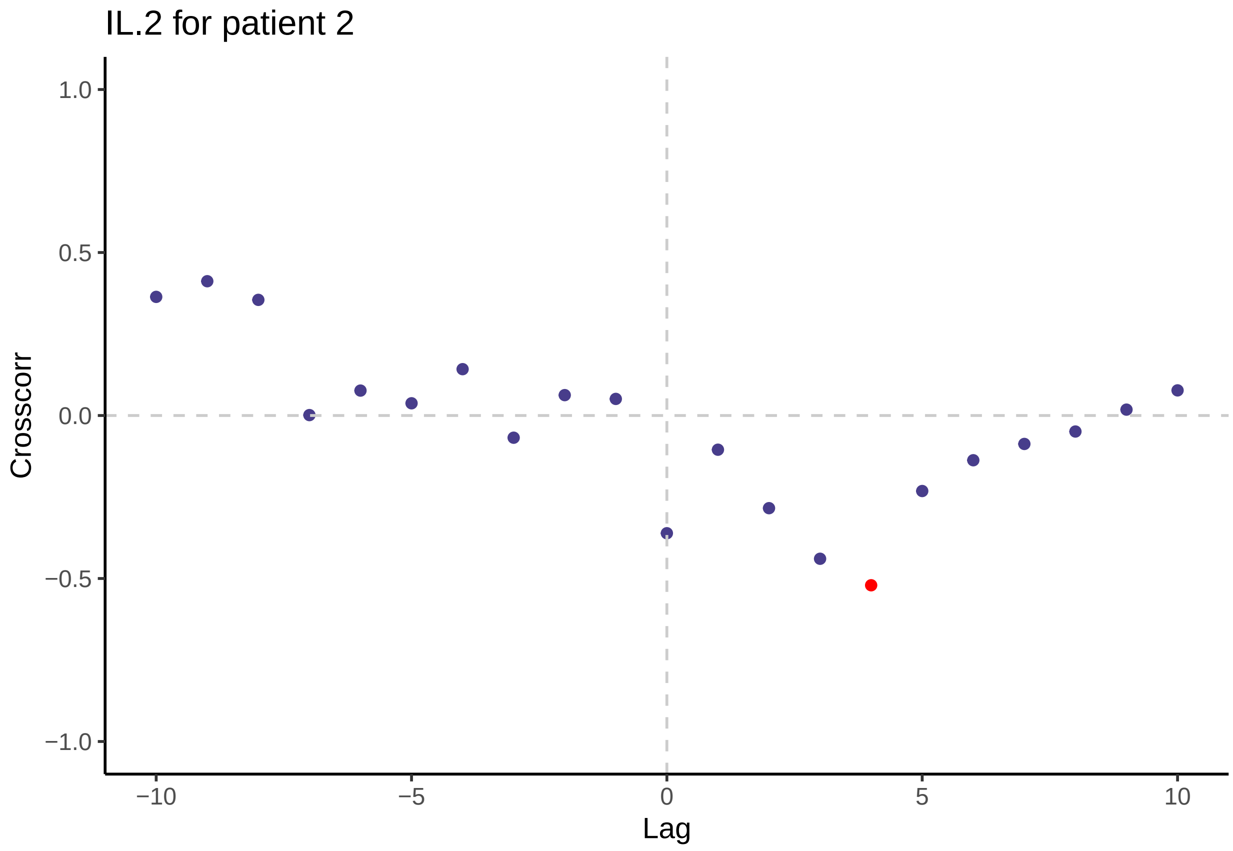


```
## [1] "IL.2 for patient 2 - p-value: 0.550514576215594"
```

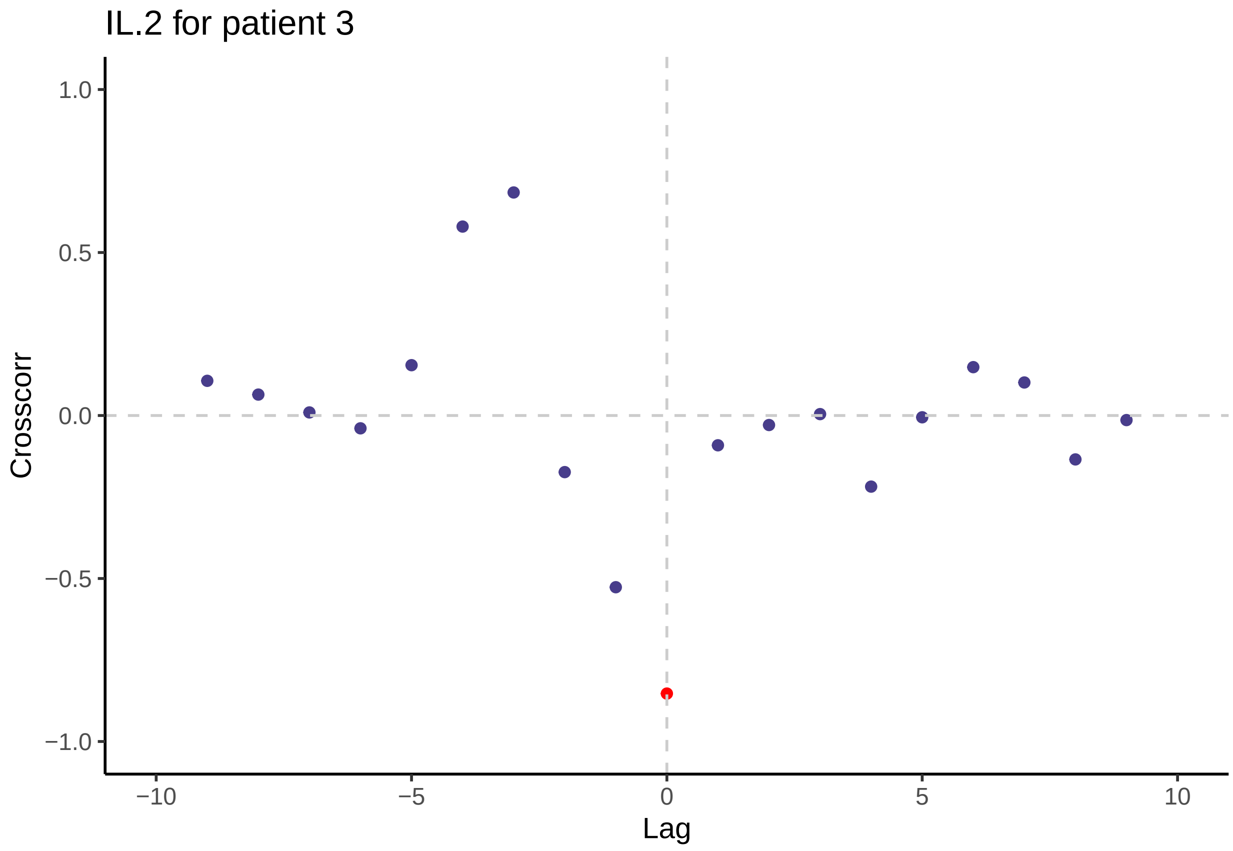


```
## [1] "IL.2 for patient 3 - p-value: 0.873834224504089"
```

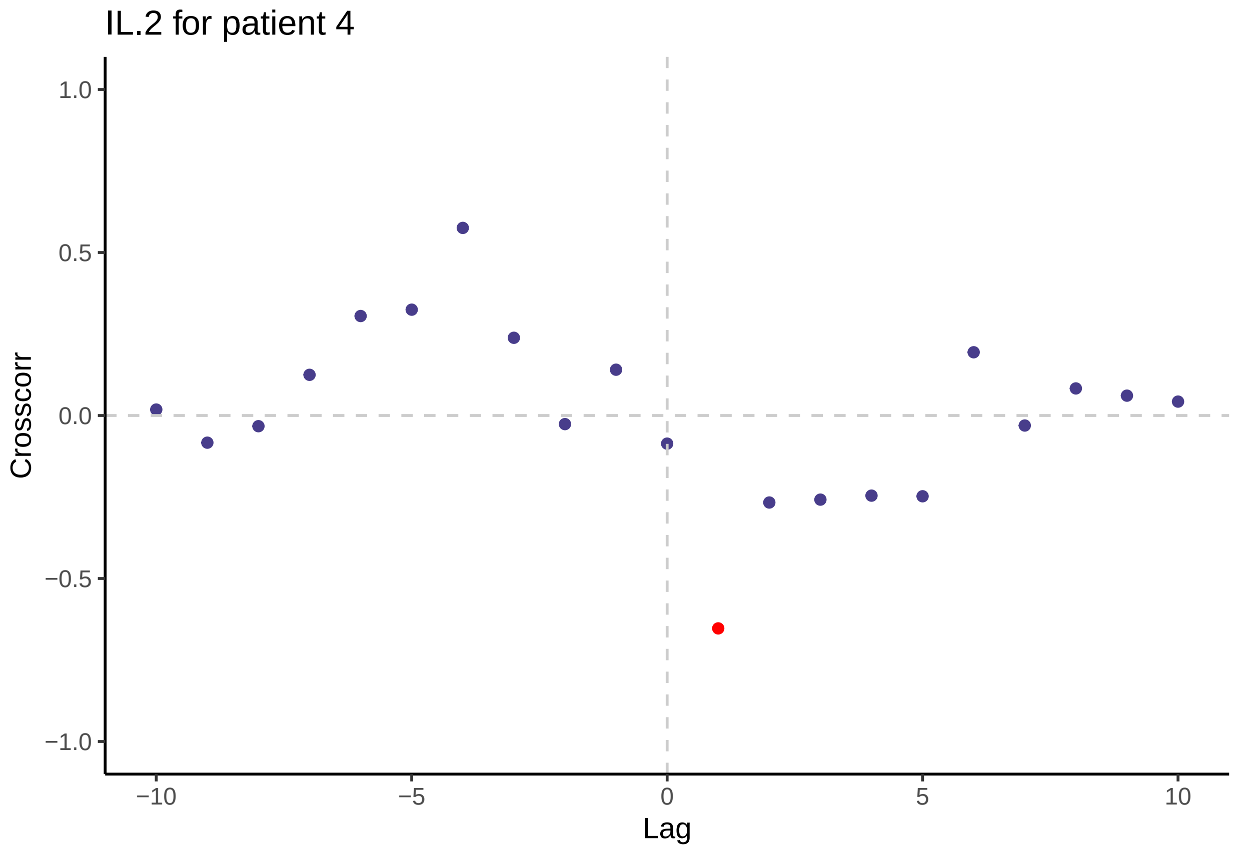


```
## [1] "IL.2 for patient 4 - p-value: 0.88415733223937"
## Warning: Removed 2 rows containing missing values (geom_point).
```

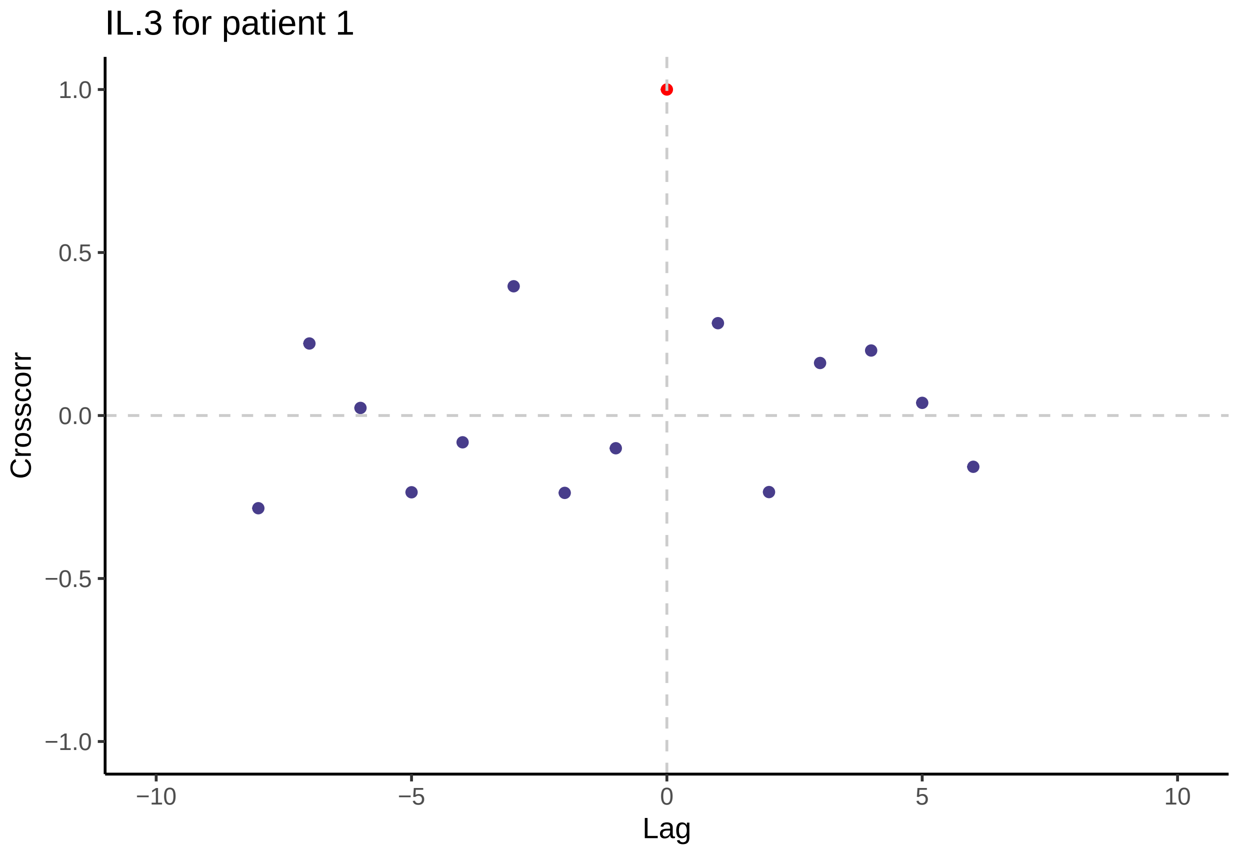


```
## [1] "IL.3 for patient 1 - p-value: 0.458389091989199"
## Warning: Removed 12 rows containing missing values (geom_point).
```

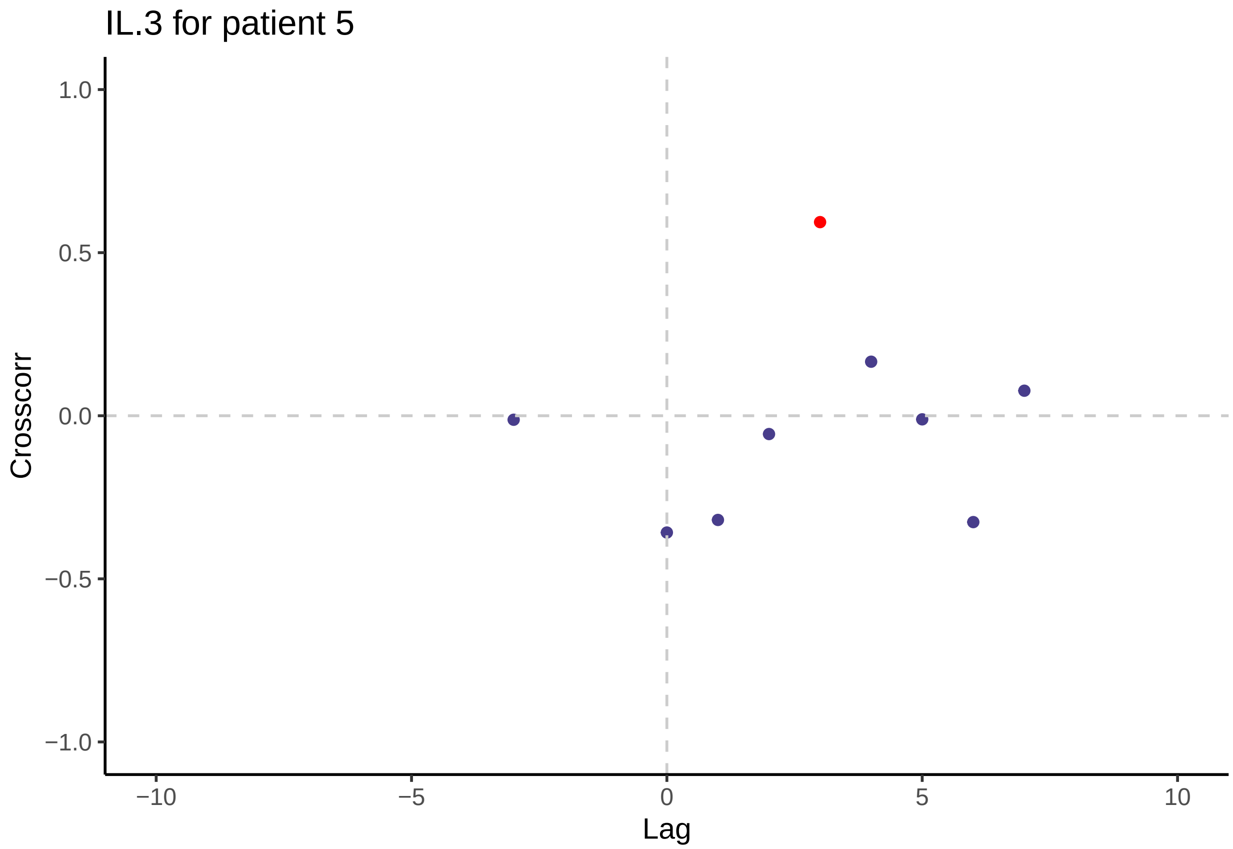


```
## [1] "IL.3 for patient 5 - p-value: 0.790815405473005"
```

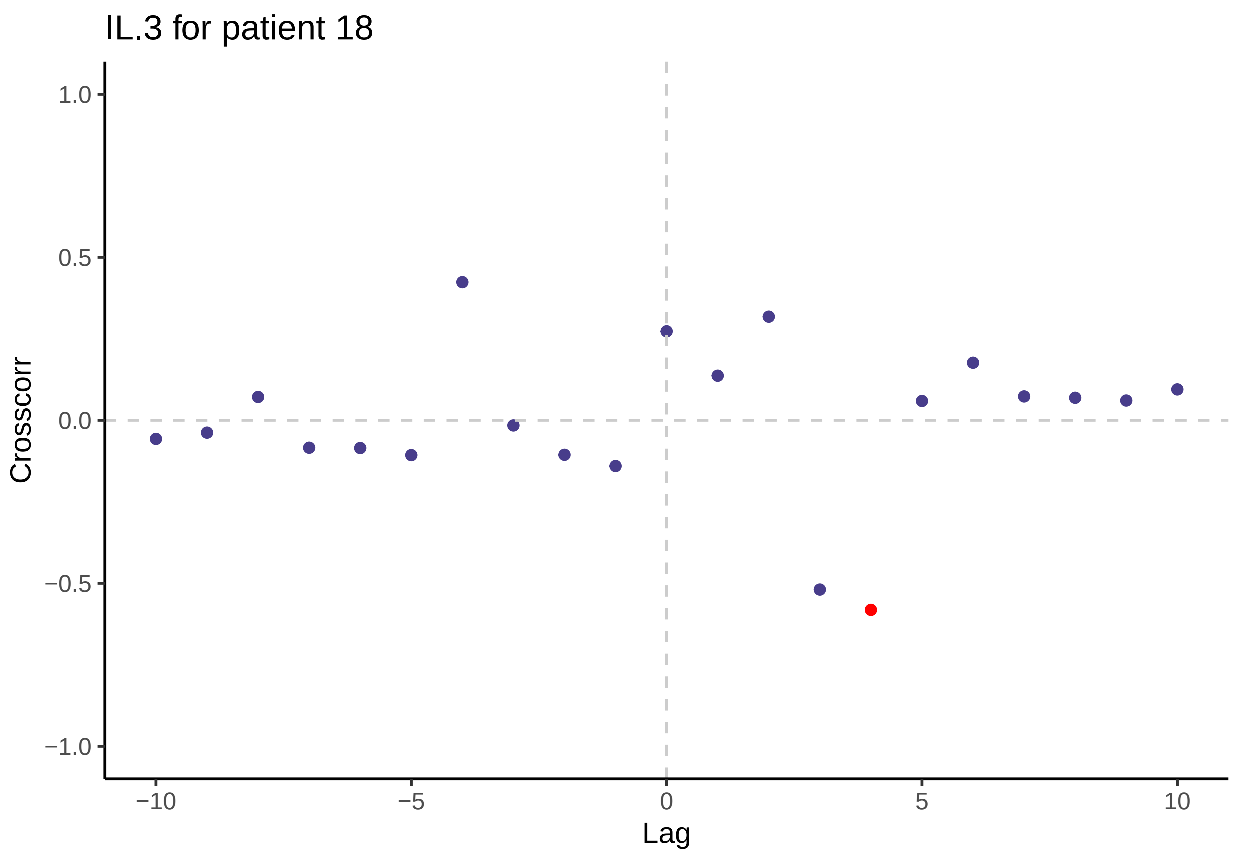


```
## [1] "IL.3 for patient 18 - p-value: 0.98485880203144"
```

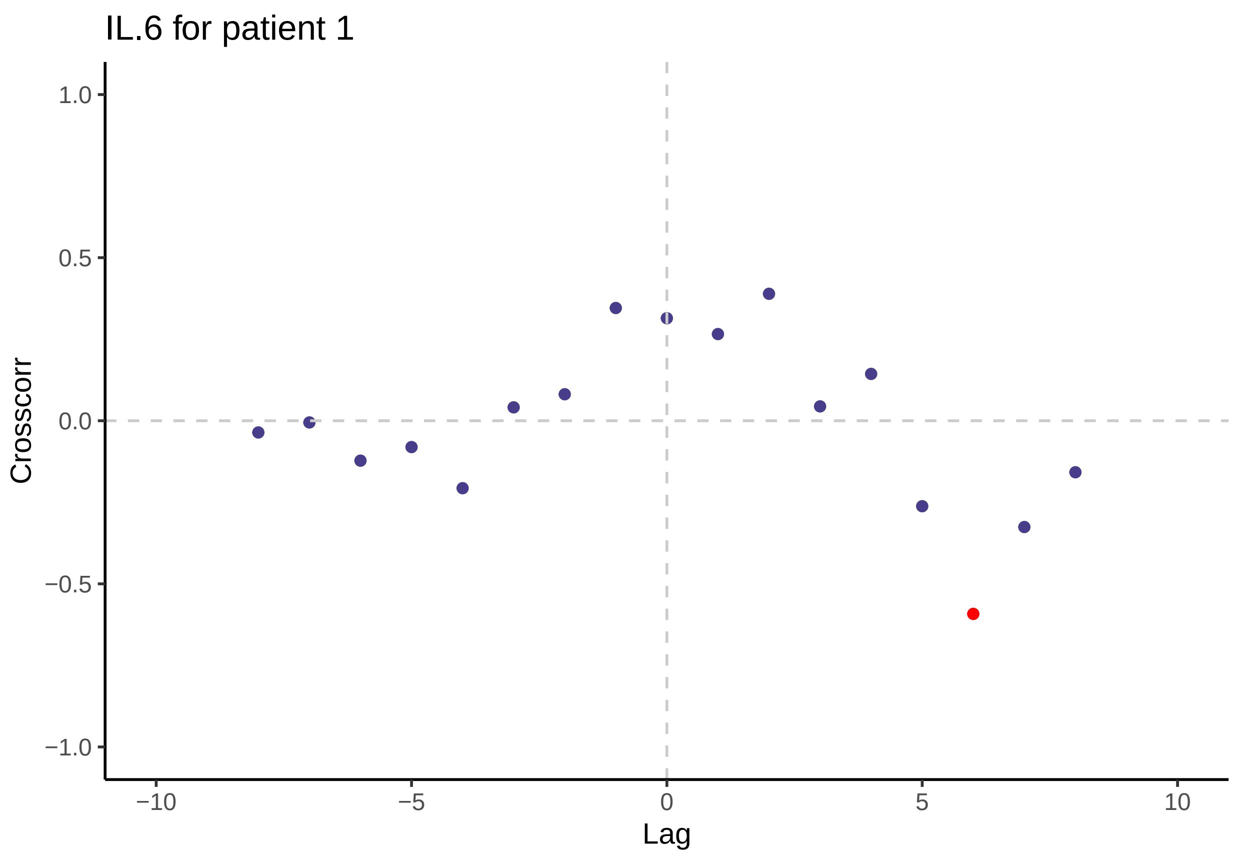


```
## [1] "IL.6 for patient 1 - p-value: 0.880810448404764"
```

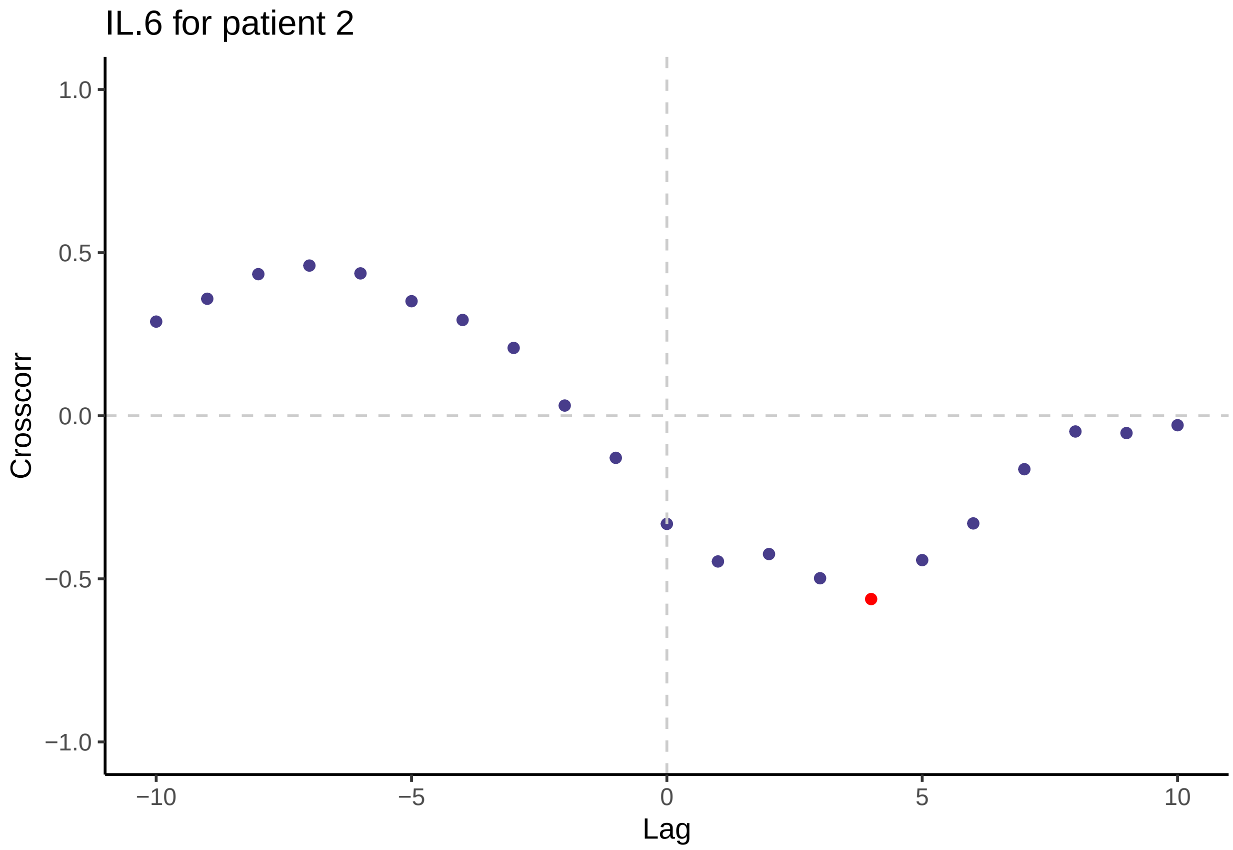


```
## [1] "IL.6 for patient 2 - p-value: 0.714089669021856"
```

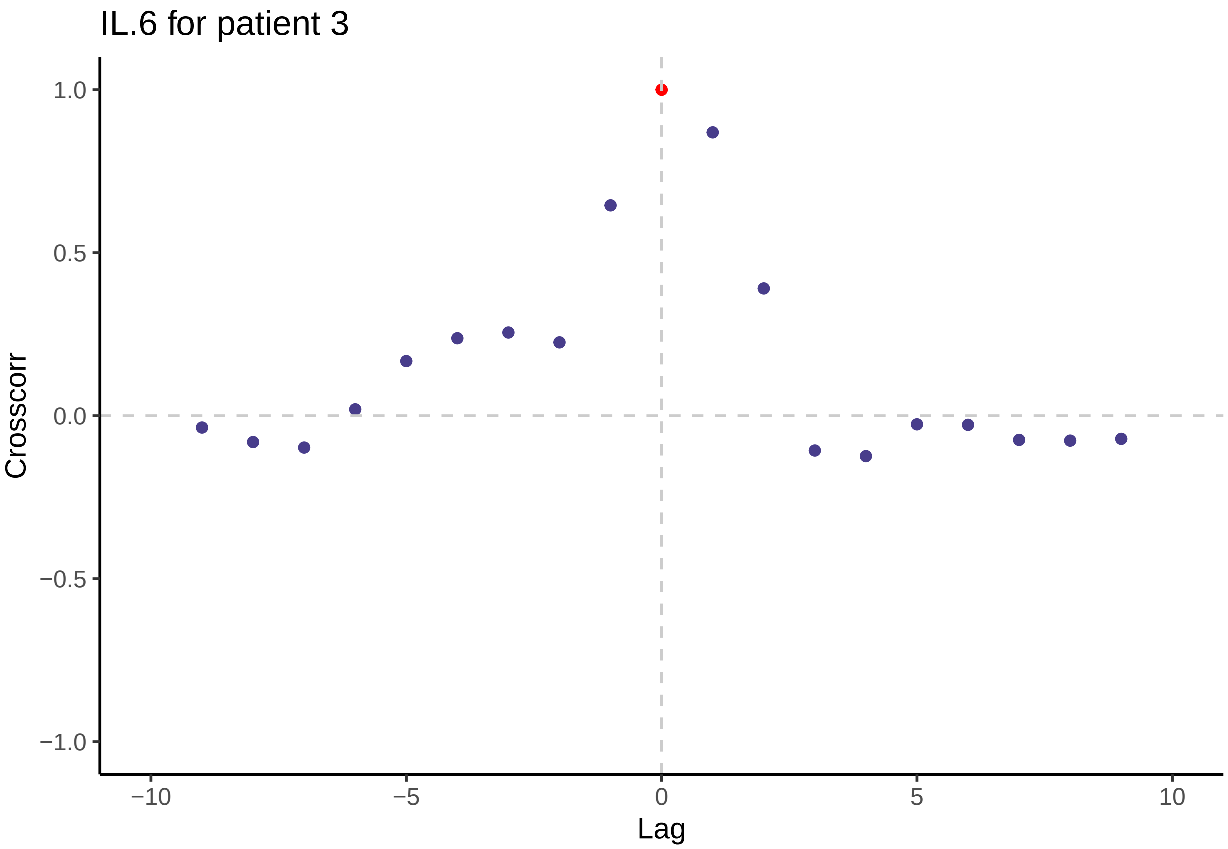


```
## [1] "IL.6 for patient 3 - p-value: 0.0517178557673809"
```

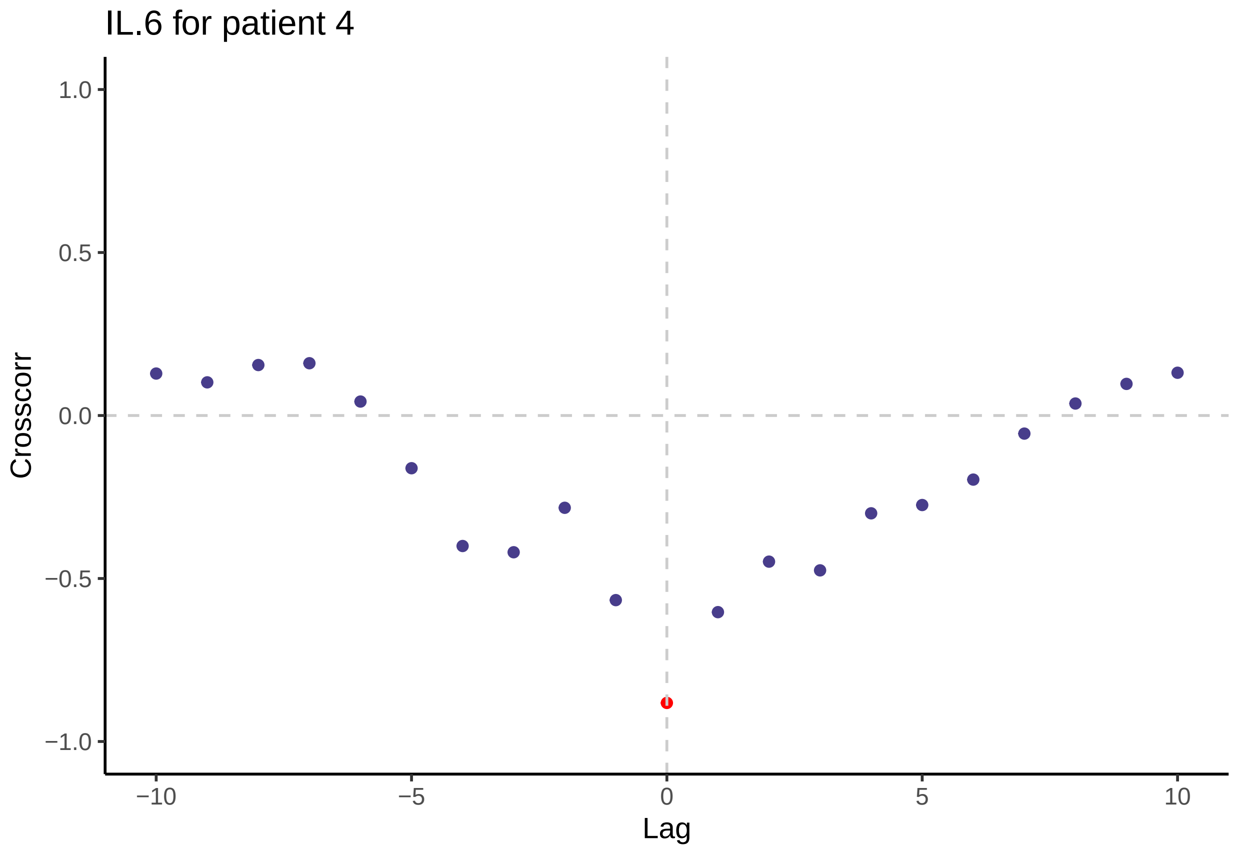


```
## [1] "IL.6 for patient 4 - p-value: 0.00612121607143279"
```

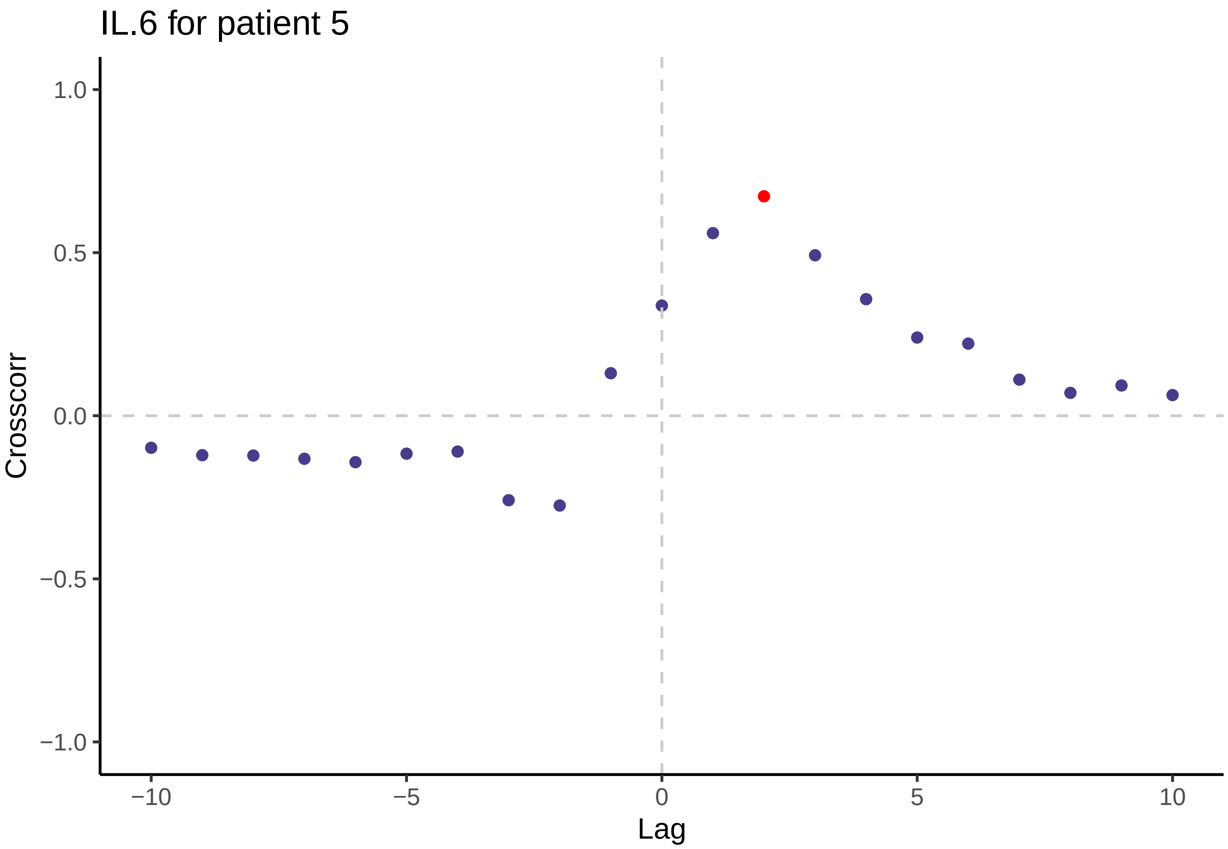


```
## [1] "IL.6 for patient 5 - p-value: 0.127176720376911"
```

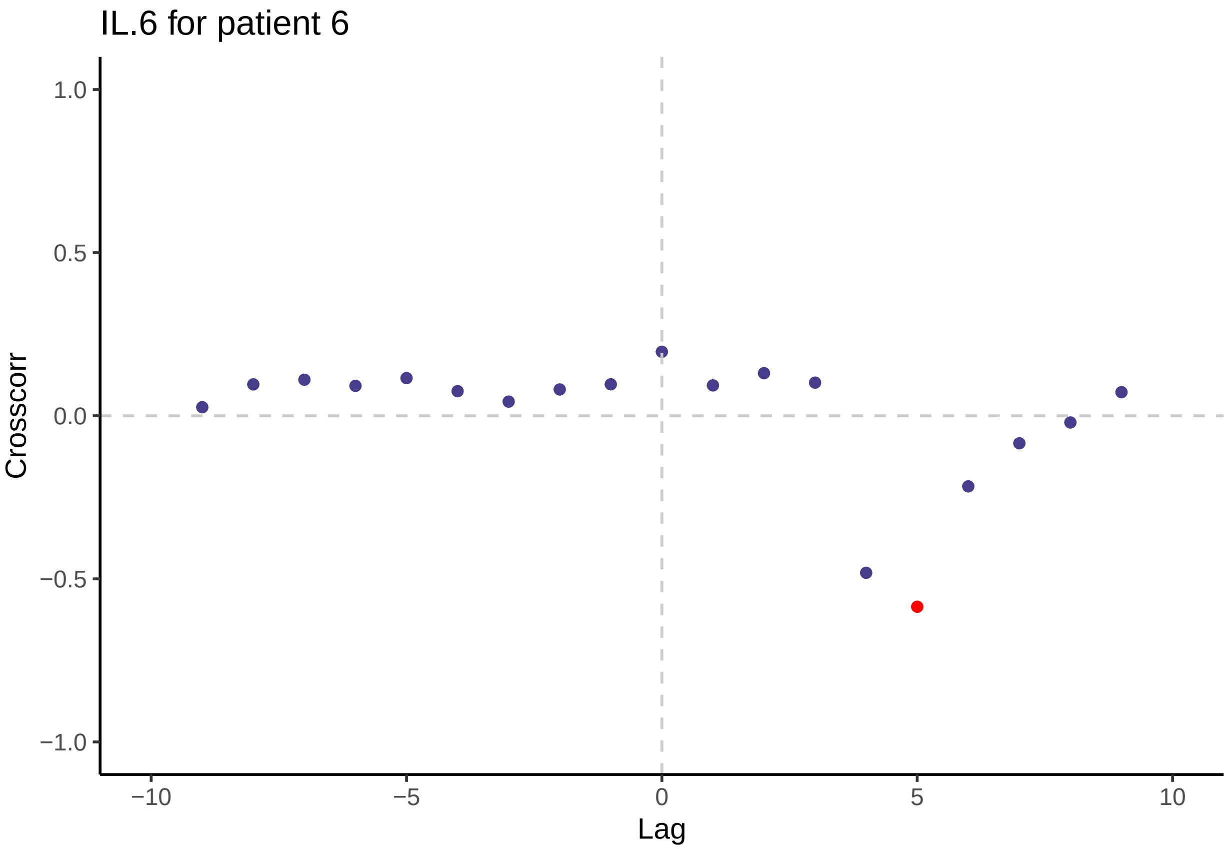


```
## [1] "IL.6 for patient 6 - p-value: 0.948036464114316"
```

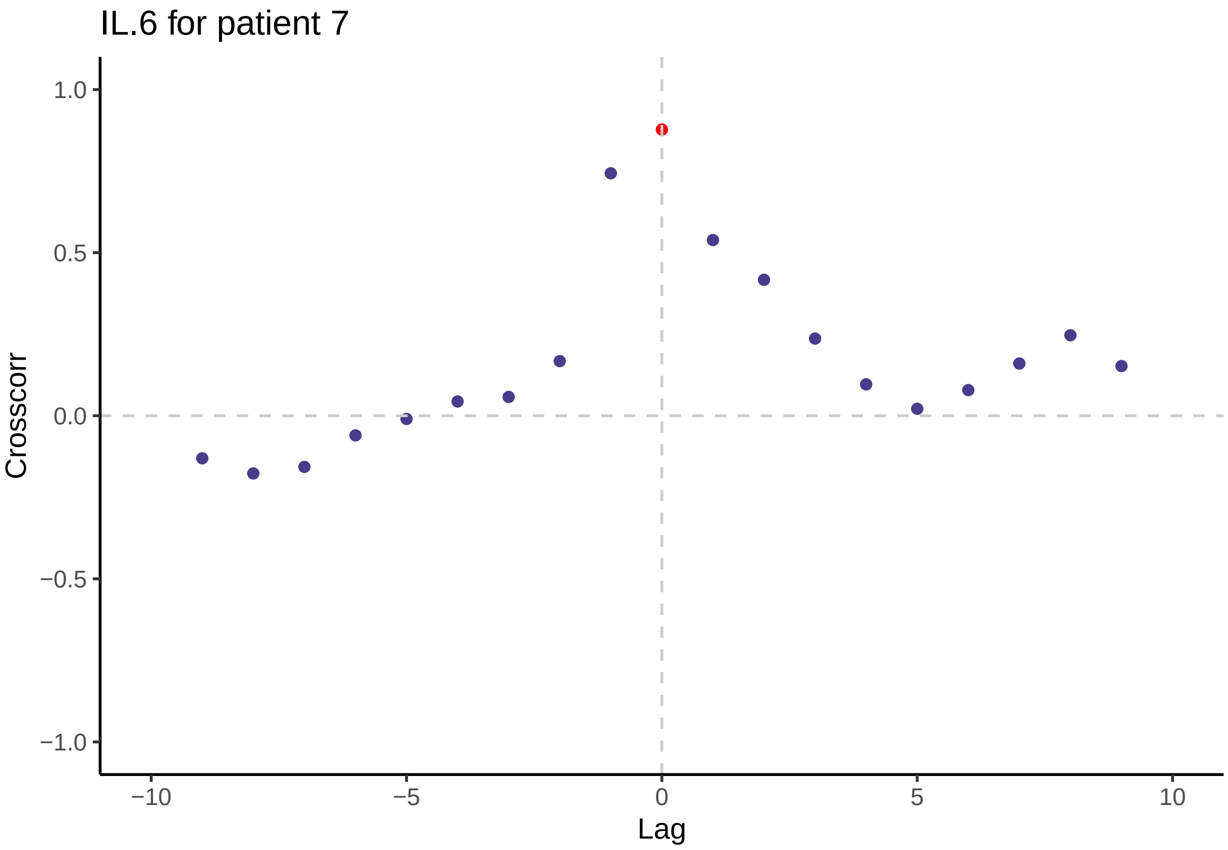


```
## [1] "IL.6 for patient 7 - p-value: 0.0172708748202394"
```

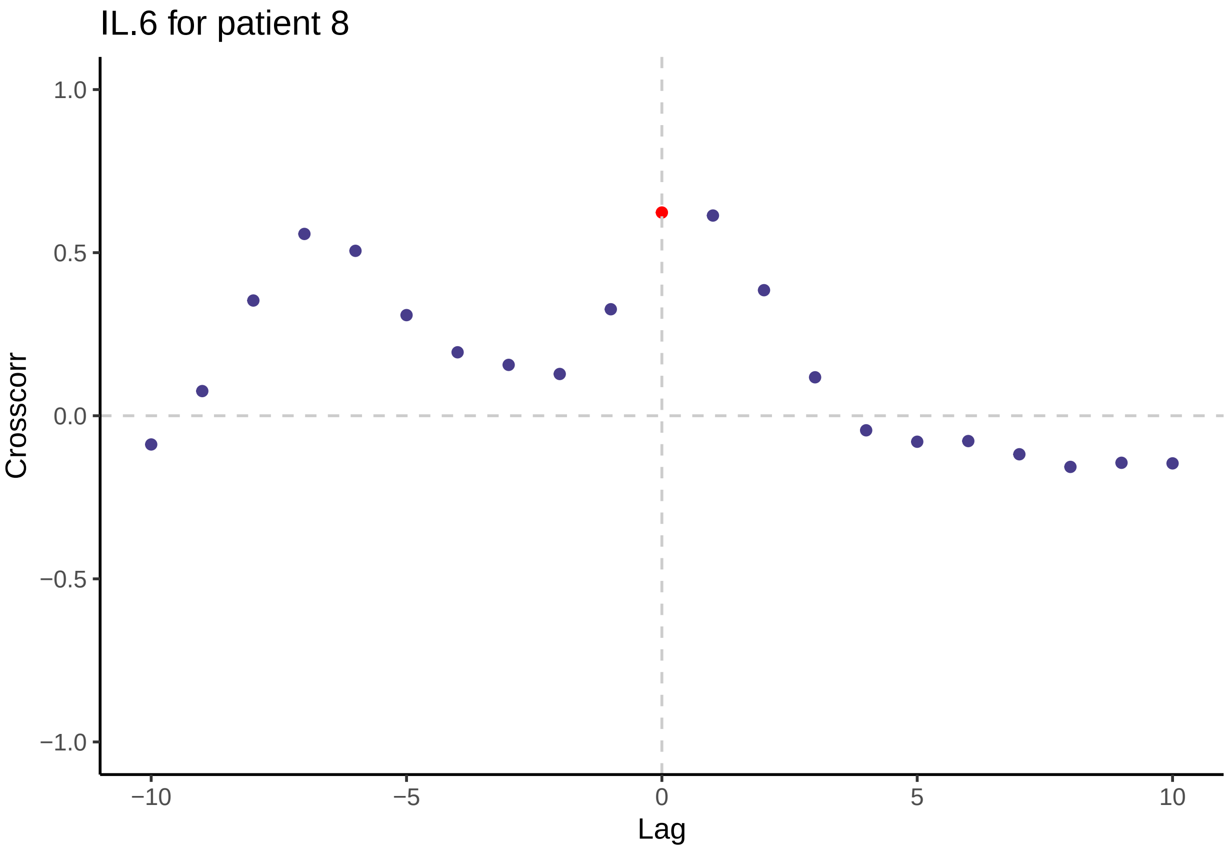


```
## [1] "IL.6 for patient 8 - p-value: 0.00985994571203789"
```

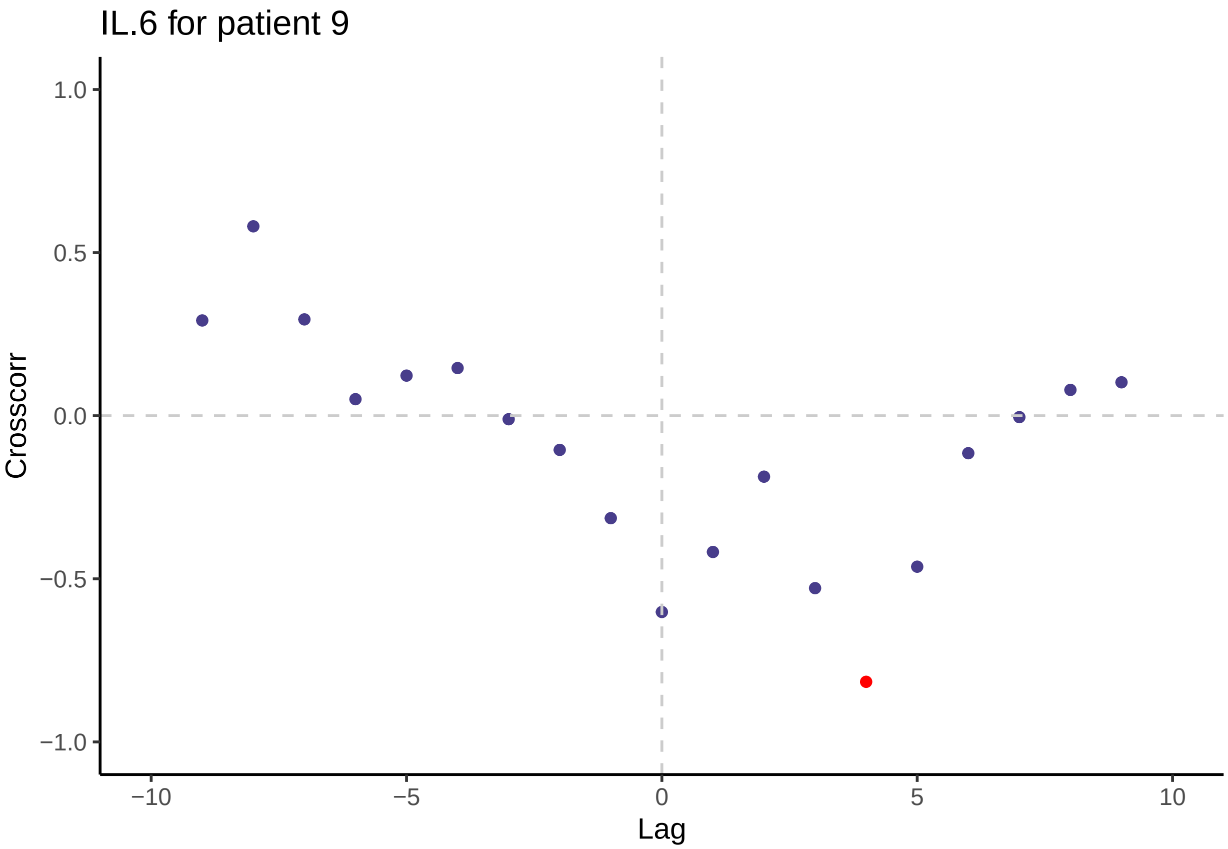


```
## [1] "IL.6 for patient 9 - p-value: 0.233669361347124"
```

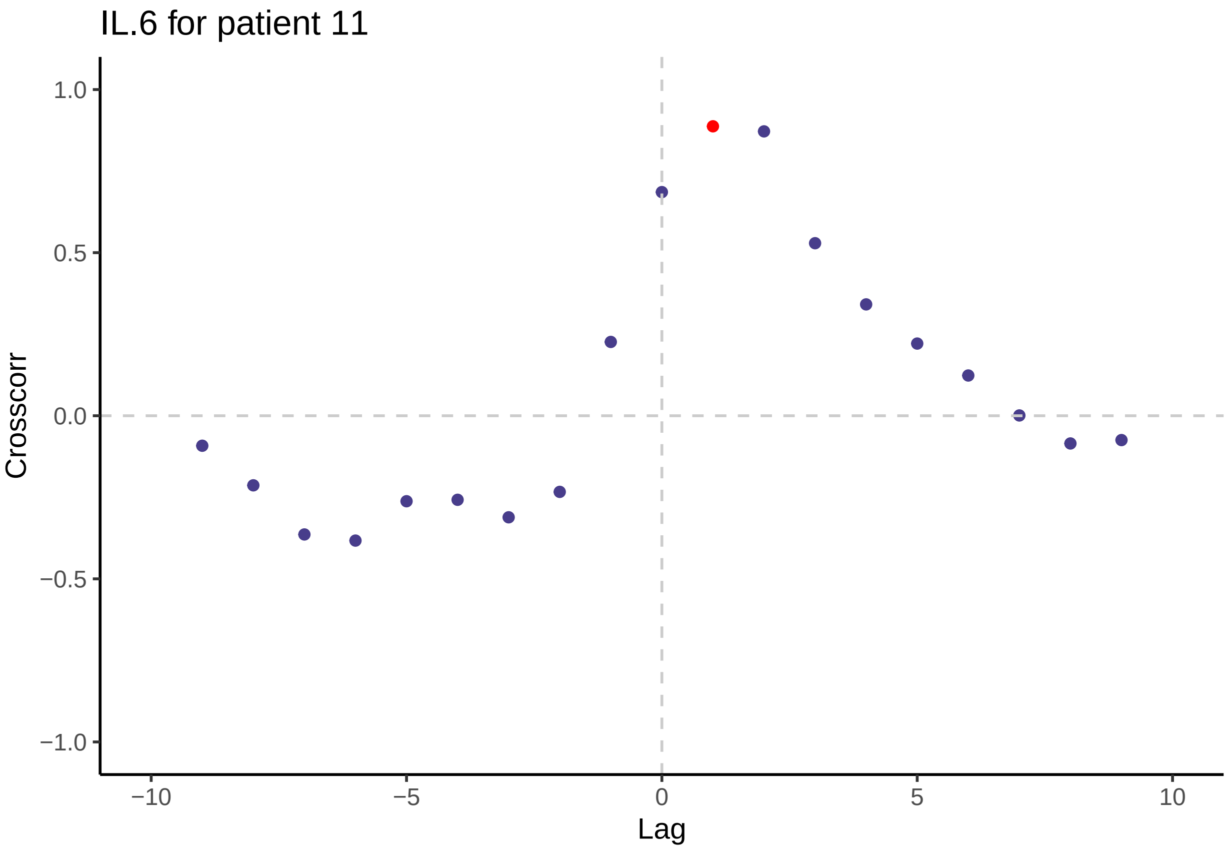


```
## [1] "IL.6 for patient 11 - p-value: 0.378952665925885"
```

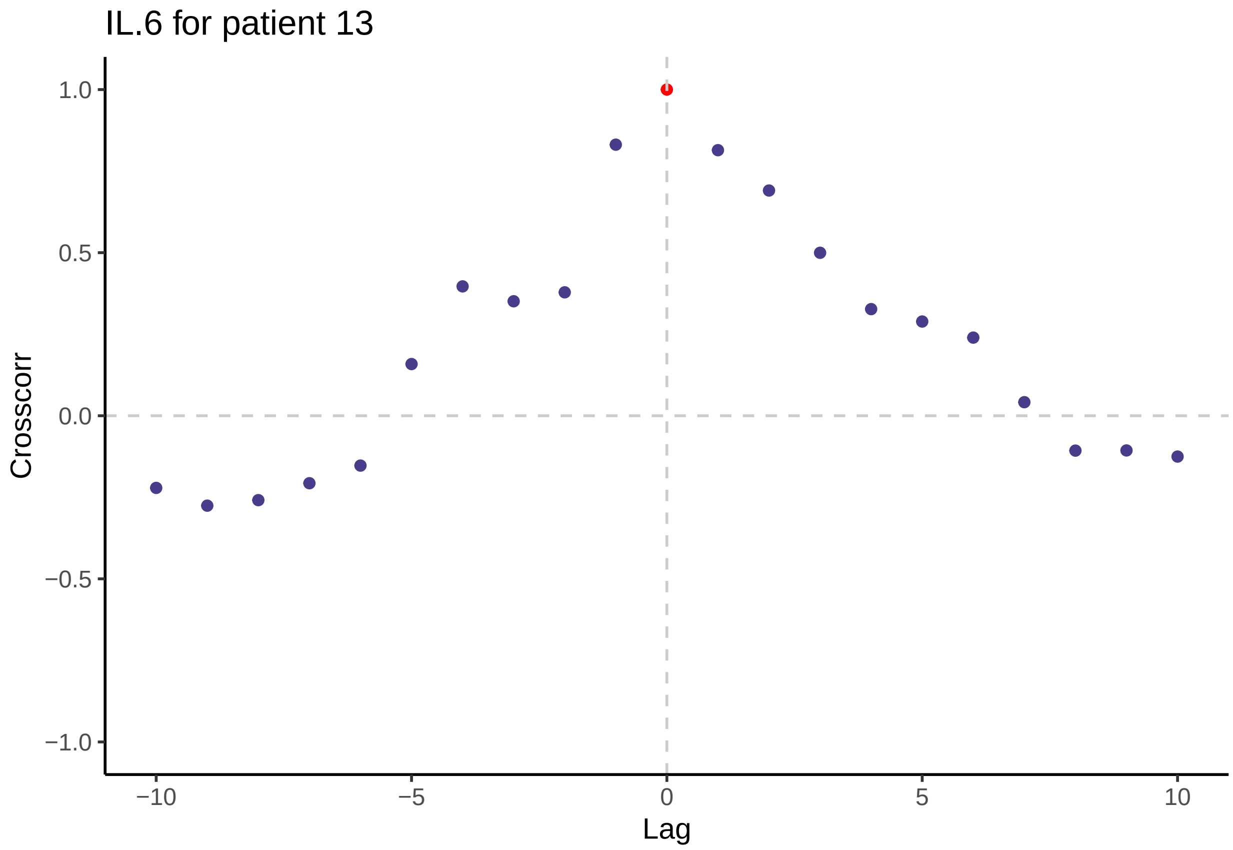


```
## [1] "IL.6 for patient 13 - p-value: 0.0196395253001243"
```

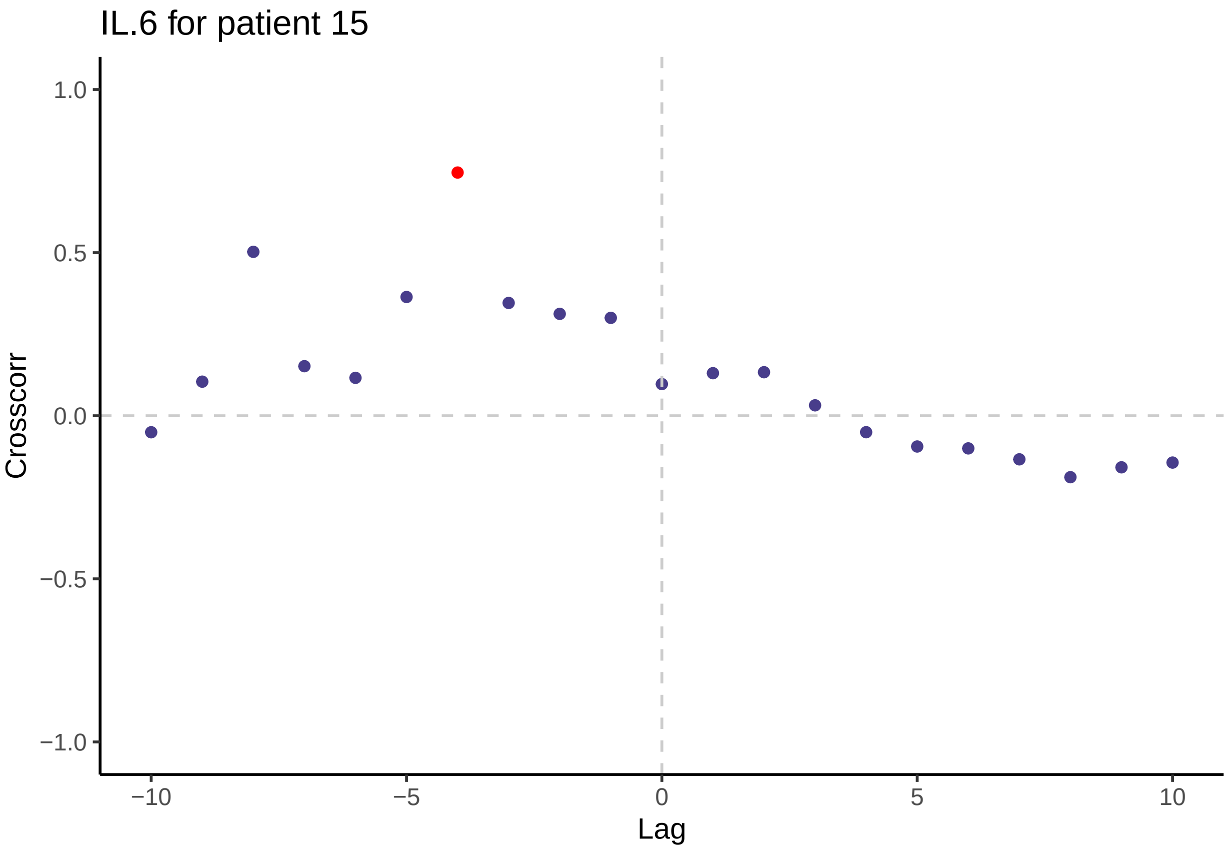


```
## [1] "IL.6 for patient 15 - p-value: 0.0425301995948818"
```

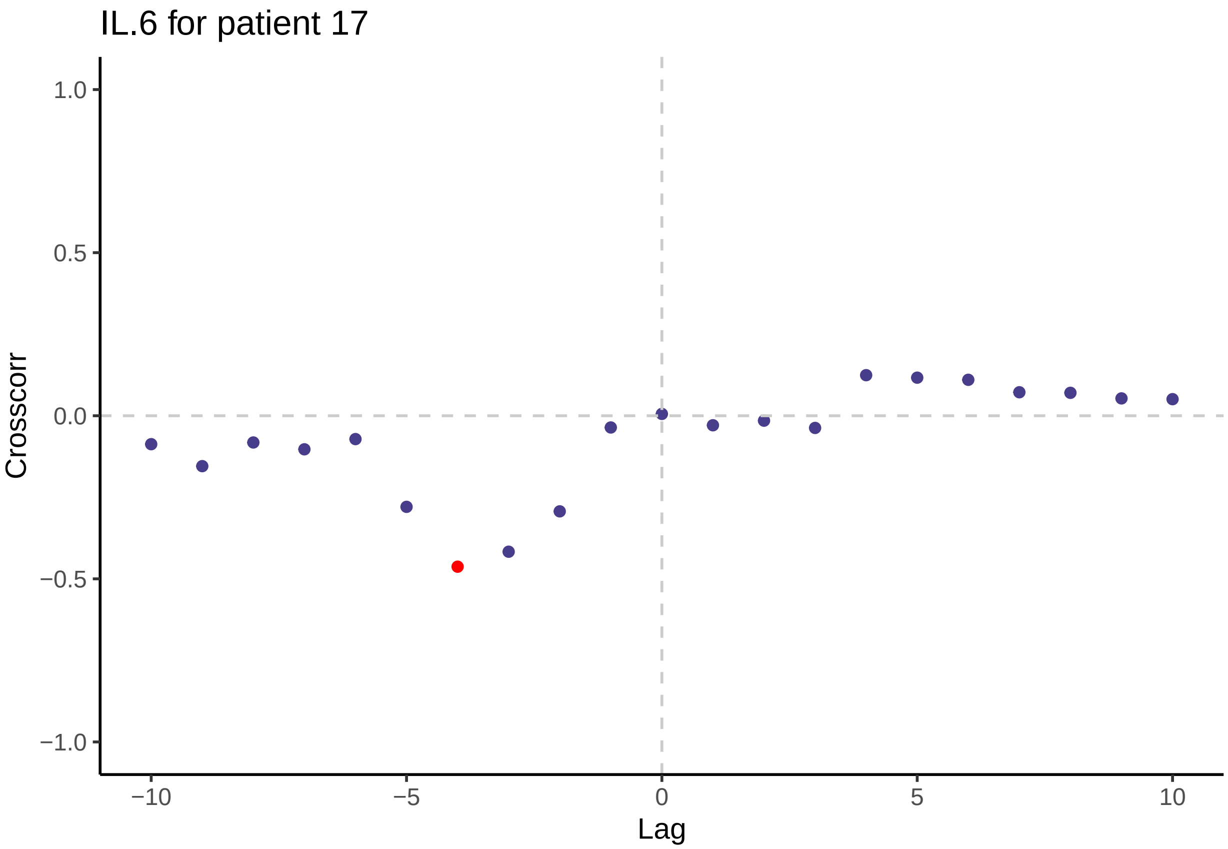


```
## [1] "IL.6 for patient 17 - p-value: 0.0716891152348136"
```

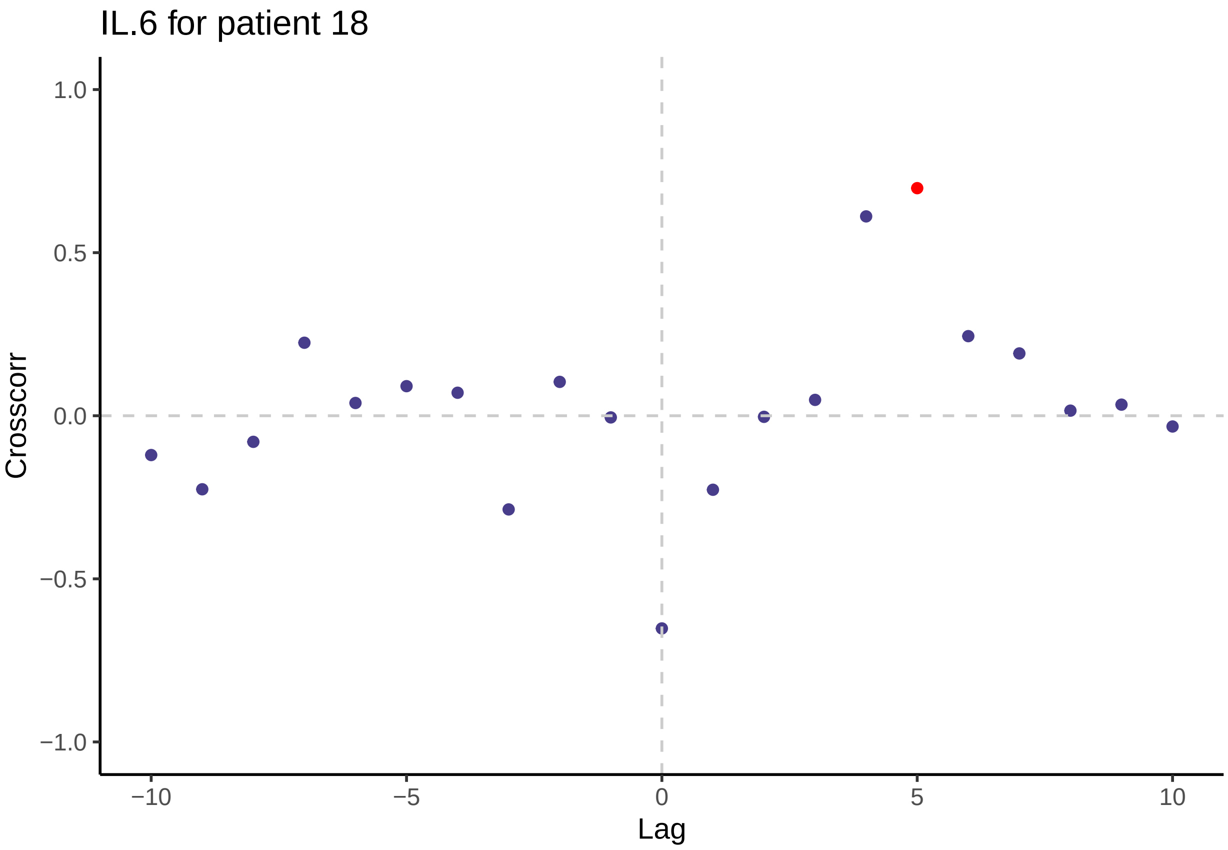


```
## [1] "IL.6 for patient 18 - p-value: 0.580581062969761"
## Warning: Removed 1 rows containing missing values (geom_point).
```

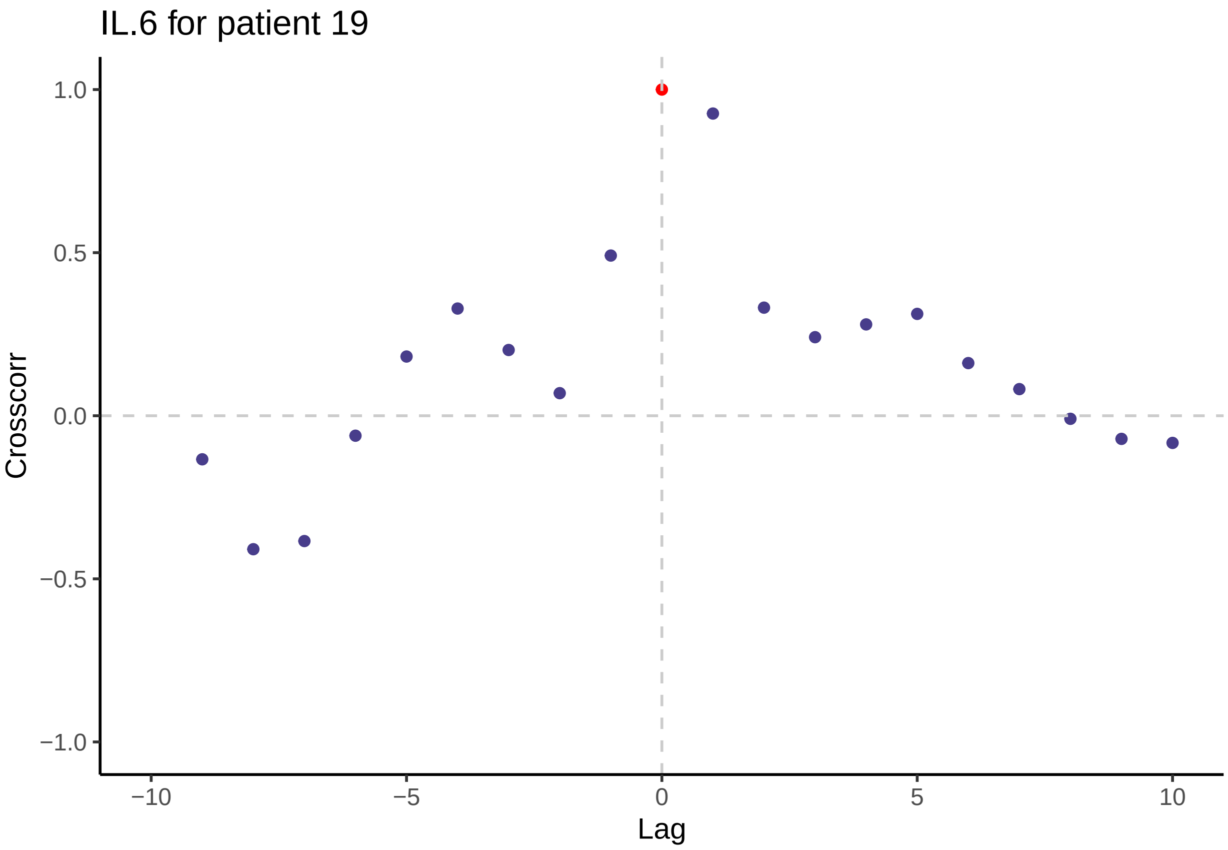


```
## [1] "IL.6 for patient 19 - p-value: 0.0431150026852153"
```

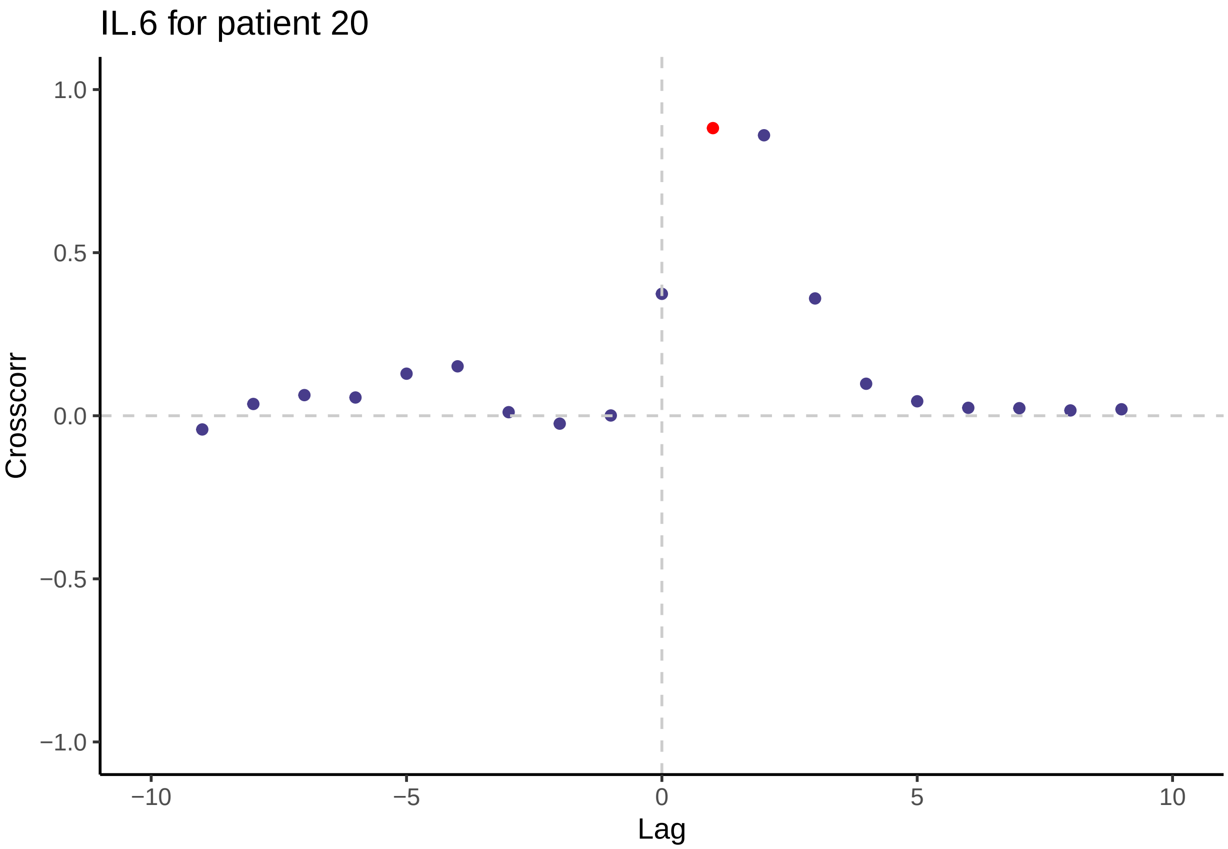


```
## [1] "IL.6 for patient 20 - p-value: 0.0187664047409164"
## Warning: Removed 2 rows containing missing values (geom_point).
```

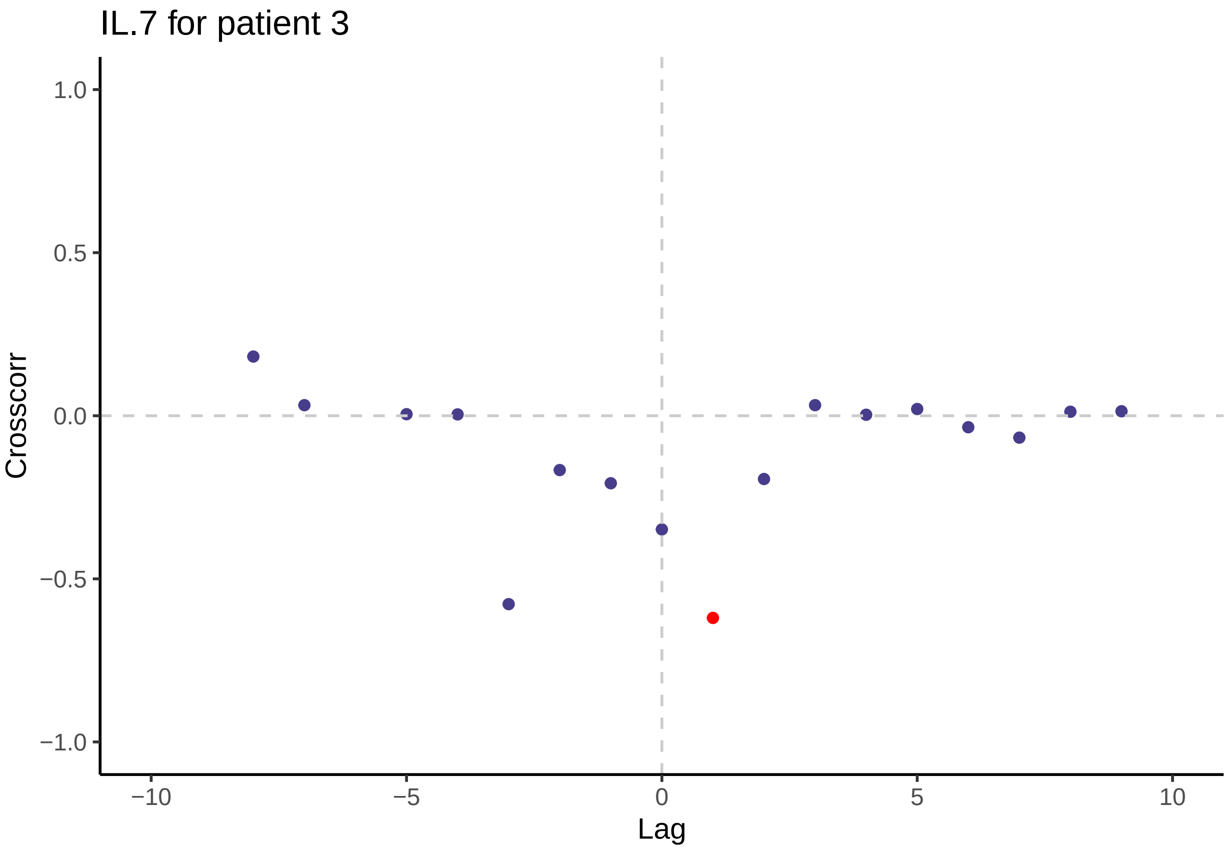


```
## [1] "IL.7 for patient 3 - p-value: 0.0513233663158157"
```

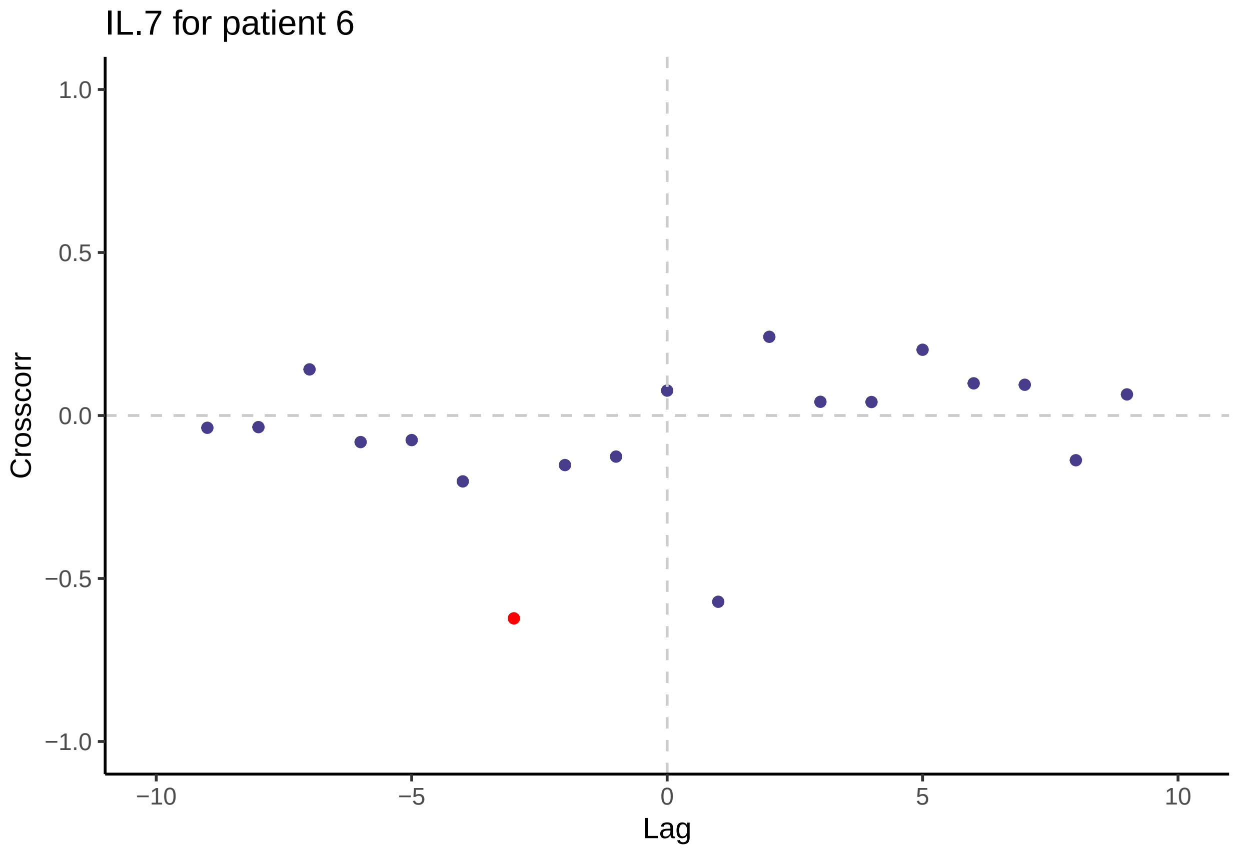


```
## [1] "IL.7 for patient 6 - p-value: 0.306183180392261"
```

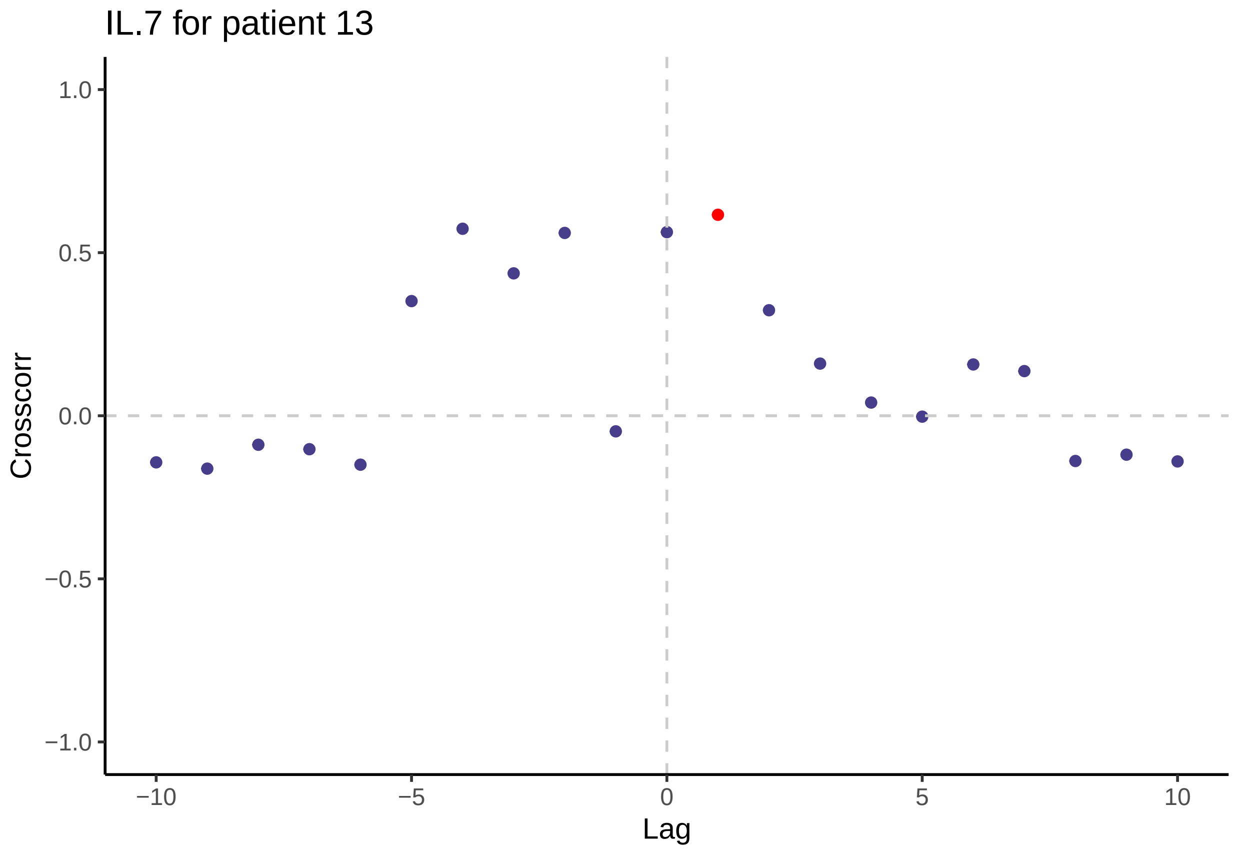


```
## [1] "IL.7 for patient 13 - p-value: 0.0410015908511051"
```

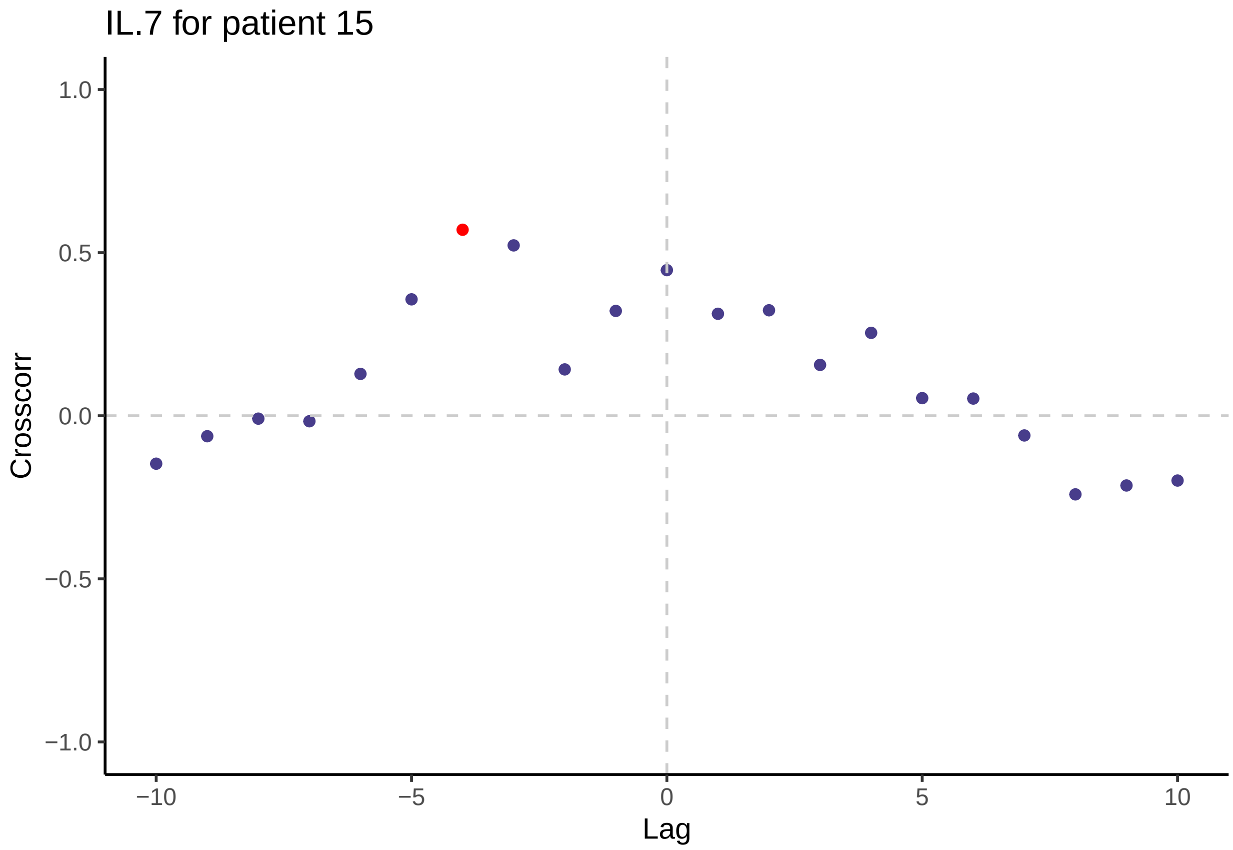


```
## [1] "IL.7 for patient 15 - p-value: 0.0258023897743227"
```

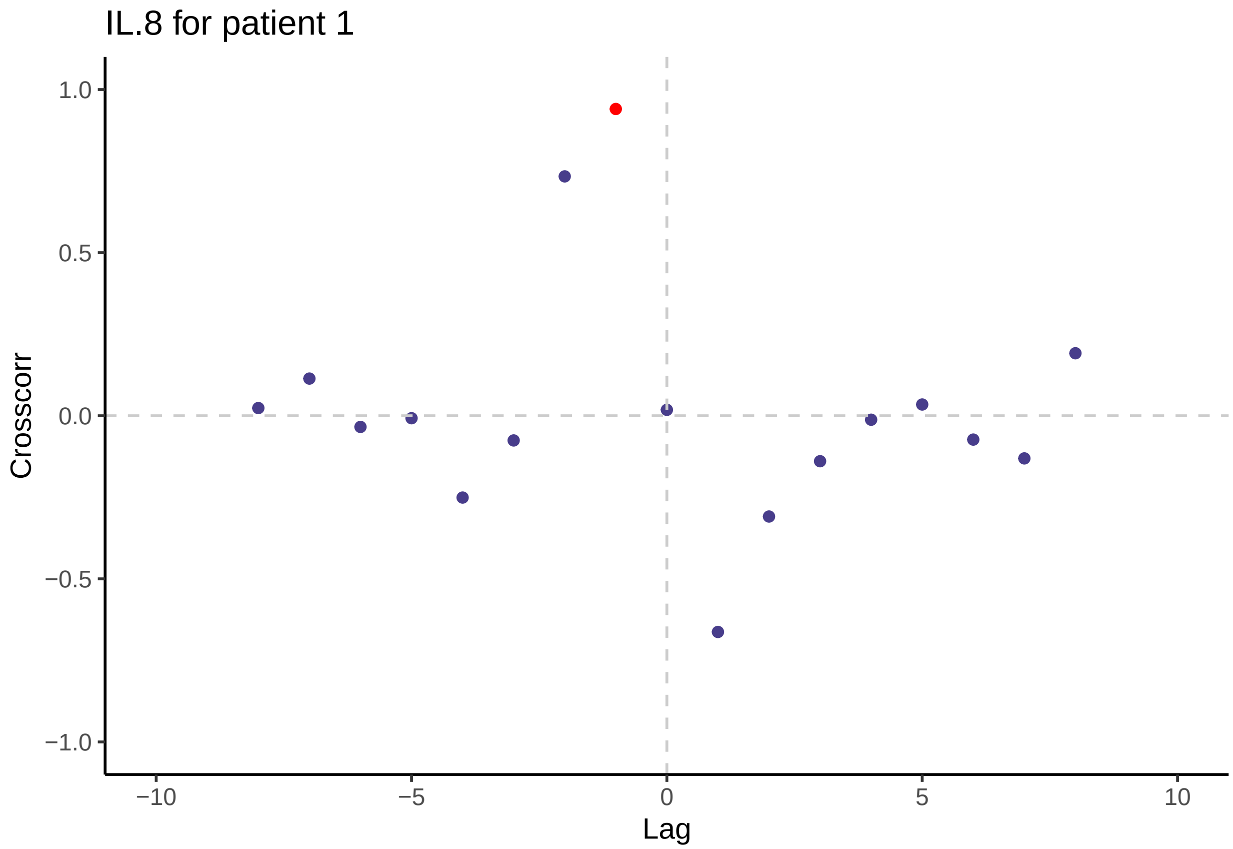


```
## [1] "IL.8 for patient 1 - p-value: 0.812681466834193"
```

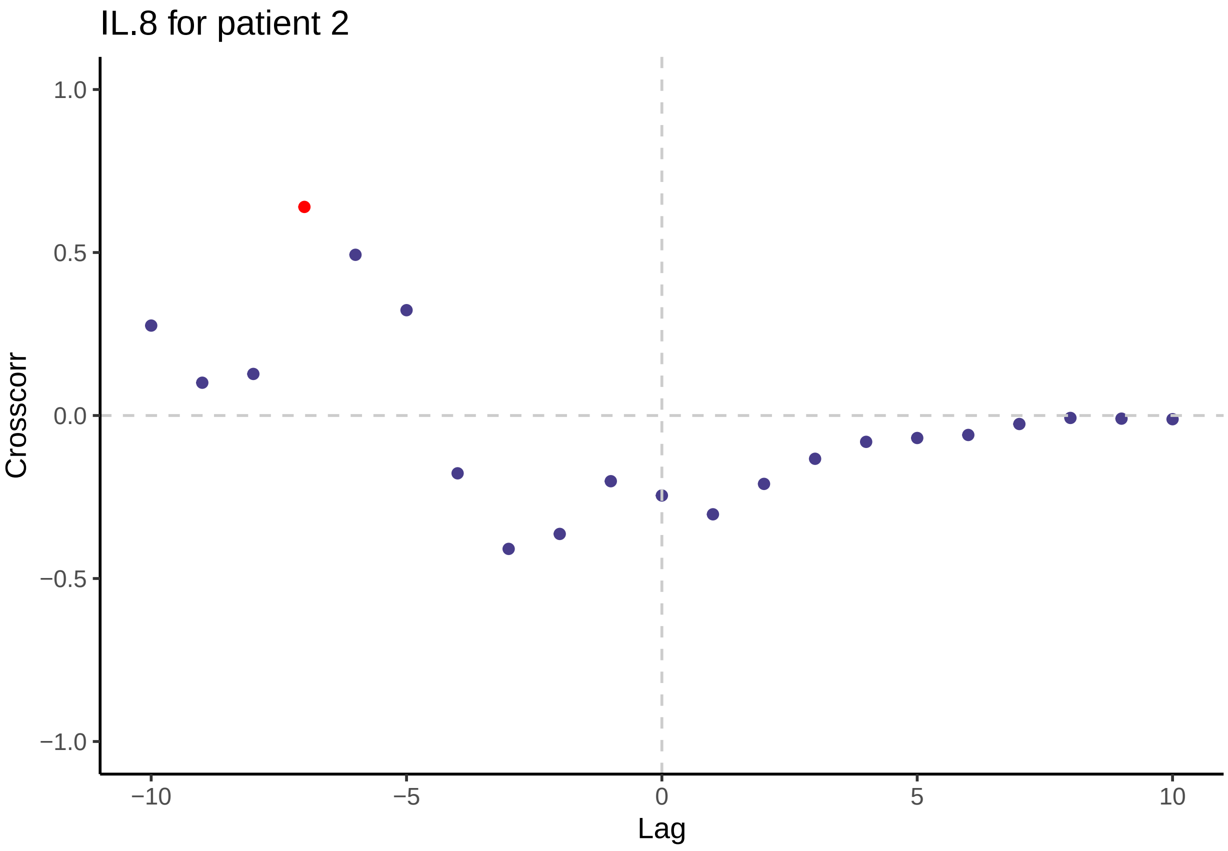


```
## [1] "IL.8 for patient 2 - p-value: 0.782398361696971"
```

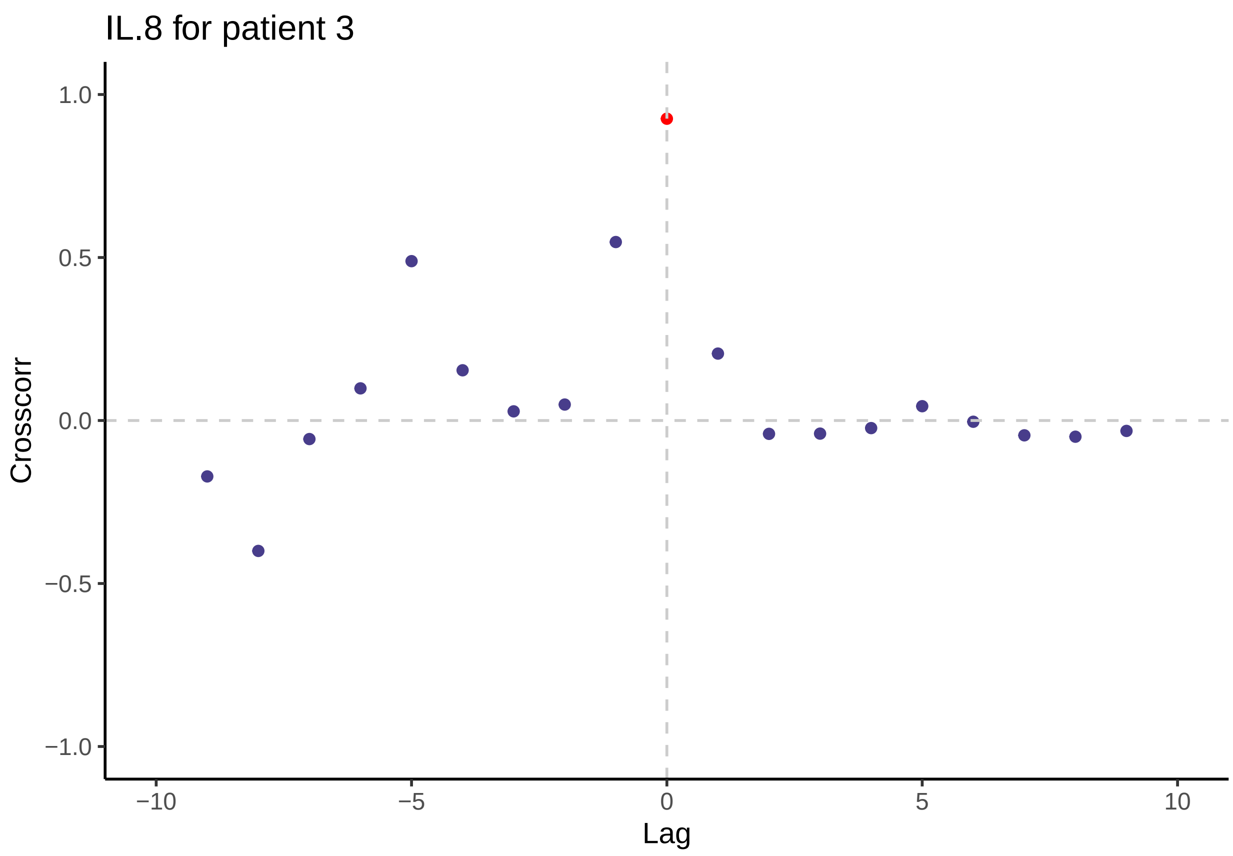


```
## [1] "IL.8 for patient 3 - p-value: 0.203483593044725"
```

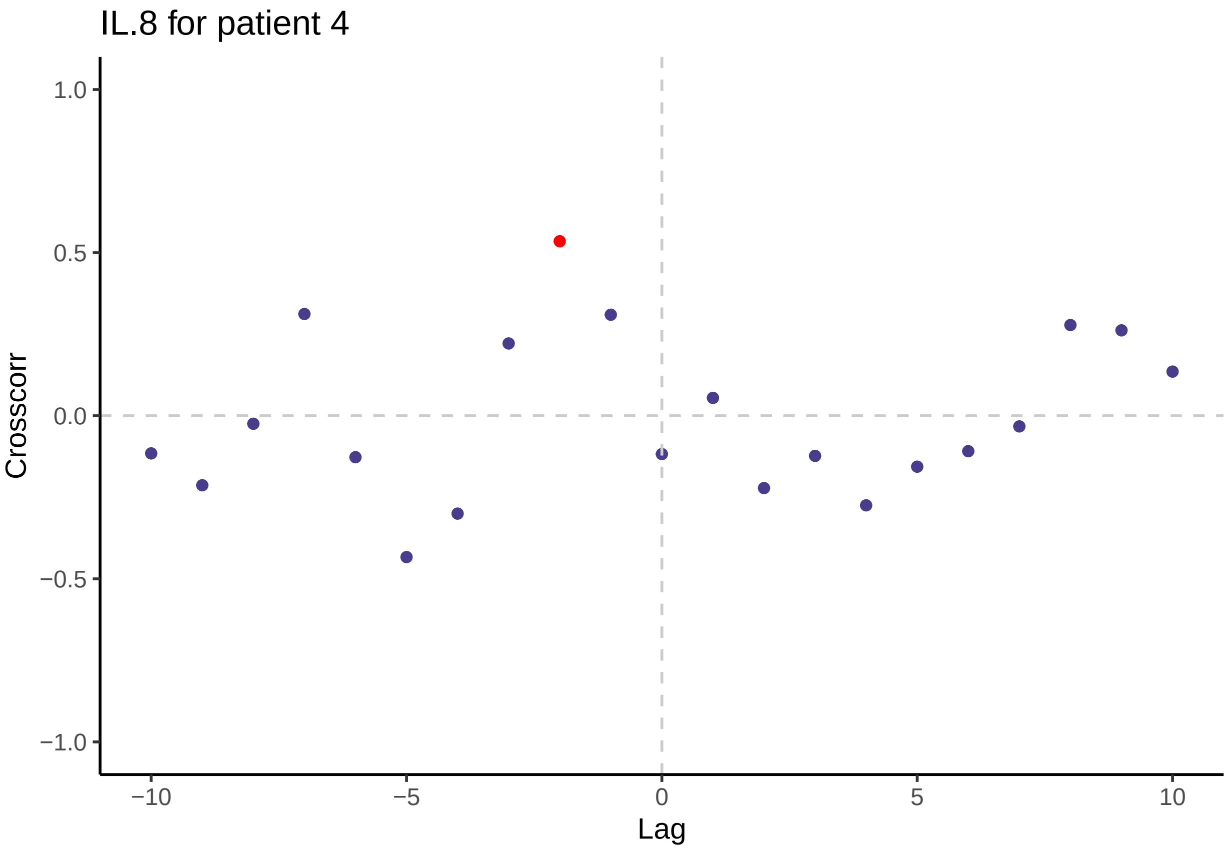


```
## [1] "IL.8 for patient 4 - p-value: 0.9040160748611"
```

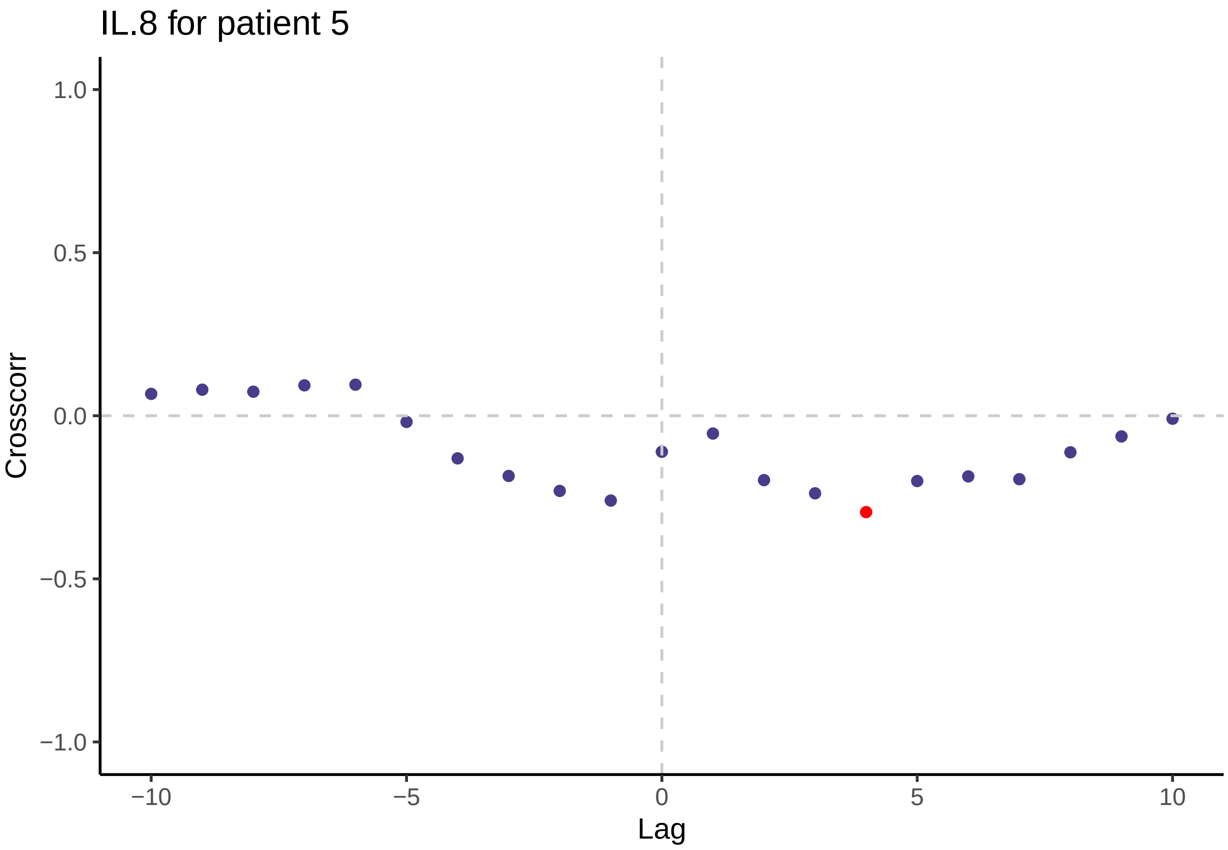


```
## [1] "IL.8 for patient 5 - p-value: 0.00206717902654269"
```

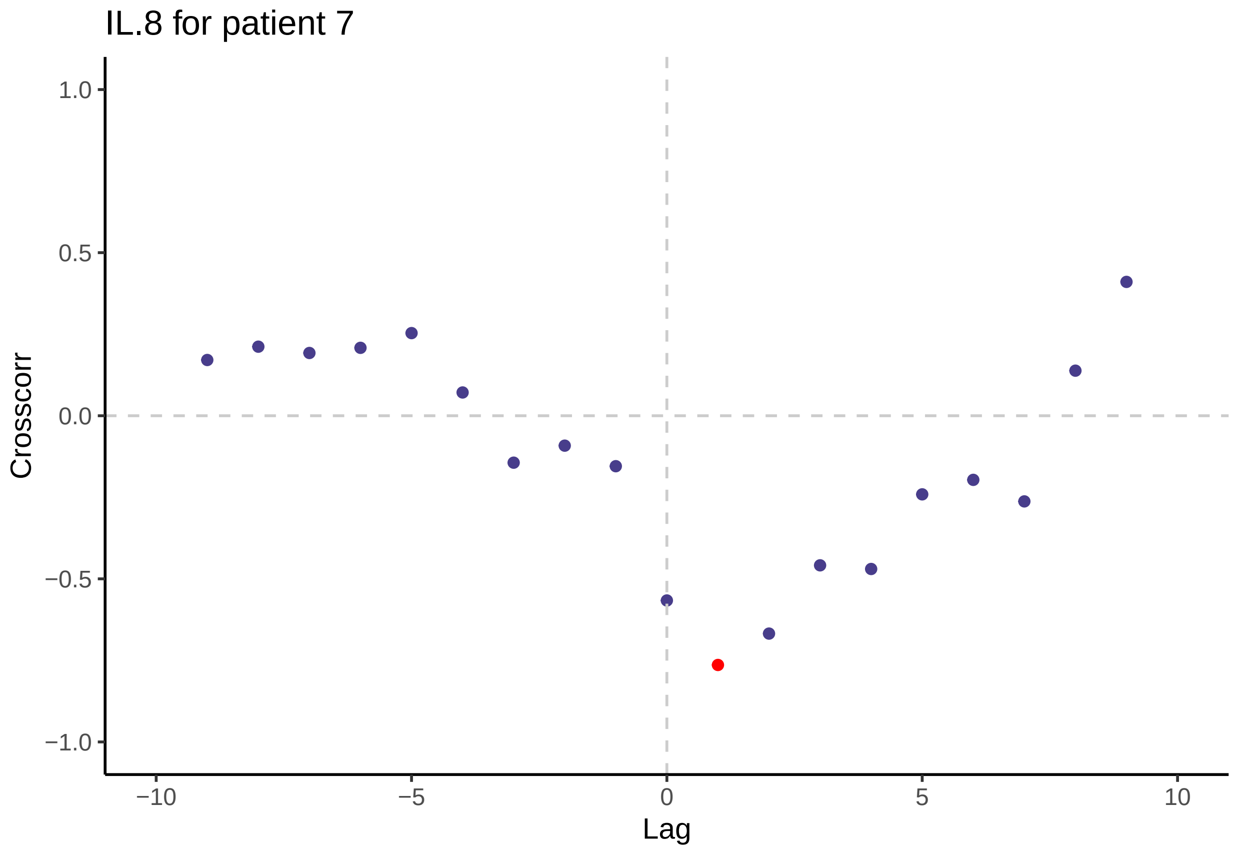


```
## [1] "IL.8 for patient 7 - p-value: 0.131943252810356"
```

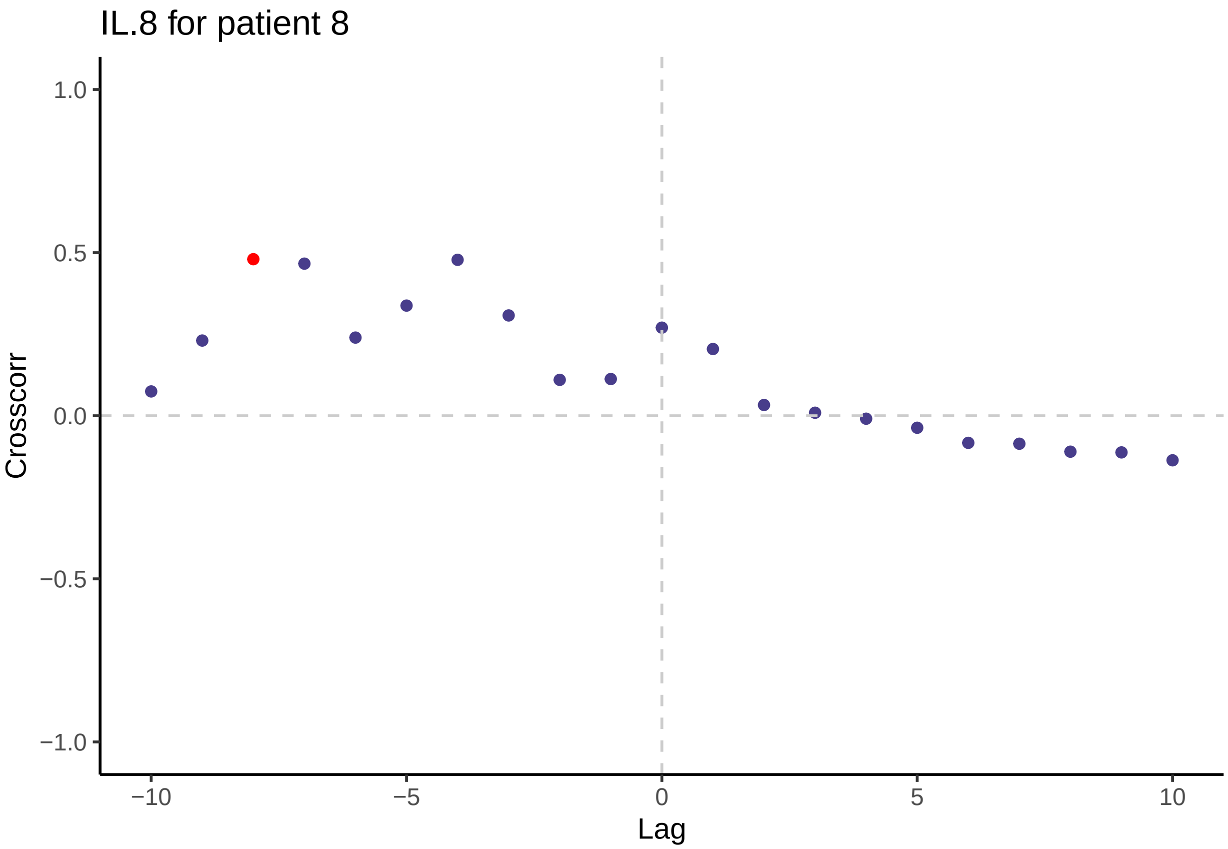


```
## [1] "IL.8 for patient 8 - p-value: 0.00751174353434095"
```

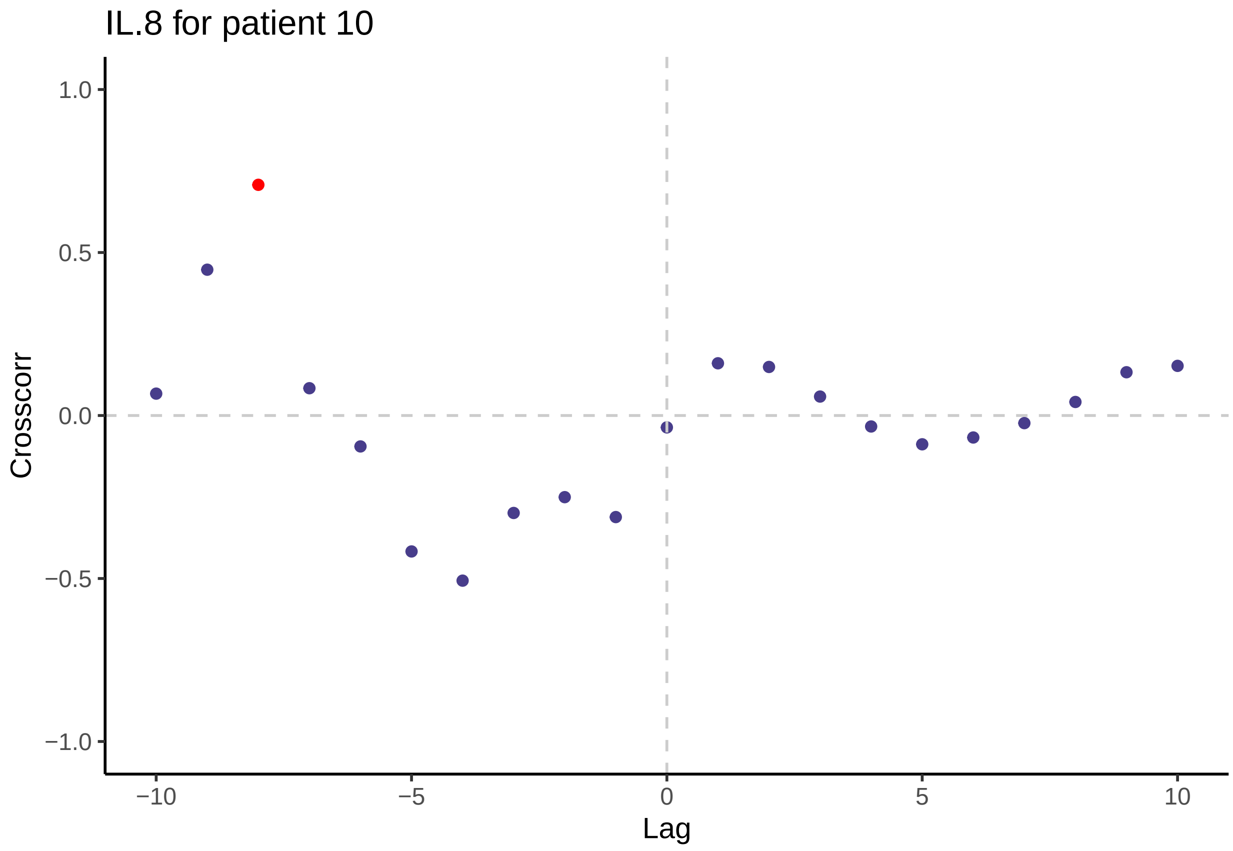


```
## [1] "IL.8 for patient 10 - p-value: 0.919380148210323"
## Warning: Removed 1 rows containing missing values (geom_point).
```

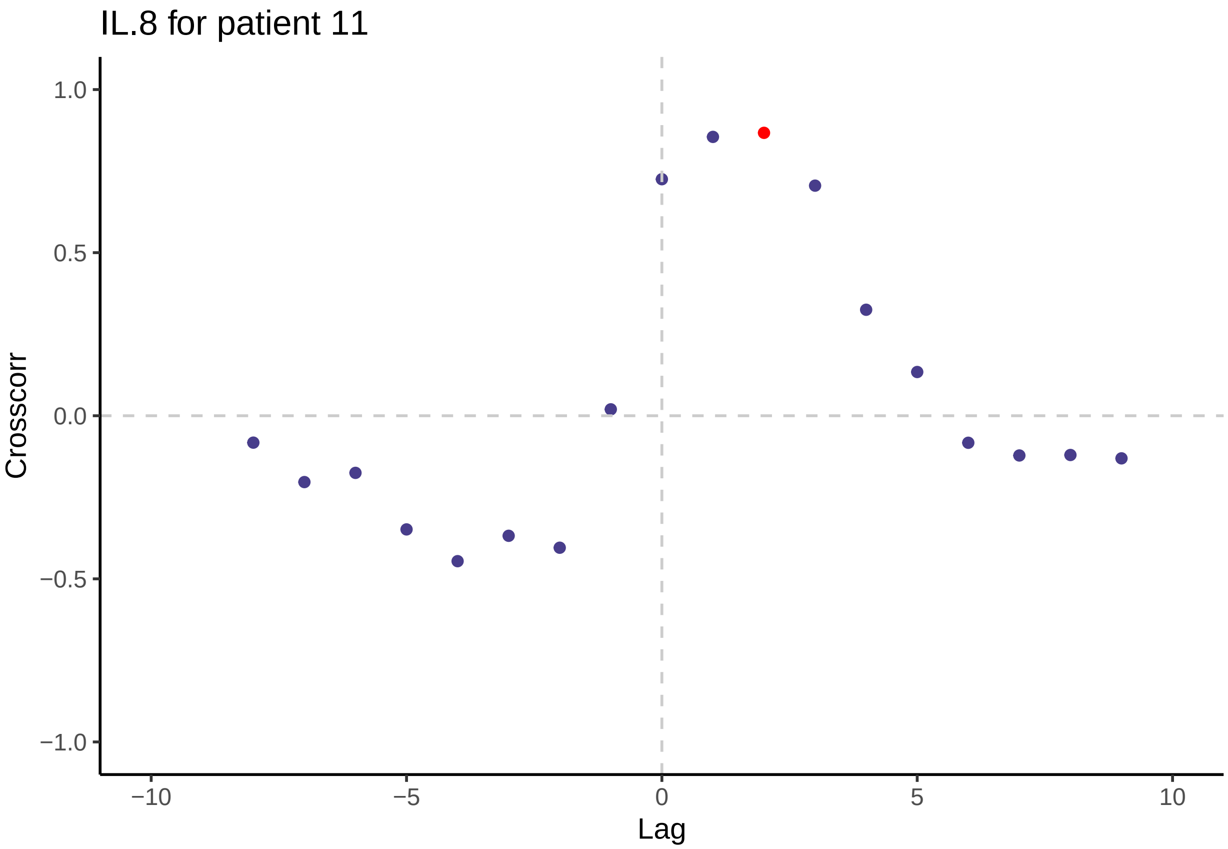


```
## [1] "IL.8 for patient 11 - p-value: 0.547558075766737"
```

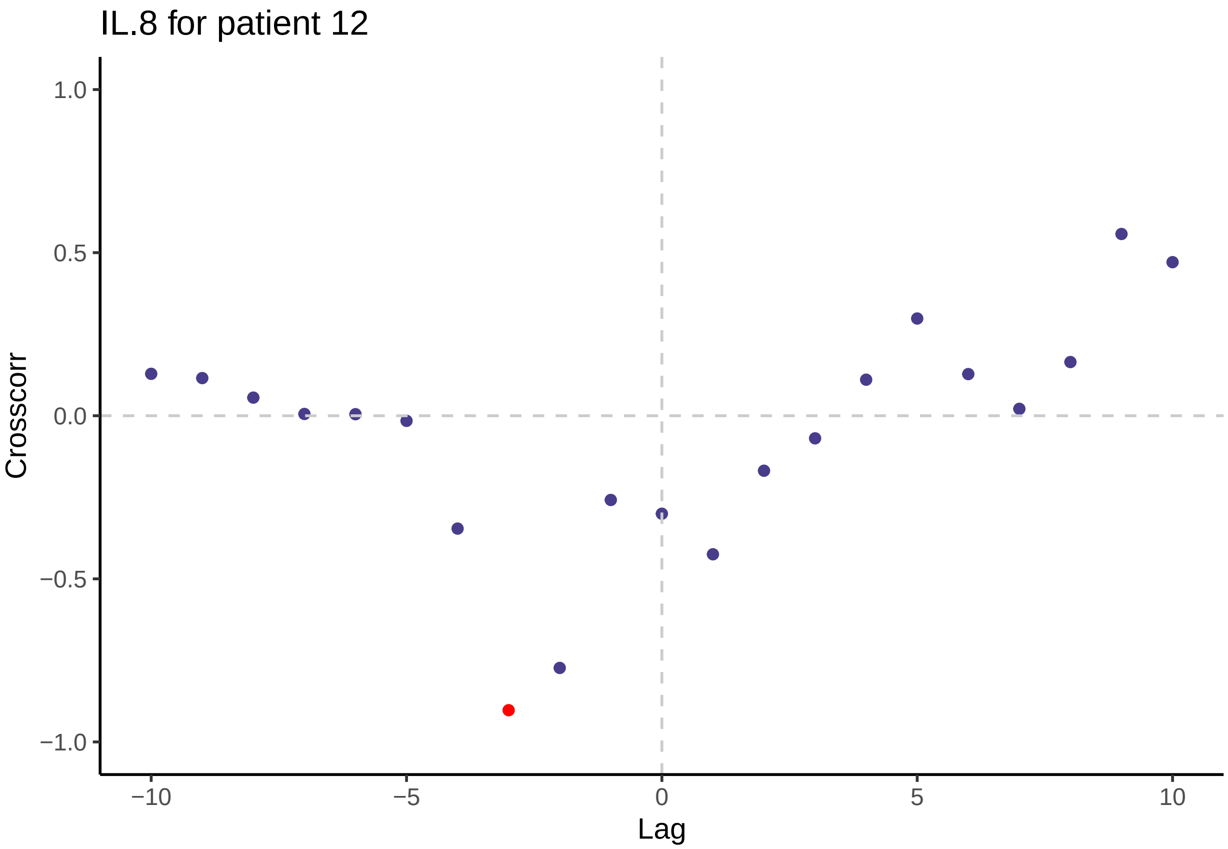


```
## [1] "IL.8 for patient 12 - p-value: 0.47217696761474"
```

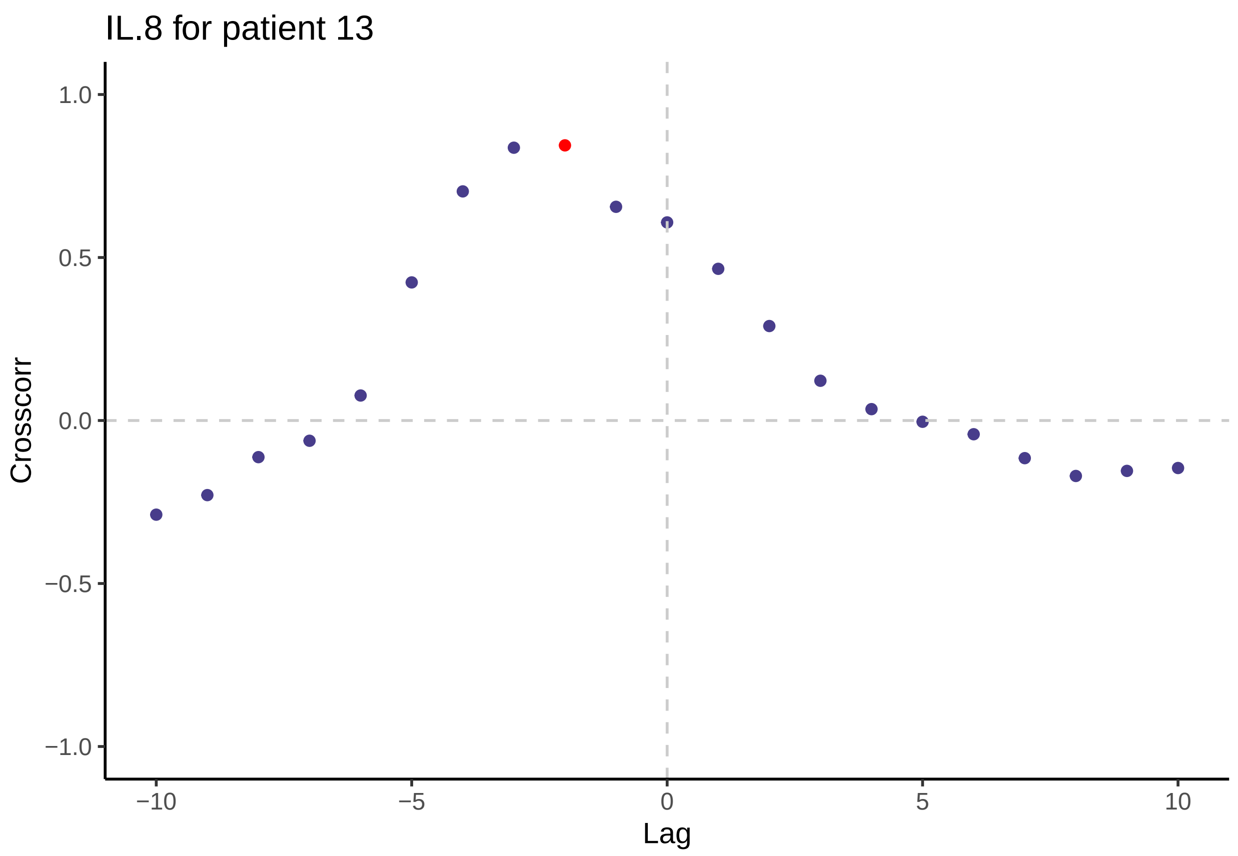


```
## [1] "IL.8 for patient 13 - p-value: 0.0410090647352864"
```

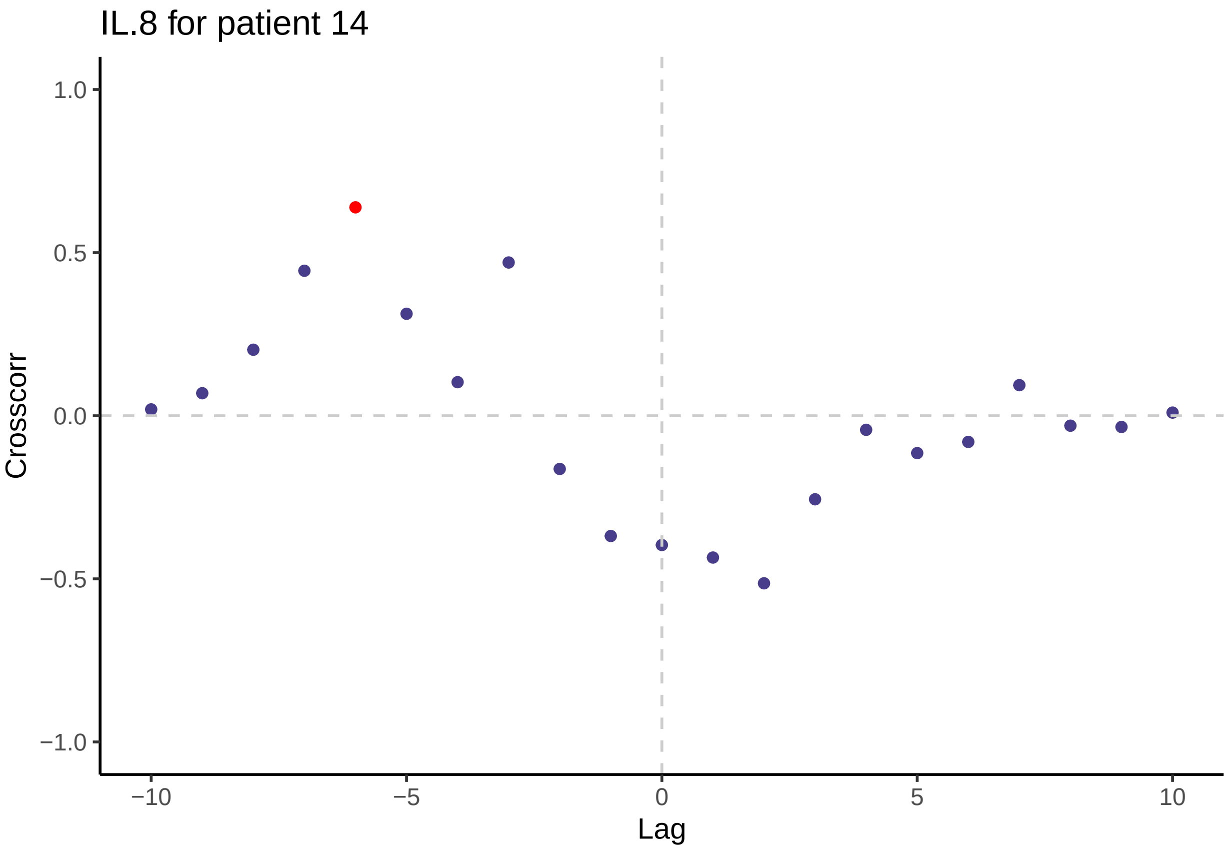


```
## [1] "IL.8 for patient 14 - p-value: 0.959540103185904"
```

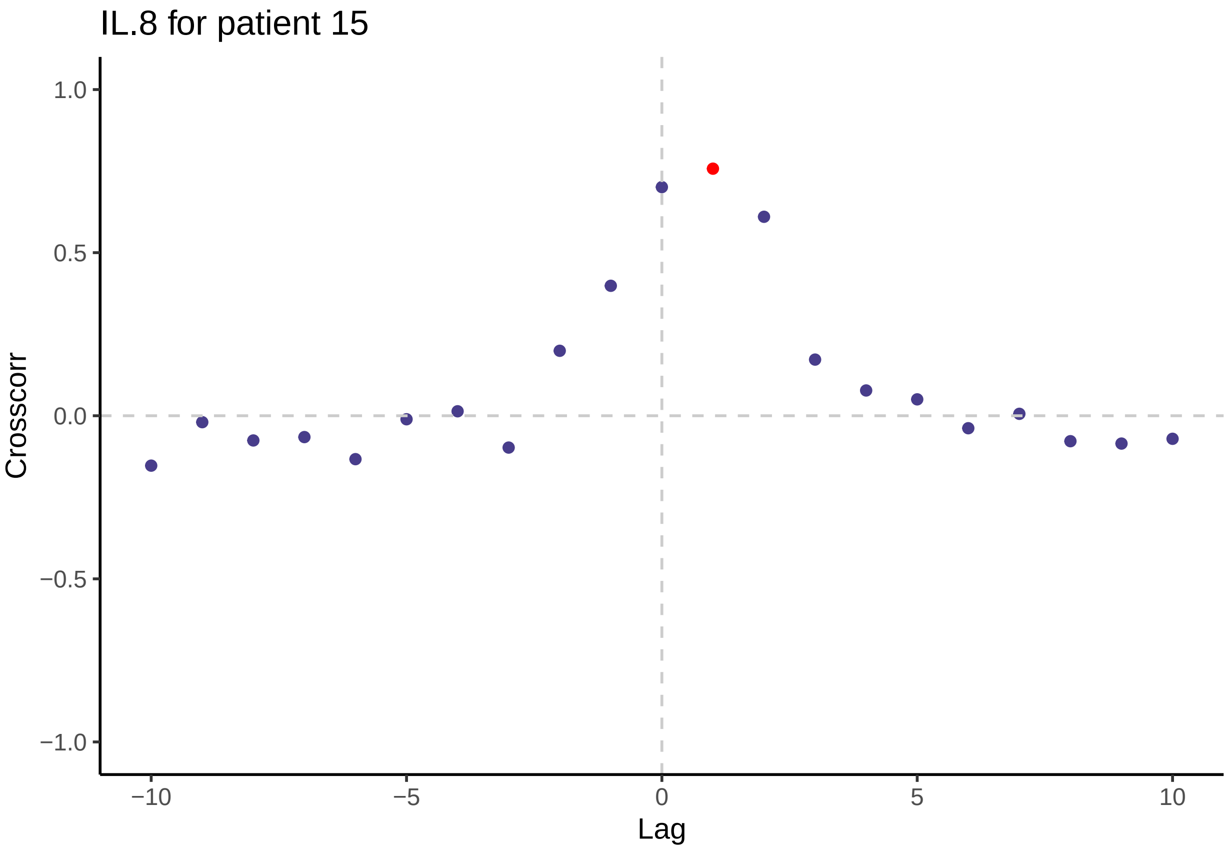


```
## [1] "IL.8 for patient 15 - p-value: 0.104396013085705"
```

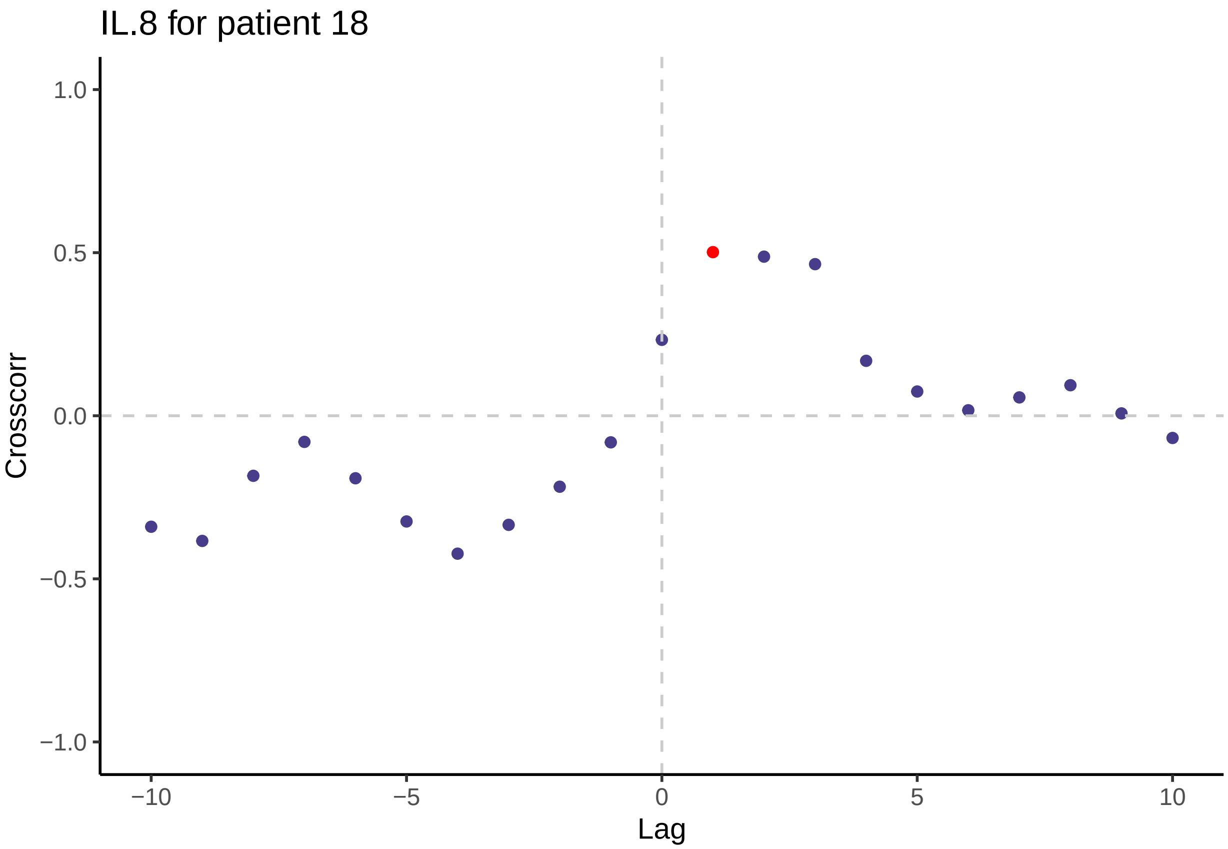


```
## [1] "IL.8 for patient 18 - p-value: 0.688778443121665"
```

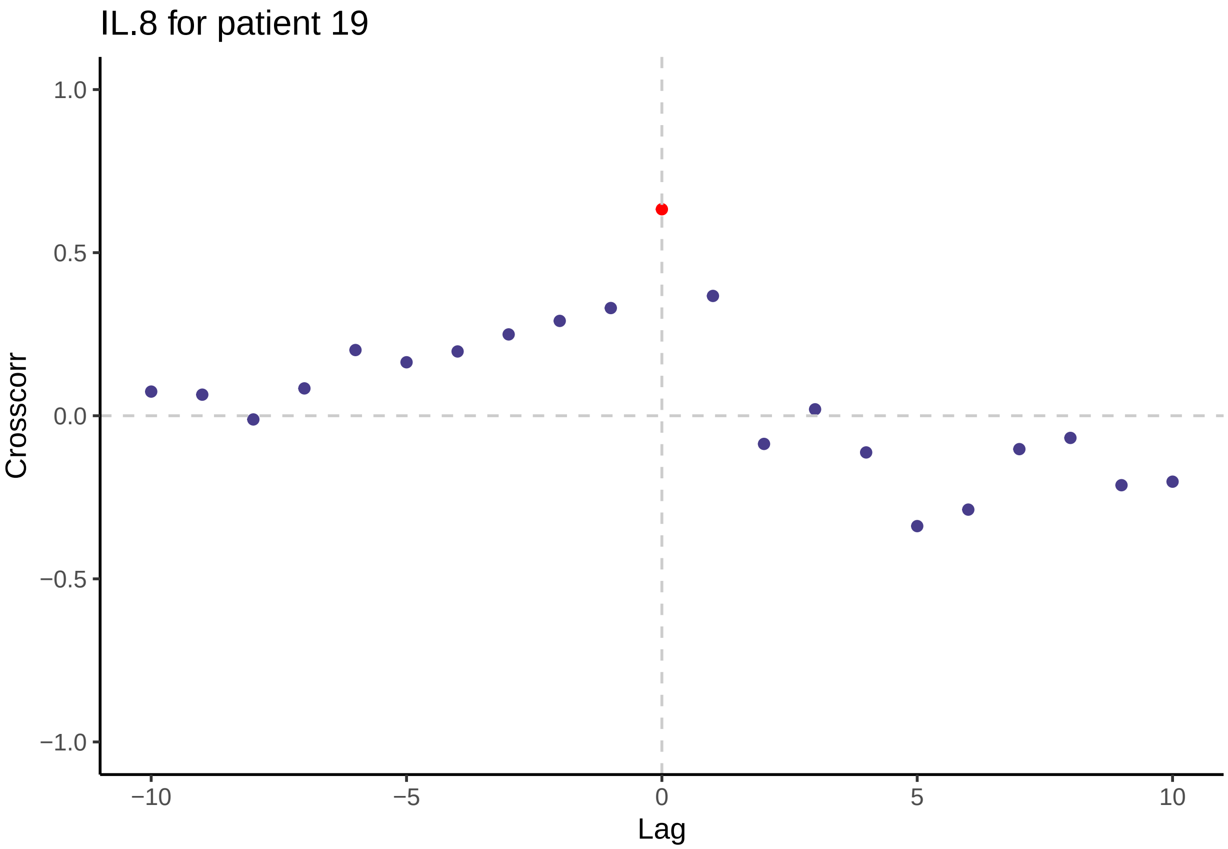


```
## [1] "IL.8 for patient 19 - p-value: 0.268832596066694"
```

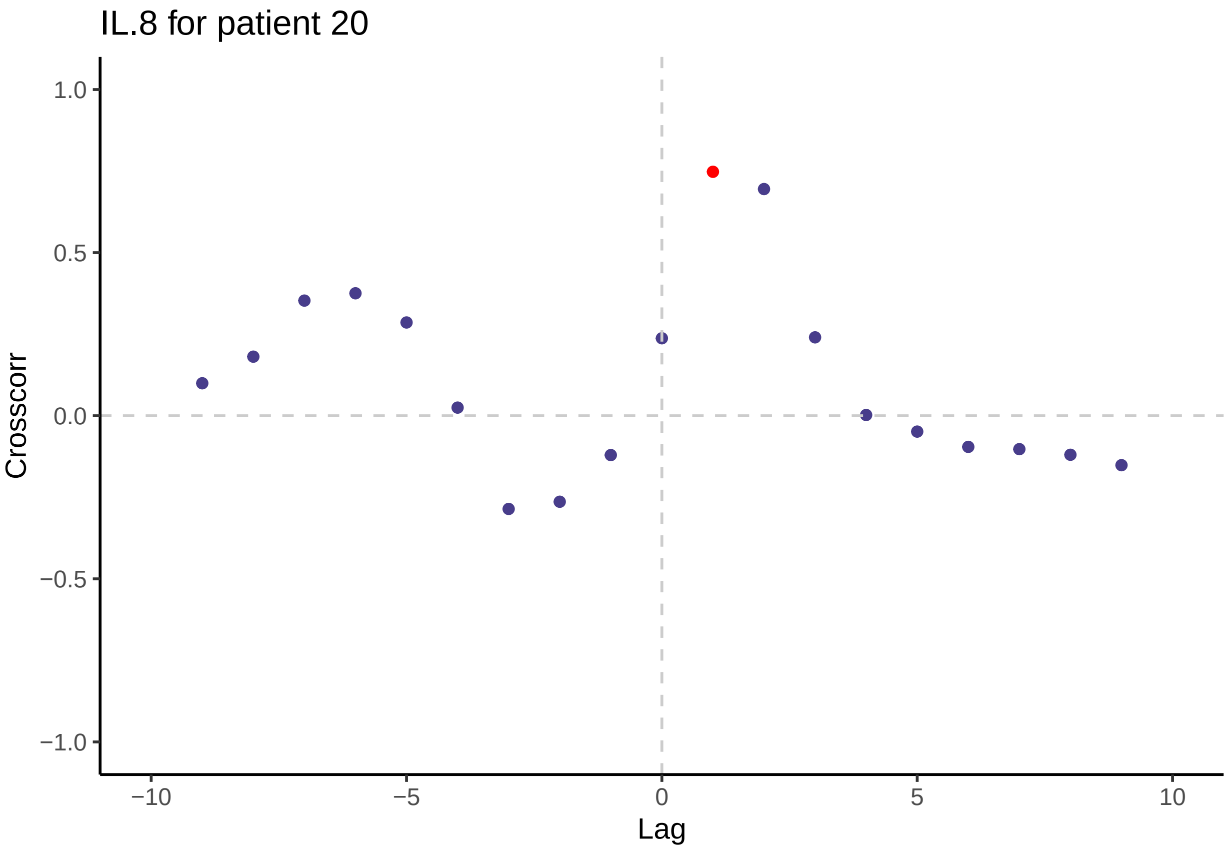


```
## [1] "IL.8 for patient 20 - p-value: 0.125319404472271"
```

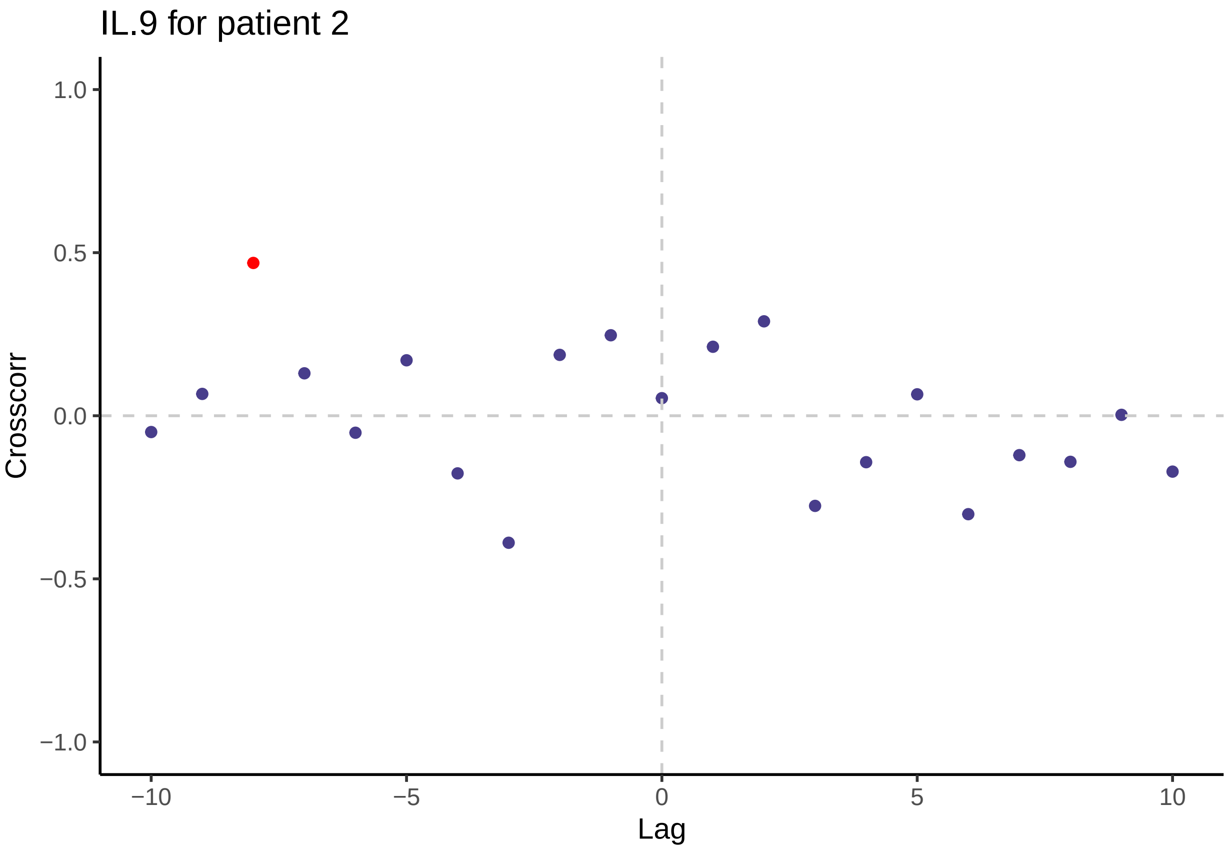


```
## [1] "IL.9 for patient 2 - p-value: 0.943982475617428"
```

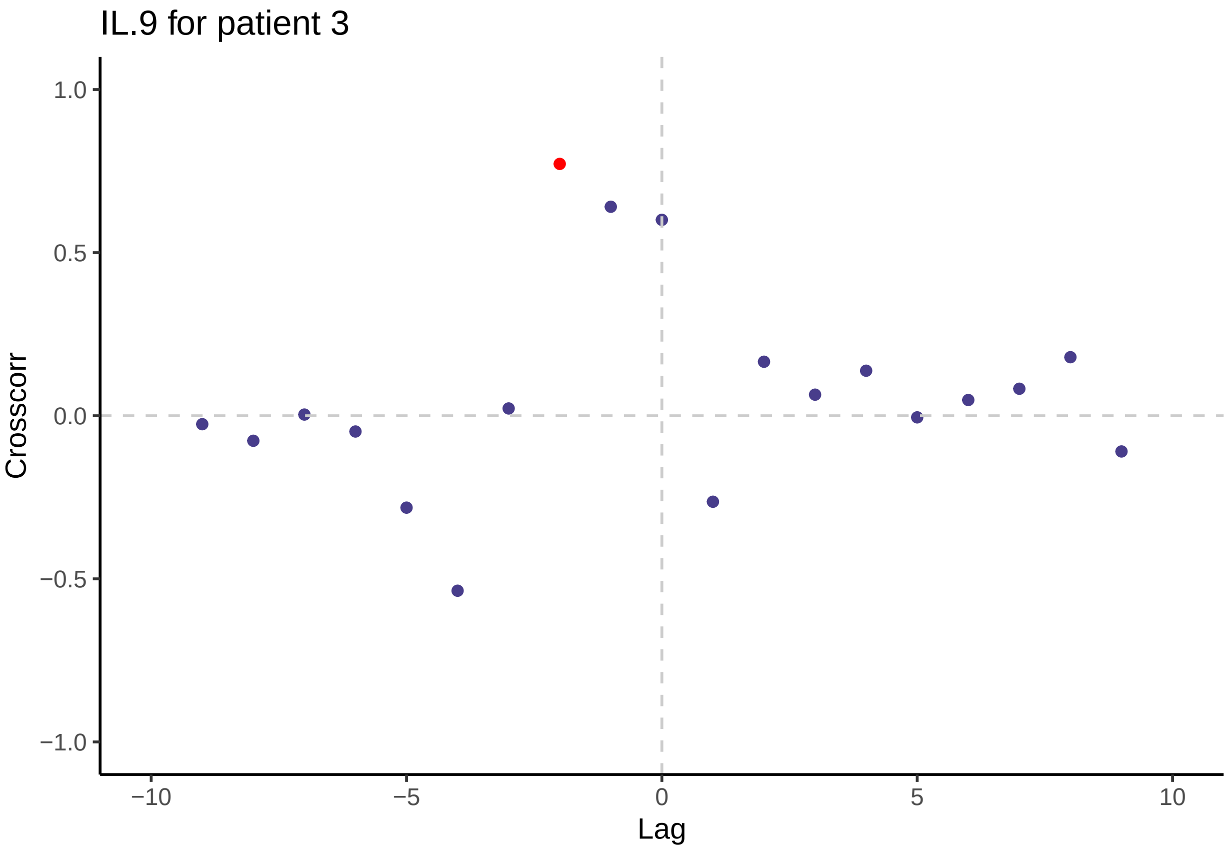


```
## [1] "IL.9 for patient 3 - p-value: 0.335302202095664"
```

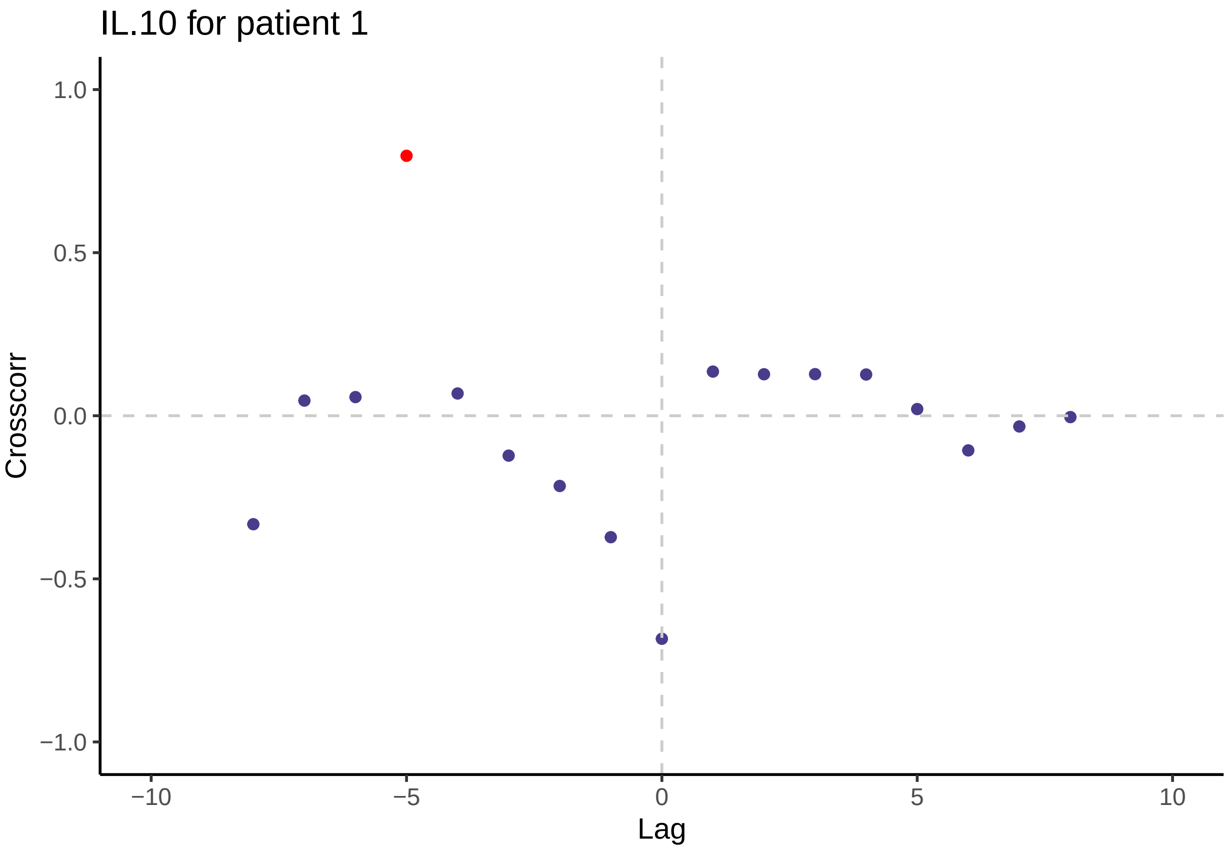


```
## [1] "IL.10 for patient 1 - p-value: 0.777052930727755"
```

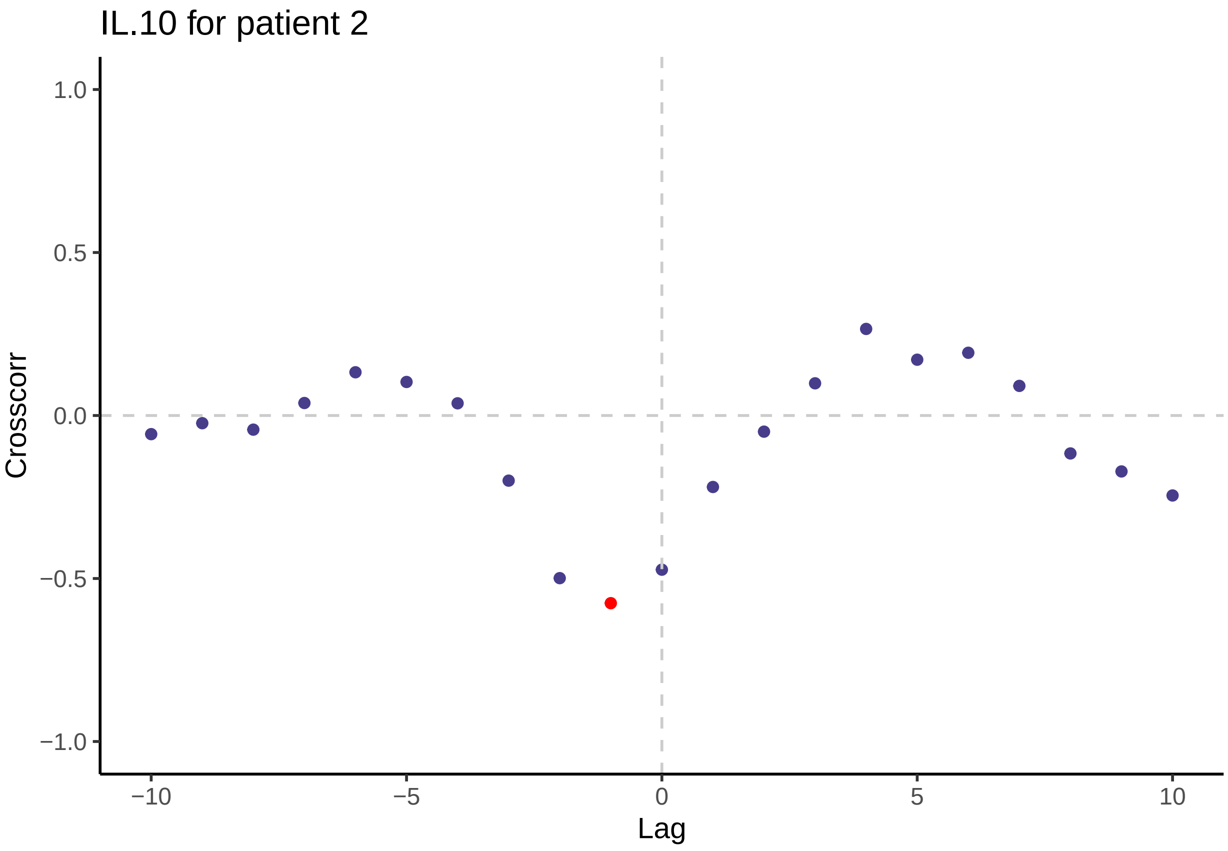


```
## [1] "IL.10 for patient 2 - p-value: 0.162029576741714"
```

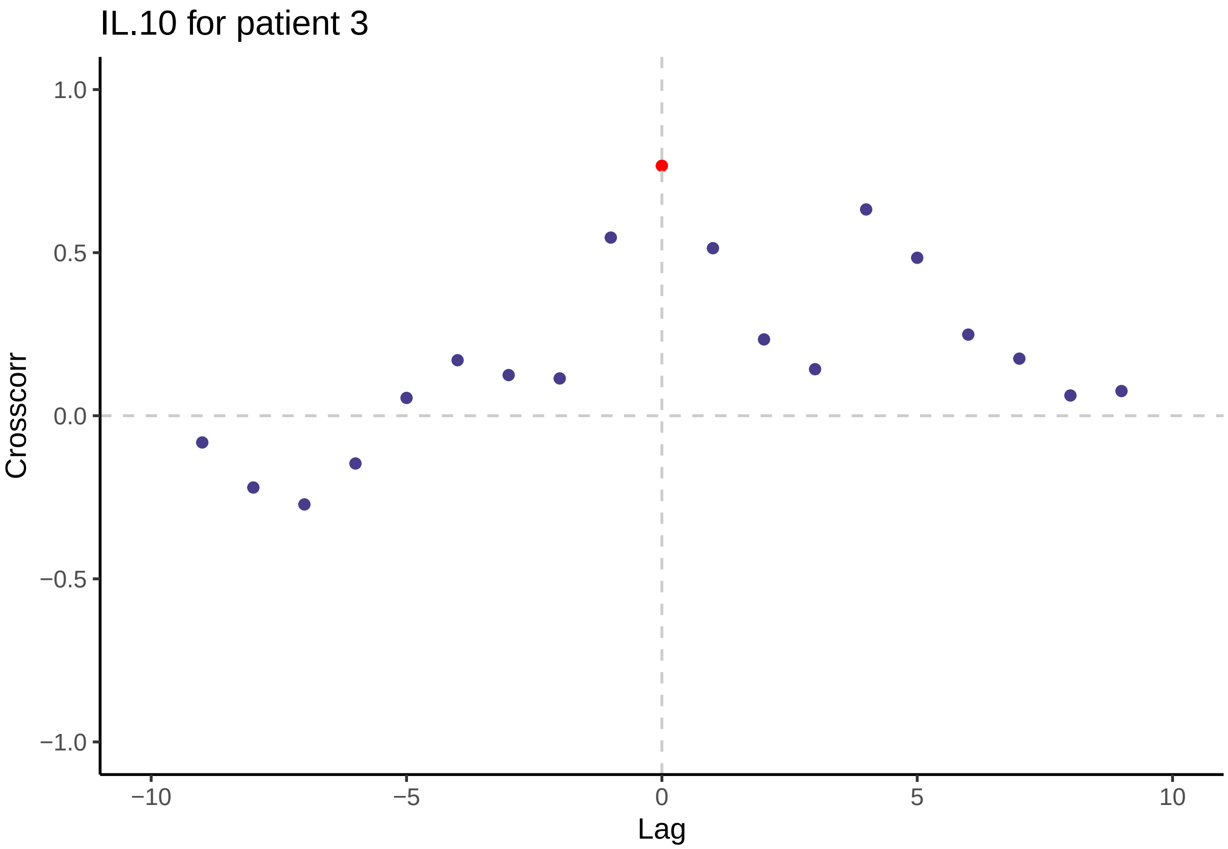


```
## [1] "IL.10 for patient 3 - p-value: 0.00954406432949252"
```

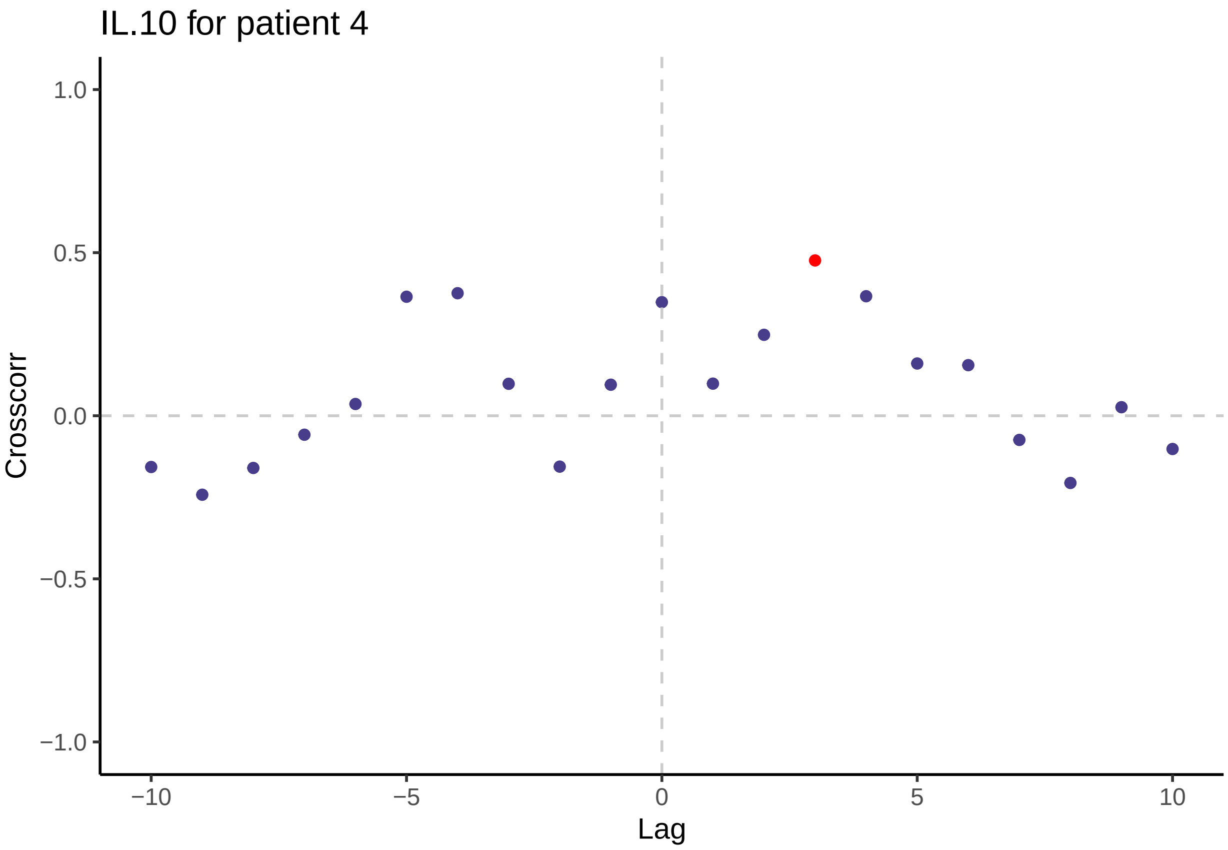


```
## [1] "IL.10 for patient 4 - p-value: 0.106553063505966"
```

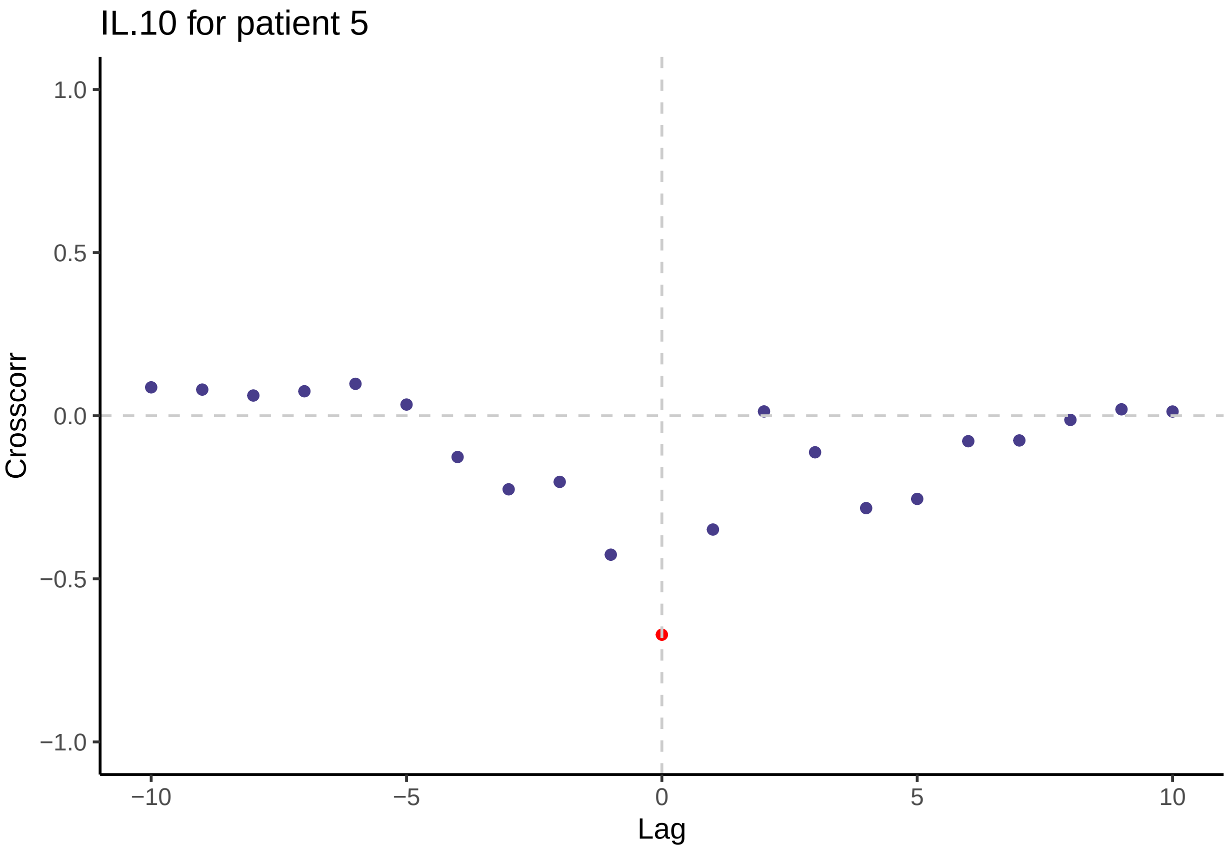


```
## [1] "IL.10 for patient 5 - p-value: 0.0196213587401657"
```

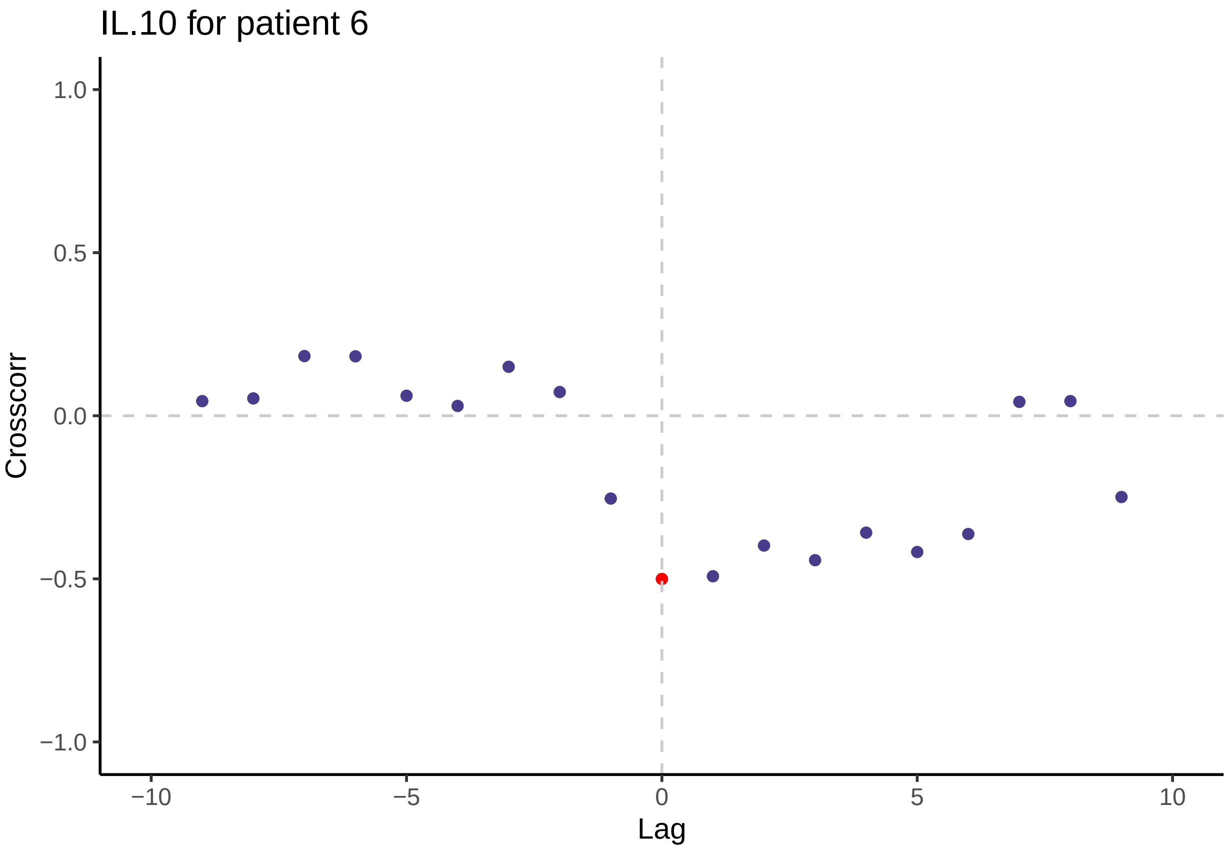


```
## [1] "IL.10 for patient 6 - p-value: 0.0296732735786476"
```

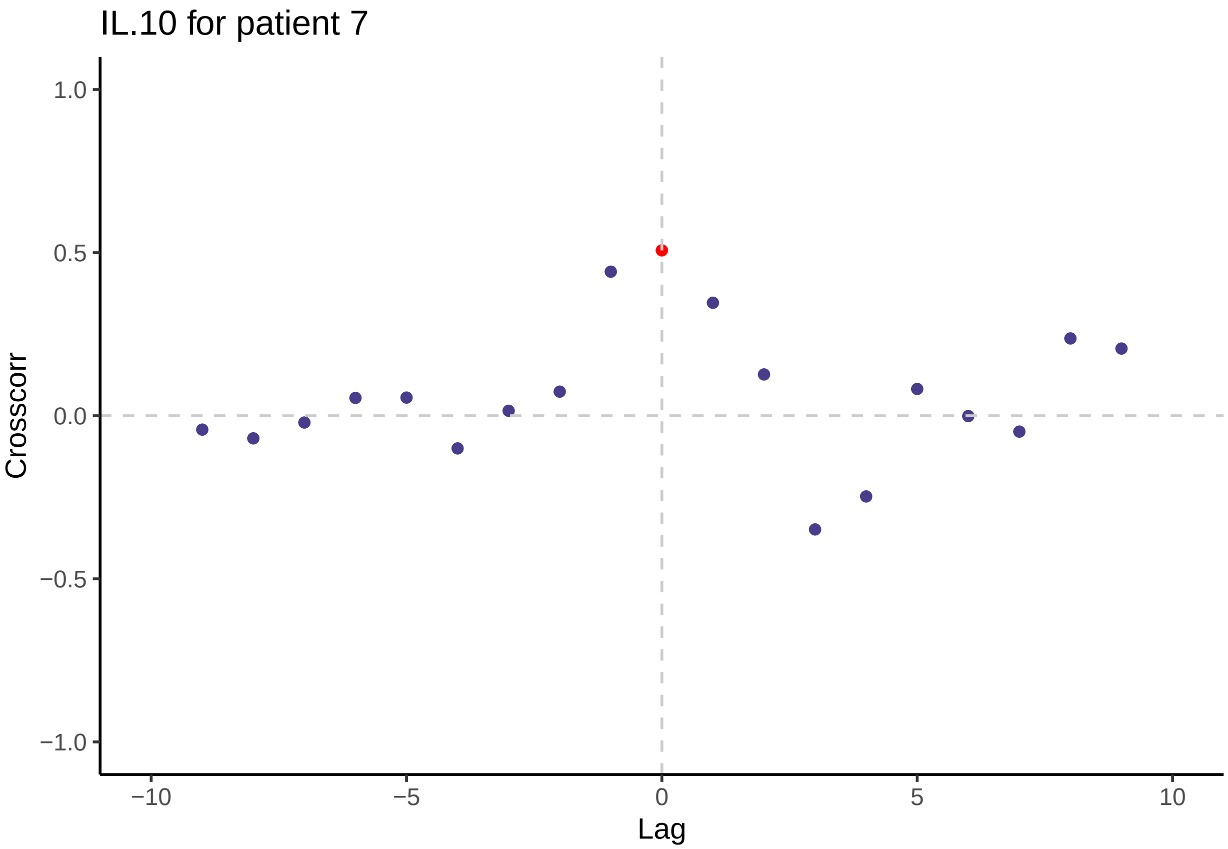


```
## [1] "IL.10 for patient 7 - p-value: 0.190310755245989"
```

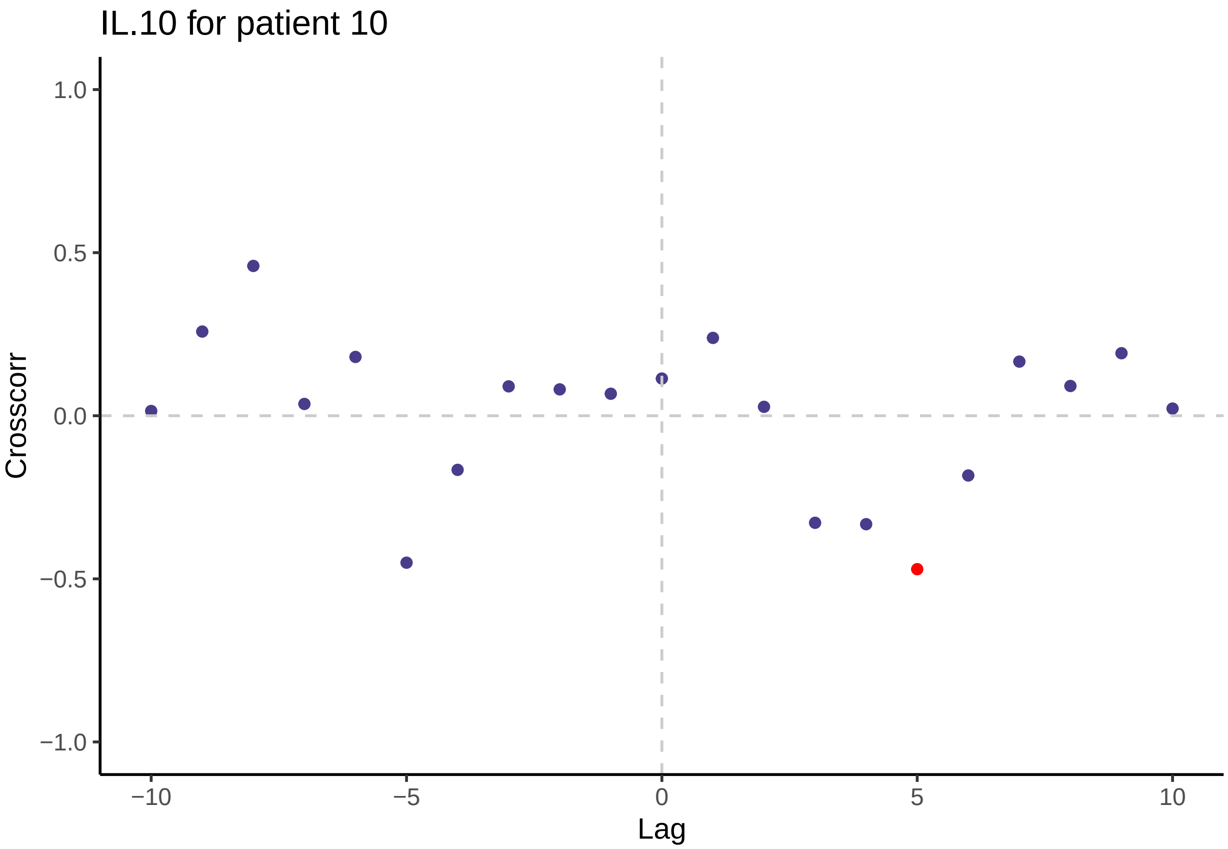


```
## [1] "IL.10 for patient 10 - p-value: 0.923654307557647"
```

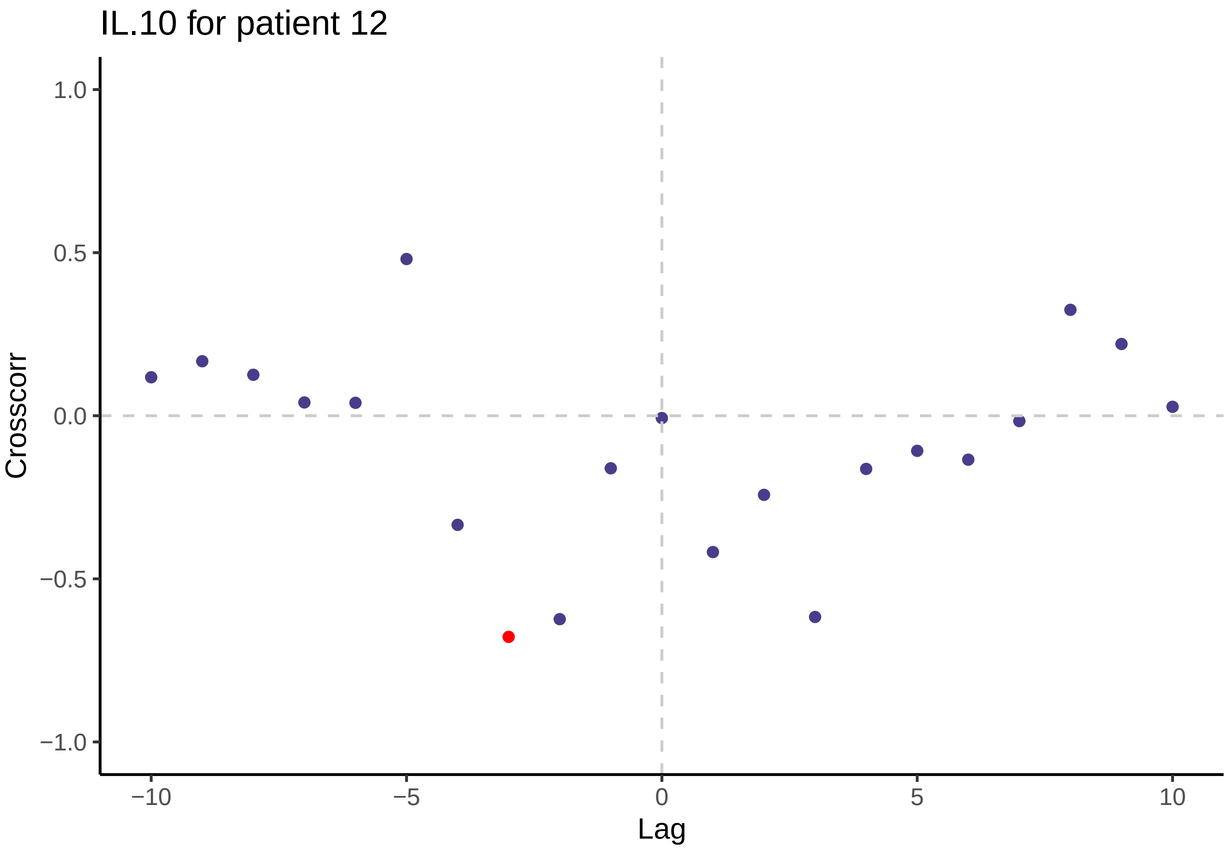


```
## [1] "IL.10 for patient 12 - p-value: 0.182945115855309"
```

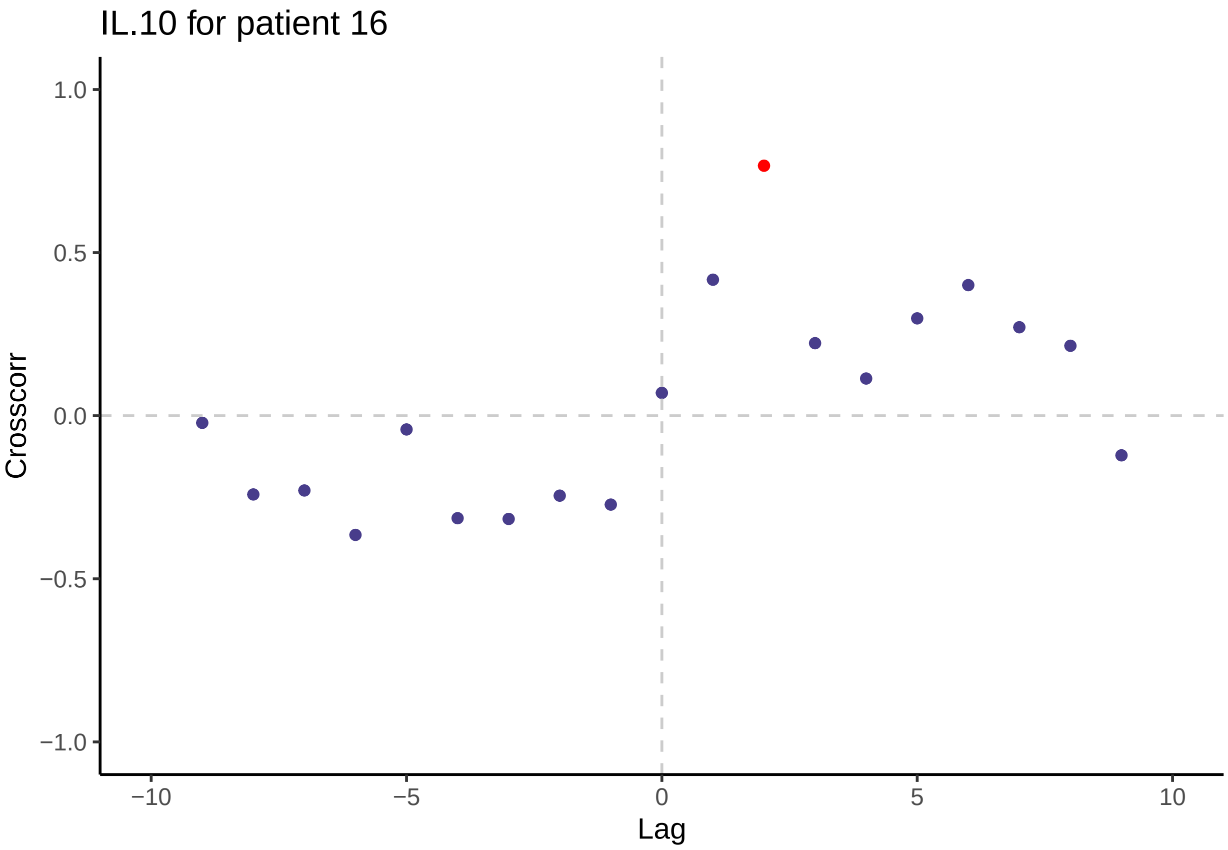


```
## [1] "IL.10 for patient 16 - p-value: 0.662569403165751"
```

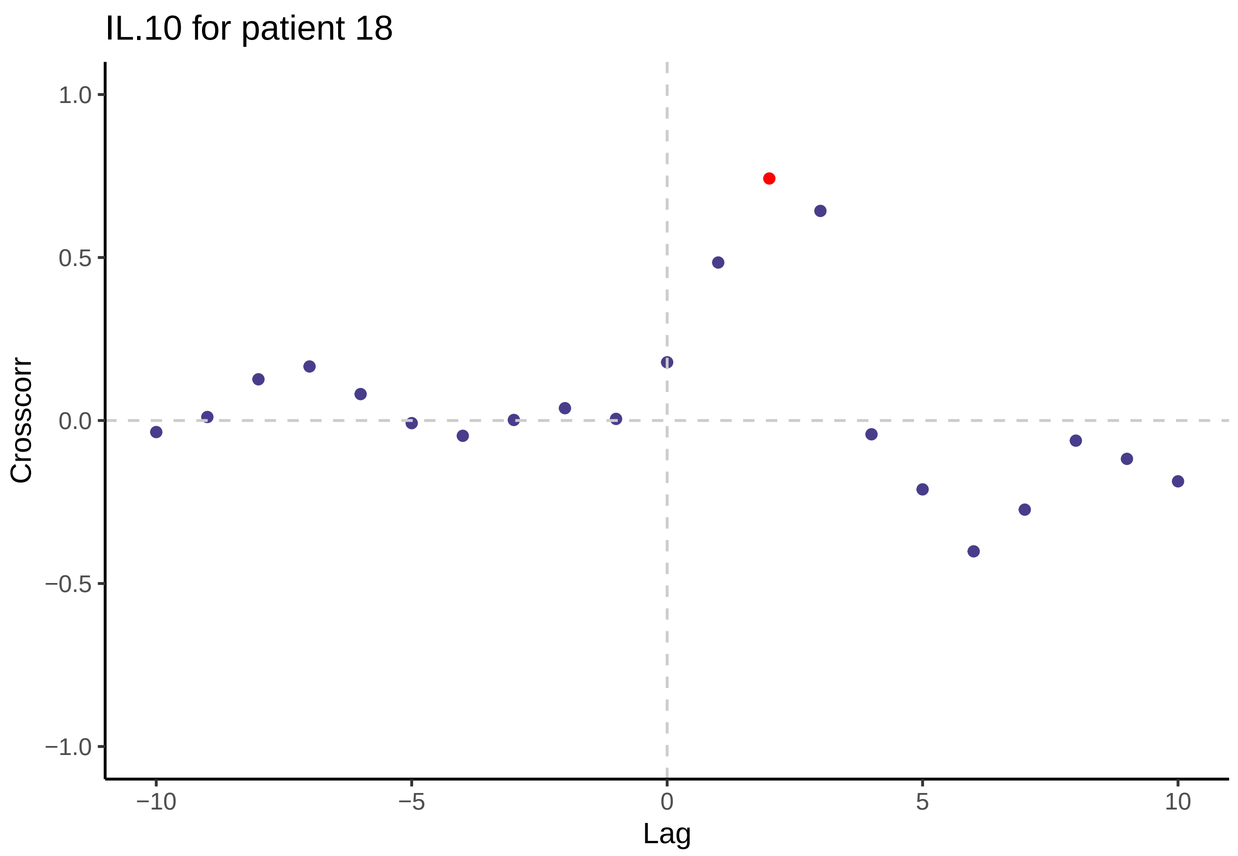


```
## [1] "IL.10 for patient 18 - p-value: 0.404043775019719"
```

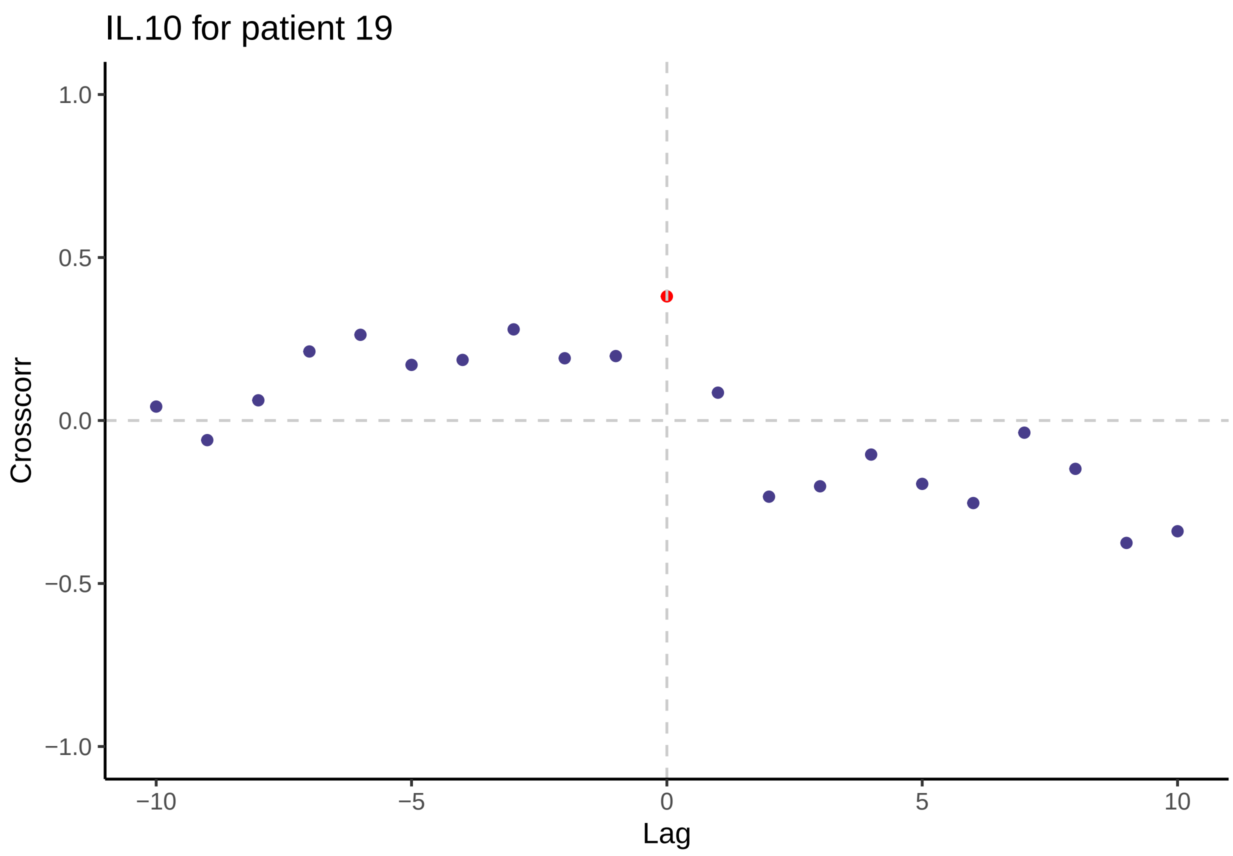


```
## [1] "IL.10 for patient 19 - p-value: 0.905749944112402"
## Warning: Removed 5 rows containing missing values (geom_point).
```

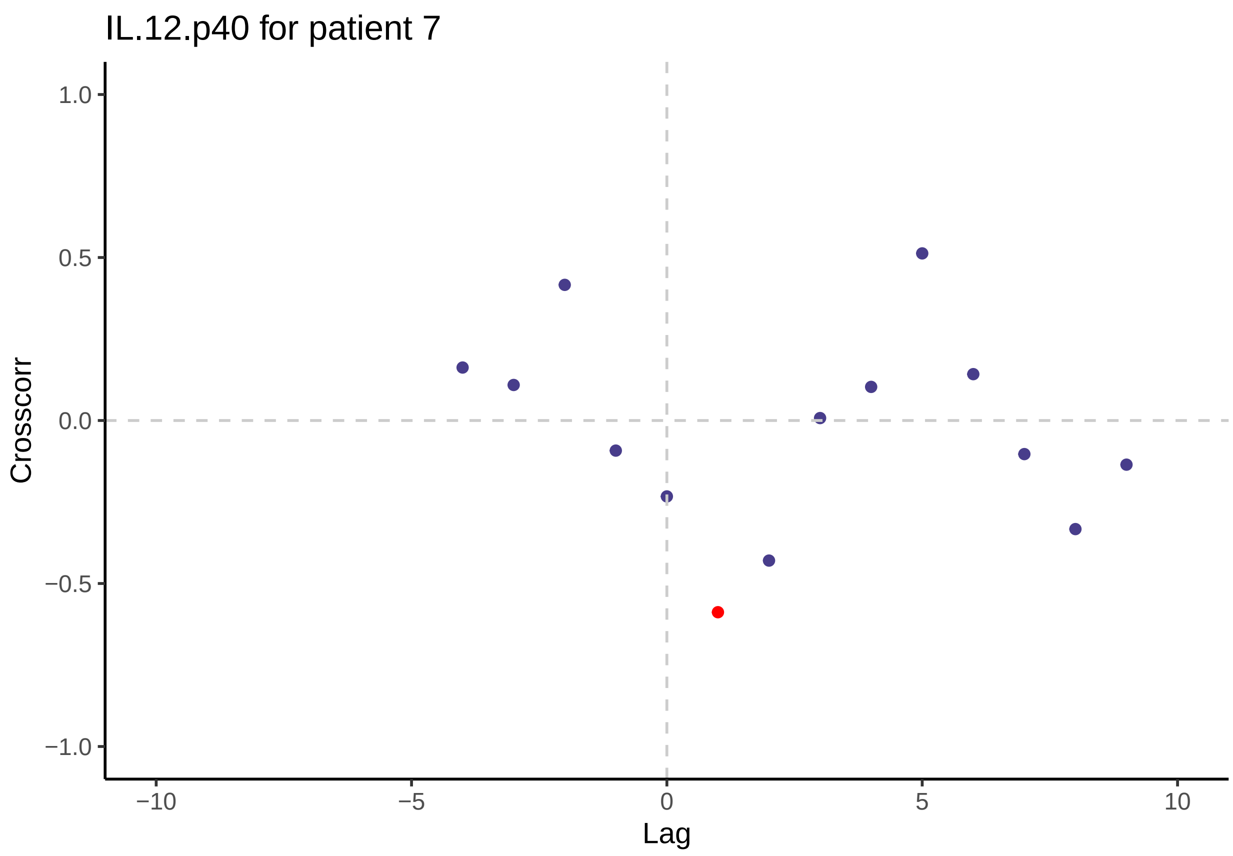


```
## [1] "IL.12.p40 for patient 7 - p-value: 0.694251649504261"
```

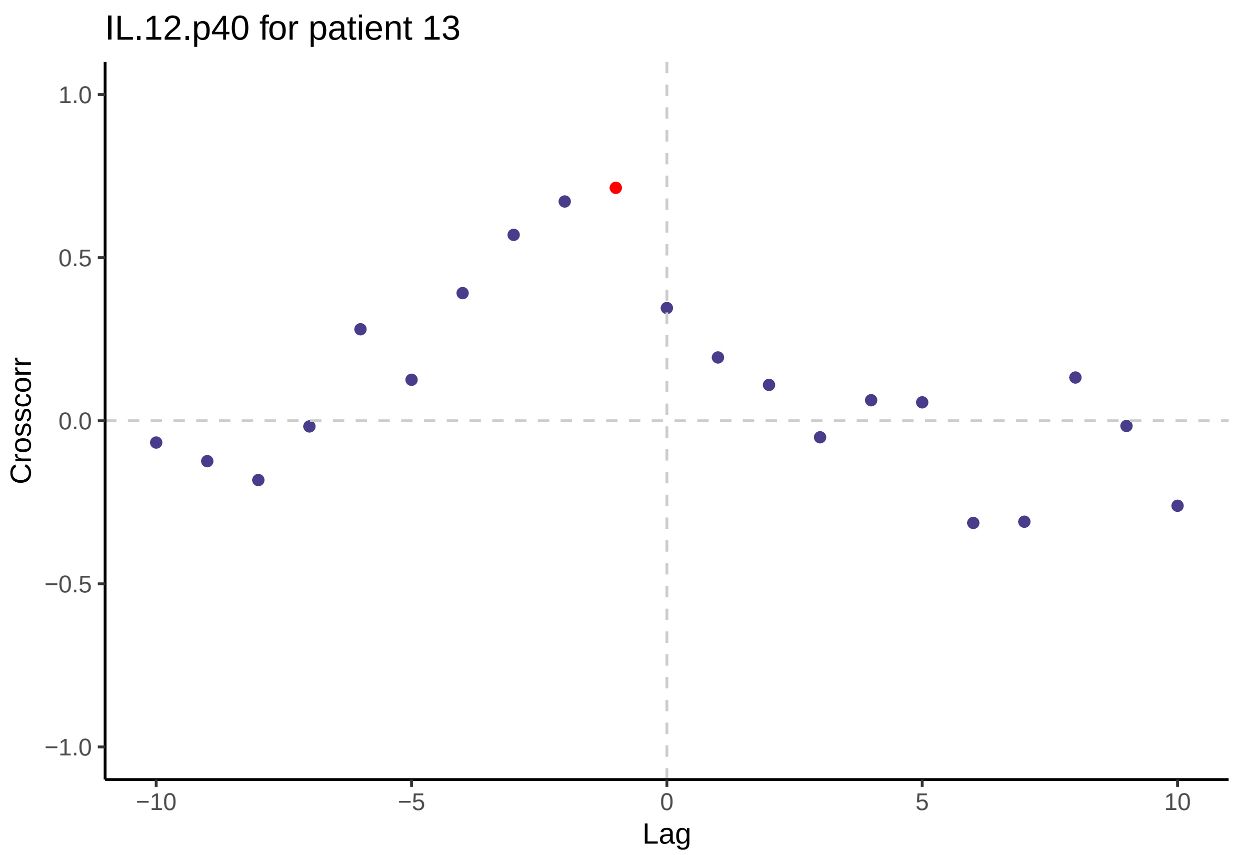


```
## [1] "IL.12.p40 for patient 13 - p-value: 0.106796701482695"
```

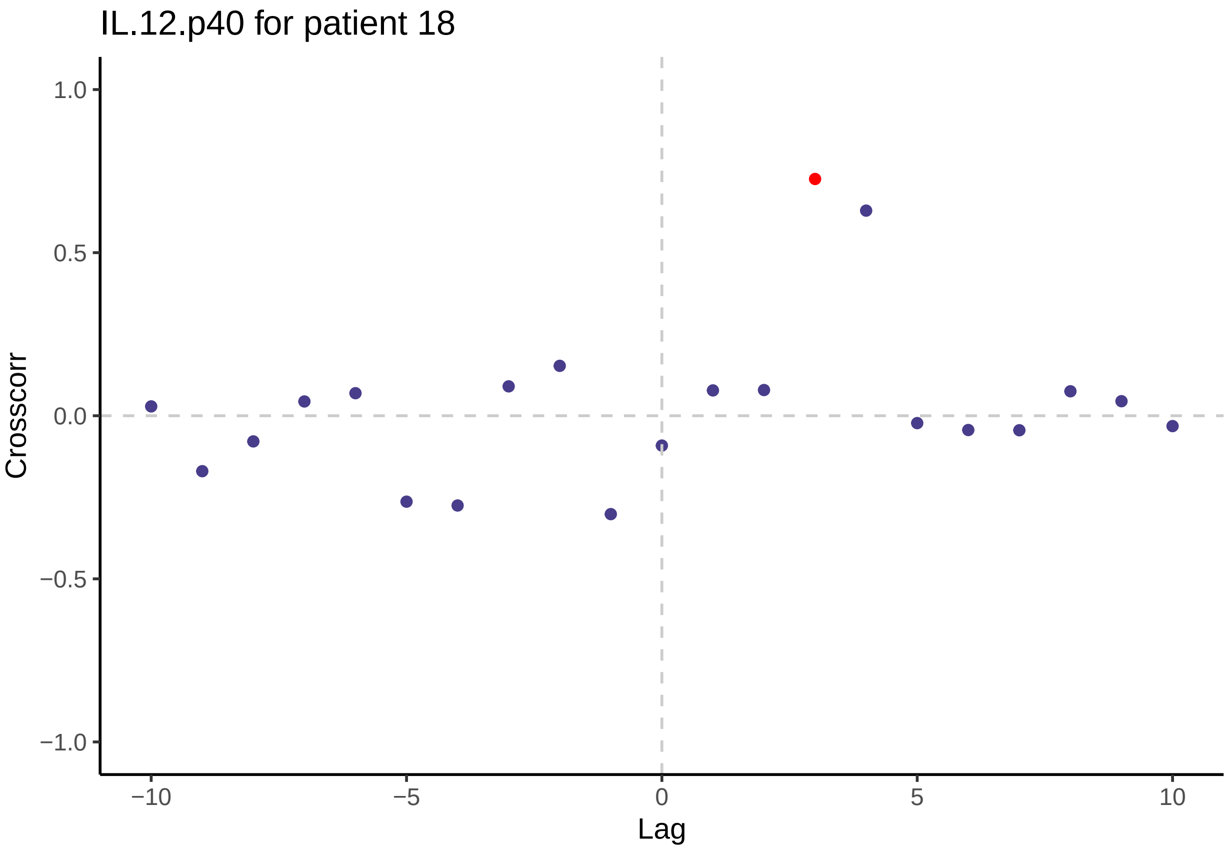


```
## [1] "IL.12.p40 for patient 18 - p-value: 0.5508424283298"
```

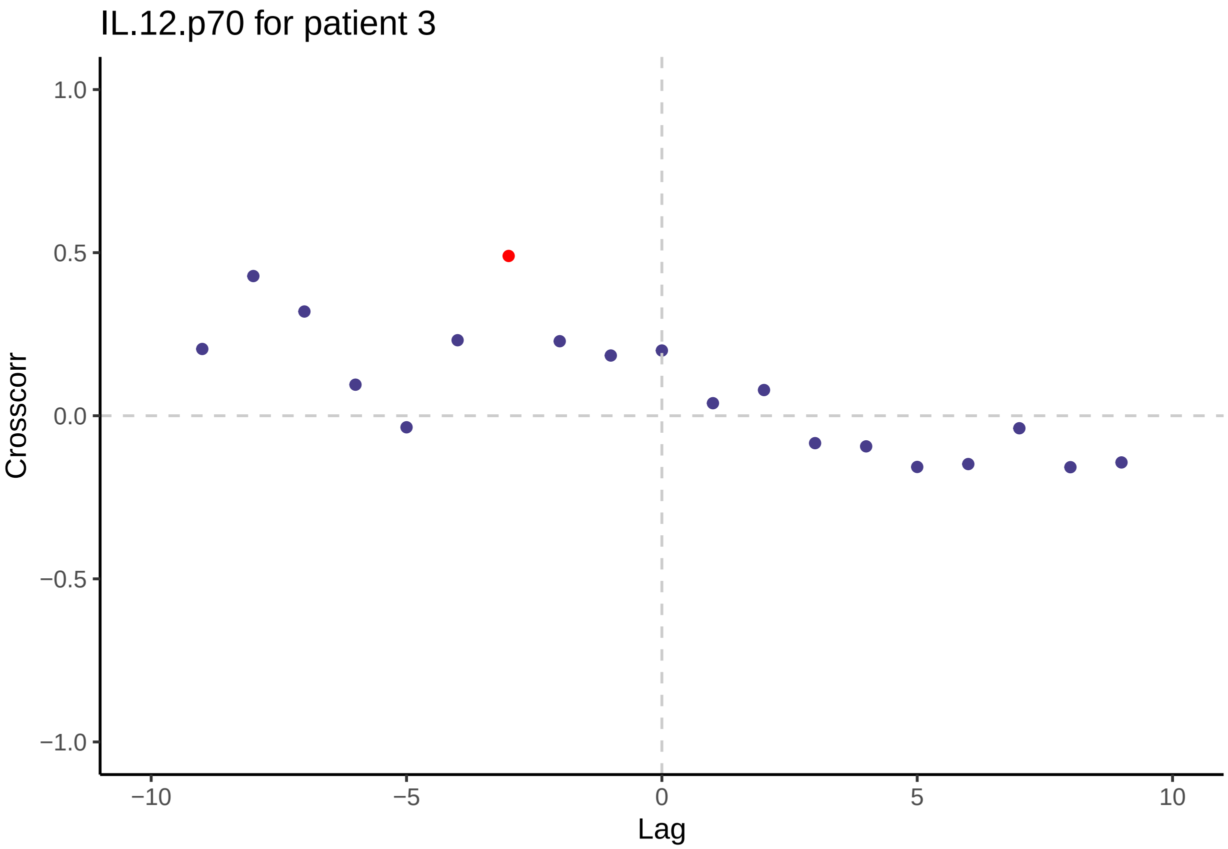


```
## [1] "IL.12.p70 for patient 3 - p-value: 0.0781274462950831"
```

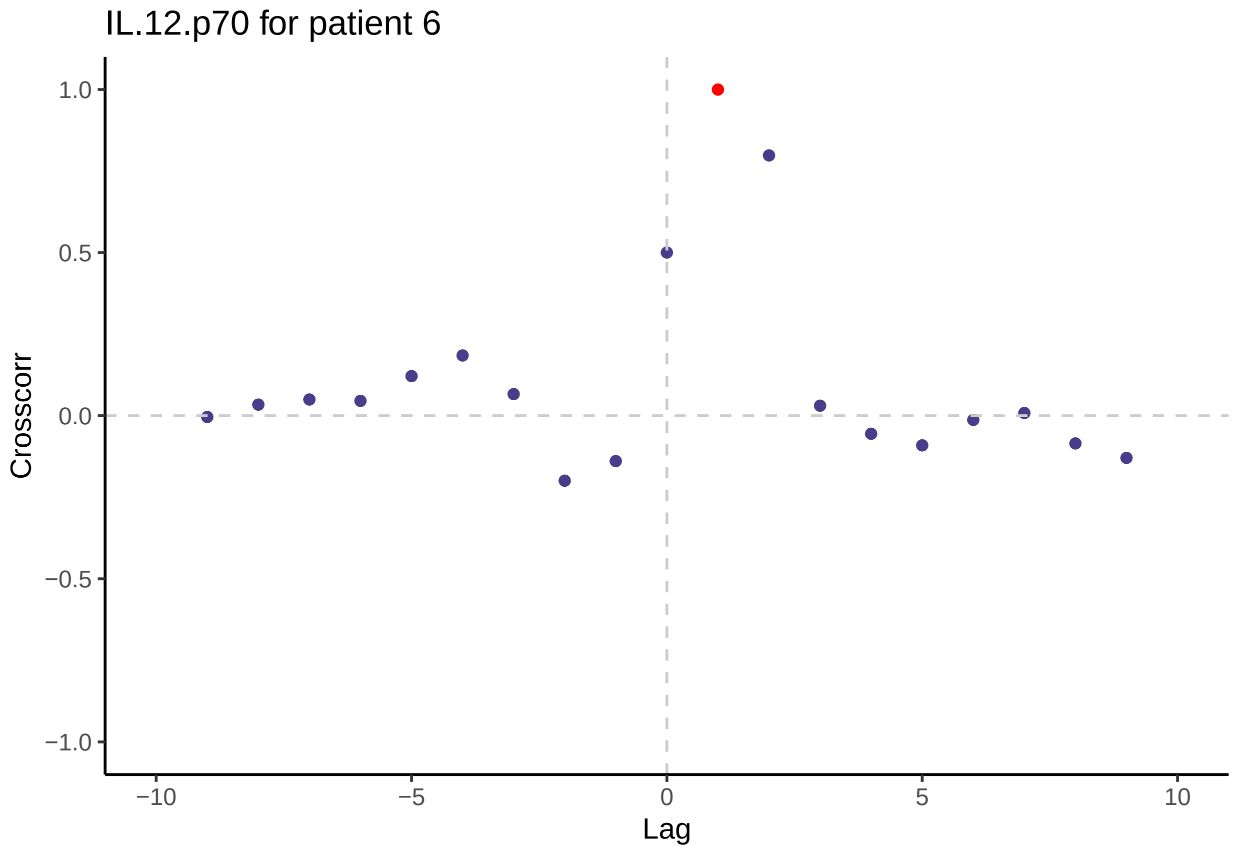


```
## [1] "IL.12.p70 for patient 6 - p-value: 0.140917885110438"
```

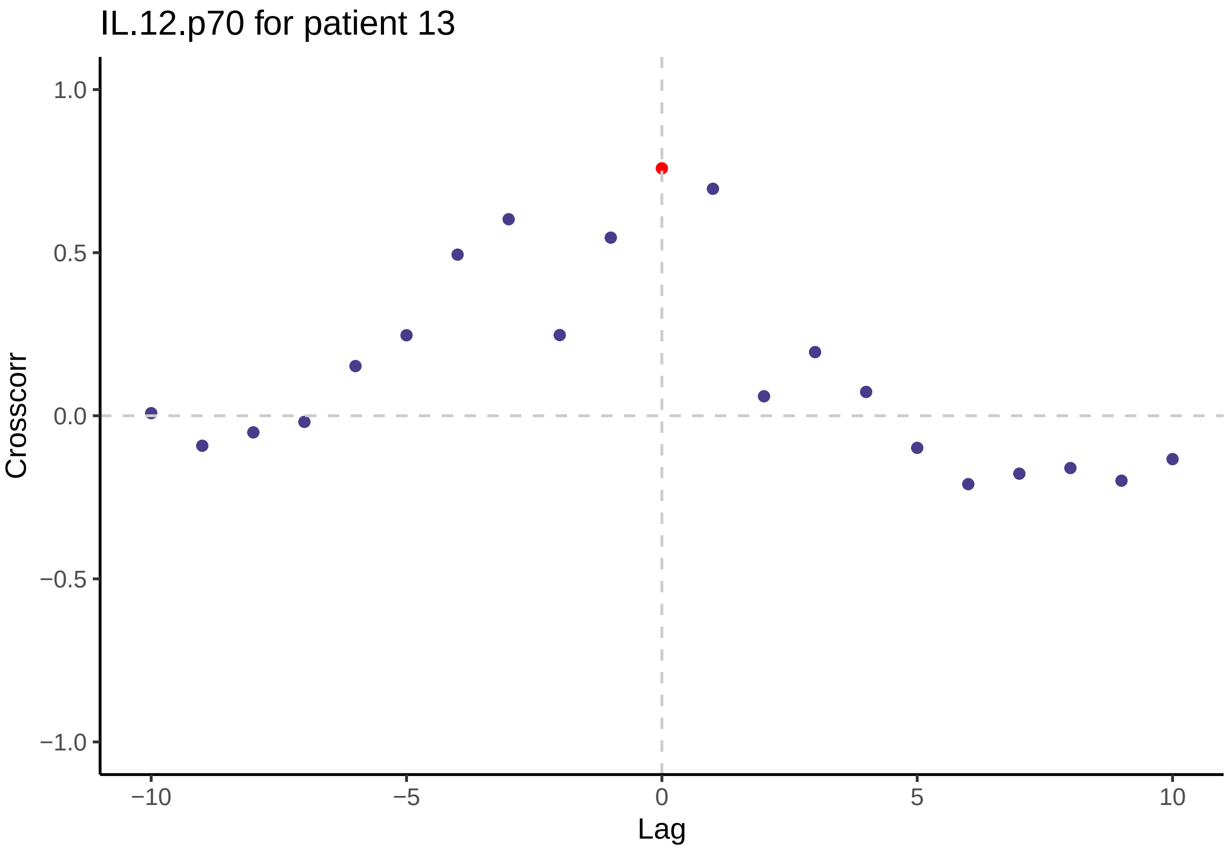


```
## [1] "IL.12.p70 for patient 13 - p-value: 0.0518663043168202"
```

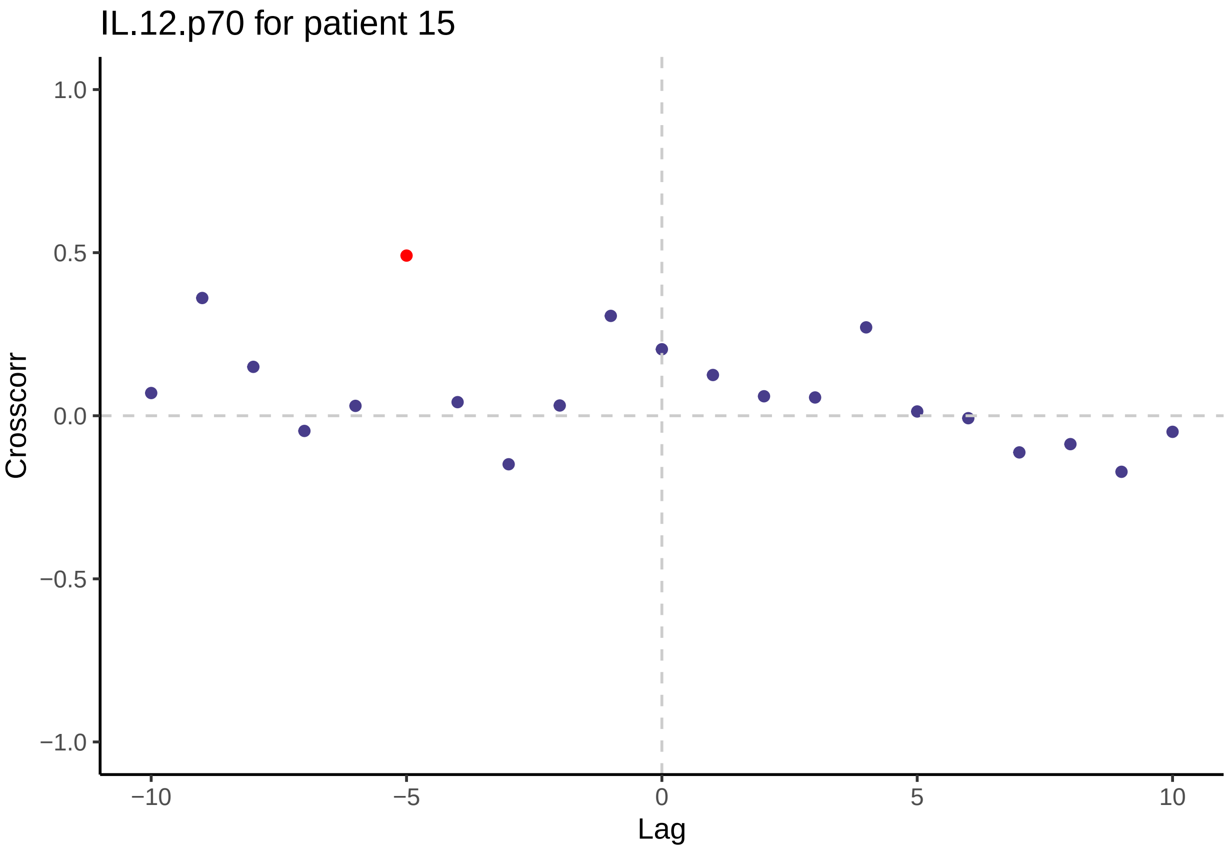


```
## [1] "IL.12.p70 for patient 15 - p-value: 0.0574647629138899"
```

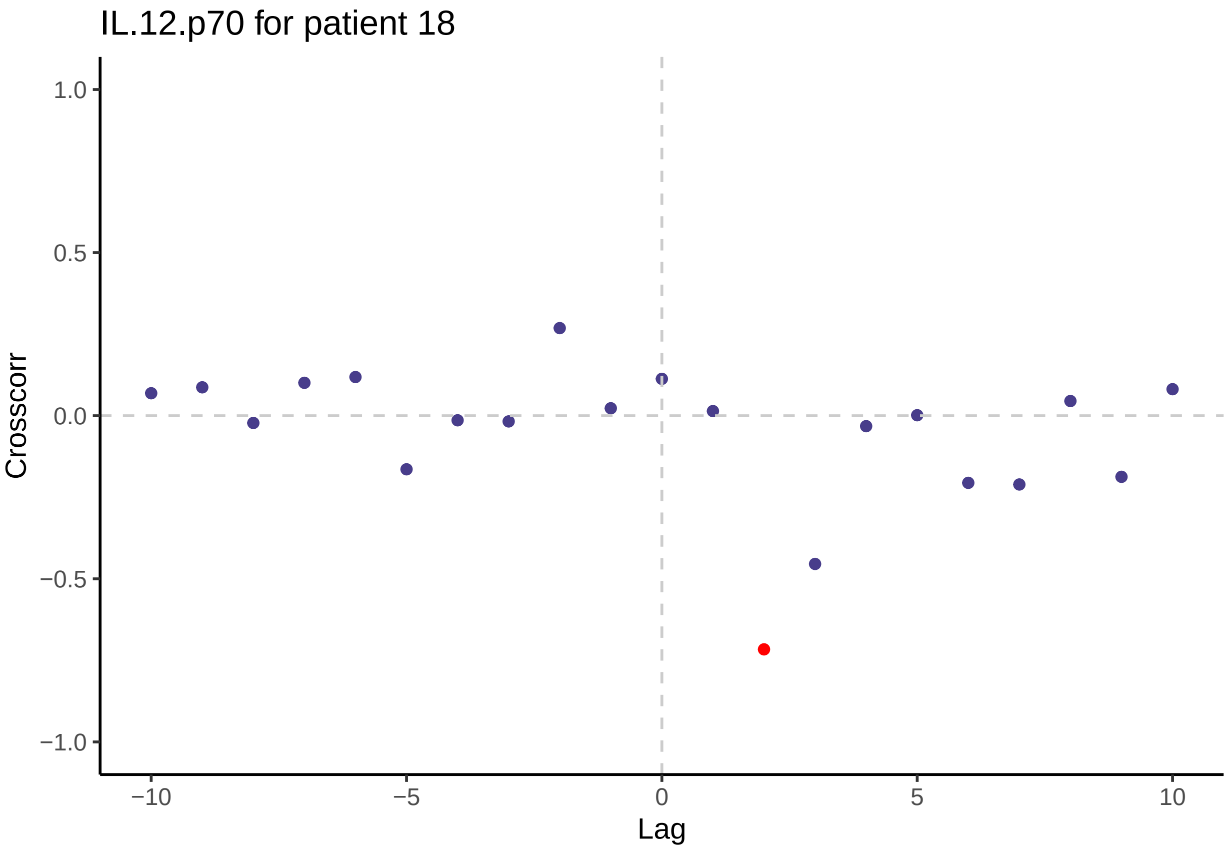


```
## [1] "IL.12.p70 for patient 18 - p-value: 0.281937281212826"
```

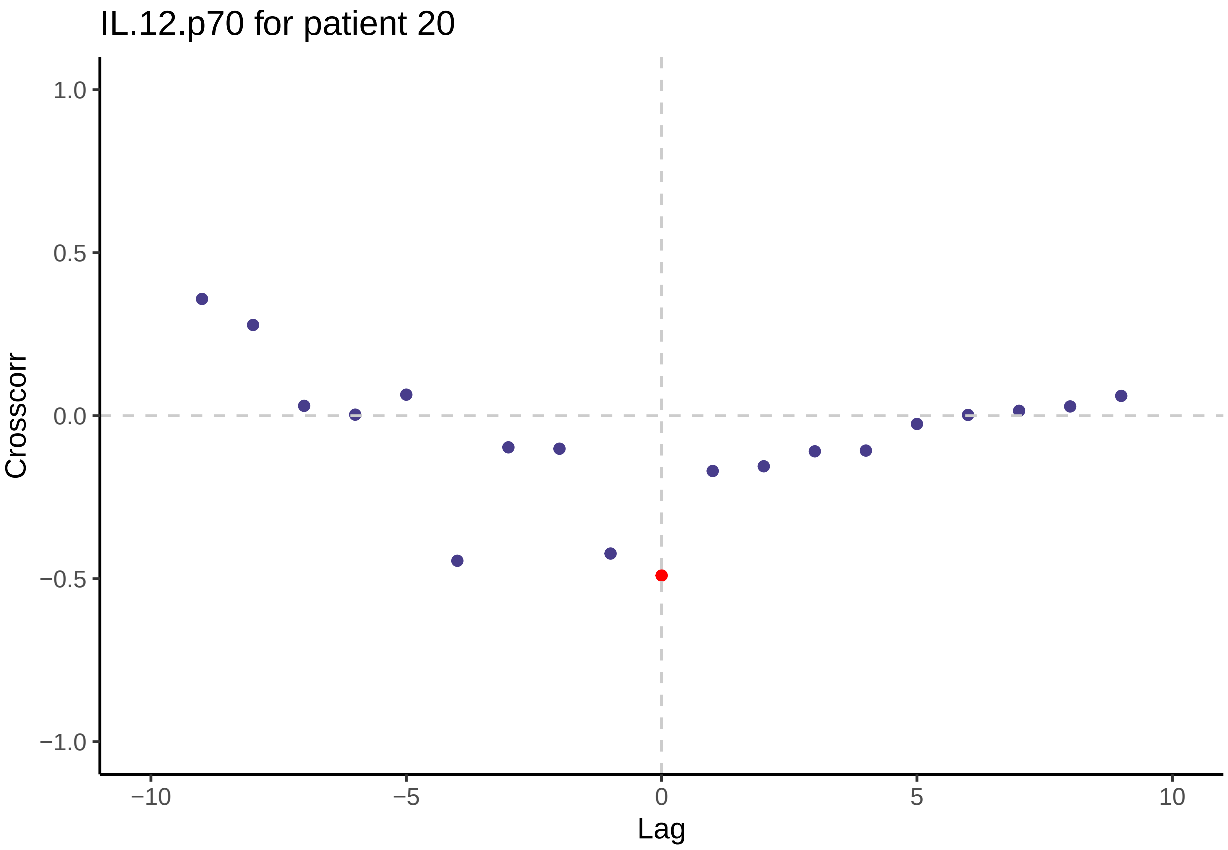


```
## [1] "IL.12.p70 for patient 20 - p-value: 0.192221430095954"
## Warning: Removed 2 rows containing missing values (geom_point).
```

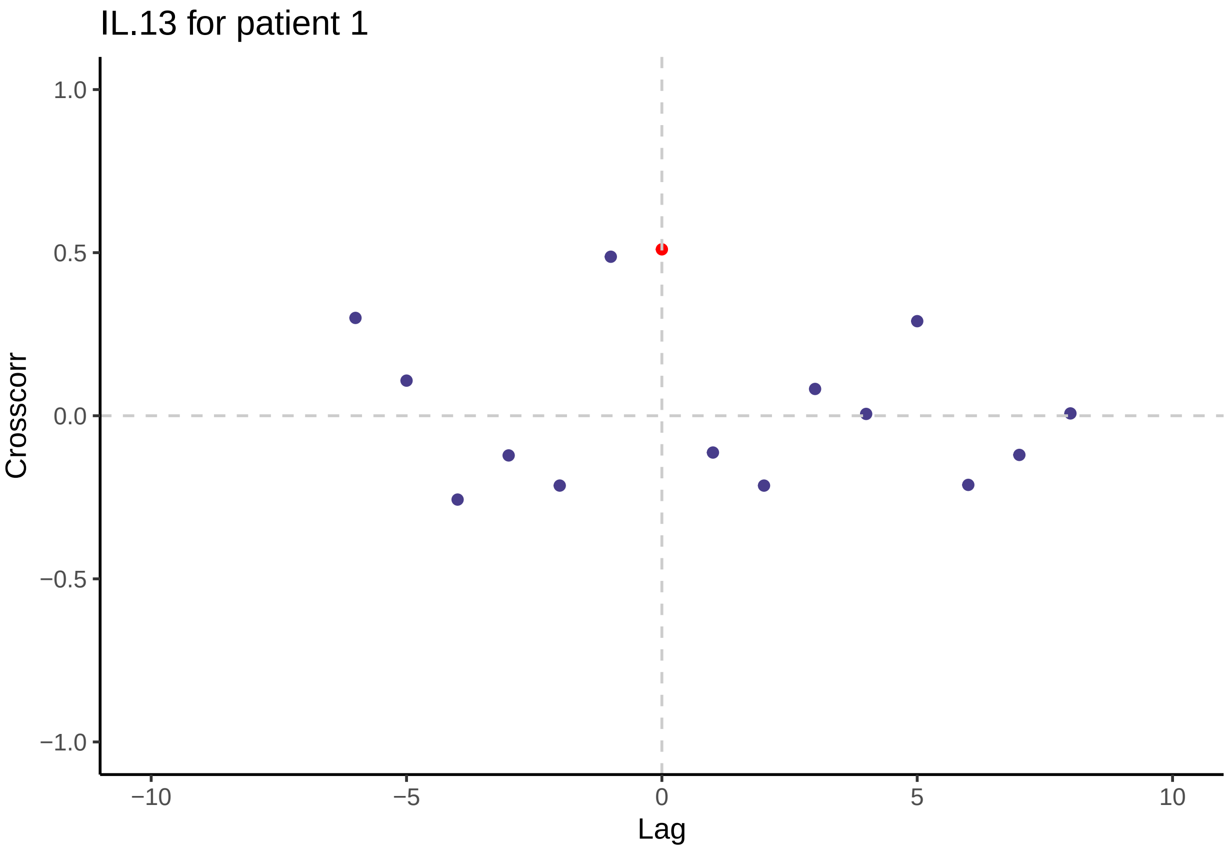


```
## [1] "IL.13 for patient 1 - p-value: 0.59497477616279"
```

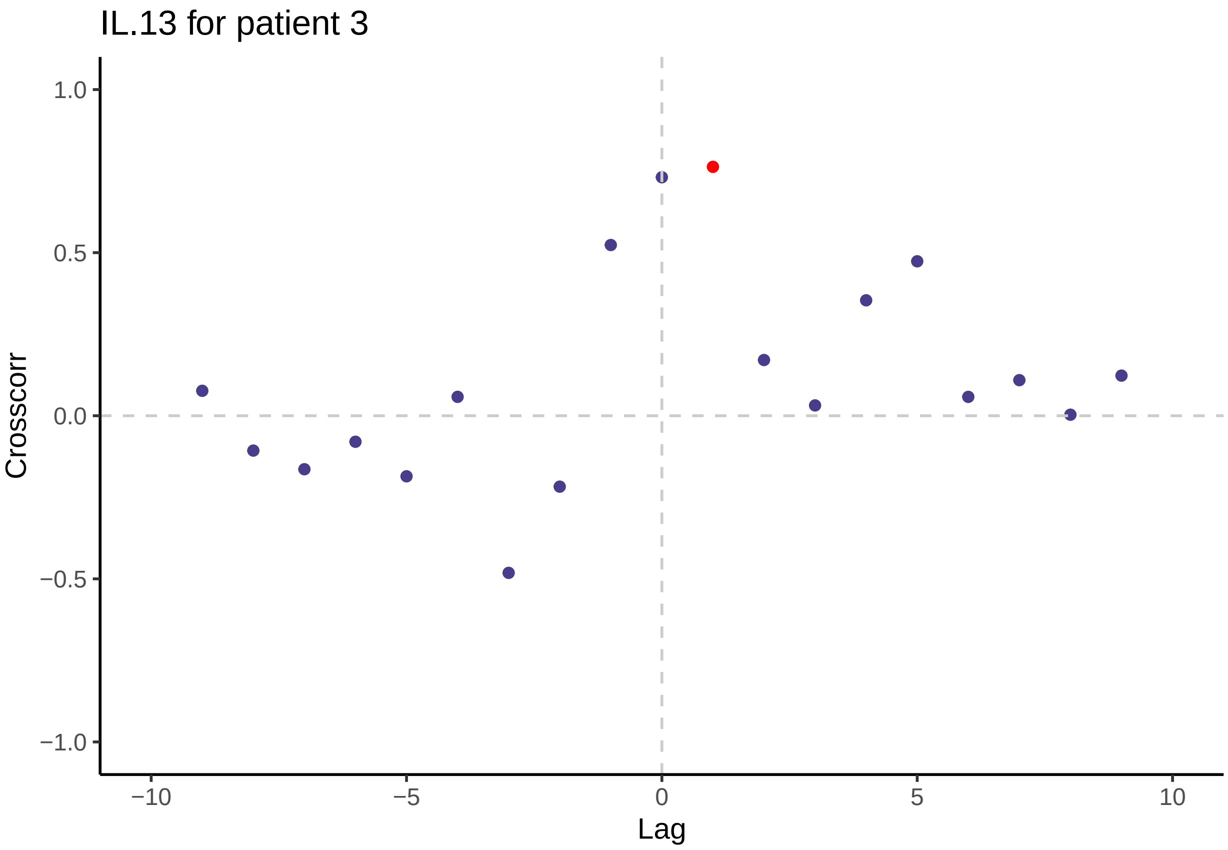


```
## [1] "IL.13 for patient 3 - p-value: 0.131405959822654"
## Warning: Removed 3 rows containing missing values (geom_point).
```

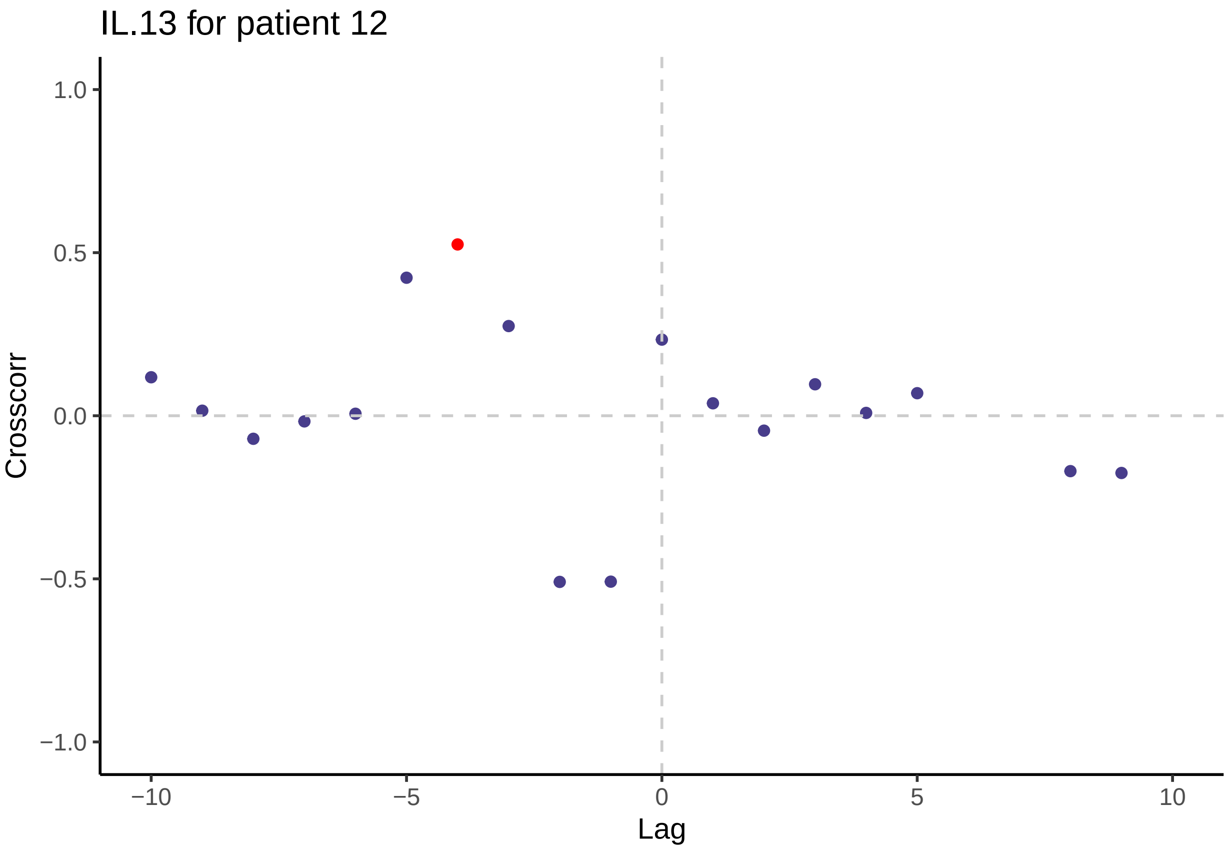


```
## [1] "IL.13 for patient 12 - p-value: 0.784635517462609"
```

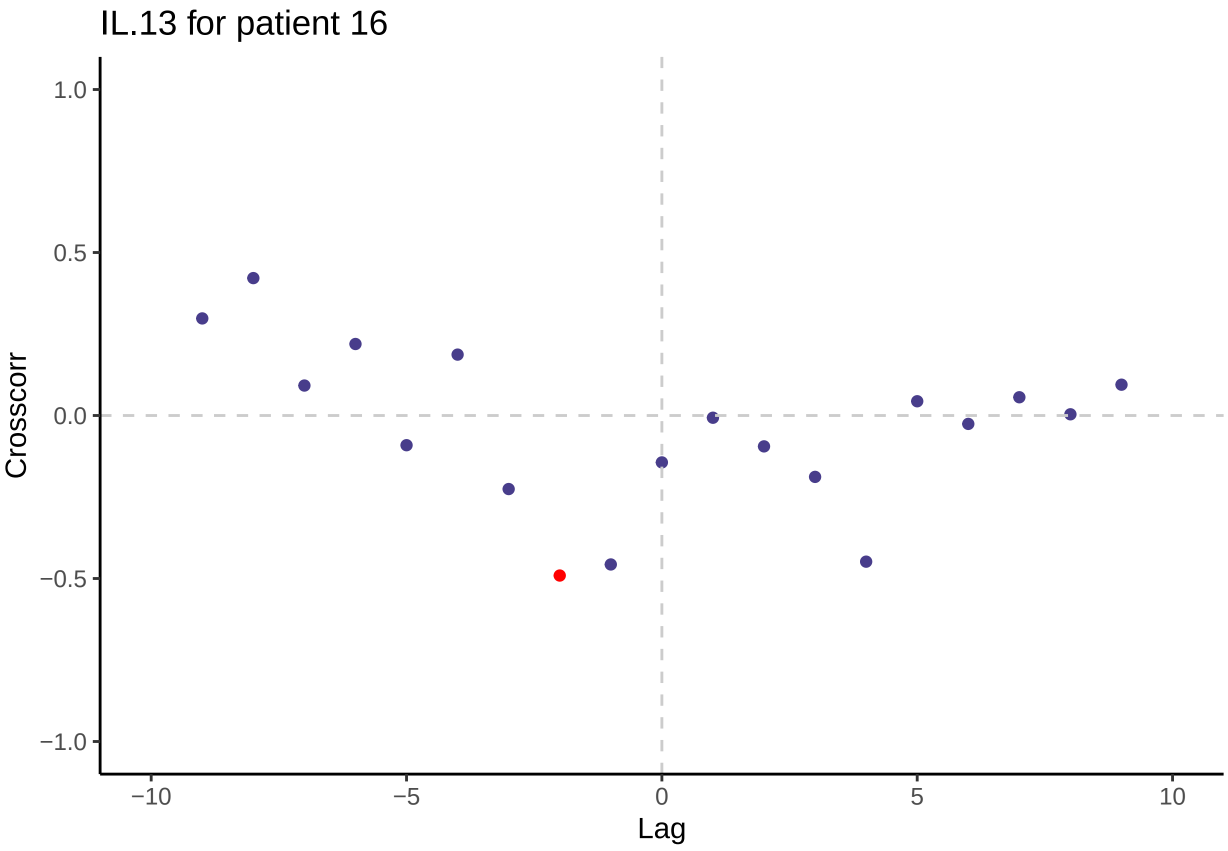


```
## [1] "IL.13 for patient 16 - p-value: 0.494858588414717"
```

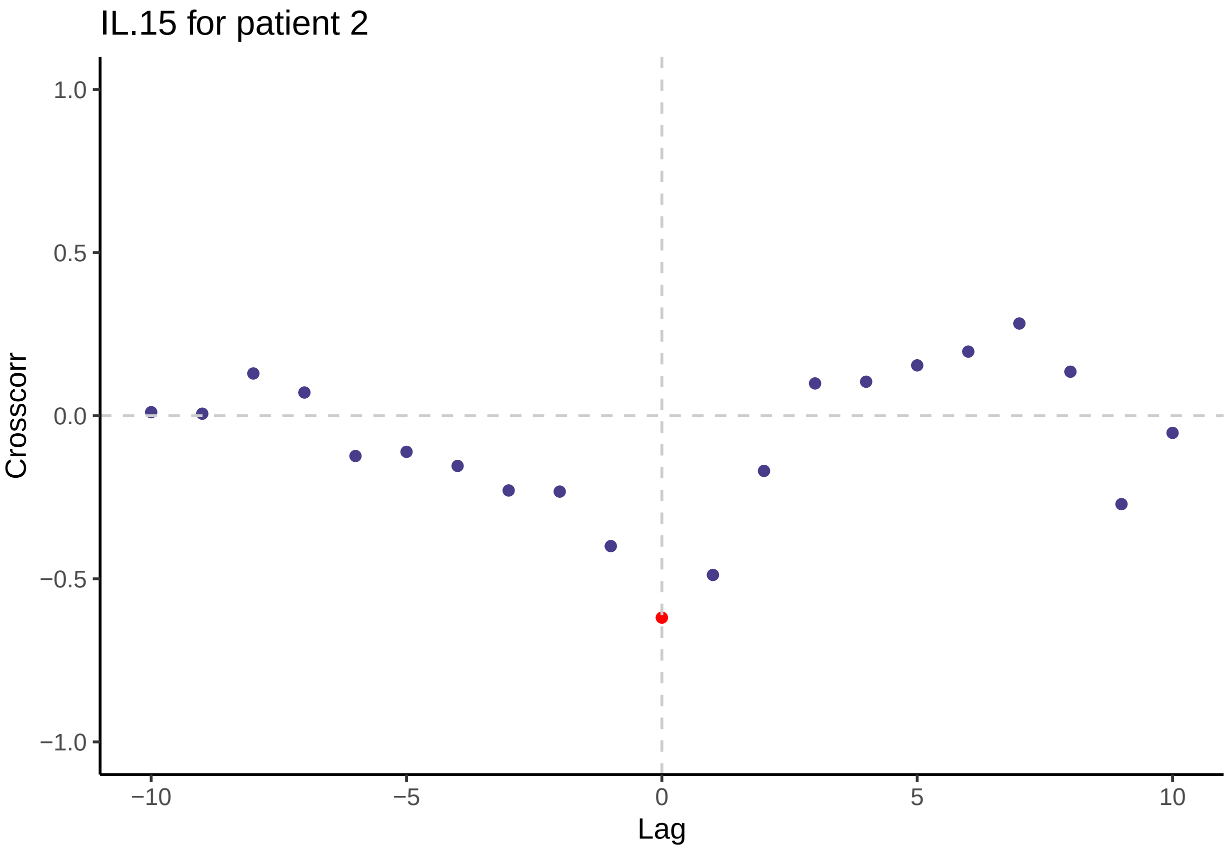


```
## [1] "IL.15 for patient 2 - p-value: 0.141273908777531"
## Warning: Removed 1 rows containing missing values (geom_point).
```

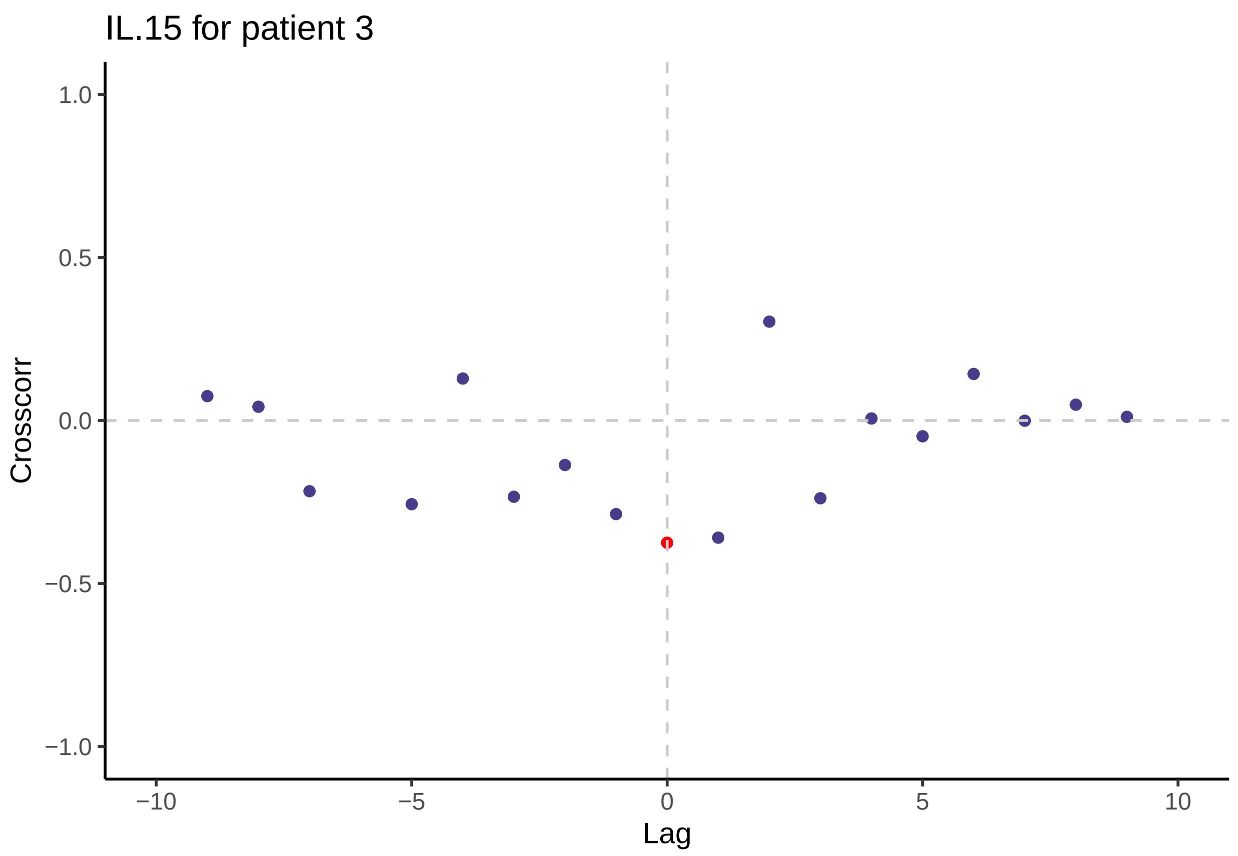


```
## [1] "IL.15 for patient 3 - p-value: 0.105425681564696"
```

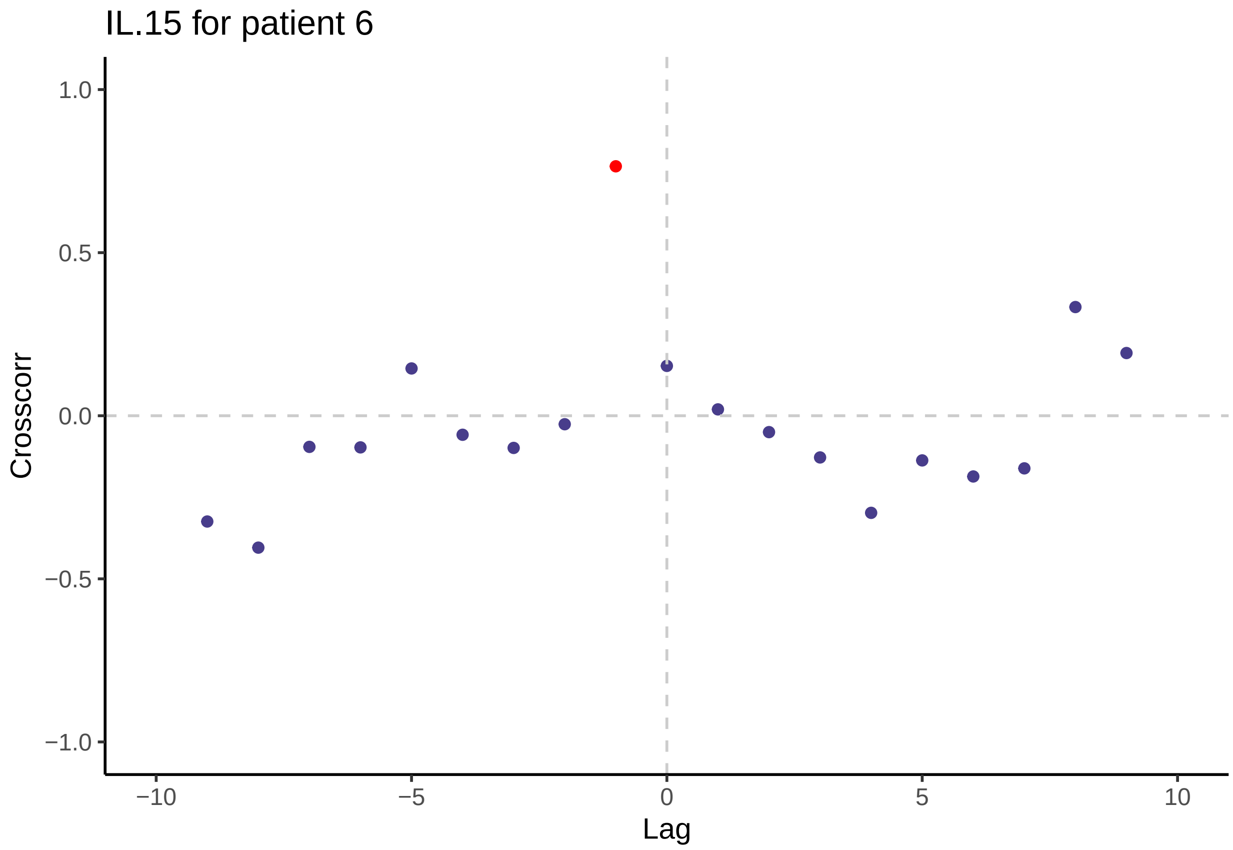


```
## [1] "IL.15 for patient 6 - p-value: 0.696548649250475"
```

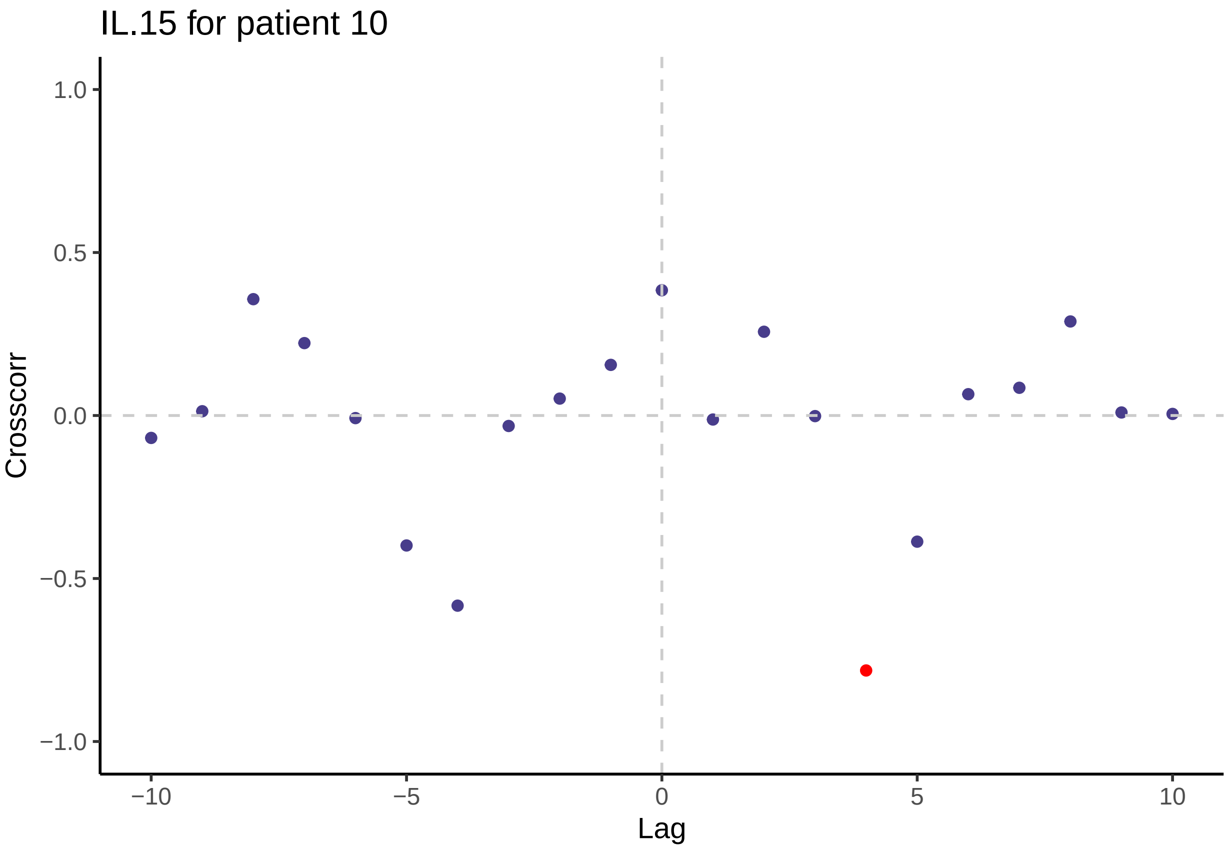


```
## [1] "IL.15 for patient 10 - p-value: 0.783278000303938"
```

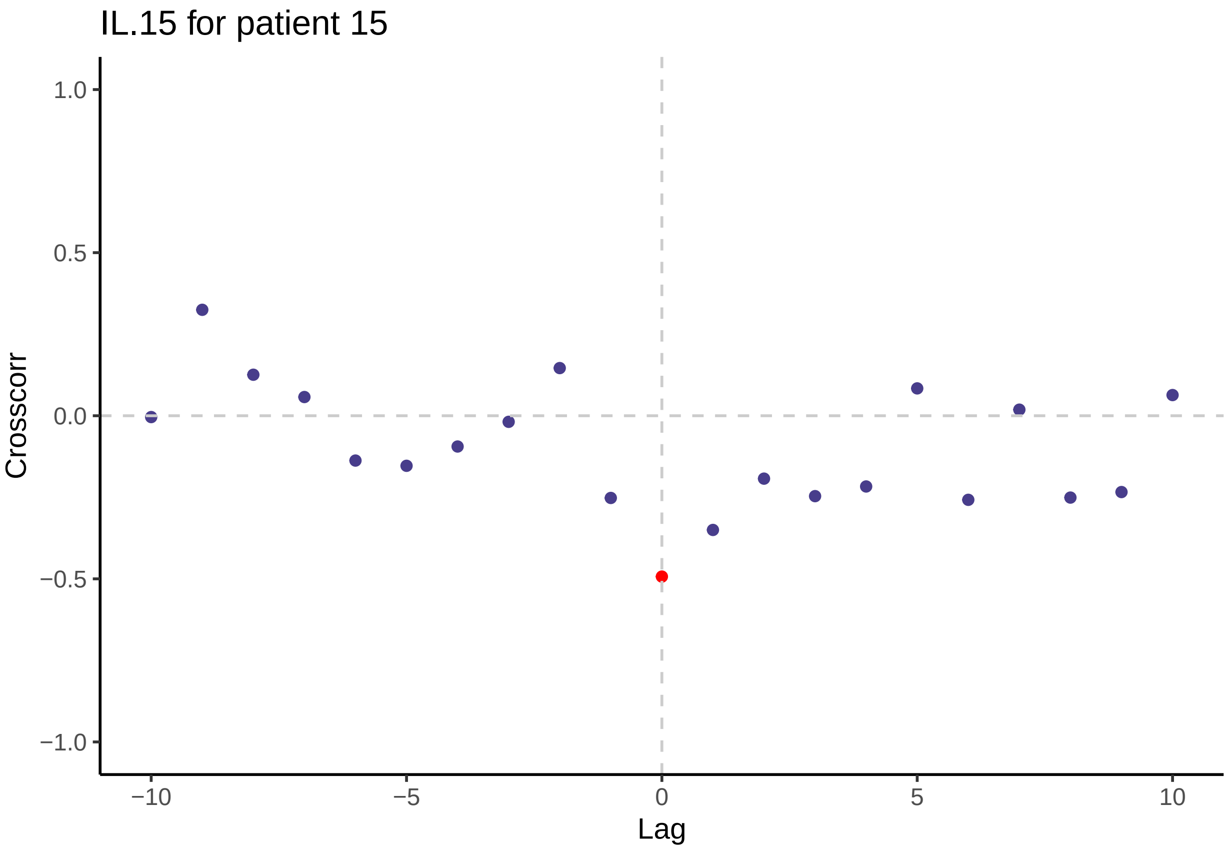


```
## [1] "IL.15 for patient 15 - p-value: 0.0305078937931928"
```

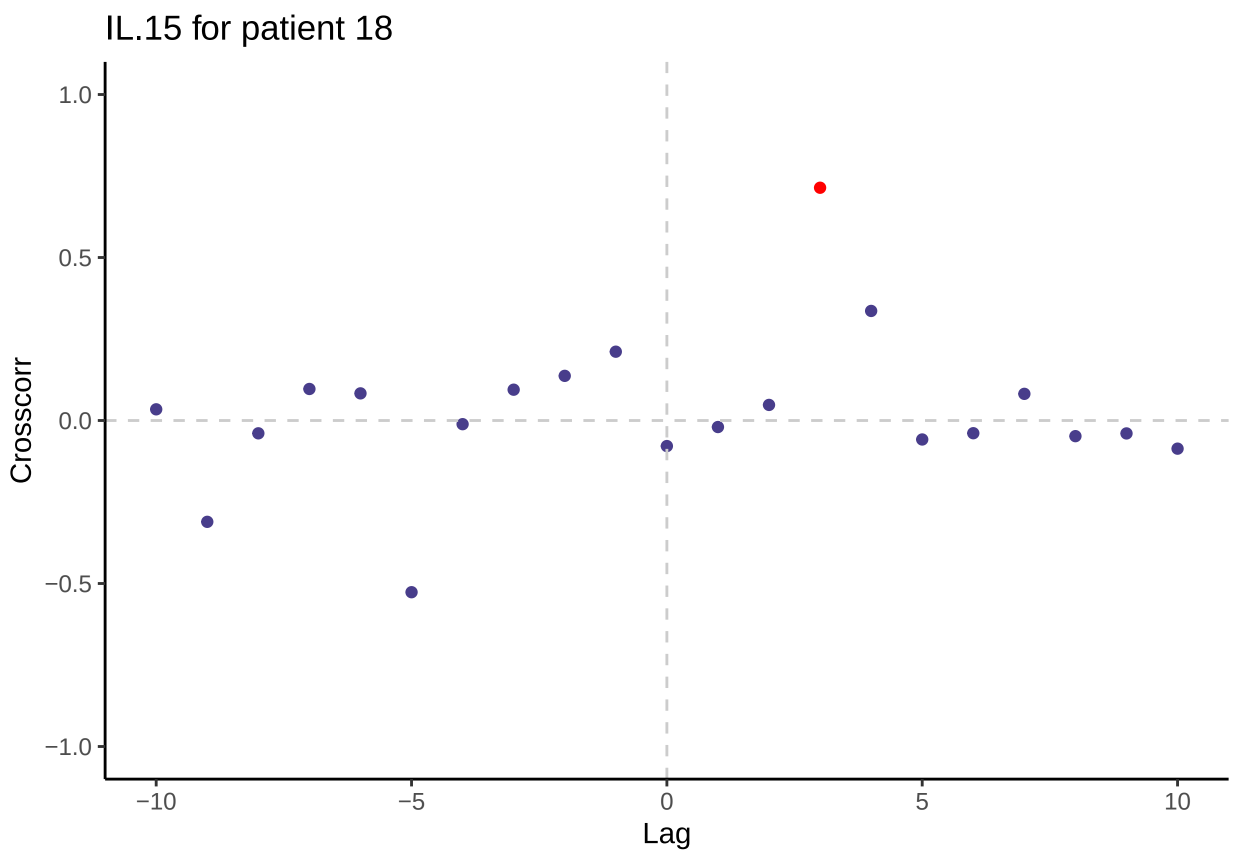


```
## [1] "IL.15 for patient 18 - p-value: 0.596533923166627"
## Warning: Removed 1 rows containing missing values (geom_point).
```

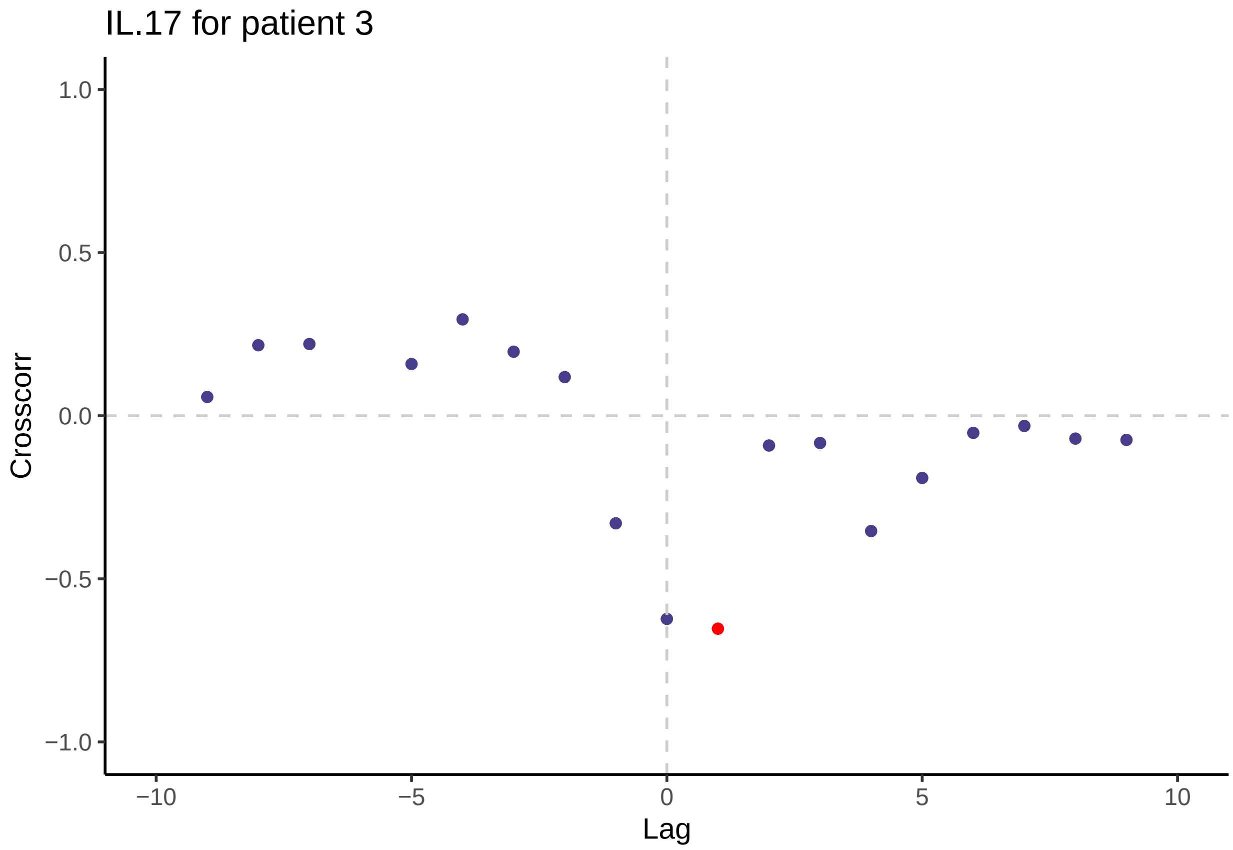


```
## [1] "IL.17 for patient 3 - p-value: 0.284235007104235"
## Warning: Removed 1 rows containing missing values (geom_point).
```

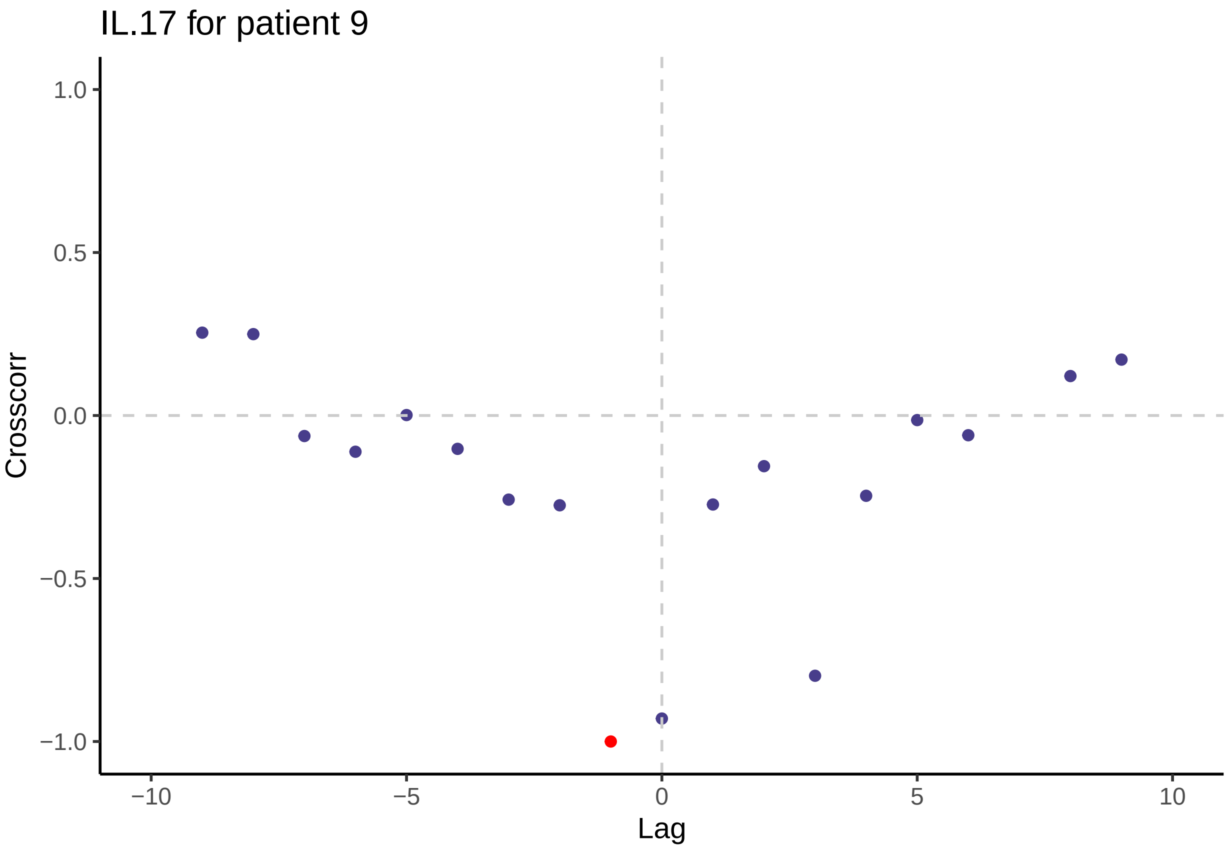


```
## [1] "IL.17 for patient 9 - p-value: 0.0401326324260126"
```

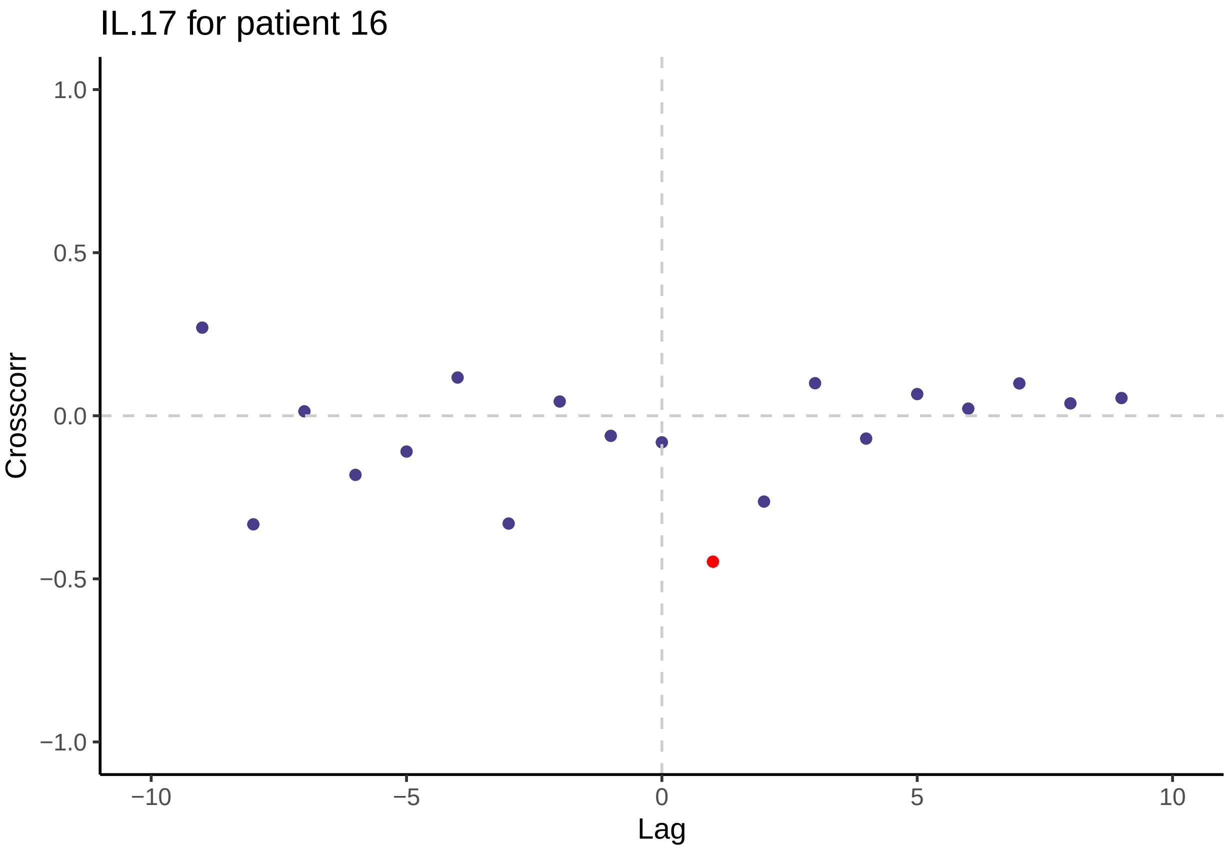


```
## [1] "IL.17 for patient 16 - p-value: 0.205325733322208"
```

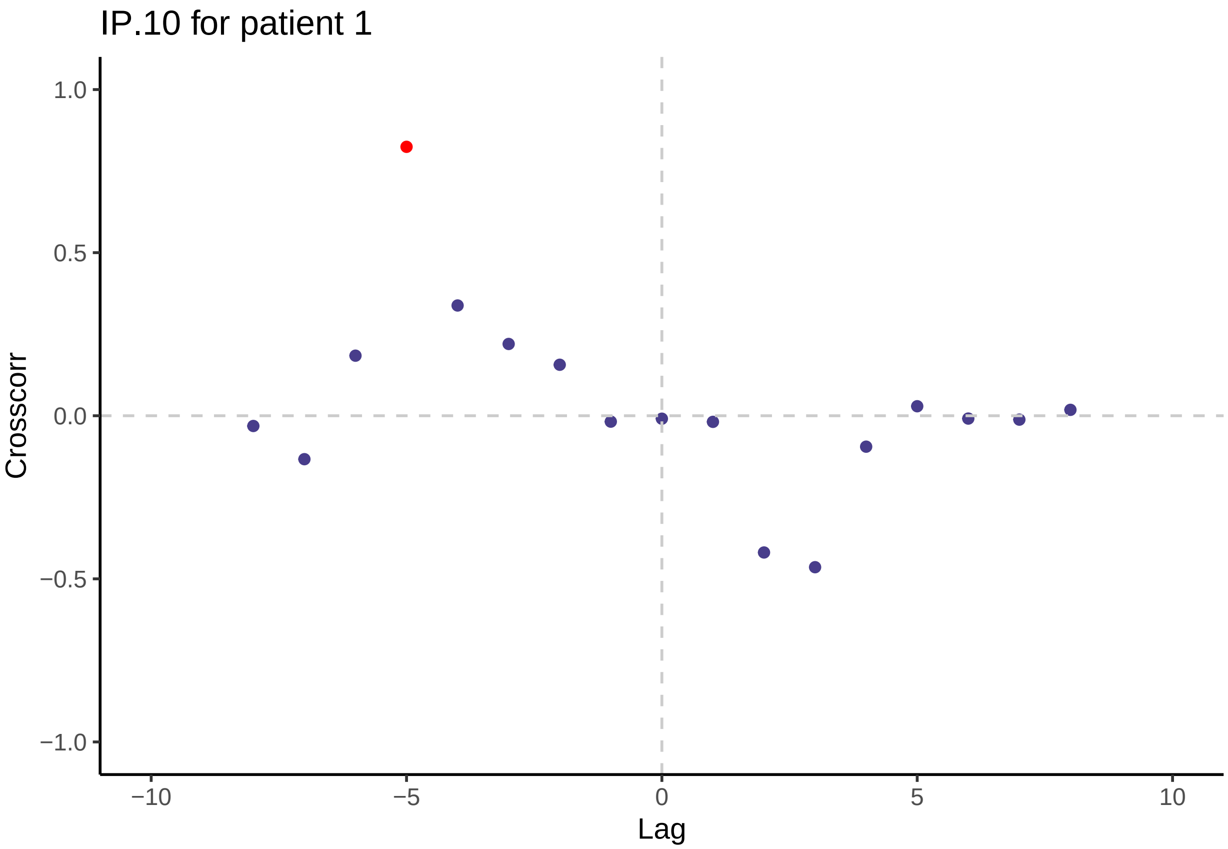


```
## [1] "IP.10 for patient 1 - p-value: 0.639740775582575"
```

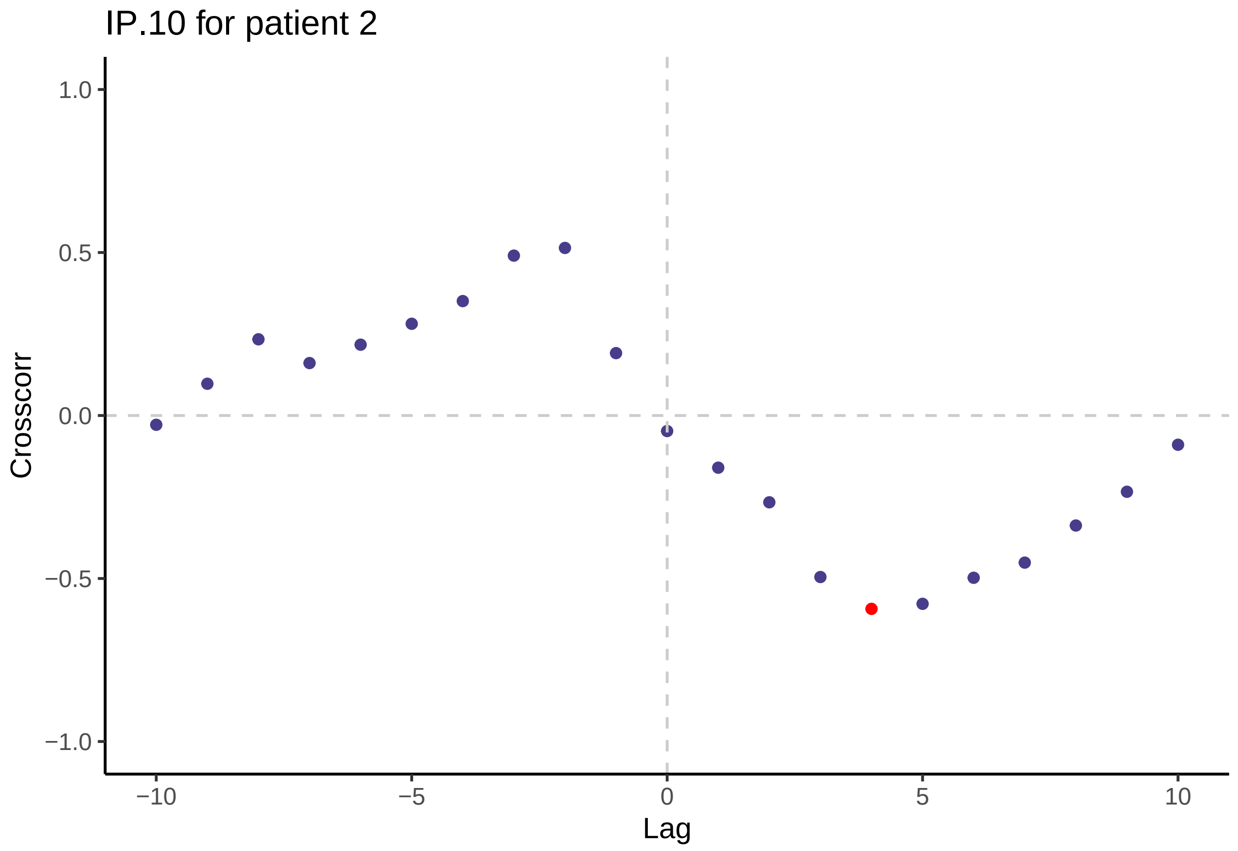


```
## [1] "IP.10 for patient 2 - p-value: 0.449642691425198"
```

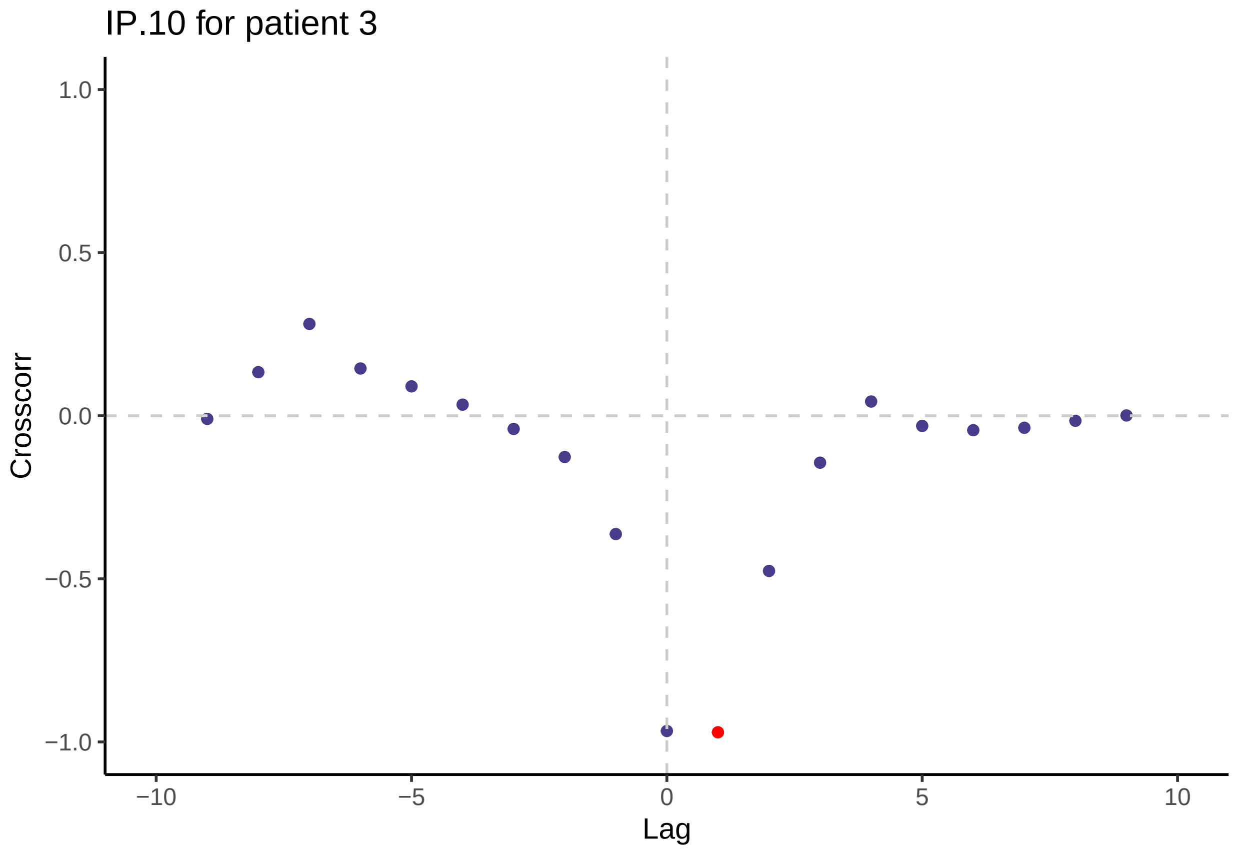


```
## [1] "IP.10 for patient 3 - p-value: 0.1093247817056"
```

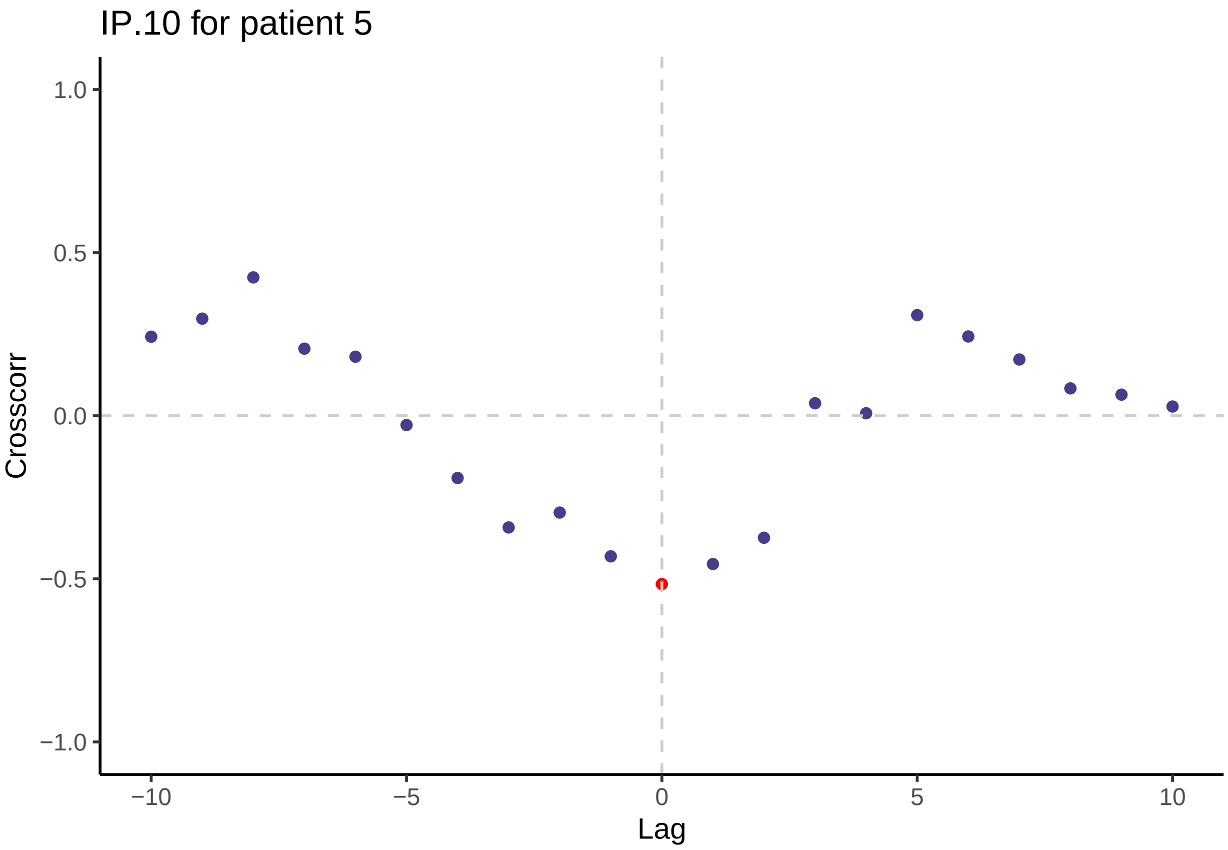


```
## [1] "IP.10 for patient 5 - p-value: 0.800062523006296"
```

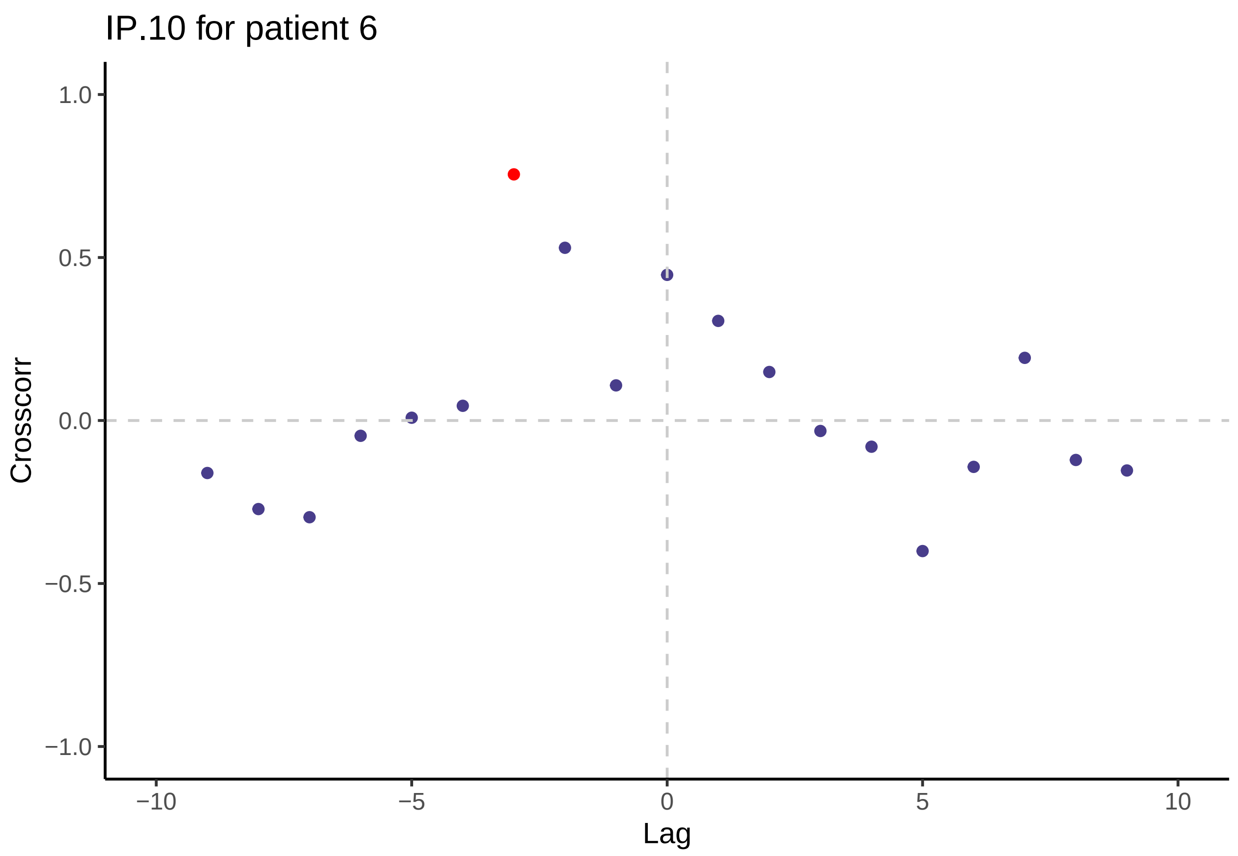


```
## [1] "IP.10 for patient 6 - p-value: 0.528606757012059"
```

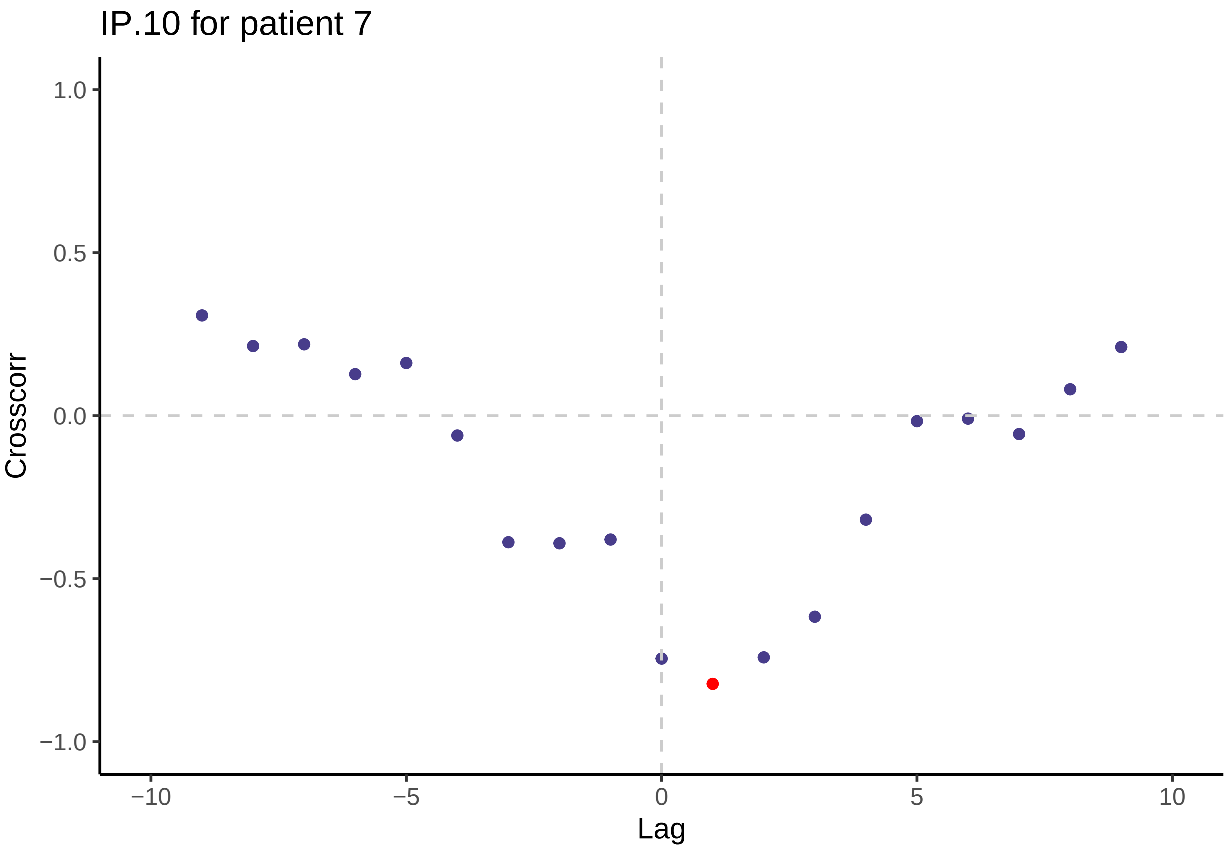


```
## [1] "IP.10 for patient 7 - p-value: 0.0605141180965169"
```

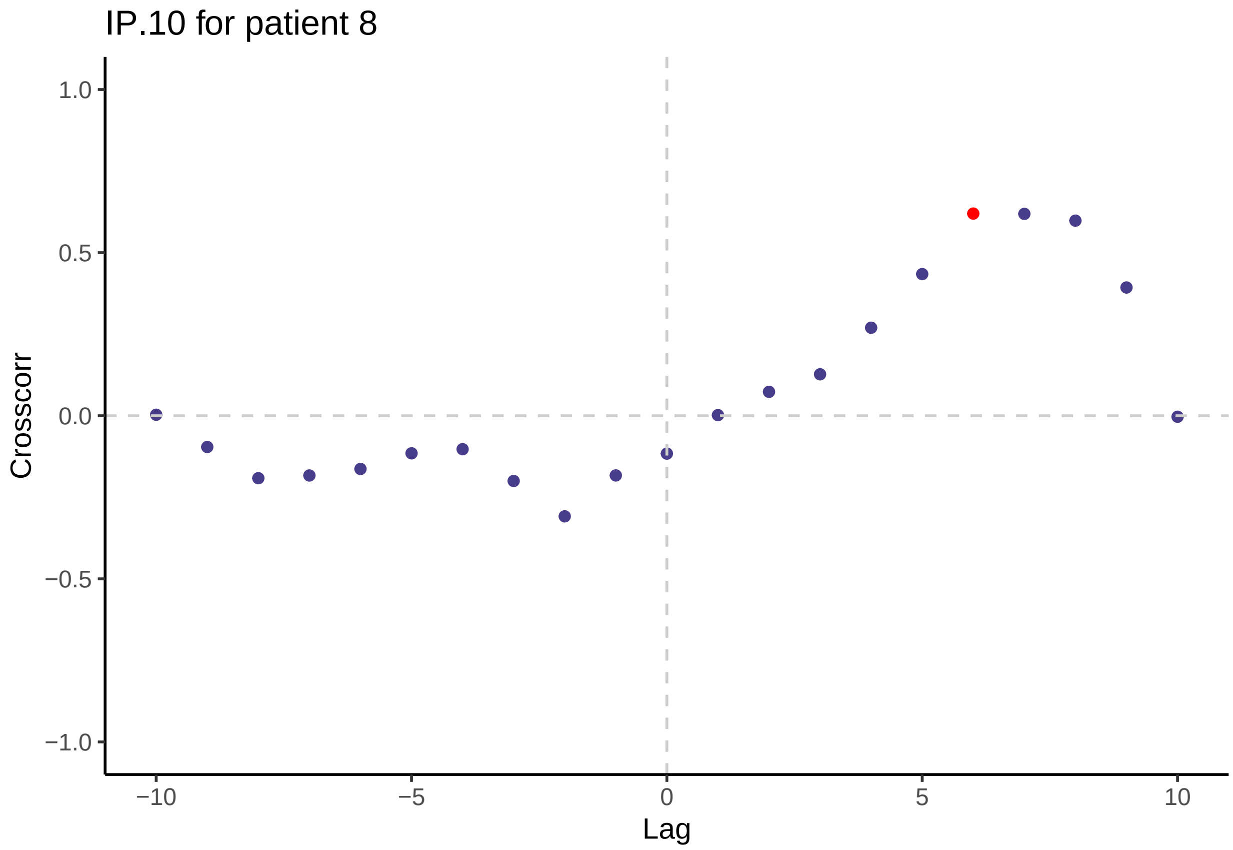


```
## [1] "IP.10 for patient 8 - p-value: 0.291161759642741"
```

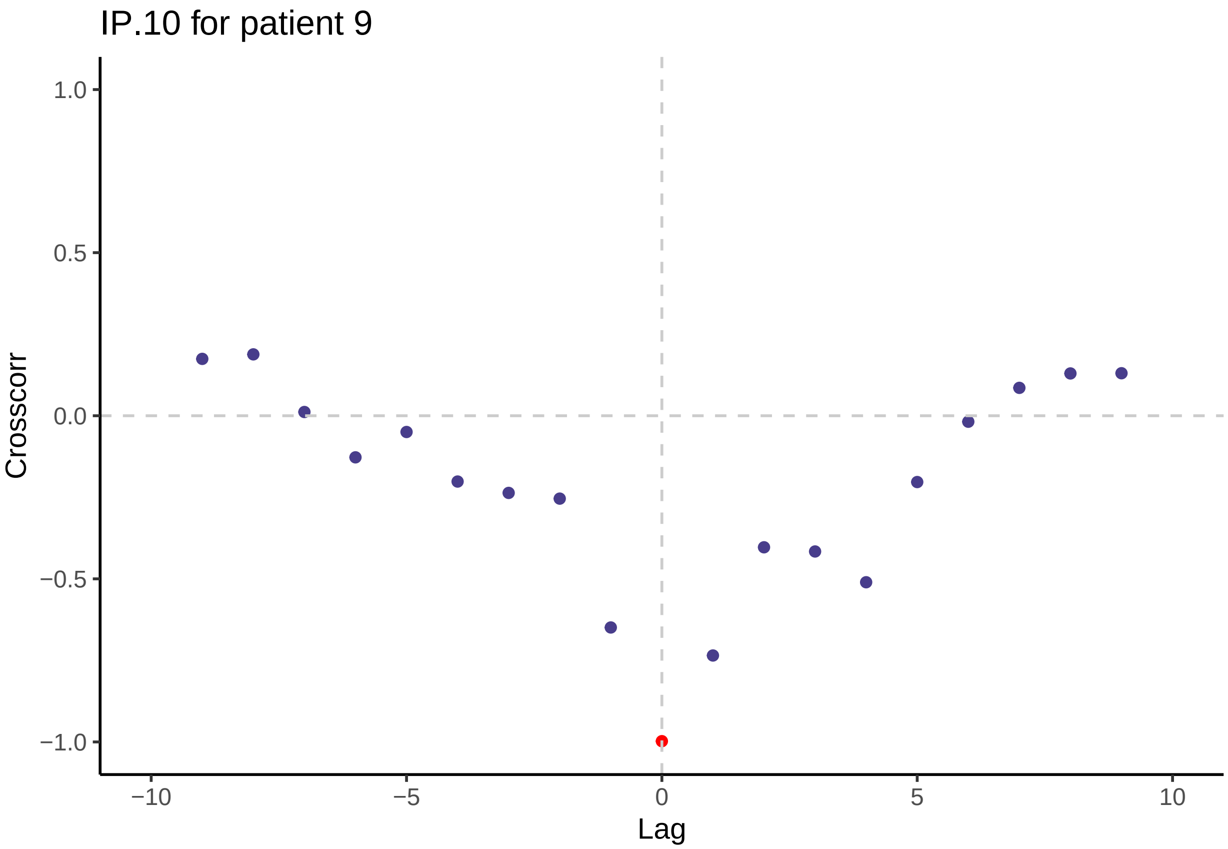


```
## [1] "IP.10 for patient 9 - p-value: 0.011662968481357"
```

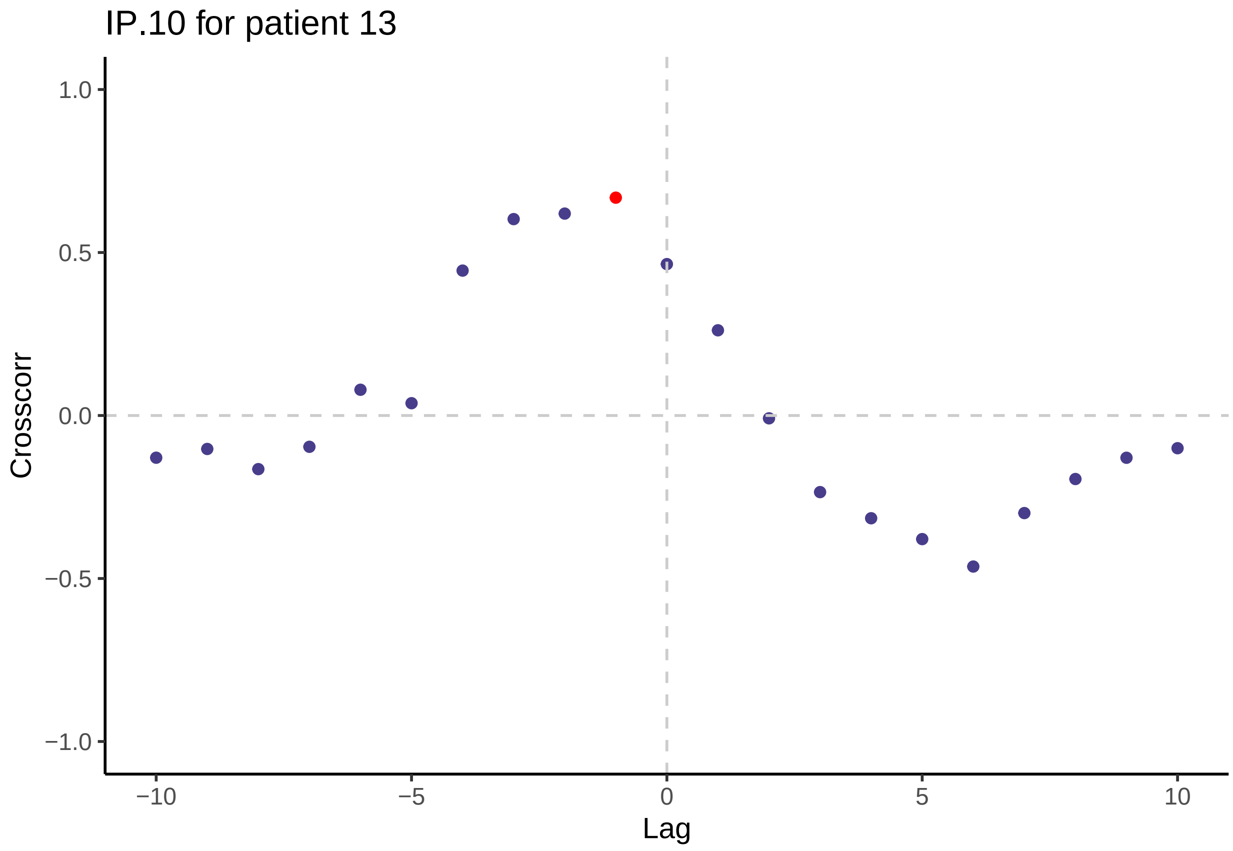


```
## [1] "IP.10 for patient 13 - p-value: 0.728091472845261"
```

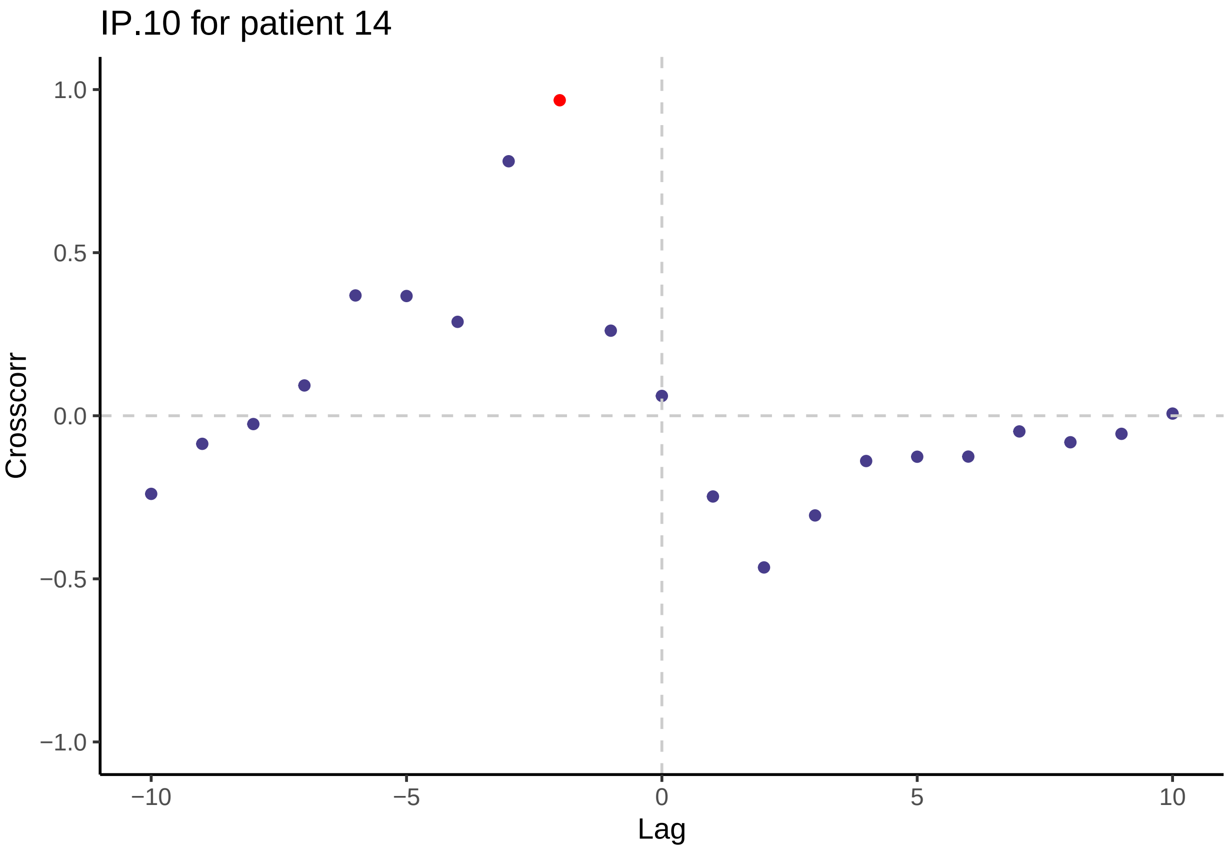


```
## [1] "IP.10 for patient 14 - p-value: 0.440889025685934"
```

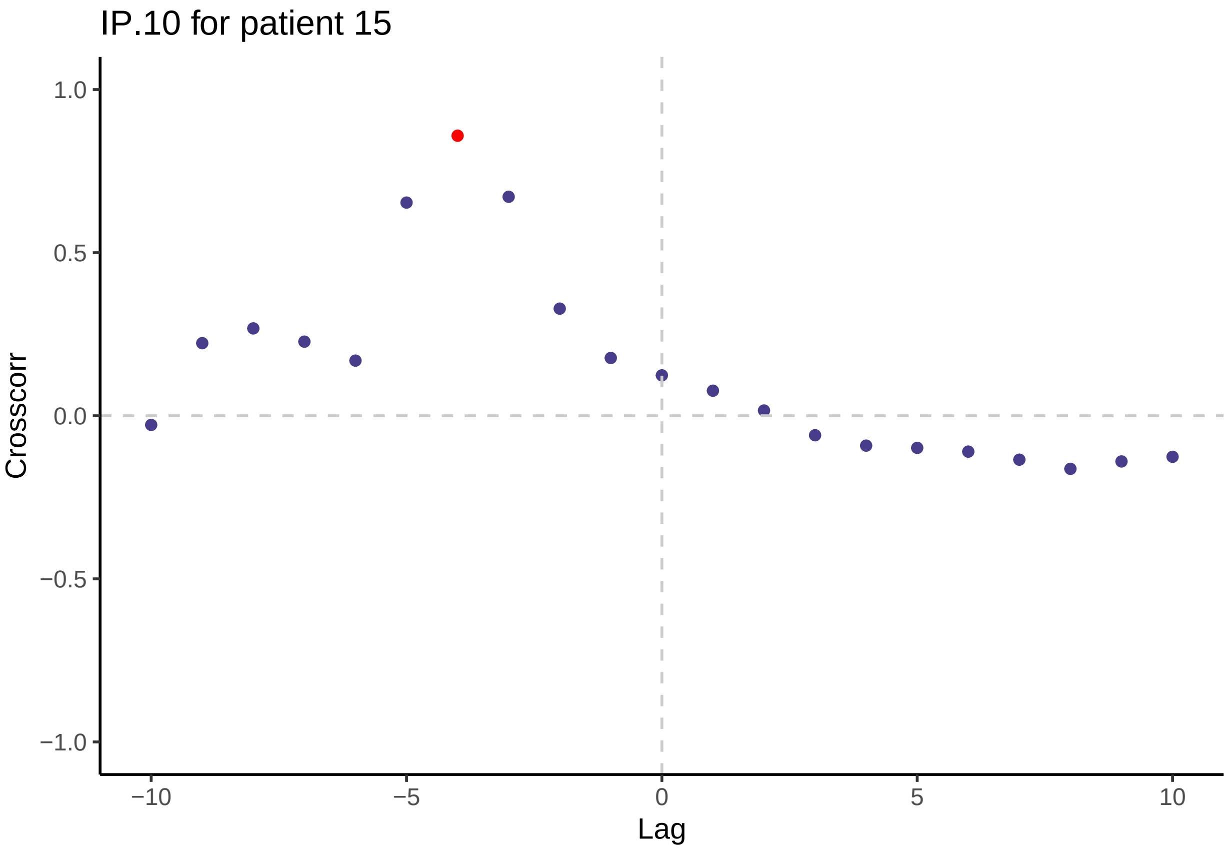


```
## [1] "IP.10 for patient 15 - p-value: 0.0465113534035932"
```

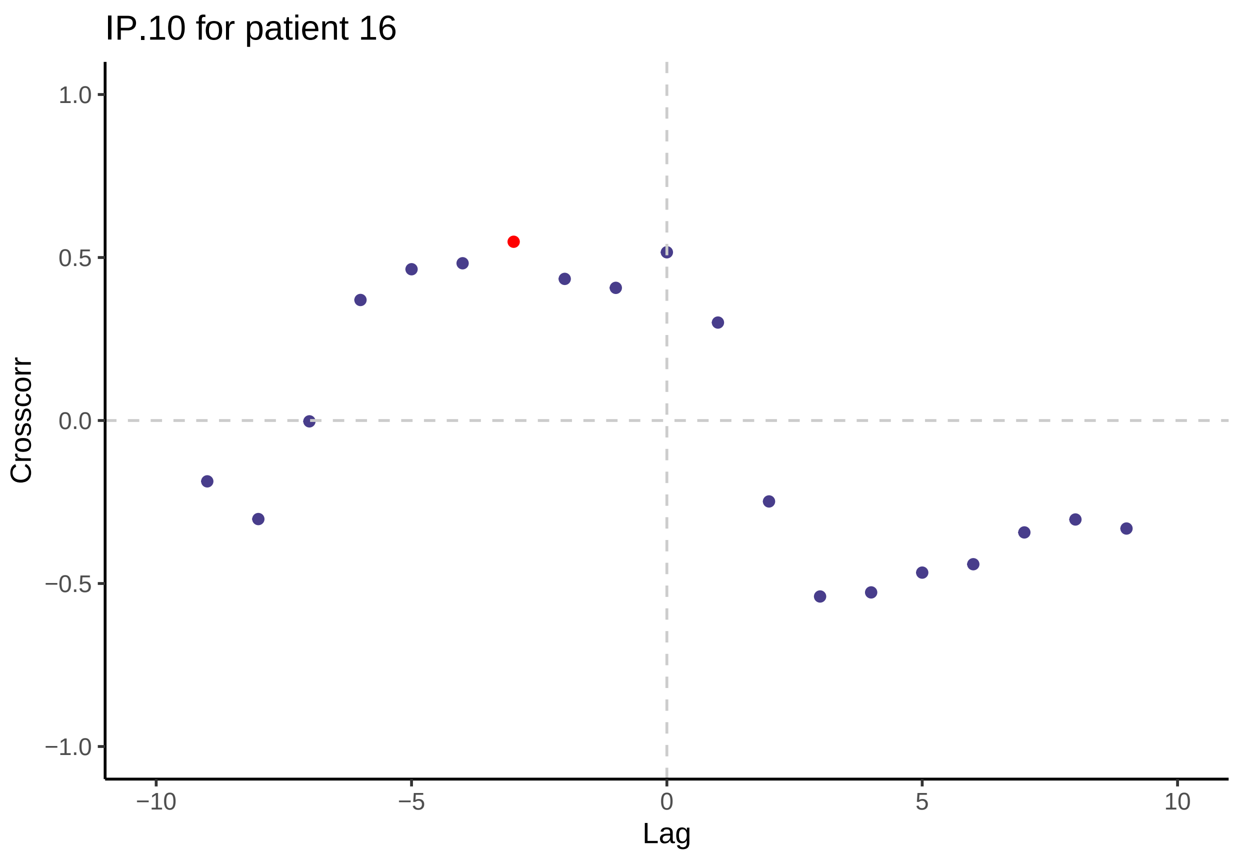


```
## [1] "IP.10 for patient 16 - p-value: 0.926021414053078"
```

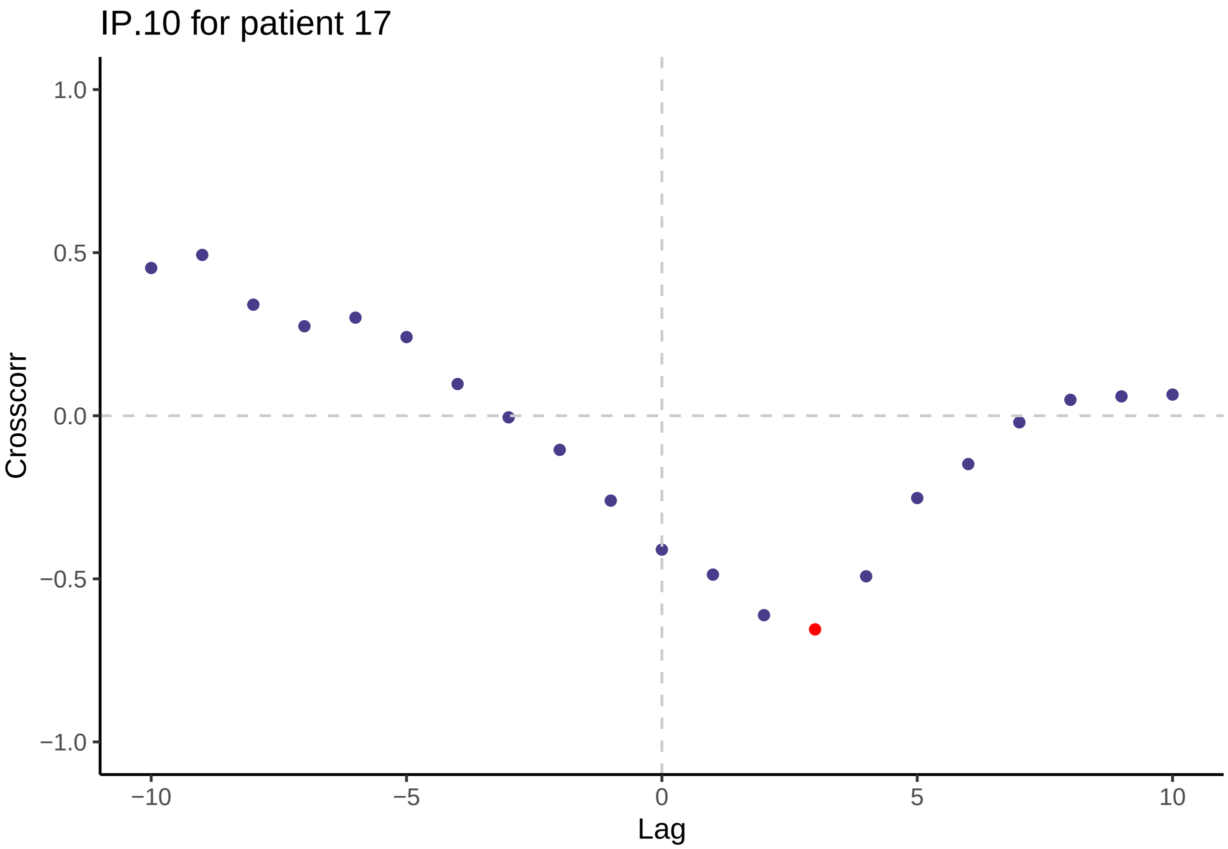


```
## [1] "IP.10 for patient 17 - p-value: 0.504229588068624"
```

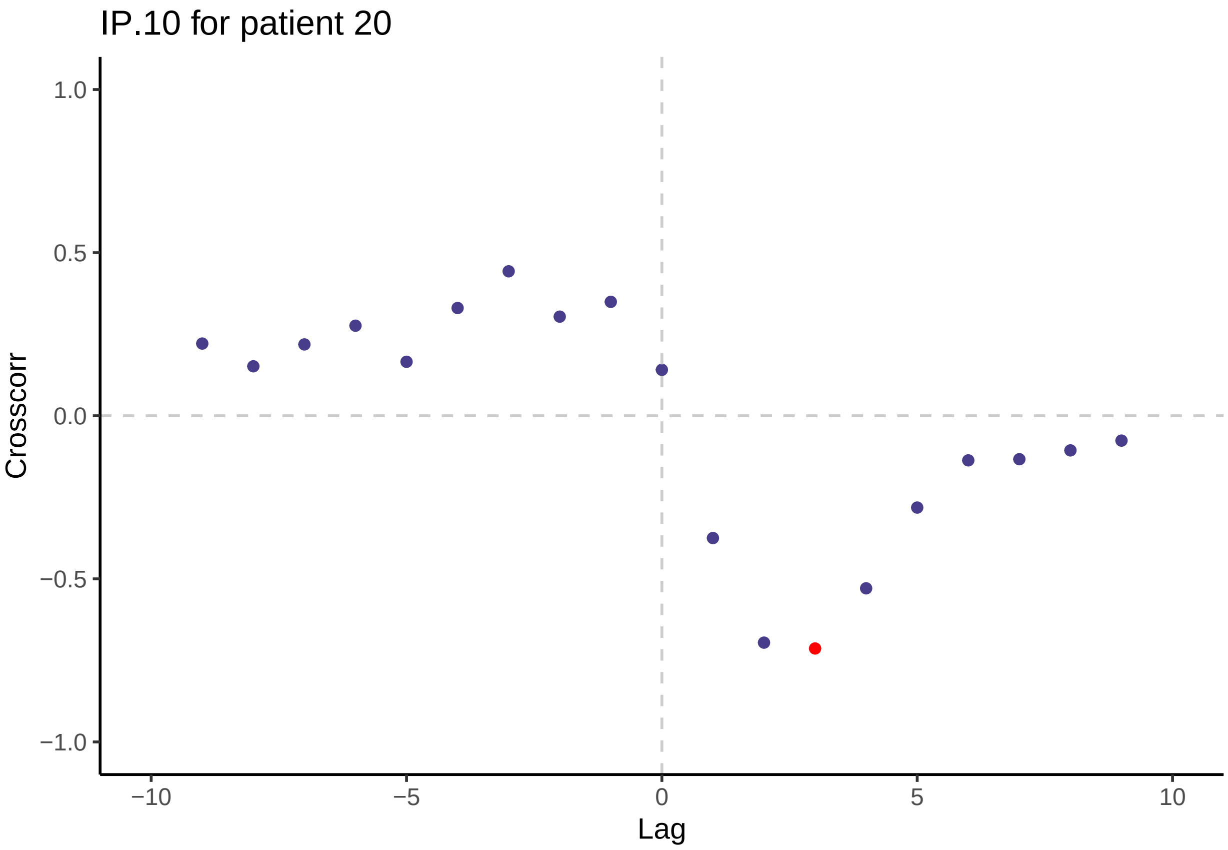


```
## [1] "IP.10 for patient 20 - p-value: 0.777651137559125"
## Warning: Removed 1 rows containing missing values (geom_point).
```

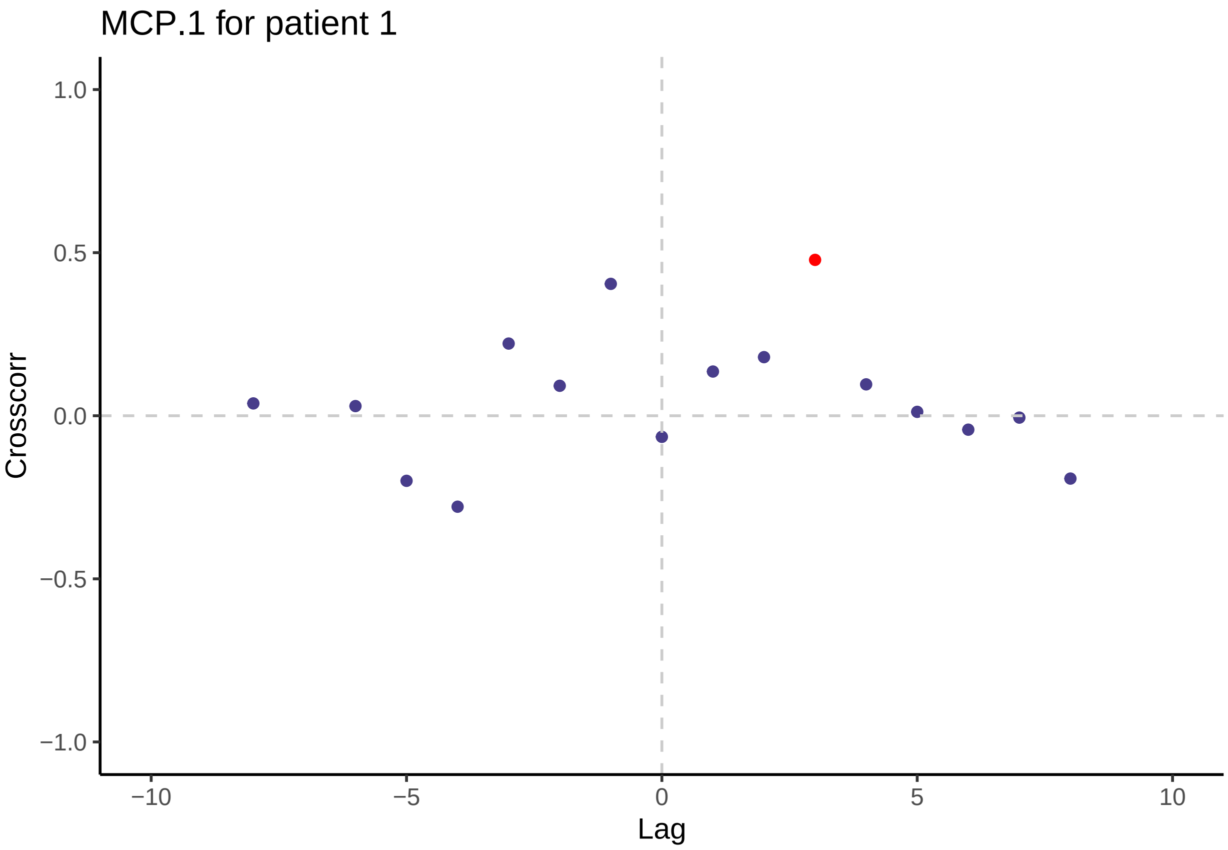


```
## [1] "MCP.1 for patient 1 - p-value: 0.285047994452936"
```

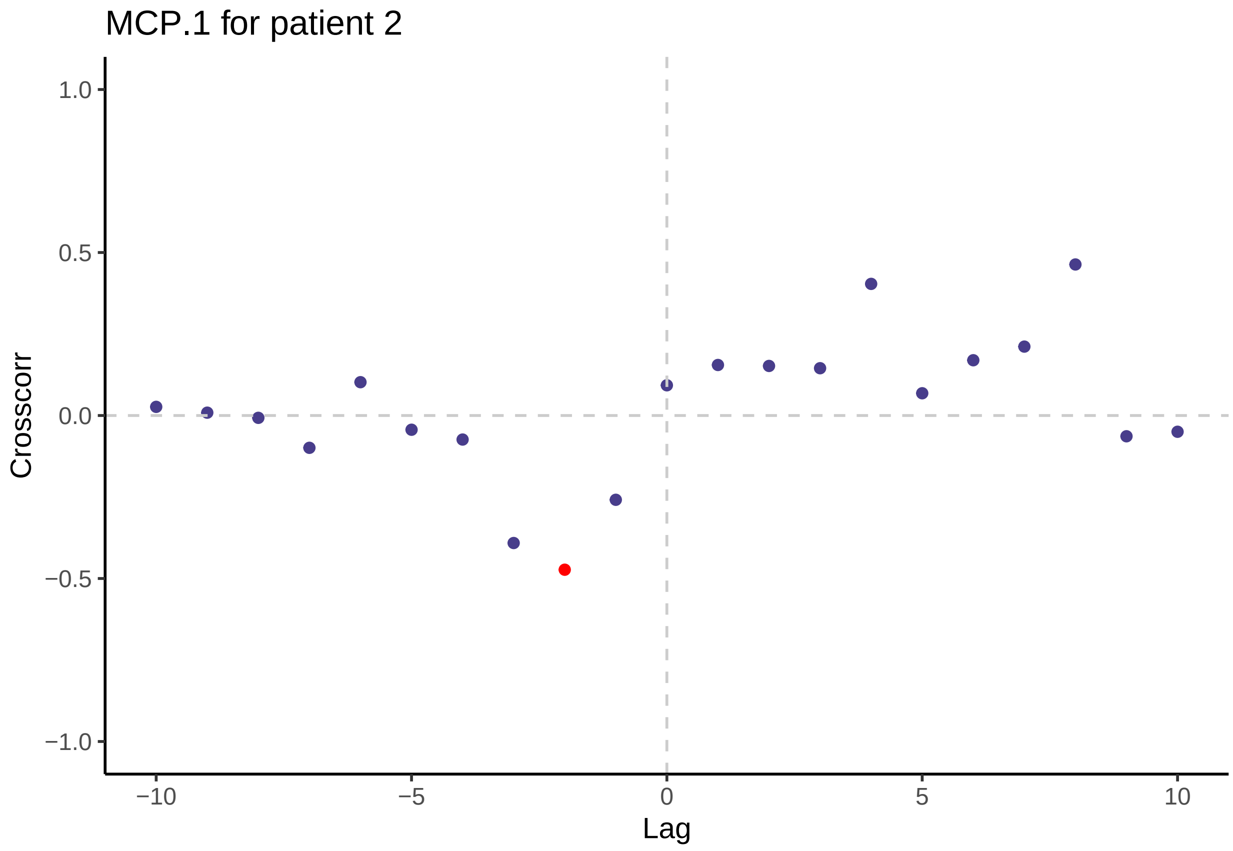


```
## [1] "MCP.1 for patient 2 - p-value: 0.604184556726904"
```

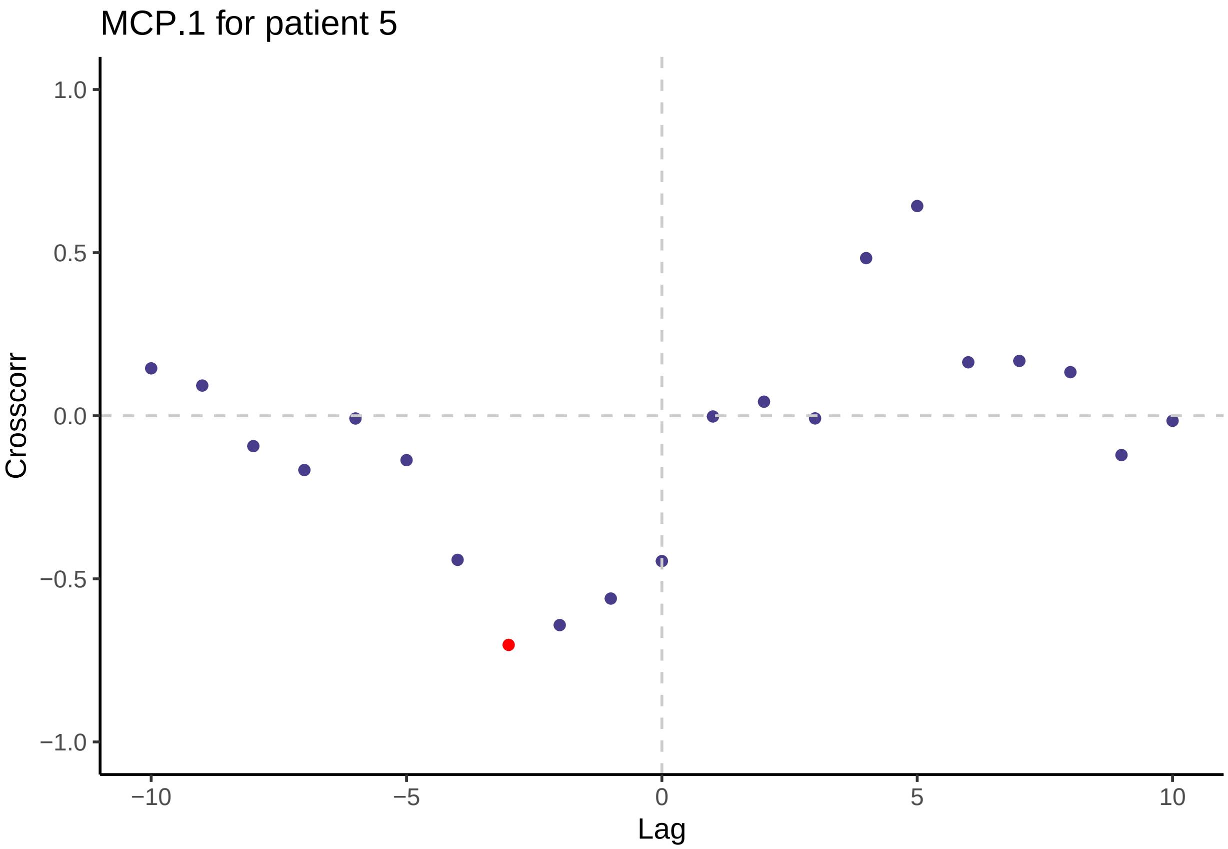


```
## [1] "MCP.1 for patient 5 - p-value: 0.358848497431132"
```

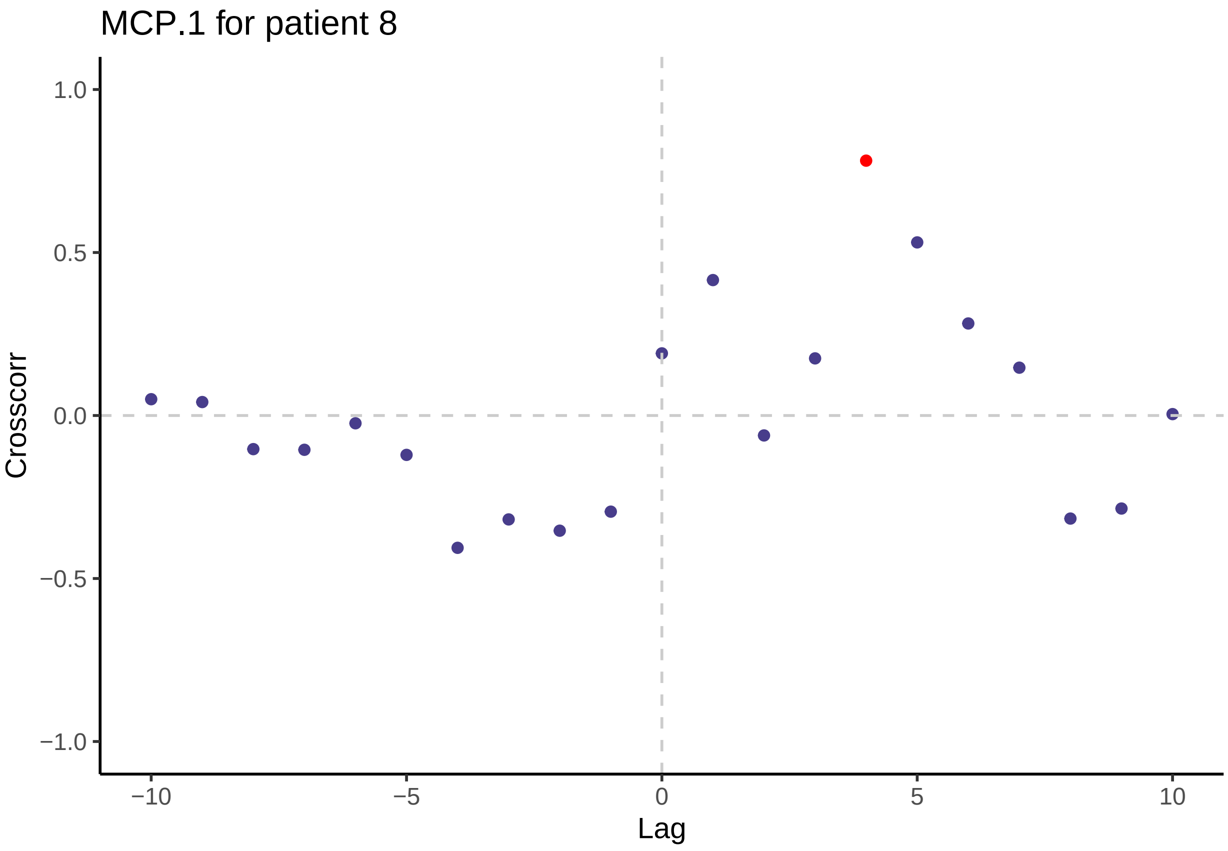


```
## [1] "MCP.1 for patient 8 - p-value: 0.873196409045232"
## Warning: Removed 1 rows containing missing values (geom_point).
```

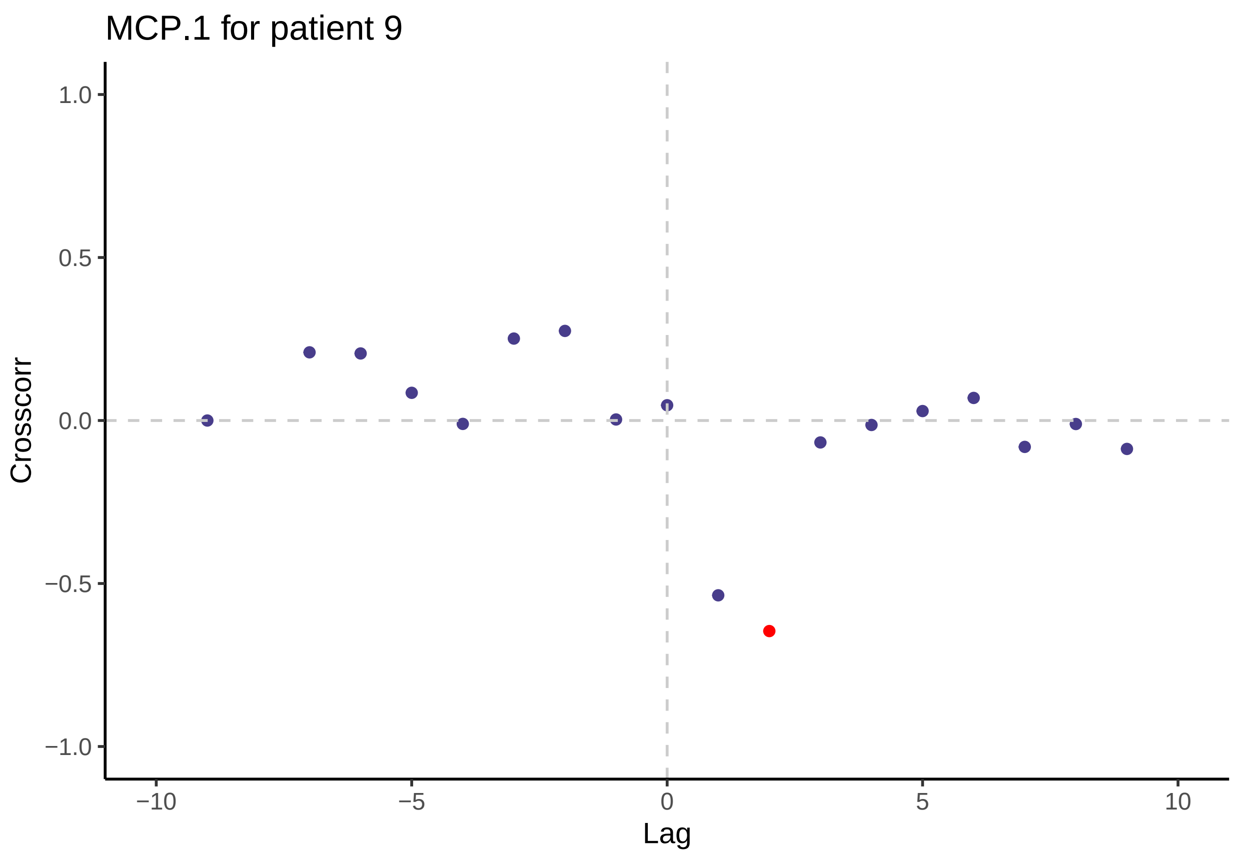


```
## [1] "MCP.1 for patient 9 - p-value: 0.786075144998025"
```

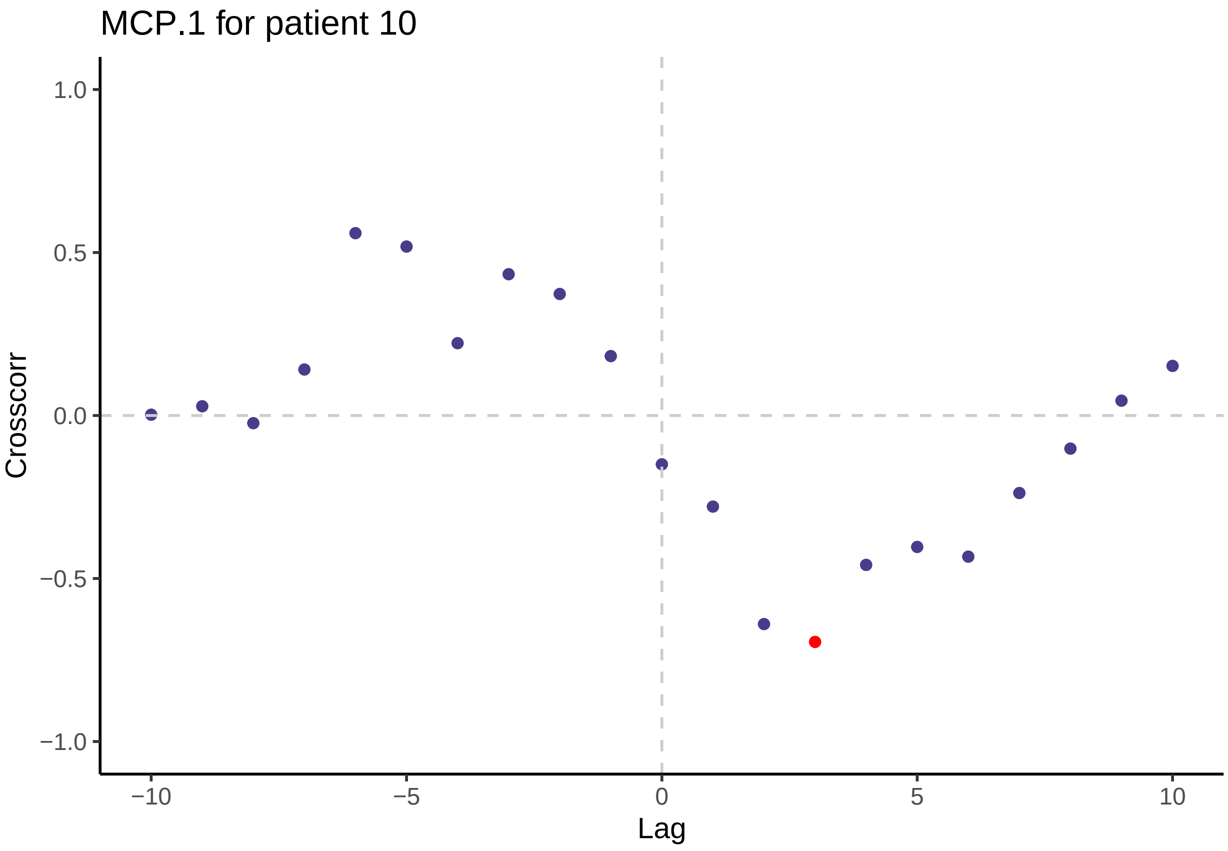


```
## [1] "MCP.1 for patient 10 - p-value: 0.651052780034176"
```

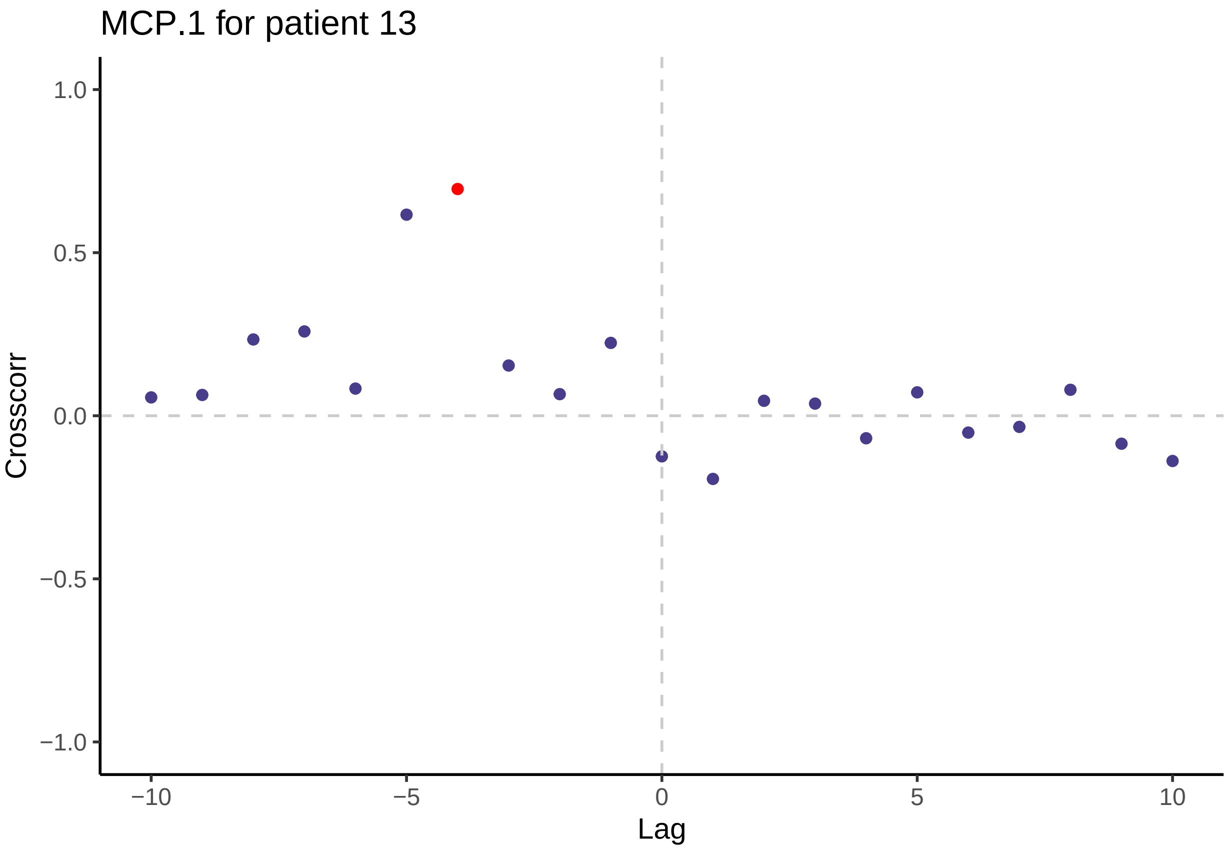


```
## [1] "MCP.1 for patient 13 - p-value: 0.0655677987758038"
## Warning: Removed 2 rows containing missing values (geom_point).
```

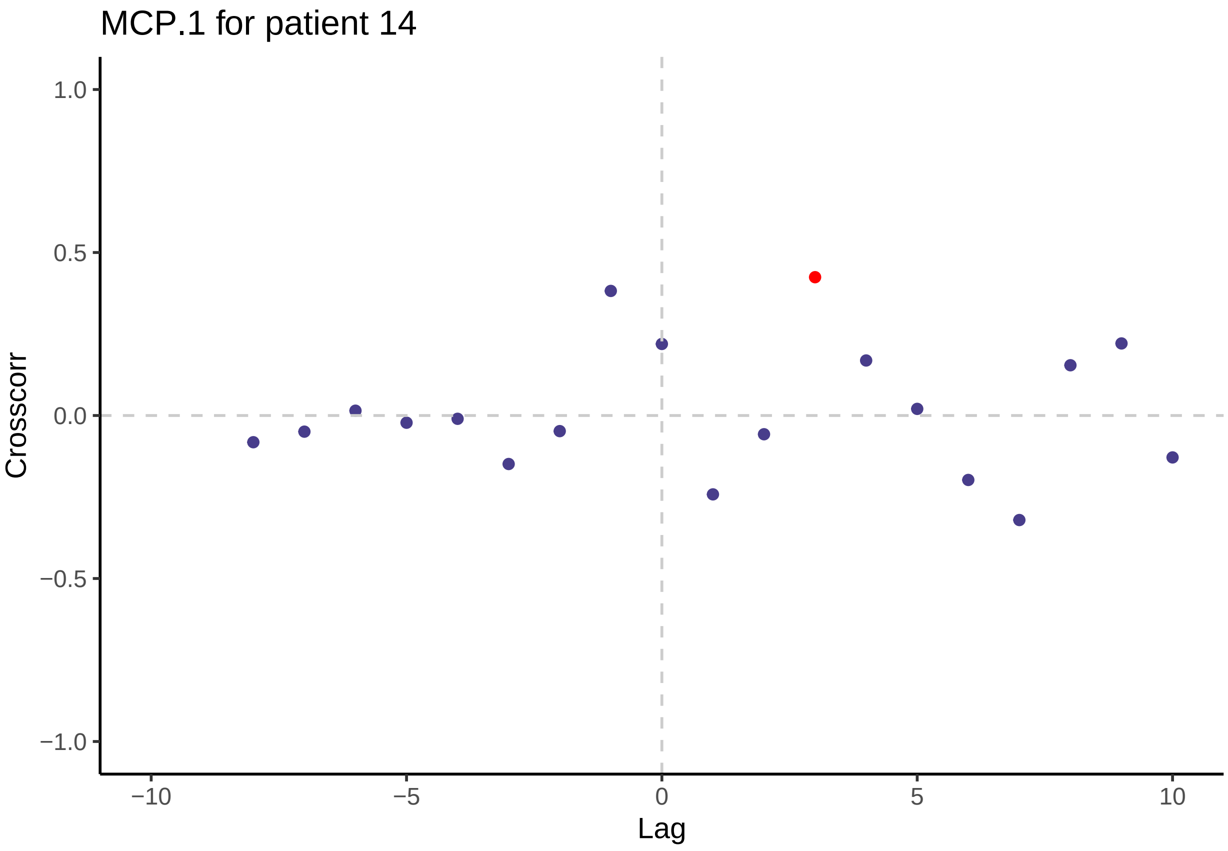


```
## [1] "MCP.1 for patient 14 - p-value: 0.736900969526215"
```

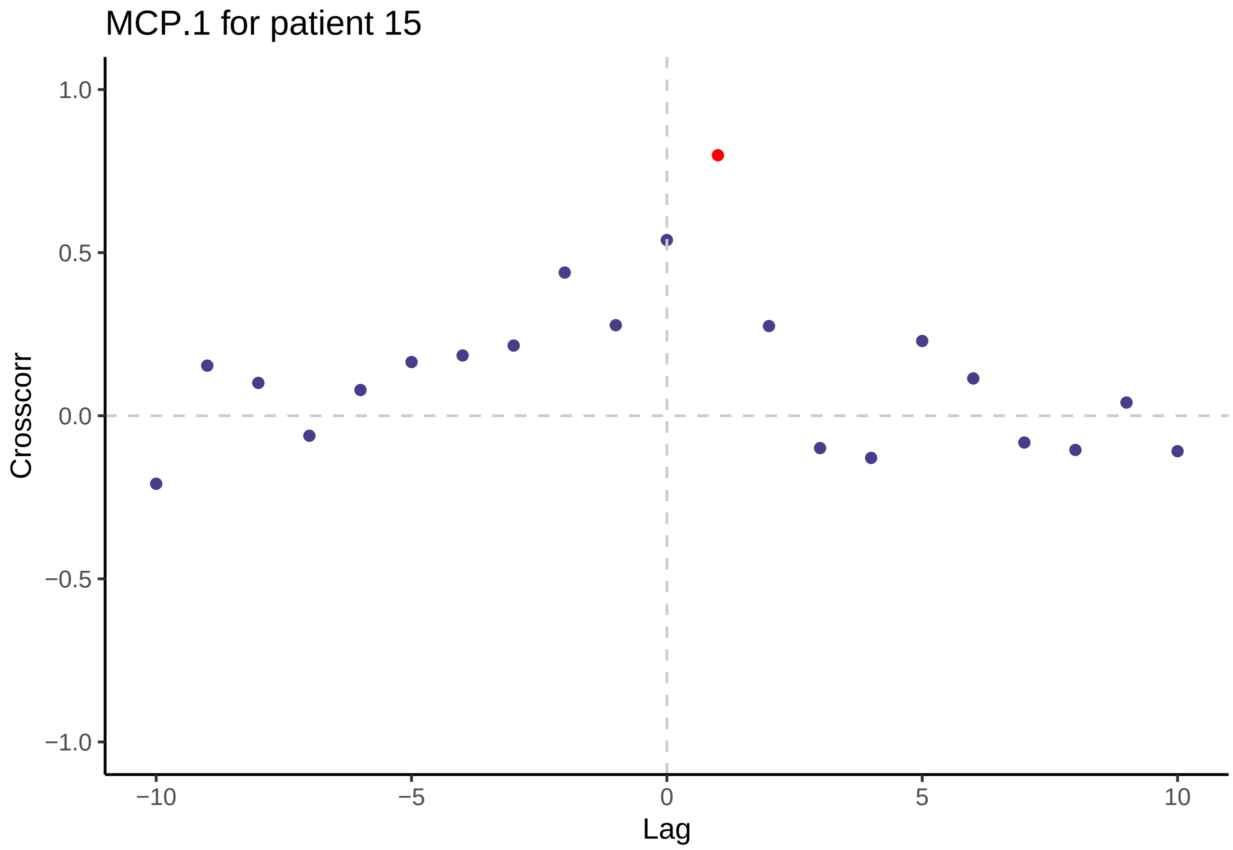


```
## [1] "MCP.1 for patient 15 - p-value: 0.0214212603051621"
```

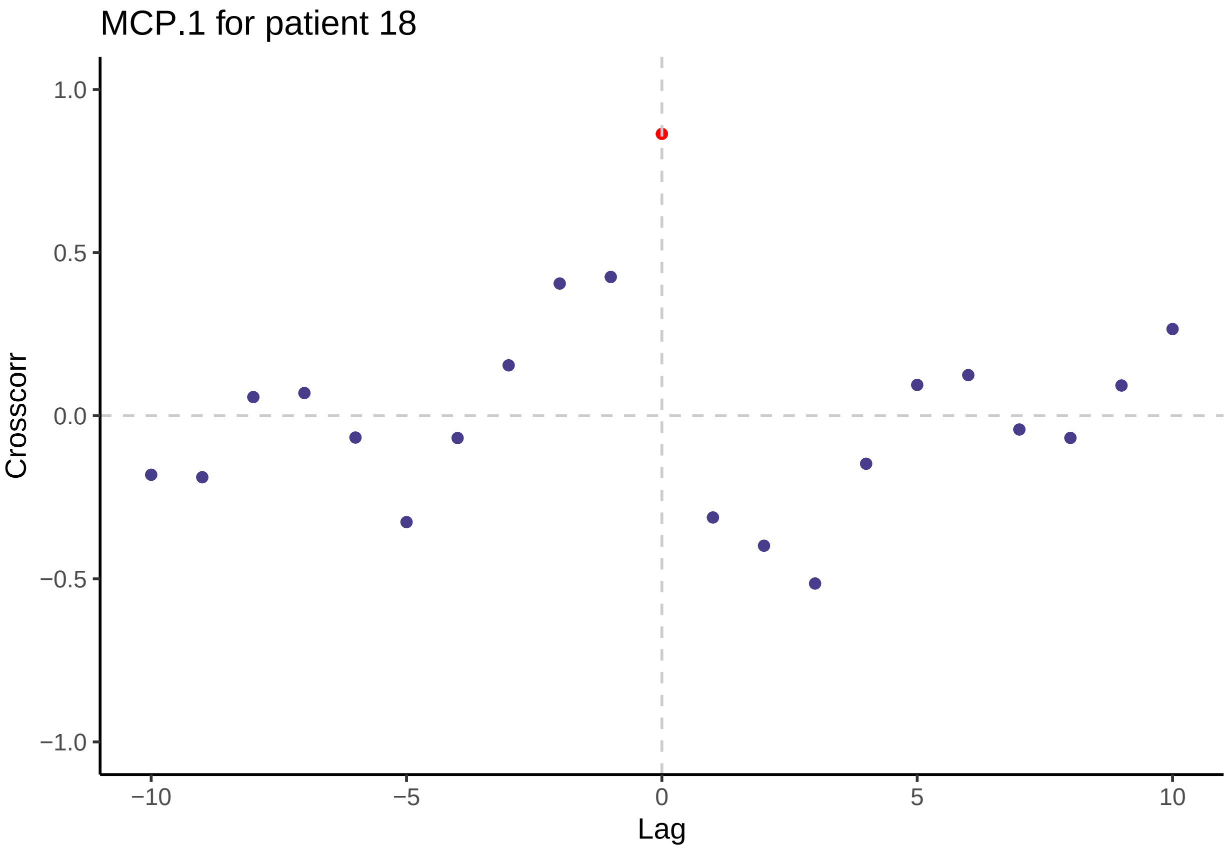


```
## [1] "MCP.1 for patient 18 - p-value: 0.867681190571504"
```

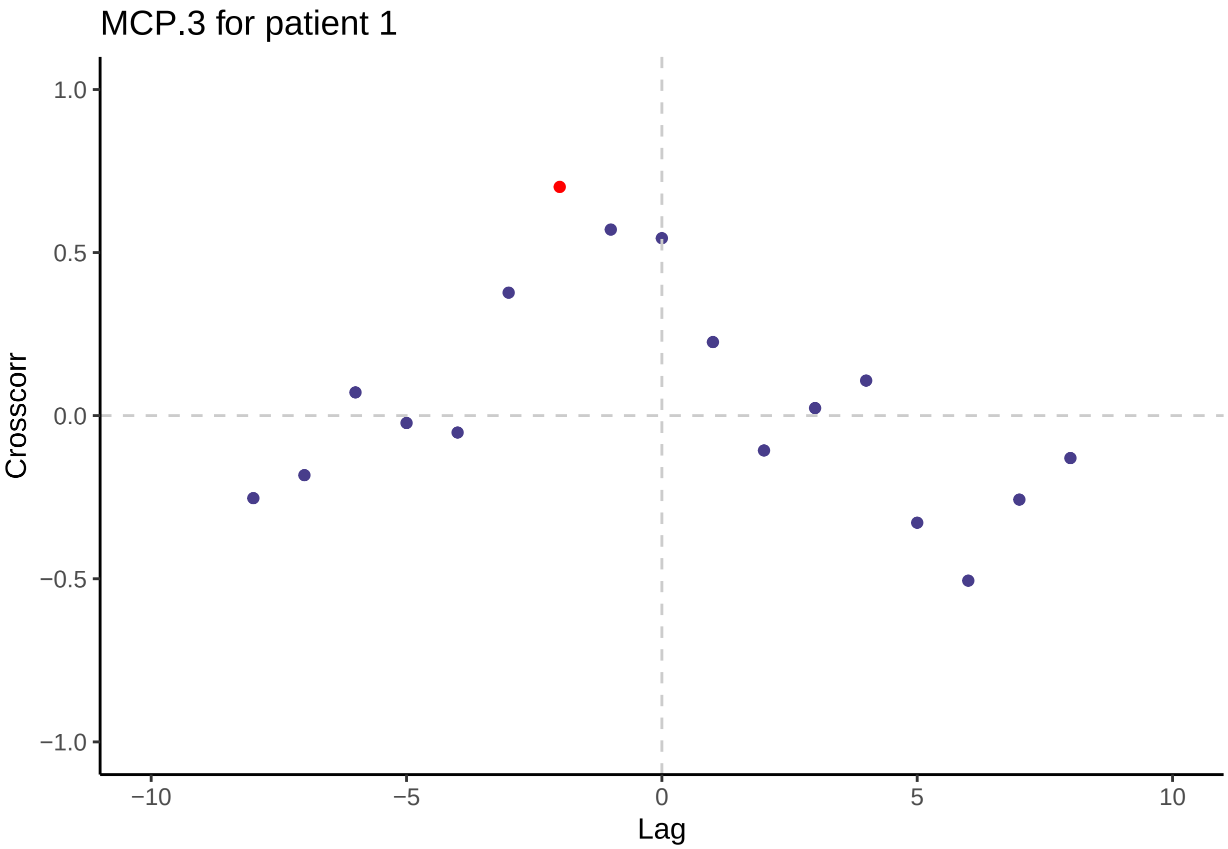


```
## [1] "MCP.3 for patient 1 - p-value: 0.581765783067311"
```

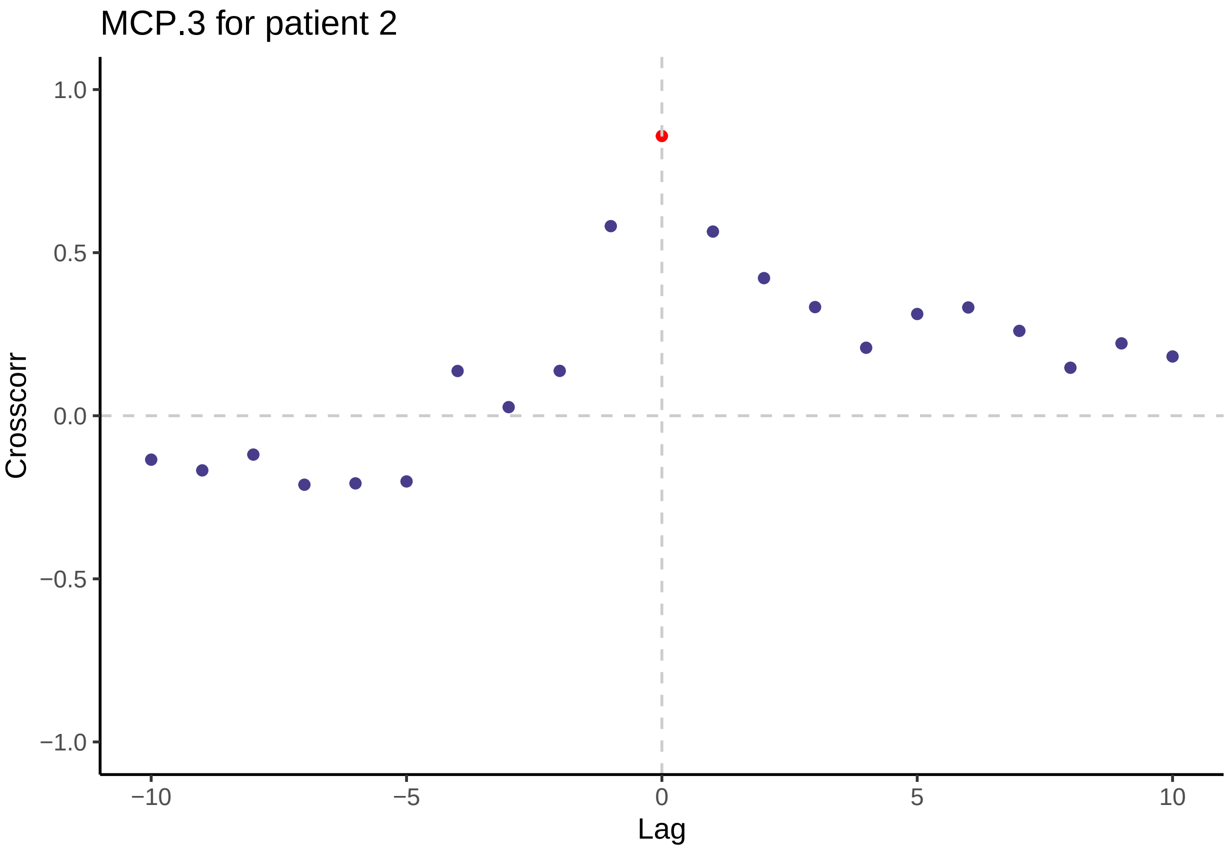


```
## [1] "MCP.3 for patient 2 - p-value: 0.0118641986879193"
```

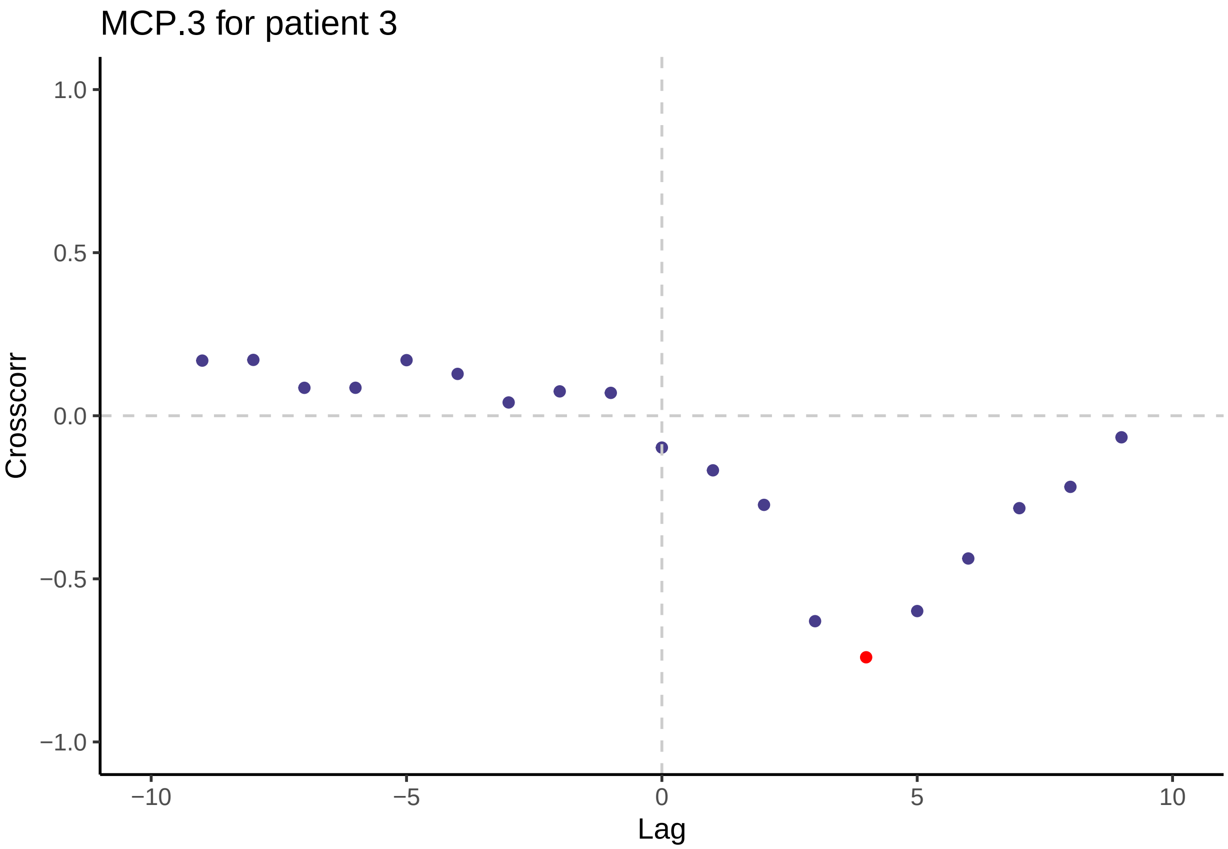


```
## [1] "MCP.3 for patient 3 - p-value: 0.0637261204106438"
```

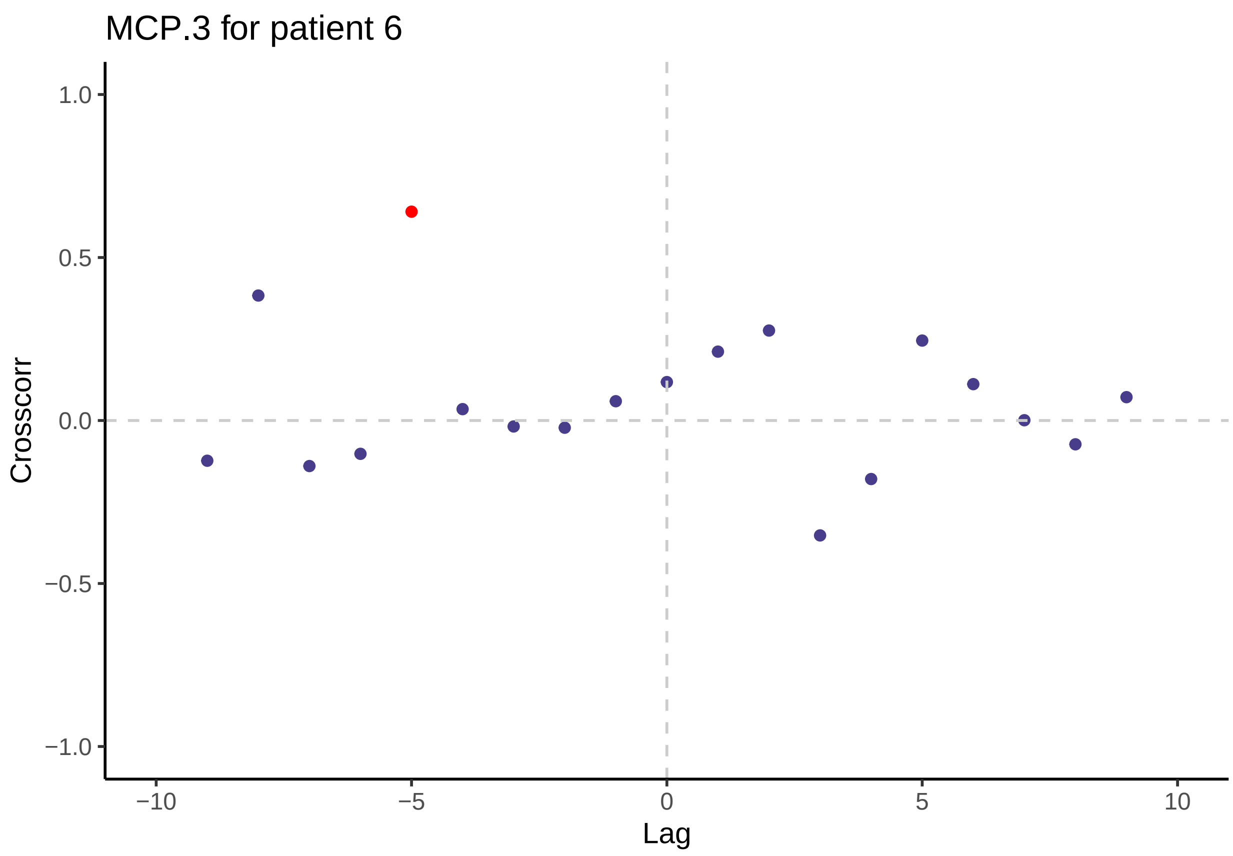


```
## [1] "MCP.3 for patient 6 - p-value: 0.260178653073132"
```

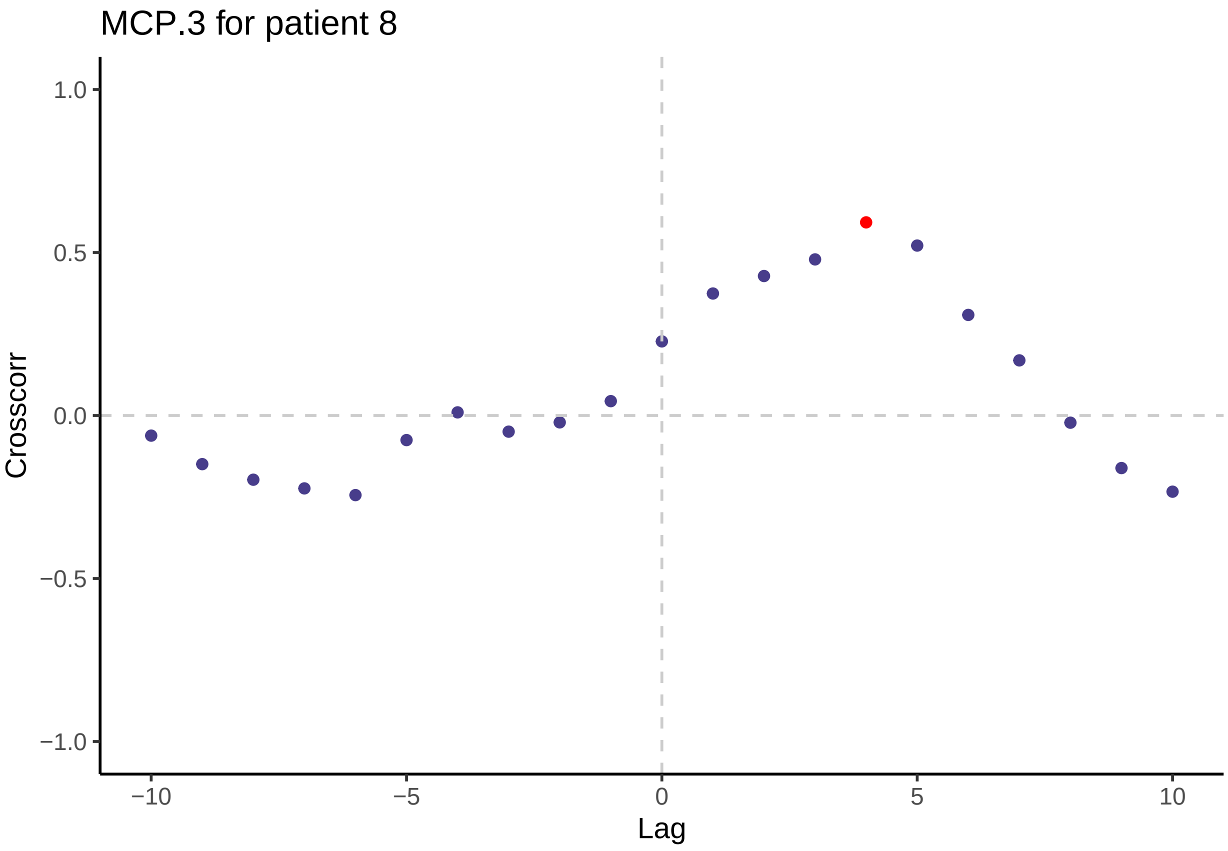


```
## [1] "MCP.3 for patient 8 - p-value: 0.183643340203562"
```

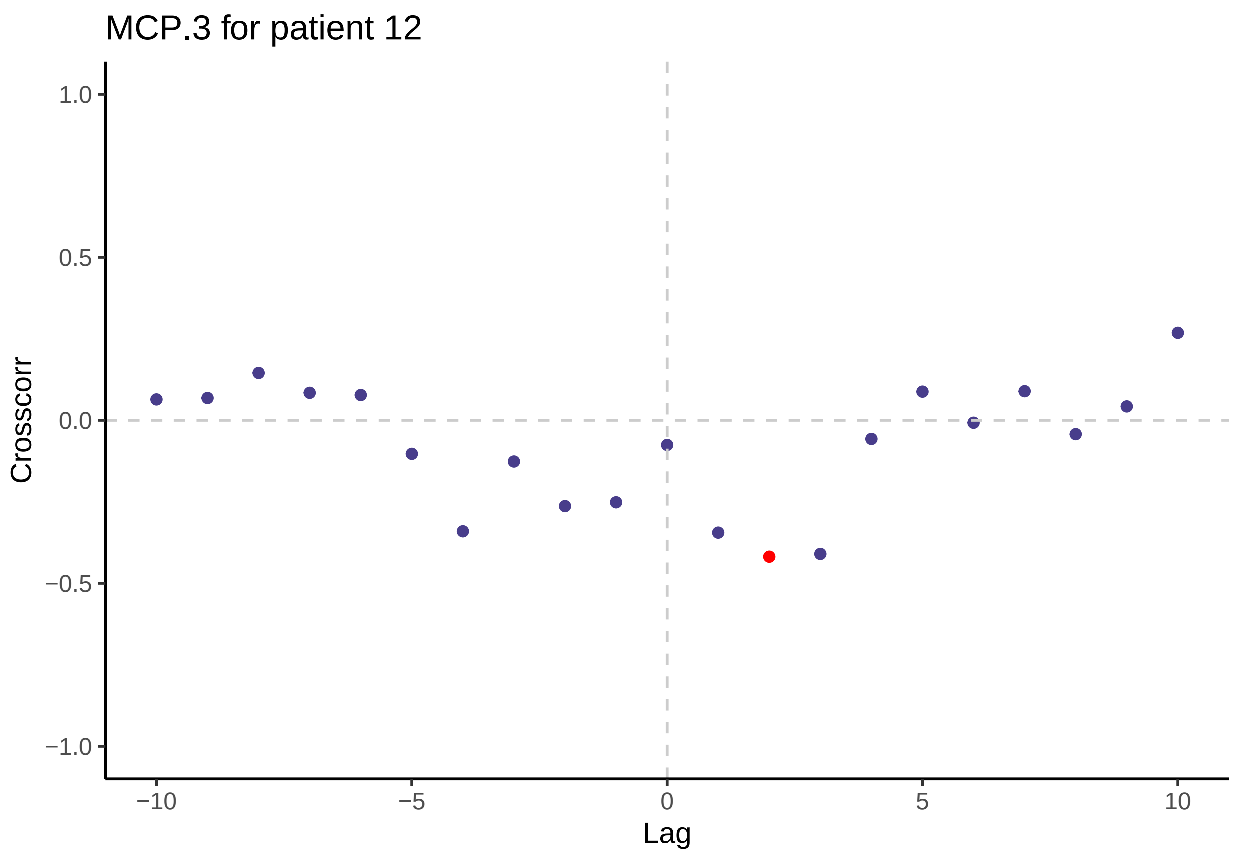


```
## [1] "MCP.3 for patient 12 - p-value: 0.107972943626631"
```

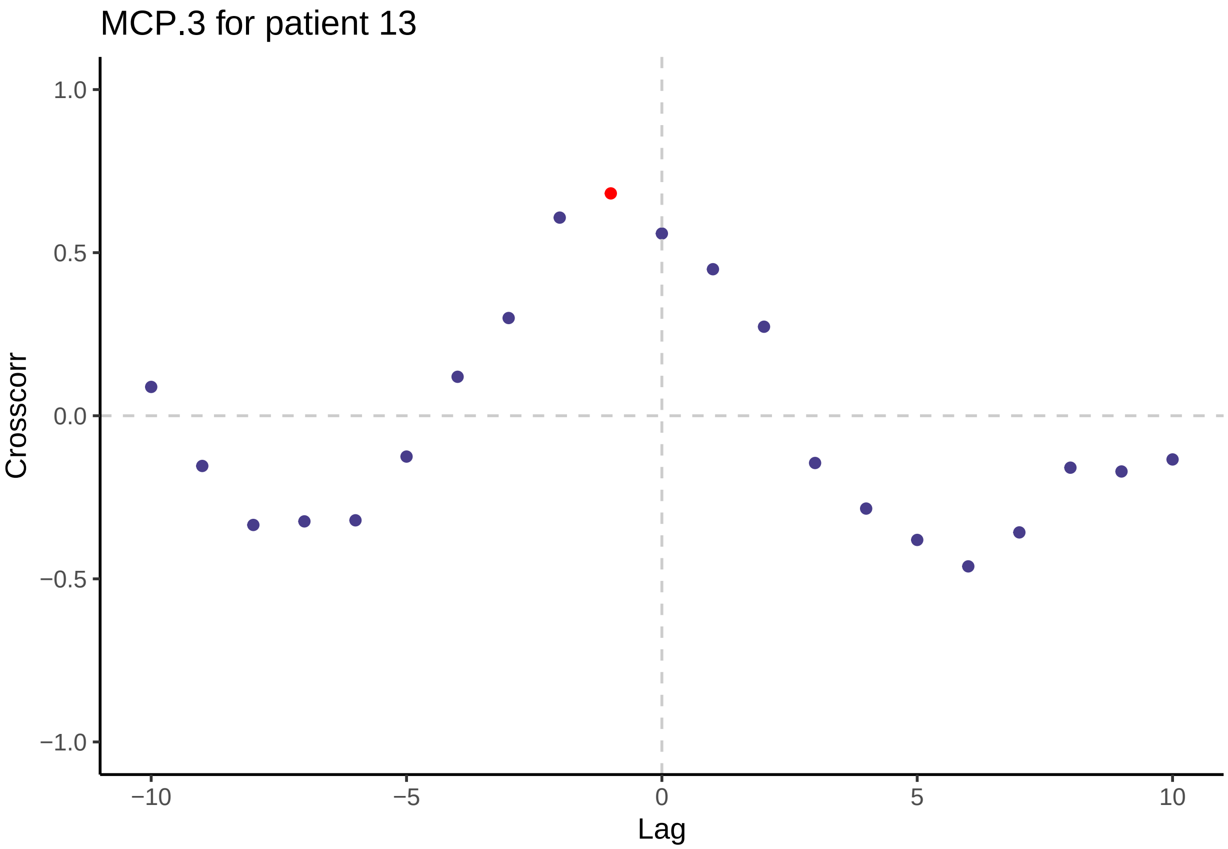


```
## [1] "MCP.3 for patient 13 - p-value: 0.868710169953029"
```

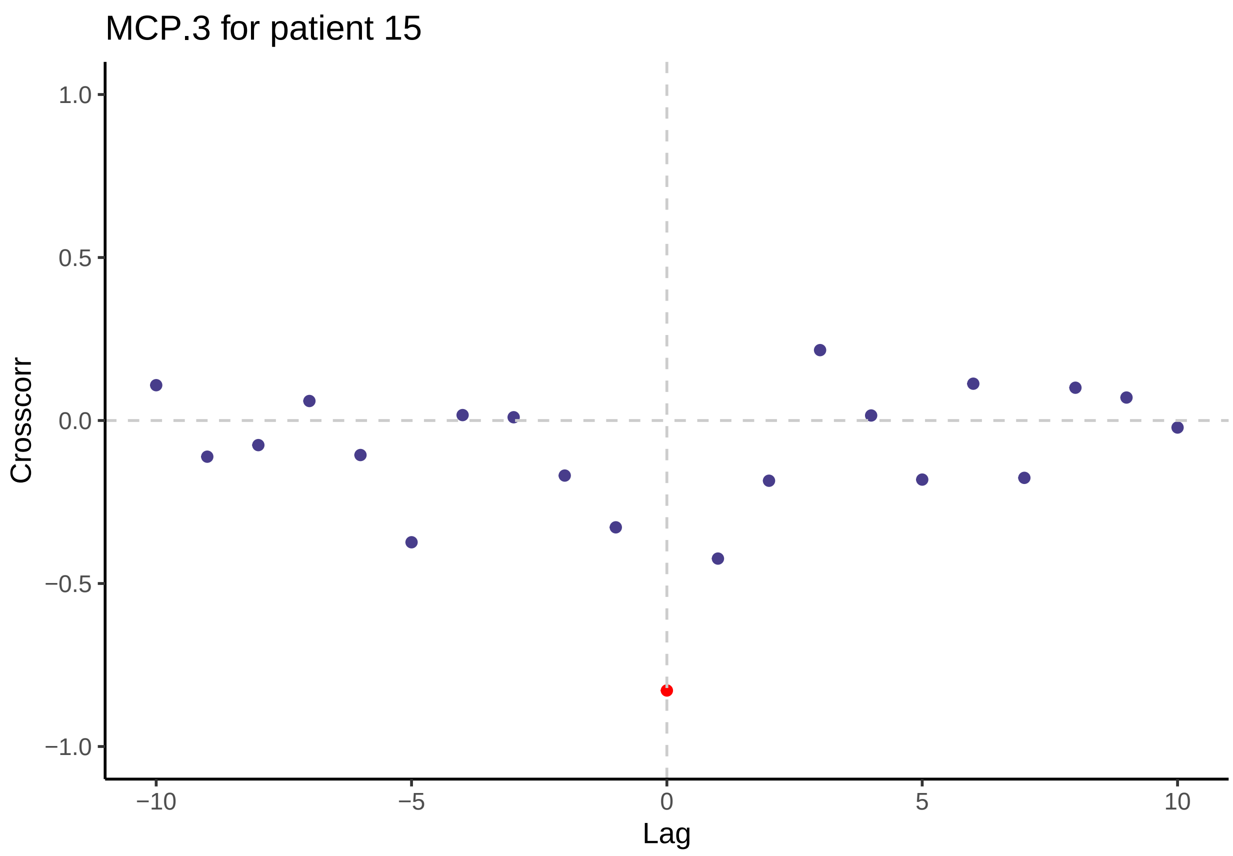


```
## [1] "MCP.3 for patient 15 - p-value: 0.0489655039112289"
```

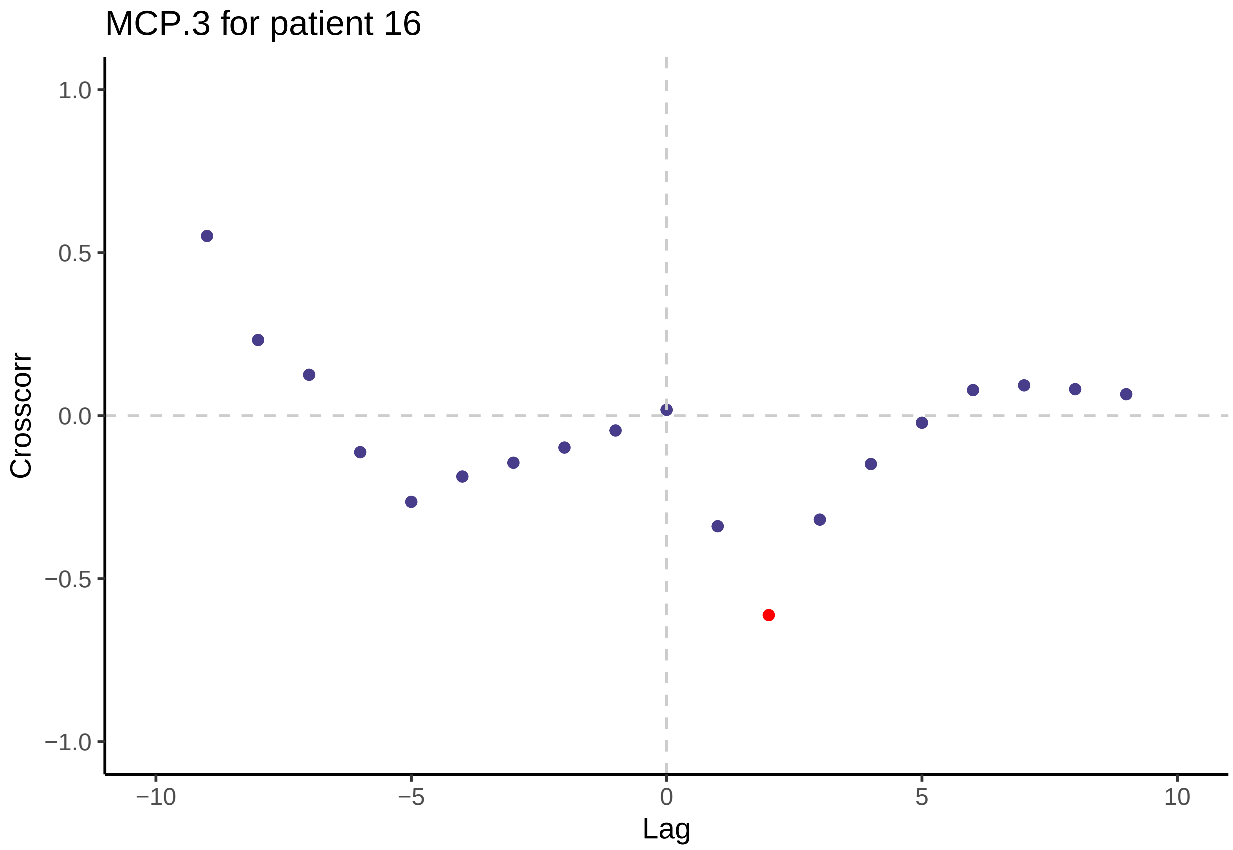


```
## [1] "MCP.3 for patient 16 - p-value: 0.348300461795745"
```

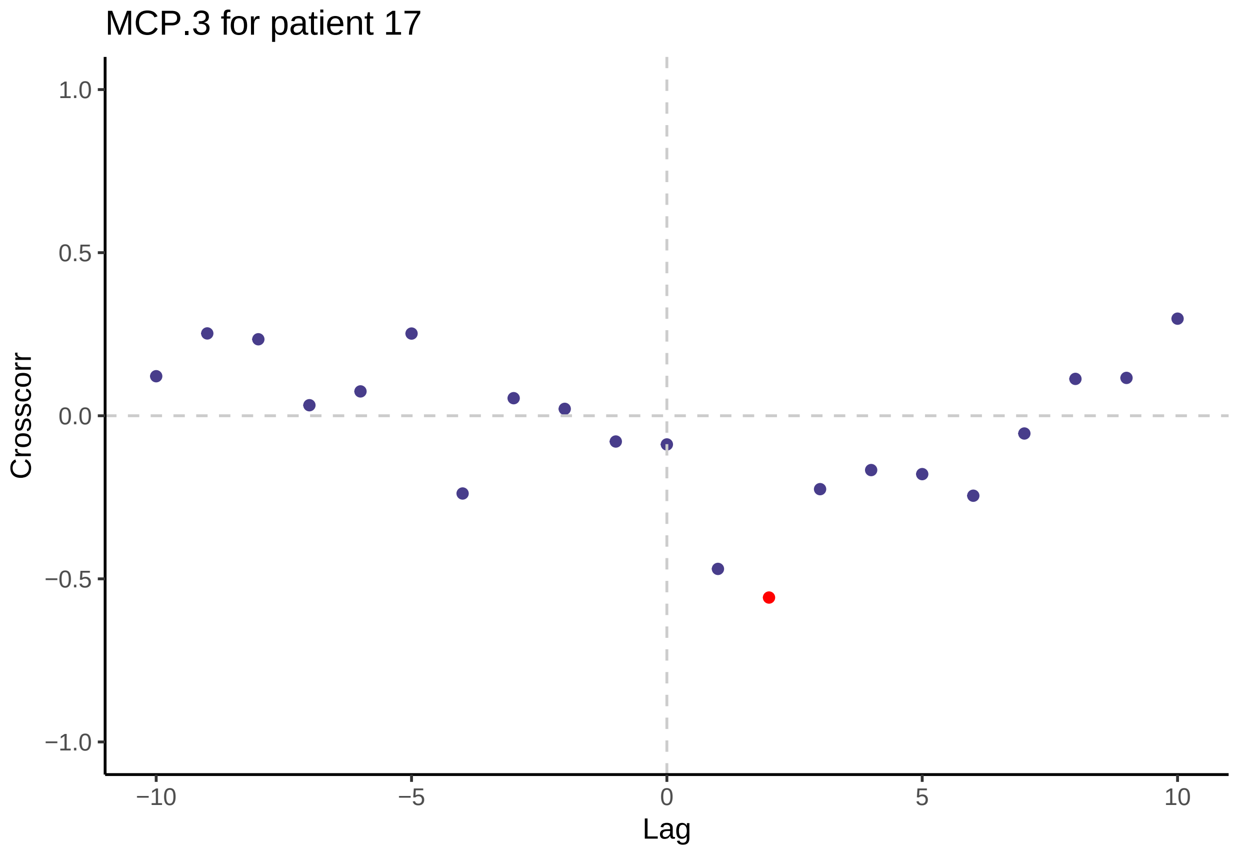


```
## [1] "MCP.3 for patient 17 - p-value: 0.497379201472858"
```

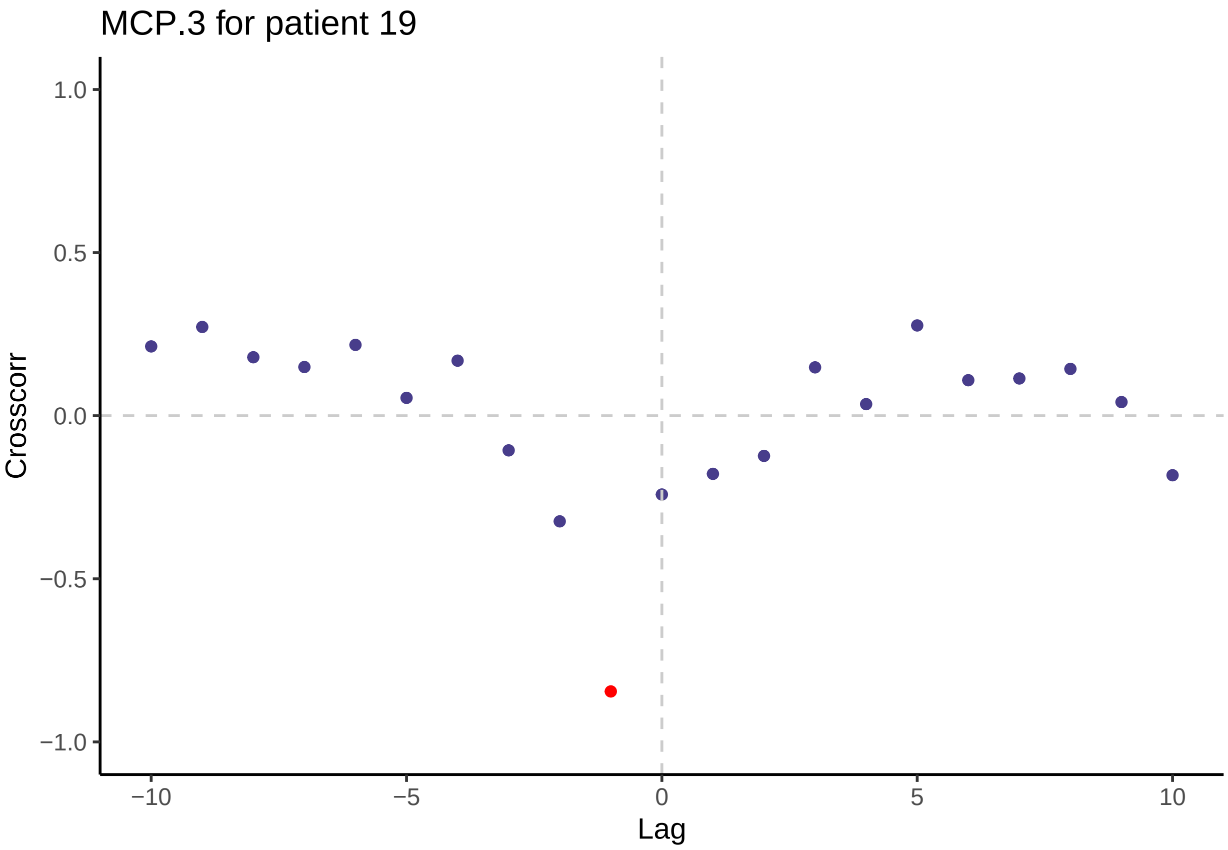


```
## [1] "MCP.3 for patient 19 - p-value: 0.91838949246731"
```

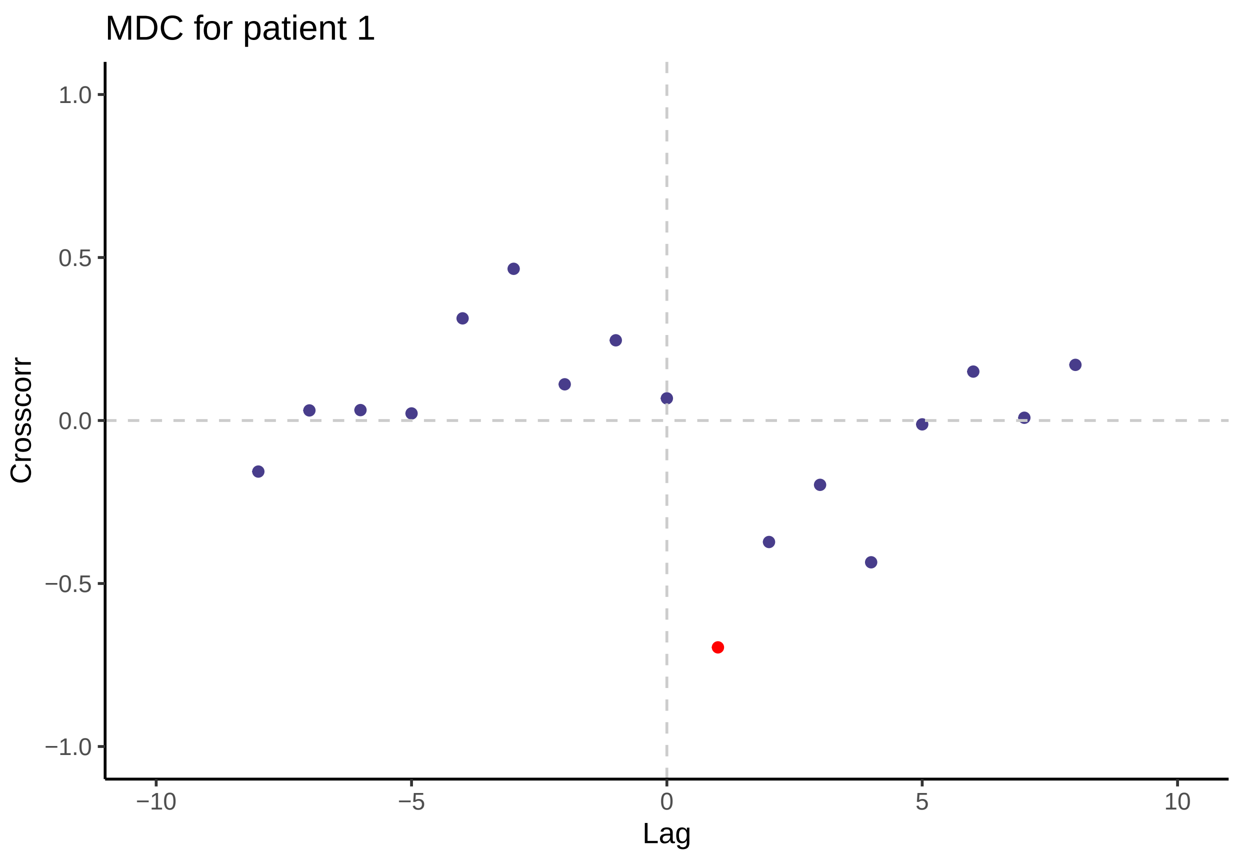


```
## [1] "MDC for patient 1 - p-value: 0.834543539522257"
```

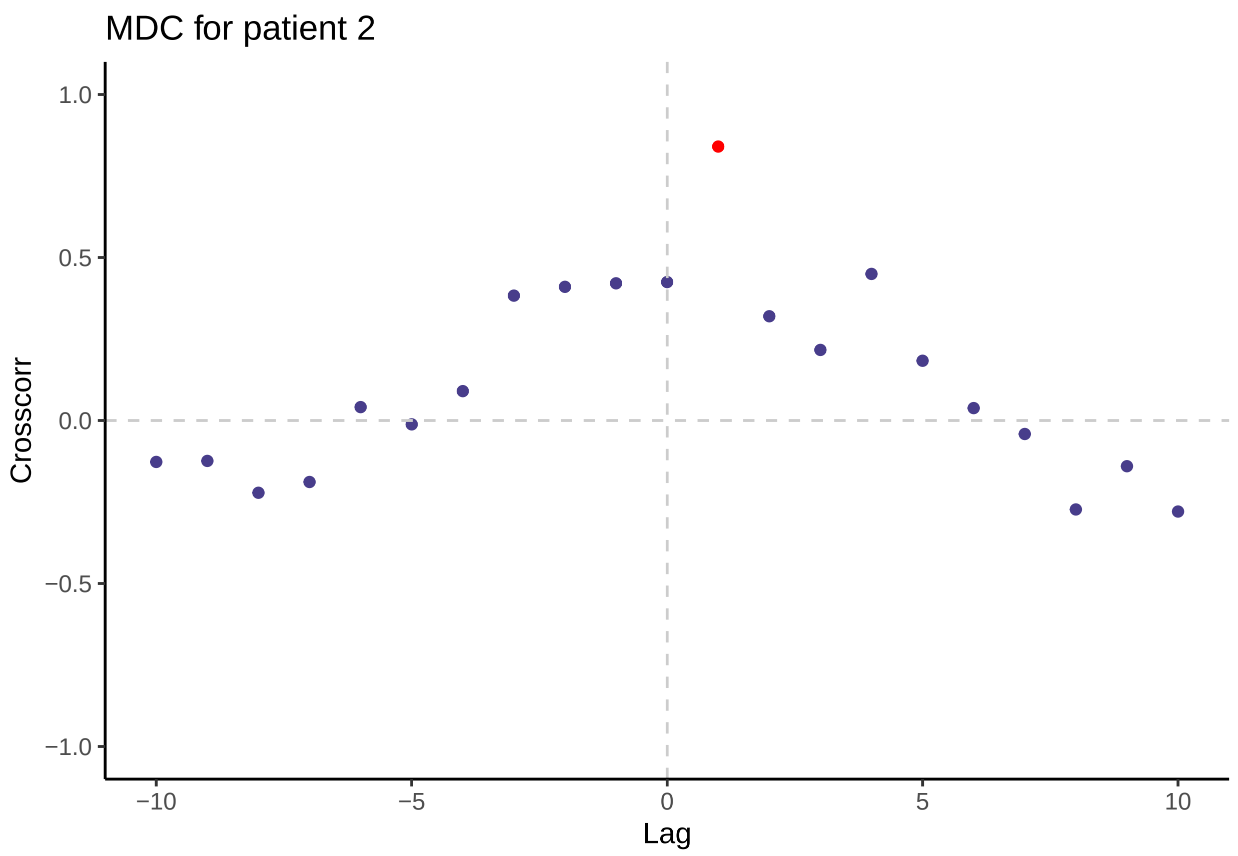


```
## [1] "MDC for patient 2 - p-value: 0.0937636552637185"
```

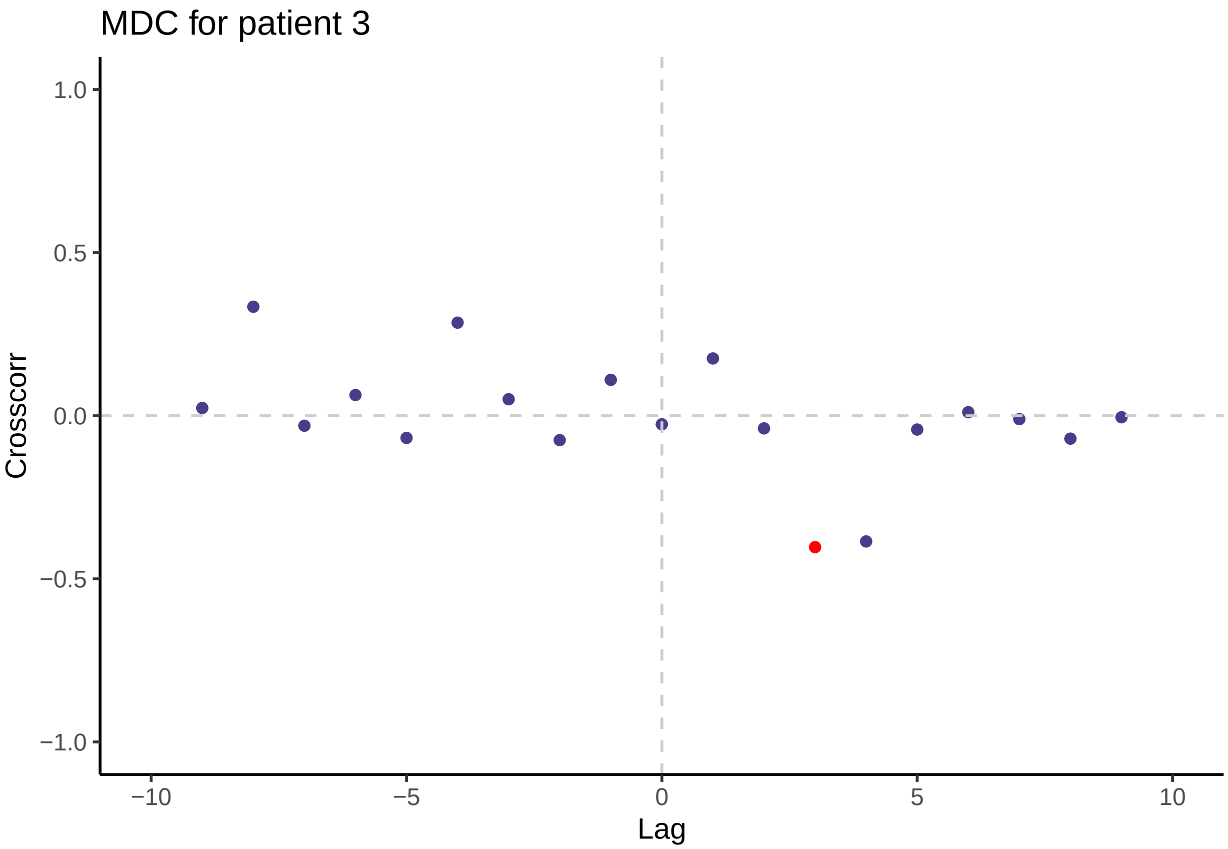


```
## [1] "MDC for patient 3 - p-value: 0.899834226158082"
```

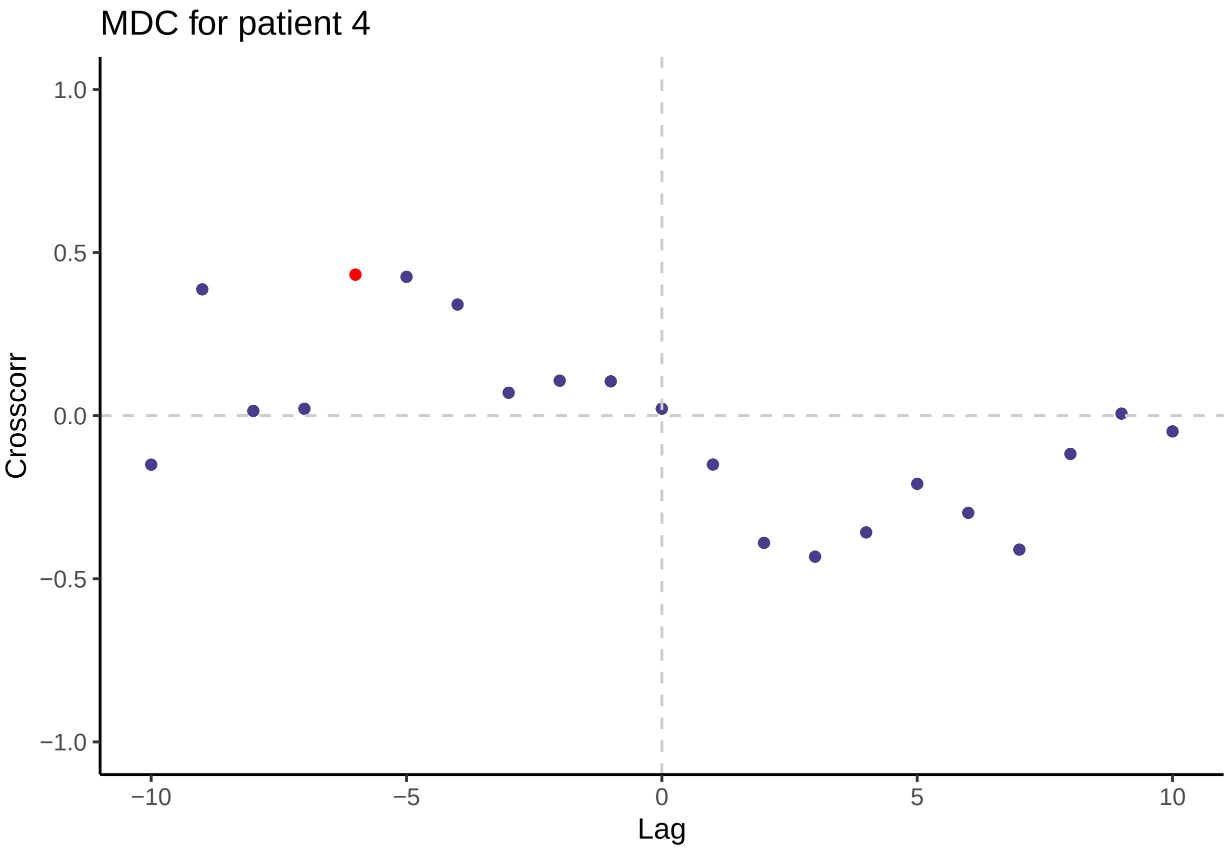


```
## [1] "MDC for patient 4 - p-value: 0.620758659118859"
```

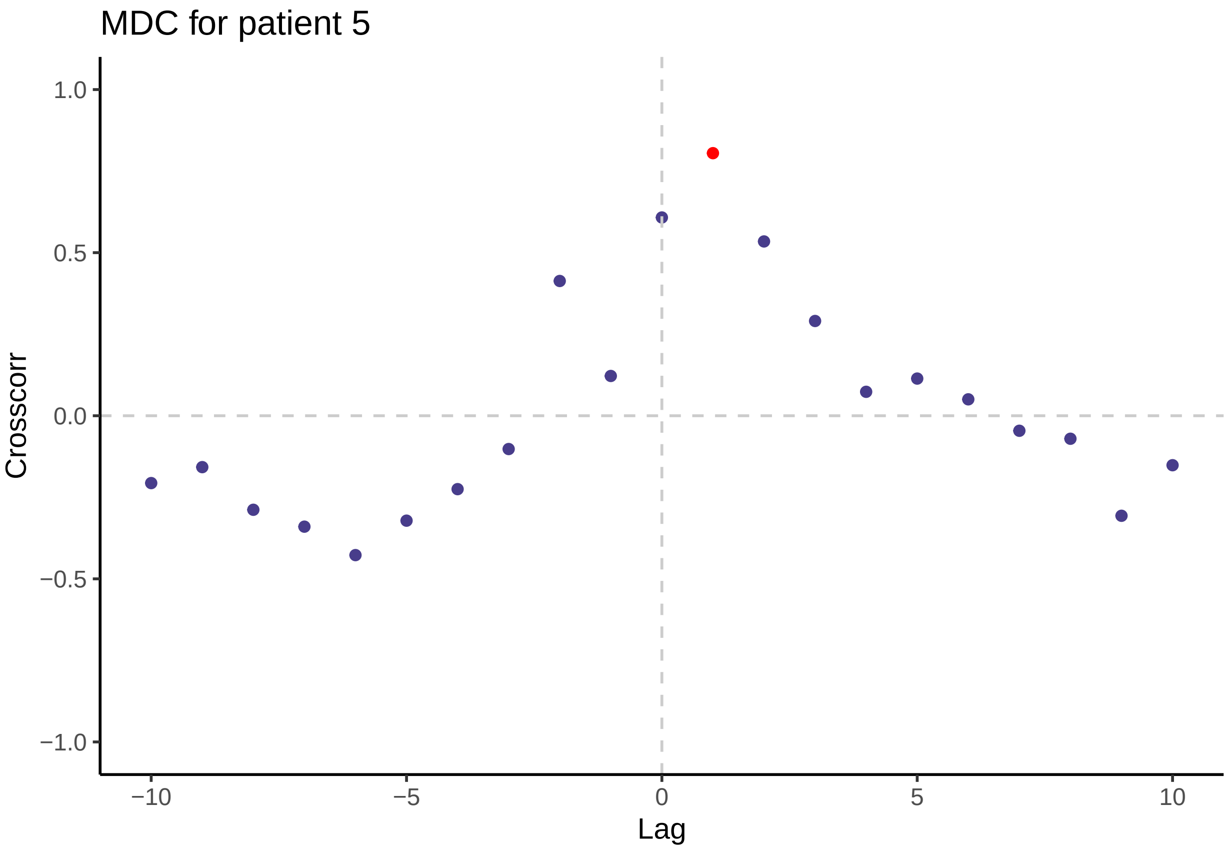


```
## [1] "MDC for patient 5 - p-value: 0.81611923570943"
```

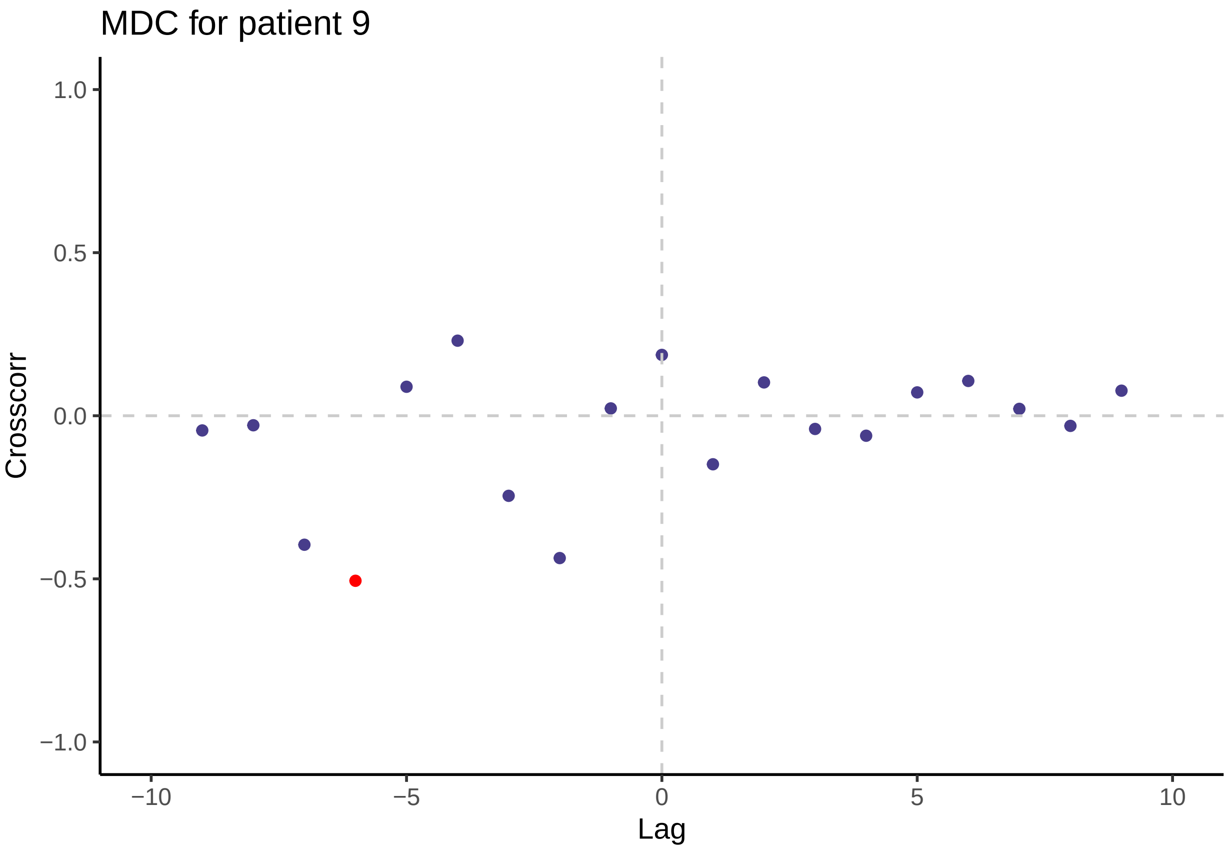


```
## [1] "MDC for patient 9 - p-value: 0.266757076197184"
```

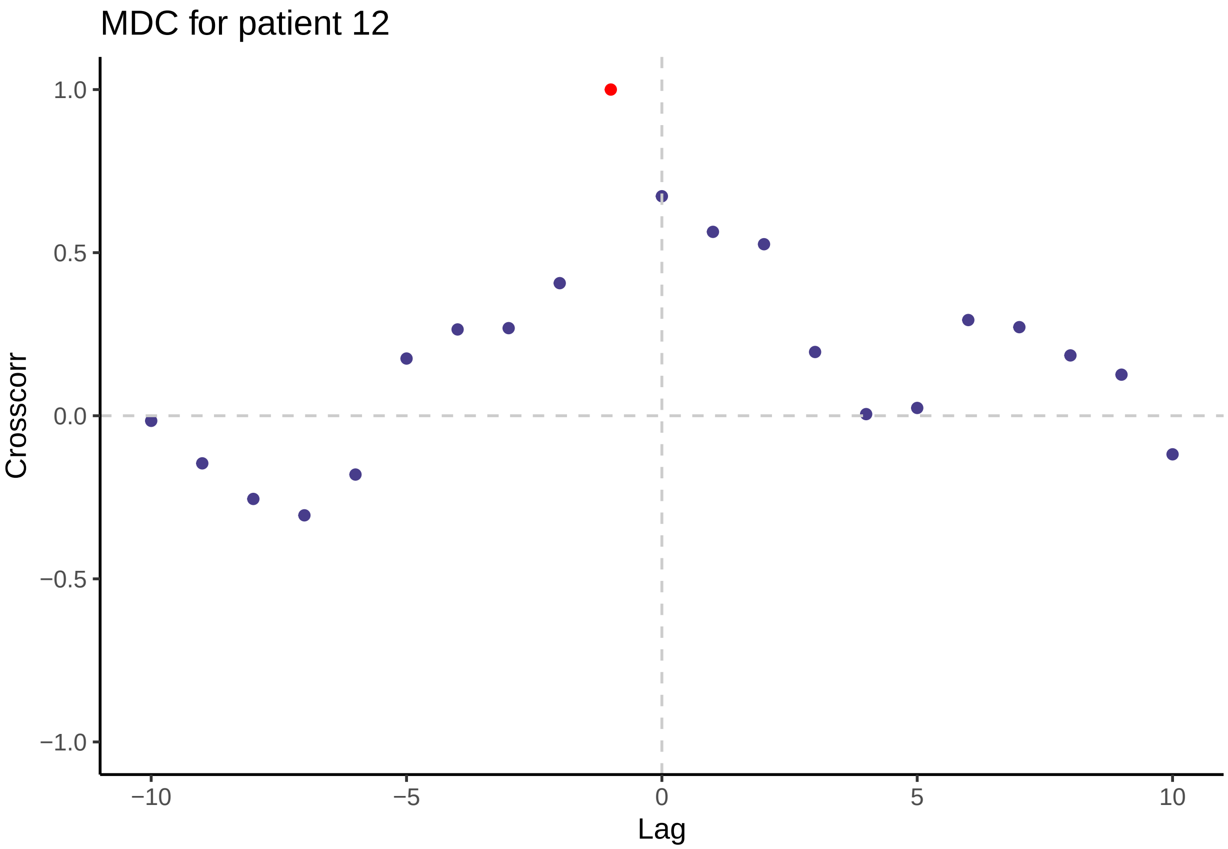


```
## [1] "MDC for patient 12 - p-value: 0.0151493941737596"
```

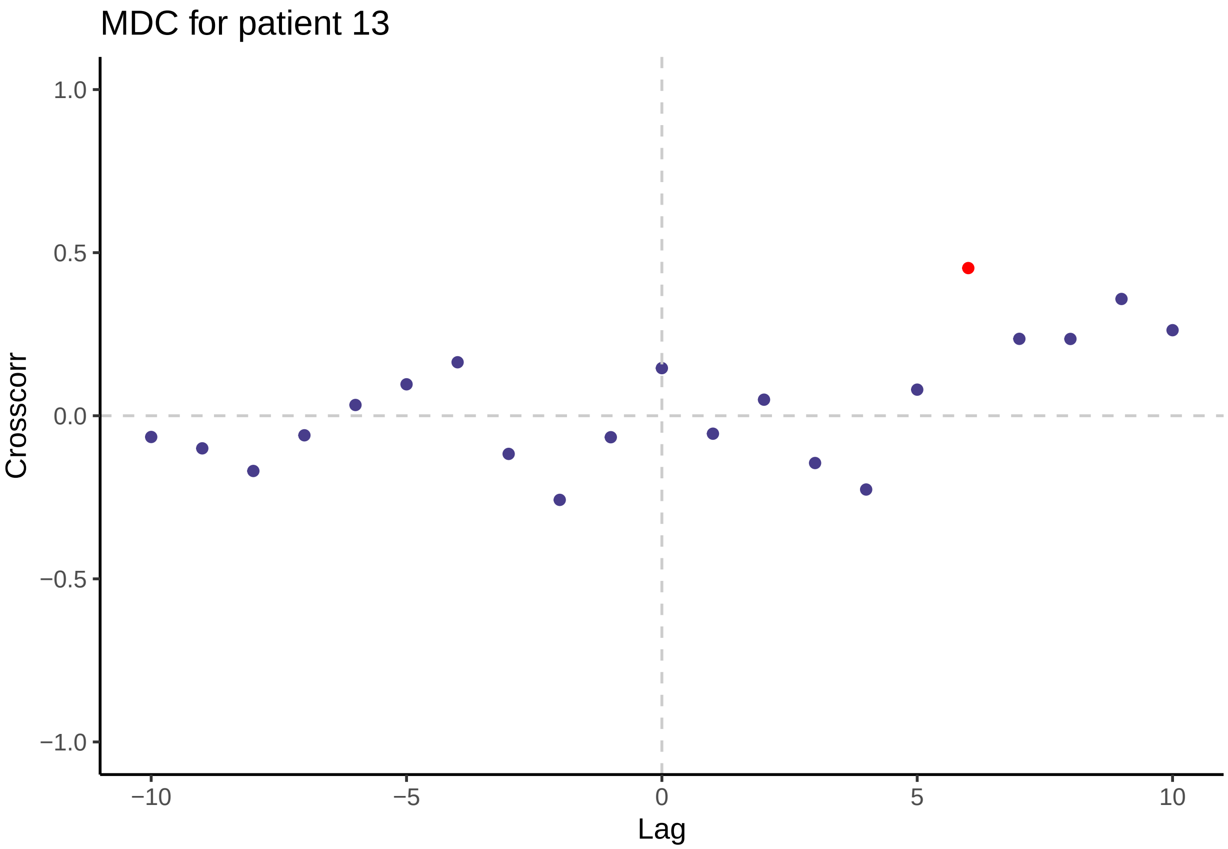


```
## [1] "MDC for patient 13 - p-value: 0.348797516493189"
```

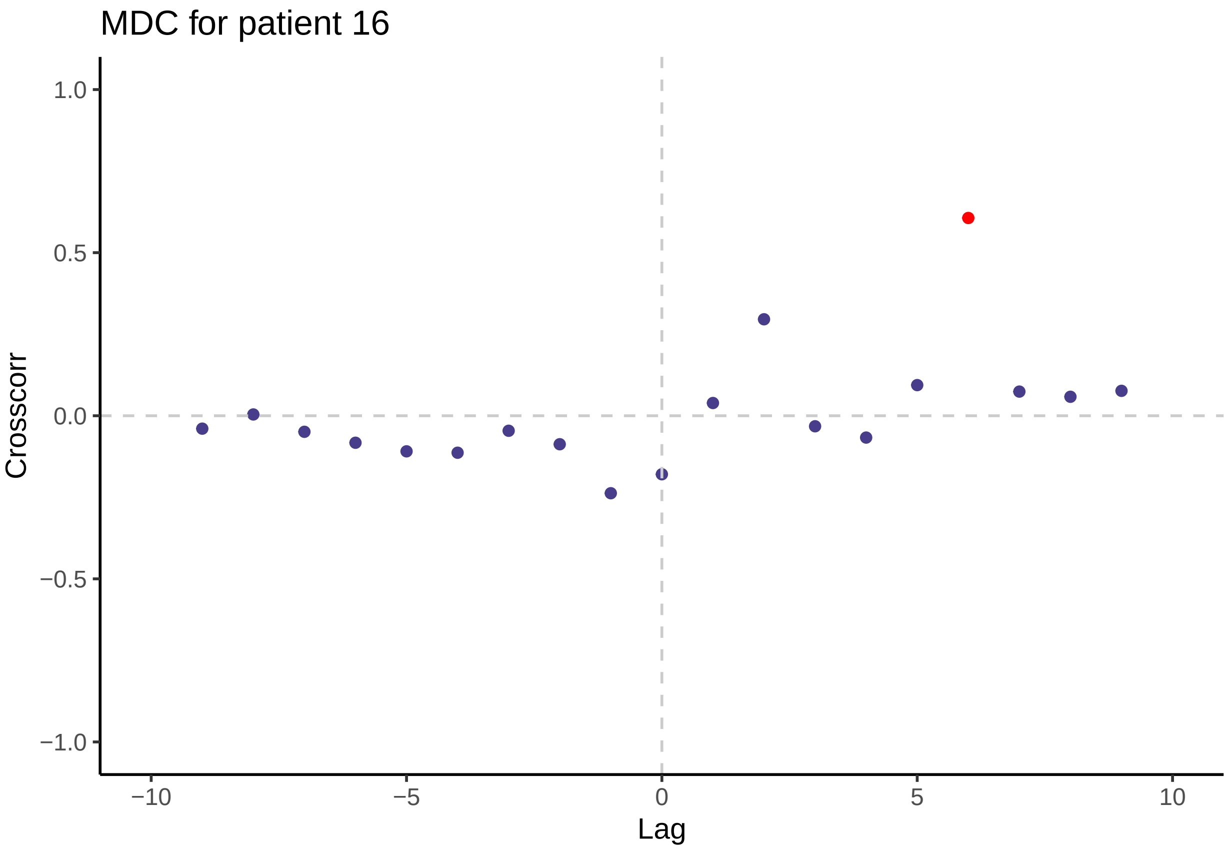


```
## [1] "MDC for patient 16 - p-value: 0.802712176585513"
```

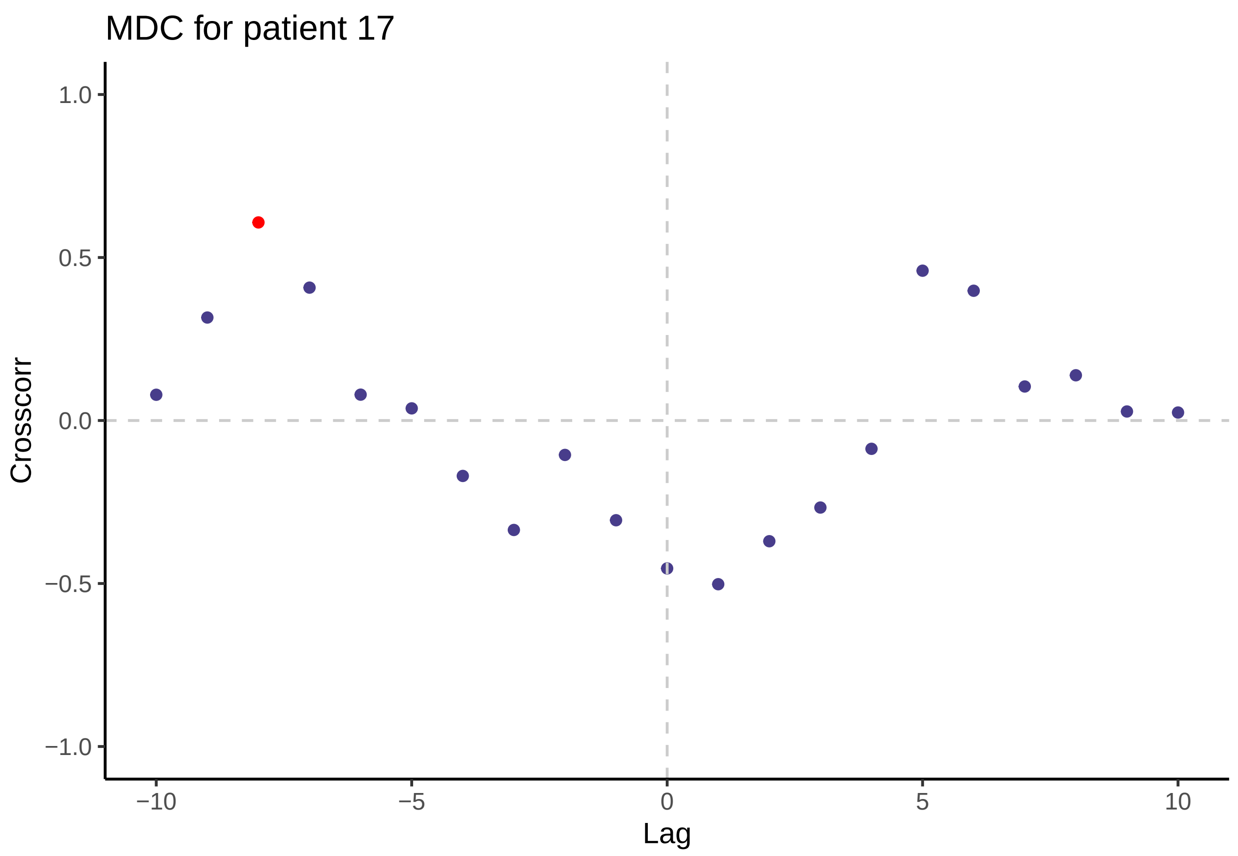


```
## [1] "MDC for patient 17 - p-value: 0.954927292185895"
```

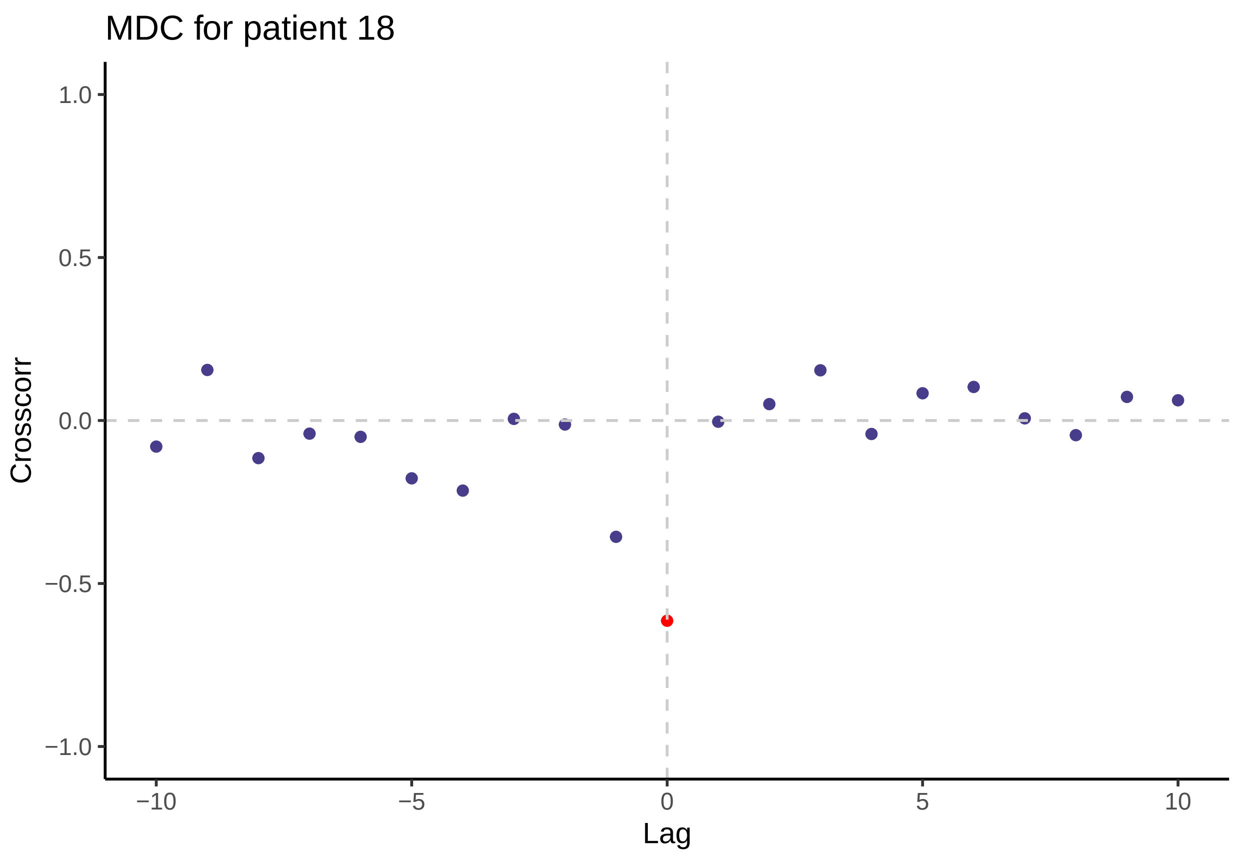


```
## [1] "MDC for patient 18 - p-value: 0.208814697031383"
```

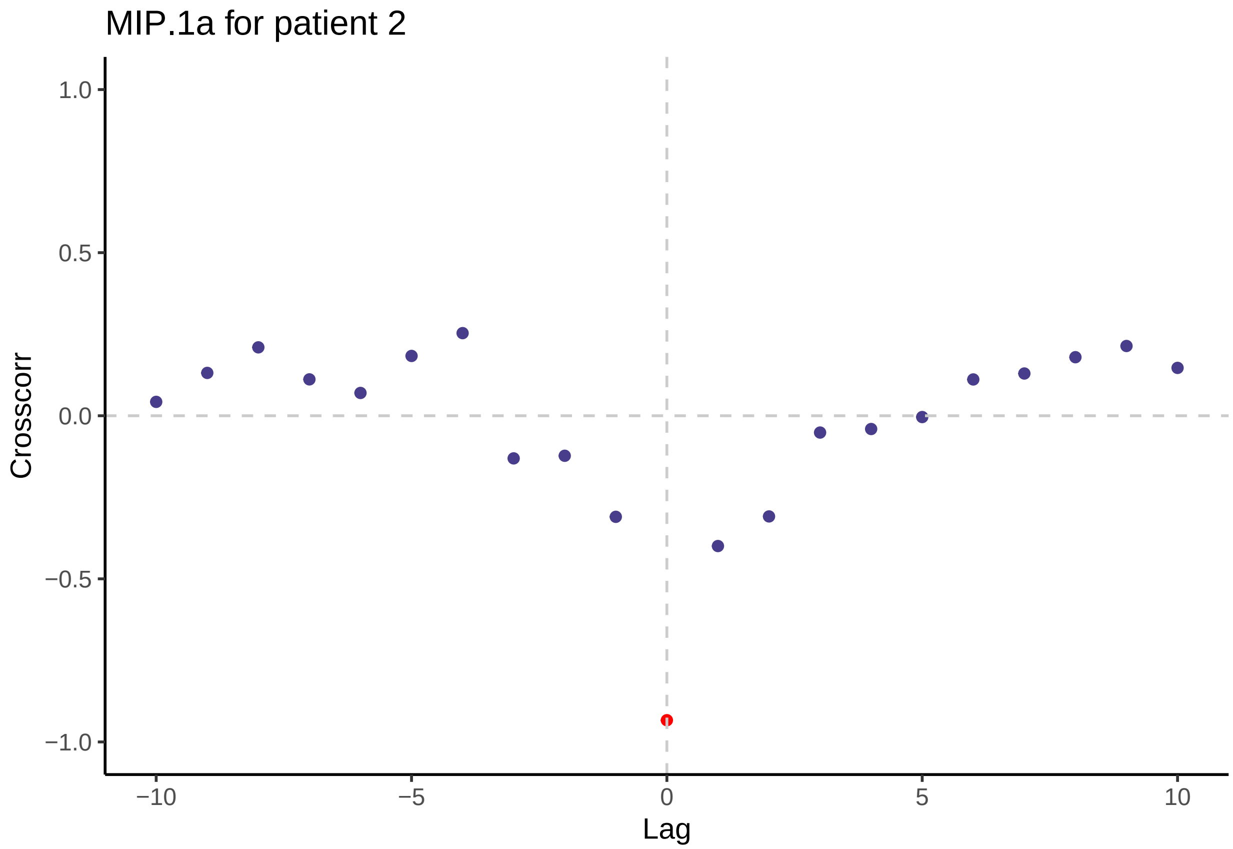


```
## [1] "MIP.1a for patient 2 - p-value: 0.689346957112172"
```

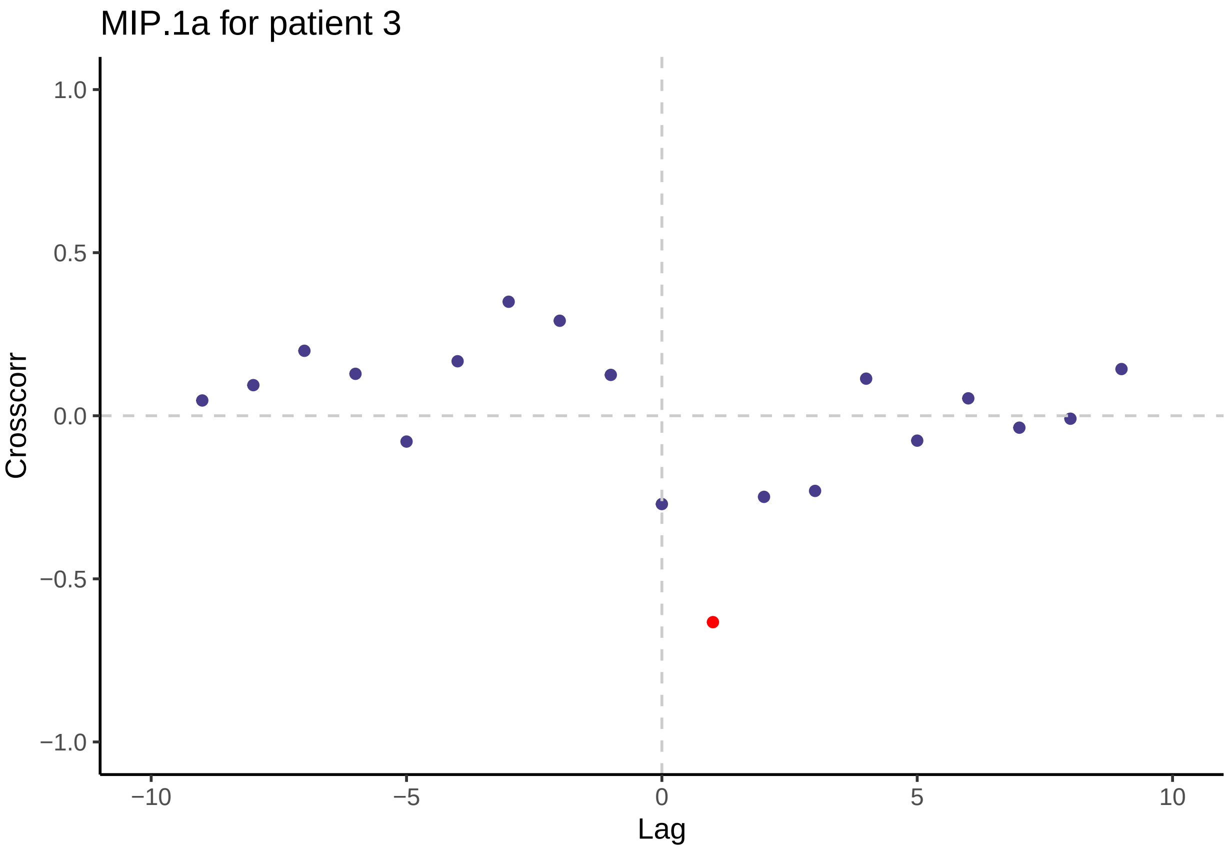


```
## [1] "MIP.1a for patient 3 - p-value: 0.899589763694176"
```

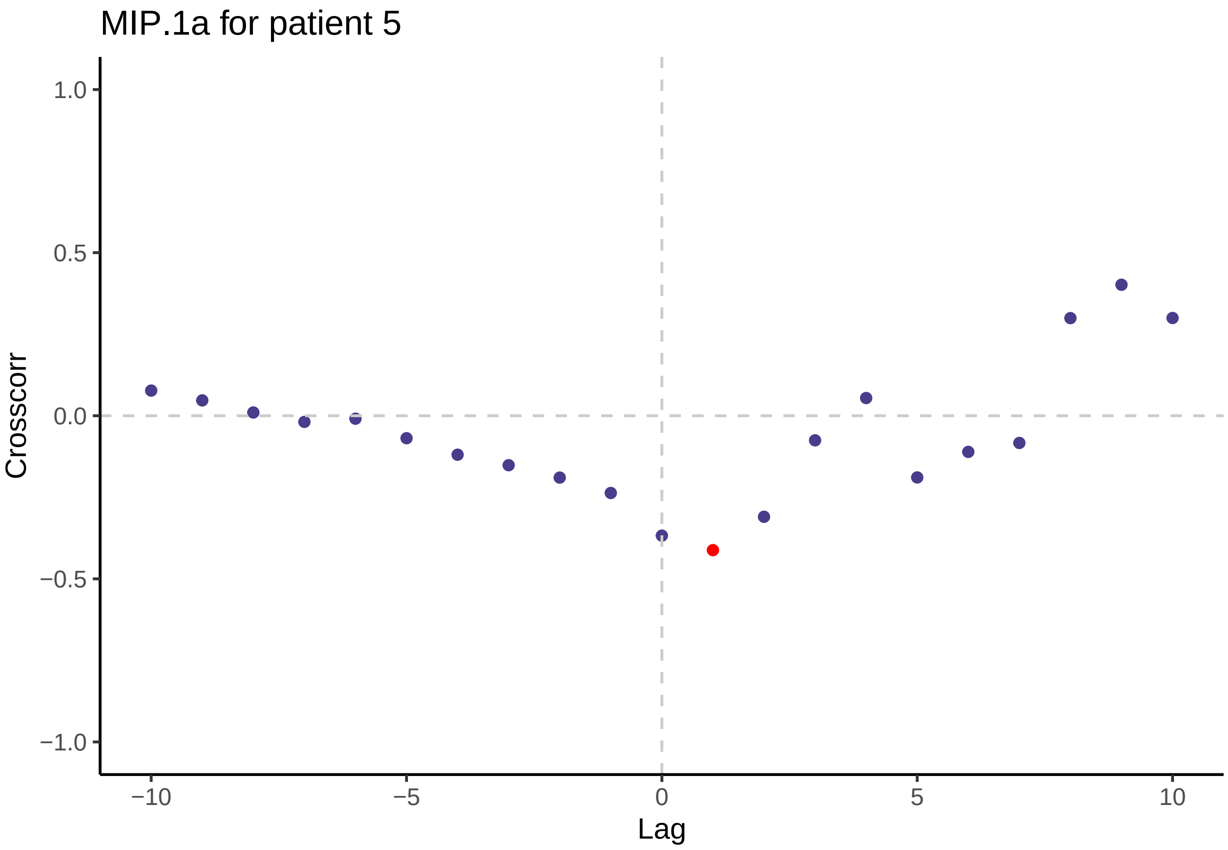


```
## [1] "MIP.1a for patient 5 - p-value: 0.245704460737188"
```

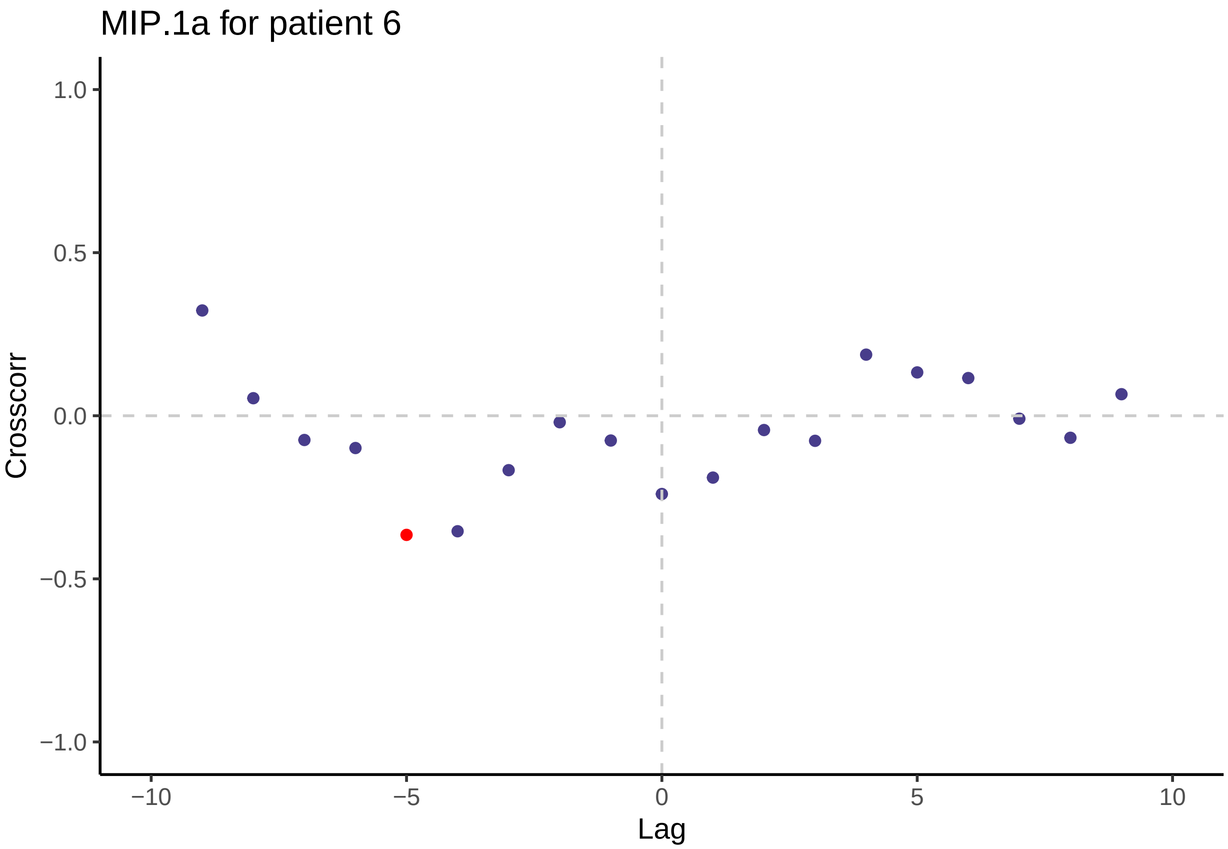


```
## [1] "MIP.1a for patient 6 - p-value: 0.250619376687361"
```

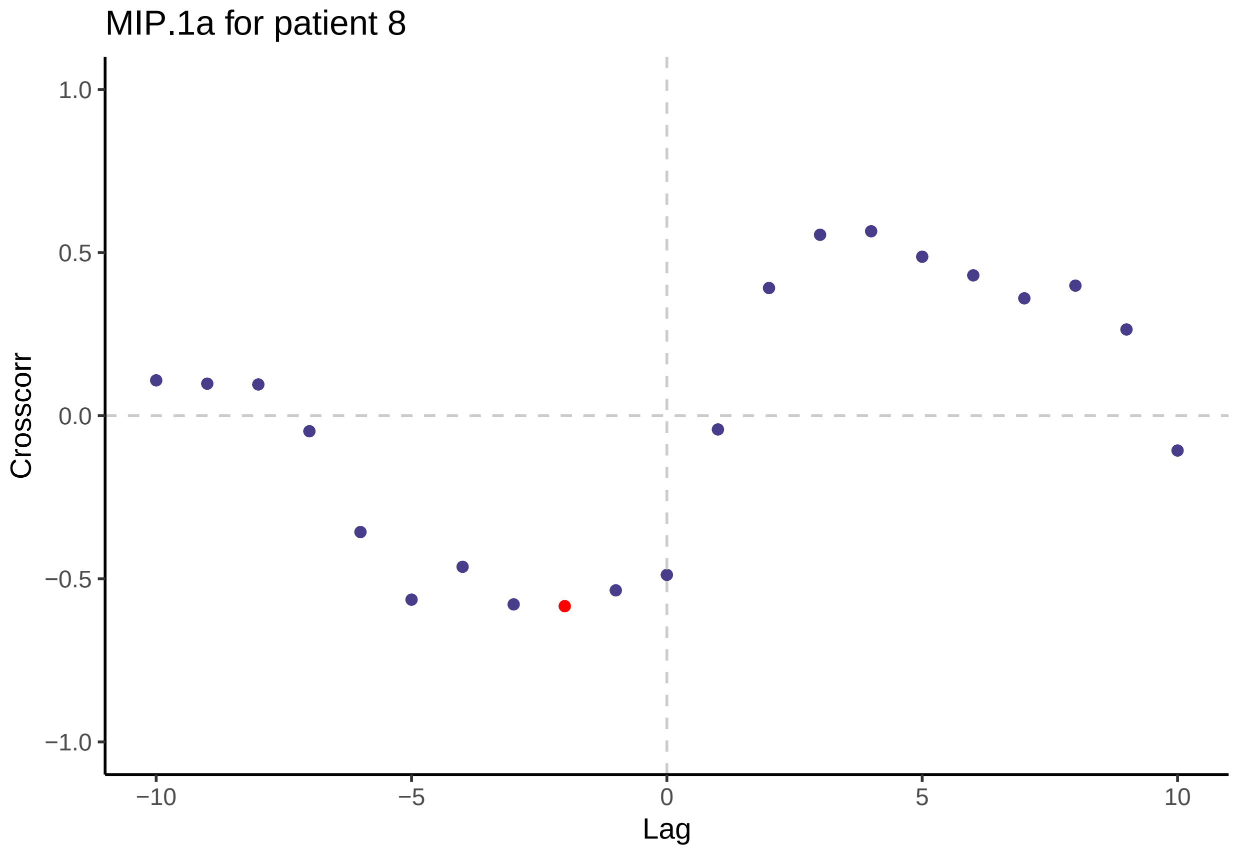


```
## [1] "MIP.1a for patient 8 - p-value: 0.996490276328955"
```

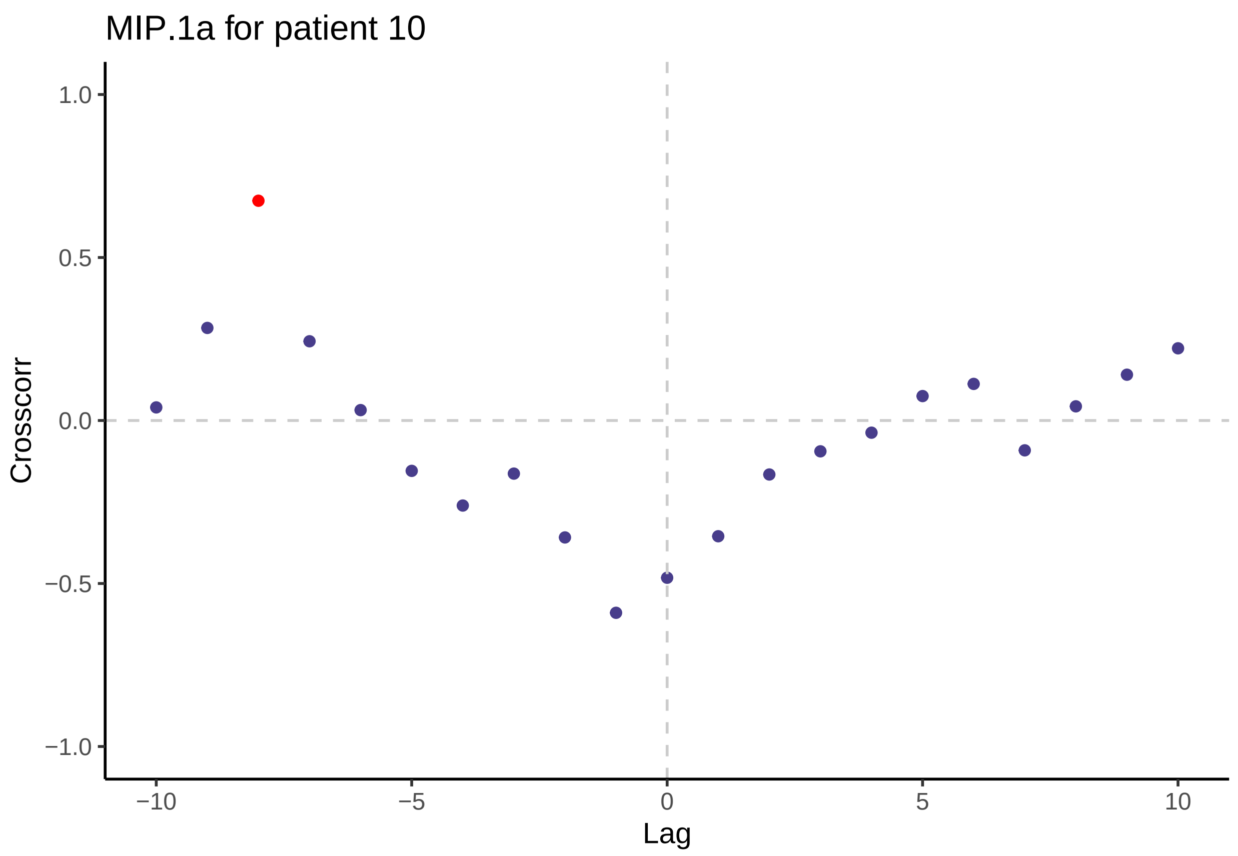


```
## [1] "MIP.1a for patient 10 - p-value: 0.507922282542812"
```

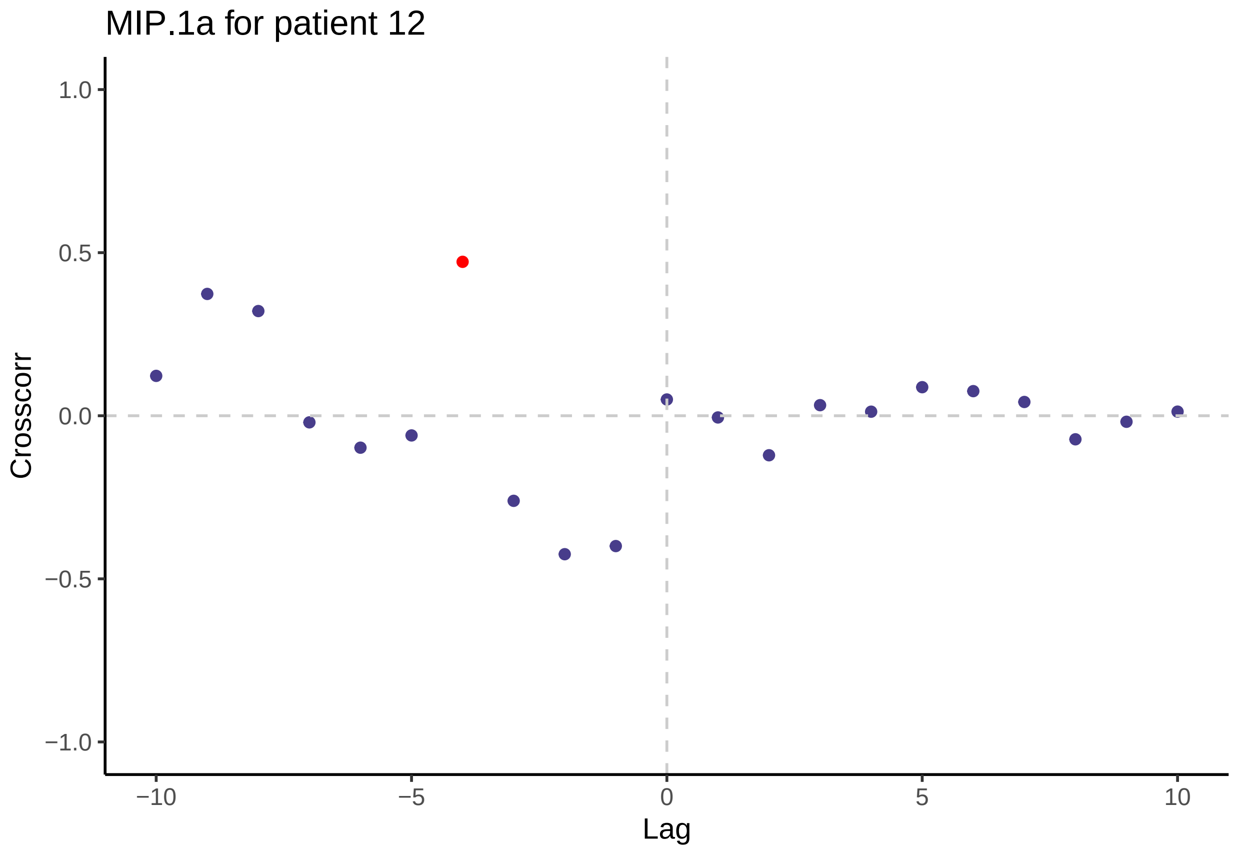


```
## [1] "MIP.1a for patient 12 - p-value: 0.904134846851753"
```

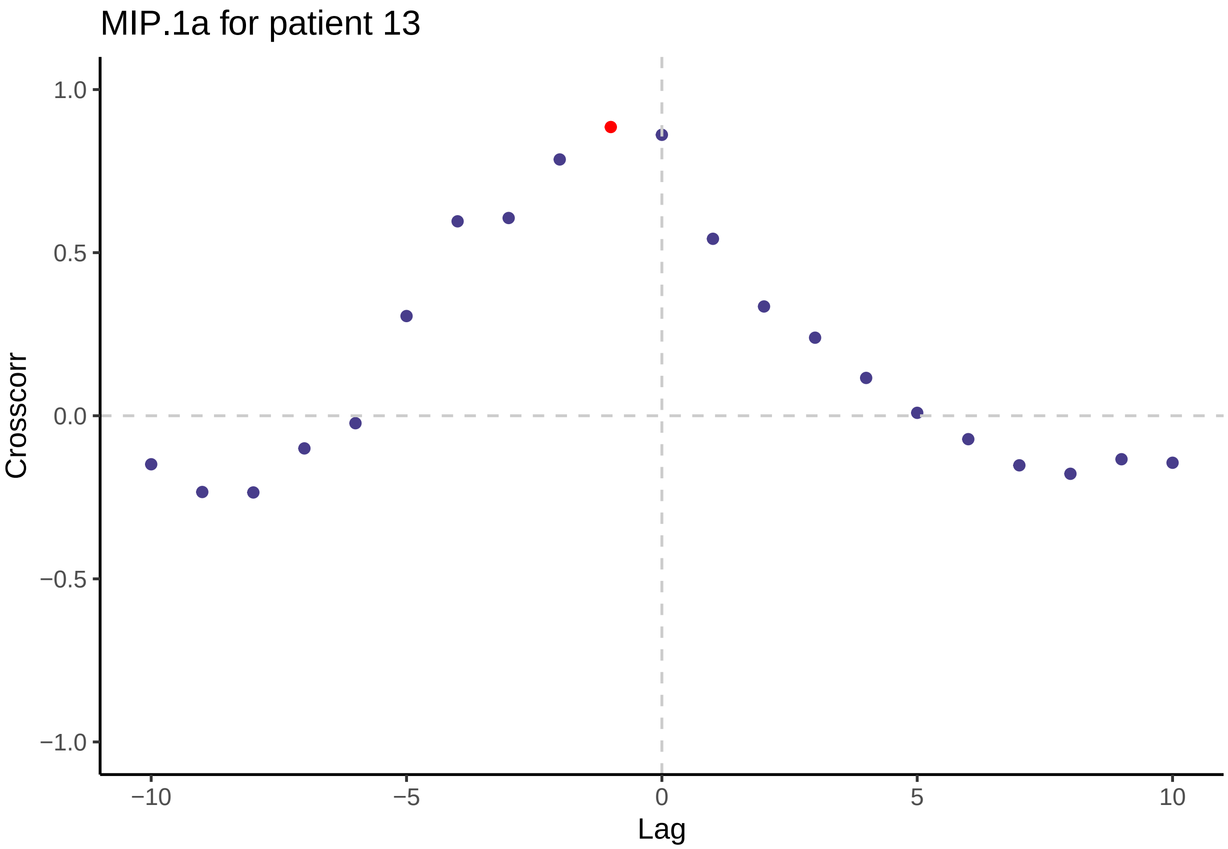


```
## [1] "MIP.1a for patient 13 - p-value: 0.0406870898691801"
```

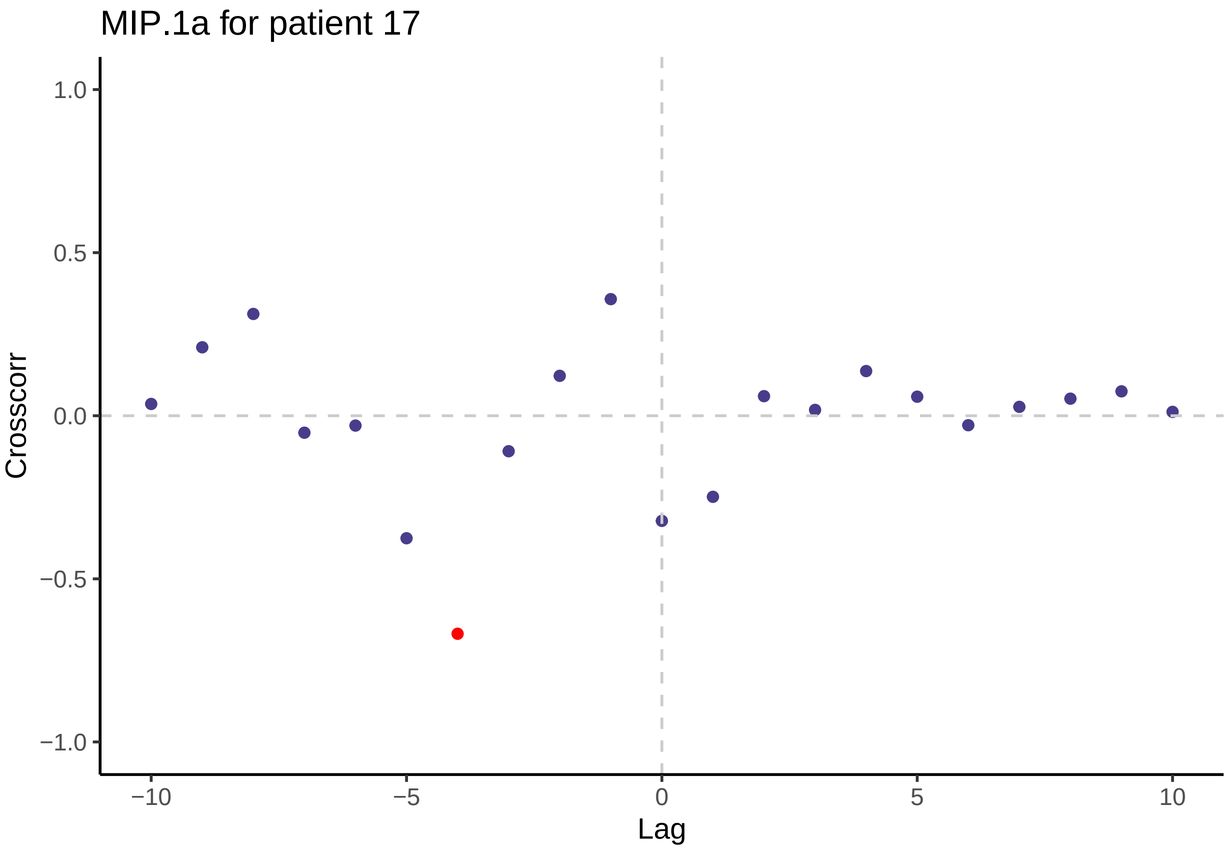


```
## [1] "MIP.1a for patient 17 - p-value: 0.741984632226767"
```

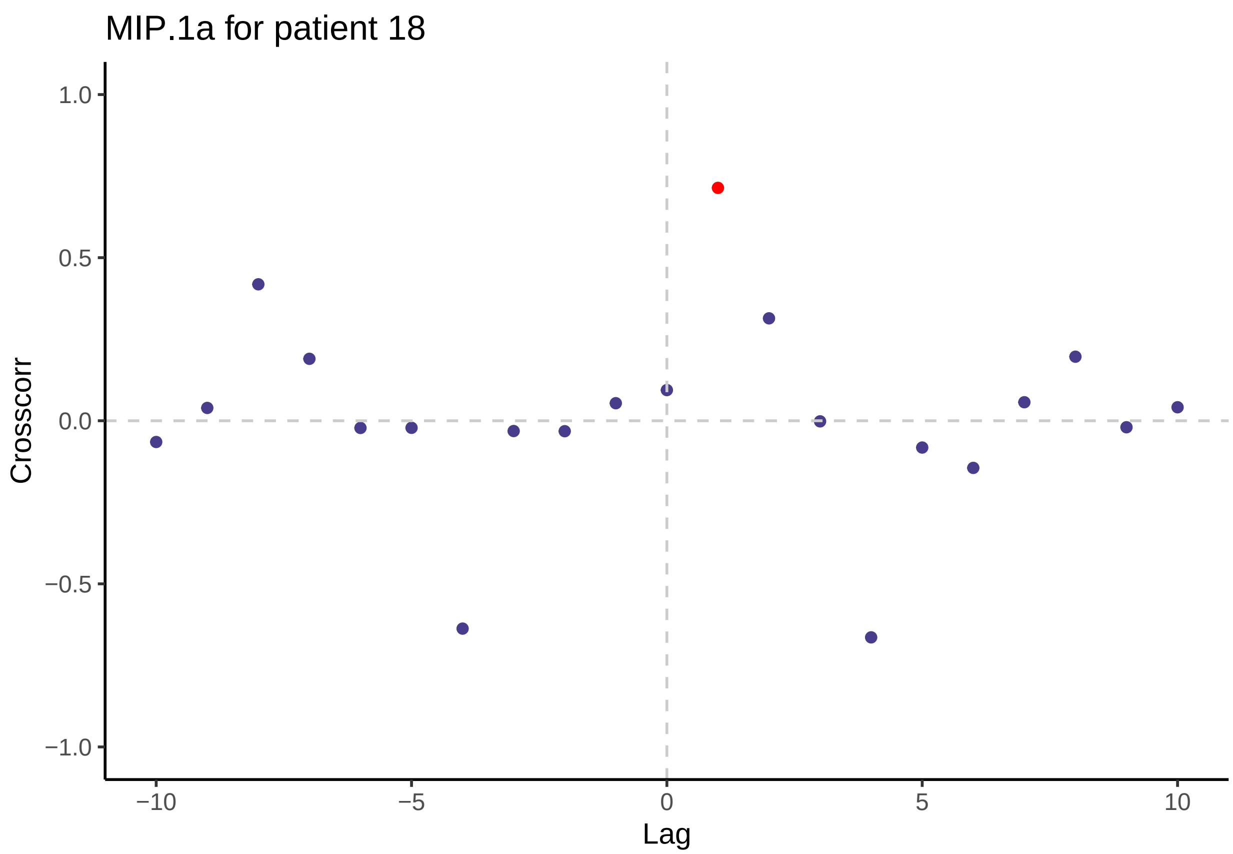


```
## [1] "MIP.1a for patient 18 - p-value: 0.773230944641706"
## Warning: Removed 1 rows containing missing values (geom_point).
```

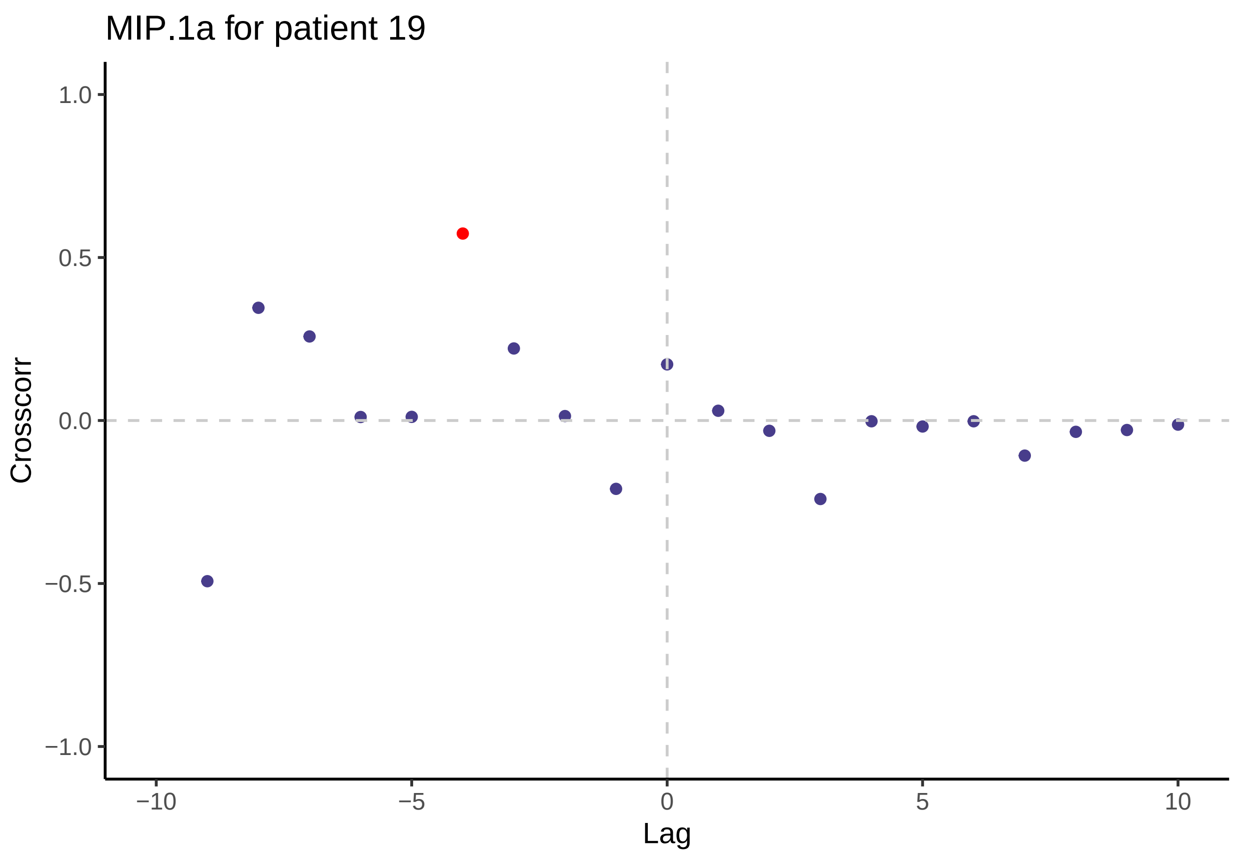


```
## [1] "MIP.1a for patient 19 - p-value: 0.654403619359678"
```

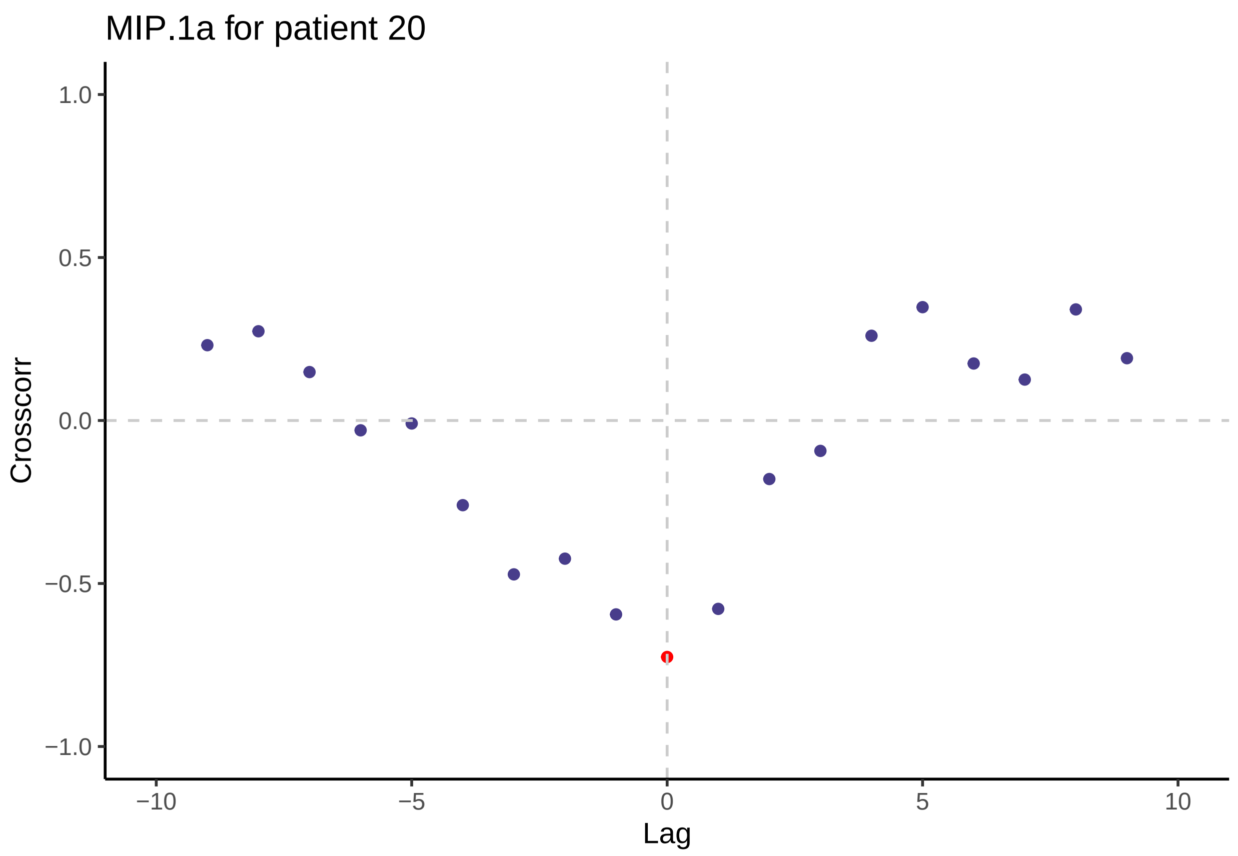


```
## [1] "MIP.1a for patient 20 - p-value: 0.412647424827353"
```

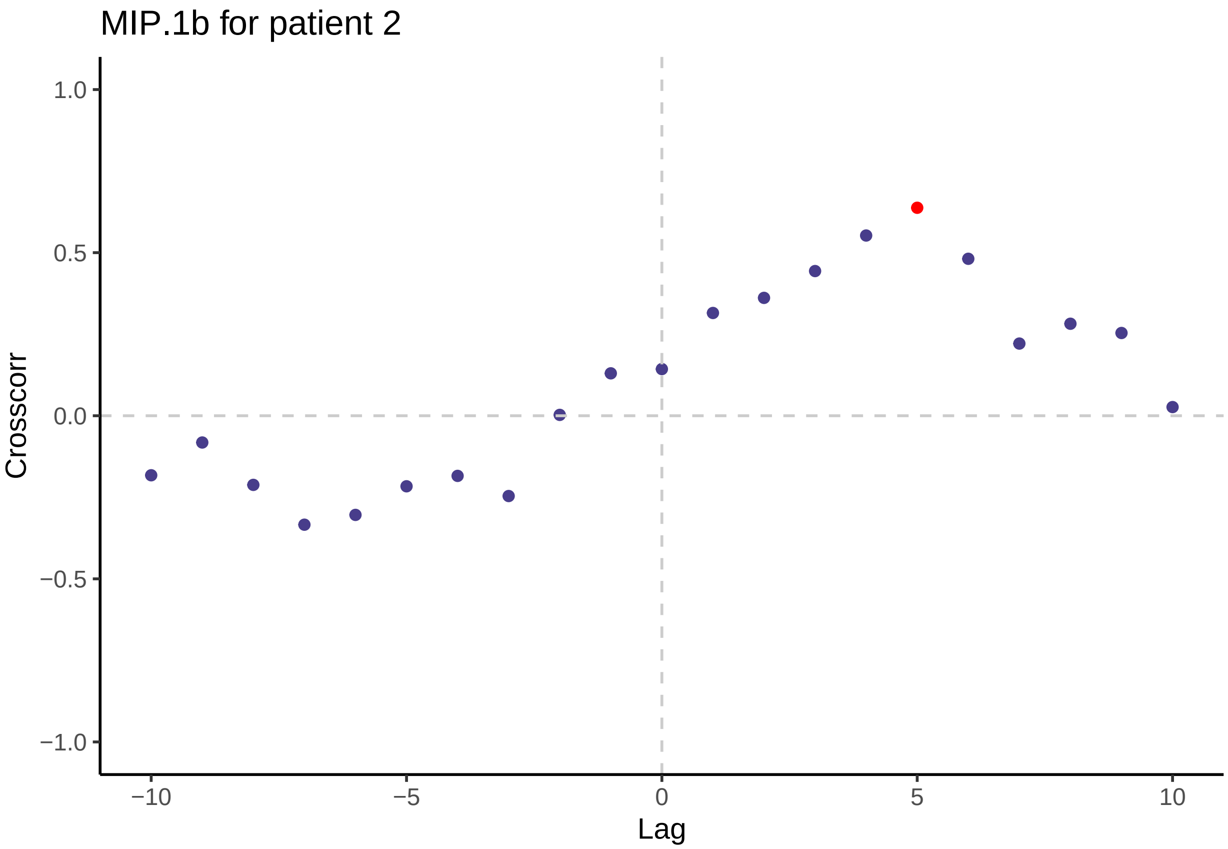


```
## [1] "MIP.1b for patient 2 - p-value: 0.146487457464424"
```

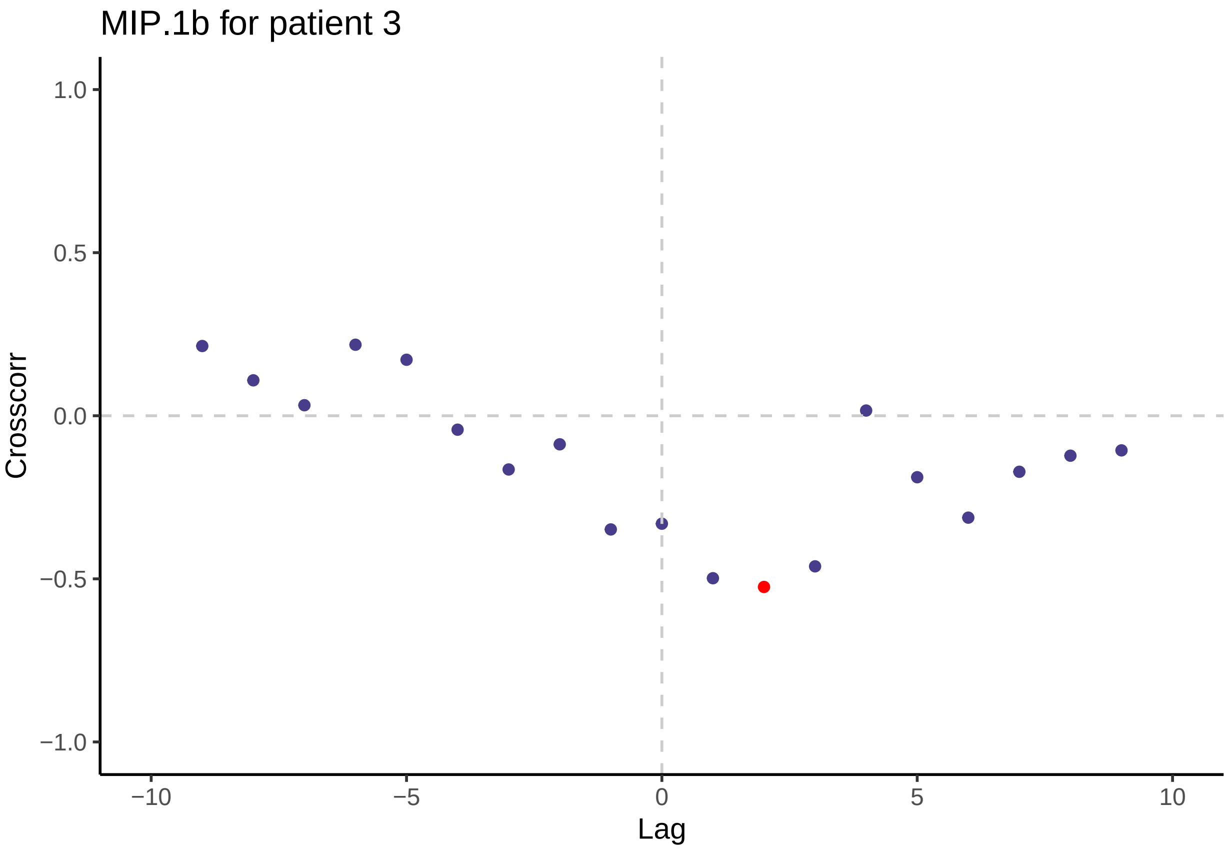


```
## [1] "MIP.1b for patient 3 - p-value: 0.0194552050219632"
```

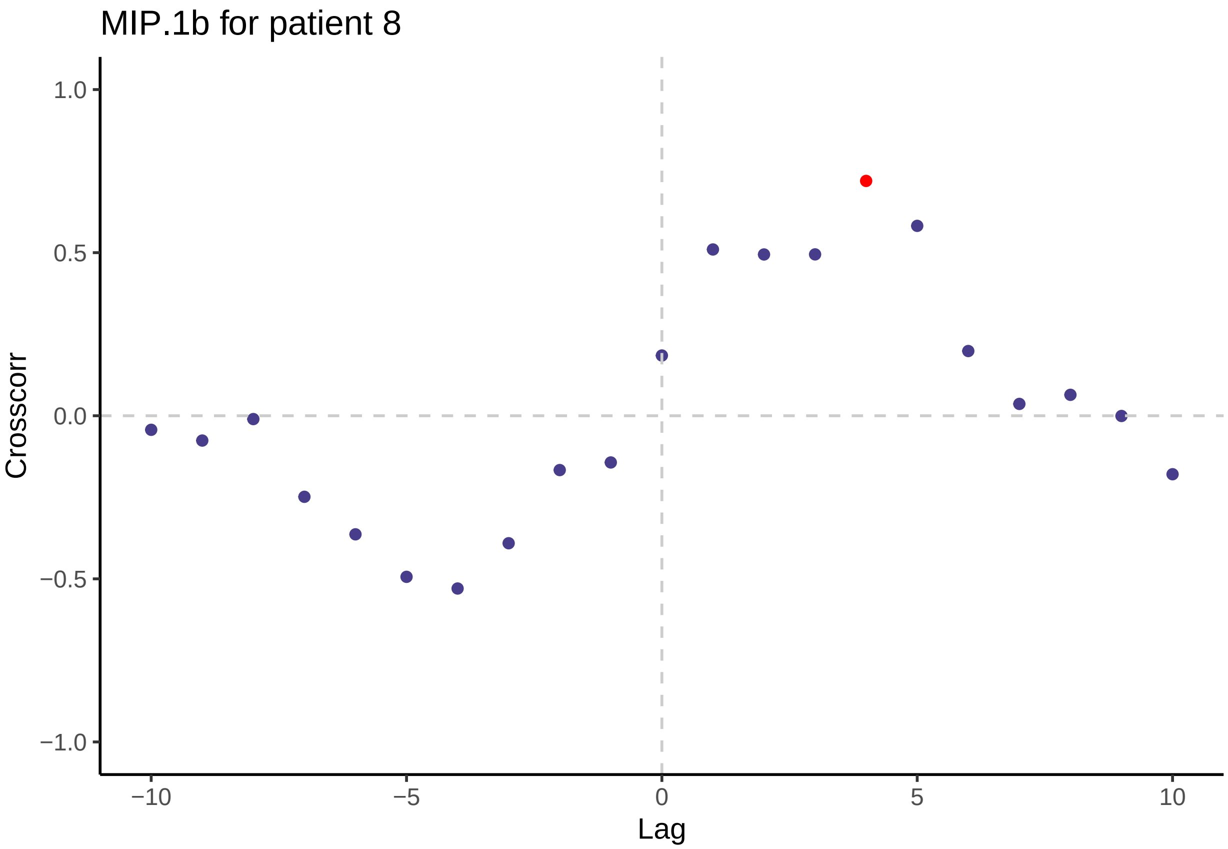


```
## [1] "MIP.1b for patient 8 - p-value: 0.704170697921509"
```

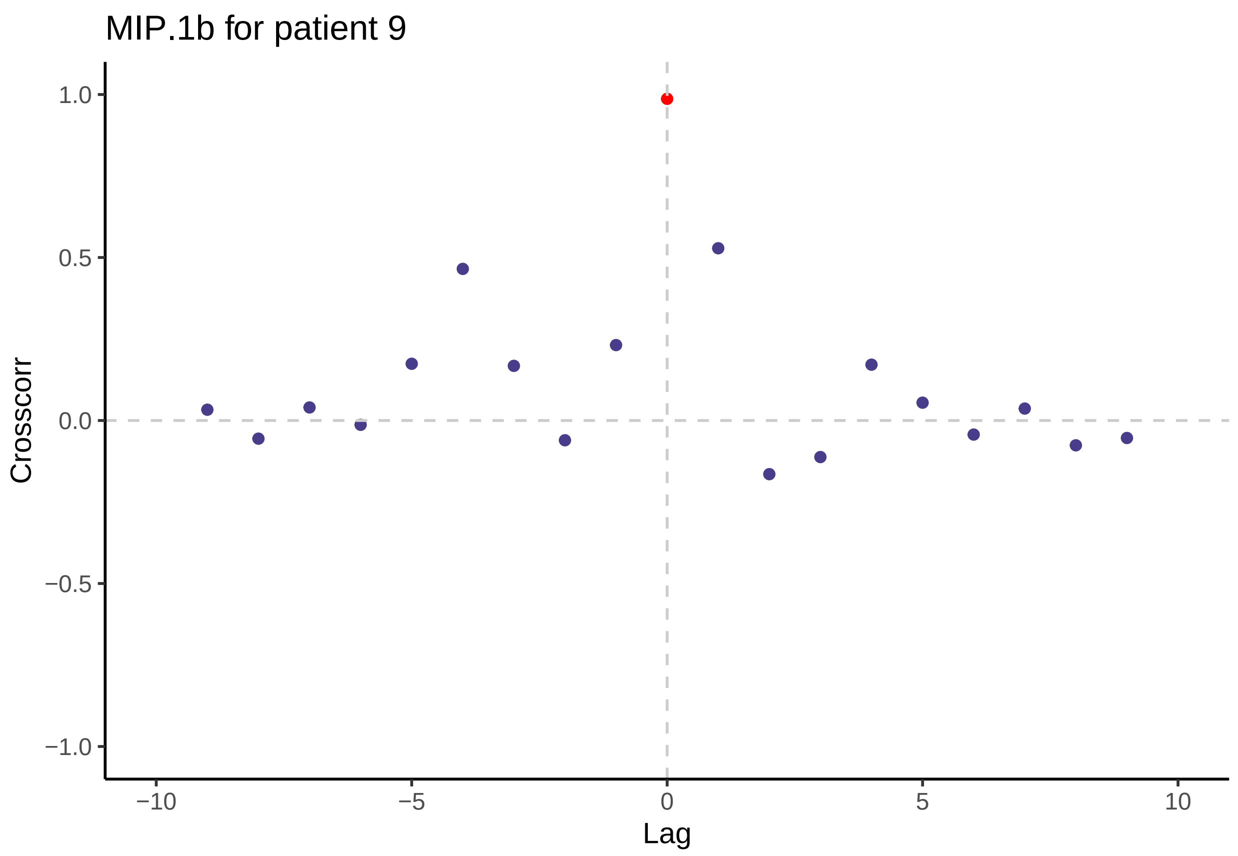


```
## [1] "MIP.1b for patient 9 - p-value: 0.0729377545967853"
## Warning: Removed 7 rows containing missing values (geom_point).
```

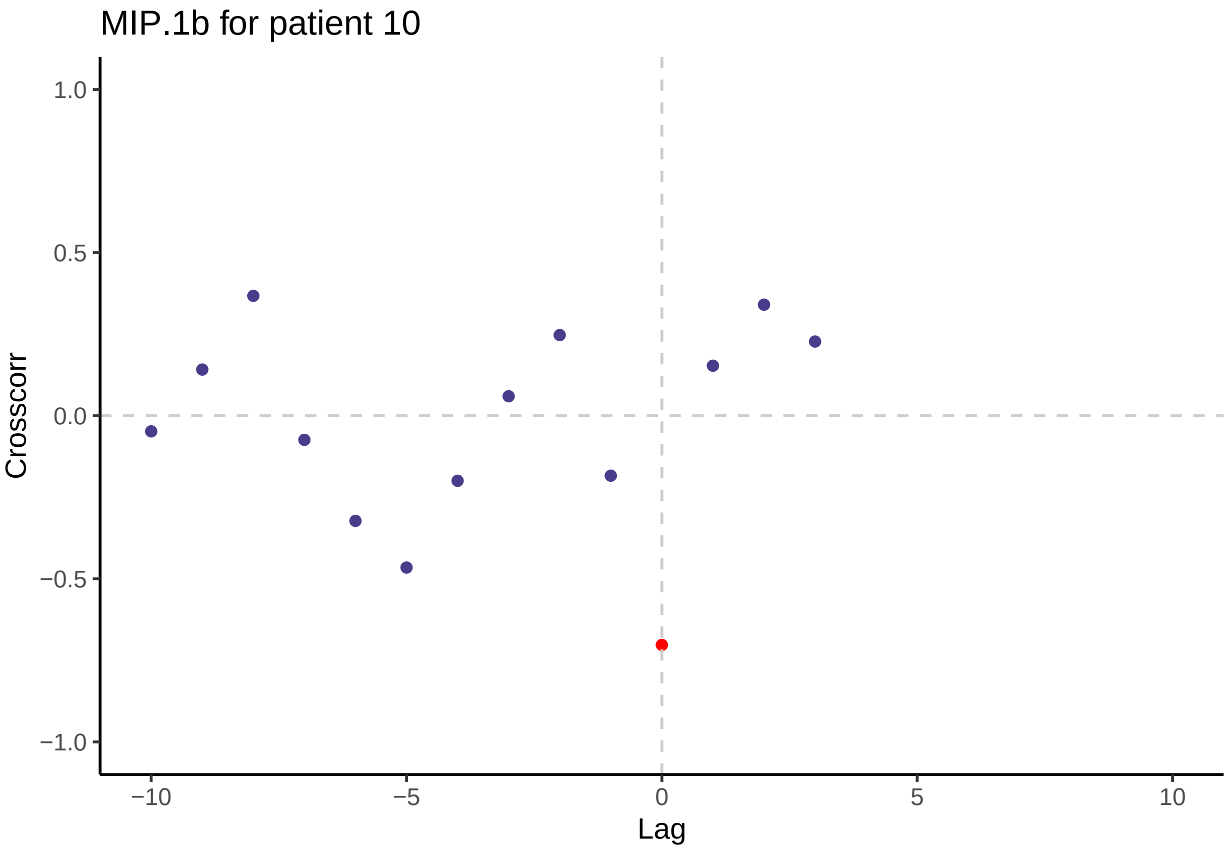


```
## [1] "MIP.1b for patient 10 - p-value: 0.704498508552991"
```

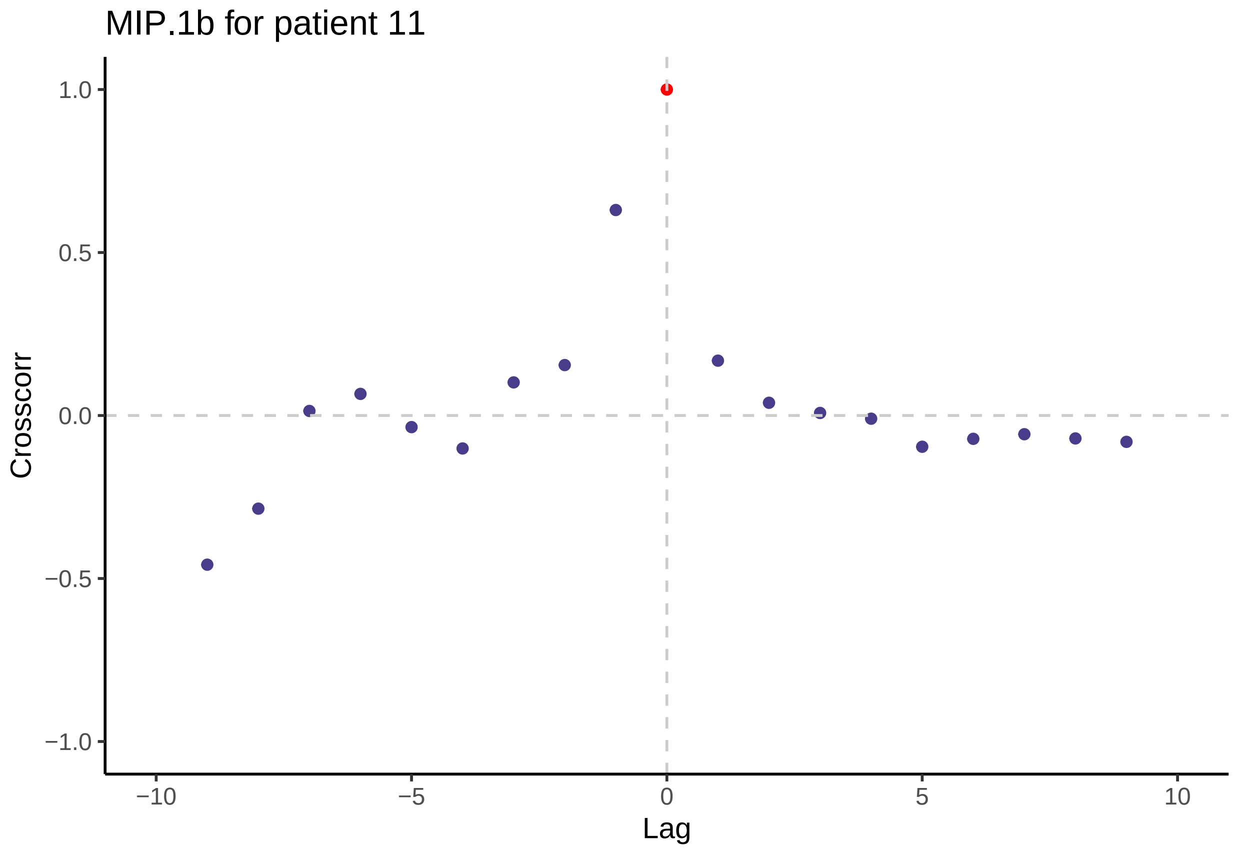


```
## [1] "MIP.1b for patient 11 - p-value: 0.509083683558237"
```

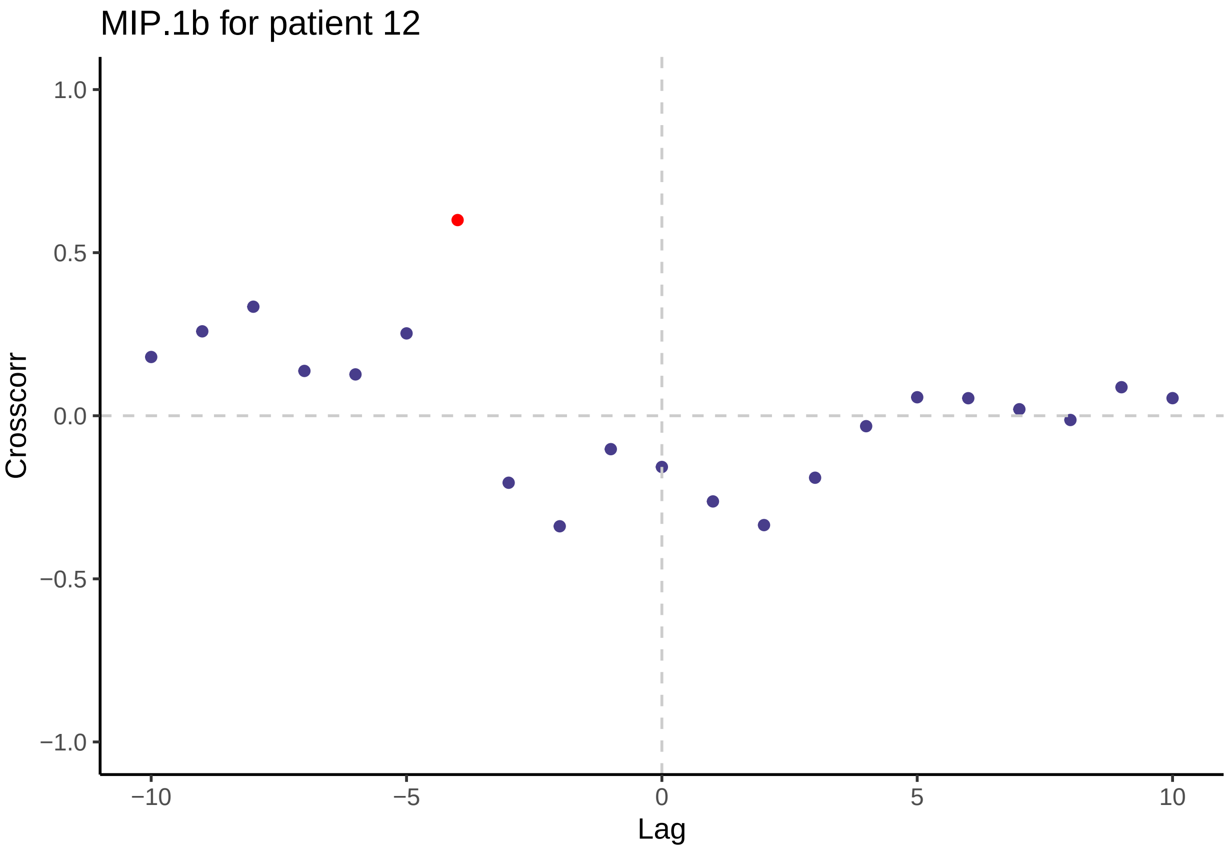


```
## [1] "MIP.1b for patient 12 - p-value: 0.627725304871118"
```

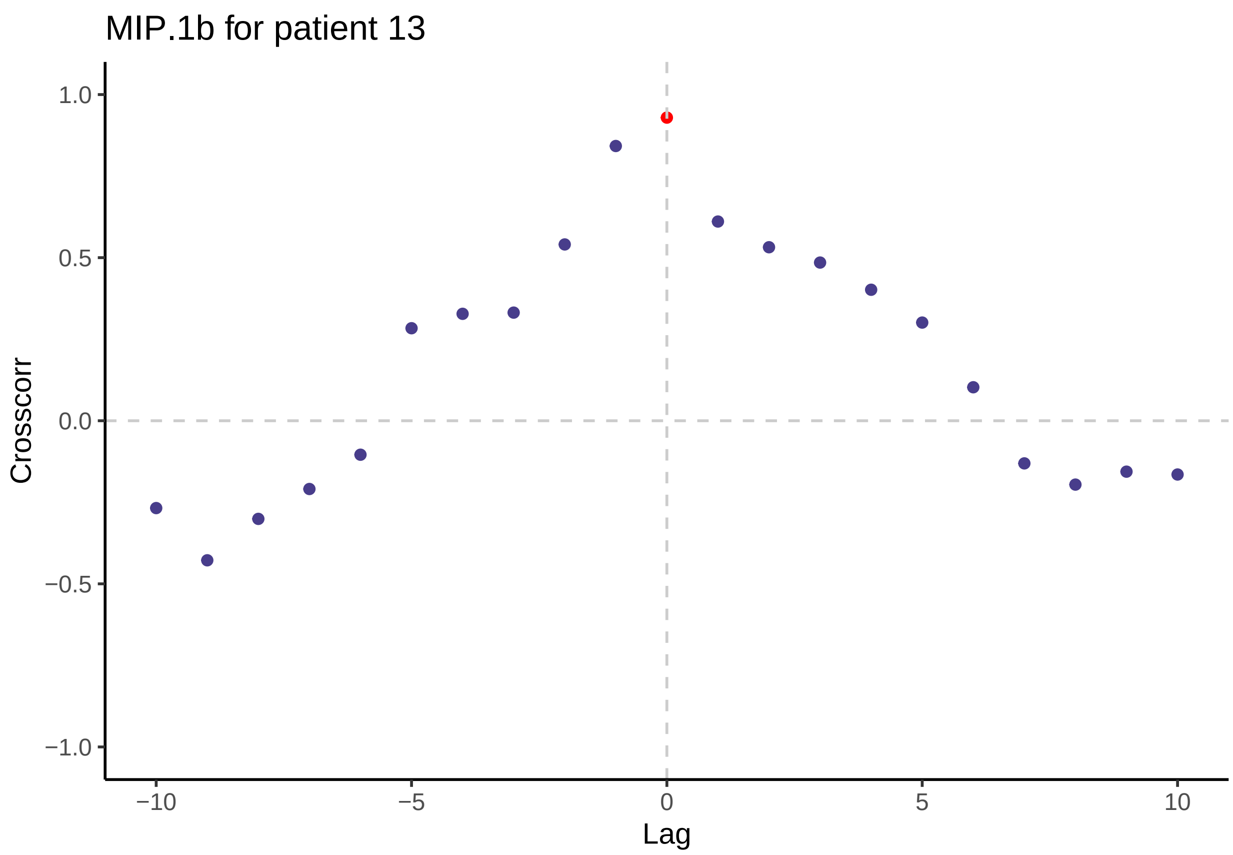


```
## [1] "MIP.1b for patient 13 - p-value: 0.0538728518822861"
```

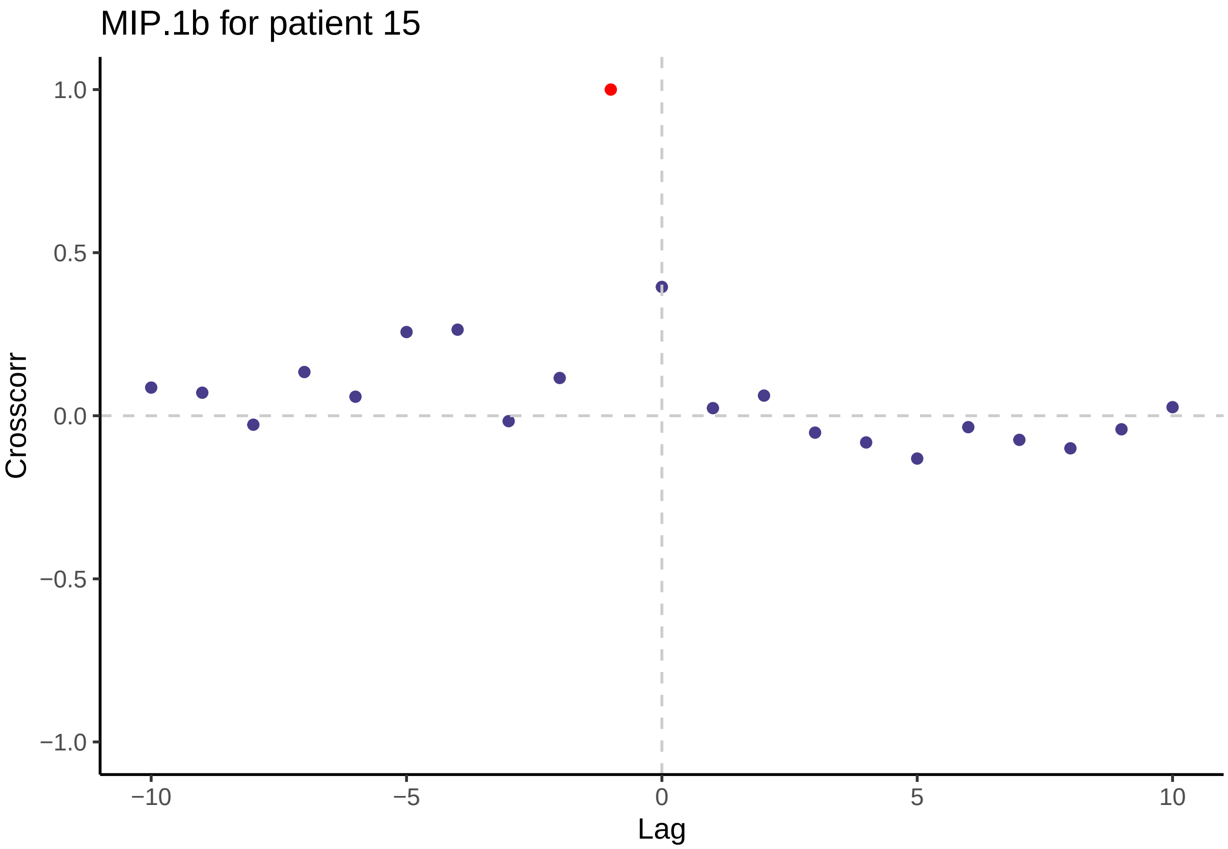


```
## [1] "MIP.1b for patient 15 - p-value: 0.102032222839539"
```

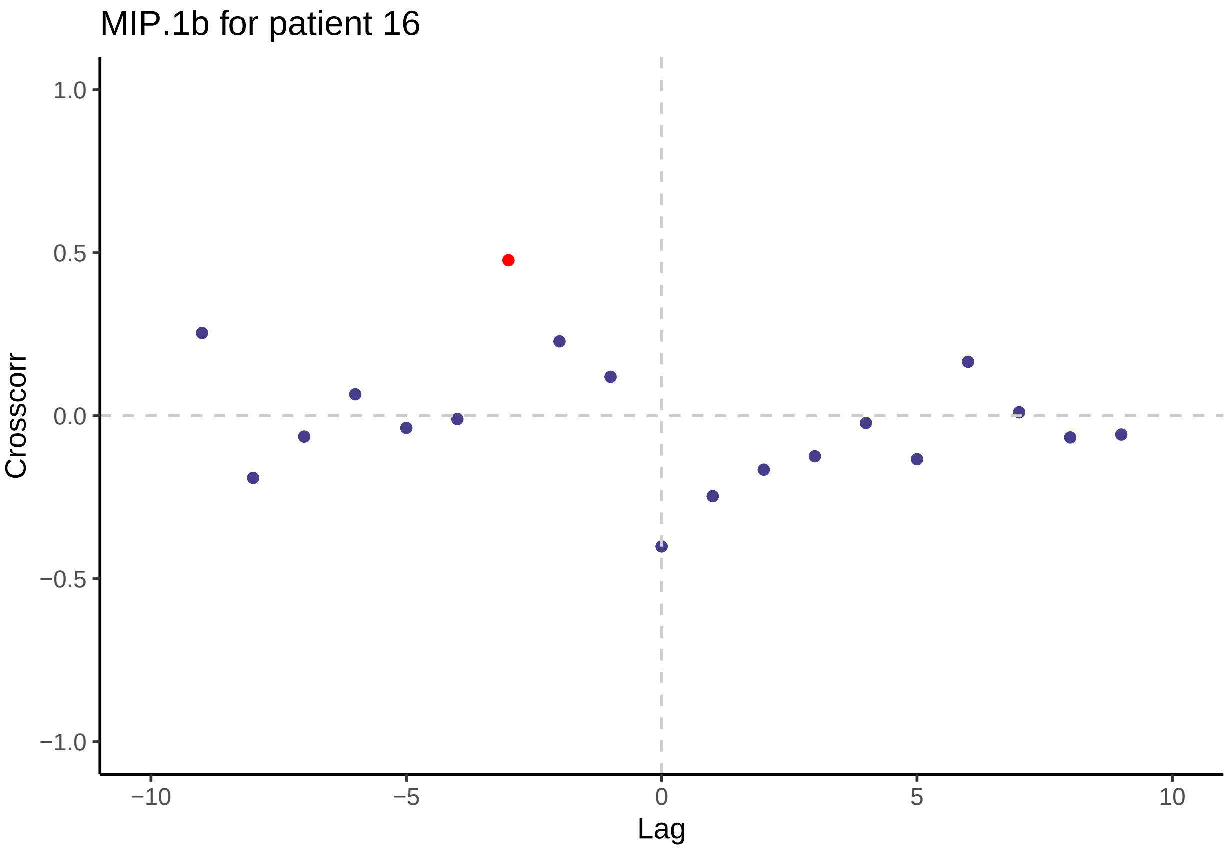


```
## [1] "MIP.1b for patient 16 - p-value: 0.824364673567531"
```

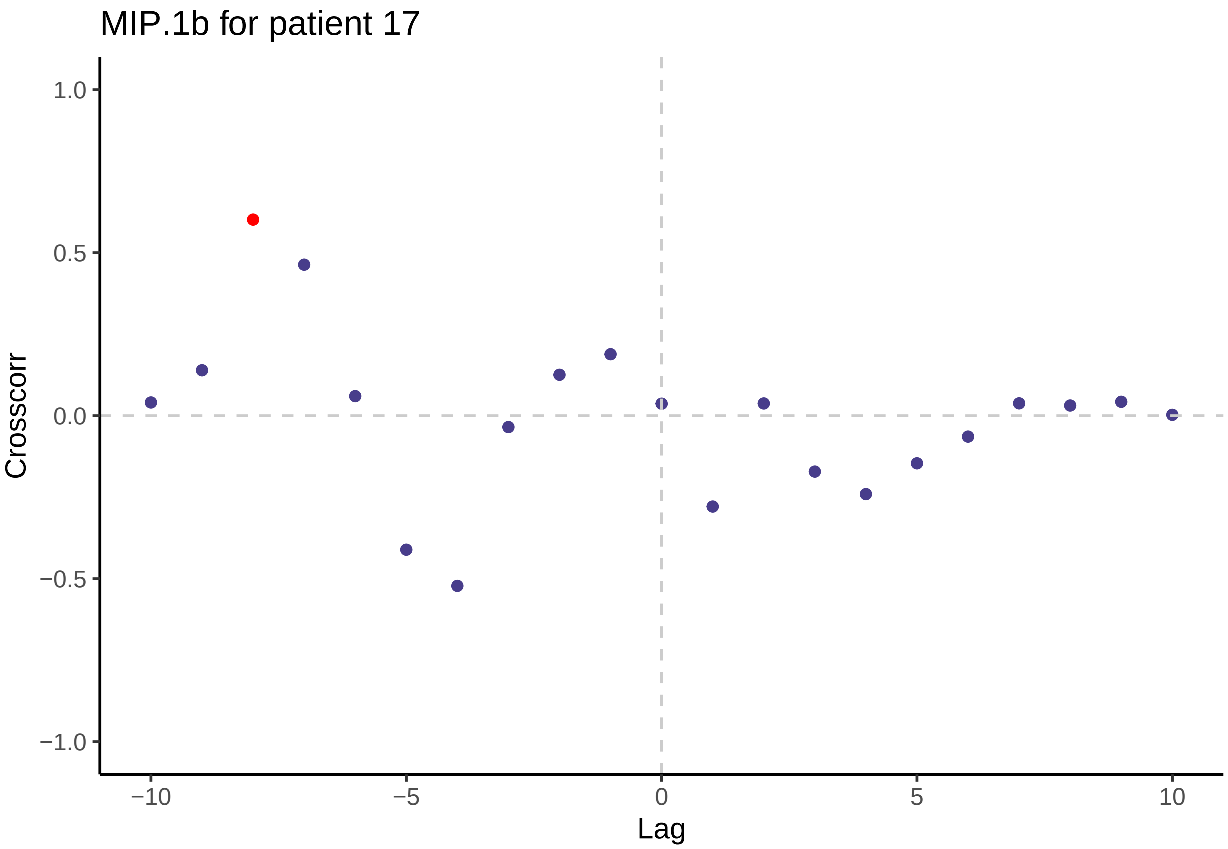


```
## [1] "MIP.1b for patient 17 - p-value: 0.961574493994563"
```

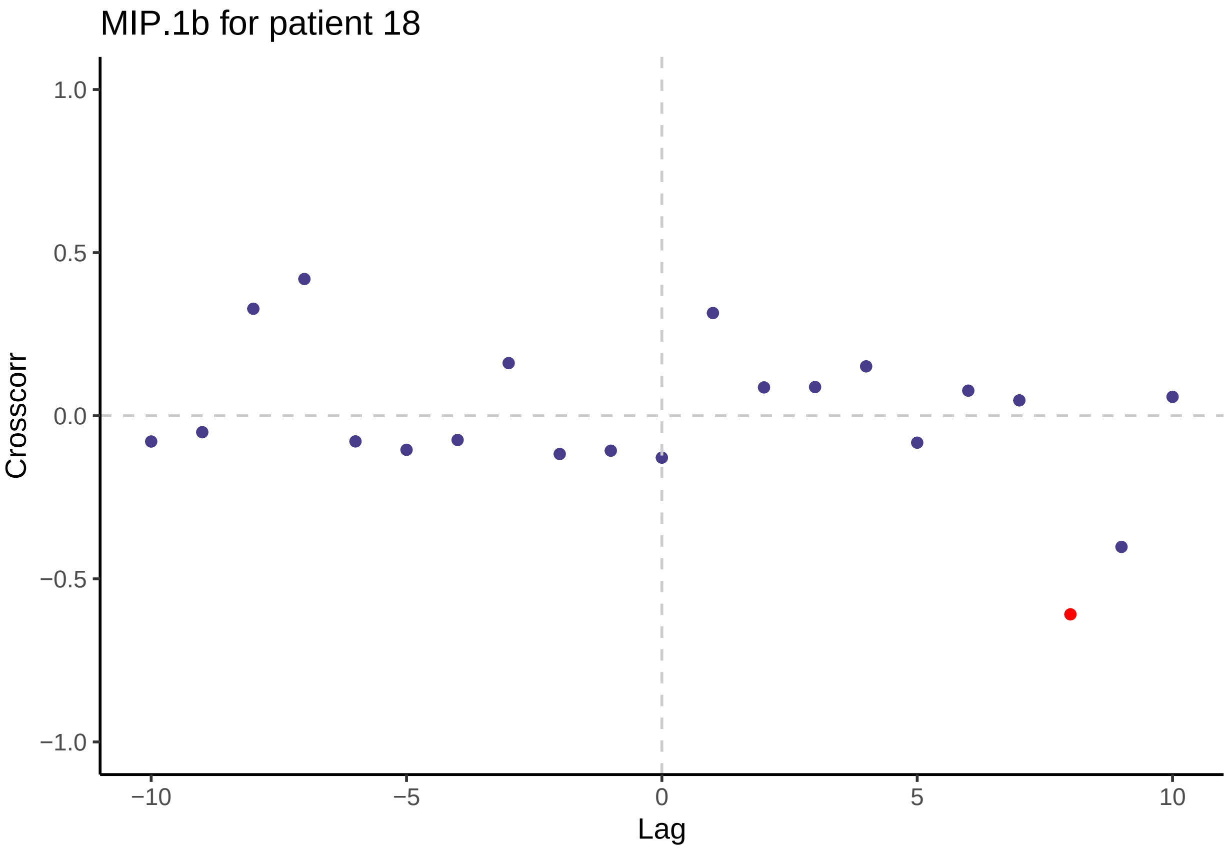


```
## [1] "MIP.1b for patient 18 - p-value: 0.924977169466141"
```

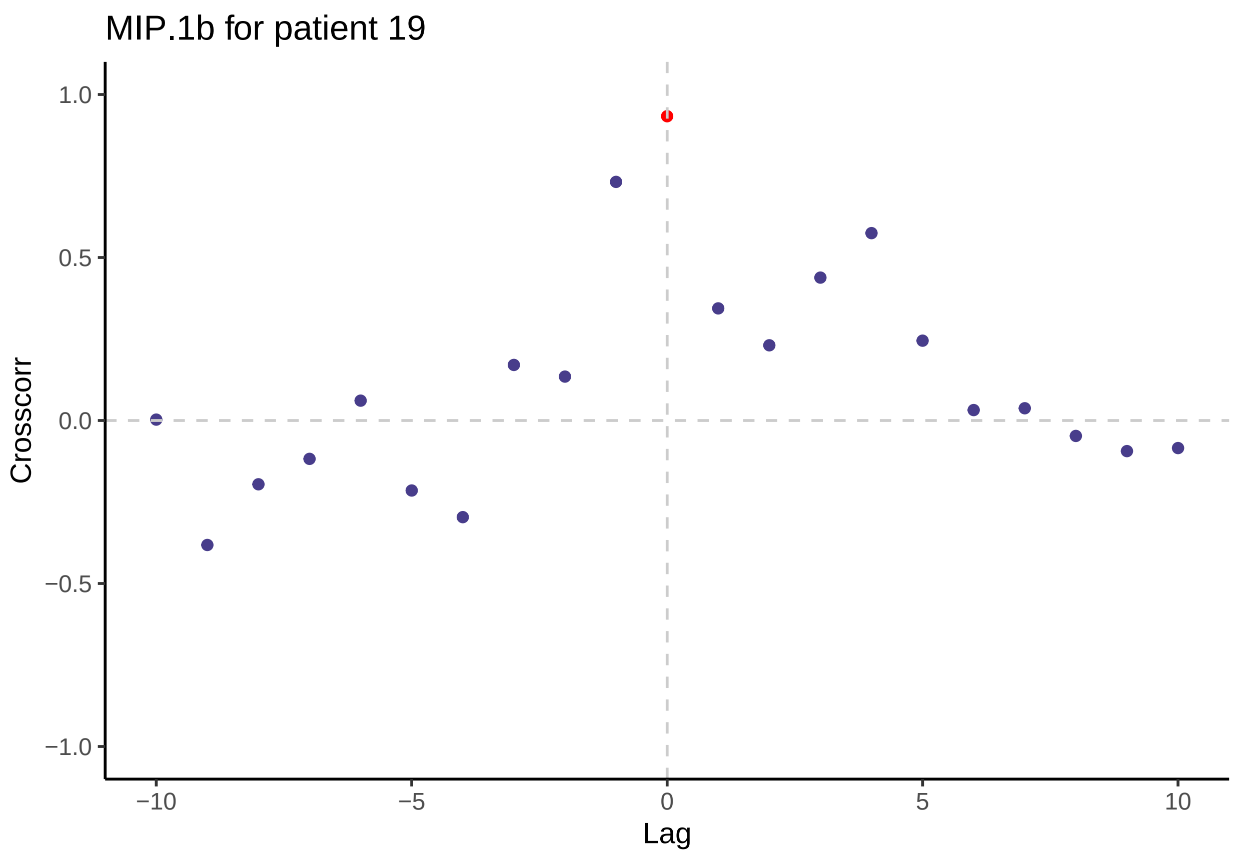


```
## [1] "MIP.1b for patient 19 - p-value: 0.120043369438718"
```

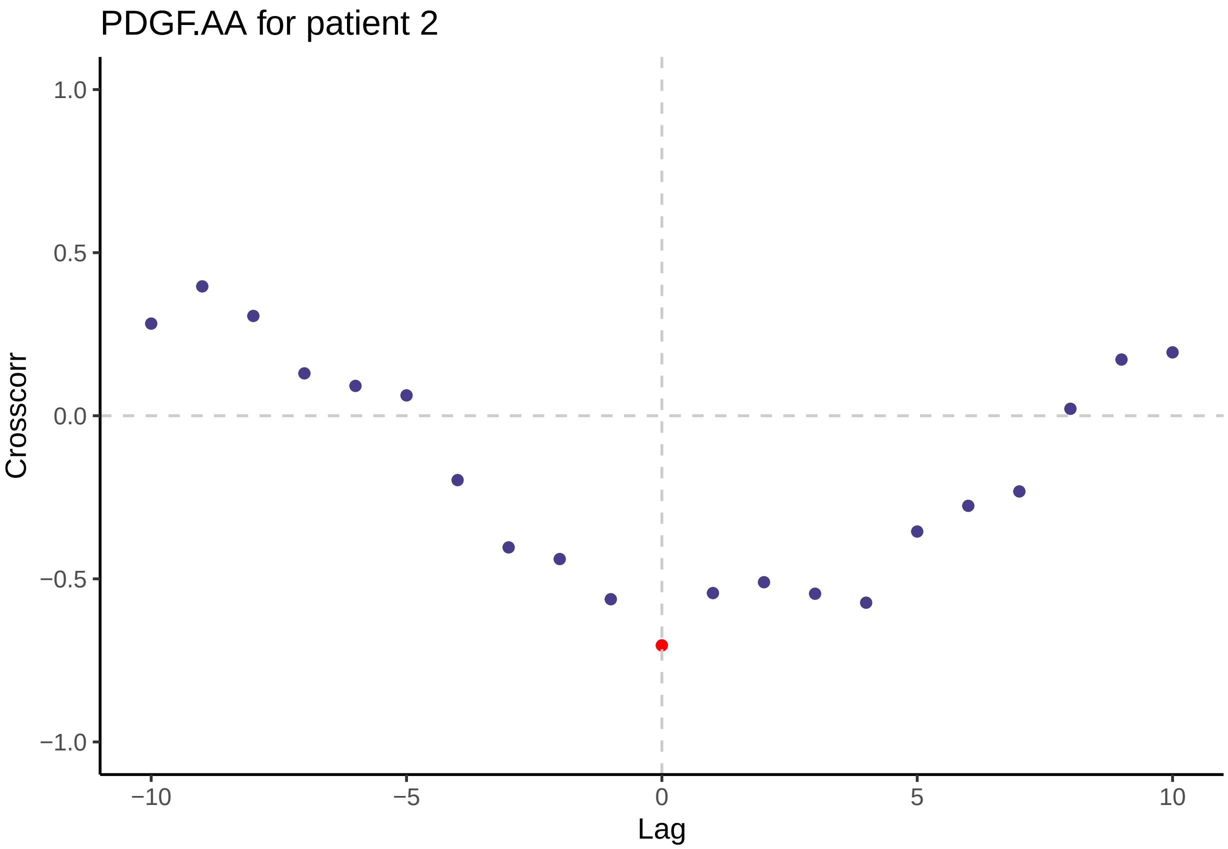


```
## [1] "PDGF.AA for patient 2 - p-value: 0.0317482267211318"
```

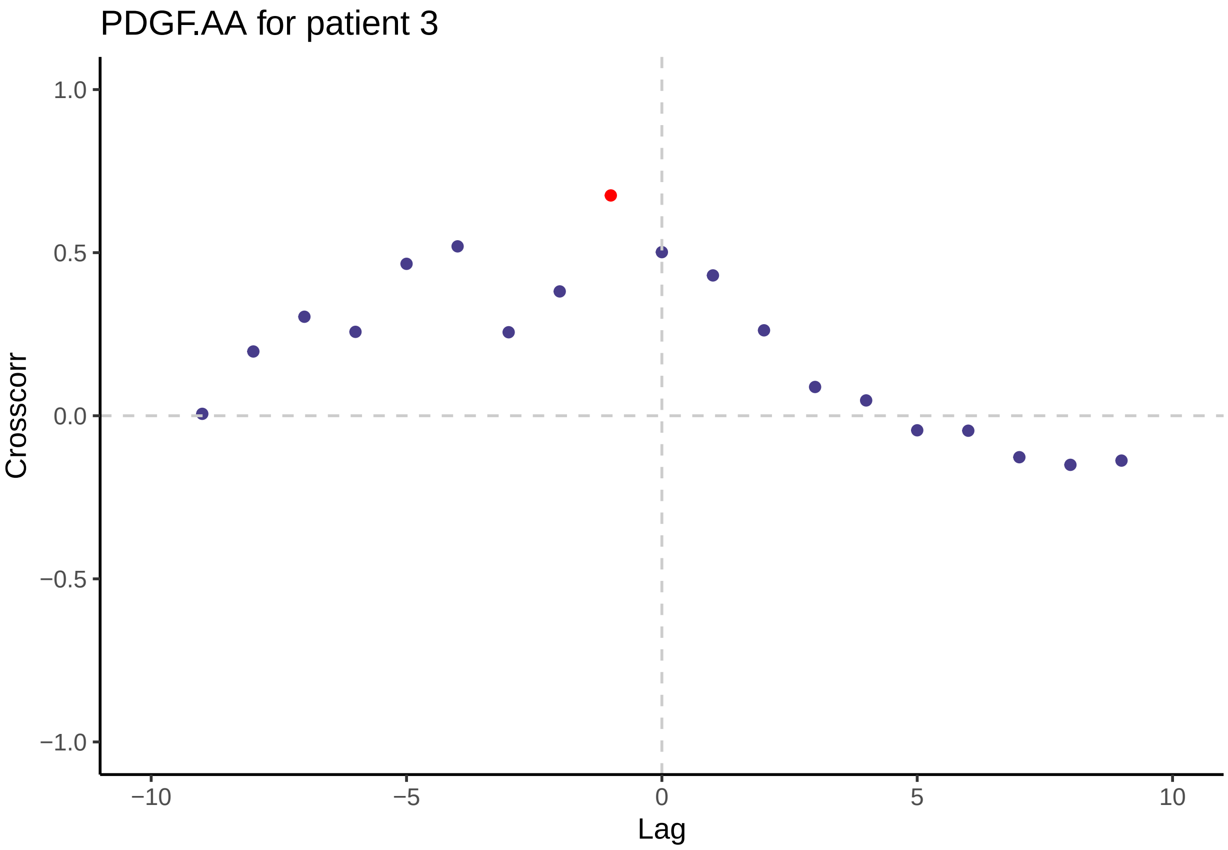


```
## [1] "PDGF.AA for patient 3 - p-value: 0.00227928360811027"
```

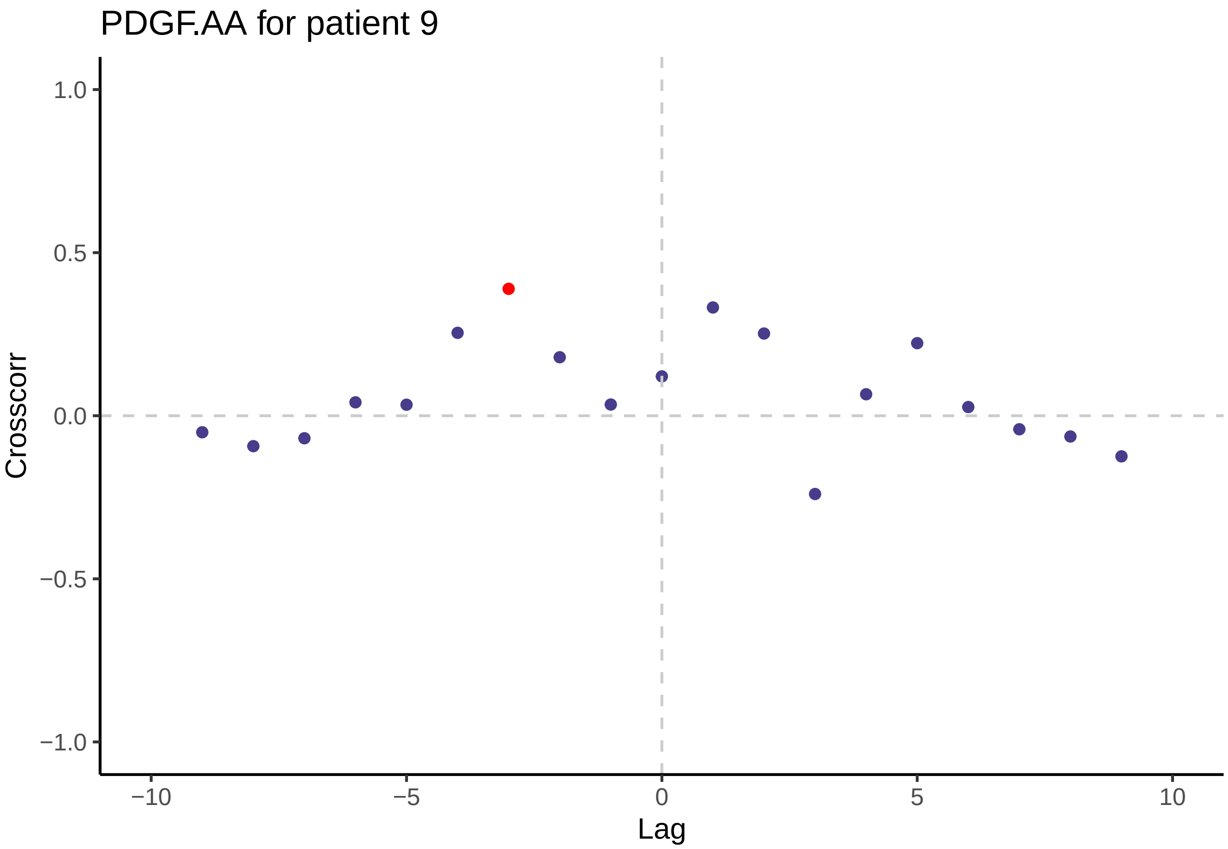


```
## [1] "PDGF.AA for patient 9 - p-value: 0.0990896212931473"
```

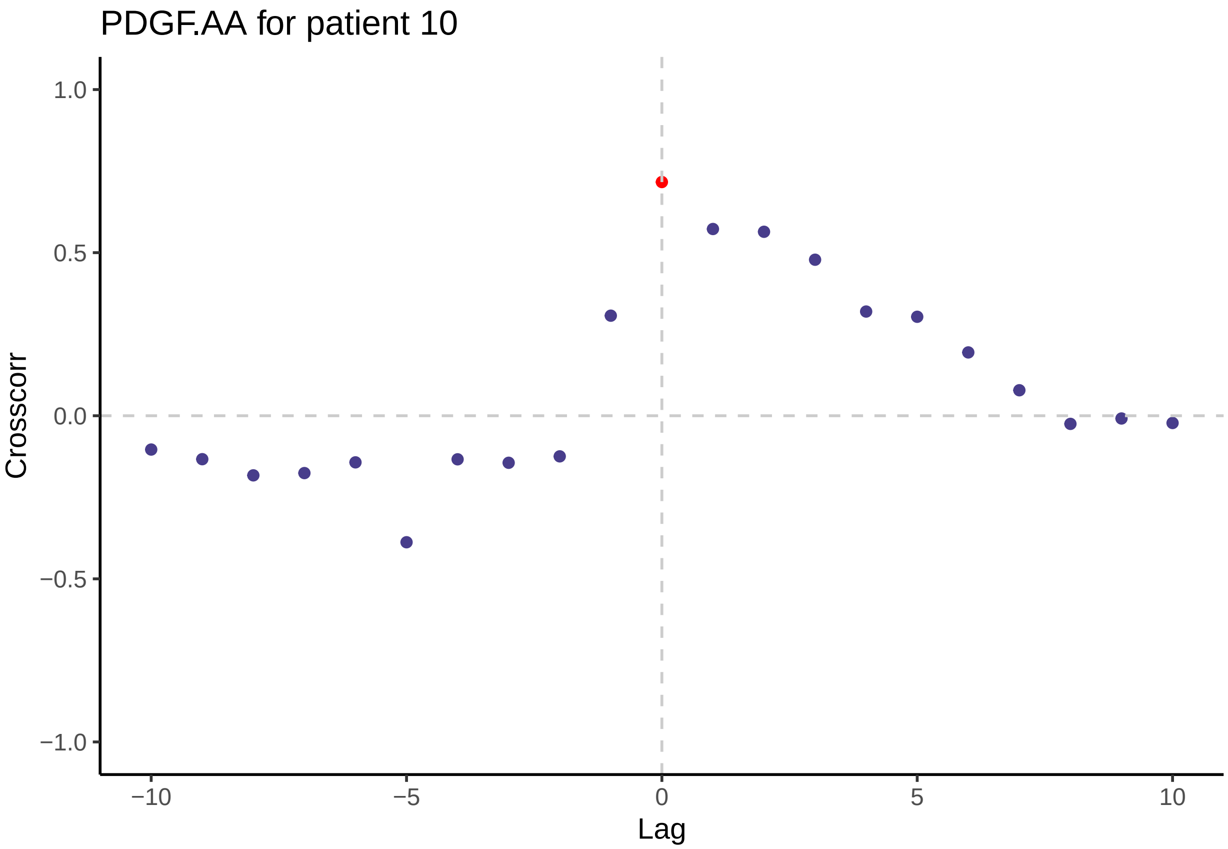


```
## [1] "PDGF.AA for patient 10 - p-value: 0.17798678022646"
```

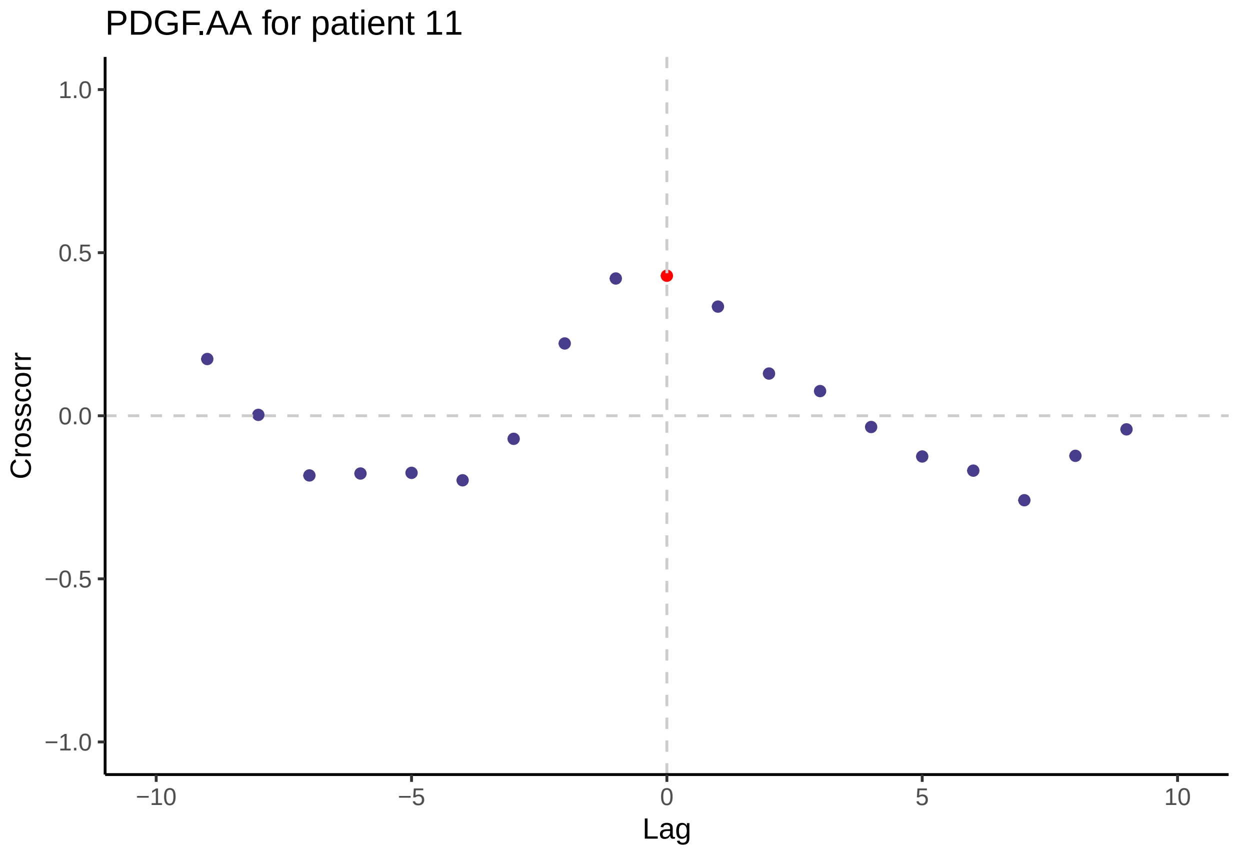


```
## [1] "PDGF.AA for patient 11 - p-value: 0.806999619906849"
```

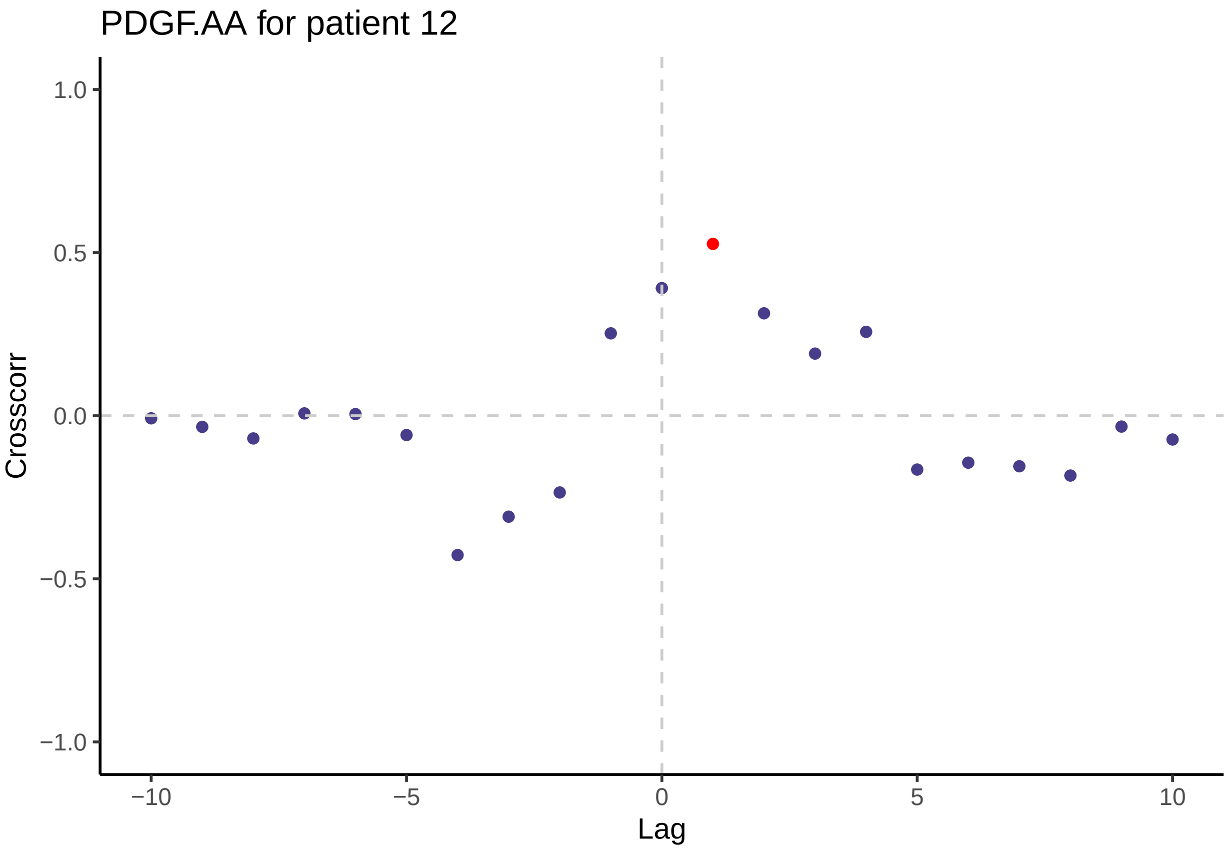


```
## [1] "PDGF.AA for patient 12 - p-value: 0.964487368662593"
```

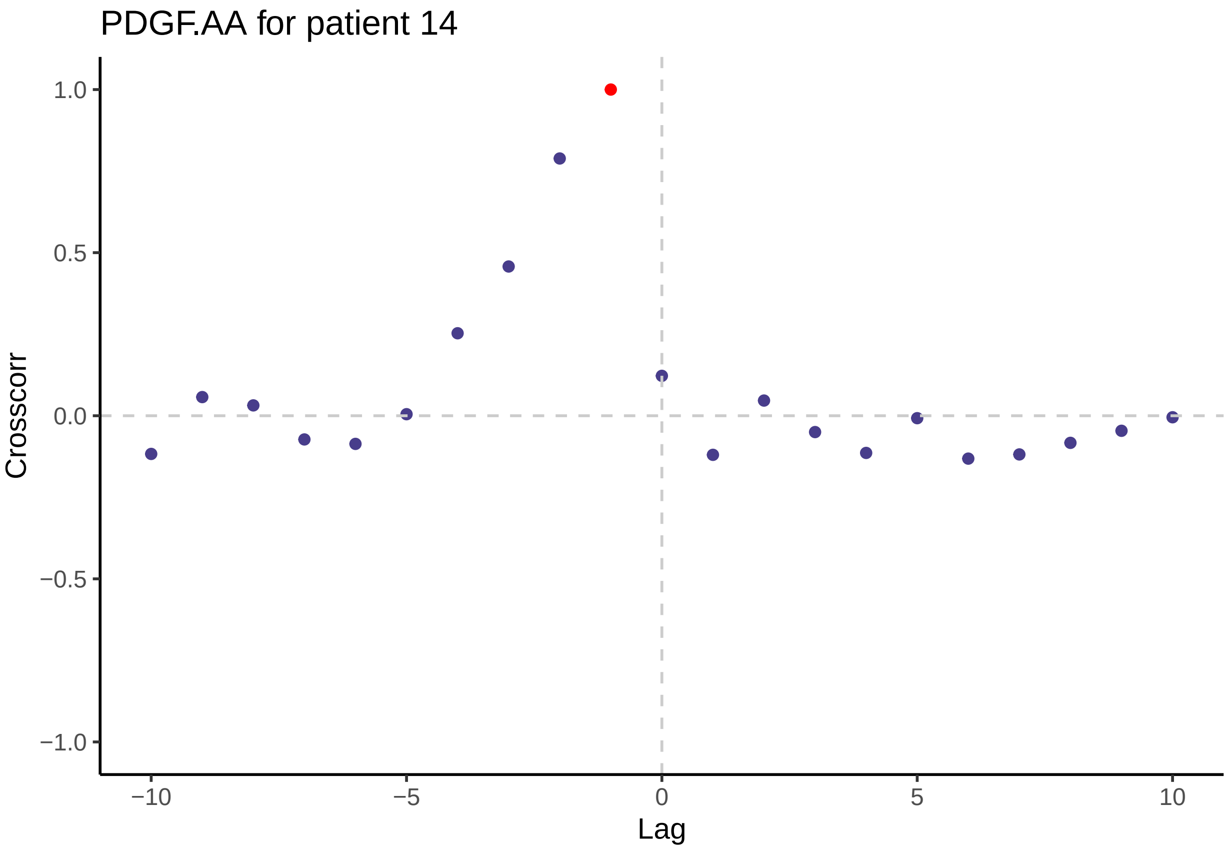


```
## [1] "PDGF.AA for patient 14 - p-value: 0.209423259110519"
```

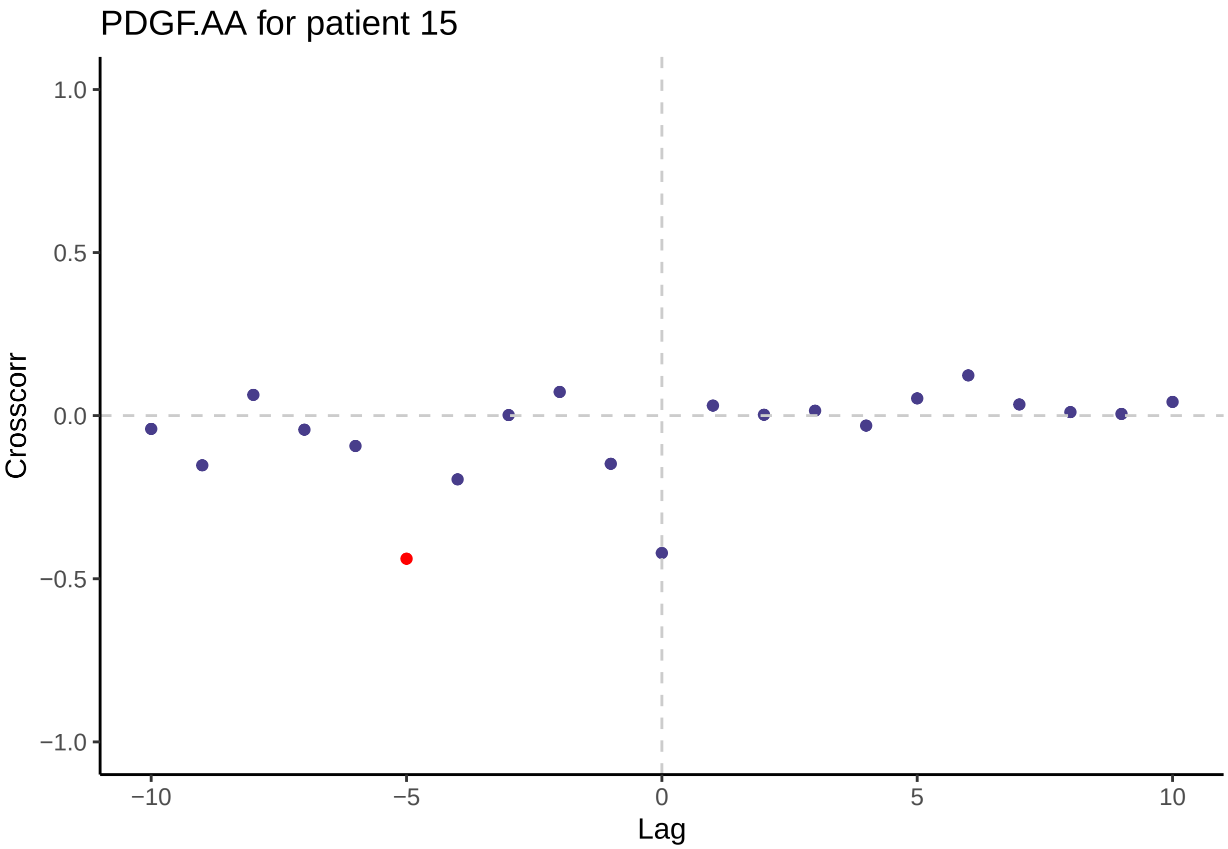


```
## [1] "PDGF.AA for patient 15 - p-value: 0.121184776497418"
```

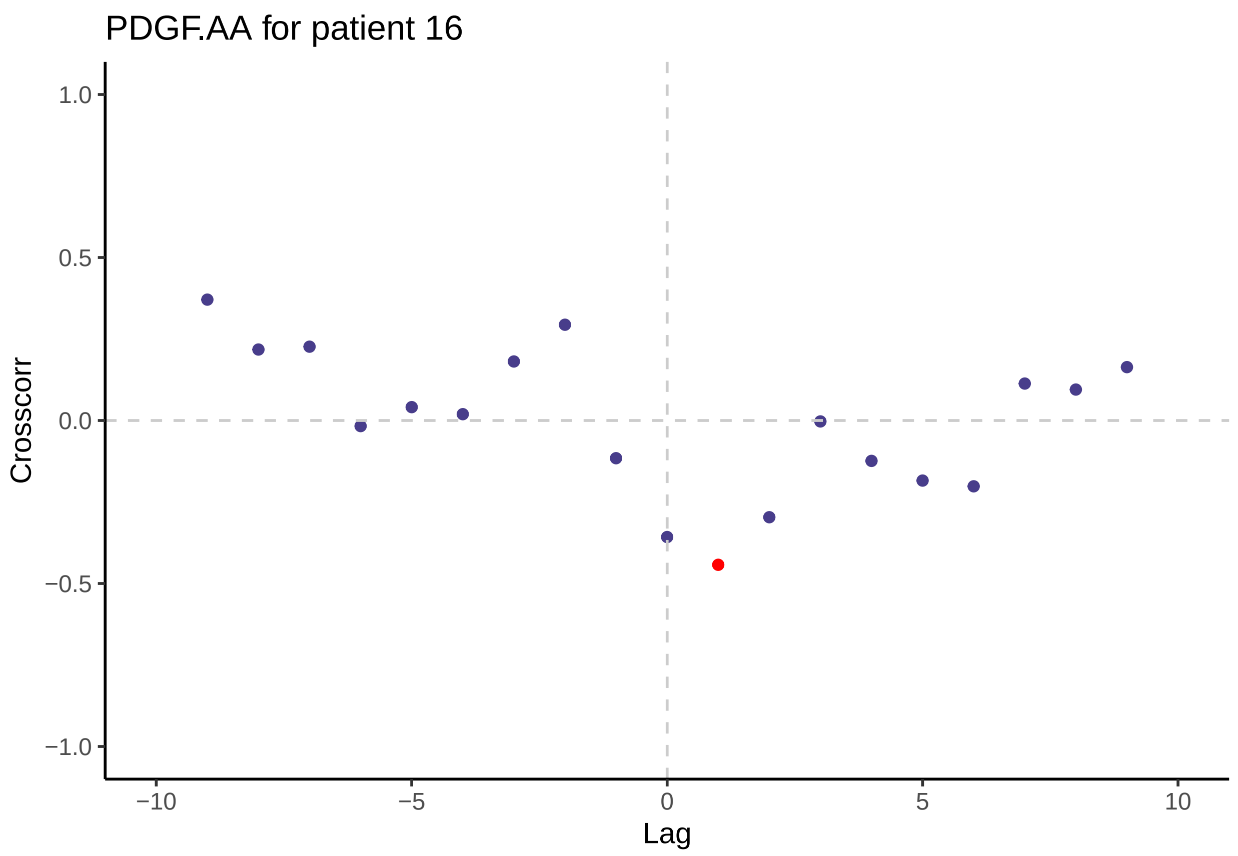


```
## [1] "PDGF.AA for patient 16 - p-value: 0.98418436255385"
```

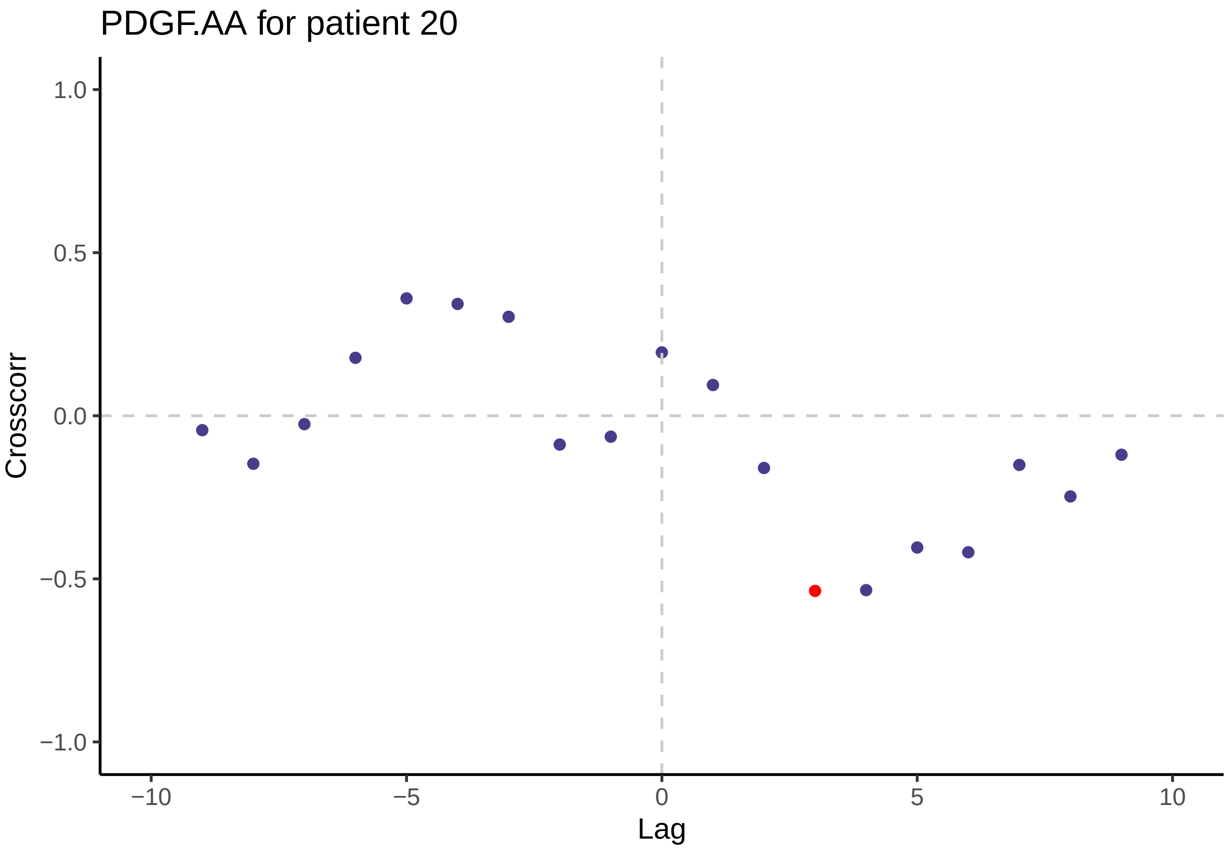


```
## [1] "PDGF.AA for patient 20 - p-value: 0.238531102274643"
```

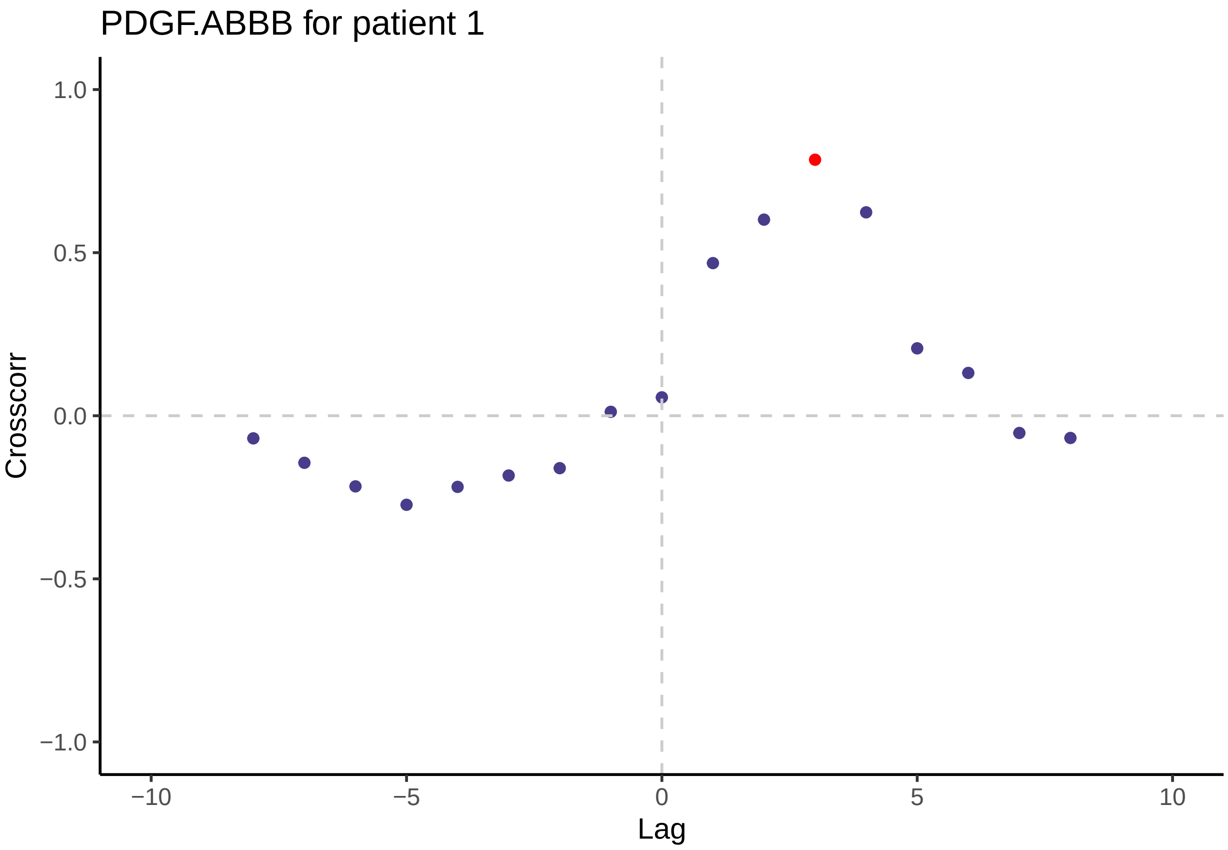


```
## [1] "PDGF.ABBB for patient 1 - p-value: 0.291782899008179"
```

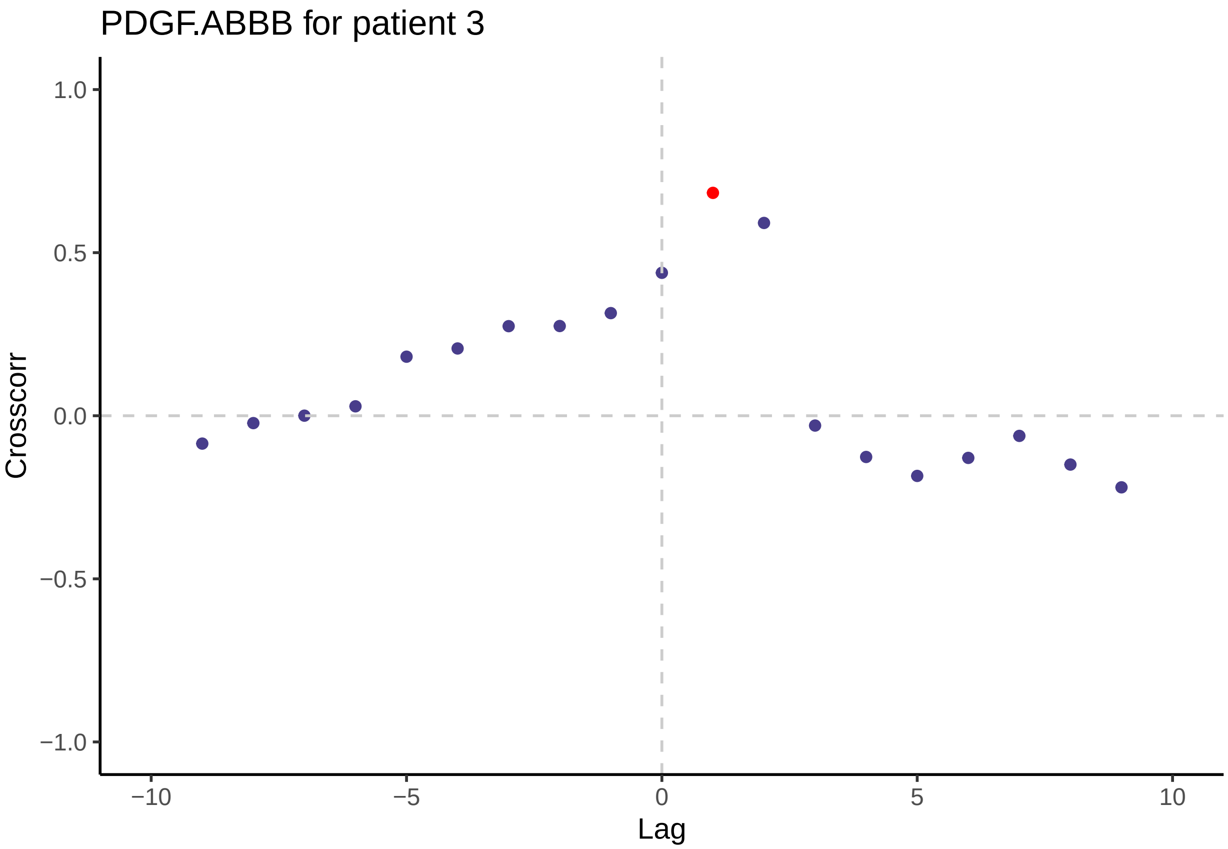


```
## [1] "PDGF.ABBB for patient 3 - p-value: 0.104535854580247"
```

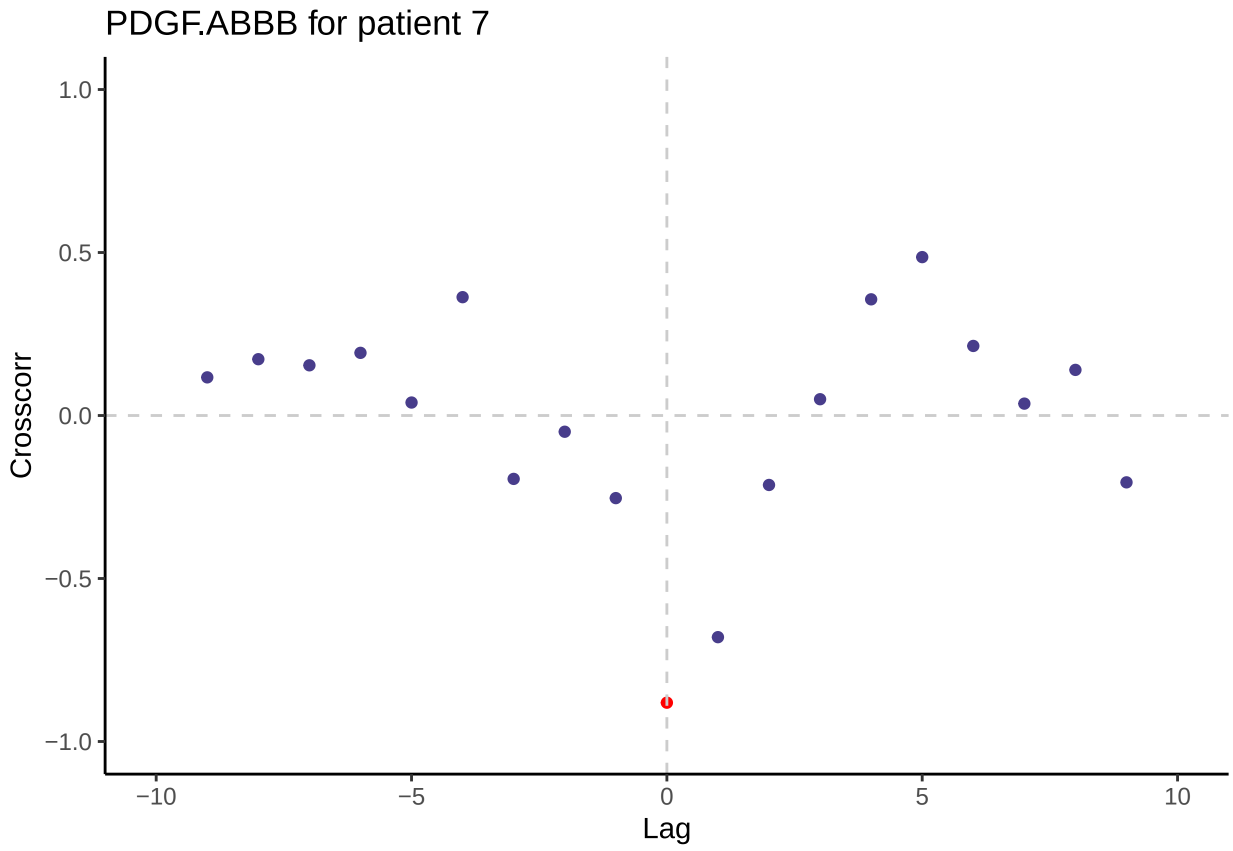


```
## [1] "PDGF.ABBB for patient 7 - p-value: 0.917751484696704"
```

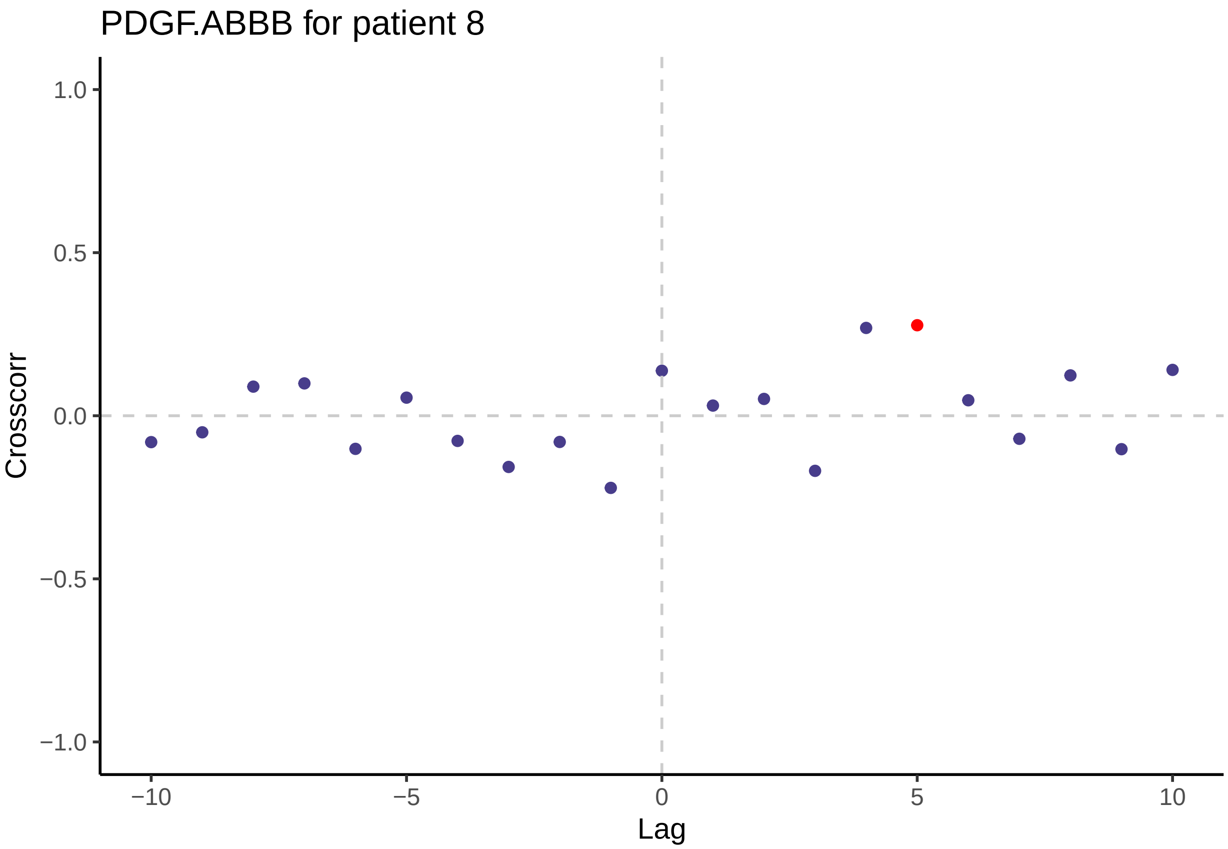


```
## [1] "PDGF.ABBB for patient 8 - p-value: 0.73876752939143"
```

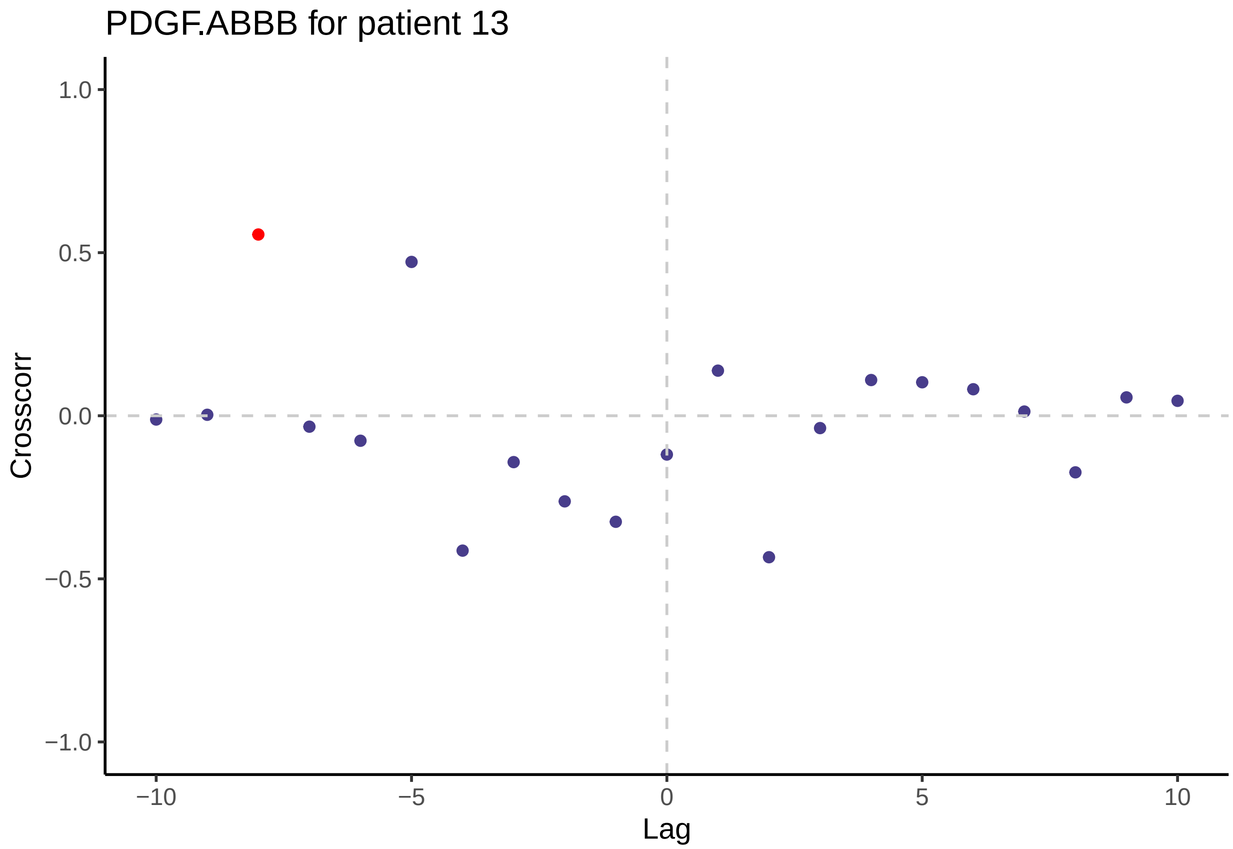


```
## [1] "PDGF.ABBB for patient 13 - p-value: 0.689456010467322"
```

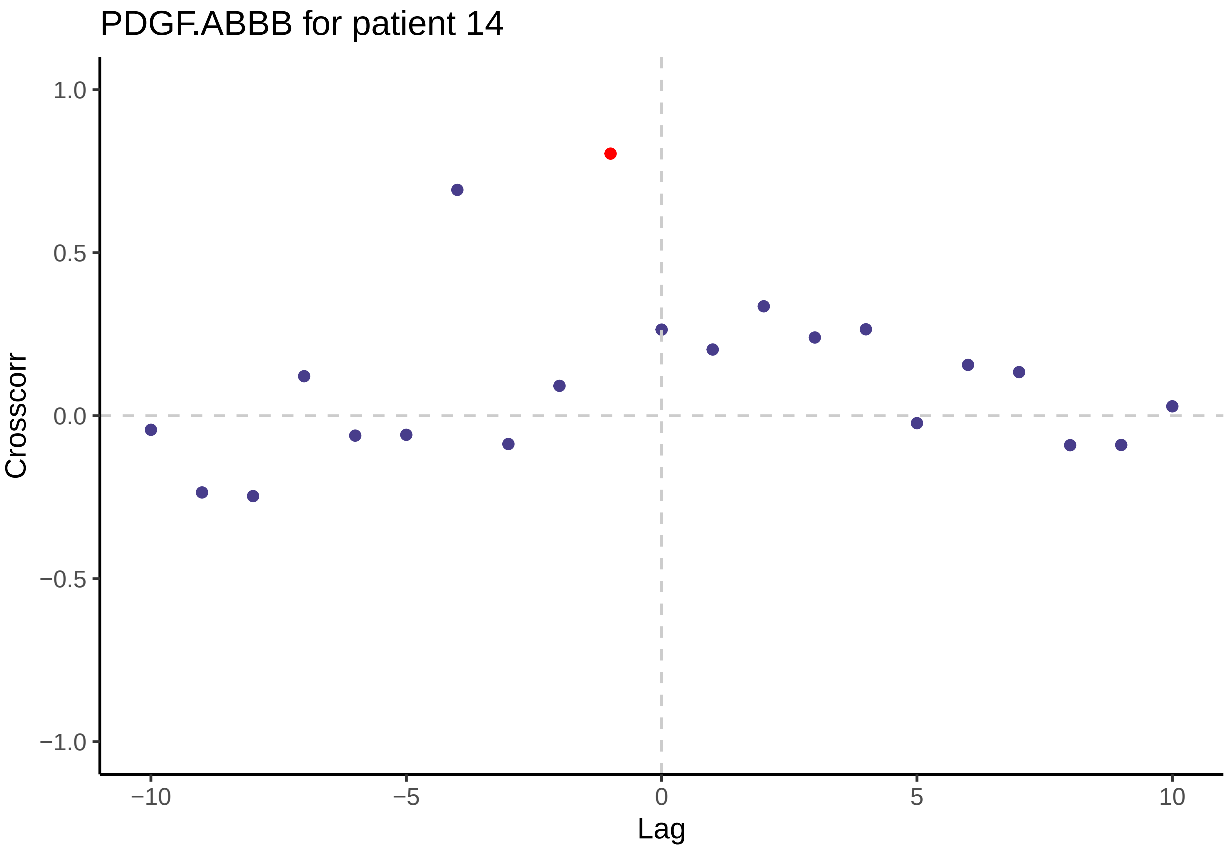


```
## [1] "PDGF.ABBB for patient 14 - p-value: 0.0628862474403086"
```

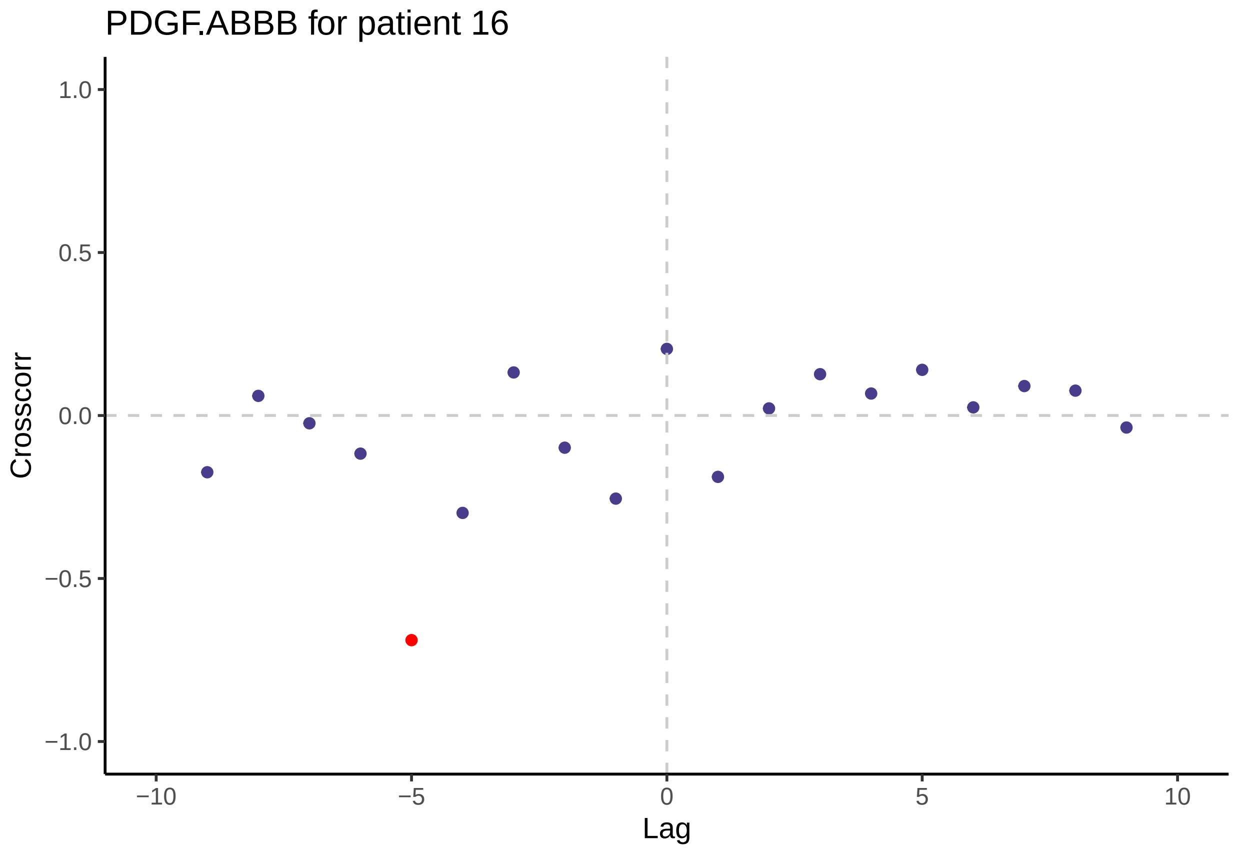


```
## [1] "PDGF.ABBB for patient 16 - p-value: 0.319157246133665"
```

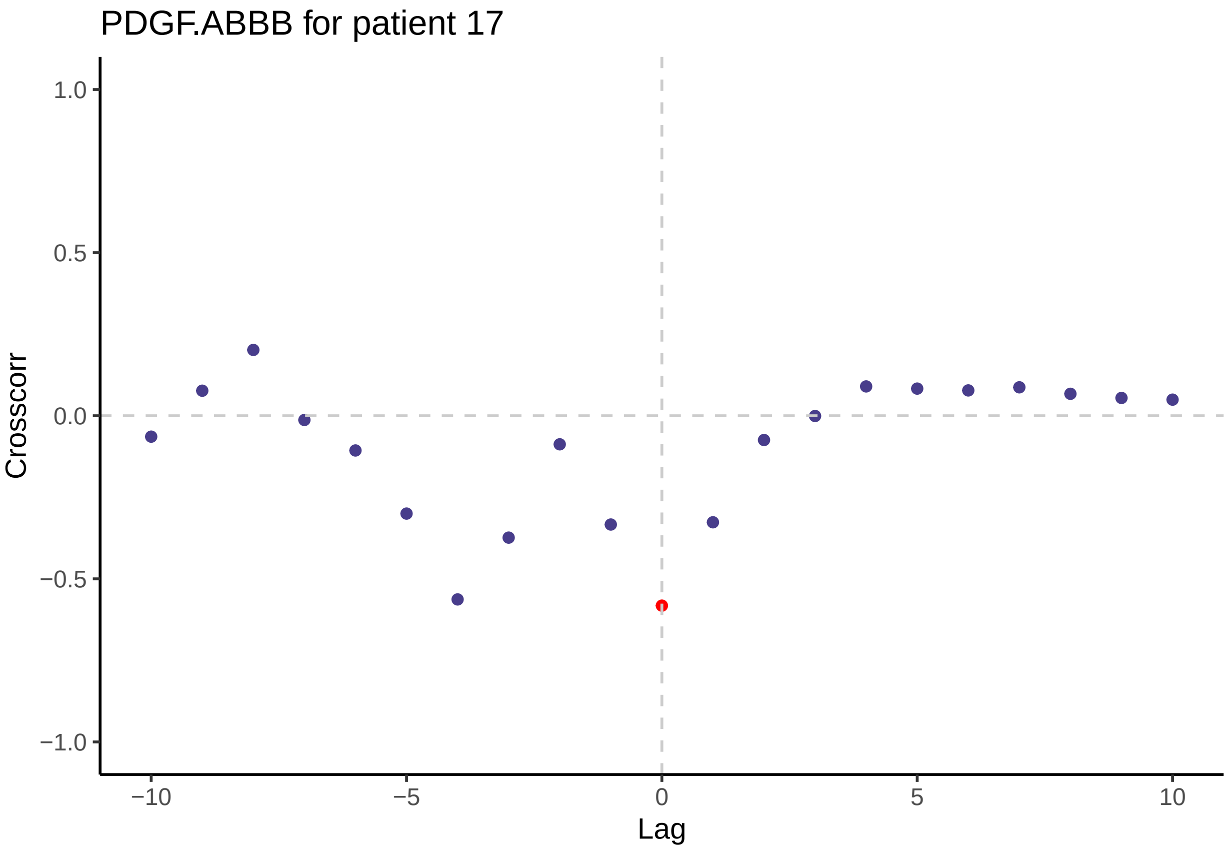


```
## [1] "PDGF.ABBB for patient 17 - p-value: 0.0629933576923409"
```

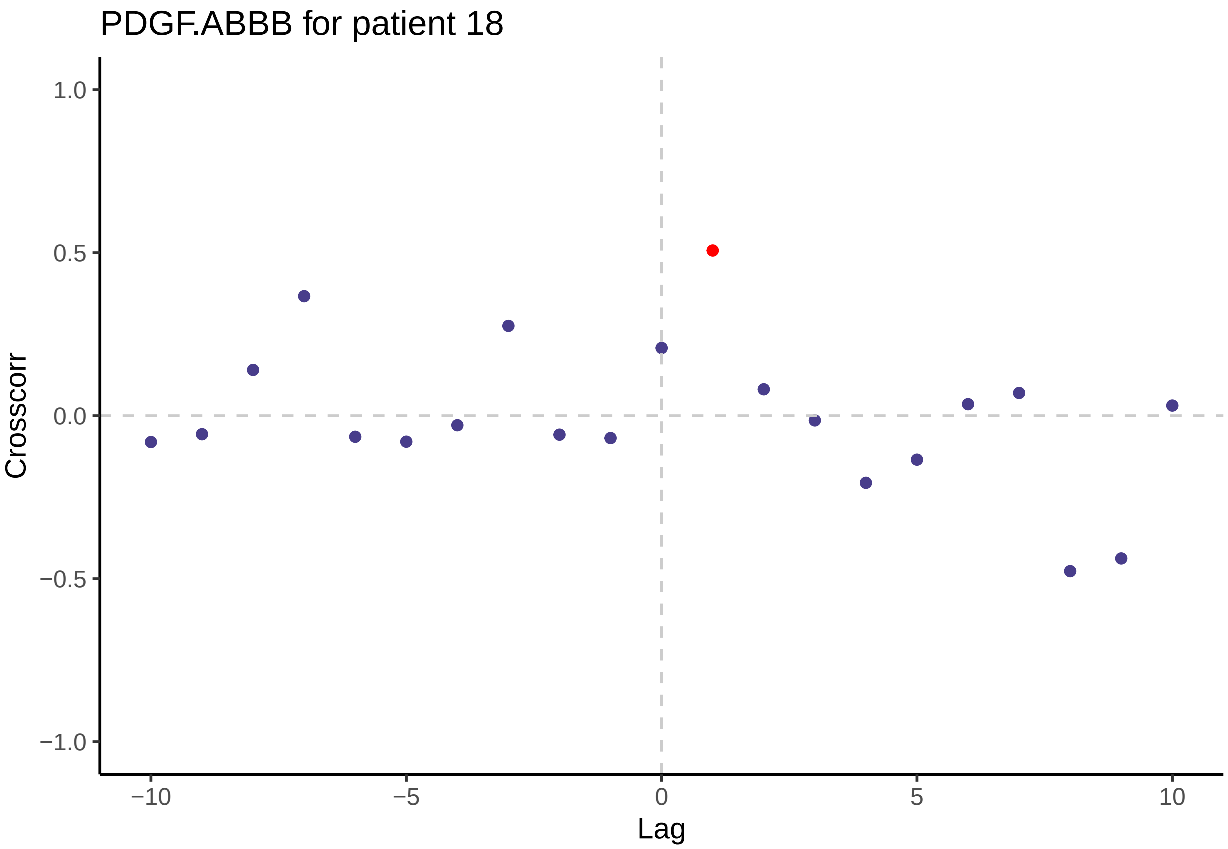


```
## [1] "PDGF.ABBB for patient 18 - p-value: 0.992229672783624"
```

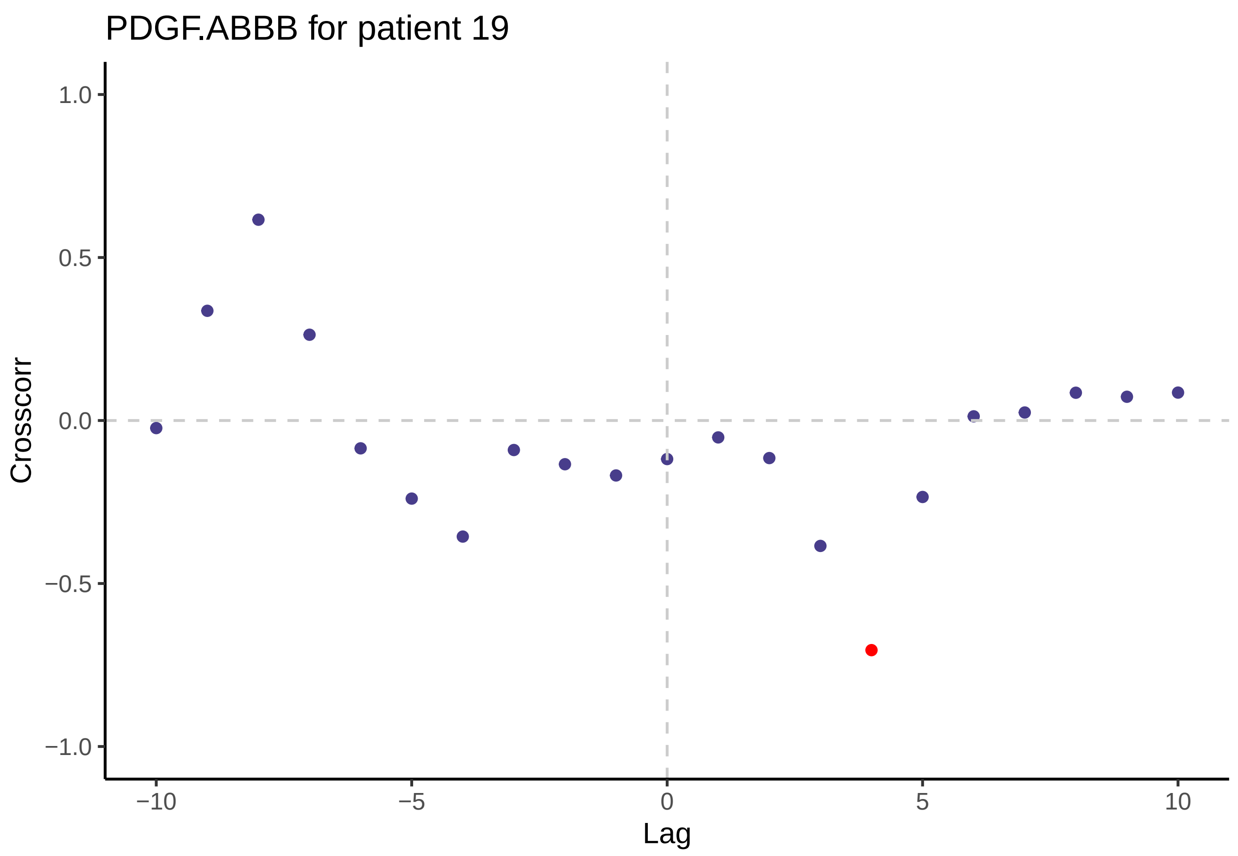


```
## [1] "PDGF.ABBB for patient 19 - p-value: 0.344871800552351"
```

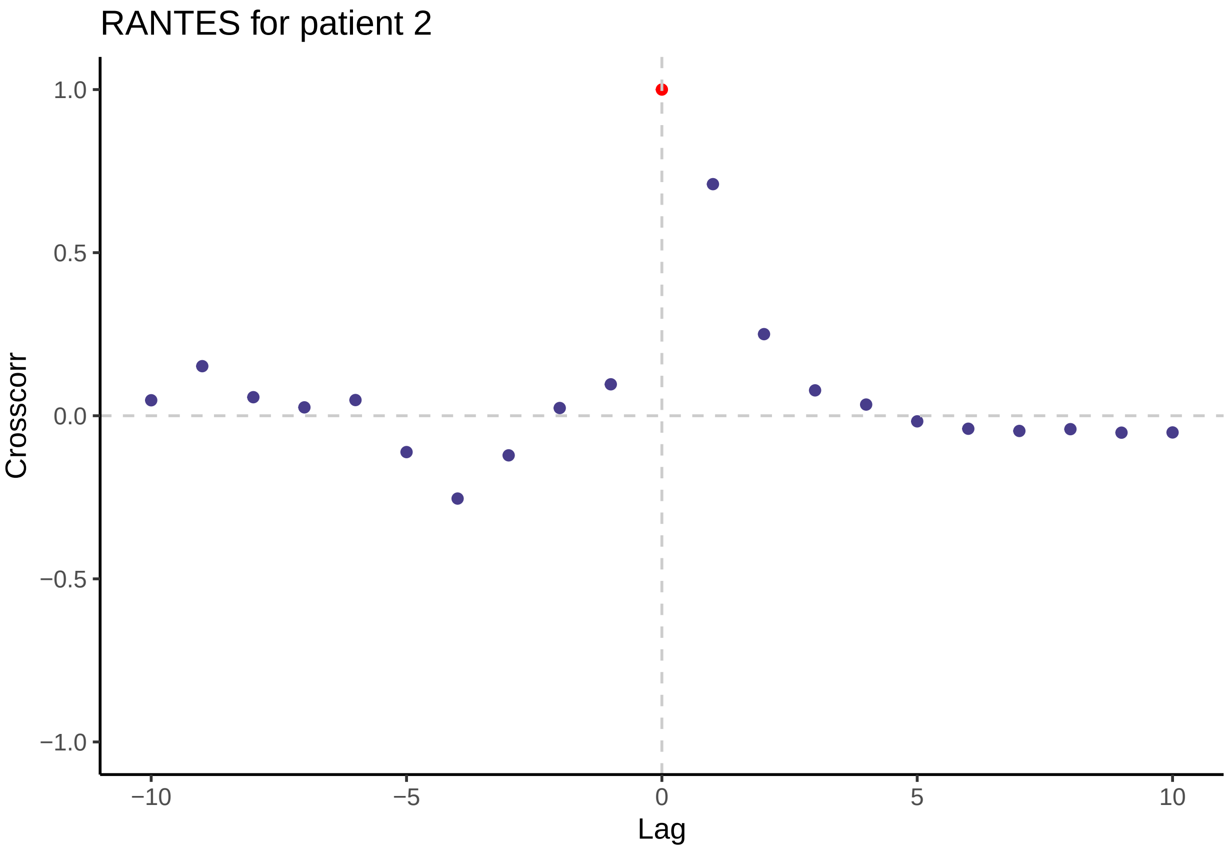


```
## [1] "RANTES for patient 2 - p-value: 0.17827564519203"
```

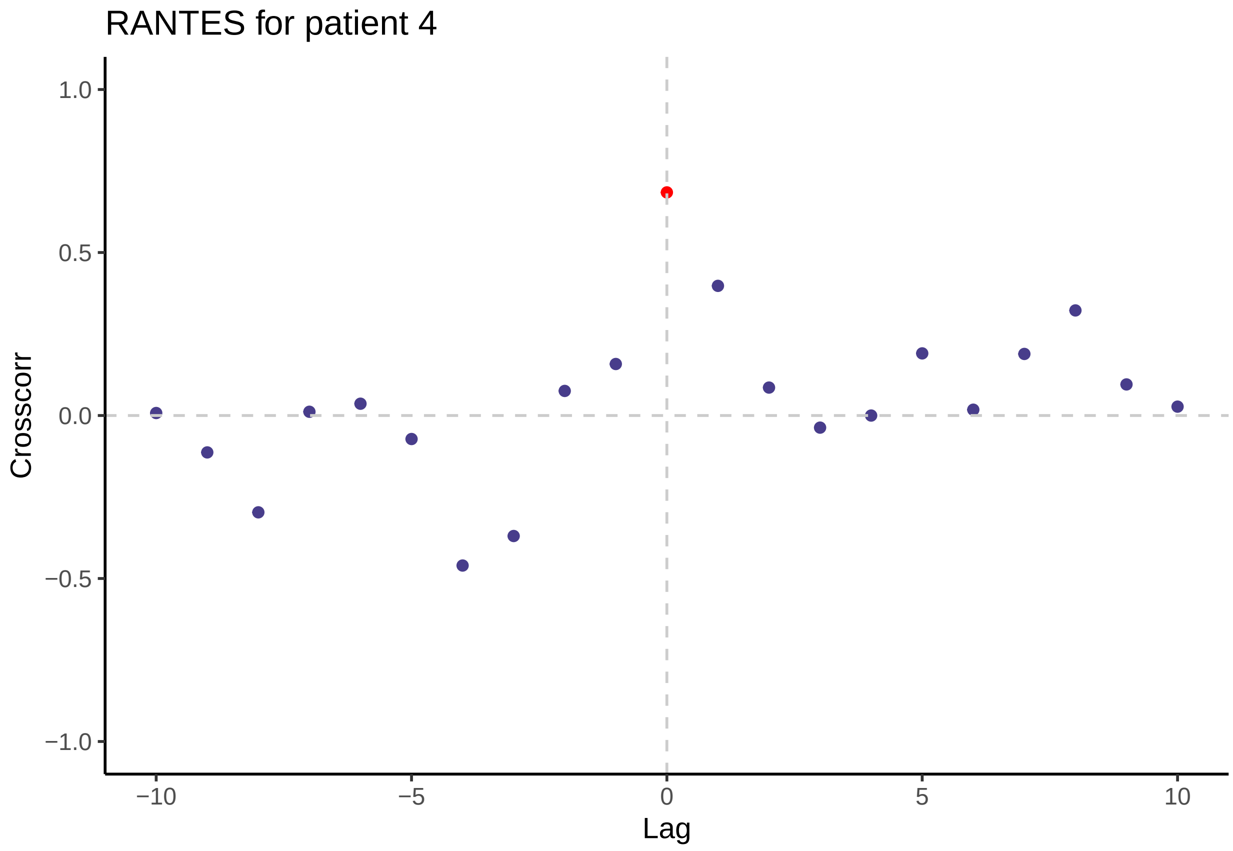


```
## [1] "RANTES for patient 4 - p-value: 0.419761968706203"
```

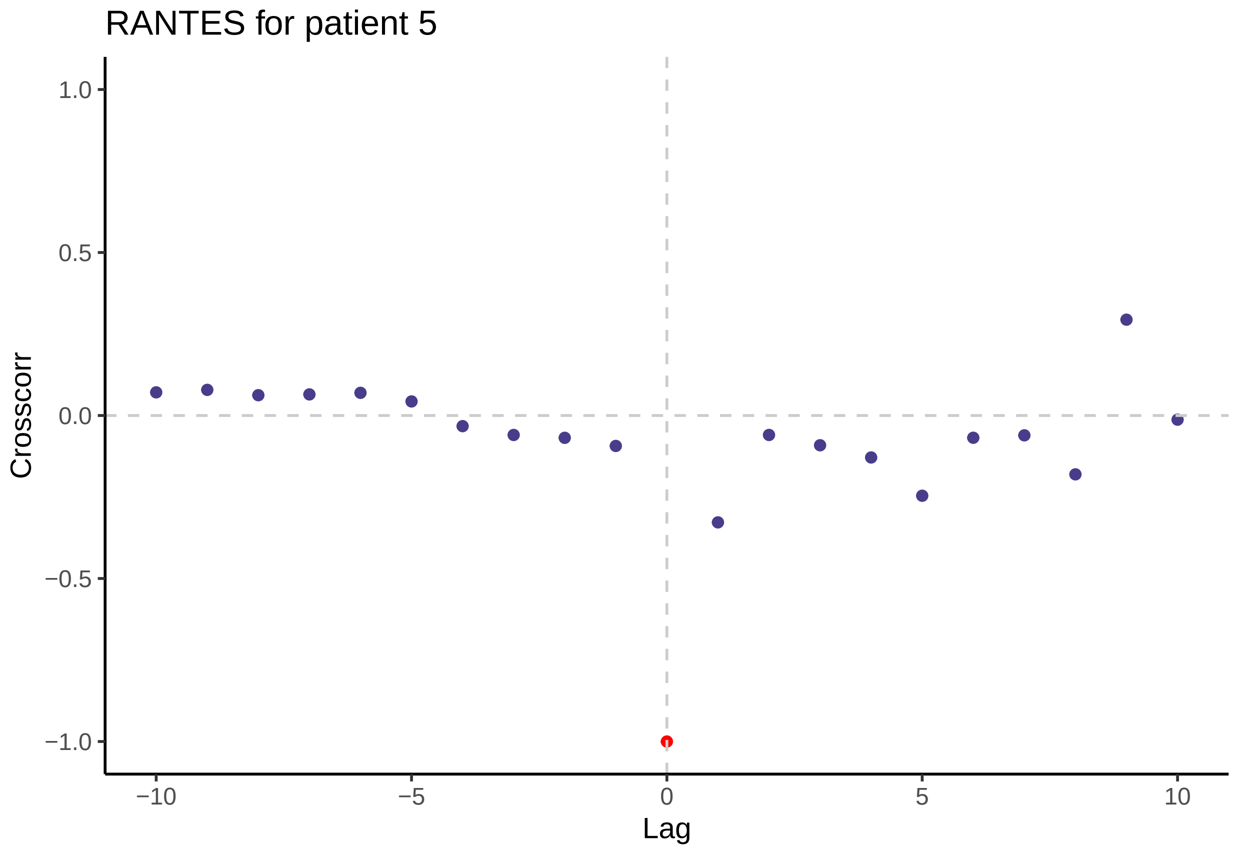


```
## [1] "RANTES for patient 5 - p-value: 0.1389058542817"
```

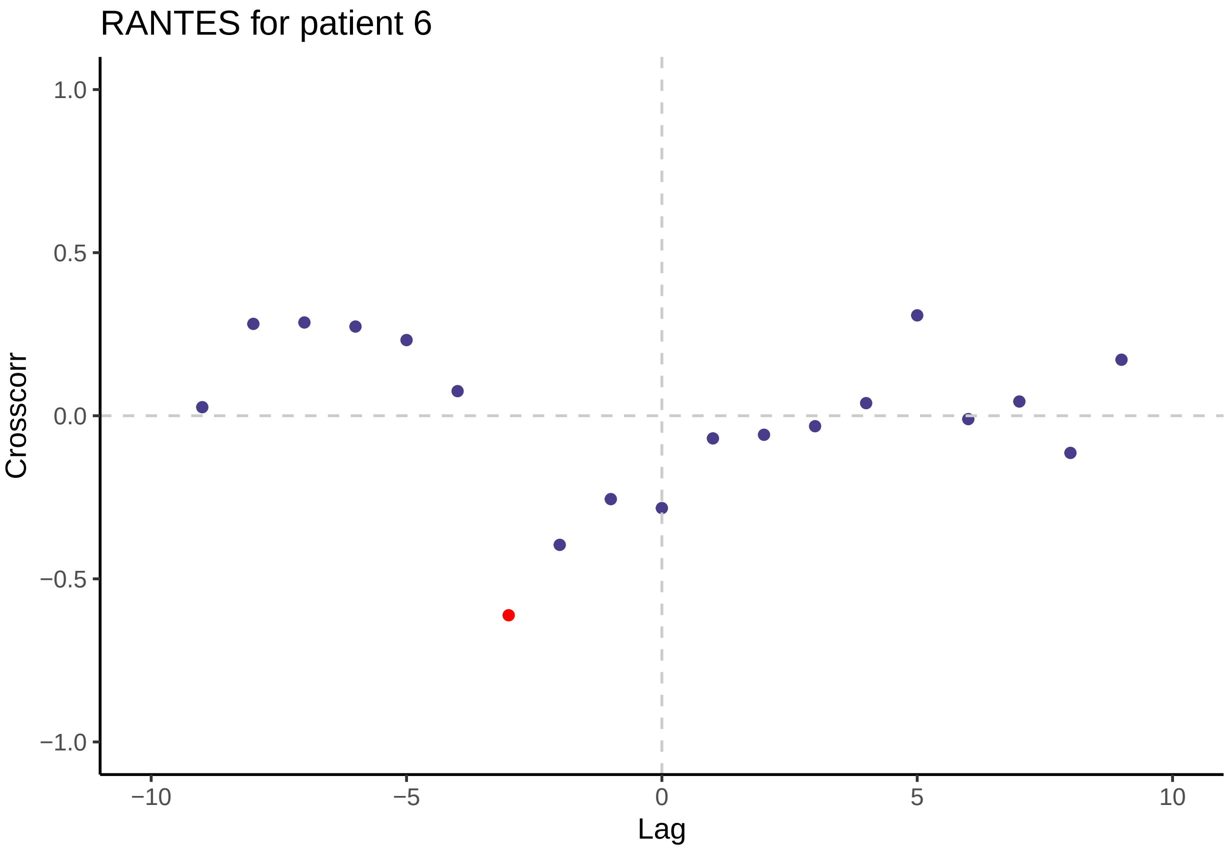


```
## [1] "RANTES for patient 6 - p-value: 0.932854097249304"
```

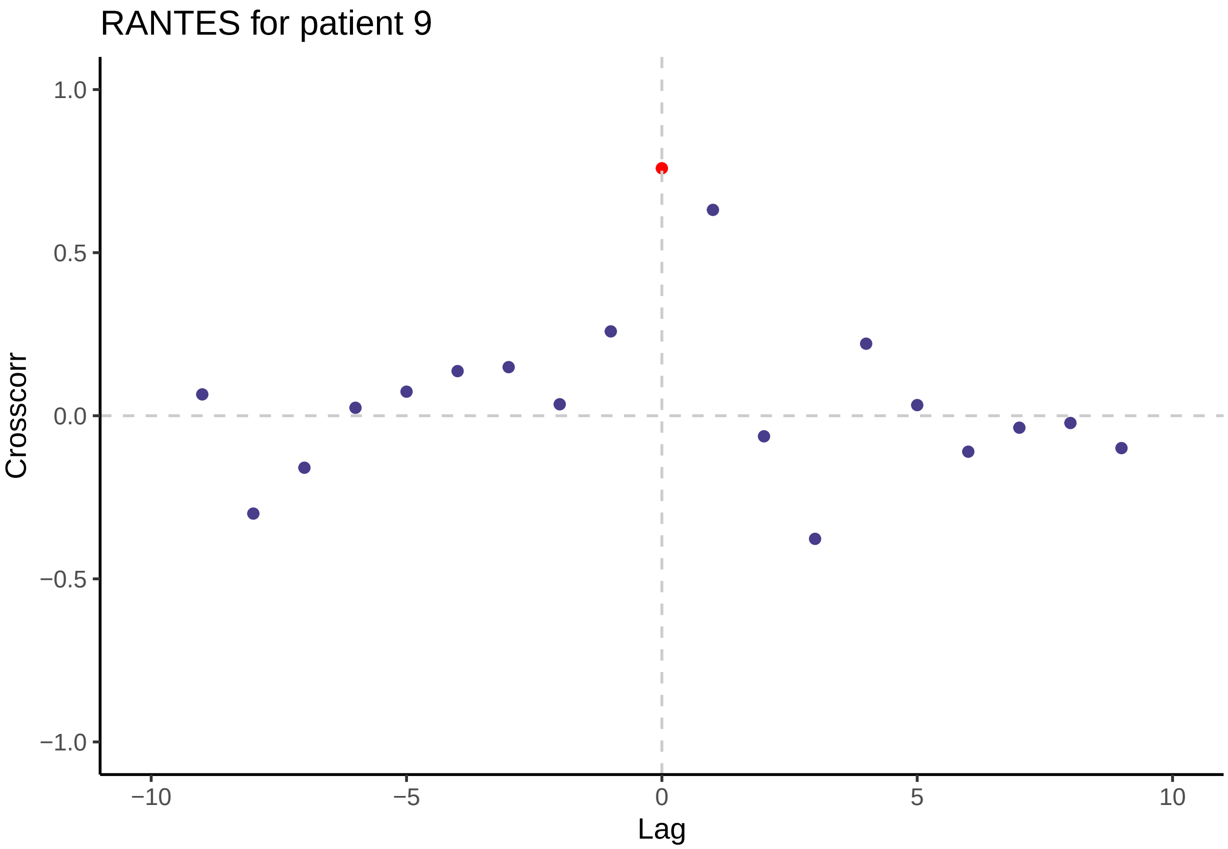


```
## [1] "RANTES for patient 9 - p-value: 0.320633697796283"
```

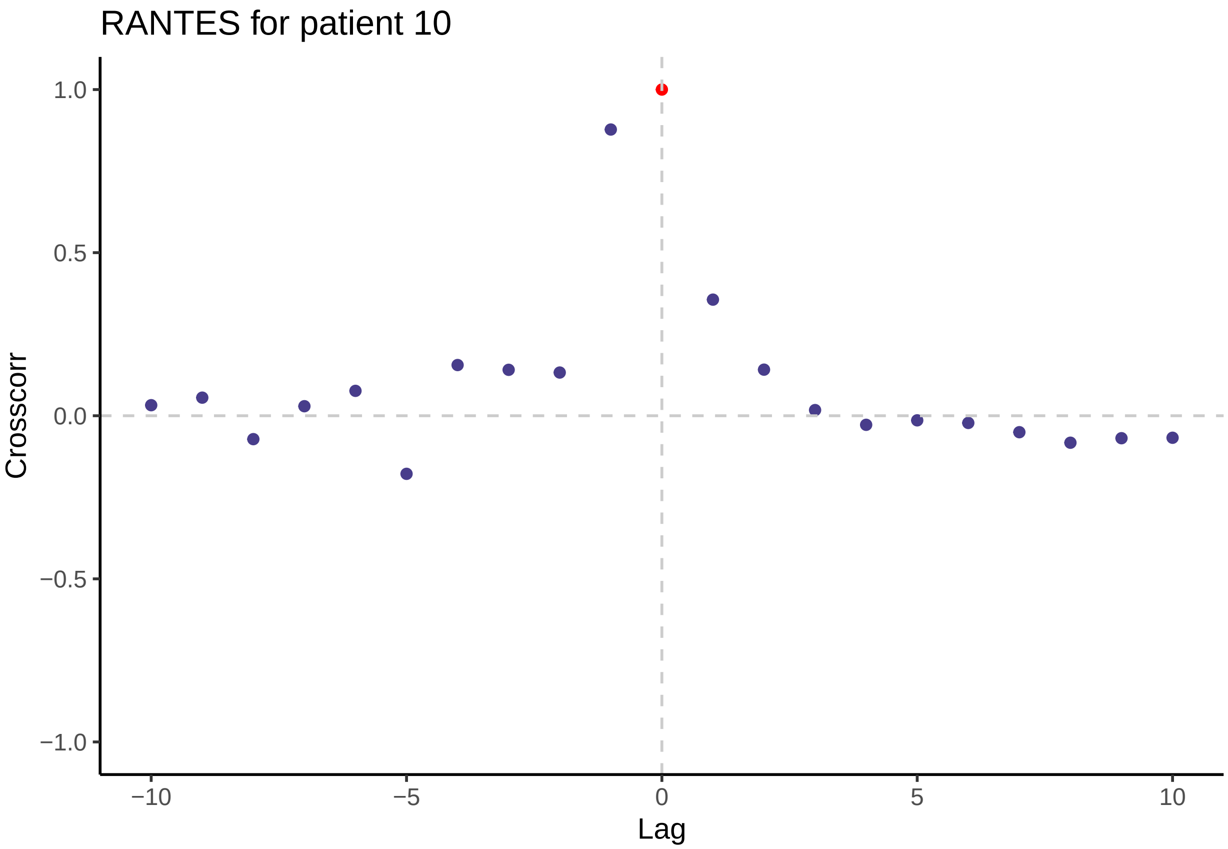


```
## [1] "RANTES for patient 10 - p-value: 0.089227759492657"
```

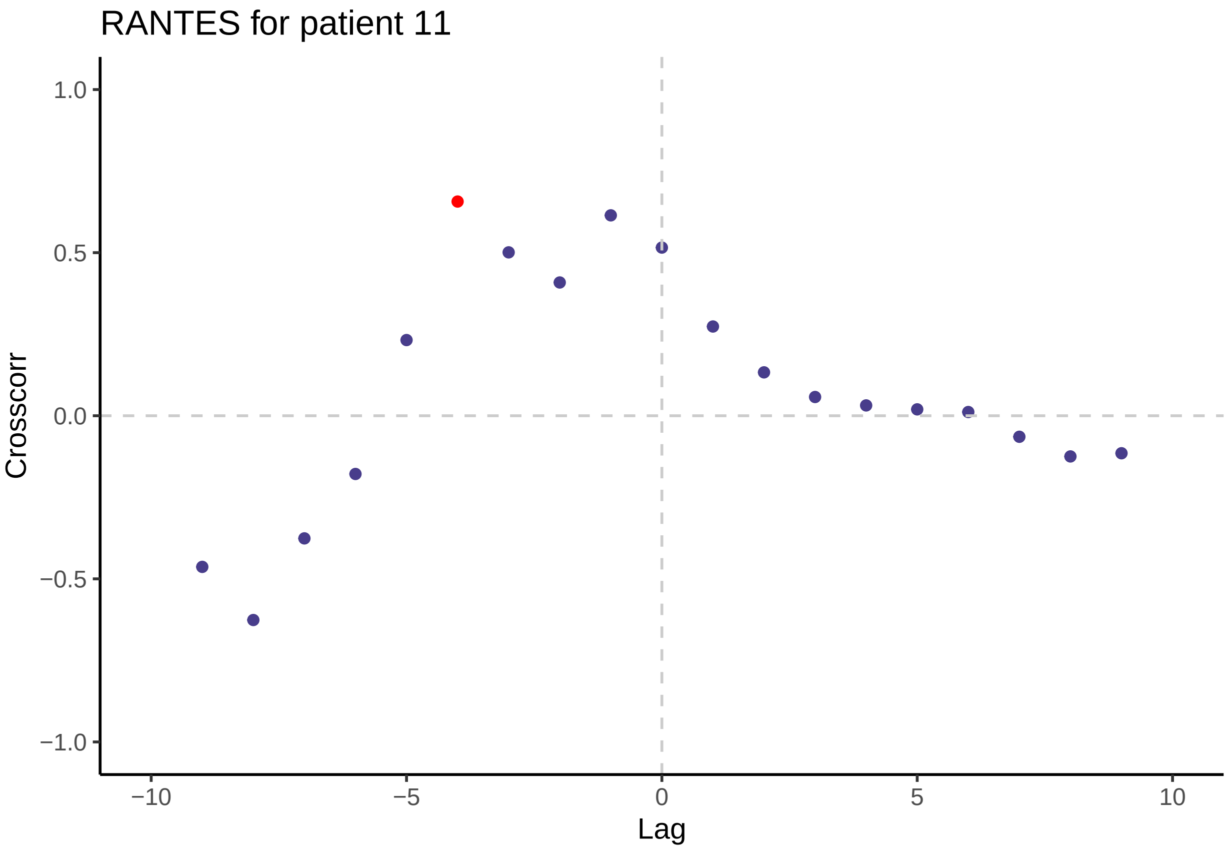


```
## [1] "RANTES for patient 11 - p-value: 0.34993082040782"
```

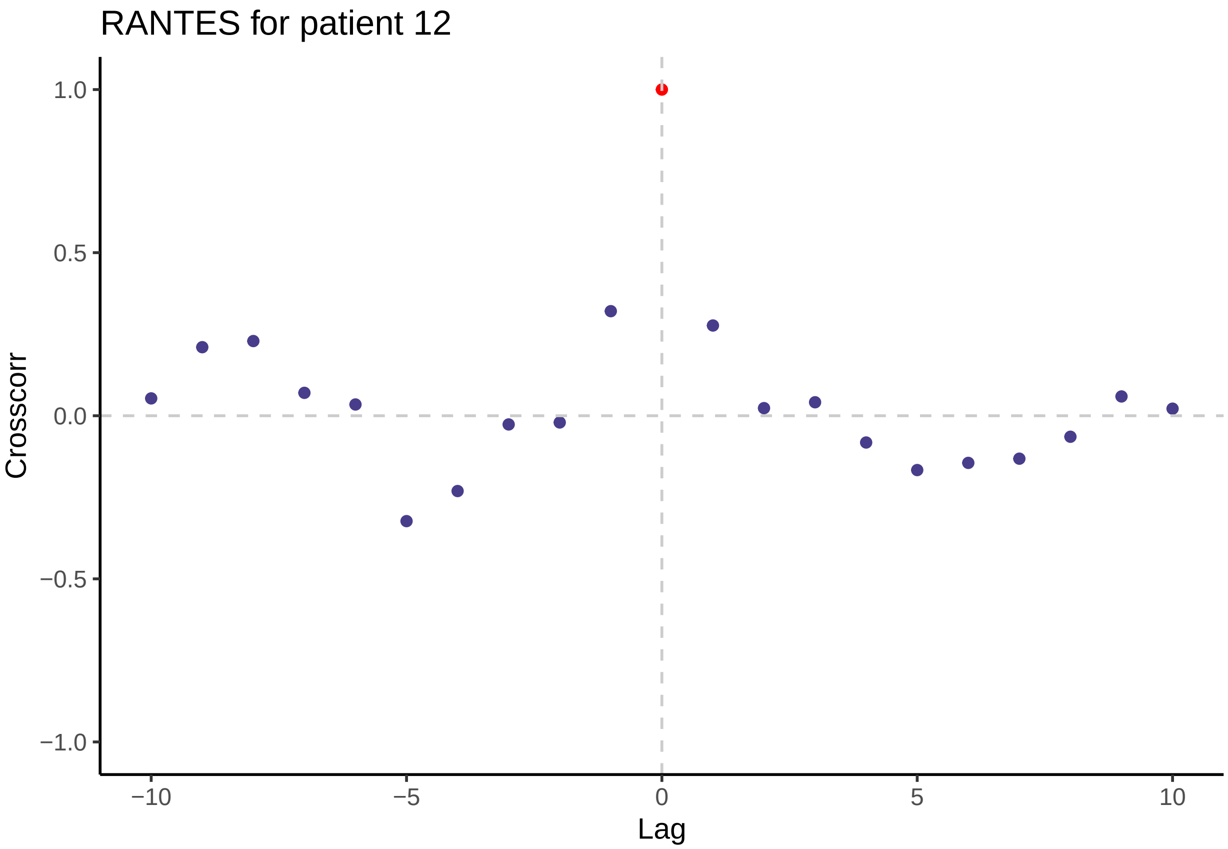


```
## [1] "RANTES for patient 12 - p-value: 0.364594904038136"
```

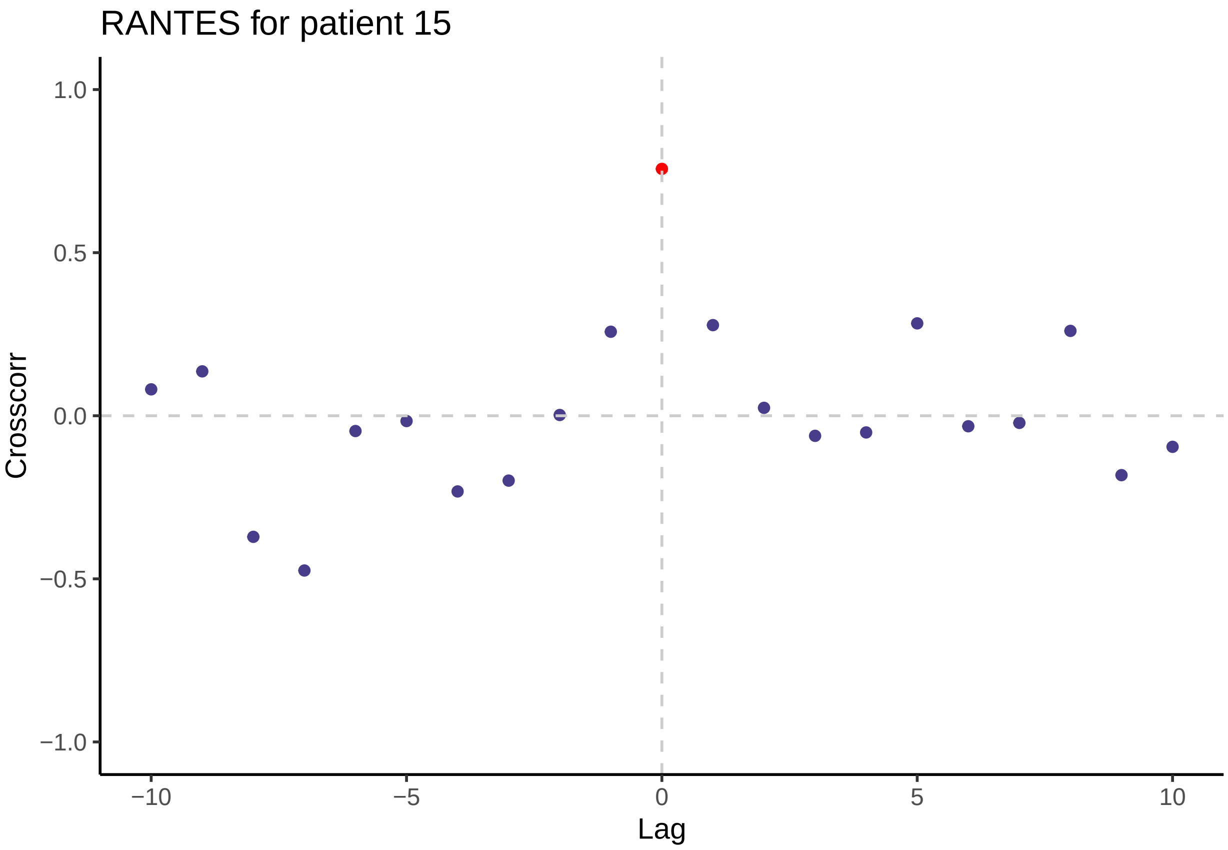


```
## [1] "RANTES for patient 15 - p-value: 0.809506519899383"
```

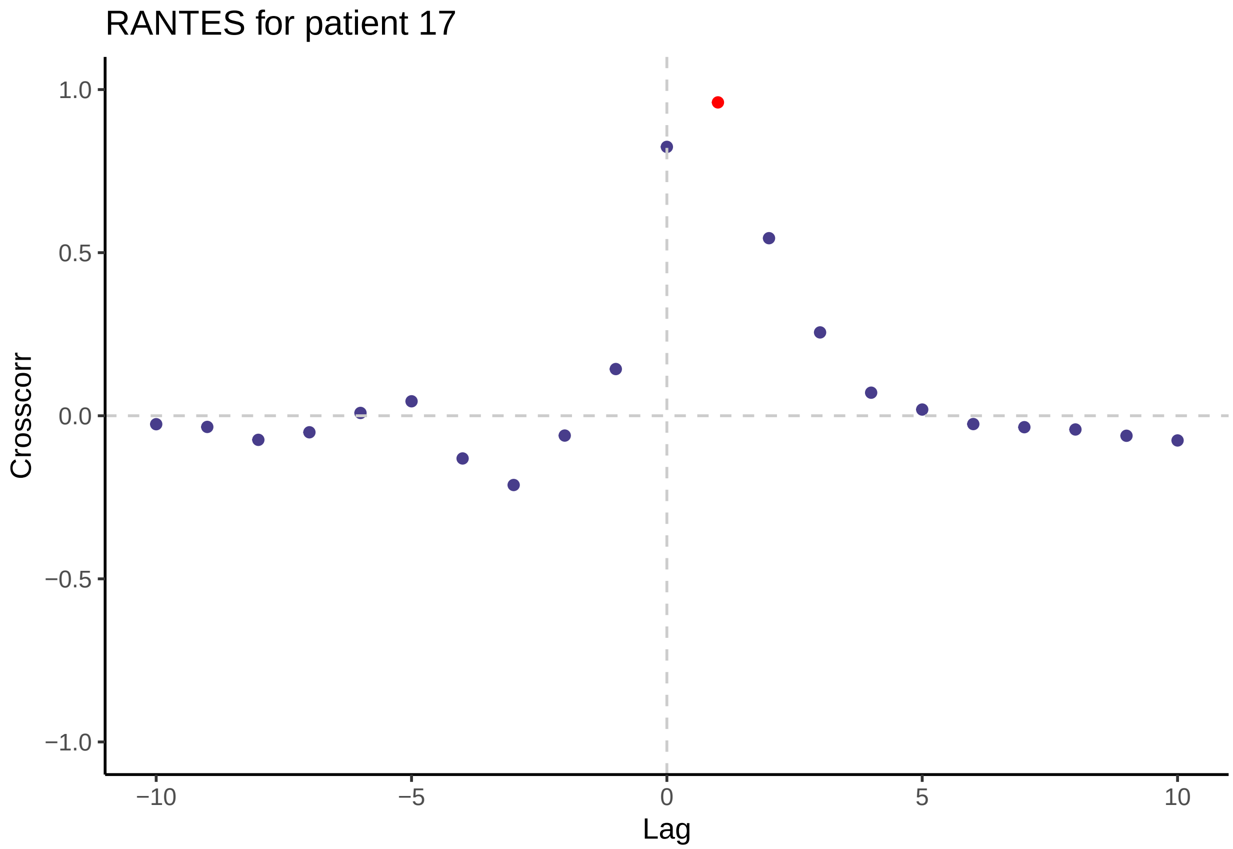


```
## [1] "RANTES for patient 17 - p-value: 0.161716482860387"
## Warning: Removed 3 rows containing missing values (geom_point).
```

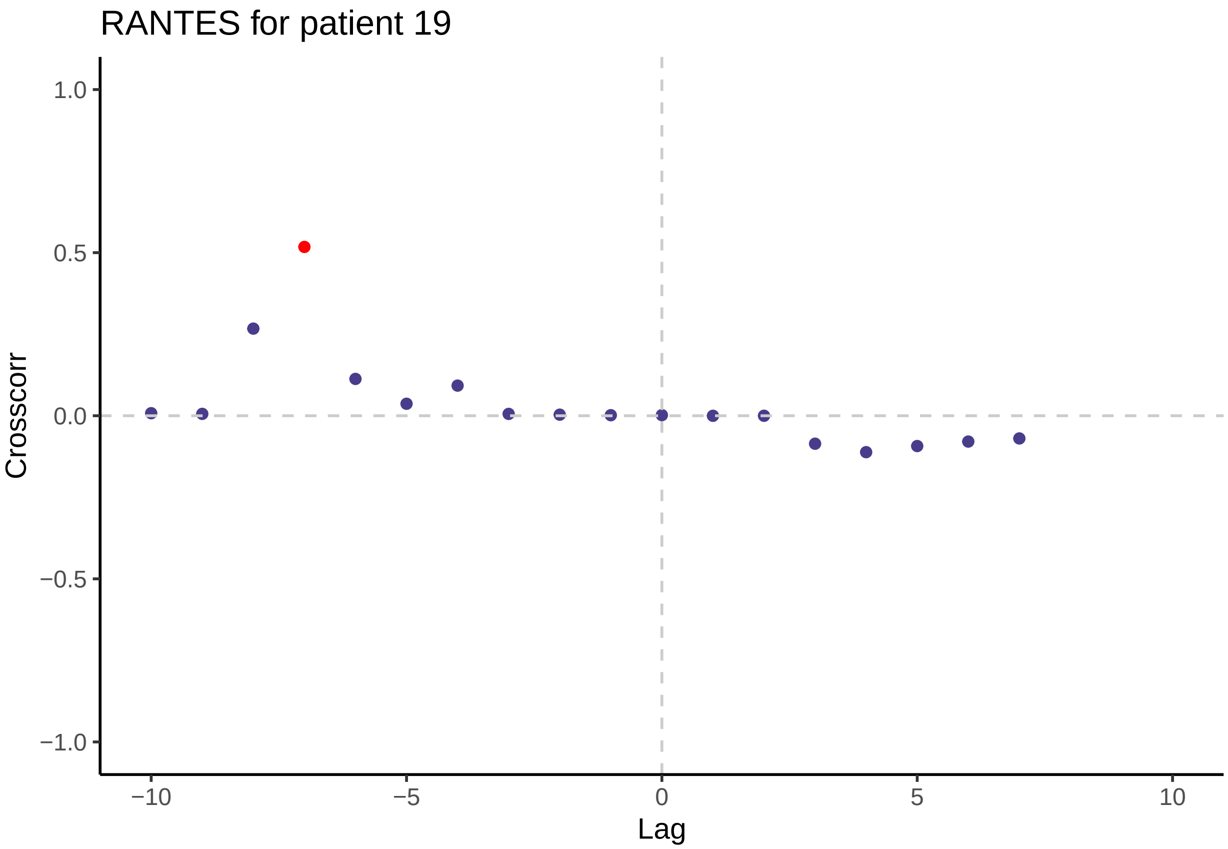


```
## [1] "RANTES for patient 19 - p-value: 0.346929837077333"
```

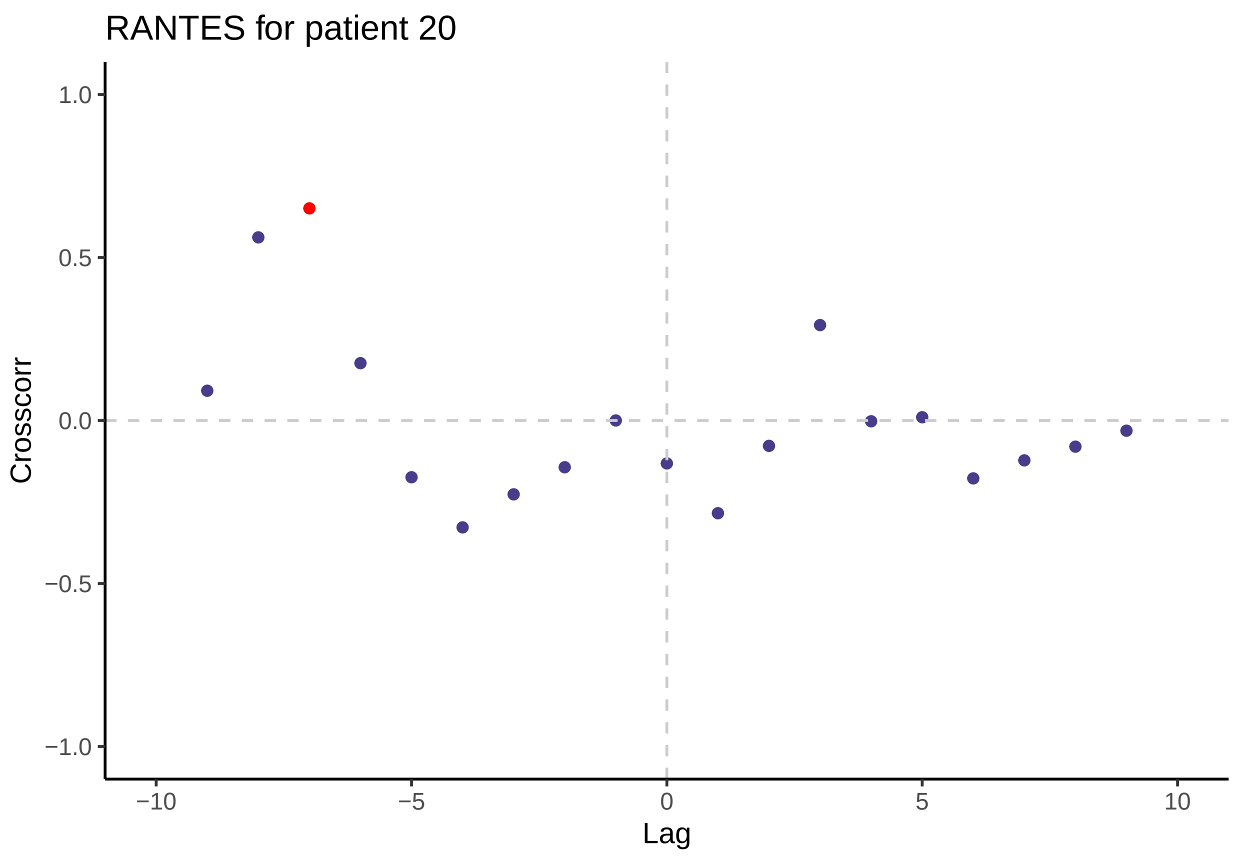


```
## [1] "RANTES for patient 20 - p-value: 0.997553316012362"
```

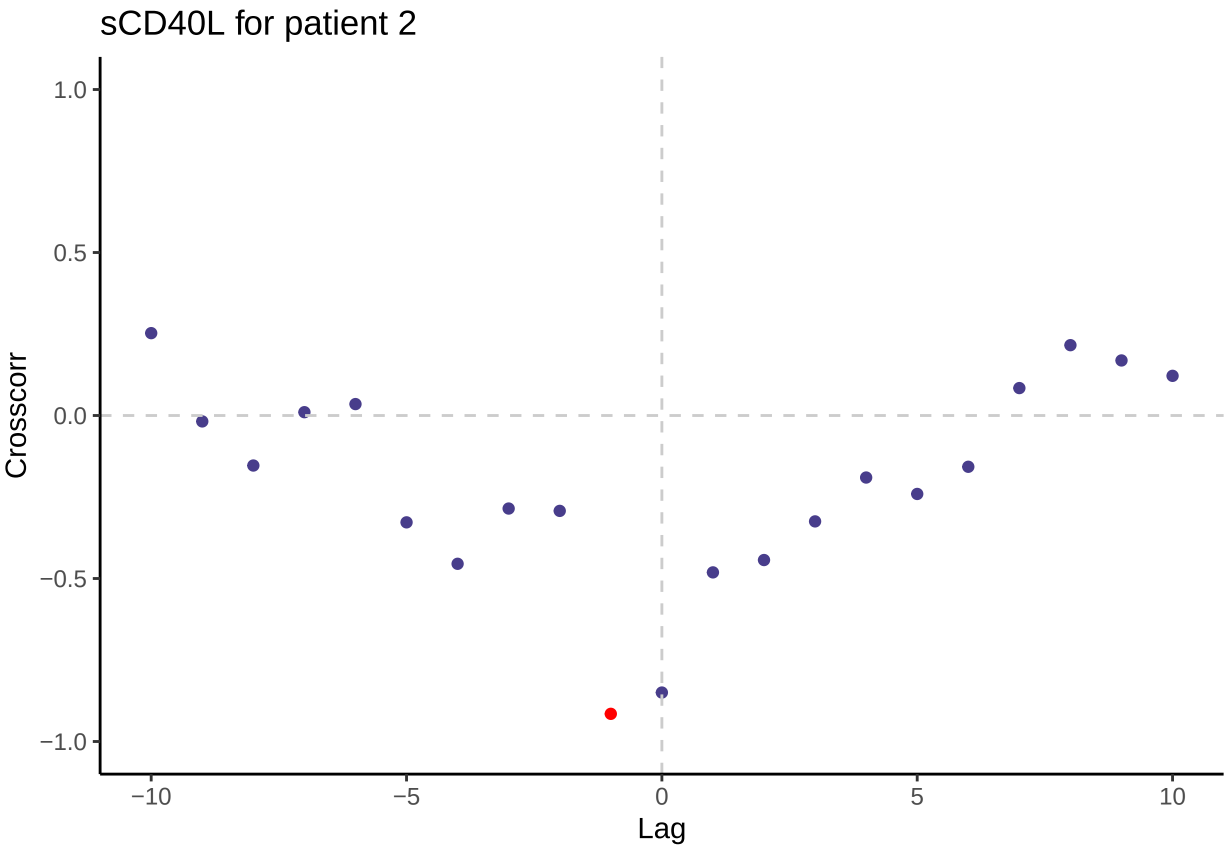


```
## [1] "sCD40L for patient 2 - p-value: 0.00856366097823449"
```

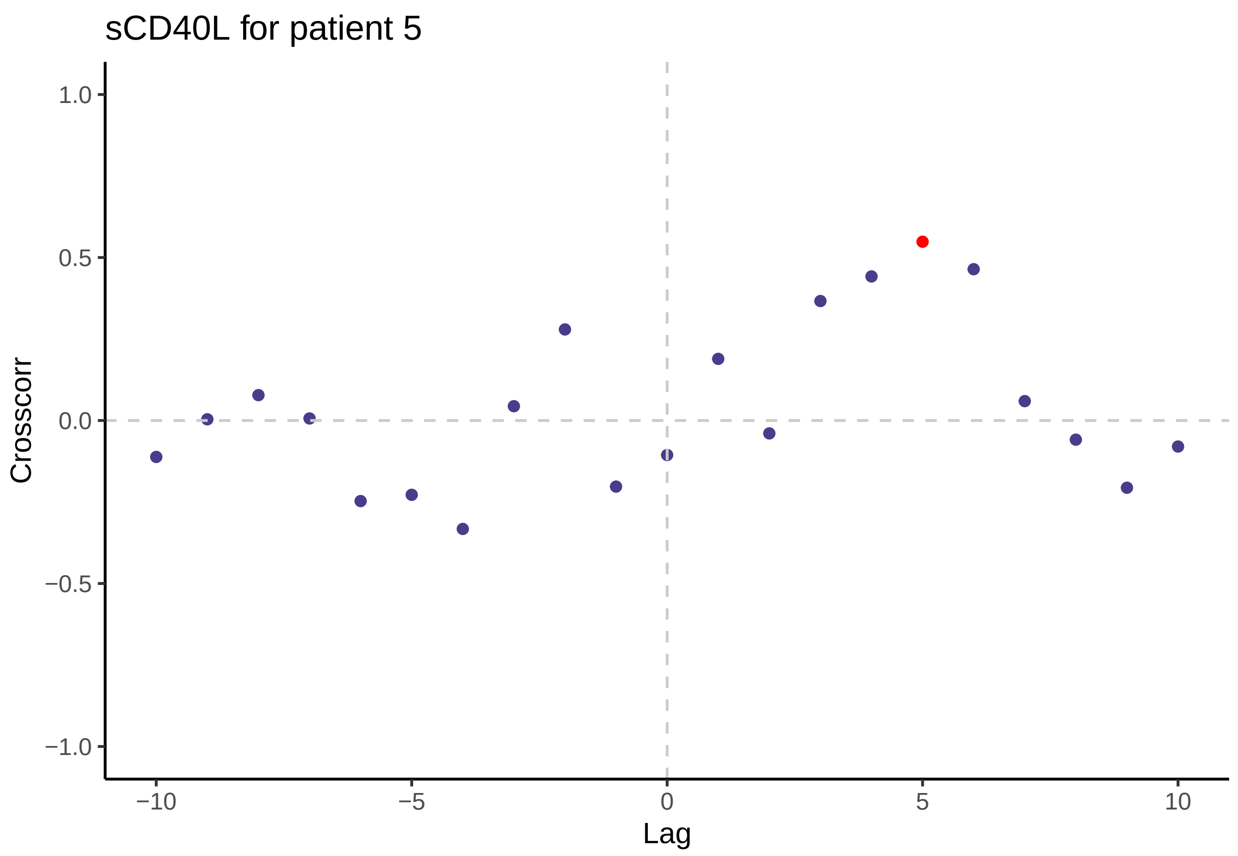


```
## [1] "sCD40L for patient 5 - p-value: 0.462181278931092"
```

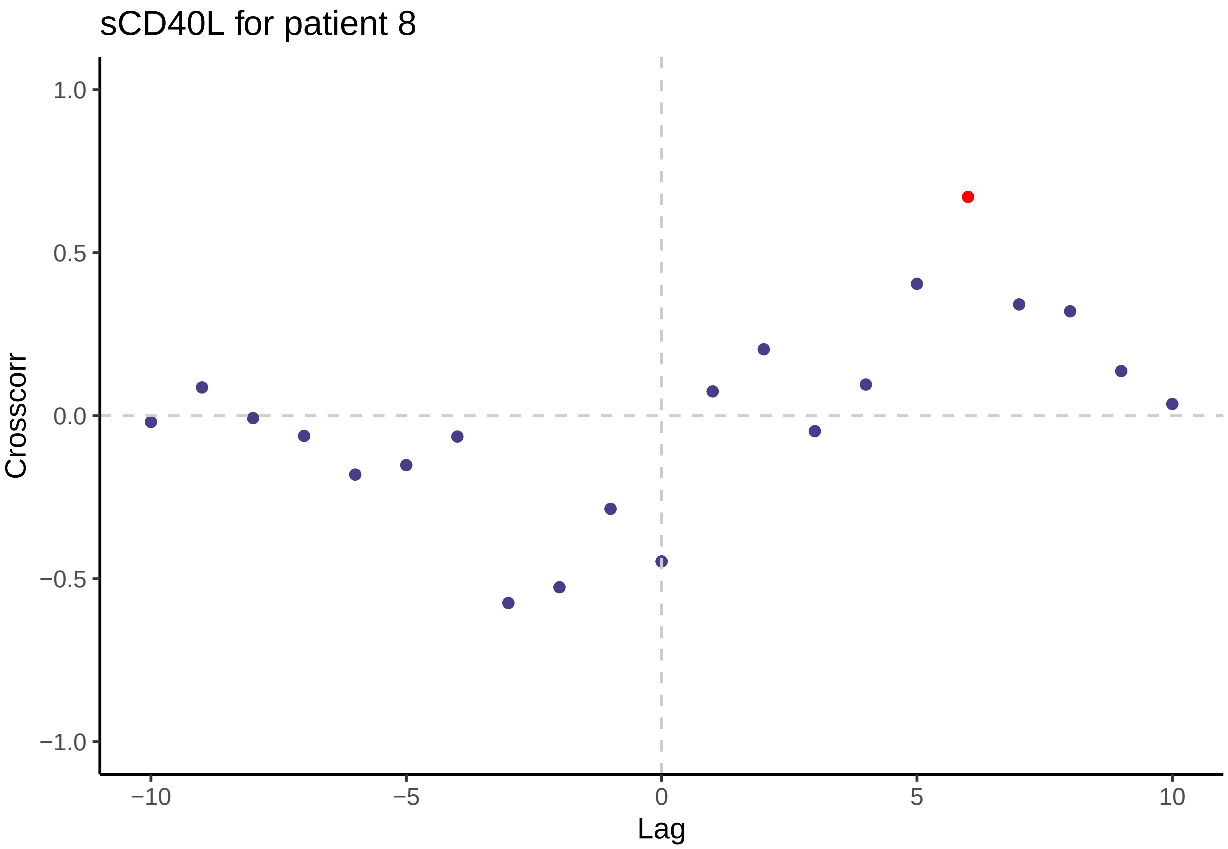


```
## [1] "sCD40L for patient 8 - p-value: 0.995198386615297"
```

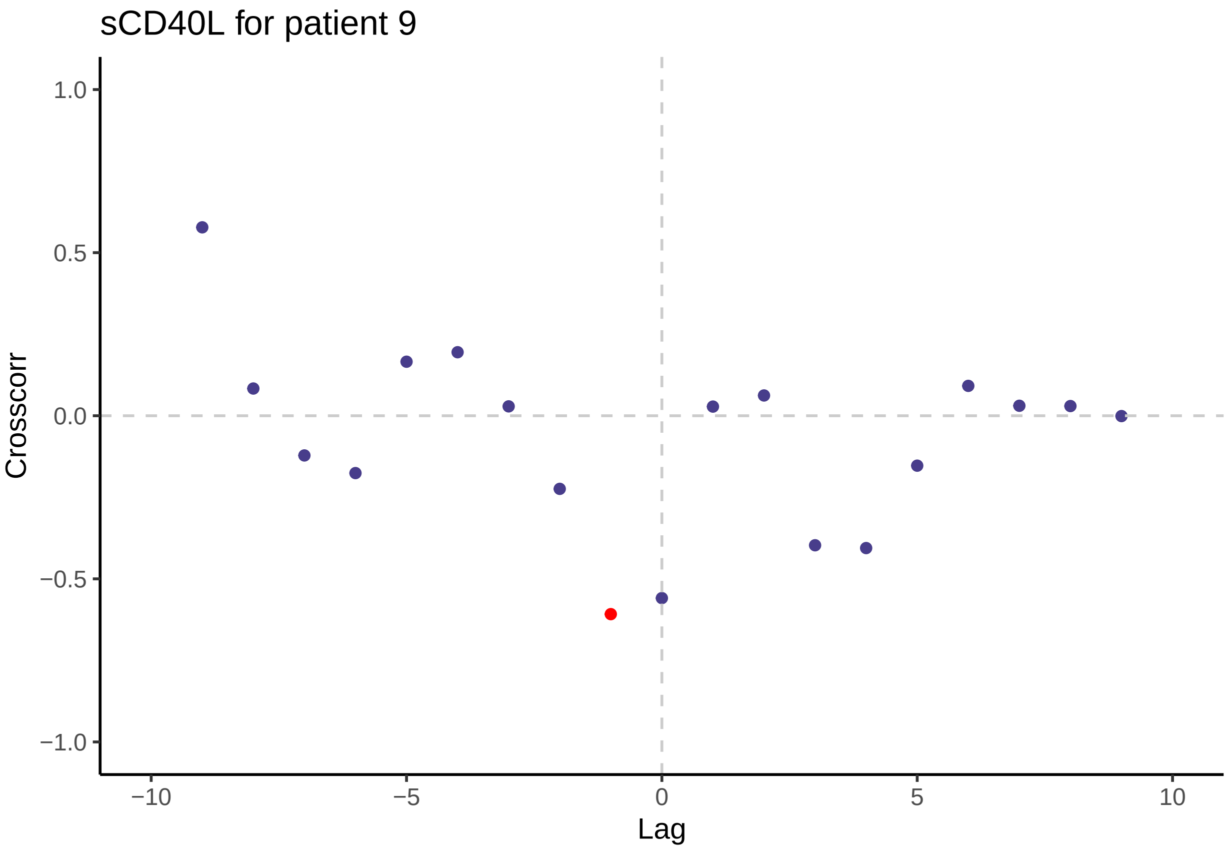


```
## [1] "sCD40L for patient 9 - p-value: 0.288599336745794"
```

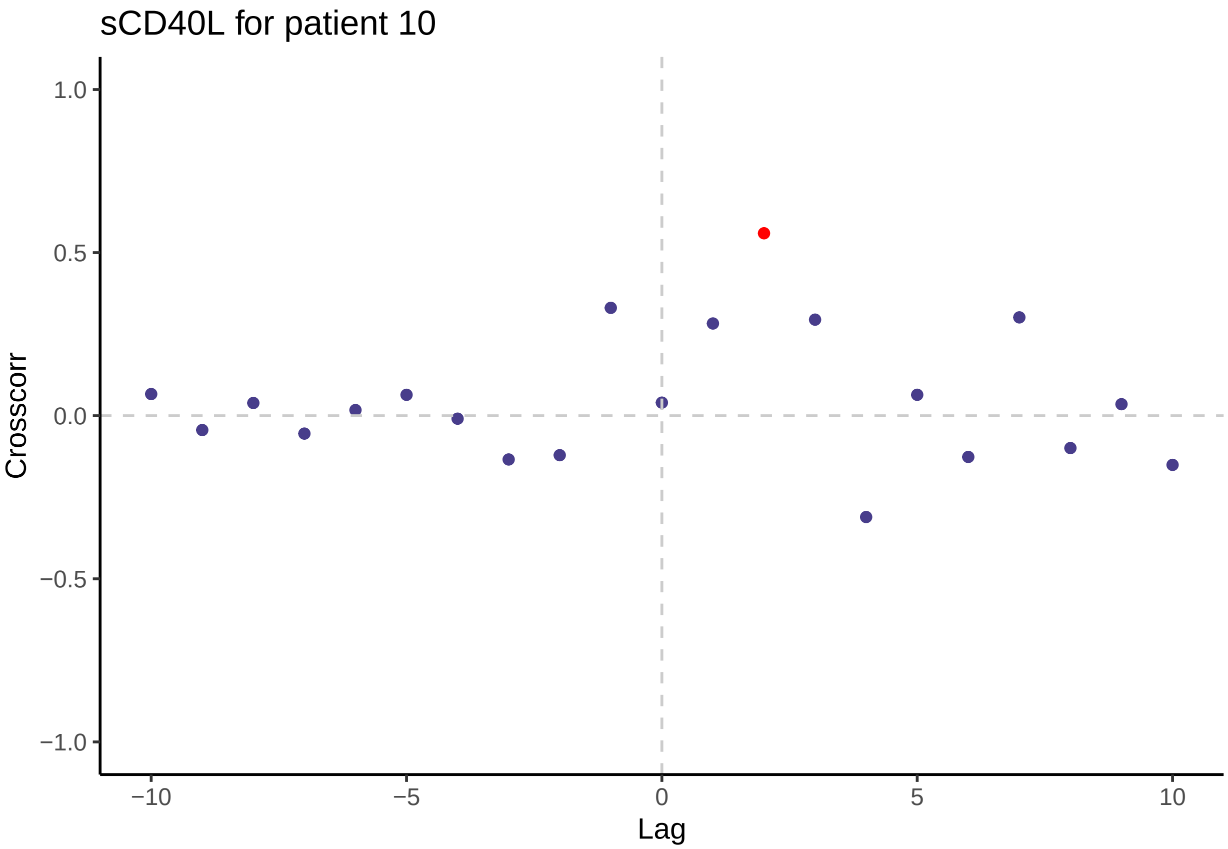


```
## [1] "sCD40L for patient 10 - p-value: 0.274305592307718"
```

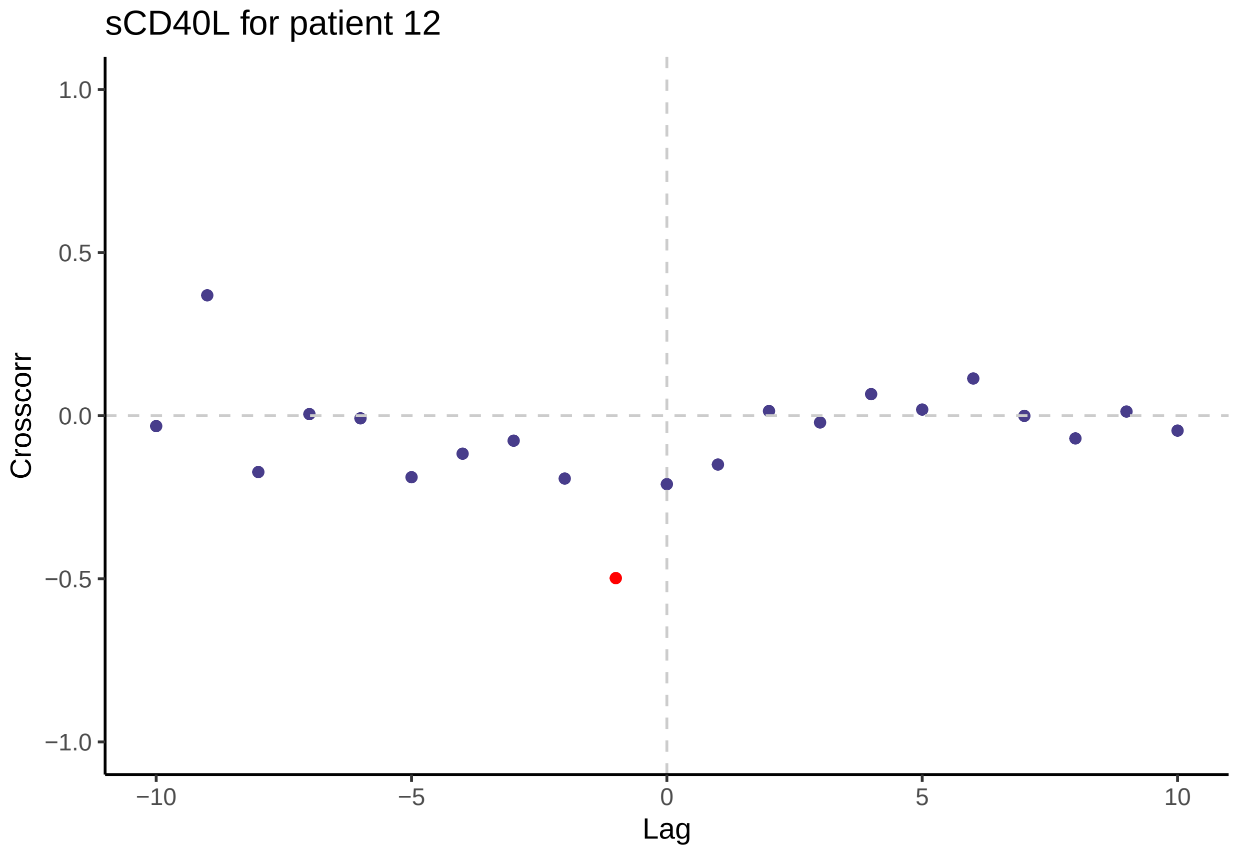


```
## [1] "sCD40L for patient 12 - p-value: 0.131061995811394"
## Warning: Removed 2 rows containing missing values (geom_point).
```

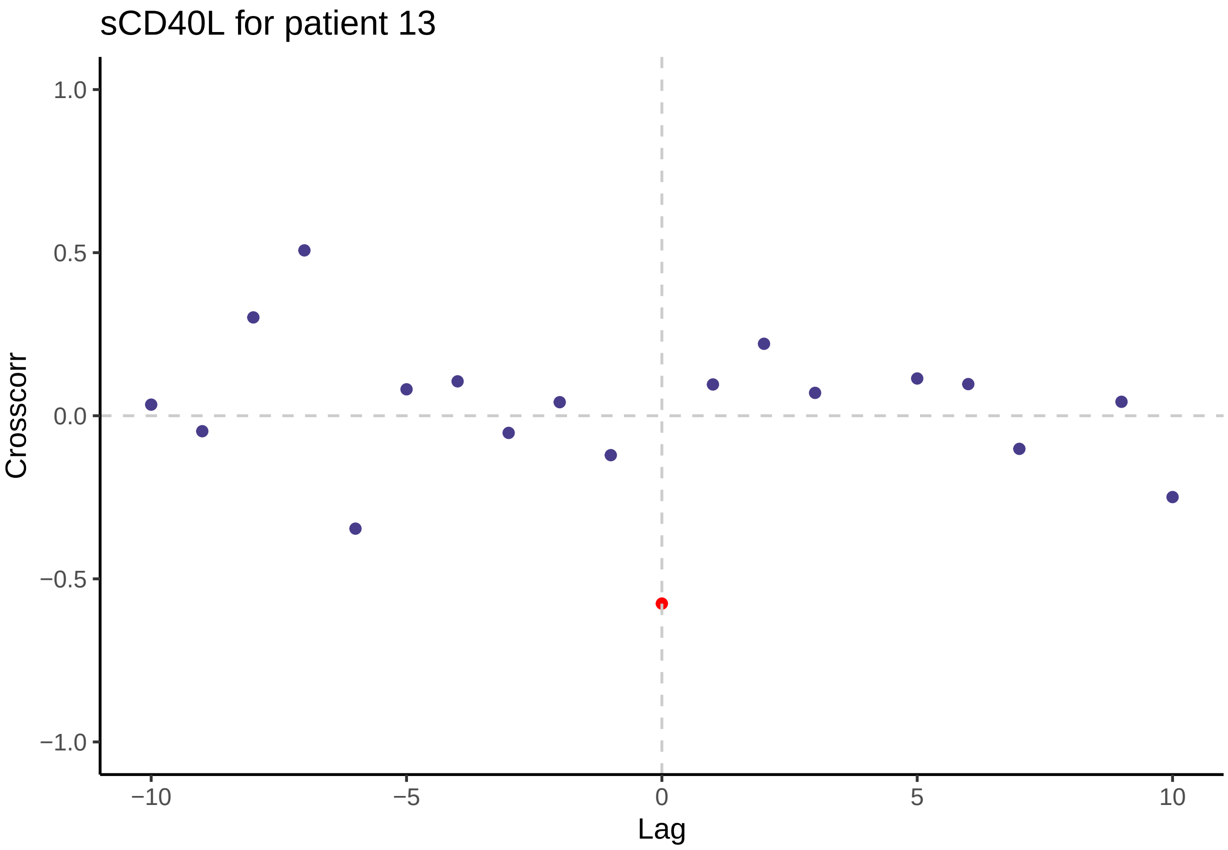


```
## [1] "sCD40L for patient 13 - p-value: 0.833660511817156"
```

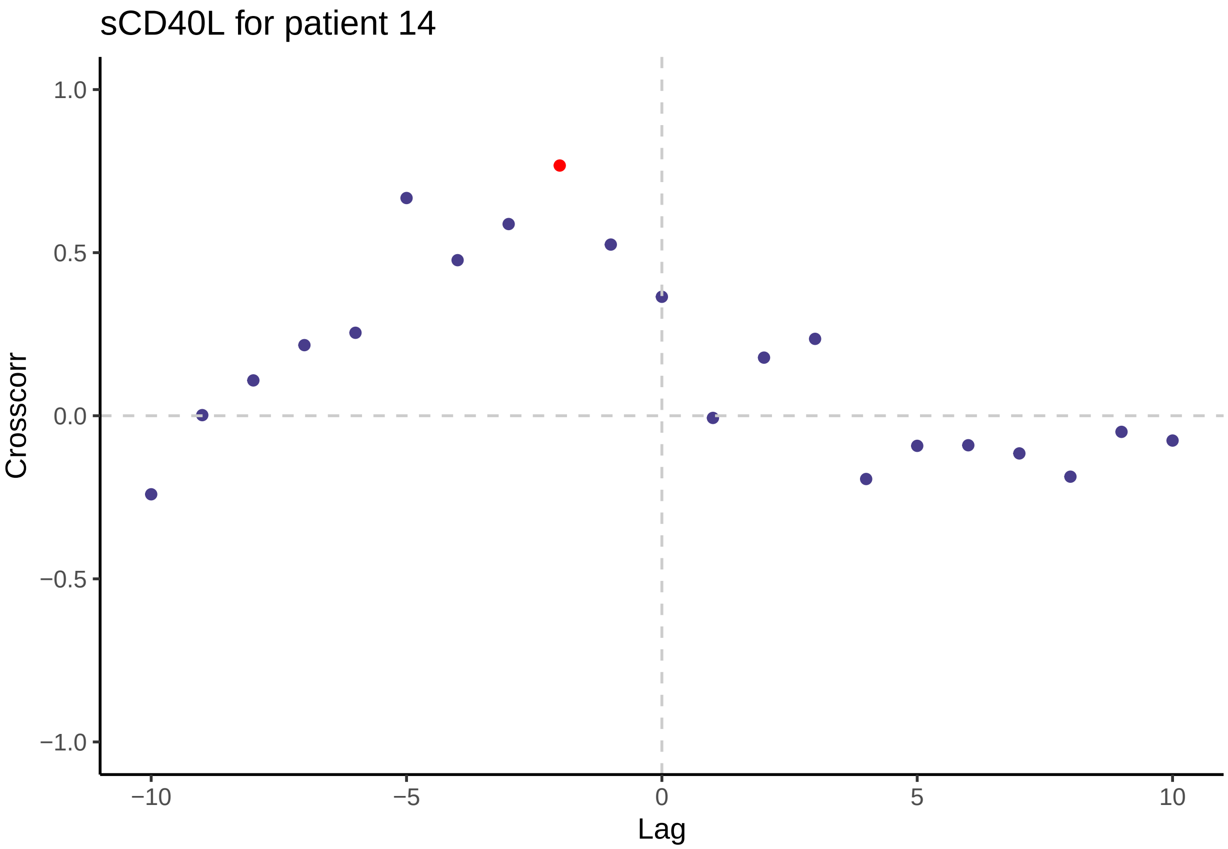


```
## [1] "sCD40L for patient 14 - p-value: 0.0273367655908593"
## Warning: Removed 1 rows containing missing values (geom_point).
```

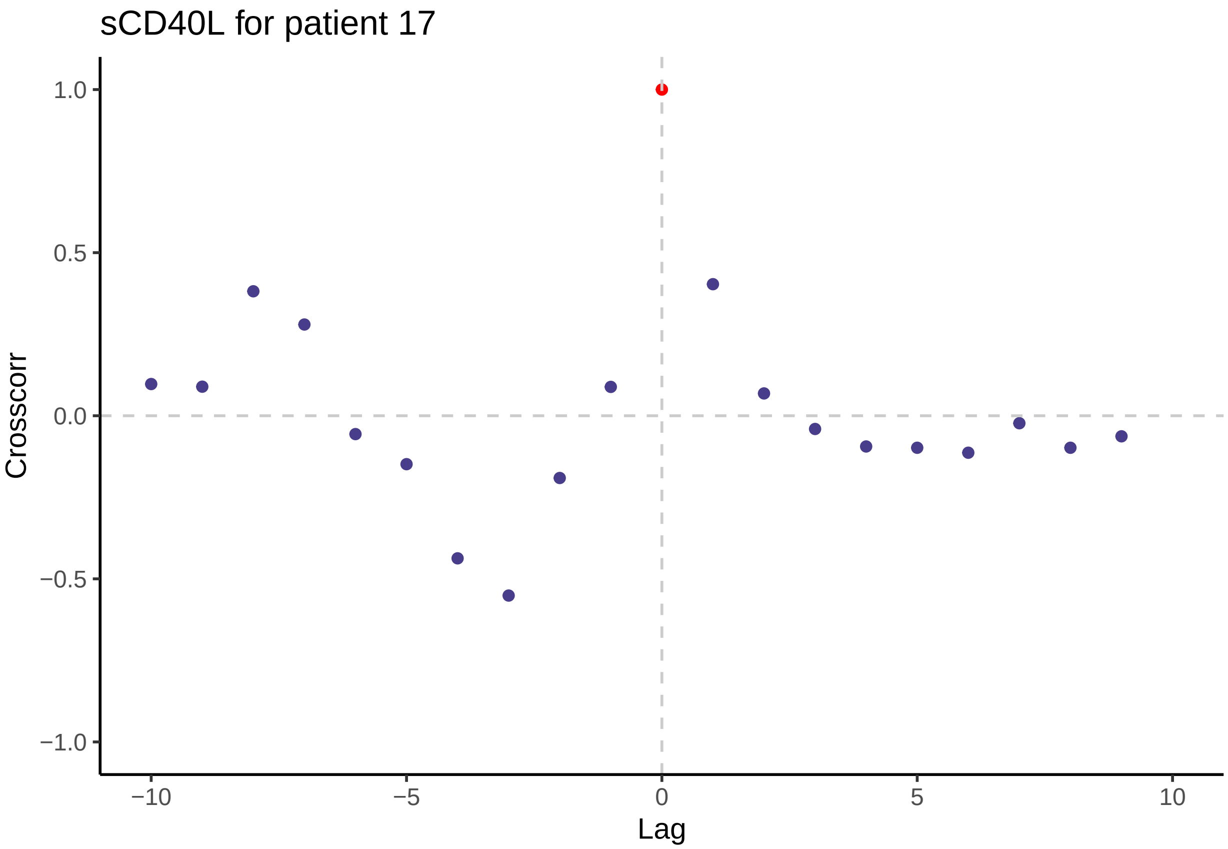


```
## [1] "sCD40L for patient 17 - p-value: 0.737733162074147"
```

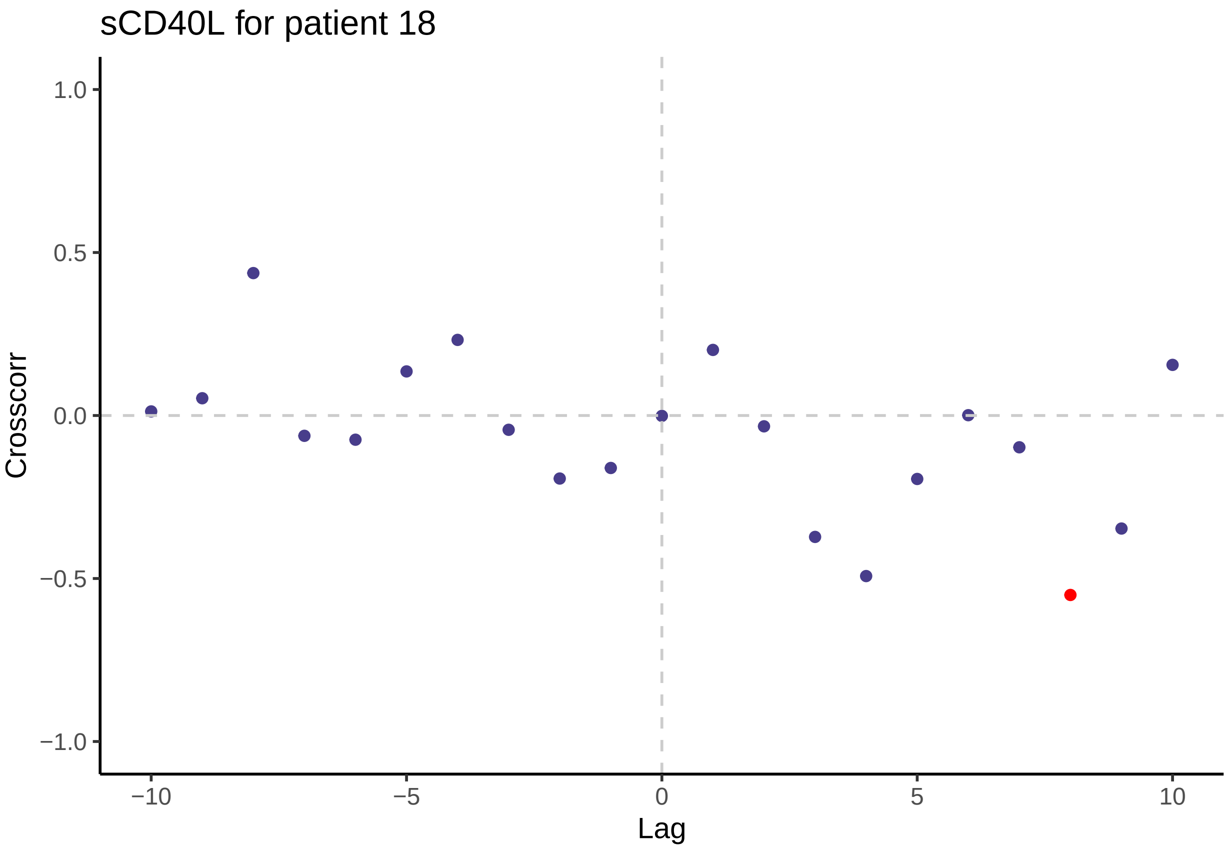


```
## [1] "sCD40L for patient 18 - p-value: 0.221431719021208"
```

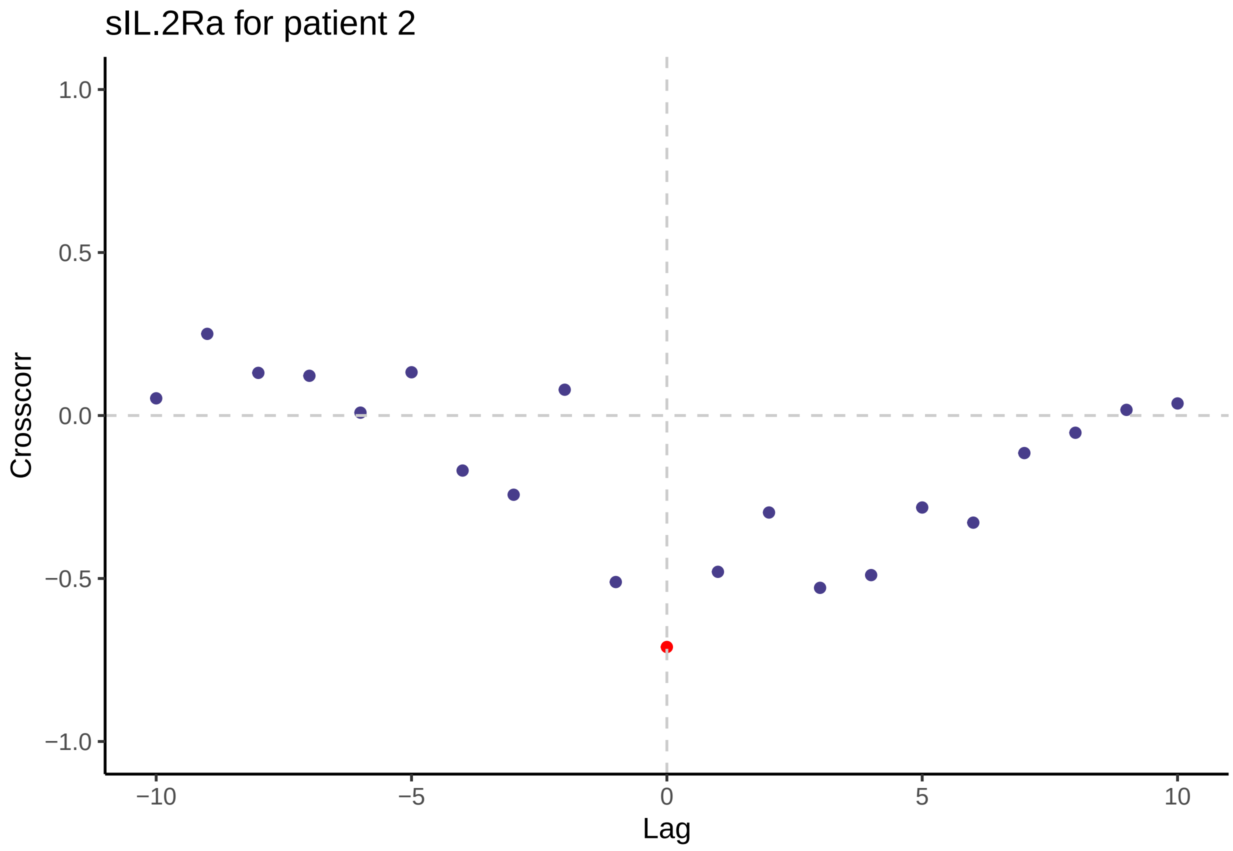


```
## [1] "sIL.2Ra for patient 2 - p-value: 0.013375193675771"
```

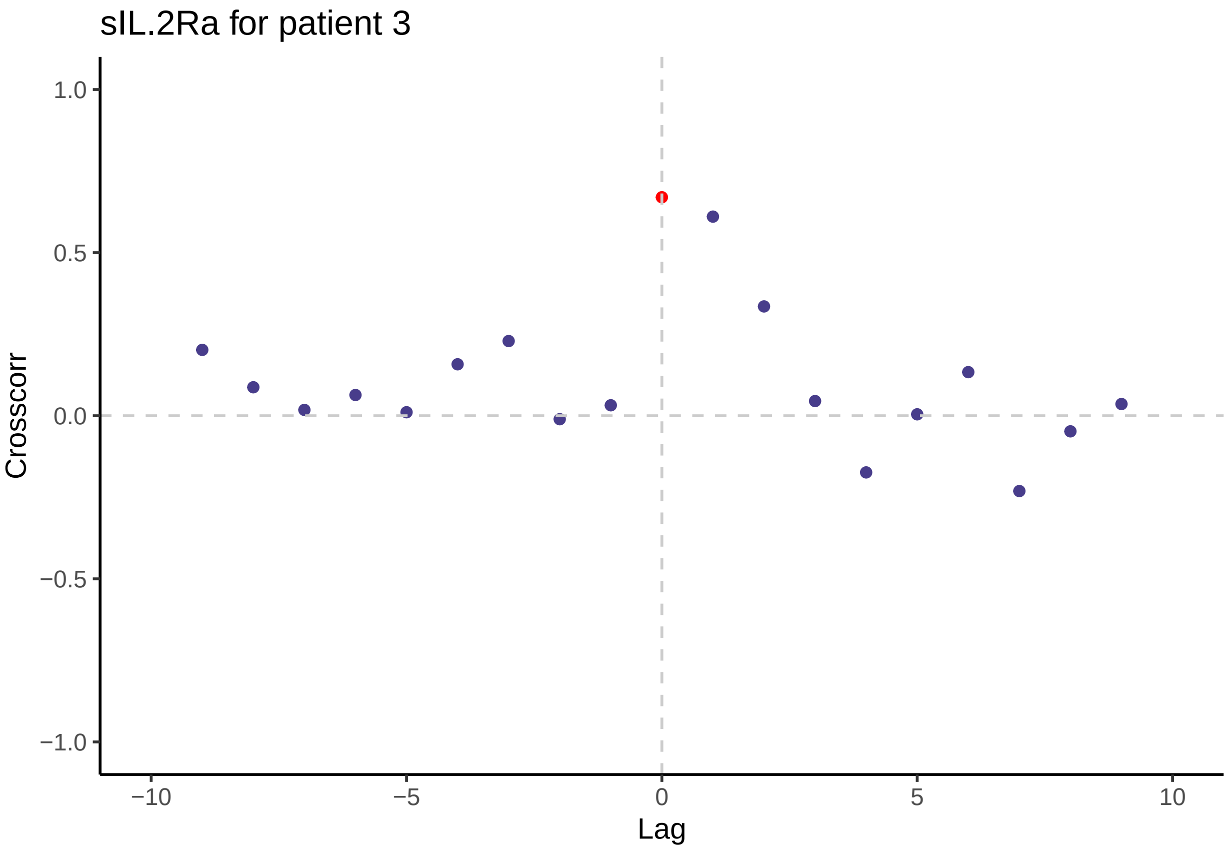


```
## [1] "sIL.2Ra for patient 3 - p-value: 0.0411498320028593"
```

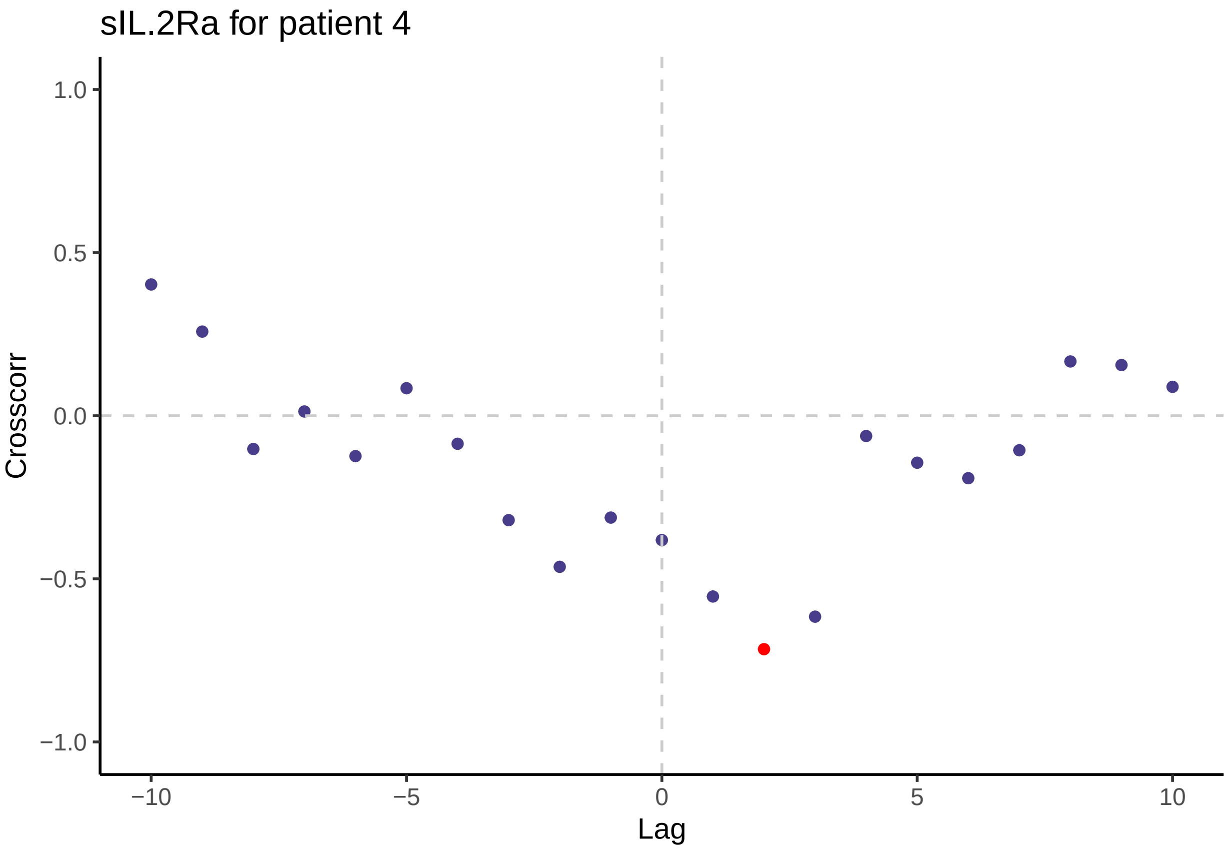


```
## [1] "sIL.2Ra for patient 4 - p-value: 0.0371248829854298"
```

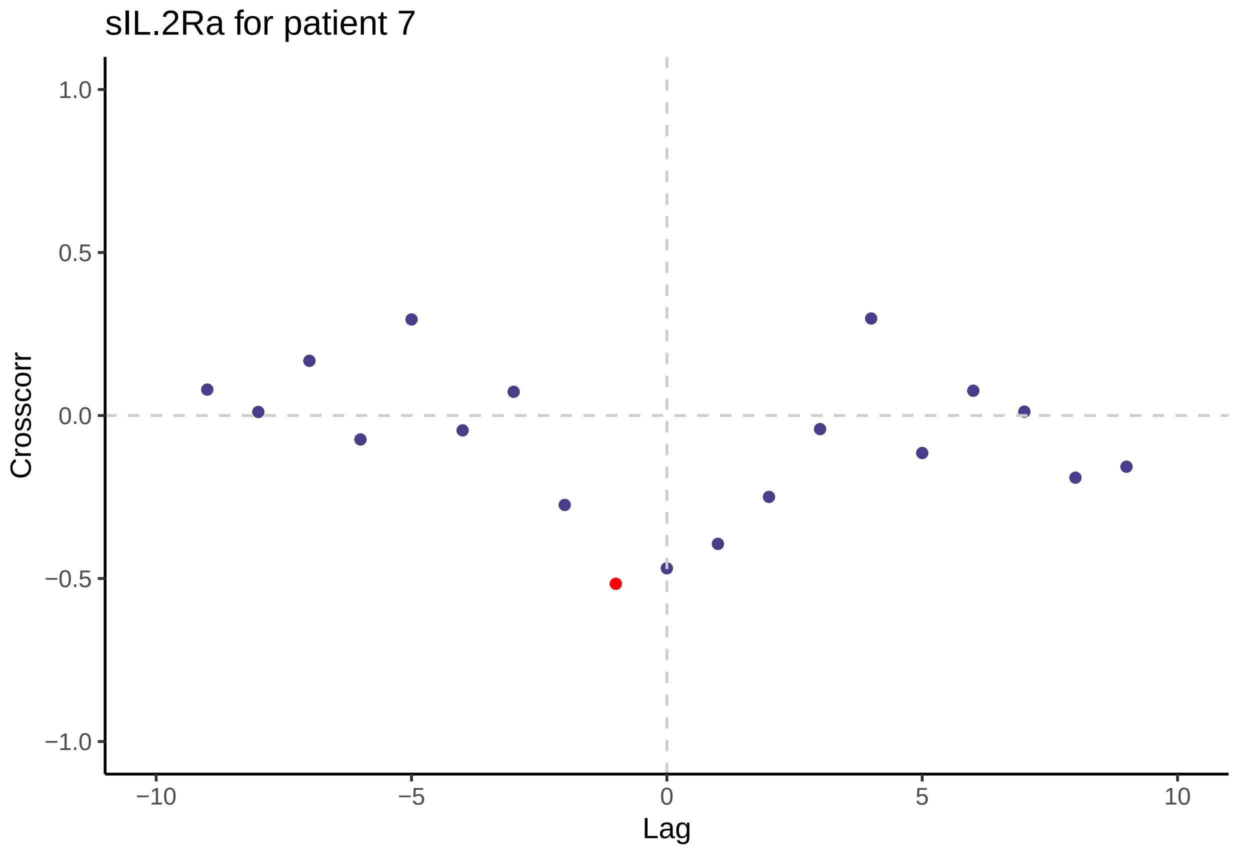


```
## [1] "sIL.2Ra for patient 7 - p-value: 0.150169255148928"
```

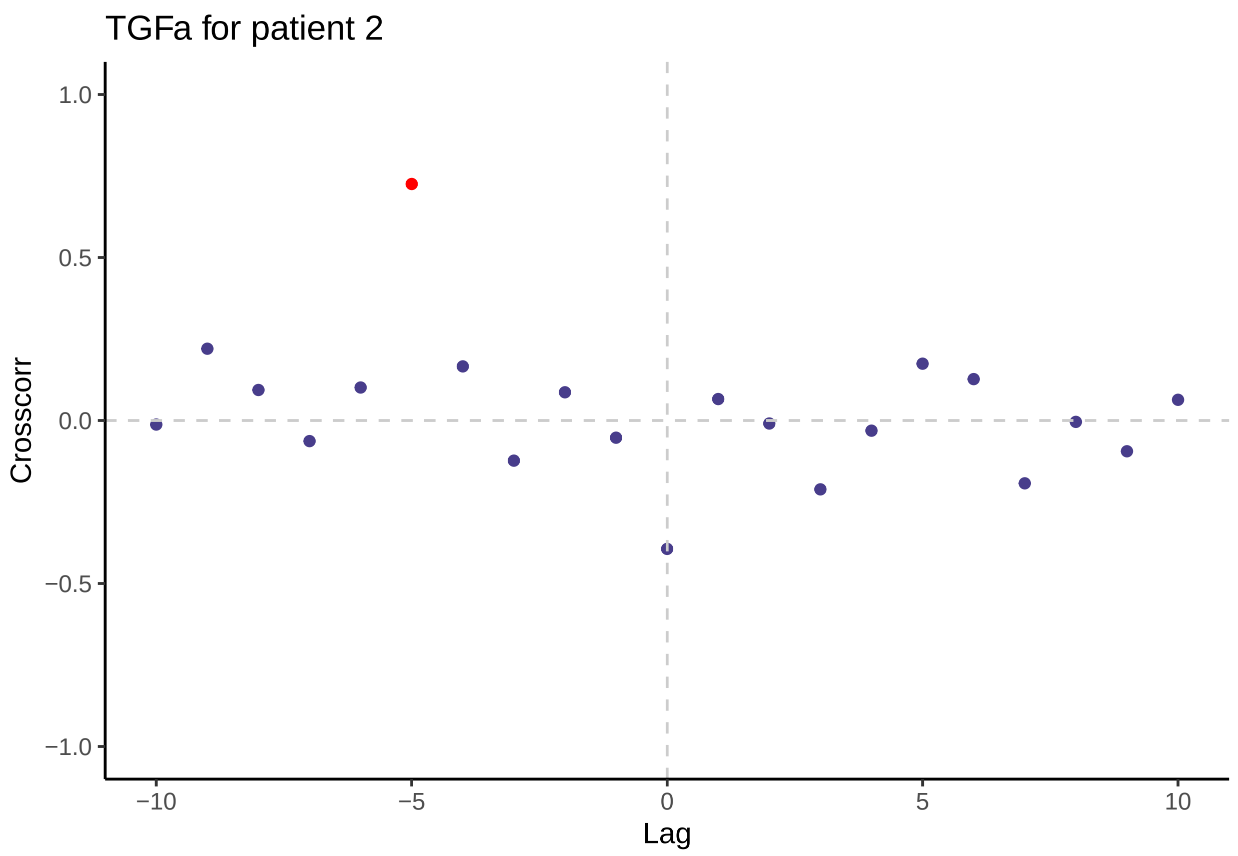


```
## [1] "TGFa for patient 2 - p-value: 0.528014215661487"
```

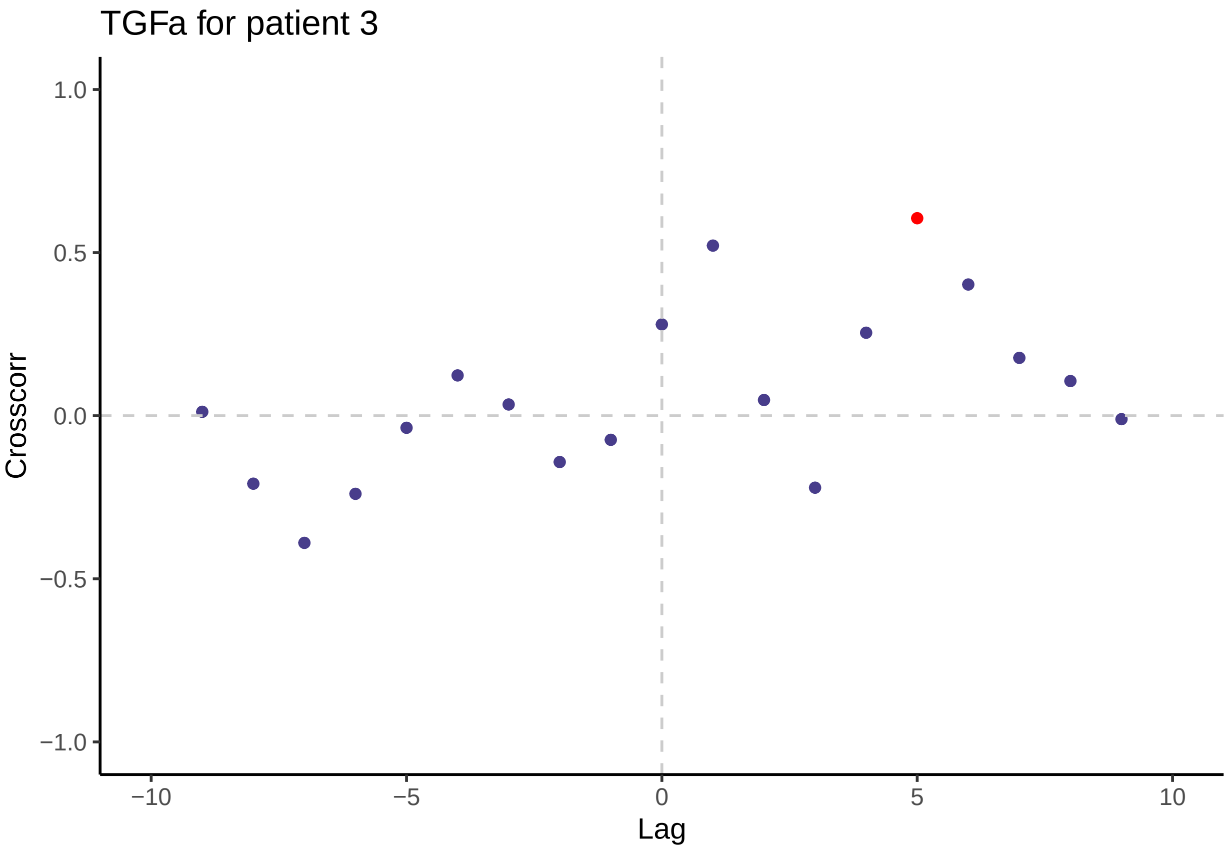


```
## [1] "TGFa for patient 3 - p-value: 0.292672907423354"
```

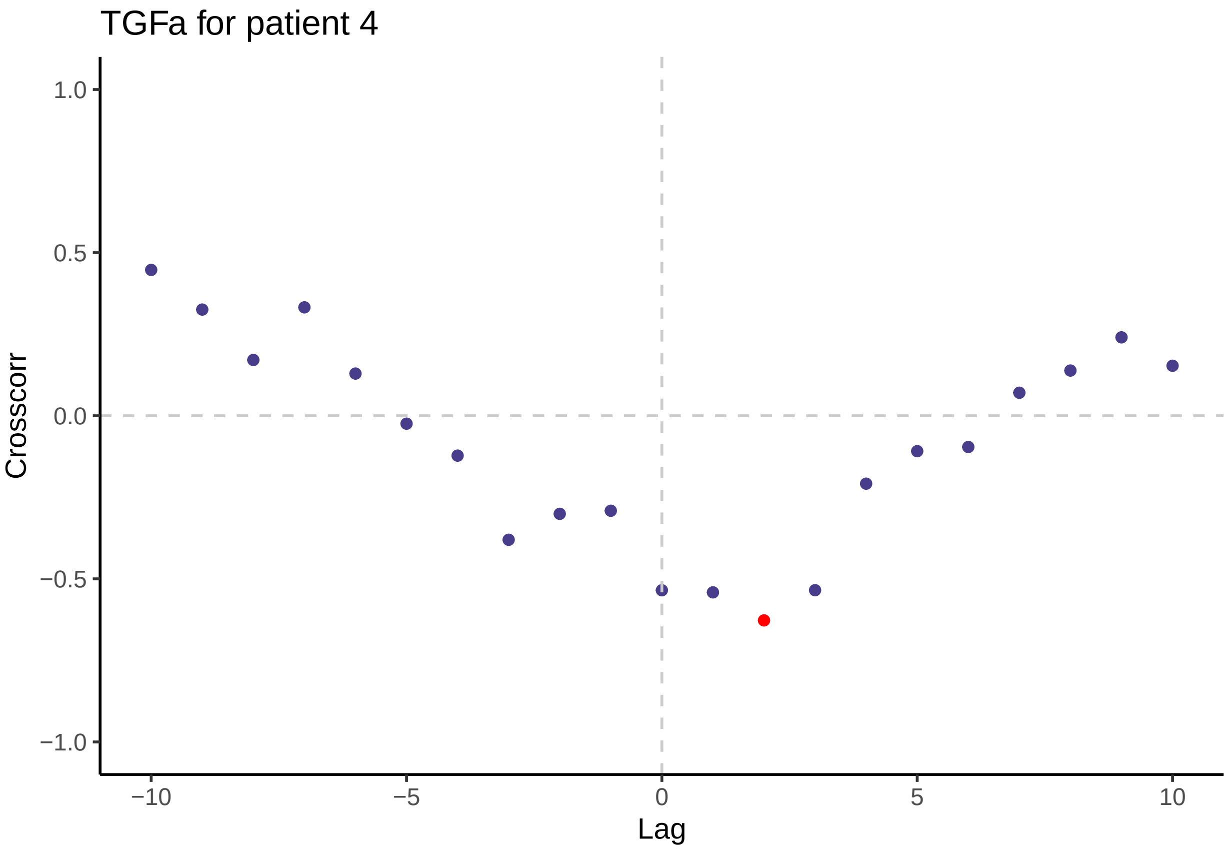


```
## [1] "TGFa for patient 4 - p-value: 0.247086624033394"
```

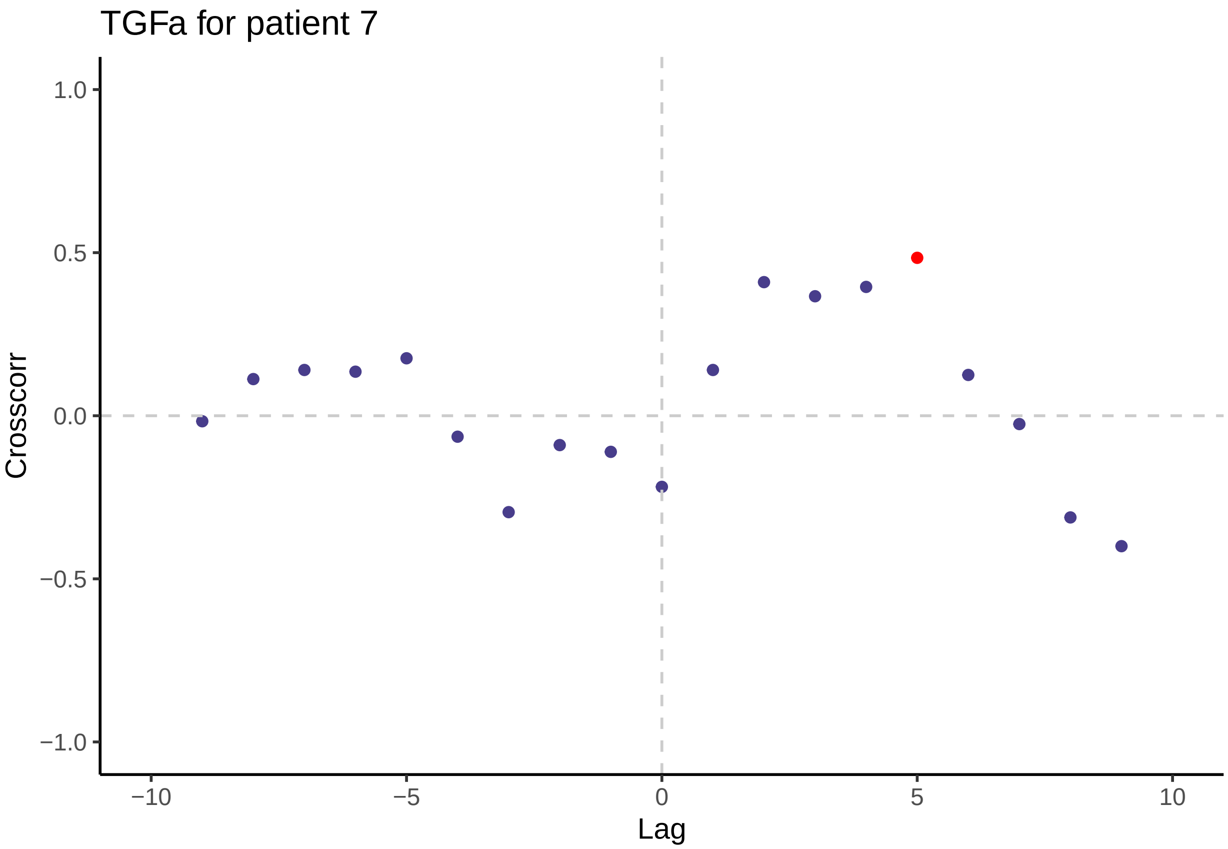


```
## [1] "TGFa for patient 7 - p-value: 0.404309543898263"
```

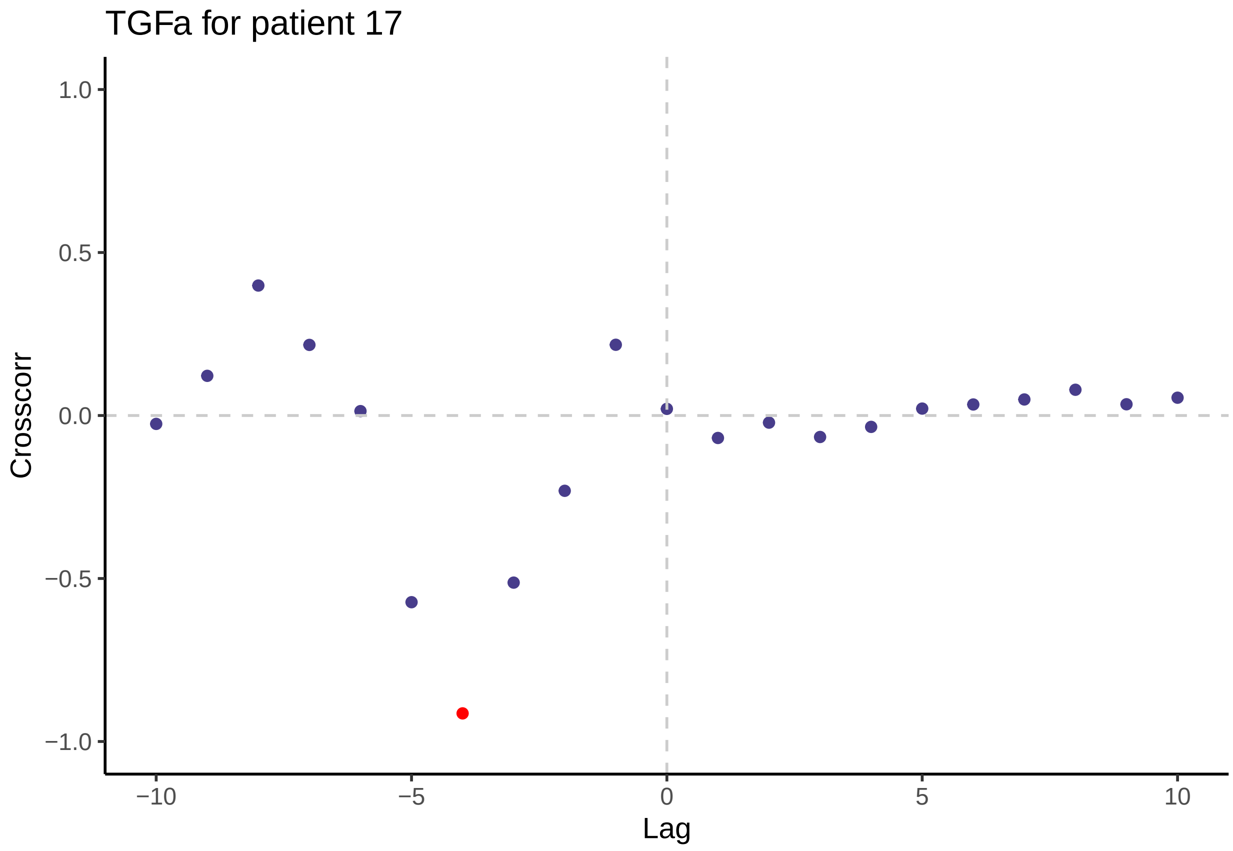


```
## [1] "TGFa for patient 17 - p-value: 0.385713402292932"
```

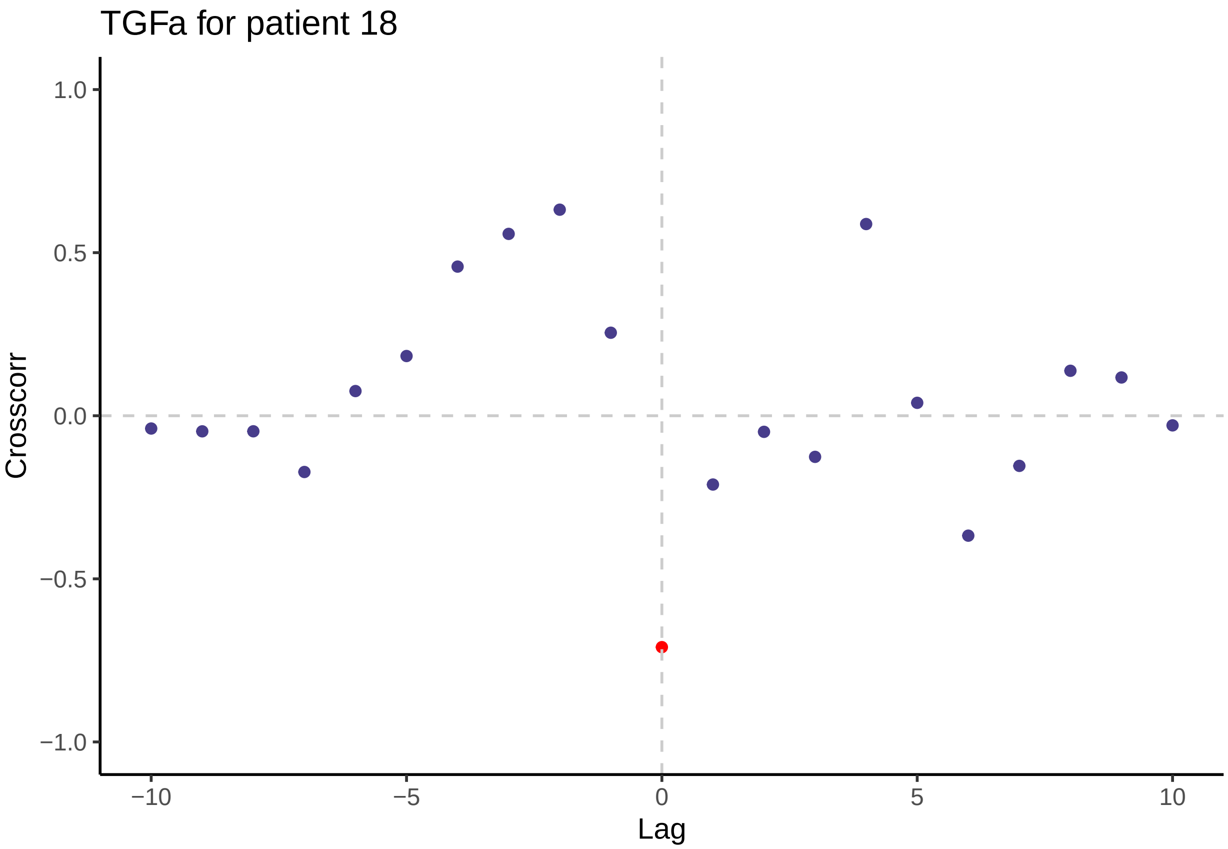


```
## [1] "TGFa for patient 18 - p-value: 0.472512682085531"
```

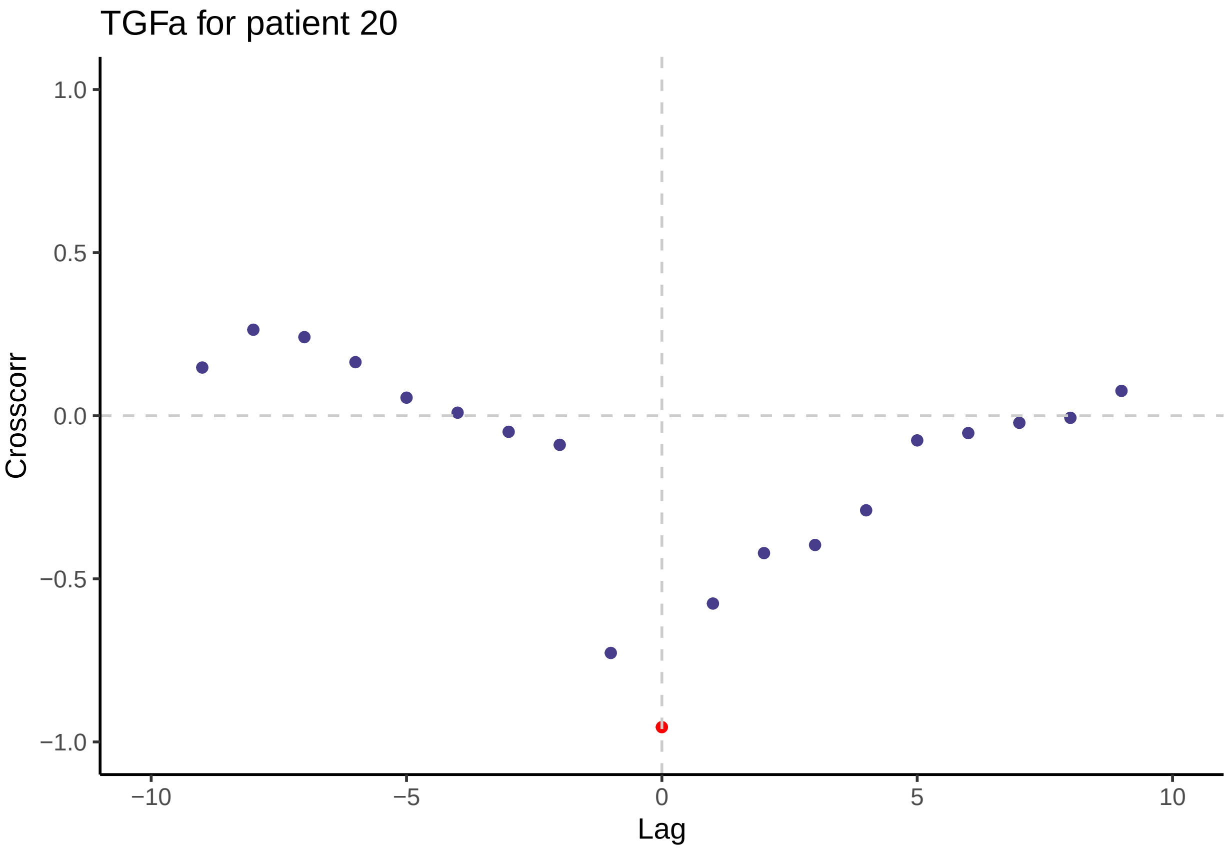


```
## [1] "TGFa for patient 20 - p-value: 0.0801430449618368"
```

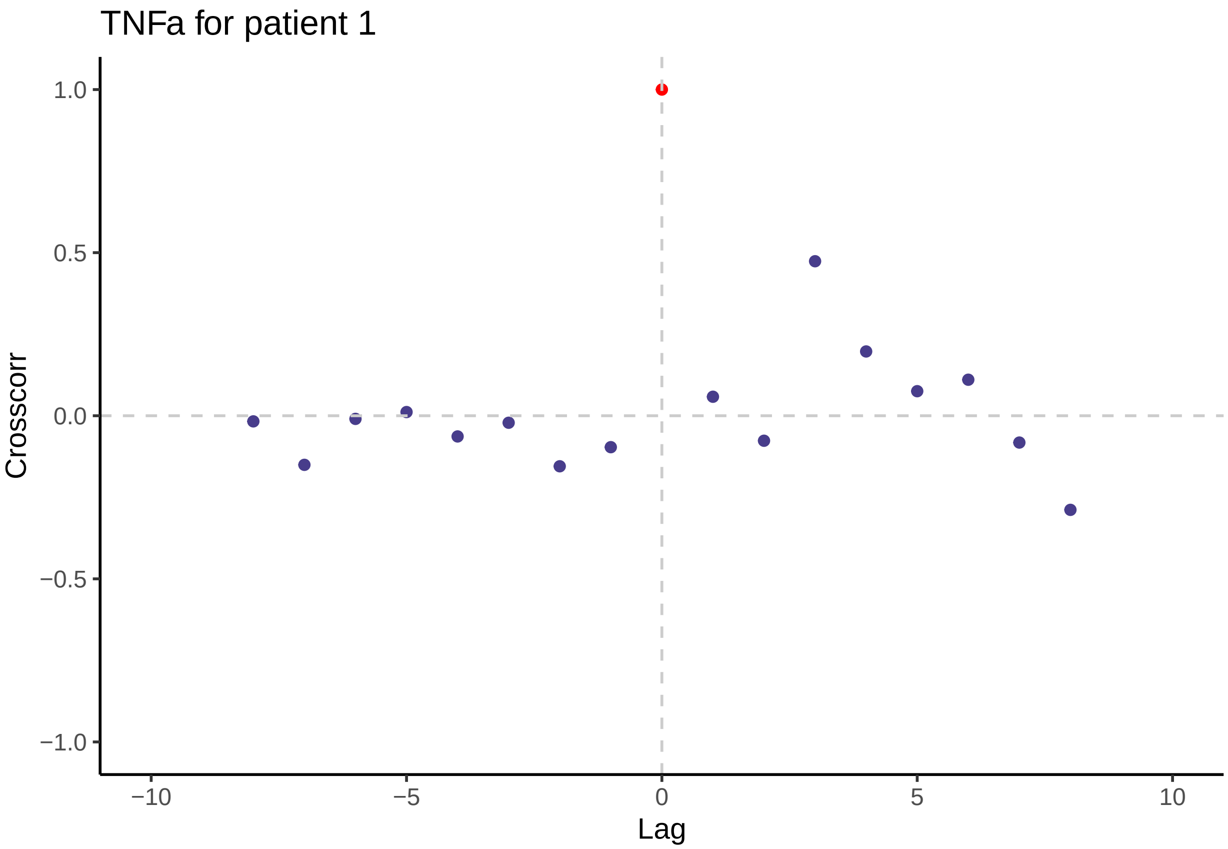


```
## [1] "TNFa for patient 1 - p-value: 0.437831450331867"
```

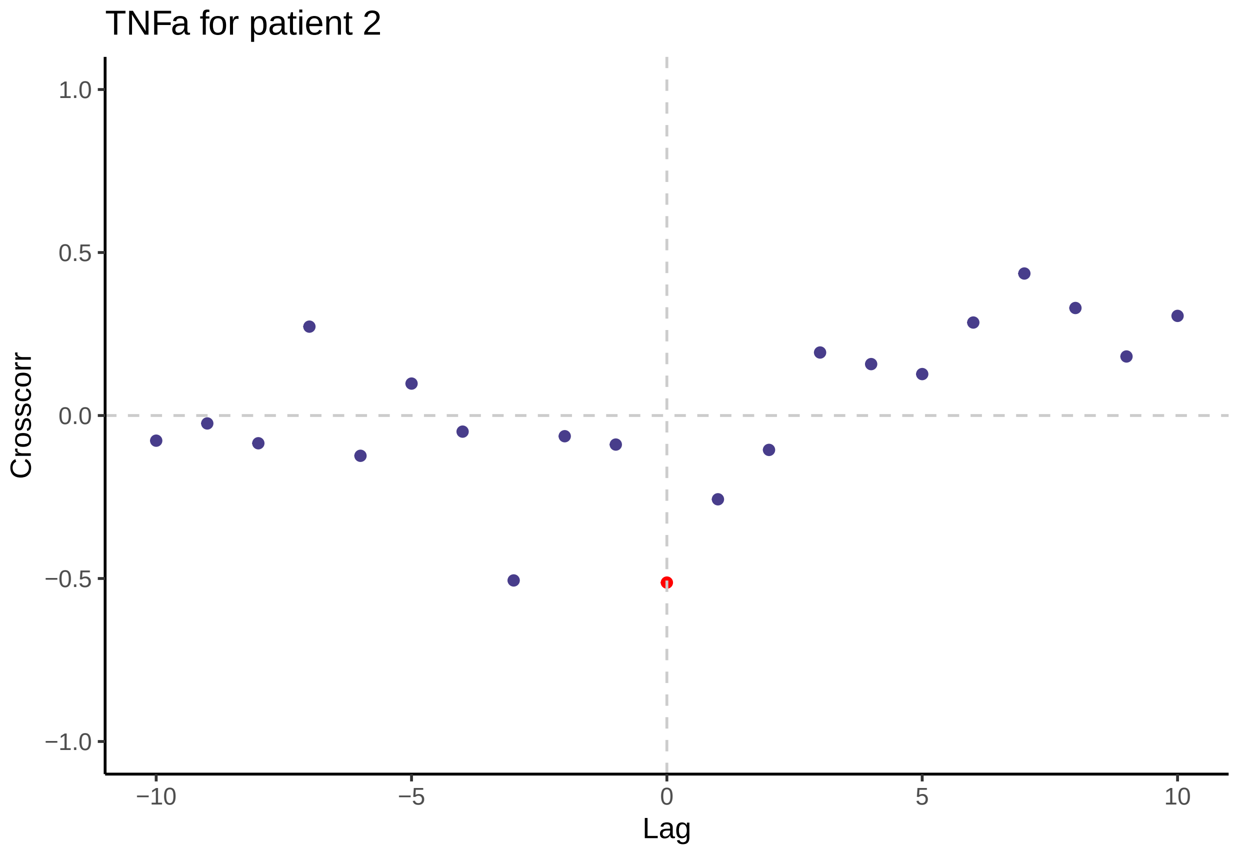


```
## [1] "TNFa for patient 2 - p-value: 0.677223249280793"
```

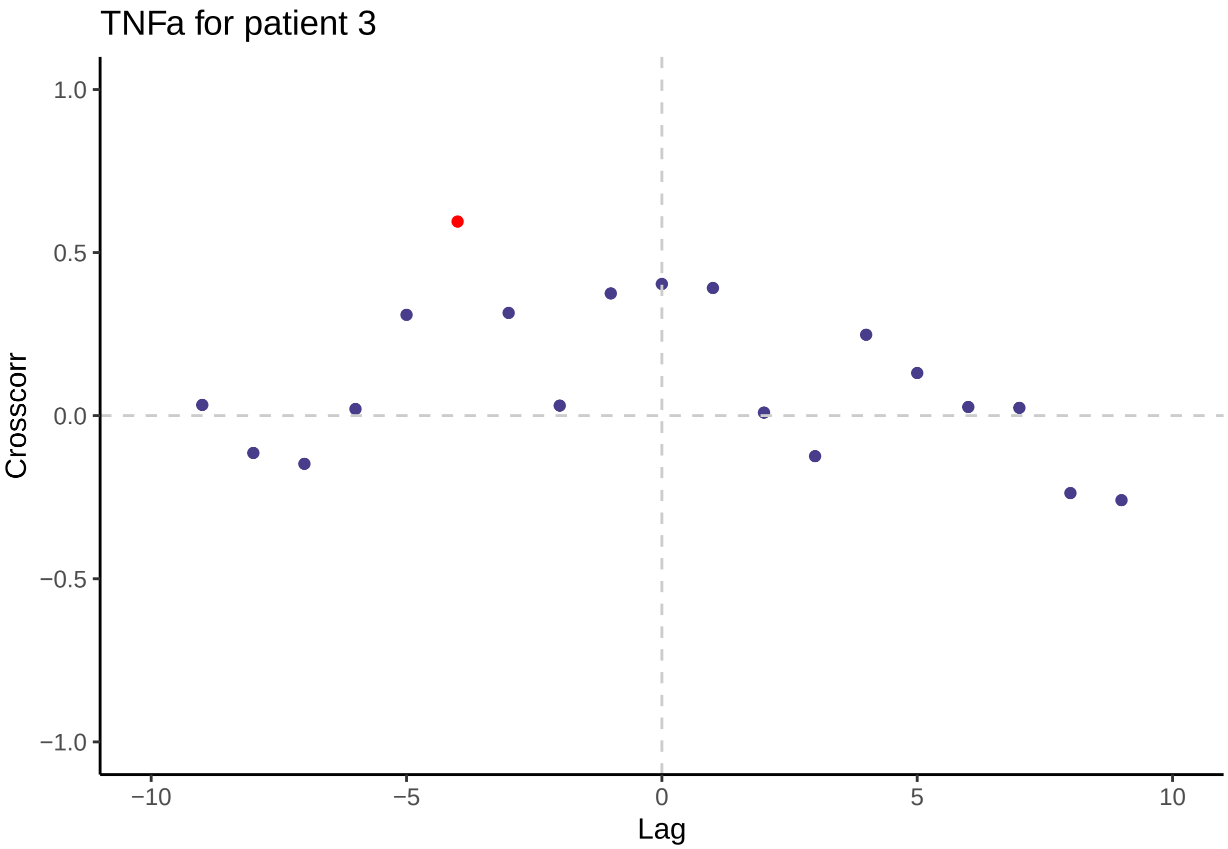


```
## [1] "TNFa for patient 3 - p-value: 0.0685564811171613"
```

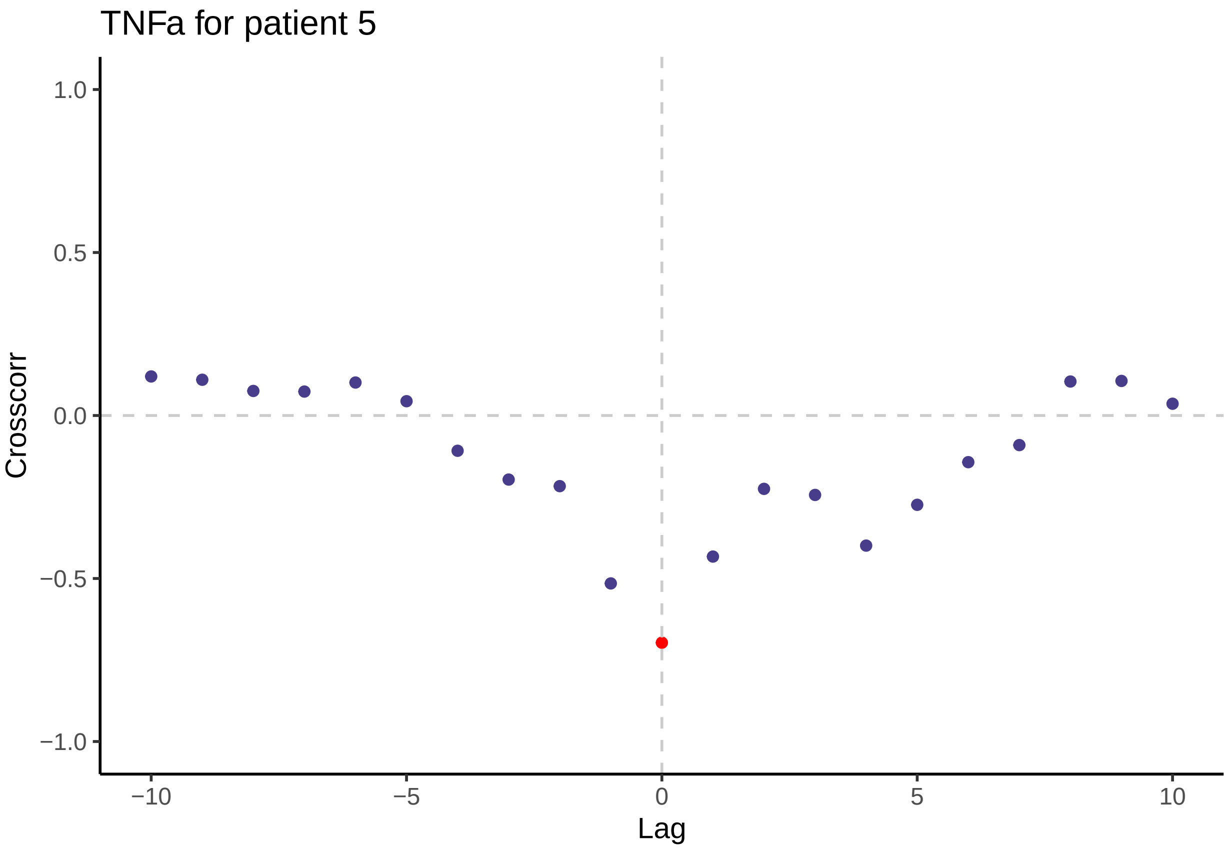


```
## [1] "TNFa for patient 5 - p-value: 0.0186348341556511"
```

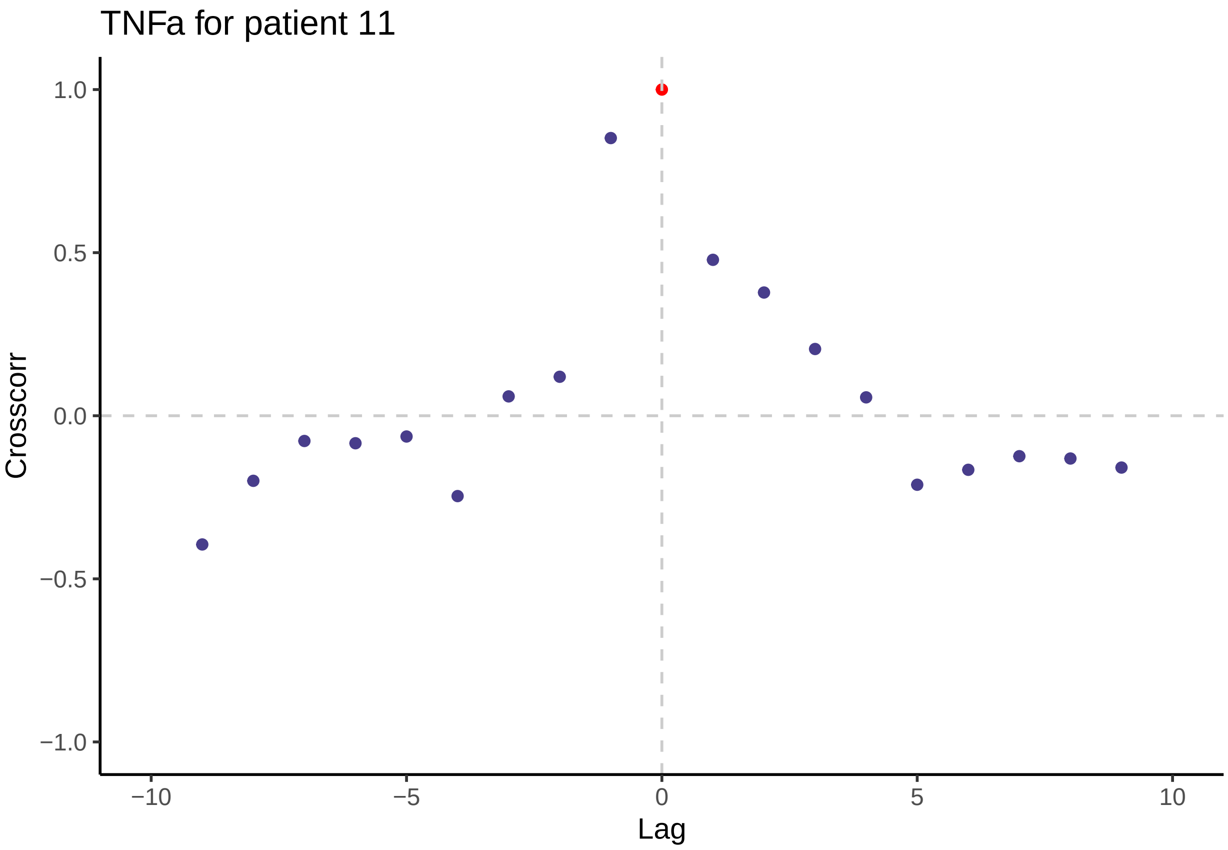


```
## [1] "TNFa for patient 11 - p-value: 0.435019274091684"
```

```
## Warning: Removed 3 rows containing missing values (geom_point).
```

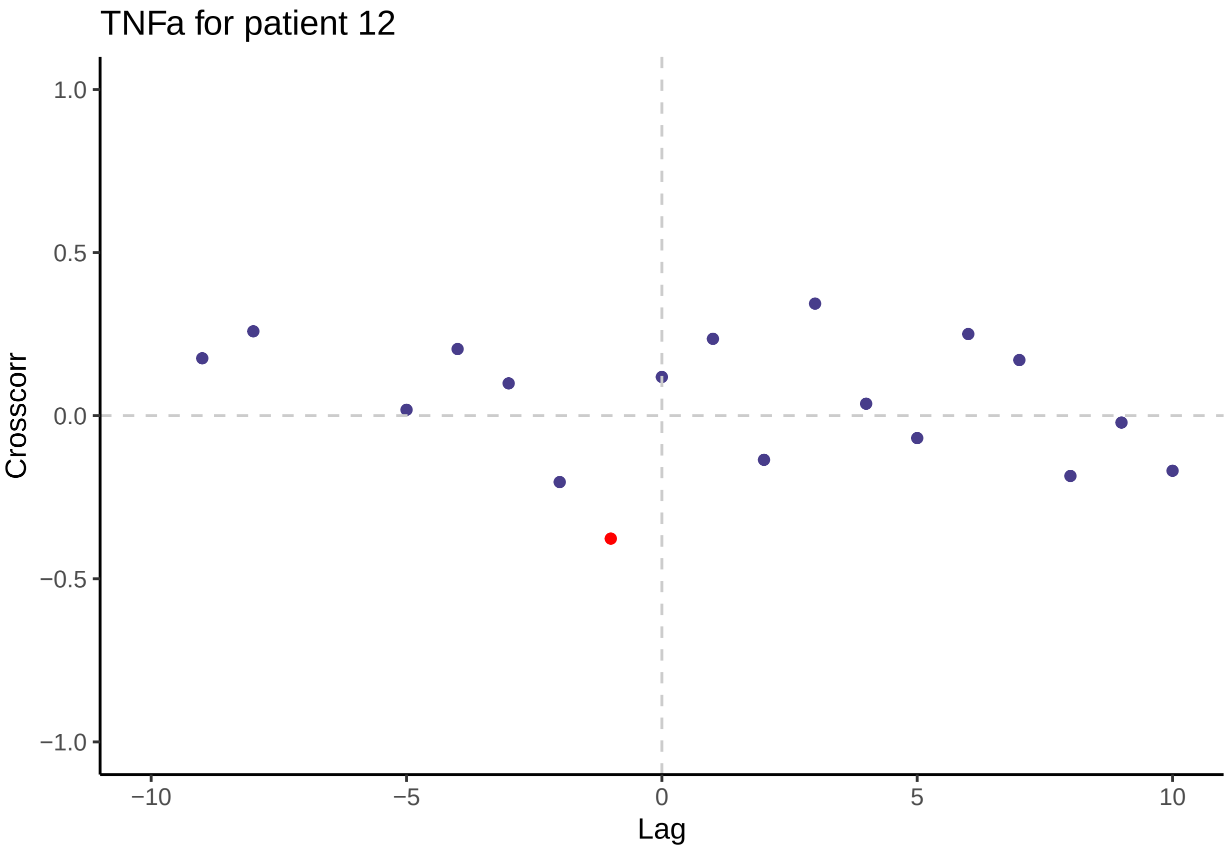


```
## [1] "TNFa for patient 12 - p-value: 0.381201053611328"
```

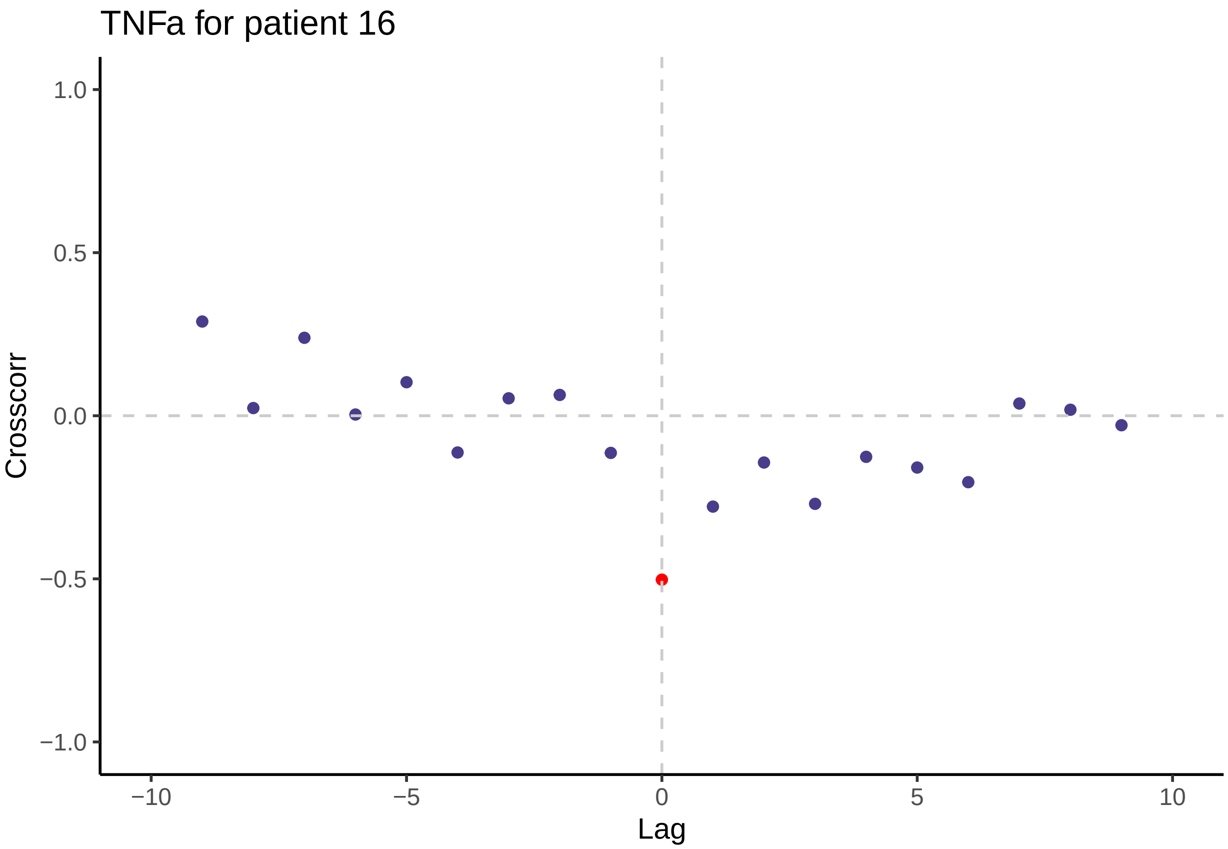


```
## [1] "TNFa for patient 16 - p-value: 0.190927834999029"
```

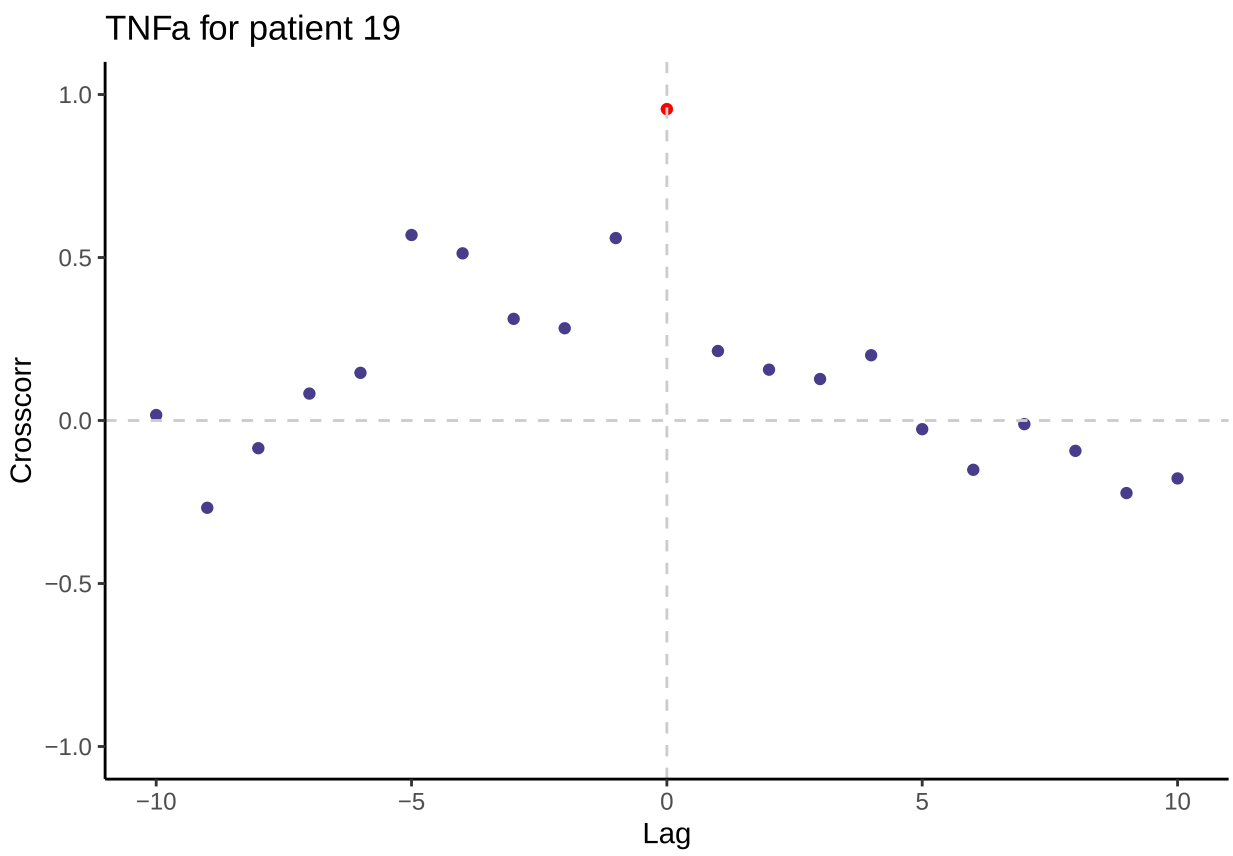


```
## [1] "TNFa for patient 19 - p-value: 0.0392474937084566"
```

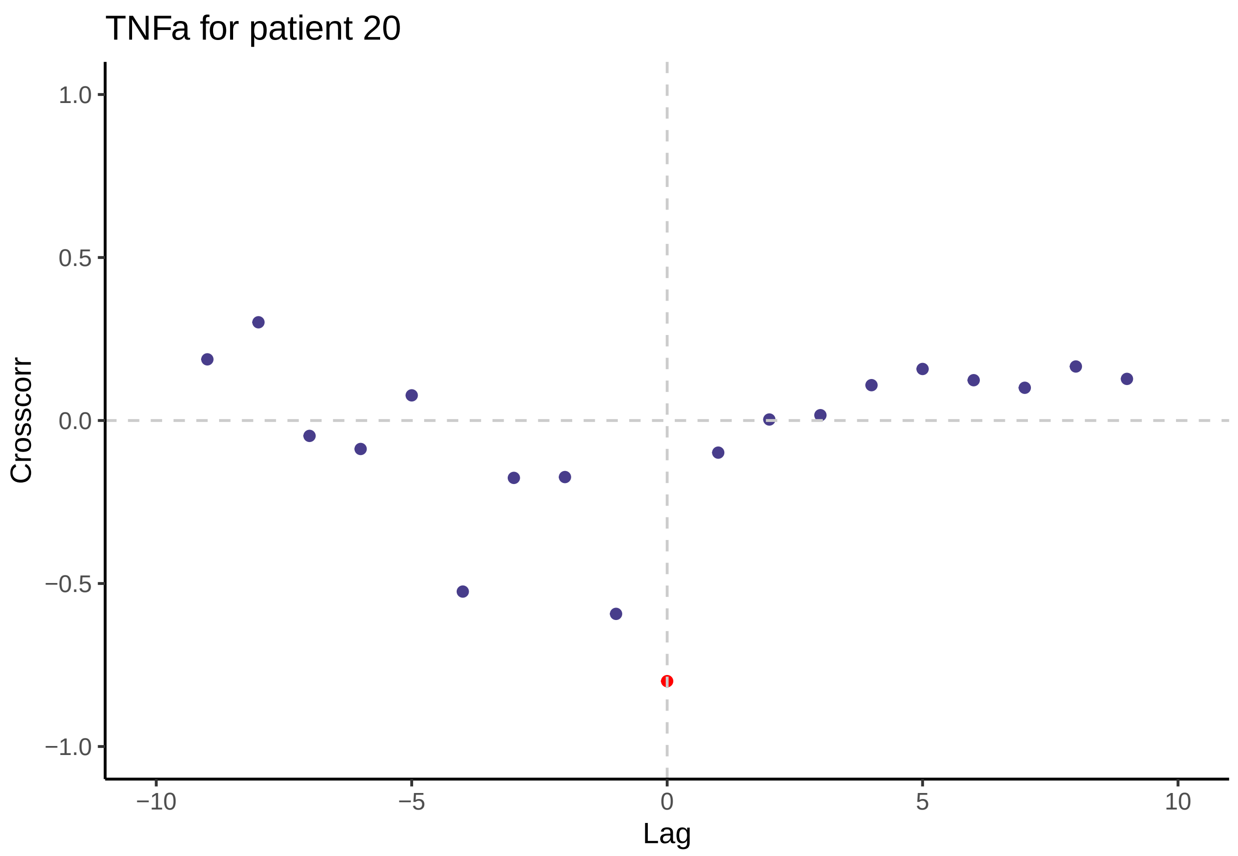


```
## [1] "TNFa for patient 20 - p-value: 0.383766361983102"
```

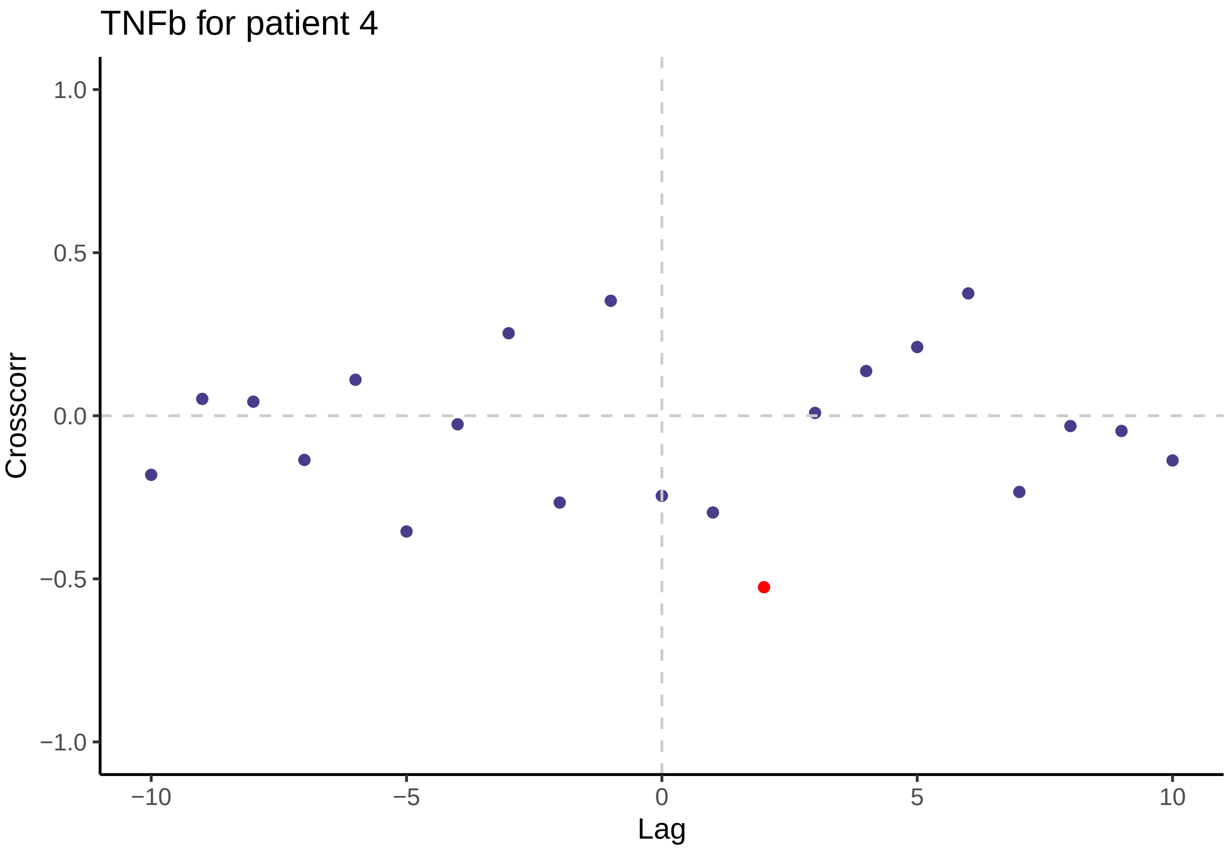


```
## [1] "TNFb for patient 4 - p-value: 0.396653081953921"
```

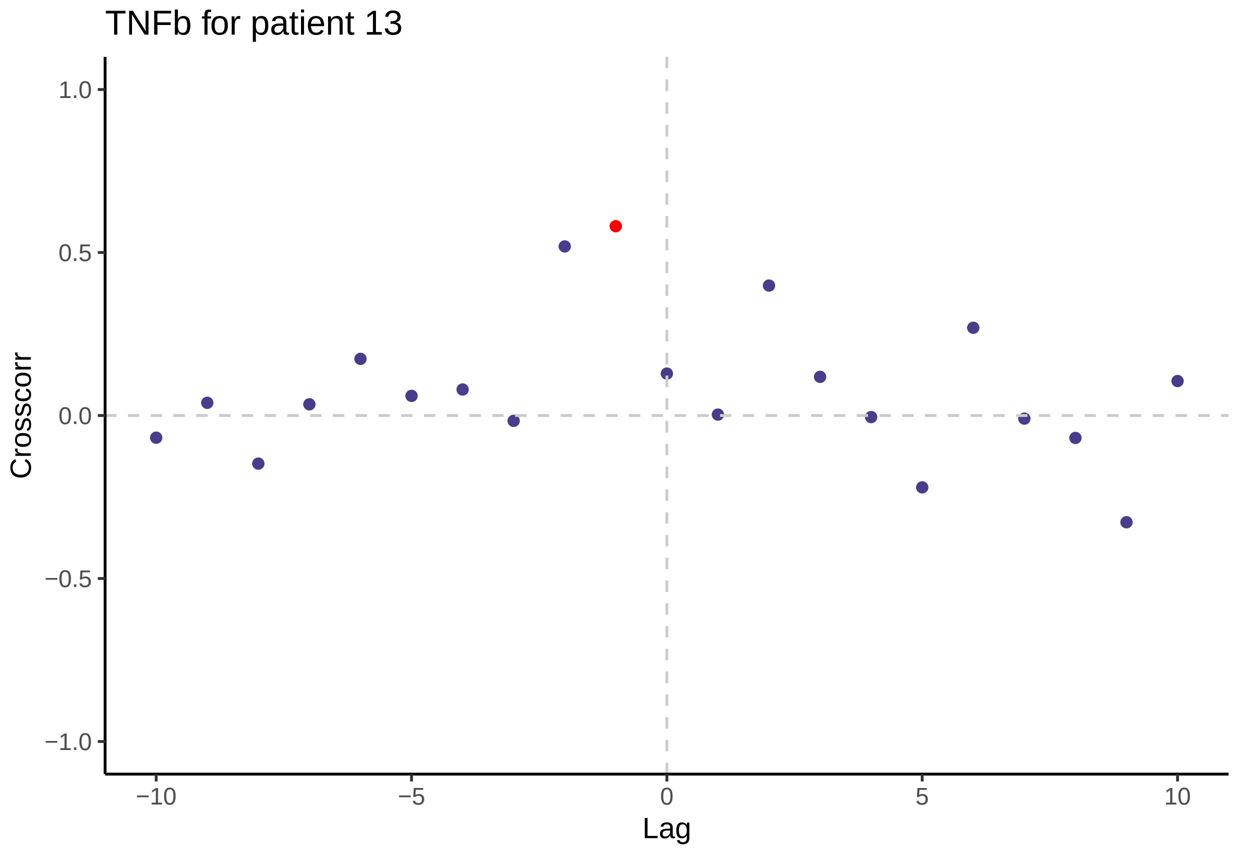


```
## [1] "TNFb for patient 13 - p-value: 0.120059144113981"
```

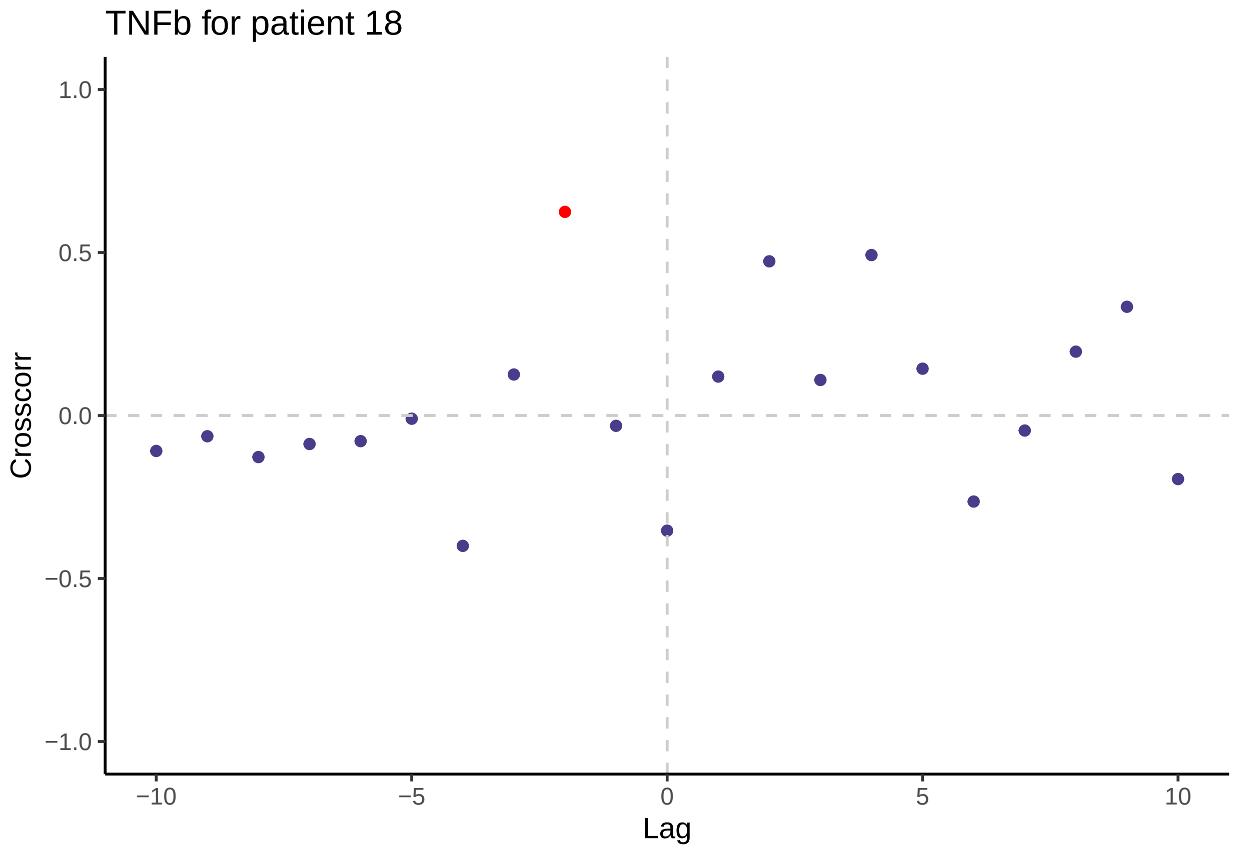


```
## [1] "TNFb for patient 18 - p-value: 0.501698548104019"
## Warning: Removed 2 rows containing missing values (geom_point).
```

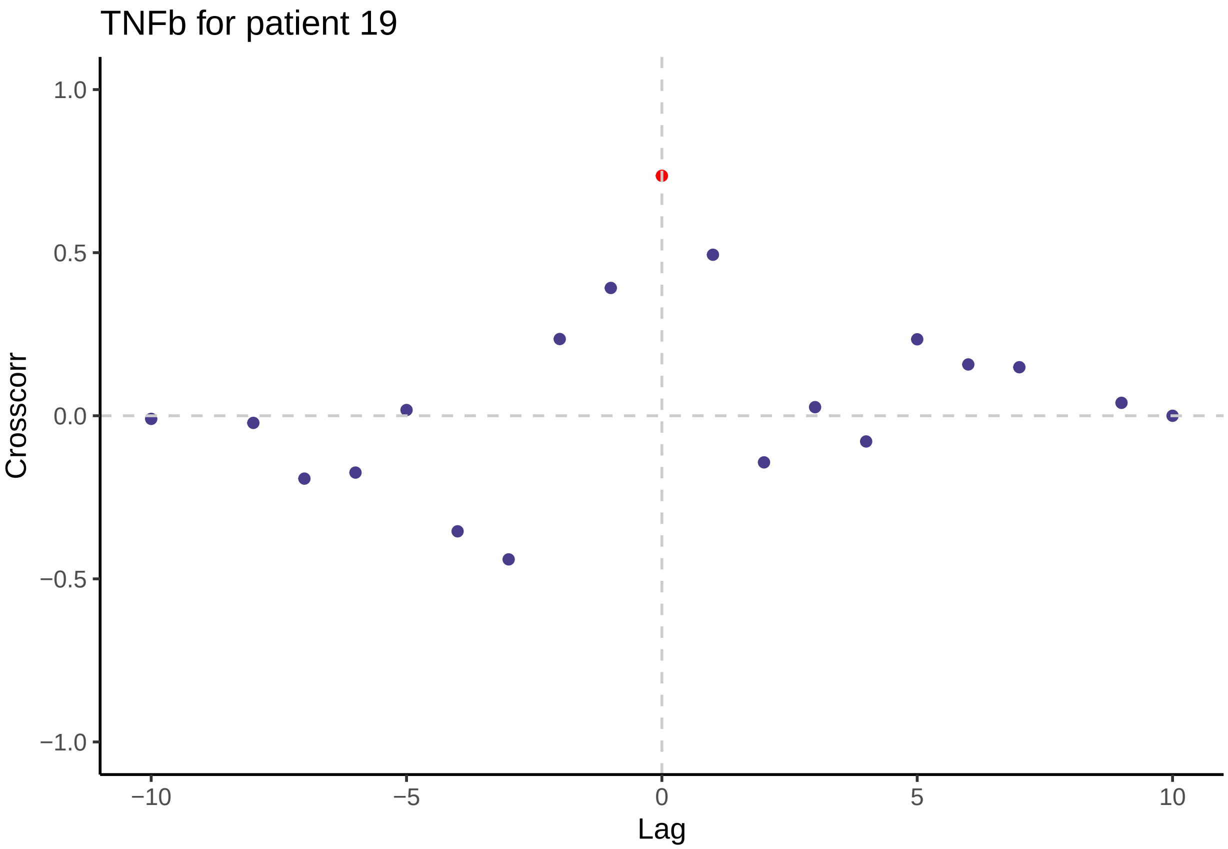


```
## [1] "TNFb for patient 19 - p-value: 0.400619041046448"
```

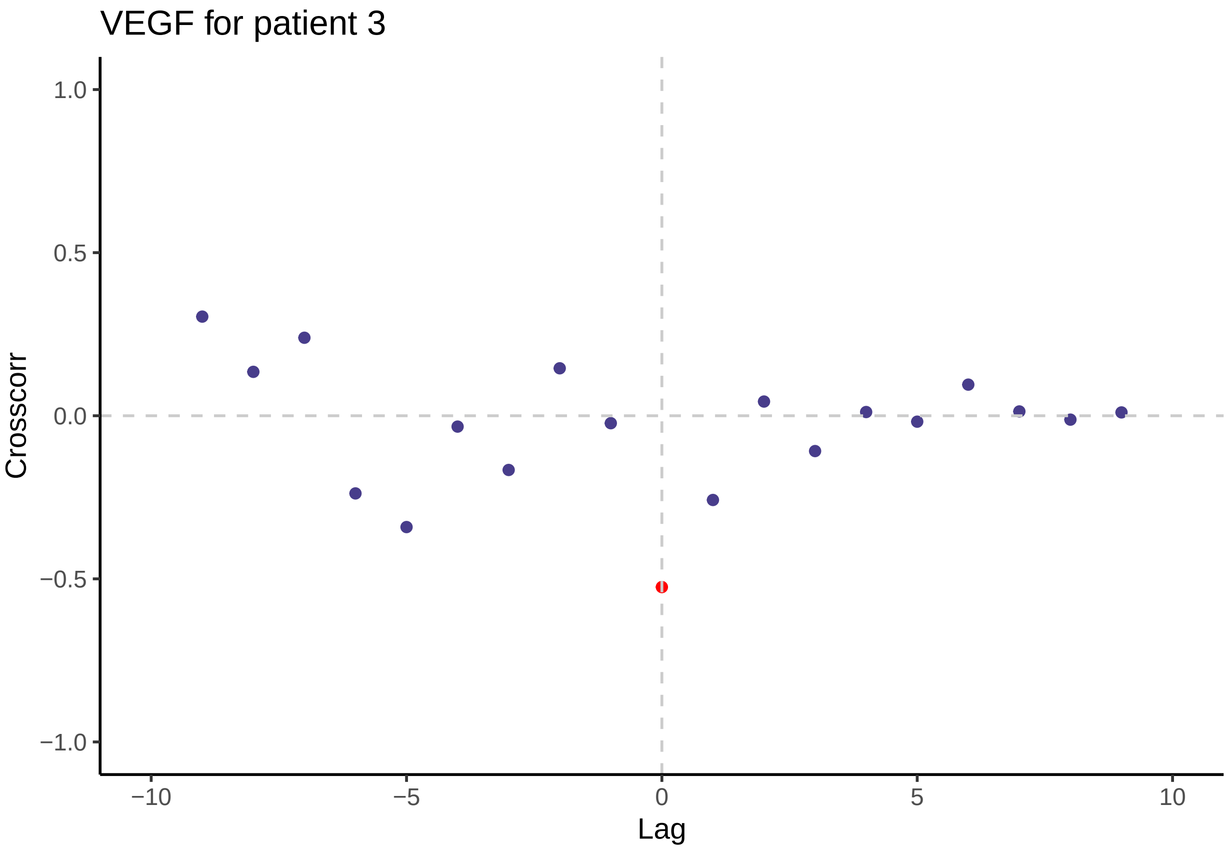


```
## [1] "VEGF for patient 3 - p-value: 0.41882384271283"
```

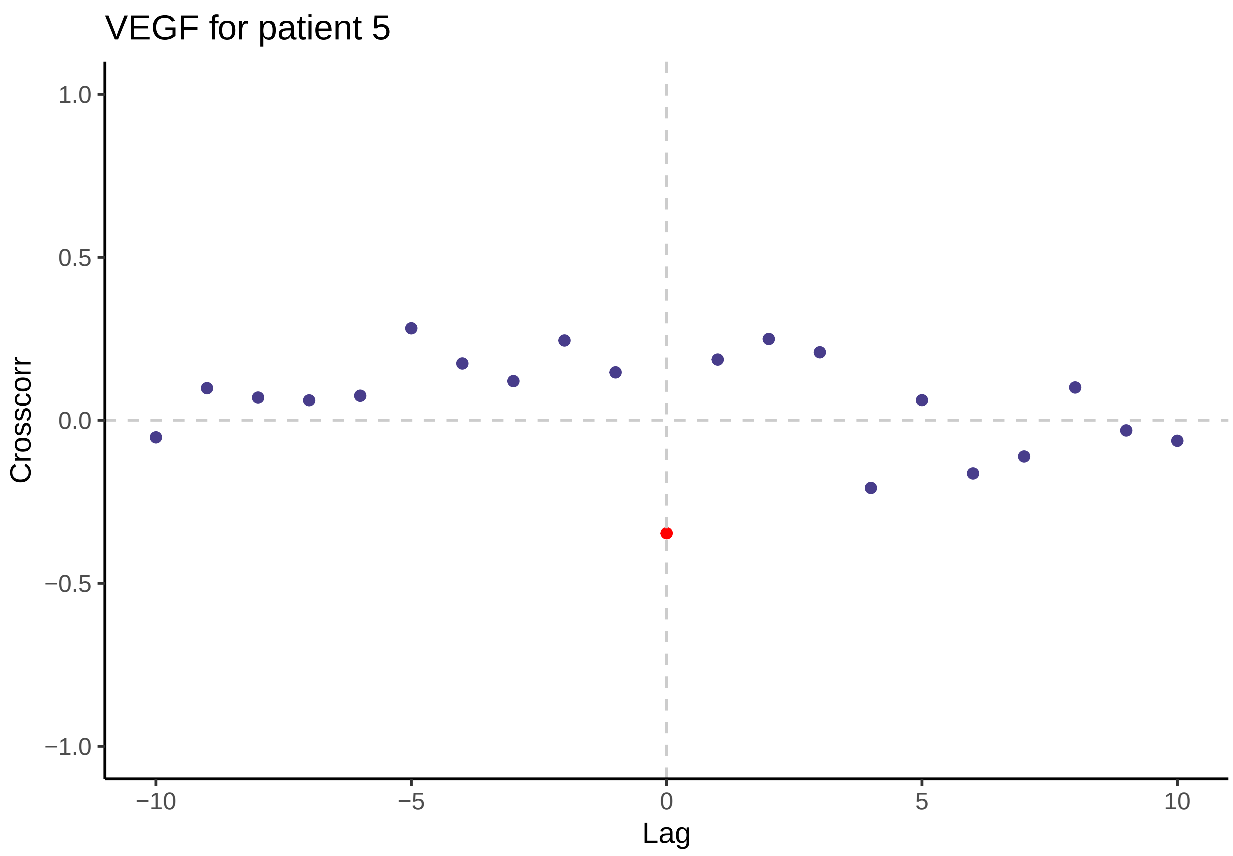


```
## [1] "VEGF for patient 5 - p-value: 0.156570648853007"
```

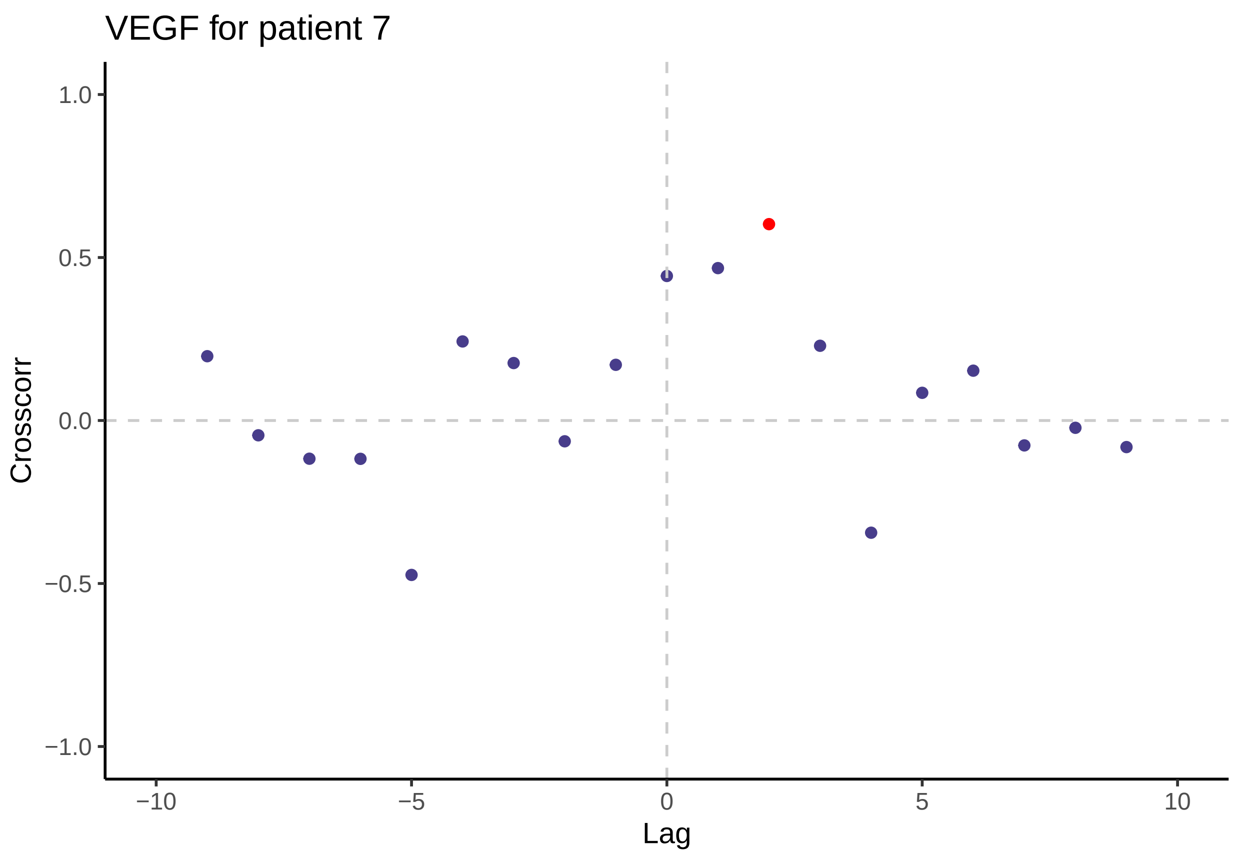


```
## [1] "VEGF for patient 7 - p-value: 0.239727241576102"
```

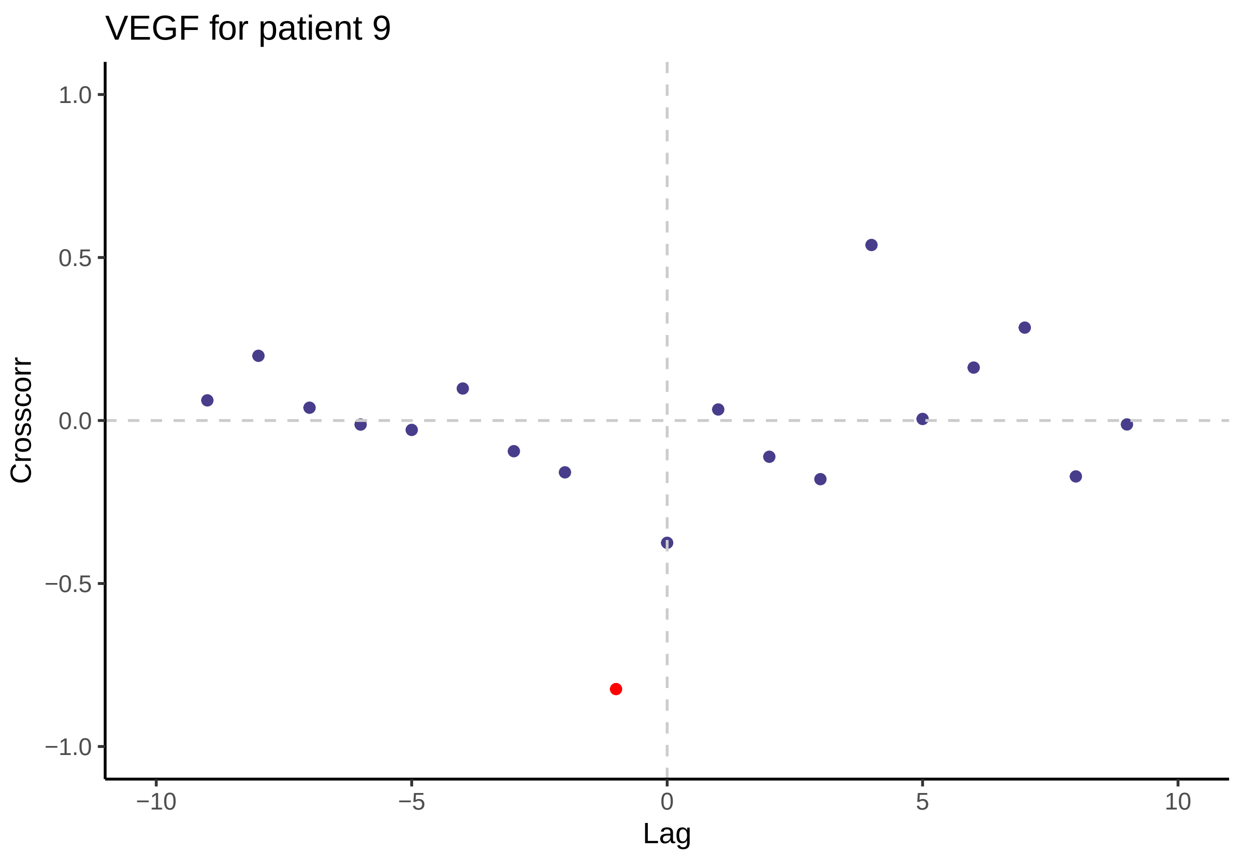


```
## [1] "VEGF for patient 9 - p-value: 0.654834793469433"
```

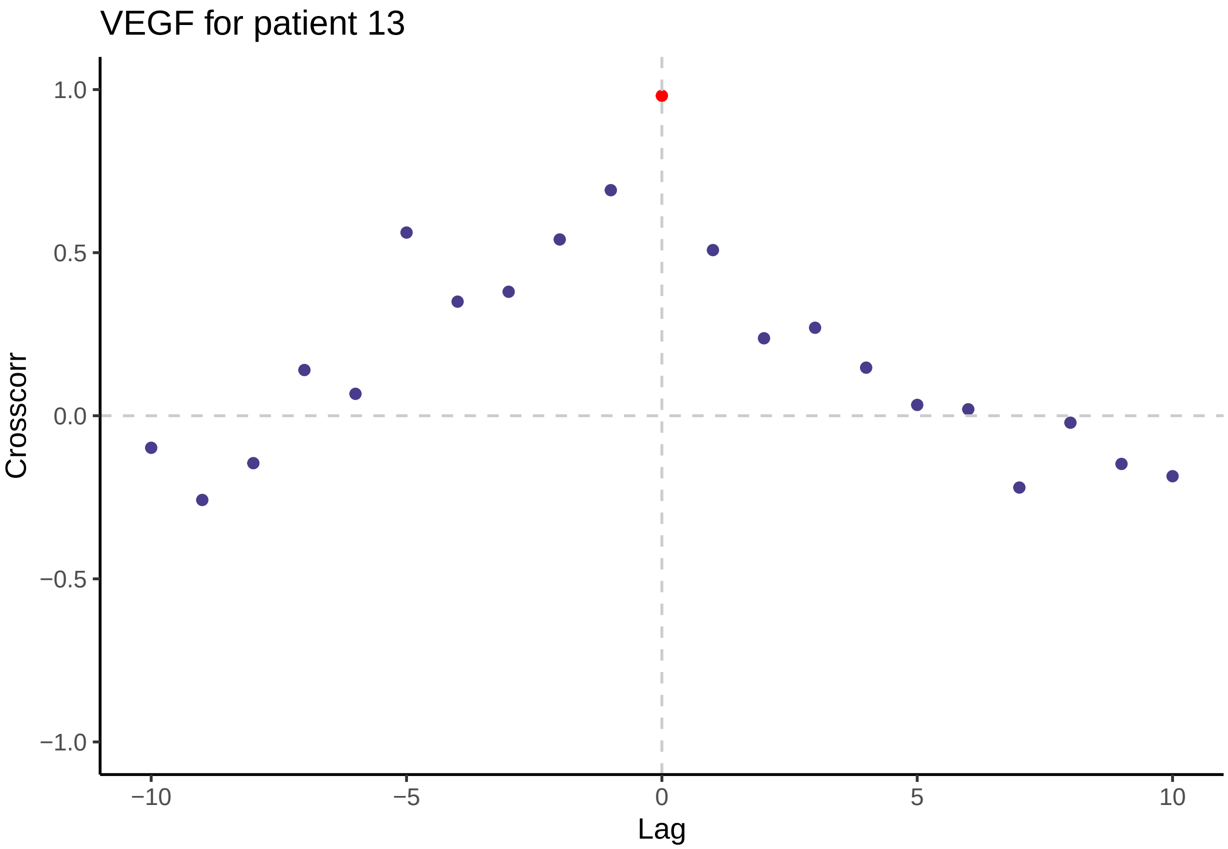


```
## [1] "VEGF for patient 13 - p-value: 0.0208538548799299"
```

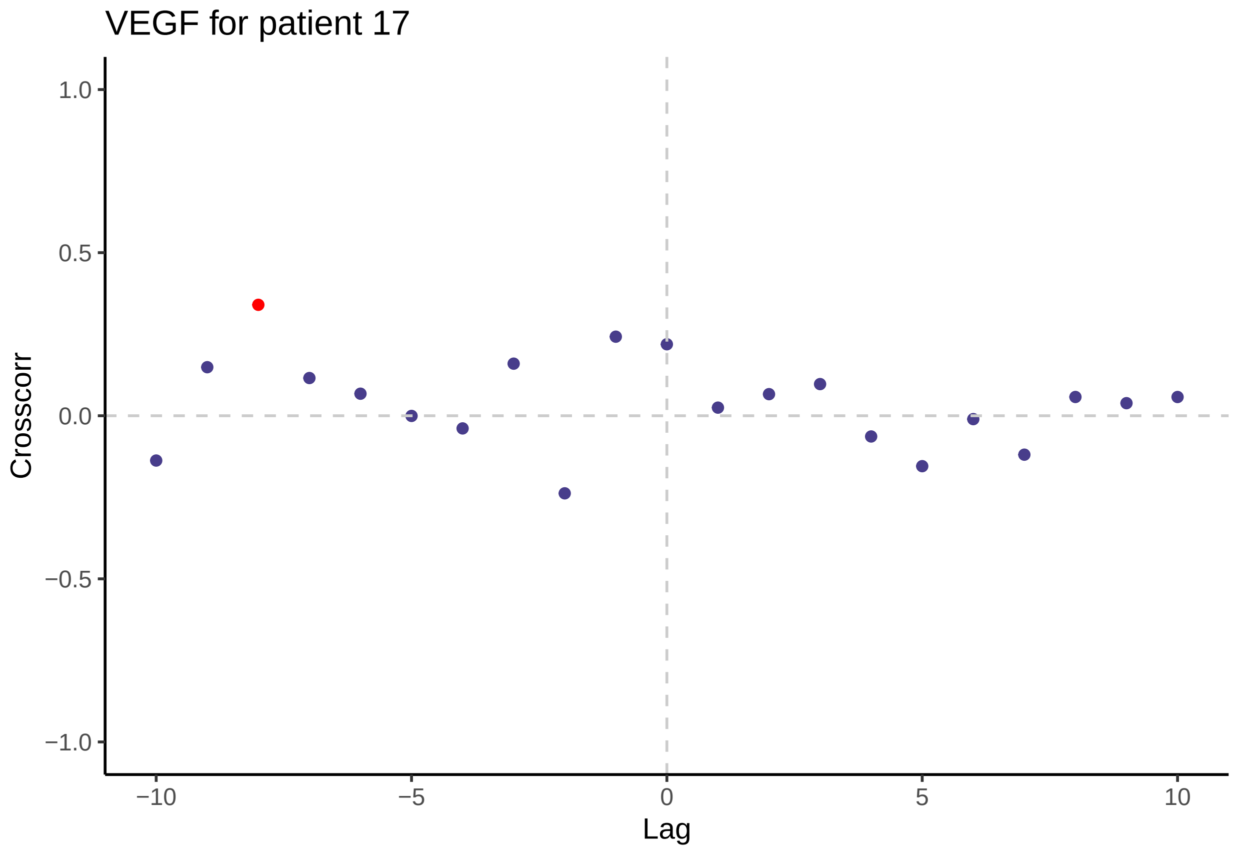


```
## [1] "VEGF for patient 17 - p-value: 0.189416381218946"
```

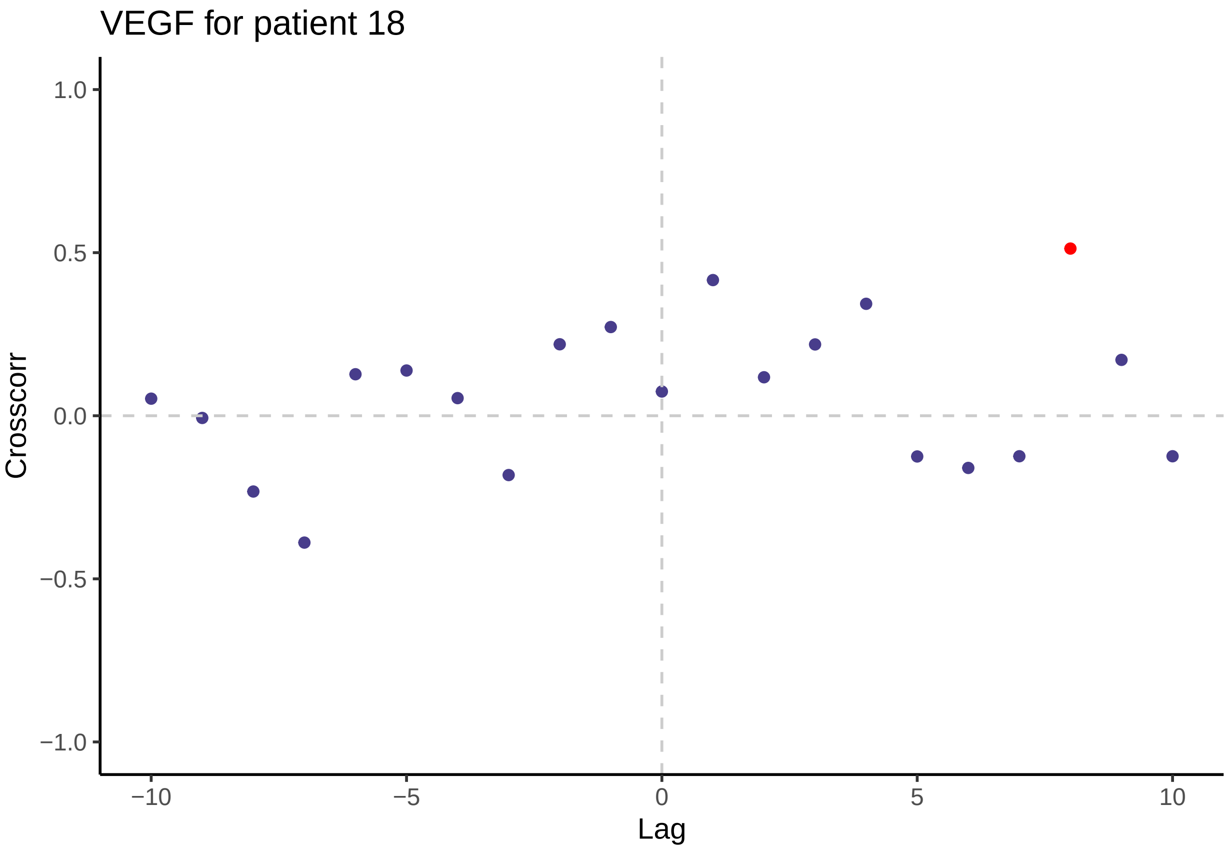


```
## [1] "VEGF for patient 18 - p-value: 0.201764911265744"
```

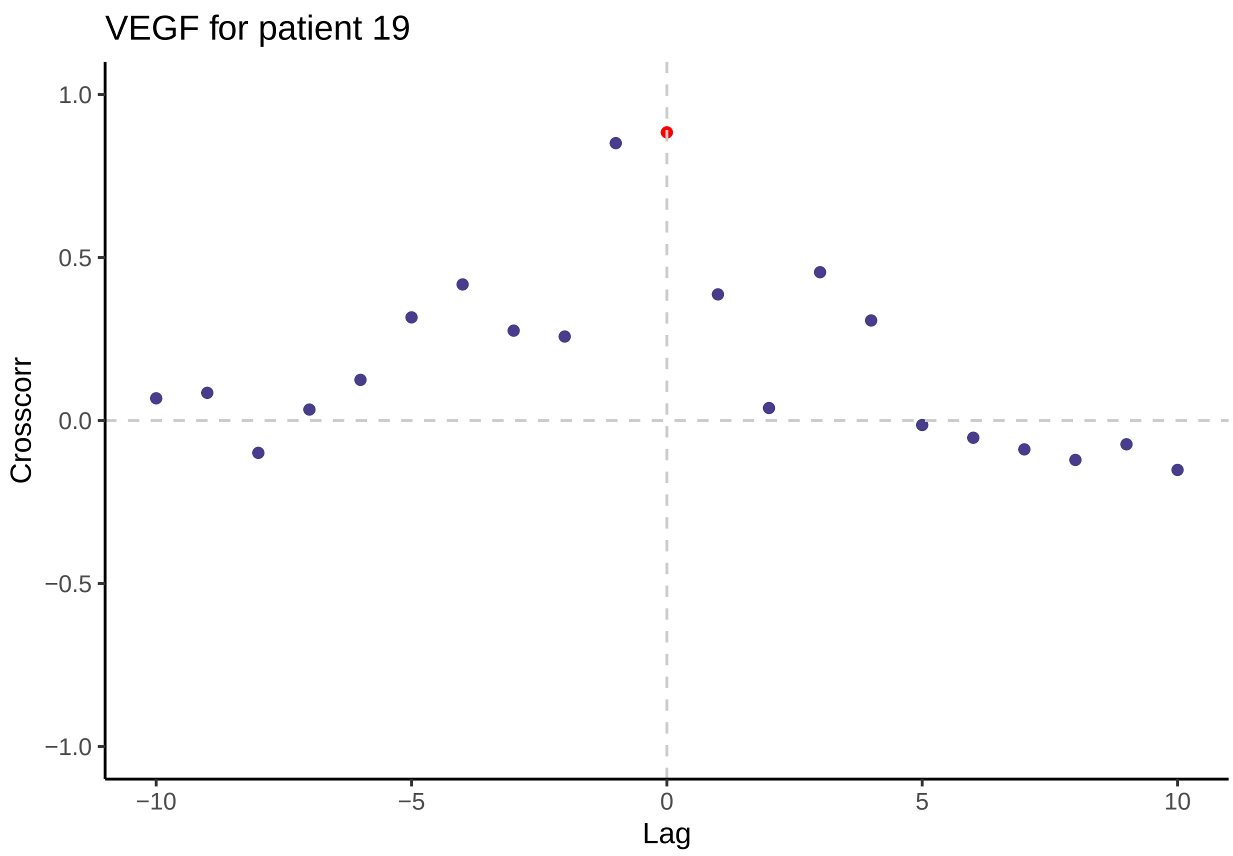


```
## [1] "VEGF for patient 19 - p-value: 0.00917959762127627"
```

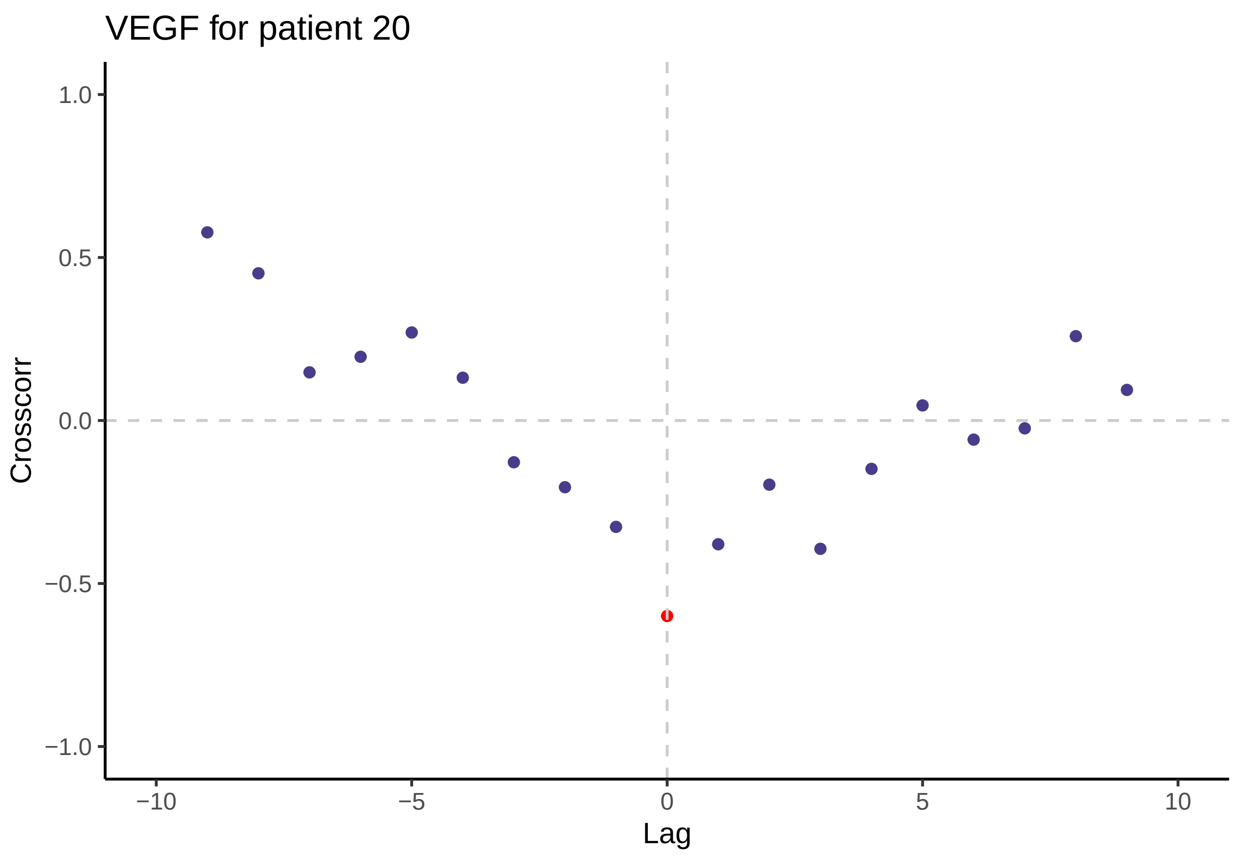


```
## [1] "VEGF for patient 20 - p-value: 0.82994477584649"
```

```
write.xlsx(output, "crosstable200906.xlsx")

output_compact <-
  gather(data.frame(output_compact),
         Reason_for_exclusion, Frequency, 2:(length(reasons)+1))
output_compact$Cytokine <-
  gsub("[.]", "-", output_compact$Cytokine)
output_compact$Reason_for_exclusion <-
  gsub("[.]", " ", output_compact$Reason_for_exclusion)
write.xlsx(output_compact, "crosstable_compact200906.xlsx")

output_compact$Cytokine <- factor(output_compact$Cytokine,
                                  levels = rev(output_compact$Cytokine[1:42]))

p2 <- ggplot(data = output_compact, aes(fill = Reason_for_exclusion,
                                        x = Cytokine, y = Frequency)) +
  geom_bar(position = "stack", stat = "identity") +
  coord_flip() +
  theme_classic()
p2$labels$fill <- gsub("_", " ", p2$labels$fill)
print(p2)
```

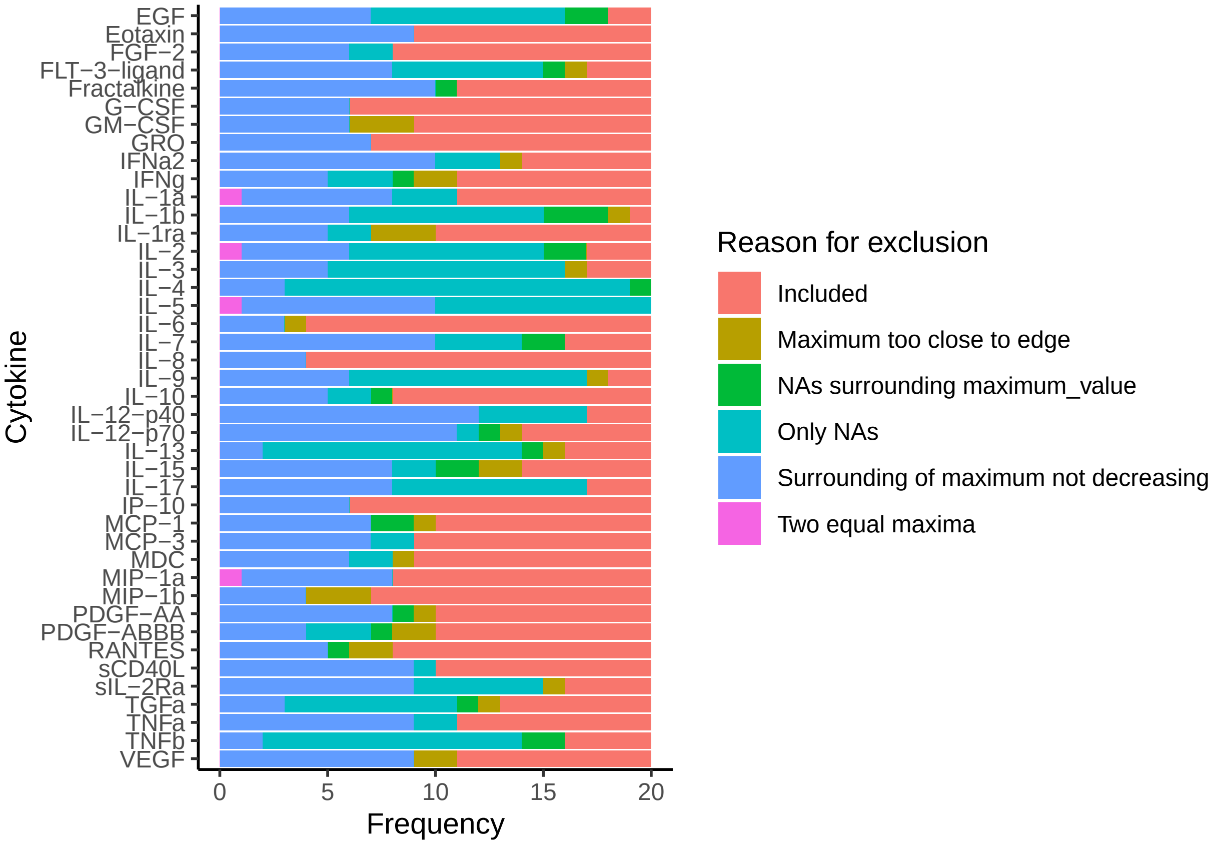


```
ggsave("crossexcluded200906.pdf",
       plot = p2,
       device = "pdf",
       width = 8,
       height = 8,
       units = "in")
```

Plot relevant ccfs

```
meansdall <- data.frame(lagmean = mean(output$Lag, na.rm = TRUE),
                        ccmean = mean(output$Maximum_value, na.rm = TRUE),
                        lagsd = sd(output$Lag, na.rm = TRUE),
                        ccsd = sd(output$Maximum_value, na.rm = TRUE))
p3a <- ggplot(data = output, aes(x = Lag, y = Maximum_value)) +
  geom_point() +
  # geom_point(data = meansdall, aes(x = lagmean, y = ccmean,
  #                                  shape = "All", size = "All")) +
  scale_shape_manual(values = 1, guide = FALSE) +
  scale_size_manual(values = 5, guide = FALSE) +
  # geom_errorbar(data = meansdall,
  #               aes(ymin = ifelse(ccmean - 1.96 * ccsd < - 1, - 1,
  #                                 ccmean - 1.96 * ccsd),
  #                   ymax = ifelse(ccmean + 1.96 * ccsd > 1, 1,
  #                                 ccmean + 1.96 * ccsd),
  #                   x = lagmean),
```

```
  #                width = 0, inherit.aes = FALSE) +
  # geom_errorbarh(data = meansdall, aes(xmin = lagmean - 1.96 * lagsd,
  #                                      xmax = lagmean + 1.96 * lagsd,
  #                                      y = ccmean,
  #                                      height = 0),
  #                 inherit.aes = FALSE) +
  theme_classic() +
  # ggtitle("All cytokines together") +
  ylab("Maximum cross-correlation") +
  geom_hline(yintercept = 0, linetype = "dashed", color = "grey80") +
  geom_vline(xintercept = 0, linetype = "dashed", color = "grey80") +
  xlim(-10, 10) +
  ylim(-1, 1)
print(p3a)
```

```
## Warning: Removed 506 rows containing missing values (geom_point).
```

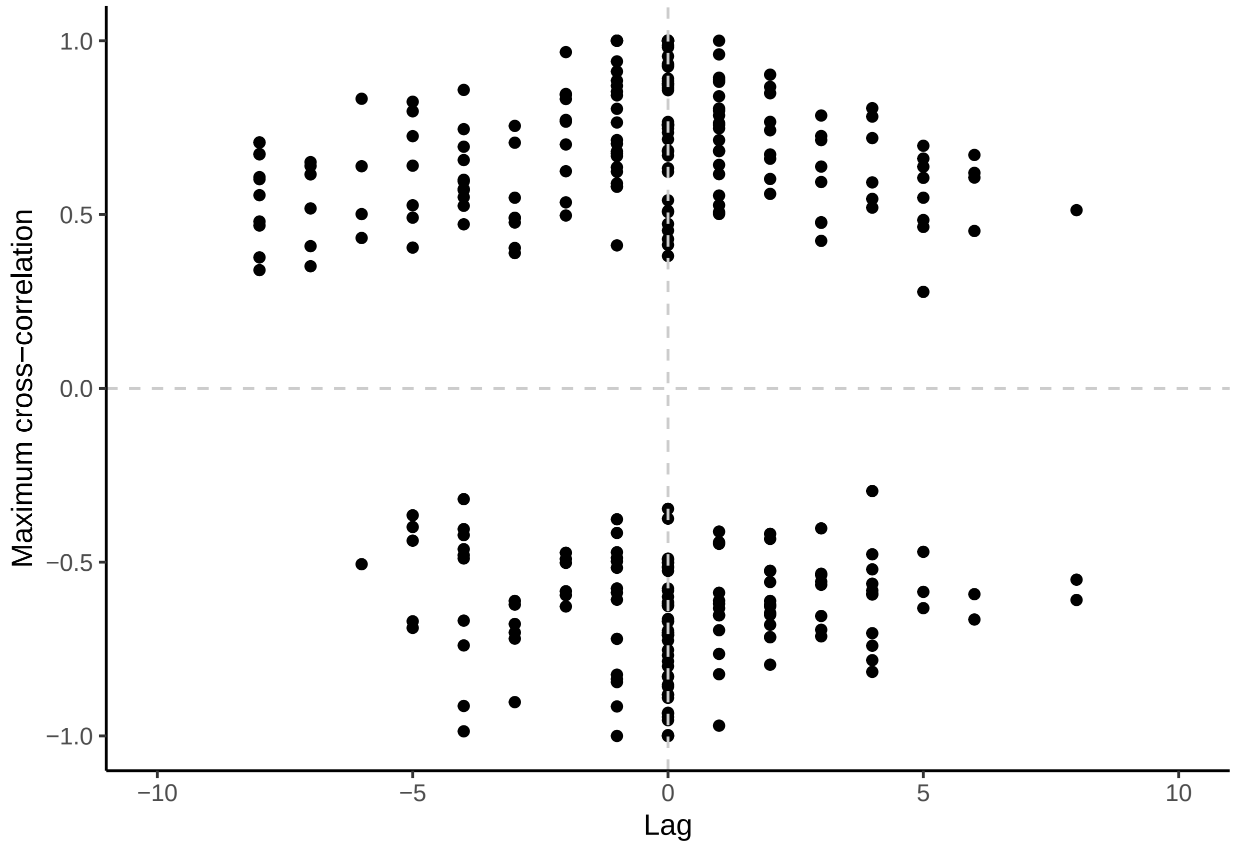


```
# ggsave("crossall200906.pdf",
#        plot = p3a,
#        device = "pdf",
#        width = 8,
#        height = 4,
#        units = "in")

ggsave("crossall201013.pdf",
       plot = p3a,
       device = "pdf",
```

```
       width = 6,
       height = 4,
       units = "in")
```

```
## Warning: Removed 506 rows containing missing values (geom_point).
```

```
ggsave("crossall201013.tiff",
       plot = p3a,
       device = "tiff",
       width = 6,
       height = 4,
       units = "in",
       dpi = "retina")
```

```
## Warning: Removed 506 rows containing missing values (geom_point).
```

```
p3 <- function(data_all, name, title) {
  meansd_spec <- data.frame(lagmean_no = mean(data_all[data_all[ , name] == "No",
                                                        "Lag"], na.rm = TRUE),
                            ccmean_no = mean(data_all[data_all[ , name] == "No",
                                                       "Maximum_value"], na.rm = TRUE),
                            lagsd_no = sd(data_all[data_all[ , name] == "No",
                                                    "Lag"], na.rm = TRUE),
                            ccsd_no = sd(data_all[data_all[ , name] == "No",
                                                   "Maximum_value"], na.rm = TRUE),
                            lagmean_yes = mean(data_all[data_all[ , name] == "Yes",
                                                         "Lag"], na.rm = TRUE),
                            ccmean_yes = mean(data_all[data_all[ , name] == "Yes",
                                                        "Maximum_value"], na.rm = TRUE),
                            lagsd_yes = sd(data_all[data_all[ , name] == "Yes",
                                                     "Lag"], na.rm = TRUE),
                            ccsd_yes = sd(data_all[data_all[ , name] == "Yes",
                                                    "Maximum_value"], na.rm = TRUE))
  meansd_spec[is.na(meansd_spec)] <- 0

  p3c <- ggplot(data = data_all, aes(x = Lag, y = Maximum_value)) +
    geom_point(aes(color = data_all[ , name])) +
    theme_classic() +
    geom_point(data = meansd_spec, aes(x = lagmean_no, y = ccmean_no,
                                       shape = "Mean", size = "Mean",
                                       color = "No"),
               na.rm = TRUE) +
    scale_shape_manual(values = 1, guide = FALSE) +
    scale_size_manual(values = 5, guide = FALSE) +
    geom_errorbar(data = meansd_spec,
                  aes(ymin = ifelse(ccmean_no - 1.96 * ccsd_no < - 1, - 1,
                                    ccmean_no - 1.96 * ccsd_no),
                      ymax = ifelse(ccmean_no + 1.96 * ccsd_no > 1, 1,
                                    ccmean_no + 1.96 * ccsd_no),
                      x = lagmean_no, color = "No"),
                  width = 0, inherit.aes = FALSE,
                  na.rm = TRUE) +
    geom_errorbarh(data = meansd_spec,
                   aes(xmin = ifelse(lagmean_no - 1.96 * lagsd_no < -10, -10,
                                     lagmean_no - 1.96 * lagsd_no),
```

```
                     xmax = ifelse(lagmean_no + 1.96 * lagsd_no > 10, 10,
                                   lagmean_no + 1.96 * lagsd_no),
                     y = ccmean_no, color = "No",
                     height = 0),
                 inherit.aes = FALSE,
                 na.rm = TRUE) +
    geom_point(data = meansd_spec, aes(x = lagmean_yes, y = ccmean_yes,
                                       shape = "Mean", size = "Mean",
                                       color = "Yes"),
               na.rm = TRUE) +
    geom_errorbar(data = meansd_spec,
                  aes(ymin = ifelse(ccmean_yes - 1.96 * ccsd_yes < - 1, - 1,
                                    ccmean_yes - 1.96 * ccsd_yes),
                      ymax = ifelse(ccmean_yes + 1.96 * ccsd_yes > 1, 1,
                                    ccmean_yes + 1.96 * ccsd_yes),
                      x = lagmean_yes, color = "Yes"),
                  width = 0, inherit.aes = FALSE,
                  na.rm = TRUE) +
    geom_errorbarh(data = meansd_spec,
                   aes(xmin = ifelse(lagmean_yes - 1.96 * lagsd_yes < -10, -10,
                                     lagmean_yes - 1.96 * lagsd_yes),
                       xmax = ifelse(lagmean_yes + 1.96 * lagsd_yes > 10, 10,
                                     lagmean_yes + 1.96 * lagsd_yes),
                       y = ccmean_yes, color = "Yes",
                       height = 0),
                   inherit.aes = FALSE,
                   na.rm = TRUE) +
    ggtitle(title) +
    ylab("Maximum cross-correlation") +
    geom_hline(yintercept = 0, linetype = "dashed", color = "grey80") +
    geom_vline(xintercept = 0, linetype = "dashed", color = "grey80") +
    xlim(-10, 10) +
    ylim(-1, 1) +
    labs(color = name)
  print(p3c)

  return(p3c)
}

for (vars in 7:12) {
  names <- colnames(output)[vars]
  p3c_out <- p3(data_all = output, name = names, title = "All cytokines together")

  ggsave(paste0("cross", names, "200906", ".pdf"),
       plot = p3c_out,
       device = "pdf",
       width = 8,
       height = 4,
       units = "in")
}
```

```
## Warning: Removed 506 rows containing missing values (geom_point).

## Warning: Removed 506 rows containing missing values (geom_point).
```

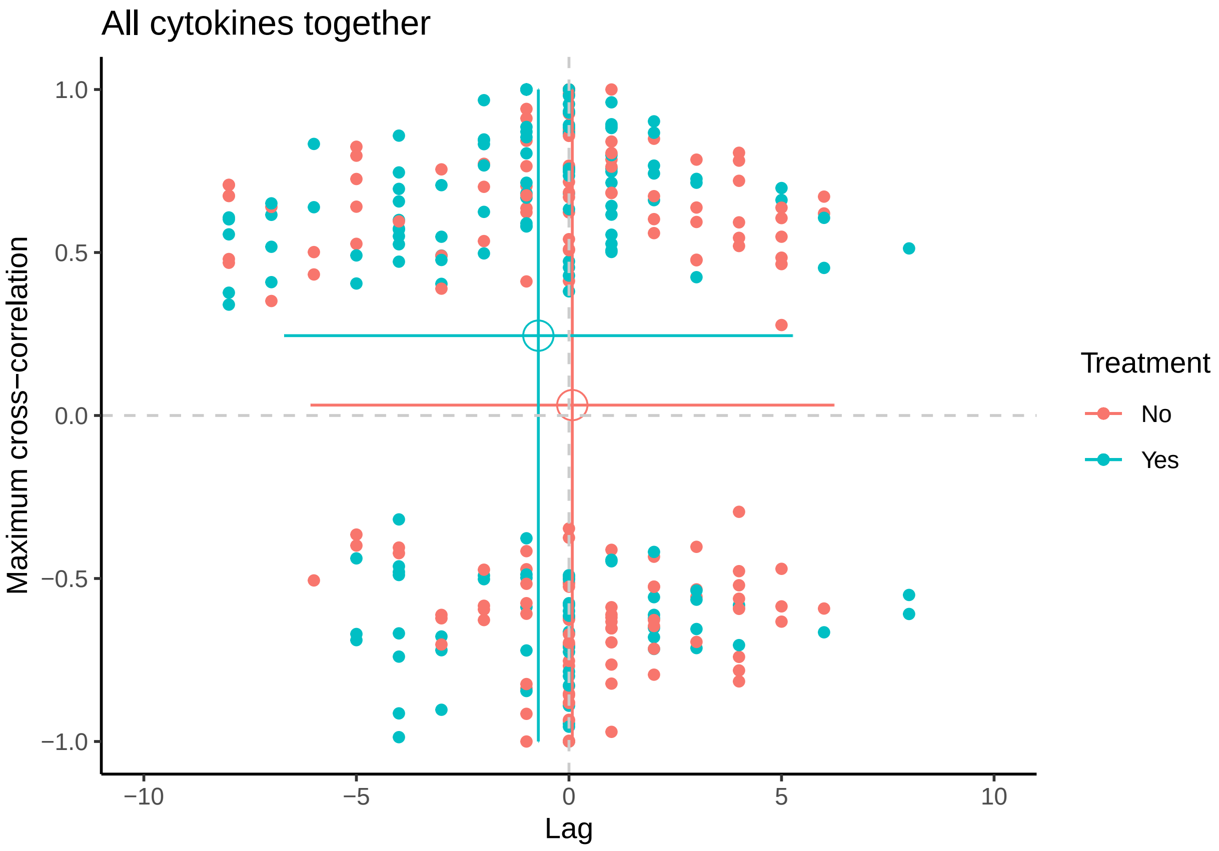


```
## Warning: Removed 506 rows containing missing values (geom_point).

## Warning: Removed 506 rows containing missing values (geom_point).
```

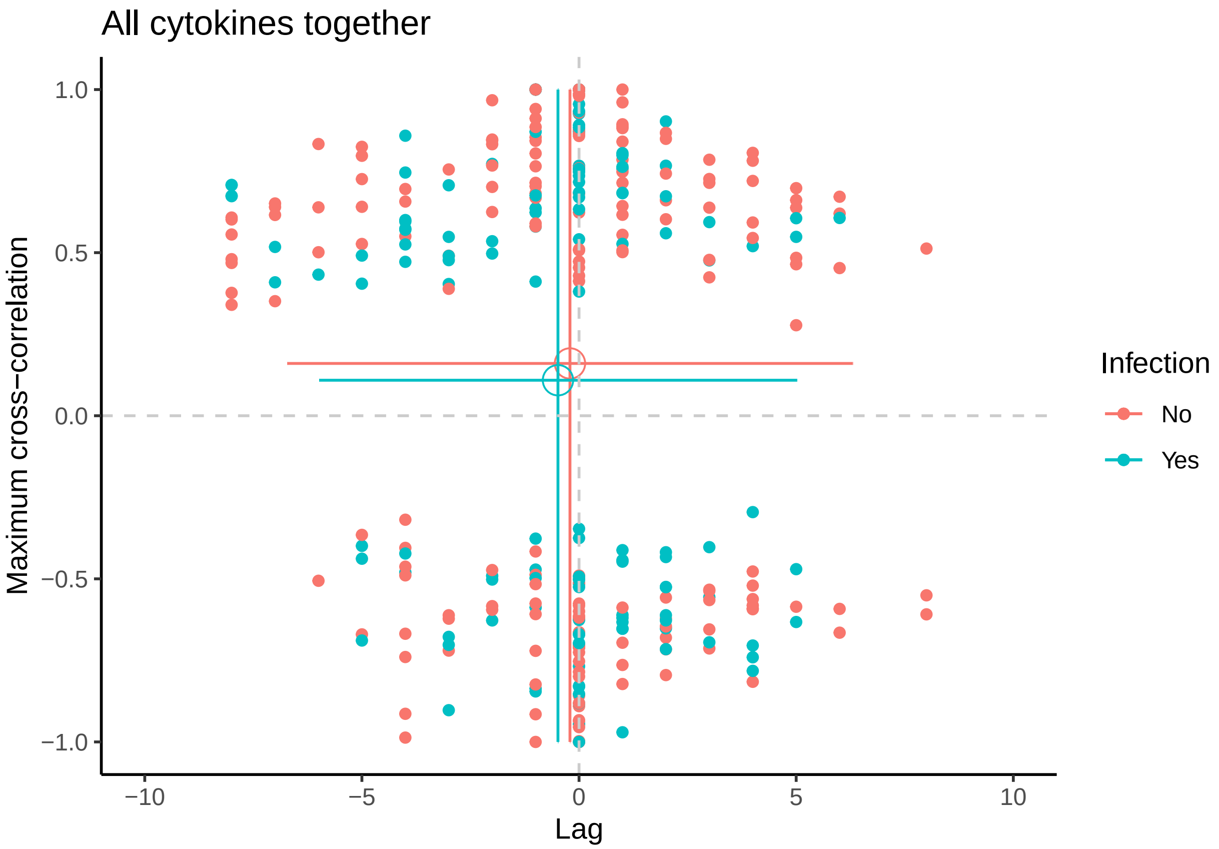


```
## Warning: Removed 506 rows containing missing values (geom_point).

## Warning: Removed 506 rows containing missing values (geom_point).
```

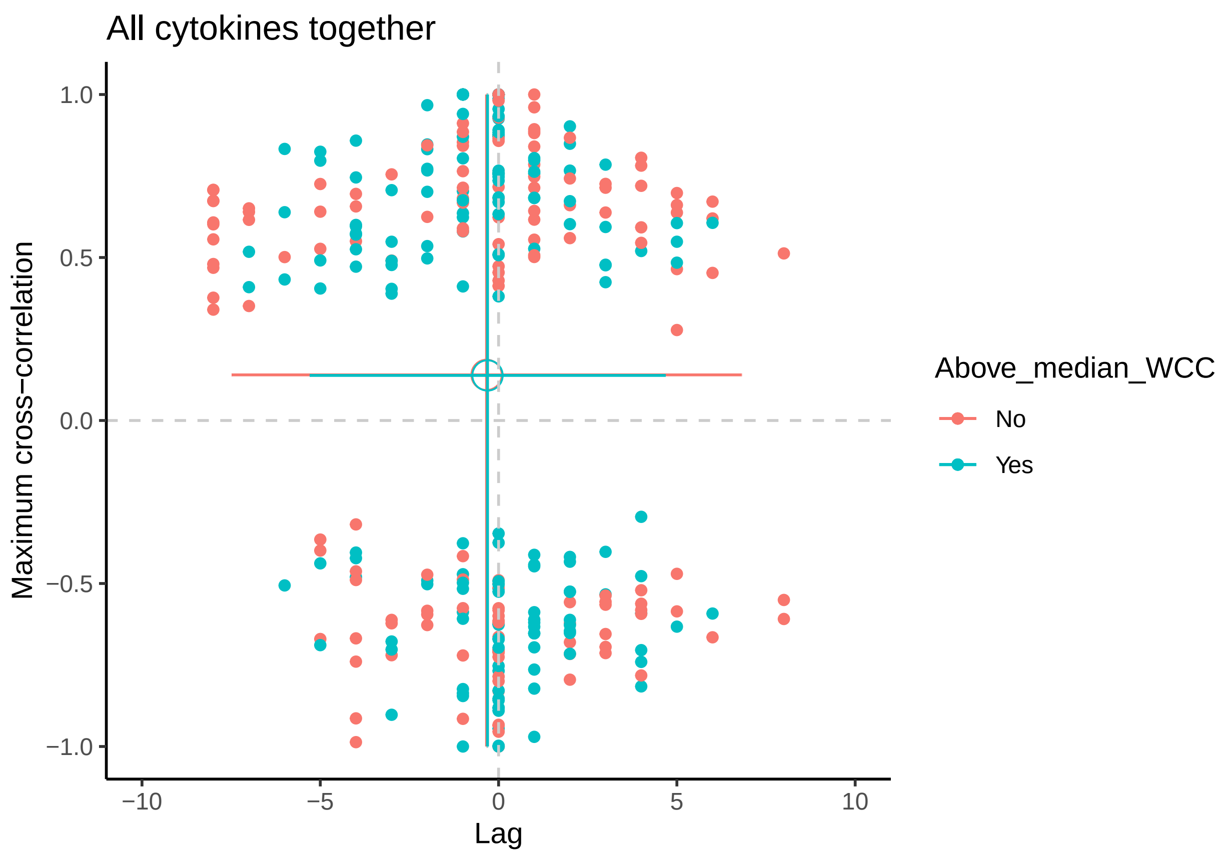


```
## Warning: Removed 506 rows containing missing values (geom_point).

## Warning: Removed 506 rows containing missing values (geom_point).
```

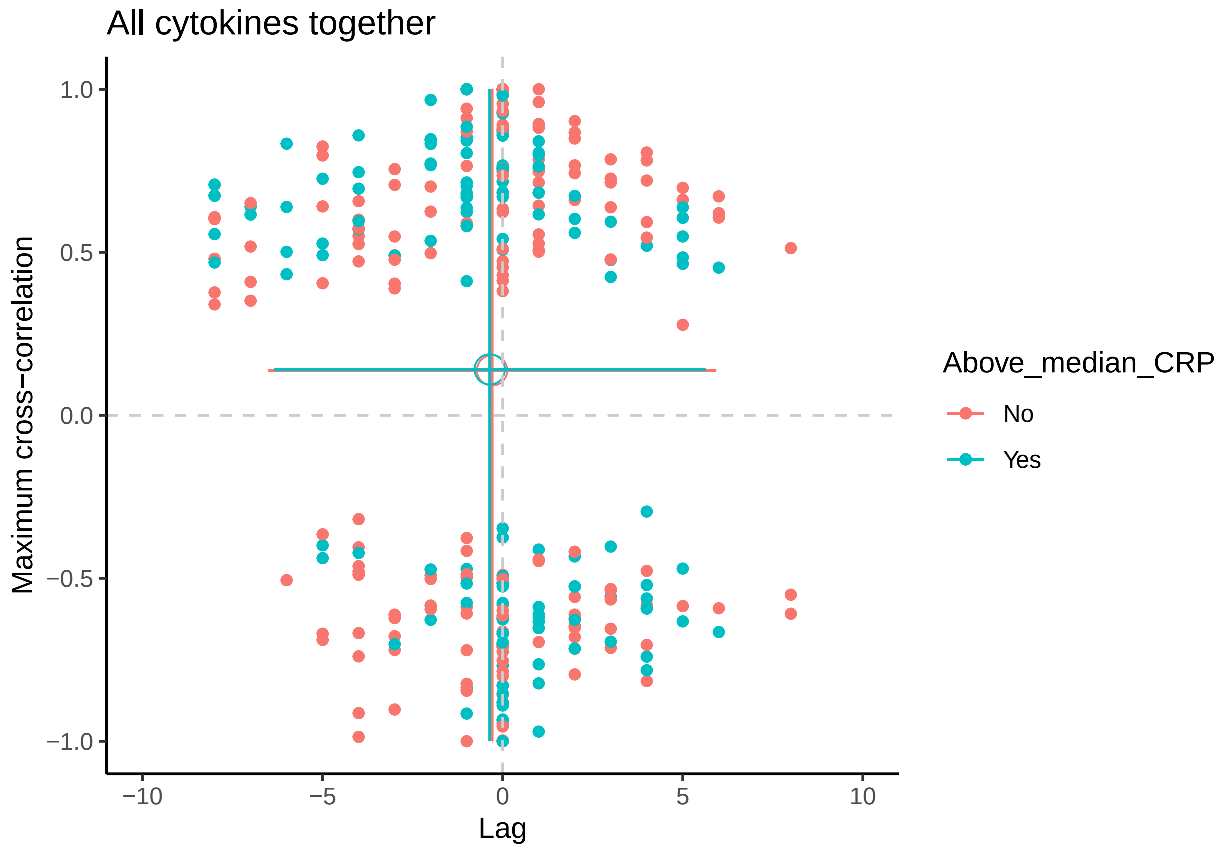


```
## Warning: Removed 506 rows containing missing values (geom_point).

## Warning: Removed 506 rows containing missing values (geom_point).
```

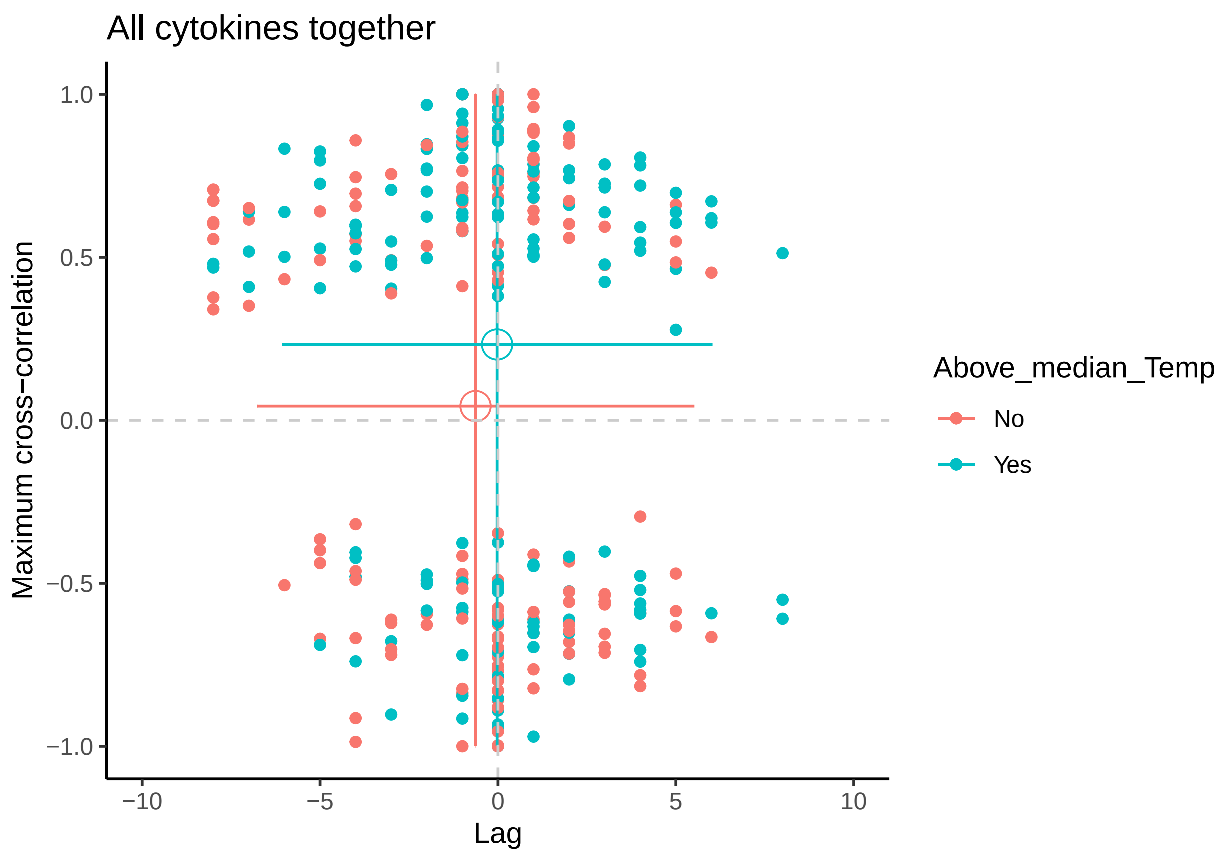


```
## Warning: Removed 506 rows containing missing values (geom_point).

## Warning: Removed 506 rows containing missing values (geom_point).
```

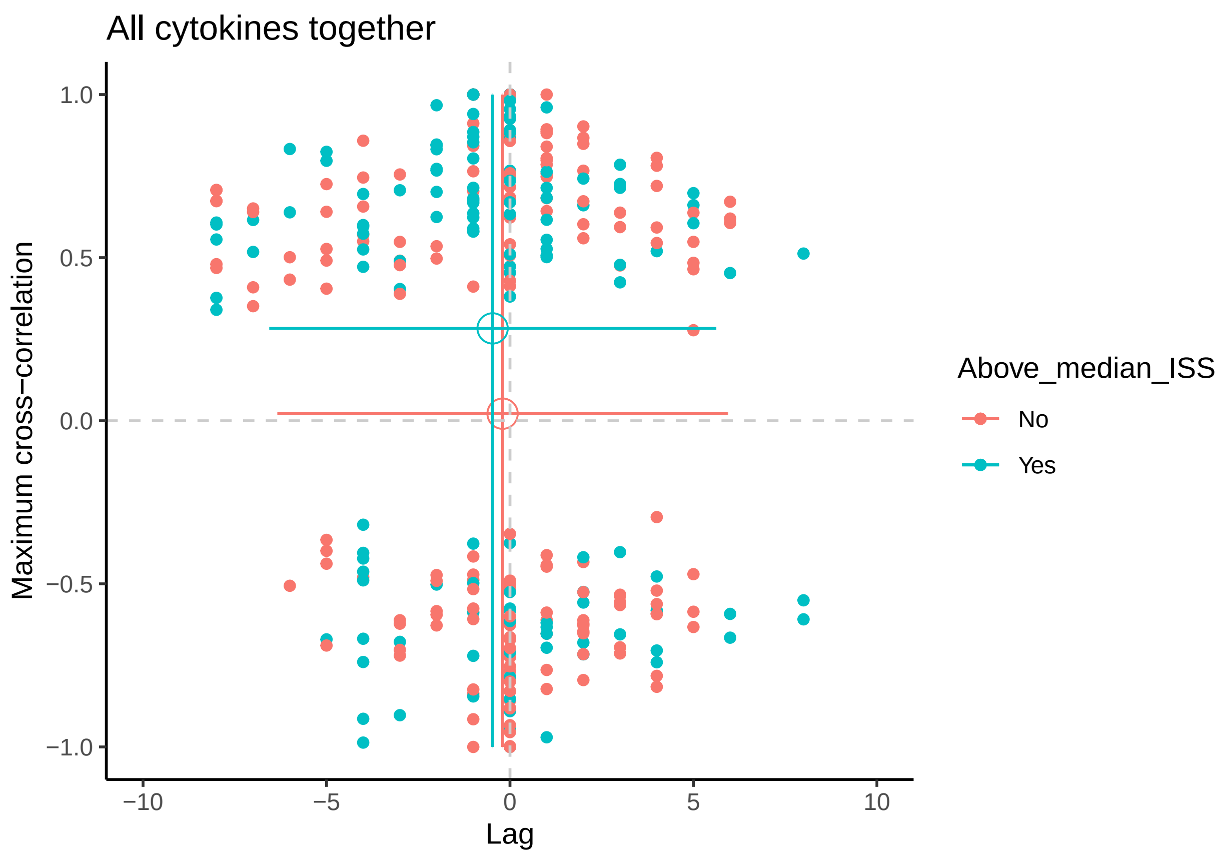


```
for (cytokine in 1:42) {
  local_data <- output[((cytokine - 1) * 20 + 1):(cytokine * 20), ]
  local_data <- local_data[local_data$Included == "Yes", ]

  for (vars in 7:12) {
    names <- colnames(output)[vars]
    p3(data_all = local_data, name = names,
       title = gsub("[.]", "-",
                    substr(colnames(ip_data)[cytokine*3+5], start = 4, stop = 100)))
  }
}
```

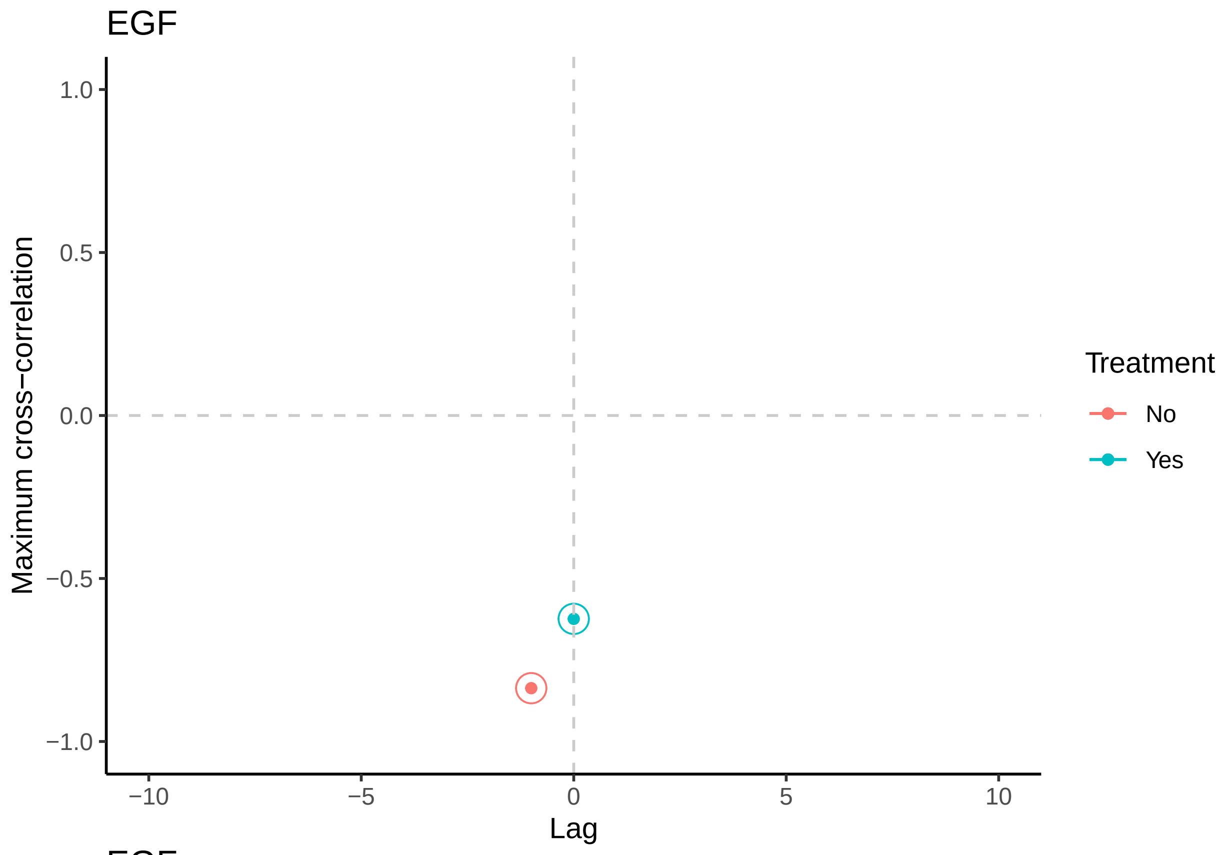
EGF
Maximum cross−correlation
1.0
0.5
0.0
−0.5
−1.0
−10
−5
0
5
10
Lag
Treatment
No
Yes

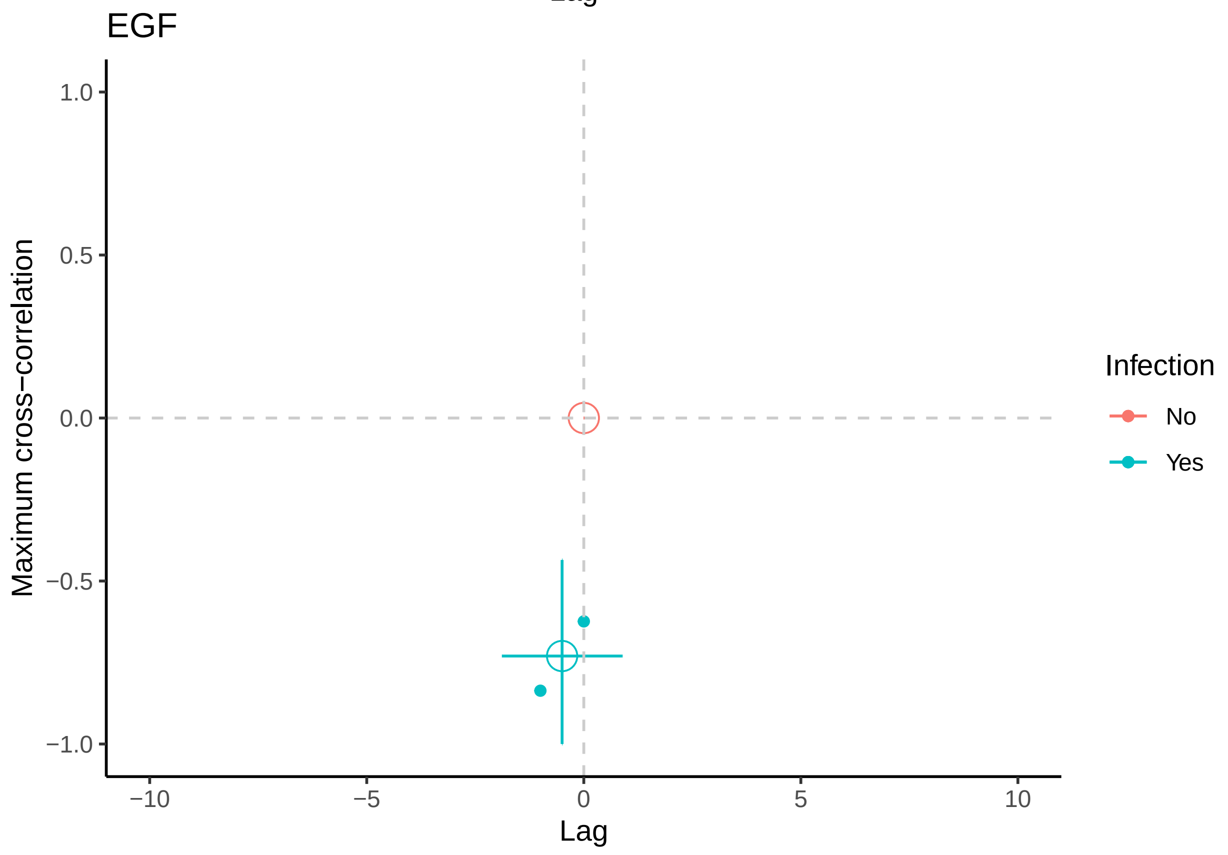
EGF
Maximum cross−correlation
1.0
0.5
0.0
−0.5
−1.0
−10
−5
0
5
10
Lag
Infection
No
Yes

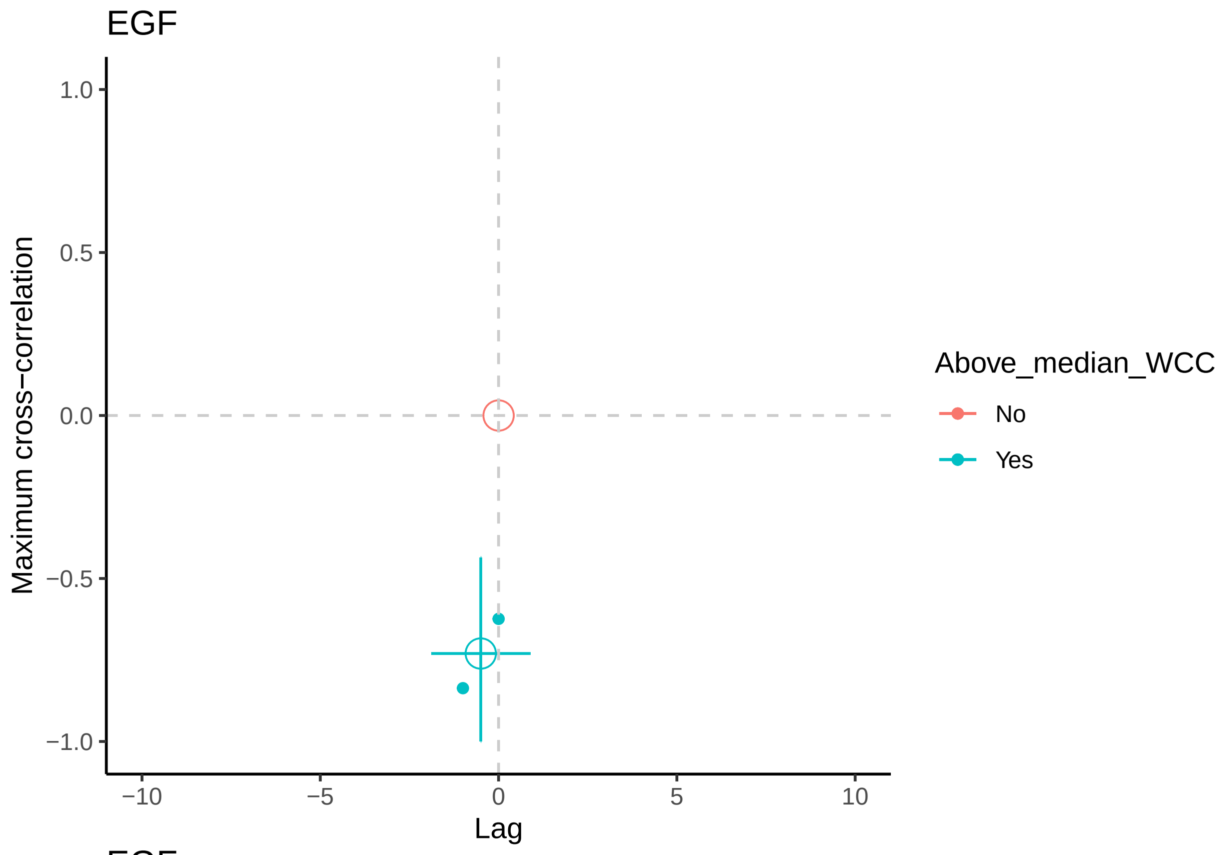
EGF
Maximum cross−correlation
1.0
0.5
0.0
−0.5
−1.0
−10
−5
0
5
10
Lag
Above_median_WCC
No
Yes

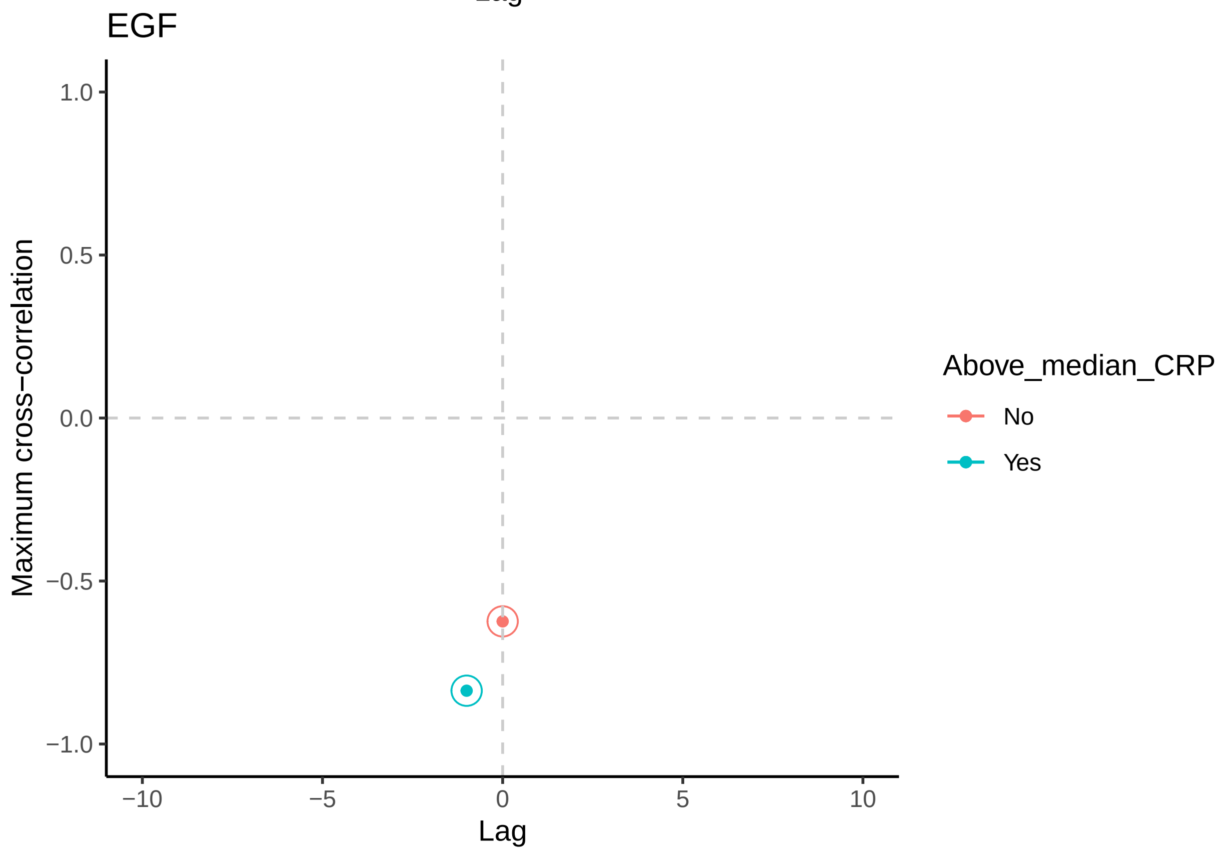
EGF
Maximum cross−correlation
1.0
0.5
0.0
−0.5
−1.0
−10
−5
0
5
10
Lag
Above_median_CRP
No
Yes

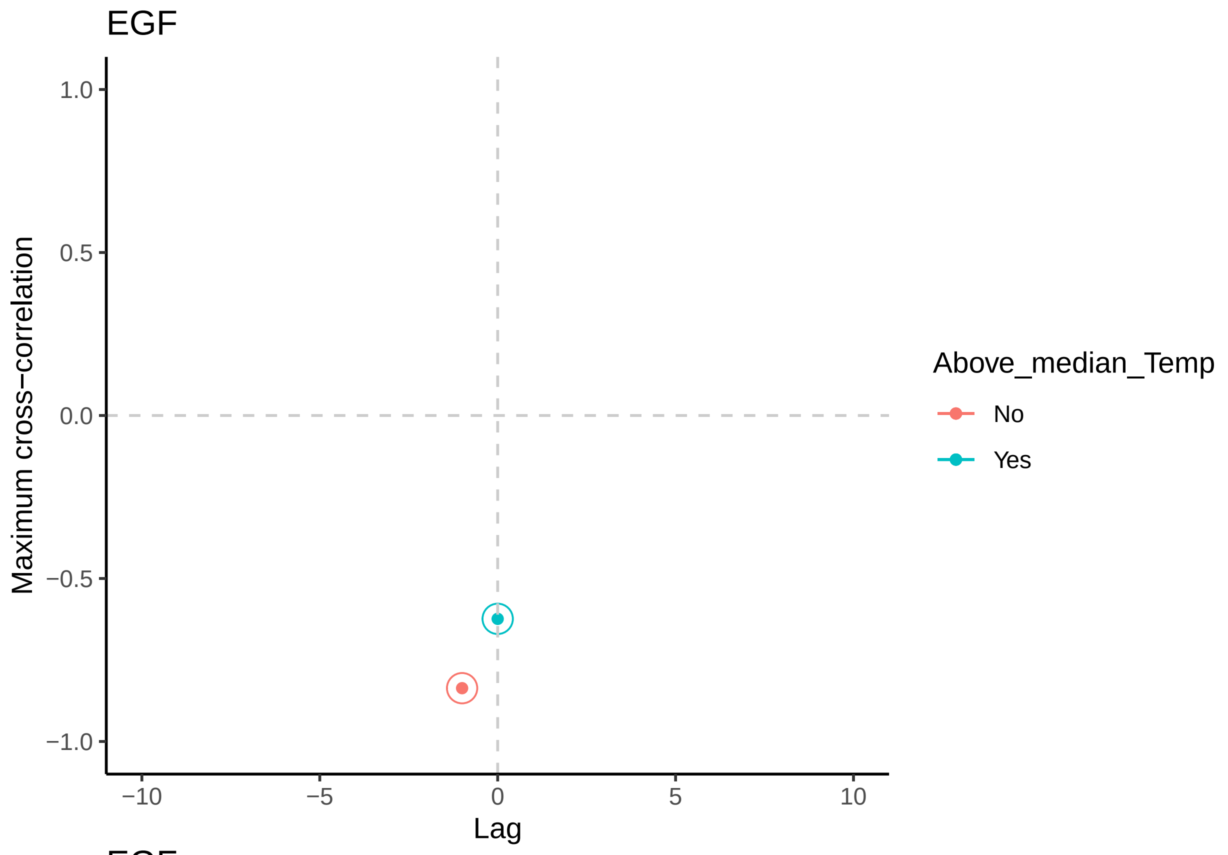

EGF
Maximum cross−correlation
1.0
0.5
0.0
−0.5
−1.0
−10
−5
0
5
10
Lag
Above_median_Temp
No
Yes

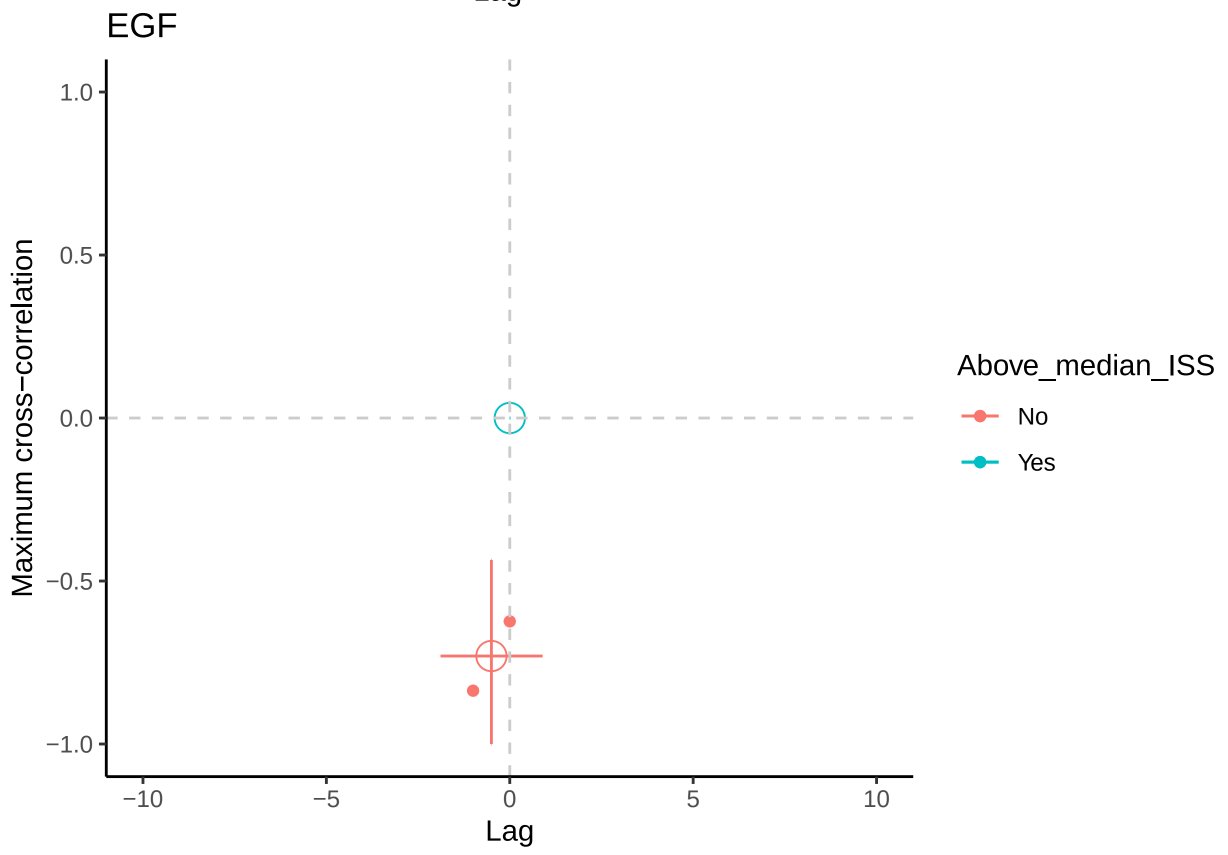

EGF
Maximum cross−correlation
1.0
0.5
0.0
−0.5
−1.0
−10
−5
0
5
10
Lag
Above_median_ISS
No
Yes

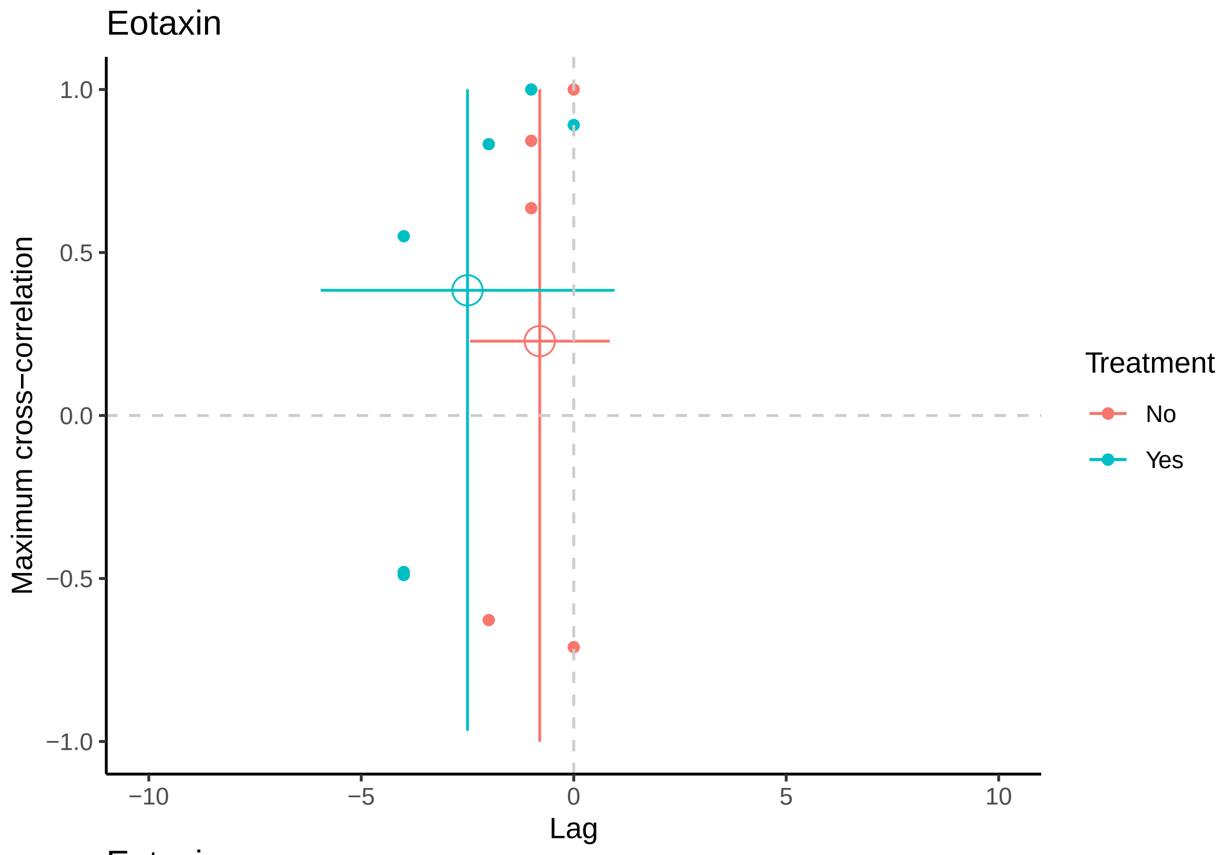
Eotaxin
Maximum cross−correlation
1.0
0.5
0.0
−0.5
−1.0
−10
−5
0
5
10
Lag
Treatment
No
Yes

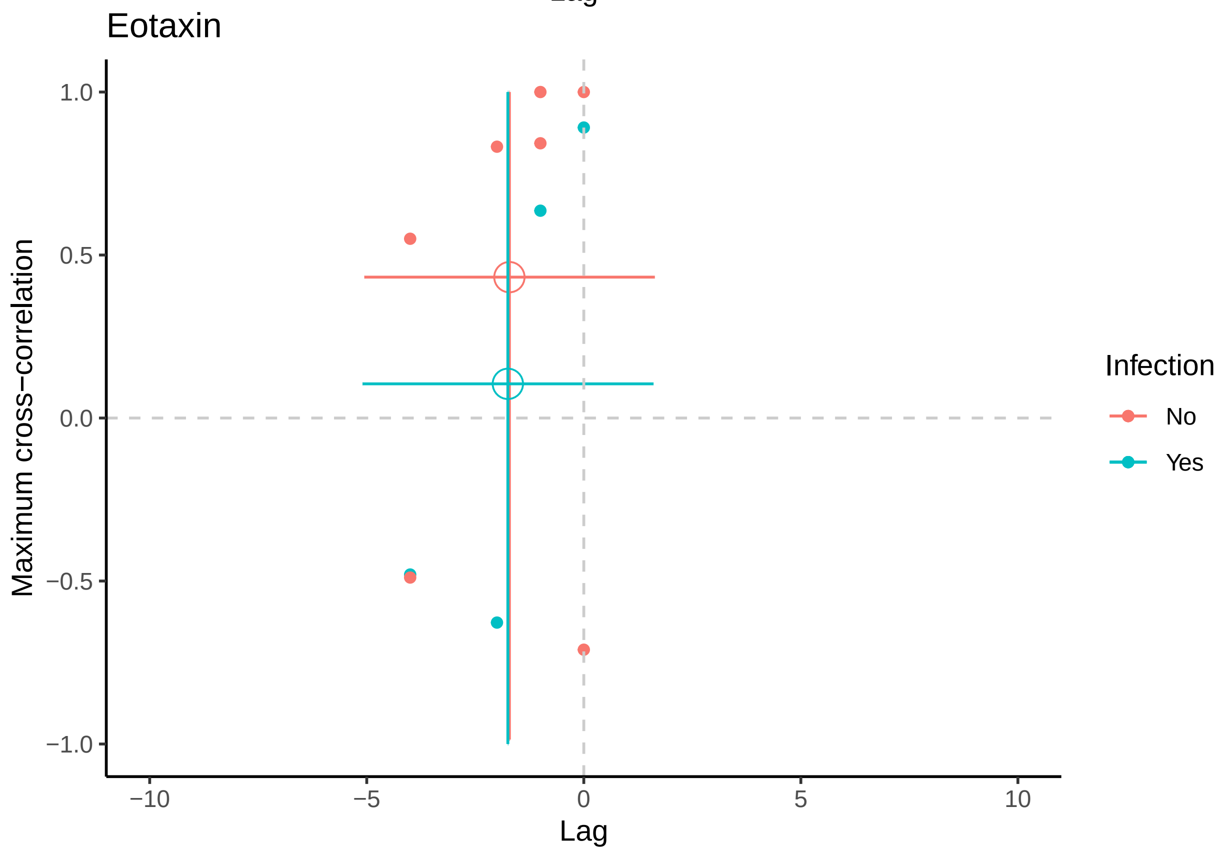
Eotaxin
Maximum cross−correlation
1.0
0.5
0.0
−0.5
−1.0
−10
−5
0
5
10
Lag
Infection
No
Yes

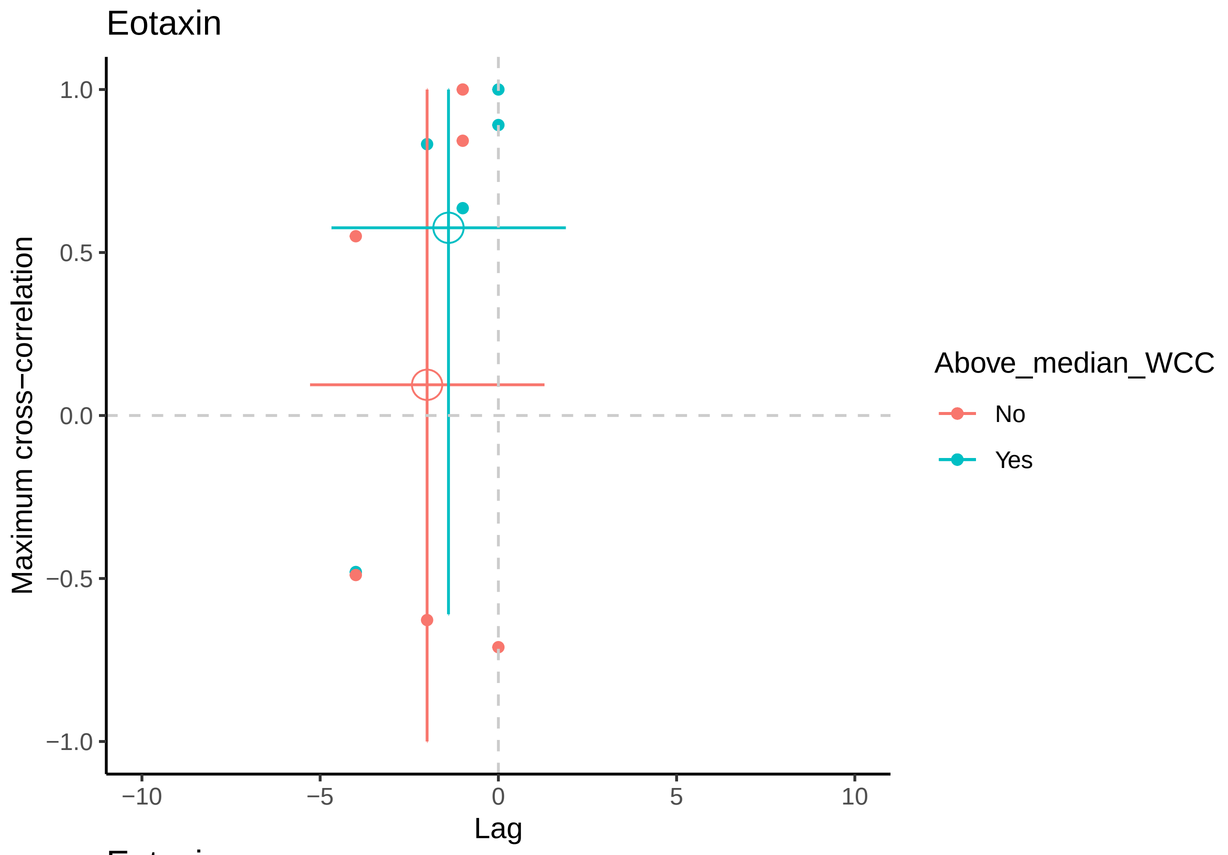
Eotaxin
Maximum cross−correlation
1.0
0.5
0.0
−0.5
−1.0
−10
−5
0
5
10
Lag
Above_median_WCC
No
Yes

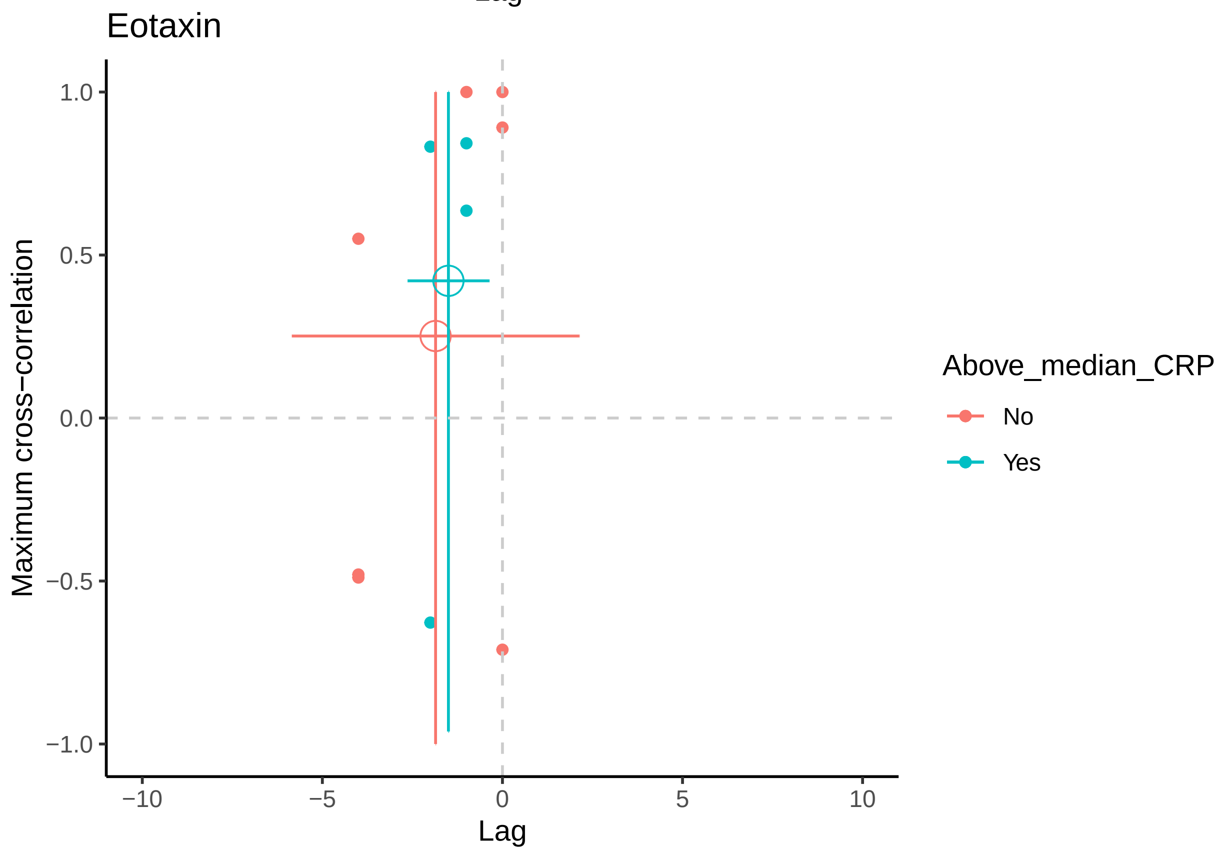
Eotaxin
Maximum cross−correlation
1.0
0.5
0.0
−0.5
−1.0
−10
−5
0
5
10
Lag
Above_median_CRP
No
Yes

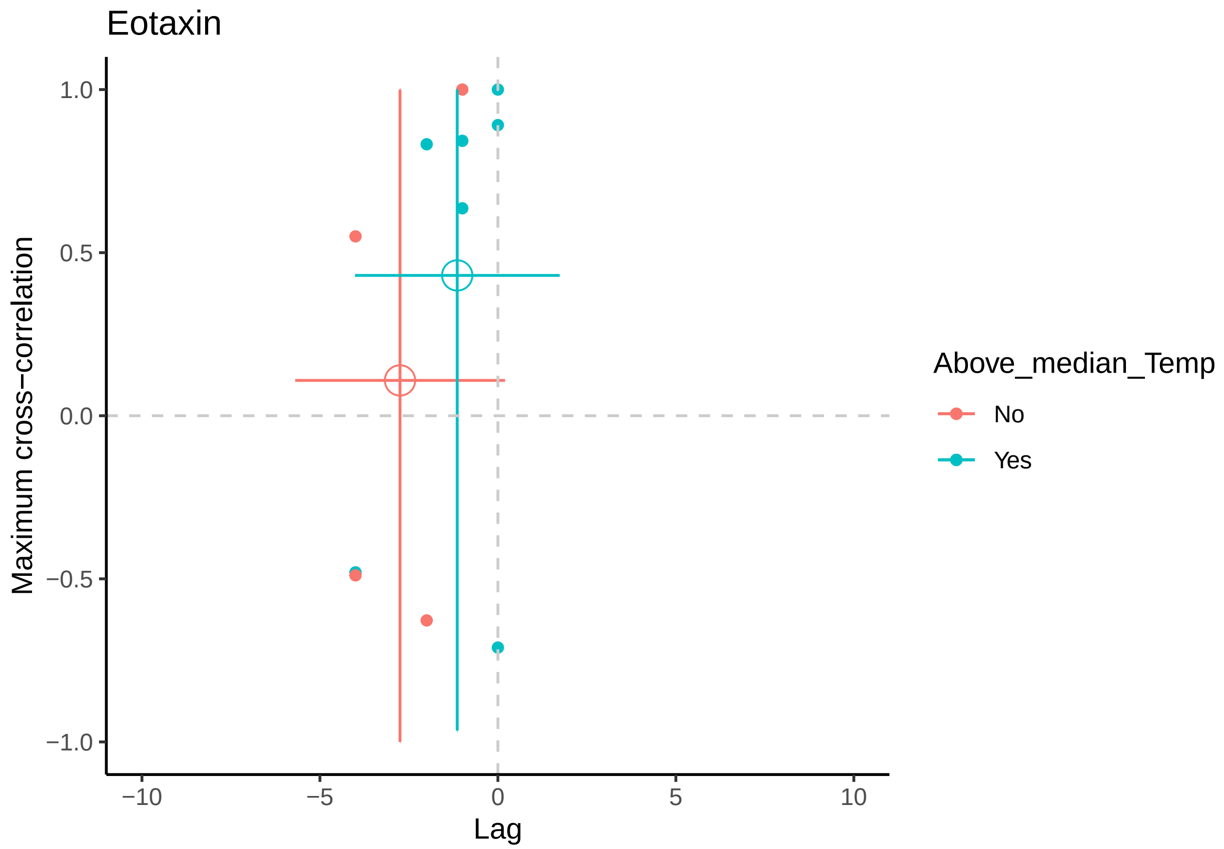
Eotaxin
Maximum cross−correlation
1.0
0.5
0.0
−0.5
−1.0
−10
−5
0
5
10
Lag
Above_median_Temp
No
Yes

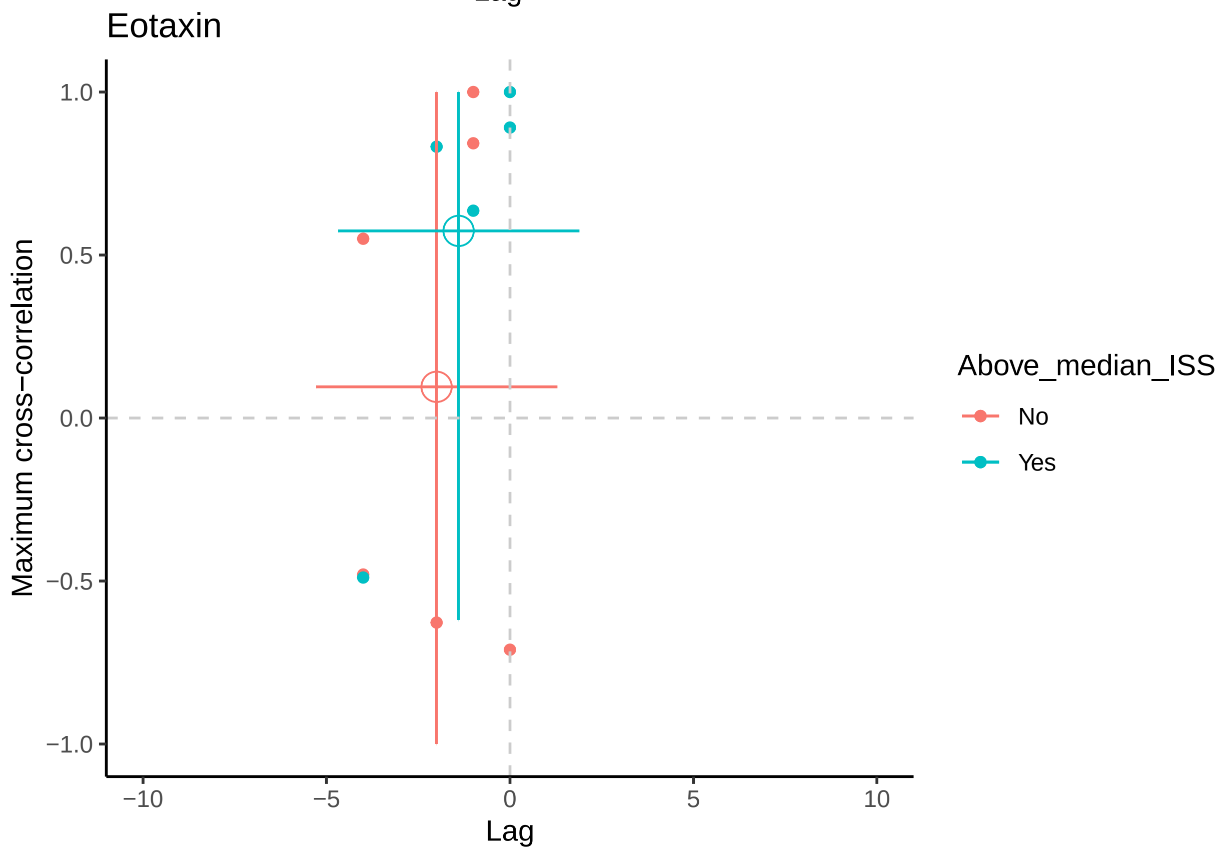
Eotaxin
Maximum cross−correlation
1.0
0.5
0.0
−0.5
−1.0
−10
−5
0
5
10
Lag
Above_median_ISS
No
Yes

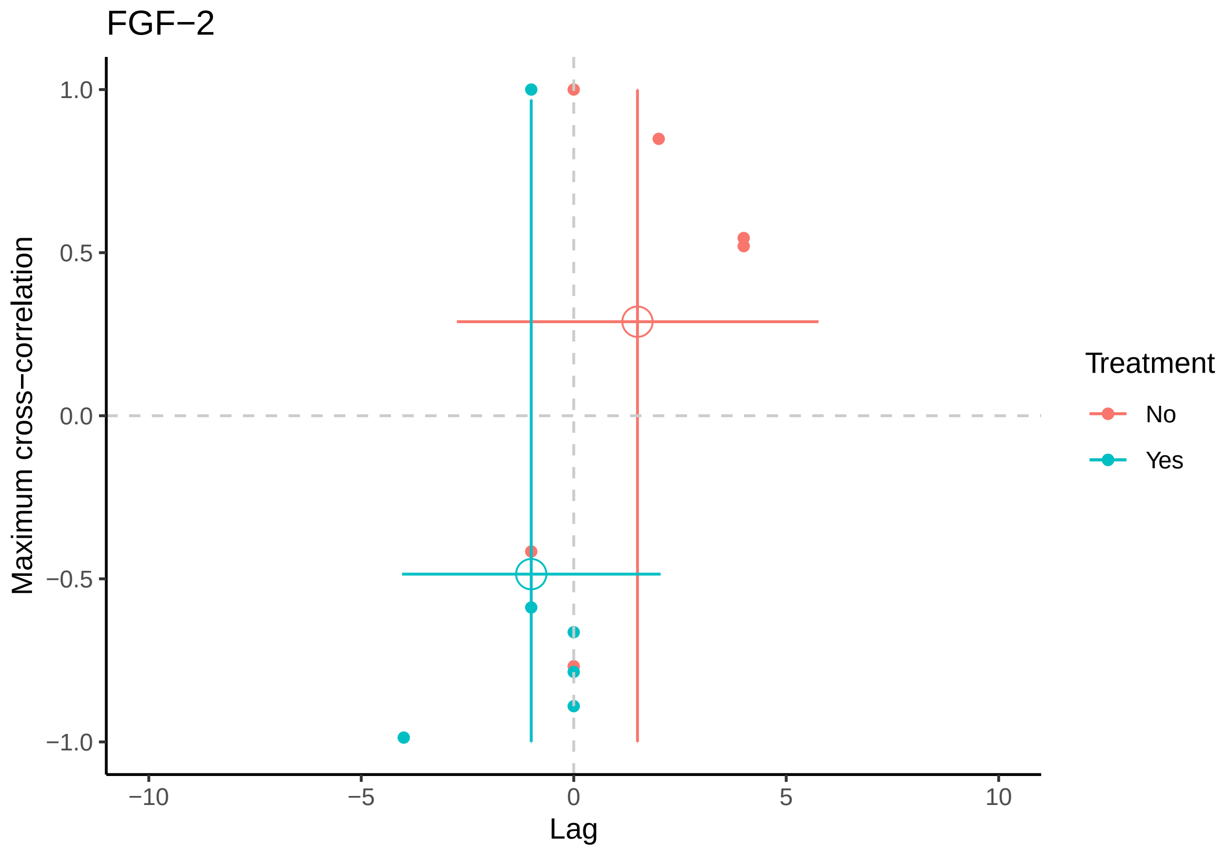
FGF−2
Maximum cross−correlation
1.0
0.5
0.0
−0.5
−1.0
−10
−5
0
5
10
Lag
Treatment
No
Yes

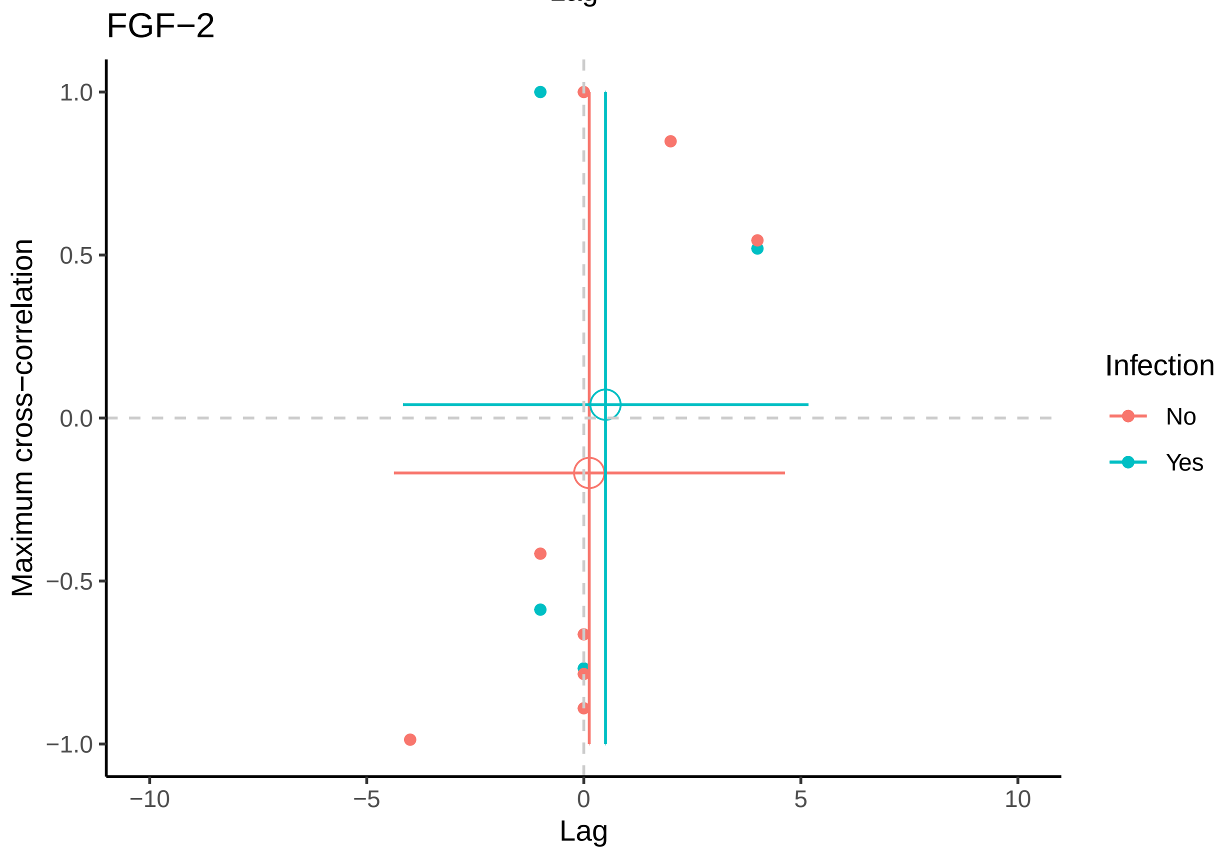
FGF−2
Maximum cross−correlation
1.0
0.5
0.0
−0.5
−1.0
−10
−5
0
5
10
Lag
Infection
No
Yes

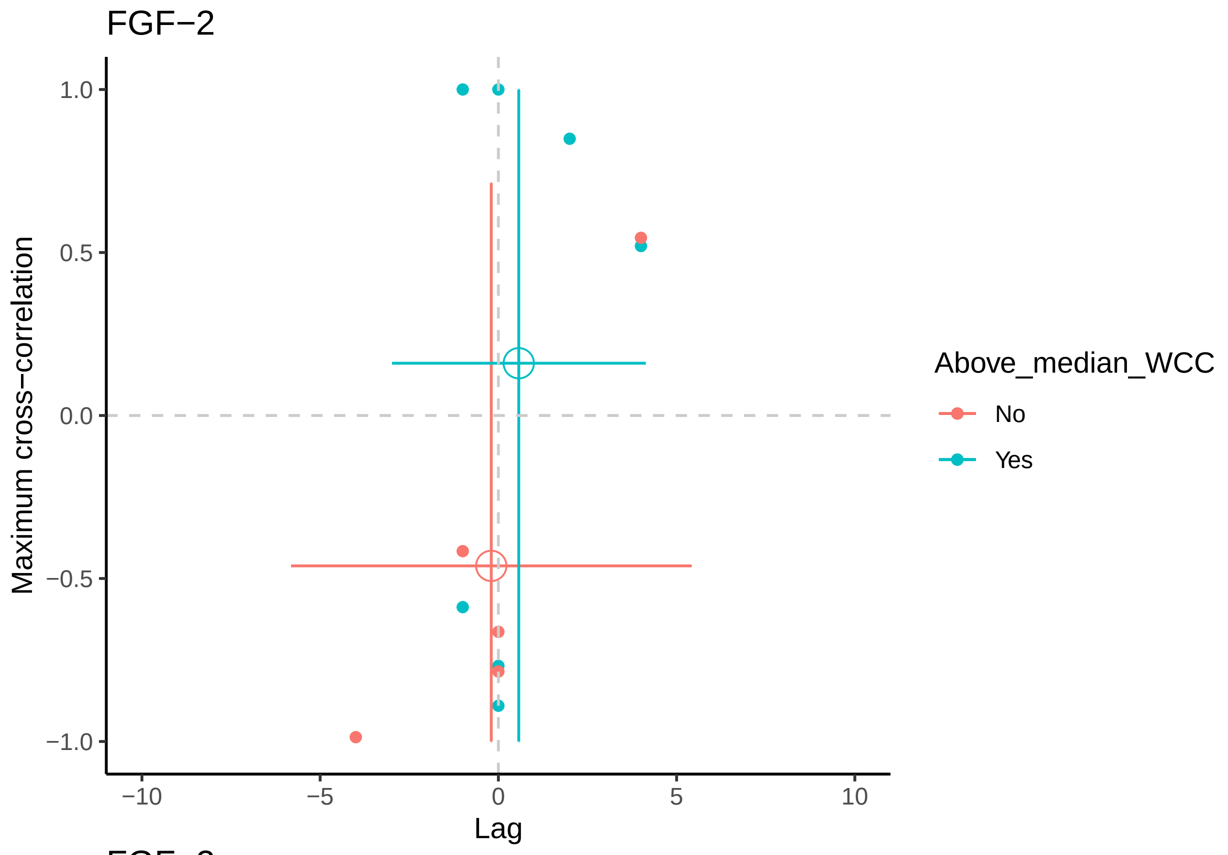

FGF−2
Maximum cross−correlation
1.0
0.5
0.0
−0.5
−1.0
−10
−5
0
5
10
Lag
Above_median_WCC
No
Yes

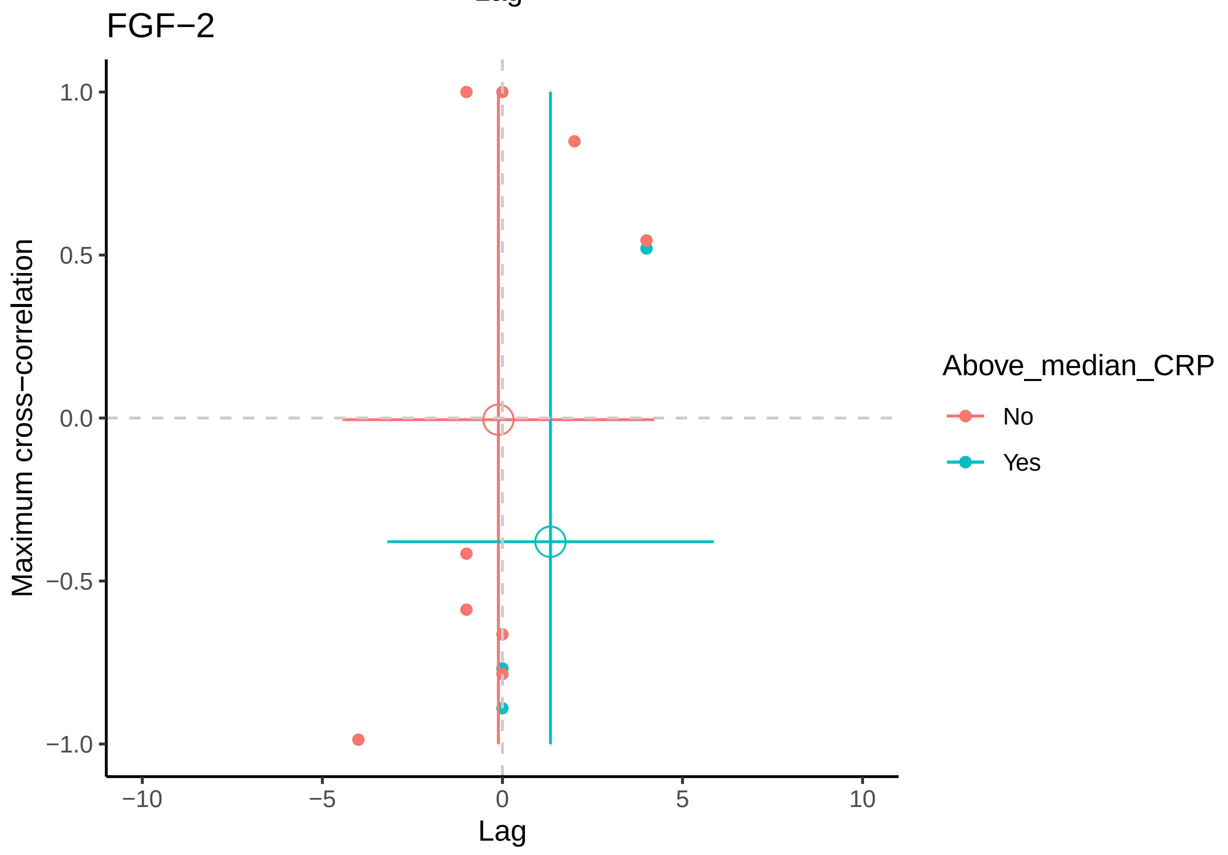

FGF−2
Maximum cross−correlation
1.0
0.5
0.0
−0.5
−1.0
−10
−5
0
5
10
Lag
Above_median_CRP
No
Yes

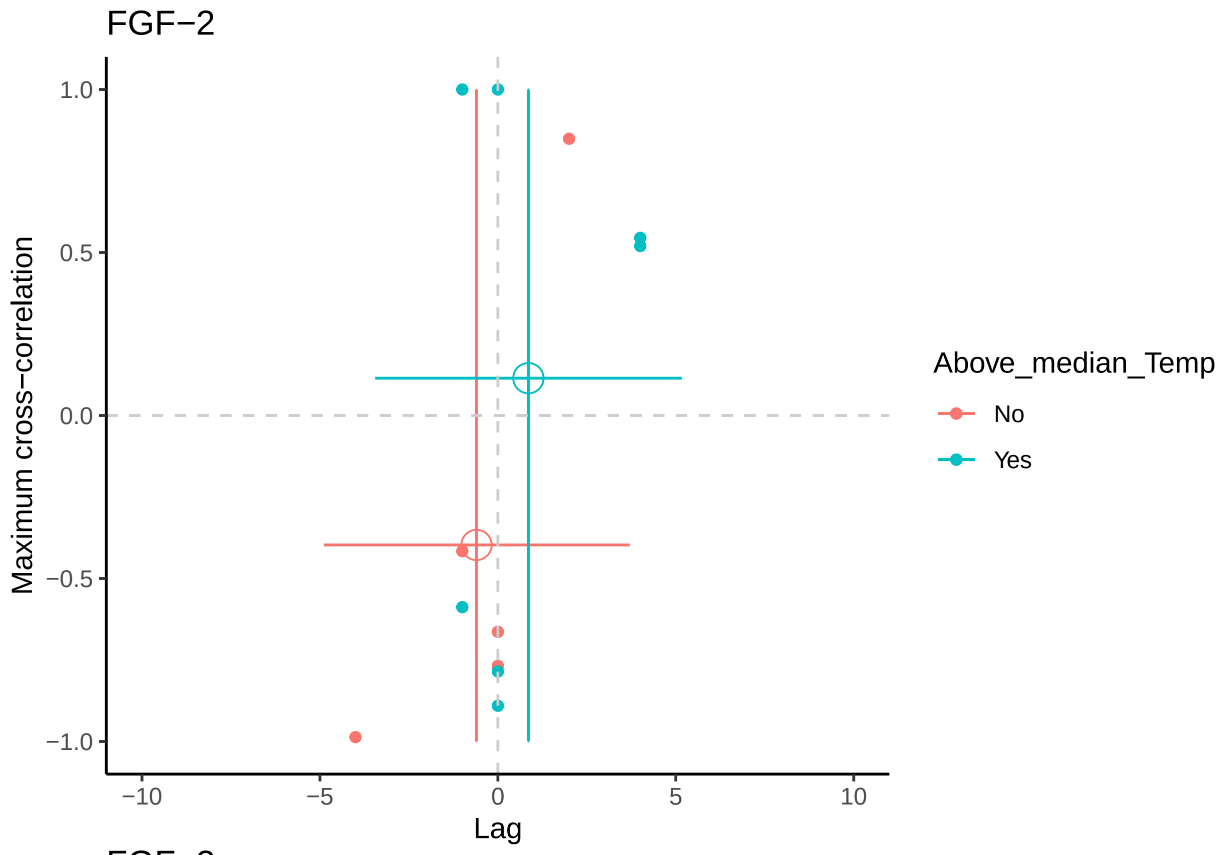

FGF−2
Maximum cross−correlation
1.0
0.5
0.0
−0.5
−1.0
−10
−5
0
5
10
Lag
Above_median_Temp
No
Yes

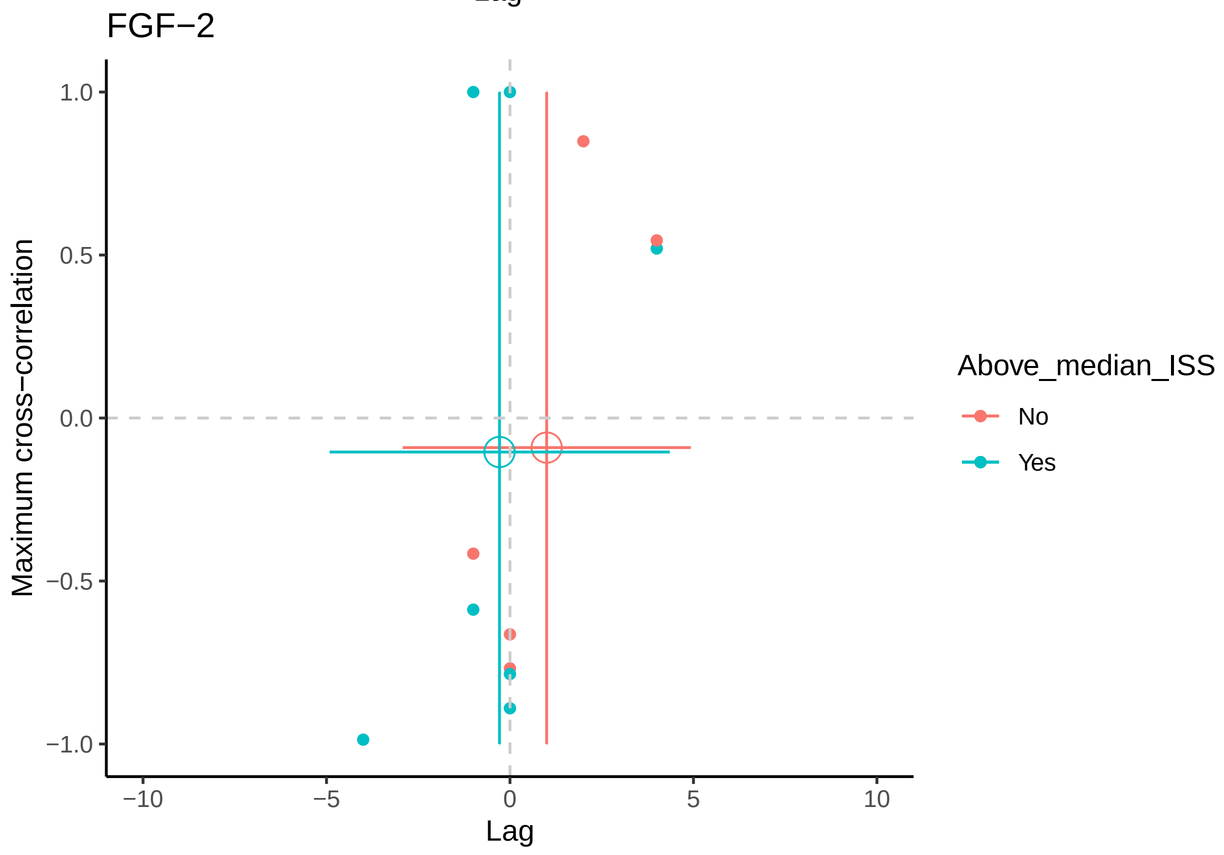

FGF−2
Maximum cross−correlation
1.0
0.5
0.0
−0.5
−1.0
−10
−5
0
5
10
Lag
Above_median_ISS
No
Yes

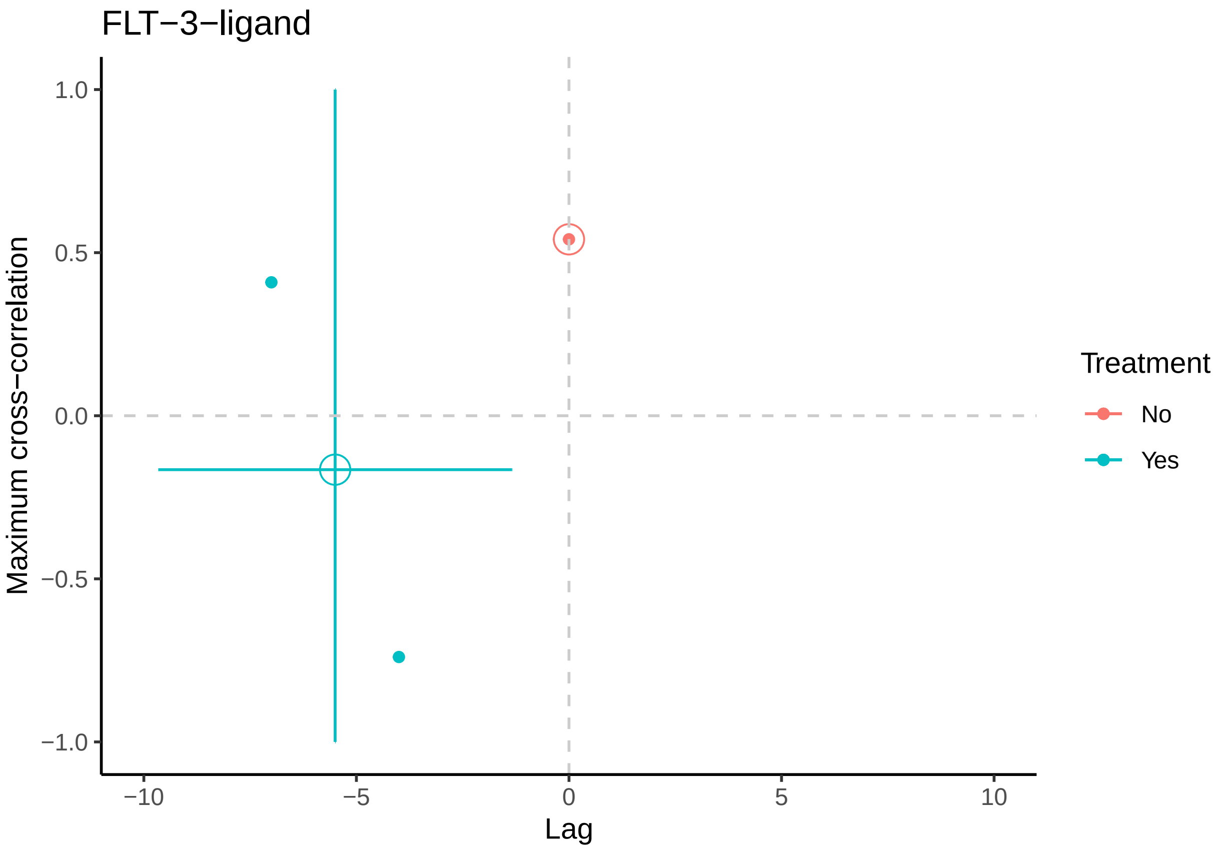
FLT−3−ligand
Maximum cross−correlation
1.0
0.5
0.0
−0.5
−1.0
−10
−5
0
5
10
Lag
Treatment
No
Yes

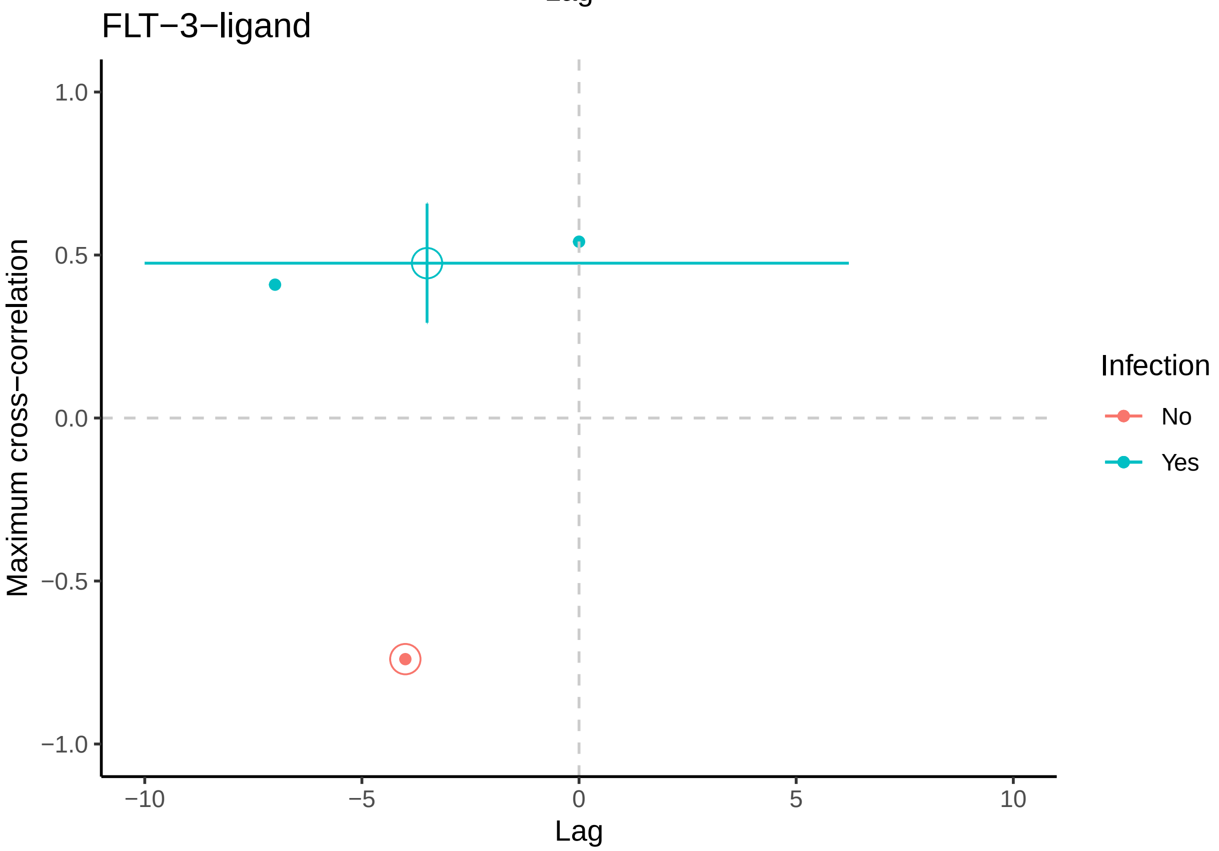
FLT−3−ligand
Maximum cross−correlation
1.0
0.5
0.0
−0.5
−1.0
−10
−5
0
5
10
Lag
Infection
No
Yes

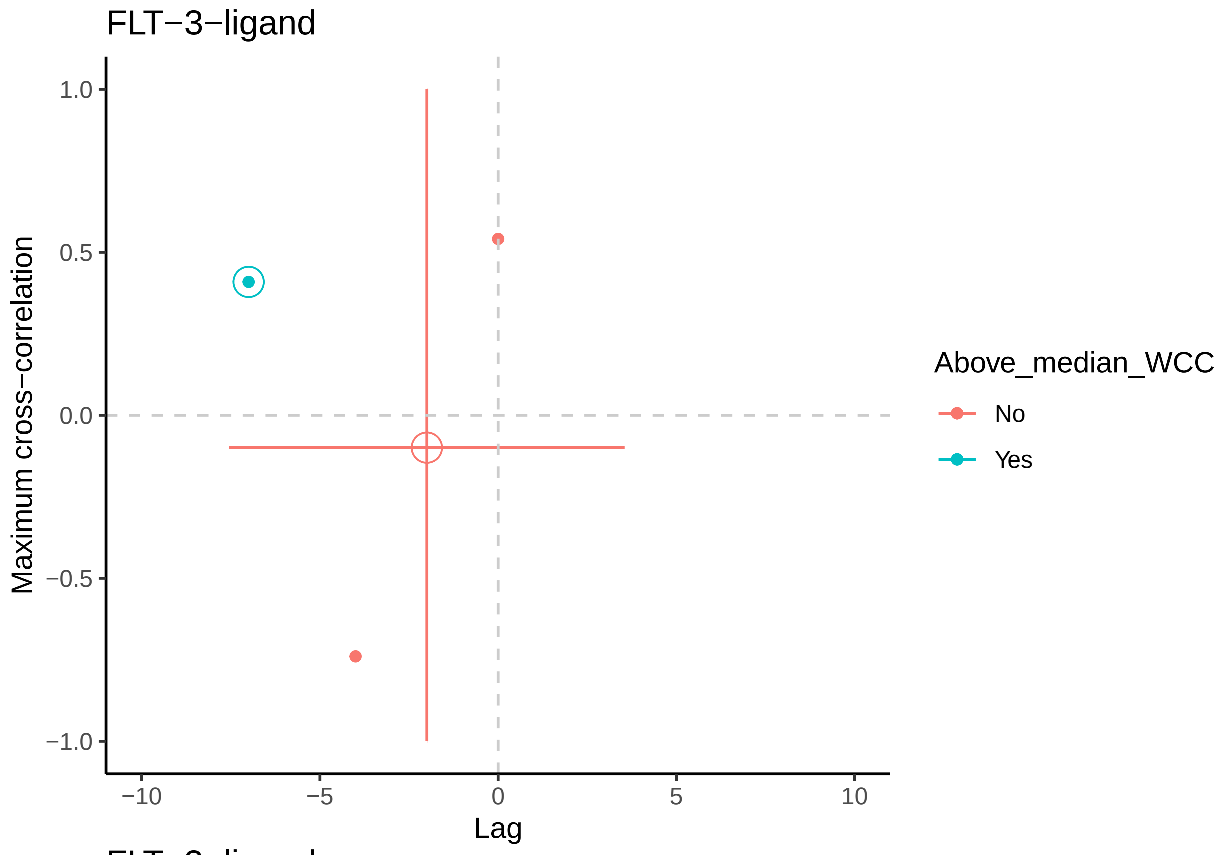
FLT−3−ligand
Maximum cross−correlation
1.0
0.5
0.0
−0.5
−1.0
−10
−5
0
5
10
Lag
Above_median_WCC
No
Yes

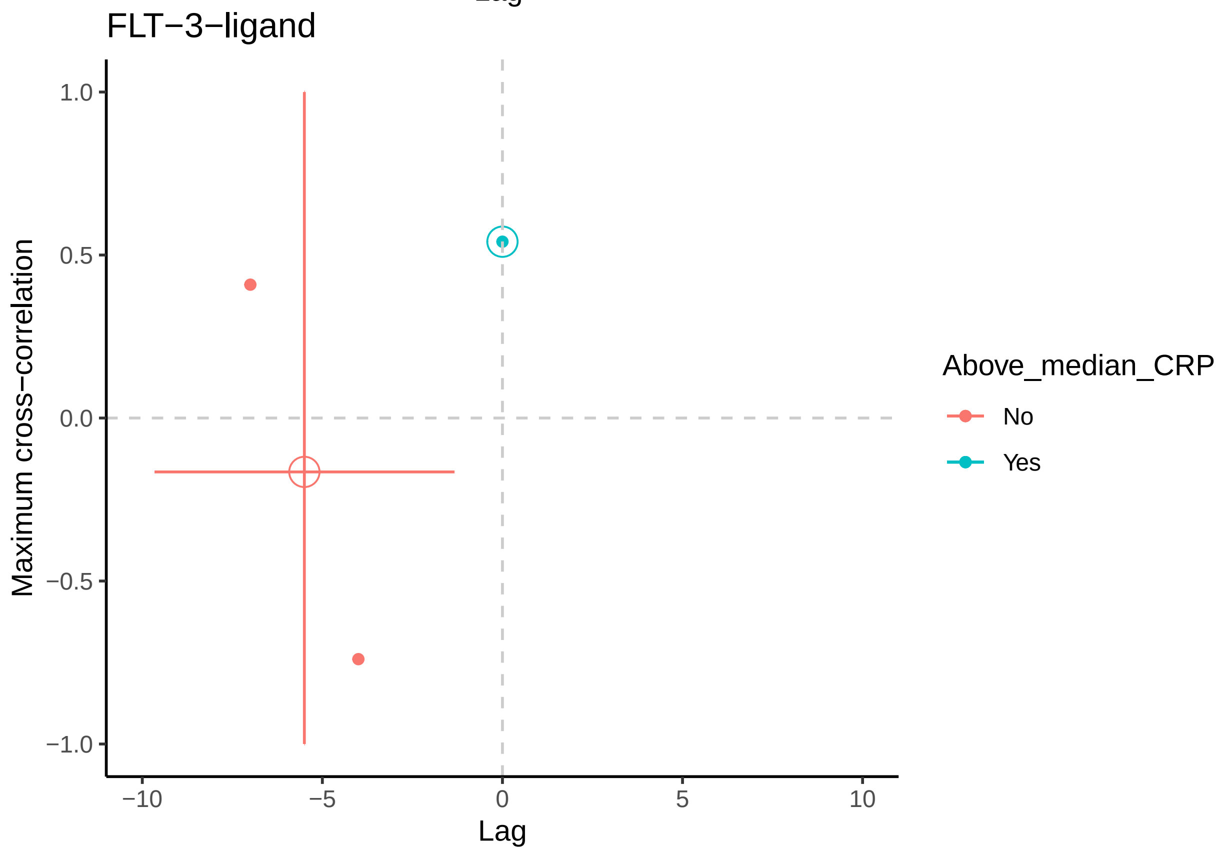
FLT−3−ligand
Maximum cross−correlation
1.0
0.5
0.0
−0.5
−1.0
−10
−5
0
5
10
Lag
Above_median_CRP
No
Yes

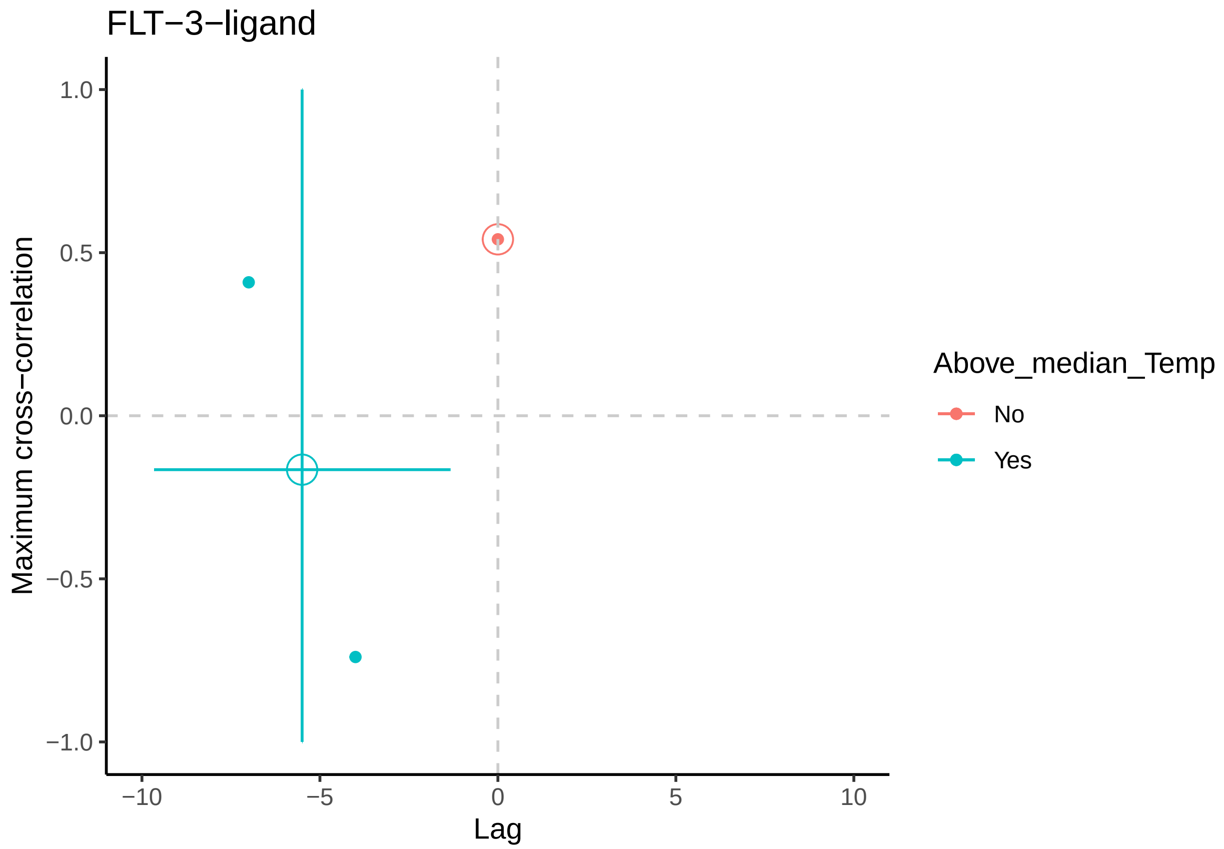
FLT−3−ligand
Maximum cross−correlation
1.0
0.5
0.0
−0.5
−1.0
−10
−5
0
5
10
Lag
Above_median_Temp
No
Yes

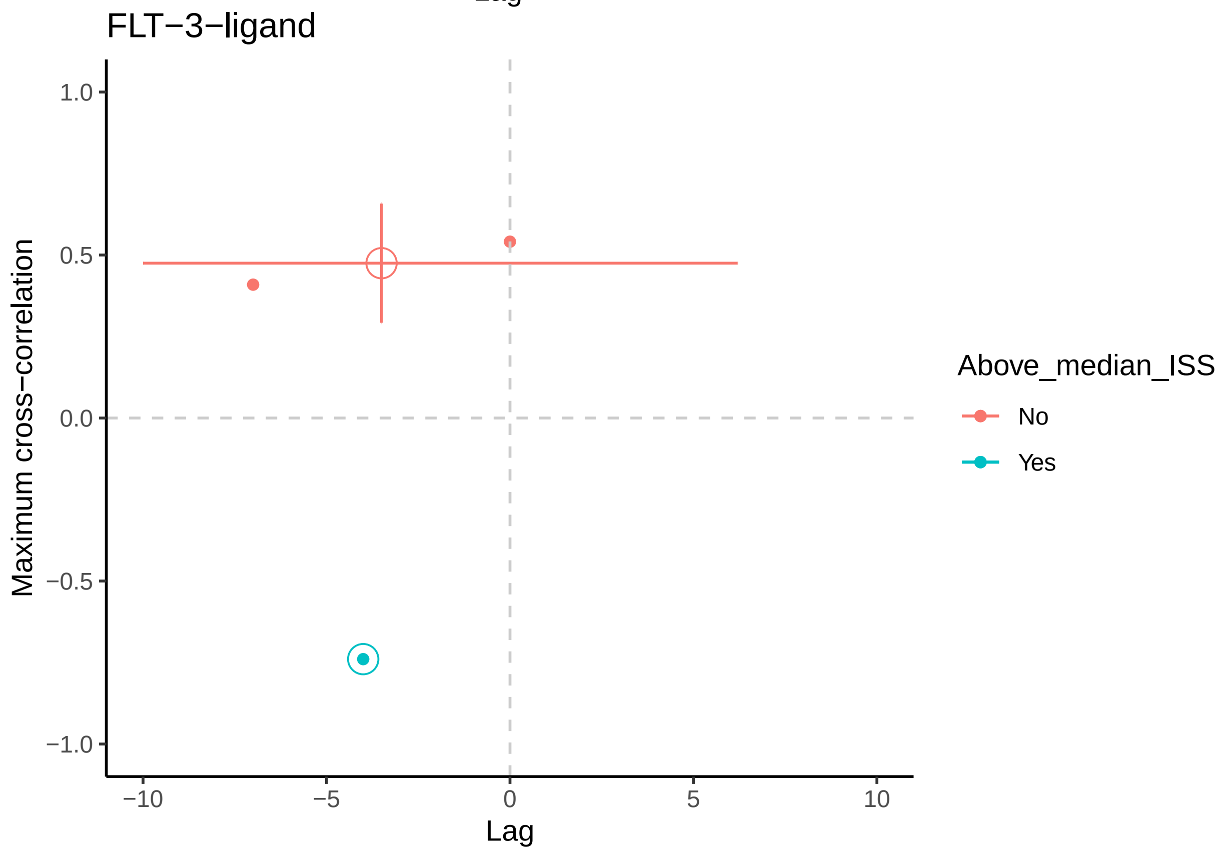
FLT−3−ligand
Maximum cross−correlation
1.0
0.5
0.0
−0.5
−1.0
−10
−5
0
5
10
Lag
Above_median_ISS
No
Yes

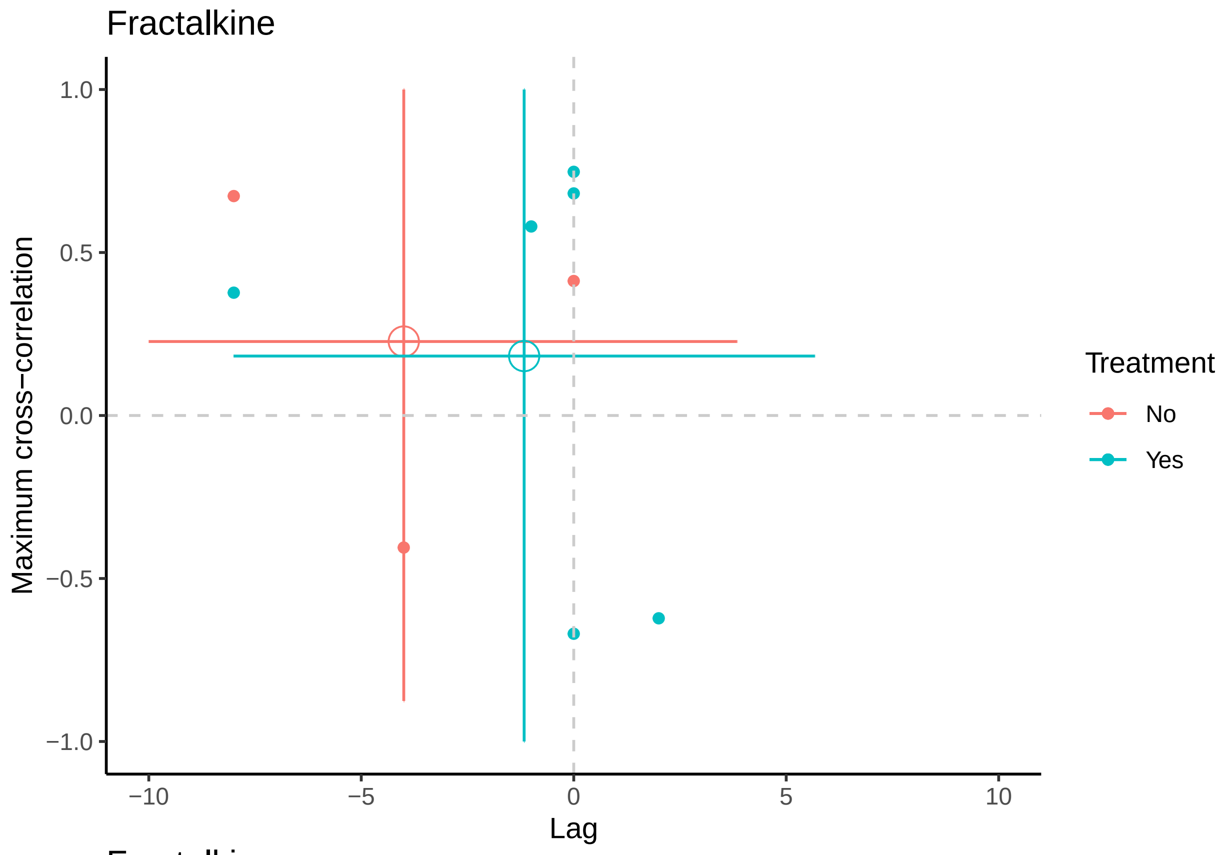
Fractalkine
Maximum cross−correlation
Lag
Treatment
No
Yes
1.0
0.5
0.0
−0.5
−1.0
−10
−5
0
5
10

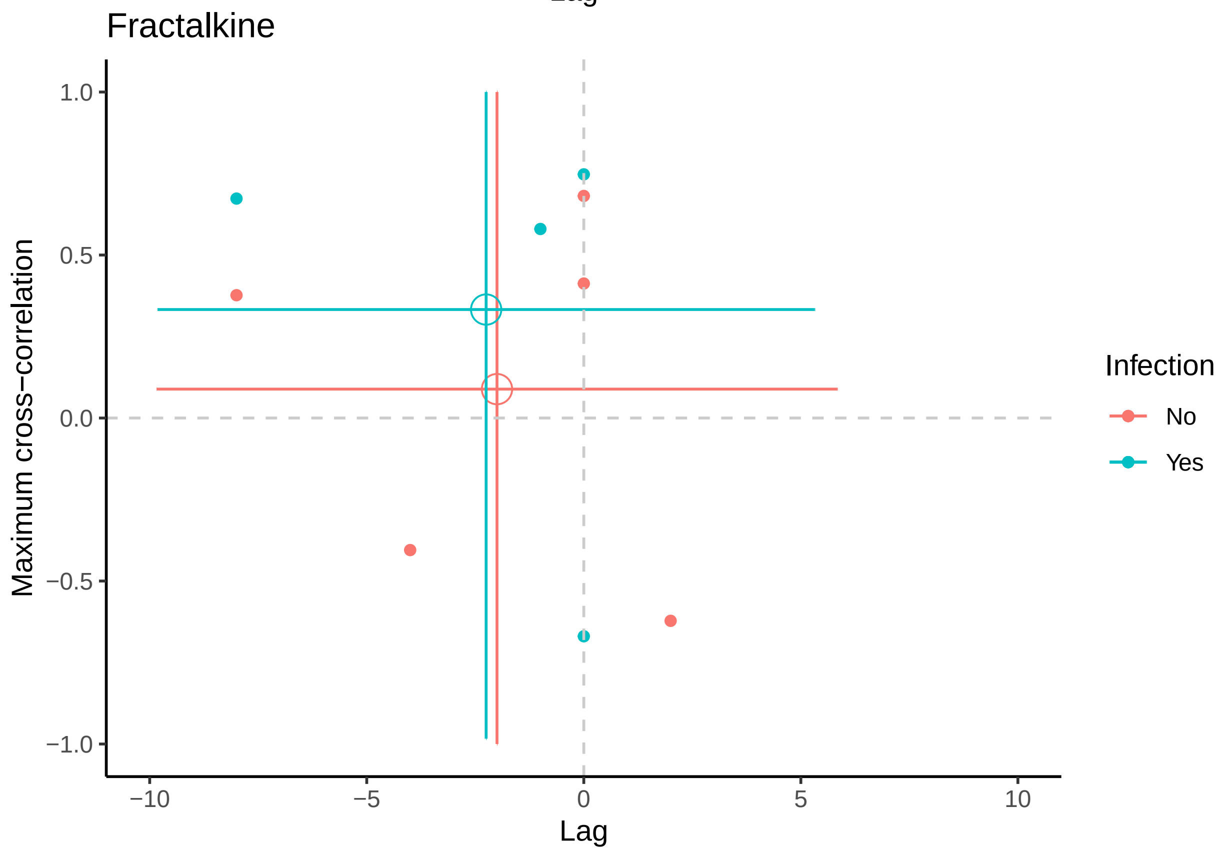
Fractalkine
Maximum cross−correlation
Lag
Infection
No
Yes
1.0
0.5
0.0
−0.5
−1.0
−10
−5
0
5
10

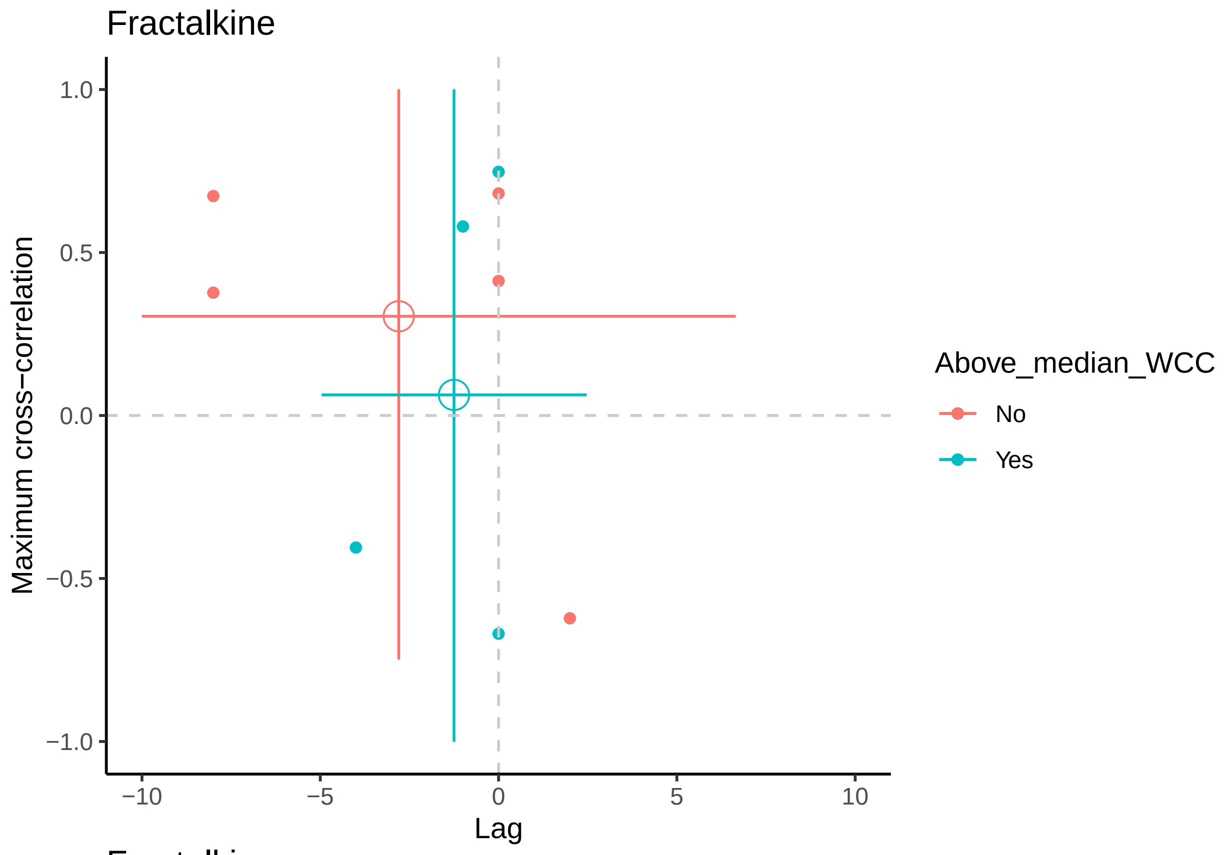
Fractalkine
Maximum cross−correlation
Lag
1.0
0.5
0.0
−0.5
−1.0
−10
−5
0
5
10
Above_median_WCC
No
Yes

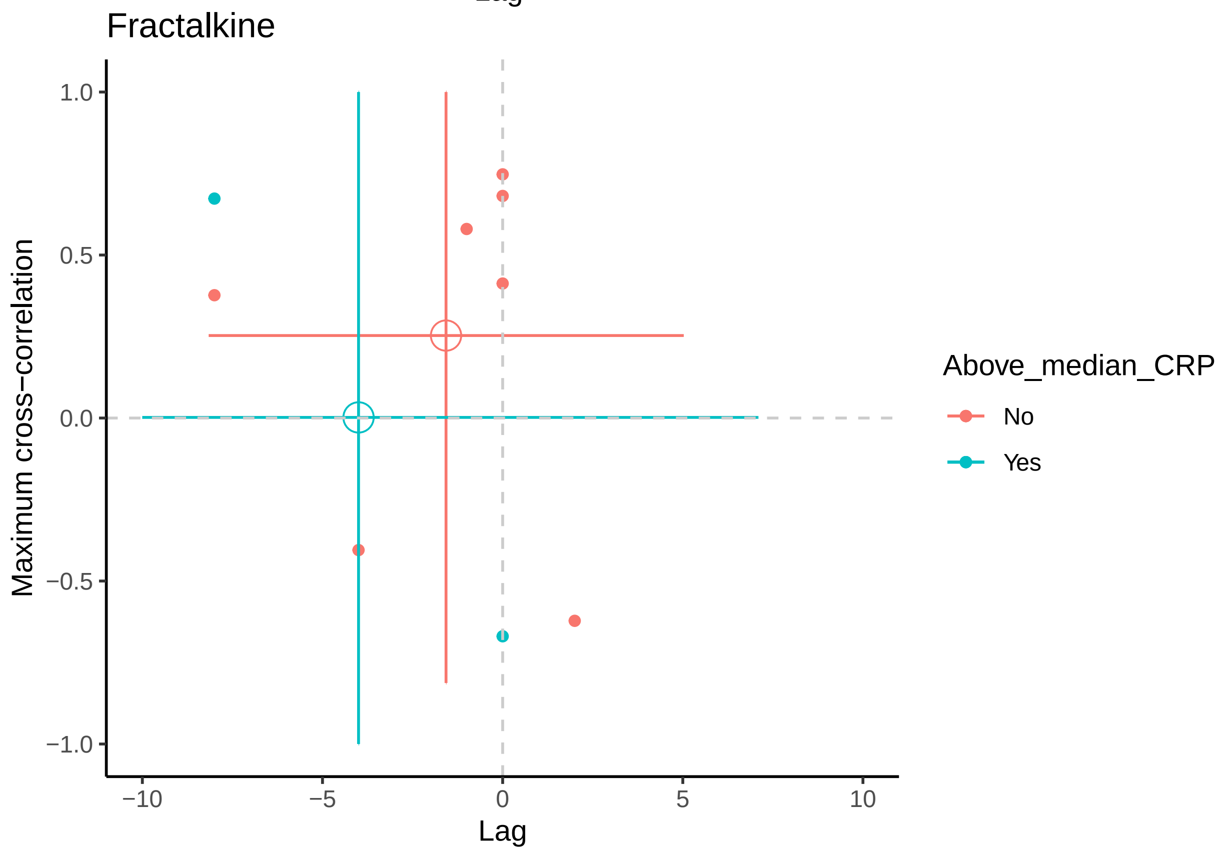
Fractalkine
Maximum cross−correlation
Lag
1.0
0.5
0.0
−0.5
−1.0
−10
−5
0
5
10
Above_median_CRP
No
Yes

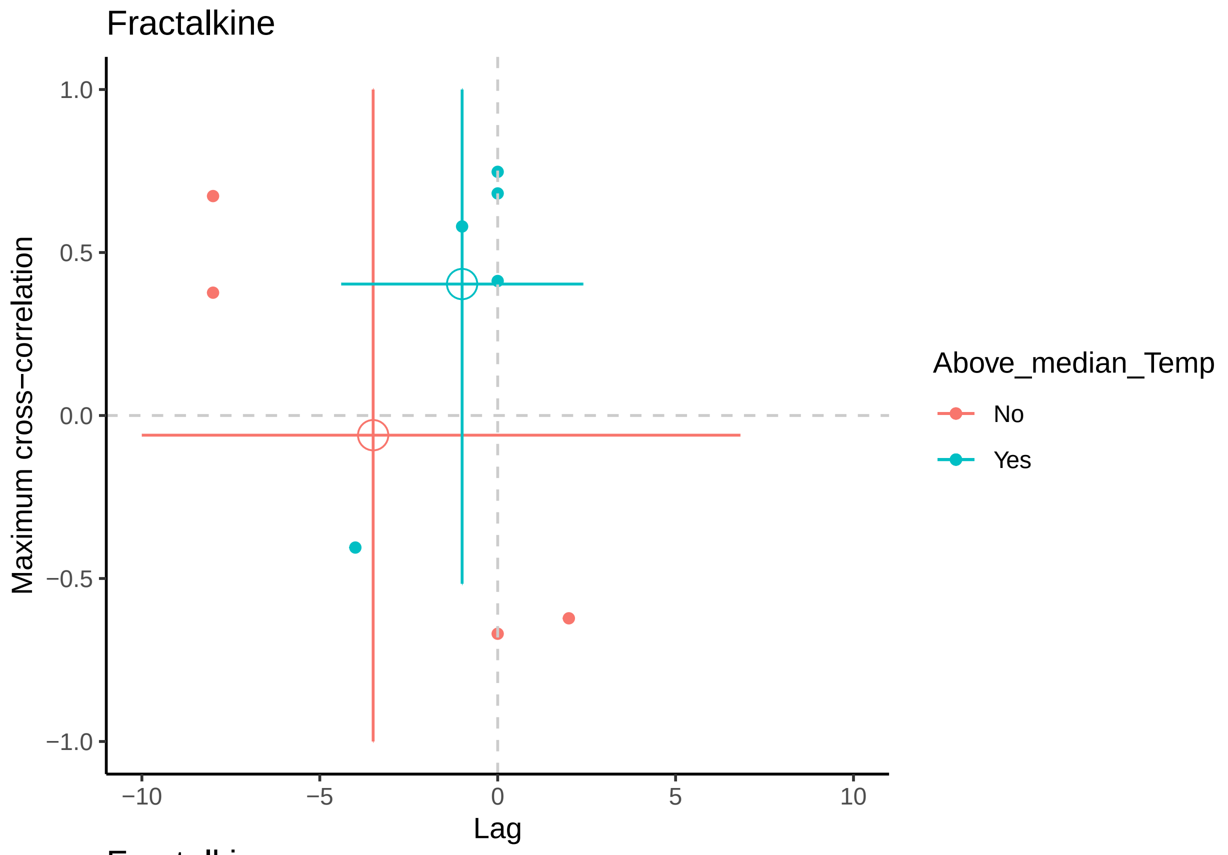
Fractalkine
Maximum cross−correlation
Lag
1.0
0.5
0.0
−0.5
−1.0
−10
−5
0
5
10
Above_median_Temp
No
Yes

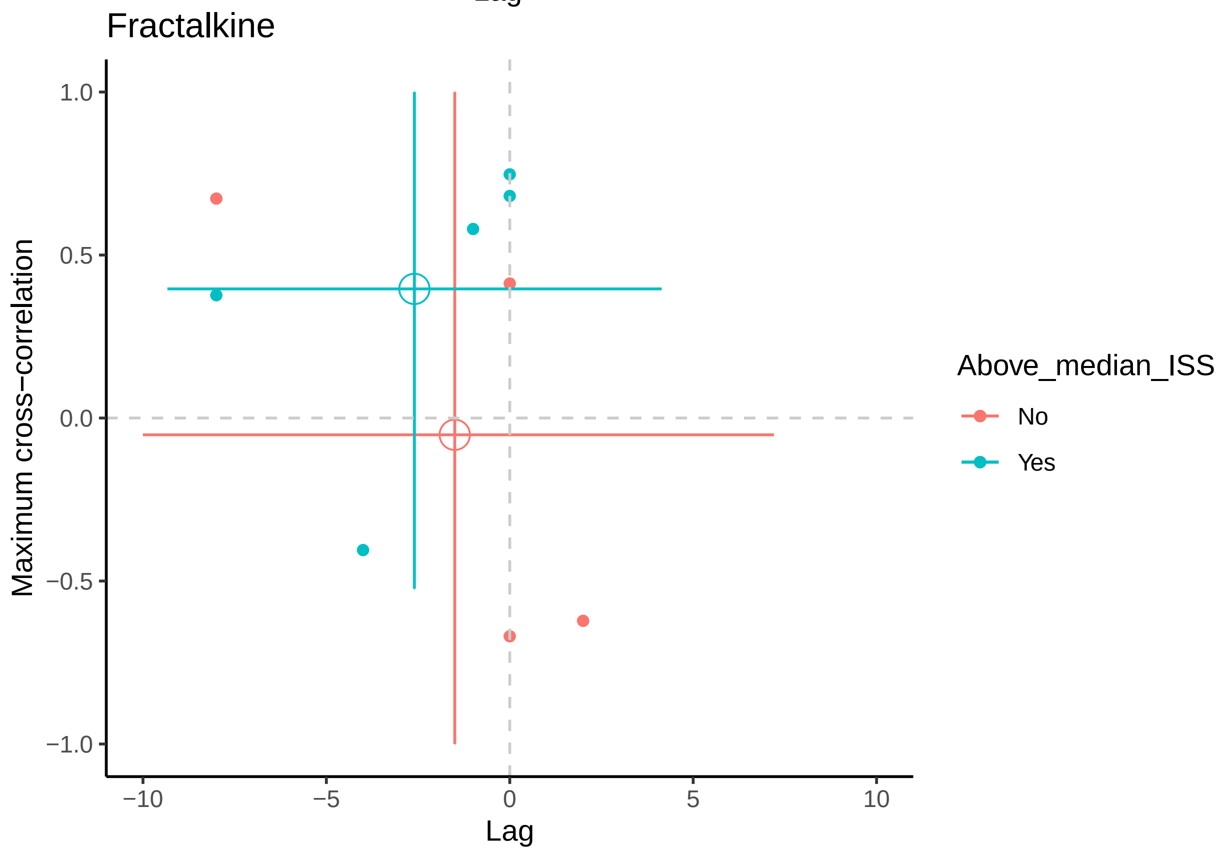
Fractalkine
Maximum cross−correlation
Lag
1.0
0.5
0.0
−0.5
−1.0
−10
−5
0
5
10
Above_median_ISS
No
Yes

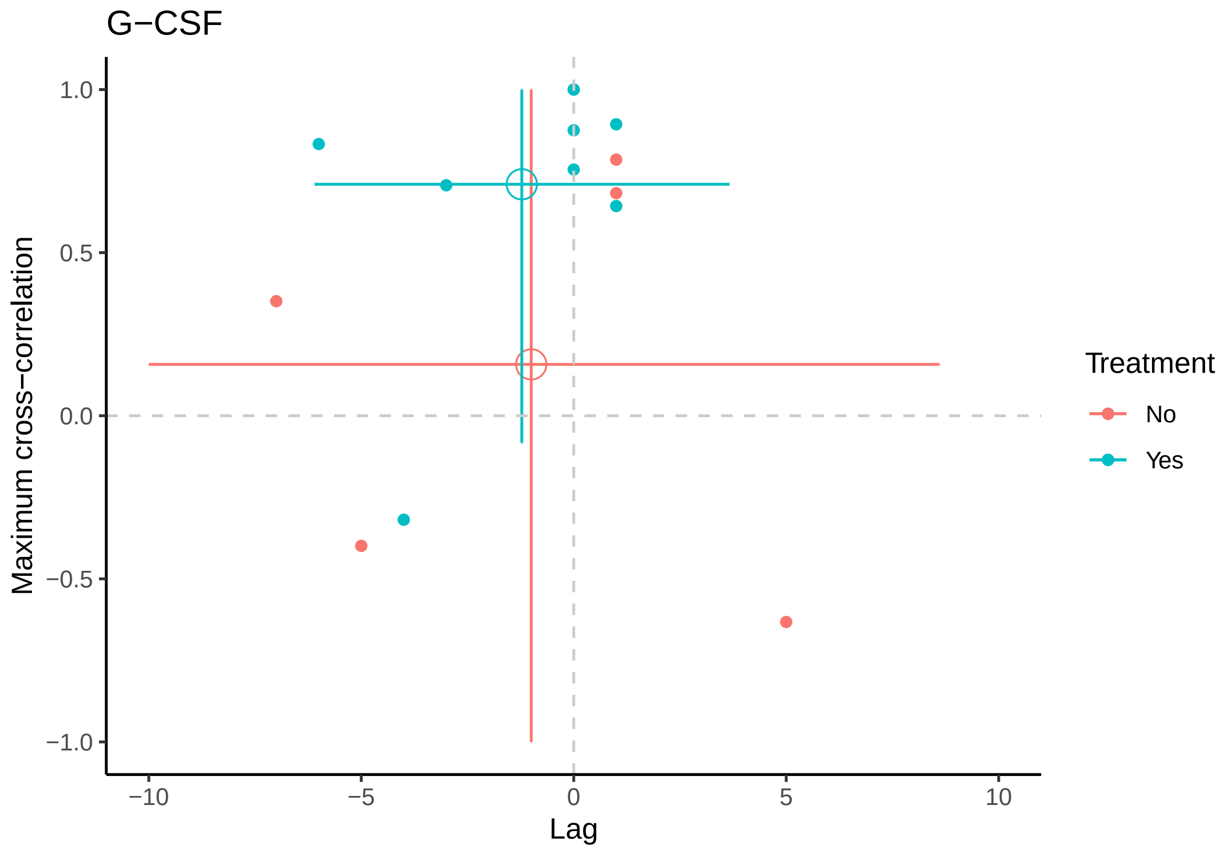
G−CSF
Maximum cross−correlation
Lag
Treatment
No
Yes
1.0
0.5
0.0
−0.5
−1.0
−10
−5
0
5
10

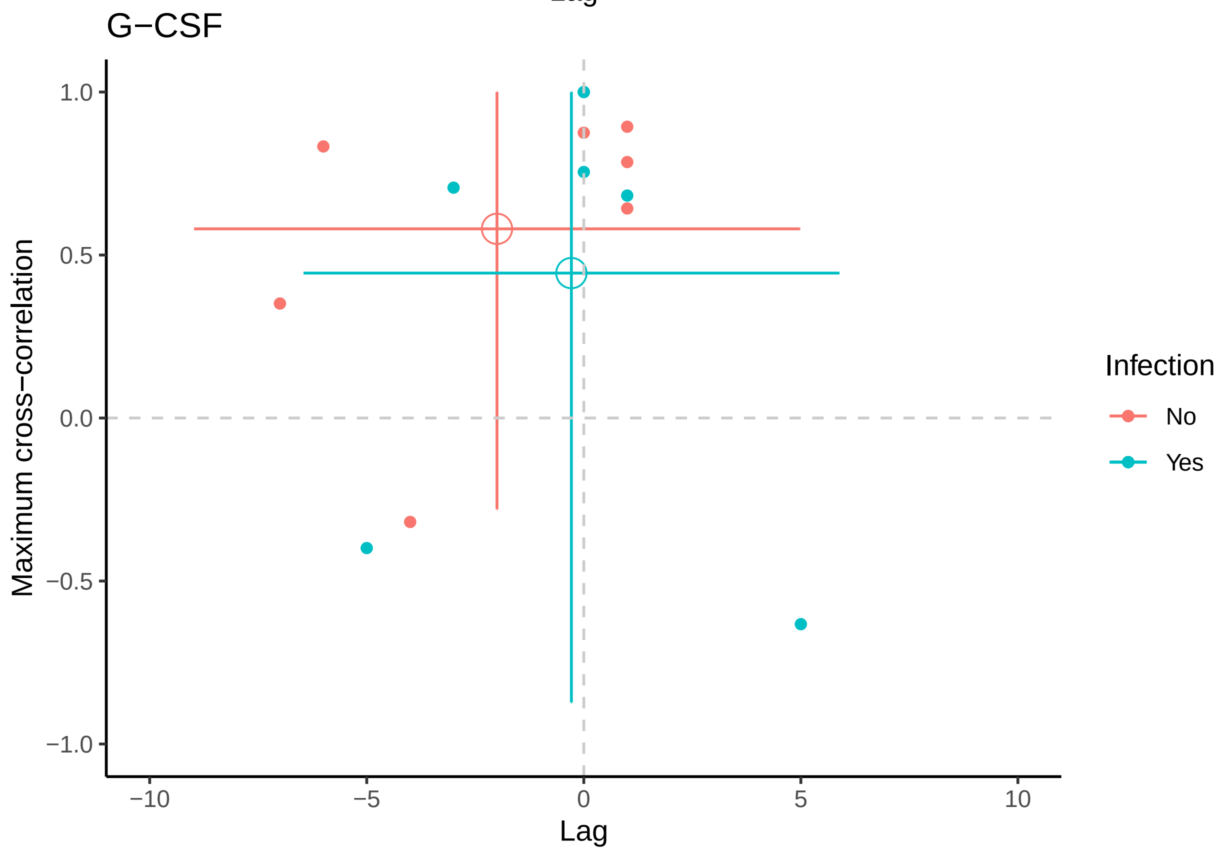
G−CSF
Maximum cross−correlation
Lag
Infection
No
Yes
1.0
0.5
0.0
−0.5
−1.0
−10
−5
0
5
10

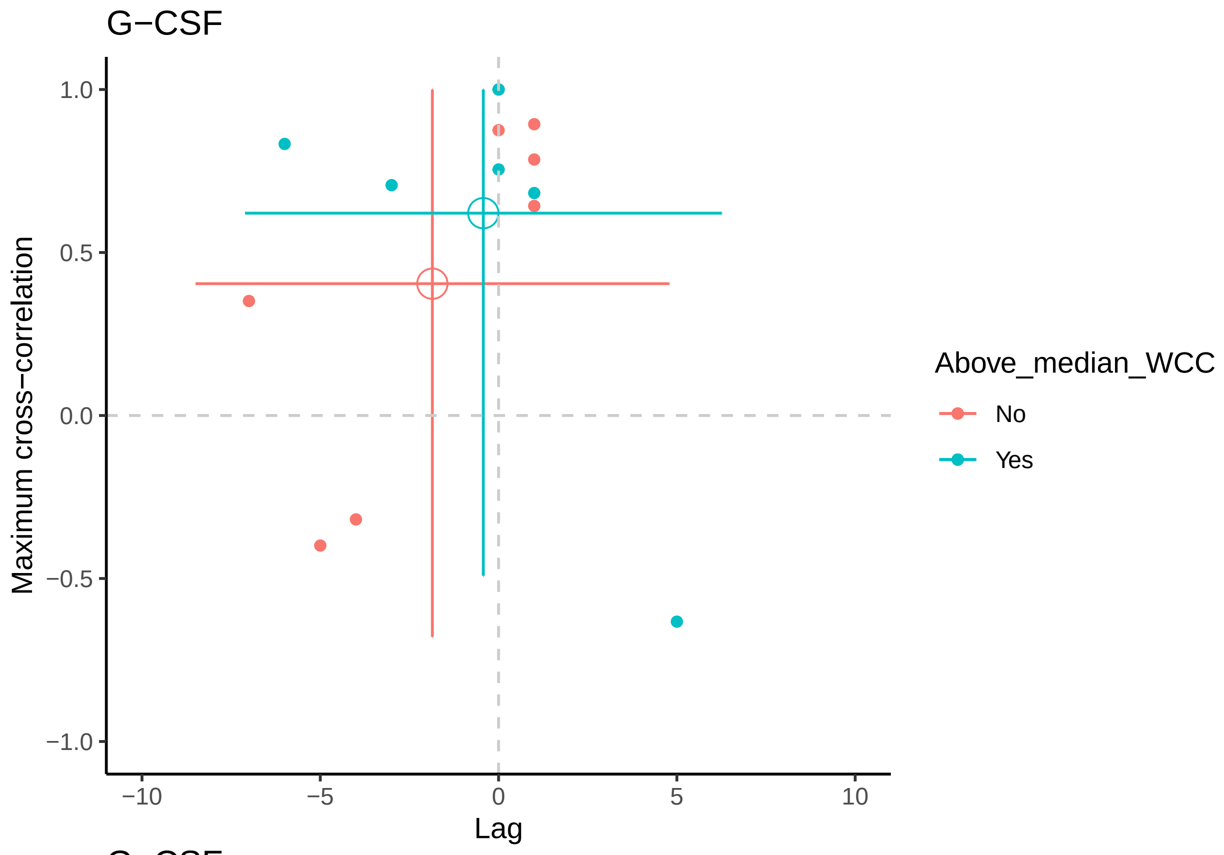
G−CSF
Maximum cross−correlation
Lag
Above_median_WCC
No
Yes
1.0
0.5
0.0
−0.5
−1.0
−10
−5
0
5
10

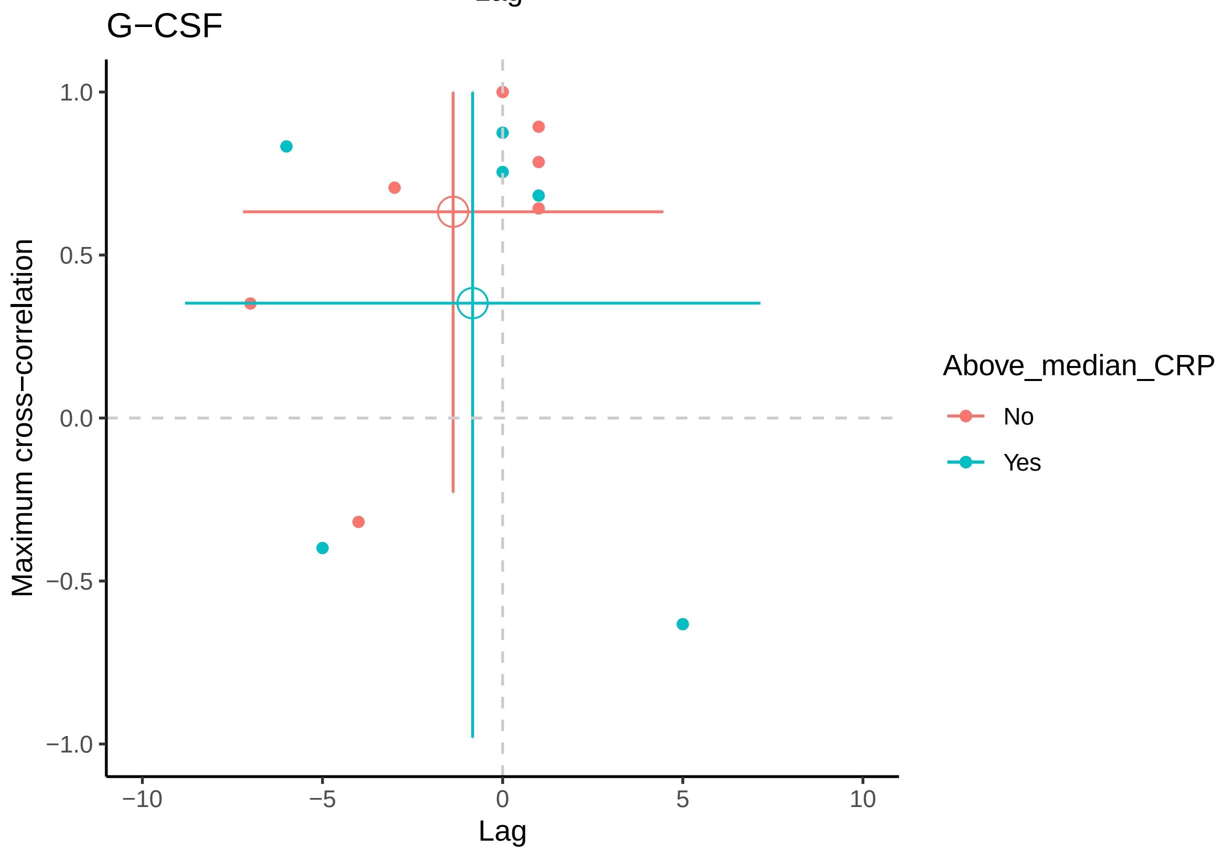
G−CSF
Maximum cross−correlation
Lag
Above_median_CRP
No
Yes
1.0
0.5
0.0
−0.5
−1.0
−10
−5
0
5
10

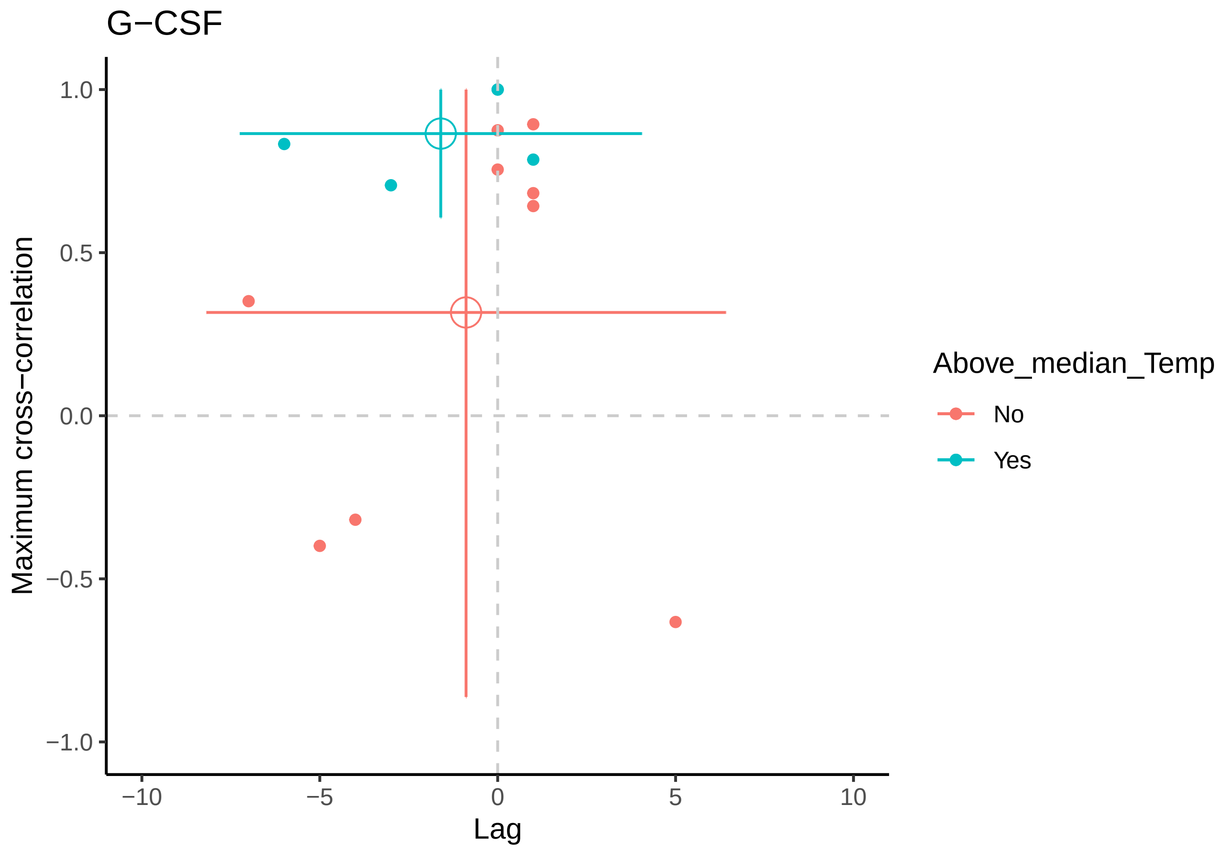
G−CSF
Maximum cross−correlation
1.0
0.5
0.0
−0.5
−1.0
−10
−5
0
5
10
Lag
Above_median_Temp
No
Yes

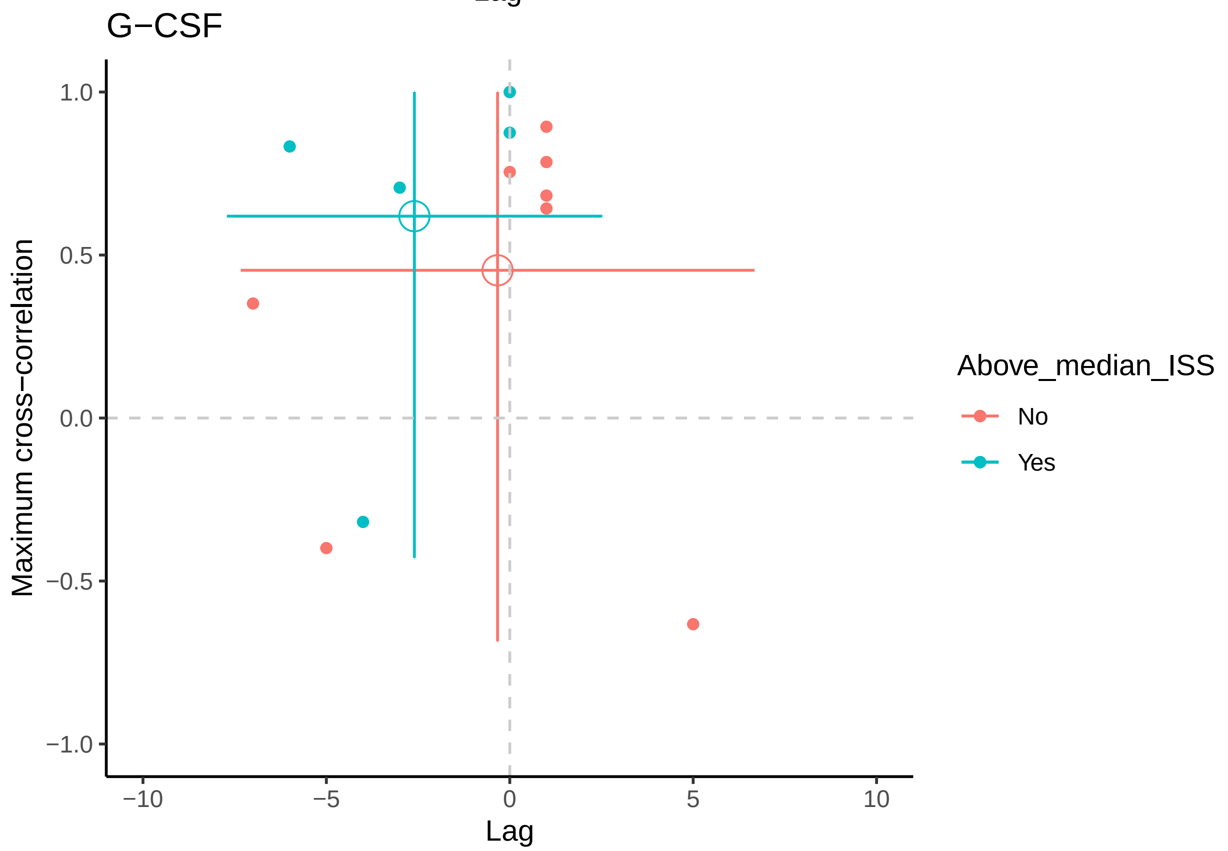
G−CSF
Maximum cross−correlation
1.0
0.5
0.0
−0.5
−1.0
−10
−5
0
5
10
Lag
Above_median_ISS
No
Yes

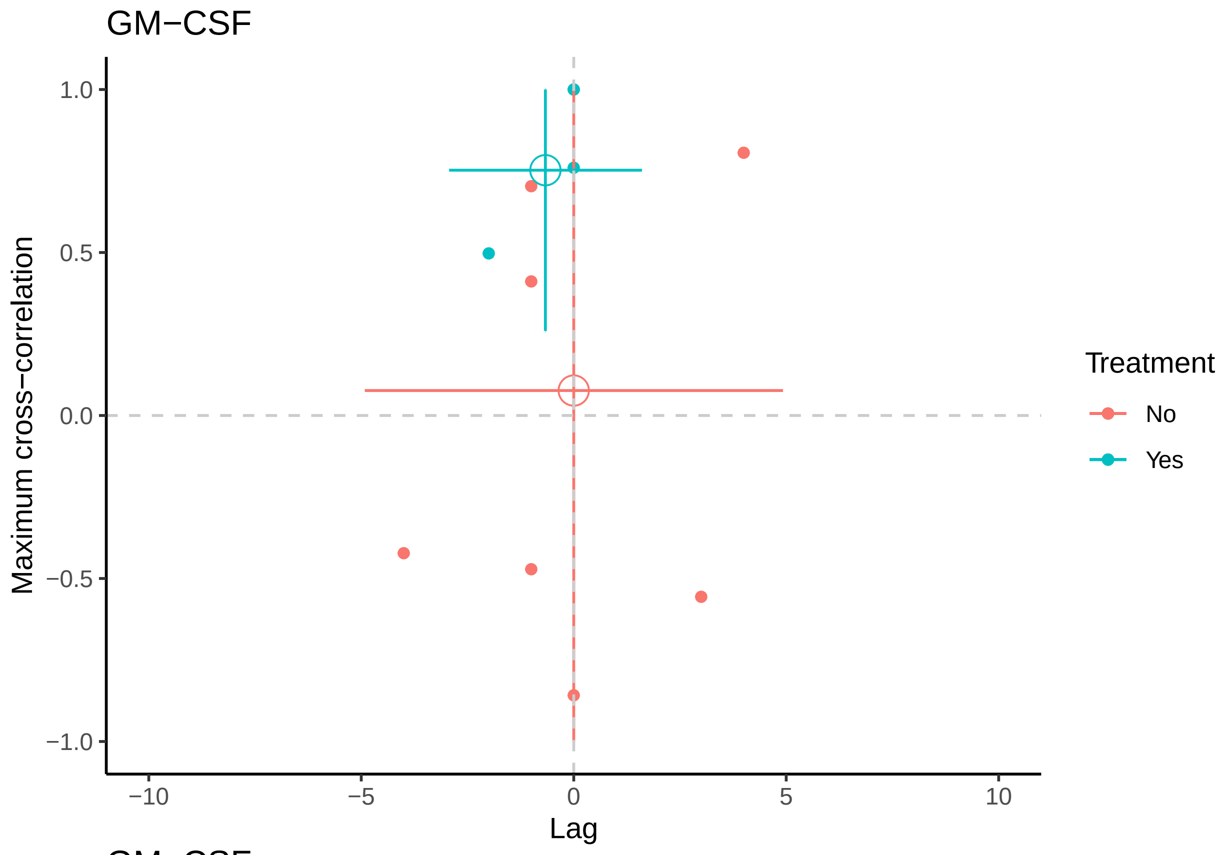
GM−CSF
Maximum cross−correlation
1.0
0.5
0.0
−0.5
−1.0
−10
−5
0
5
10
Lag
Treatment
No
Yes

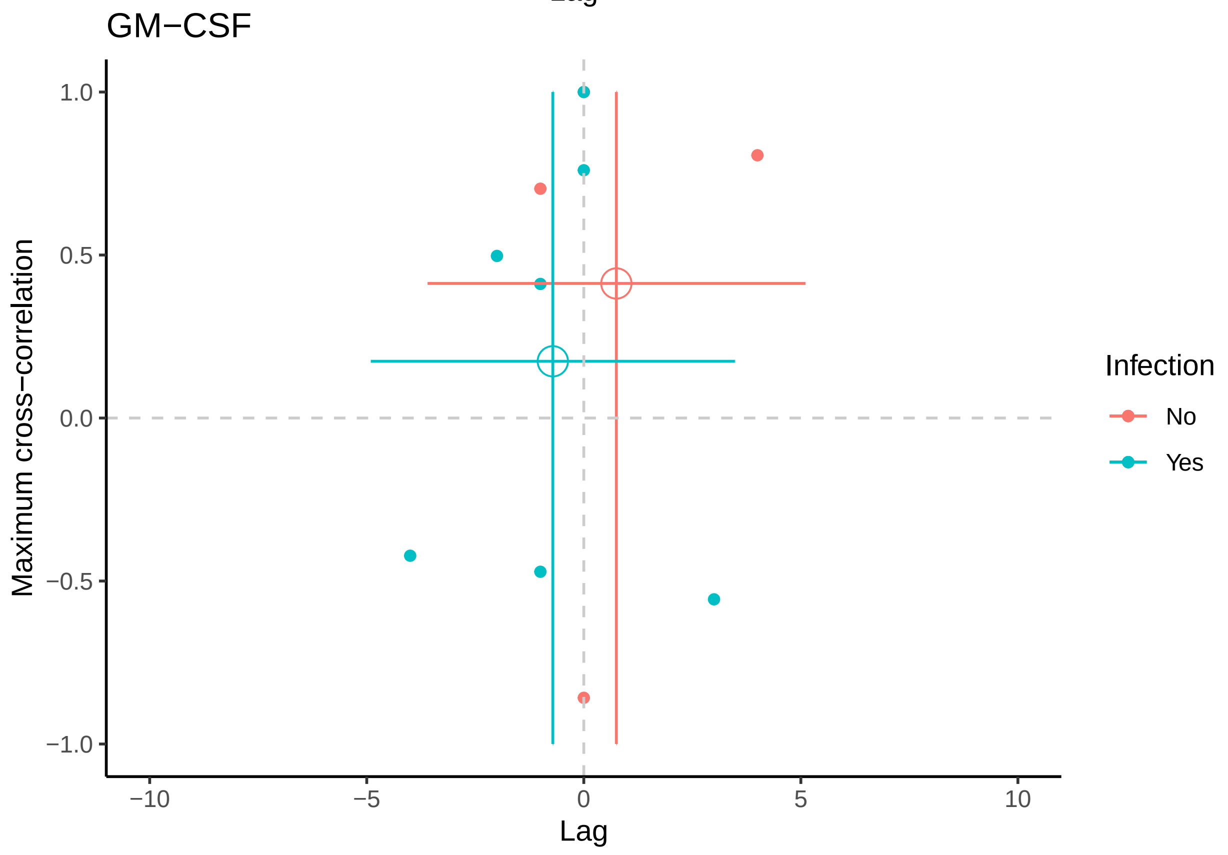
GM−CSF
Maximum cross−correlation
1.0
0.5
0.0
−0.5
−1.0
−10
−5
0
5
10
Lag
Infection
No
Yes

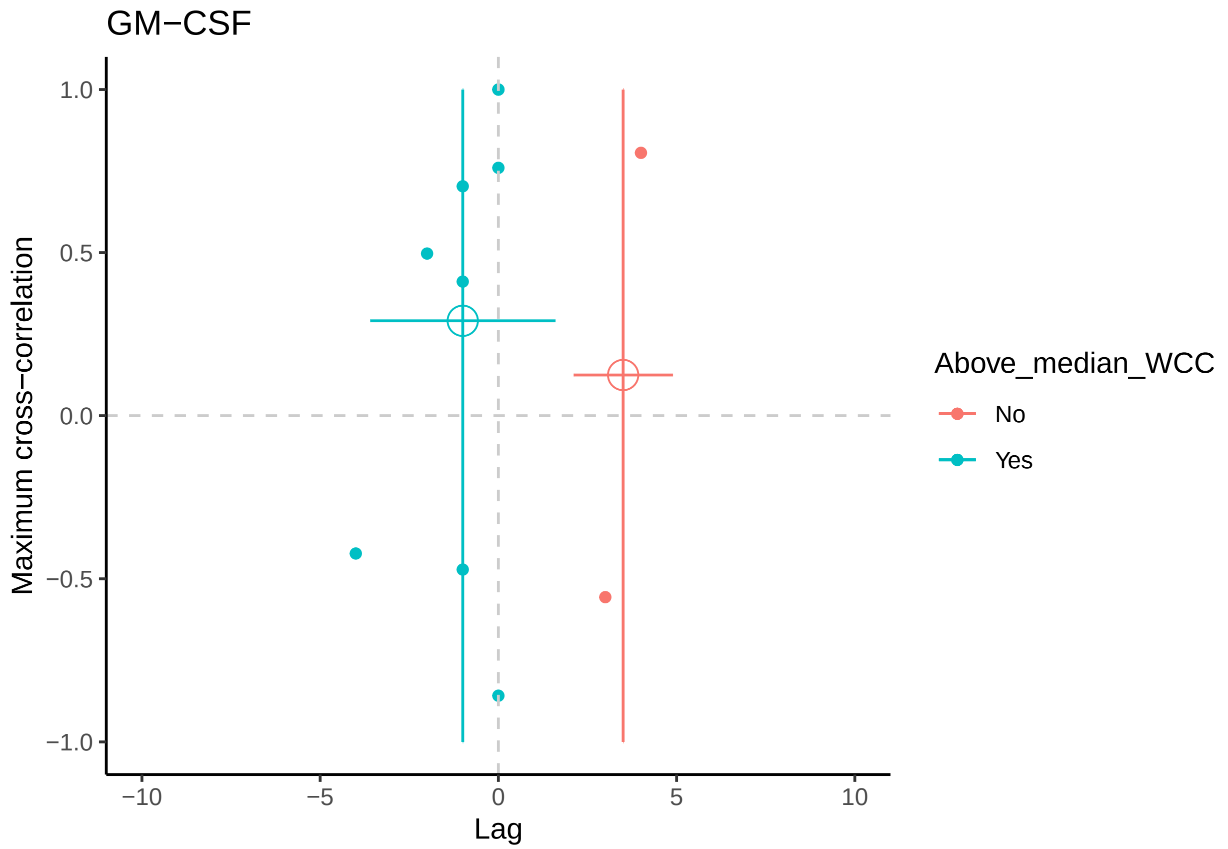
GM−CSF
Maximum cross−correlation
Lag
Above_median_WCC
No
Yes

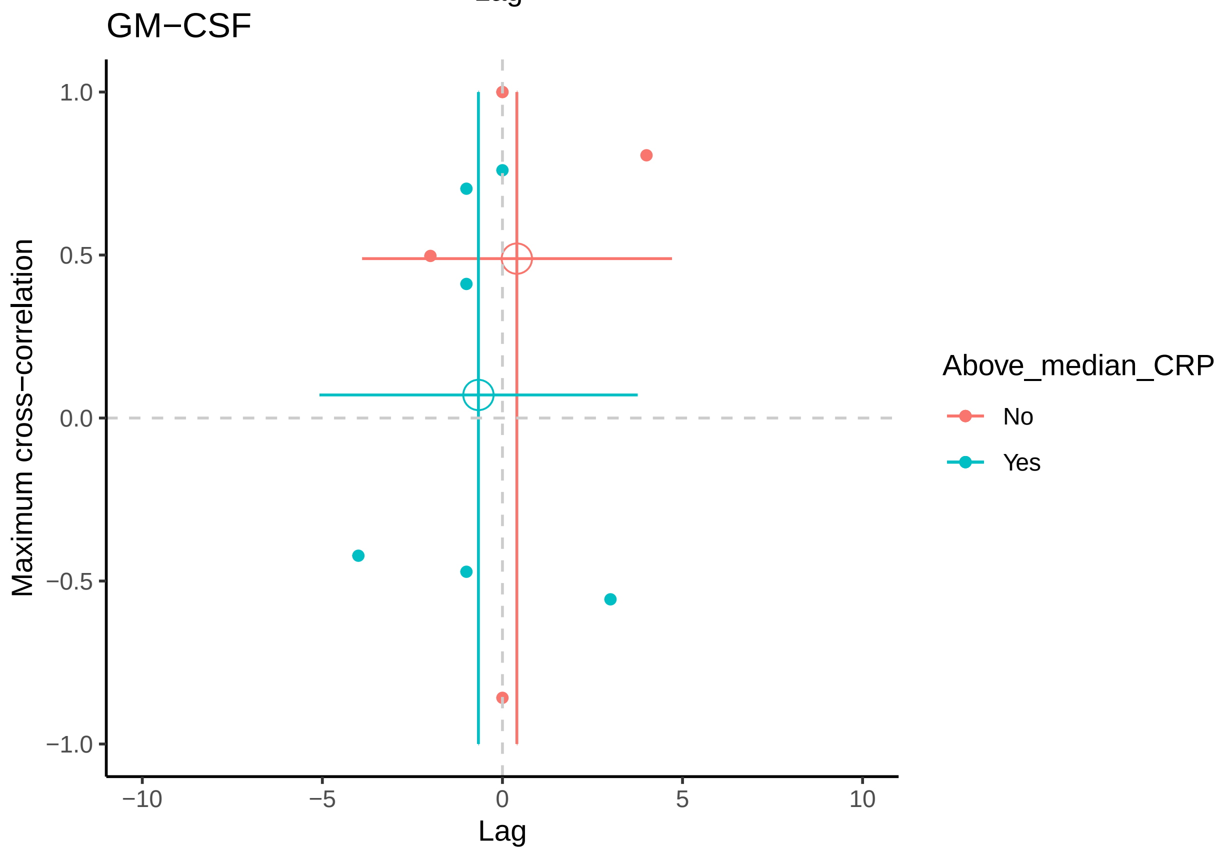
GM−CSF
Maximum cross−correlation
Lag
Above_median_CRP
No
Yes

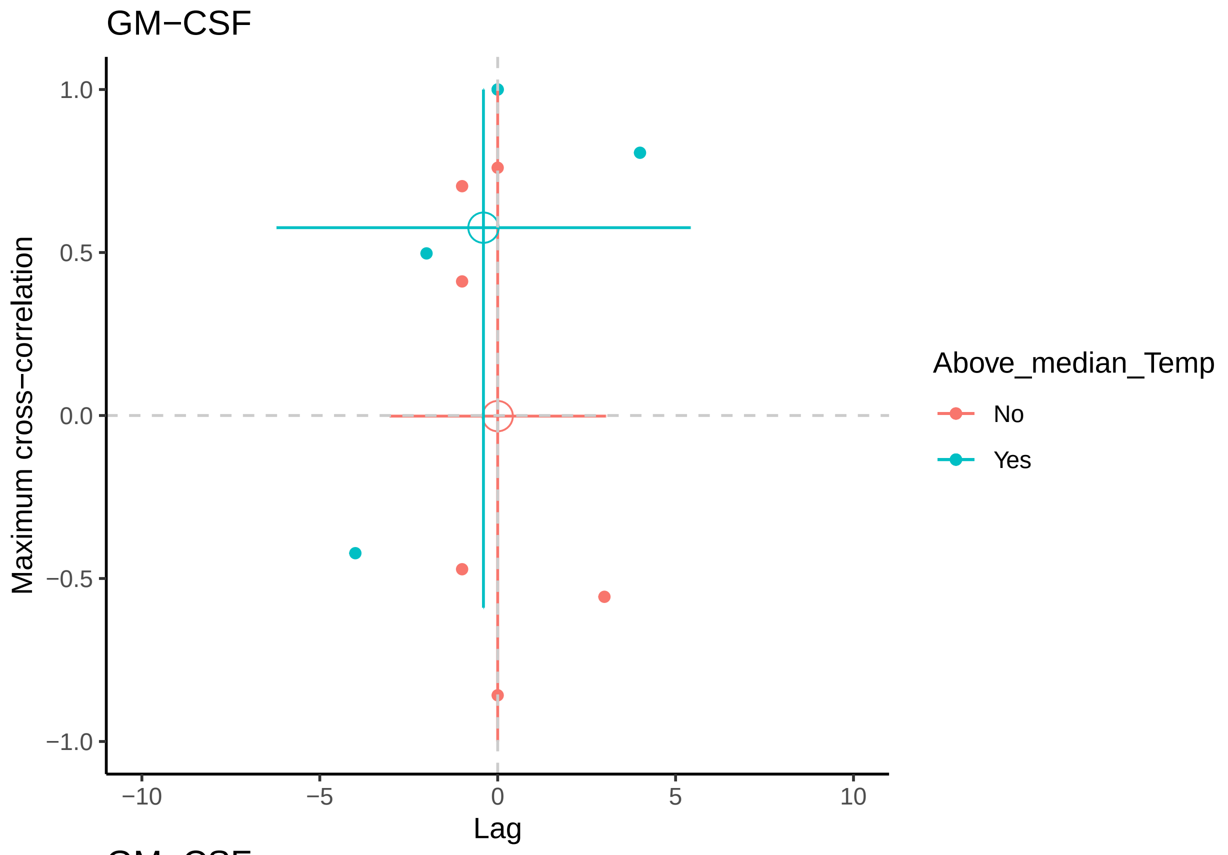
GM−CSF
Maximum cross−correlation
Lag
Above_median_Temp
No
Yes

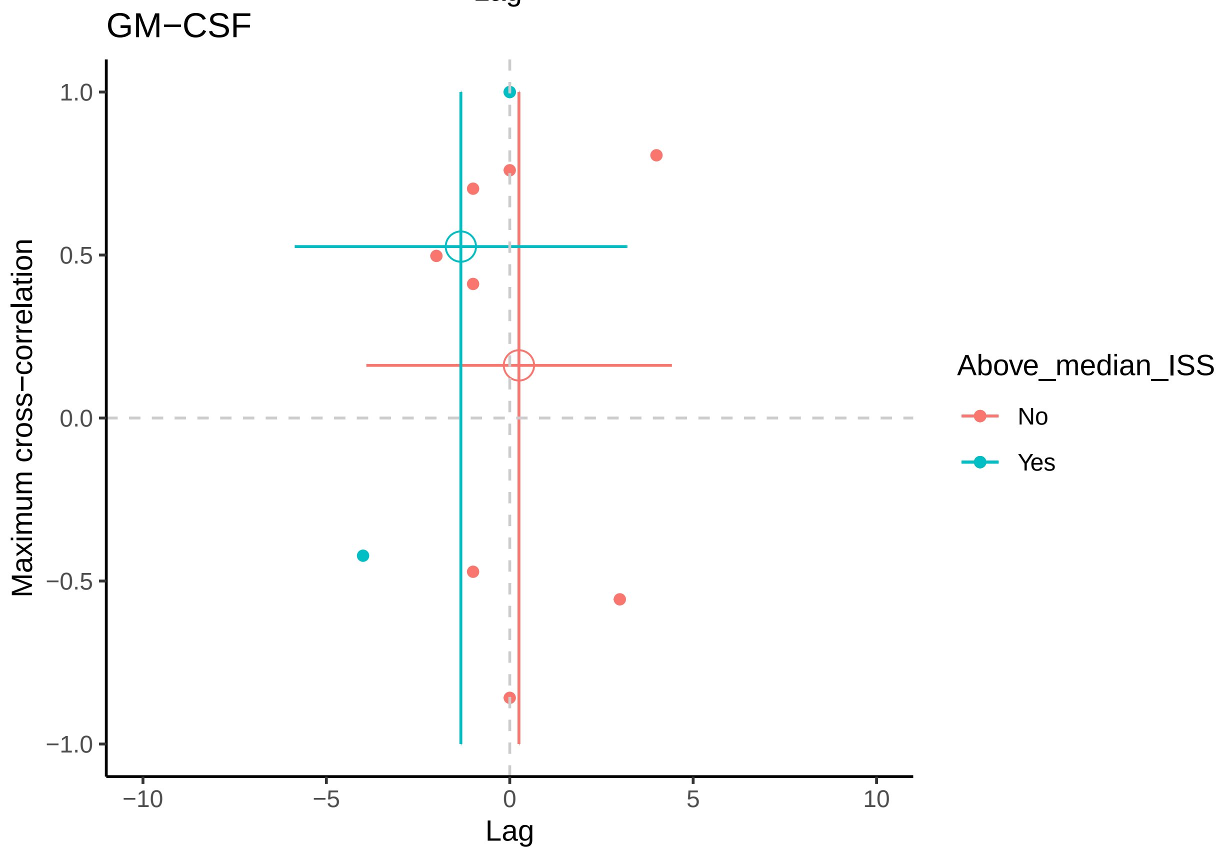
GM−CSF
Maximum cross−correlation
Lag
Above_median_ISS
No
Yes

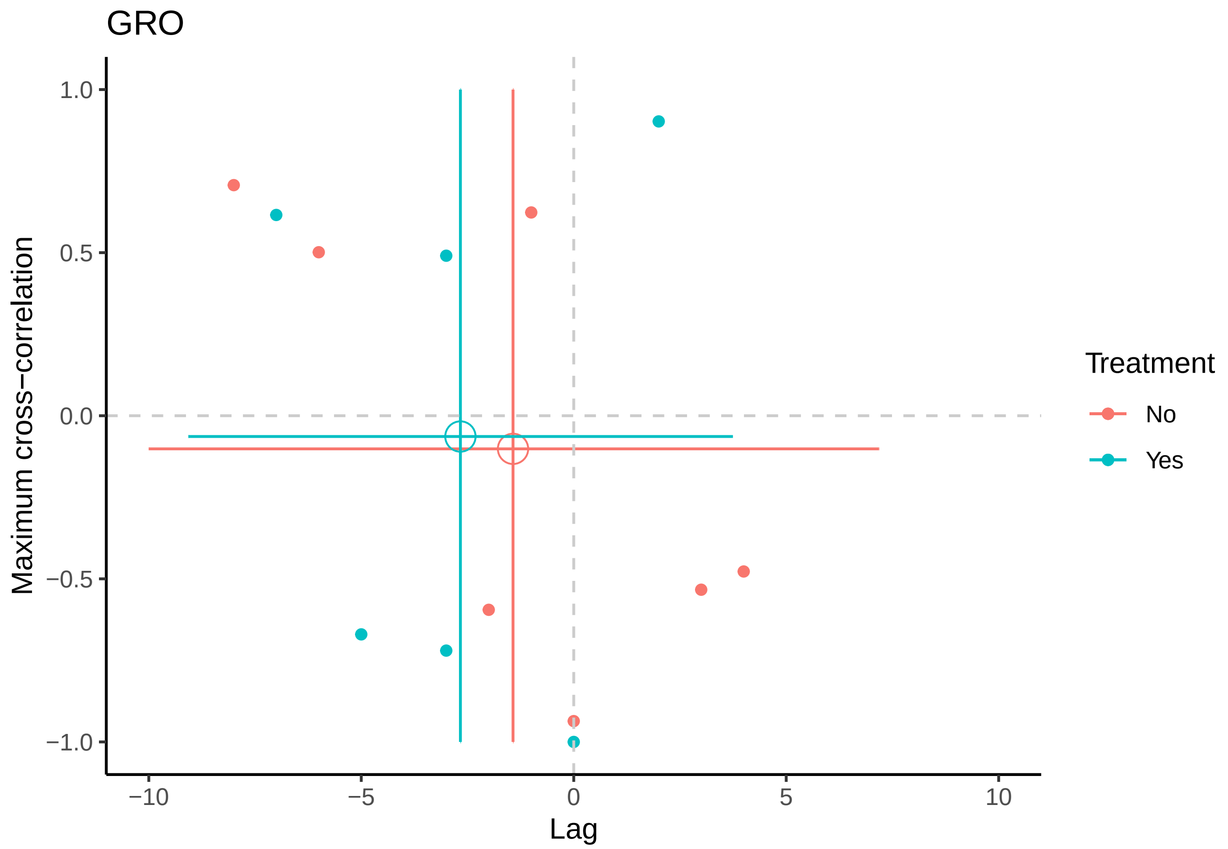
GRO
Maximum cross−correlation
Lag
Treatment
No
Yes
1.0
0.5
0.0
−0.5
−1.0
−10
−5
0
5
10

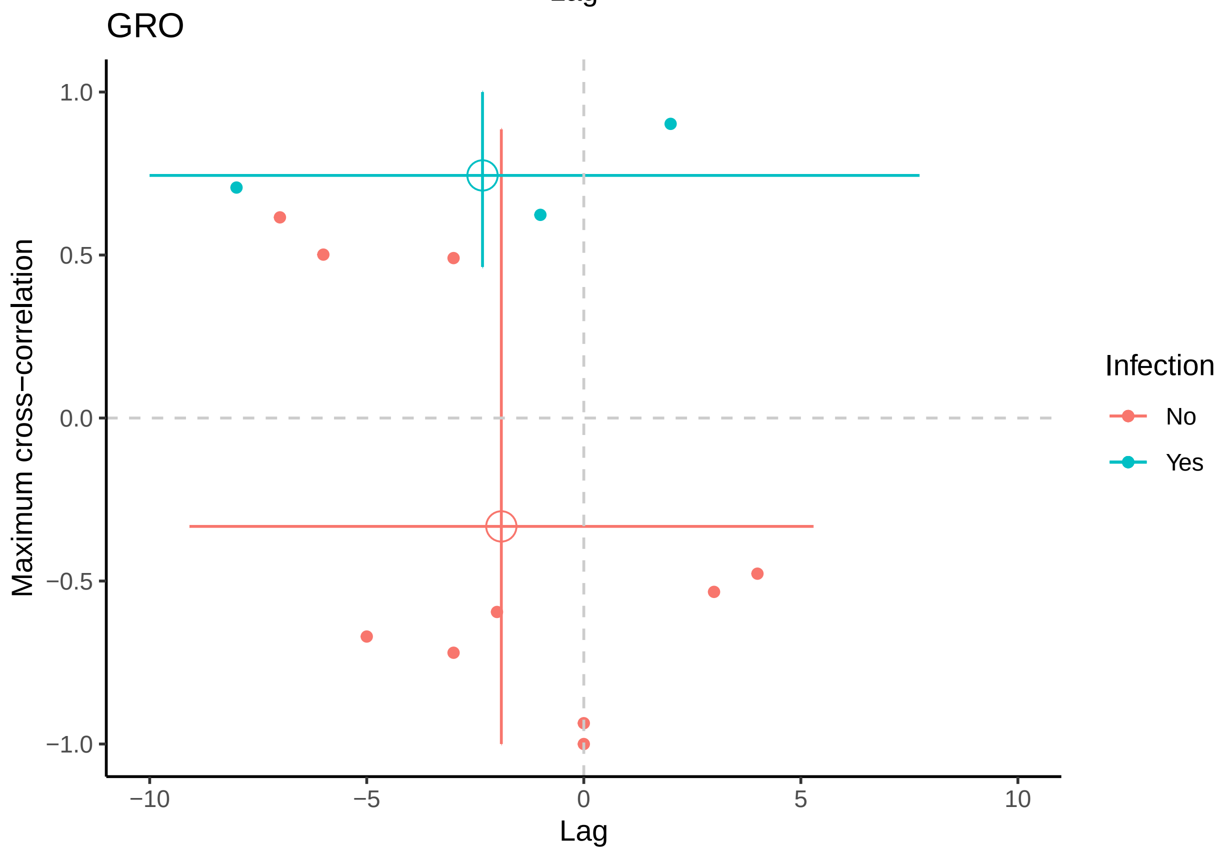
GRO
Maximum cross−correlation
Lag
Infection
No
Yes
1.0
0.5
0.0
−0.5
−1.0
−10
−5
0
5
10

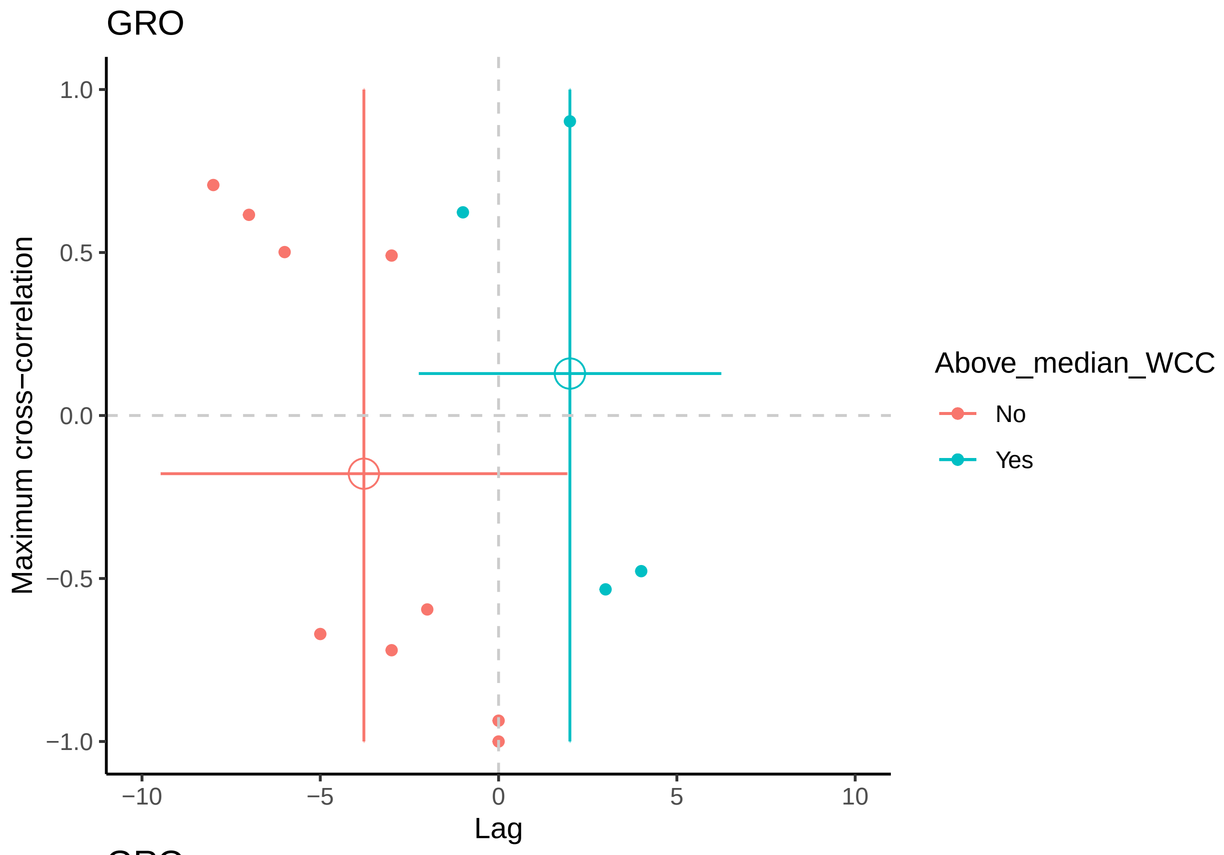
GRO
Maximum cross−correlation
1.0
0.5
0.0
−0.5
−1.0
−10
−5
0
5
10
Lag
Above_median_WCC
No
Yes

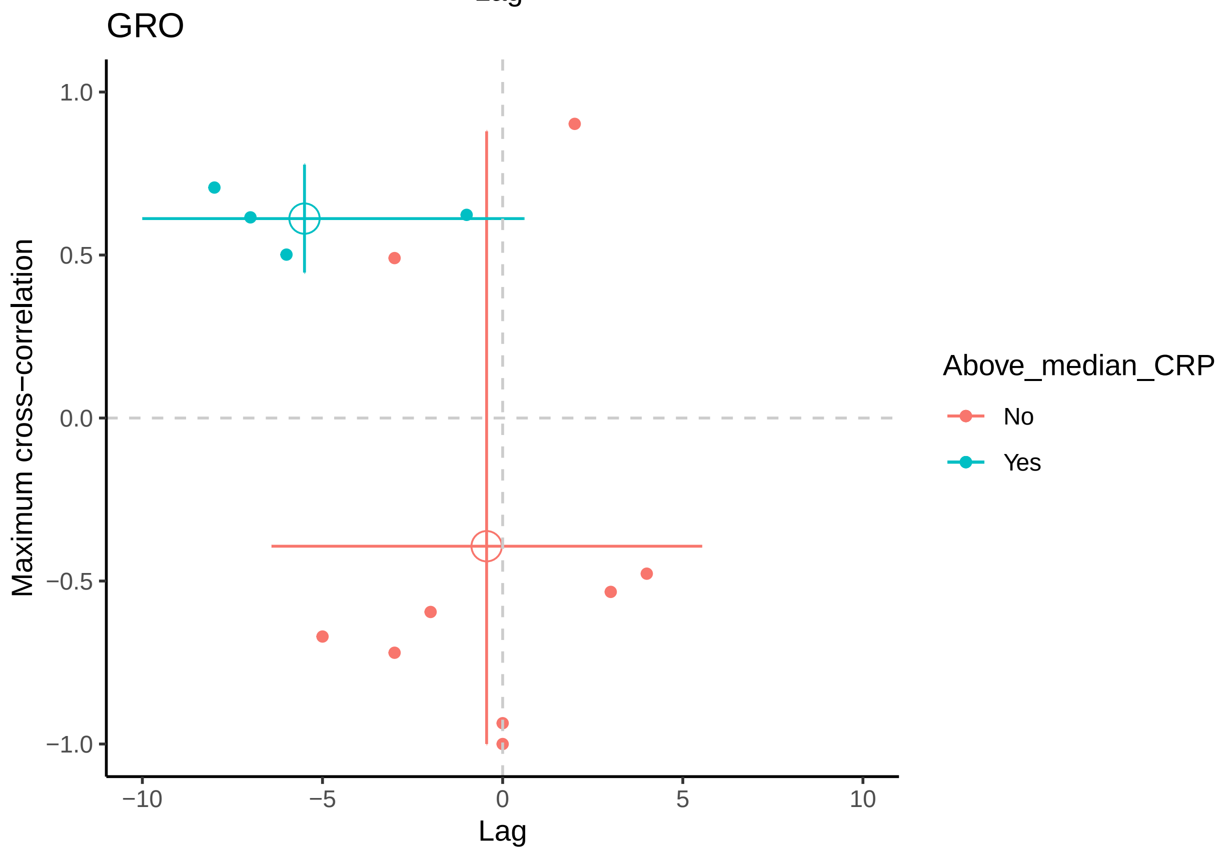
GRO
Maximum cross−correlation
1.0
0.5
0.0
−0.5
−1.0
−10
−5
0
5
10
Lag
Above_median_CRP
No
Yes

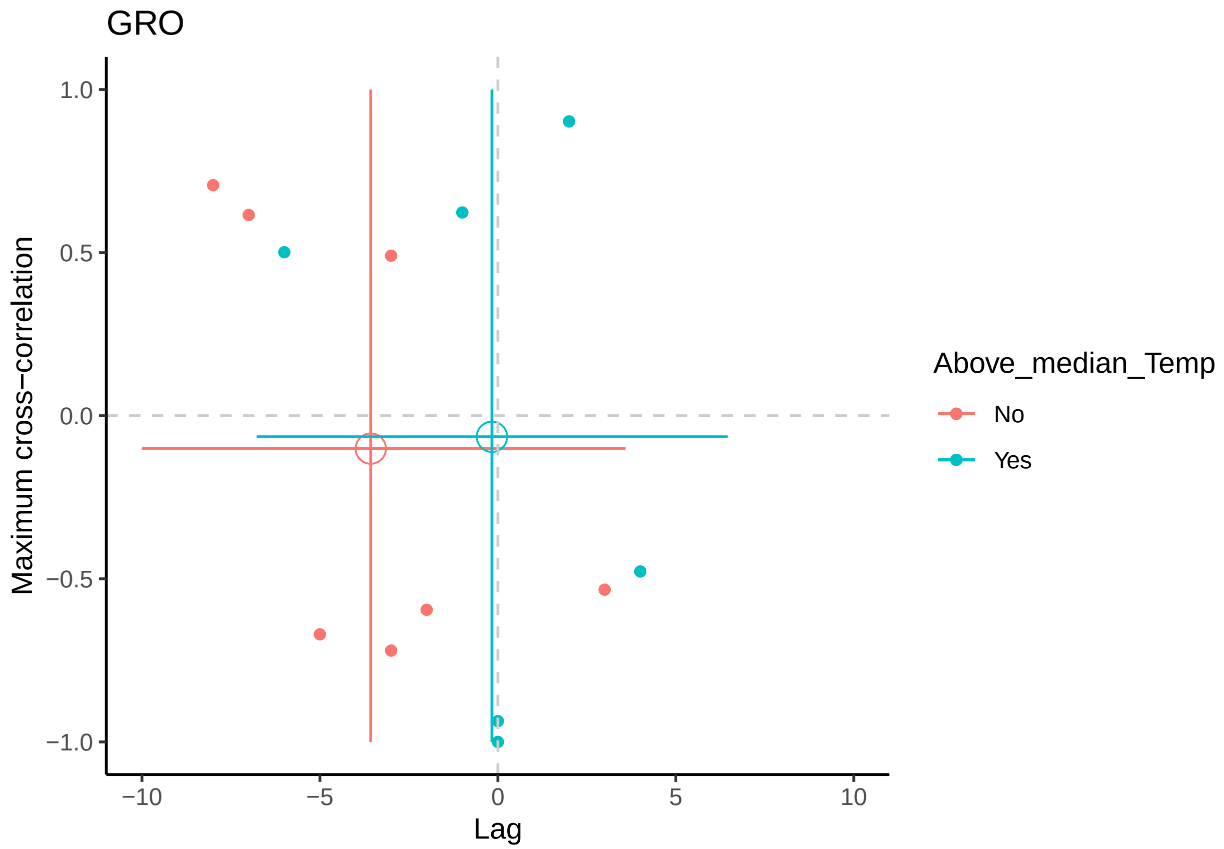
GRO
Maximum cross−correlation
1.0
0.5
0.0
−0.5
−1.0
−10
−5
0
5
10
Lag
Above_median_Temp
No
Yes

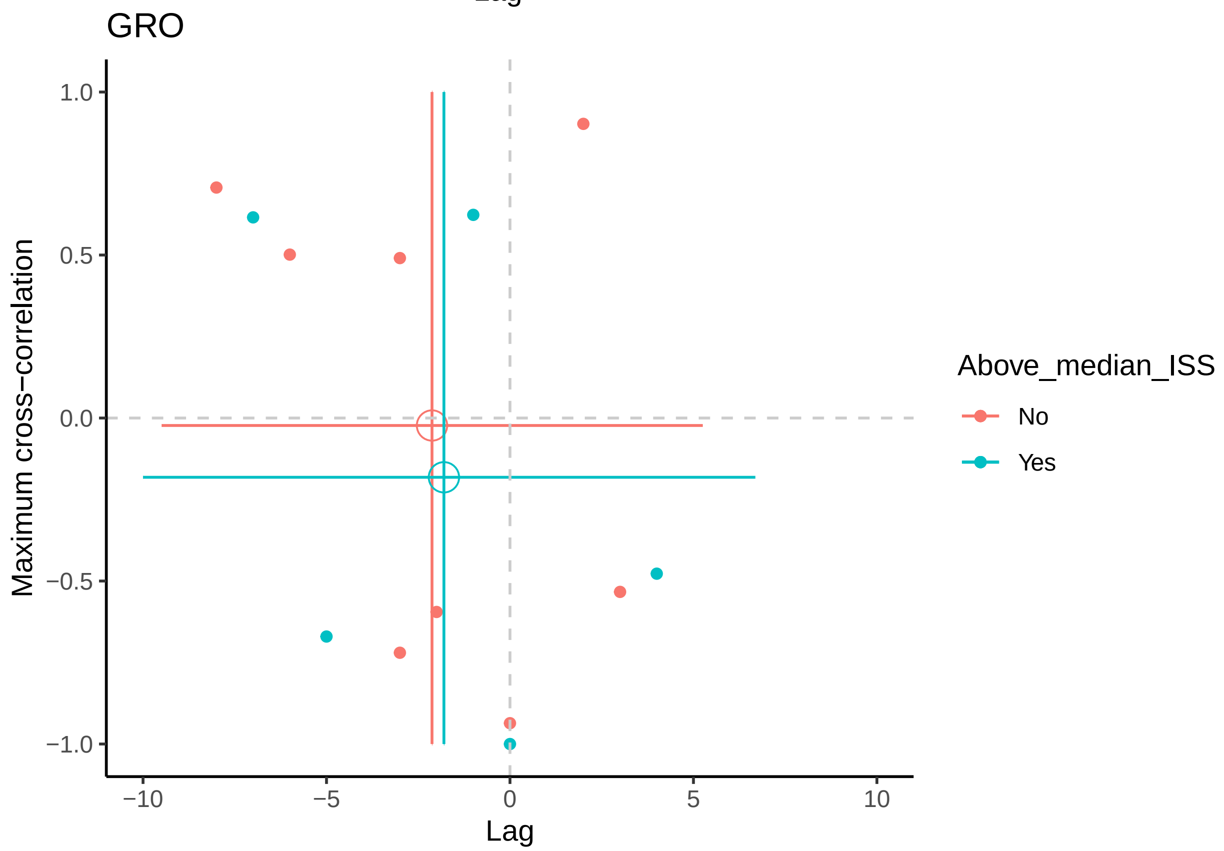
GRO
Maximum cross−correlation
1.0
0.5
0.0
−0.5
−1.0
−10
−5
0
5
10
Lag
Above_median_ISS
No
Yes

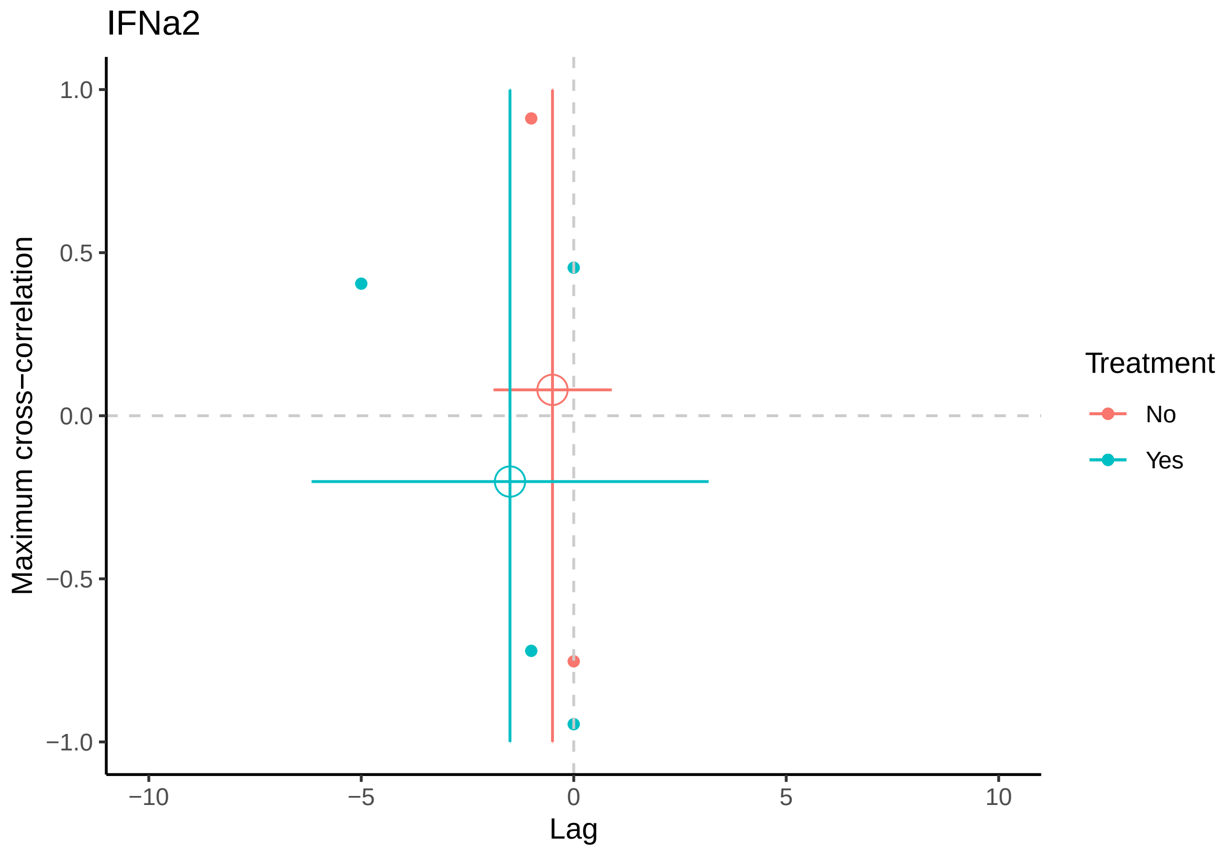
IFNa2
Maximum cross−correlation
1.0
0.5
0.0
−0.5
−1.0
−10
−5
0
5
10
Lag
Treatment
No
Yes

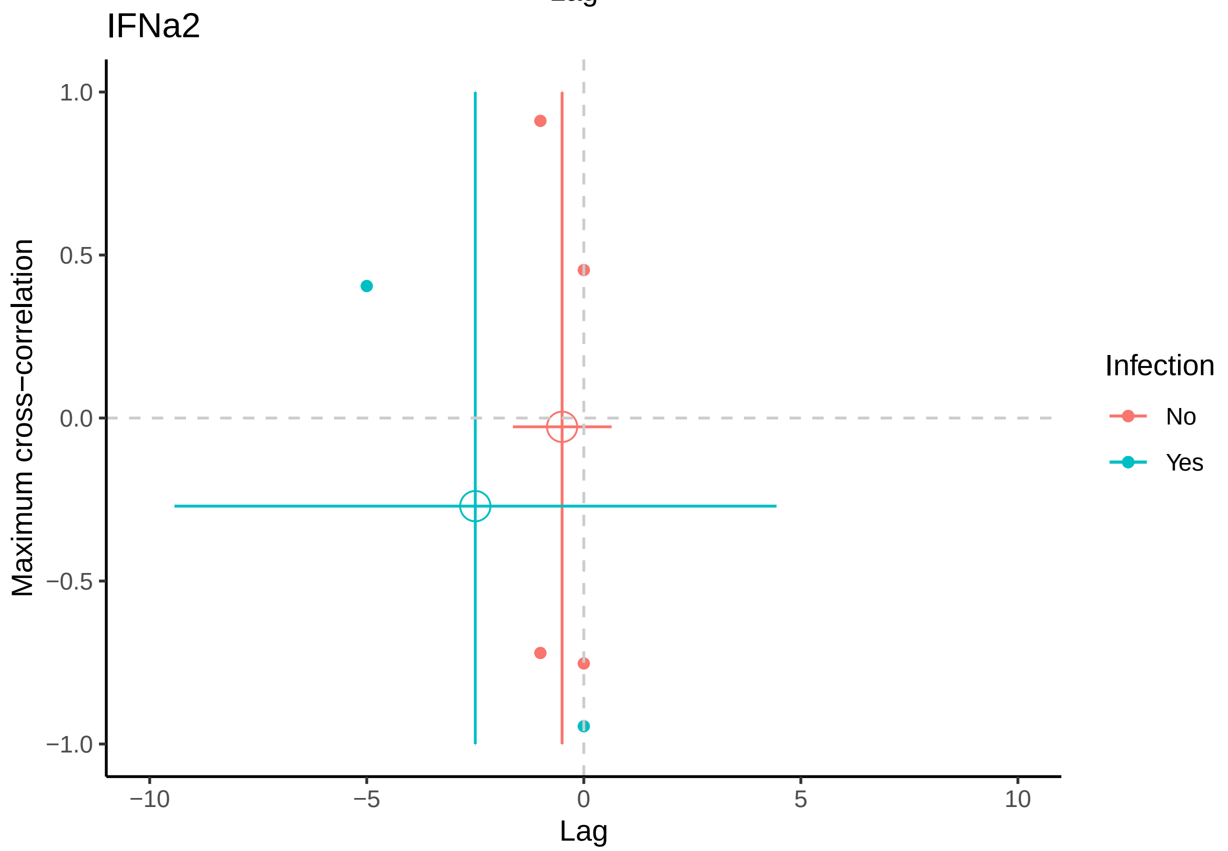
IFNa2
Maximum cross−correlation
1.0
0.5
0.0
−0.5
−1.0
−10
−5
0
5
10
Lag
Infection
No
Yes

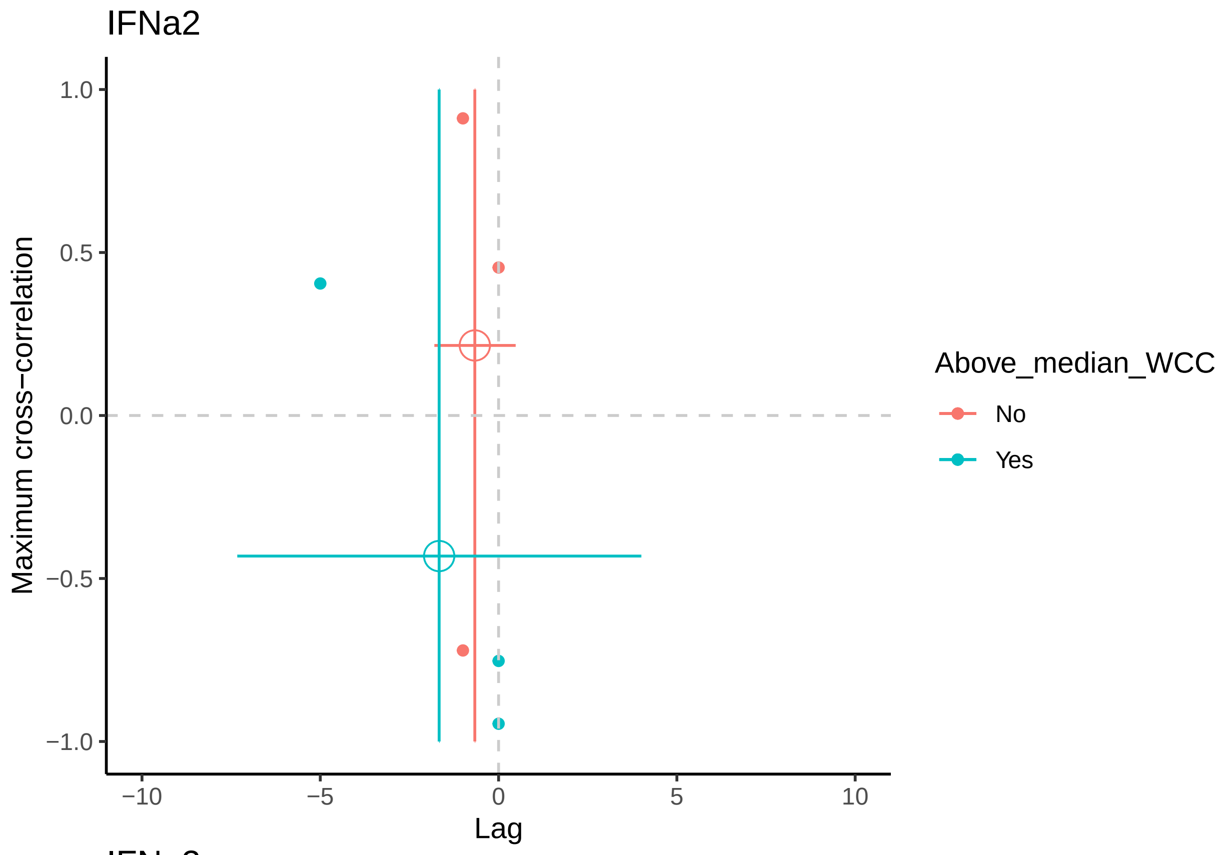
IFNa2
Maximum cross−correlation
1.0
0.5
0.0
−0.5
−1.0
−10
−5
0
5
10
Lag
Above_median_WCC
No
Yes

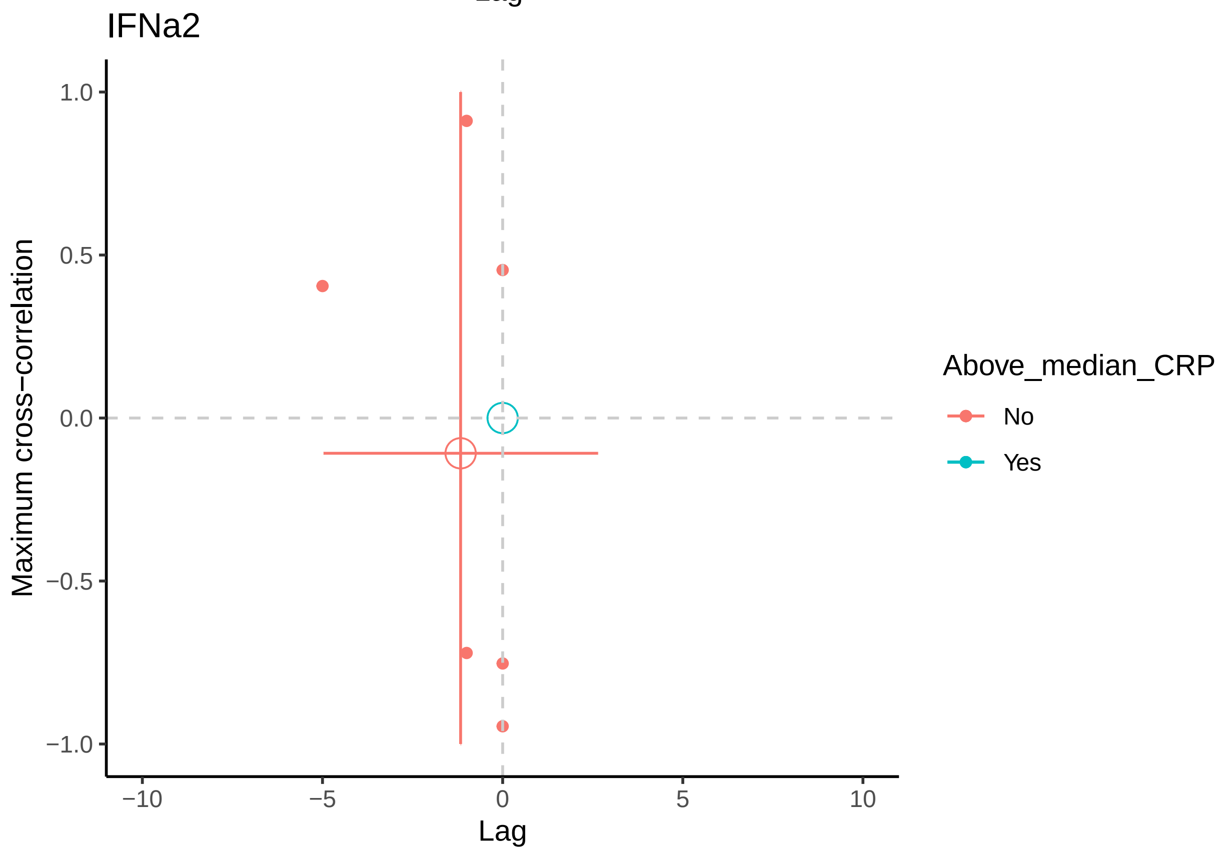
IFNa2
Maximum cross−correlation
1.0
0.5
0.0
−0.5
−1.0
−10
−5
0
5
10
Lag
Above_median_CRP
No
Yes

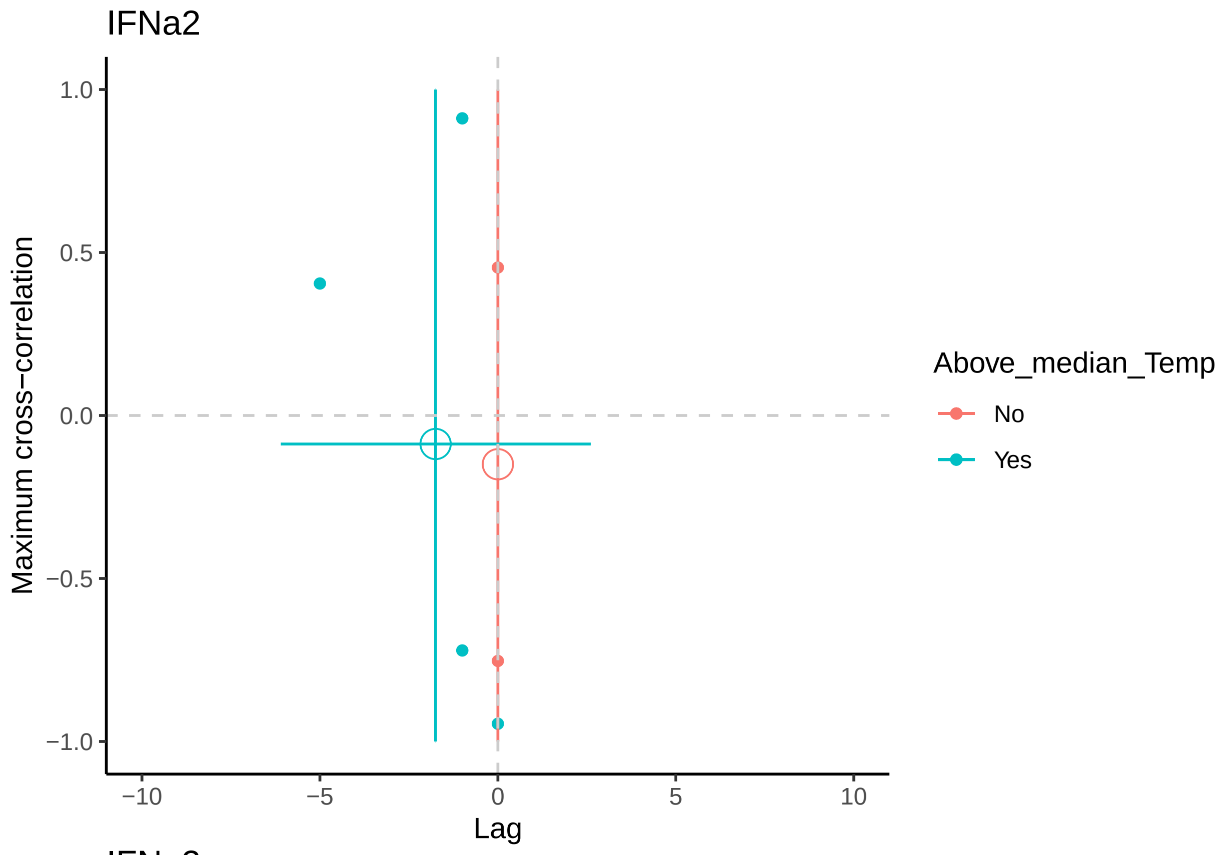

IFNa2
Maximum cross−correlation
1.0
0.5
0.0
−0.5
−1.0
−10
−5
0
5
10
Lag
Above_median_Temp
No
Yes

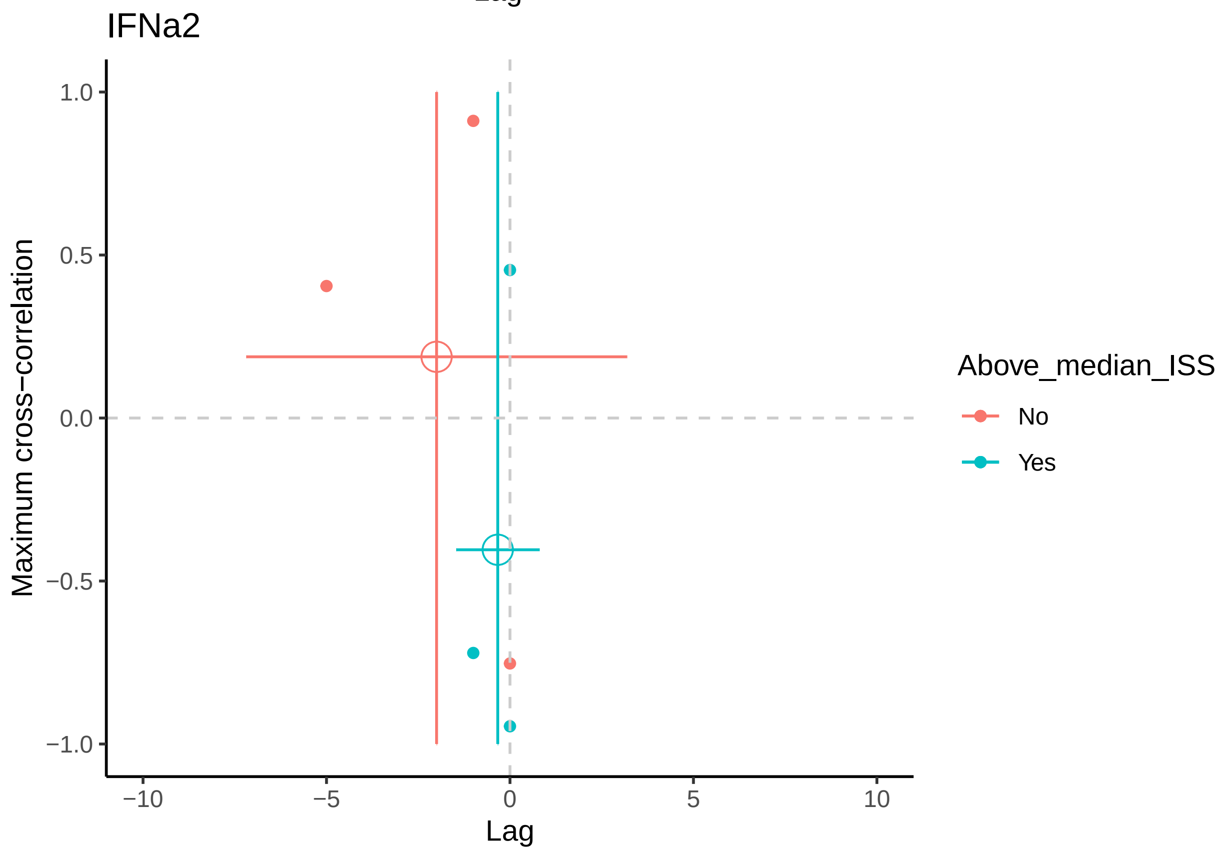

IFNa2
Maximum cross−correlation
1.0
0.5
0.0
−0.5
−1.0
−10
−5
0
5
10
Lag
Above_median_ISS
No
Yes

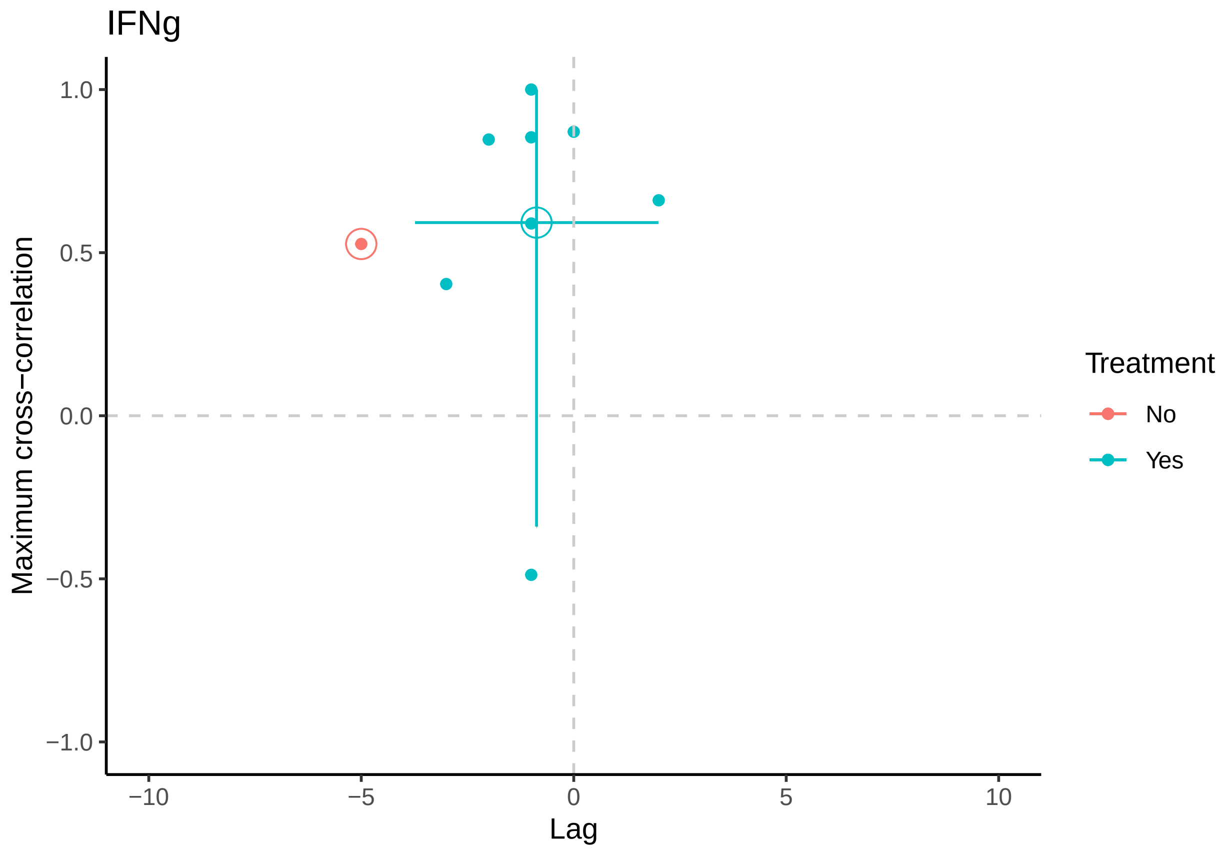
IFNg
Maximum cross−correlation
1.0
0.5
0.0
−0.5
−1.0
−10
−5
0
5
10
Lag
Treatment
No
Yes

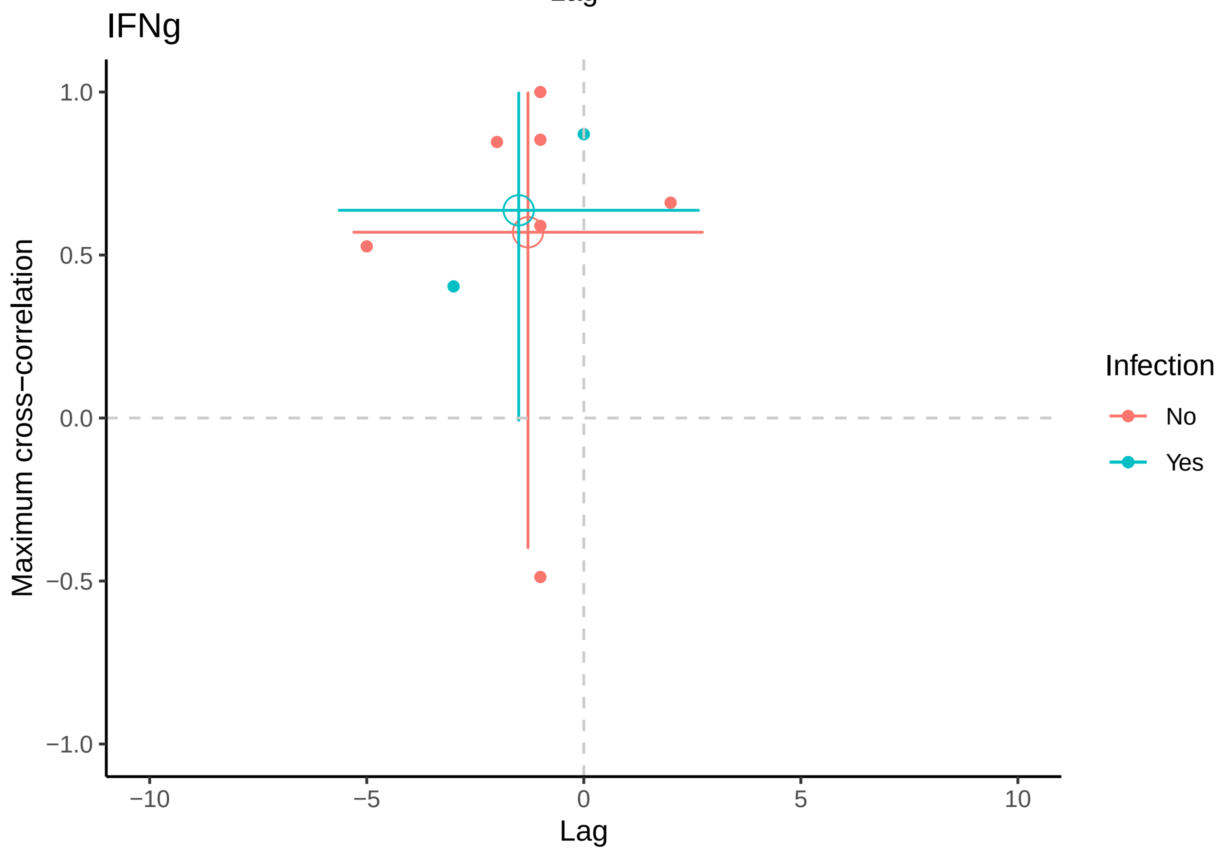
IFNg
Maximum cross−correlation
1.0
0.5
0.0
−0.5
−1.0
−10
−5
0
5
10
Lag
Infection
No
Yes

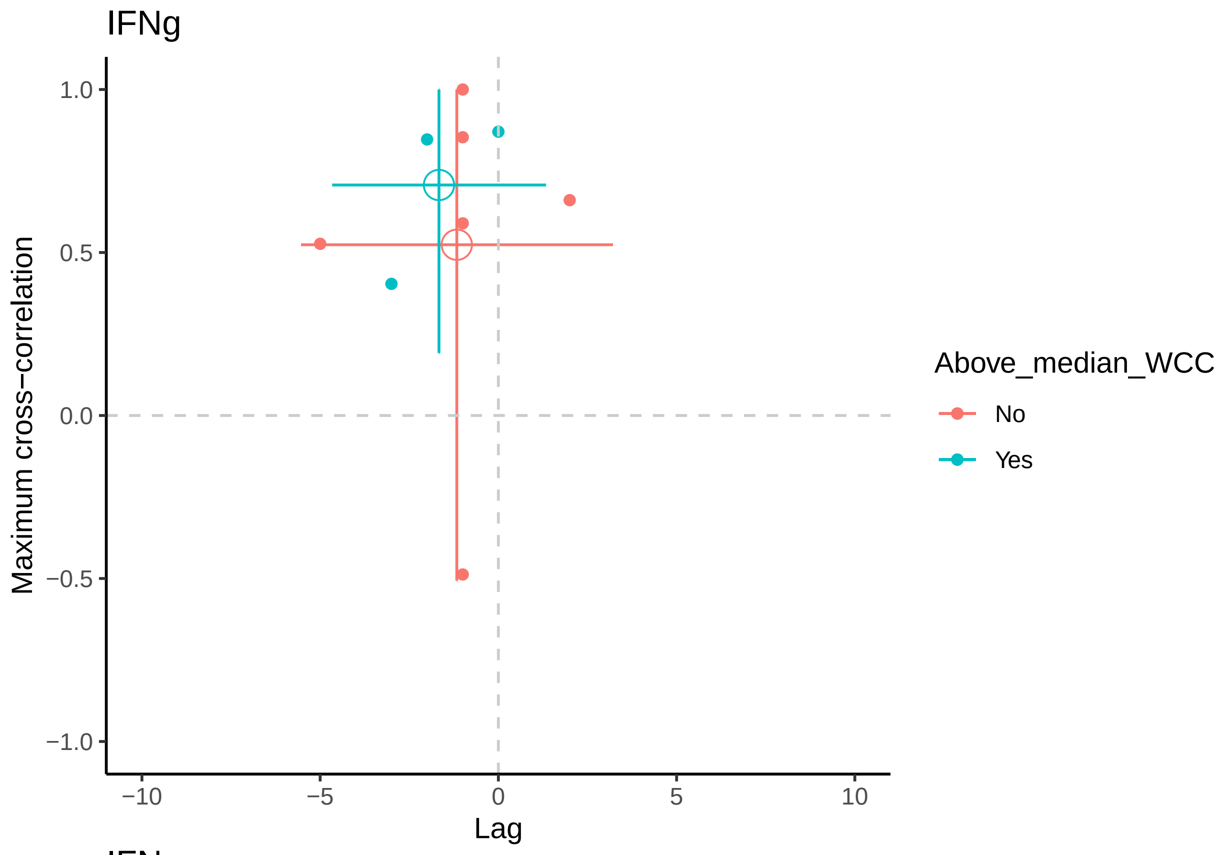
IFNg
Maximum cross−correlation
1.0
0.5
0.0
−0.5
−1.0
−10
−5
0
5
10
Lag
Above_median_WCC
No
Yes

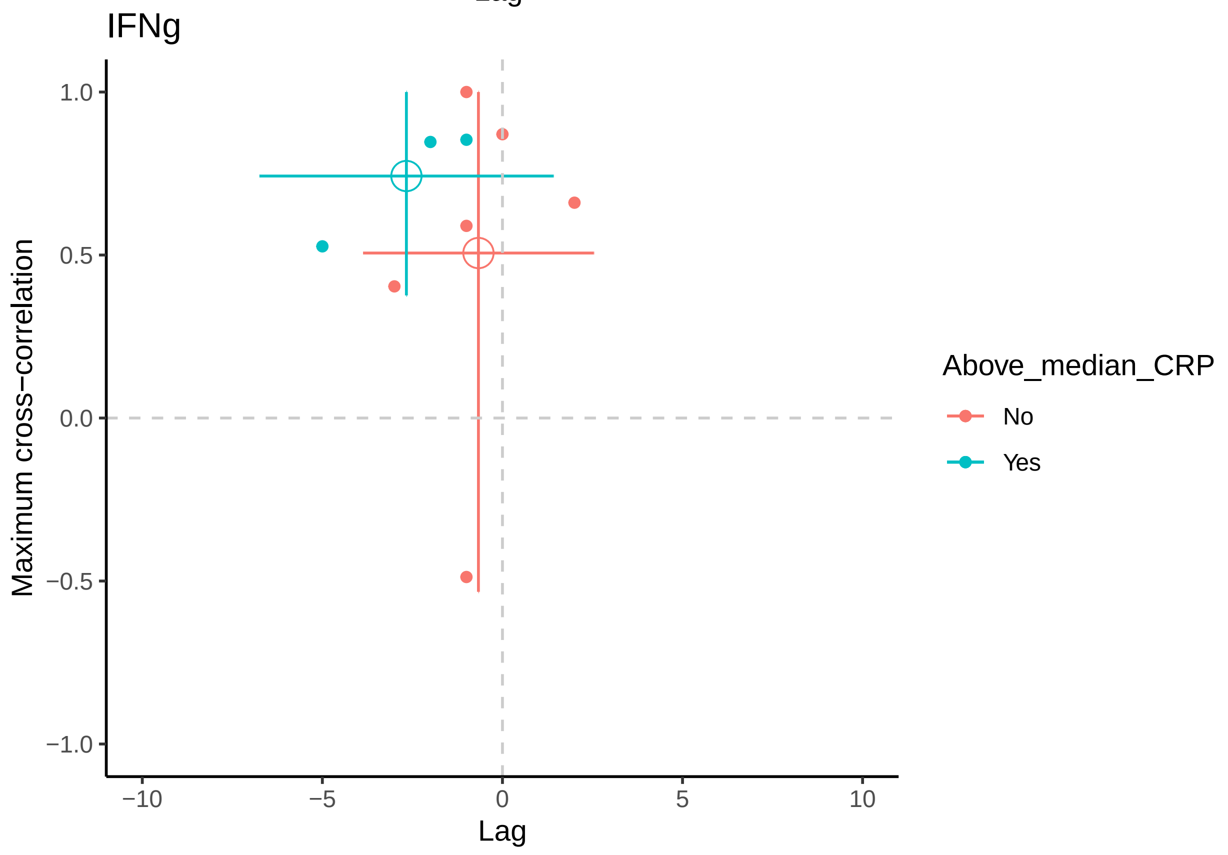
IFNg
Maximum cross−correlation
1.0
0.5
0.0
−0.5
−1.0
−10
−5
0
5
10
Lag
Above_median_CRP
No
Yes

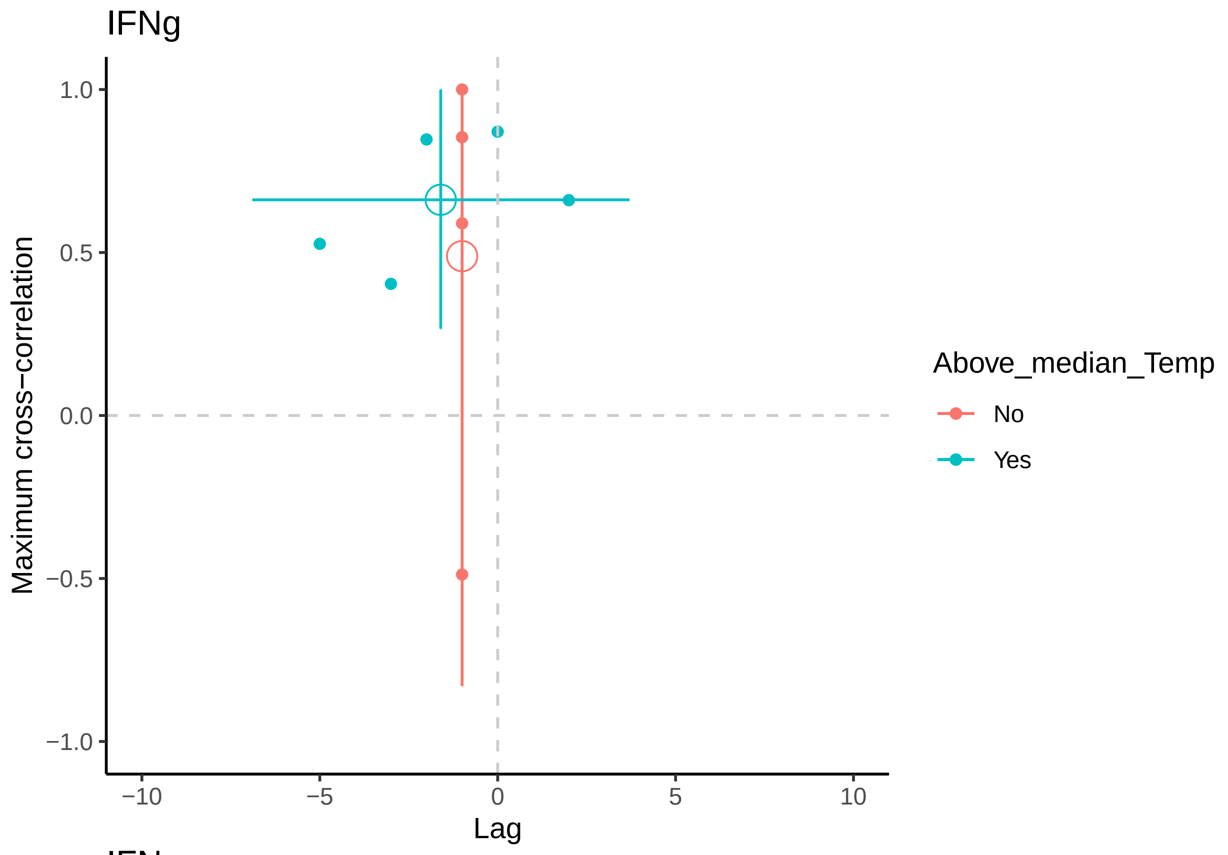
IFNg
Maximum cross−correlation
1.0
0.5
0.0
−0.5
−1.0
−10
−5
0
5
10
Lag
Above_median_Temp
No
Yes

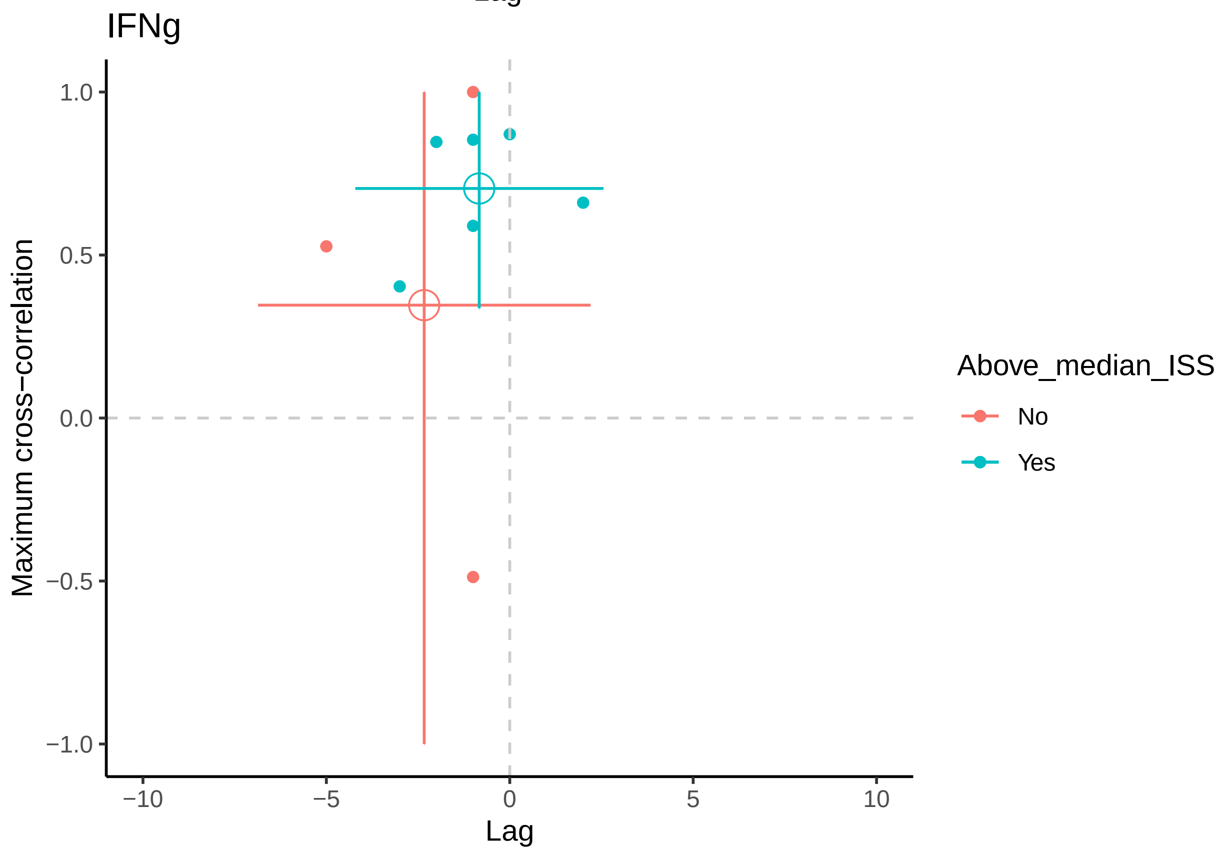
IFNg
Maximum cross−correlation
1.0
0.5
0.0
−0.5
−1.0
−10
−5
0
5
10
Lag
Above_median_ISS
No
Yes

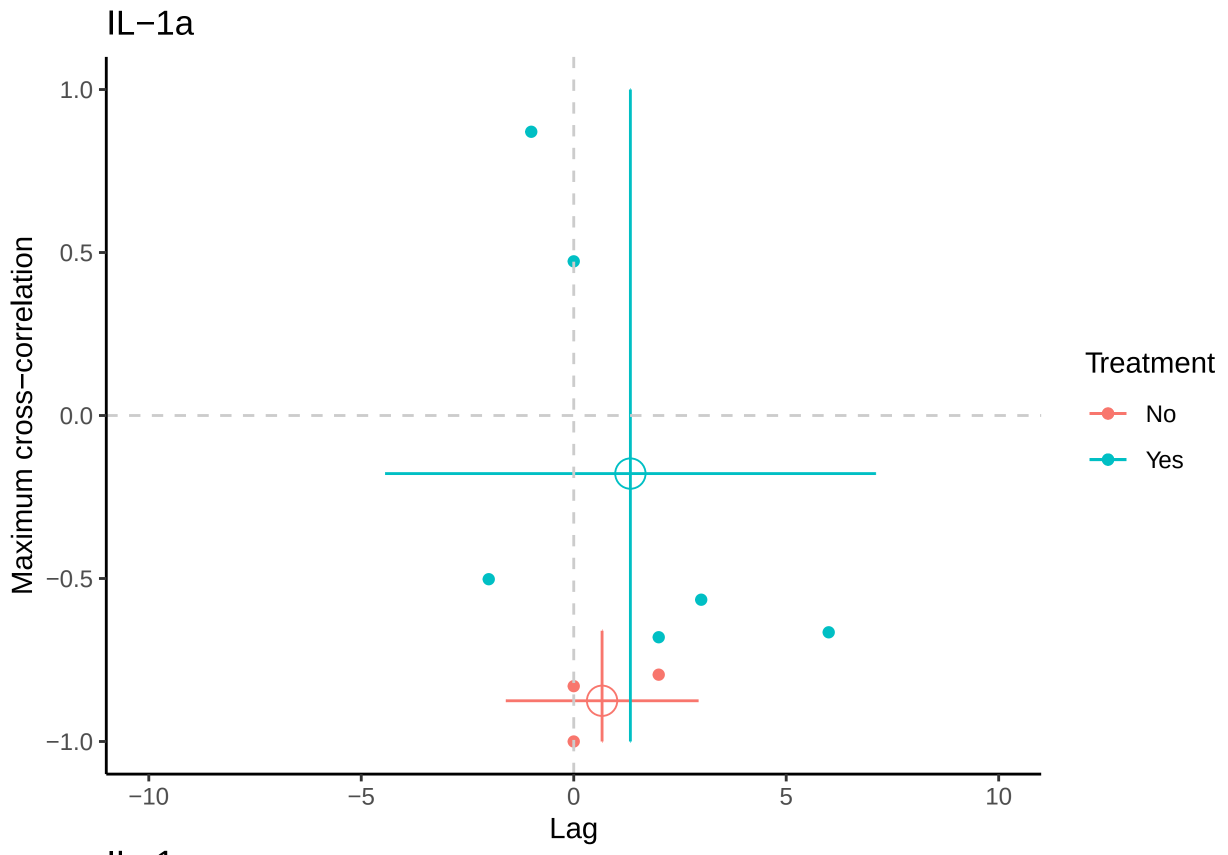
IL−1a
Maximum cross−correlation
1.0
0.5
0.0
−0.5
−1.0
−10
−5
0
5
10
Lag
Treatment
No
Yes

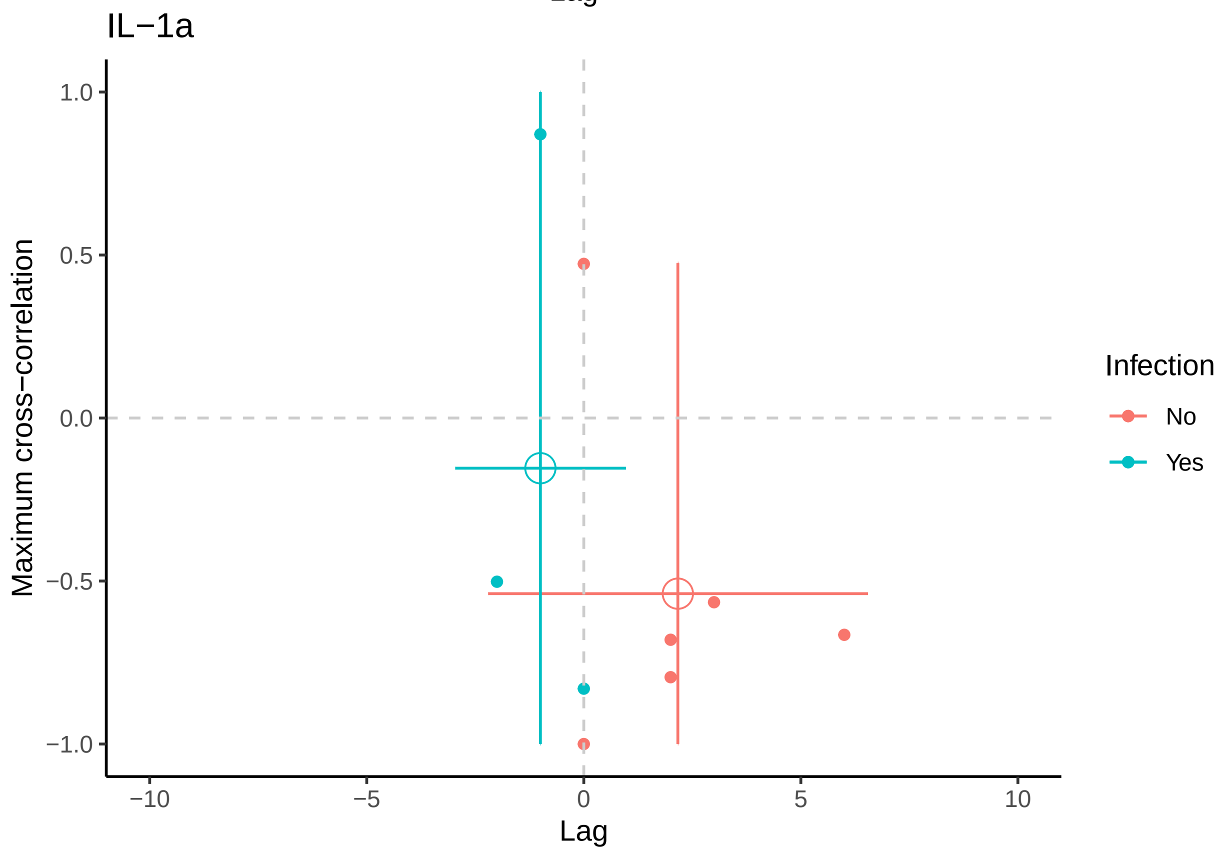
IL−1a
Maximum cross−correlation
1.0
0.5
0.0
−0.5
−1.0
−10
−5
0
5
10
Lag
Infection
No
Yes

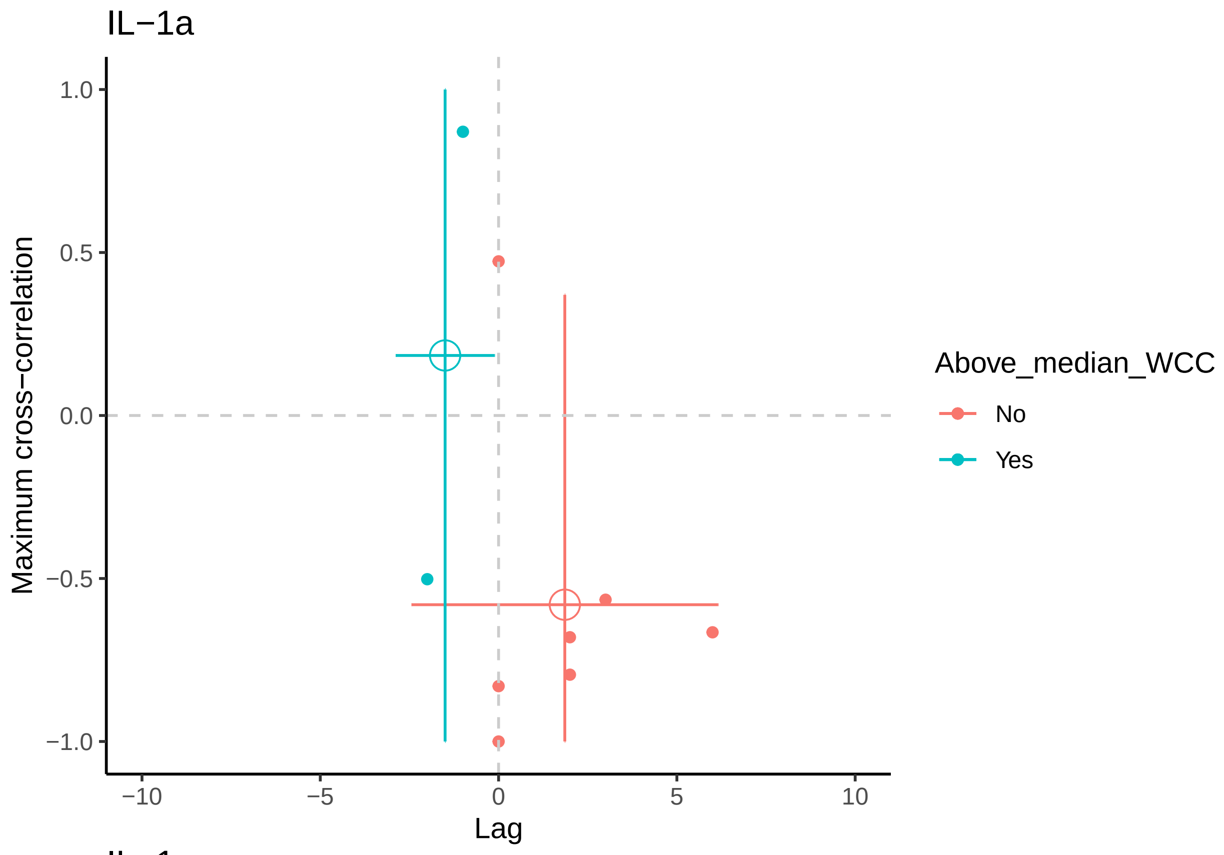
IL−1a
Maximum cross−correlation
1.0
0.5
0.0
−0.5
−1.0
−10
−5
0
5
10
Lag
Above_median_WCC
No
Yes

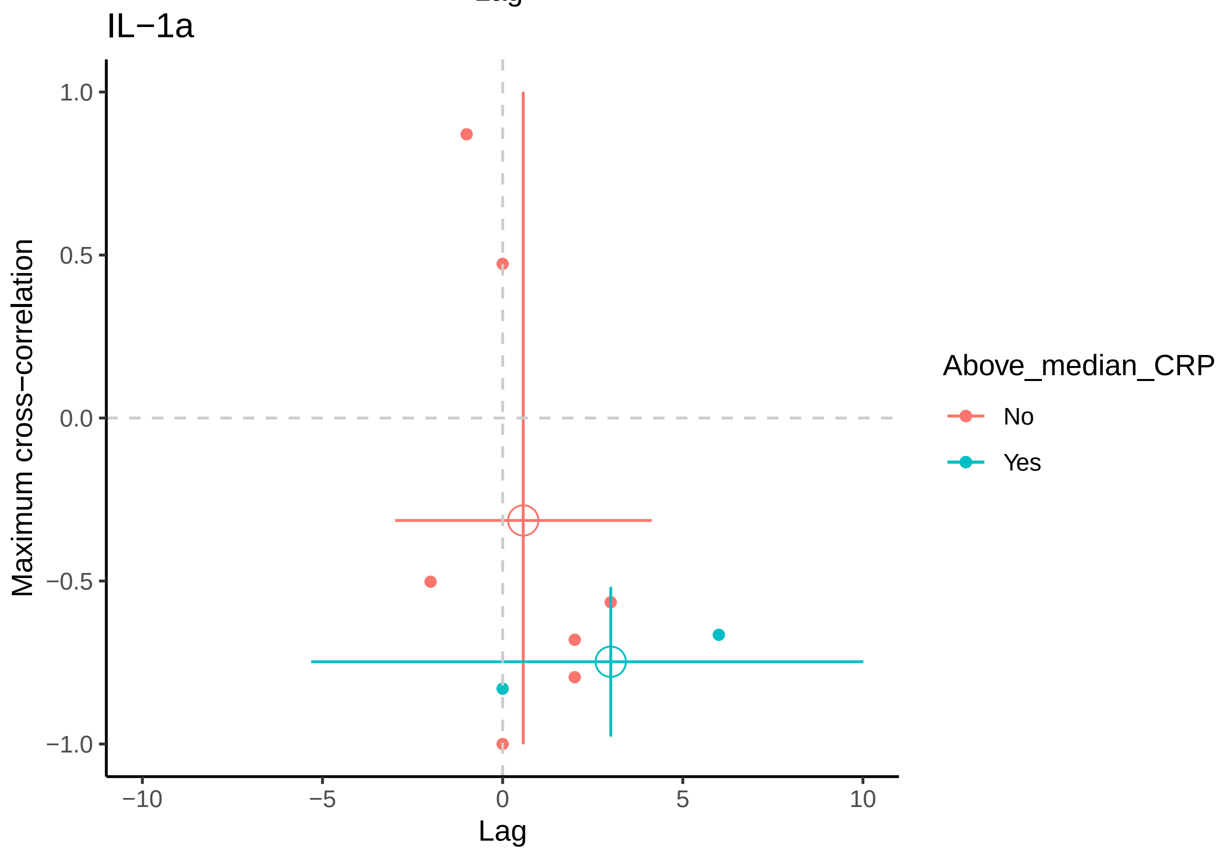
IL−1a
Maximum cross−correlation
1.0
0.5
0.0
−0.5
−1.0
−10
−5
0
5
10
Lag
Above_median_CRP
No
Yes

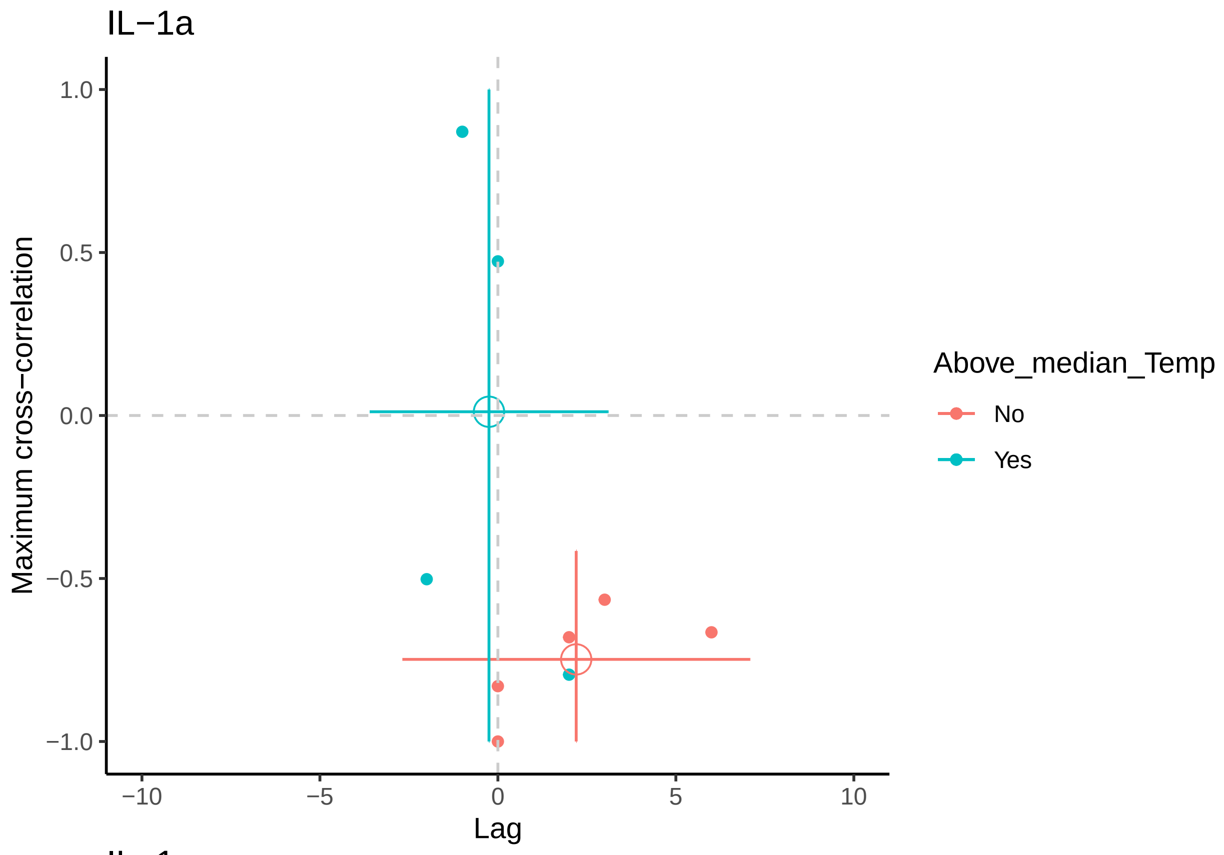
IL−1a
Maximum cross−correlation
1.0
0.5
0.0
−0.5
−1.0
−10
−5
0
5
10
Lag
Above_median_Temp
No
Yes

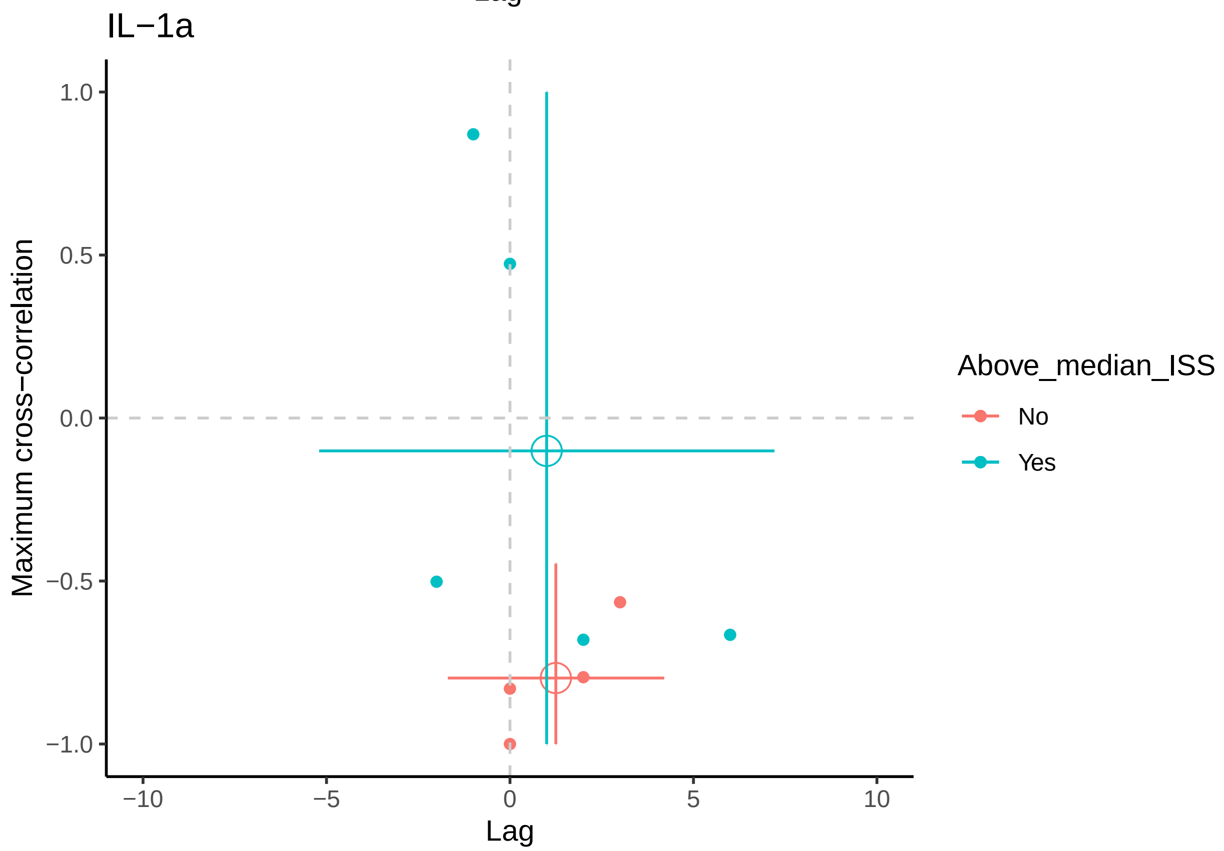
IL−1a
Maximum cross−correlation
1.0
0.5
0.0
−0.5
−1.0
−10
−5
0
5
10
Lag
Above_median_ISS
No
Yes

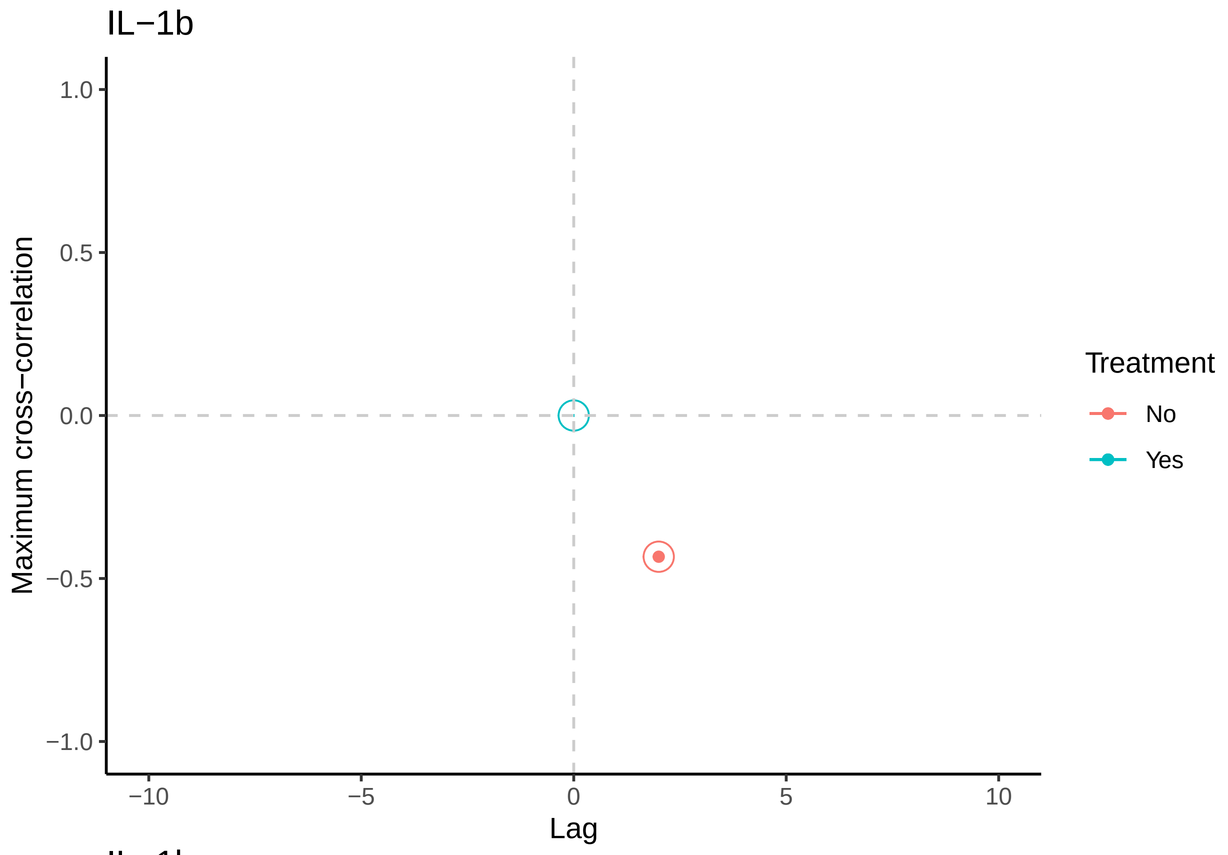
IL−1b
Maximum cross−correlation
1.0
0.5
0.0
−0.5
−1.0
−10
−5
0
5
10
Lag
Treatment
No
Yes

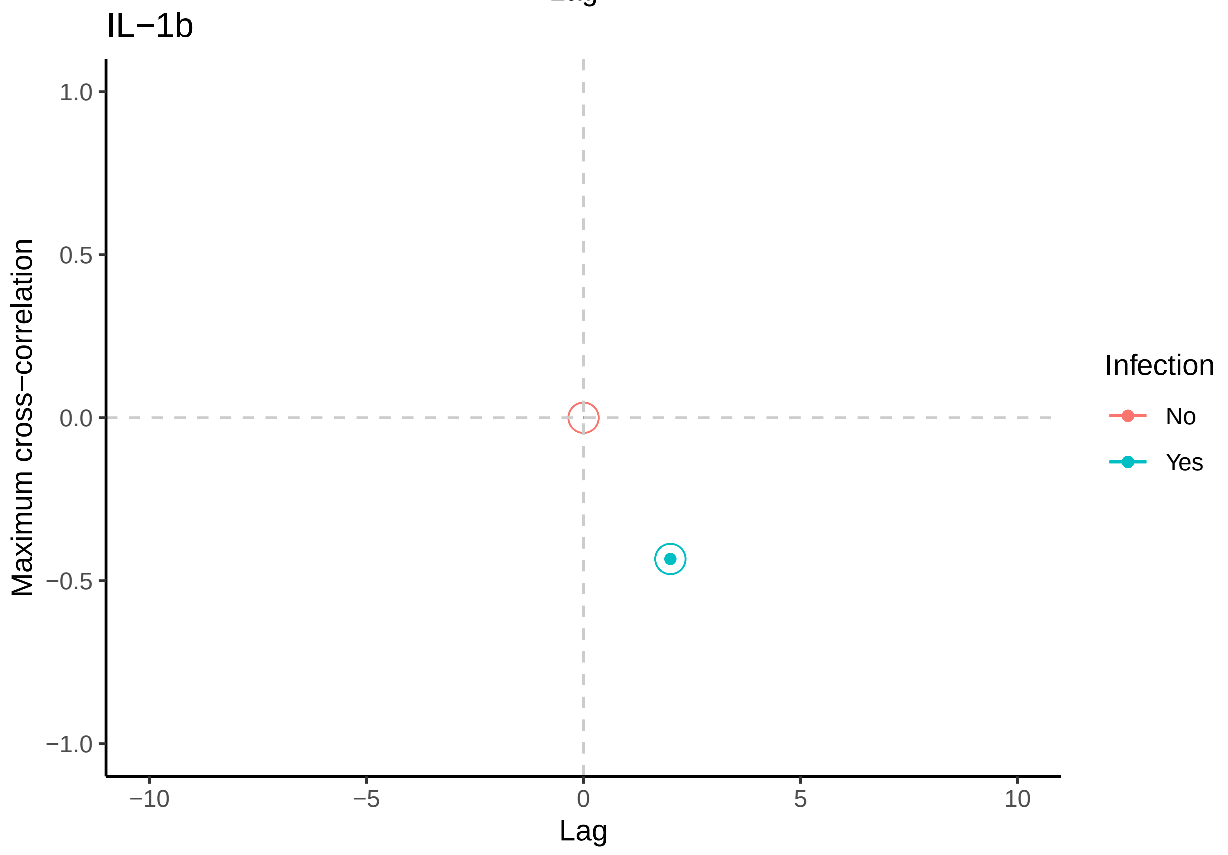
IL−1b
Maximum cross−correlation
1.0
0.5
0.0
−0.5
−1.0
−10
−5
0
5
10
Lag
Infection
No
Yes

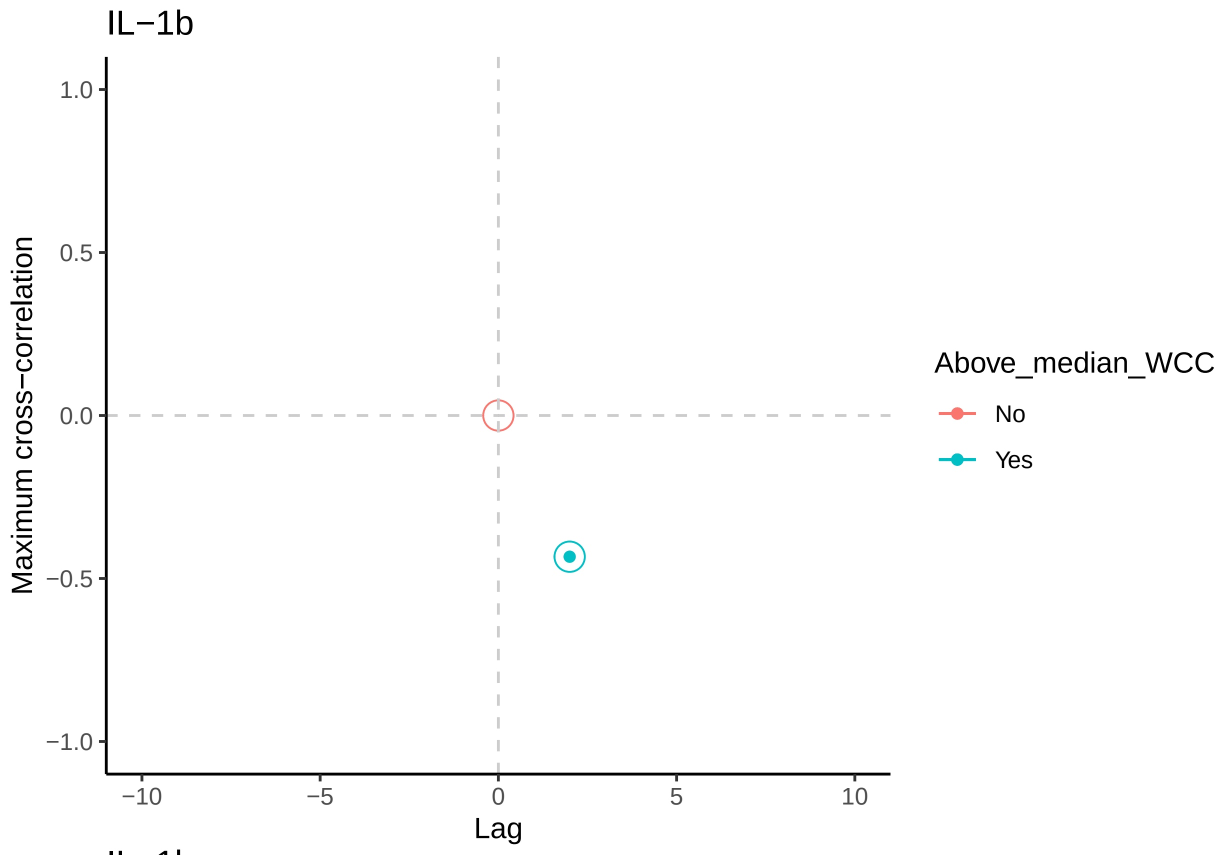
IL−1b
Maximum cross−correlation
1.0
0.5
0.0
−0.5
−1.0
−10
−5
0
5
10
Lag
Above_median_WCC
No
Yes

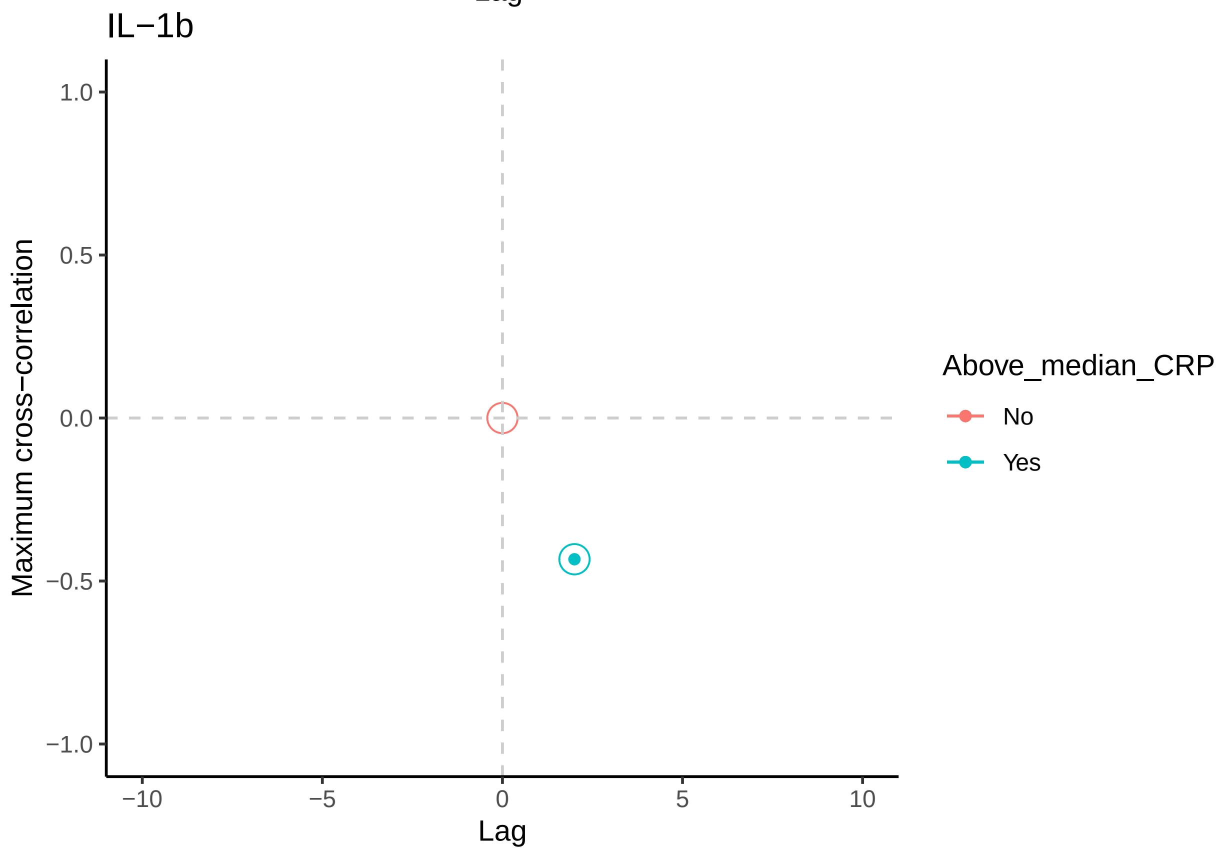
IL−1b
Maximum cross−correlation
1.0
0.5
0.0
−0.5
−1.0
−10
−5
0
5
10
Lag
Above_median_CRP
No
Yes

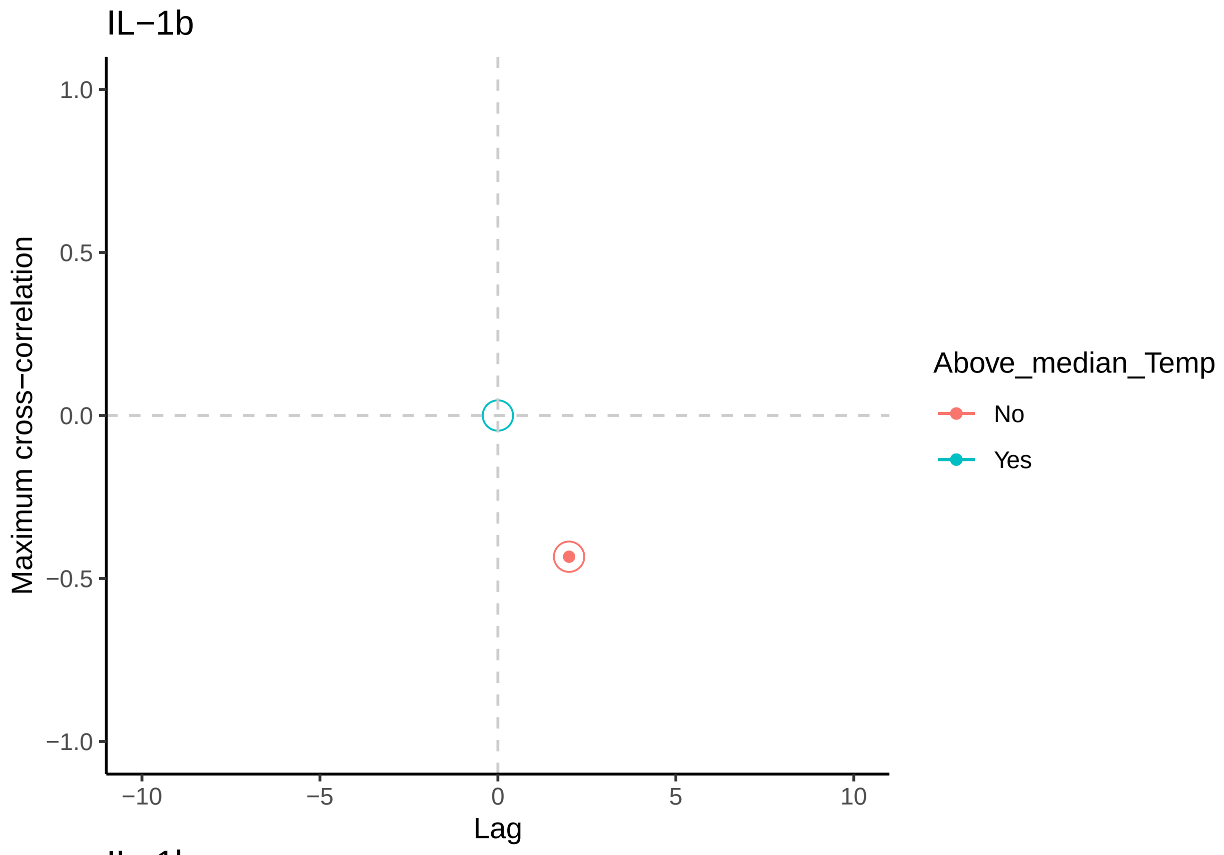
IL−1b
Maximum cross−correlation
1.0
0.5
0.0
−0.5
−1.0
−10
−5
0
5
10
Lag
Above_median_Temp
No
Yes

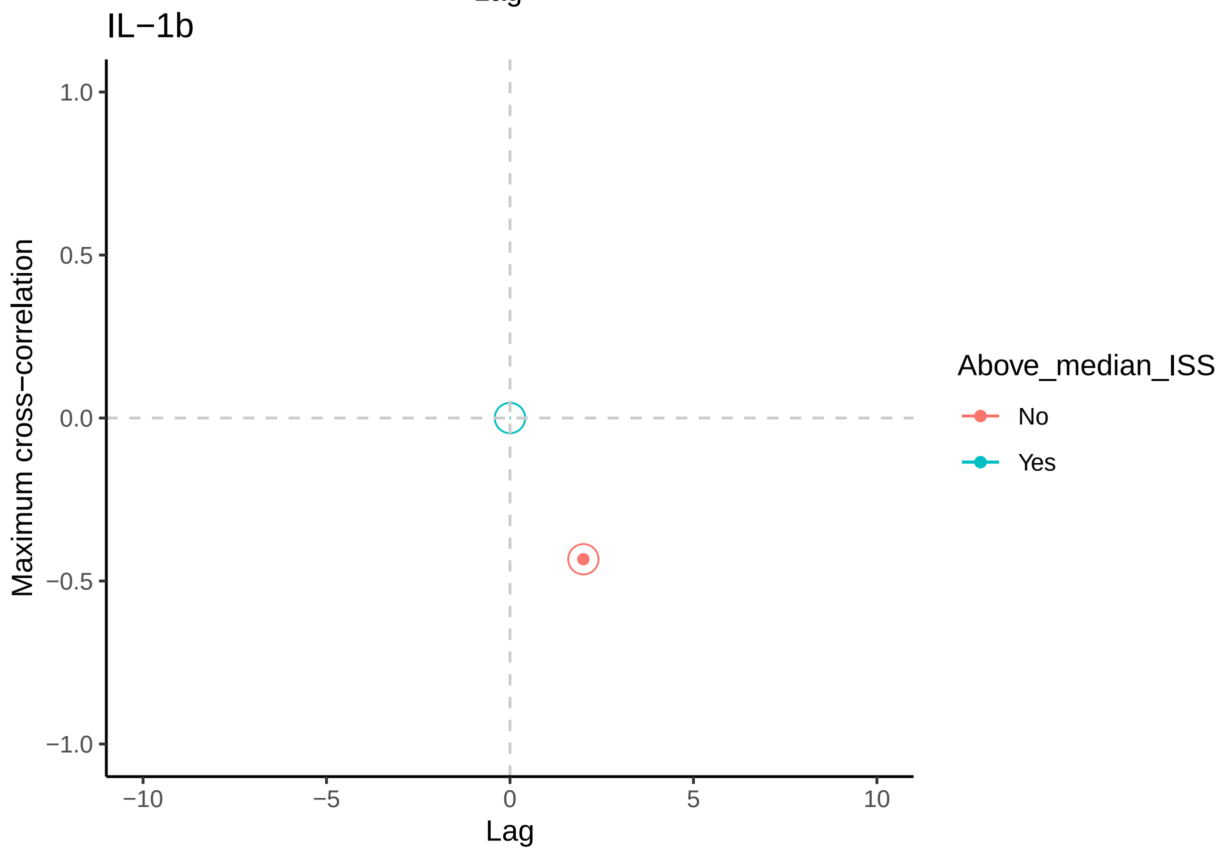
IL−1b
Maximum cross−correlation
1.0
0.5
0.0
−0.5
−1.0
−10
−5
0
5
10
Lag
Above_median_ISS
No
Yes

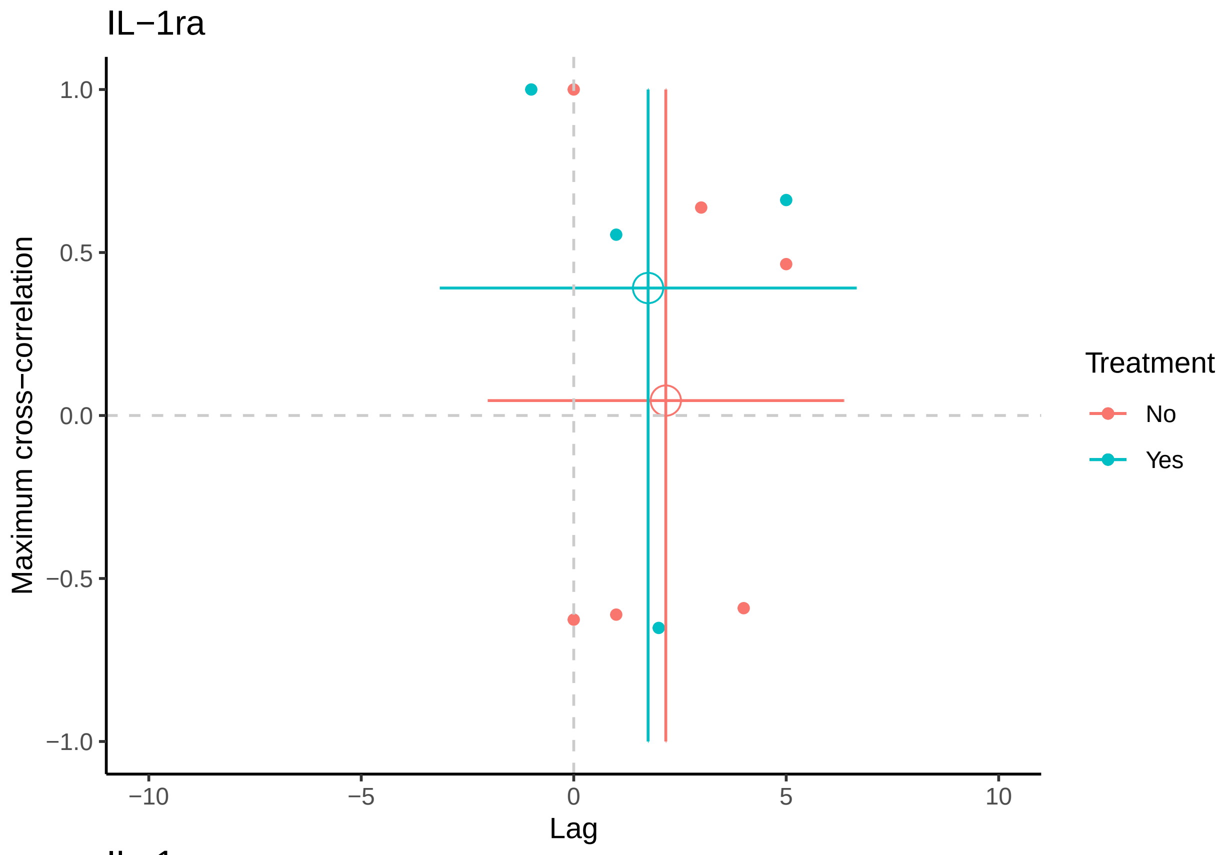
IL−1ra
Maximum cross−correlation
1.0
0.5
0.0
−0.5
−1.0
−10
−5
0
5
10
Lag
Treatment
No
Yes

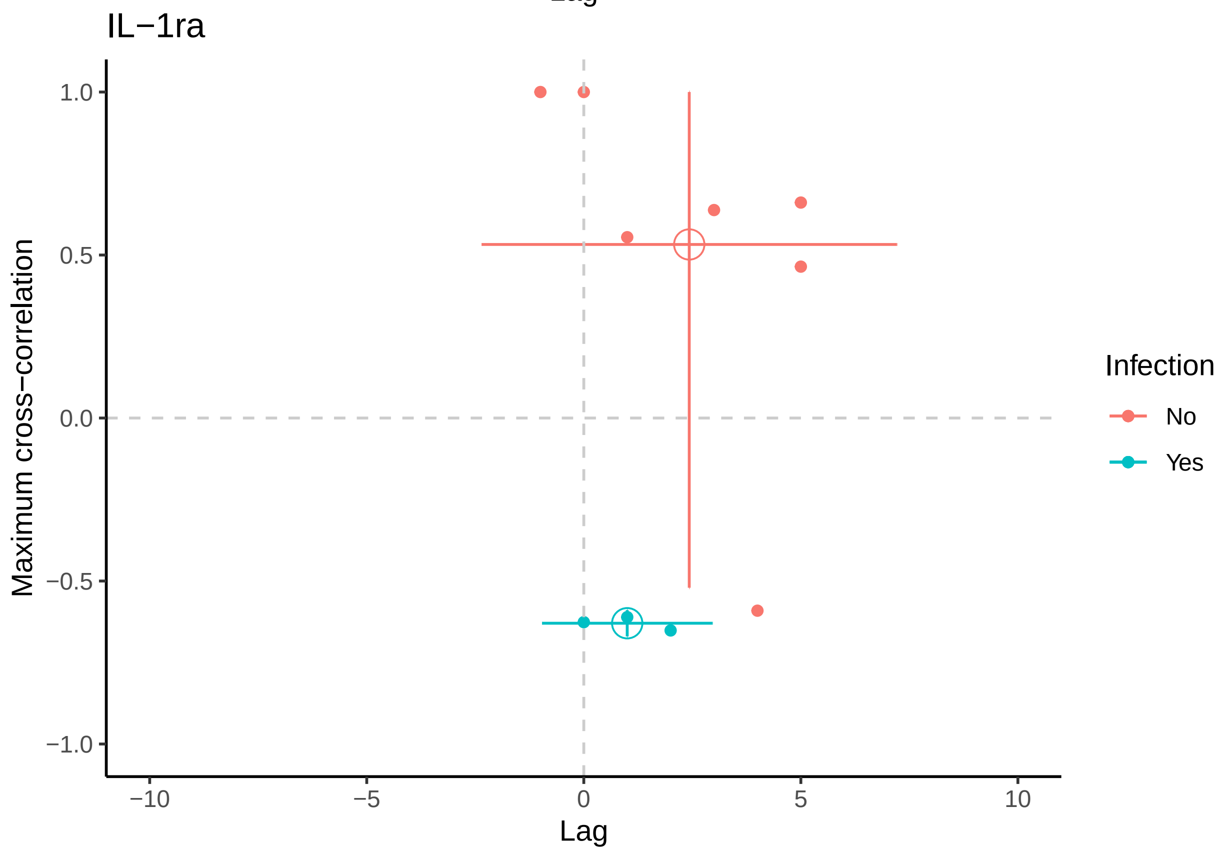
IL−1ra
Maximum cross−correlation
1.0
0.5
0.0
−0.5
−1.0
−10
−5
0
5
10
Lag
Infection
No
Yes

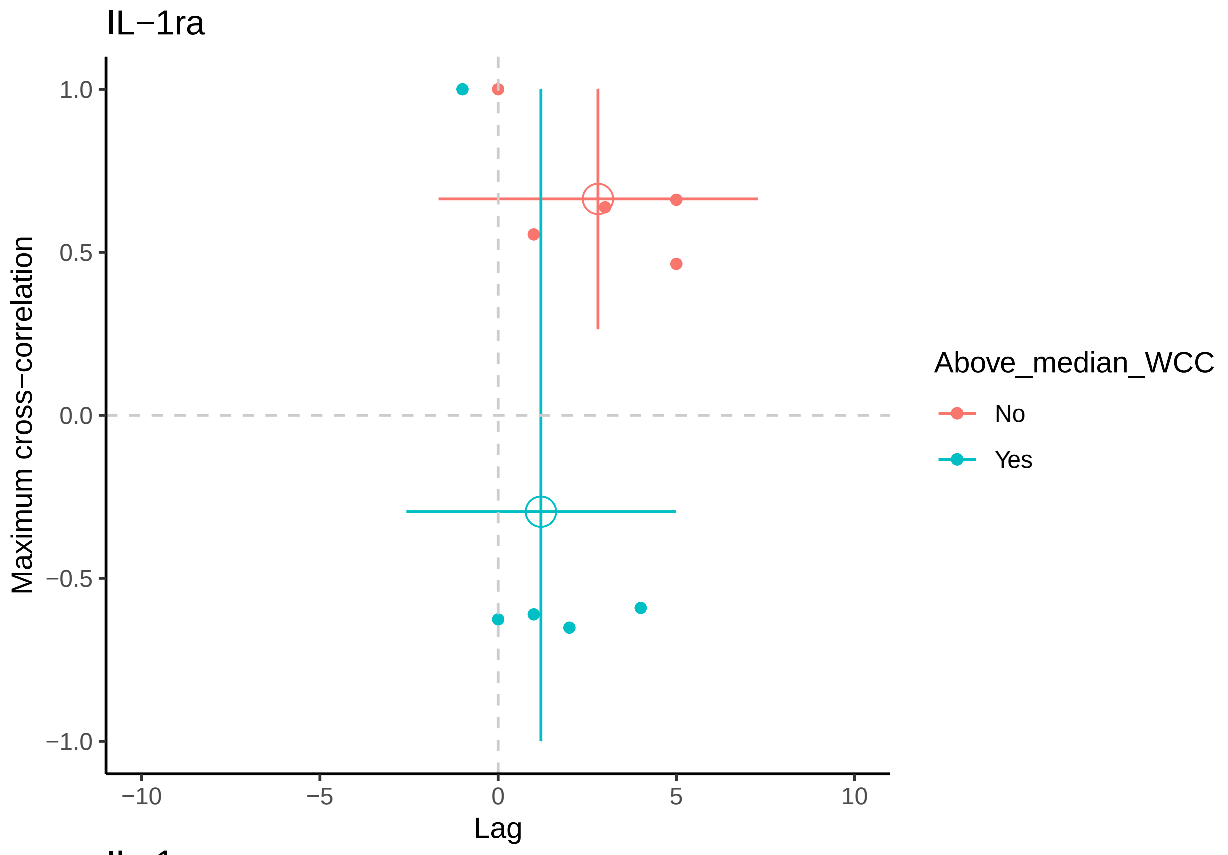
IL−1ra
Maximum cross−correlation
Lag
Above_median_WCC
No
Yes

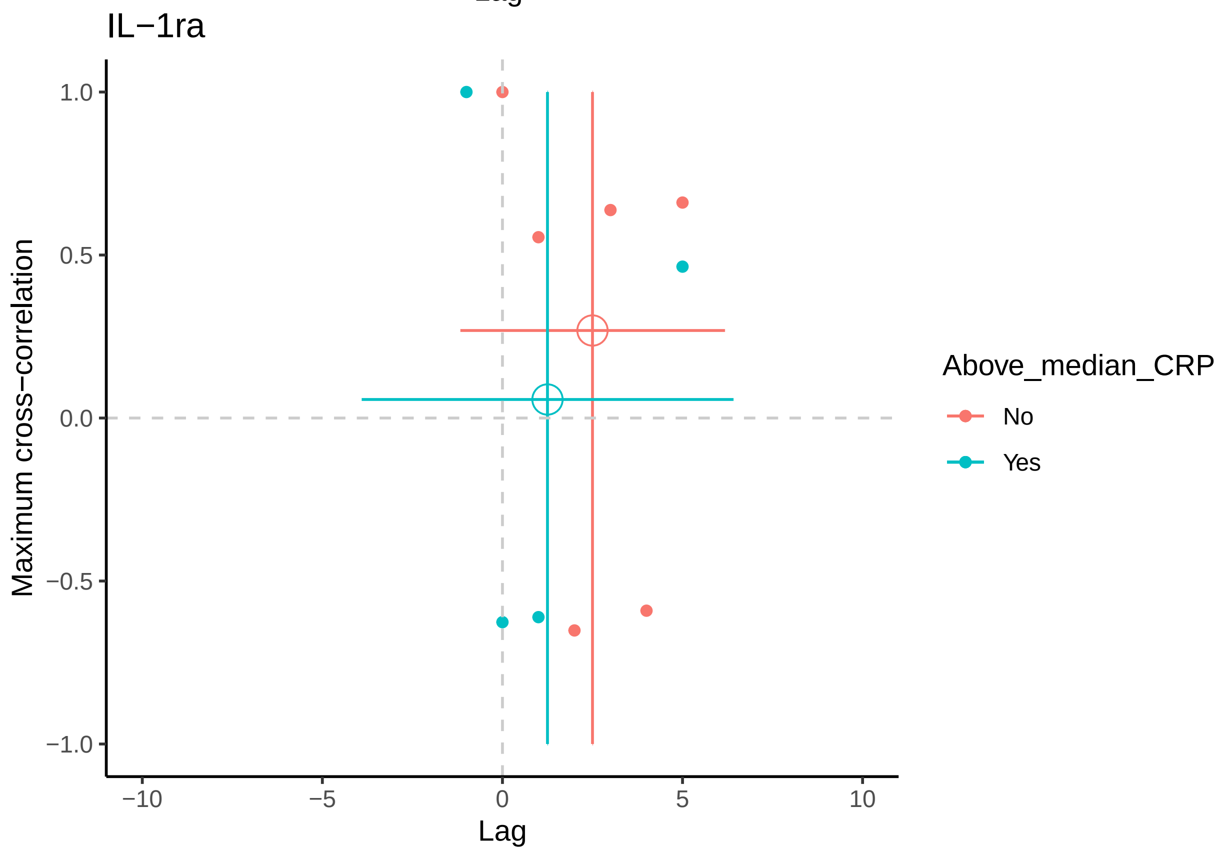
IL−1ra
Maximum cross−correlation
Lag
Above_median_CRP
No
Yes

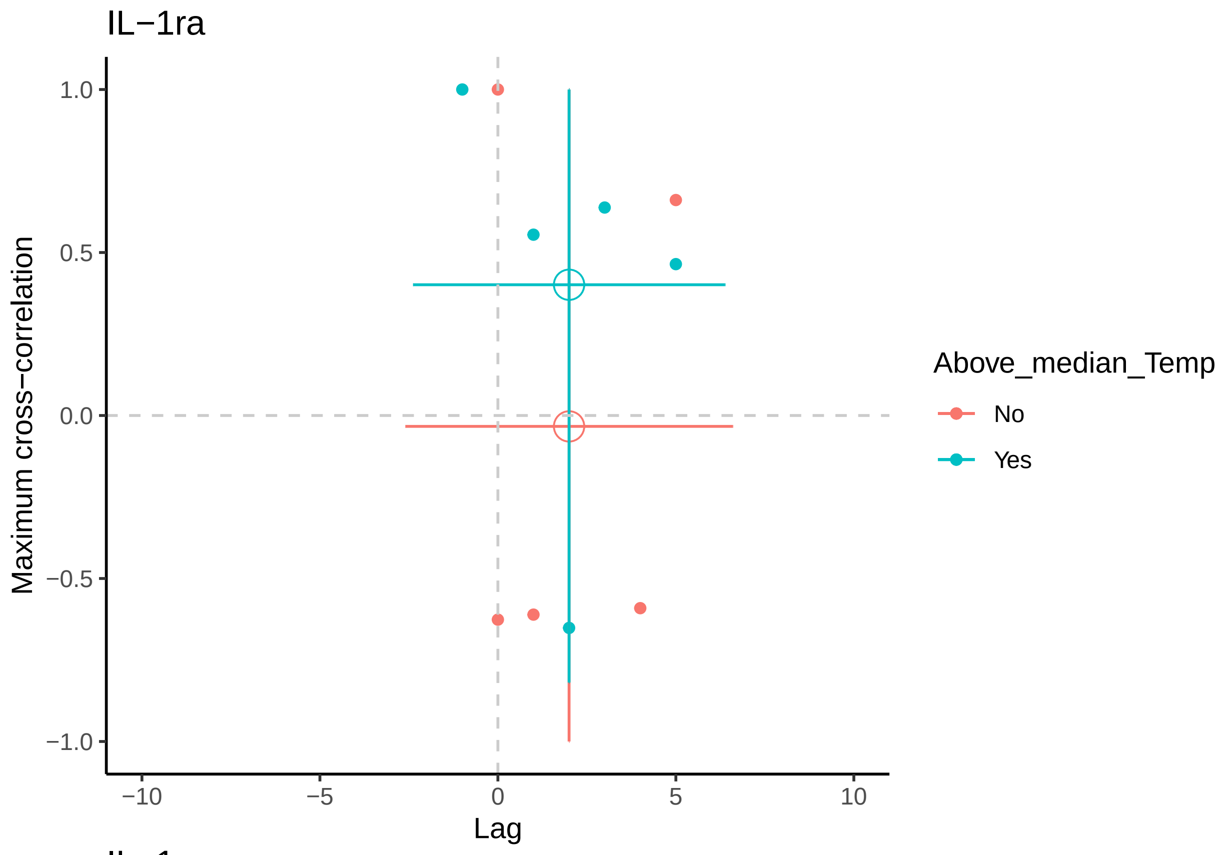
IL−1ra
Maximum cross−correlation
1.0
0.5
0.0
−0.5
−1.0
−10
−5
0
5
10
Lag
Above_median_Temp
No
Yes

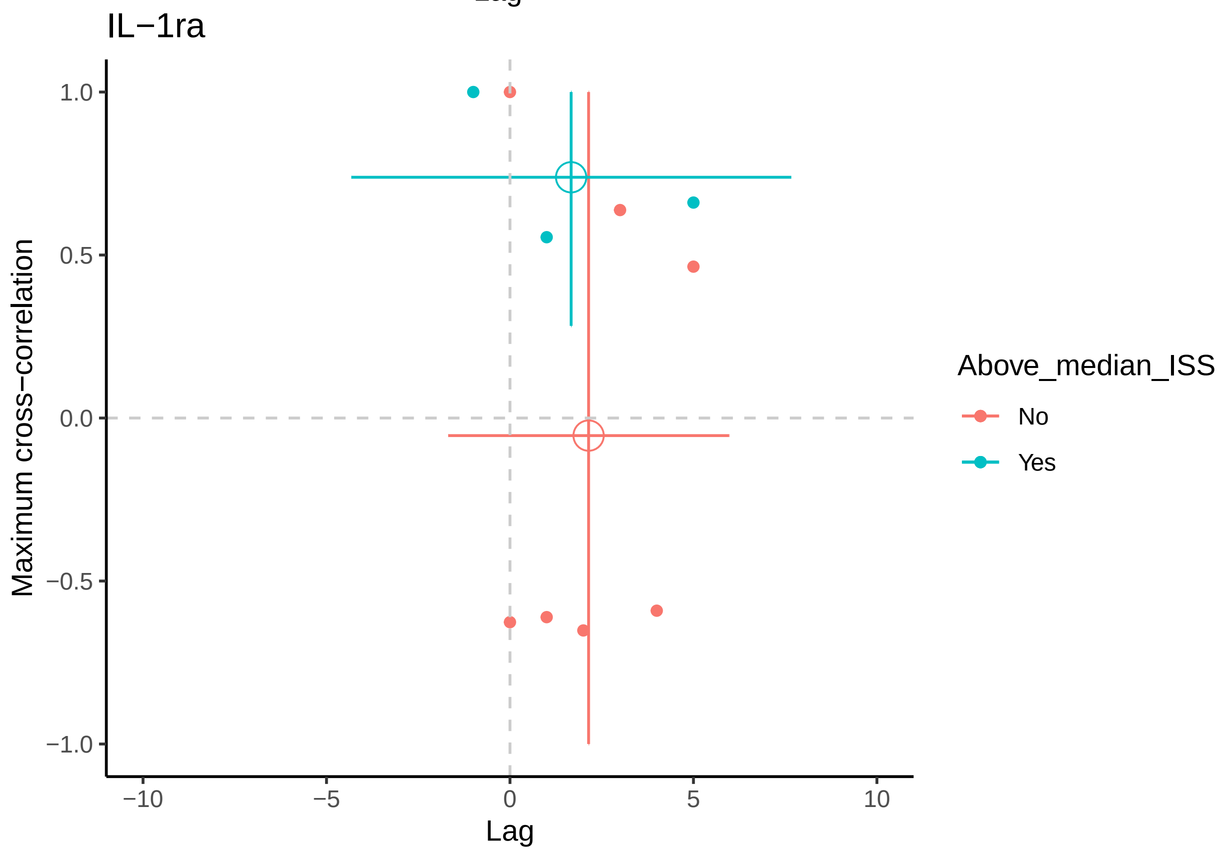
IL−1ra
Maximum cross−correlation
1.0
0.5
0.0
−0.5
−1.0
−10
−5
0
5
10
Lag
Above_median_ISS
No
Yes

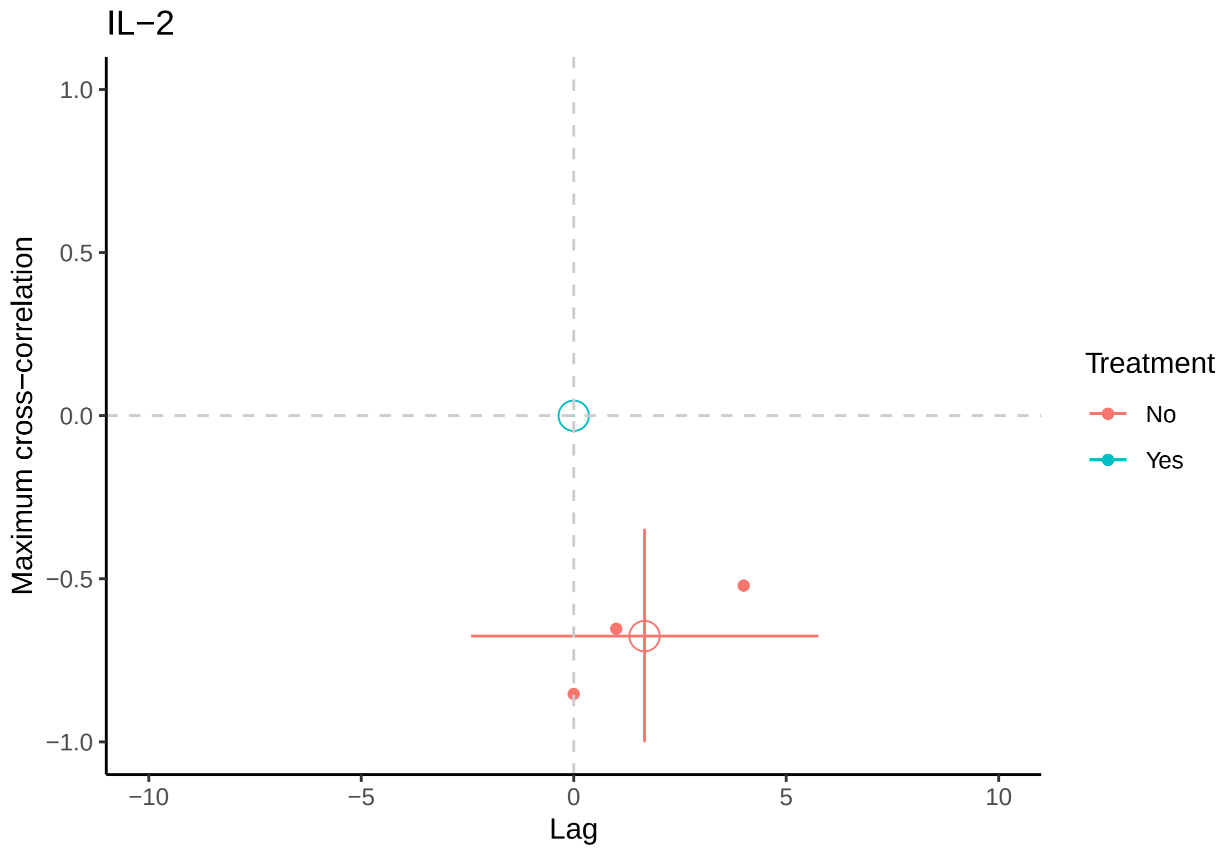
IL−2
Maximum cross−correlation
1.0
0.5
0.0
−0.5
−1.0
−10
−5
0
5
10
Lag
Treatment
No
Yes

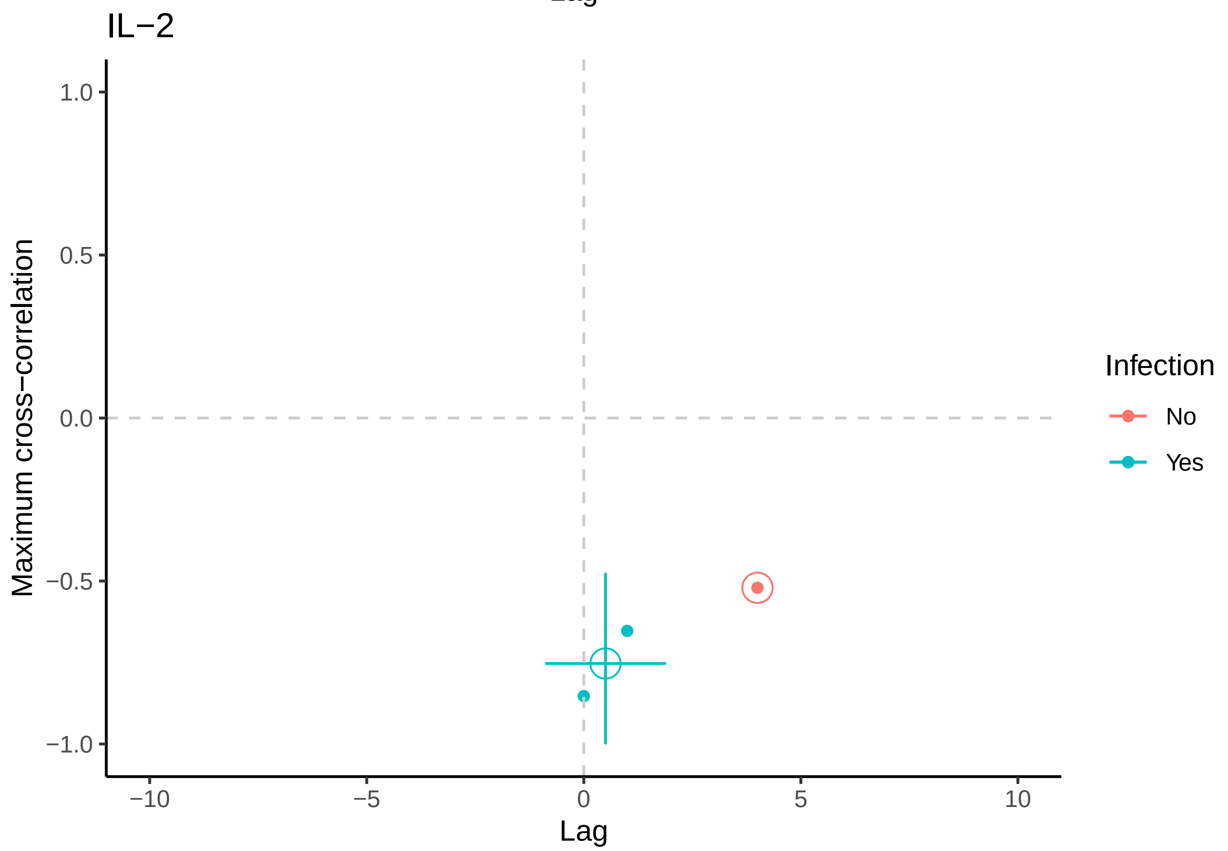
IL−2
Maximum cross−correlation
1.0
0.5
0.0
−0.5
−1.0
−10
−5
0
5
10
Lag
Infection
No
Yes

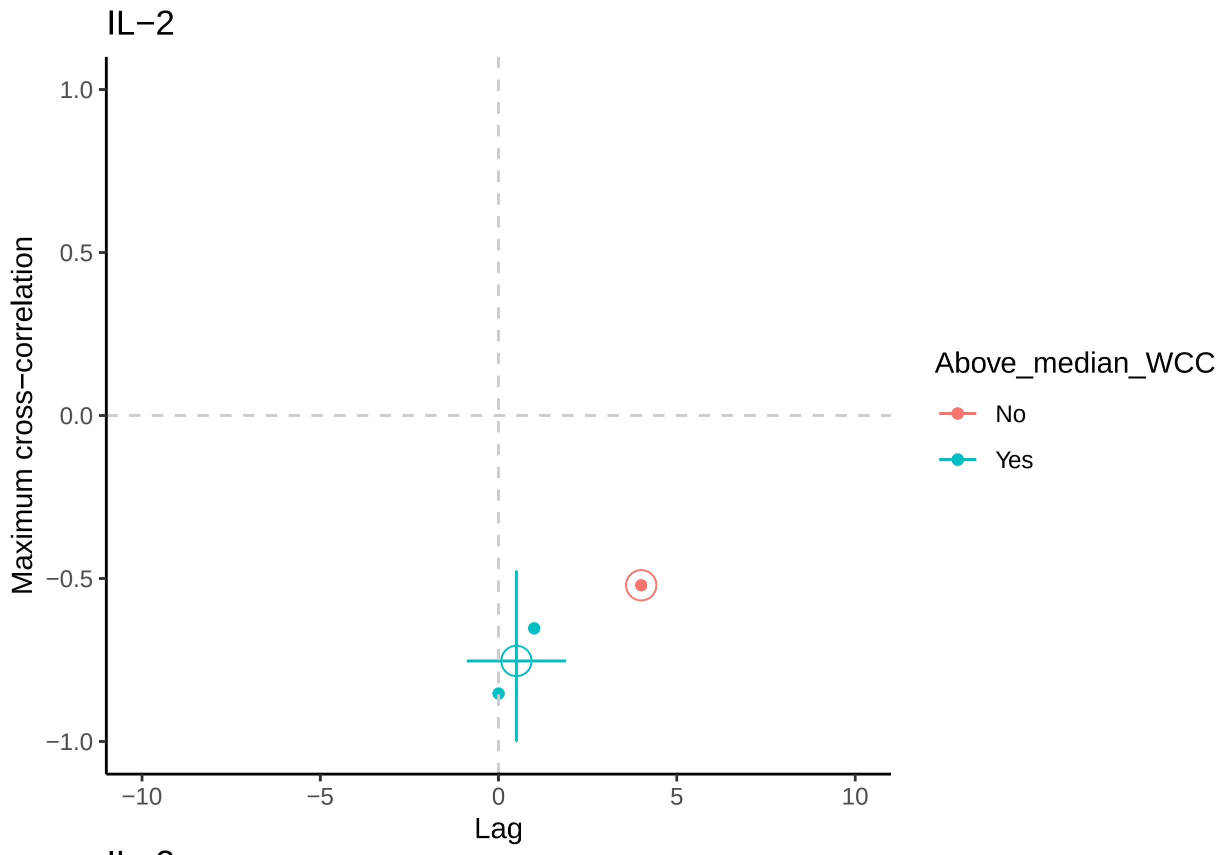
IL−2
Maximum cross−correlation
1.0
0.5
0.0
−0.5
−1.0
−10
−5
0
5
10
Lag
Above_median_WCC
No
Yes

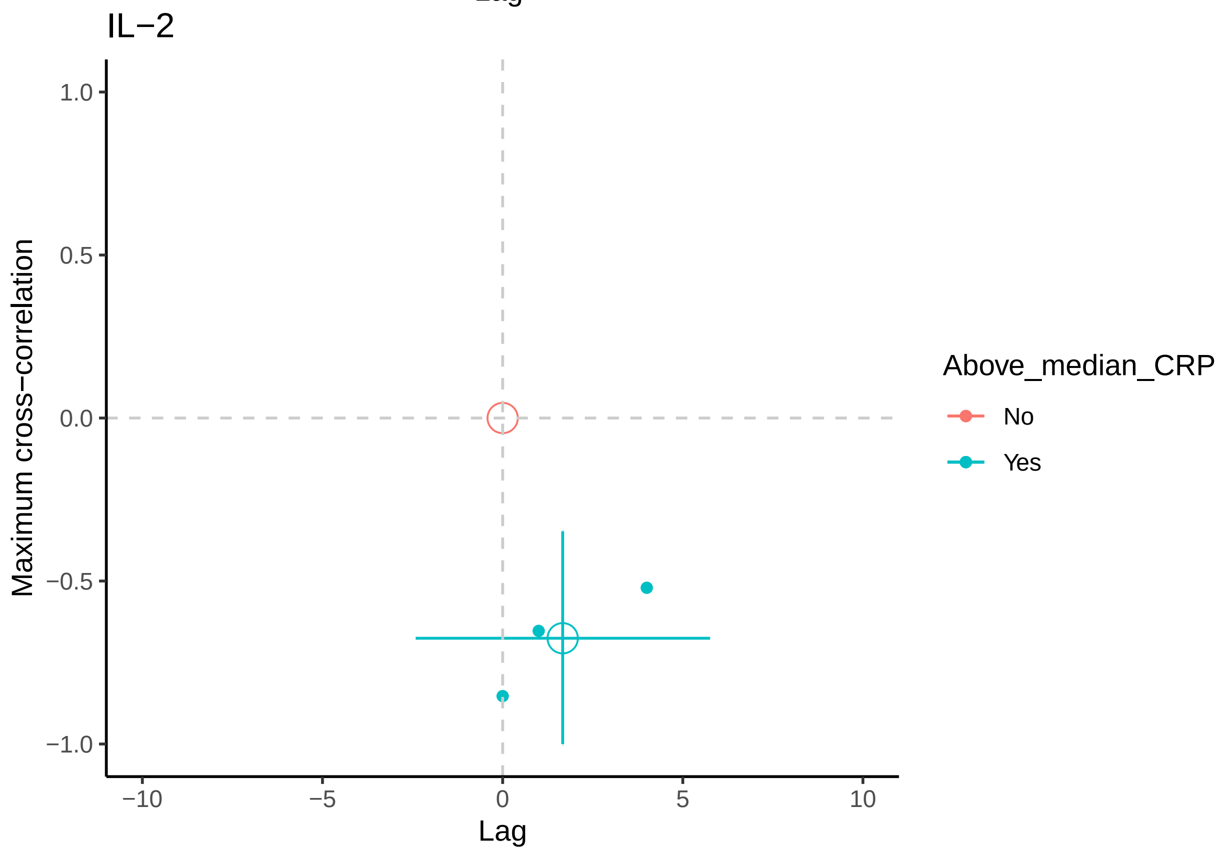
IL−2
Maximum cross−correlation
1.0
0.5
0.0
−0.5
−1.0
−10
−5
0
5
10
Lag
Above_median_CRP
No
Yes

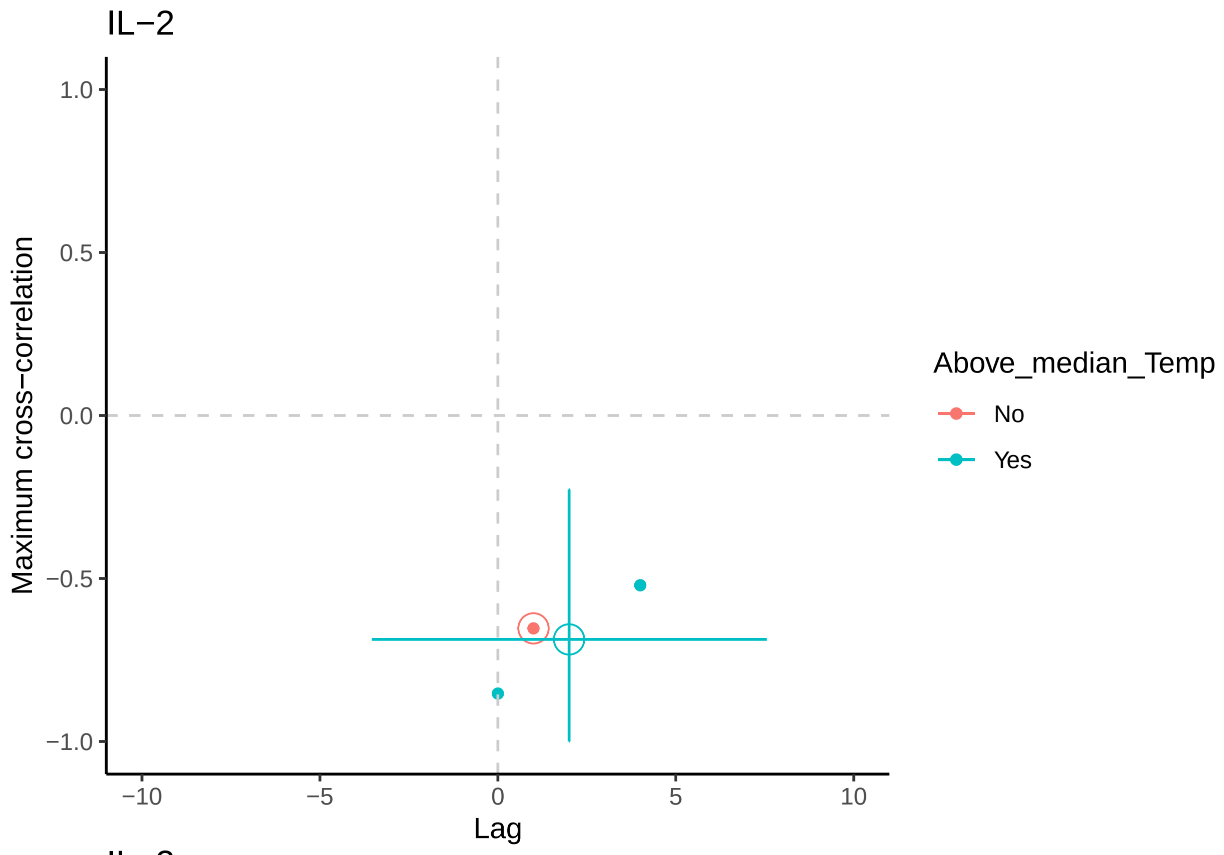
IL−2
Maximum cross−correlation
1.0
0.5
0.0
−0.5
−1.0
−10
−5
0
5
10
Lag
Above_median_Temp
No
Yes

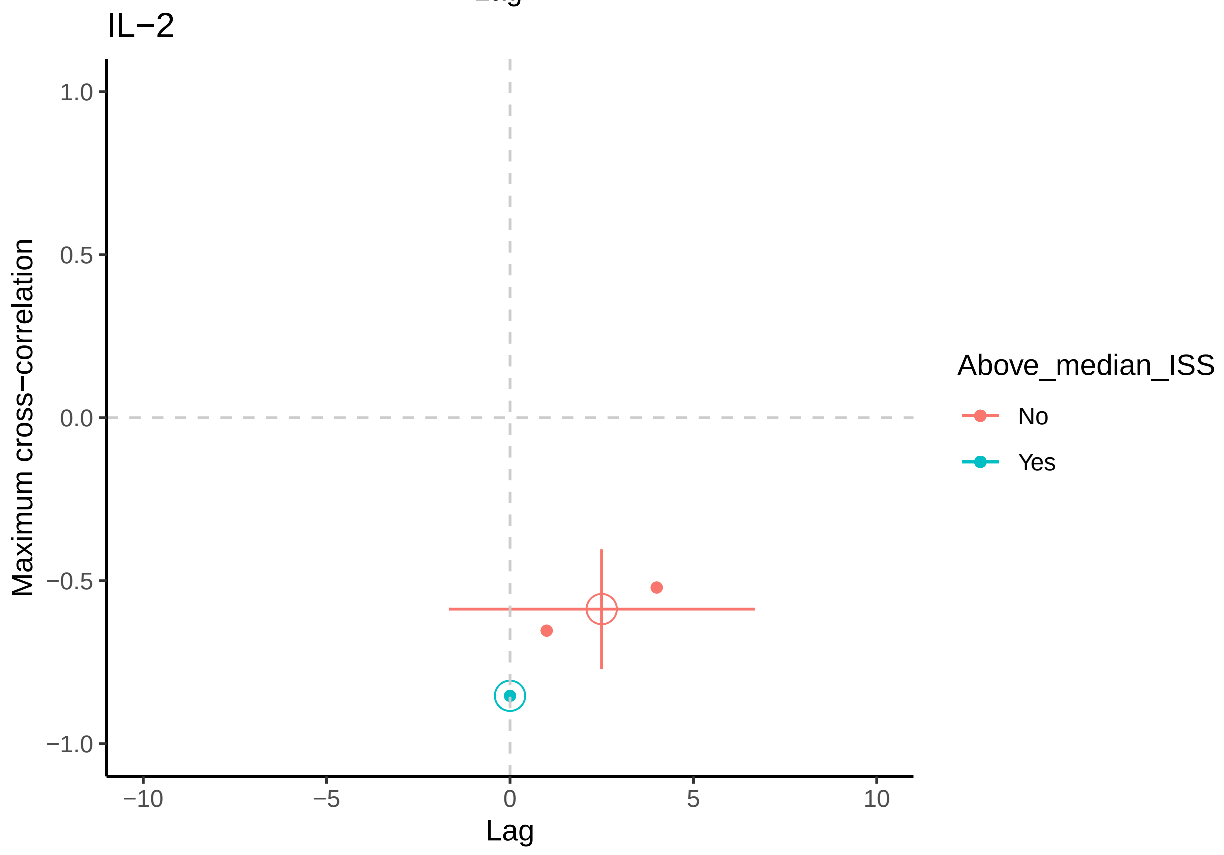
IL−2
Maximum cross−correlation
1.0
0.5
0.0
−0.5
−1.0
−10
−5
0
5
10
Lag
Above_median_ISS
No
Yes

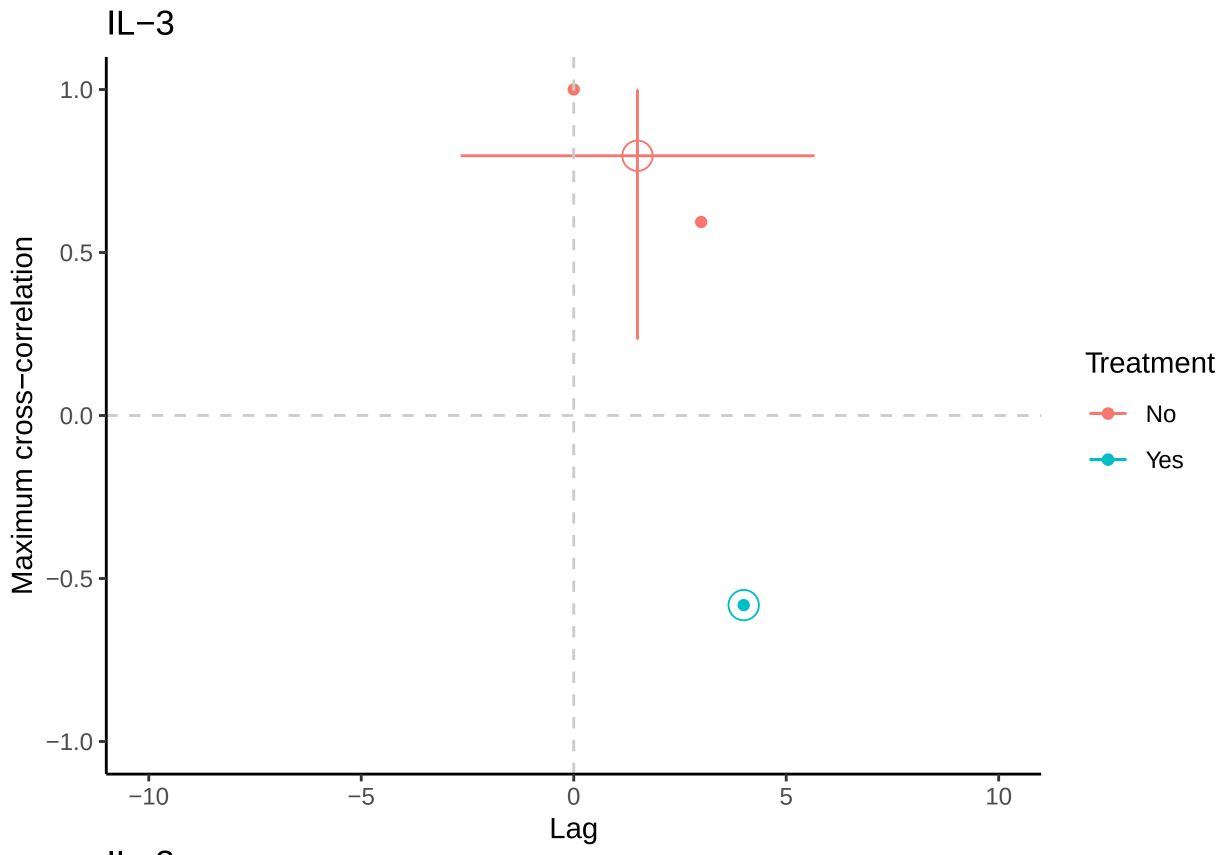
IL−3
Maximum cross−correlation
1.0
0.5
0.0
−0.5
−1.0
−10
−5
0
5
10
Lag
Treatment
No
Yes

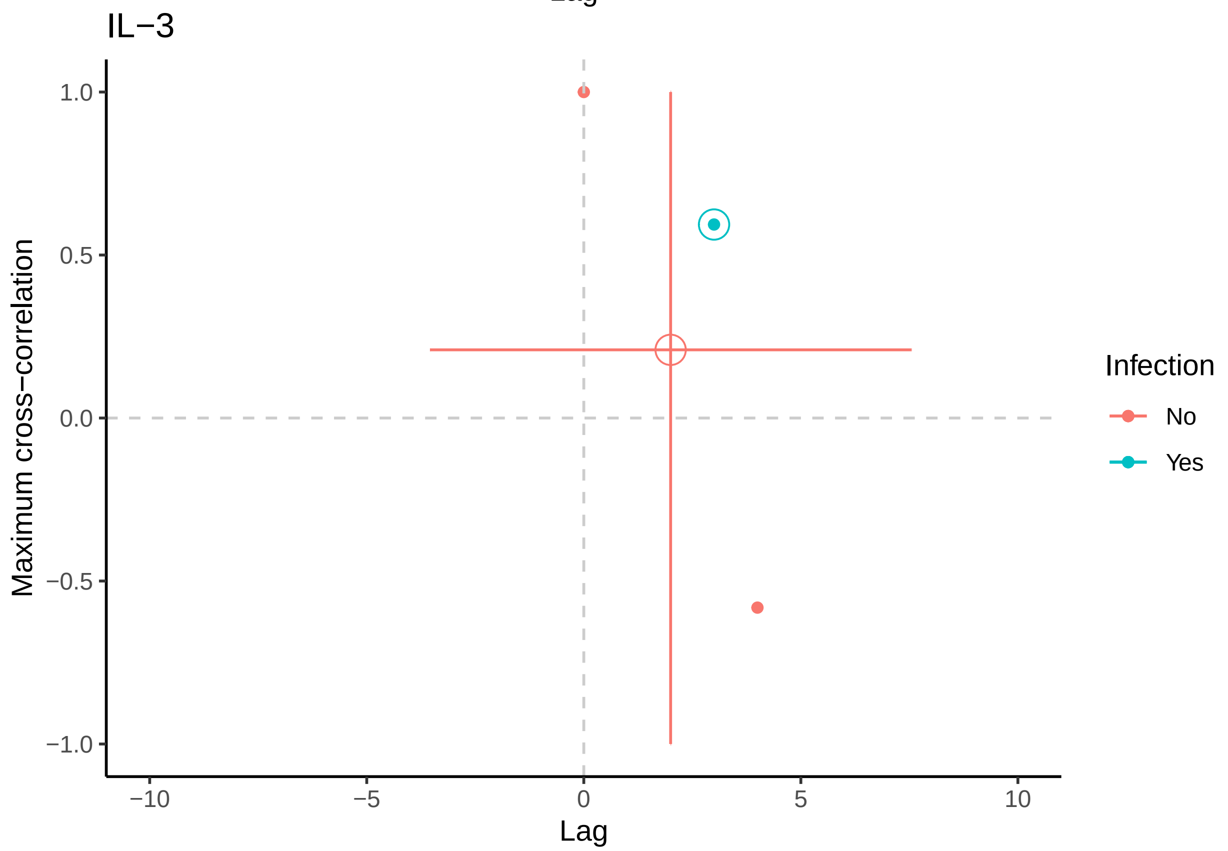
IL−3
Maximum cross−correlation
1.0
0.5
0.0
−0.5
−1.0
−10
−5
0
5
10
Lag
Infection
No
Yes

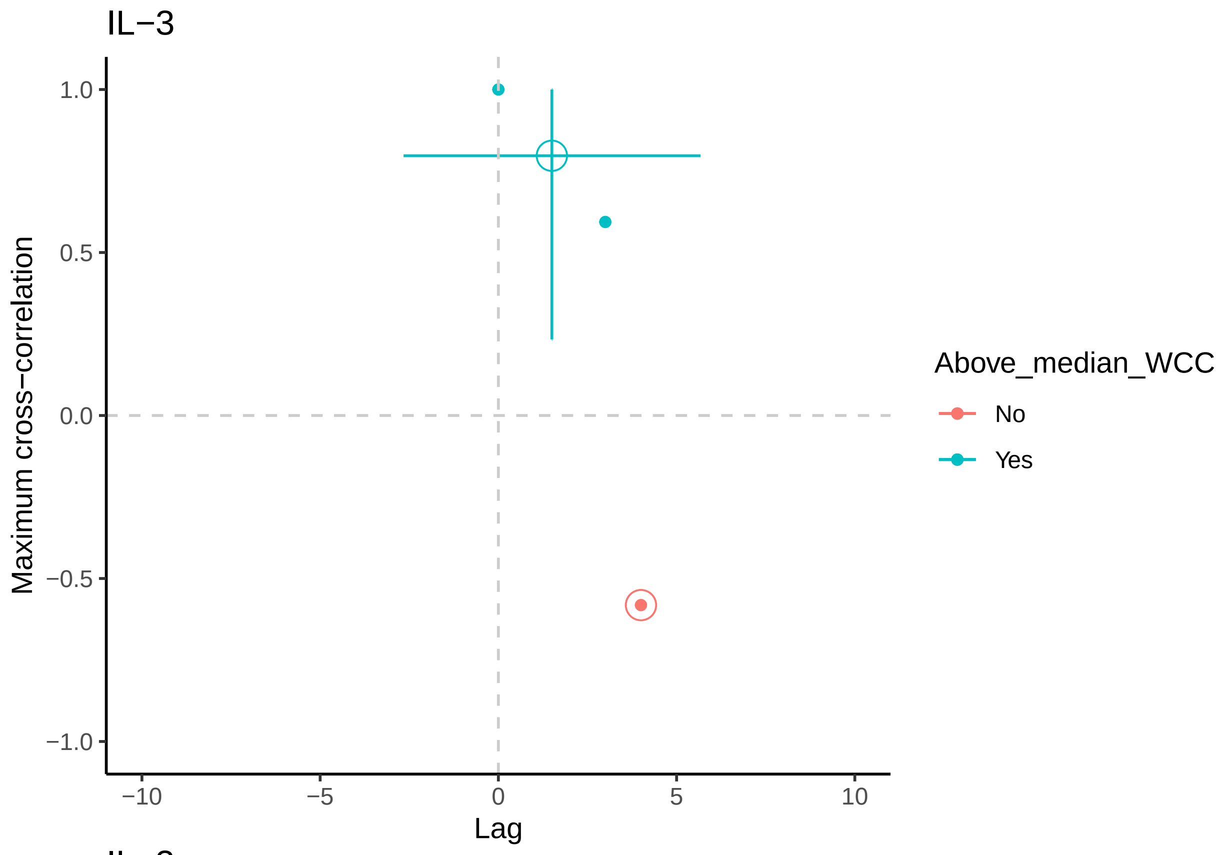
IL−3
Maximum cross−correlation
1.0
0.5
0.0
−0.5
−1.0
−10
−5
0
5
10
Lag
Above_median_WCC
No
Yes

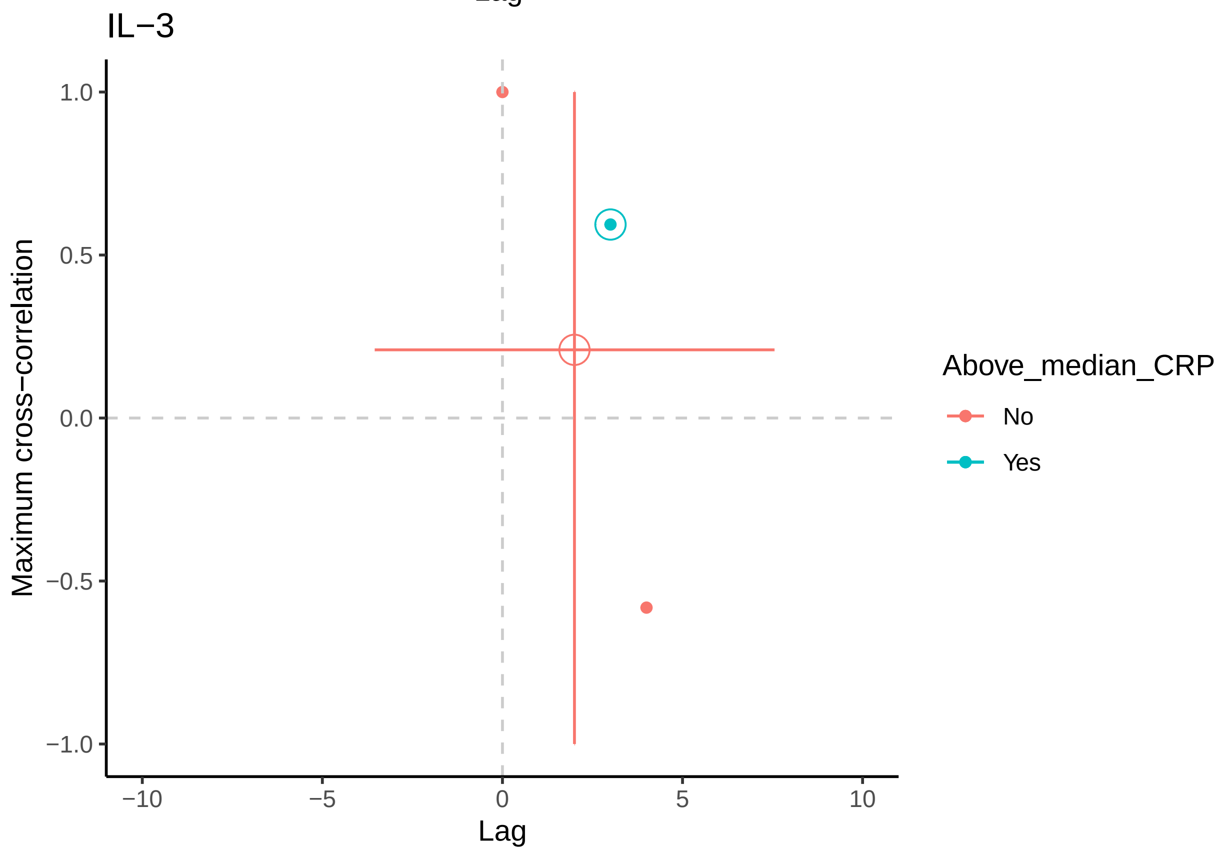
IL−3
Maximum cross−correlation
1.0
0.5
0.0
−0.5
−1.0
−10
−5
0
5
10
Lag
Above_median_CRP
No
Yes

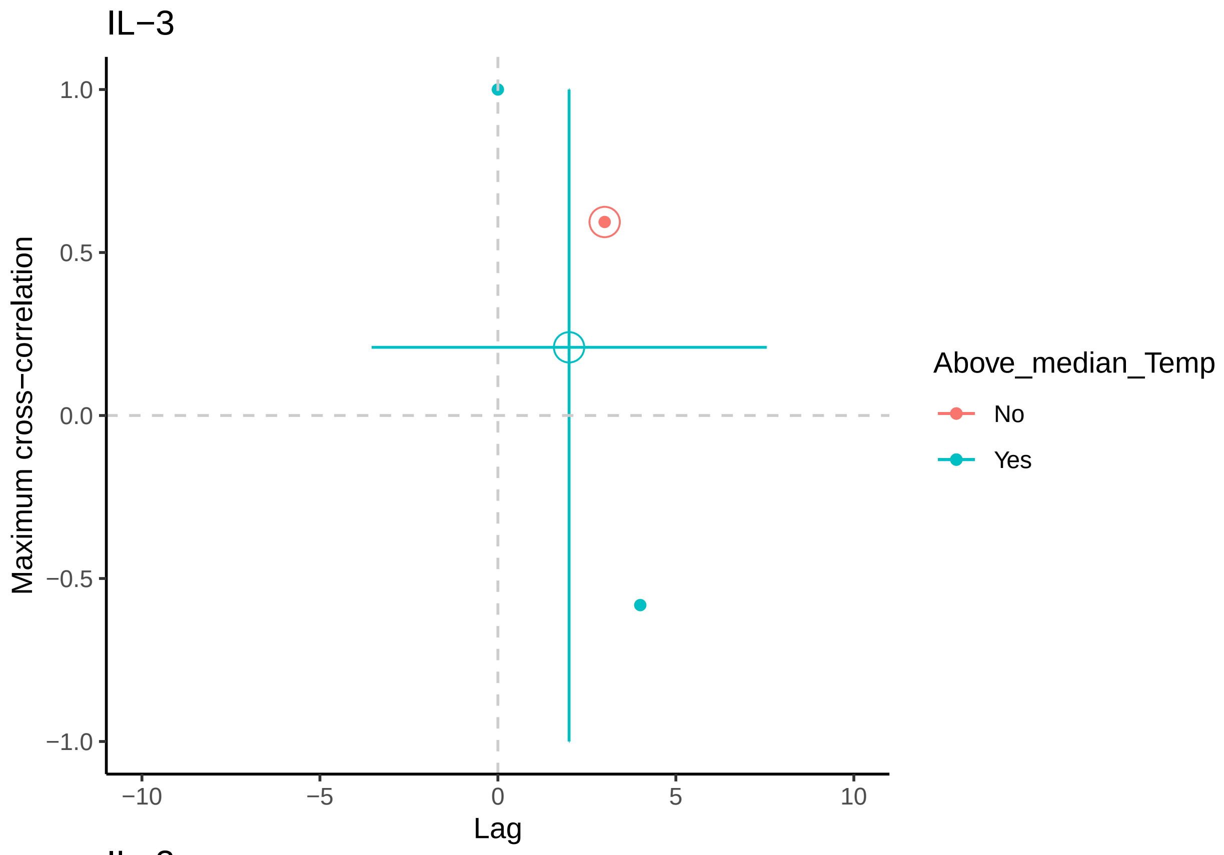
IL−3
Maximum cross−correlation
1.0
0.5
0.0
−0.5
−1.0
−10
−5
0
5
10
Lag
Above_median_Temp
No
Yes

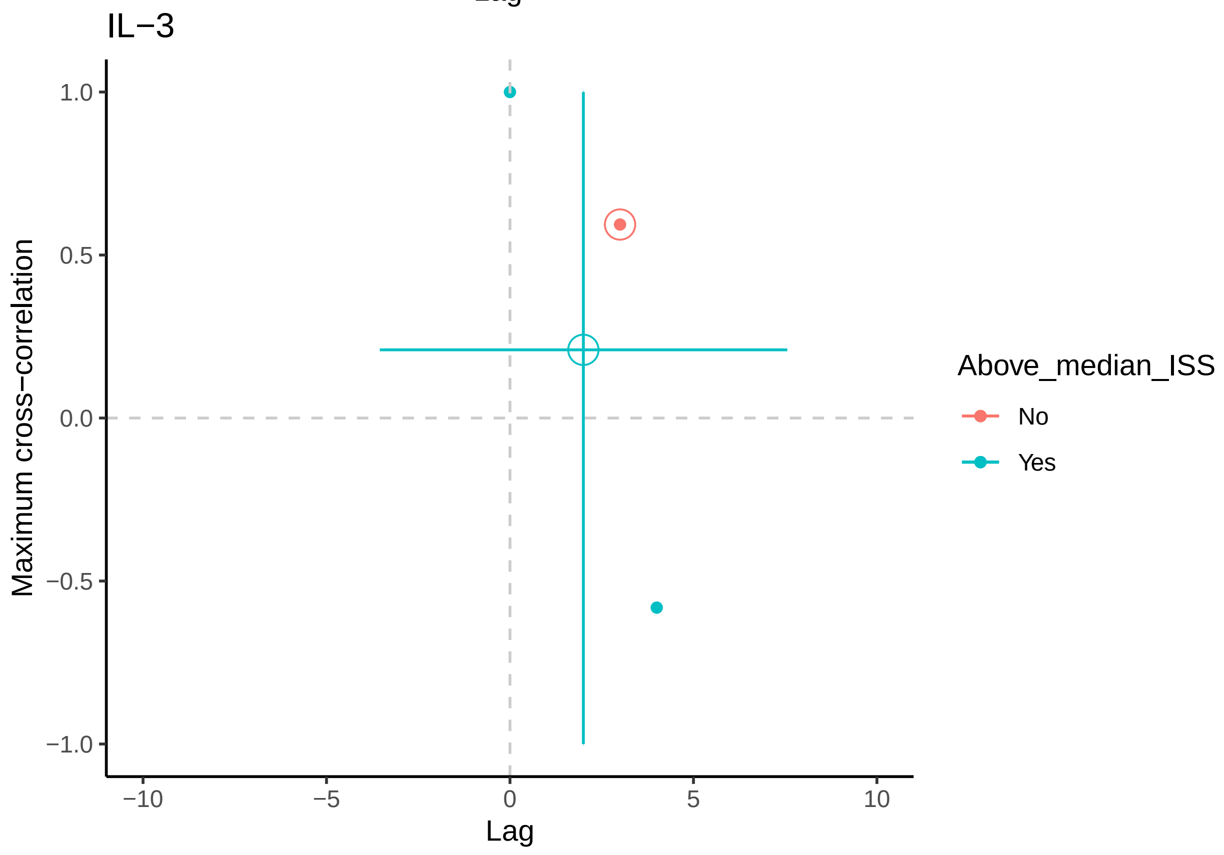
IL−3
Maximum cross−correlation
1.0
0.5
0.0
−0.5
−1.0
−10
−5
0
5
10
Lag
Above_median_ISS
No
Yes

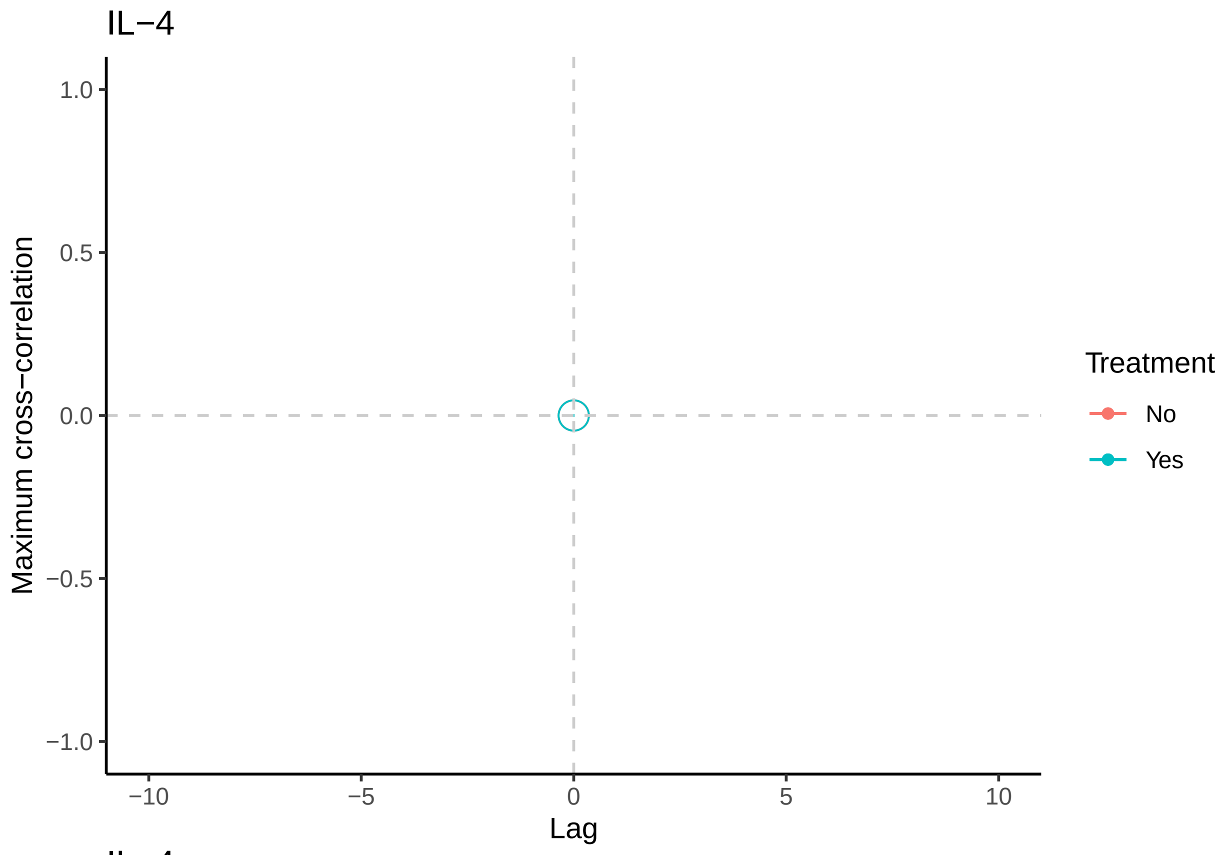
IL−4
Maximum cross−correlation
1.0
0.5
0.0
−0.5
−1.0
−10
−5
0
5
10
Lag
Treatment
No
Yes

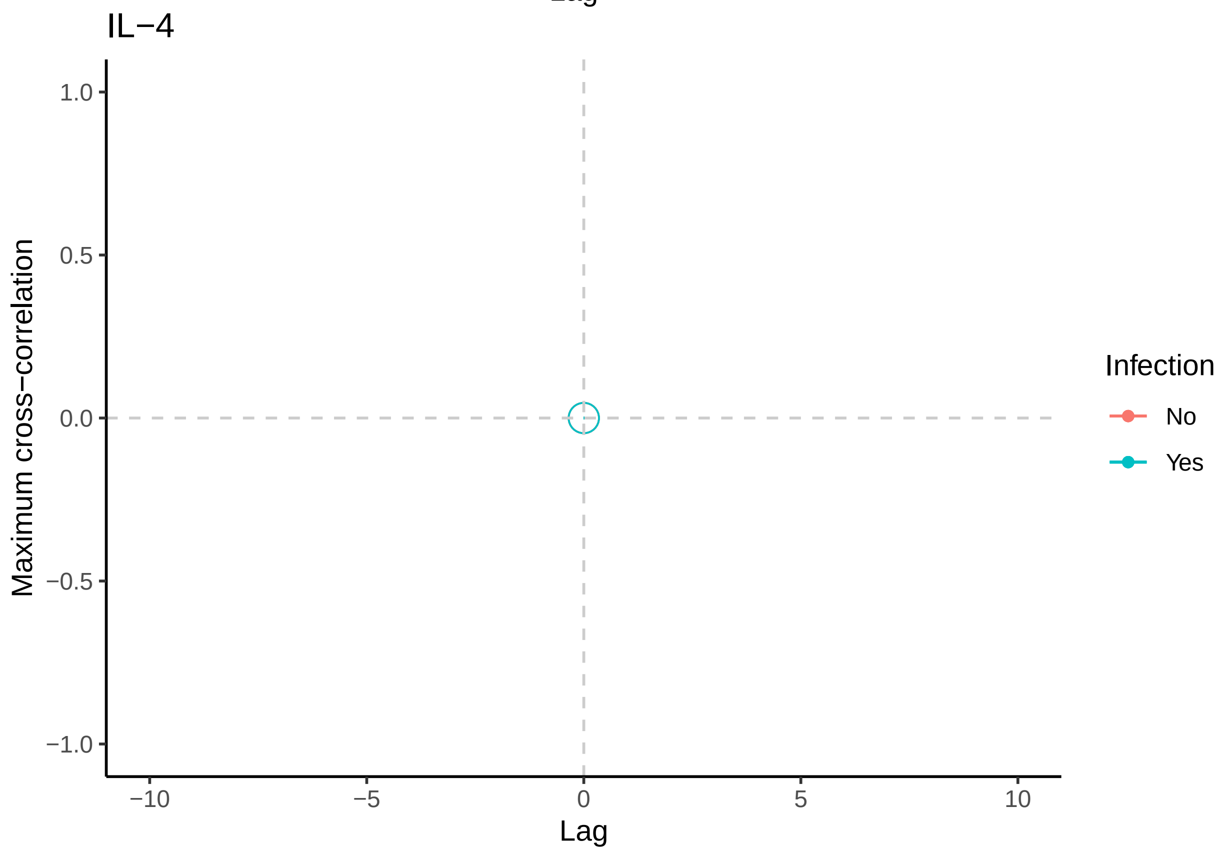
IL−4
Maximum cross−correlation
1.0
0.5
0.0
−0.5
−1.0
−10
−5
0
5
10
Lag
Infection
No
Yes

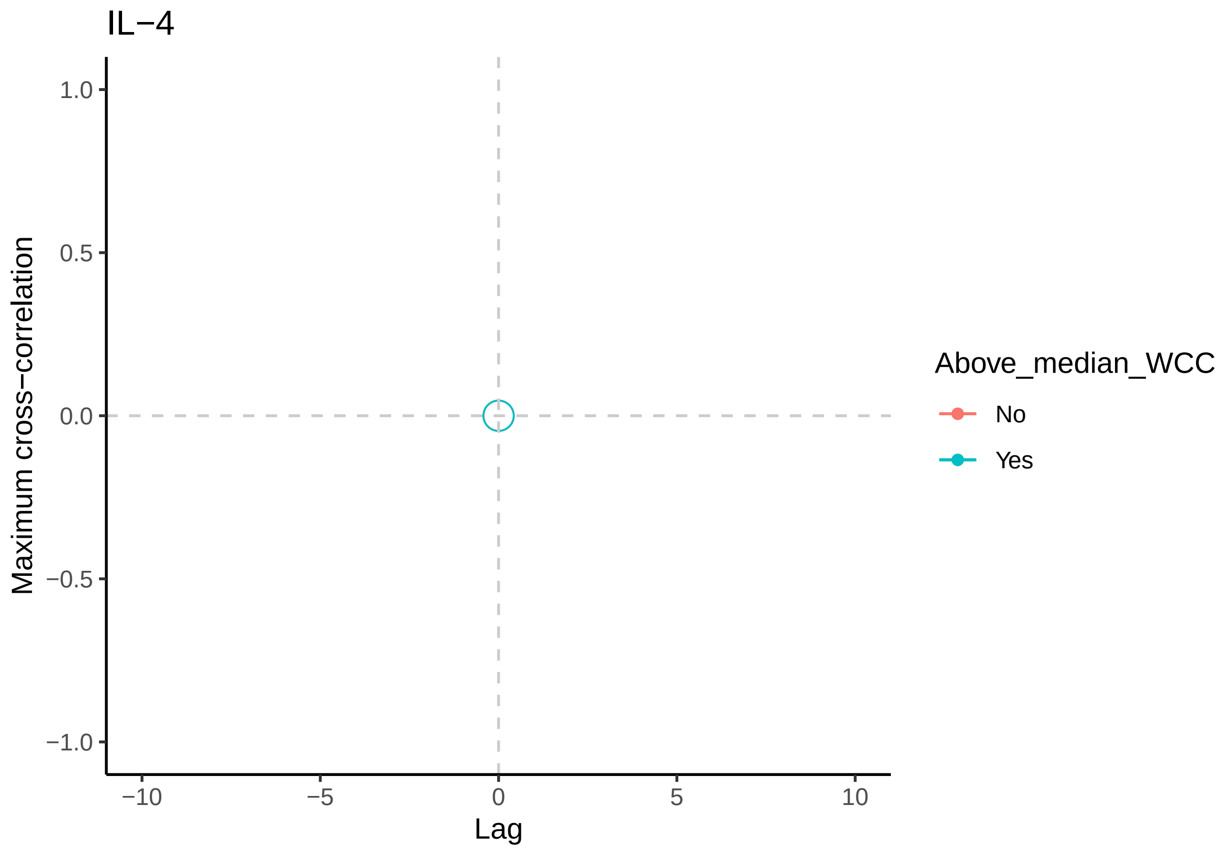

IL−4
Maximum cross−correlation
1.0
0.5
0.0
−0.5
−1.0
−10
−5
0
5
10
Lag
Above_median_WCC
No
Yes

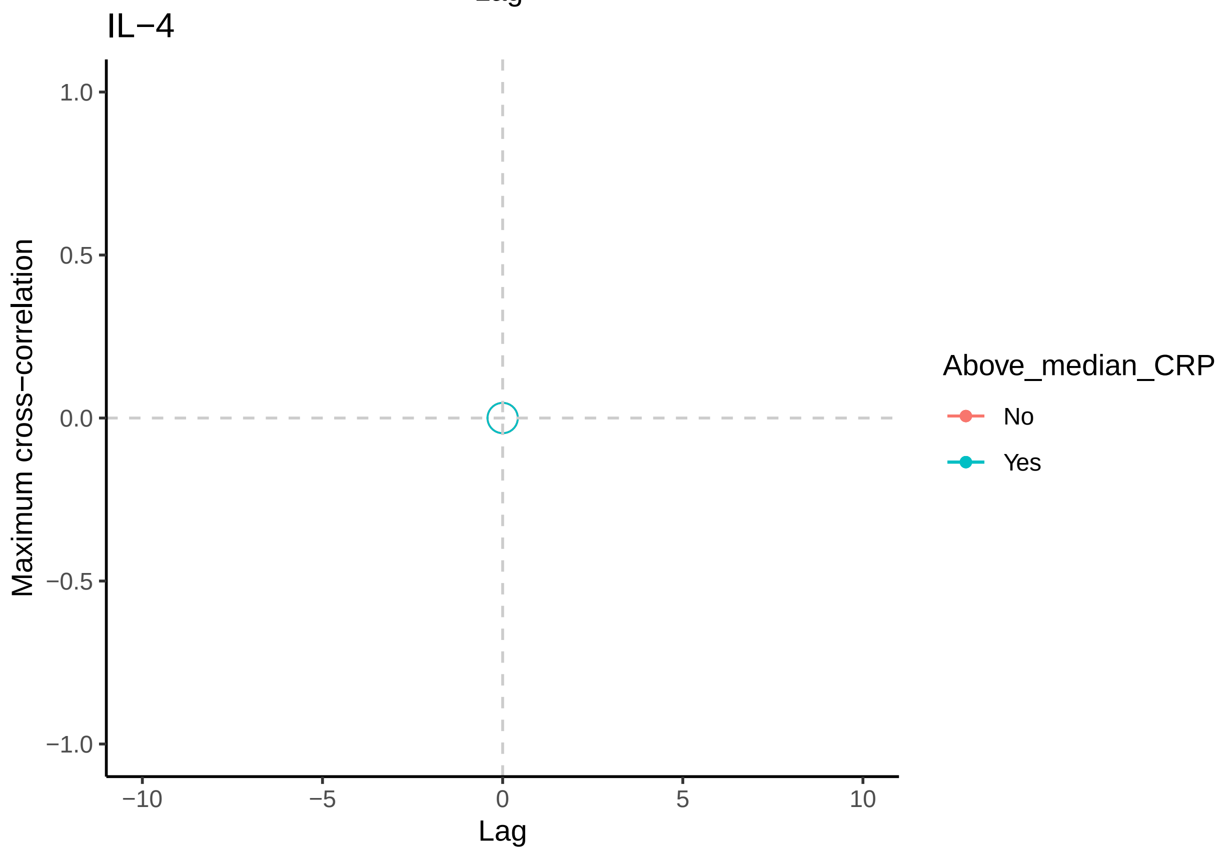

IL−4
Maximum cross−correlation
1.0
0.5
0.0
−0.5
−1.0
−10
−5
0
5
10
Lag
Above_median_CRP
No
Yes

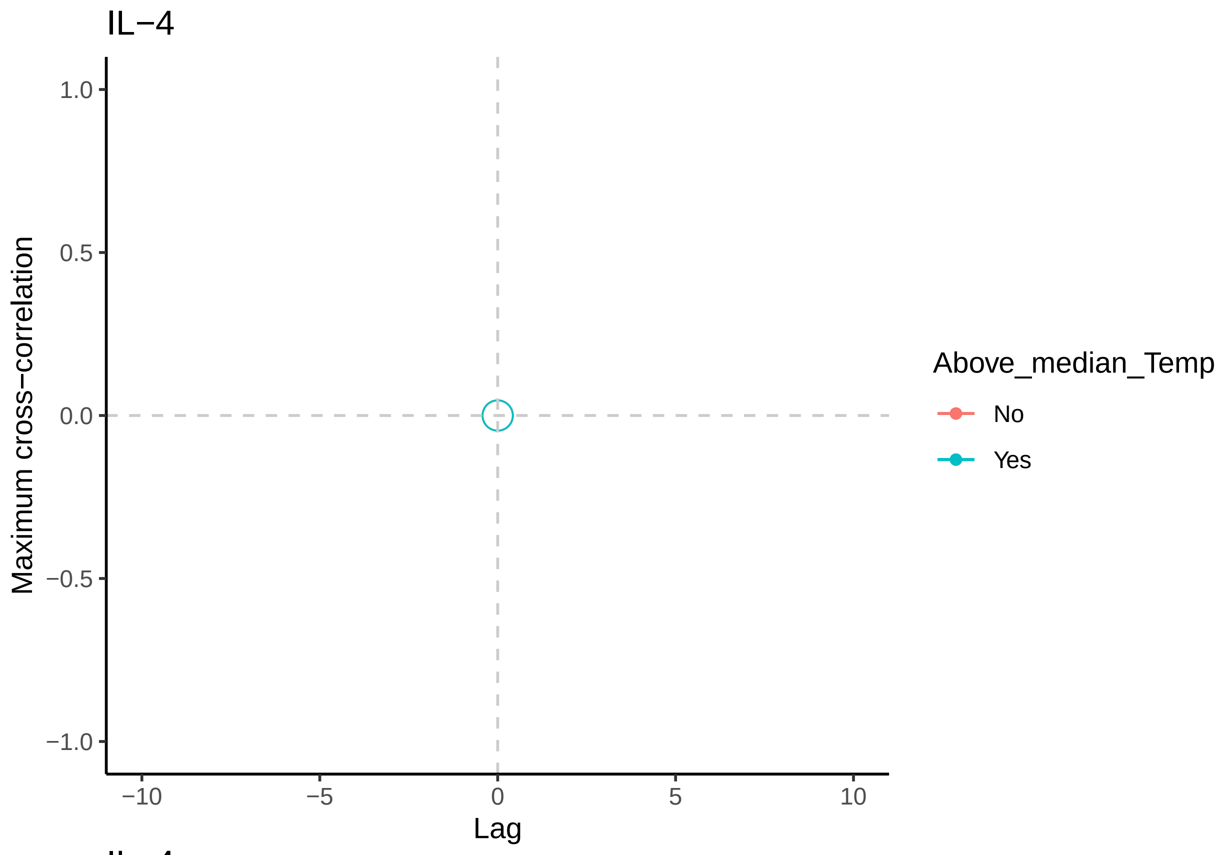
IL−4
Maximum cross−correlation
1.0
0.5
0.0
−0.5
−1.0
−10
−5
0
5
10
Lag
Above_median_Temp
No
Yes

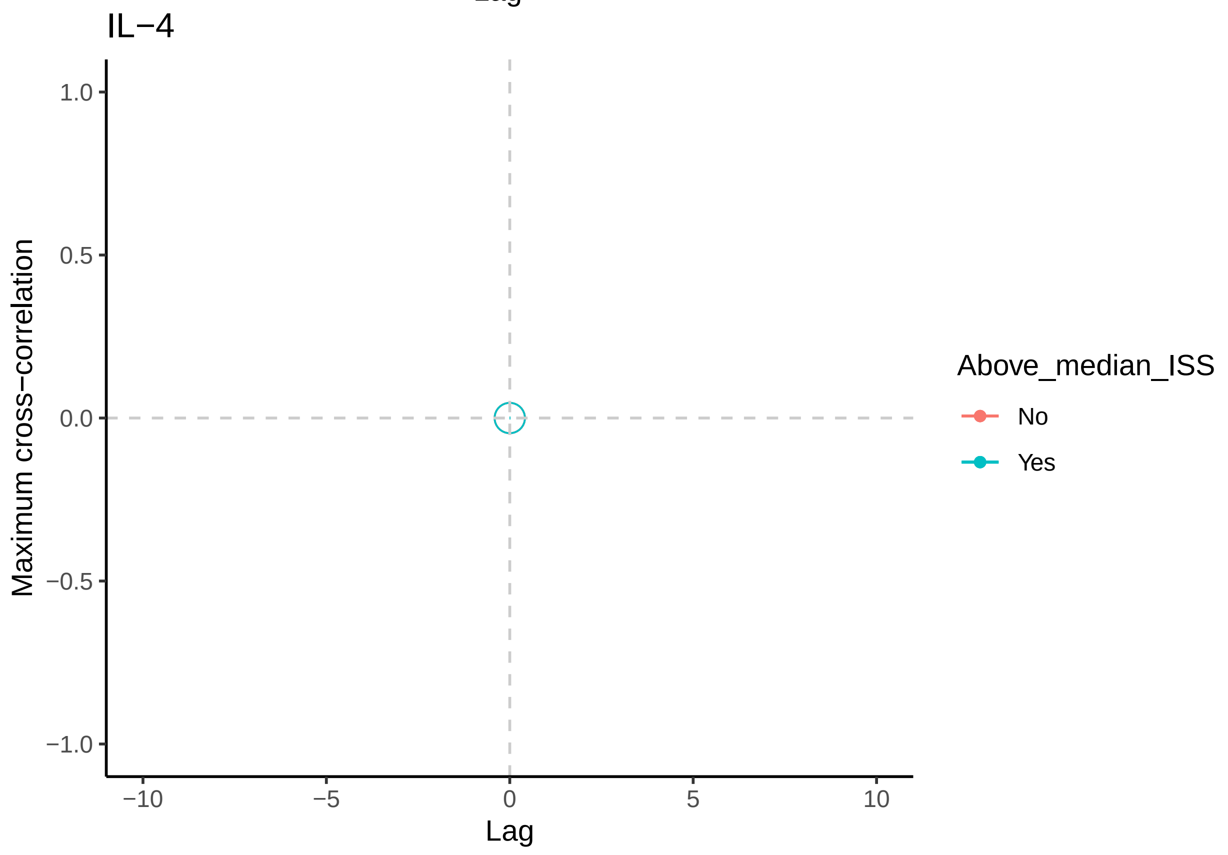
IL−4
Maximum cross−correlation
1.0
0.5
0.0
−0.5
−1.0
−10
−5
0
5
10
Lag
Above_median_ISS
No
Yes

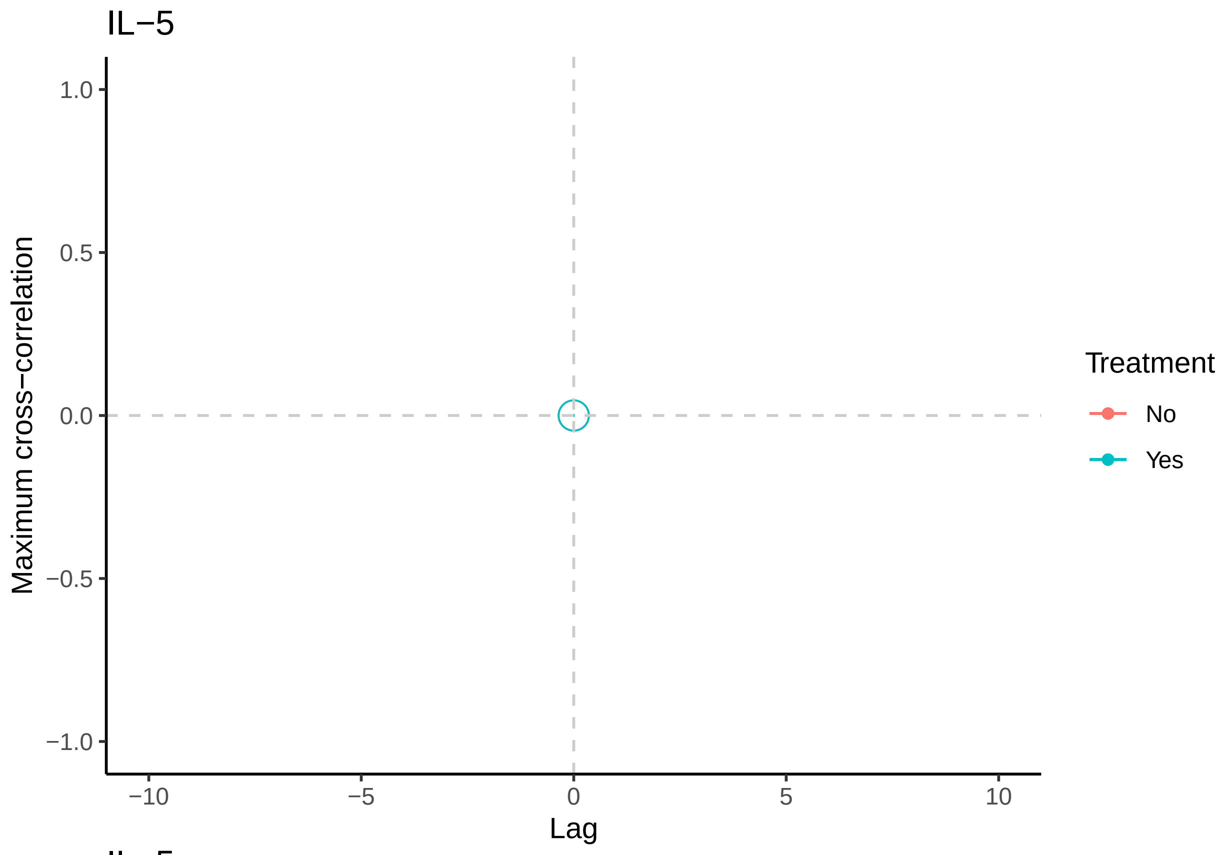
IL−5
1.0
0.5
0.0
−0.5
−1.0
Maximum cross−correlation
−10
−5
0
5
10
Lag
Treatment
No
Yes

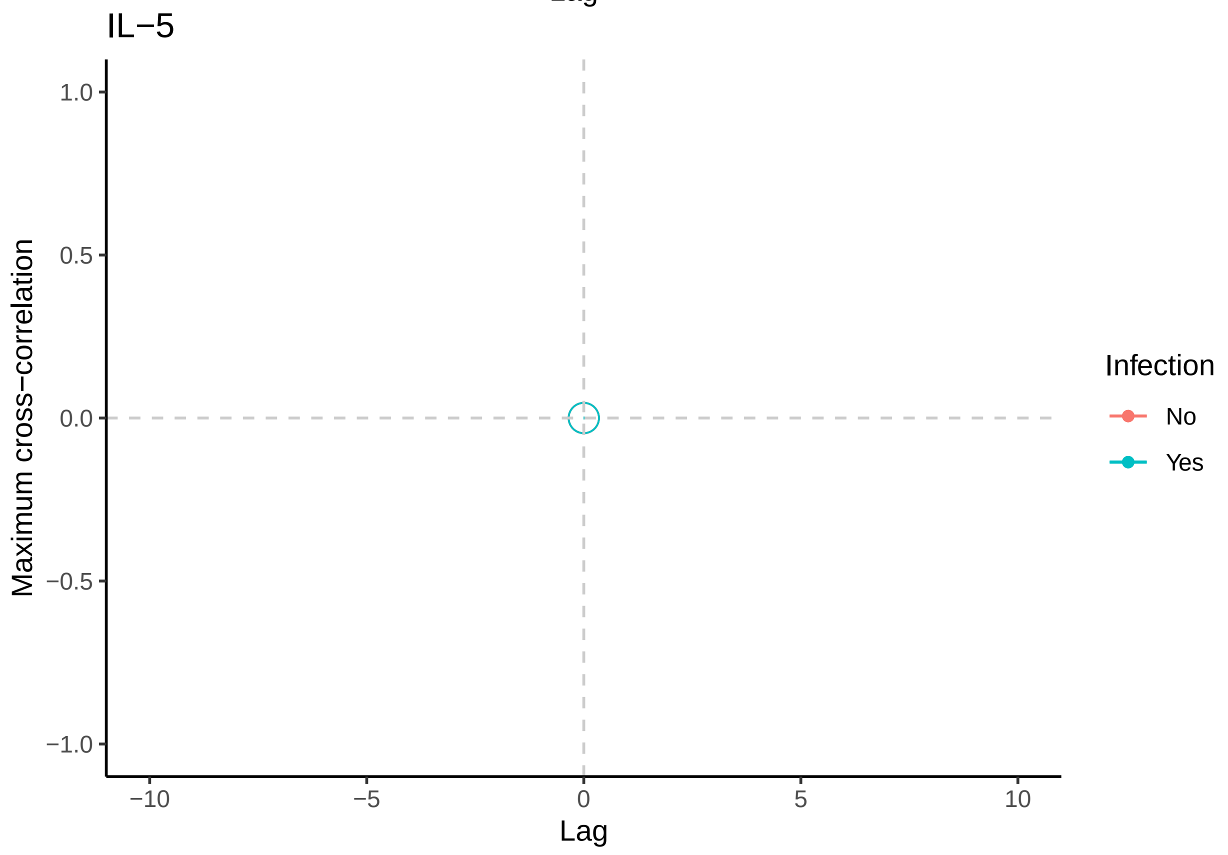
IL−5
1.0
0.5
0.0
−0.5
−1.0
Maximum cross−correlation
−10
−5
0
5
10
Lag
Infection
No
Yes

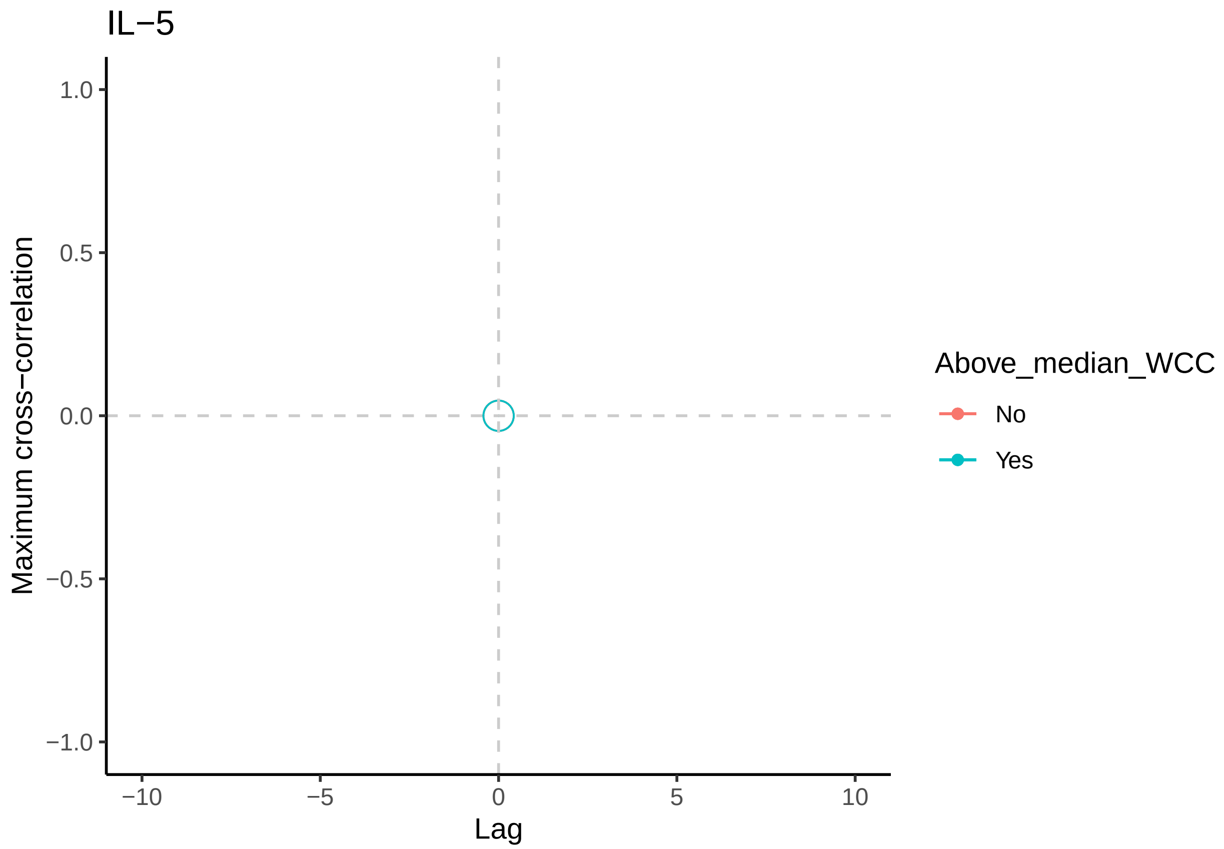
IL−5
Maximum cross−correlation
1.0
0.5
0.0
−0.5
−1.0
−10
−5
0
5
10
Lag
Above_median_WCC
No
Yes

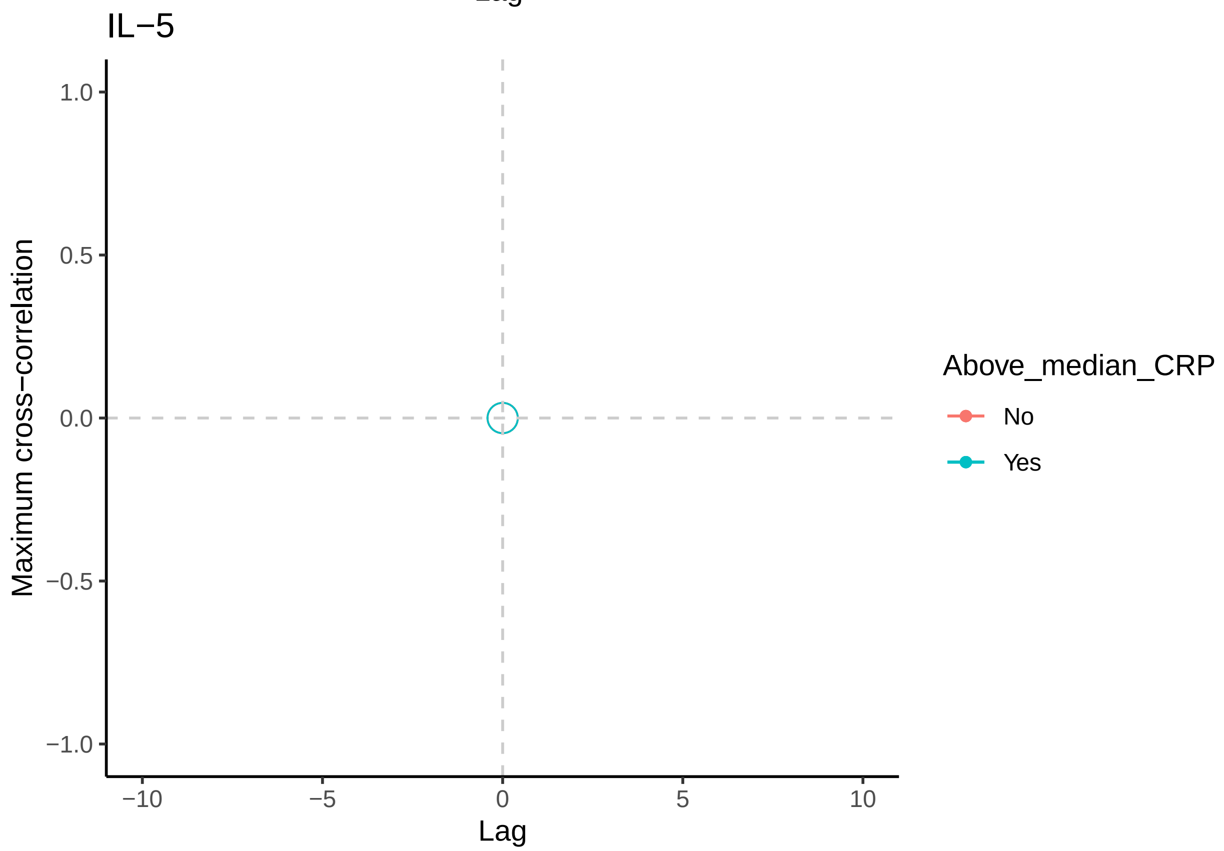
IL−5
Maximum cross−correlation
1.0
0.5
0.0
−0.5
−1.0
−10
−5
0
5
10
Lag
Above_median_CRP
No
Yes

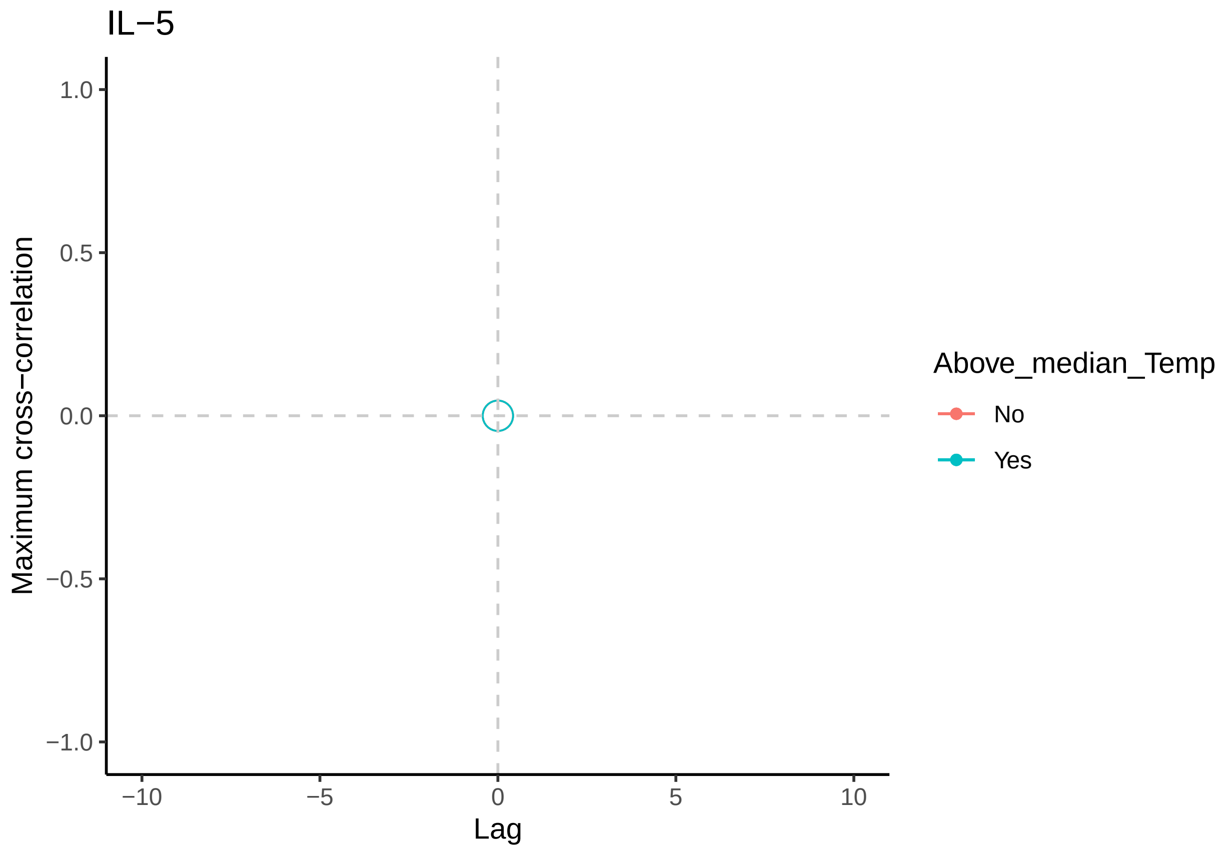
IL−5
Maximum cross−correlation
1.0
0.5
0.0
−0.5
−1.0
−10
−5
0
5
10
Lag
Above_median_Temp
No
Yes

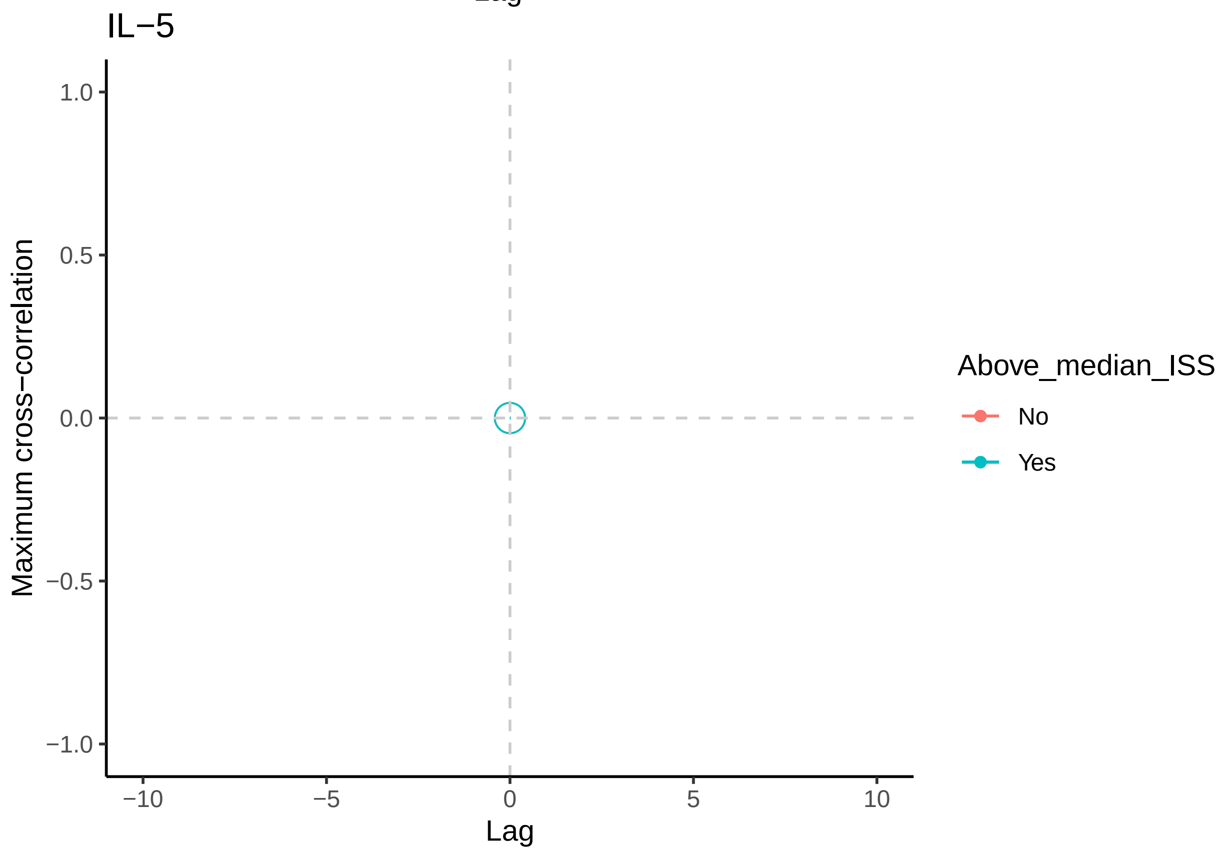
IL−5
Maximum cross−correlation
1.0
0.5
0.0
−0.5
−1.0
−10
−5
0
5
10
Lag
Above_median_ISS
No
Yes

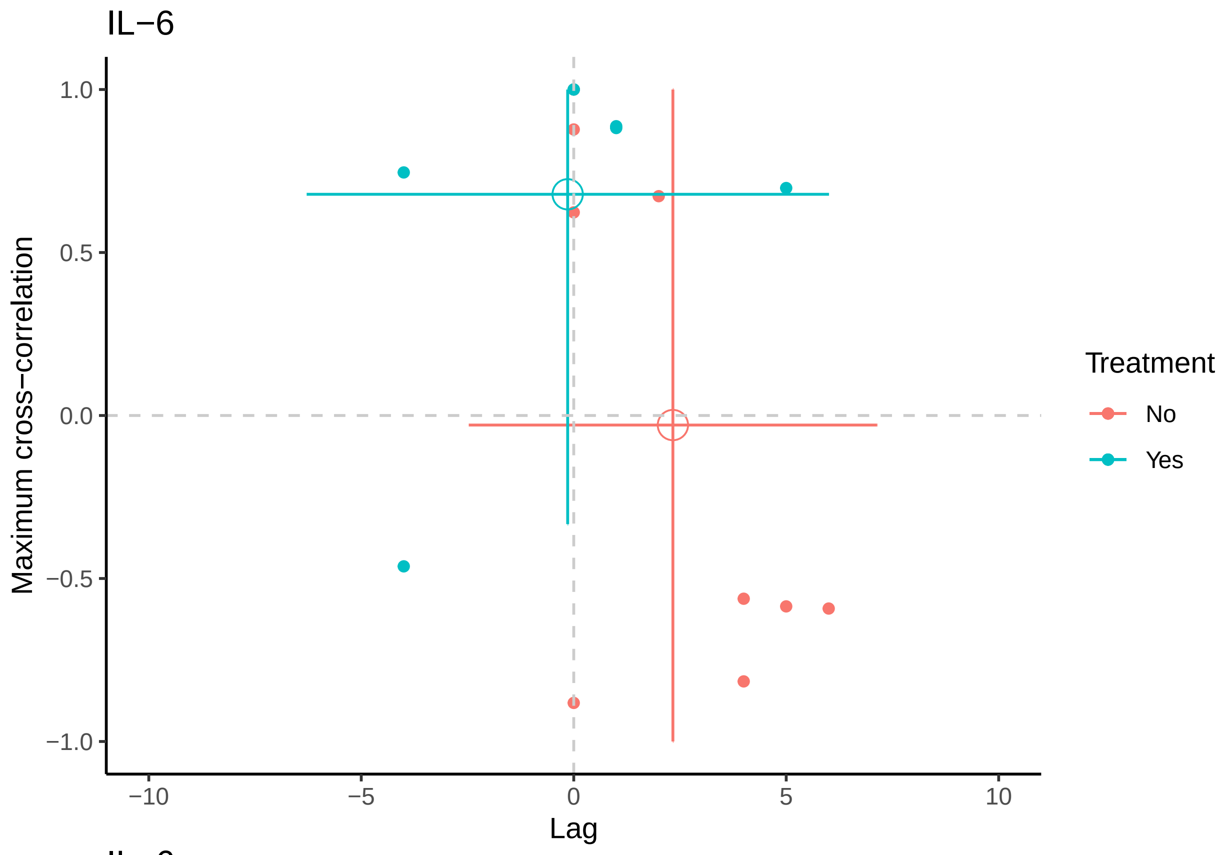
IL−6
Maximum cross−correlation
1.0
0.5
0.0
−0.5
−1.0
−10
−5
0
5
10
Lag
Treatment
No
Yes

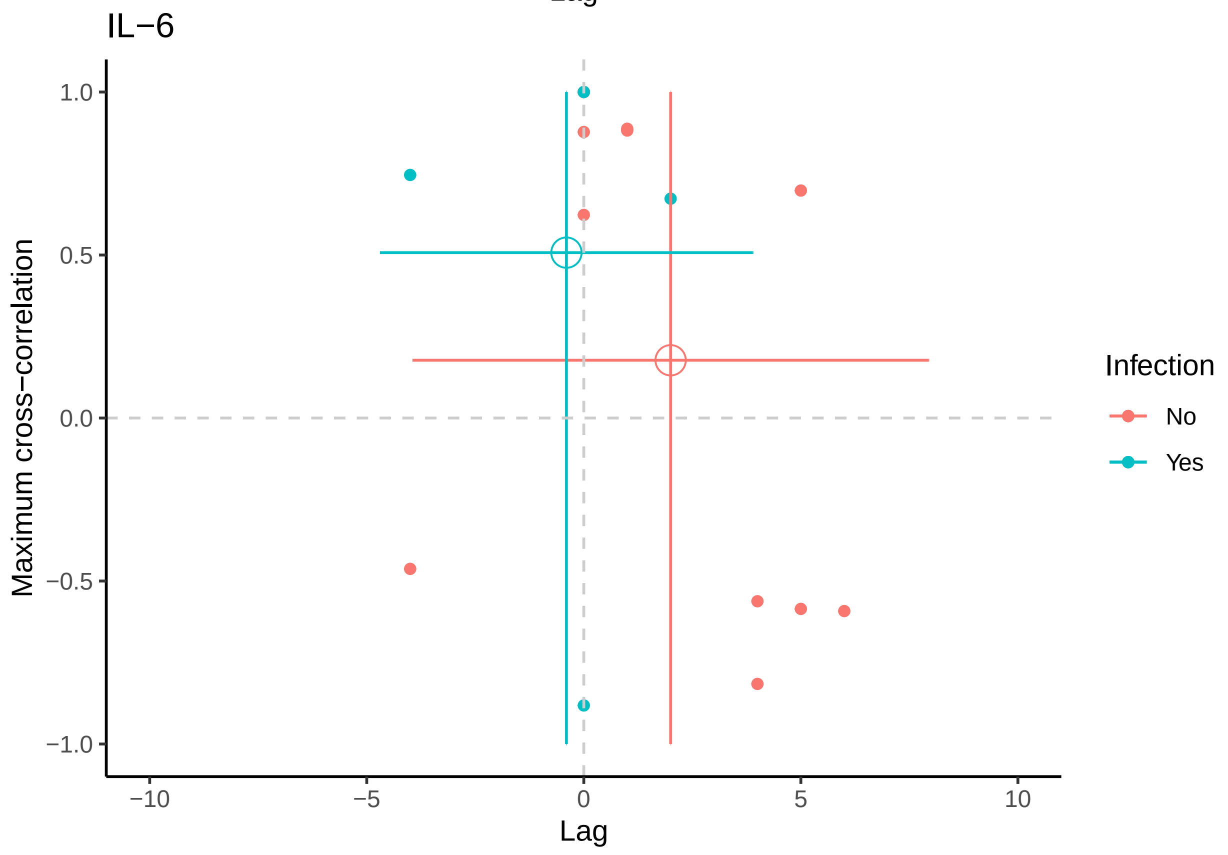
IL−6
Maximum cross−correlation
1.0
0.5
0.0
−0.5
−1.0
−10
−5
0
5
10
Lag
Infection
No
Yes

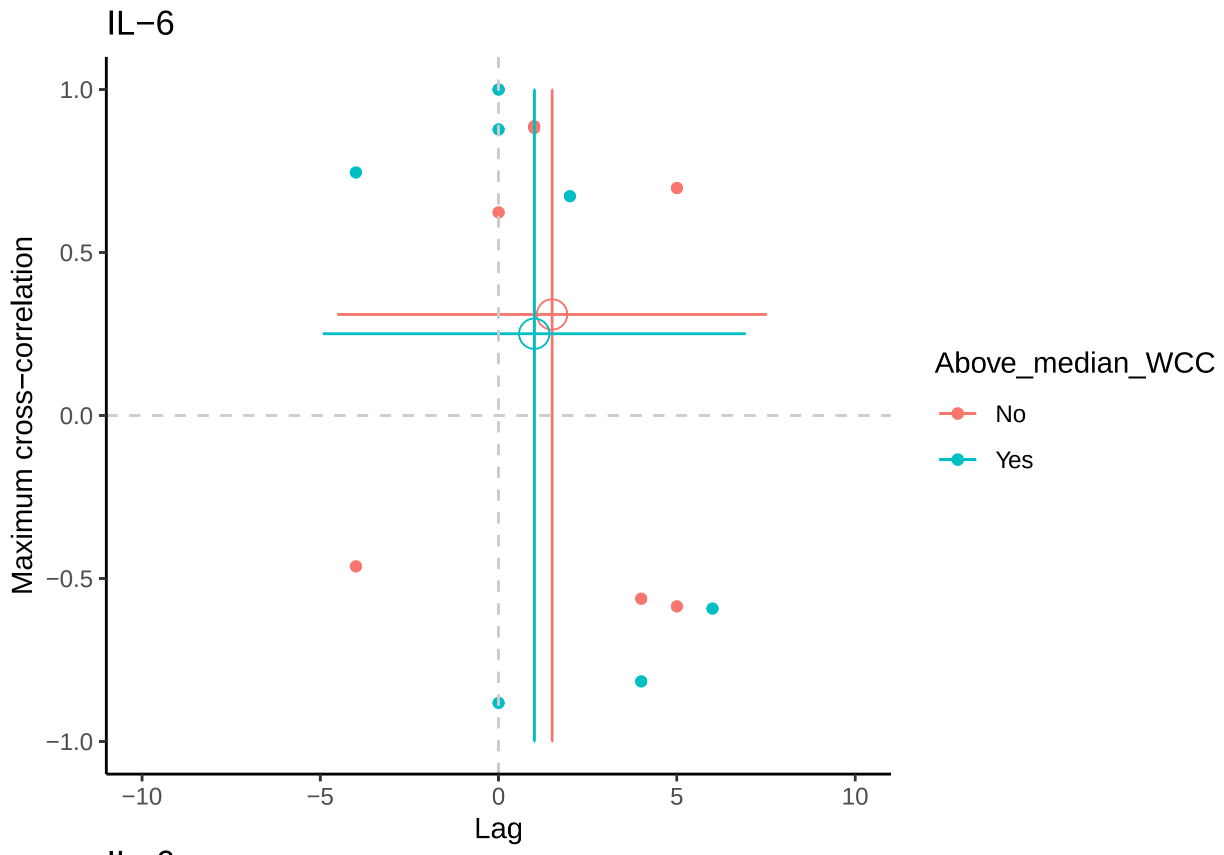
IL−6
Maximum cross−correlation
Lag
Above_median_WCC
No
Yes

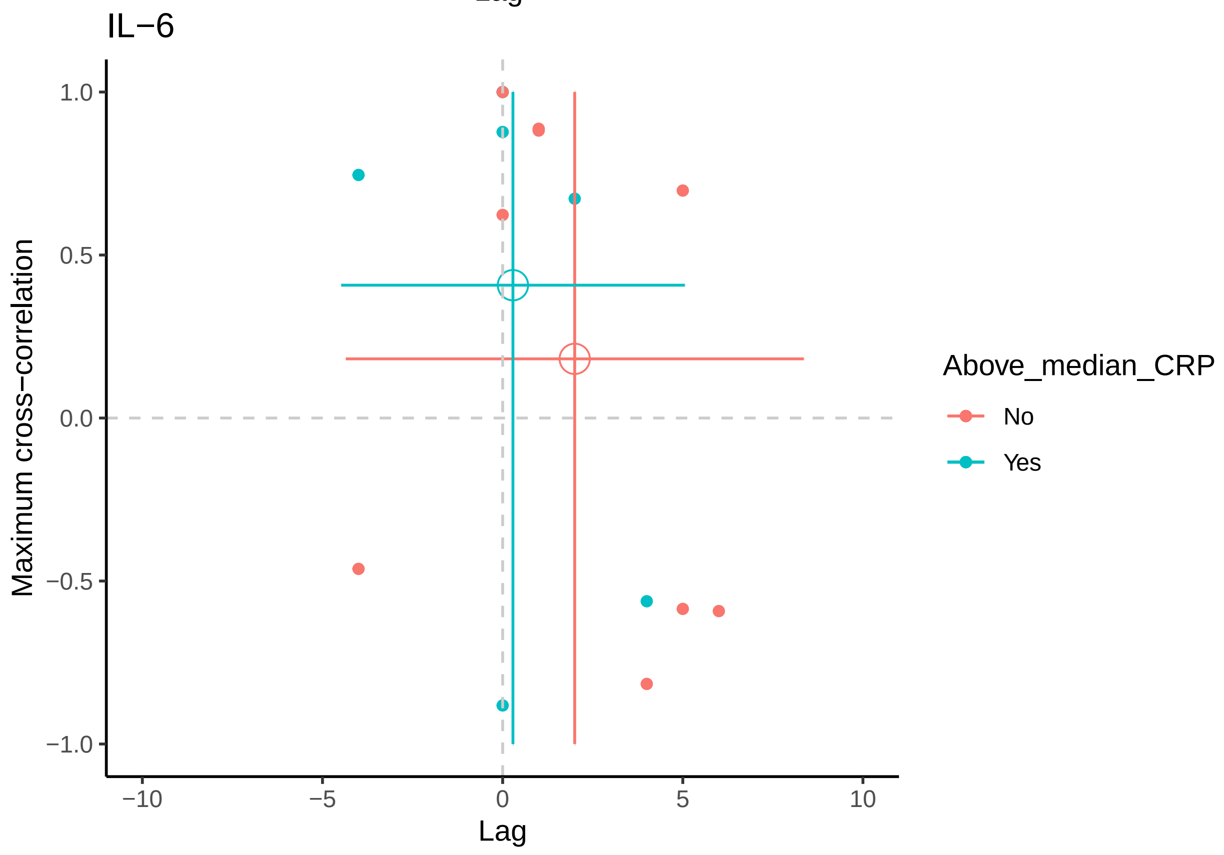
IL−6
Maximum cross−correlation
Lag
Above_median_CRP
No
Yes

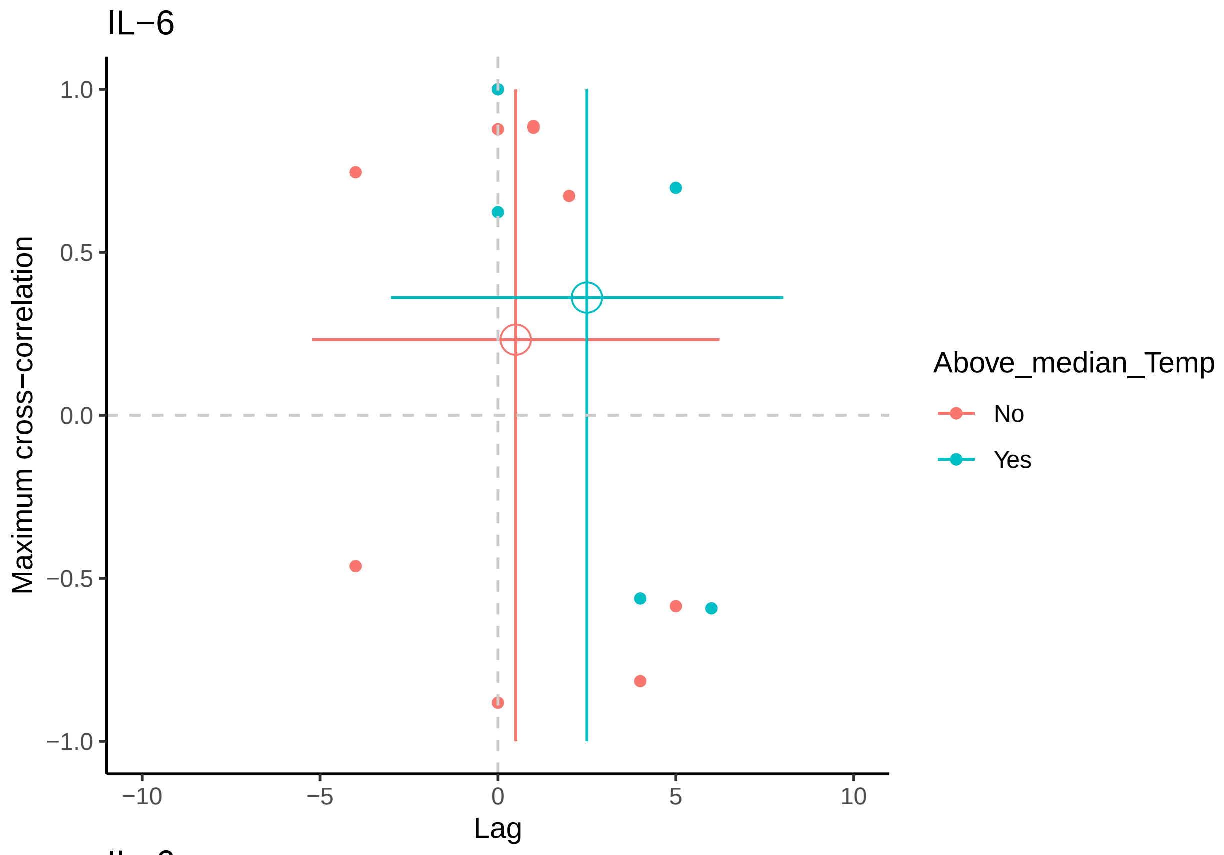
IL−6
Maximum cross−correlation
1.0
0.5
0.0
−0.5
−1.0
−10
−5
0
5
10
Lag
Above_median_Temp
No
Yes

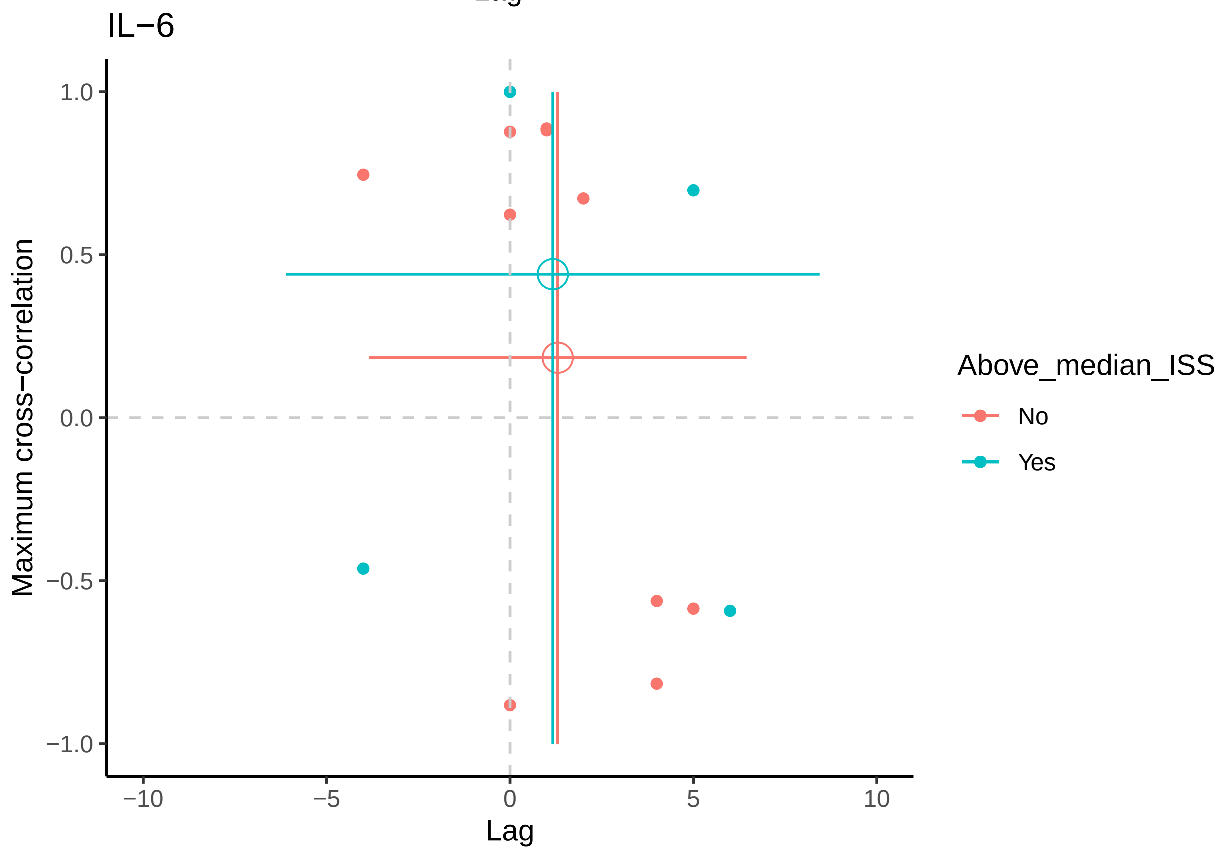
IL−6
Maximum cross−correlation
1.0
0.5
0.0
−0.5
−1.0
−10
−5
0
5
10
Lag
Above_median_ISS
No
Yes

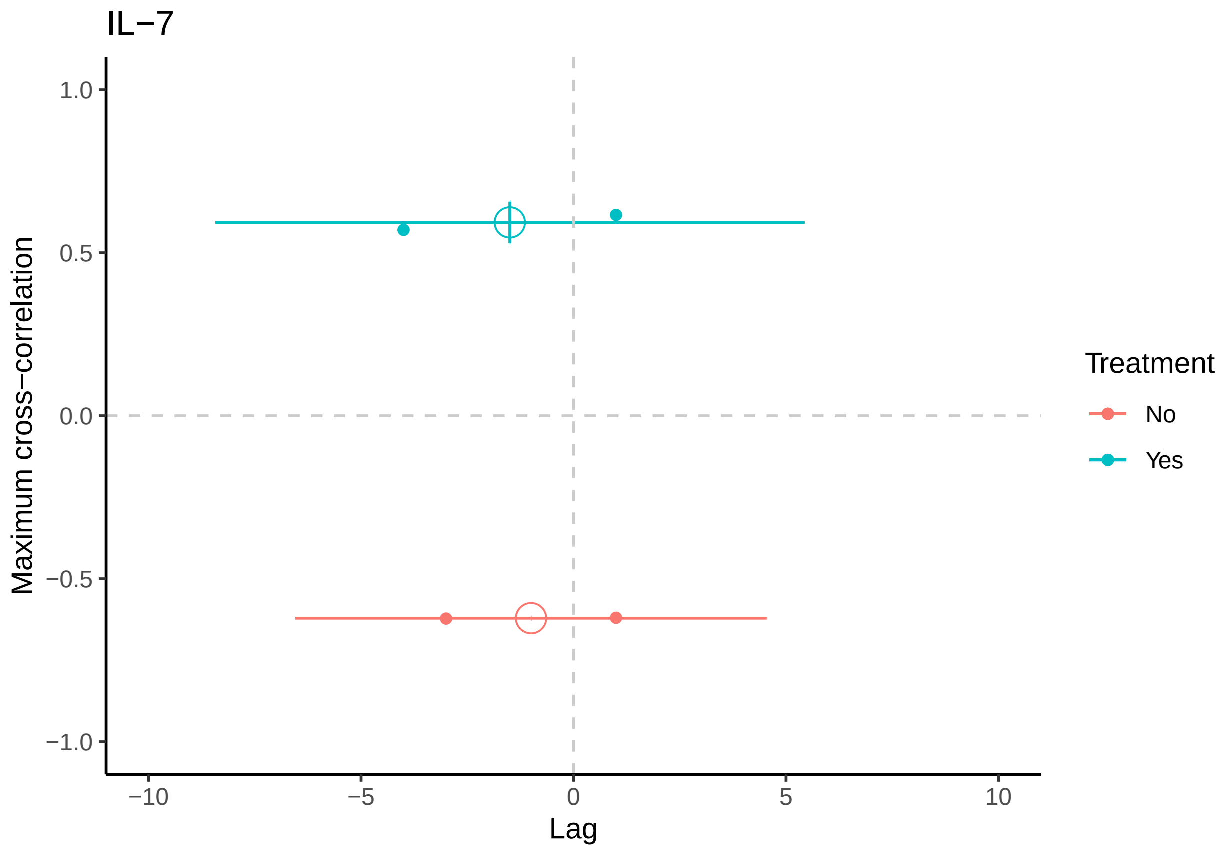
IL−7
Maximum cross−correlation
1.0
0.5
0.0
−0.5
−1.0
−10
−5
0
5
10
Lag
Treatment
No
Yes

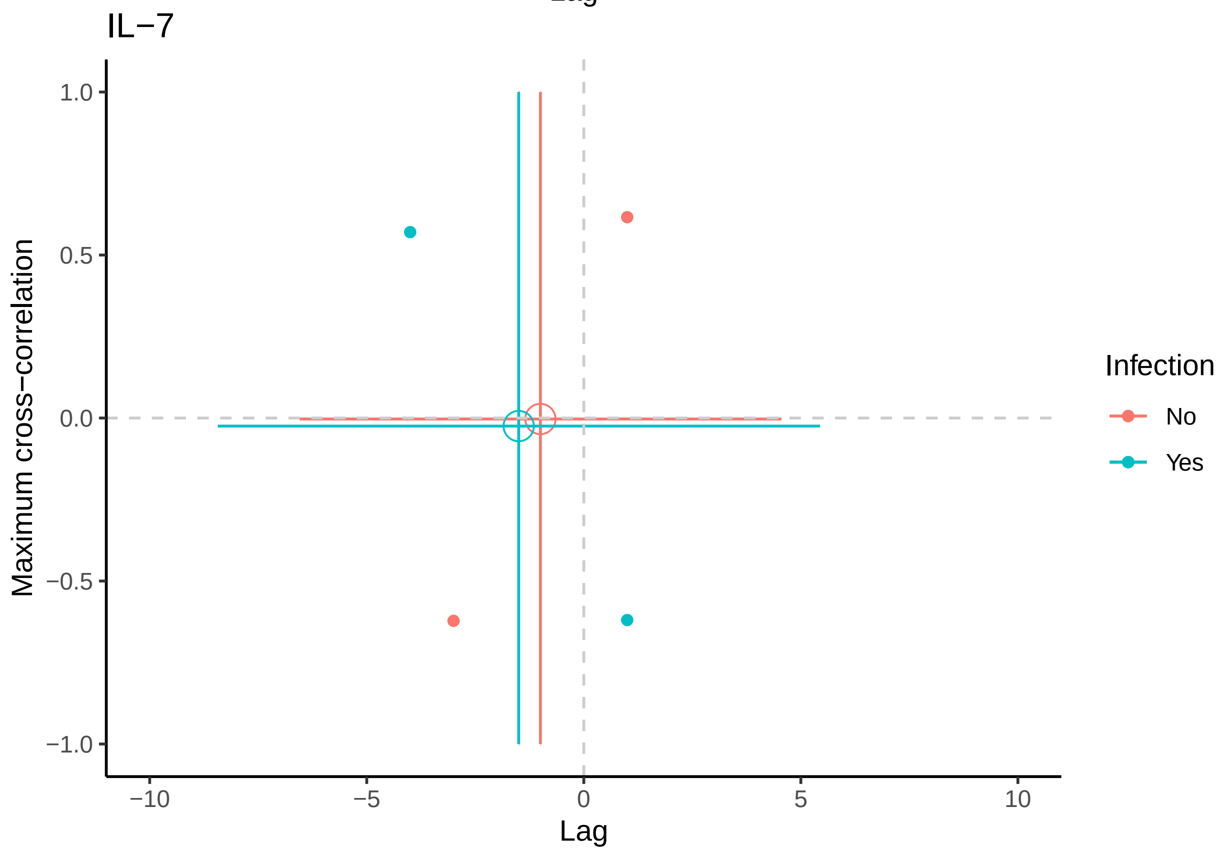
IL−7
Maximum cross−correlation
1.0
0.5
0.0
−0.5
−1.0
−10
−5
0
5
10
Lag
Infection
No
Yes

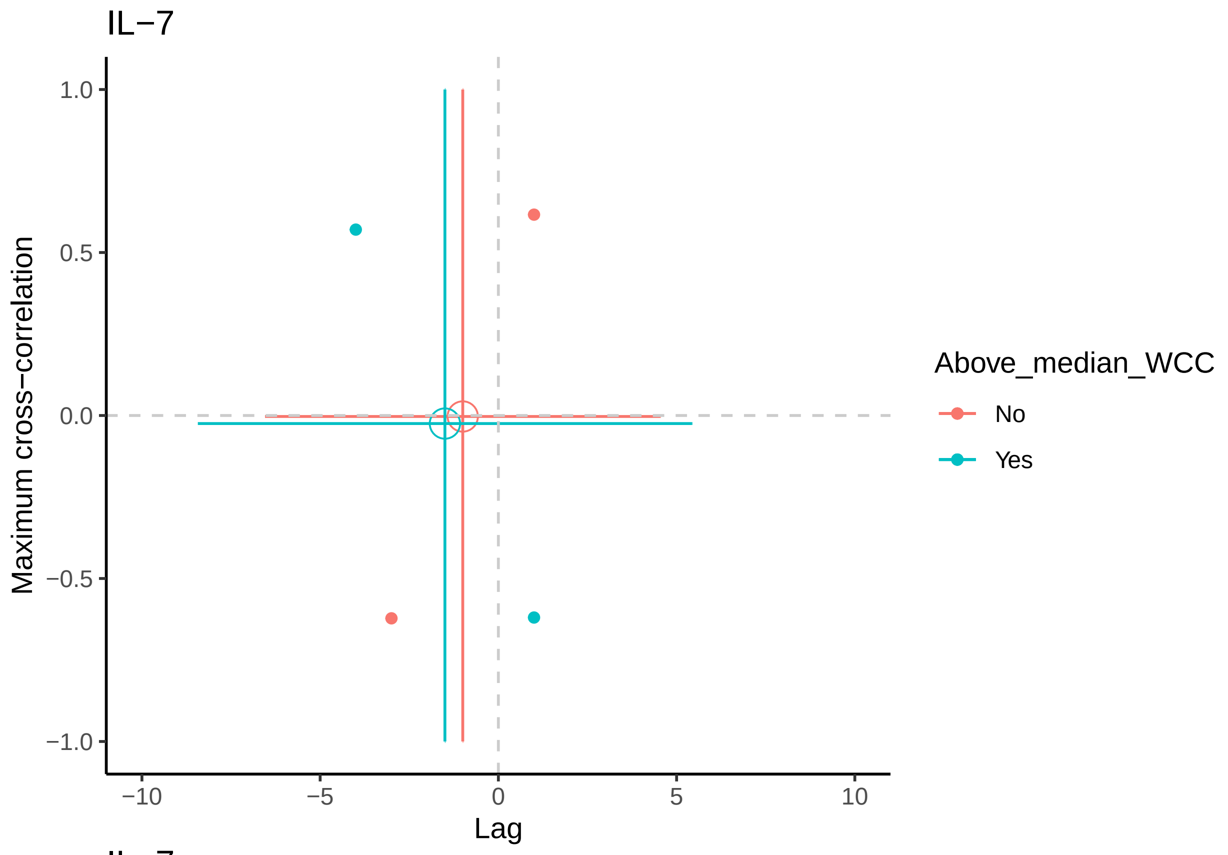
IL−7
Maximum cross−correlation
1.0
0.5
0.0
−0.5
−1.0
−10
−5
0
5
10
Lag
Above_median_WCC
No
Yes

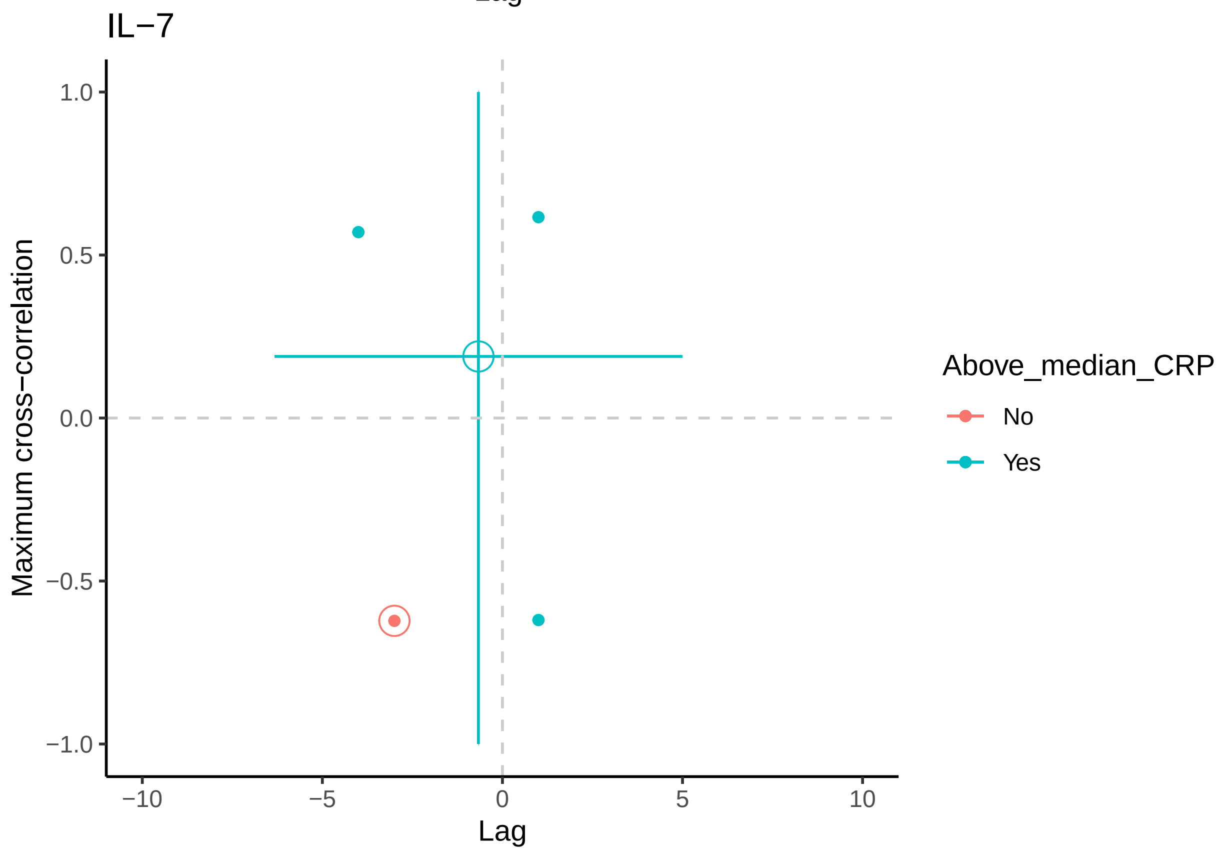
IL−7
Maximum cross−correlation
1.0
0.5
0.0
−0.5
−1.0
−10
−5
0
5
10
Lag
Above_median_CRP
No
Yes

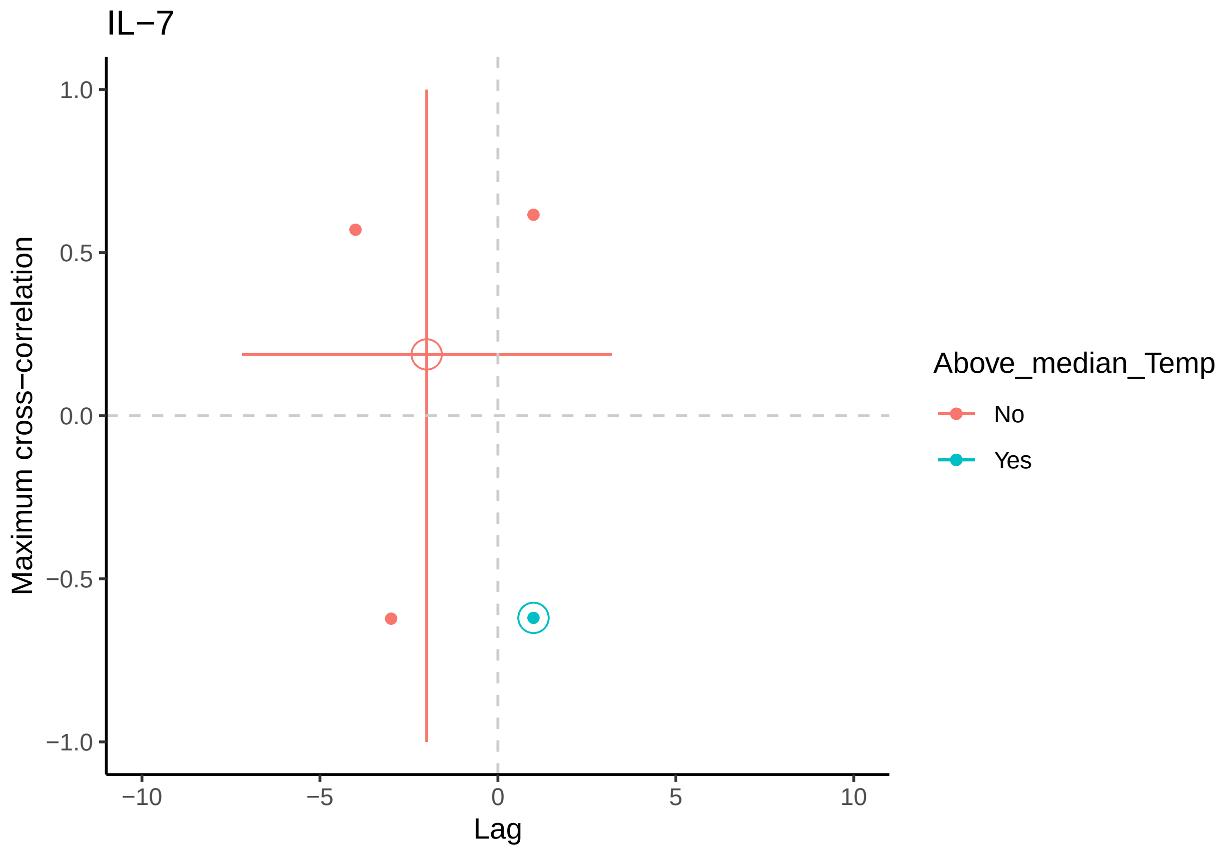
IL−7
Maximum cross−correlation
1.0
0.5
0.0
−0.5
−1.0
−10
−5
0
5
10
Lag
Above_median_Temp
No
Yes

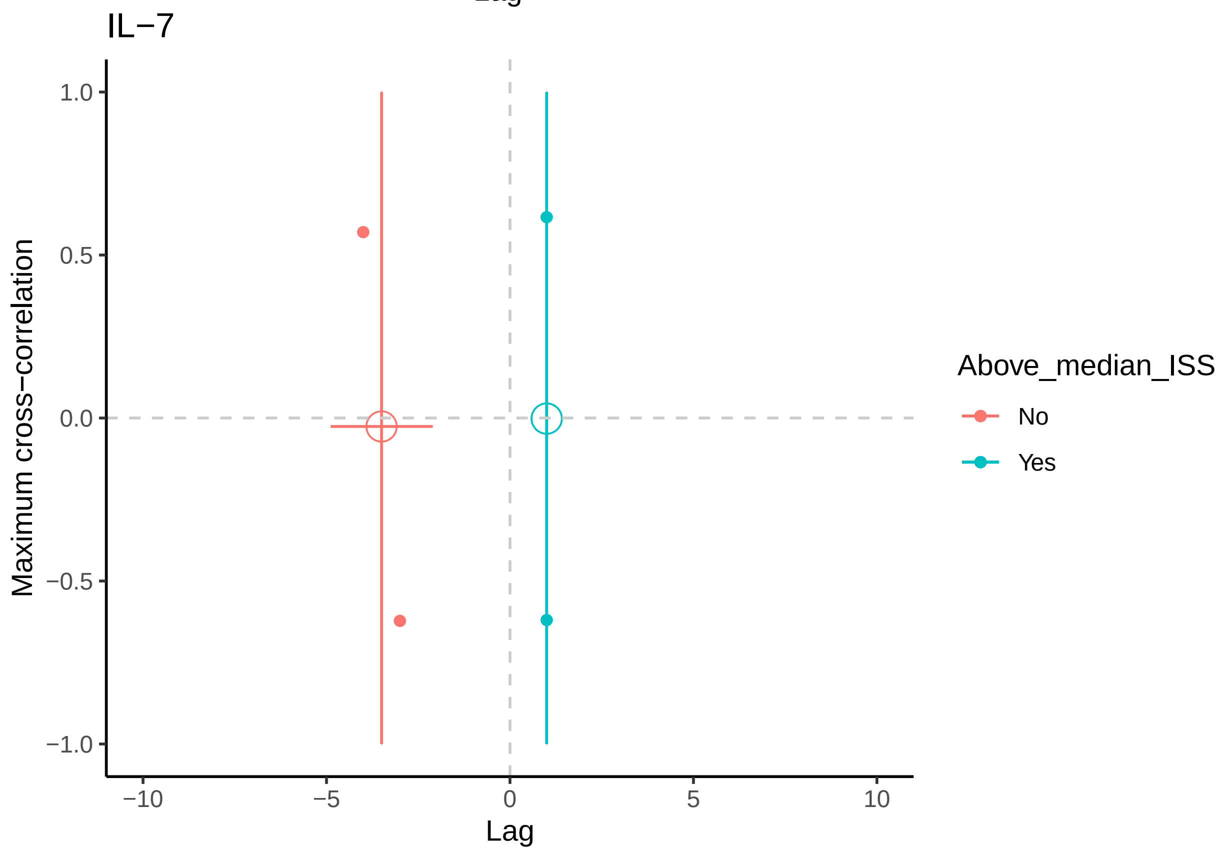
IL−7
Maximum cross−correlation
1.0
0.5
0.0
−0.5
−1.0
−10
−5
0
5
10
Lag
Above_median_ISS
No
Yes

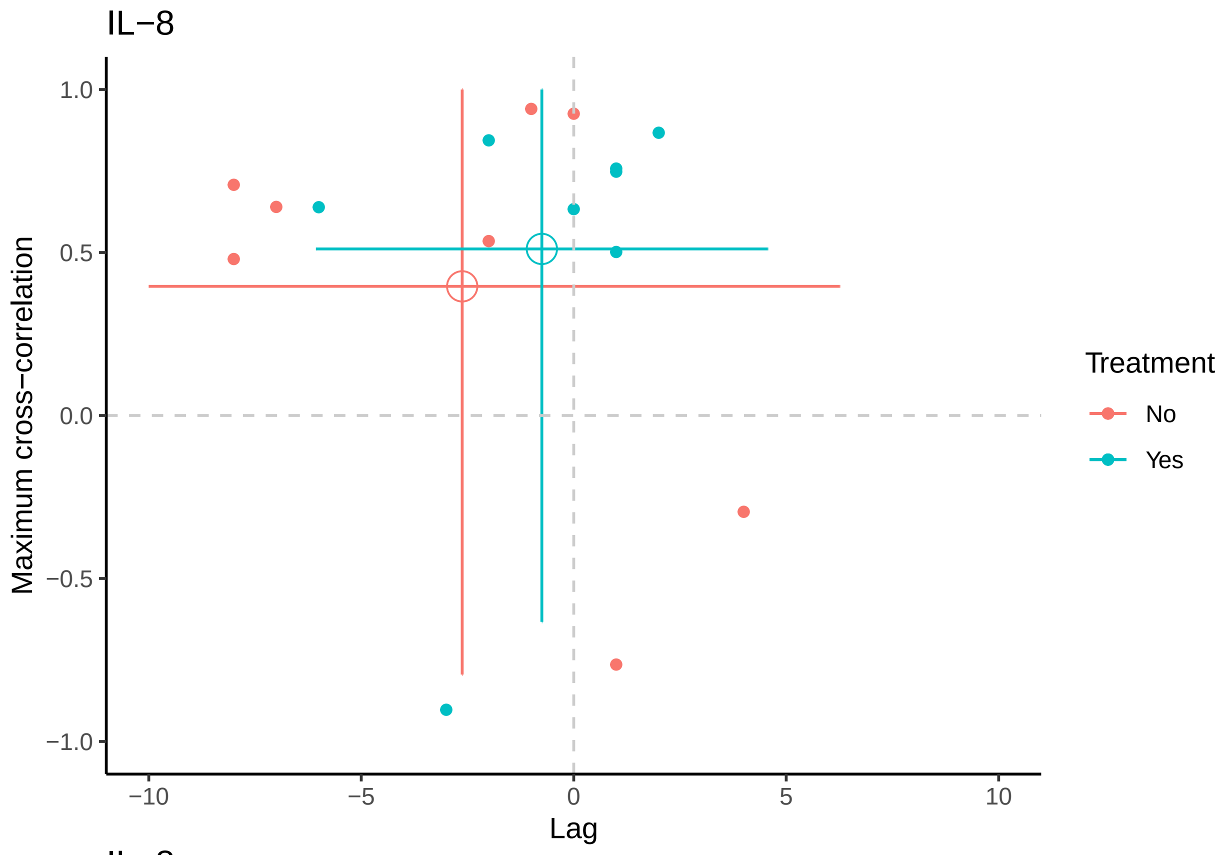
IL−8
Maximum cross−correlation
Lag
Treatment
No
Yes
1.0
0.5
0.0
−0.5
−1.0
−10
−5
0
5
10

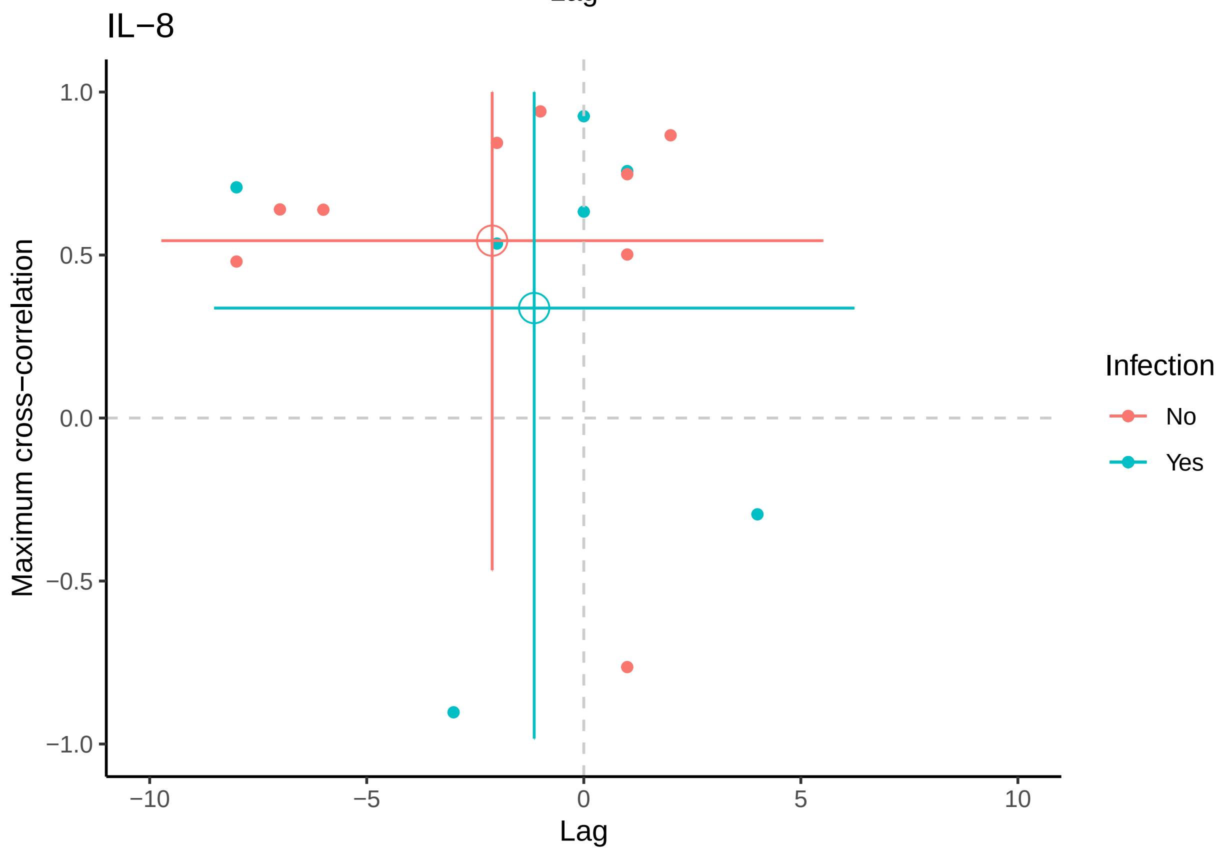
IL−8
Maximum cross−correlation
Lag
Infection
No
Yes
1.0
0.5
0.0
−0.5
−1.0
−10
−5
0
5
10

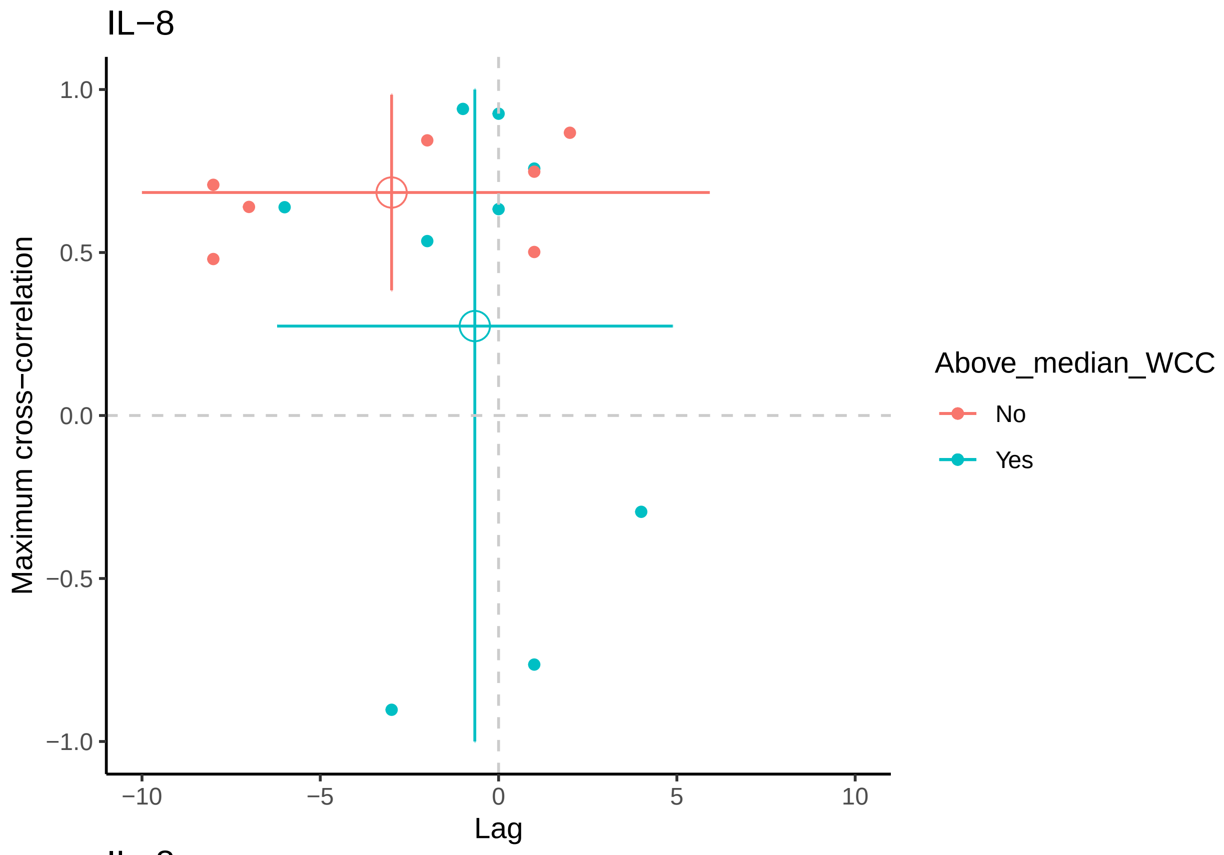
IL−8
Maximum cross−correlation
Lag
Above_median_WCC
No
Yes

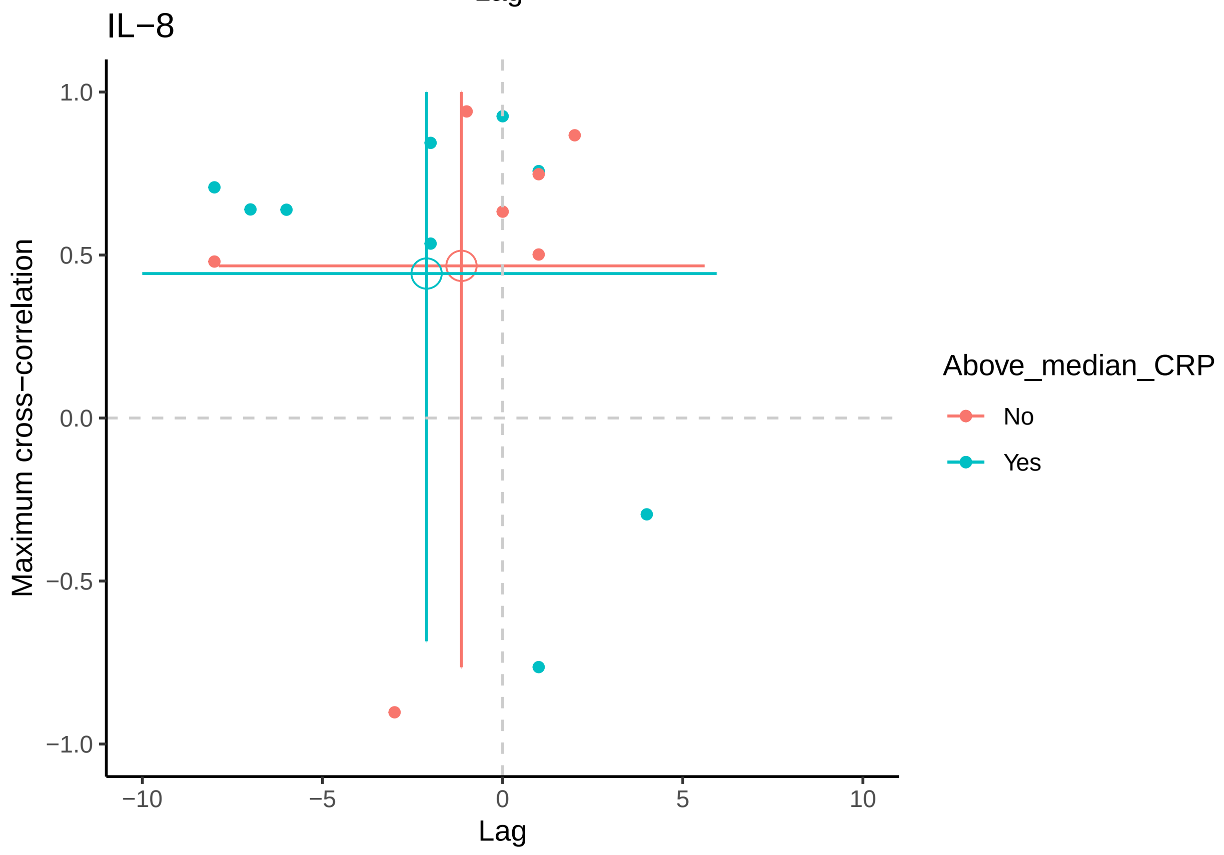
IL−8
Maximum cross−correlation
Lag
Above_median_CRP
No
Yes

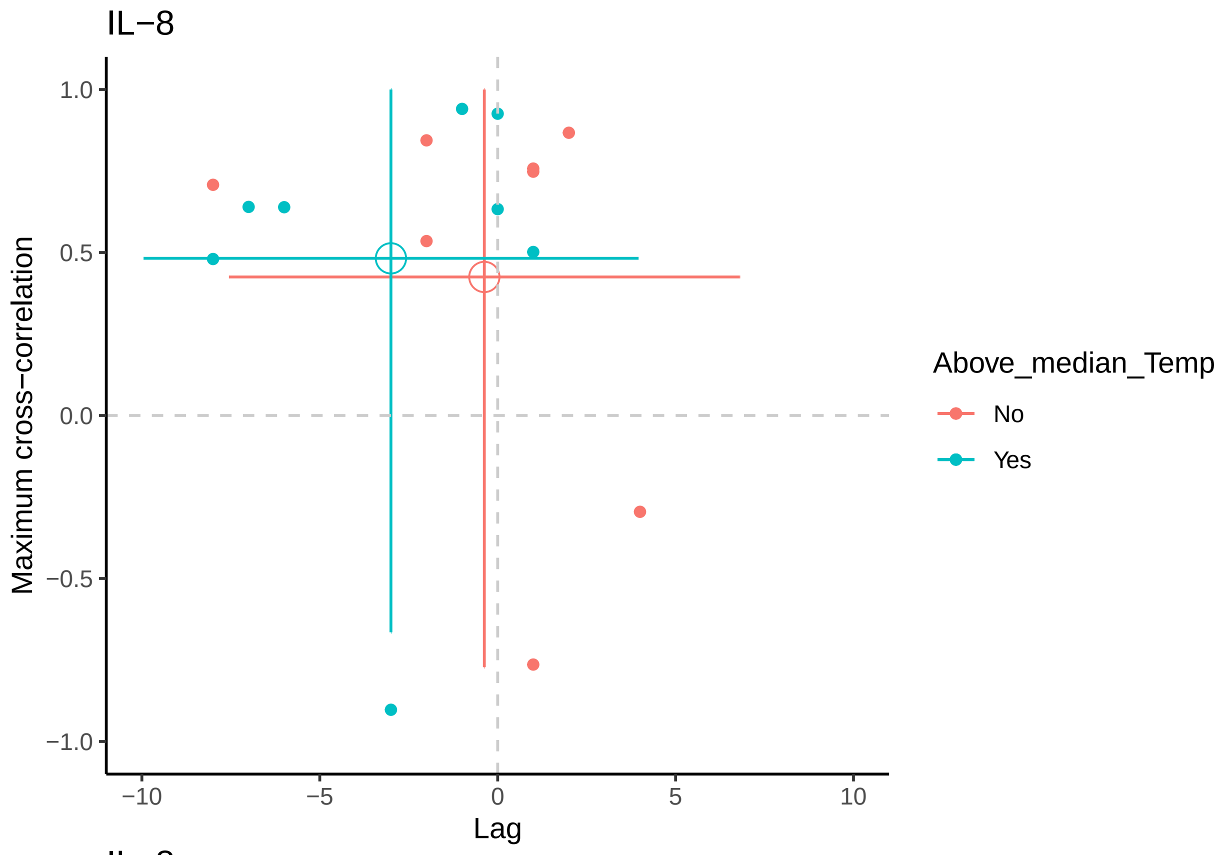
IL−8
Maximum cross−correlation
1.0
0.5
0.0
−0.5
−1.0
−10
−5
0
5
10
Lag
Above_median_Temp
No
Yes

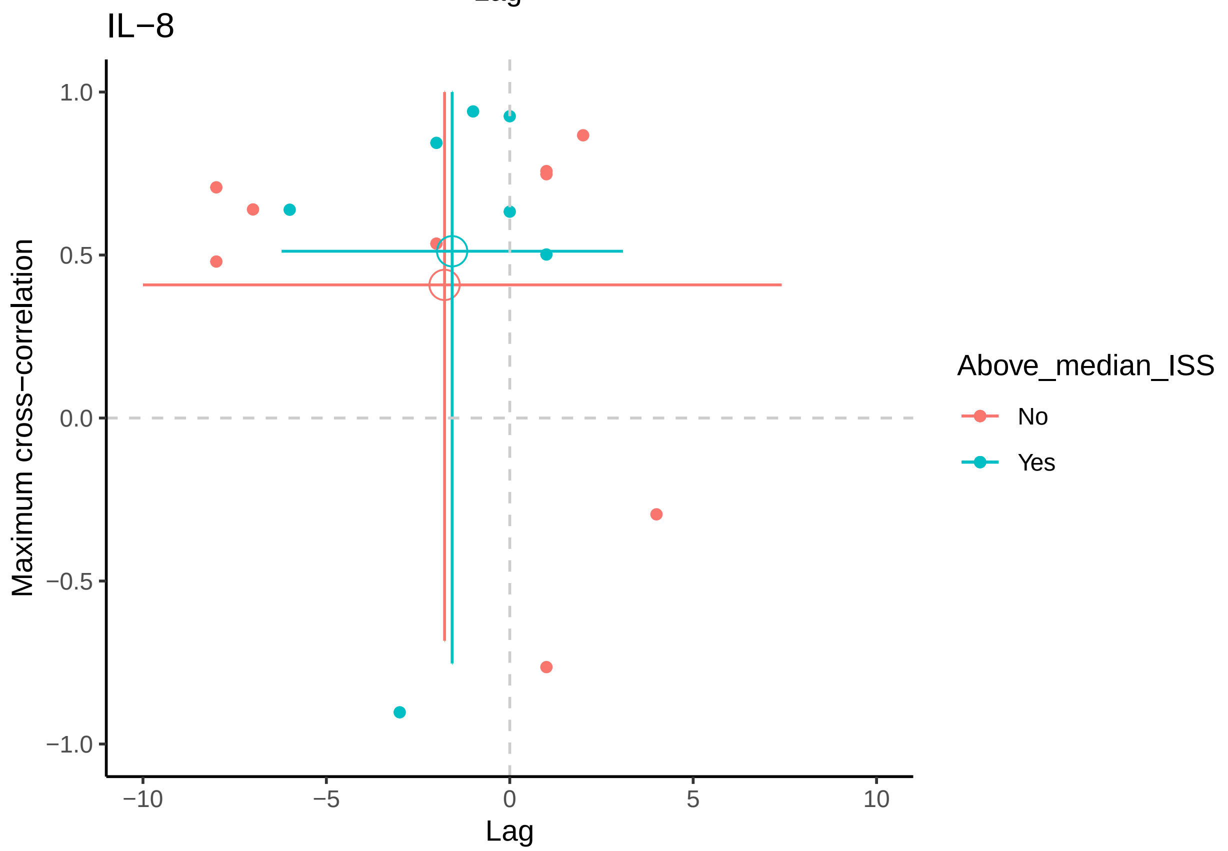
IL−8
Maximum cross−correlation
1.0
0.5
0.0
−0.5
−1.0
−10
−5
0
5
10
Lag
Above_median_ISS
No
Yes

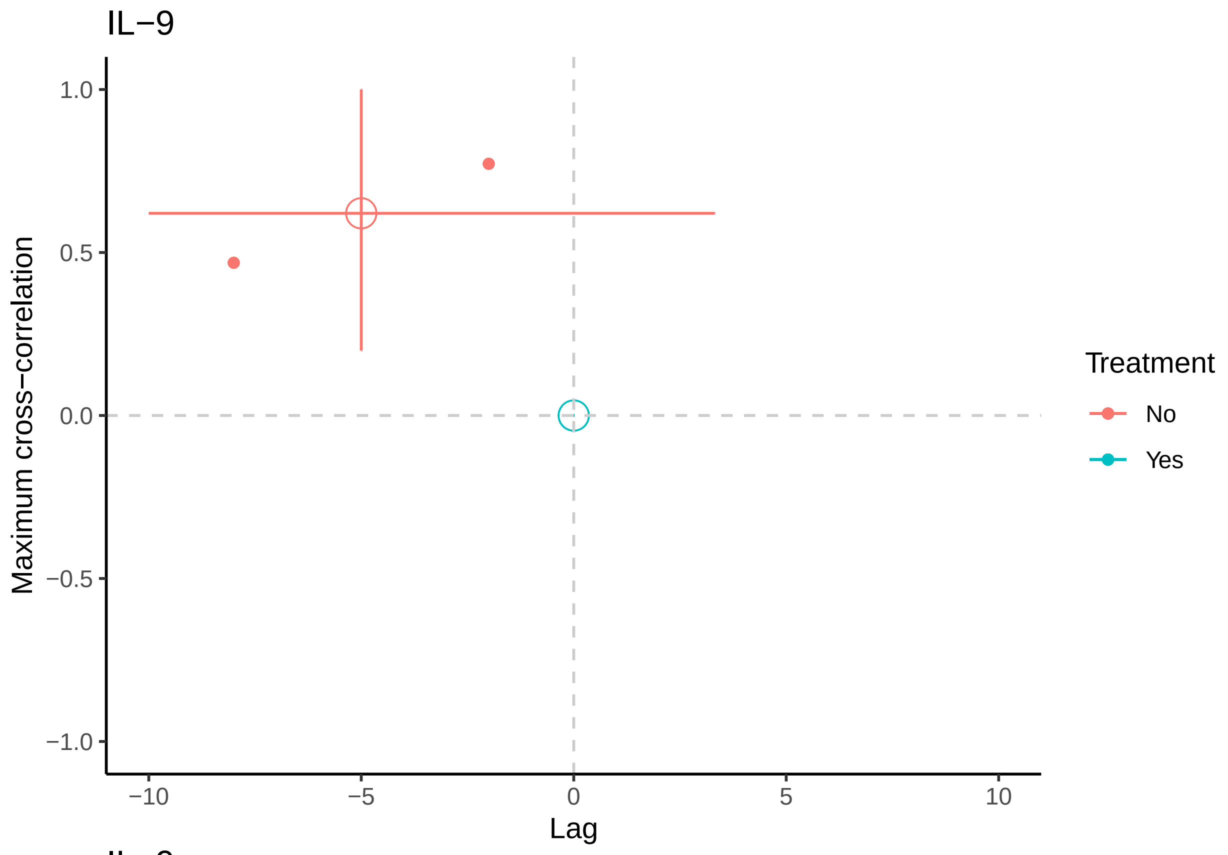
IL−9
Maximum cross−correlation
1.0
0.5
0.0
−0.5
−1.0
−10
−5
0
5
10
Lag
Treatment
No
Yes

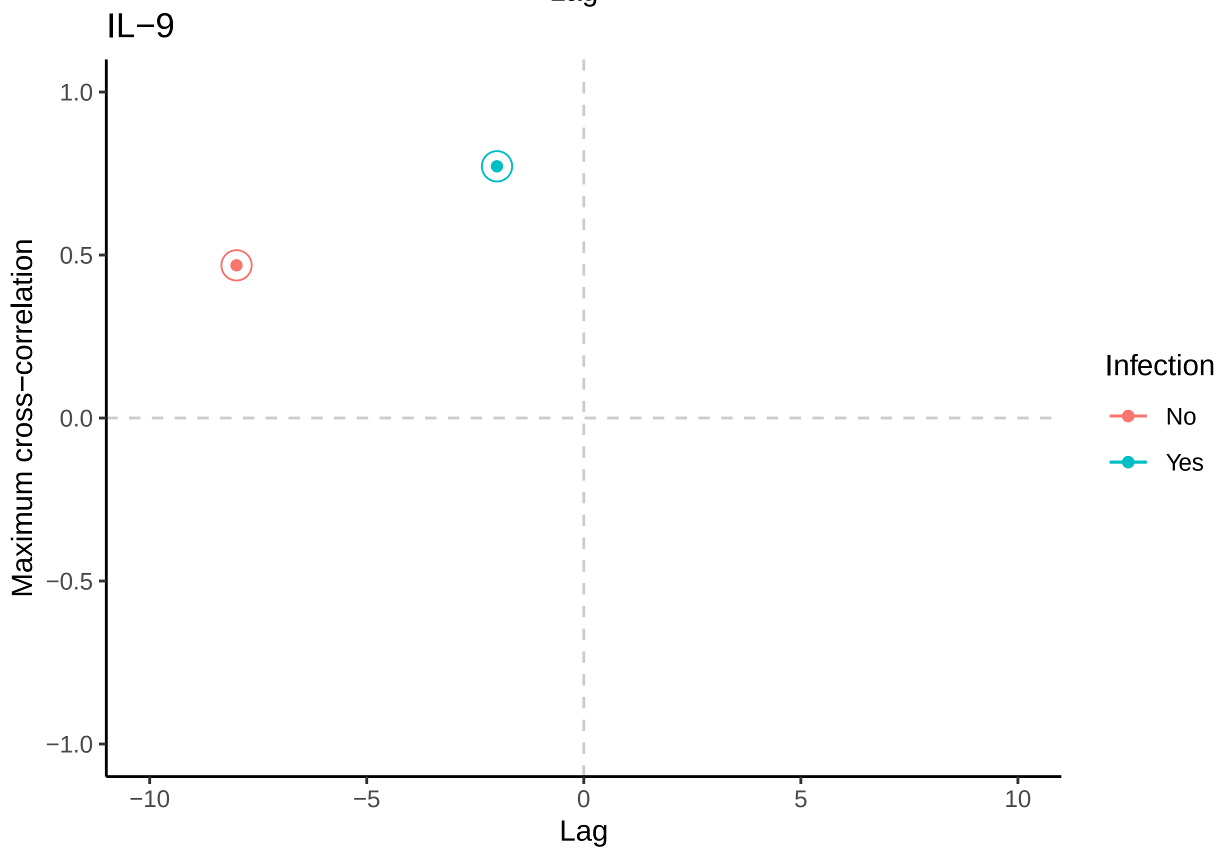
IL−9
Maximum cross−correlation
1.0
0.5
0.0
−0.5
−1.0
−10
−5
0
5
10
Lag
Infection
No
Yes

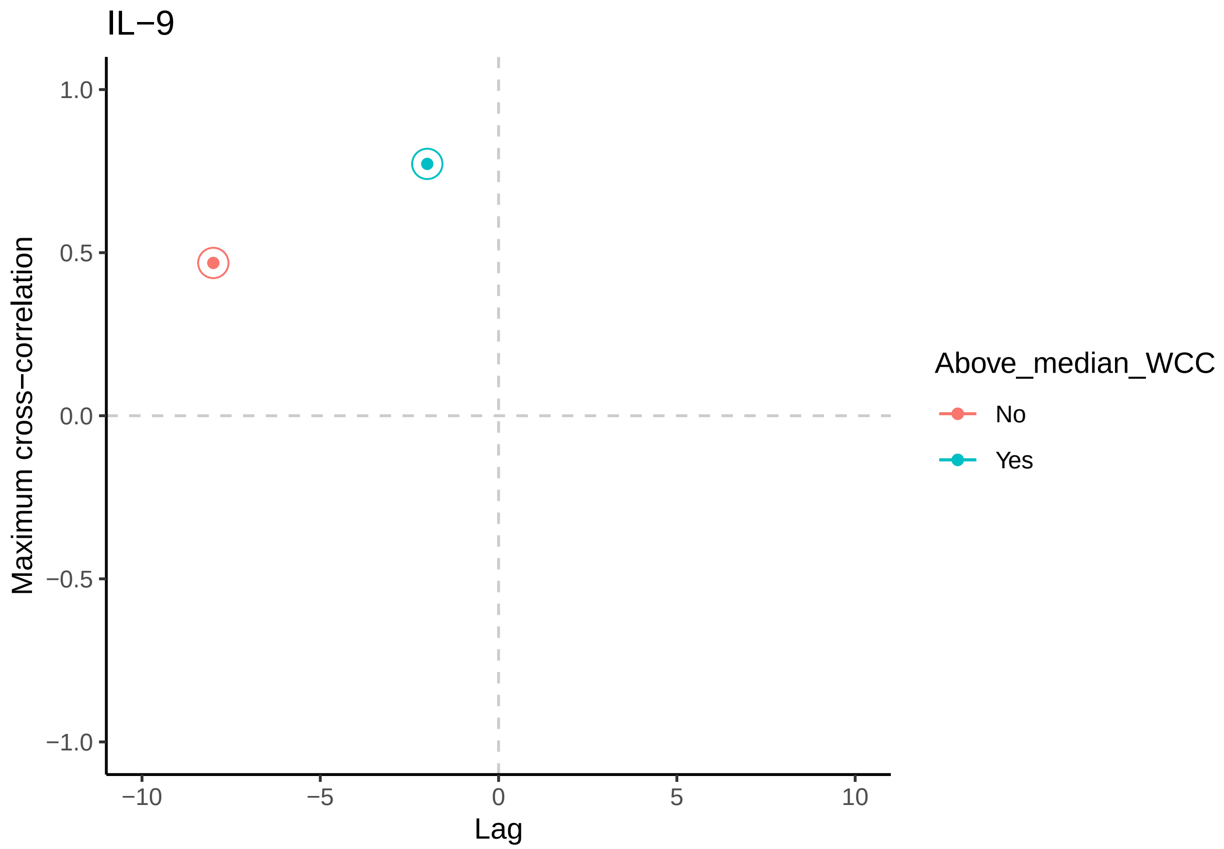
IL−9
Maximum cross−correlation
1.0
0.5
0.0
−0.5
−1.0
−10
−5
0
5
10
Lag
Above_median_WCC
No
Yes

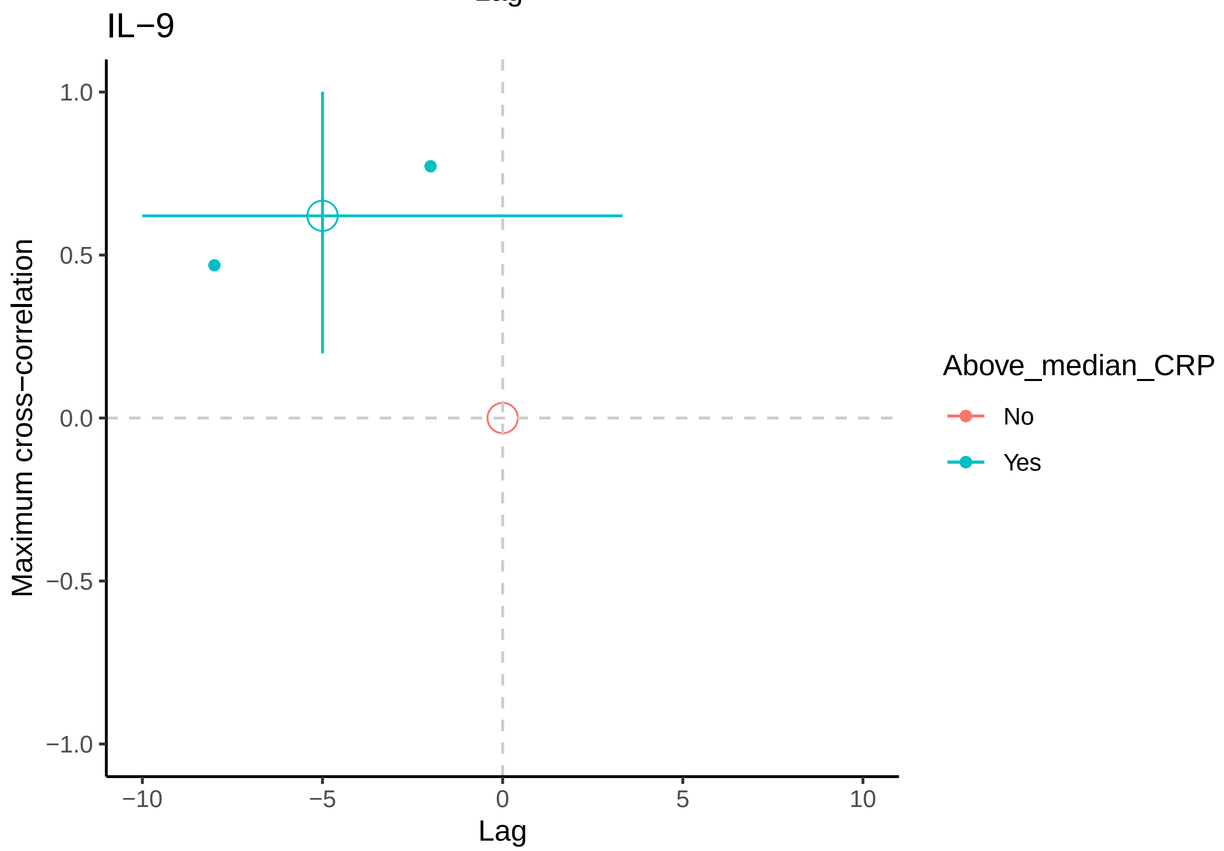
IL−9
Maximum cross−correlation
1.0
0.5
0.0
−0.5
−1.0
−10
−5
0
5
10
Lag
Above_median_CRP
No
Yes

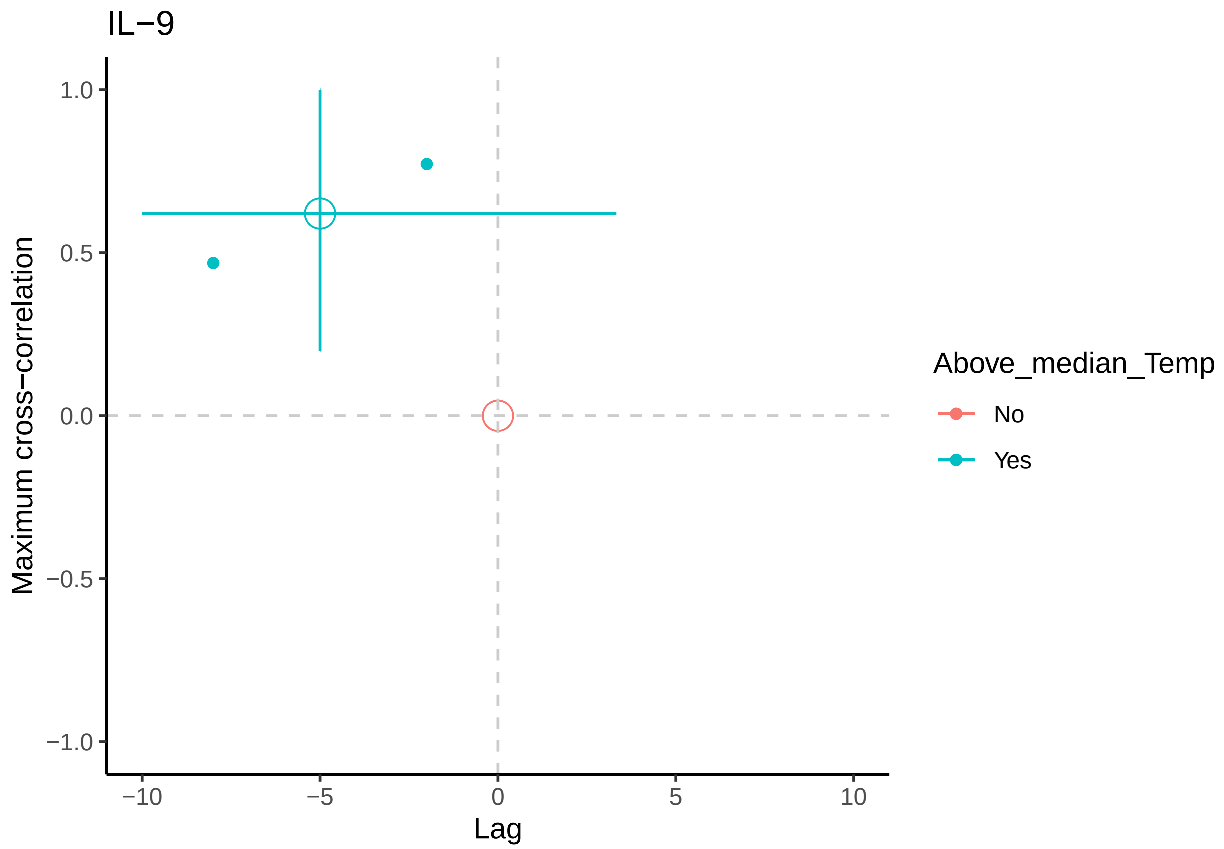
IL−9
Maximum cross−correlation
1.0
0.5
0.0
−0.5
−1.0
−10
−5
0
5
10
Lag
Above_median_Temp
No
Yes

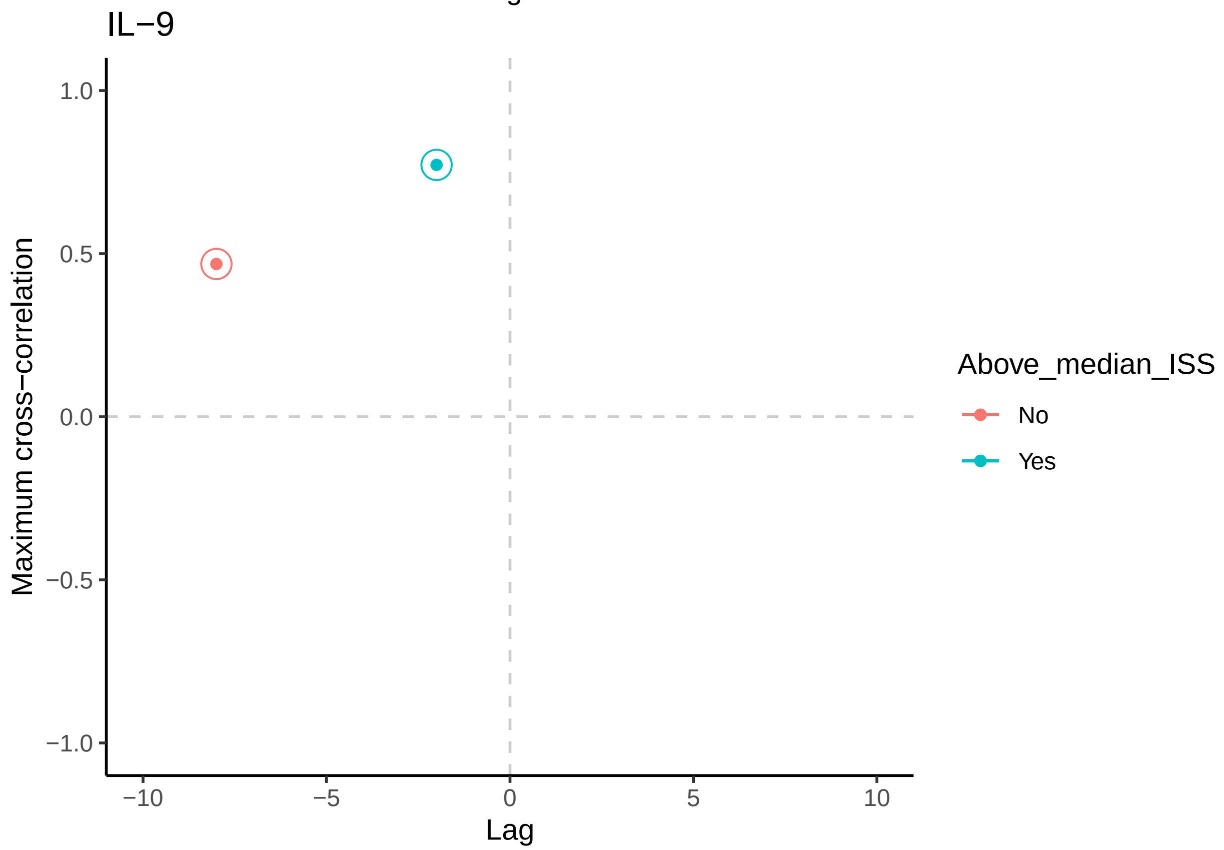
IL−9
Maximum cross−correlation
1.0
0.5
0.0
−0.5
−1.0
−10
−5
0
5
10
Lag
Above_median_ISS
No
Yes

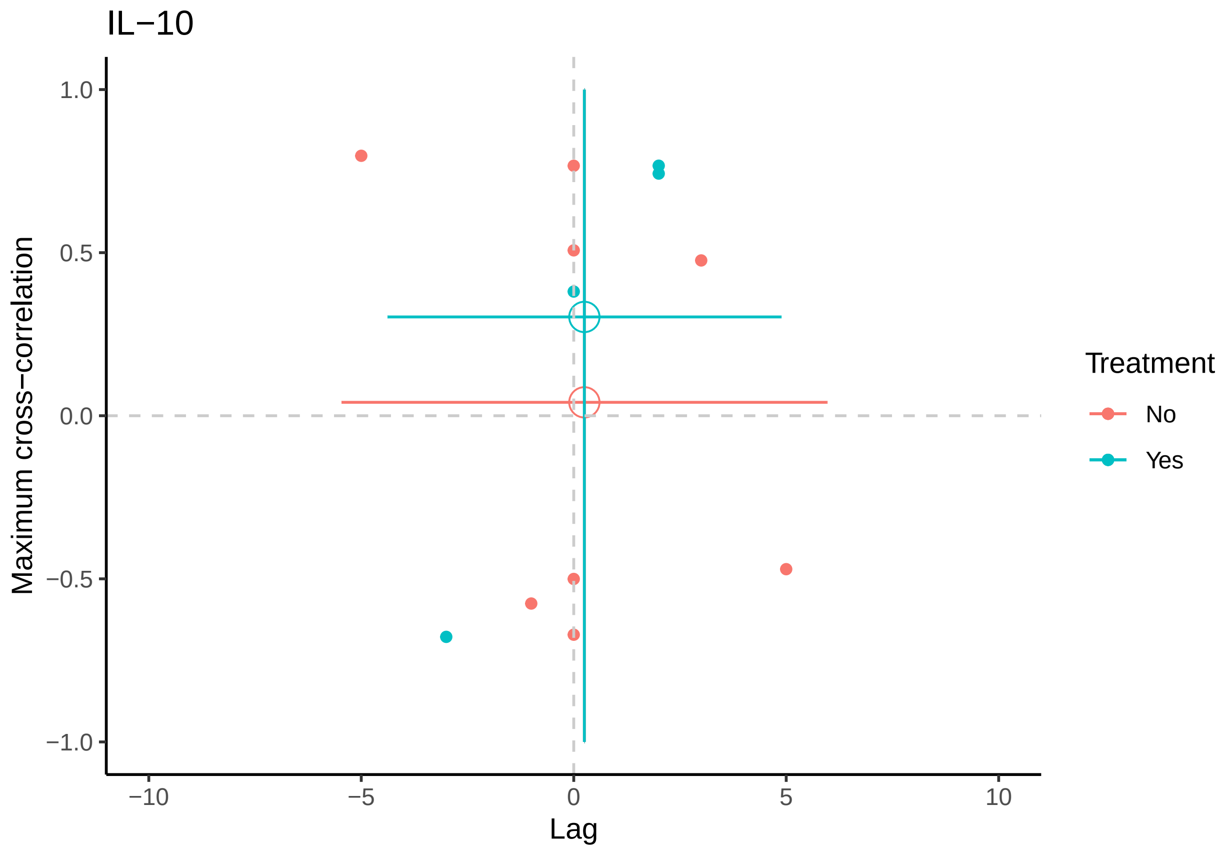
IL−10
Maximum cross−correlation
Lag
Treatment
No
Yes
1.0
0.5
0.0
−0.5
−1.0
−10
−5
0
5
10

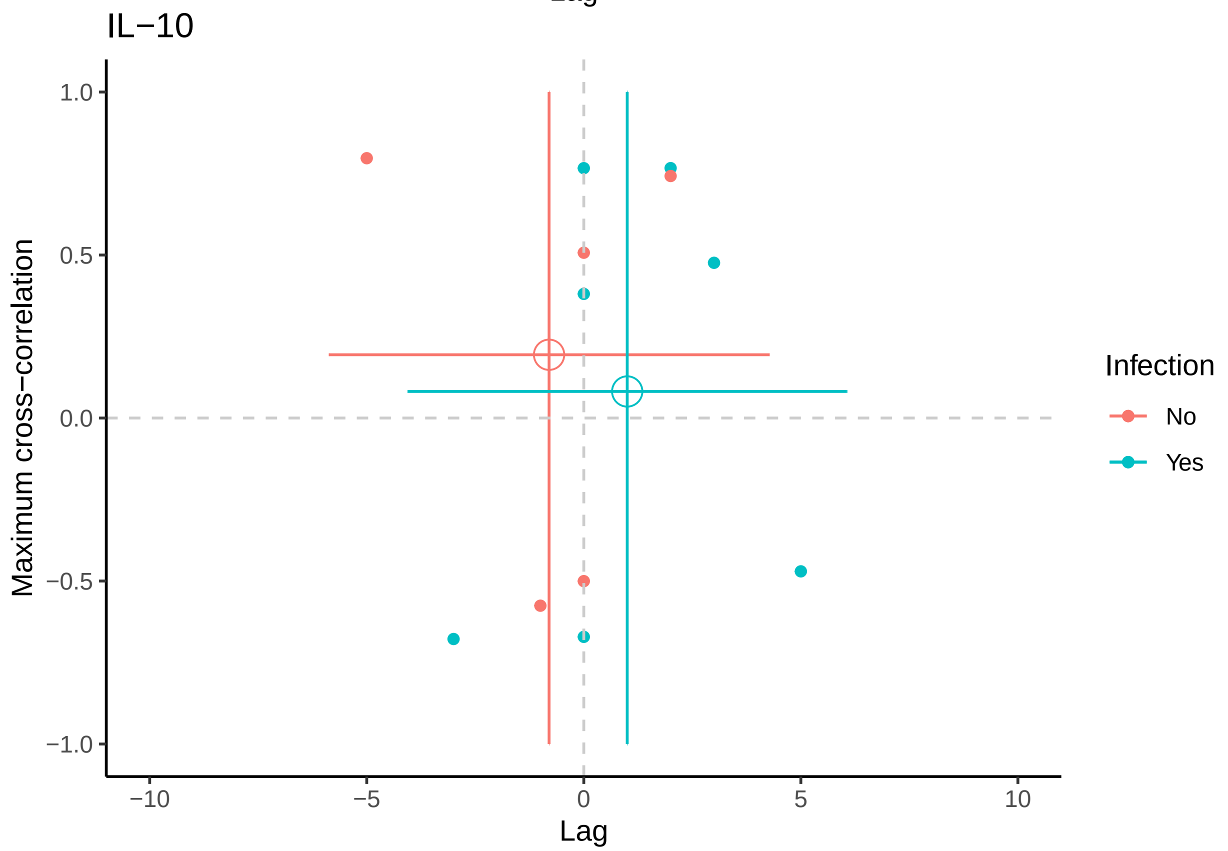
IL−10
Maximum cross−correlation
Lag
Infection
No
Yes
1.0
0.5
0.0
−0.5
−1.0
−10
−5
0
5
10

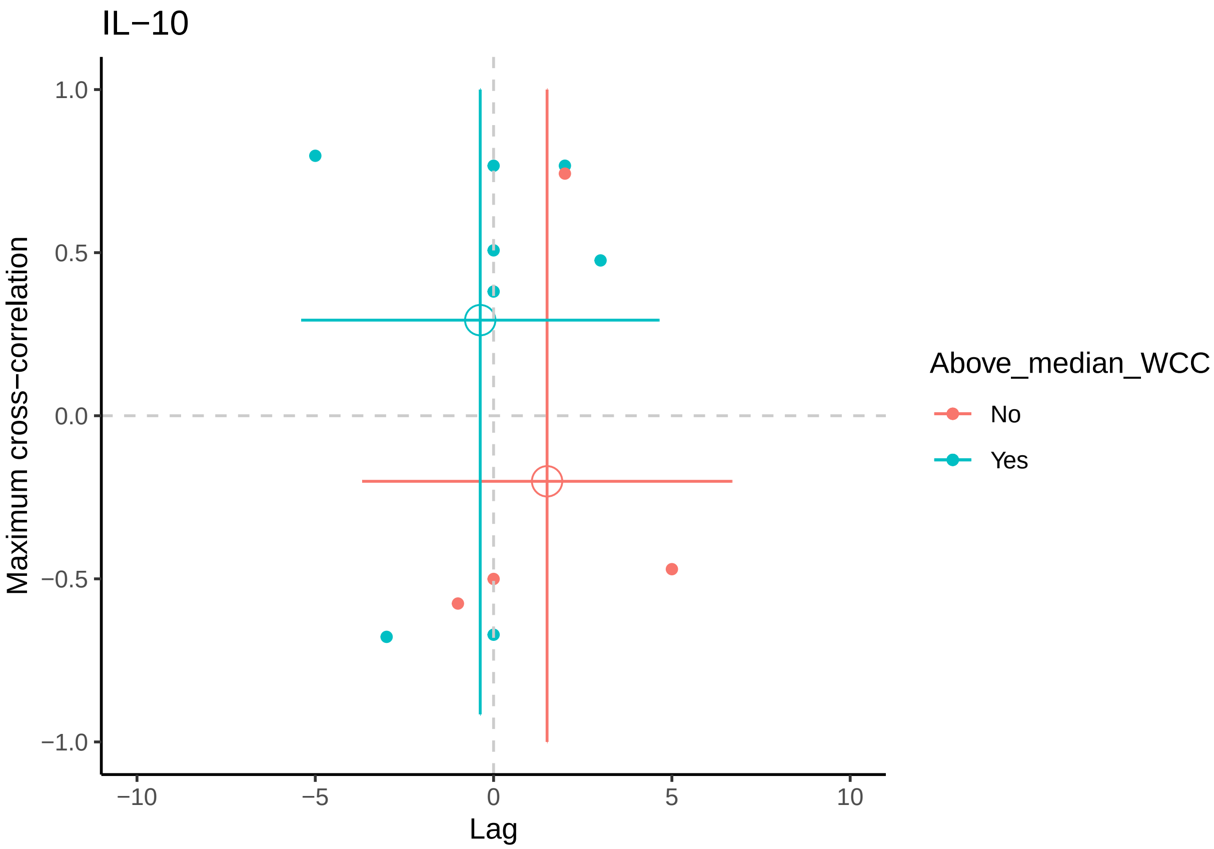
IL−10
Maximum cross−correlation
Lag
Above_median_WCC
No
Yes

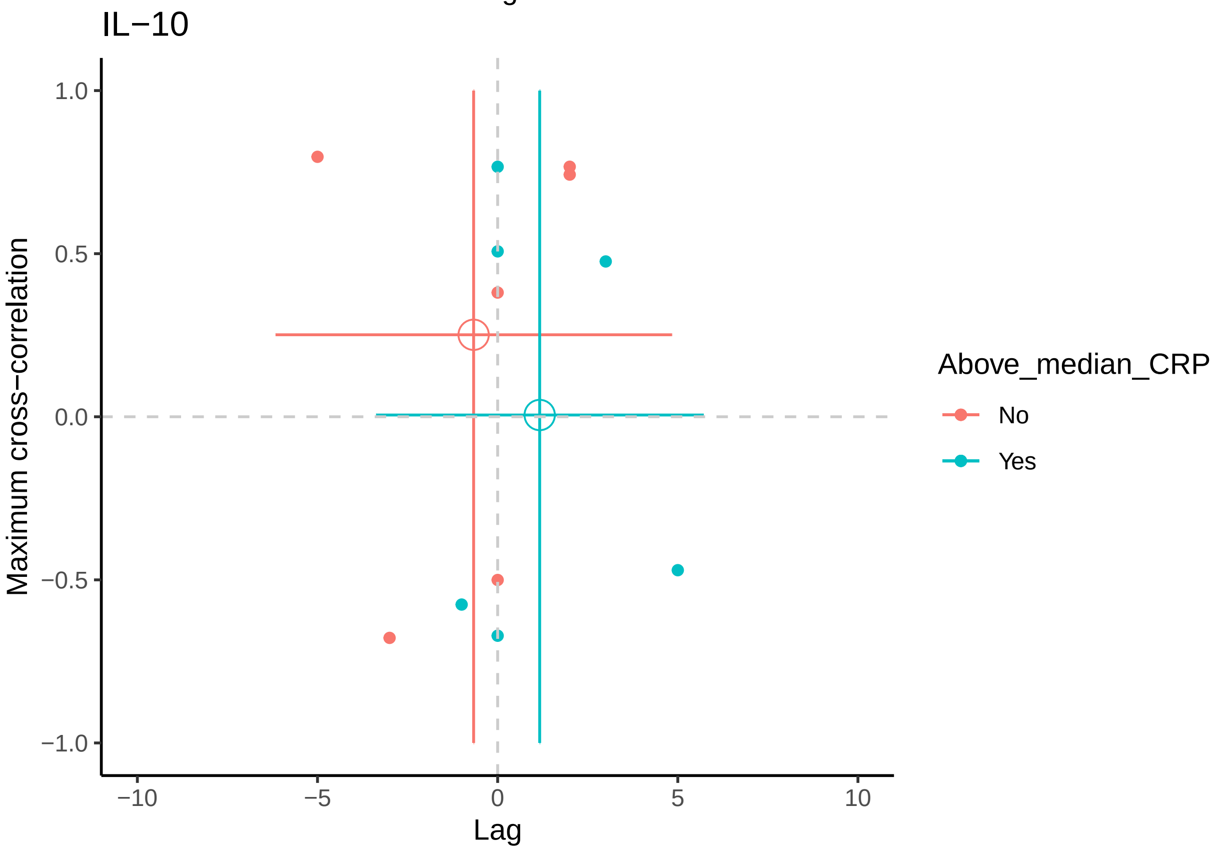
IL−10
Maximum cross−correlation
Lag
Above_median_CRP
No
Yes

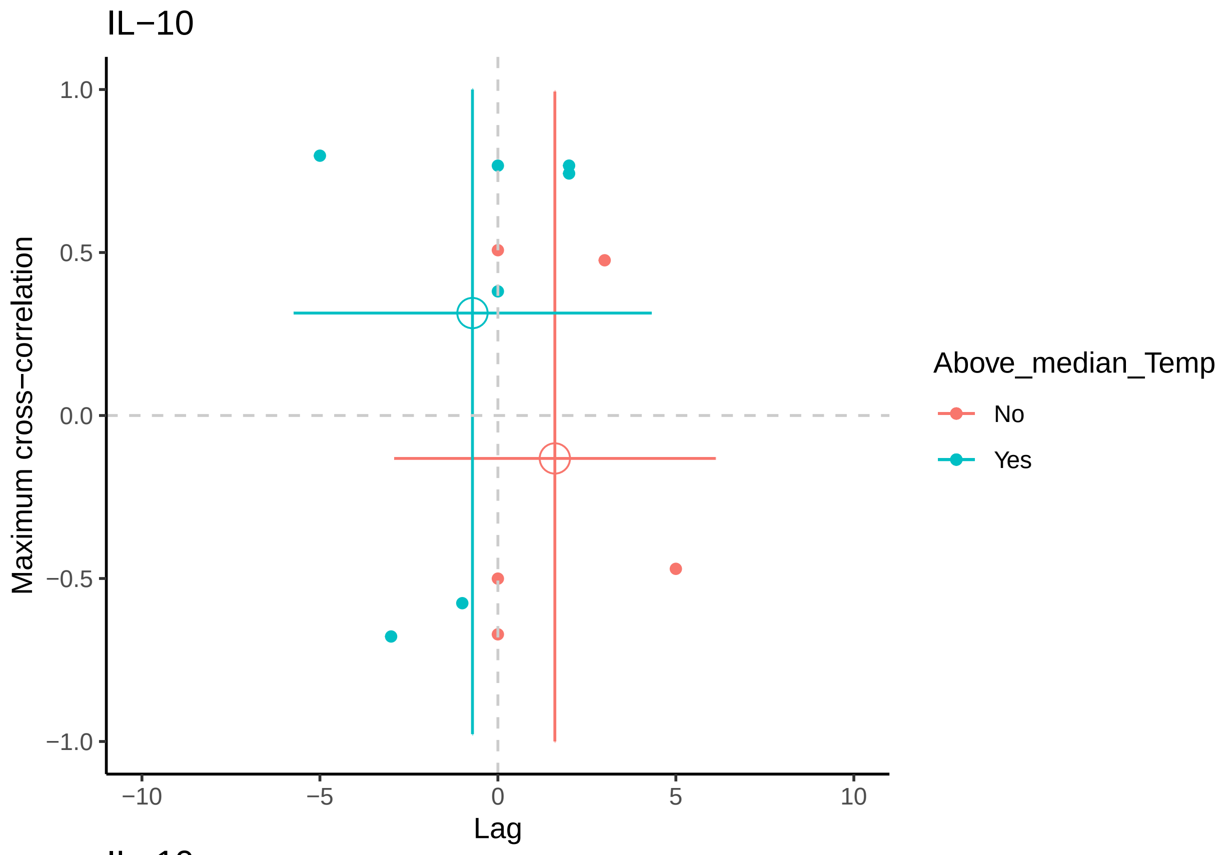
IL−10
Maximum cross−correlation
1.0
0.5
0.0
−0.5
−1.0
−10
−5
0
5
10
Lag
Above_median_Temp
No
Yes

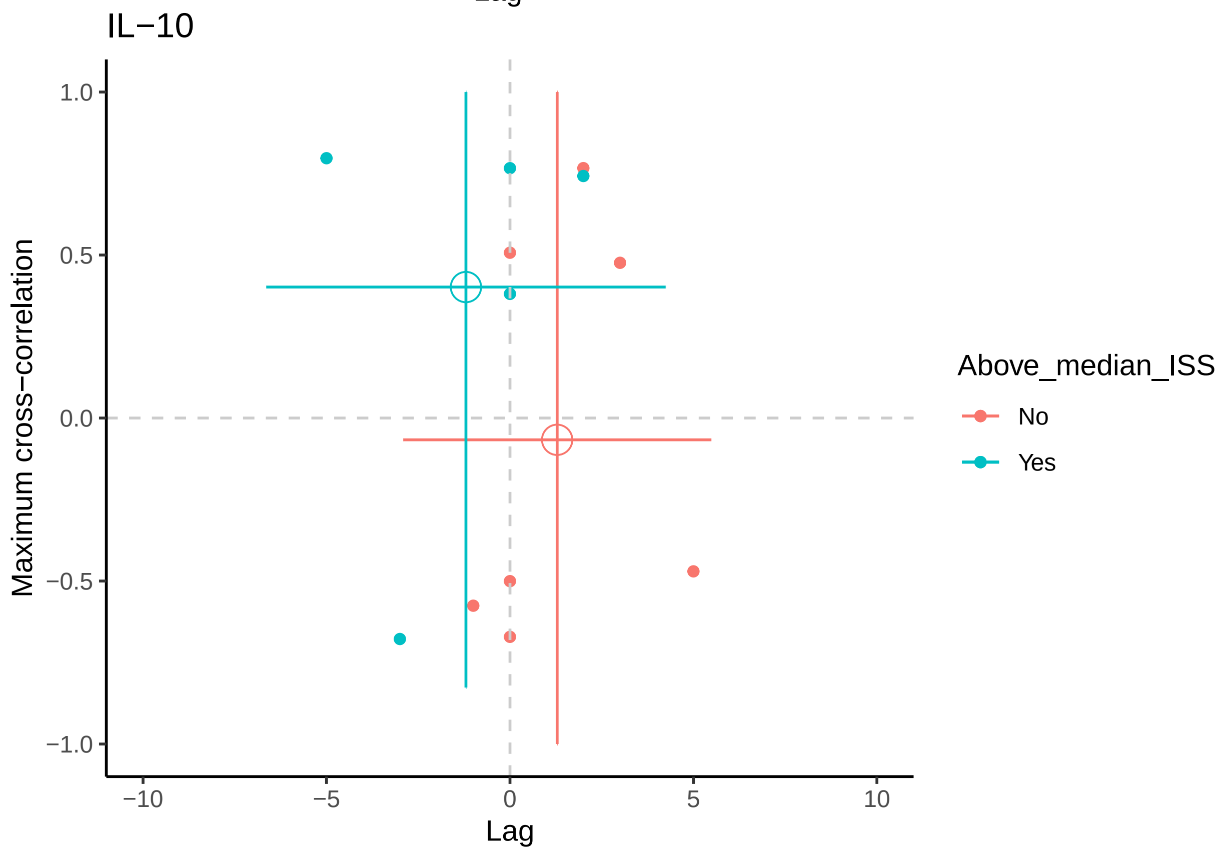
IL−10
Maximum cross−correlation
1.0
0.5
0.0
−0.5
−1.0
−10
−5
0
5
10
Lag
Above_median_ISS
No
Yes

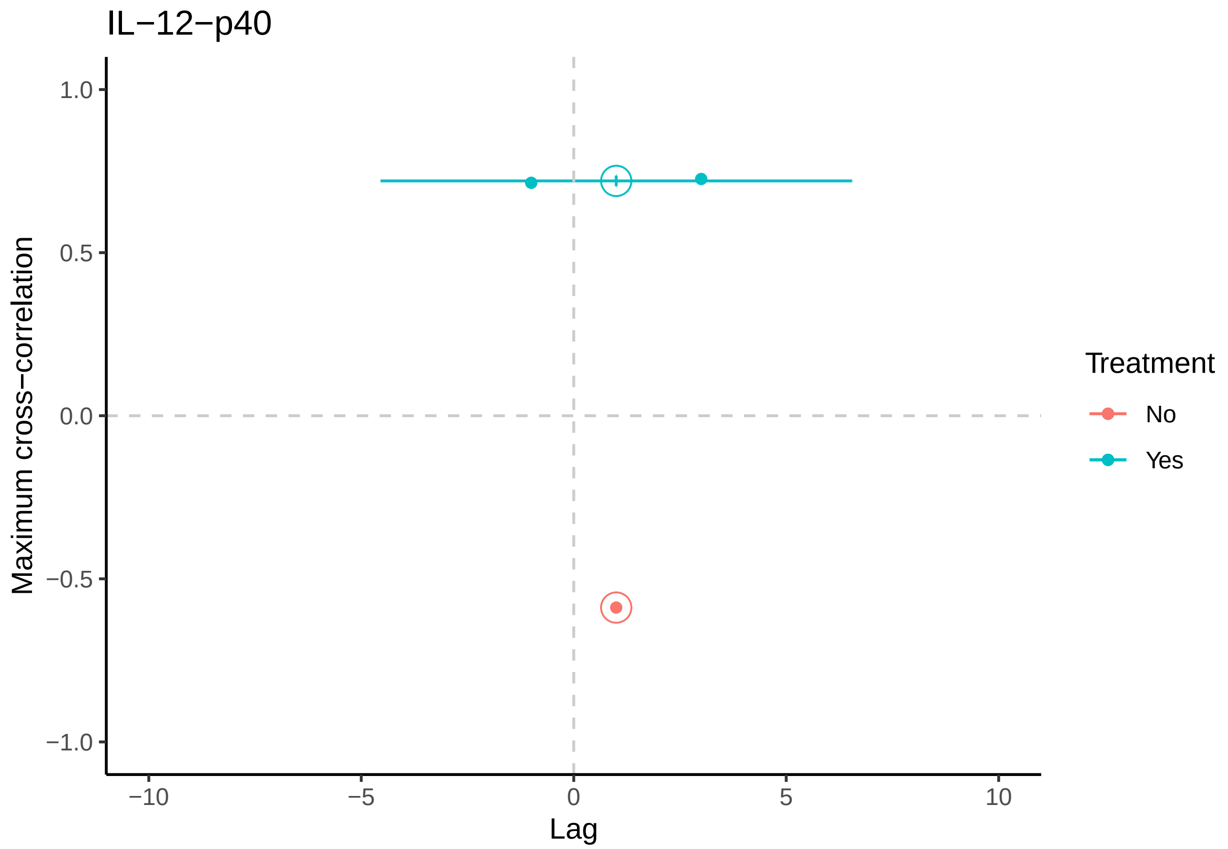
IL−12−p40
Maximum cross−correlation
1.0
0.5
0.0
−0.5
−1.0
−10
−5
0
5
10
Lag
Treatment
No
Yes

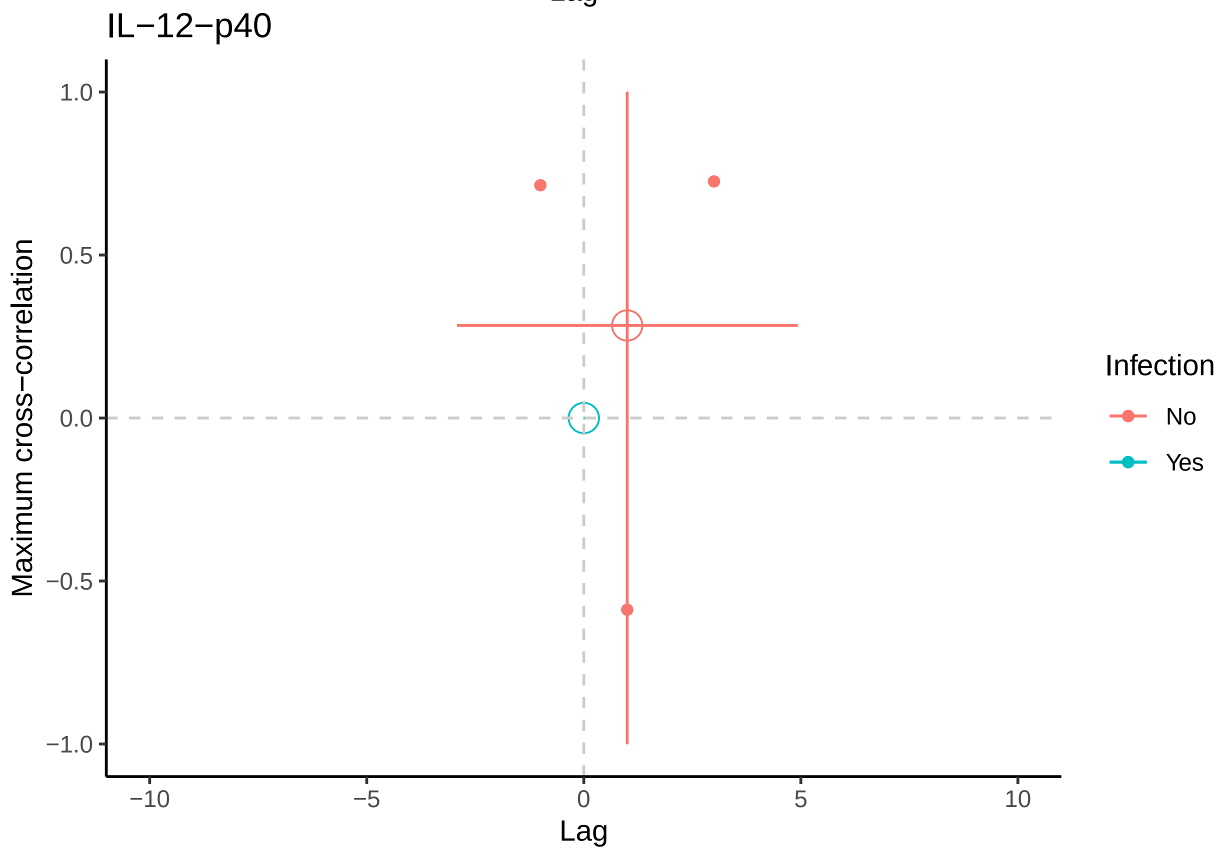
IL−12−p40
Maximum cross−correlation
1.0
0.5
0.0
−0.5
−1.0
−10
−5
0
5
10
Lag
Infection
No
Yes

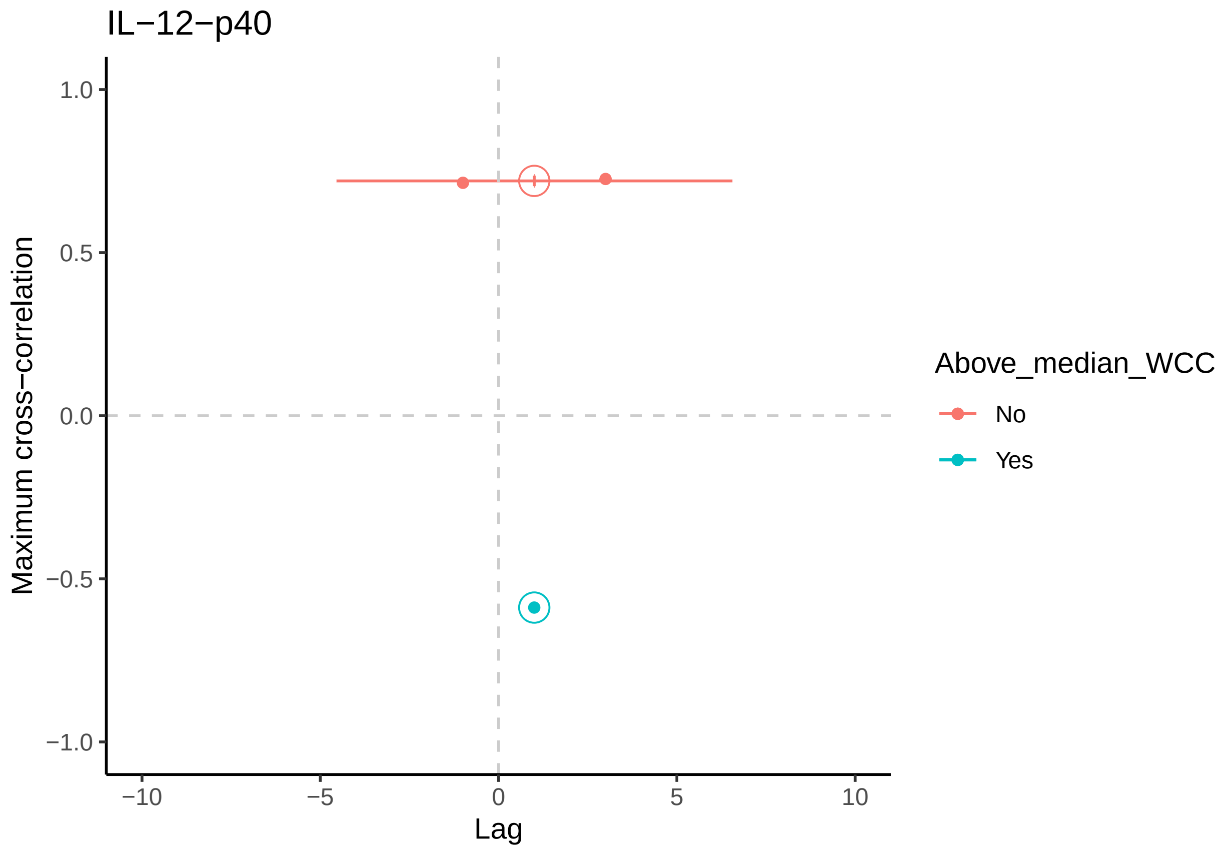
IL−12−p40
Maximum cross−correlation
1.0
0.5
0.0
−0.5
−1.0
−10
−5
0
5
10
Lag
Above_median_WCC
No
Yes

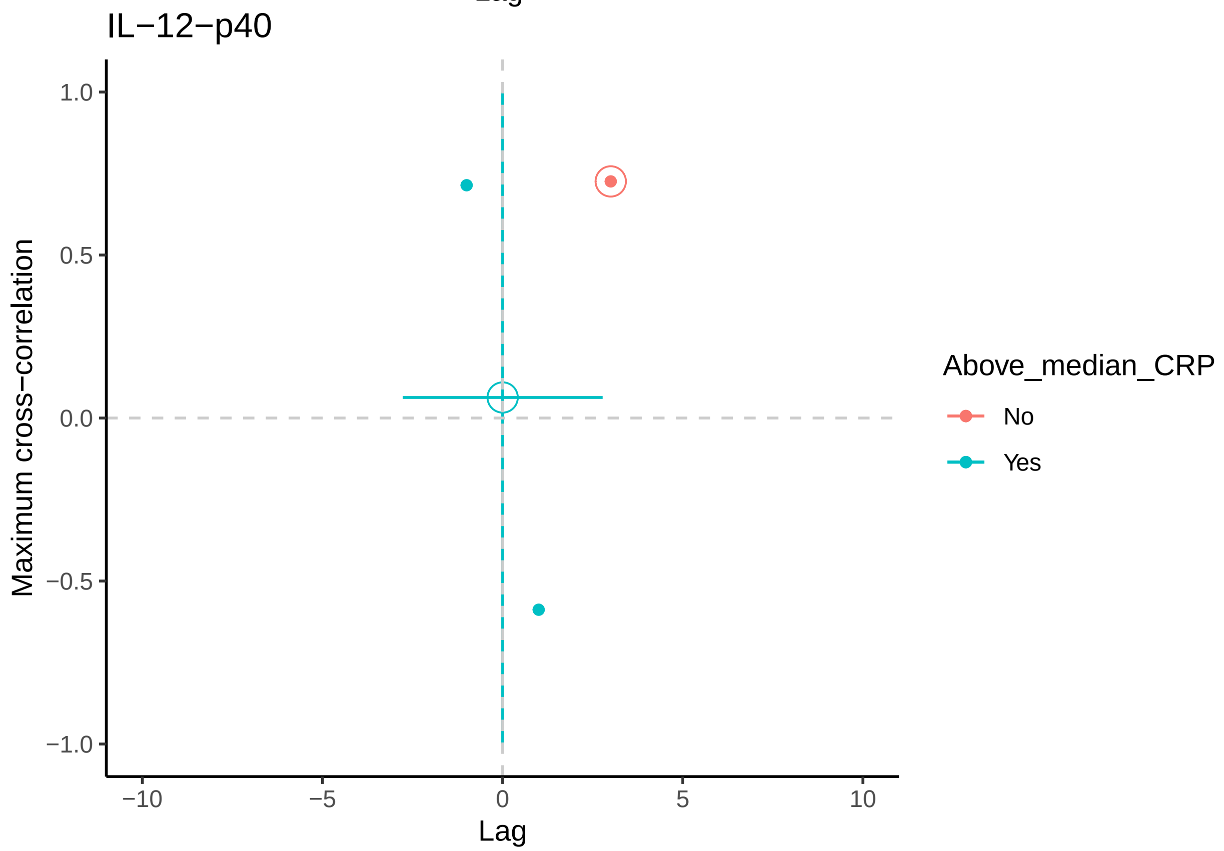
IL−12−p40
Maximum cross−correlation
1.0
0.5
0.0
−0.5
−1.0
−10
−5
0
5
10
Lag
Above_median_CRP
No
Yes

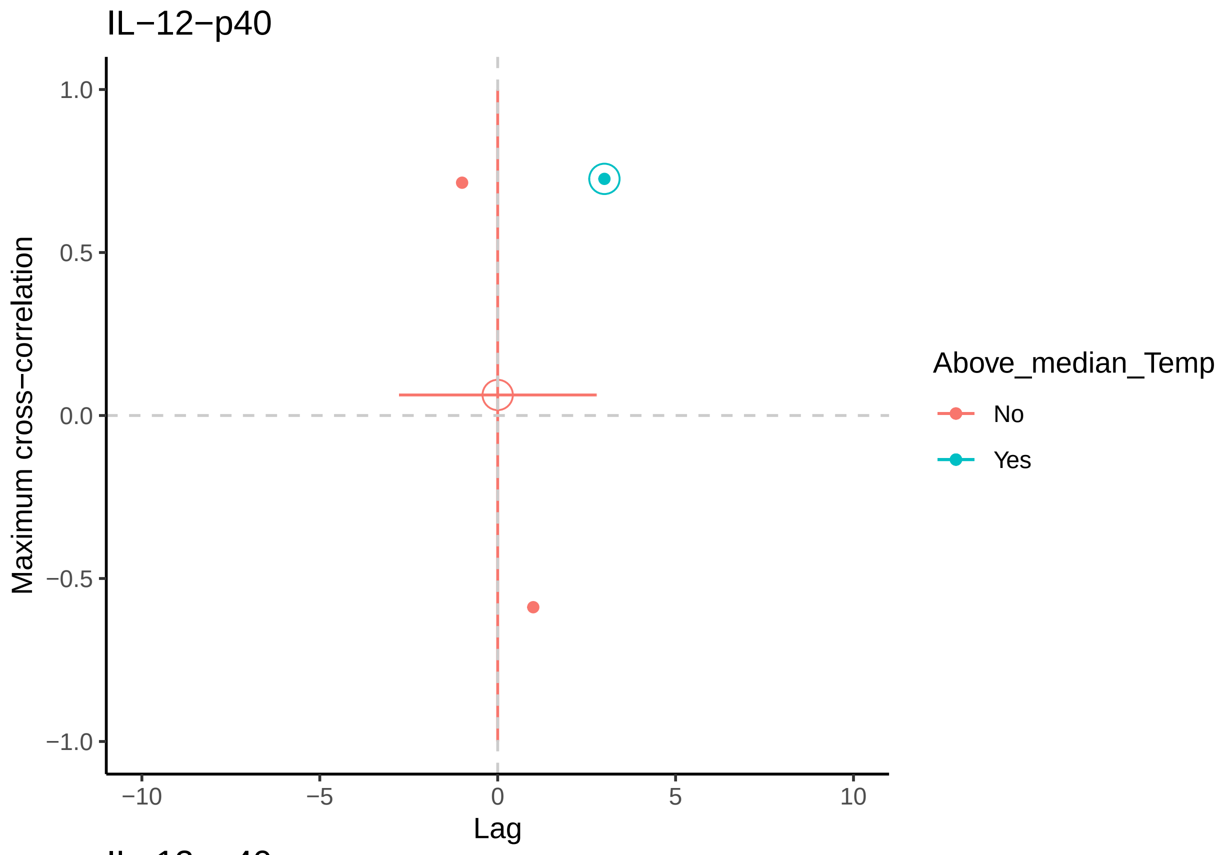
IL−12−p40
Maximum cross−correlation
1.0
0.5
0.0
−0.5
−1.0
−10
−5
0
5
10
Lag
Above_median_Temp
No
Yes

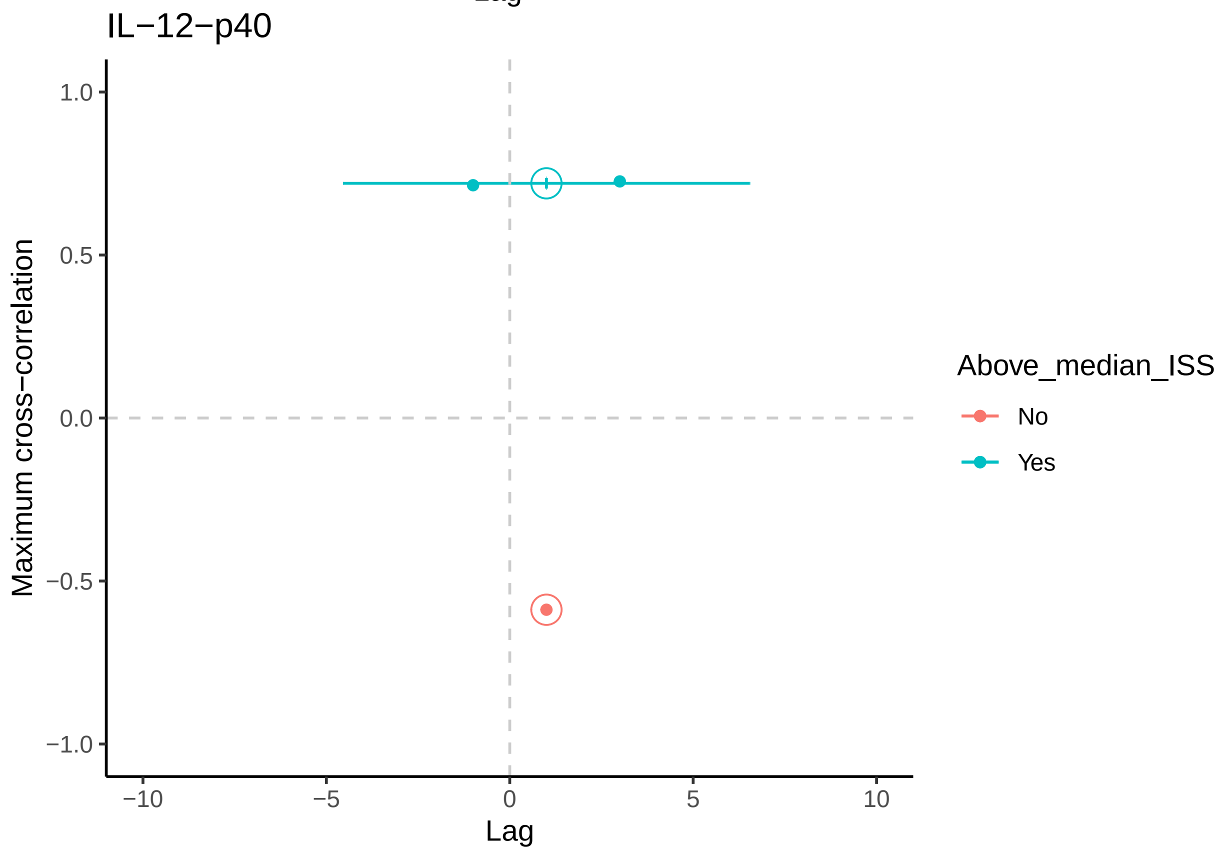
IL−12−p40
Maximum cross−correlation
1.0
0.5
0.0
−0.5
−1.0
−10
−5
0
5
10
Lag
Above_median_ISS
No
Yes

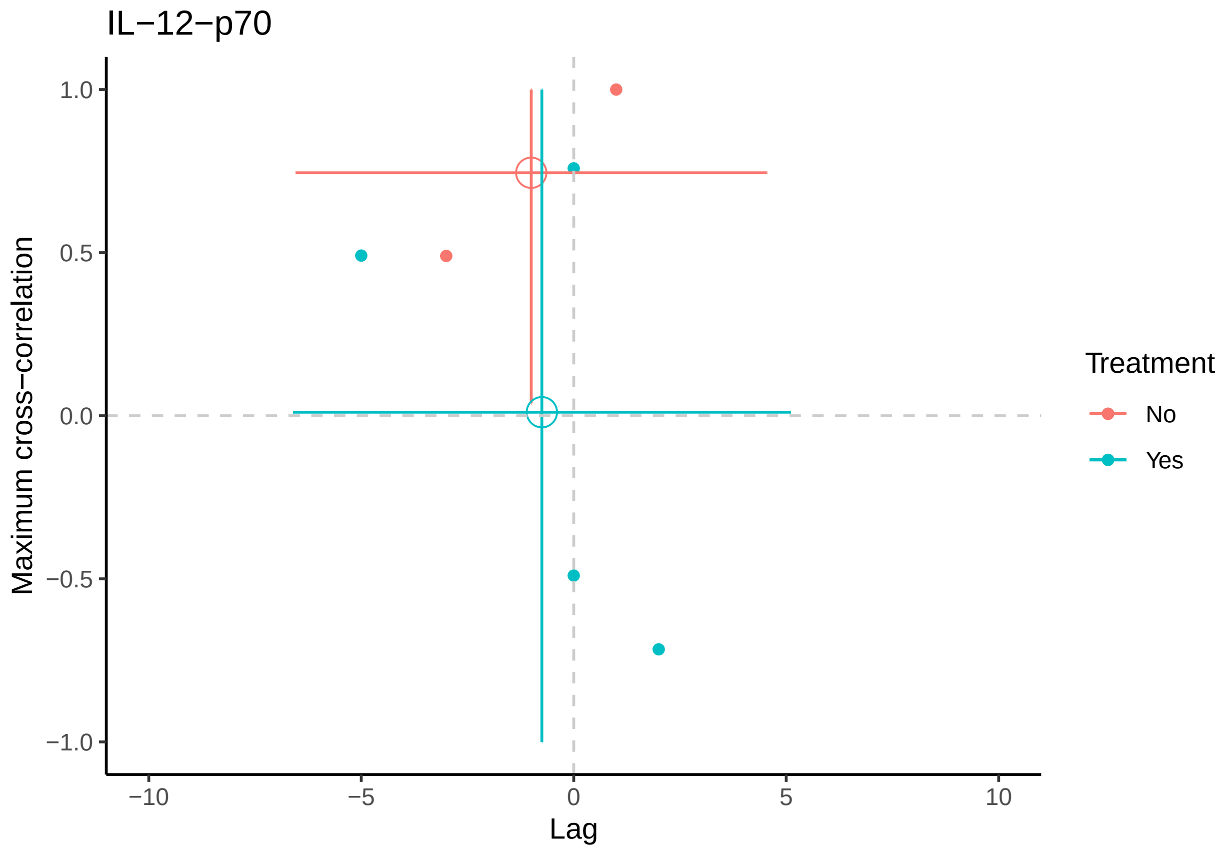
IL−12−p70
Maximum cross−correlation
1.0
0.5
0.0
−0.5
−1.0
−10
−5
0
5
10
Lag
Treatment
No
Yes

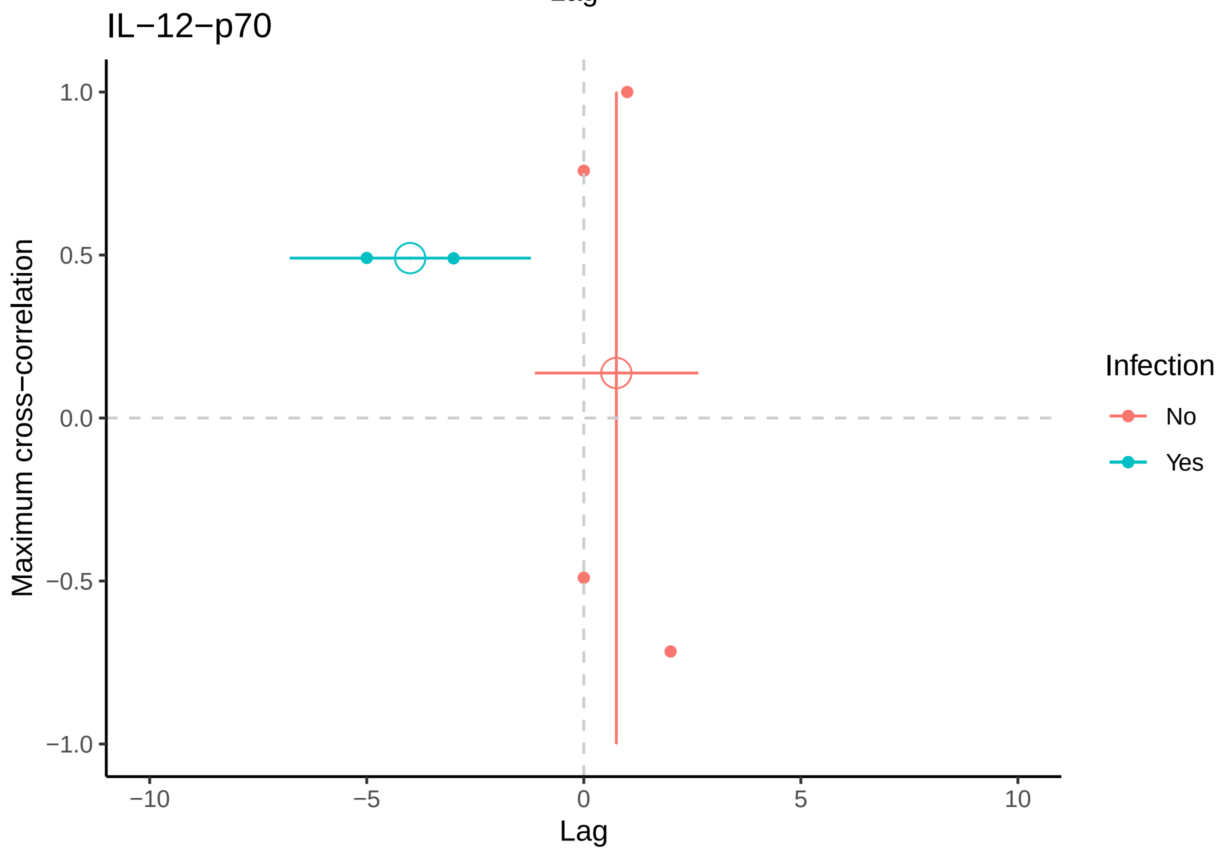
IL−12−p70
Maximum cross−correlation
1.0
0.5
0.0
−0.5
−1.0
−10
−5
0
5
10
Lag
Infection
No
Yes

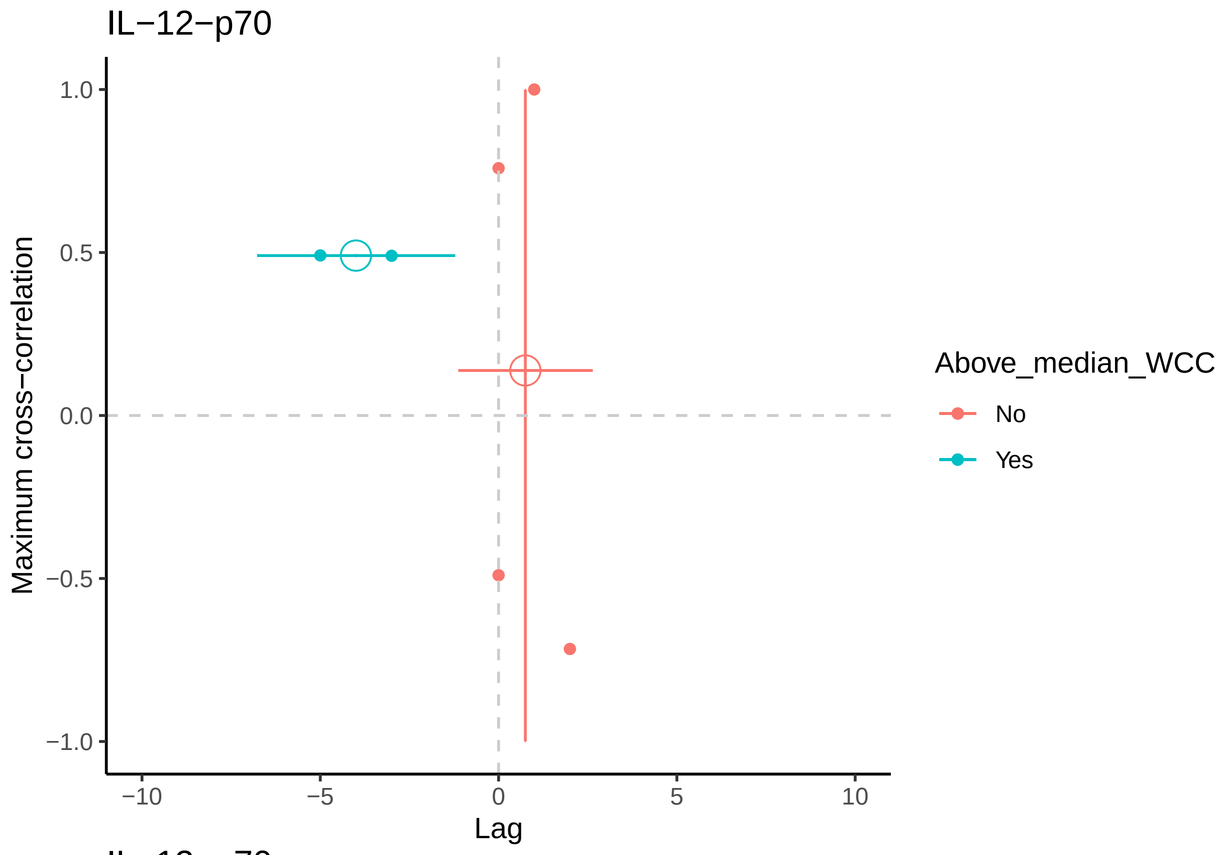
IL−12−p70
1.0
0.5
0.0
−0.5
−1.0
Maximum cross−correlation
−10
−5
0
5
10
Lag
Above_median_WCC
No
Yes

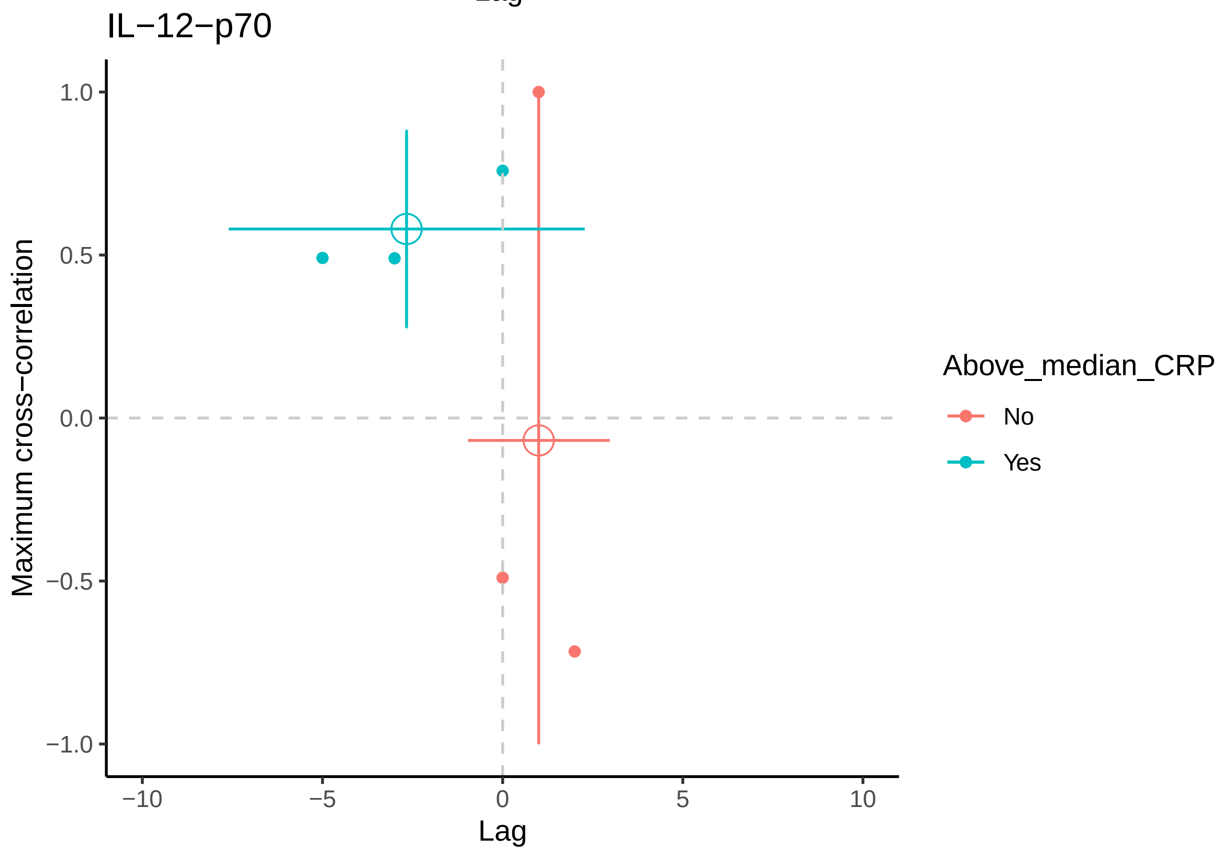
IL−12−p70
1.0
0.5
0.0
−0.5
−1.0
Maximum cross−correlation
−10
−5
0
5
10
Lag
Above_median_CRP
No
Yes

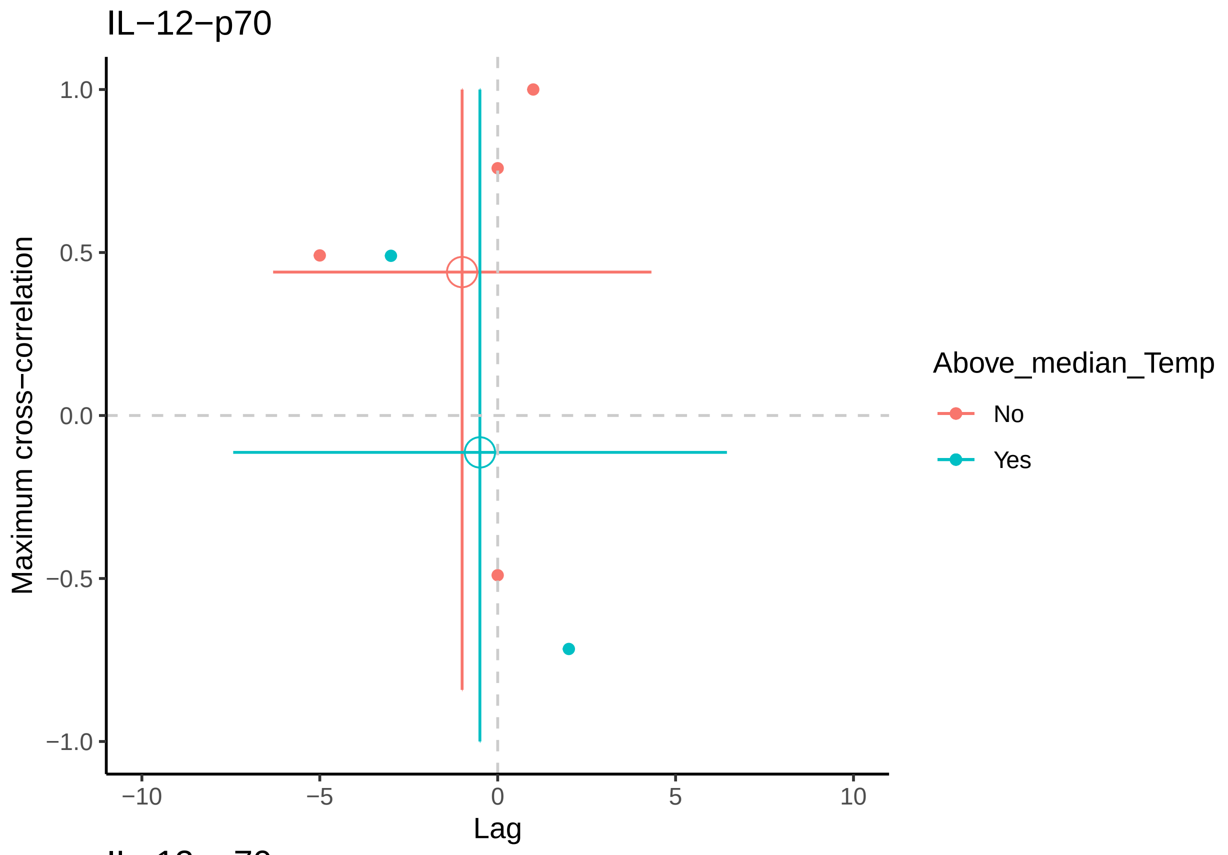
IL−12−p70
Maximum cross−correlation
1.0
0.5
0.0
−0.5
−1.0
−10
−5
0
5
10
Lag
Above_median_Temp
No
Yes

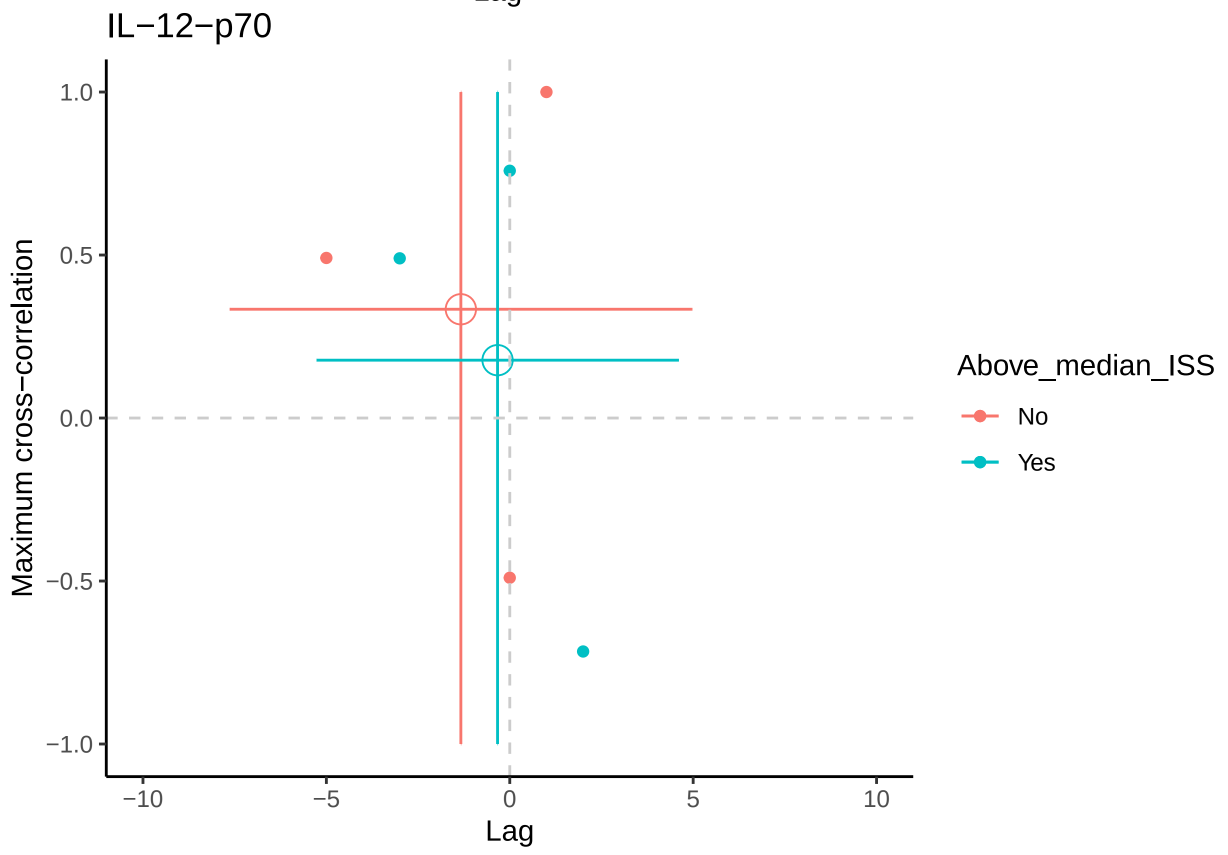
IL−12−p70
Maximum cross−correlation
1.0
0.5
0.0
−0.5
−1.0
−10
−5
0
5
10
Lag
Above_median_ISS
No
Yes

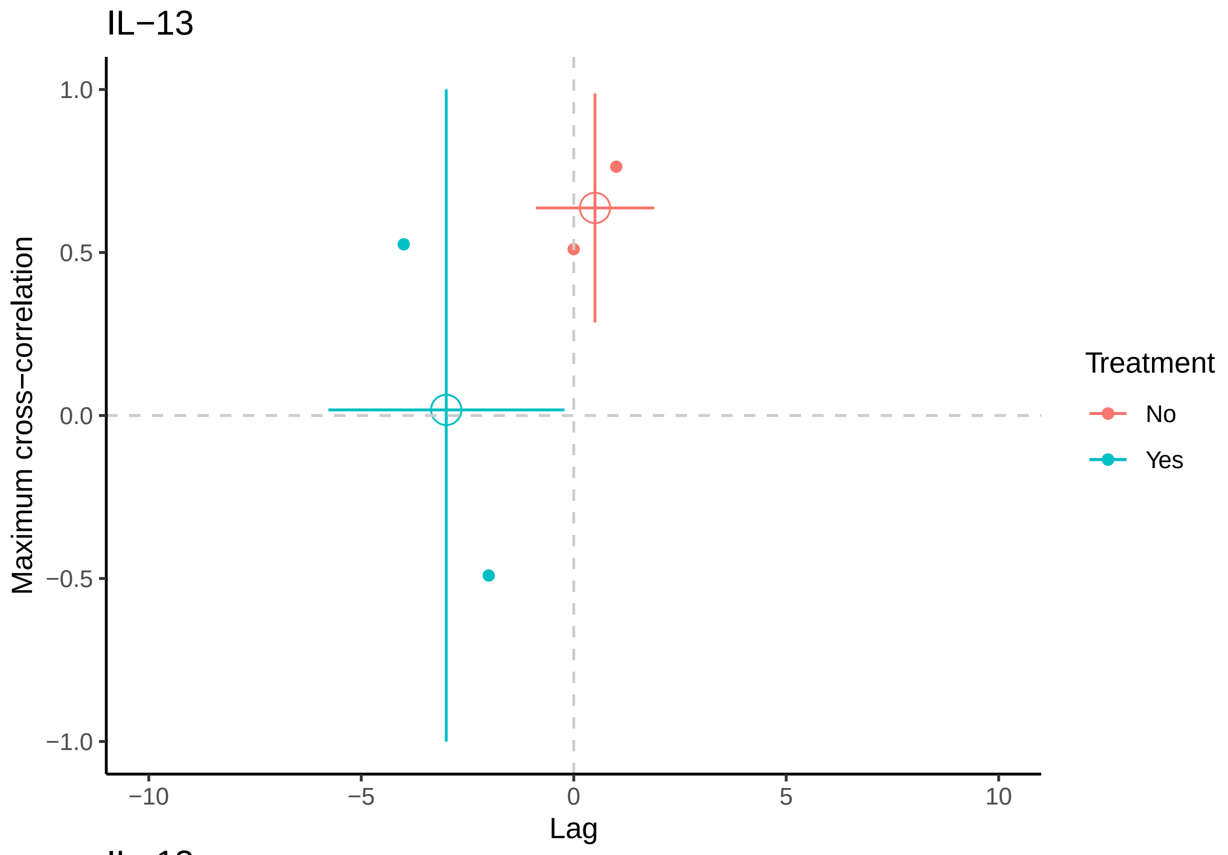
IL−13
1.0
0.5
0.0
−0.5
−1.0
Maximum cross−correlation
−10
−5
0
5
10
Lag
Treatment
No
Yes

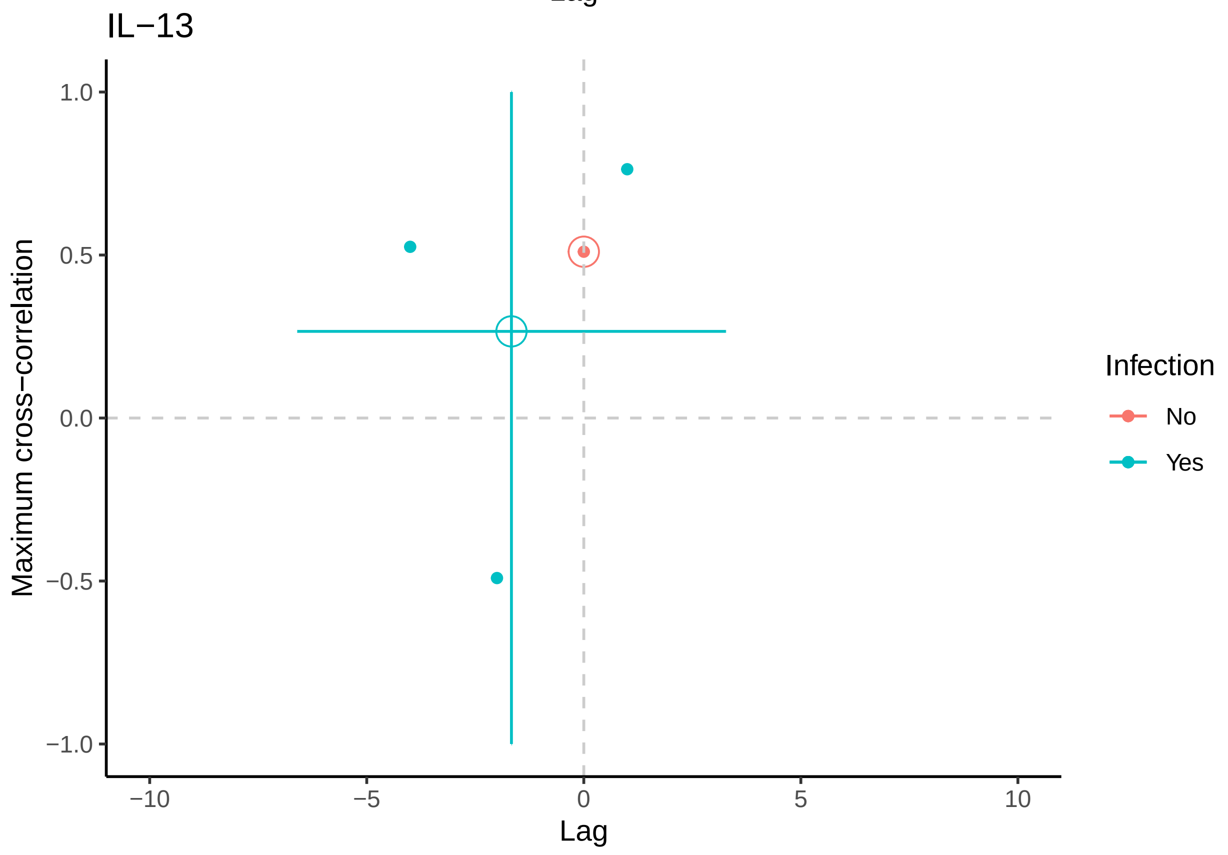
IL−13
1.0
0.5
0.0
−0.5
−1.0
Maximum cross−correlation
−10
−5
0
5
10
Lag
Infection
No
Yes

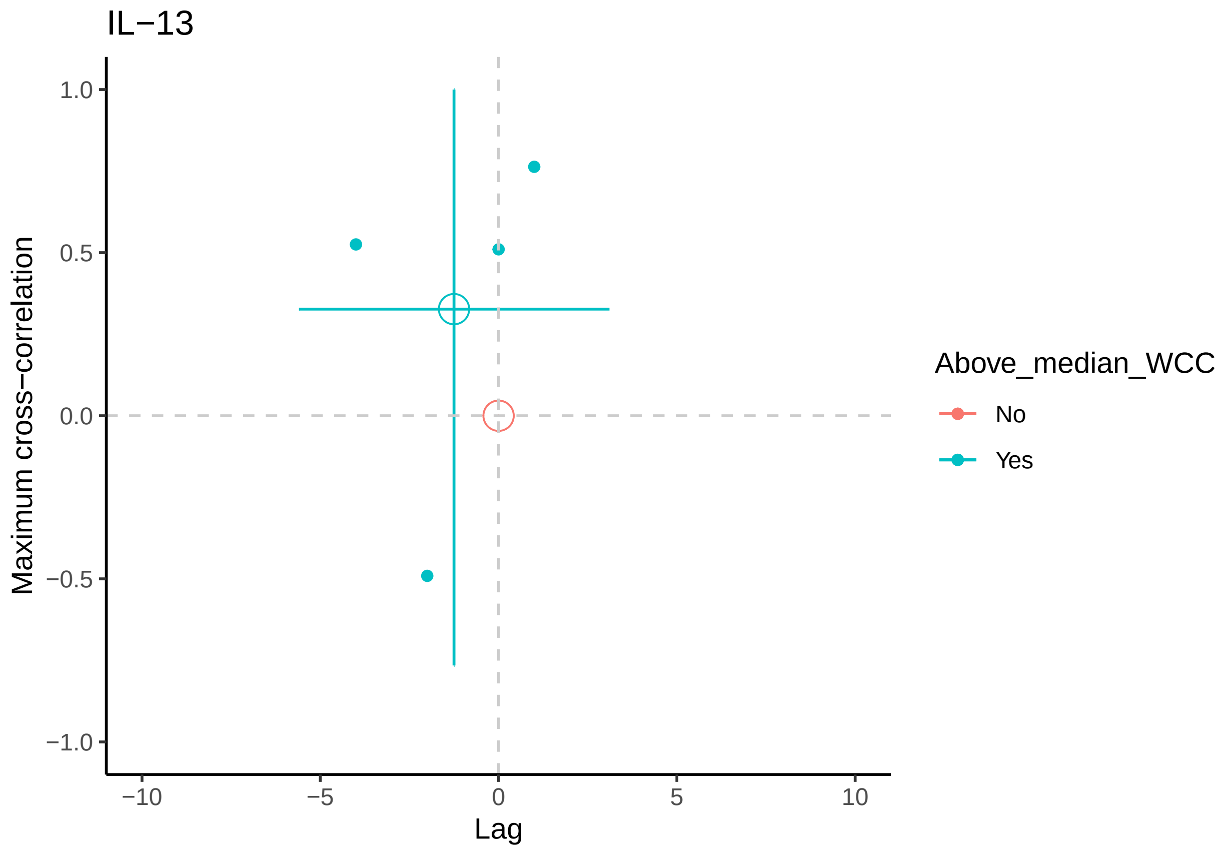
IL−13
Maximum cross−correlation
1.0
0.5
0.0
−0.5
−1.0
−10
−5
0
5
10
Lag
Above_median_WCC
No
Yes

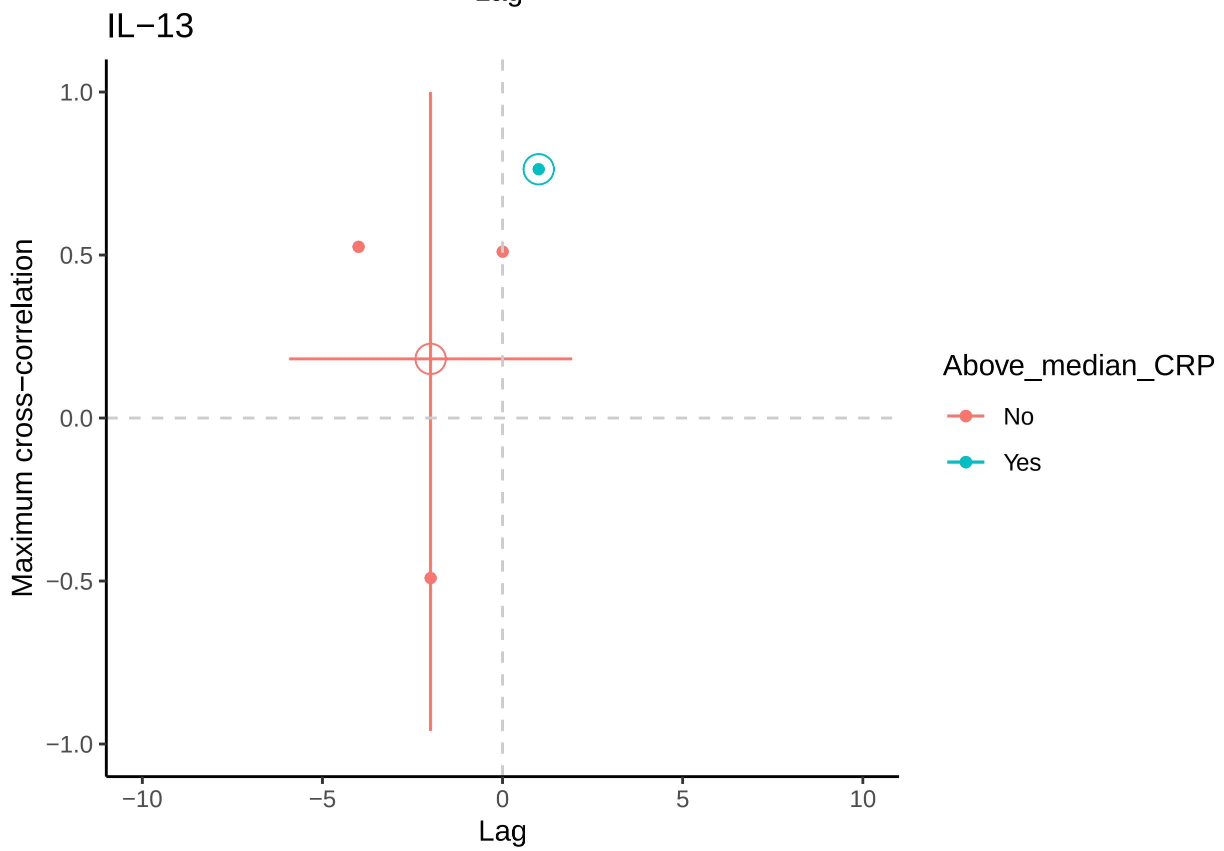
IL−13
Maximum cross−correlation
1.0
0.5
0.0
−0.5
−1.0
−10
−5
0
5
10
Lag
Above_median_CRP
No
Yes

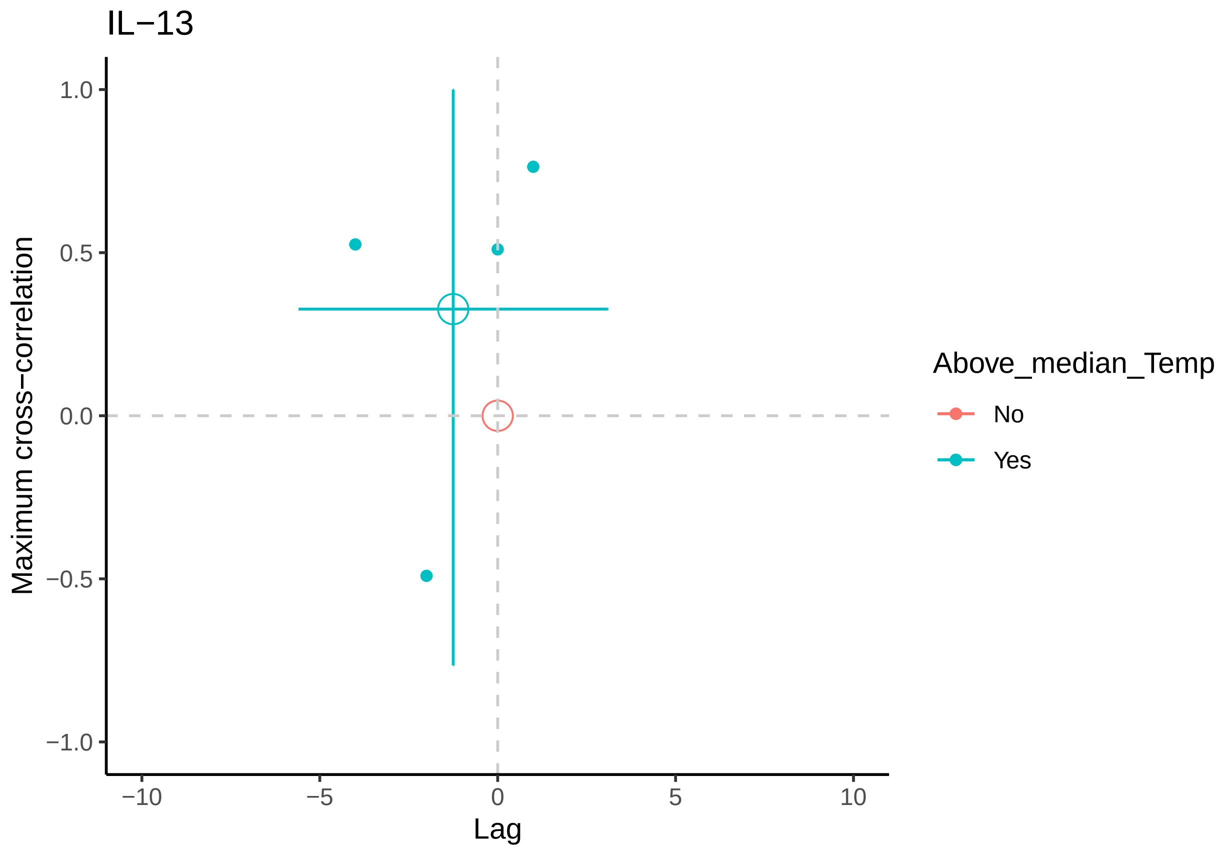
IL−13
1.0
0.5
0.0
−0.5
−1.0
Maximum cross−correlation
−10
−5
0
5
10
Lag
Above_median_Temp
No
Yes

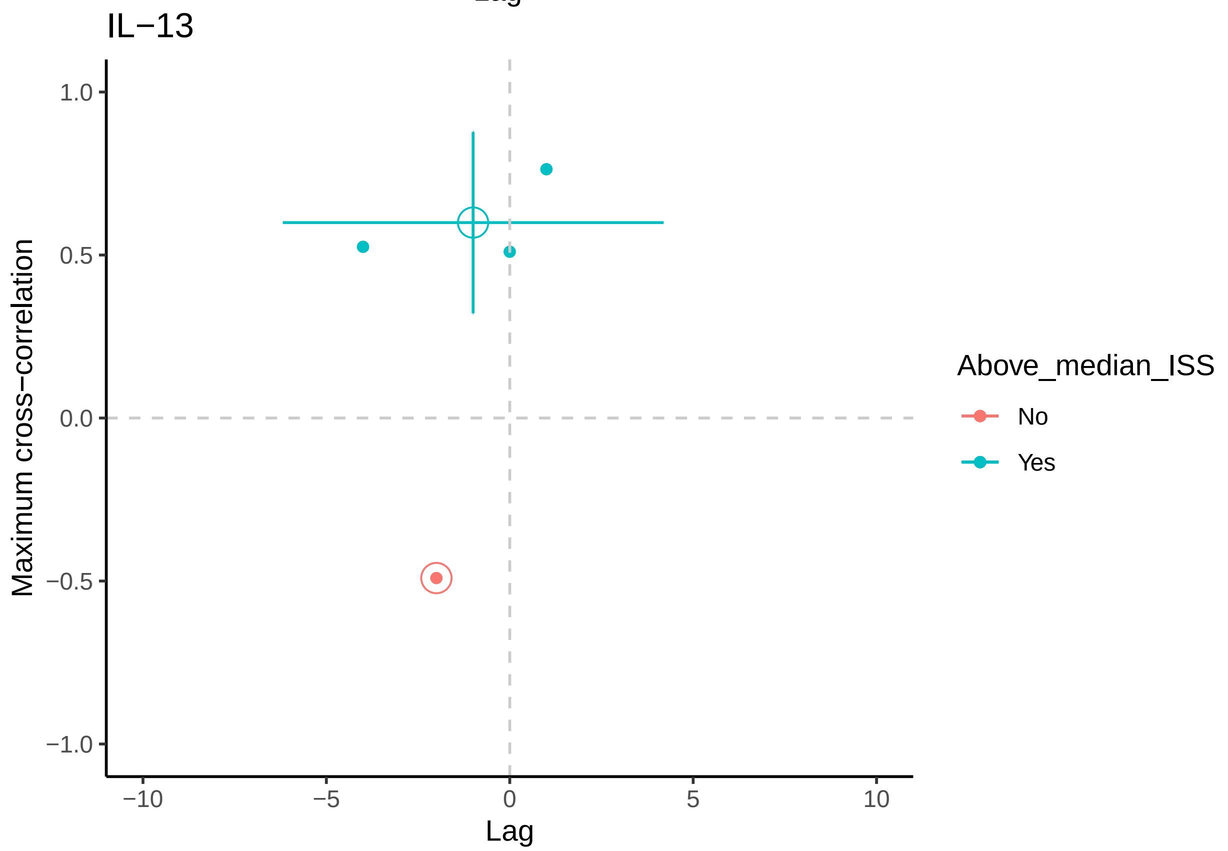
IL−13
1.0
0.5
0.0
−0.5
−1.0
Maximum cross−correlation
−10
−5
0
5
10
Lag
Above_median_ISS
No
Yes

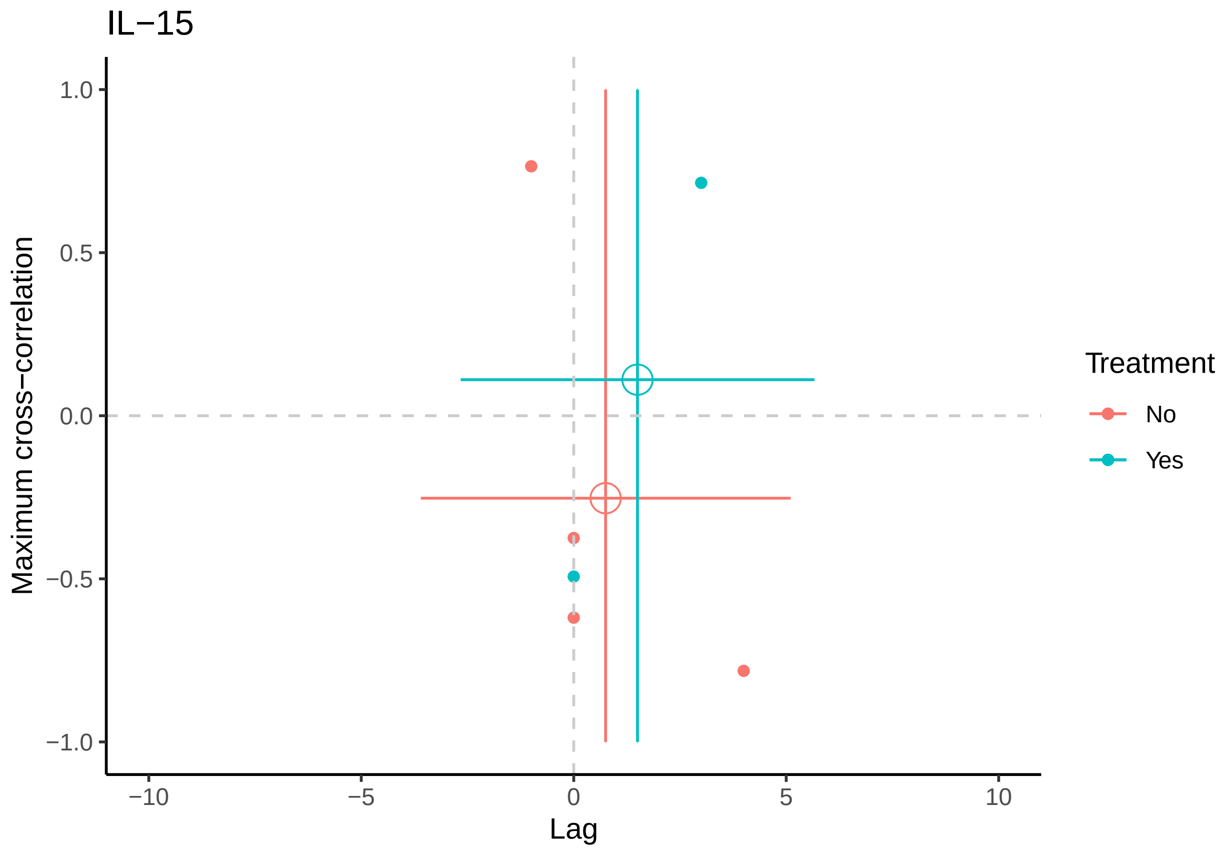
IL−15
Maximum cross−correlation
1.0
0.5
0.0
−0.5
−1.0
−10
−5
0
5
10
Lag
Treatment
No
Yes

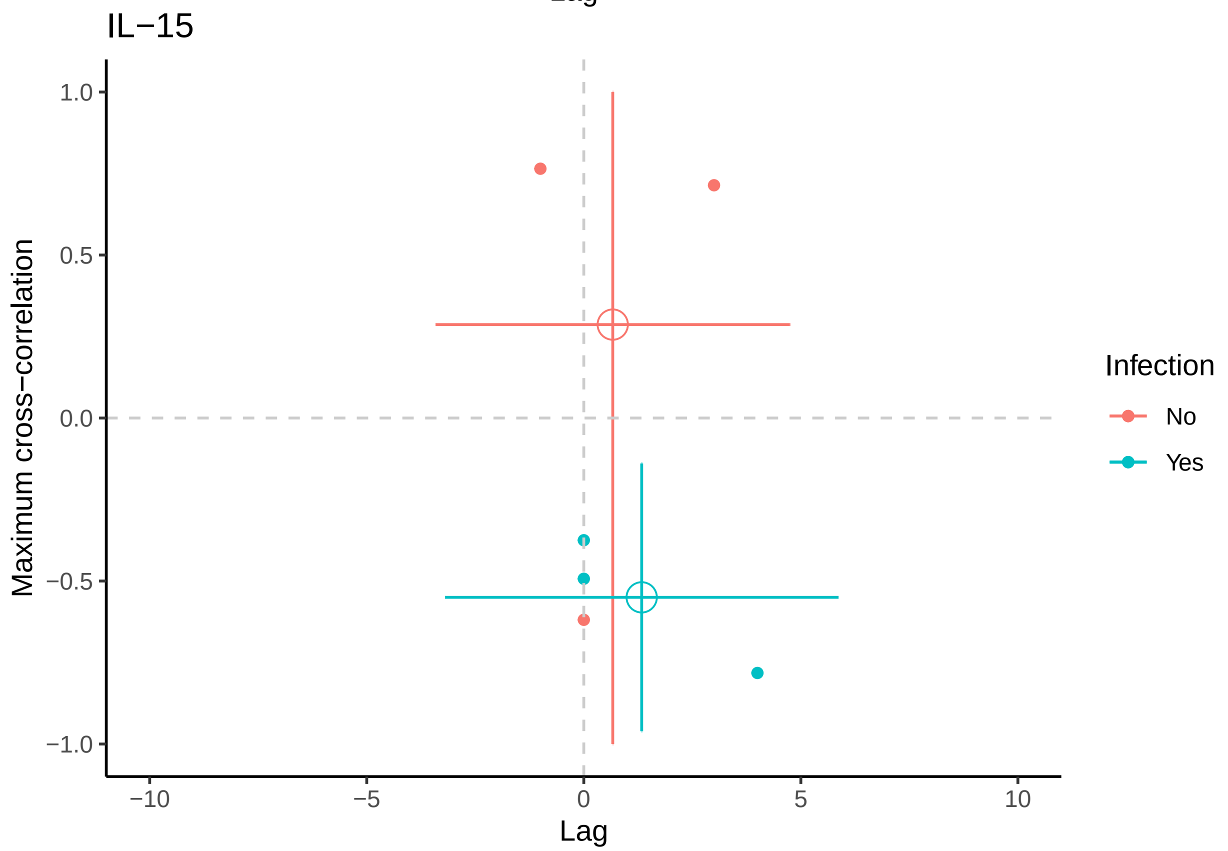
IL−15
Maximum cross−correlation
1.0
0.5
0.0
−0.5
−1.0
−10
−5
0
5
10
Lag
Infection
No
Yes

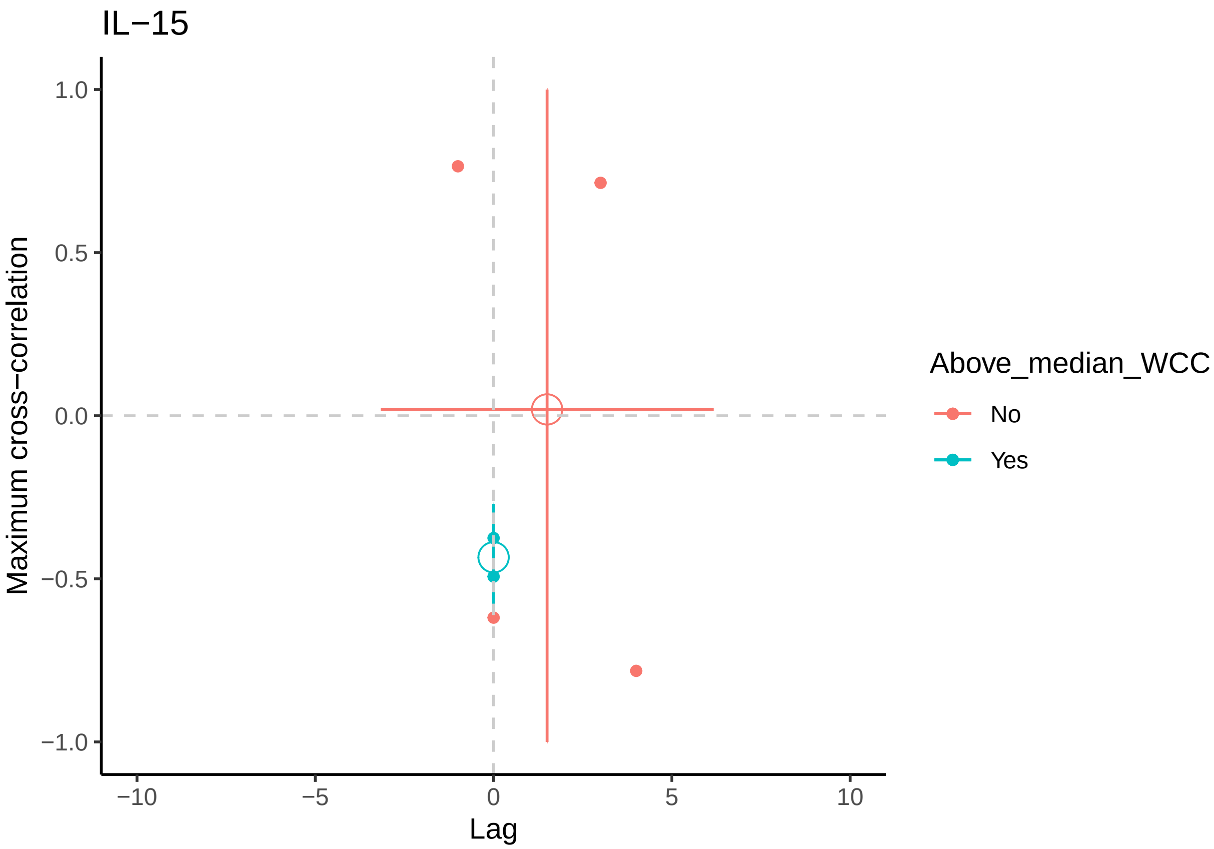
IL−15
Maximum cross−correlation
1.0
0.5
0.0
−0.5
−1.0
−10
−5
0
5
10
Lag
Above_median_WCC
No
Yes

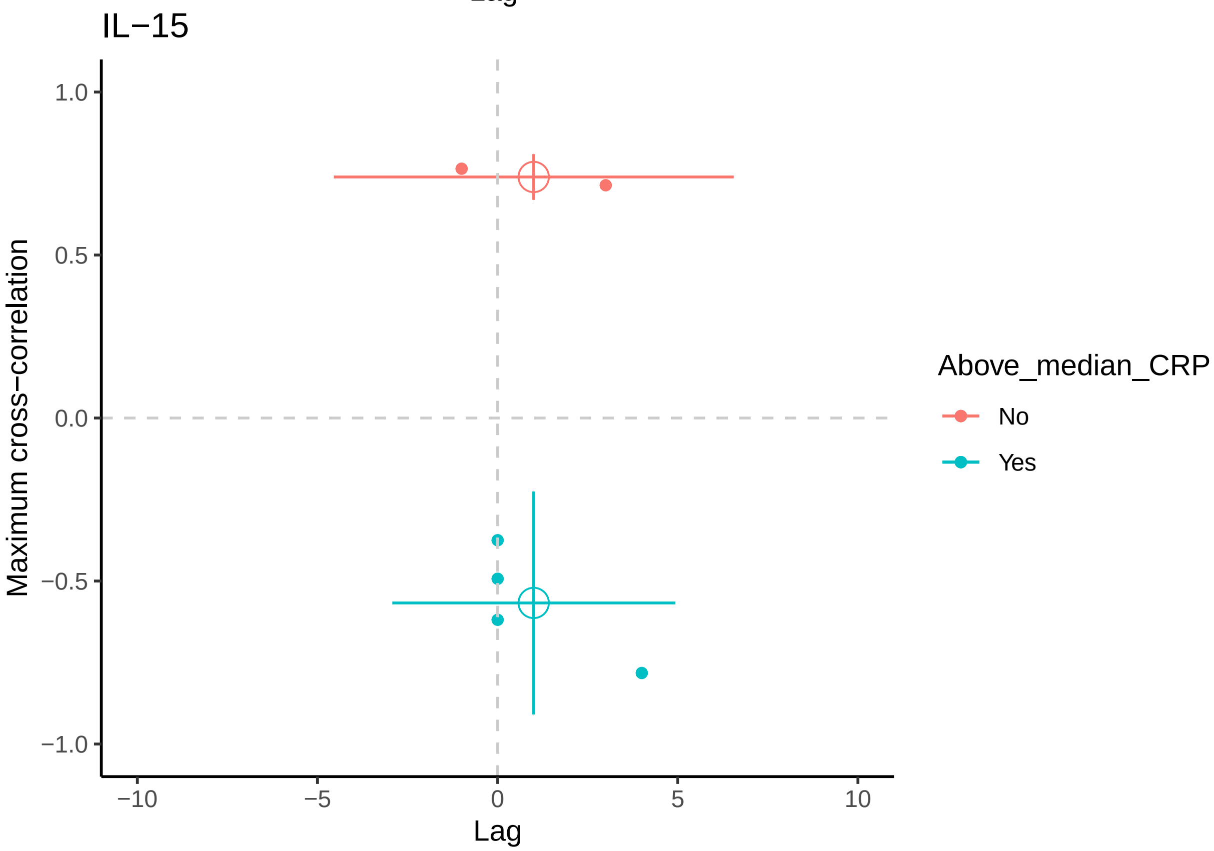
IL−15
Maximum cross−correlation
1.0
0.5
0.0
−0.5
−1.0
−10
−5
0
5
10
Lag
Above_median_CRP
No
Yes

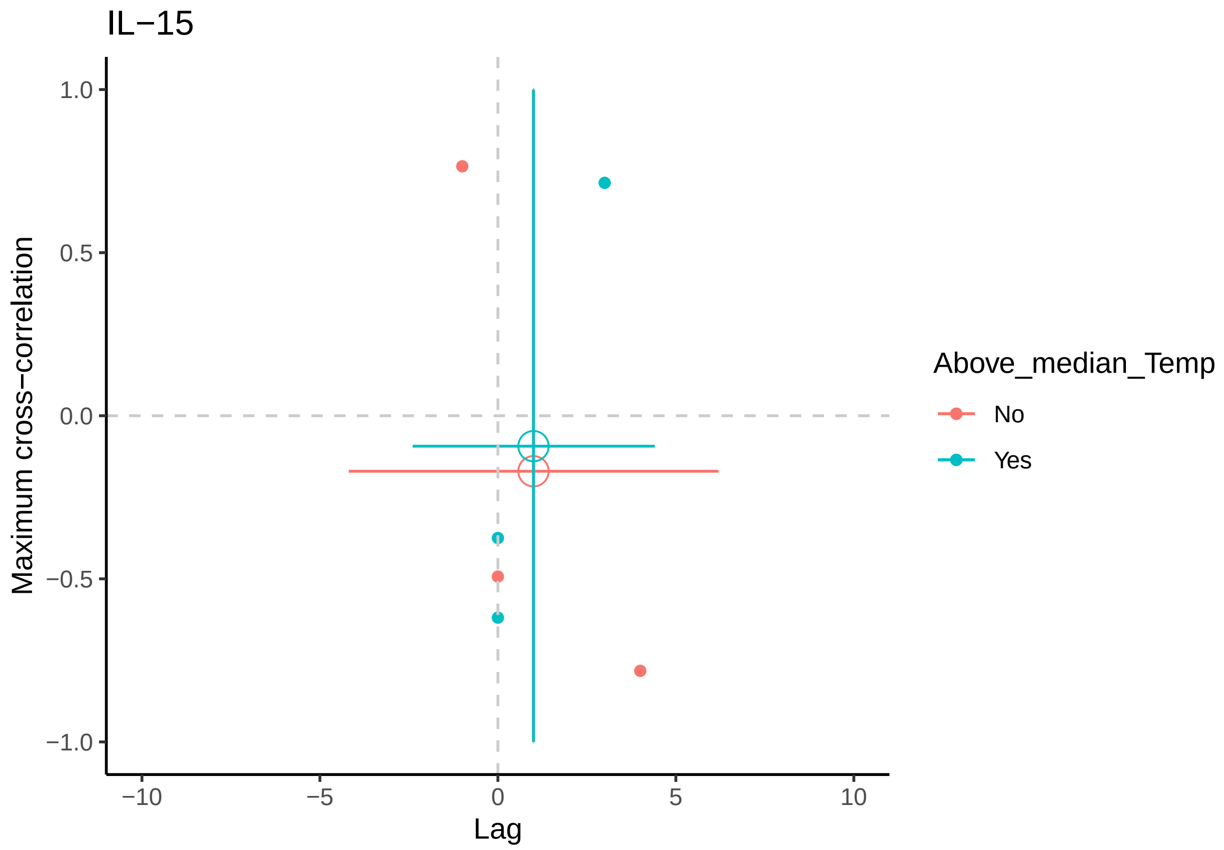
IL−15
Maximum cross−correlation
1.0
0.5
0.0
−0.5
−1.0
−10
−5
0
5
10
Lag
Above_median_Temp
No
Yes

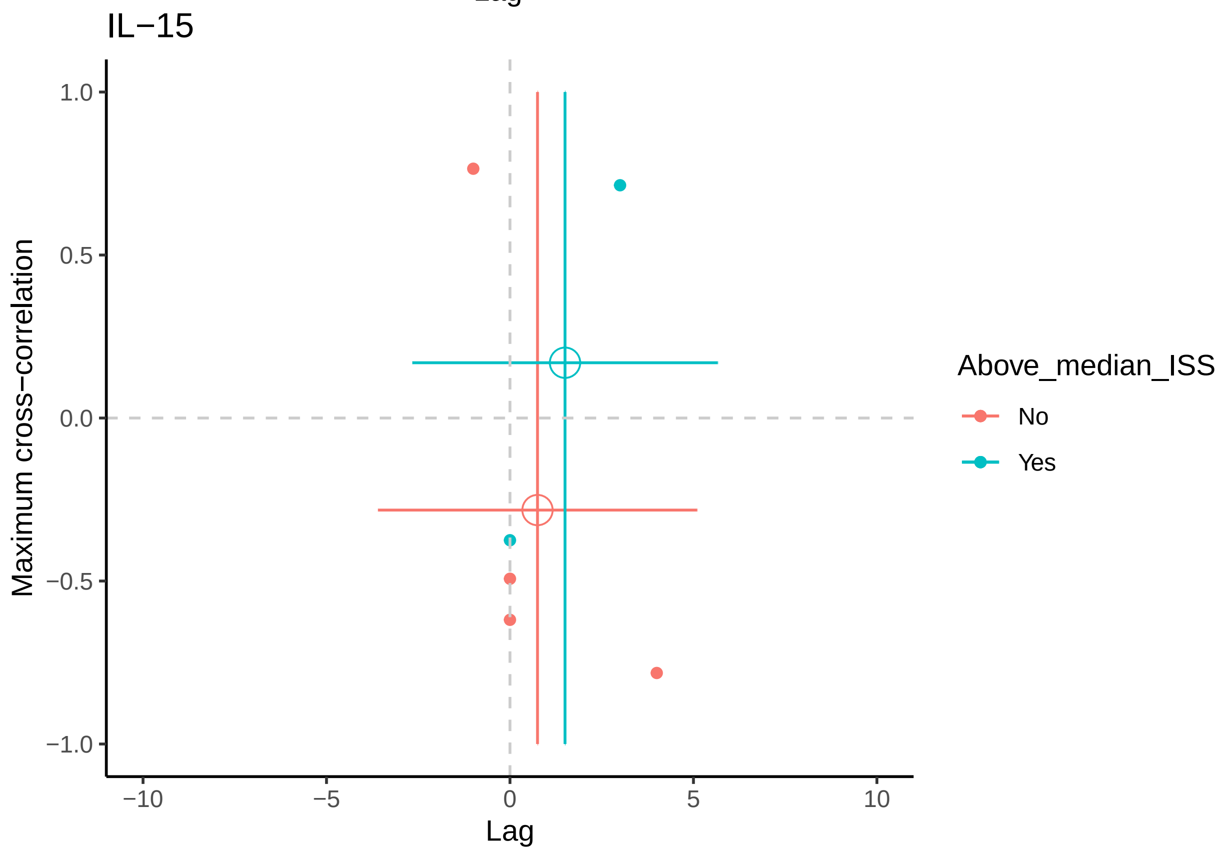
IL−15
Maximum cross−correlation
1.0
0.5
0.0
−0.5
−1.0
−10
−5
0
5
10
Lag
Above_median_ISS
No
Yes

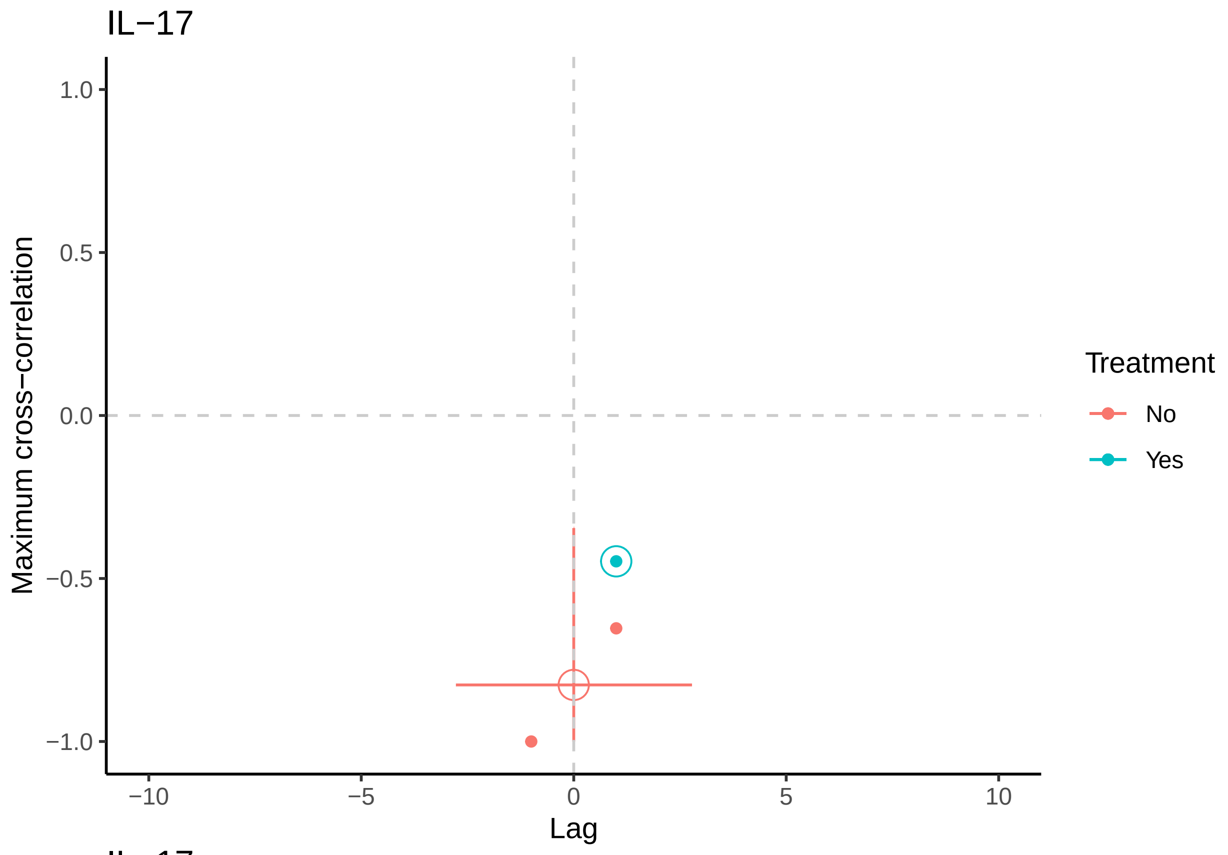
IL−17
Maximum cross−correlation
1.0
0.5
0.0
−0.5
−1.0
−10
−5
0
5
10
Lag
Treatment
No
Yes

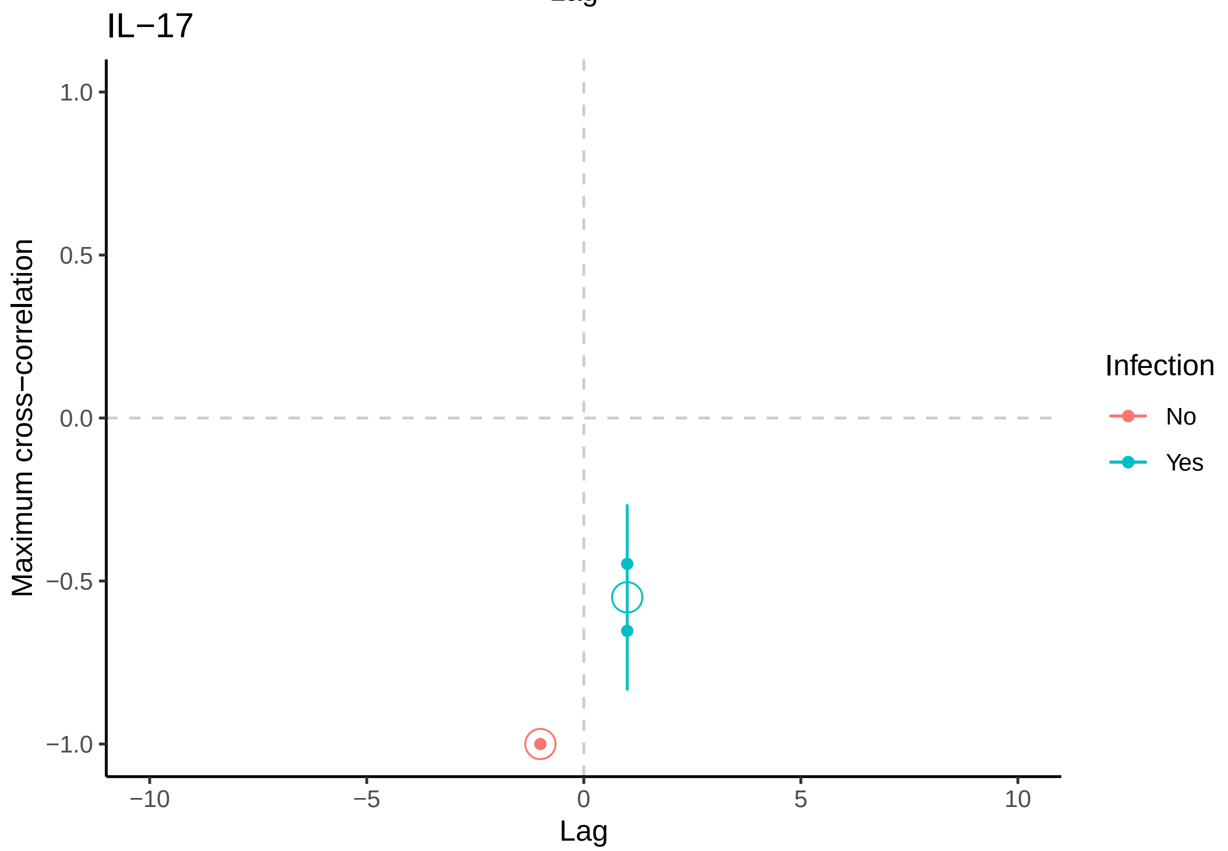
IL−17
Maximum cross−correlation
1.0
0.5
0.0
−0.5
−1.0
−10
−5
0
5
10
Lag
Infection
No
Yes

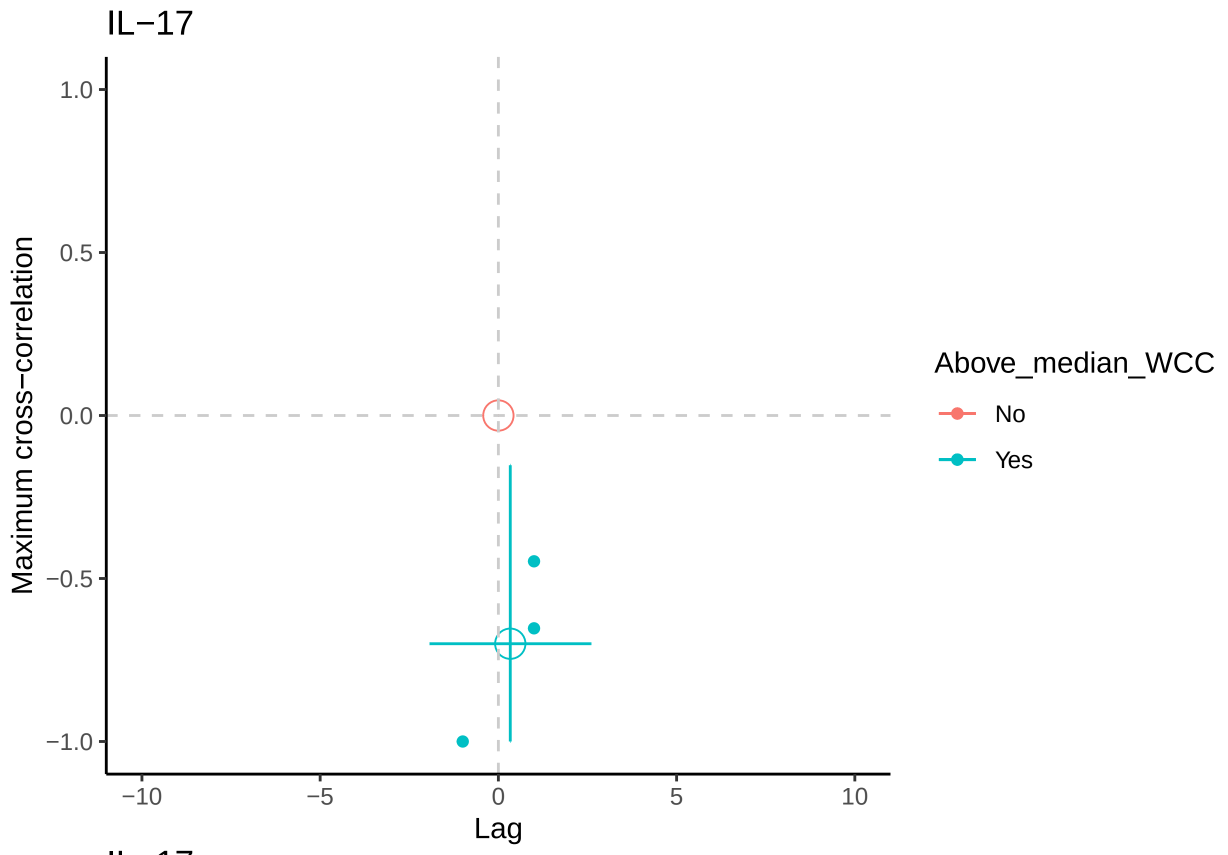
IL−17
Maximum cross−correlation
1.0
0.5
0.0
−0.5
−1.0
−10
−5
0
5
10
Lag
Above_median_WCC
No
Yes

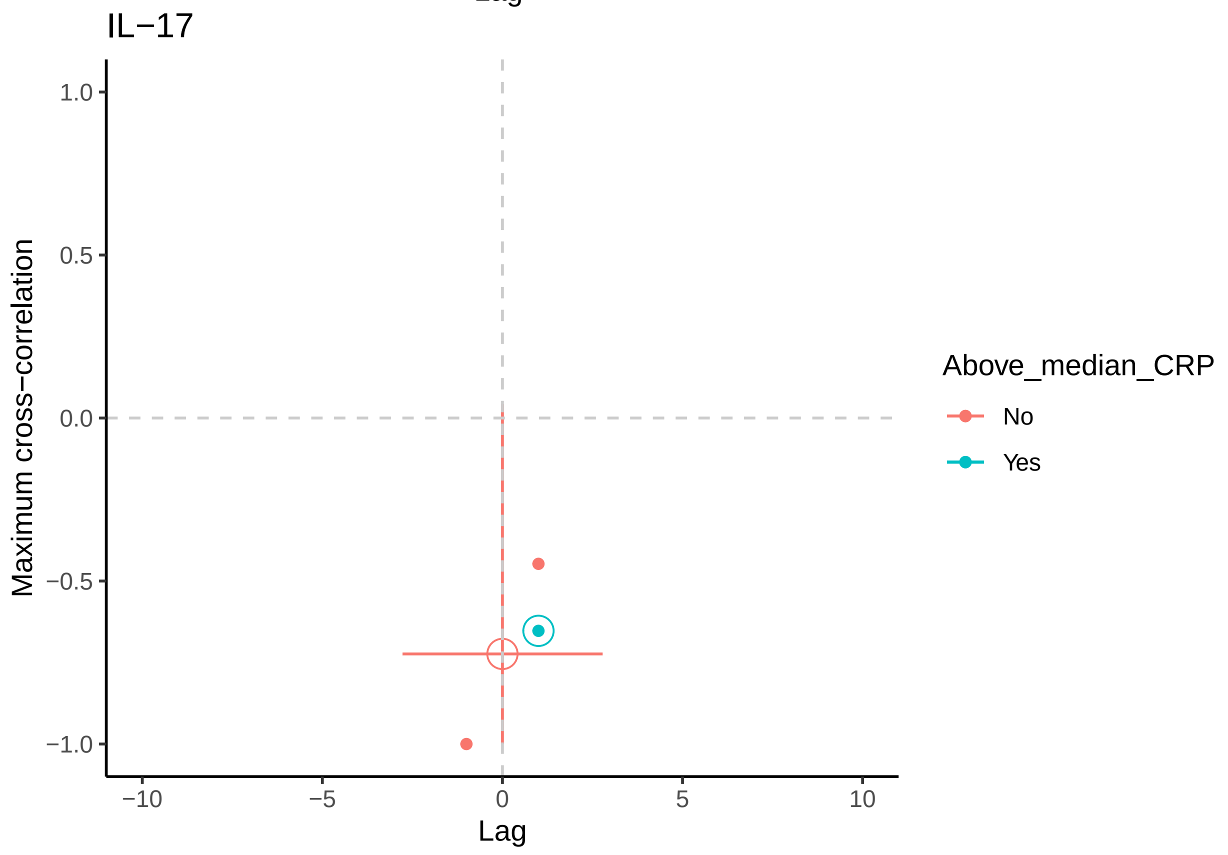
IL−17
Maximum cross−correlation
1.0
0.5
0.0
−0.5
−1.0
−10
−5
0
5
10
Lag
Above_median_CRP
No
Yes

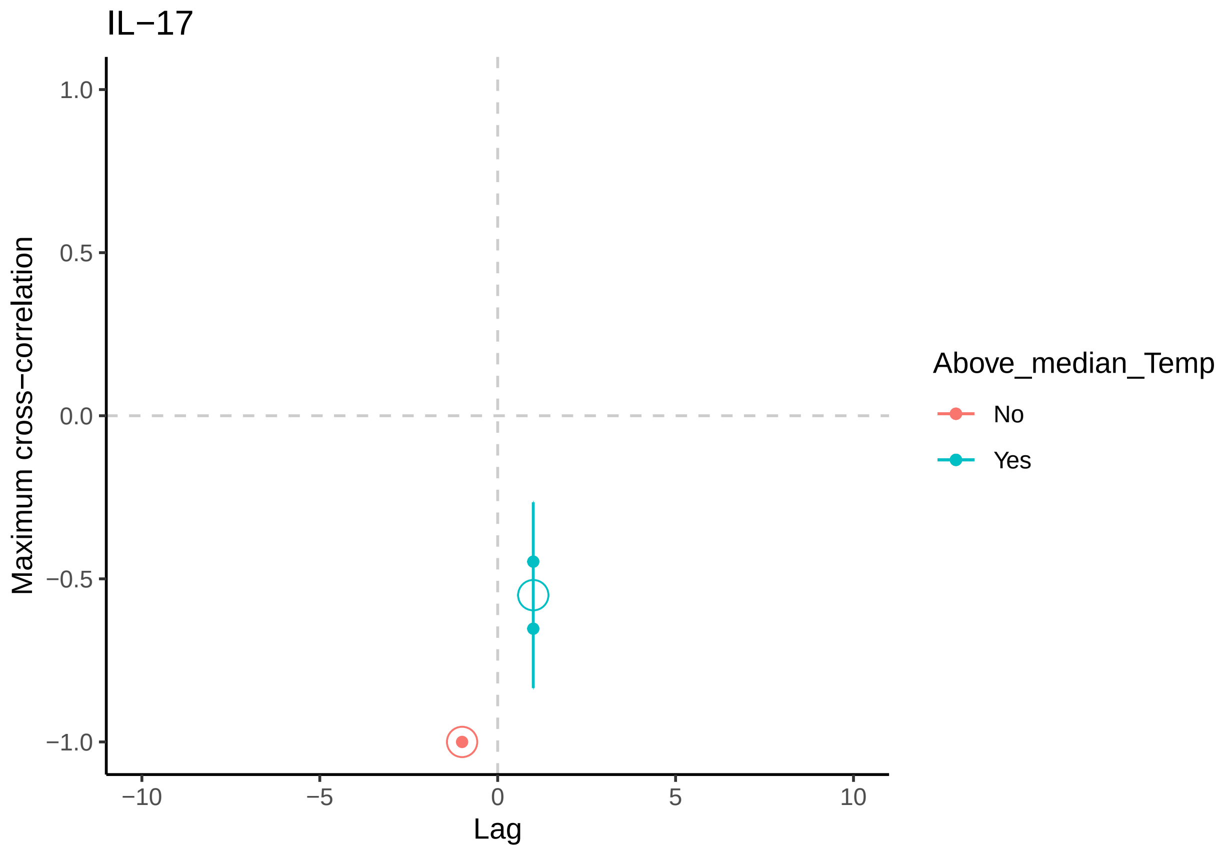
IL−17
Maximum cross−correlation
1.0
0.5
0.0
−0.5
−1.0
−10
−5
0
5
10
Lag
Above_median_Temp
No
Yes

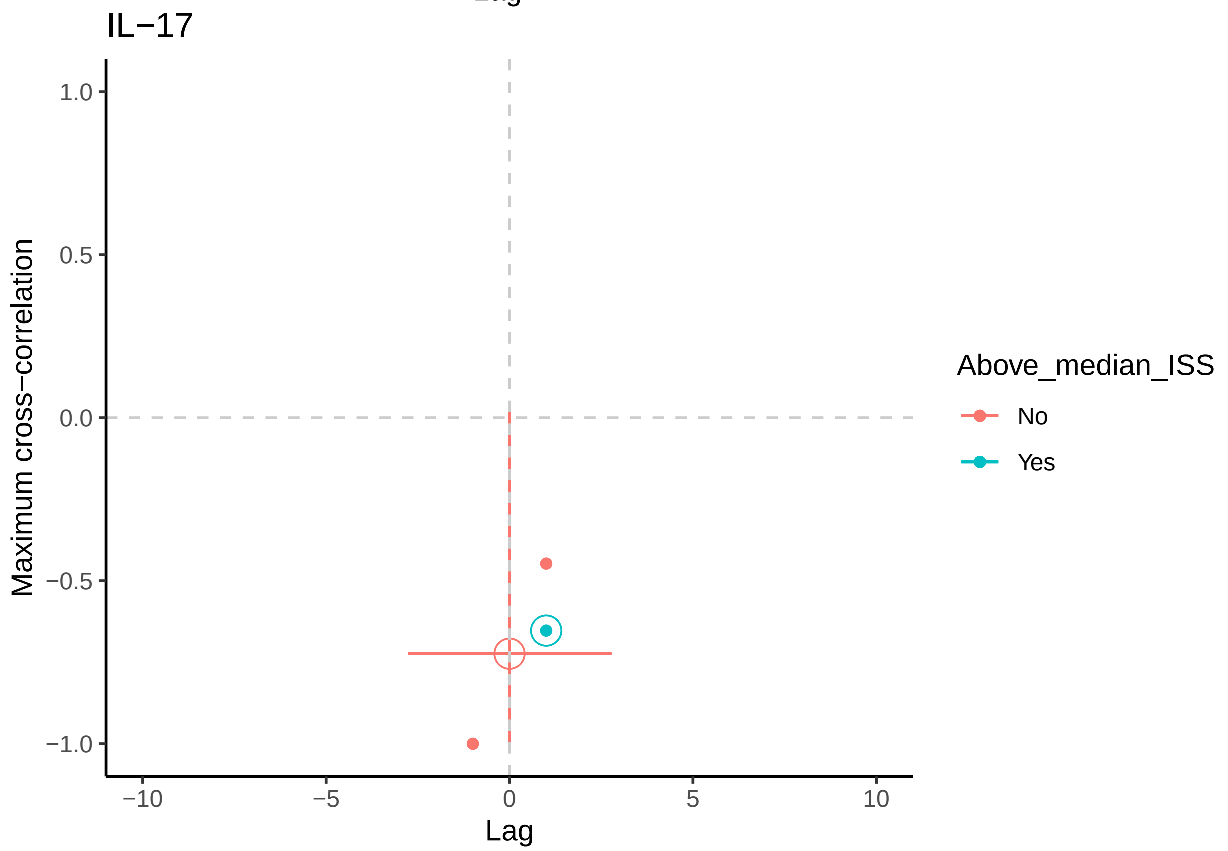
IL−17
Maximum cross−correlation
1.0
0.5
0.0
−0.5
−1.0
−10
−5
0
5
10
Lag
Above_median_ISS
No
Yes

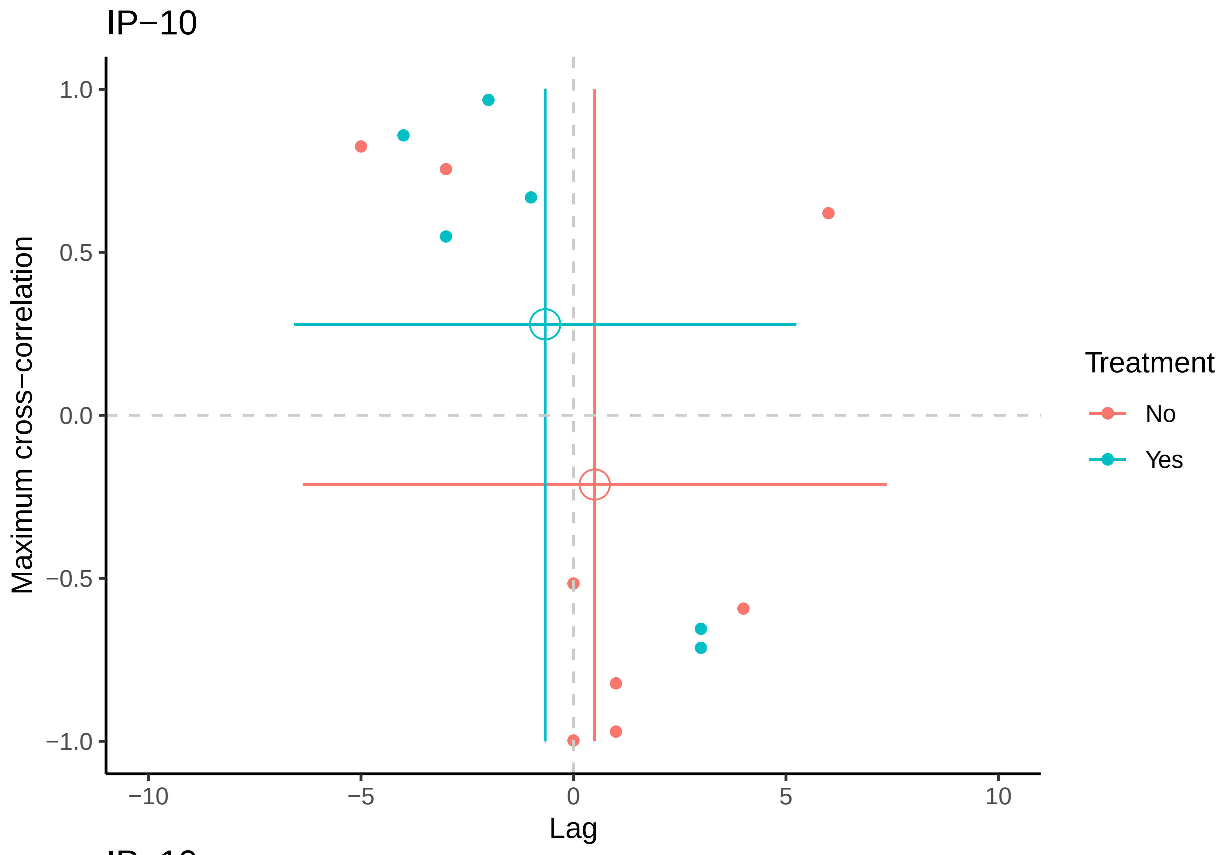
IP−10
Maximum cross−correlation
Lag
Treatment
No
Yes
1.0
0.5
0.0
−0.5
−1.0
−10
−5
0
5
10

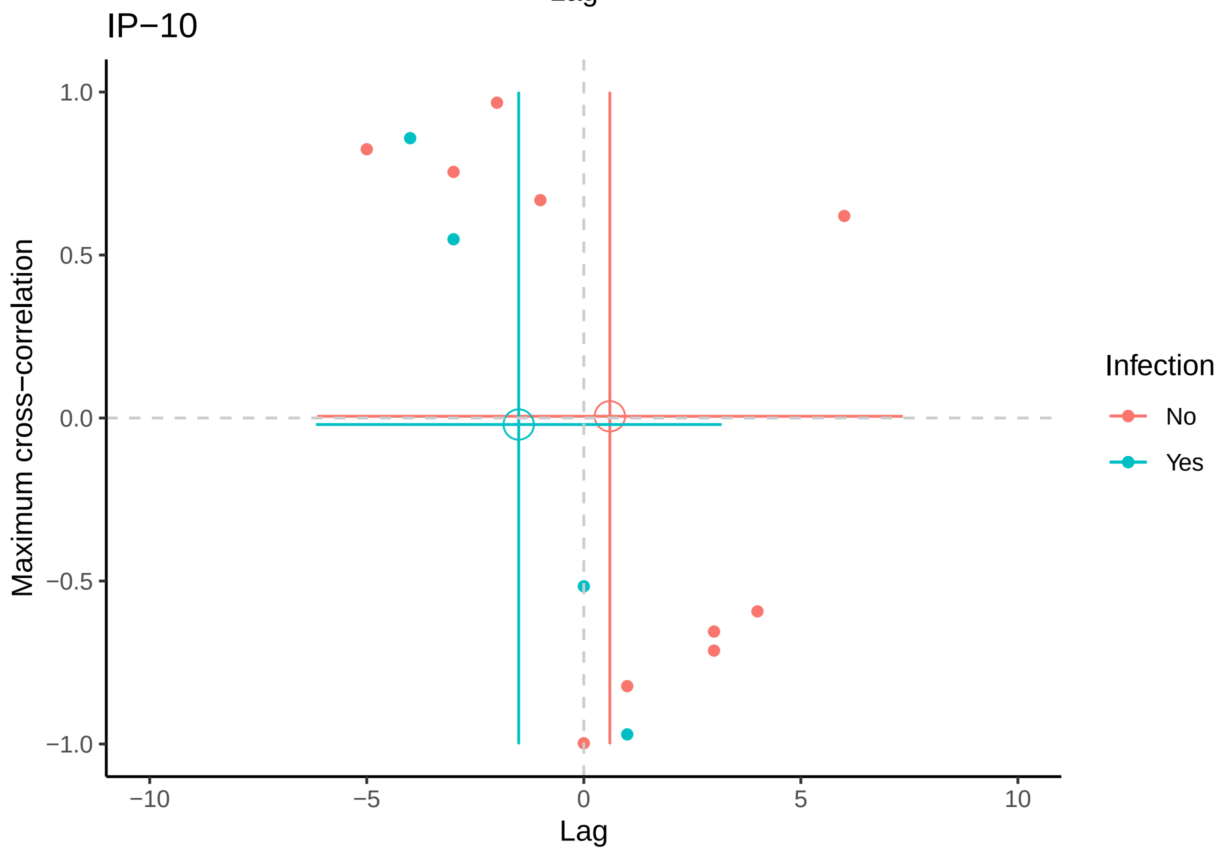
IP−10
Maximum cross−correlation
Lag
Infection
No
Yes
1.0
0.5
0.0
−0.5
−1.0
−10
−5
0
5
10

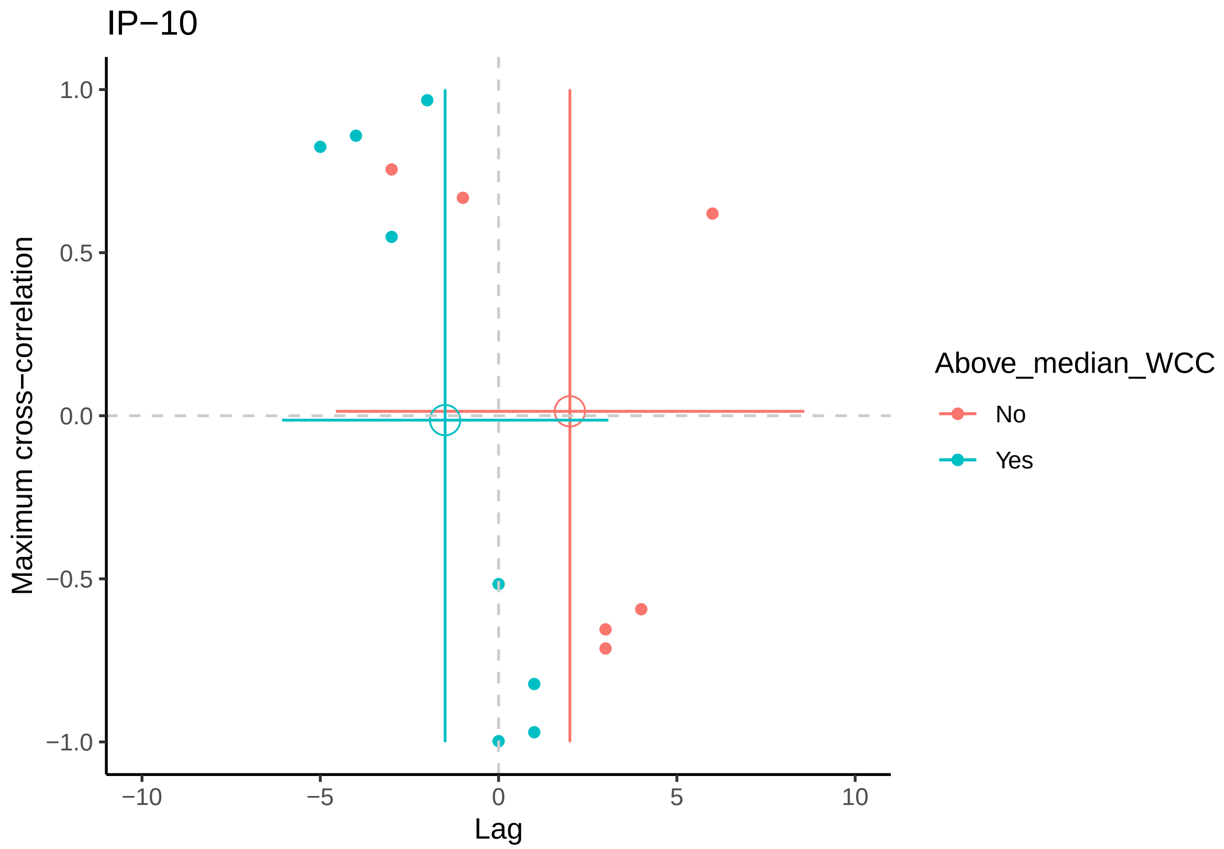
IP−10
Maximum cross−correlation
1.0
0.5
0.0
−0.5
−1.0
−10
−5
0
5
10
Lag
Above_median_WCC
No
Yes

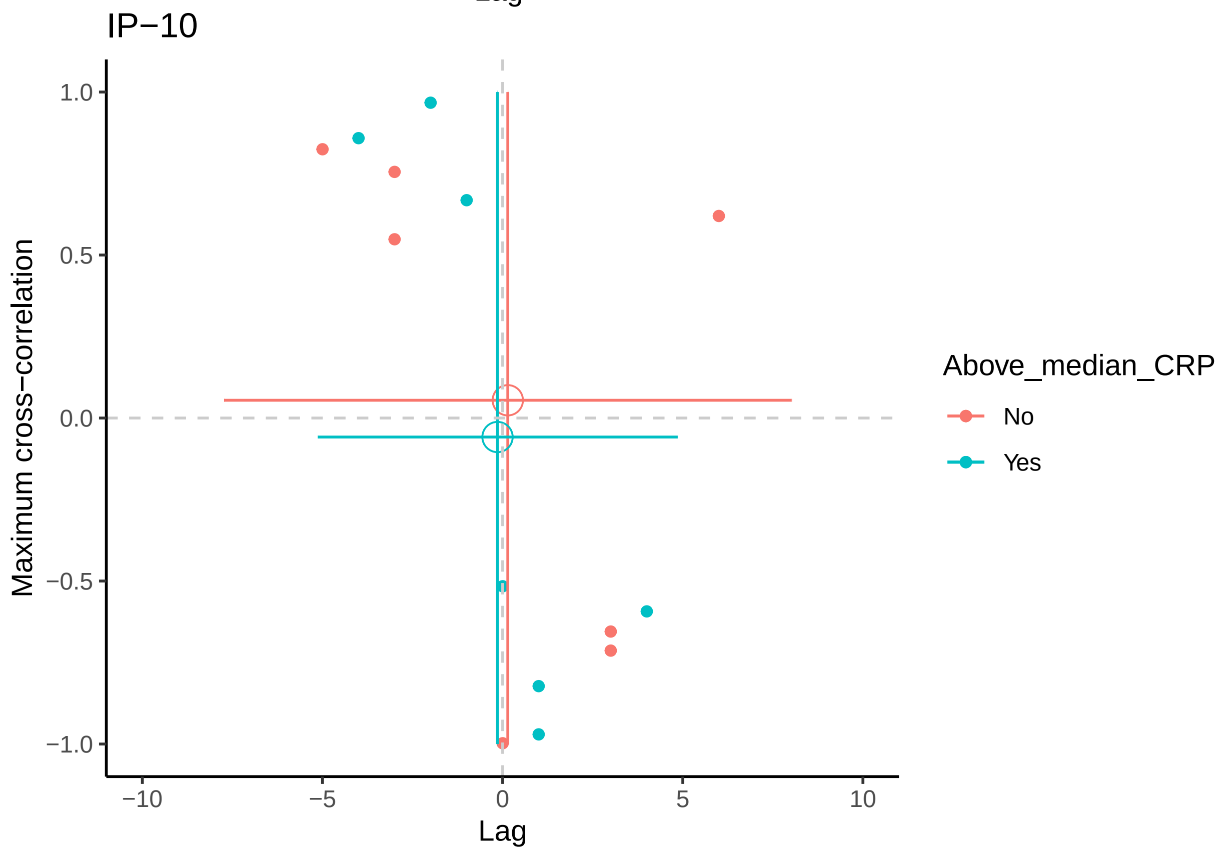
IP−10
Maximum cross−correlation
1.0
0.5
0.0
−0.5
−1.0
−10
−5
0
5
10
Lag
Above_median_CRP
No
Yes

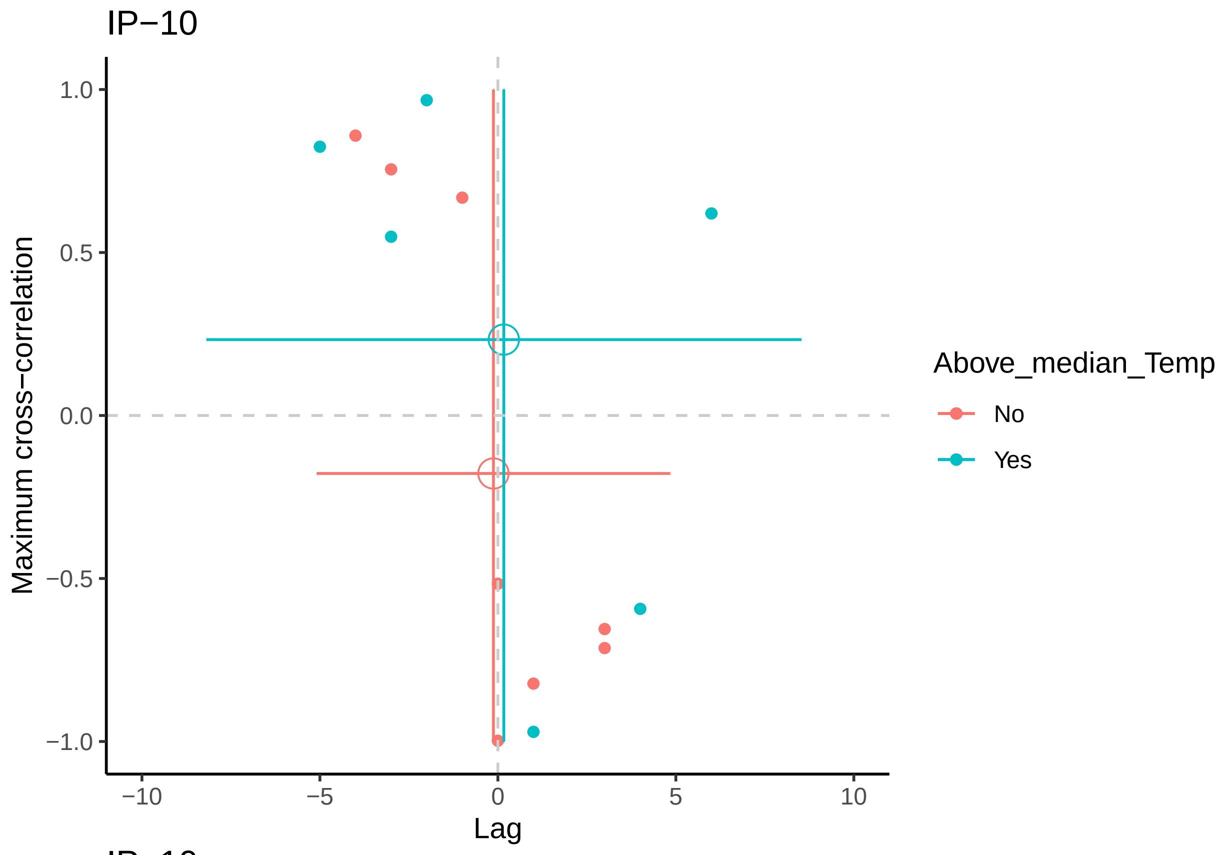
IP−10
1.0
0.5
0.0
−0.5
−1.0
Maximum cross−correlation
−10
−5
0
5
10
Lag
Above_median_Temp
No
Yes

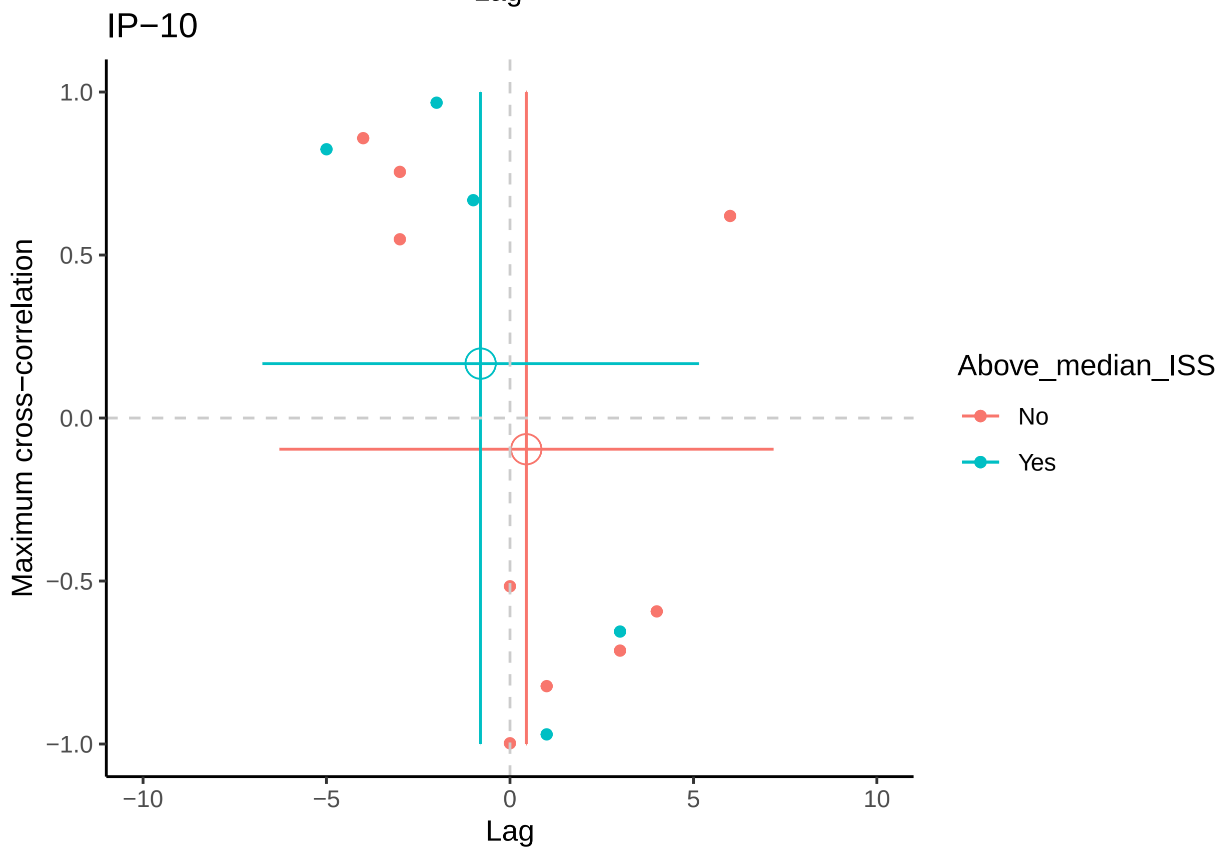
IP−10
1.0
0.5
0.0
−0.5
−1.0
Maximum cross−correlation
−10
−5
0
5
10
Lag
Above_median_ISS
No
Yes

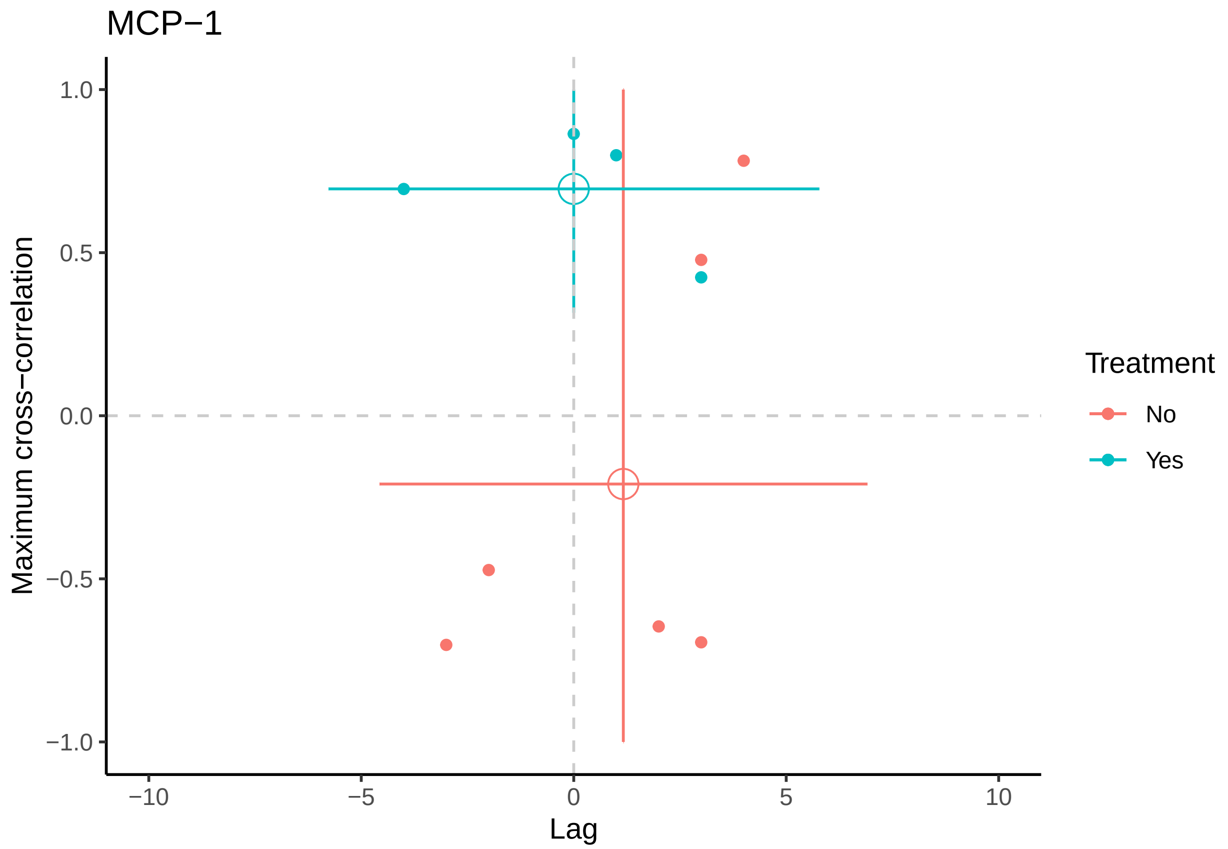
MCP−1
Maximum cross−correlation
1.0
0.5
0.0
−0.5
−1.0
−10
−5
0
5
10
Lag
Treatment
No
Yes

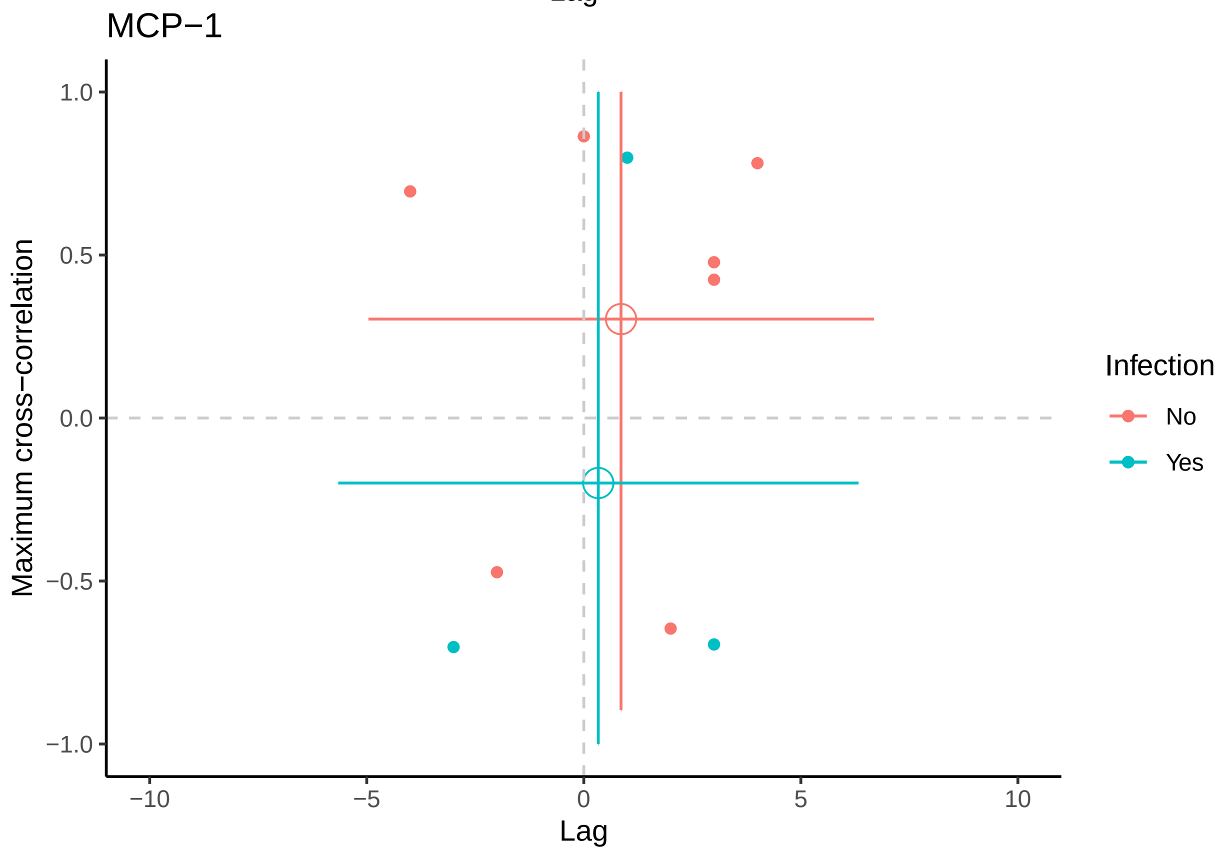
MCP−1
Maximum cross−correlation
1.0
0.5
0.0
−0.5
−1.0
−10
−5
0
5
10
Lag
Infection
No
Yes

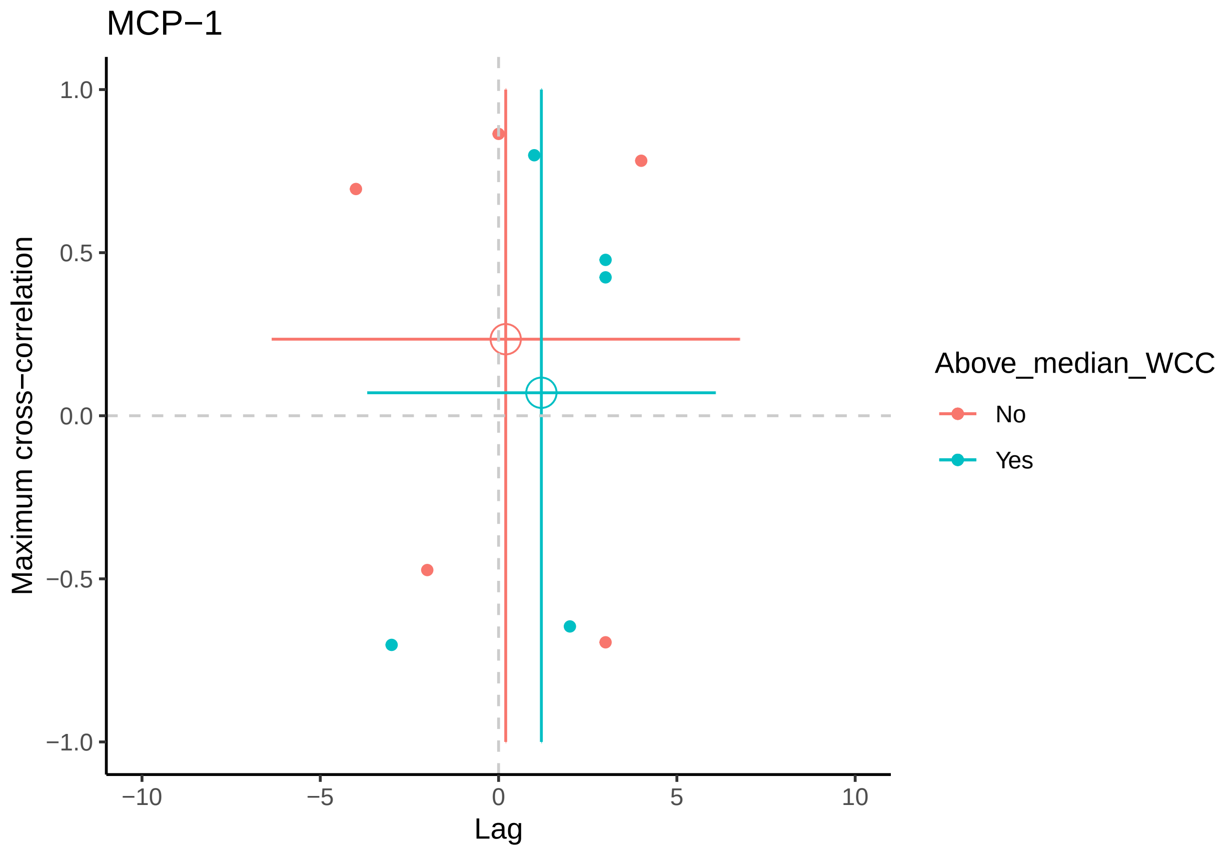
MCP−1
Maximum cross−correlation
1.0
0.5
0.0
−0.5
−1.0
−10
−5
0
5
10
Lag
Above_median_WCC
No
Yes

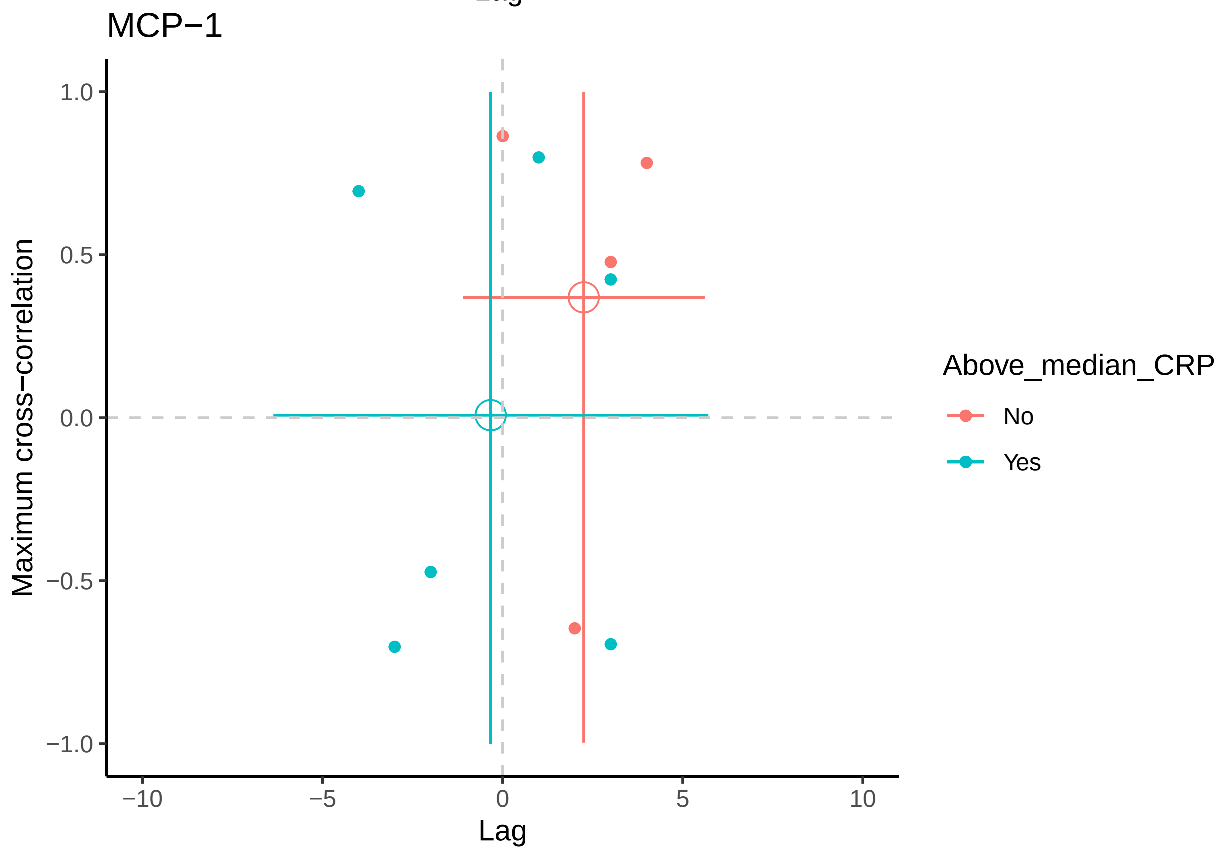
MCP−1
Maximum cross−correlation
1.0
0.5
0.0
−0.5
−1.0
−10
−5
0
5
10
Lag
Above_median_CRP
No
Yes

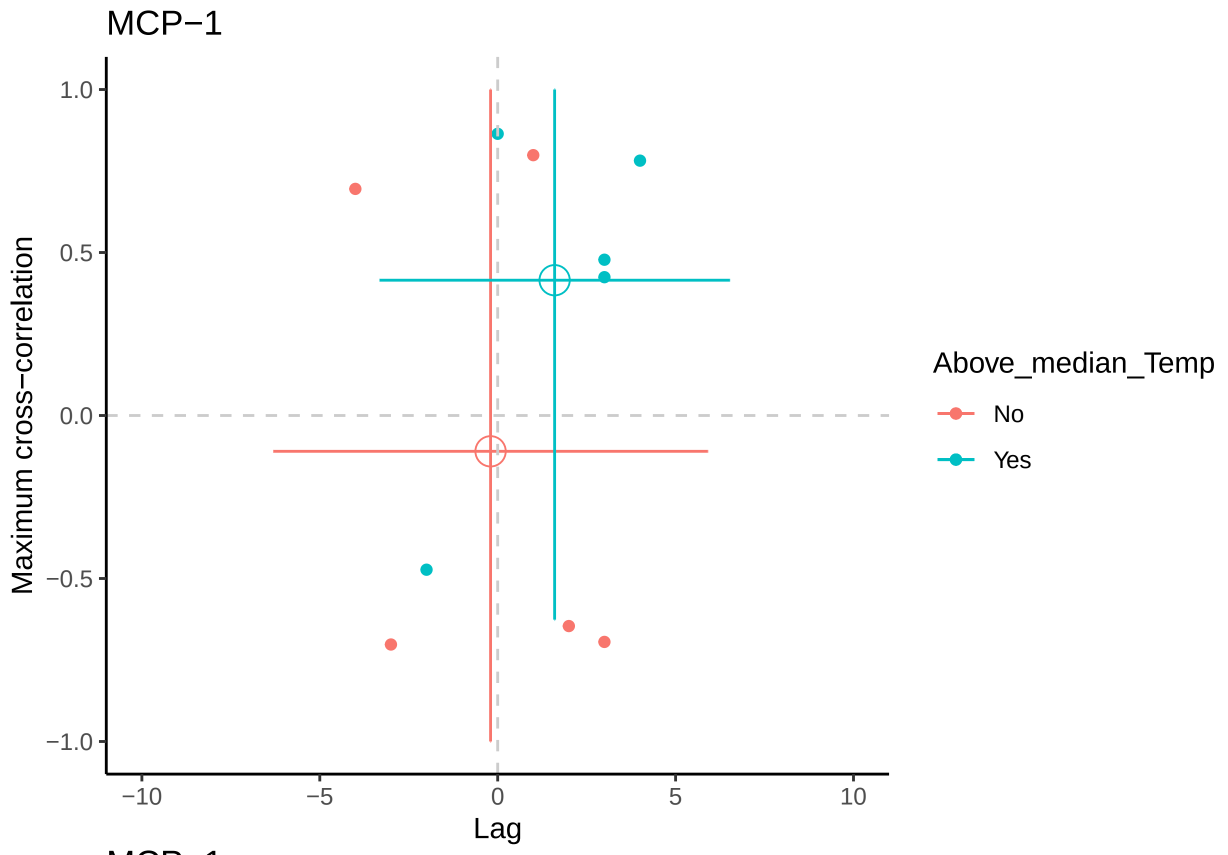
MCP−1
Maximum cross−correlation
Lag
1.0
0.5
0.0
−0.5
−1.0
−10
−5
0
5
10
Above_median_Temp
No
Yes

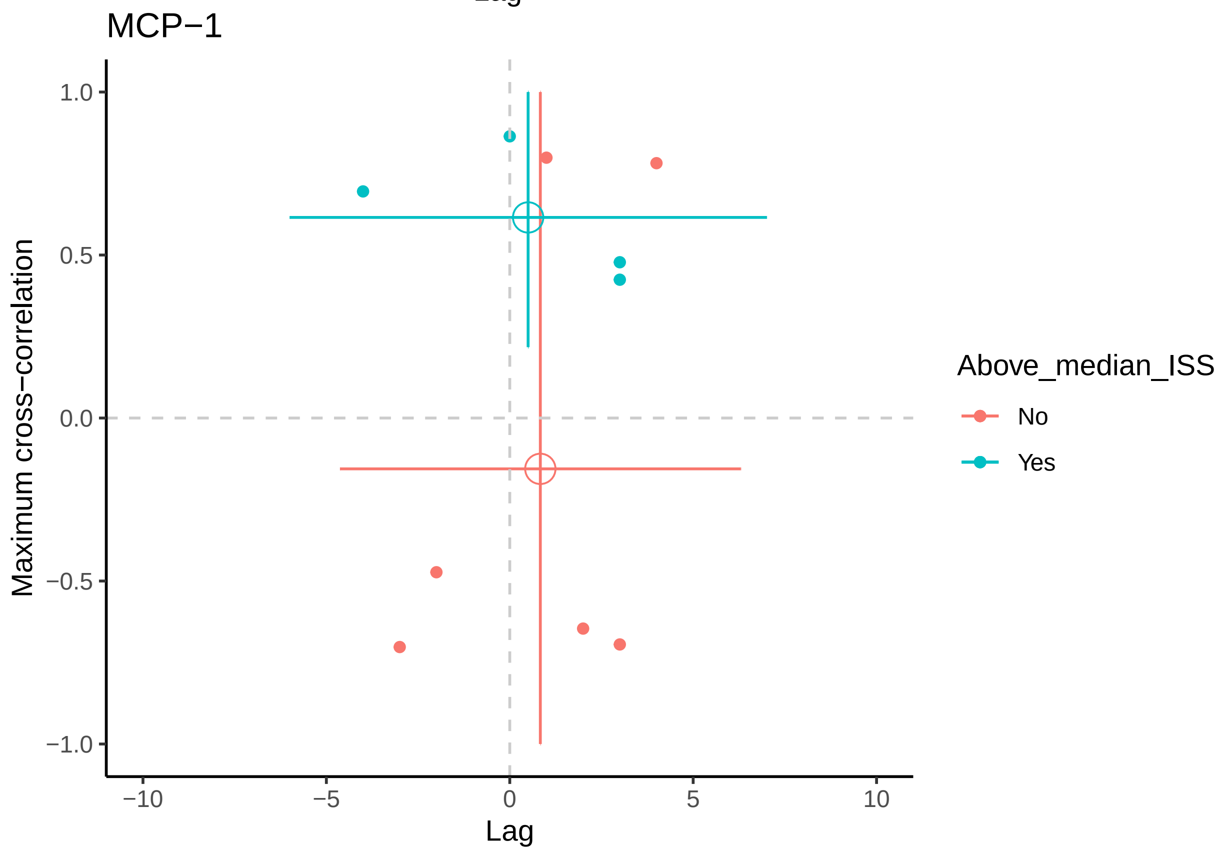
MCP−1
Maximum cross−correlation
Lag
1.0
0.5
0.0
−0.5
−1.0
−10
−5
0
5
10
Above_median_ISS
No
Yes

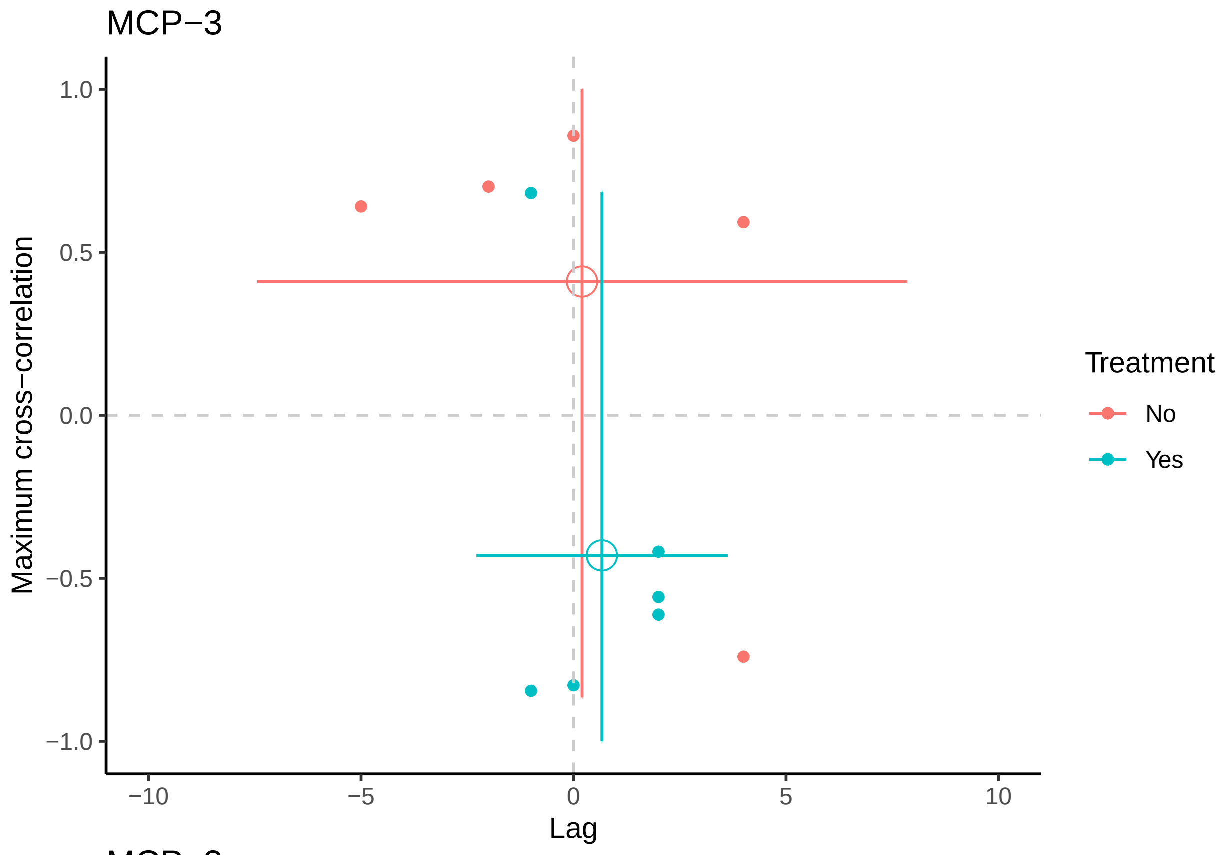
MCP−3
Maximum cross−correlation
1.0
0.5
0.0
−0.5
−1.0
−10
−5
0
5
10
Lag
Treatment
No
Yes

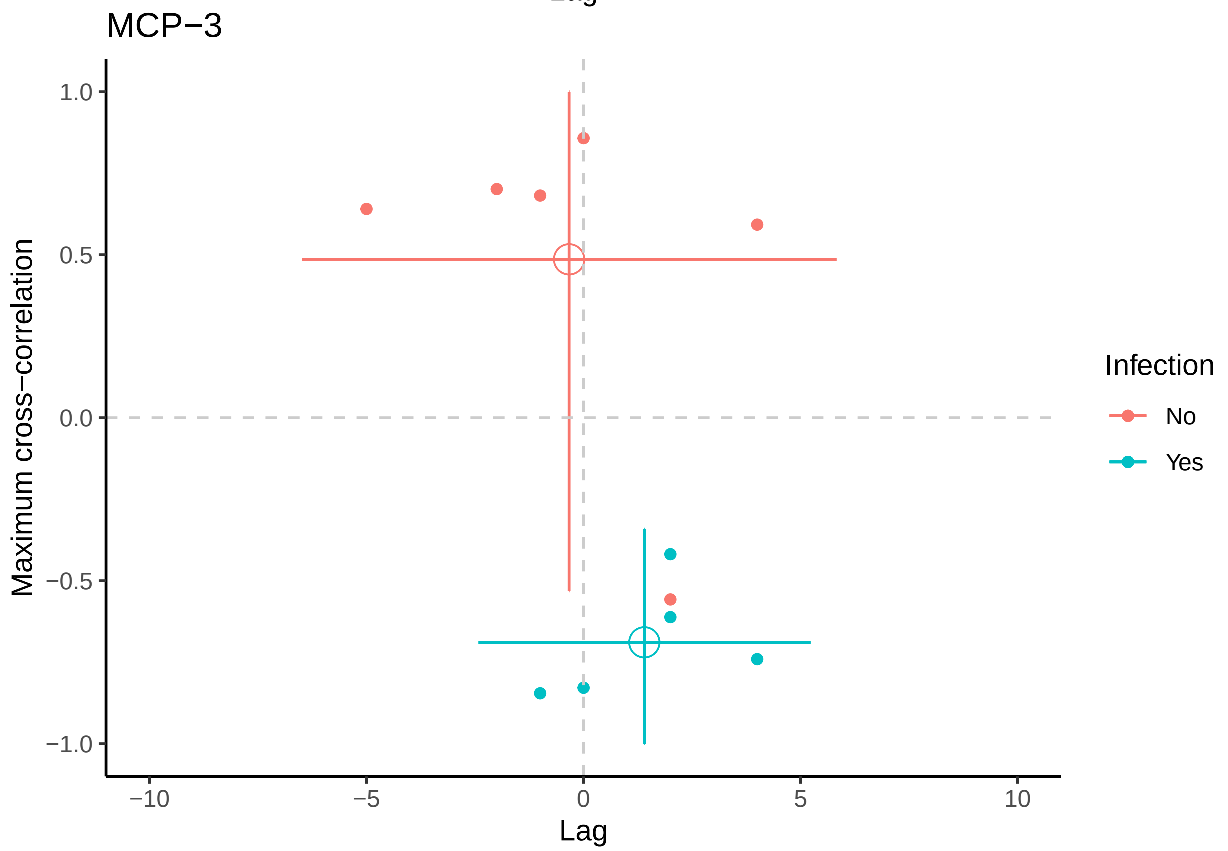
MCP−3
Maximum cross−correlation
1.0
0.5
0.0
−0.5
−1.0
−10
−5
0
5
10
Lag
Infection
No
Yes

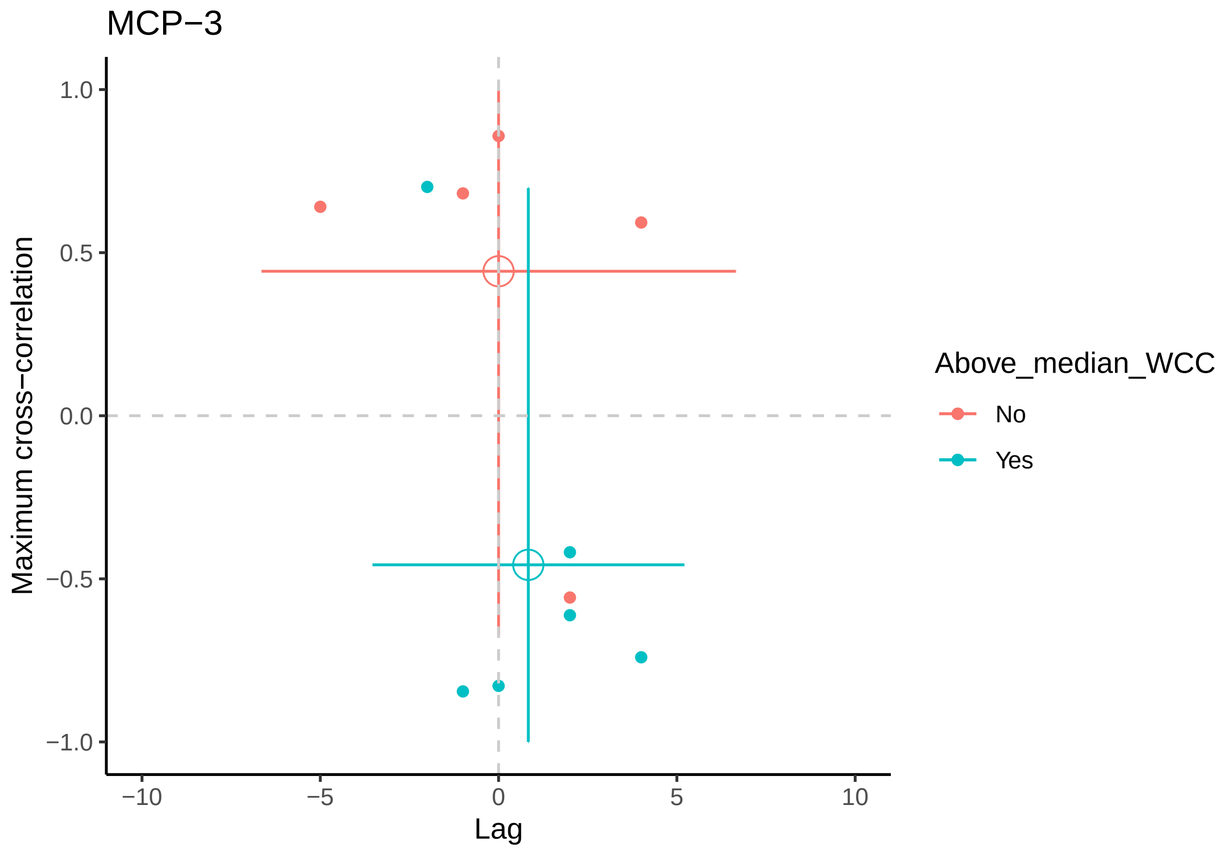
MCP−3
Maximum cross−correlation
1.0
0.5
0.0
−0.5
−1.0
−10
−5
0
5
10
Lag
Above_median_WCC
No
Yes

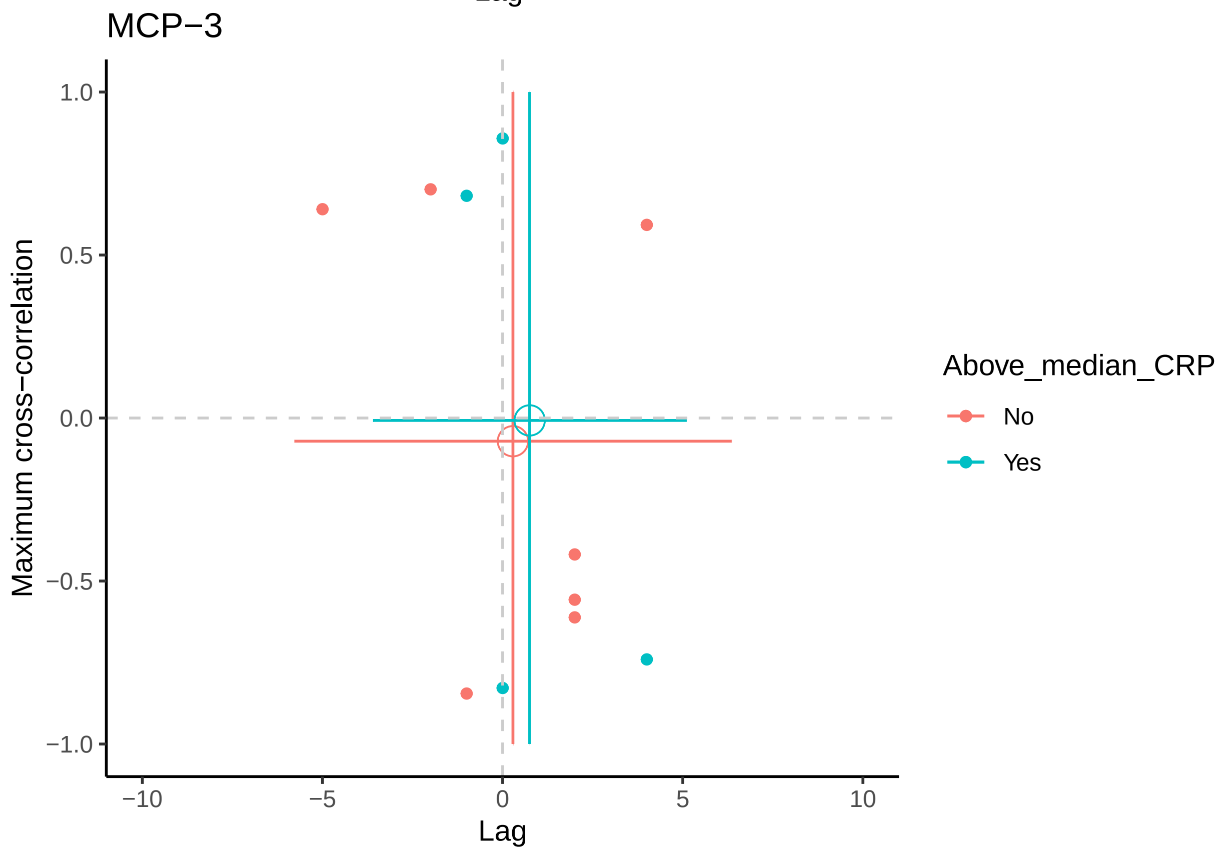
MCP−3
Maximum cross−correlation
1.0
0.5
0.0
−0.5
−1.0
−10
−5
0
5
10
Lag
Above_median_CRP
No
Yes

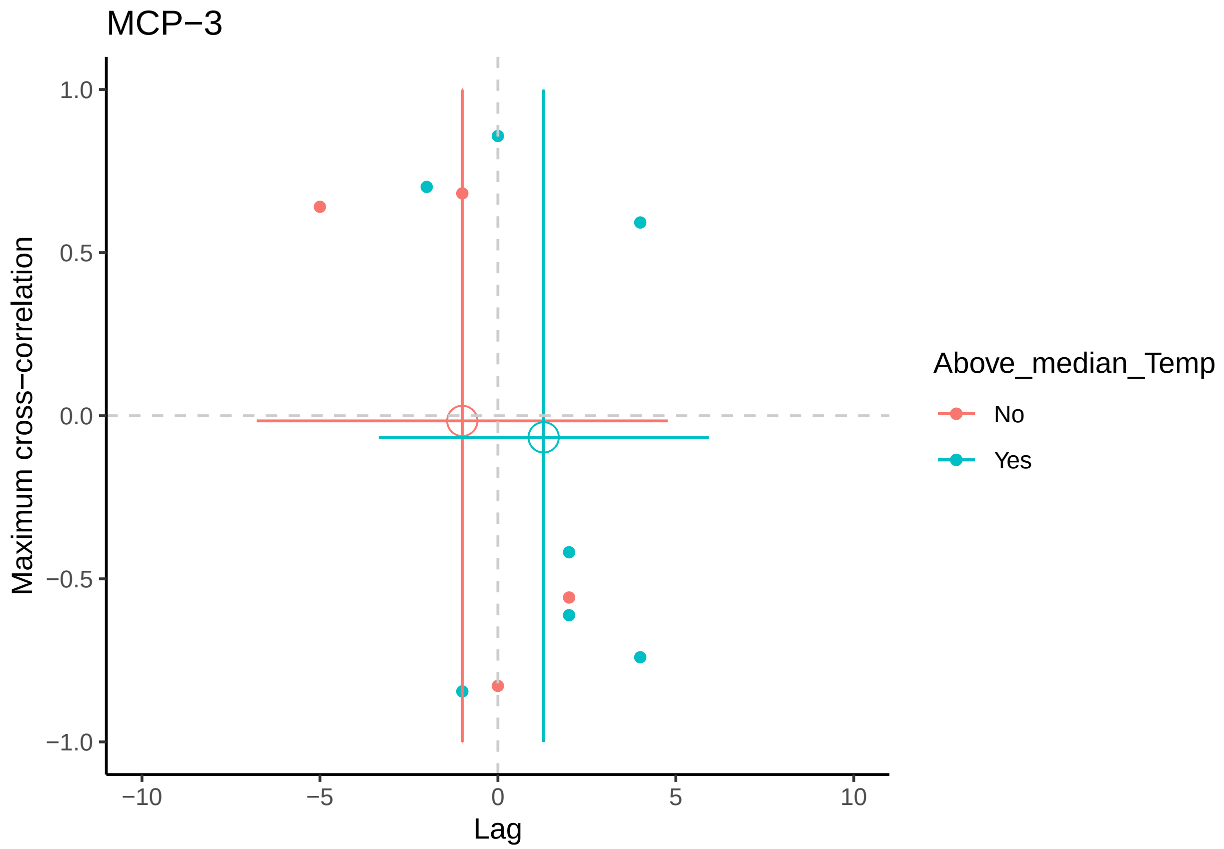
MCP−3
Maximum cross−correlation
1.0
0.5
0.0
−0.5
−1.0
−10
−5
0
5
10
Lag
Above_median_Temp
No
Yes

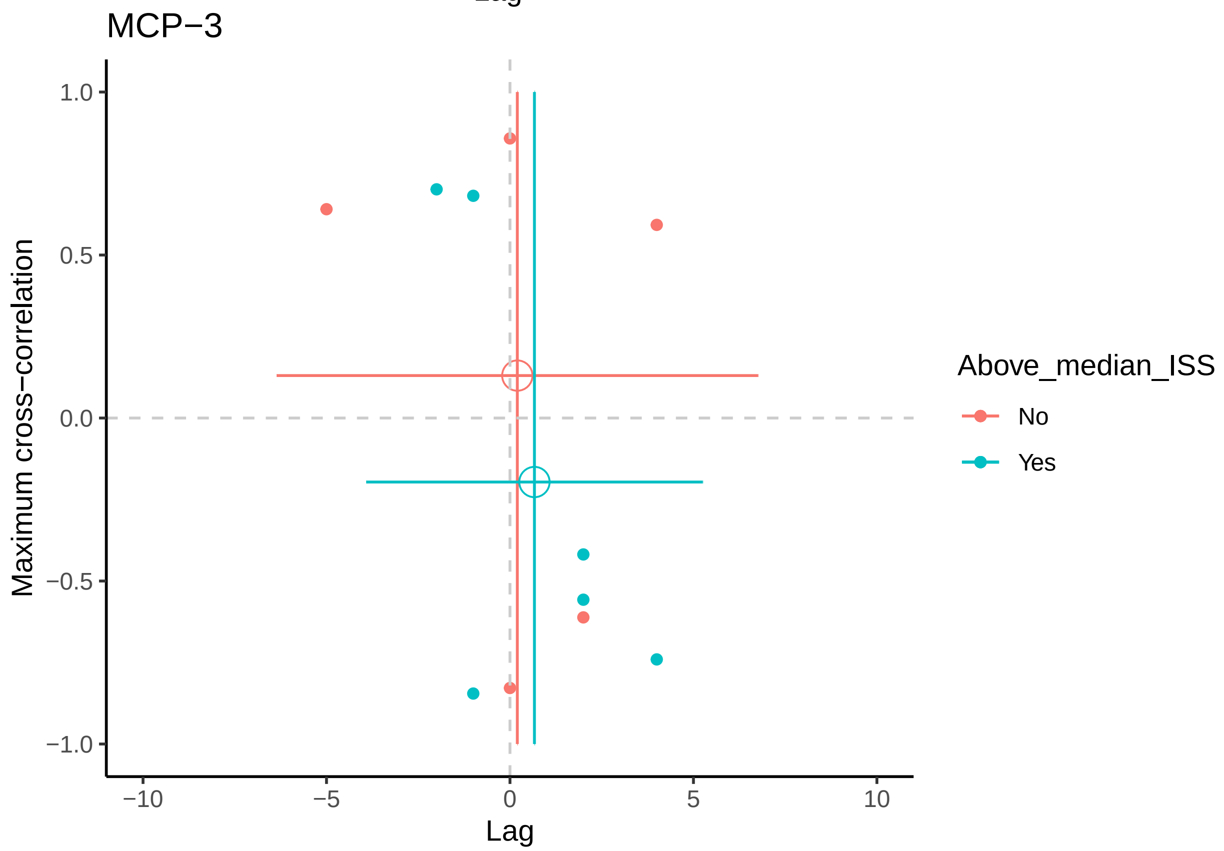
MCP−3
Maximum cross−correlation
1.0
0.5
0.0
−0.5
−1.0
−10
−5
0
5
10
Lag
Above_median_ISS
No
Yes

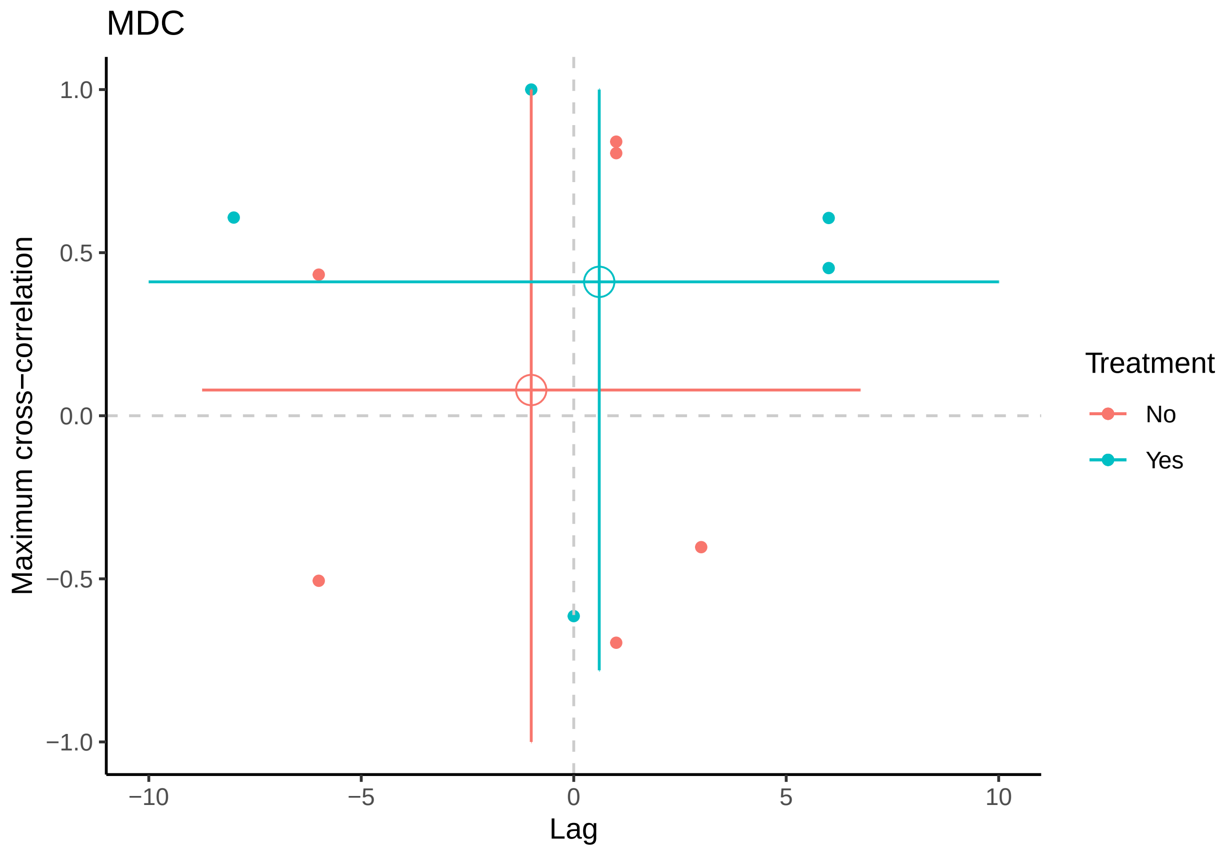

MDC
Maximum cross-correlation
Lag
Treatment
No
Yes

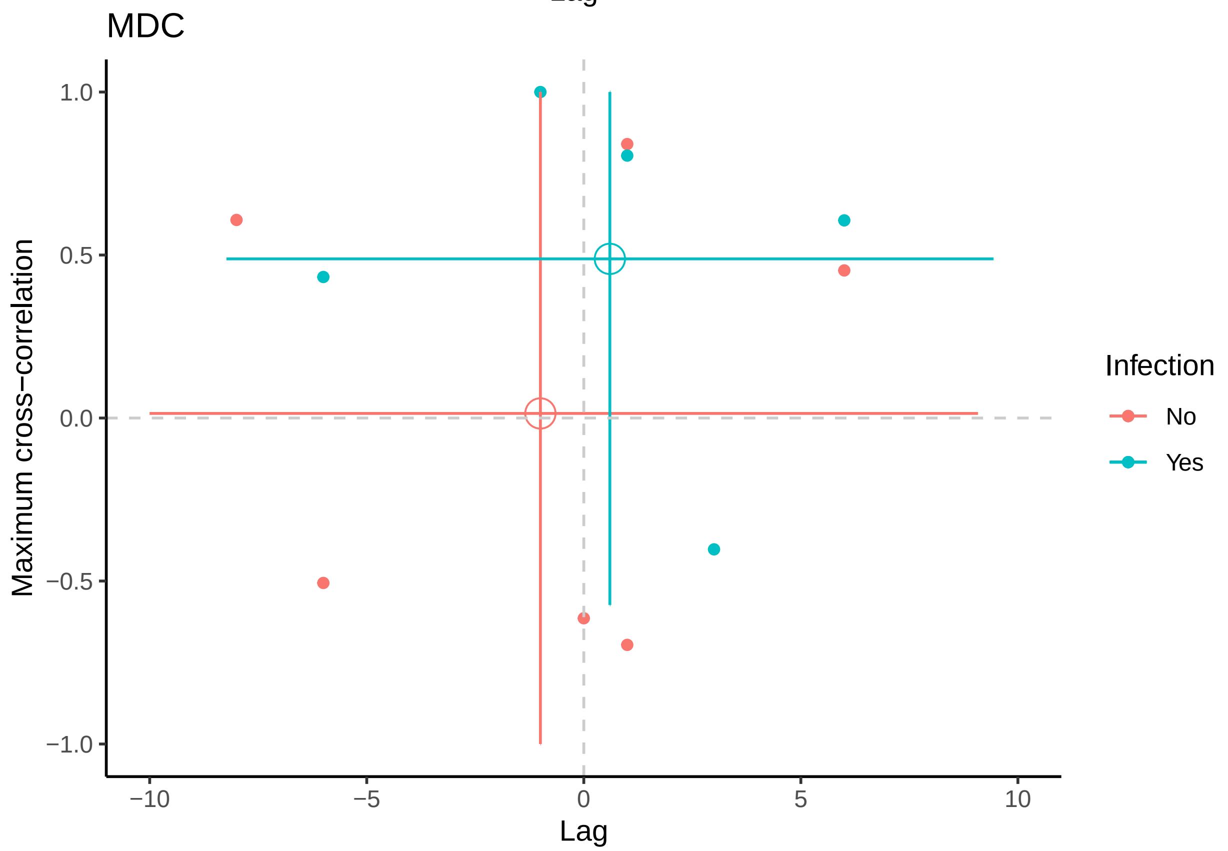

MDC
Maximum cross-correlation
Lag
Infection
No
Yes

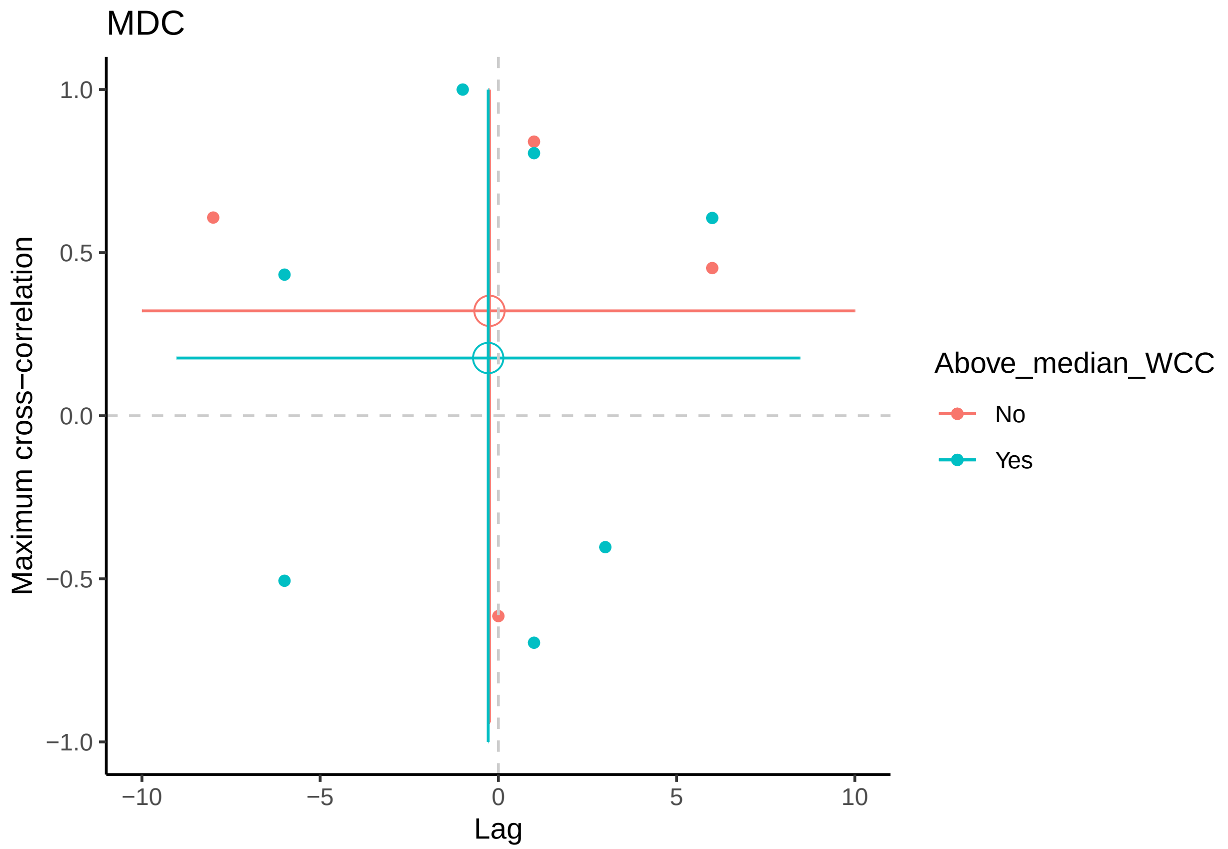
MDC
1.0
0.5
0.0
−0.5
−1.0
Maximum cross−correlation
−10
−5
0
5
10
Lag
Above_median_WCC
No
Yes

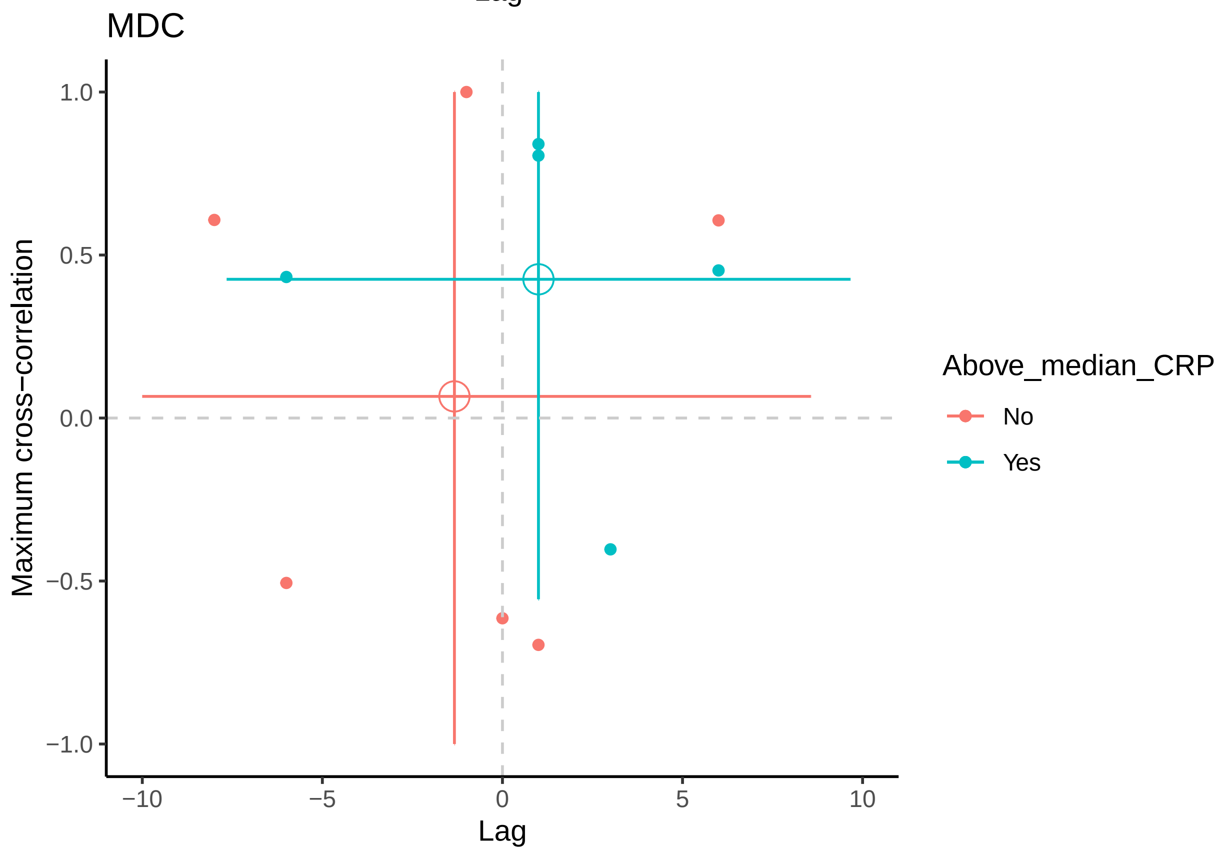
MDC
1.0
0.5
0.0
−0.5
−1.0
Maximum cross−correlation
−10
−5
0
5
10
Lag
Above_median_CRP
No
Yes

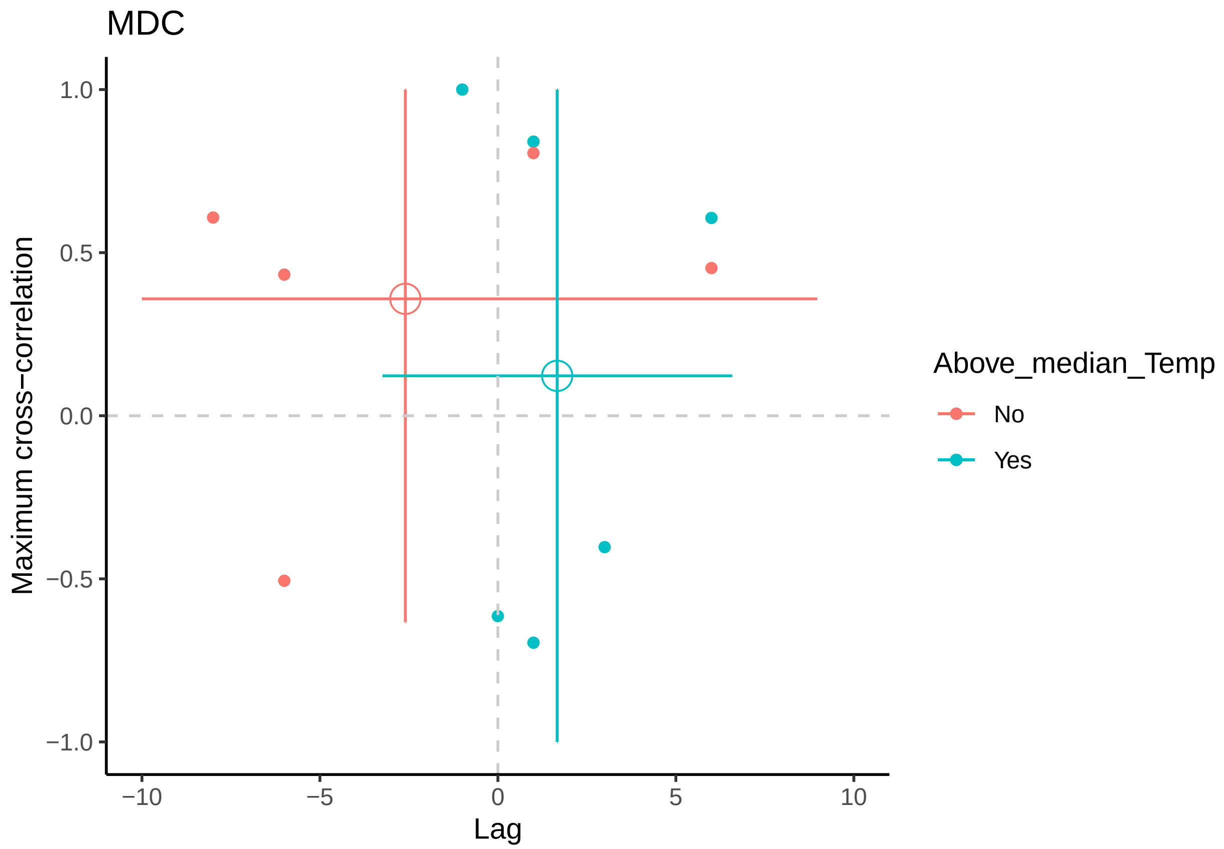
MDC
Maximum cross−correlation
Lag
Above_median_Temp
No
Yes

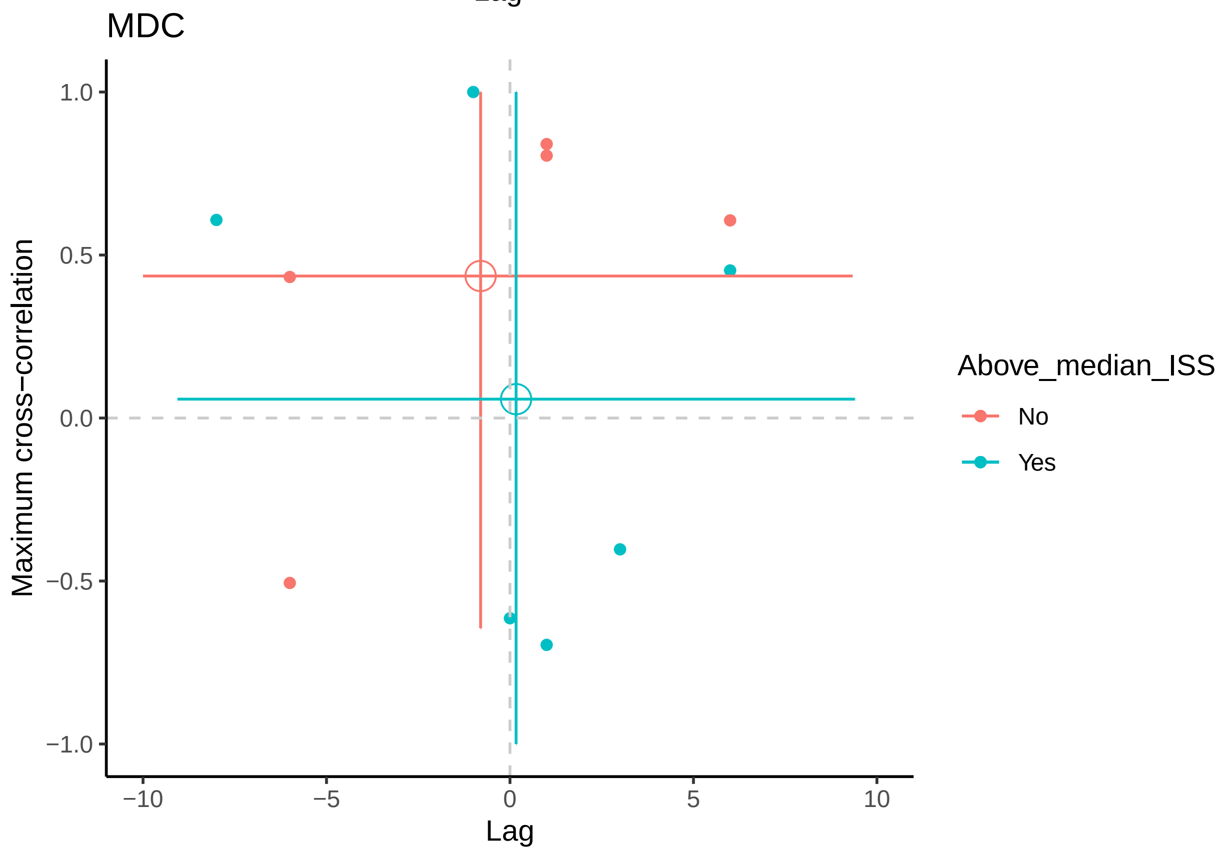
MDC
Maximum cross−correlation
Lag
Above_median_ISS
No
Yes

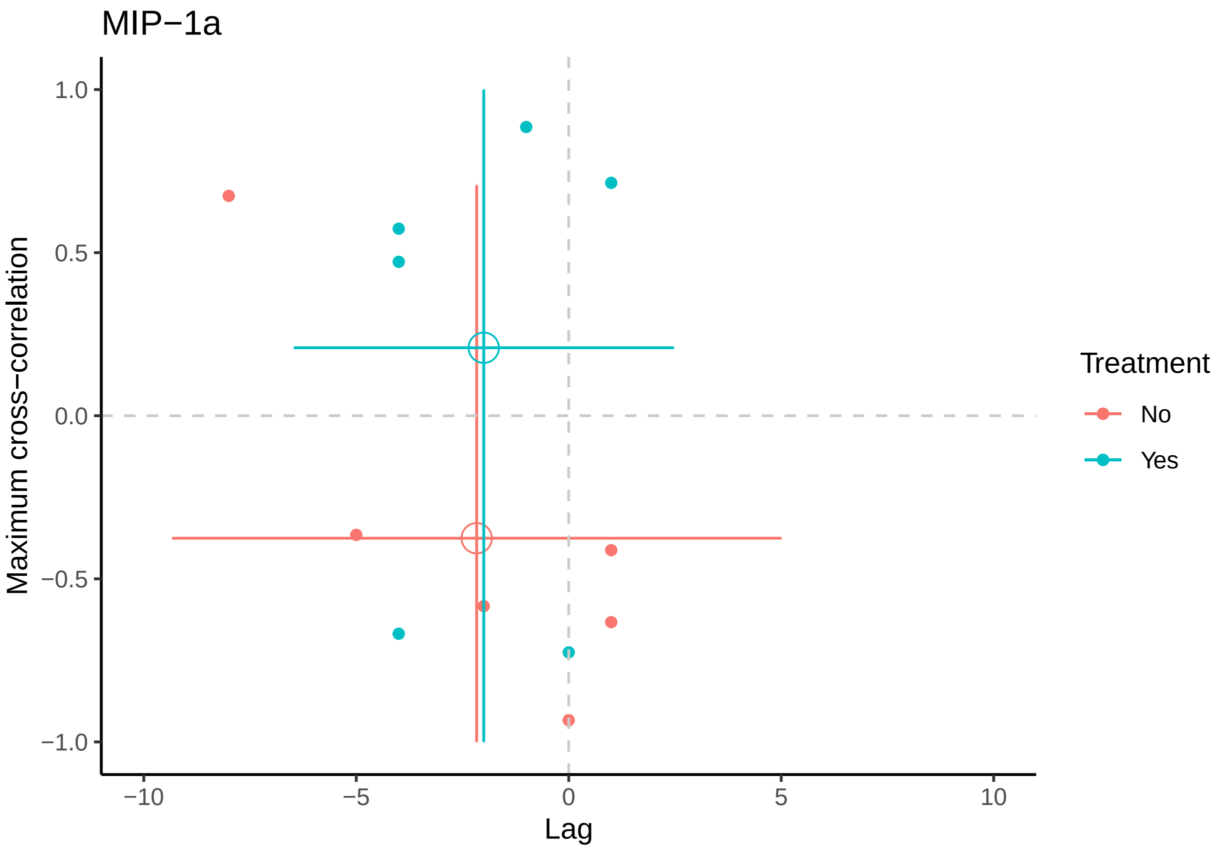
MIP−1a
Maximum cross−correlation
1.0
0.5
0.0
−0.5
−1.0
−10
−5
0
5
10
Lag
Treatment
No
Yes

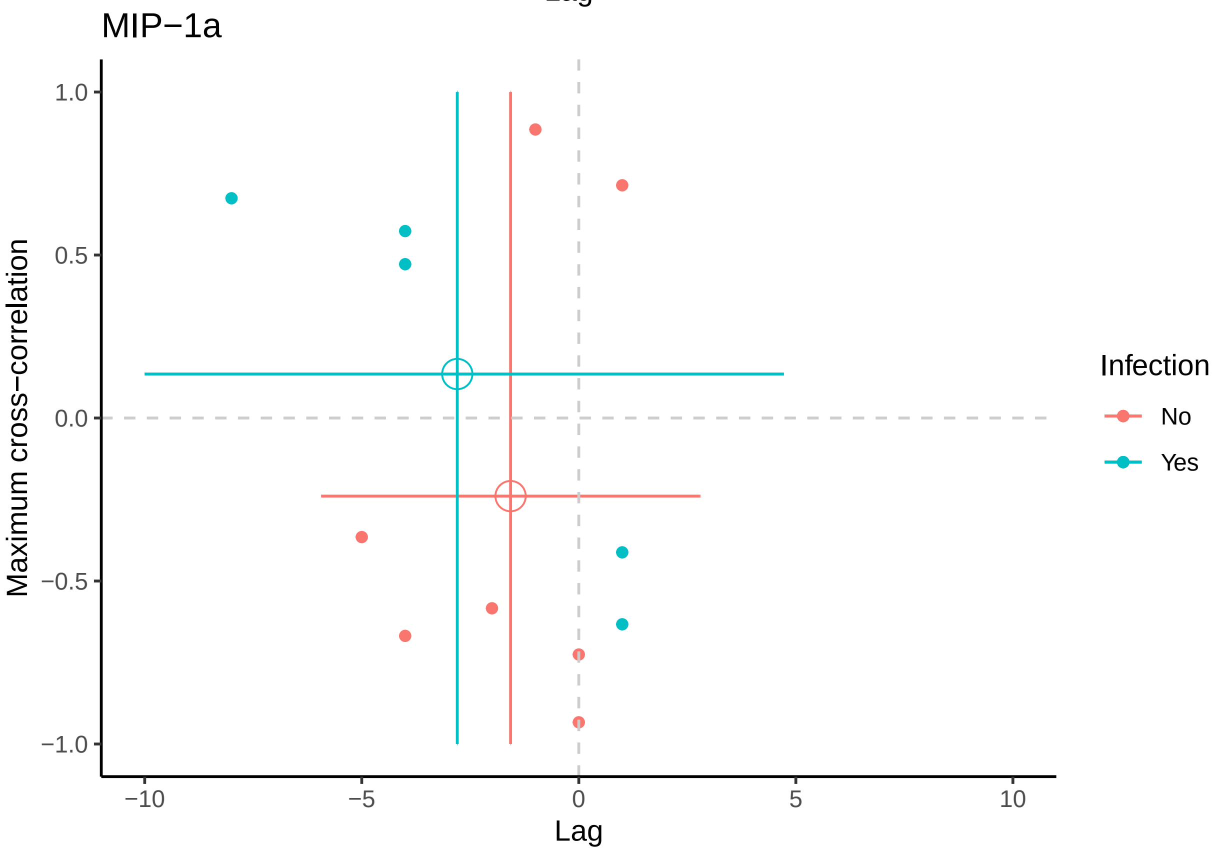
MIP−1a
Maximum cross−correlation
1.0
0.5
0.0
−0.5
−1.0
−10
−5
0
5
10
Lag
Infection
No
Yes

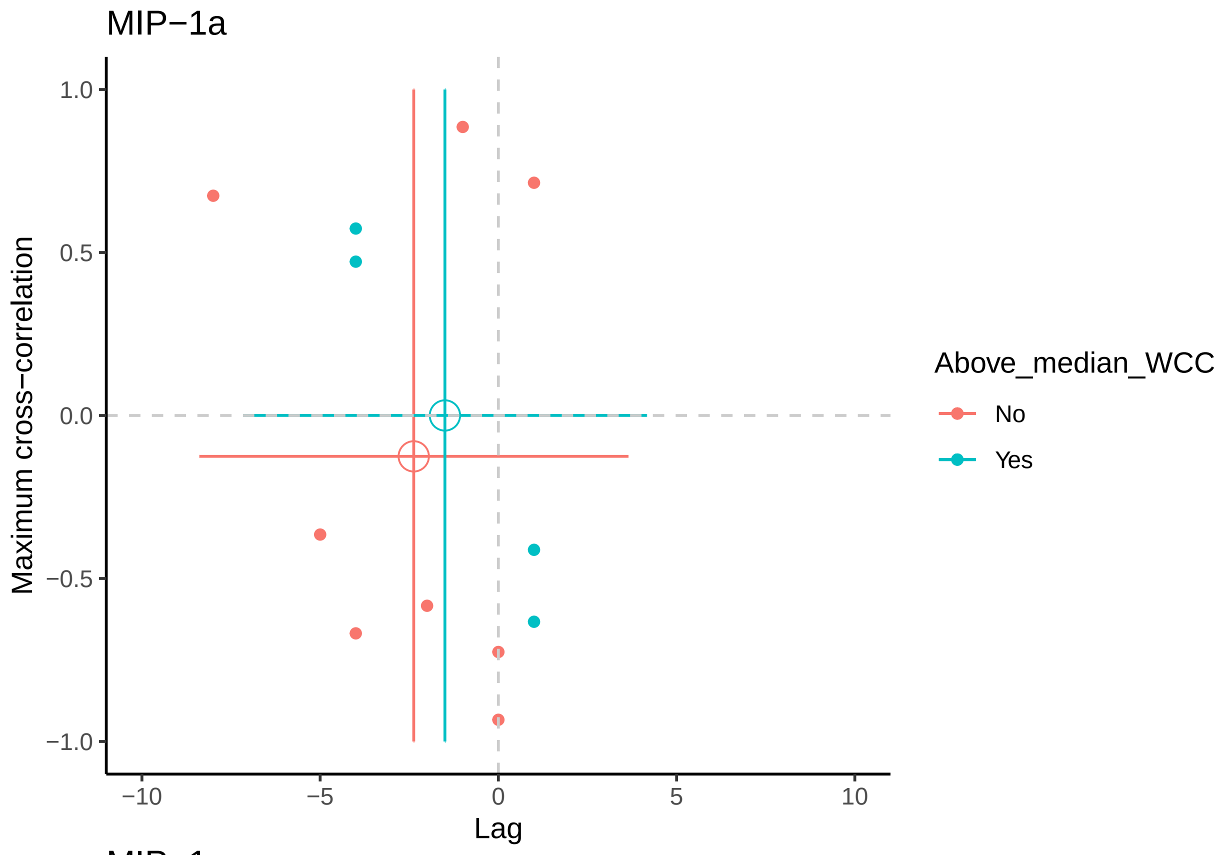
MIP−1a
Maximum cross−correlation
1.0
0.5
0.0
−0.5
−1.0
−10
−5
0
5
10
Lag
Above_median_WCC
No
Yes

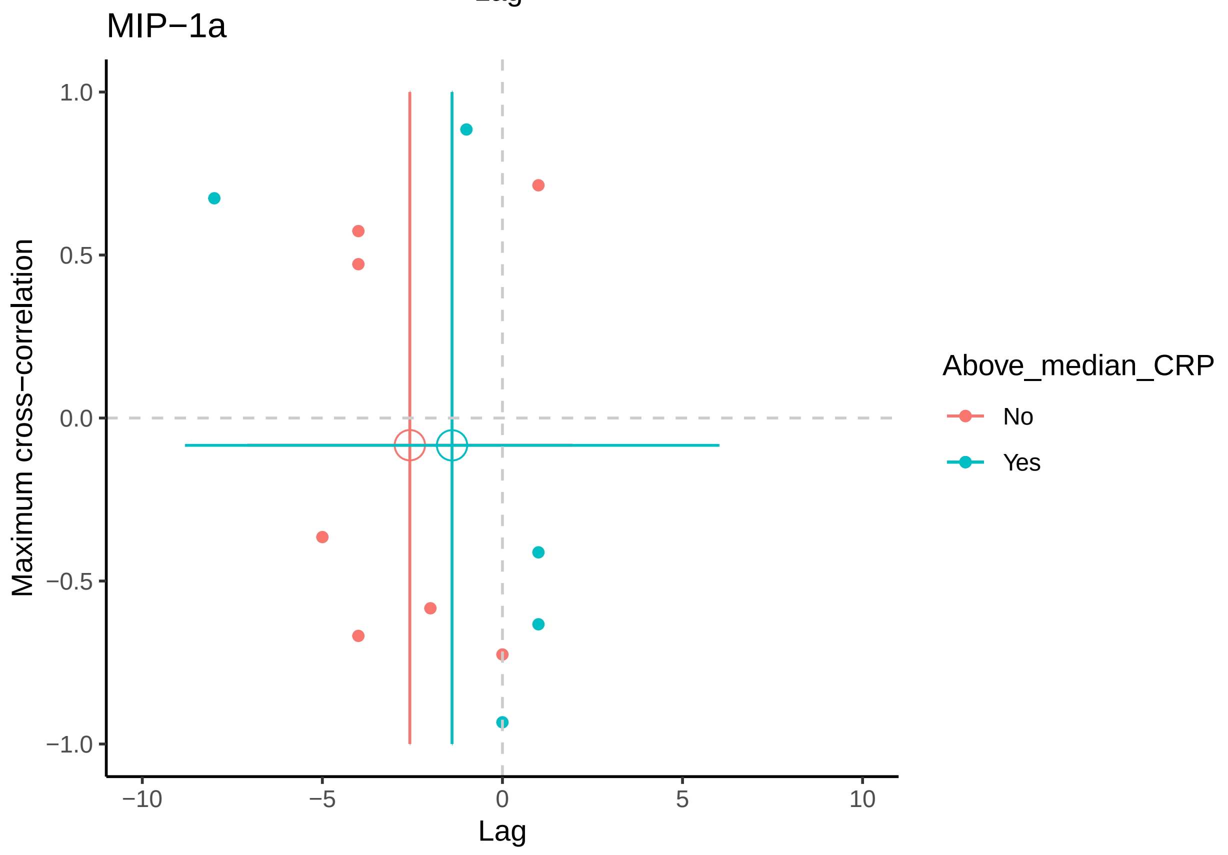
MIP−1a
Maximum cross−correlation
1.0
0.5
0.0
−0.5
−1.0
−10
−5
0
5
10
Lag
Above_median_CRP
No
Yes

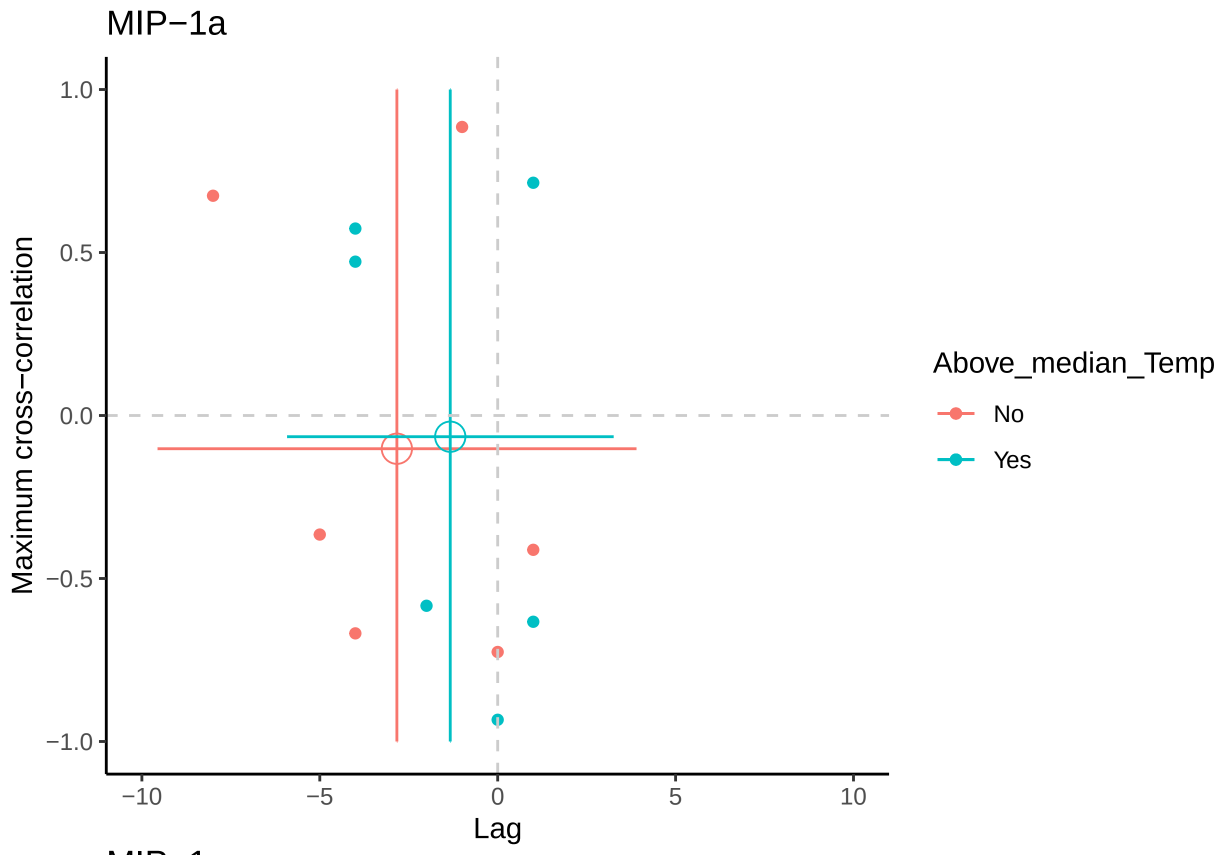
MIP−1a
Maximum cross−correlation
1.0
0.5
0.0
−0.5
−1.0
−10
−5
0
5
10
Lag
Above_median_Temp
No
Yes

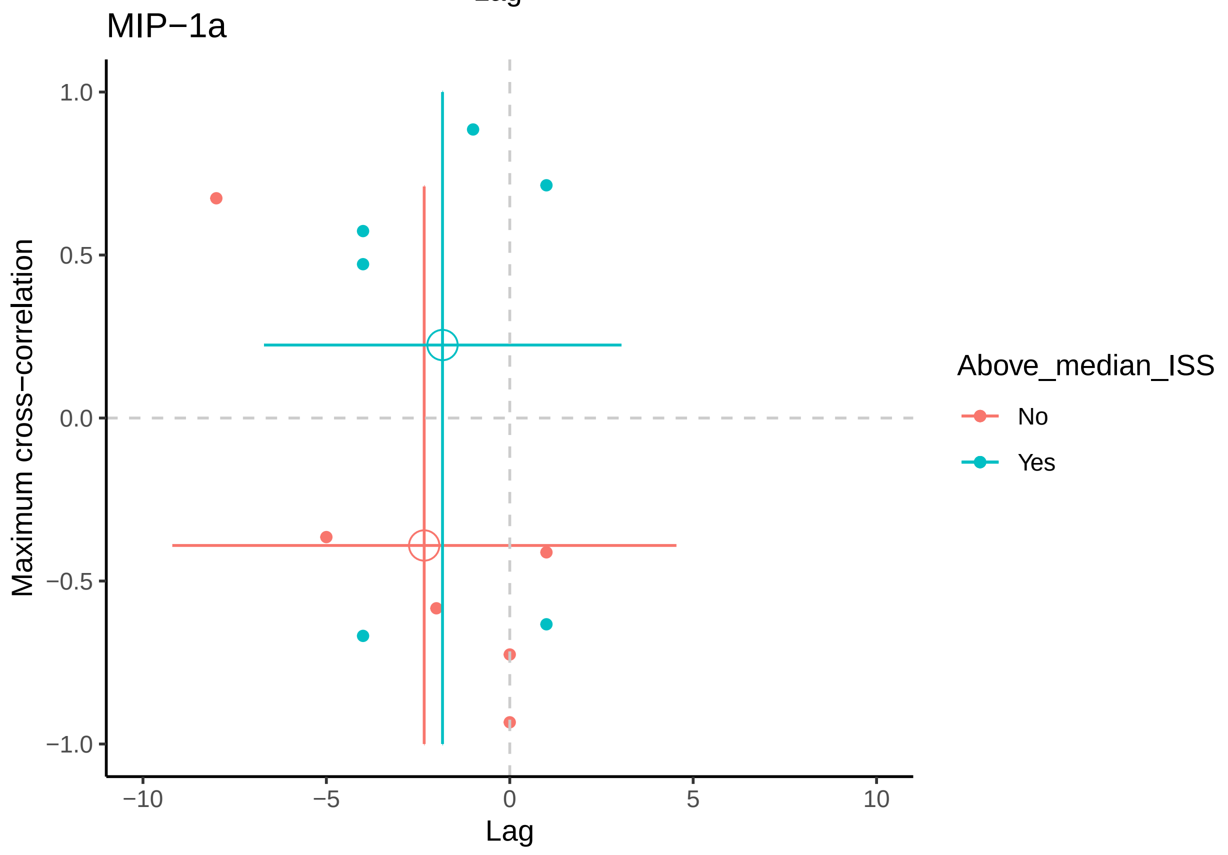
MIP−1a
Maximum cross−correlation
1.0
0.5
0.0
−0.5
−1.0
−10
−5
0
5
10
Lag
Above_median_ISS
No
Yes

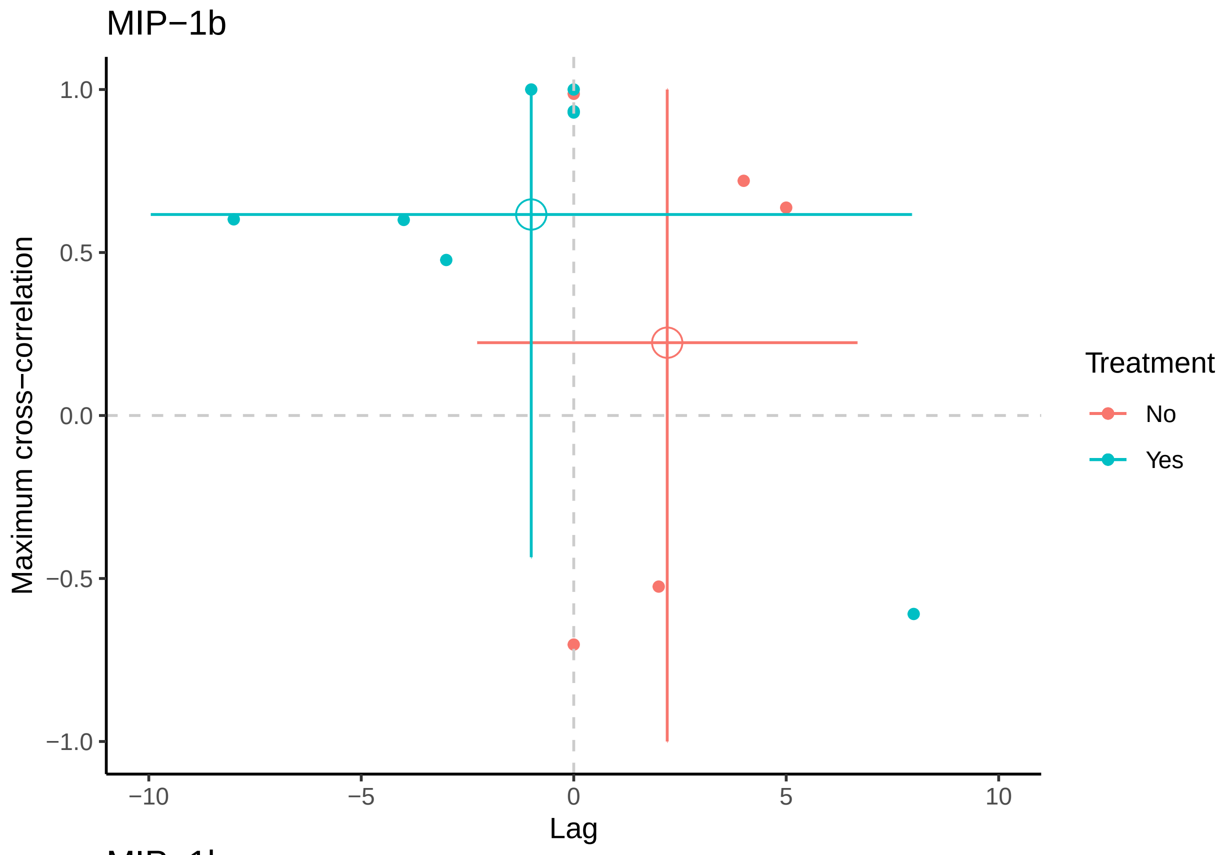
MIP−1b
Maximum cross−correlation
1.0
0.5
0.0
−0.5
−1.0
−10
−5
0
5
10
Lag
Treatment
No
Yes

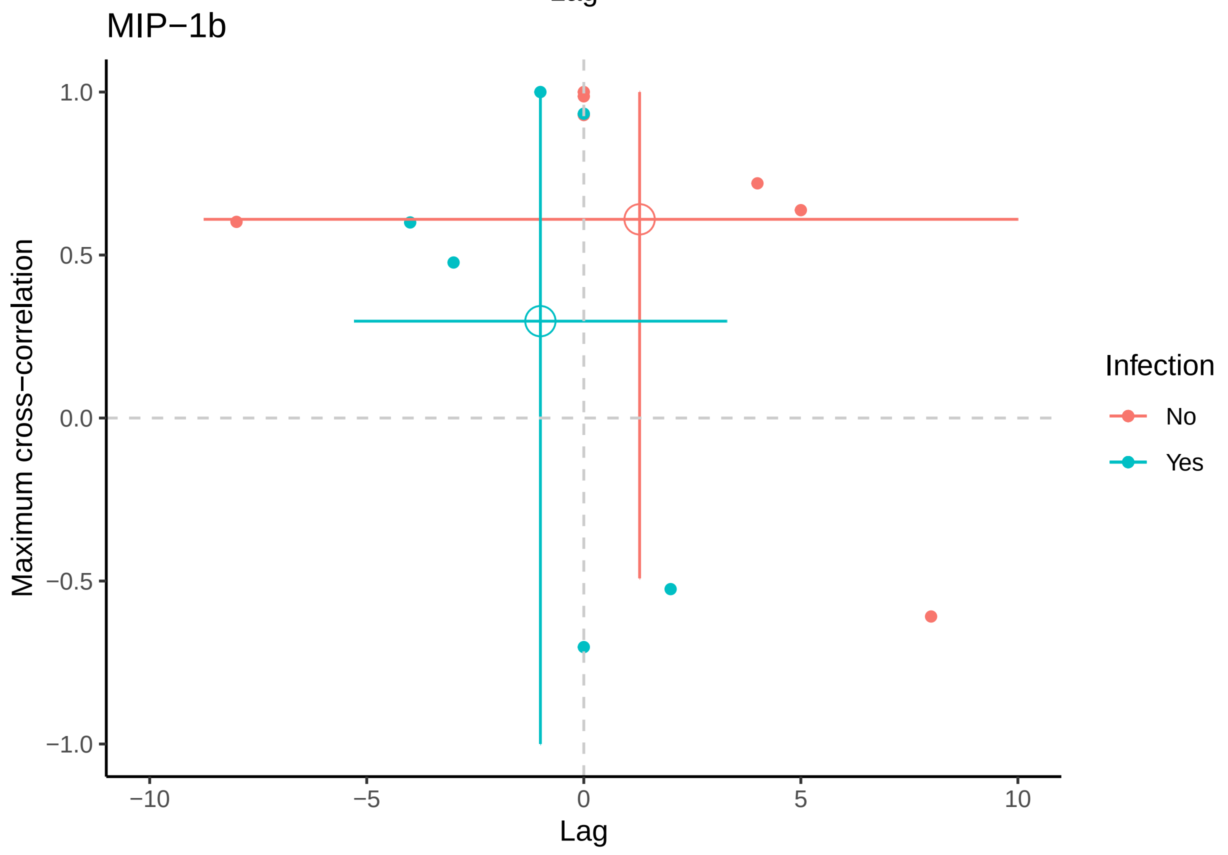
MIP−1b
Maximum cross−correlation
1.0
0.5
0.0
−0.5
−1.0
−10
−5
0
5
10
Lag
Infection
No
Yes

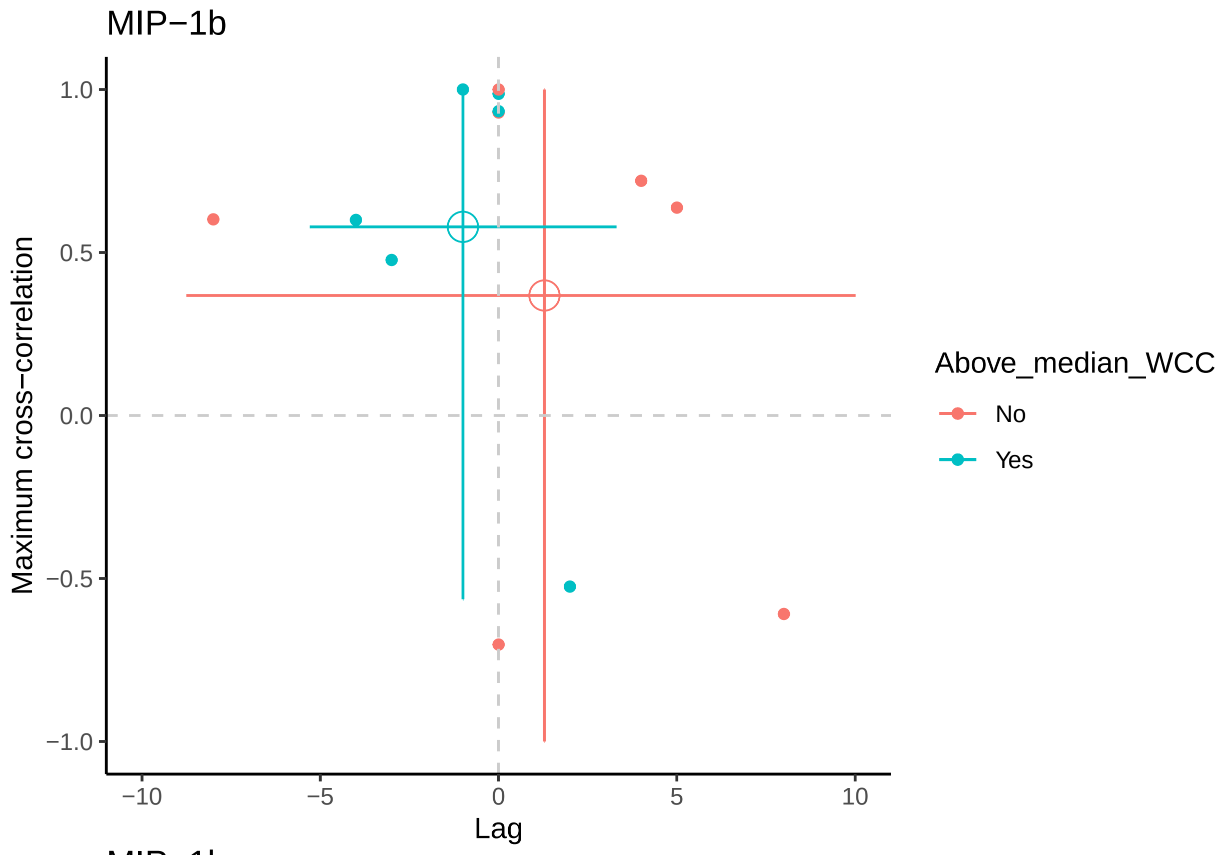
MIP−1b
Maximum cross−correlation
1.0
0.5
0.0
−0.5
−1.0
−10
−5
0
5
10
Lag
Above_median_WCC
No
Yes

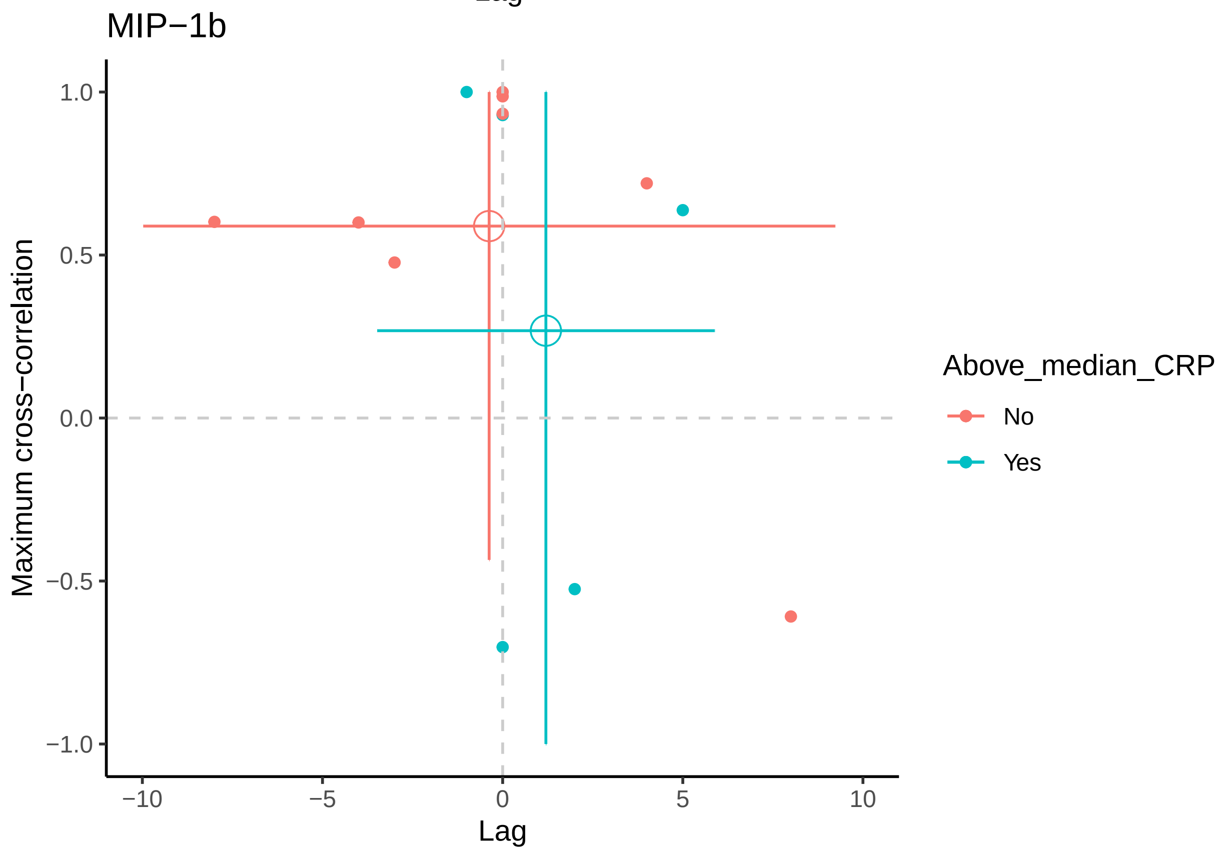
MIP−1b
Maximum cross−correlation
1.0
0.5
0.0
−0.5
−1.0
−10
−5
0
5
10
Lag
Above_median_CRP
No
Yes

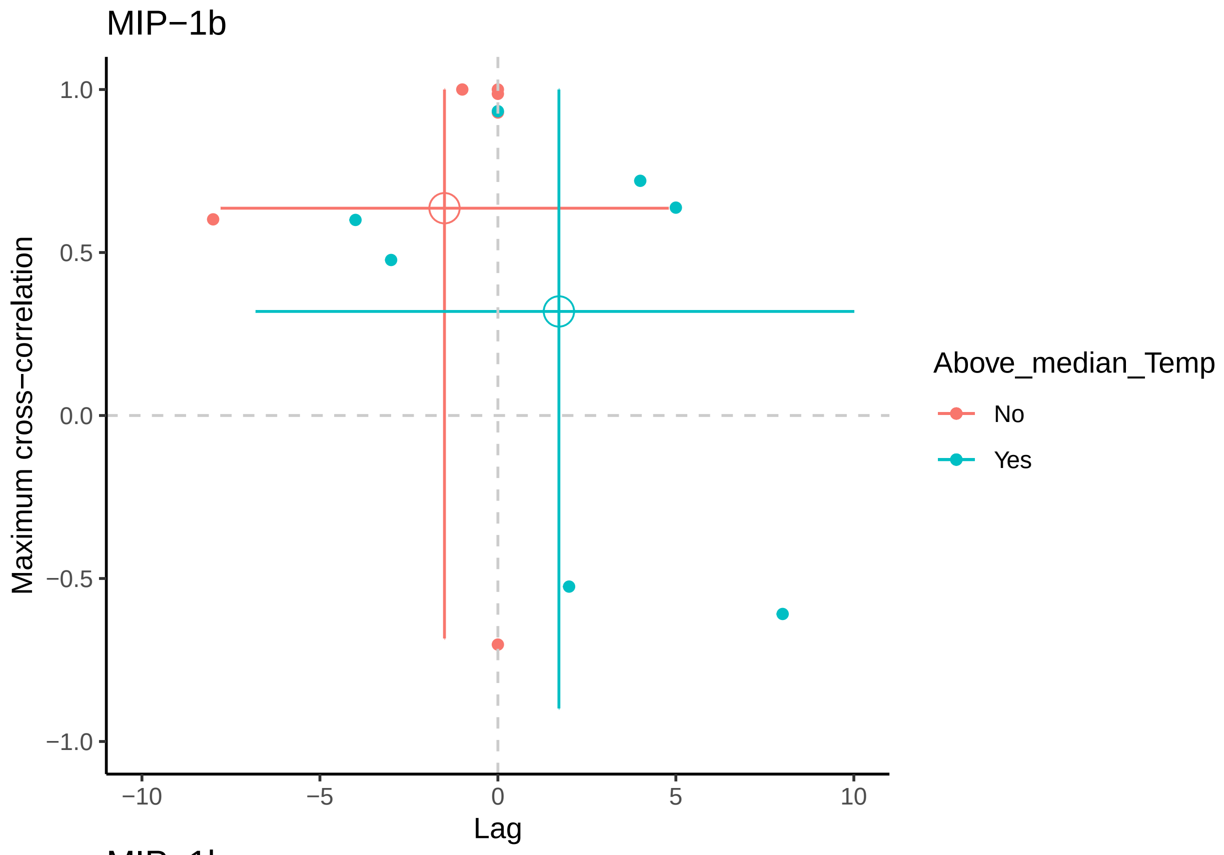
MIP−1b
Maximum cross−correlation
1.0
0.5
0.0
−0.5
−1.0
−10
−5
0
5
10
Lag
Above_median_Temp
No
Yes

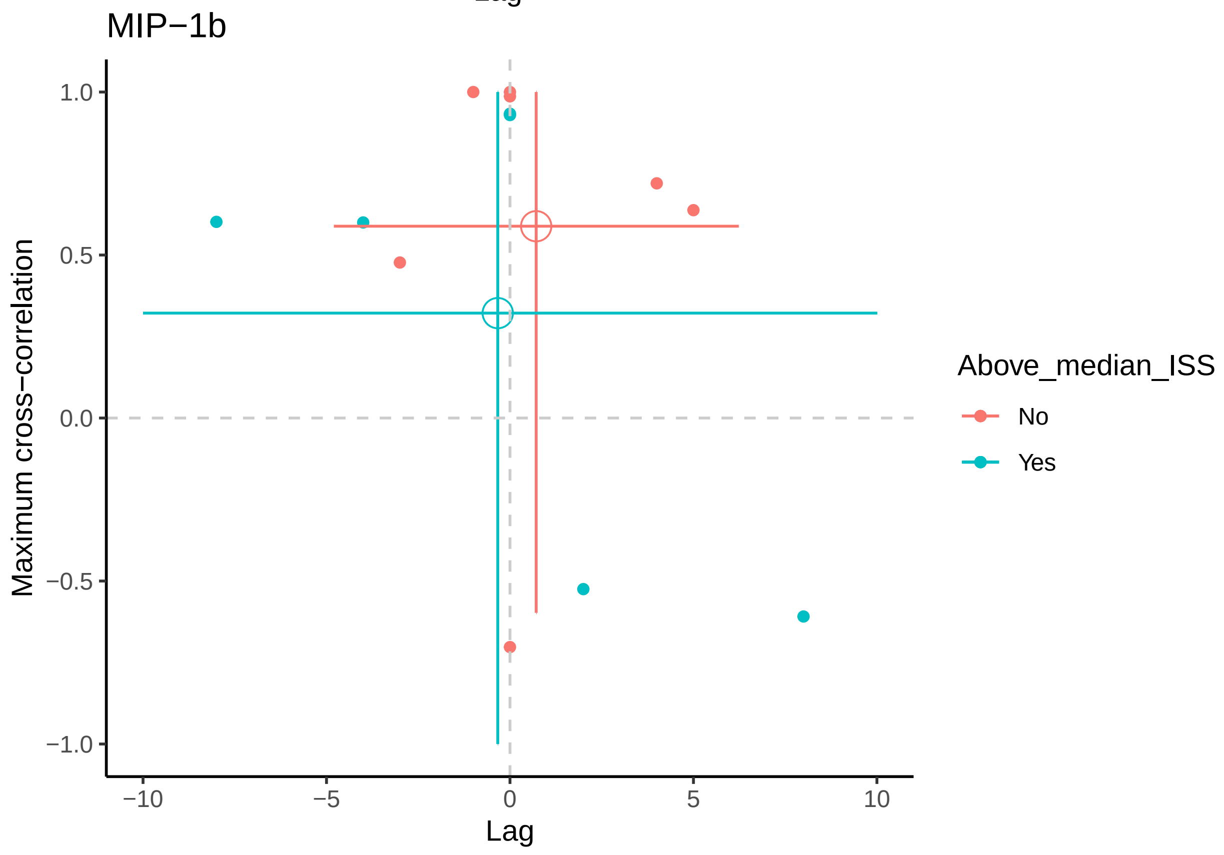
MIP−1b
Maximum cross−correlation
1.0
0.5
0.0
−0.5
−1.0
−10
−5
0
5
10
Lag
Above_median_ISS
No
Yes

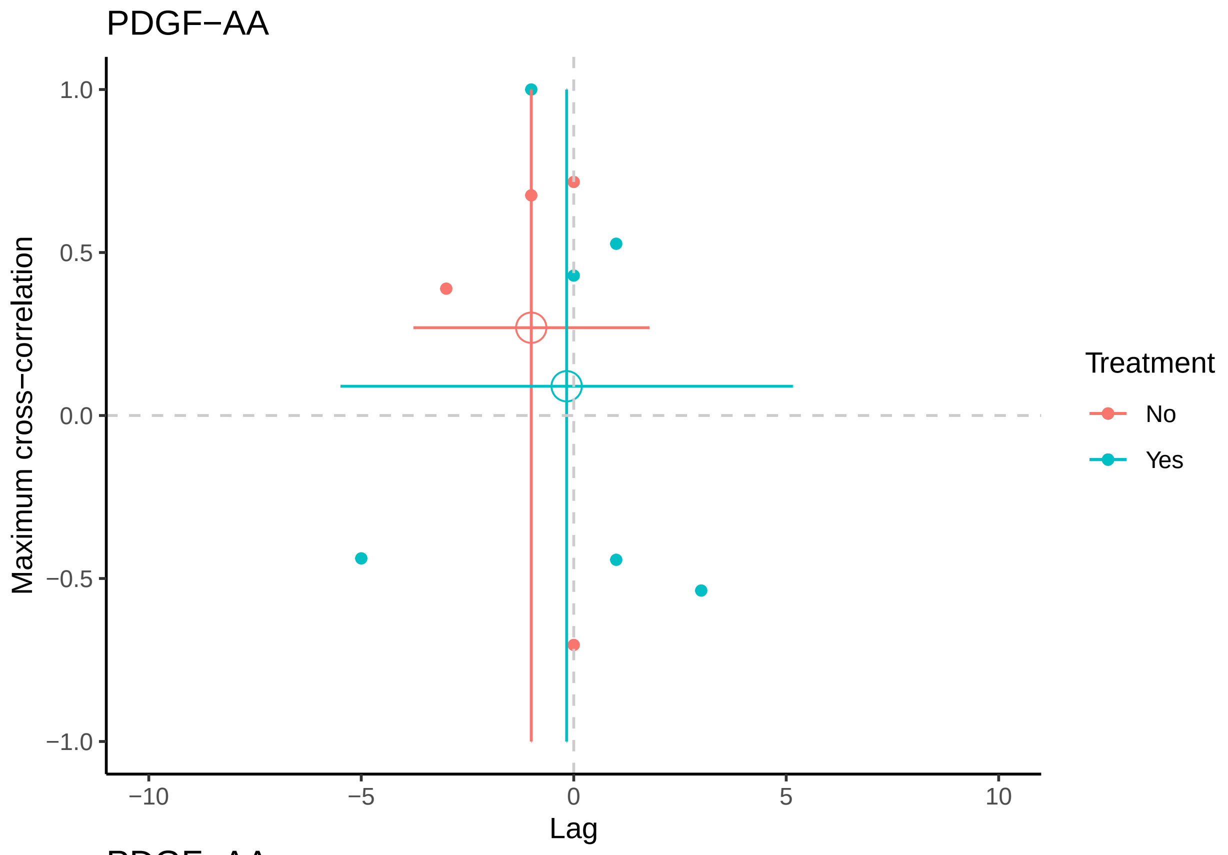
PDGF−AA
Maximum cross−correlation
1.0
0.5
0.0
−0.5
−1.0
−10
−5
0
5
10
Lag
Treatment
No
Yes

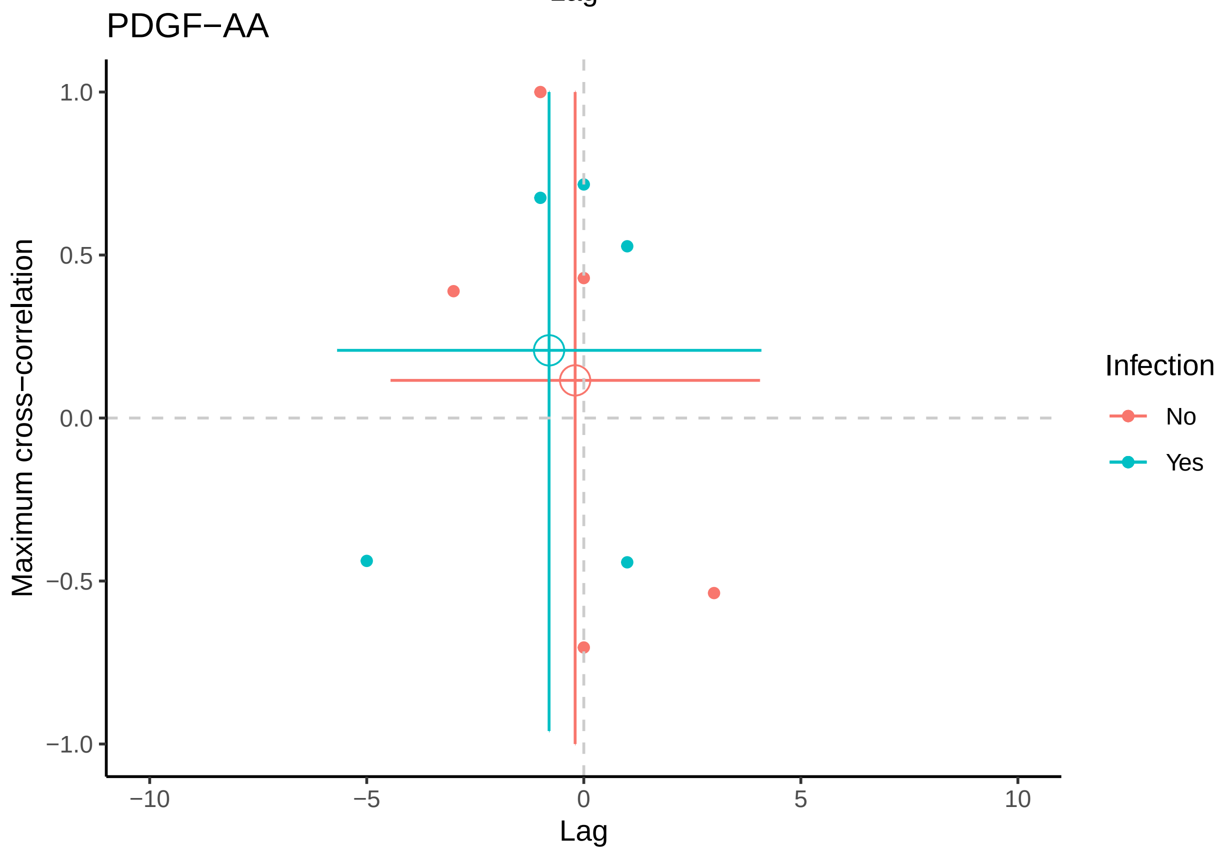
PDGF−AA
Maximum cross−correlation
1.0
0.5
0.0
−0.5
−1.0
−10
−5
0
5
10
Lag
Infection
No
Yes

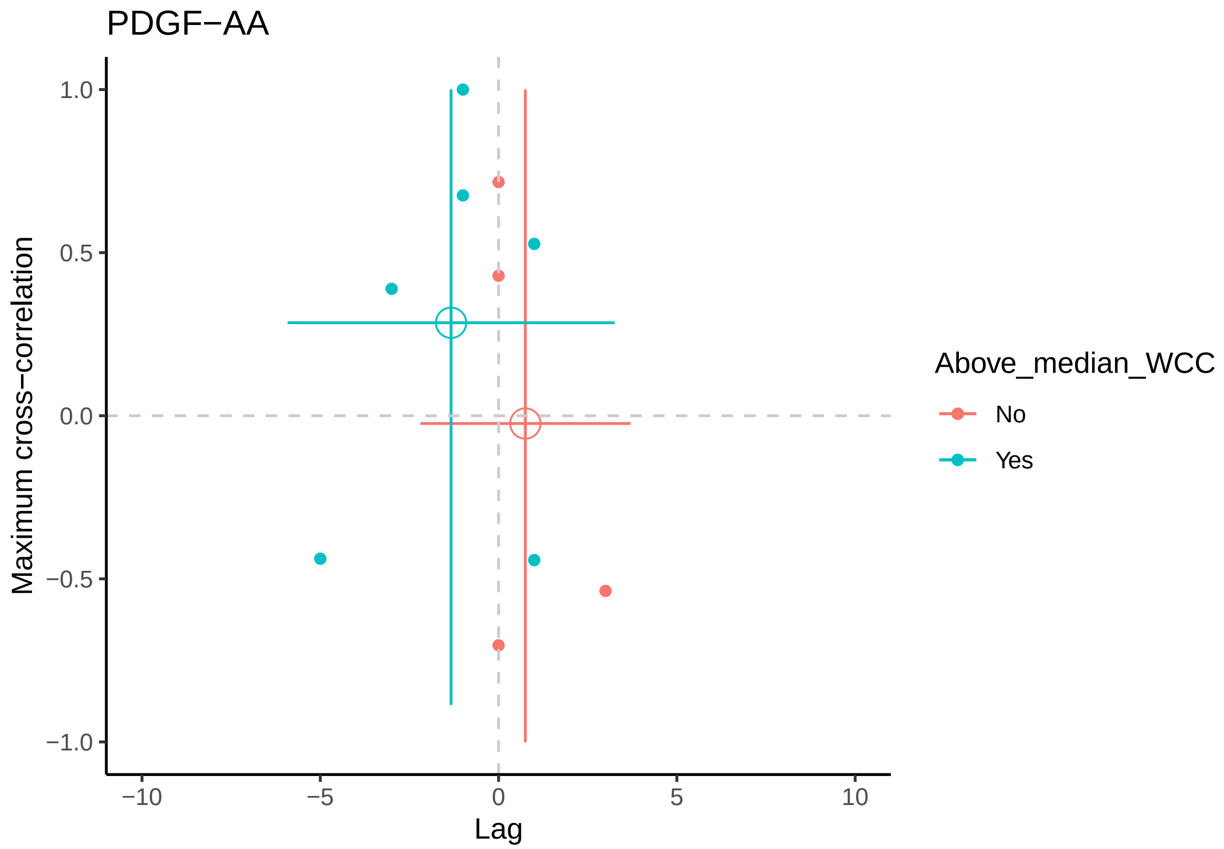
PDGF−AA
Maximum cross−correlation
Lag
1.0
0.5
0.0
−0.5
−1.0
−10
−5
0
5
10
Above_median_WCC
No
Yes

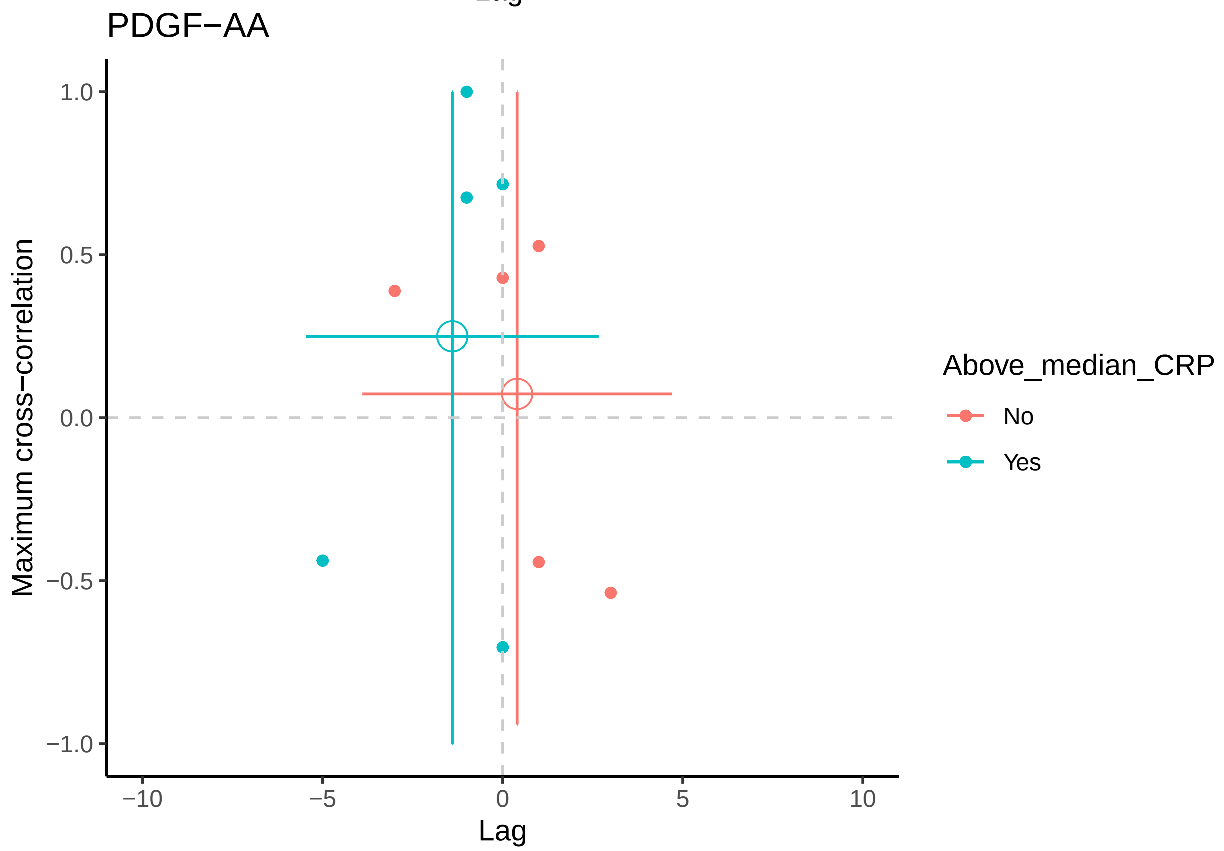
PDGF−AA
Maximum cross−correlation
Lag
1.0
0.5
0.0
−0.5
−1.0
−10
−5
0
5
10
Above_median_CRP
No
Yes

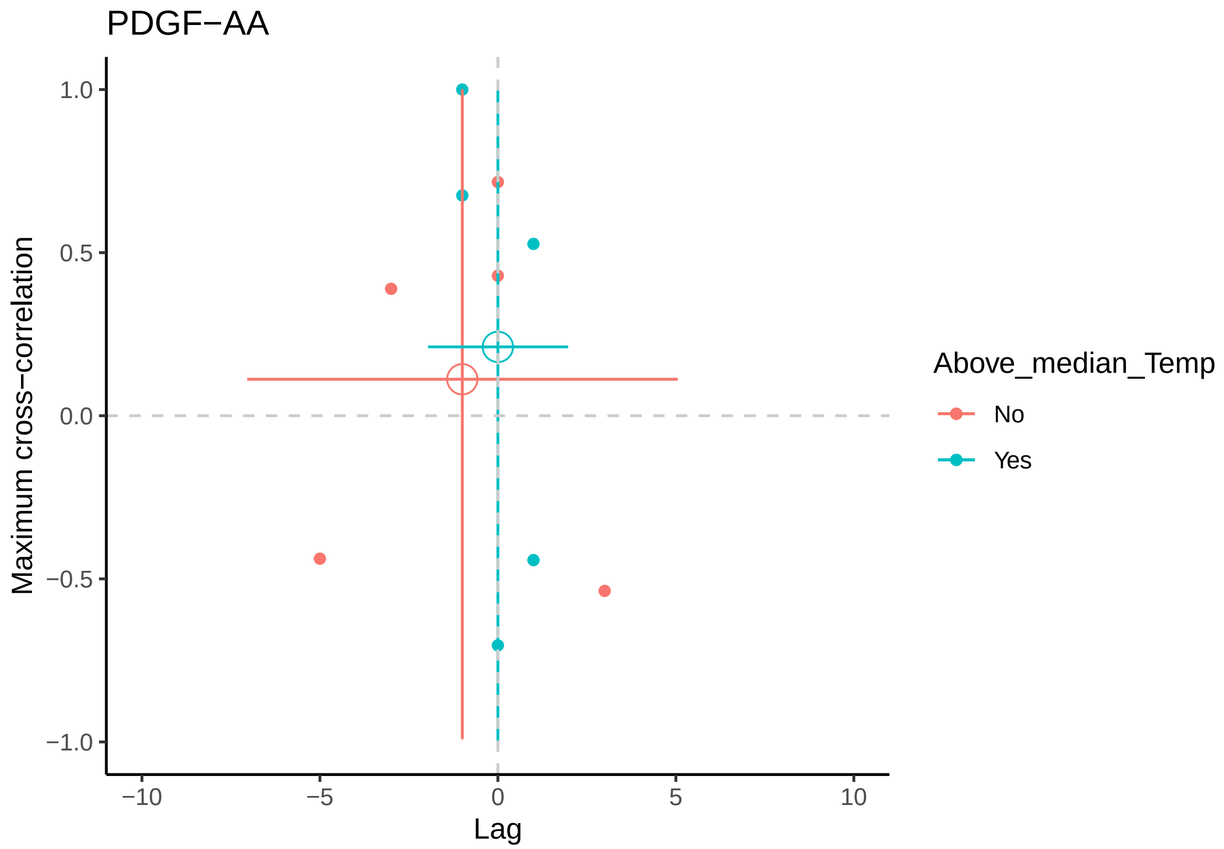
PDGF−AA
Maximum cross−correlation
1.0
0.5
0.0
−0.5
−1.0
−10
−5
0
5
10
Lag
Above_median_Temp
No
Yes

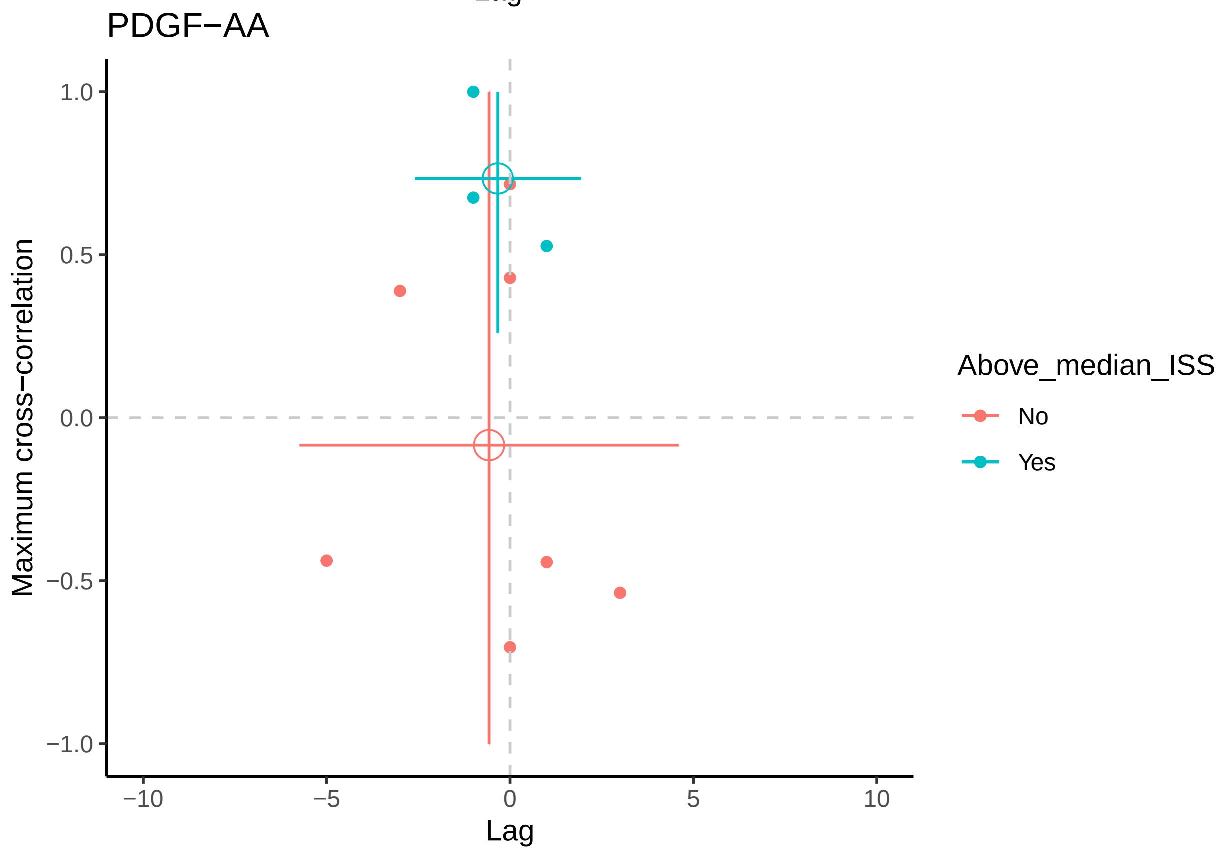
PDGF−AA
Maximum cross−correlation
1.0
0.5
0.0
−0.5
−1.0
−10
−5
0
5
10
Lag
Above_median_ISS
No
Yes

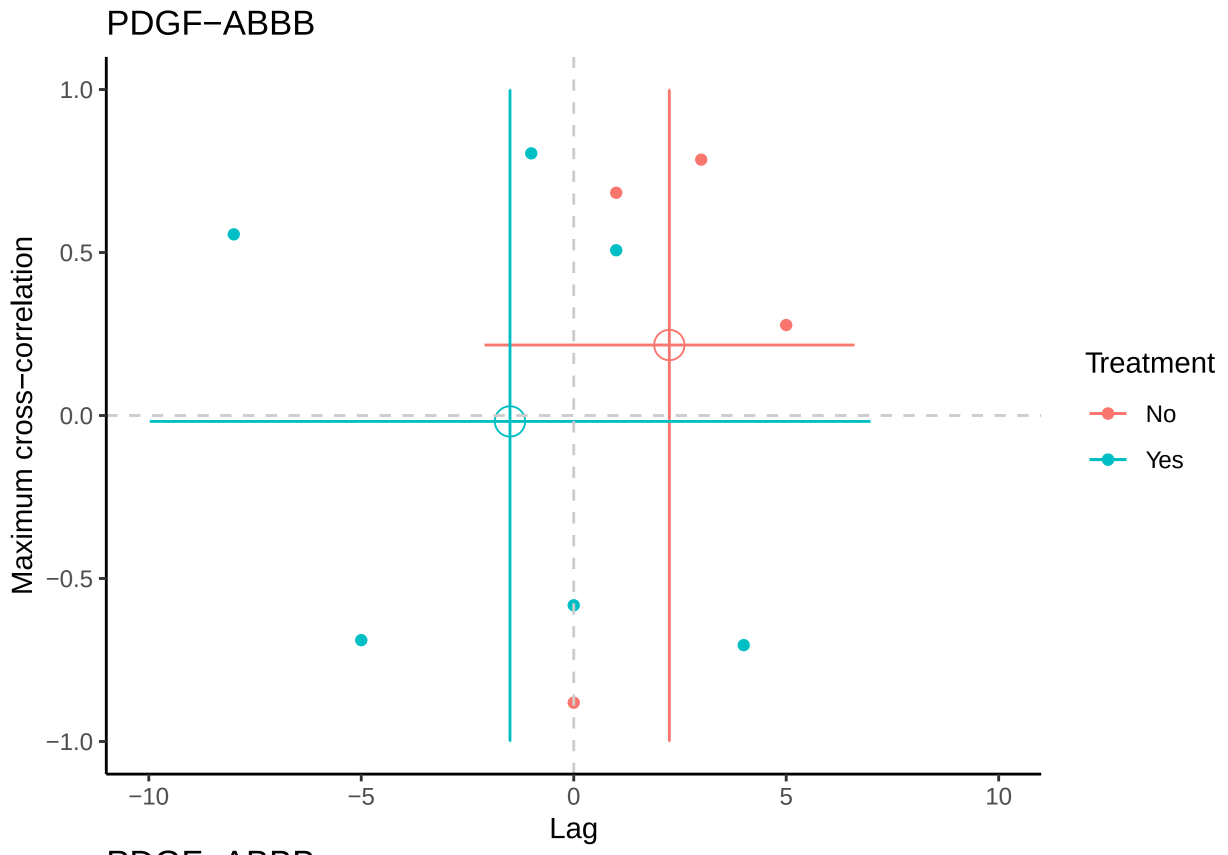
PDGF−ABBB
Maximum cross−correlation
1.0
0.5
0.0
−0.5
−1.0
−10
−5
0
5
10
Lag
Treatment
No
Yes

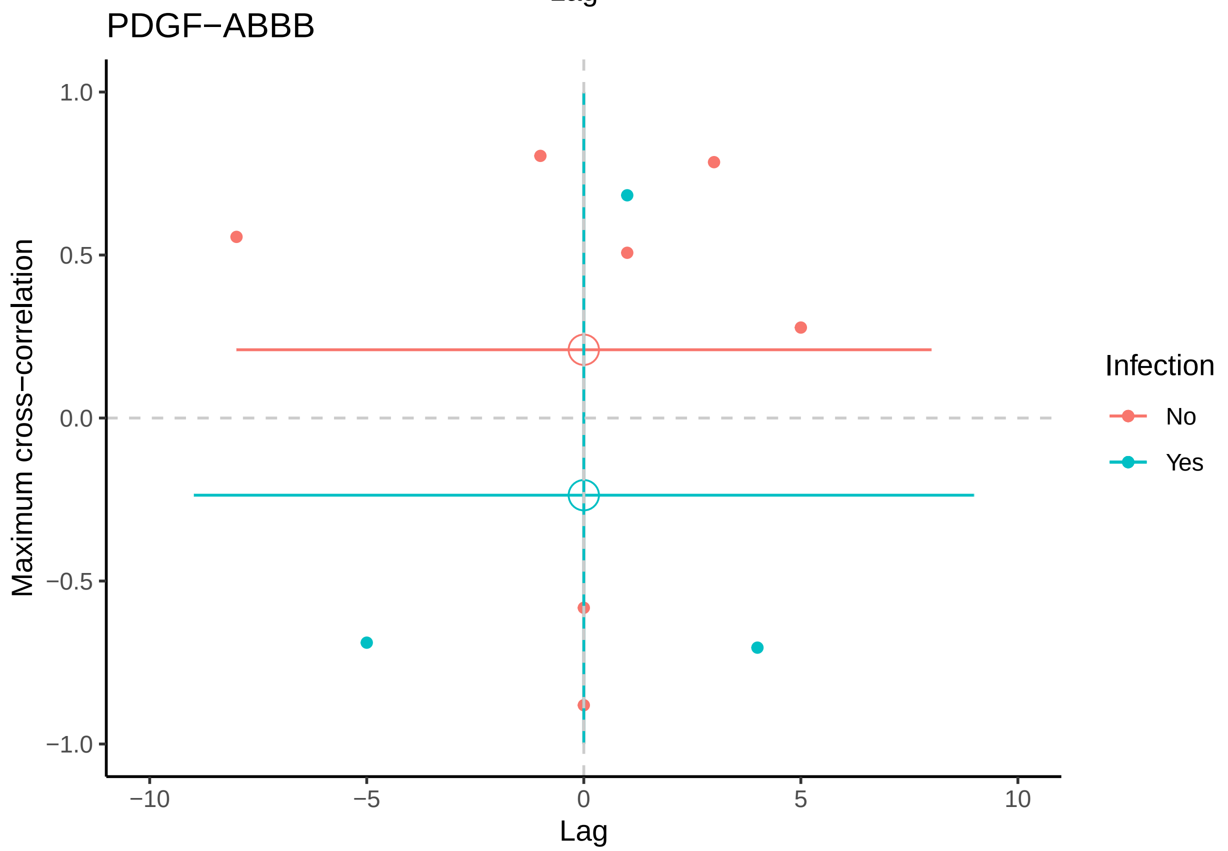
PDGF−ABBB
Maximum cross−correlation
1.0
0.5
0.0
−0.5
−1.0
−10
−5
0
5
10
Lag
Infection
No
Yes

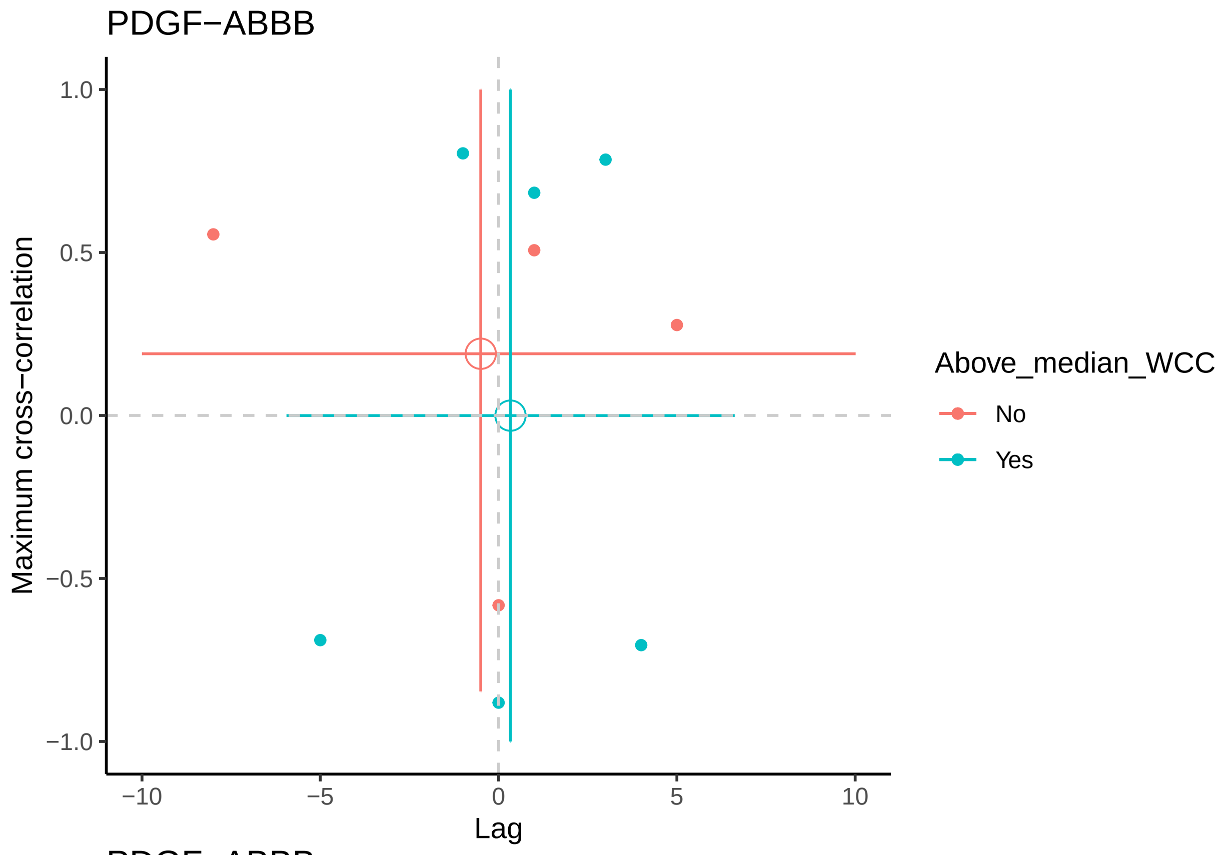
PDGF−ABBB
Maximum cross−correlation
1.0
0.5
0.0
−0.5
−1.0
−10
−5
0
5
10
Lag
Above_median_WCC
No
Yes

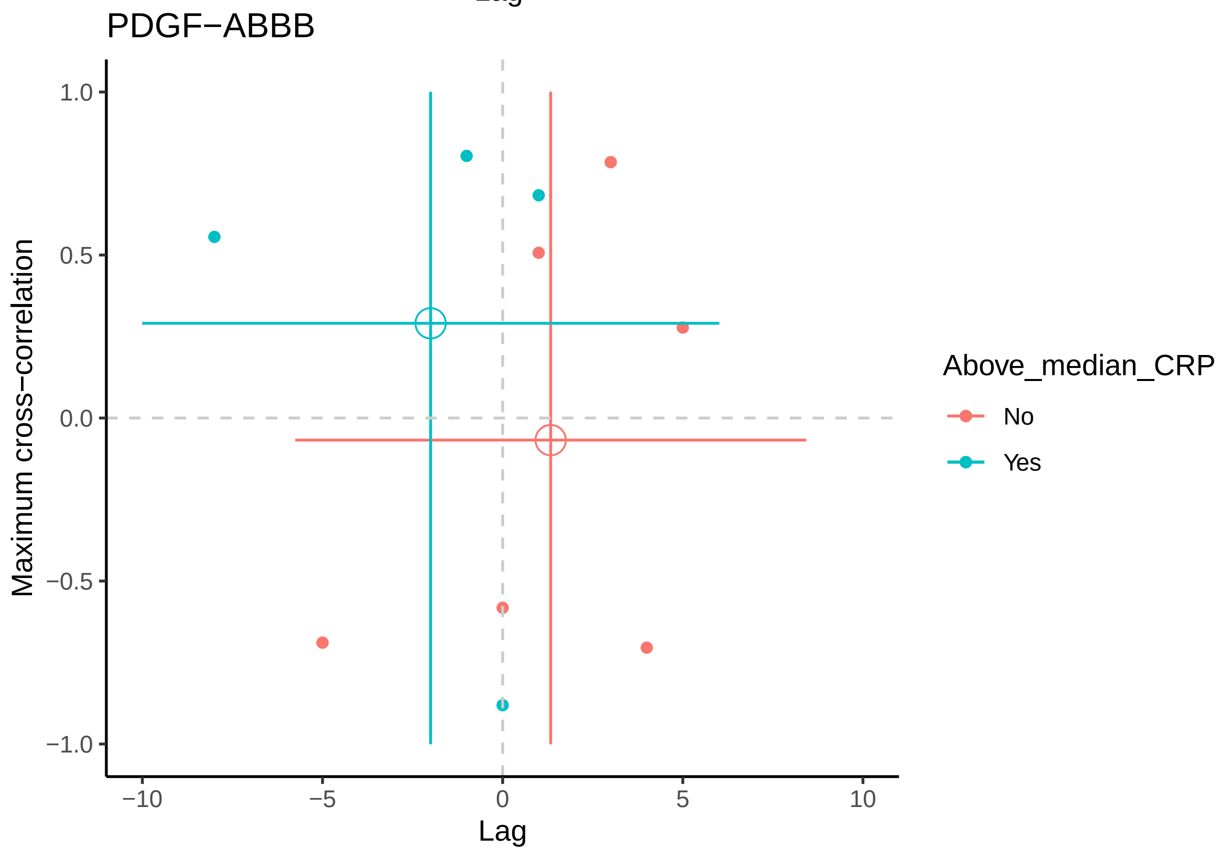
PDGF−ABBB
Maximum cross−correlation
1.0
0.5
0.0
−0.5
−1.0
−10
−5
0
5
10
Lag
Above_median_CRP
No
Yes

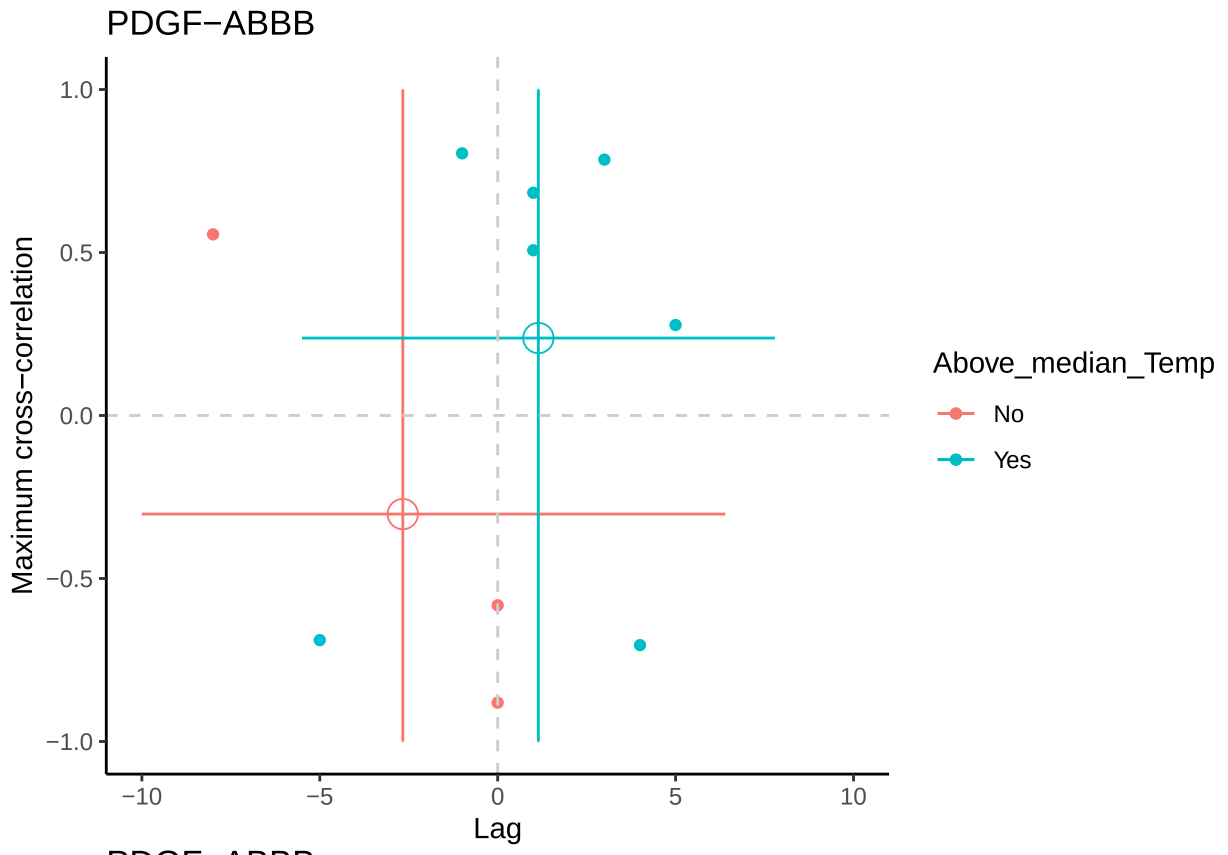
PDGF−ABBB
Maximum cross−correlation
1.0
0.5
0.0
−0.5
−1.0
−10
−5
0
5
10
Lag
Above_median_Temp
No
Yes

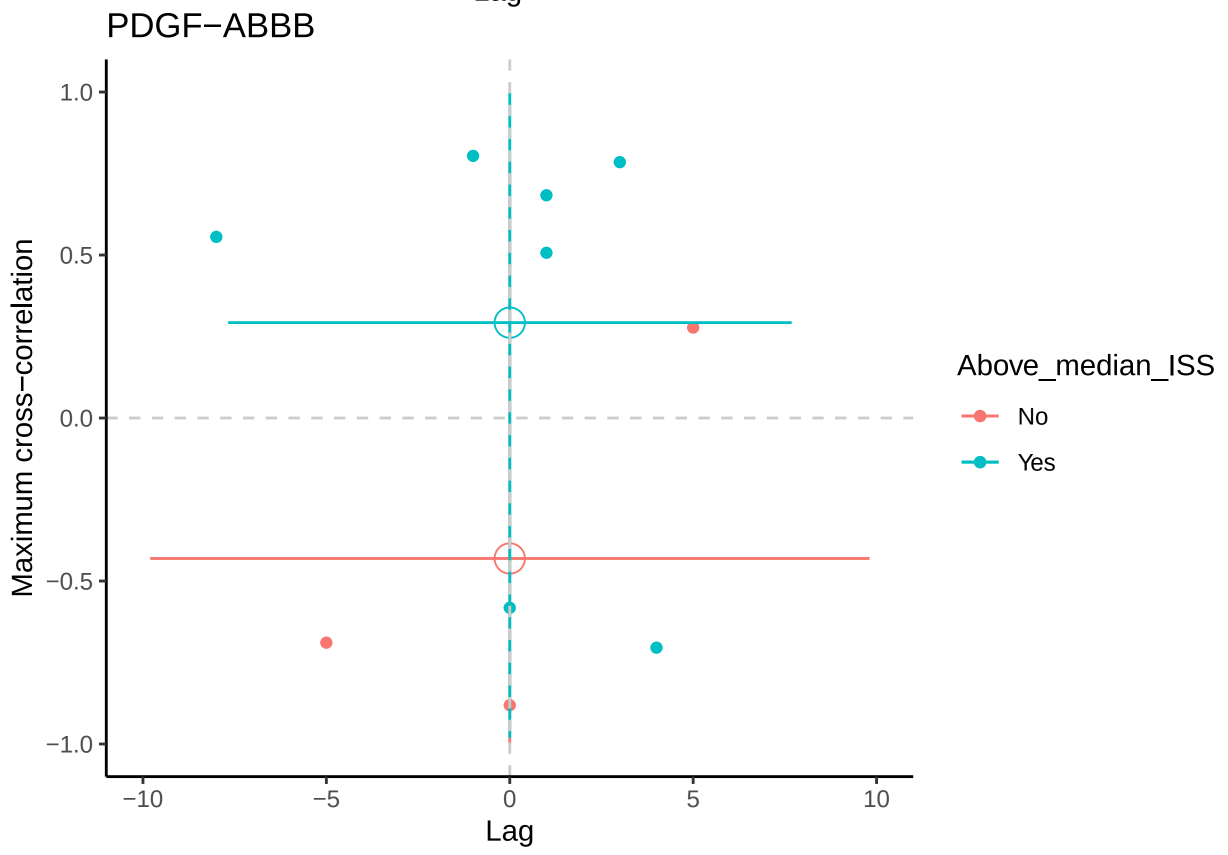
PDGF−ABBB
Maximum cross−correlation
1.0
0.5
0.0
−0.5
−1.0
−10
−5
0
5
10
Lag
Above_median_ISS
No
Yes

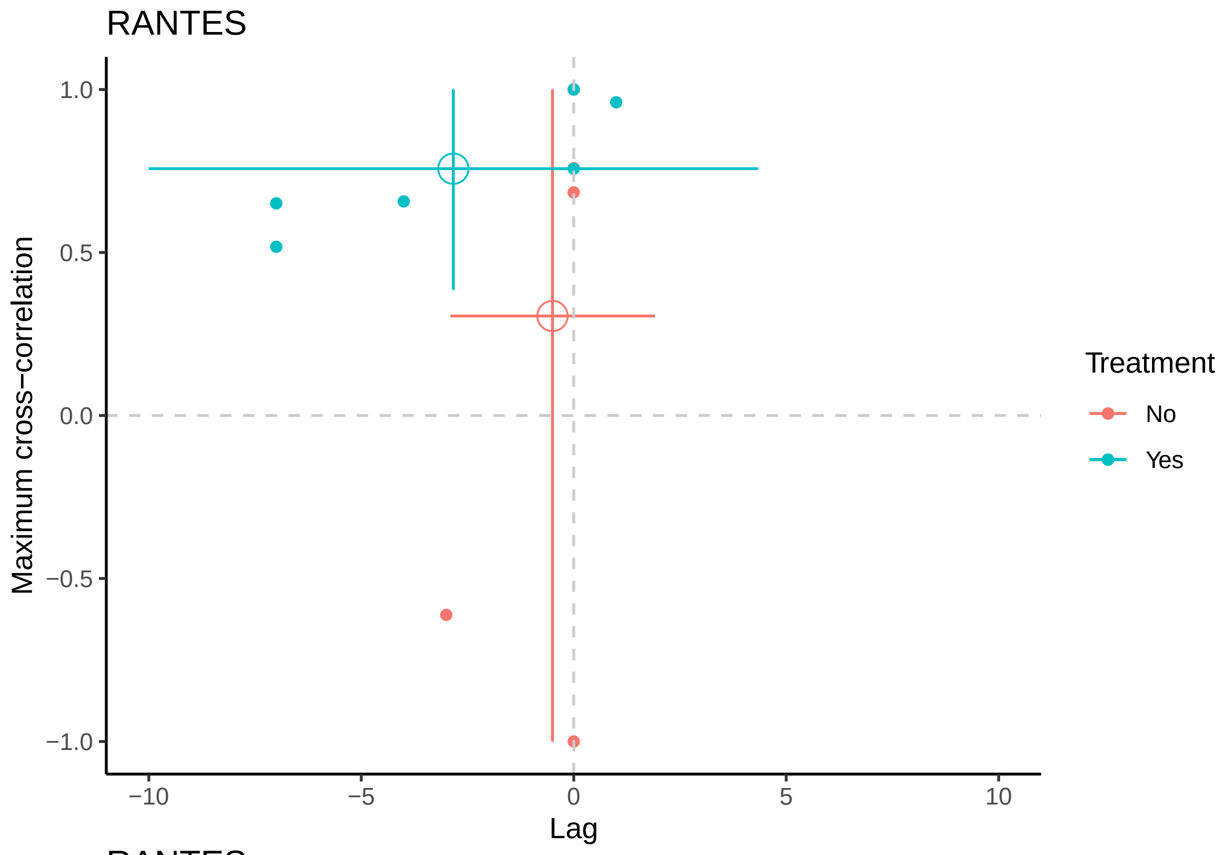
RANTES
Maximum cross−correlation
Lag
1.0
0.5
0.0
−0.5
−1.0
−10
−5
0
5
10
Treatment
No
Yes

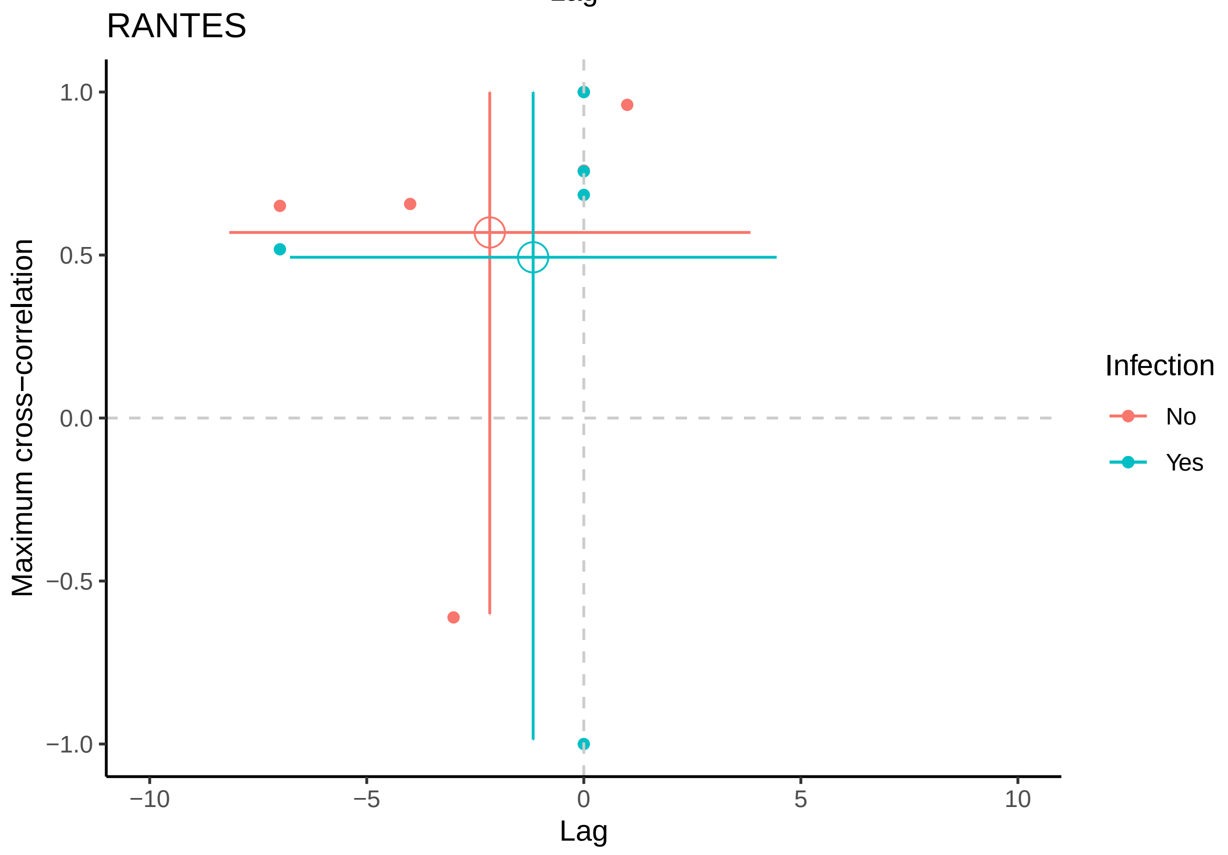
RANTES
Maximum cross−correlation
Lag
1.0
0.5
0.0
−0.5
−1.0
−10
−5
0
5
10
Infection
No
Yes

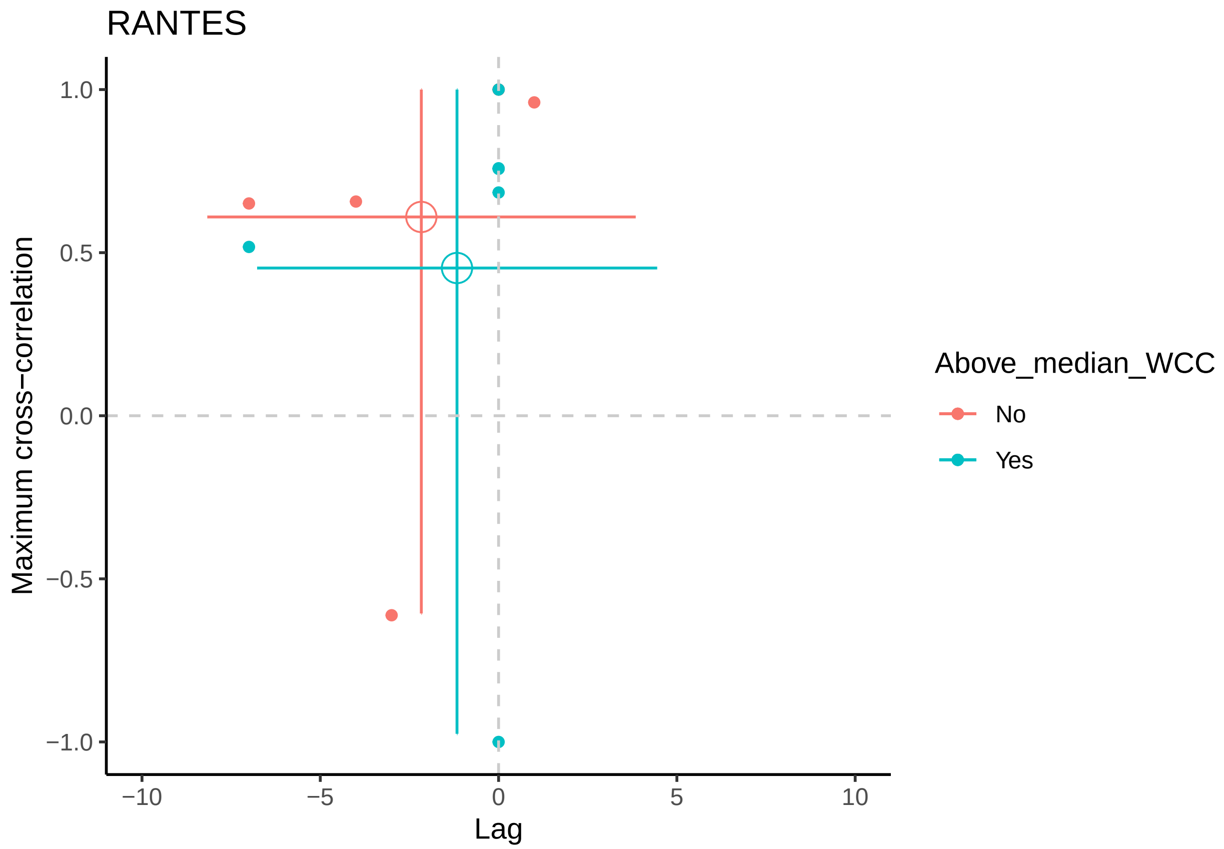
RANTES
Maximum cross−correlation
1.0
0.5
0.0
−0.5
−1.0
−10
−5
0
5
10
Lag
Above_median_WCC
No
Yes

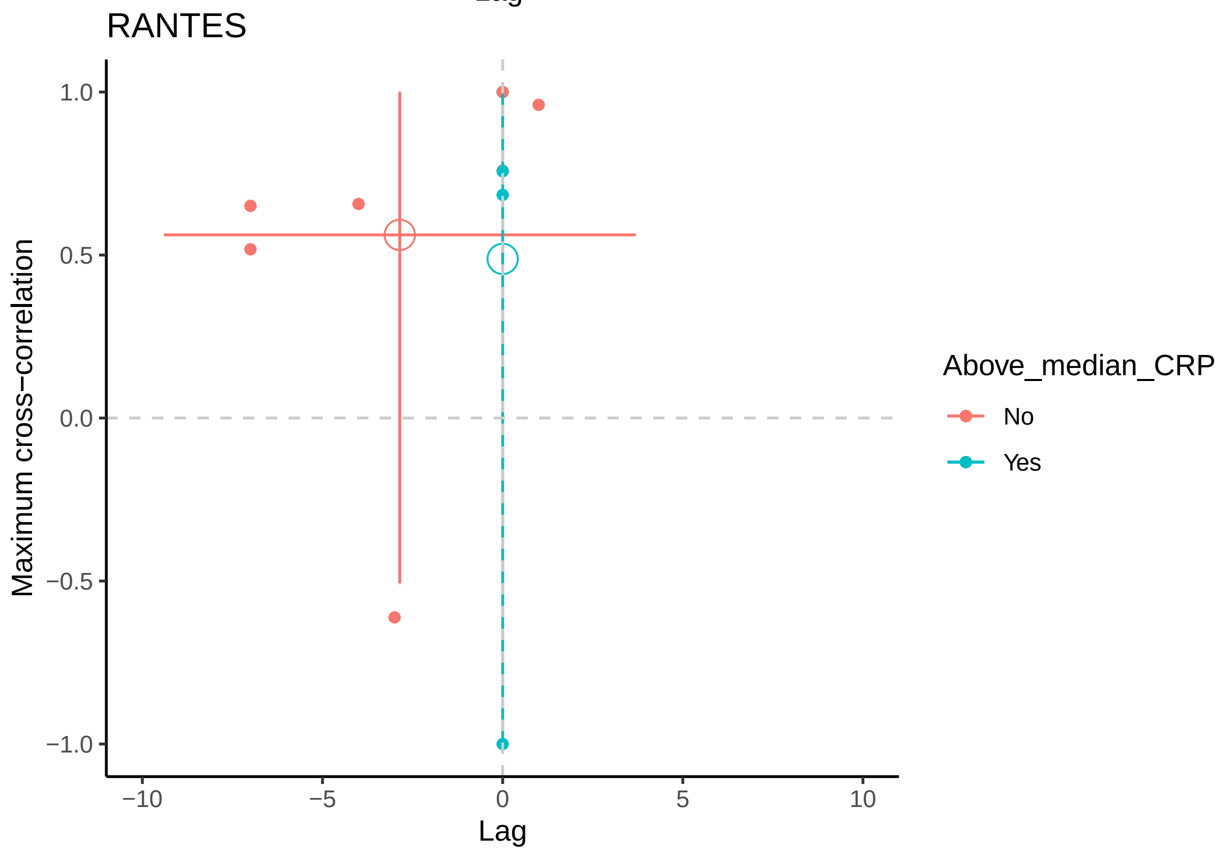
RANTES
Maximum cross−correlation
1.0
0.5
0.0
−0.5
−1.0
−10
−5
0
5
10
Lag
Above_median_CRP
No
Yes

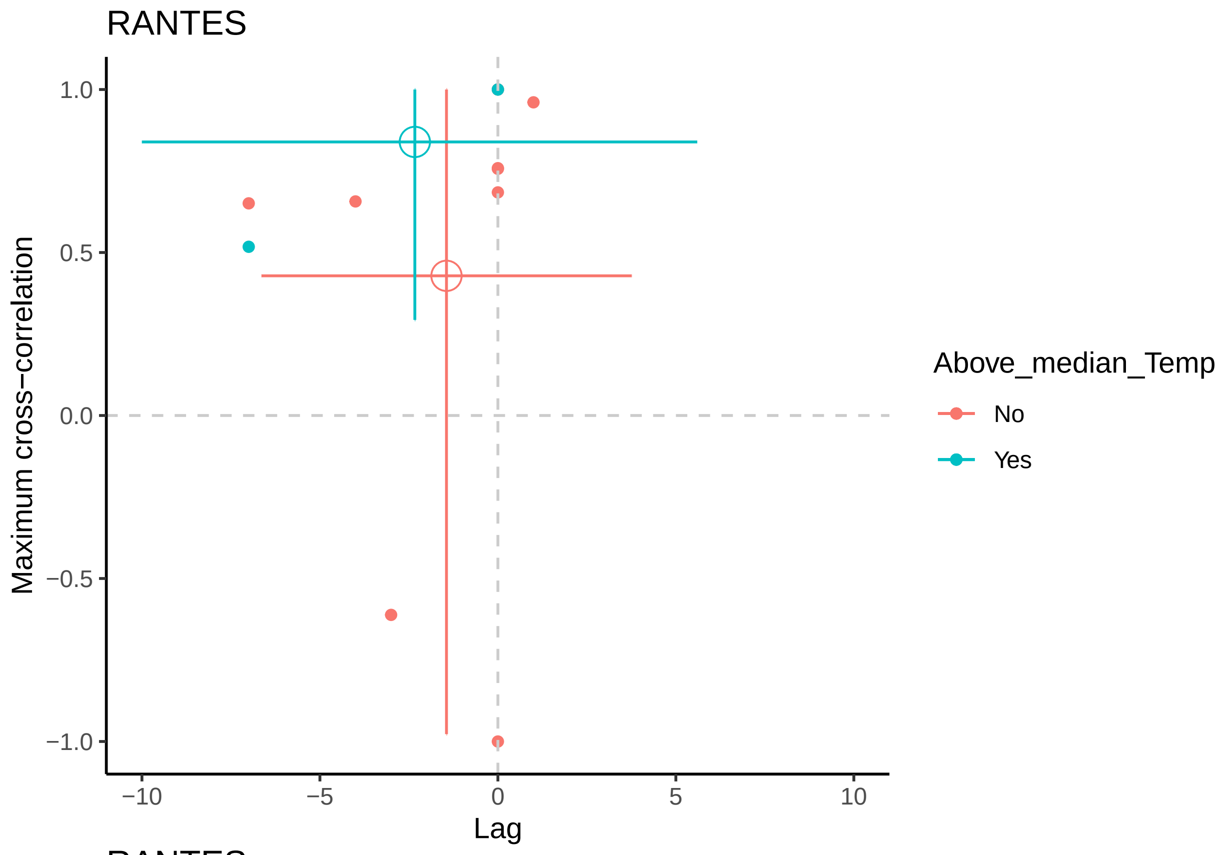
RANTES
Maximum cross−correlation
1.0
0.5
0.0
−0.5
−1.0
−10
−5
0
5
10
Lag
Above_median_Temp
No
Yes

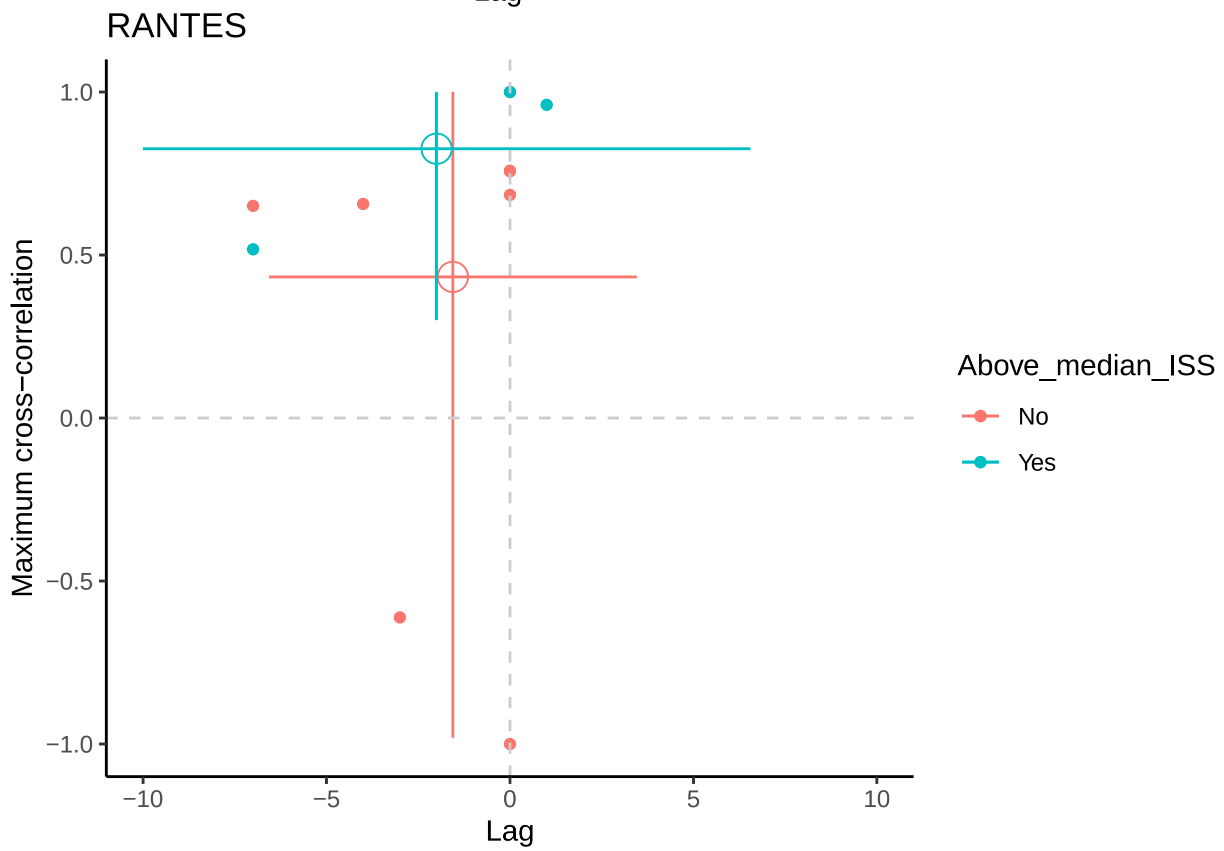
RANTES
Maximum cross−correlation
1.0
0.5
0.0
−0.5
−1.0
−10
−5
0
5
10
Lag
Above_median_ISS
No
Yes

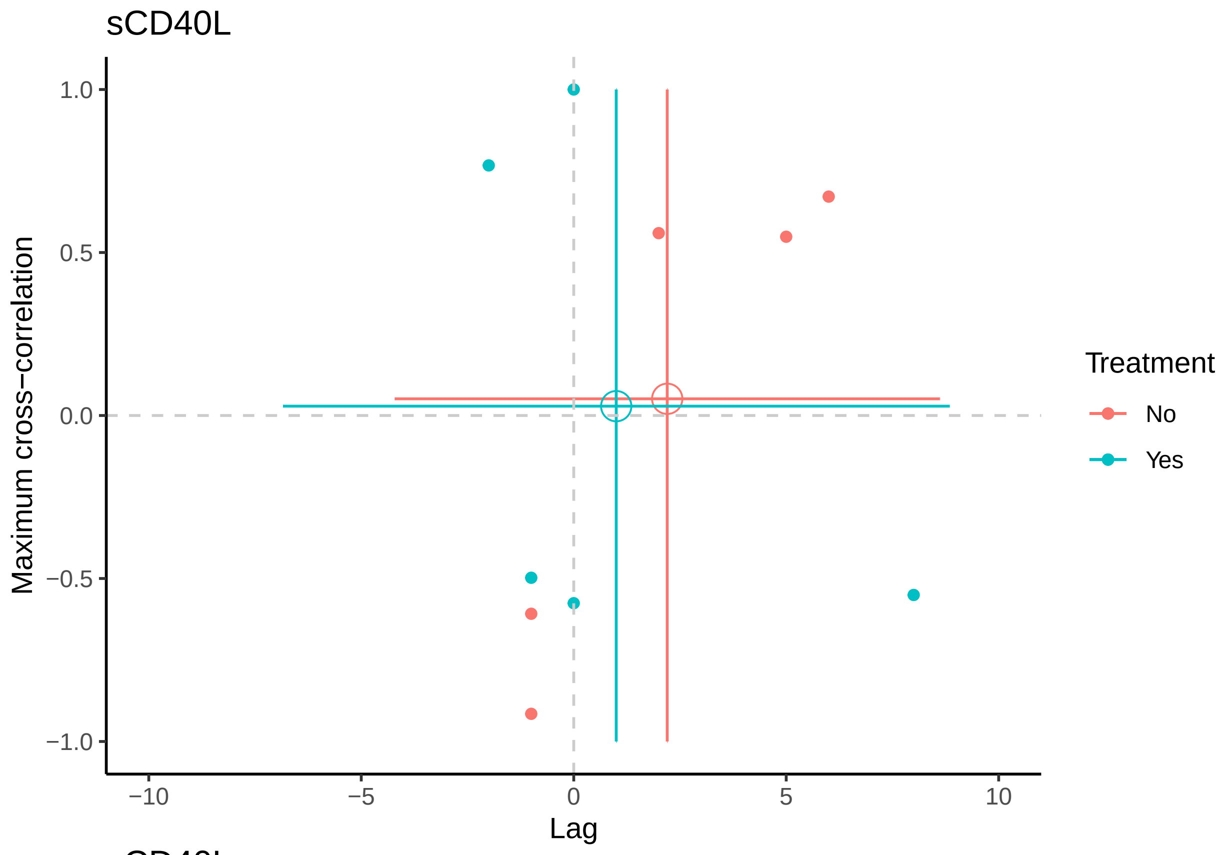

sCD40L
Maximum cross−correlation
1.0
0.5
0.0
−0.5
−1.0
−10
−5
0
5
10
Lag
Treatment
No
Yes

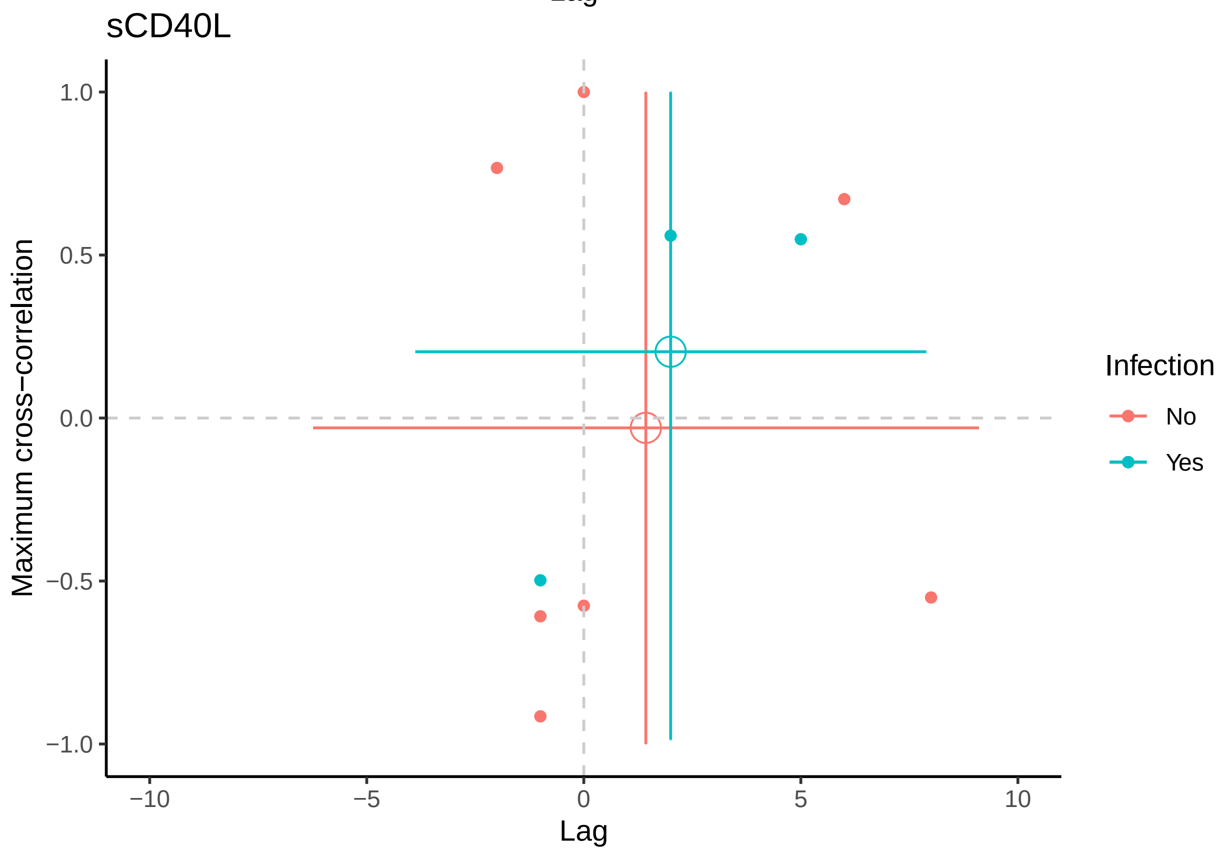

sCD40L
Maximum cross−correlation
1.0
0.5
0.0
−0.5
−1.0
−10
−5
0
5
10
Lag
Infection
No
Yes

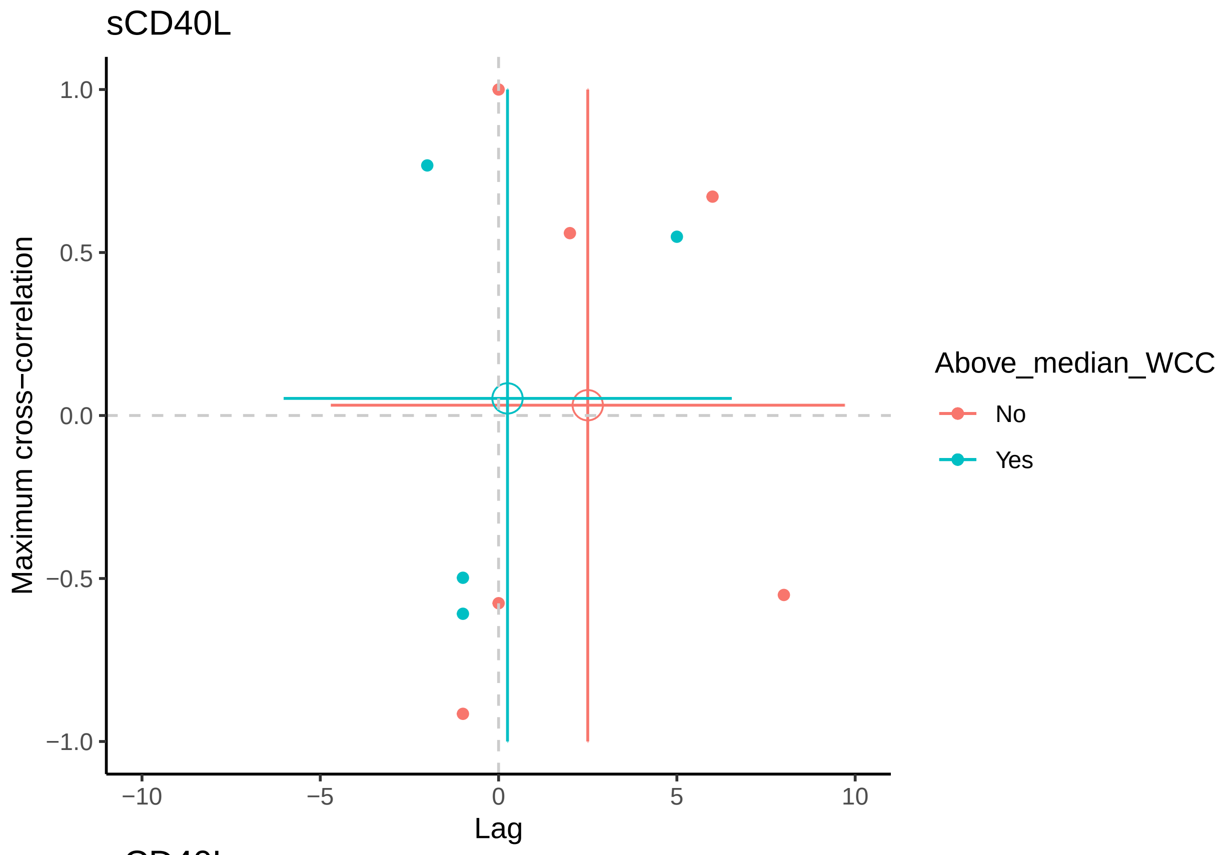
sCD40L
Maximum cross−correlation
1.0
0.5
0.0
−0.5
−1.0
−10
−5
0
5
10
Lag
Above_median_WCC
No
Yes

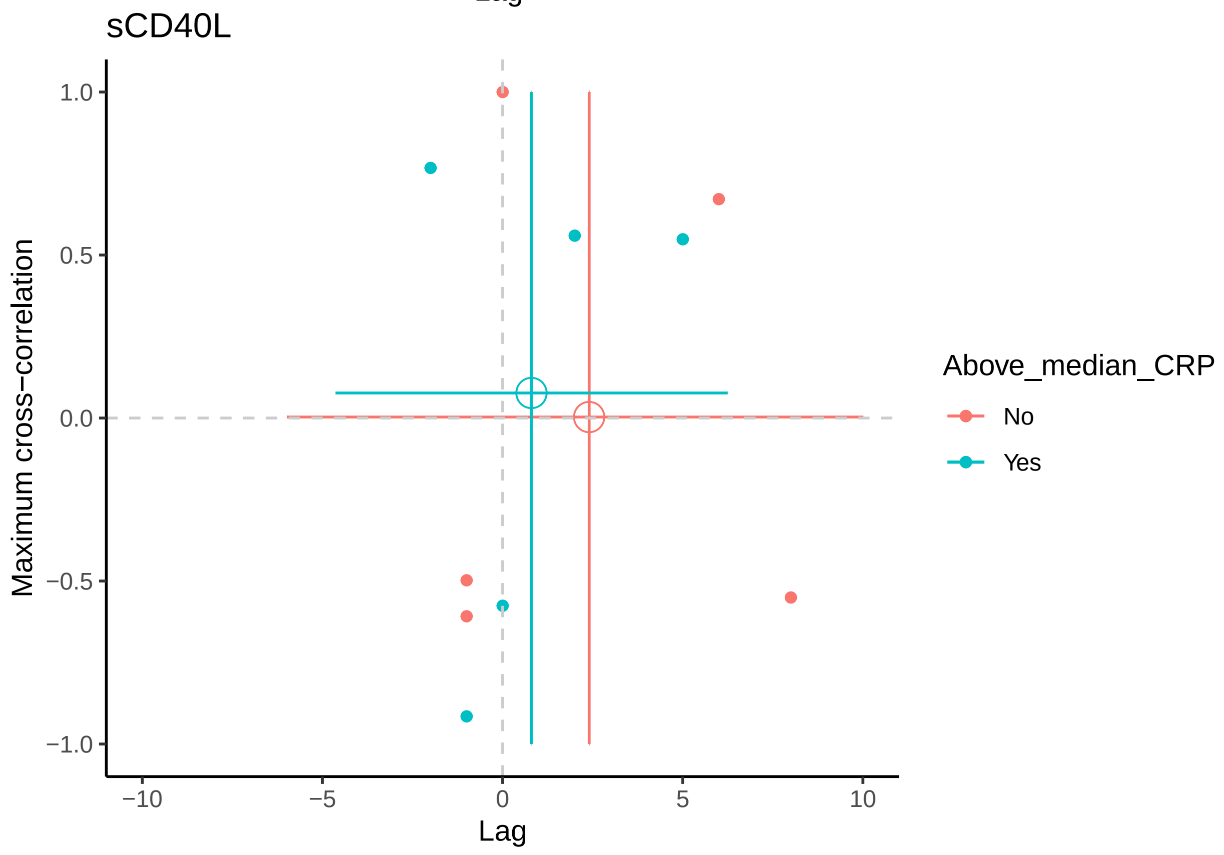
sCD40L
Maximum cross−correlation
1.0
0.5
0.0
−0.5
−1.0
−10
−5
0
5
10
Lag
Above_median_CRP
No
Yes

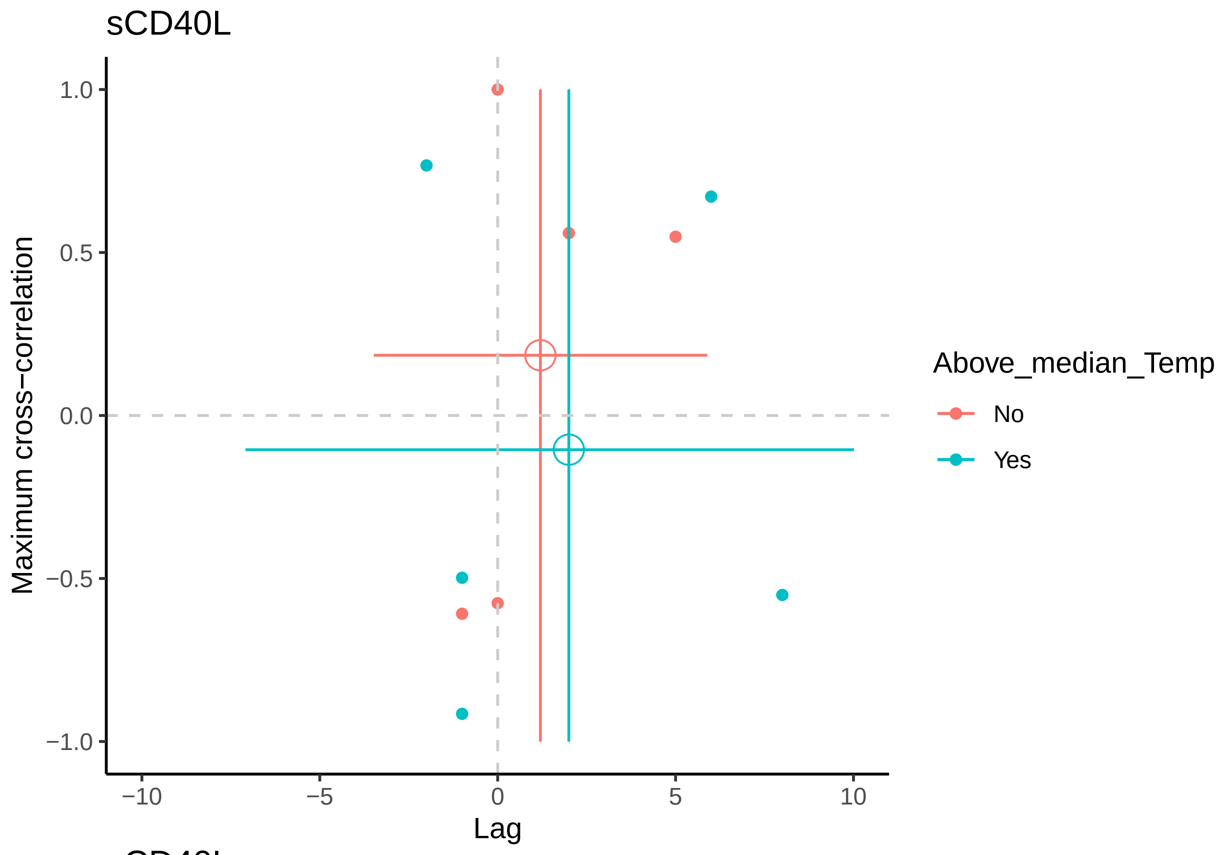
sCD40L
Maximum cross−correlation
Lag
Above_median_Temp
No
Yes
1.0
0.5
0.0
−0.5
−1.0
−10
−5
0
5
10

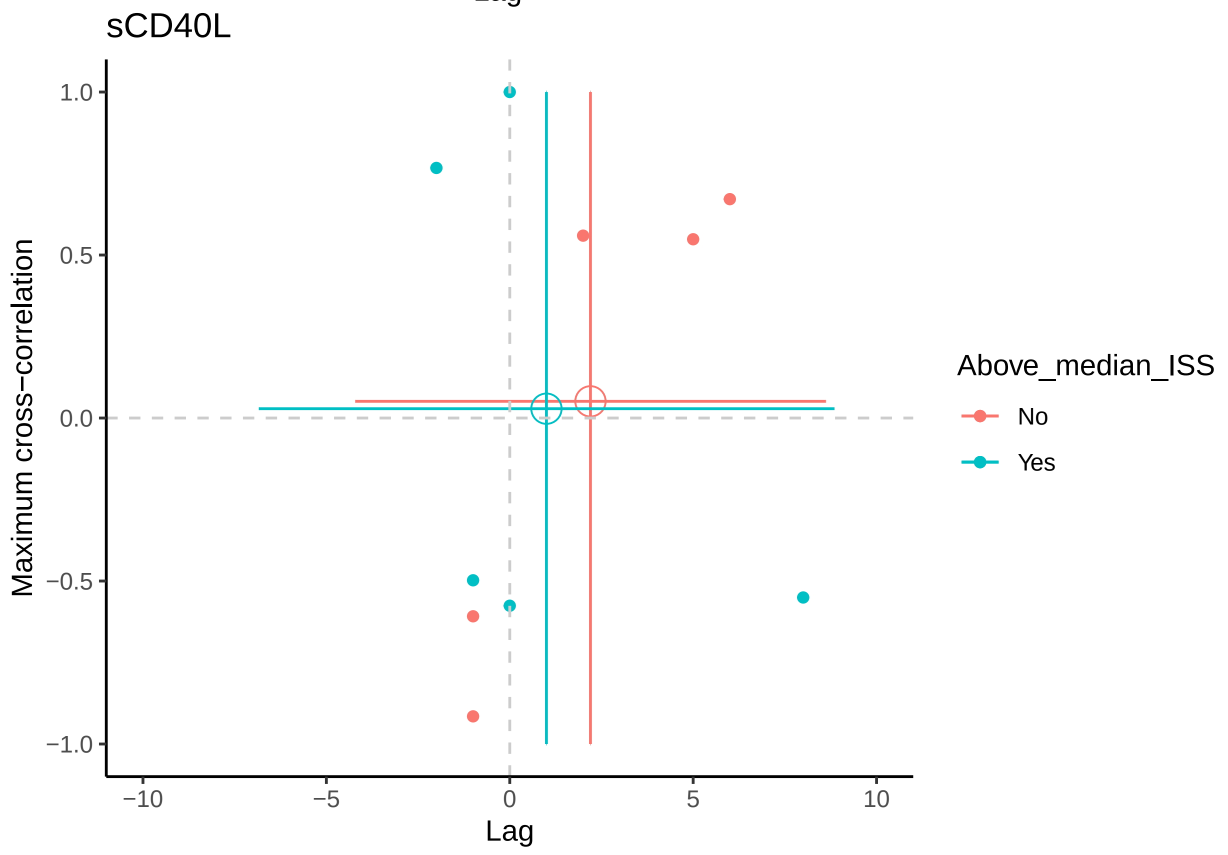
sCD40L
Maximum cross−correlation
Lag
Above_median_ISS
No
Yes
1.0
0.5
0.0
−0.5
−1.0
−10
−5
0
5
10

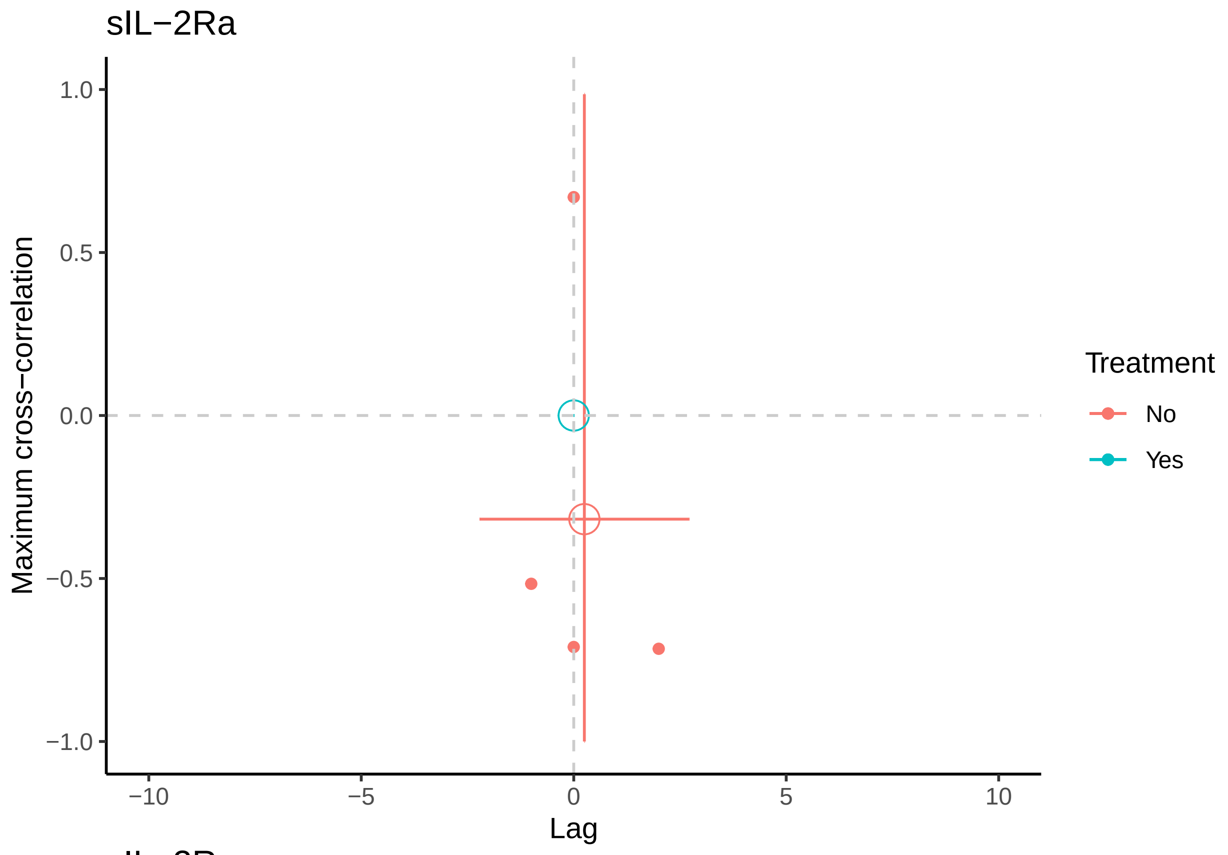
sIL−2Ra
Maximum cross−correlation
1.0
0.5
0.0
−0.5
−1.0
−10
−5
0
5
10
Lag
Treatment
No
Yes

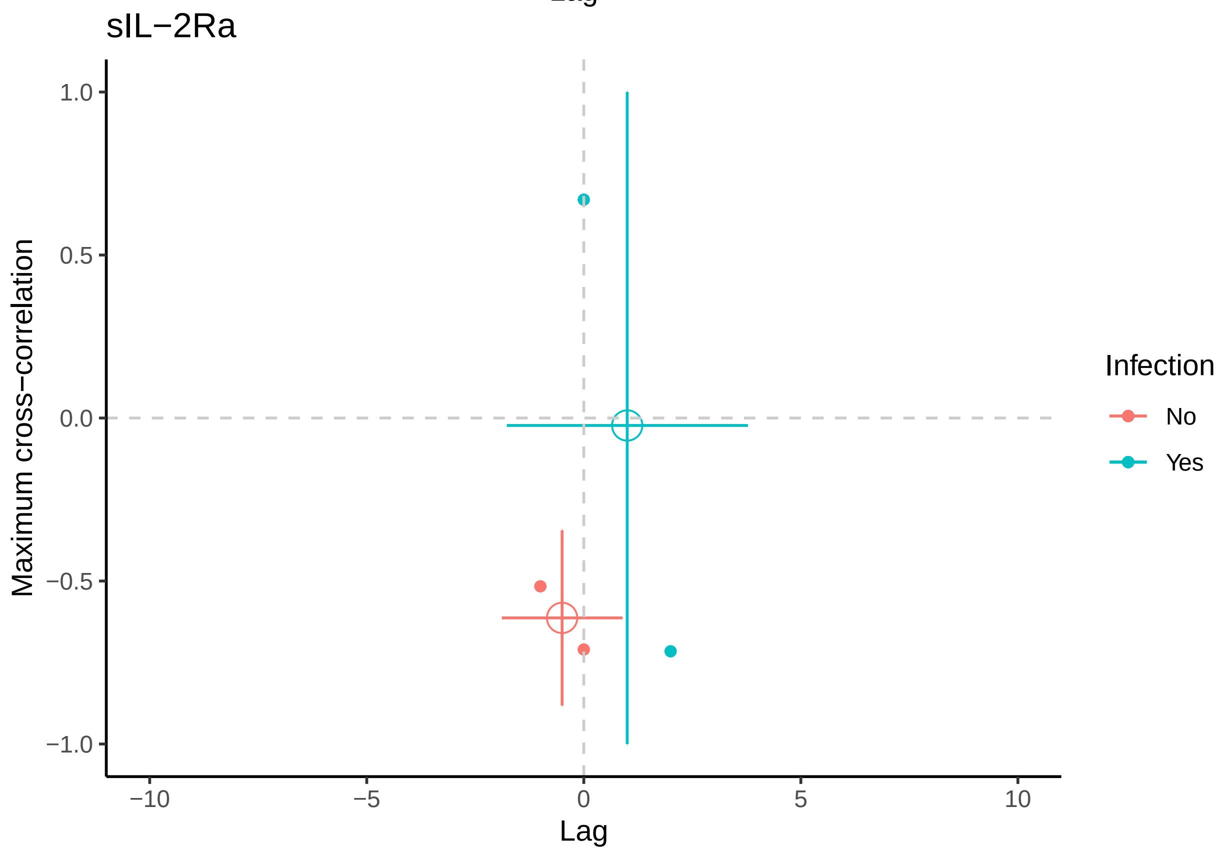
sIL−2Ra
Maximum cross−correlation
1.0
0.5
0.0
−0.5
−1.0
−10
−5
0
5
10
Lag
Infection
No
Yes

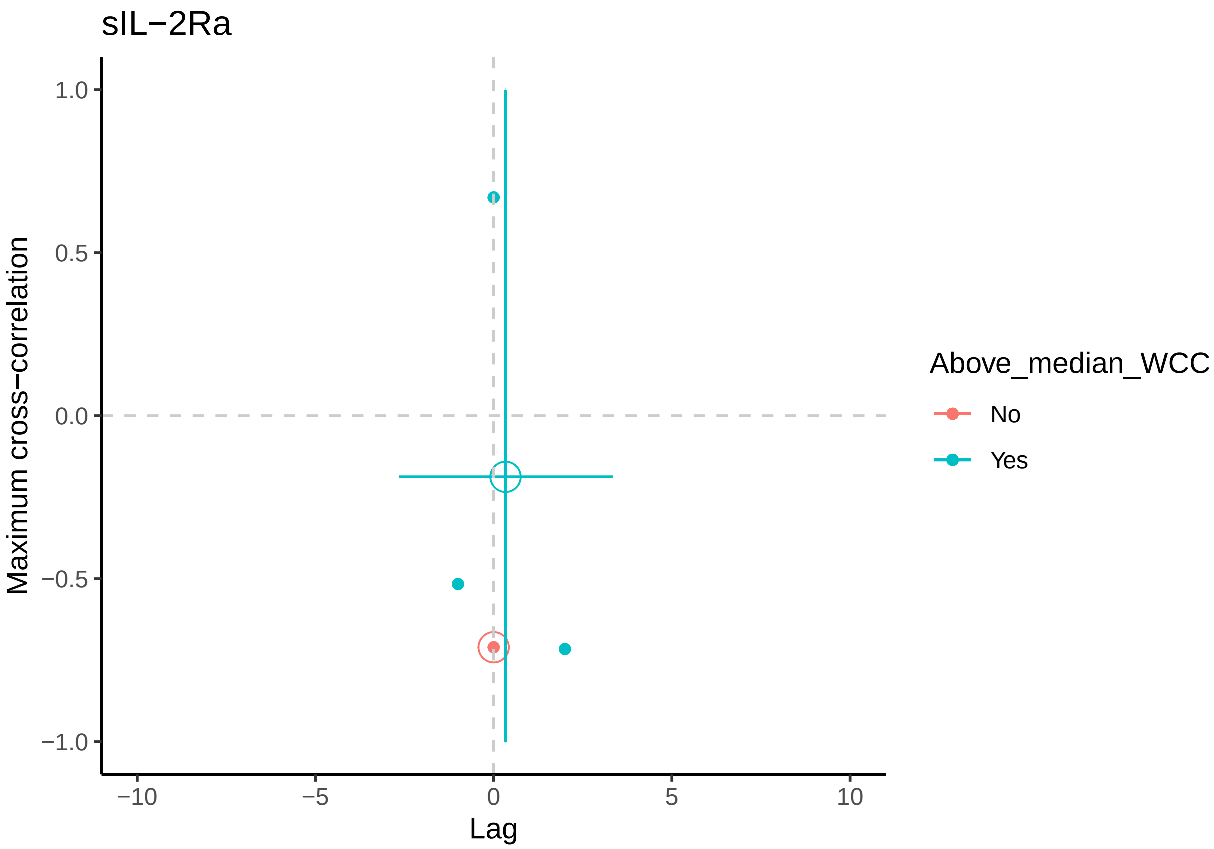
sIL−2Ra
Maximum cross−correlation
1.0
0.5
0.0
−0.5
−1.0
−10
−5
0
5
10
Lag
Above_median_WCC
No
Yes

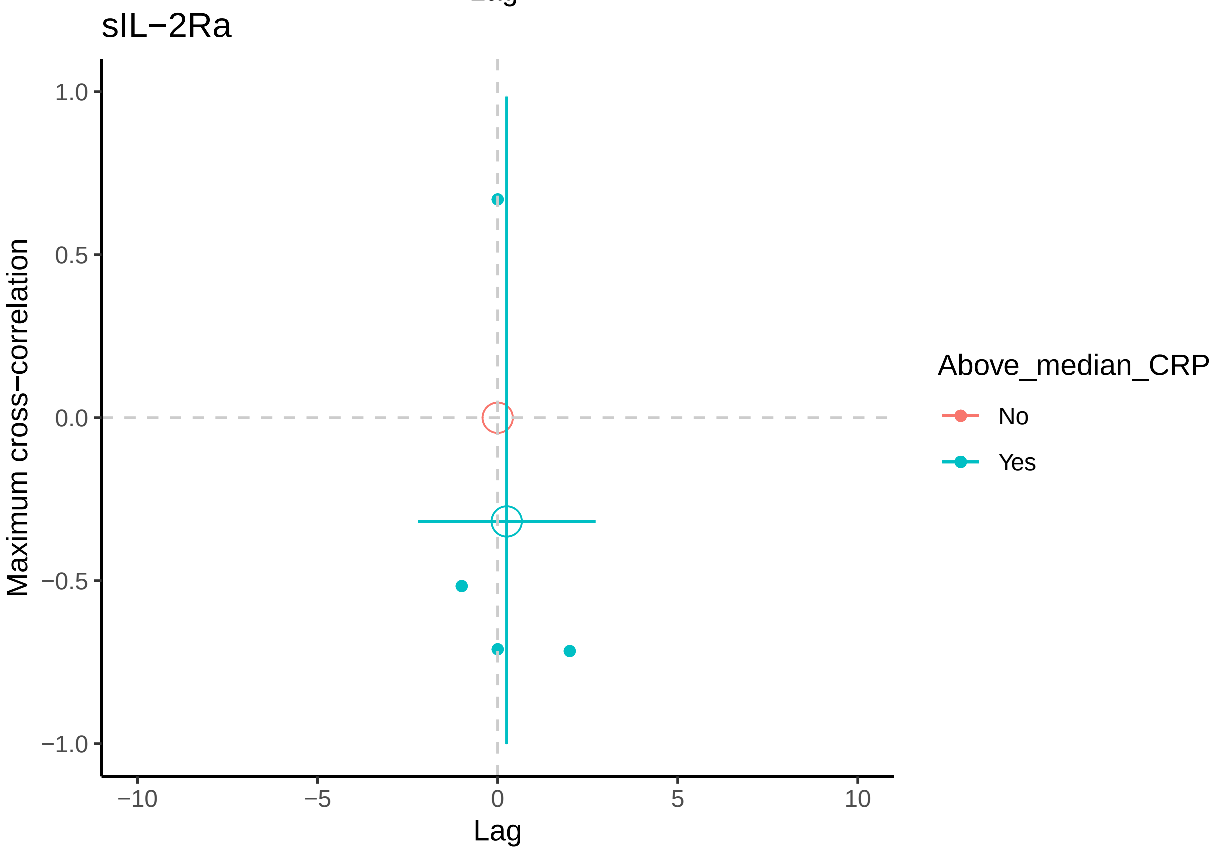
sIL−2Ra
Maximum cross−correlation
1.0
0.5
0.0
−0.5
−1.0
−10
−5
0
5
10
Lag
Above_median_CRP
No
Yes

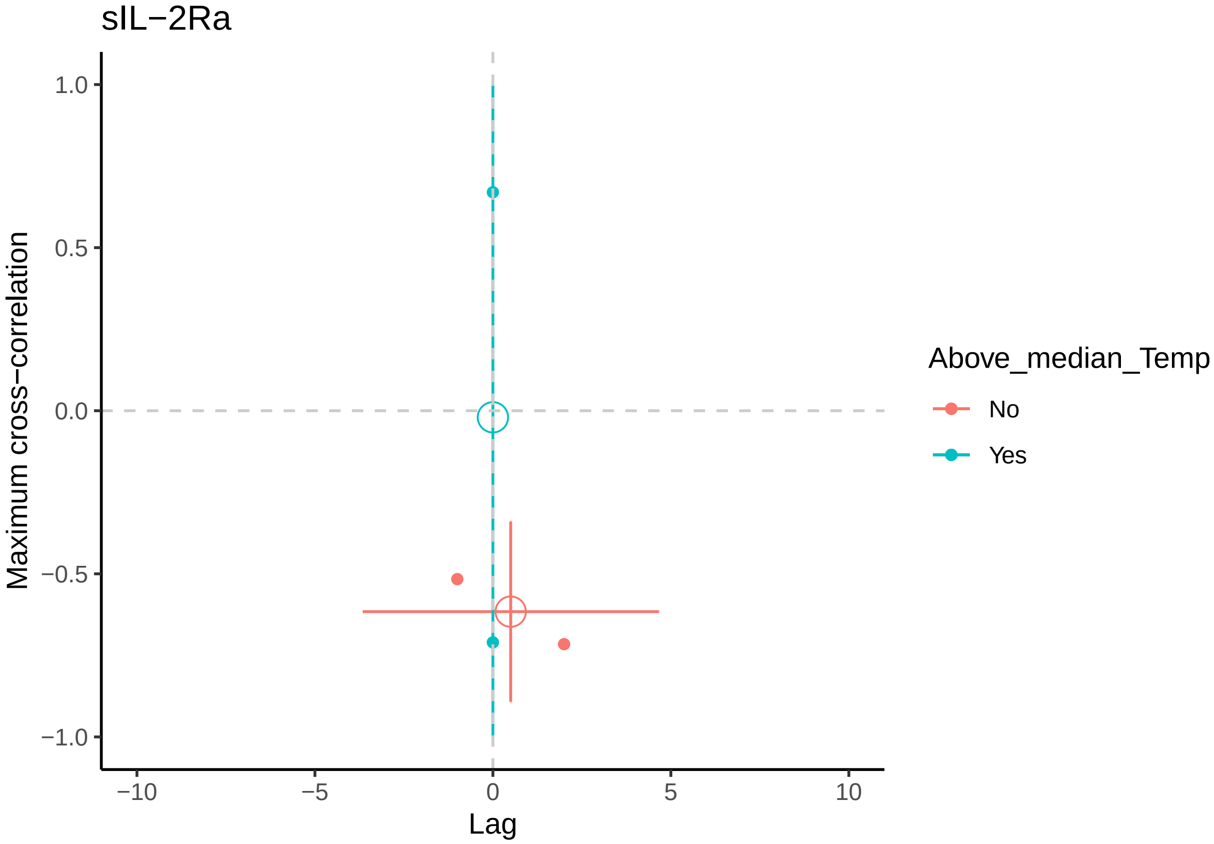
sIL−2Ra
Maximum cross−correlation
1.0
0.5
0.0
−0.5
−1.0
−10
−5
0
5
10
Lag
Above_median_Temp
No
Yes

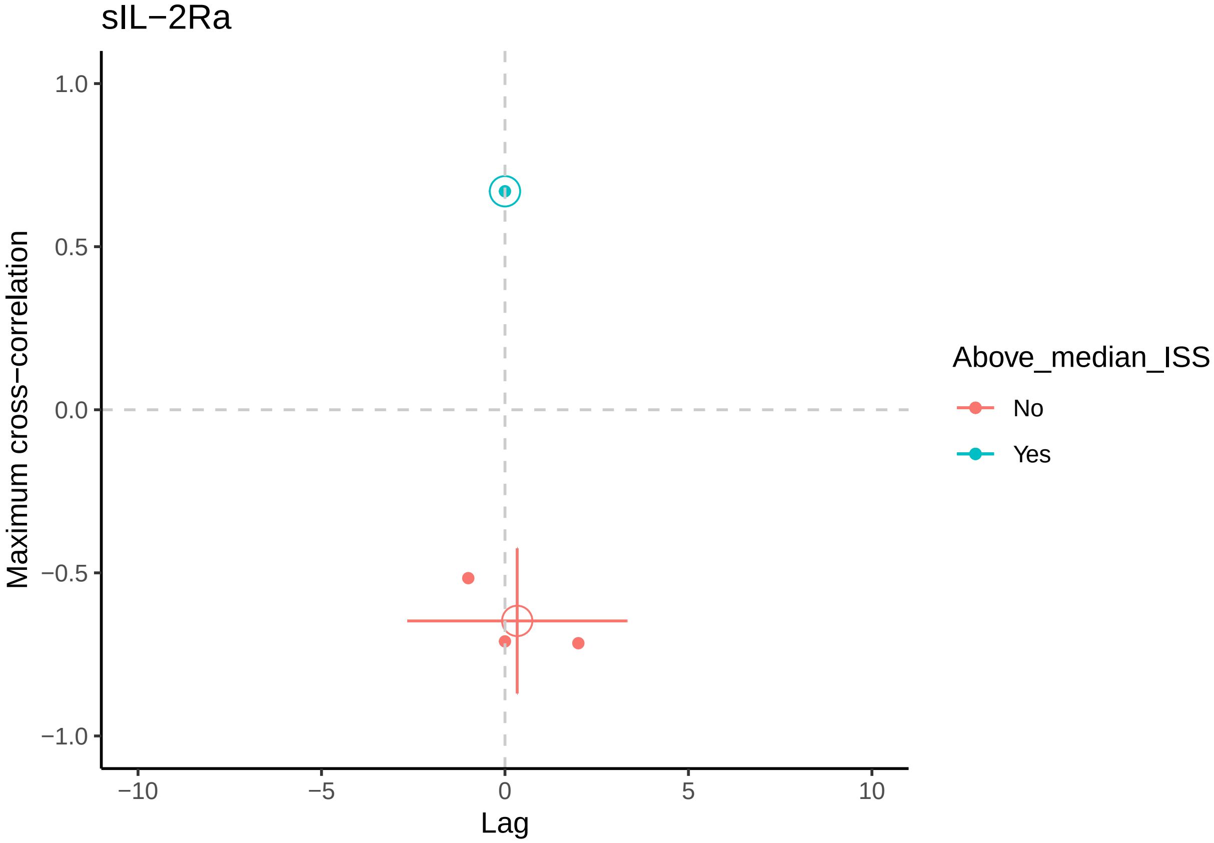
sIL−2Ra
Maximum cross−correlation
1.0
0.5
0.0
−0.5
−1.0
−10
−5
0
5
10
Lag
Above_median_ISS
No
Yes

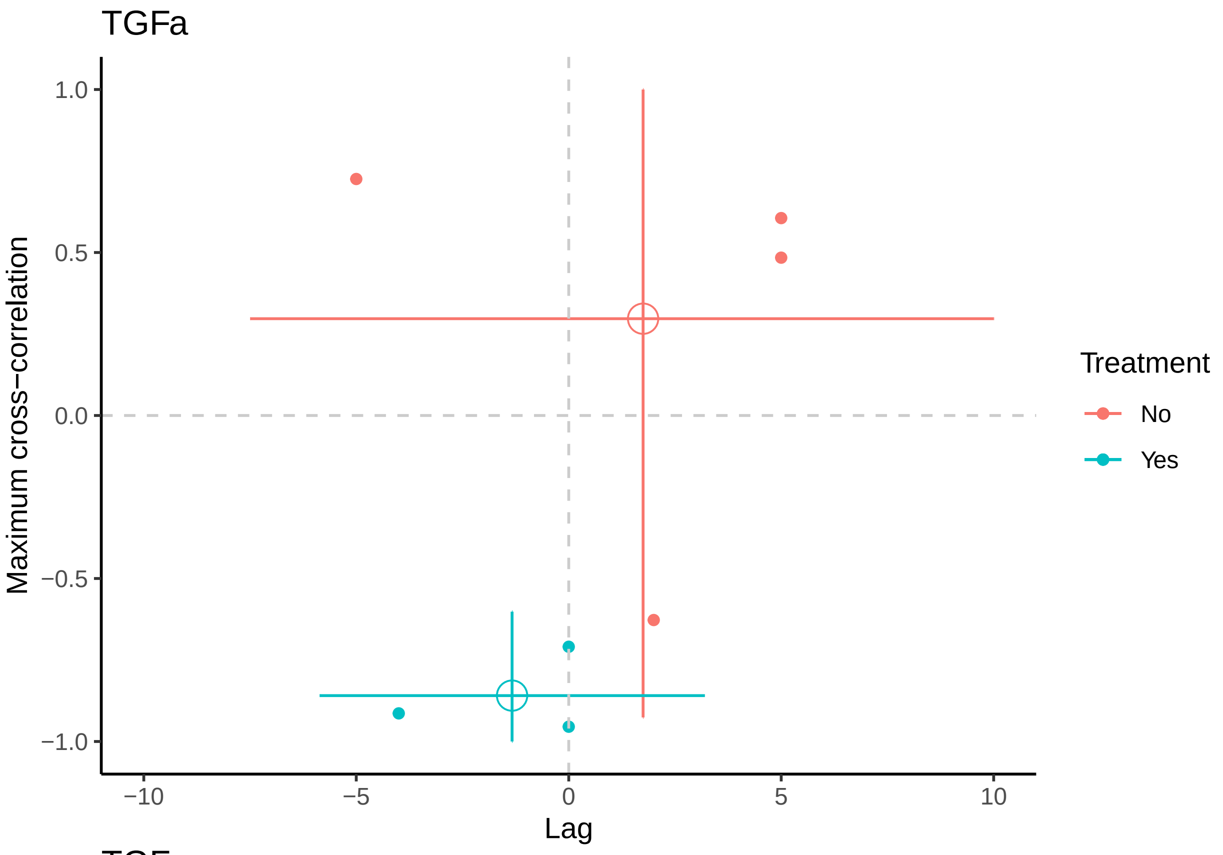
TGFa
Maximum cross−correlation
Lag
Treatment
No
Yes
1.0
0.5
0.0
−0.5
−1.0
−10
−5
0
5
10

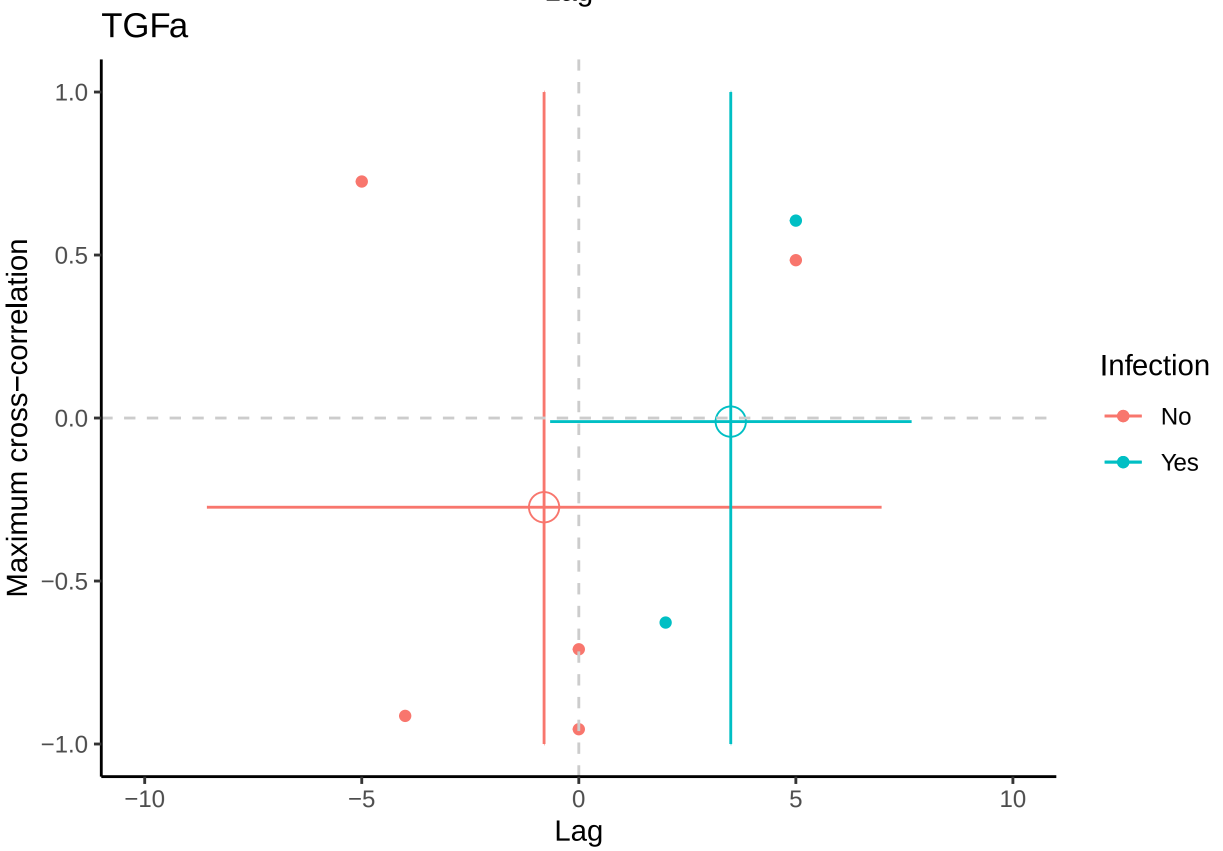
TGFa
Maximum cross−correlation
Lag
Infection
No
Yes
1.0
0.5
0.0
−0.5
−1.0
−10
−5
0
5
10

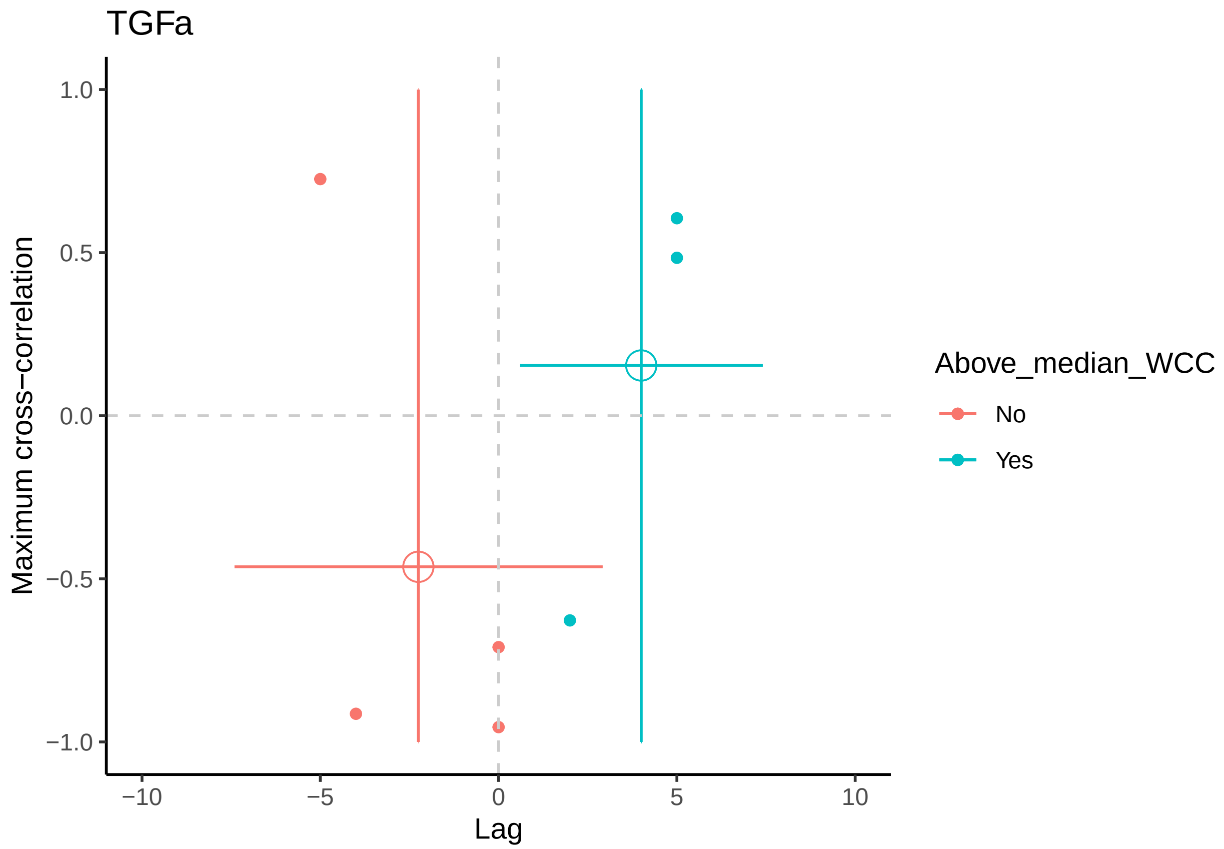
TGFa
Maximum cross−correlation
1.0
0.5
0.0
−0.5
−1.0
−10
−5
0
5
10
Lag
Above_median_WCC
No
Yes

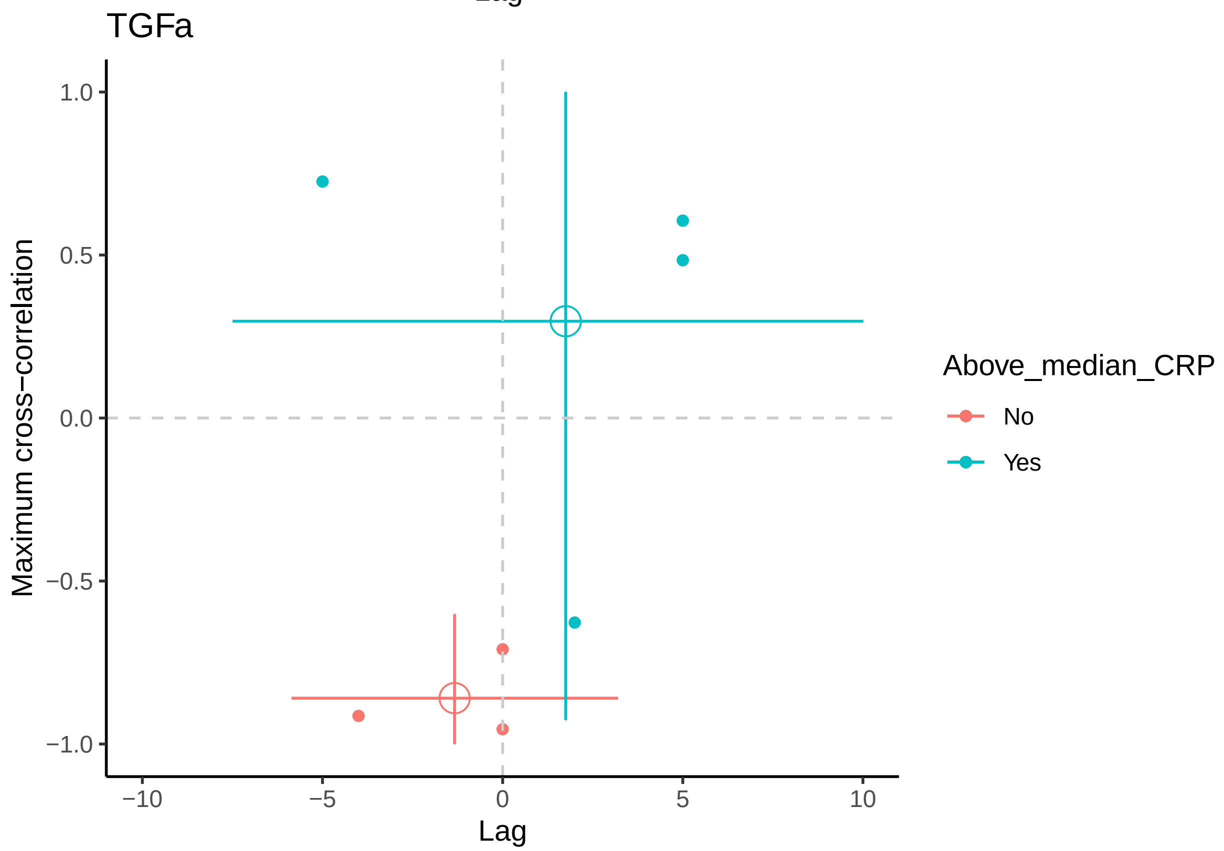
TGFa
Maximum cross−correlation
1.0
0.5
0.0
−0.5
−1.0
−10
−5
0
5
10
Lag
Above_median_CRP
No
Yes

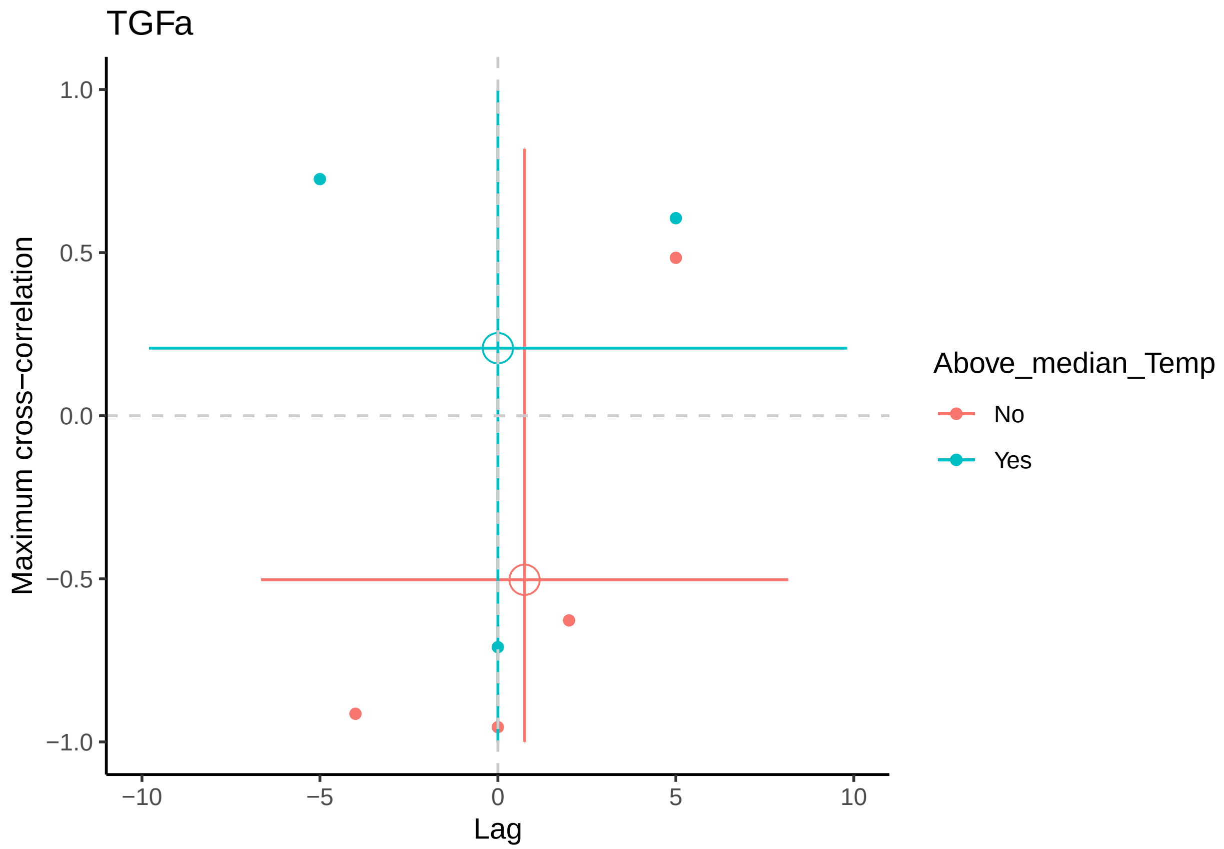
TGFa
Maximum cross−correlation
1.0
0.5
0.0
−0.5
−1.0
−10
−5
0
5
10
Lag
Above_median_Temp
No
Yes

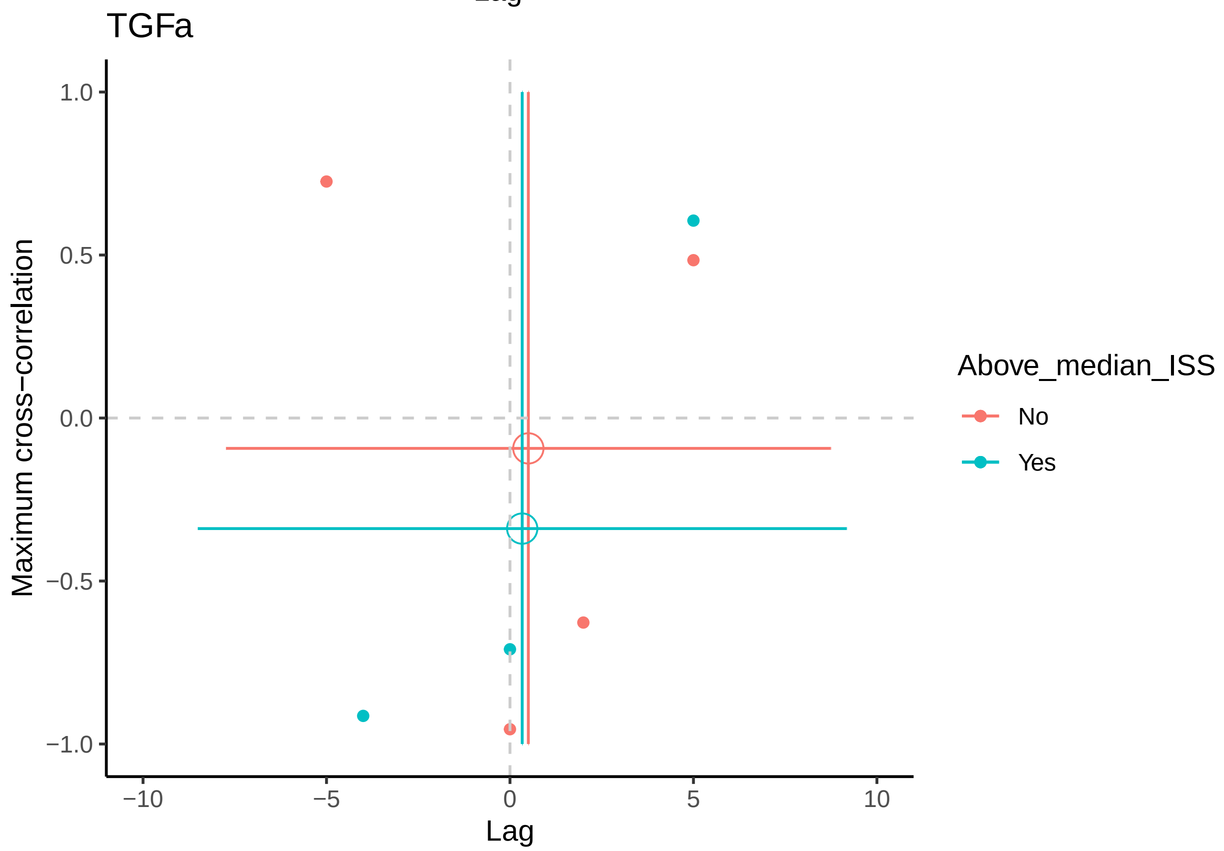
TGFa
Maximum cross−correlation
1.0
0.5
0.0
−0.5
−1.0
−10
−5
0
5
10
Lag
Above_median_ISS
No
Yes

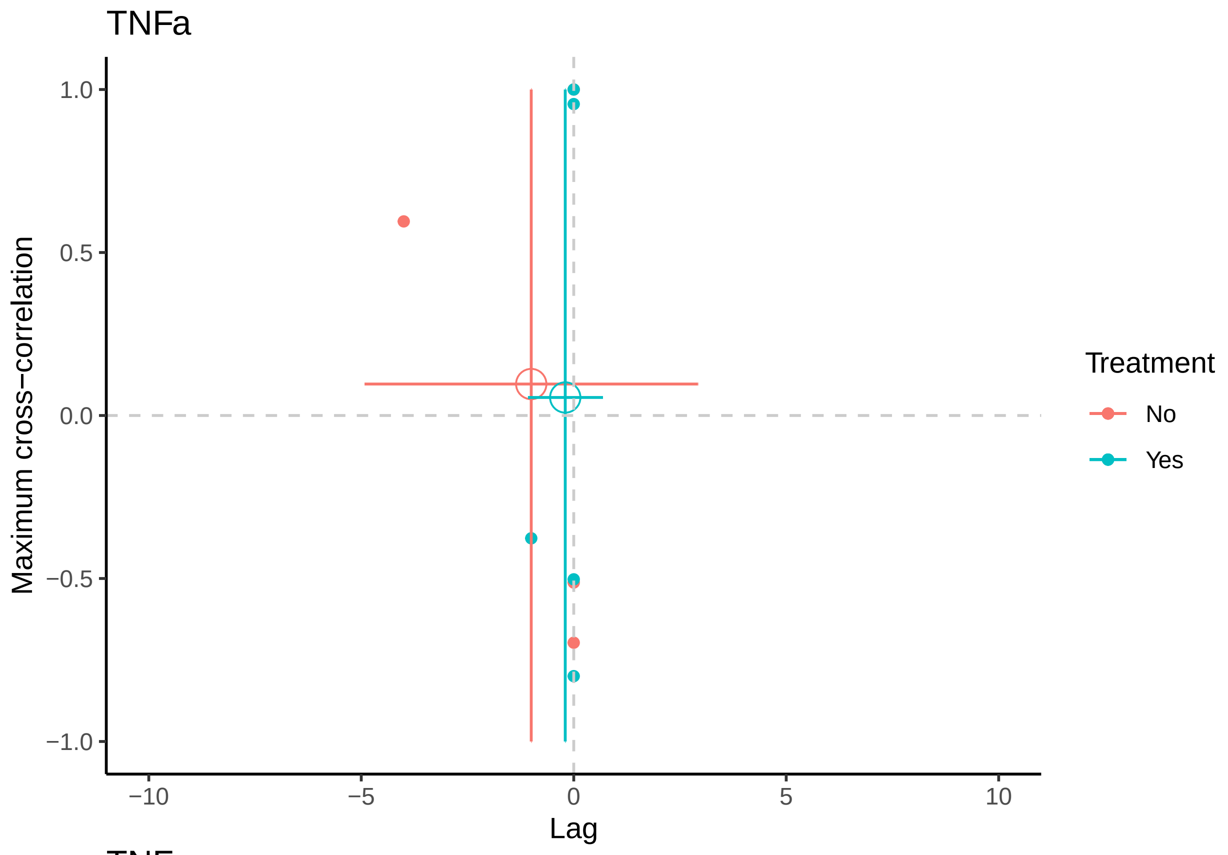
TNFa
Maximum cross−correlation
1.0
0.5
0.0
−0.5
−1.0
−10
−5
0
5
10
Lag
Treatment
No
Yes

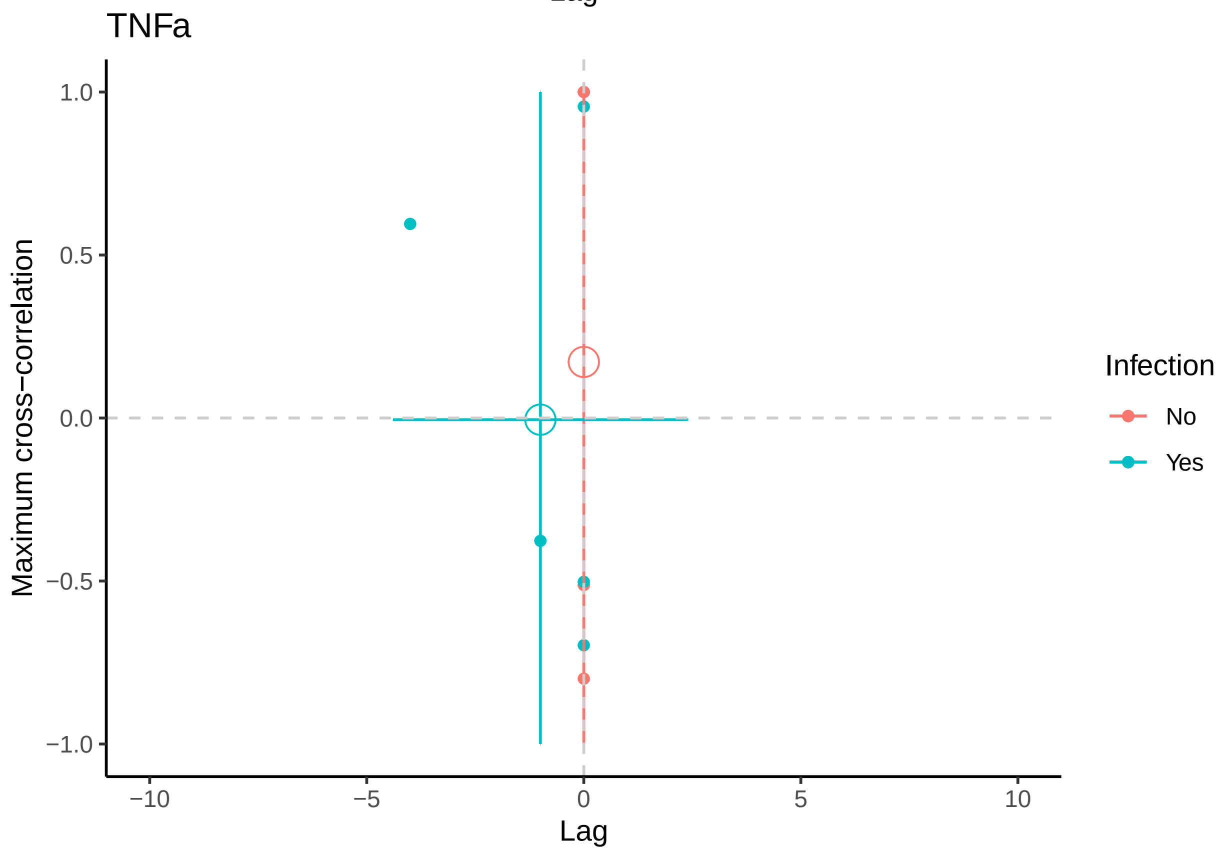
TNFa
Maximum cross−correlation
1.0
0.5
0.0
−0.5
−1.0
−10
−5
0
5
10
Lag
Infection
No
Yes

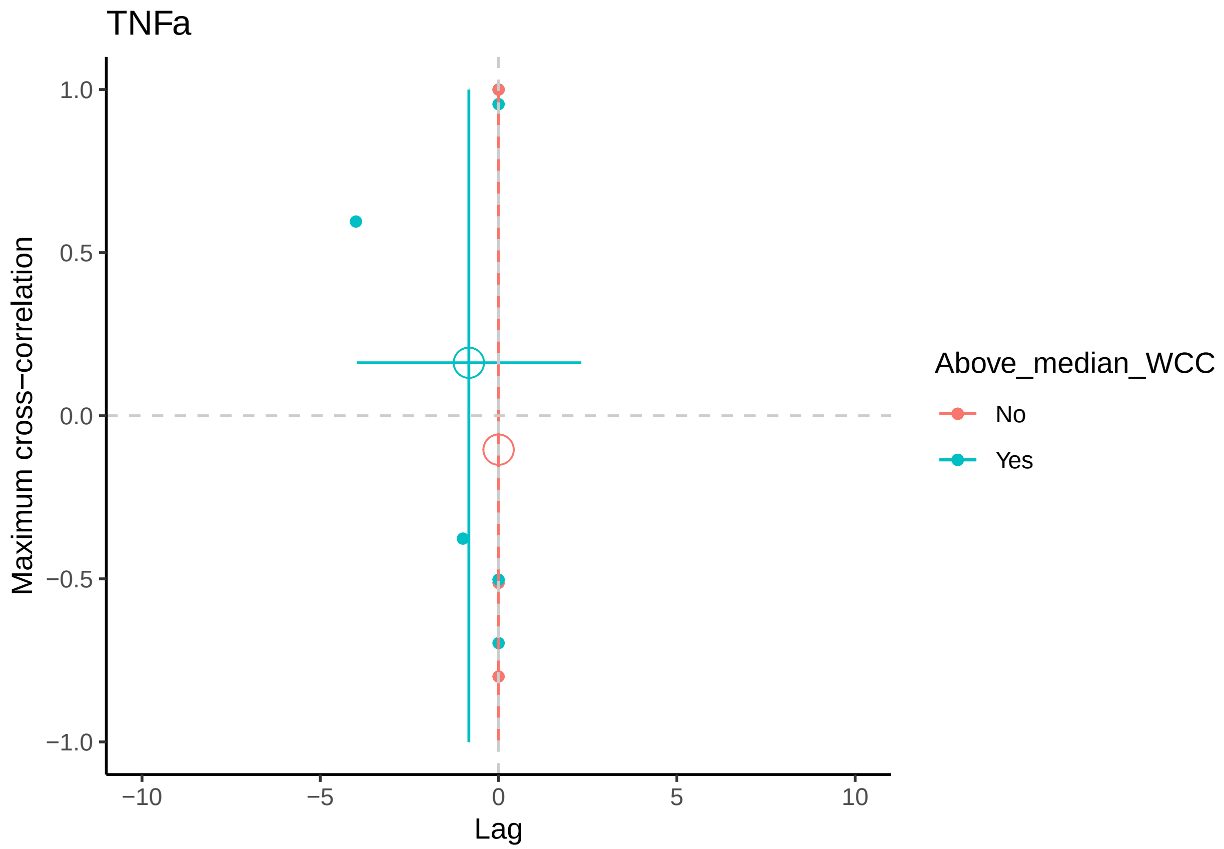
TNFa
Maximum cross−correlation
1.0
0.5
0.0
−0.5
−1.0
−10
−5
0
5
10
Lag
Above_median_WCC
No
Yes

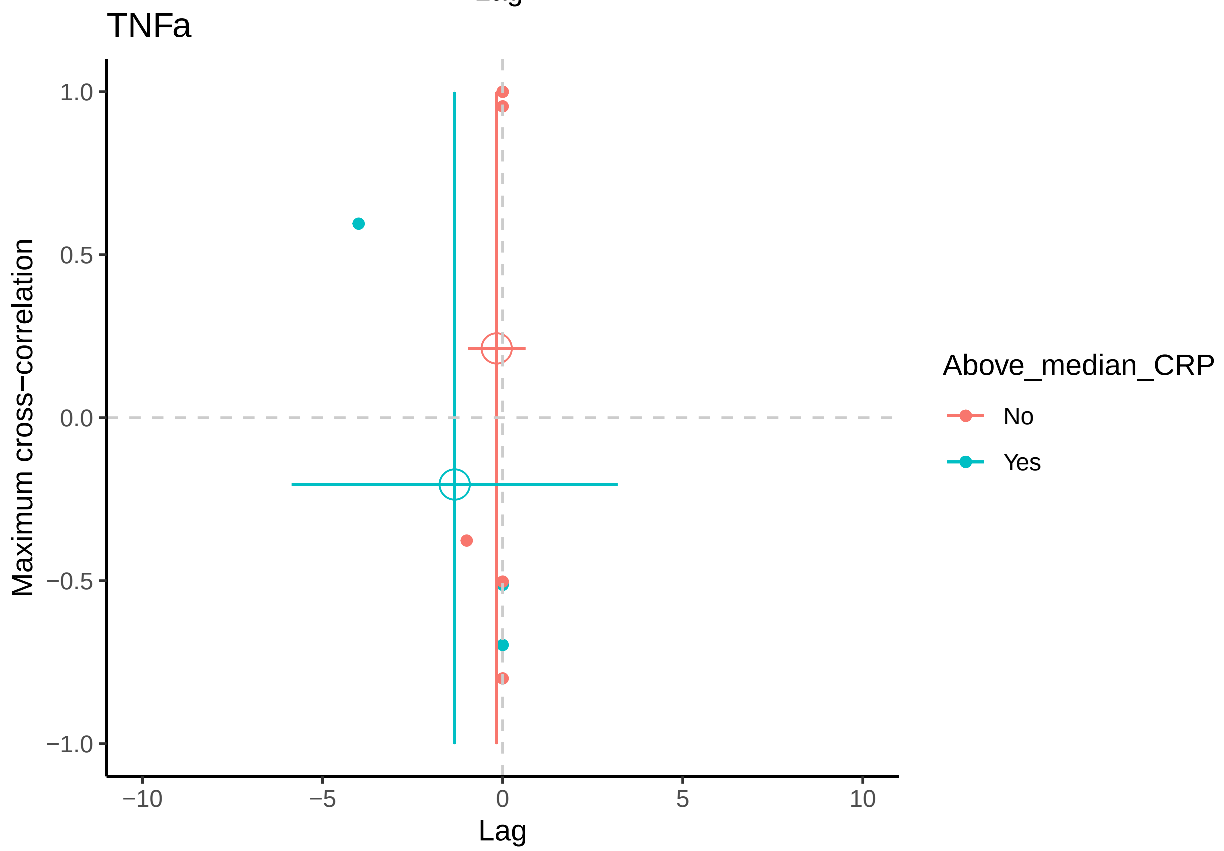
TNFa
Maximum cross−correlation
1.0
0.5
0.0
−0.5
−1.0
−10
−5
0
5
10
Lag
Above_median_CRP
No
Yes

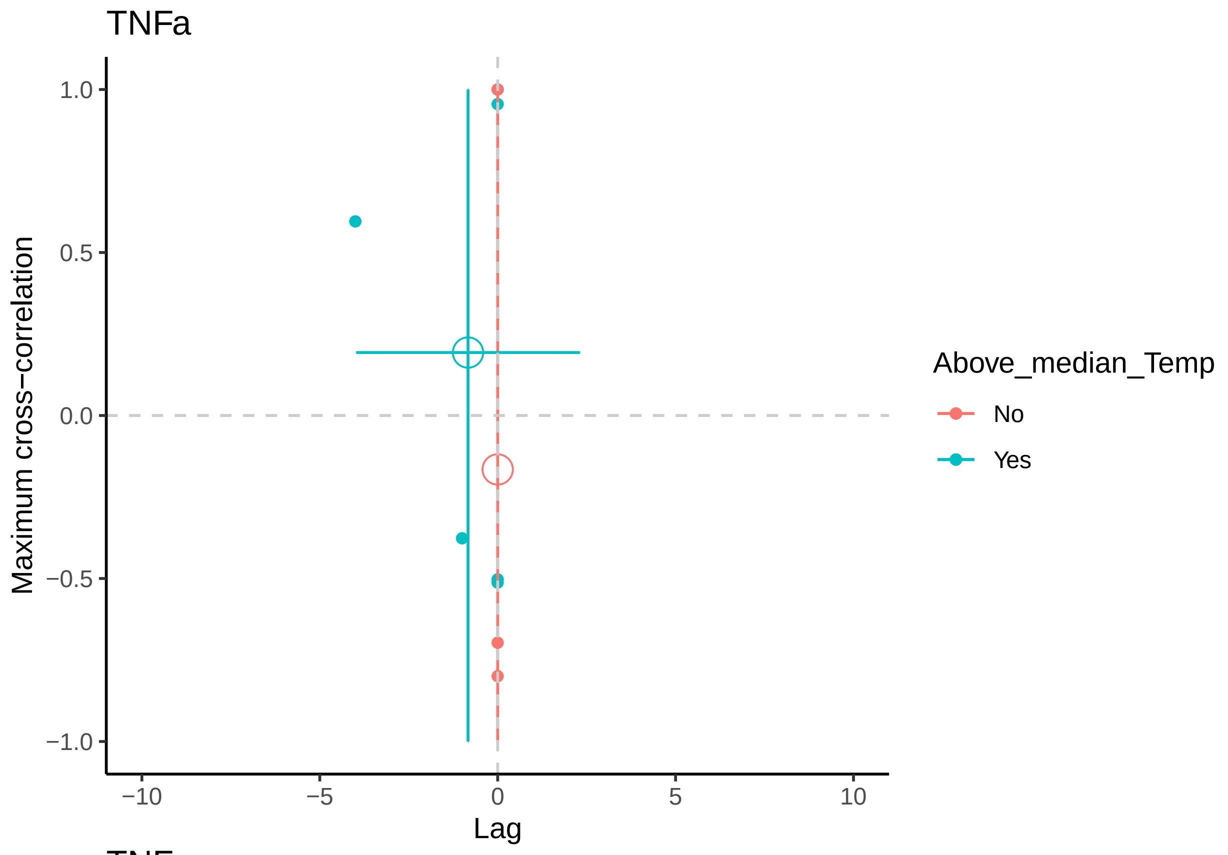
TNFa
Maximum cross−correlation
1.0
0.5
0.0
−0.5
−1.0
−10
−5
0
5
10
Lag
Above_median_Temp
No
Yes

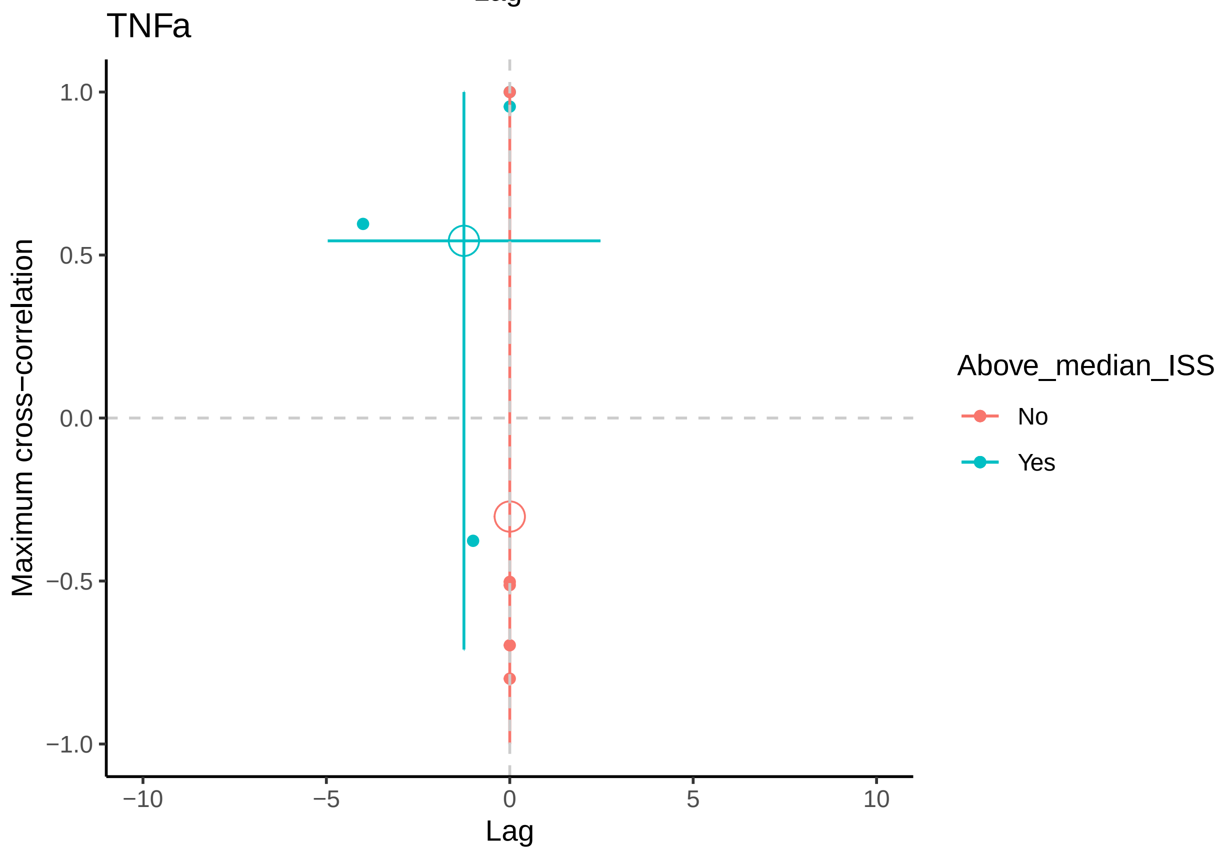
TNFa
Maximum cross−correlation
1.0
0.5
0.0
−0.5
−1.0
−10
−5
0
5
10
Lag
Above_median_ISS
No
Yes

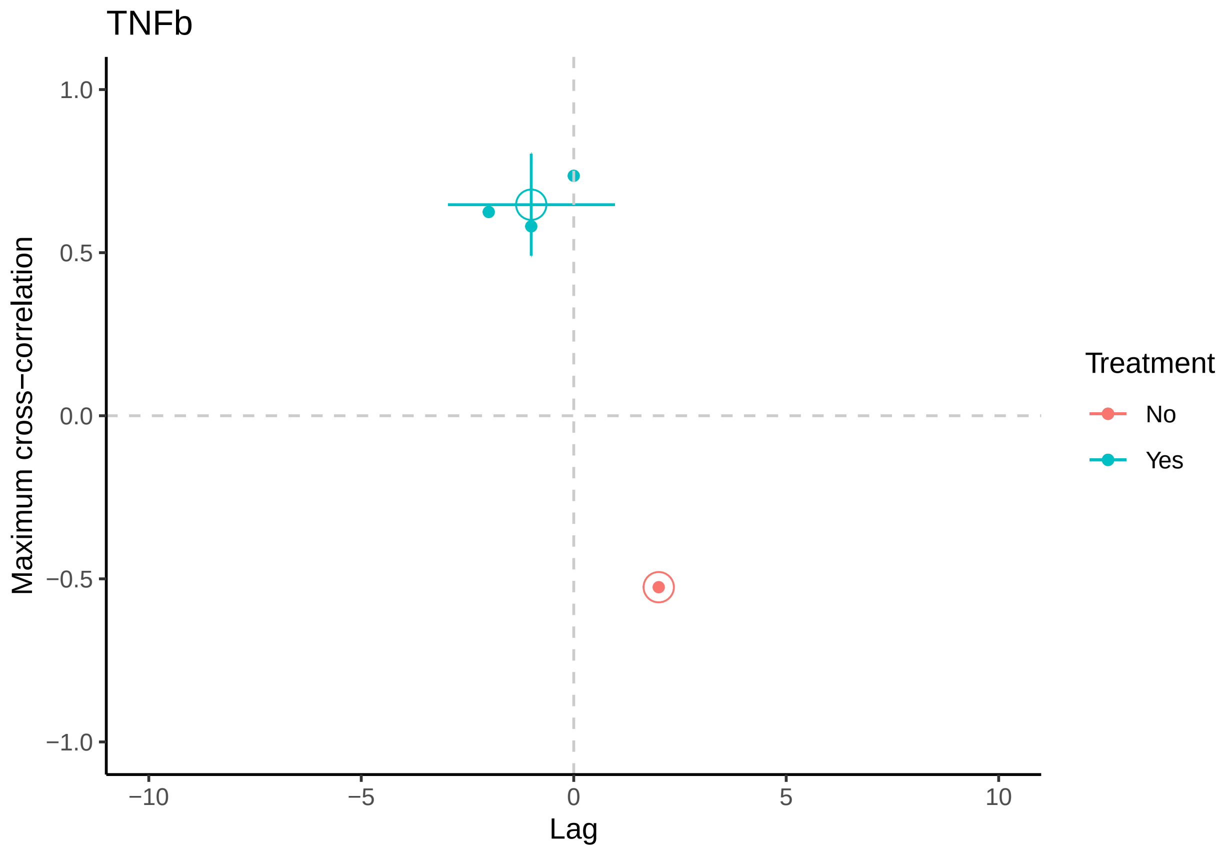
TNFb
Maximum cross−correlation
Lag
Treatment
No
Yes

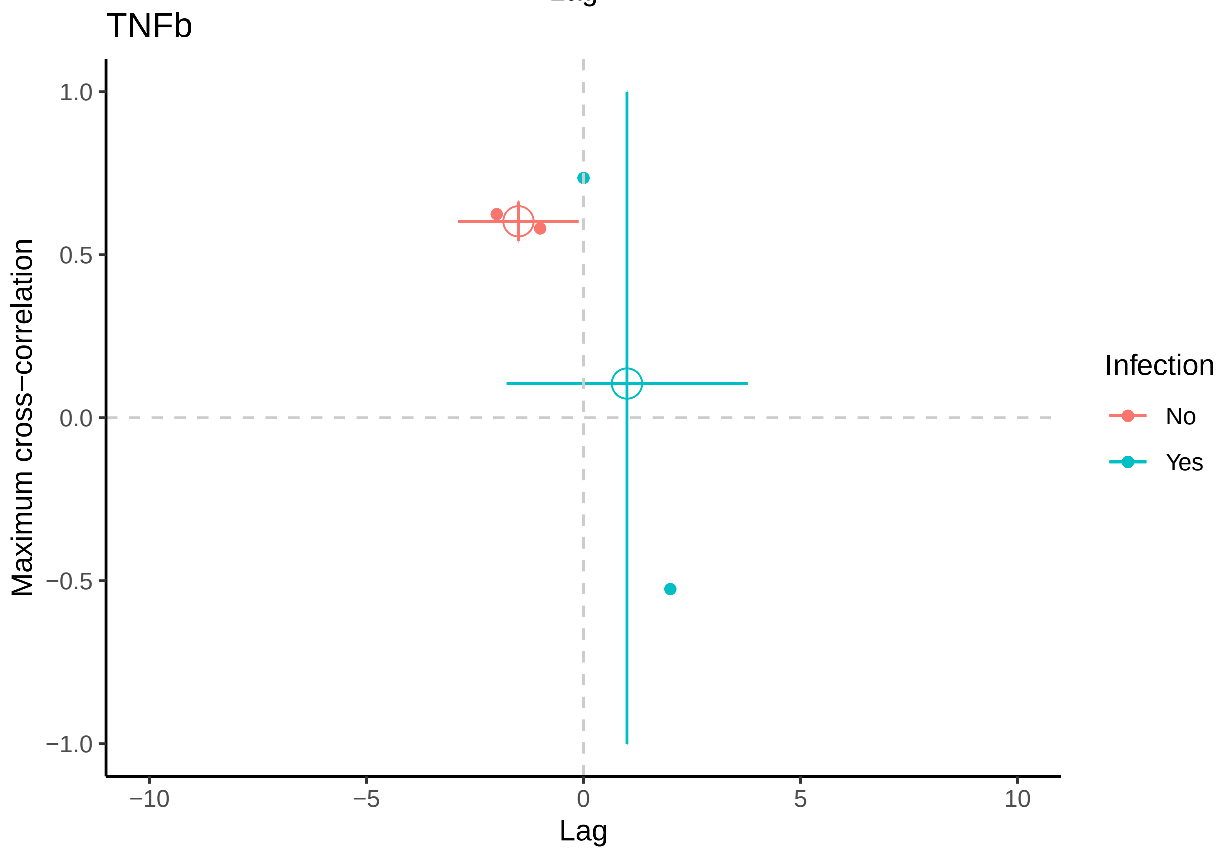
TNFb
Maximum cross−correlation
Lag
Infection
No
Yes

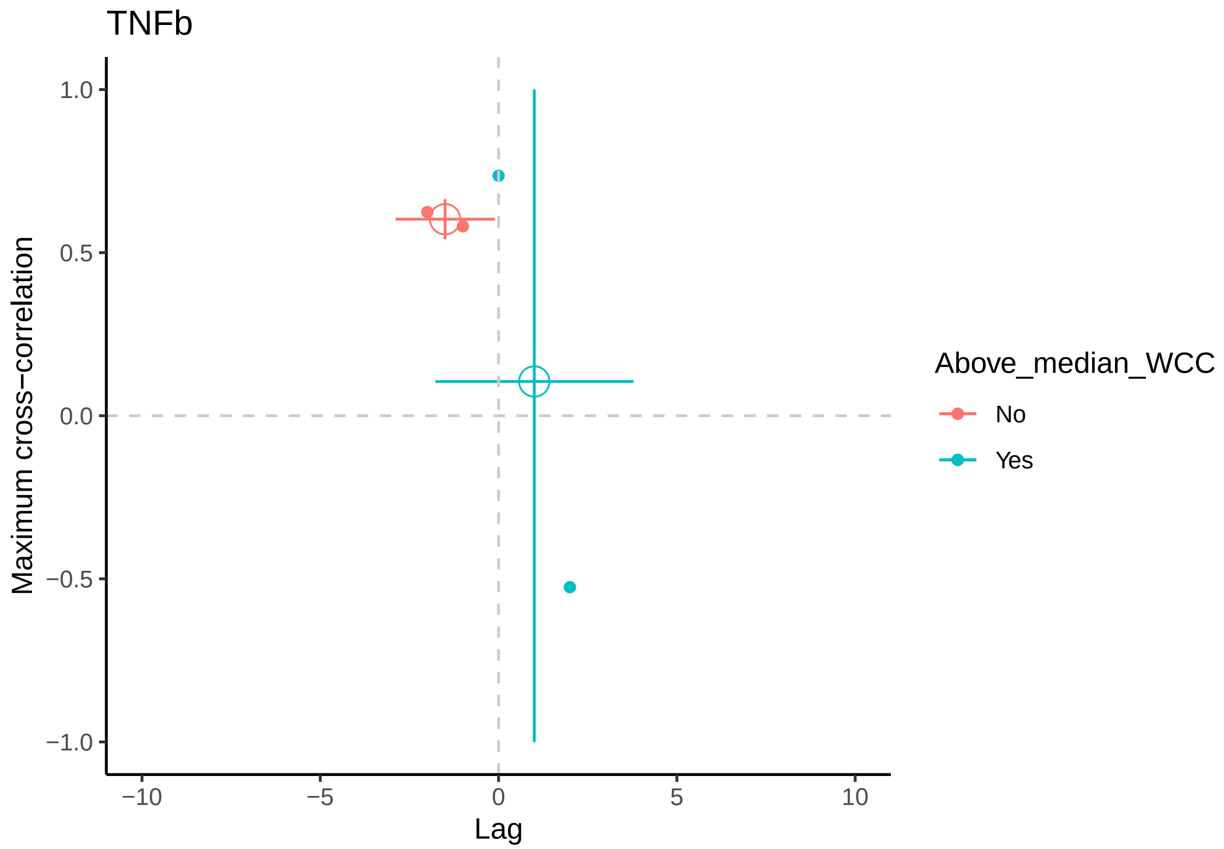
TNFb
Maximum cross−correlation
1.0
0.5
0.0
−0.5
−1.0
−10
−5
0
5
10
Lag
Above_median_WCC
No
Yes

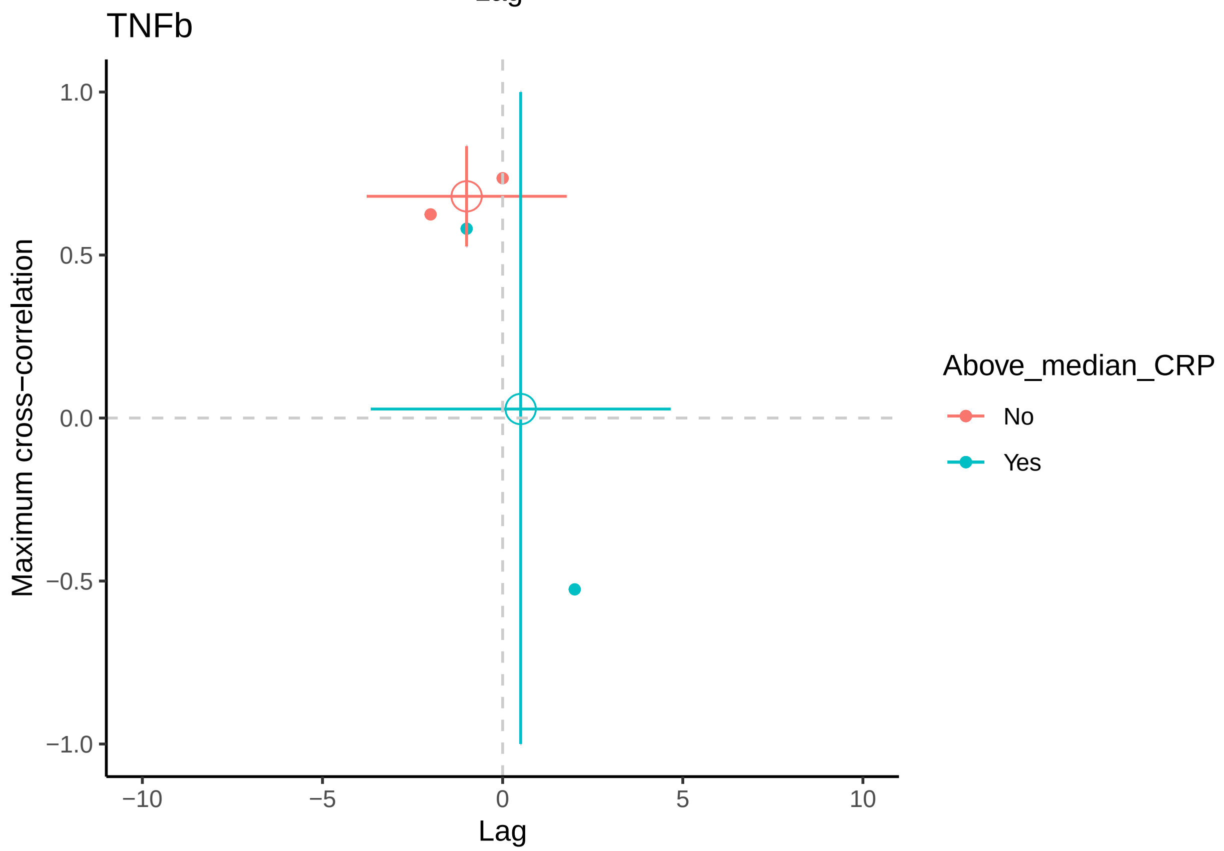
TNFb
Maximum cross−correlation
1.0
0.5
0.0
−0.5
−1.0
−10
−5
0
5
10
Lag
Above_median_CRP
No
Yes

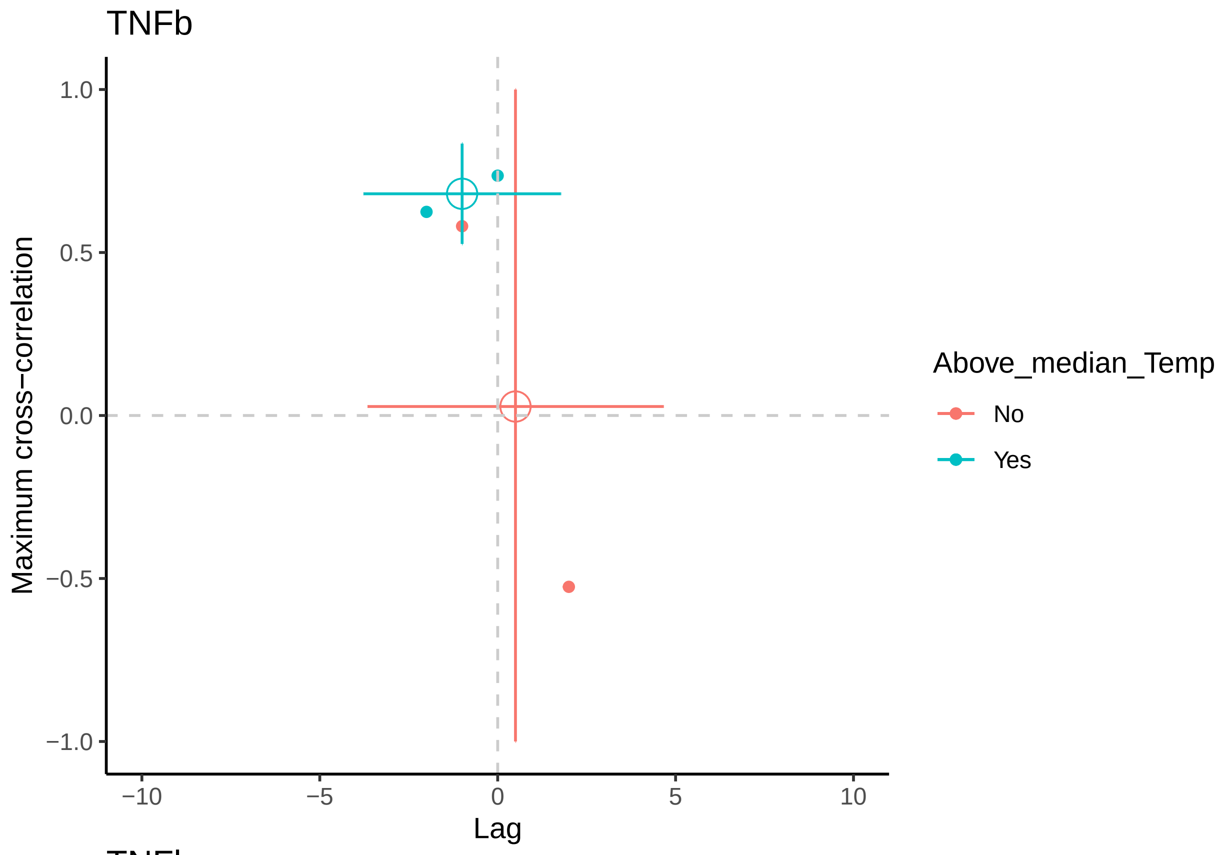
TNFb
Maximum cross−correlation
1.0
0.5
0.0
−0.5
−1.0
−10
−5
0
5
10
Lag
Above_median_Temp
No
Yes

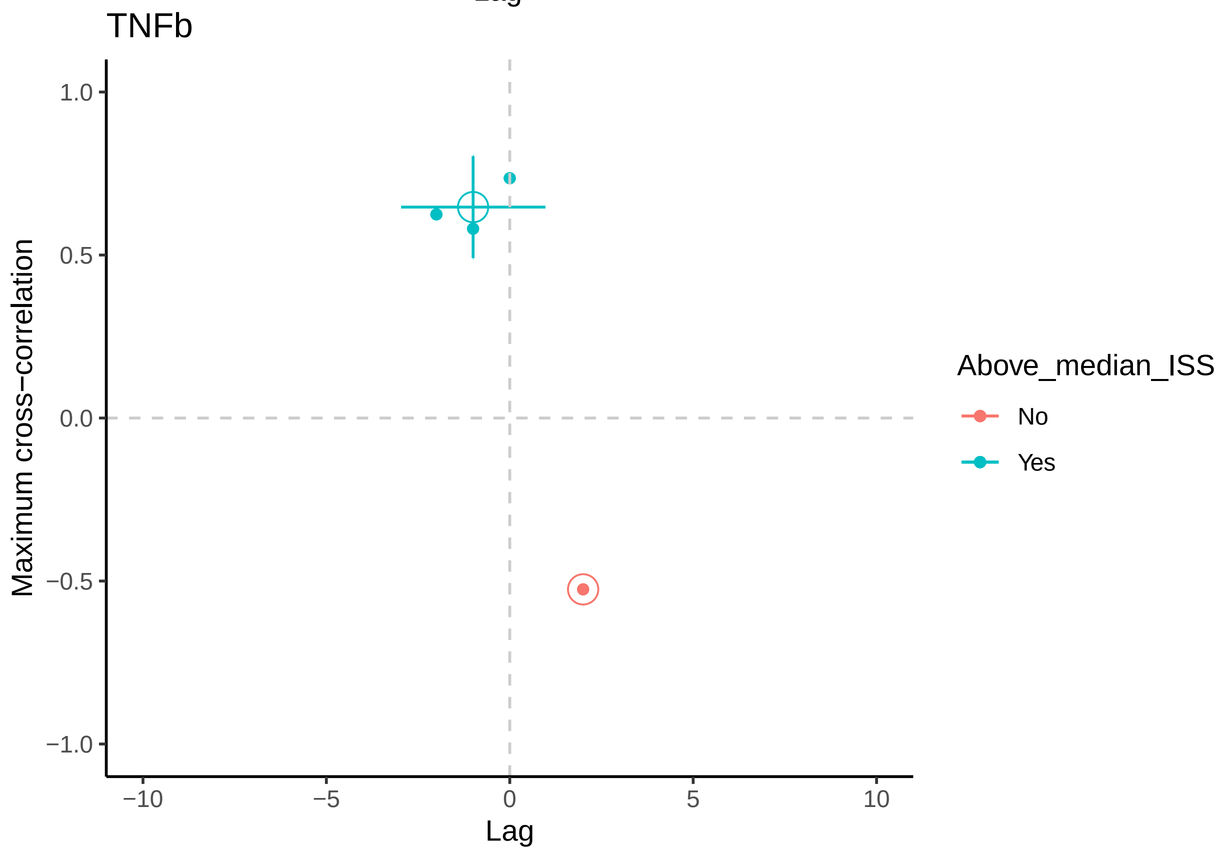
TNFb
Maximum cross−correlation
1.0
0.5
0.0
−0.5
−1.0
−10
−5
0
5
10
Lag
Above_median_ISS
No
Yes

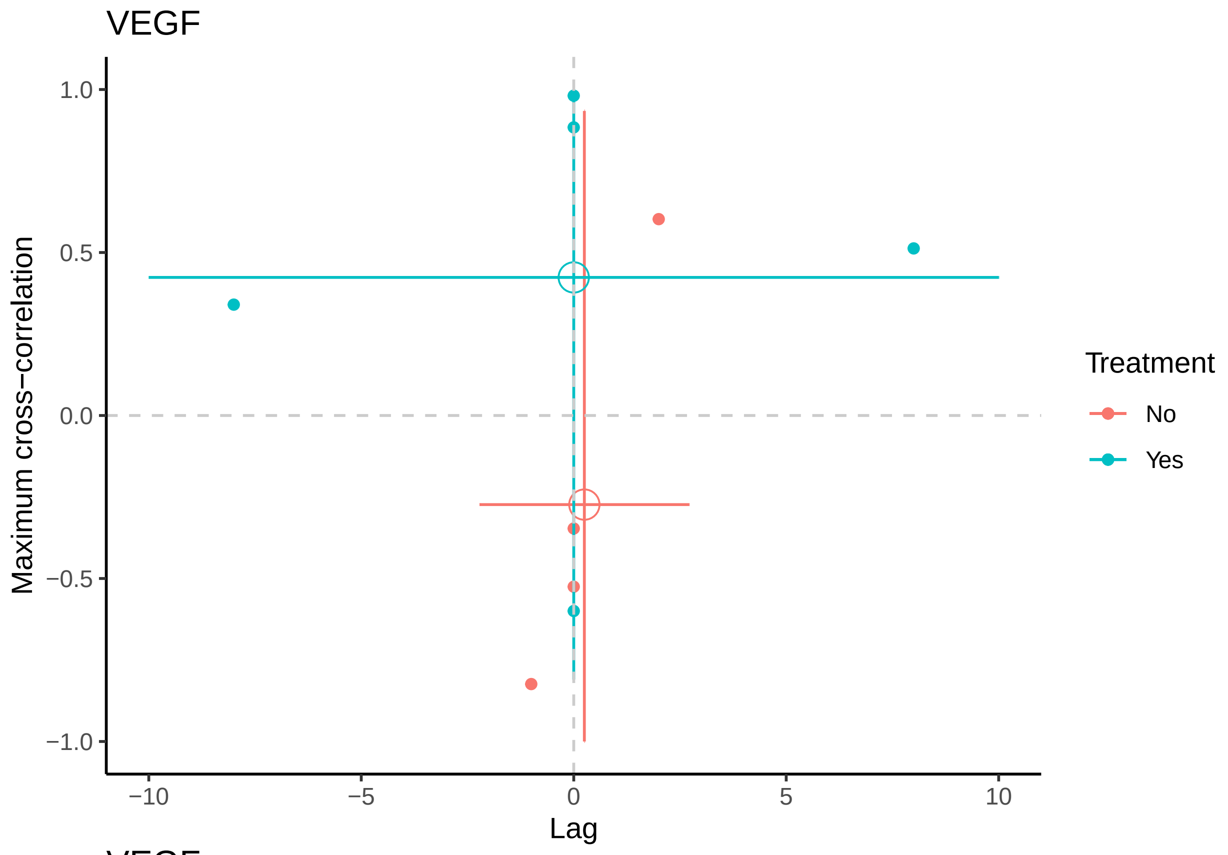
VEGF
Maximum cross−correlation
1.0
0.5
0.0
−0.5
−1.0
−10
−5
0
5
10
Lag
Treatment
No
Yes

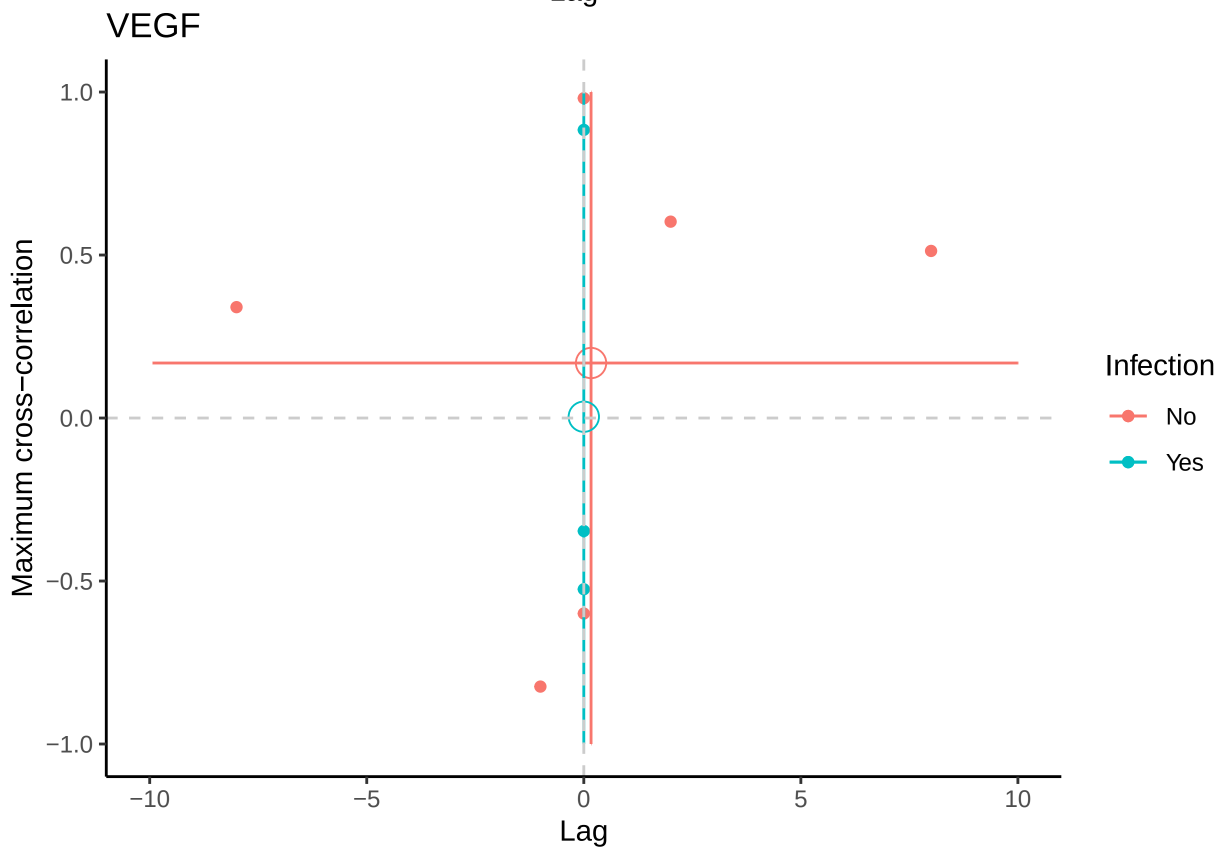
VEGF
Maximum cross−correlation
1.0
0.5
0.0
−0.5
−1.0
−10
−5
0
5
10
Lag
Infection
No
Yes

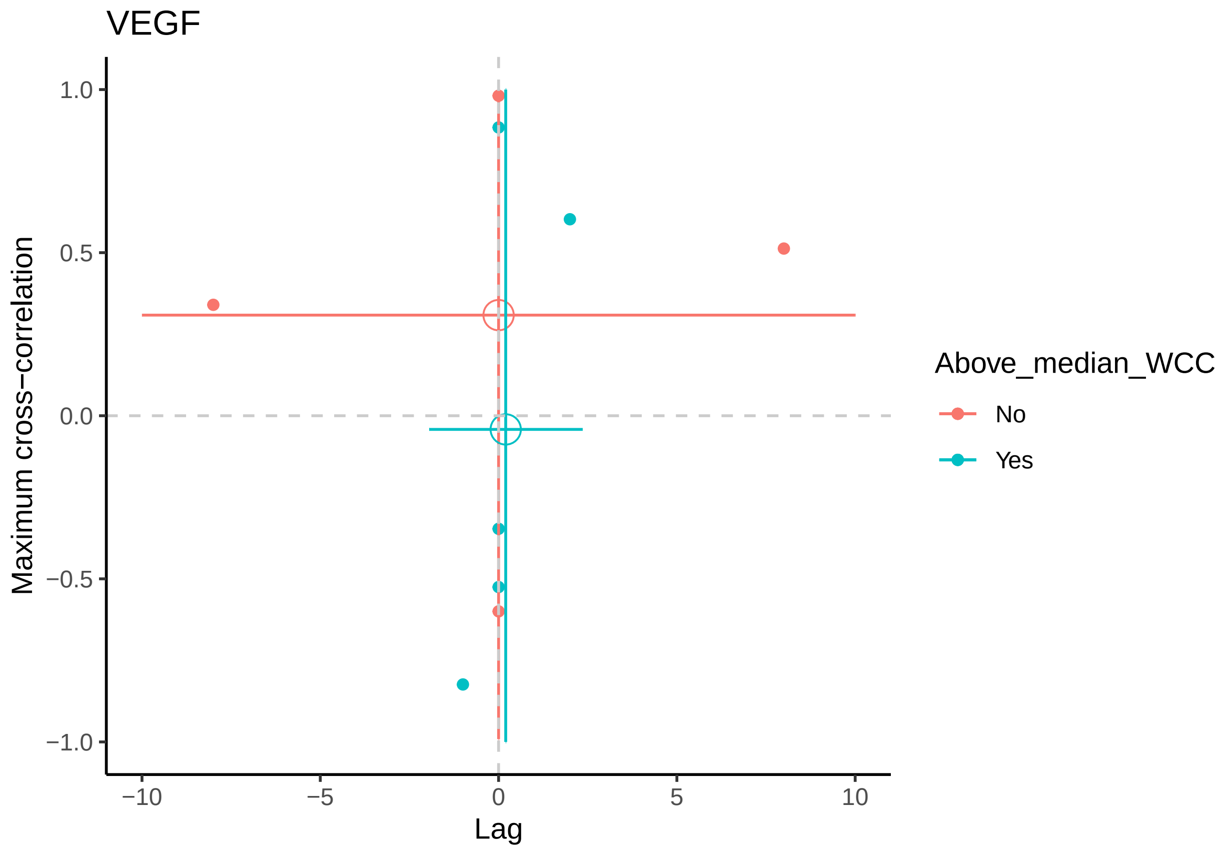
VEGF
Maximum cross−correlation
1.0
0.5
0.0
−0.5
−1.0
−10
−5
0
5
10
Lag
Above_median_WCC
No
Yes

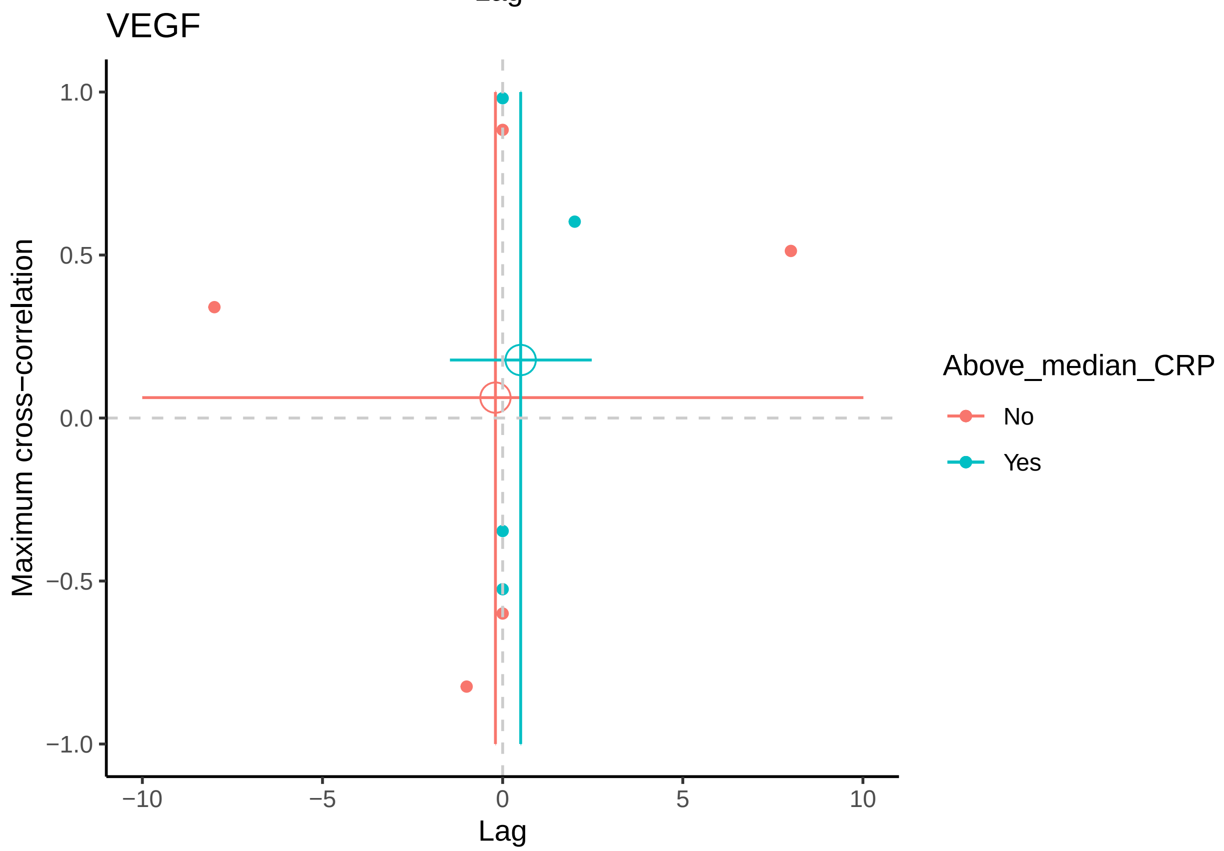
VEGF
Maximum cross−correlation
1.0
0.5
0.0
−0.5
−1.0
−10
−5
0
5
10
Lag
Above_median_CRP
No
Yes

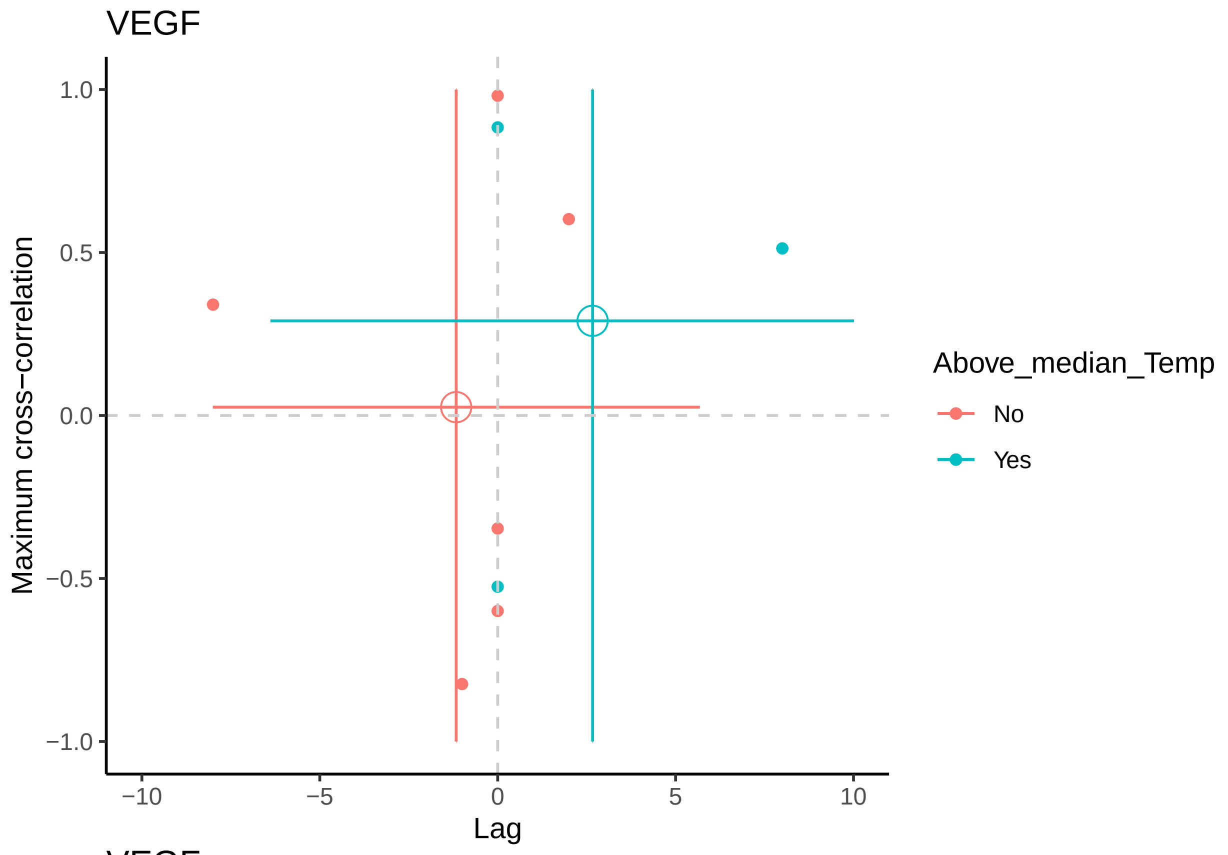

VEGF
Maximum cross−correlation
1.0
0.5
0.0
−0.5
−1.0
−10
−5
0
5
10
Lag
Above_median_Temp
No
Yes

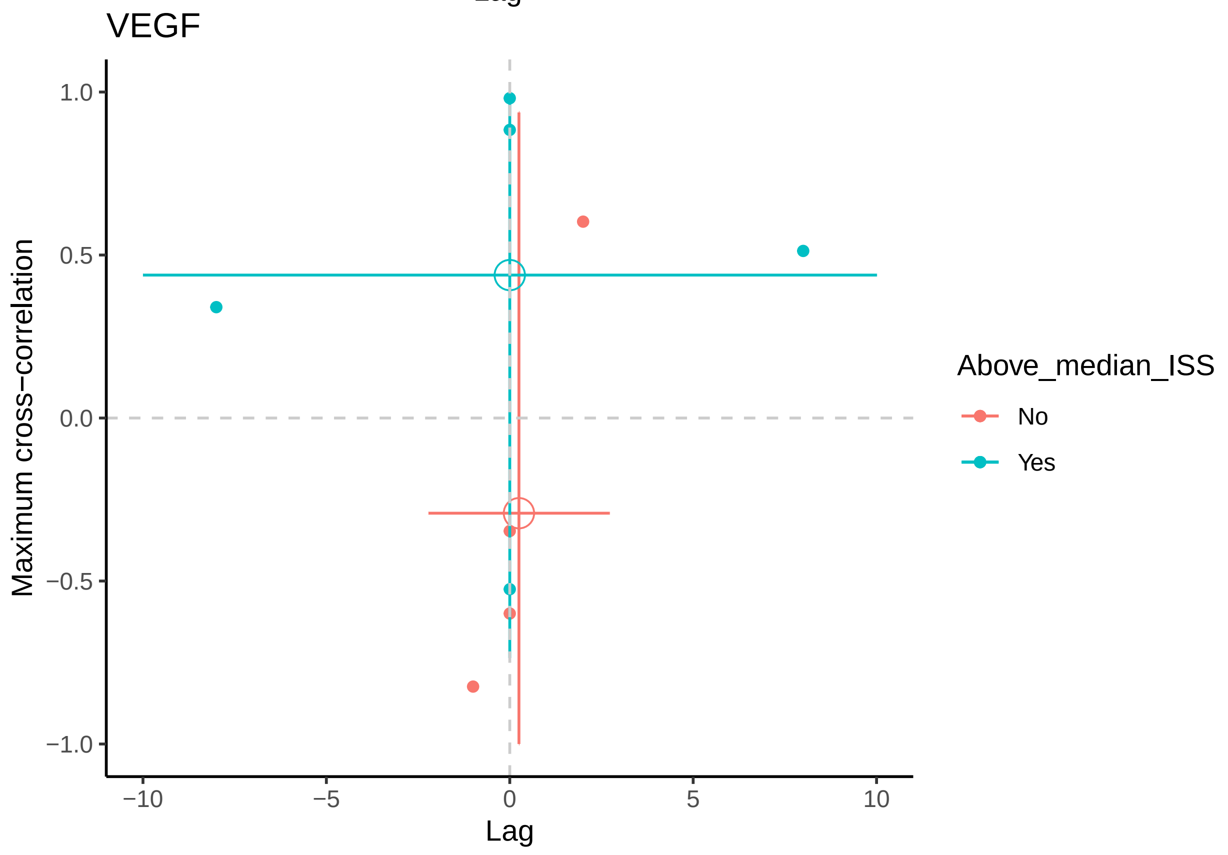

VEGF
Maximum cross−correlation
1.0
0.5
0.0
−0.5
−1.0
−10
−5
0
5
10
Lag
Above_median_ISS
No
Yes

```r
for (patient in 1:20) {
  local_data <- output[output$Patient == patient, ]
  local_data <- local_data[local_data$Included == "Yes", ]

  cap <- ""
  for (var in 7:12) {
    cap <- paste0(cap, colnames(local_data)[var], ": ",
                  local_data[local_data$Patient == patient, var][1], ".")
    if (var != 12) {
      cap <- paste0(cap, "\n")
    }
  }

  p5 <- ggplot(data = local_data, aes(x = Lag, y = Maximum_value)) +
    geom_point() +
    theme_classic() +
    ggtitle(paste("Patient", patient)) +
    ylab("Maximum cross-correlation") +
    geom_hline(yintercept = 0, linetype = "dashed", color = "grey80") +
    geom_vline(xintercept = 0, linetype = "dashed", color = "grey80") +
    xlim(-10, 10) +
    ylim(-1, 1) +
    labs(caption = cap)
  print(p5)
}
```

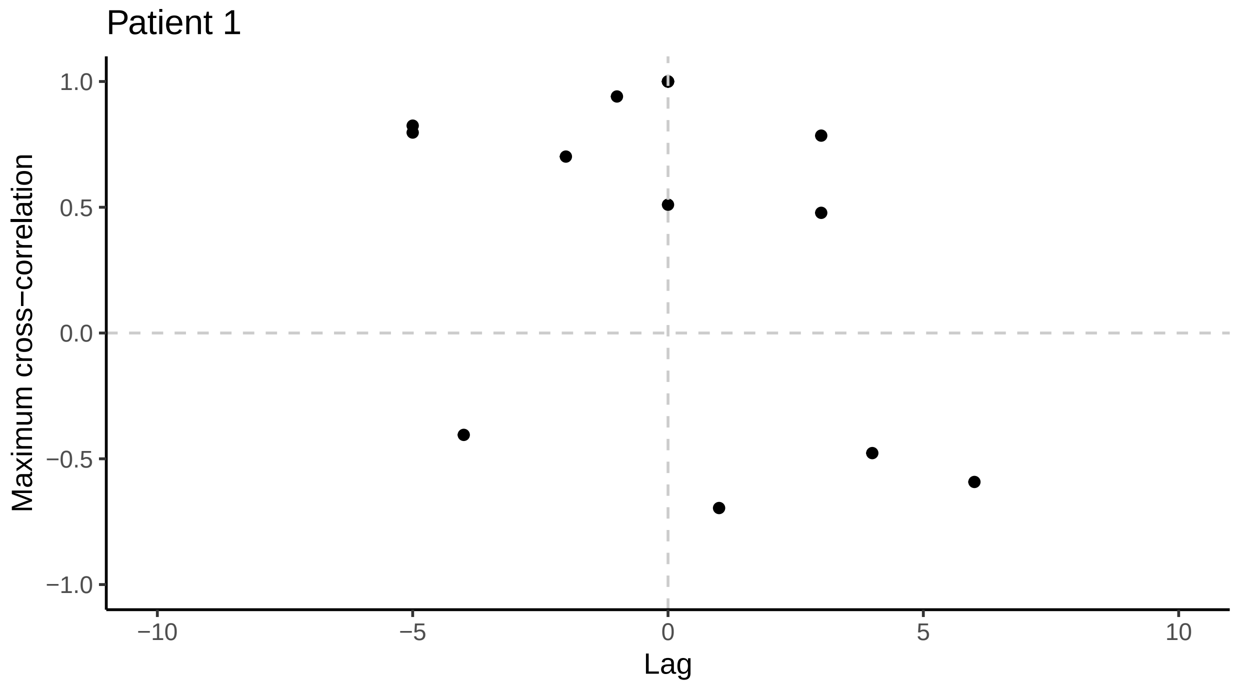

Treatment: No.
Infection: No.
Above_median_WCC: Yes.
Above_median_CRP: No.
Above_median_Temp: Yes.
Above_median_ISS: Yes.

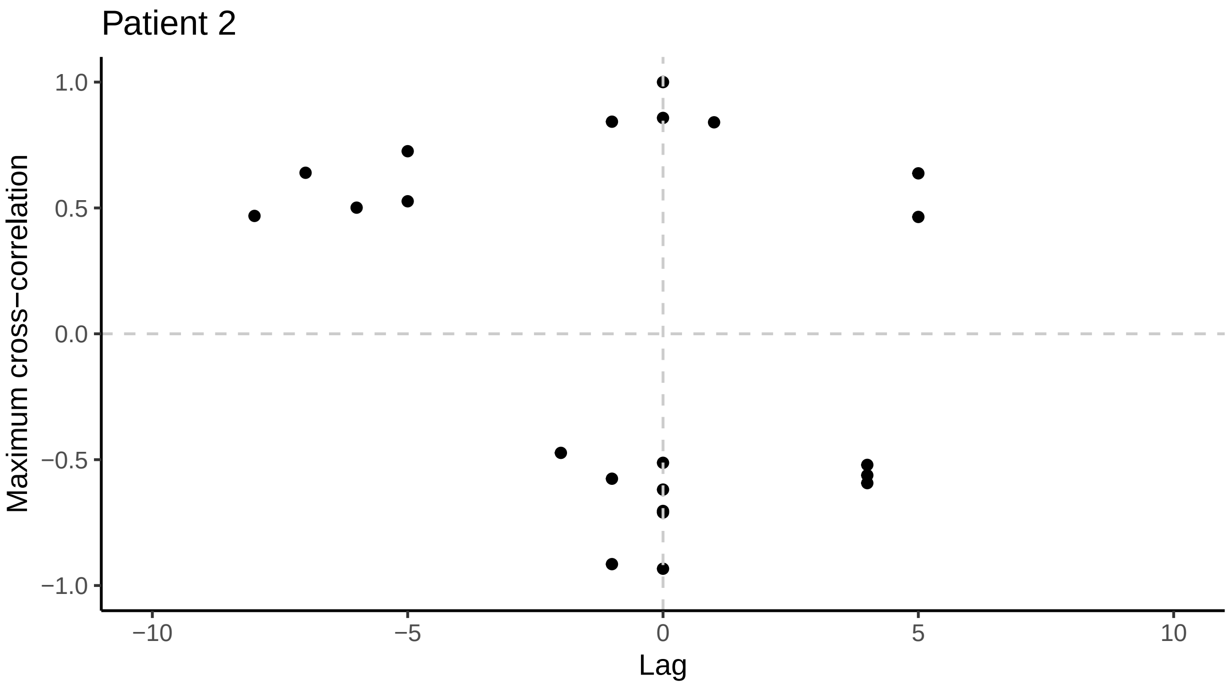

Treatment: No.
Infection: No.
Above_median_WCC: No.
Above_median_CRP: Yes.
Above_median_Temp: Yes.
Above_median_ISS: No.

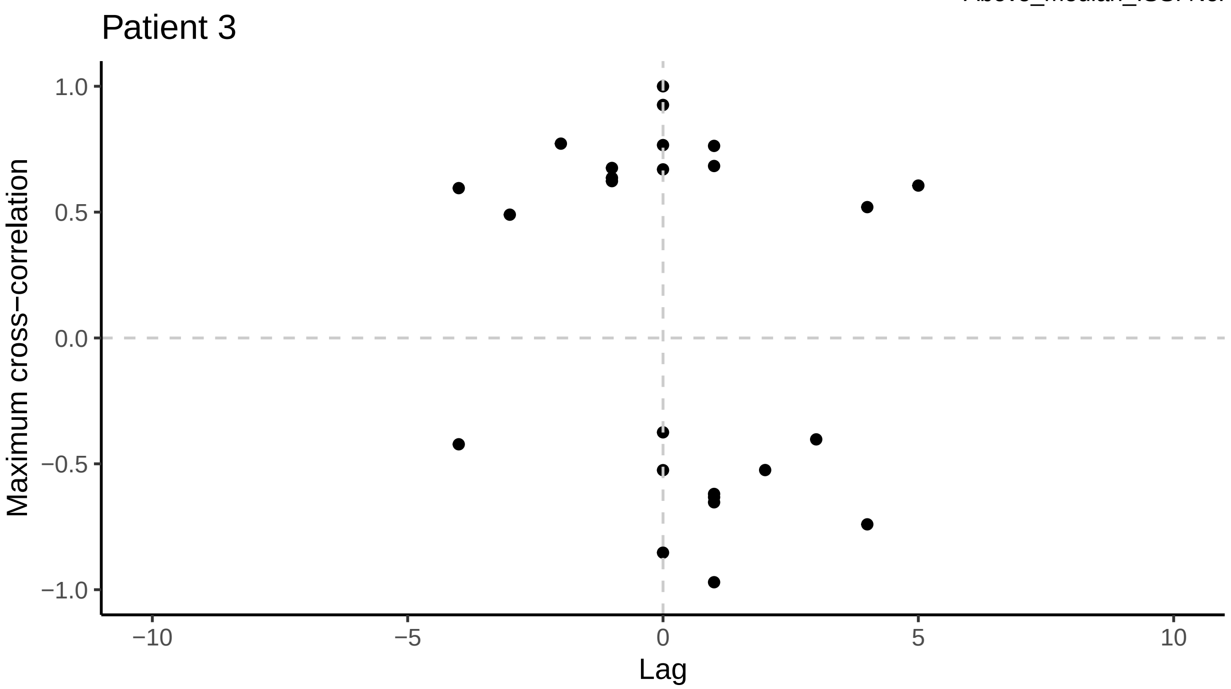

Treatment: No.
Infection: Yes.
Above_median_WCC: Yes.
Above_median_CRP: Yes.
Above_median_Temp: Yes.
Above_median_ISS: Yes.

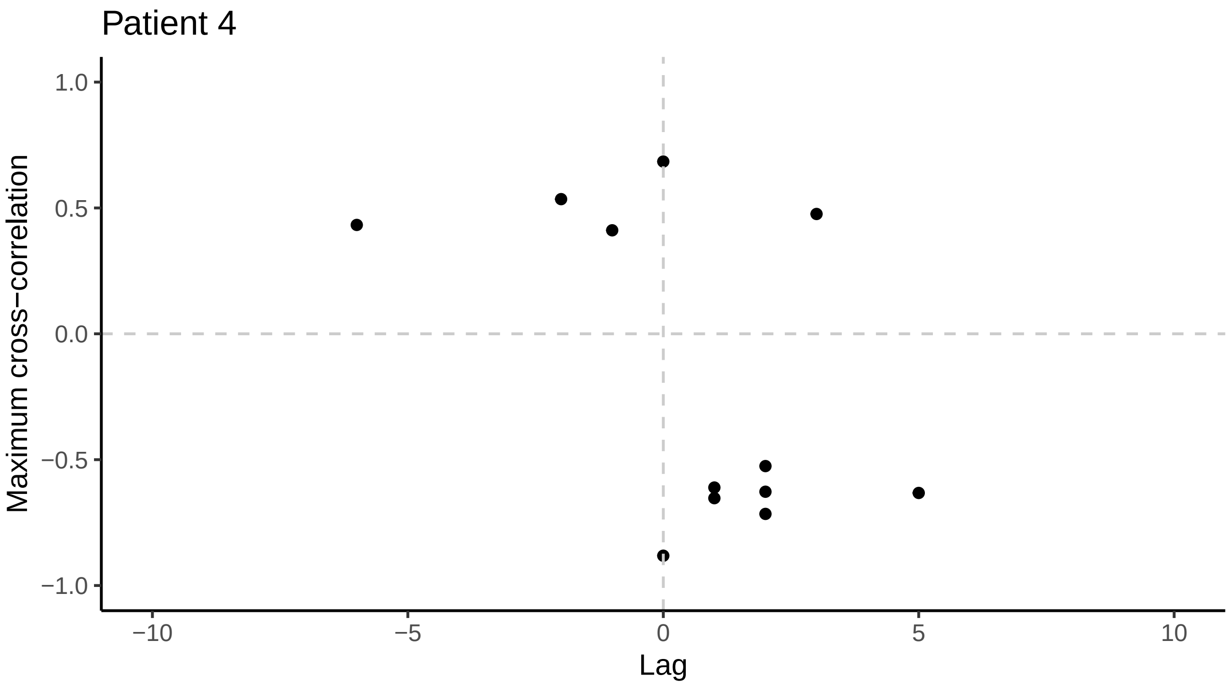

Treatment: No.
Infection: Yes.
Above_median_WCC: Yes.
Above_median_CRP: Yes.
Above_median_Temp: No.
Above_median_ISS: No.

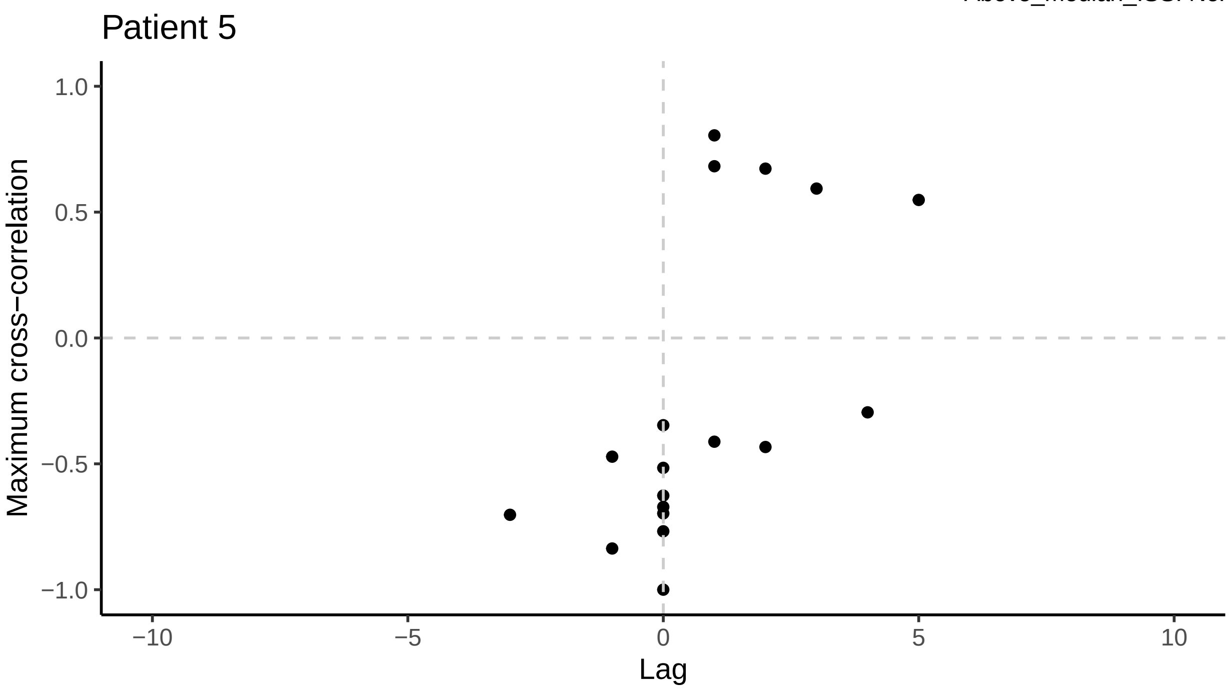

Treatment: No.
Infection: Yes.
Above_median_WCC: Yes.
Above_median_CRP: Yes.
Above_median_Temp: No.
Above_median_ISS: No.

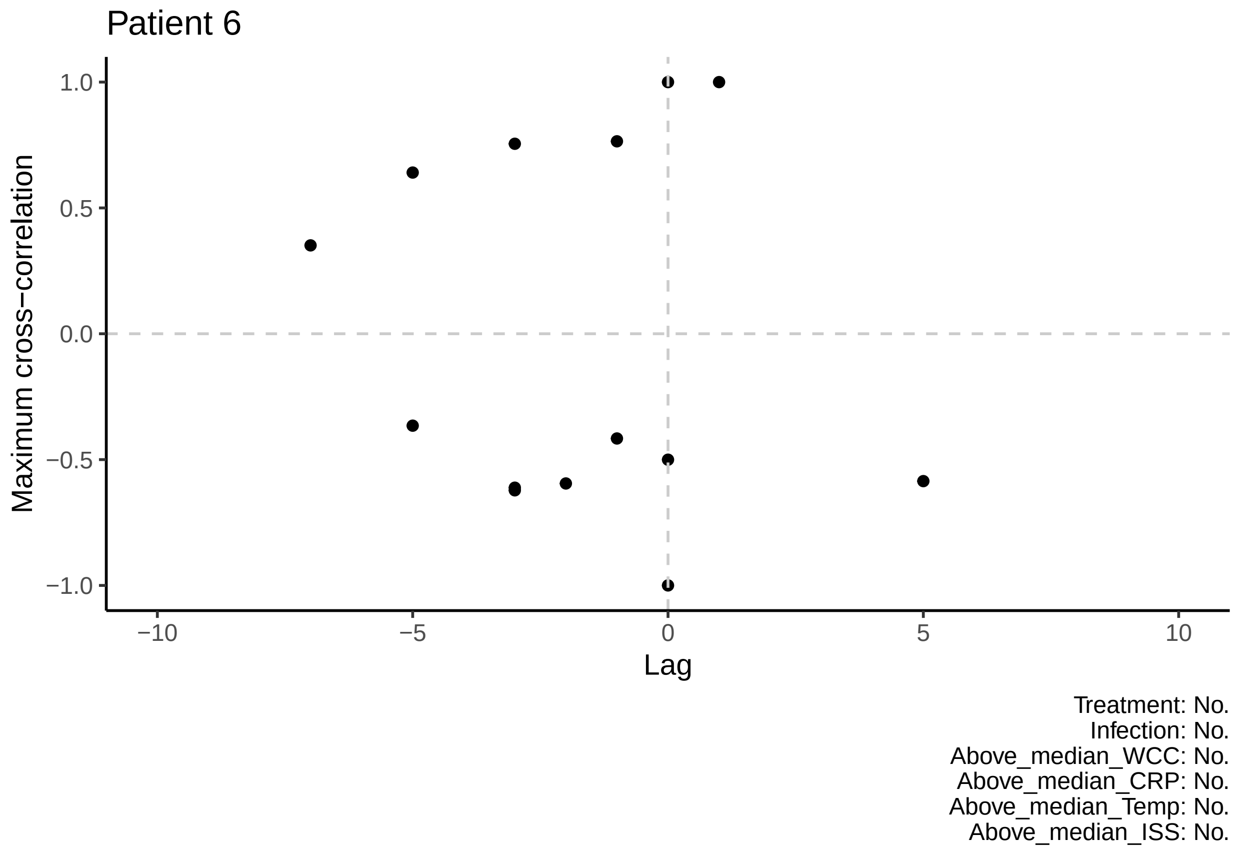
Patient 6
Maximum cross−correlation
1.0
0.5
0.0
−0.5
−1.0
−10
−5
0
5
10
Lag
Treatment: No.
Infection: No.
Above_median_WCC: No.
Above_median_CRP: No.
Above_median_Temp: No.
Above_median_ISS: No.

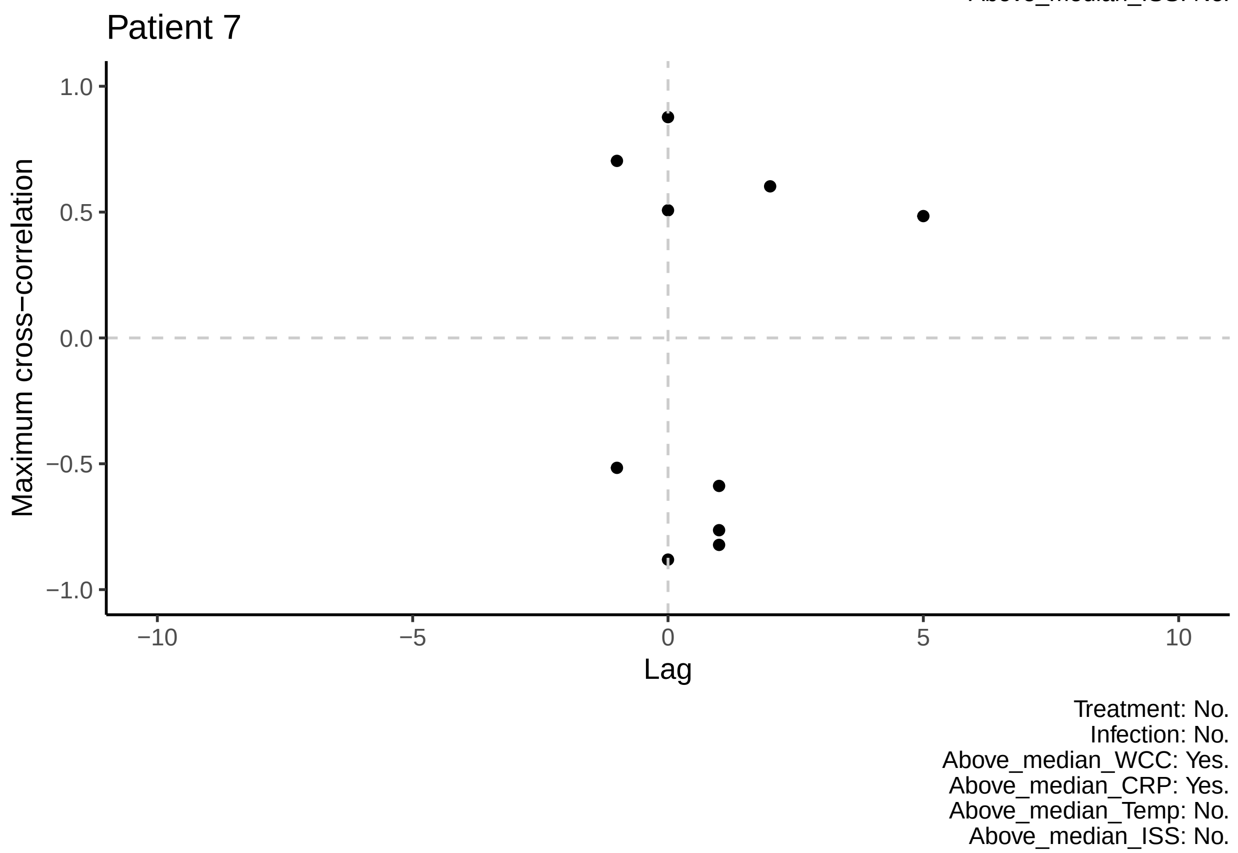
Patient 7
Maximum cross−correlation
1.0
0.5
0.0
−0.5
−1.0
−10
−5
0
5
10
Lag
Treatment: No.
Infection: No.
Above_median_WCC: Yes.
Above_median_CRP: Yes.
Above_median_Temp: No.
Above_median_ISS: No.

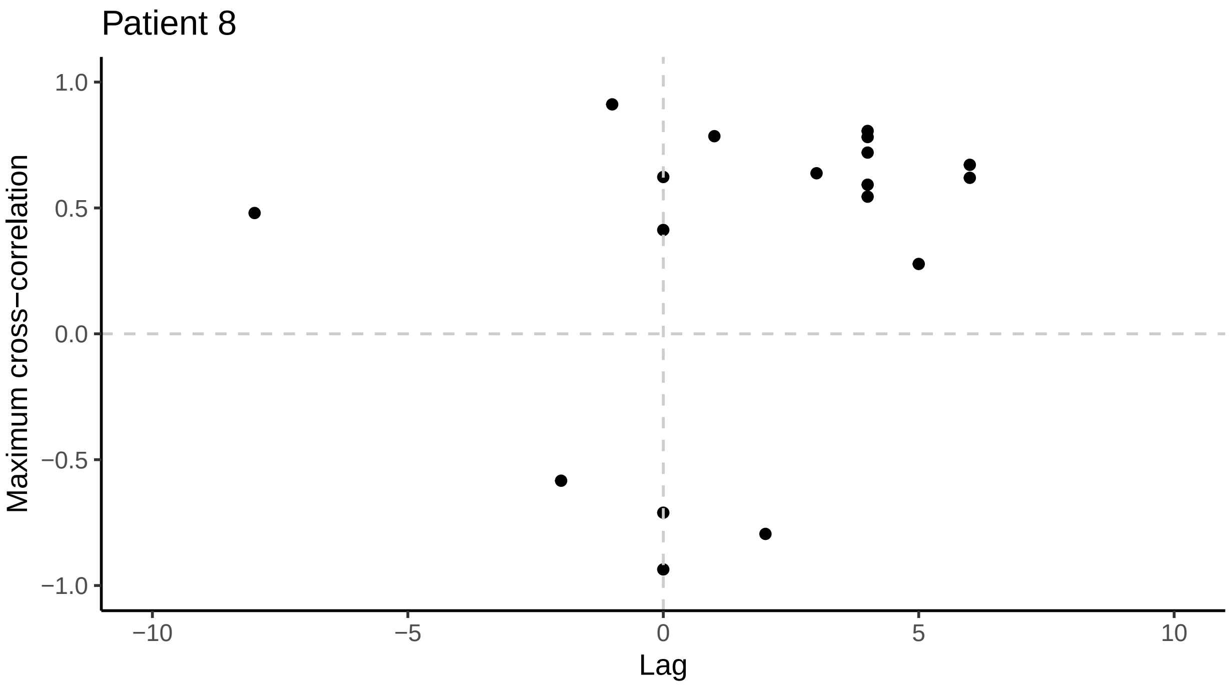

Treatment: No.
Infection: No.
Above_median_WCC: No.
Above_median_CRP: No.
Above_median_Temp: Yes.
Above_median_ISS: No.

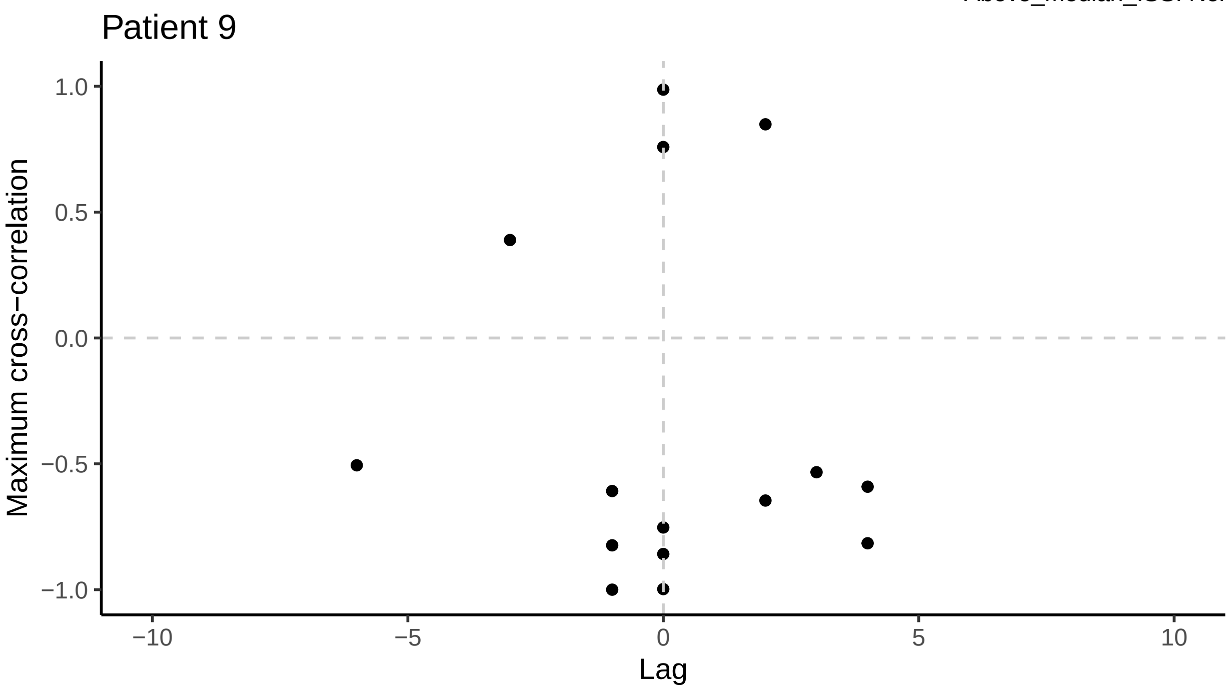

Treatment: No.
Infection: No.
Above_median_WCC: Yes.
Above_median_CRP: No.
Above_median_Temp: No.
Above_median_ISS: No.

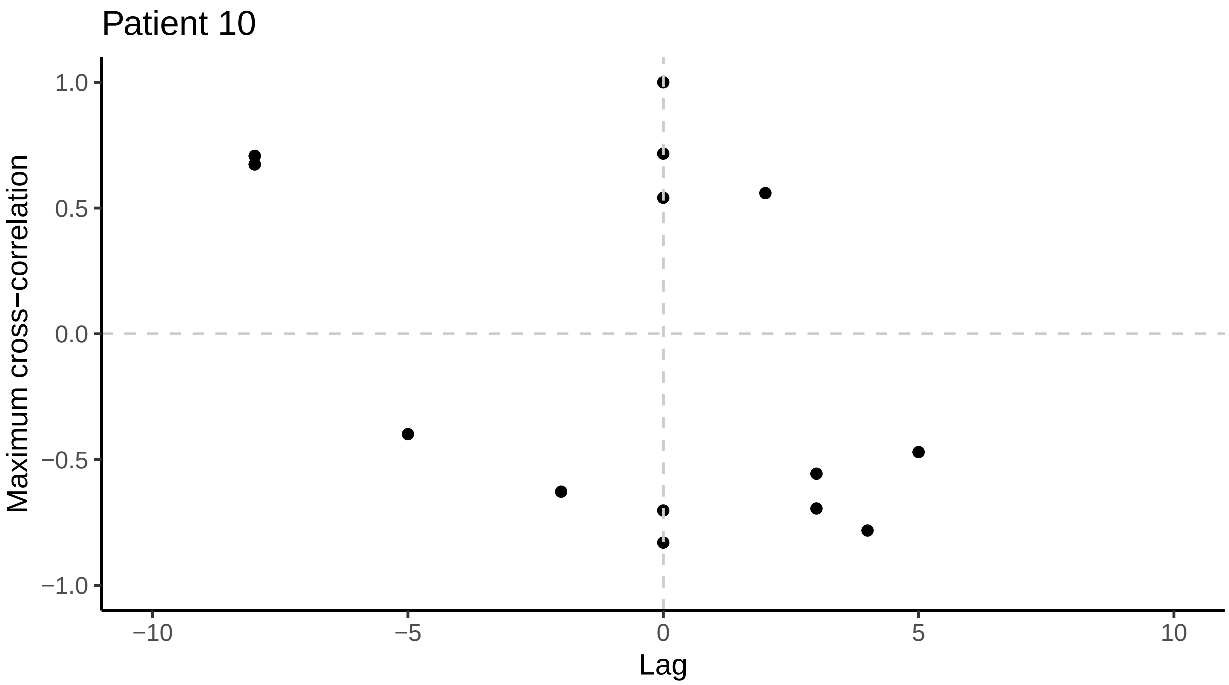

Treatment: No.
Infection: Yes.
Above_median_WCC: No.
Above_median_CRP: Yes.
Above_median_Temp: No.
Above_median_ISS: No.

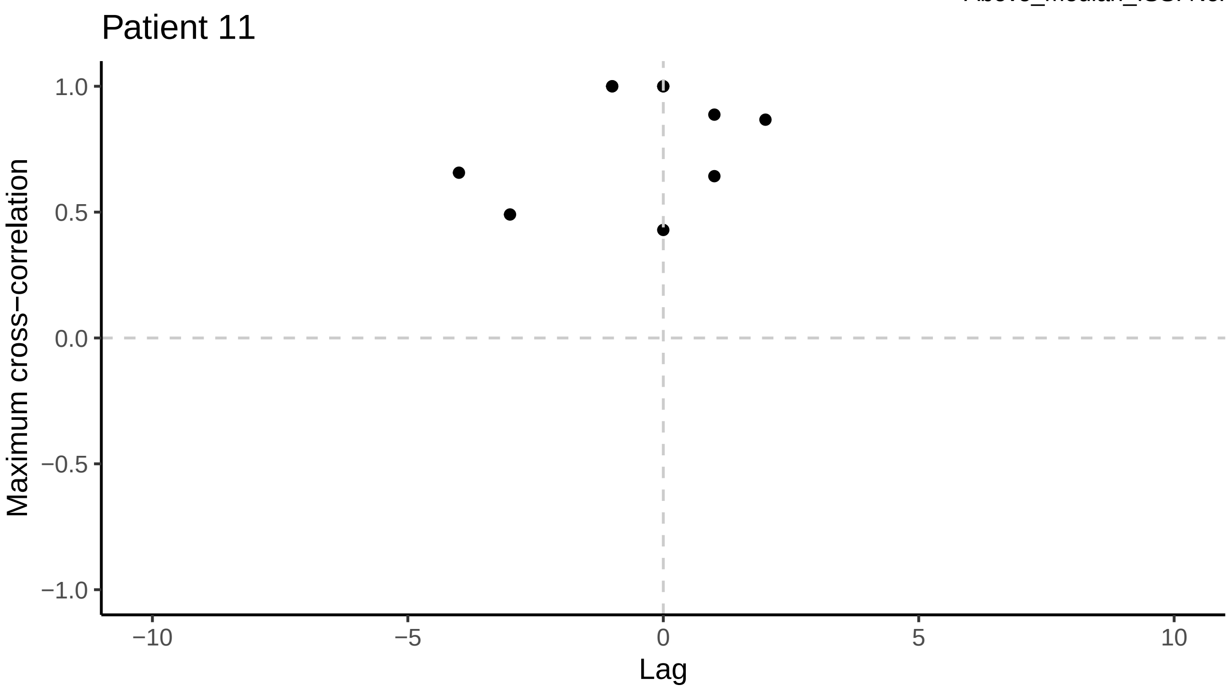

Treatment: Yes.
Infection: No.
Above_median_WCC: No.
Above_median_CRP: No.
Above_median_Temp: No.
Above_median_ISS: No.

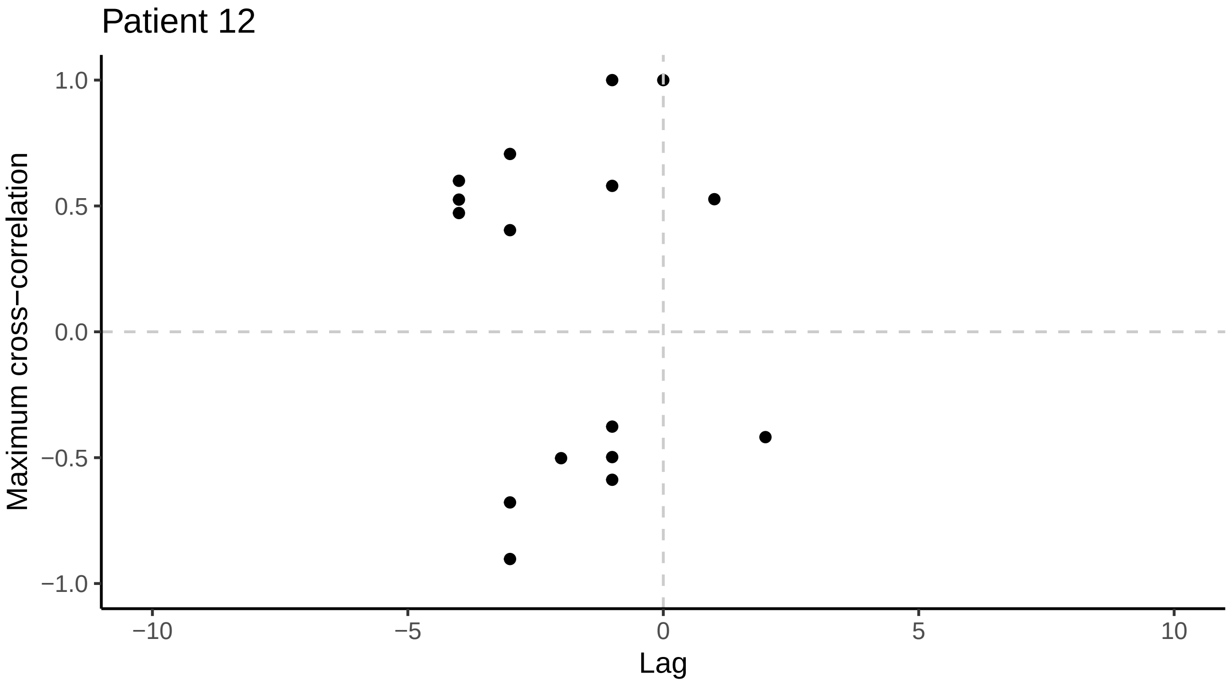

Treatment: Yes.
Infection: Yes.
Above_median_WCC: Yes.
Above_median_CRP: No.
Above_median_Temp: Yes.
Above_median_ISS: Yes.

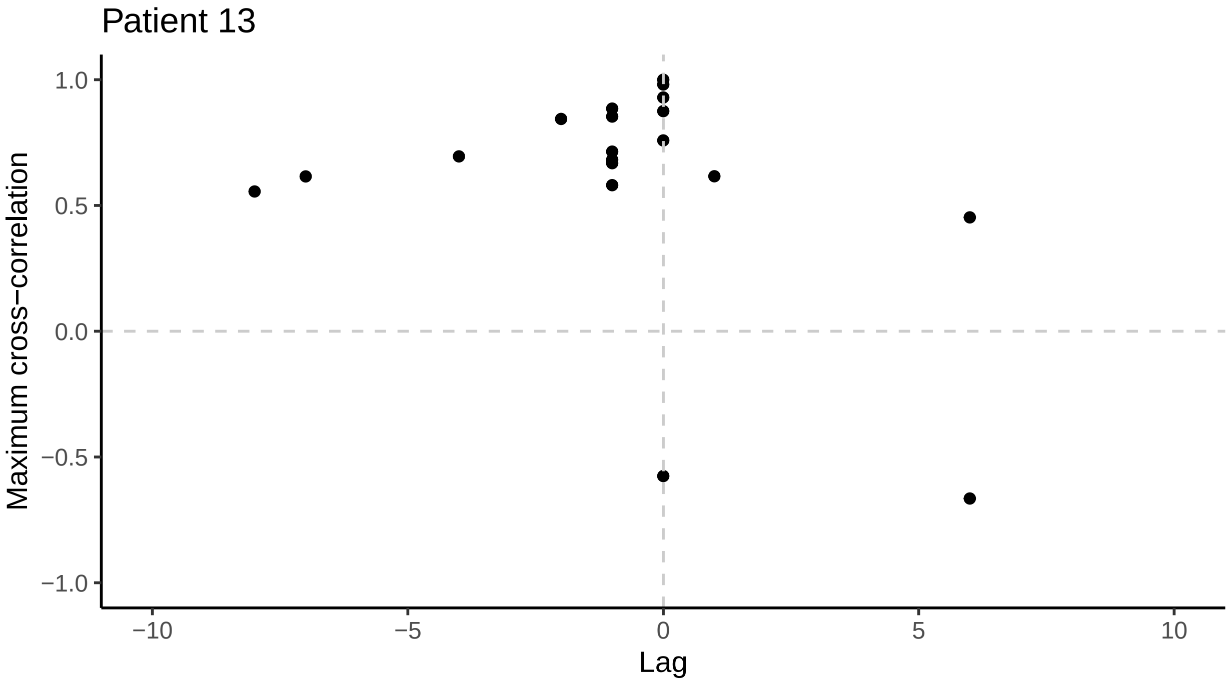

Treatment: Yes.
Infection: No.
Above_median_WCC: No.
Above_median_CRP: Yes.
Above_median_Temp: No.
Above_median_ISS: Yes.

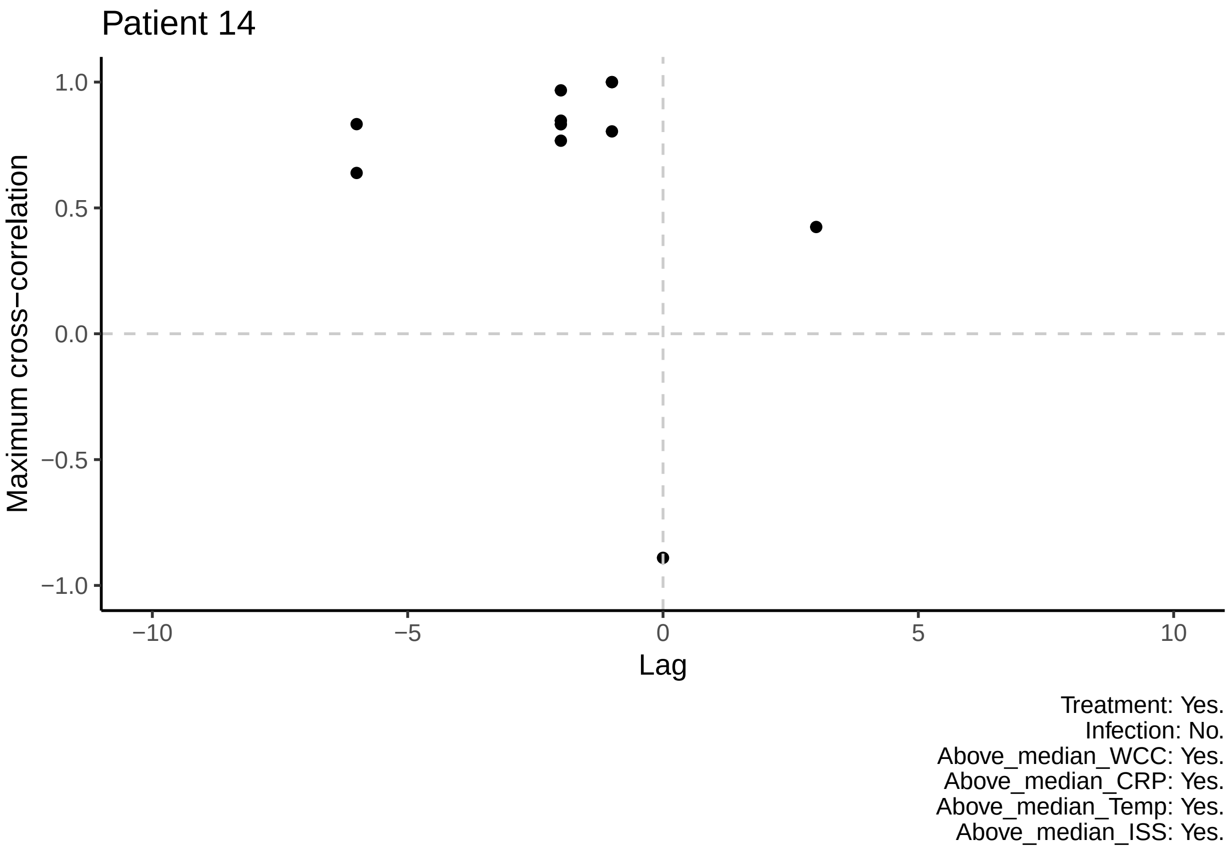
Patient 14
Maximum cross−correlation
1.0
0.5
0.0
−0.5
−1.0
−10
−5
0
5
10
Lag
Treatment: Yes.
Infection: No.
Above_median_WCC: Yes.
Above_median_CRP: Yes.
Above_median_Temp: Yes.
Above_median_ISS: Yes.

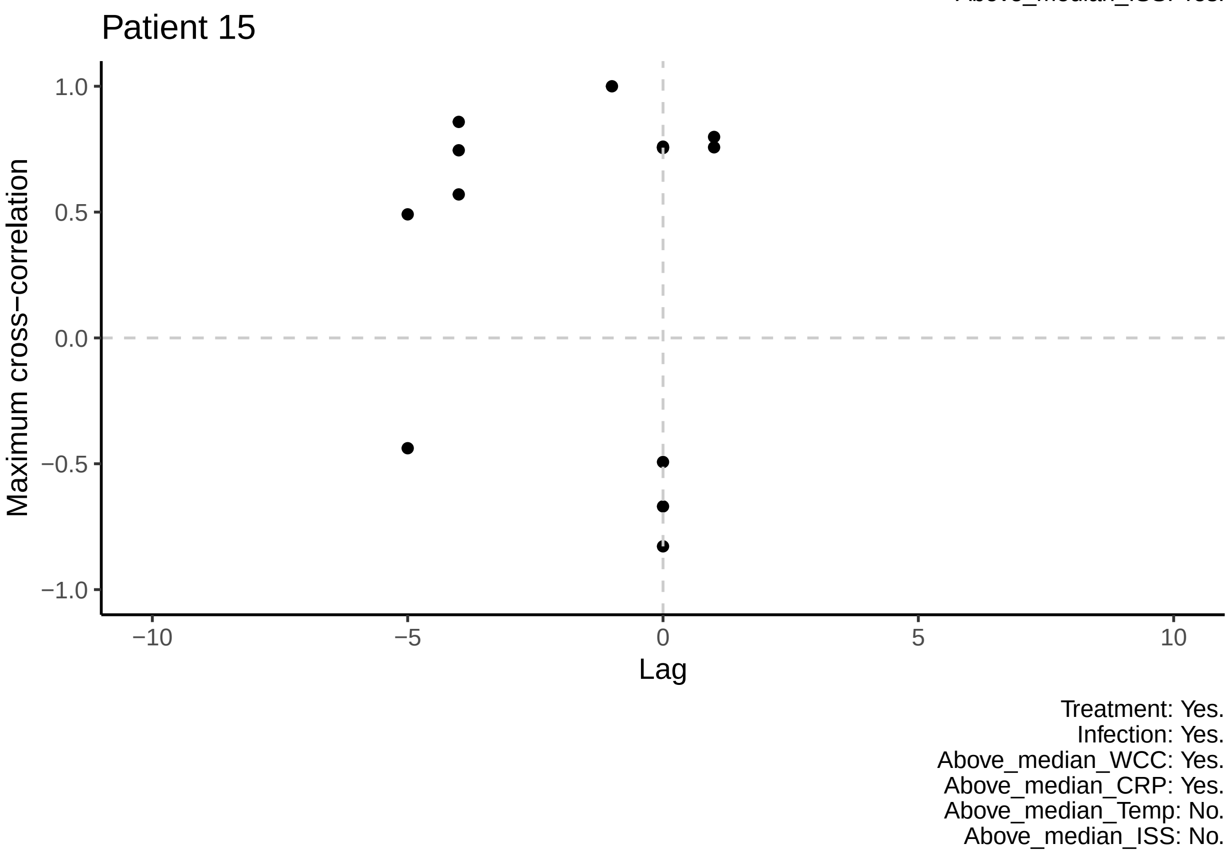
Patient 15
Maximum cross−correlation
1.0
0.5
0.0
−0.5
−1.0
−10
−5
0
5
10
Lag
Treatment: Yes.
Infection: Yes.
Above_median_WCC: Yes.
Above_median_CRP: Yes.
Above_median_Temp: No.
Above_median_ISS: No.

Patient 16

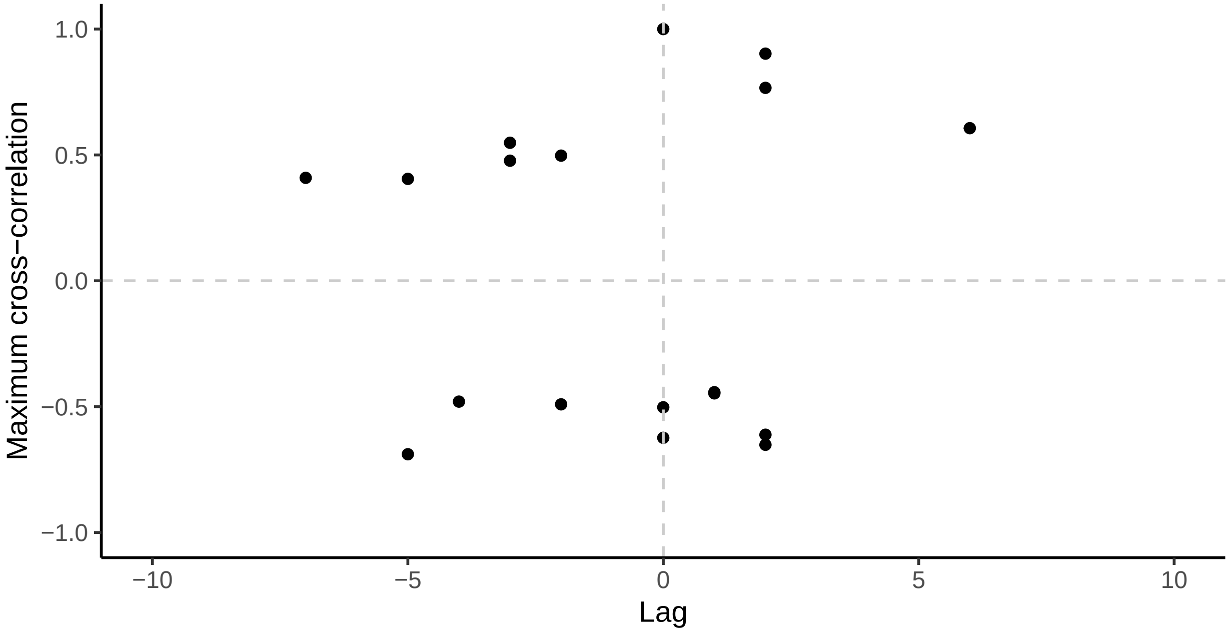

Treatment: Yes.
Infection: Yes.
Above_median_WCC: Yes.
Above_median_CRP: No.
Above_median_Temp: Yes.
Above_median_ISS: No.

Patient 17

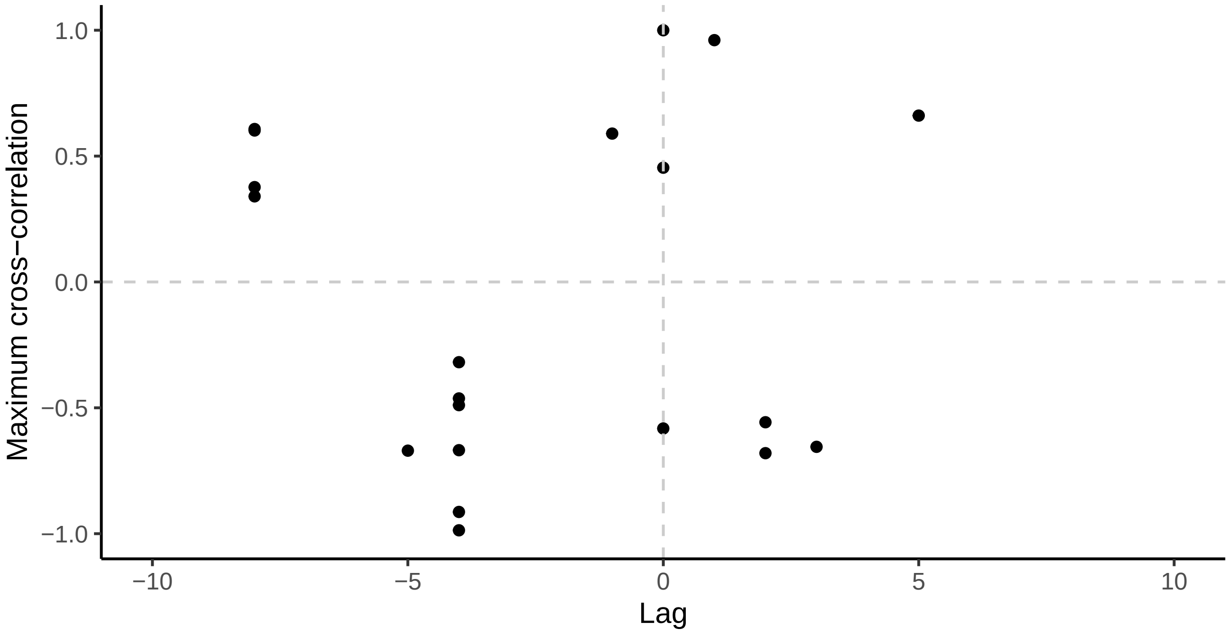

Treatment: Yes.
Infection: No.
Above_median_WCC: No.
Above_median_CRP: No.
Above_median_Temp: No.
Above_median_ISS: Yes.

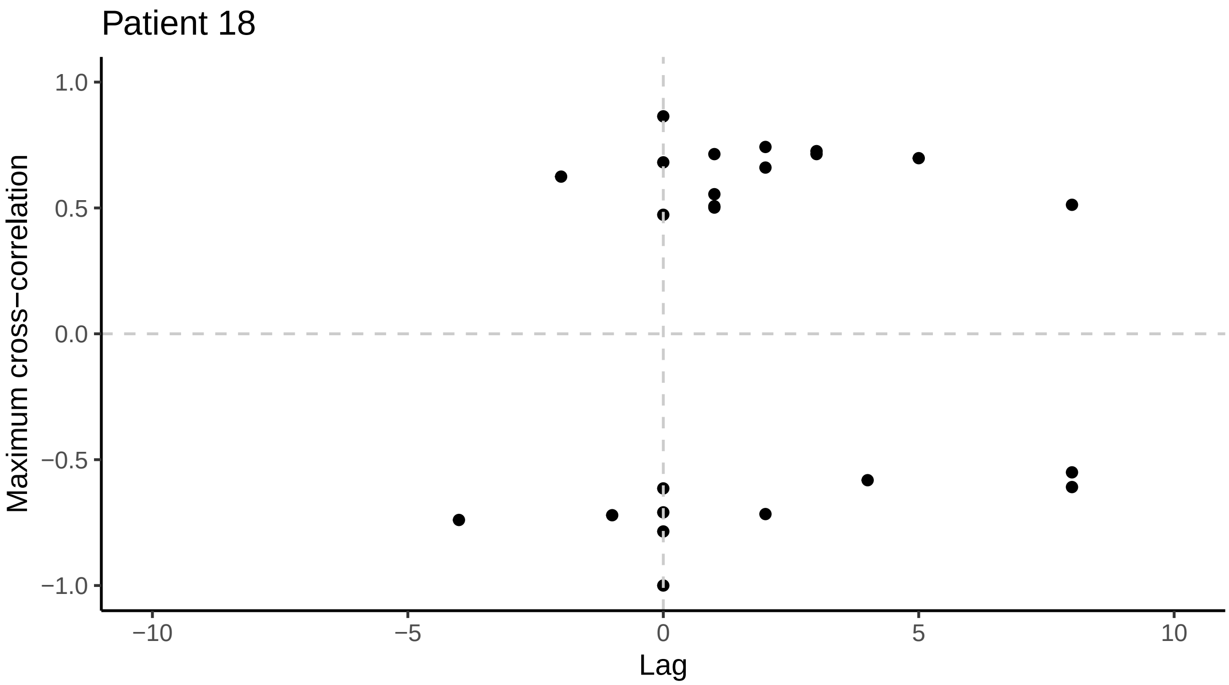

Treatment: Yes.
Infection: No.
Above_median_WCC: No.
Above_median_CRP: No.
Above_median_Temp: Yes.
Above_median_ISS: Yes.

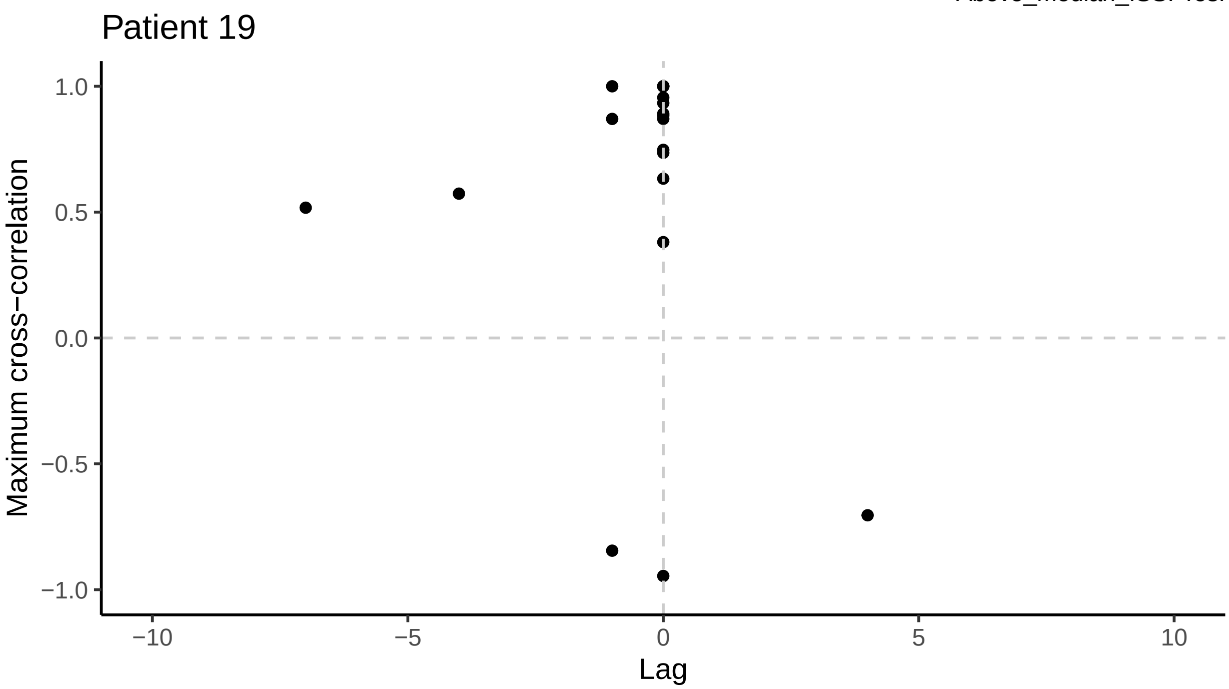

Treatment: Yes.
Infection: Yes.
Above_median_WCC: Yes.
Above_median_CRP: No.
Above_median_Temp: Yes.
Above_median_ISS: Yes.

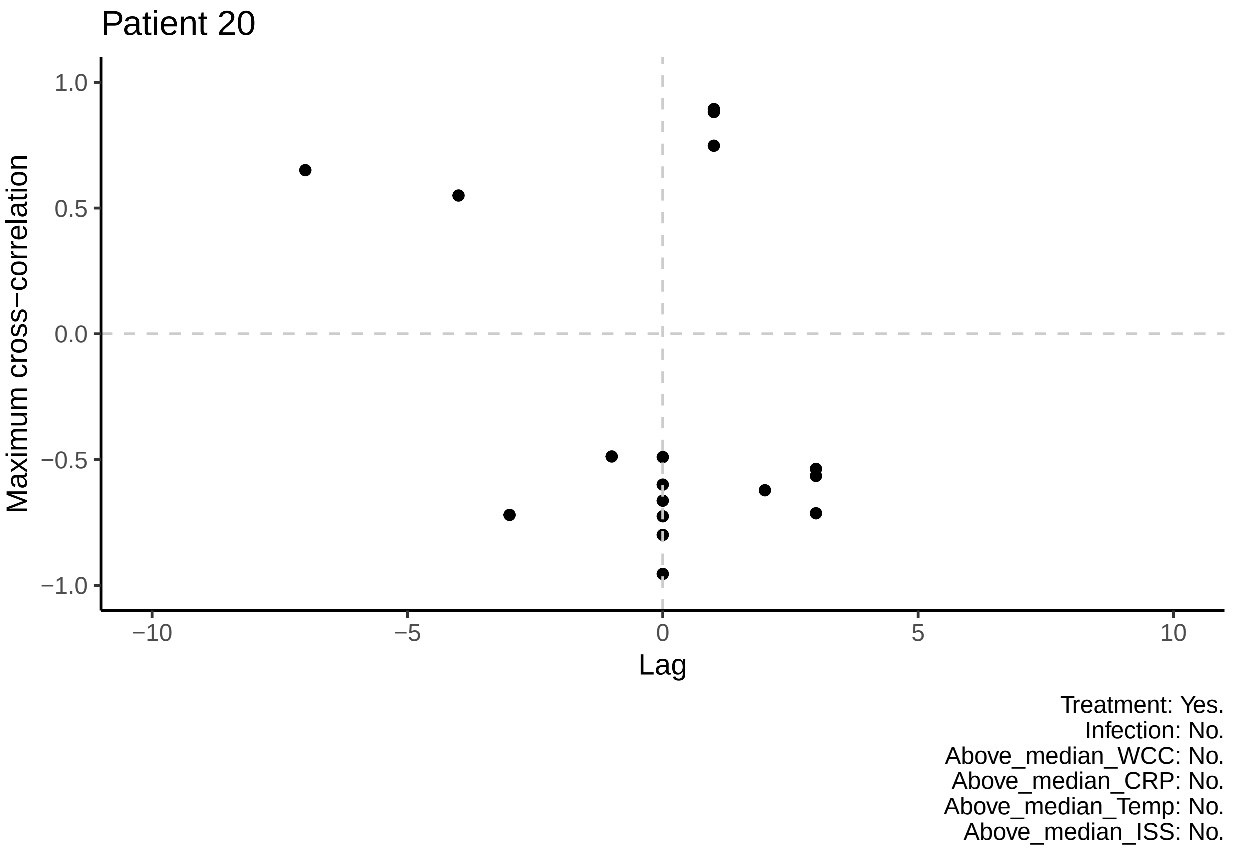
Patient 20
Maximum cross−correlation
1.0
0.5
0.0
−0.5
−1.0
−10
−5
0
5
10
Lag
Treatment: Yes.
Infection: No.
Above_median_WCC: No.
Above_median_CRP: No.
Above_median_Temp: No.
Above_median_ISS: No.

Code for Figure 3A

Philipp

Revised: May 11, 2021

Contents

Load data 1

Run nlme per patient and cytokine 1

Load data

```
library(openxlsx)
ip_data <- read.xlsx("../Dataset/Interpolated_data4.1.xlsx")
```

Run nlme per patient and cytokine

```
library(nlme)
library(ggplot2)
library(plyr)
library(pheatmap)
library(gplots)
```

```
##
## Attaching package: 'gplots'

## The following object is masked from 'package:stats':
##
##     lowess
```

```
library(made4)
```

```
## Loading required package: RColorBrewer

## Loading required package: scatterplot3d

## Loading required package: Biobase

## Loading required package: BiocGenerics

## Warning: package 'BiocGenerics' was built under R version 4.0.5

## Loading required package: parallel

##
## Attaching package: 'BiocGenerics'
```

```
## The following objects are masked from 'package:parallel':
##
##     clusterApply, clusterApplyLB, clusterCall, clusterEvalQ,
##     clusterExport, clusterMap, parApply, parCapply, parLapply,
##     parLapplyLB, parRapply, parSapply, parSapplyLB

## The following objects are masked from 'package:stats':
##
##     IQR, mad, sd, var, xtabs

## The following objects are masked from 'package:base':
##
##     anyDuplicated, append, as.data.frame, basename, cbind, colnames,
##     dirname, do.call, duplicated, eval, evalq, Filter, Find, get, grep,
##     grepl, intersect, is.unsorted, lapply, Map, mapply, match, mget,
##     order, paste, pmax, pmax.int, pmin, pmin.int, Position, rank,
##     rbind, Reduce, rownames, sapply, setdiff, sort, table, tapply,
##     union, unique, unsplit, which.max, which.min

## Welcome to Bioconductor
##
##     Vignettes contain introductory material; view with
##     'browseVignettes()'. To cite Bioconductor, see
##     'citation("Biobase")', and for packages 'citation("pkgname")'.

## Loading required package: SummarizedExperiment

## Loading required package: MatrixGenerics

## Loading required package: matrixStats

##
## Attaching package: 'matrixStats'

## The following objects are masked from 'package:Biobase':
##
##     anyMissing, rowMedians

## The following object is masked from 'package:plyr':
##
##     count

##
## Attaching package: 'MatrixGenerics'

## The following objects are masked from 'package:matrixStats':
##
##     colAlls, colAnyNAs, colAnys, colAvgsPerRowSet, colCollapse,
##     colCounts, colCummaxs, colCummins, colCumprods, colCumsums,
##     colDiffs, colIQRDiffs, colIQRs, colLogSumExps, colMadDiffs,
##     colMads, colMaxs, colMeans2, colMedians, colMins, colOrderStats,
##     colProds, colQuantiles, colRanges, colRanks, colSdDiffs, colSds,
##     colSums2, colTabulates, colVarDiffs, colVars, colWeightedMads,
##     colWeightedMeans, colWeightedMedians, colWeightedSds,
##     colWeightedVars, rowAlls, rowAnyNAs, rowAnys, rowAvgsPerColSet,
##     rowCollapse, rowCounts, rowCummaxs, rowCummins, rowCumprods,
##     rowCumsums, rowDiffs, rowIQRDiffs, rowIQRs, rowLogSumExps,
##     rowMadDiffs, rowMads, rowMaxs, rowMeans2, rowMedians, rowMins,
##     rowOrderStats, rowProds, rowQuantiles, rowRanges, rowRanks,
```

```
##     rowSdDiffs, rowSds, rowSums2, rowTabulates, rowVarDiffs, rowVars,
##     rowWeightedMads, rowWeightedMeans, rowWeightedMedians,
##     rowWeightedSds, rowWeightedVars

## The following object is masked from 'package:Biobase':
##
##     rowMedians

## Loading required package: GenomicRanges

## Loading required package: stats4

## Loading required package: S4Vectors

##
## Attaching package: 'S4Vectors'

## The following object is masked from 'package:gplots':
##
##     space

## The following object is masked from 'package:plyr':
##
##     rename

## The following object is masked from 'package:base':
##
##     expand.grid

## Loading required package: IRanges

##
## Attaching package: 'IRanges'

## The following object is masked from 'package:plyr':
##
##     desc

## The following object is masked from 'package:nlme':
##
##     collapse

## Loading required package: GenomeInfoDb

## Warning: package 'GenomeInfoDb' was built under R version 4.0.5
```

```
library(car)
```

```
## Loading required package: carData
```

```
time <- ip_data$Time
wcc <- ip_data$WCC
crp <- ip_data$CRP
temp <- ip_data$Temp
infection <- ip_data$Infection
patient <- ip_data$Patient
rhIL1ra <- ip_data$rhIL1ra

coeff_matrix <- matrix(NA,
                       nrow = 42,
                       ncol = 7)
colnames(coeff_matrix) <- c("Time",
```

```r
                            "A_cytokine",
                            "CRP",
                            "WCC",
                            "Temp",
                            "Infection",
                            "rhIl1ra")
rownames(coeff_matrix) <- colnames(ip_data)[seq(from = 8,
                                                to = 133,
                                                by = 3)]

pval_matrix <- coeff_matrix
vif_matrix <- coeff_matrix
anova_matrix <- coeff_matrix[ , 1:4]
colnames(anova_matrix) <- c("p.value", "AIC.old", "AIC.interaction", "old-interact")

sd_matrix <- matrix(NA,
                    ncol = 2,
                    nrow = 42)
colnames(sd_matrix) <- c("MD_cytokine",
                         "A_cytokine")
rownames(sd_matrix) <- substr(rownames(coeff_matrix), 4, 100)
sd_time <- sd(time, na.rm = TRUE)
sd_crp <- sd(crp, na.rm = TRUE)
sd_wcc <- sd(wcc, na.rm = TRUE)
sd_temp <- sd(temp, na.rm = TRUE)
sd_infection <- sd(infection, na.rm = TRUE)
sd_rhIL1ra <- sd(rhIL1ra, na.rm = TRUE)
sd_vector <- c(sd_time,
               NA,
               sd_crp,
               sd_wcc,
               sd_temp,
               sd_infection,
               sd_rhIL1ra)

abl_data <- matrix(NA,
                   ncol = 3,
                   nrow = 42)
colnames(abl_data) <- c("AIC",
                        "BIC",
                        "loglik")

library(predictmeans)
```

```
## Loading required package: lme4

## Loading required package: Matrix

##
## Attaching package: 'Matrix'

## The following object is masked from 'package:S4Vectors':
##
##     expand

## Registered S3 methods overwritten by 'lme4':
##   method                          from
```

```
##   cooks.distance.influence.merMod car
##   influence.merMod                car
##   dfbeta.influence.merMod         car
##   dfbetas.influence.merMod        car

##
## Attaching package: 'lme4'

## The following object is masked from 'package:nlme':
##
##     lmList

##
## Attaching package: 'predictmeans'

## The following object is masked from 'package:gplots':
##
##     residplot
```

```
for (cytokine in 1:42) {
  md_cytokine <- log(ip_data[ , cytokine * 3 + 5])
  a_cytokine <- log(ip_data[ , cytokine * 3 + 6])

  sd_matrix[cytokine, 1] <- sd(md_cytokine, na.rm = TRUE)
  sd_matrix[cytokine, 2] <- sd(a_cytokine, na.rm = TRUE)

  lme_fit <- tryCatch({
    lme(md_cytokine ~ time + a_cytokine + crp + wcc + temp + infection + rhIL1ra,
        random = ~ time | patient,
        correlation = corAR1(form = ~ time | patient),
        na.action = na.omit)
  }, error = function(e) {
    NA
  }
  )

  if (class(lme_fit) == "lme") {
    lme_names <- names(rename(lme_fit$coefficients$fixed,
                              c("a_cytokine" =
                                  colnames(ip_data)[cytokine * 3 + 6])))

    # my_plot_hook <- function(x, options)
    #     paste("\n", knitr::hook_plot_tex(x, options), "\n")
    #     knitr::knit_hooks$set(plot = my_plot_hook)
    #
    # barplot(height = lme_fit$coefficients$fixed[-1],
    #         names.arg = lme_names[-1],
    #         main = paste0("Coefficients of ",
    #                       substring(colnames(ip_data)[cytokine * 3 + 5], 4),
    #                       " for model ",
    #                       colnames(ip_data)[cytokine * 3 + 5],
    #                       " ~ ",
    #                       colnames(ip_data)[cytokine * 3 + 6],
    #                       " + time + WCC + CRP + Temp + Infection + rhIL1ra"),
    #         ylab = "Coefficient magnitude",
    #         cex.names = 0.3,
```

```r
    #         cex.main = 0.6,
    #         col = "blue")

    coeff_matrix[cytokine, ] <- lme_fit$coefficients$fixed[-1]
    pval_matrix[cytokine, ] <- anova(lme_fit)$`p-value`[2:(dim(pval_matrix)[2]+1)]
    vif_val <- vif(lme_fit)
    vif_matrix[cytokine, ] <- vif_val

    a <- tryCatch({
      anova(update(lme_fit, . ~ ., method = "ML"),
            lme(md_cytokine ~
                  time + a_cytokine + crp + wcc * temp + infection + rhIL1ra,
                random = ~ time | patient,
                correlation = corAR1(form = ~ time | patient),
                na.action = na.omit,
                method = "ML"))
    }, error = function(e) {NA})
              if (!is.na(a)) {
                anova_matrix[cytokine, 1] <-a$`p-value`[2]
                anova_matrix[cytokine, 2:3] <- a$AIC
                anova_matrix[cytokine, 4] <- a$AIC[1]-a$AIC[2]
              }

    abl_data[cytokine, 1] <- AIC(lme_fit)
    abl_data[cytokine, 2] <- BIC(lme_fit)
    abl_data[cytokine, 3] <- lme_fit$logLik

    my_plot_hook <- function(x, options)
       paste("\n", knitr::hook_plot_tex(x, options), "\n")
       knitr::knit_hooks$set(plot = my_plot_hook)
    plot(fitted(lme_fit),residuals(lme_fit),
        main = substr(colnames(ip_data)[cytokine * 3 + 6], 3, 100))
    my_plot_hook <- function(x, options)
       paste("\n", knitr::hook_plot_tex(x, options), "\n")
       knitr::knit_hooks$set(plot = my_plot_hook)
    hist(residuals(lme_fit),
        main = substr(colnames(ip_data)[cytokine * 3 + 6], 3, 100))
    my_plot_hook <- function(x, options)
       paste("\n", knitr::hook_plot_tex(x, options), "\n")
       knitr::knit_hooks$set(plot = my_plot_hook)
    qqnorm(residuals(lme_fit),
        main = paste(substr(colnames(ip_data)[cytokine * 3 + 6], 3, 100)))


  } else {
    print(paste0("Error: ",
                 colnames(ip_data)[cytokine * 3 + 5],
                 " ~ ",
                 colnames(ip_data)[cytokine * 3 + 6],
                 " + time + WCC + CRP + Temp + Infection + rhIL1ra"))
  }
}
```

```
## Warning in if (!is.na(a)) {: the condition has length > 1 and only the first
```

```
## element will be used
```

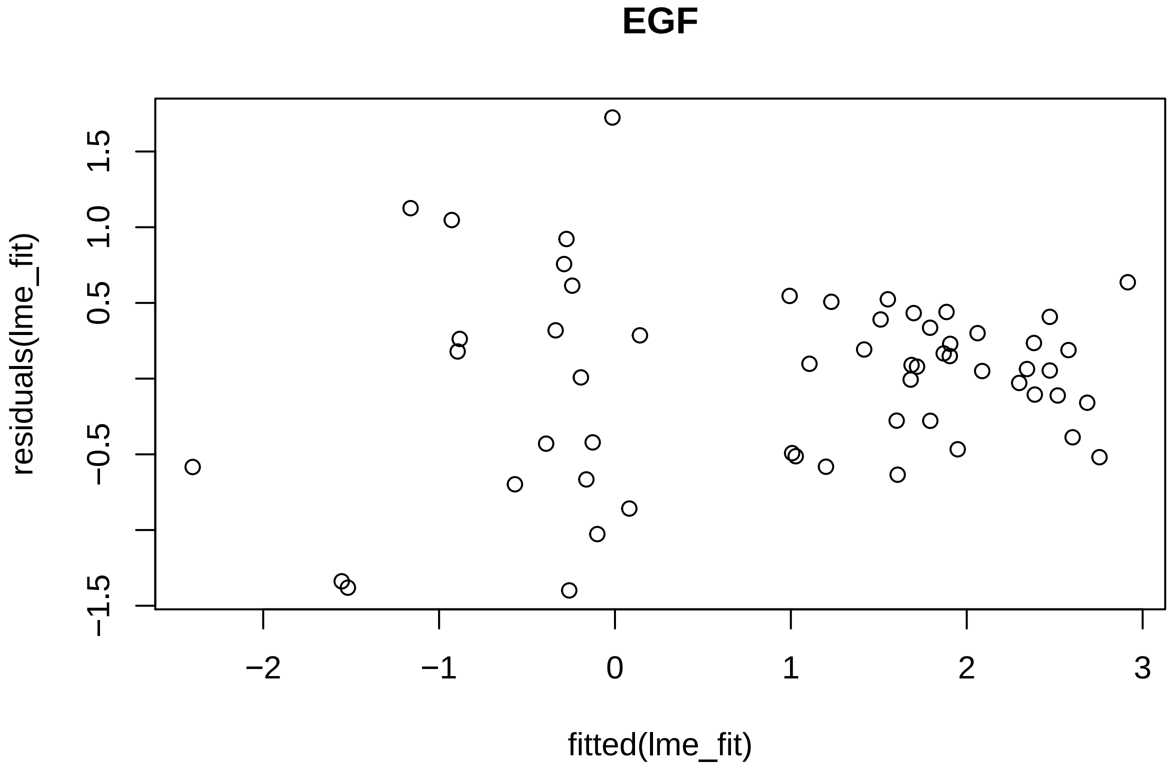

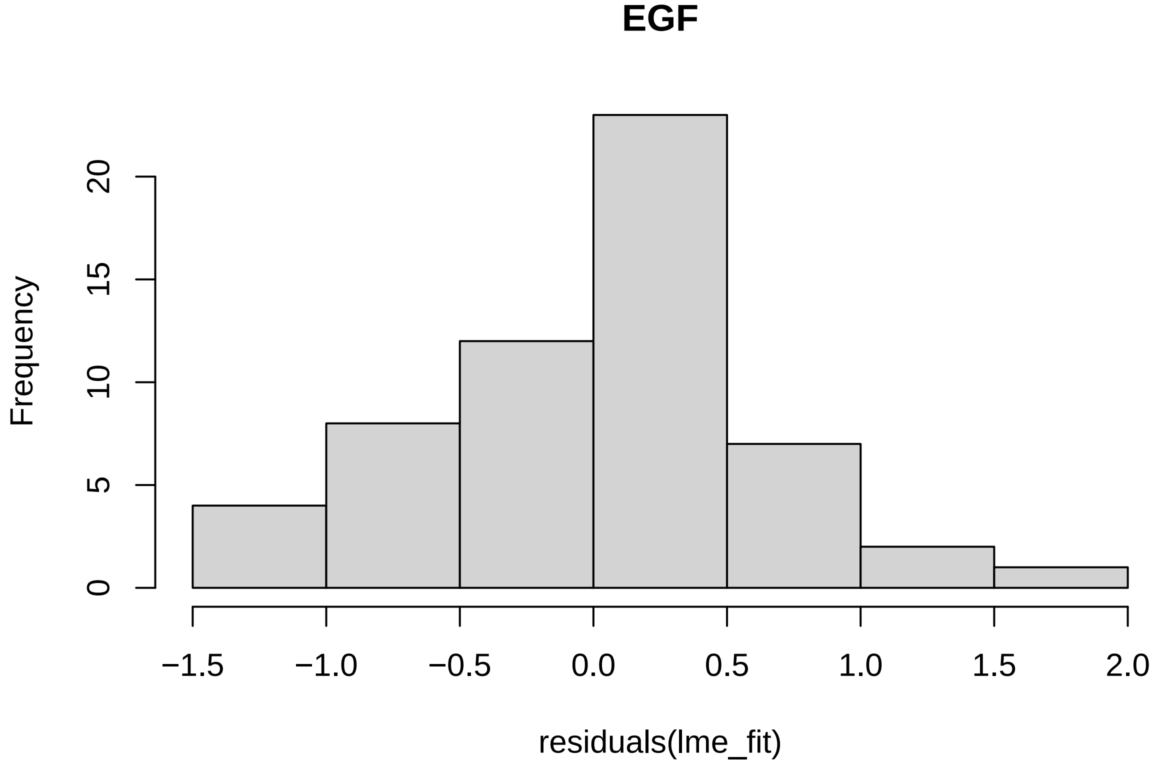


```
## Warning in if (!is.na(a)) {: the condition has length > 1 and only the first
## element will be used
```

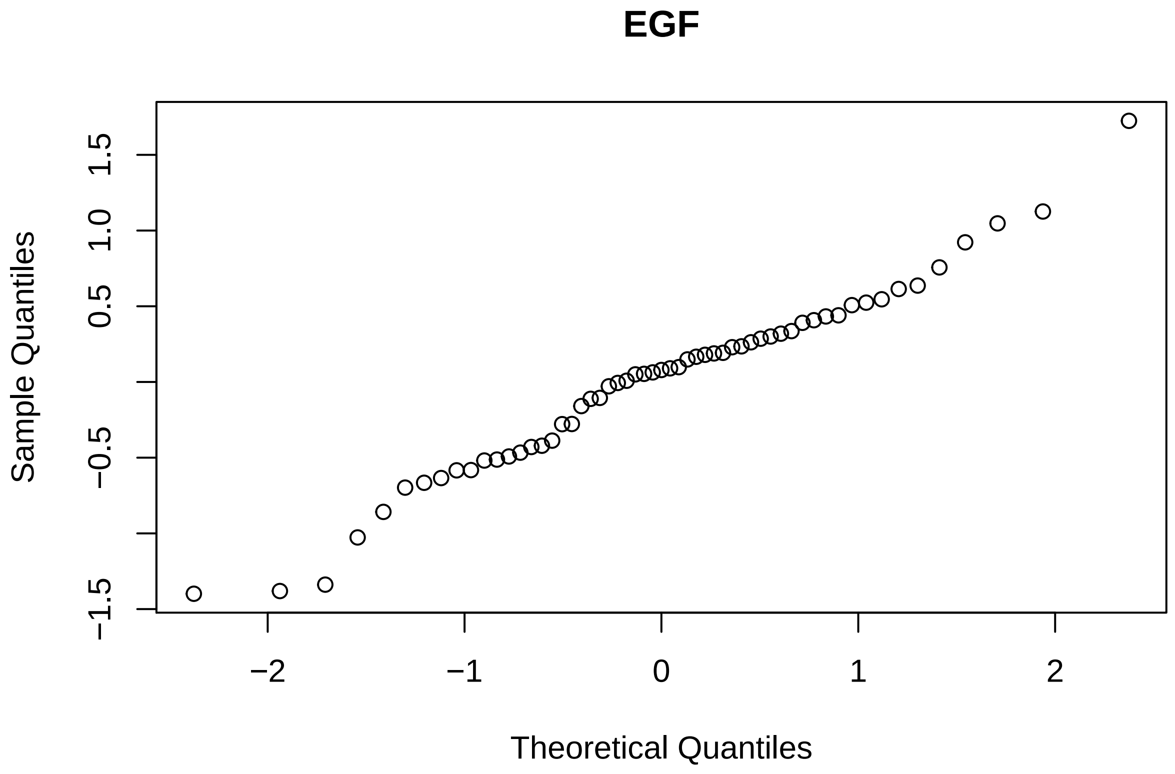
EGF
Sample Quantiles
Theoretical Quantiles
−1.5
−0.5
0.5
1.0
1.5
−2
−1
0
1
2

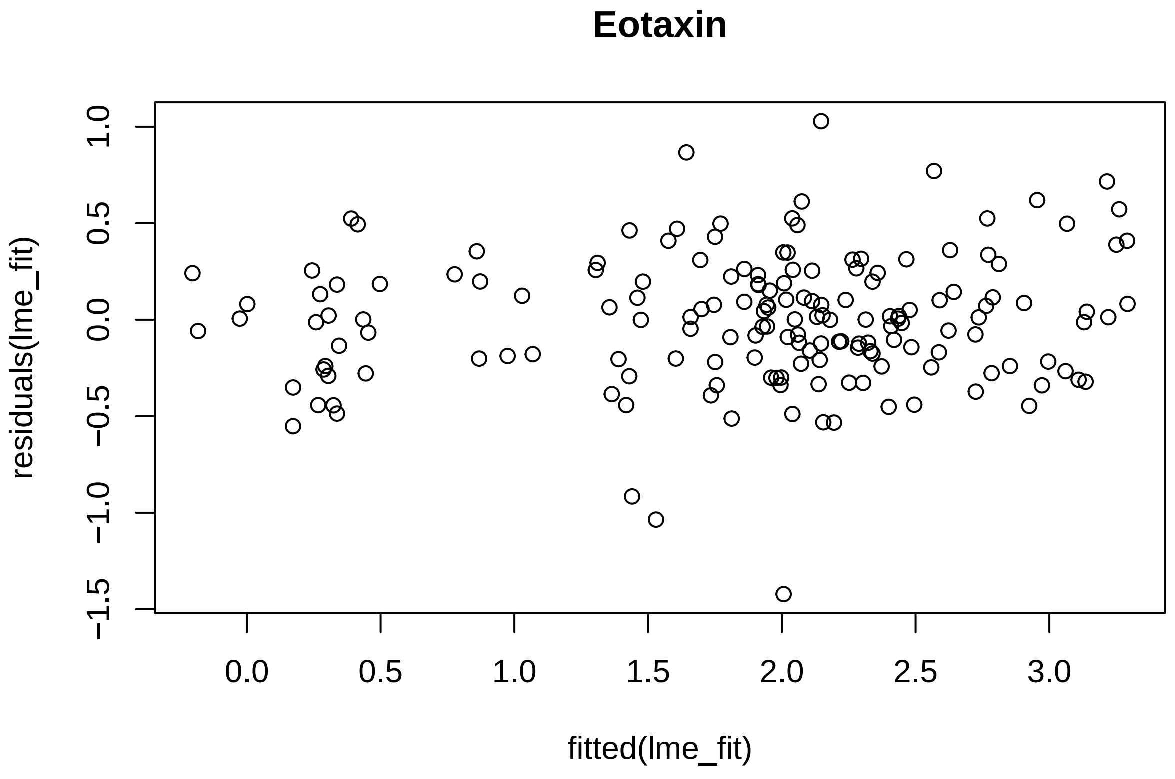
Eotaxin
residuals(lme_fit)
fitted(lme_fit)
−1.5
−1.0
−0.5
0.0
0.5
1.0
0.0
0.5
1.0
1.5
2.0
2.5
3.0

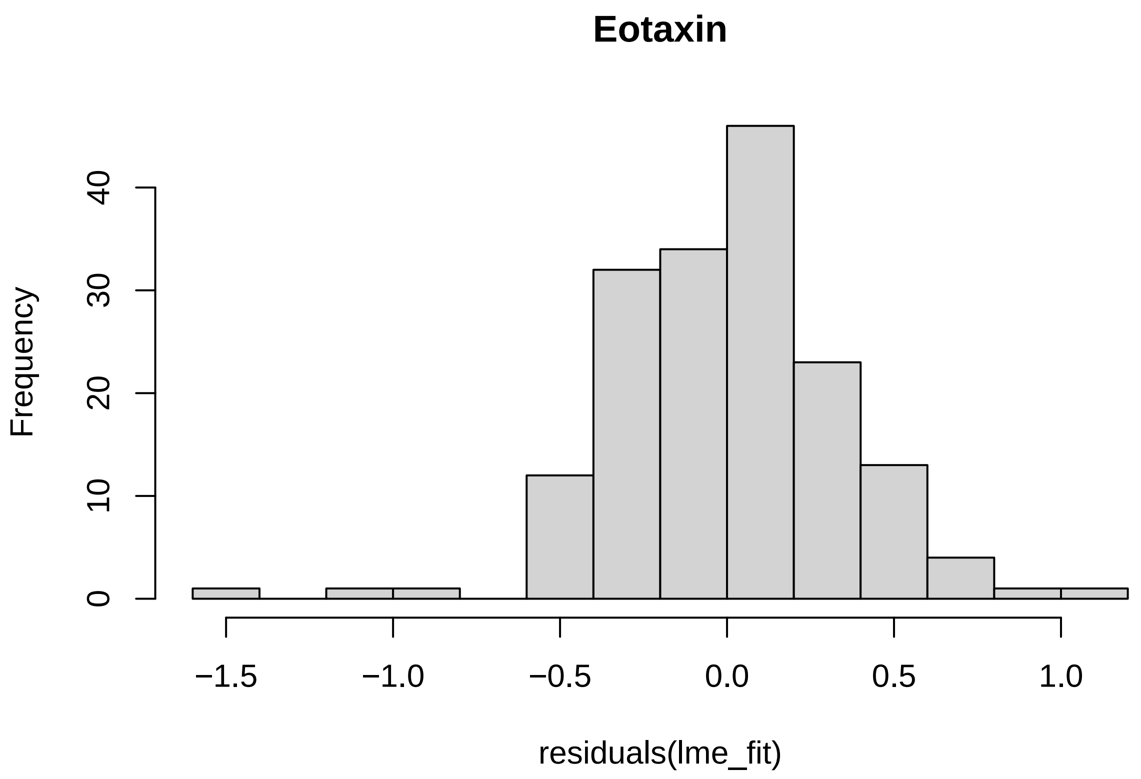


```
## Warning in if (!is.na(a)) {: the condition has length > 1 and only the first
## element will be used
```

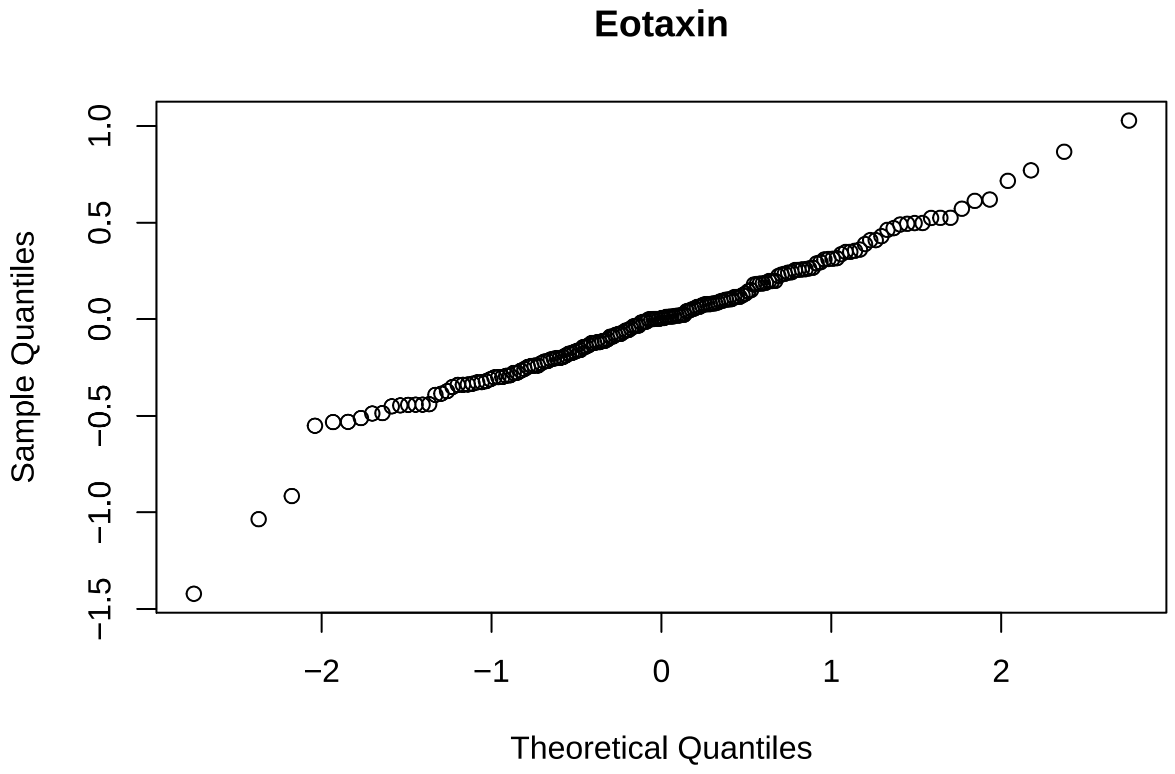

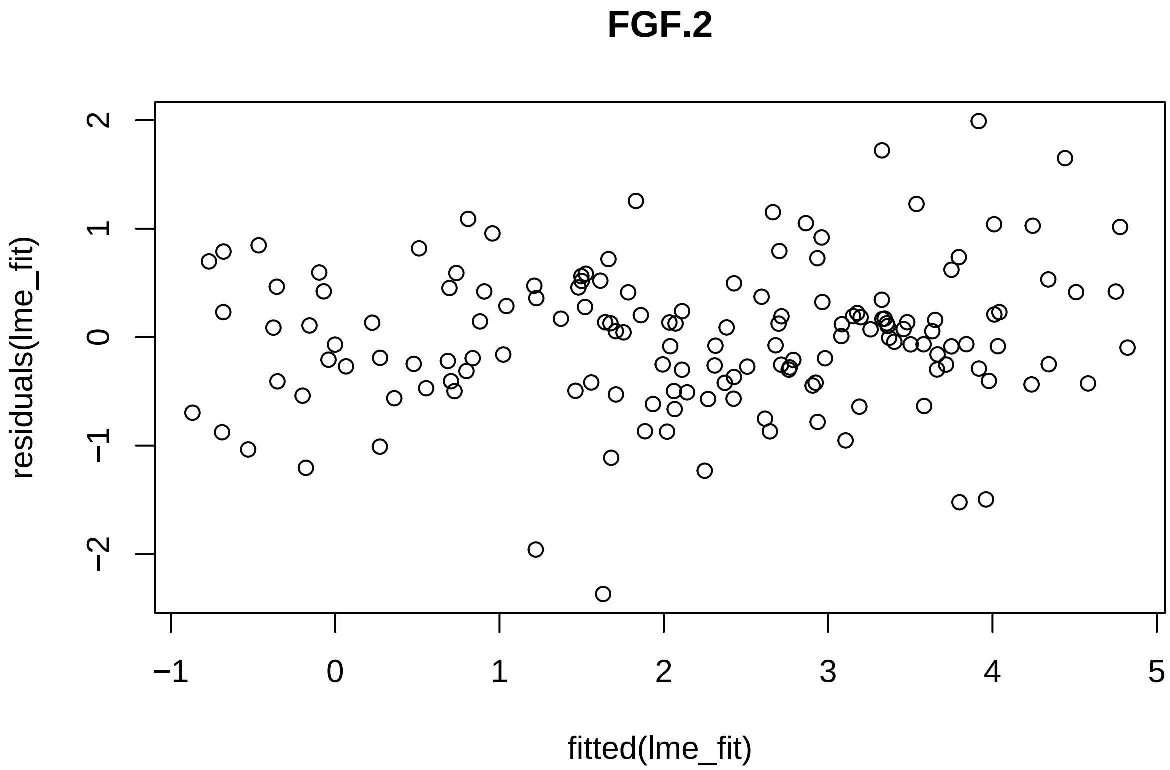

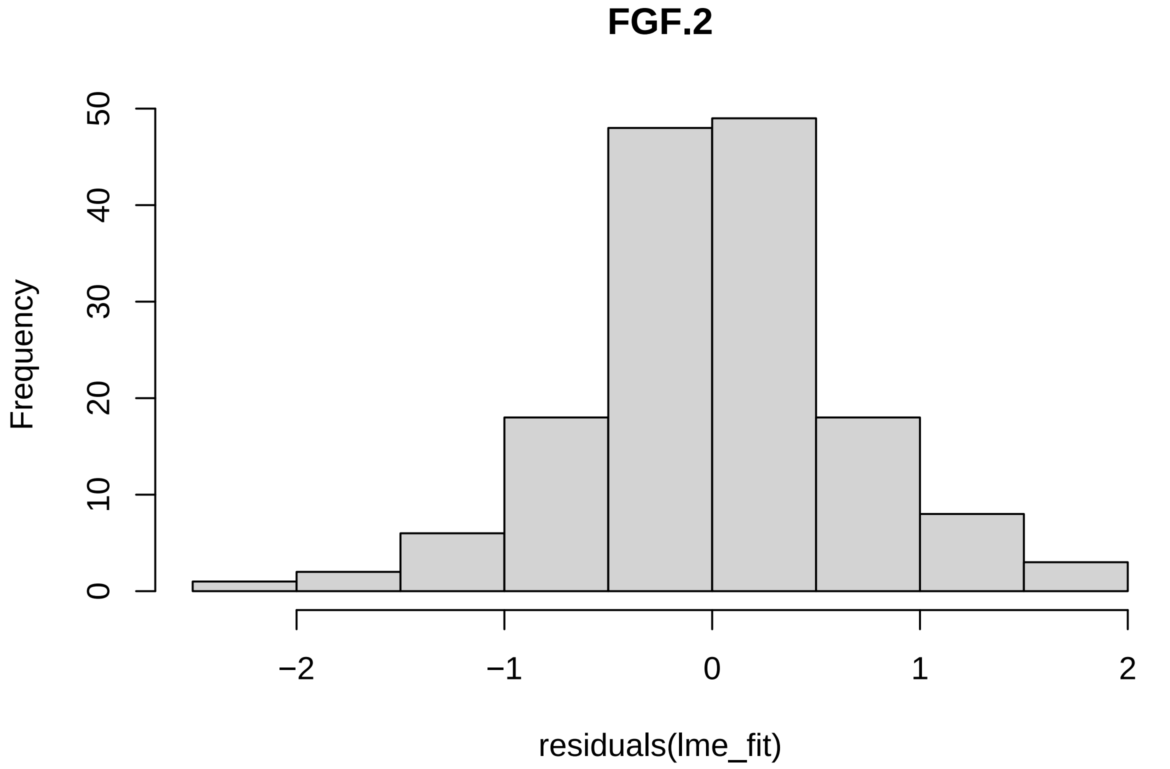


```
## Warning in if (!is.na(a)) {: the condition has length > 1 and only the first
## element will be used
```

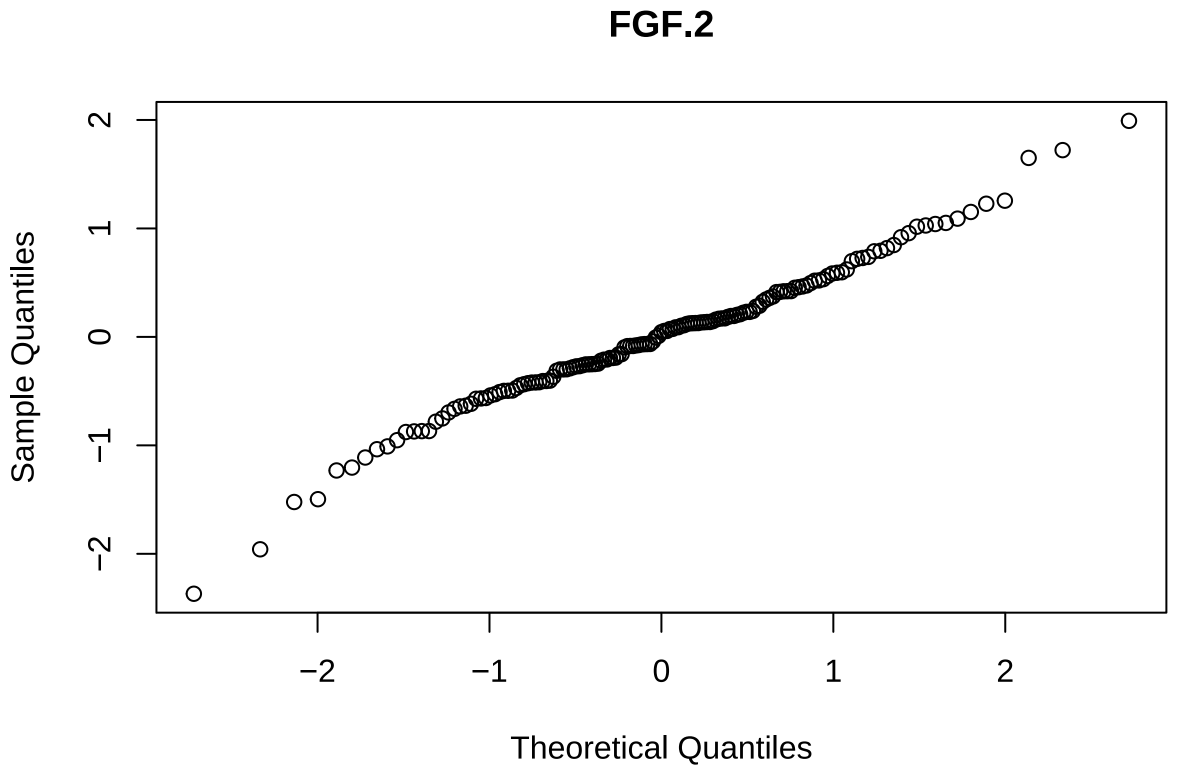
FGF.2
Sample Quantiles
Theoretical Quantiles
−2
−1
0
1
2

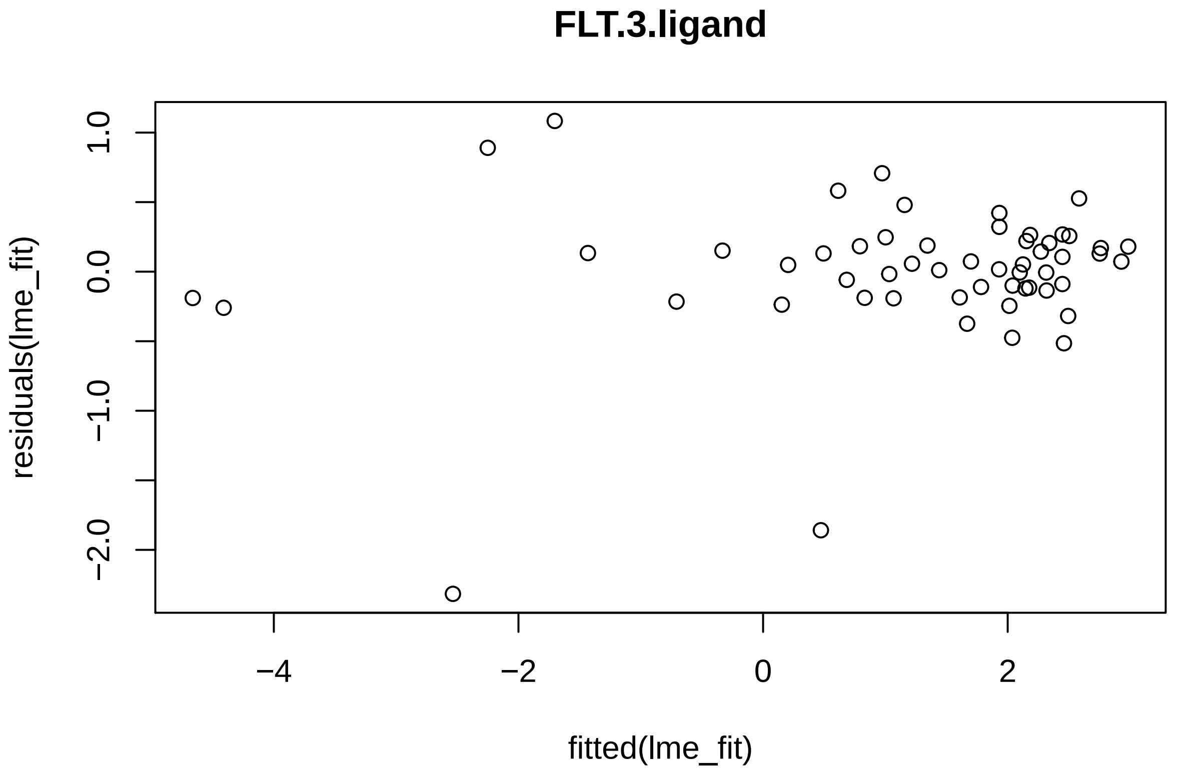
FLT.3.ligand
residuals(lme_fit)
fitted(lme_fit)
−2.0
−1.0
0.0
1.0
−4
−2
0
2

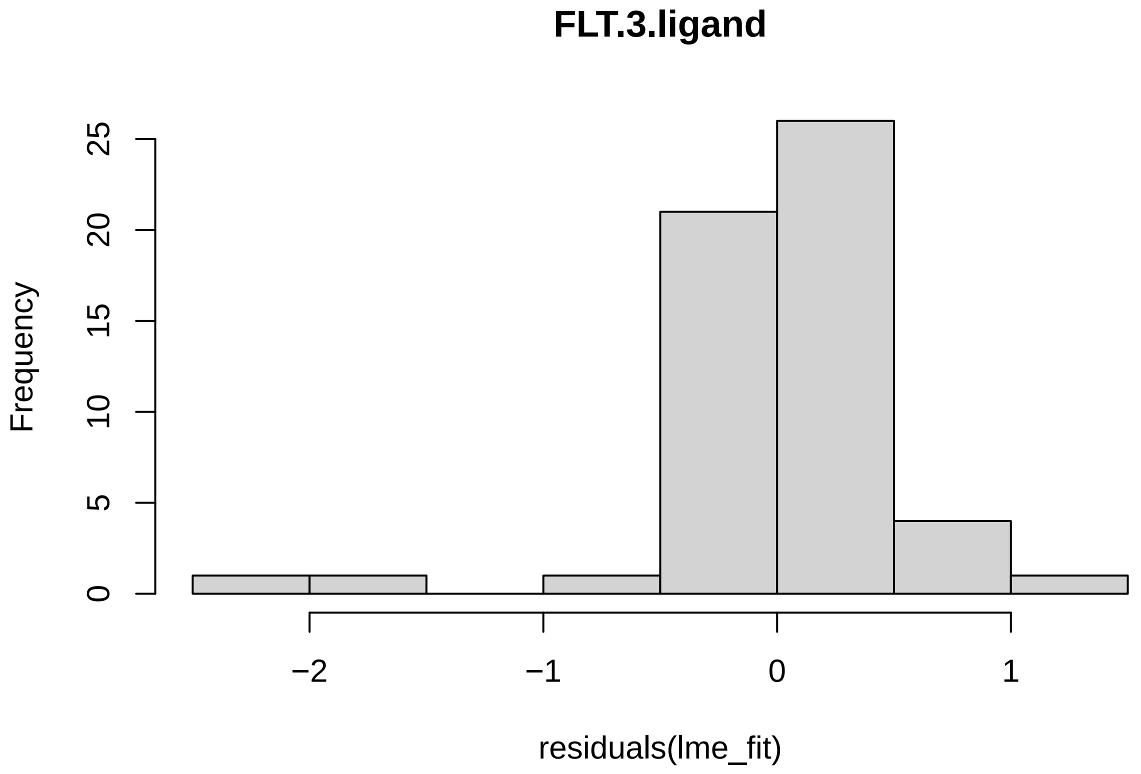


```
## Warning in if (!is.na(a)) {: the condition has length > 1 and only the first
## element will be used
```

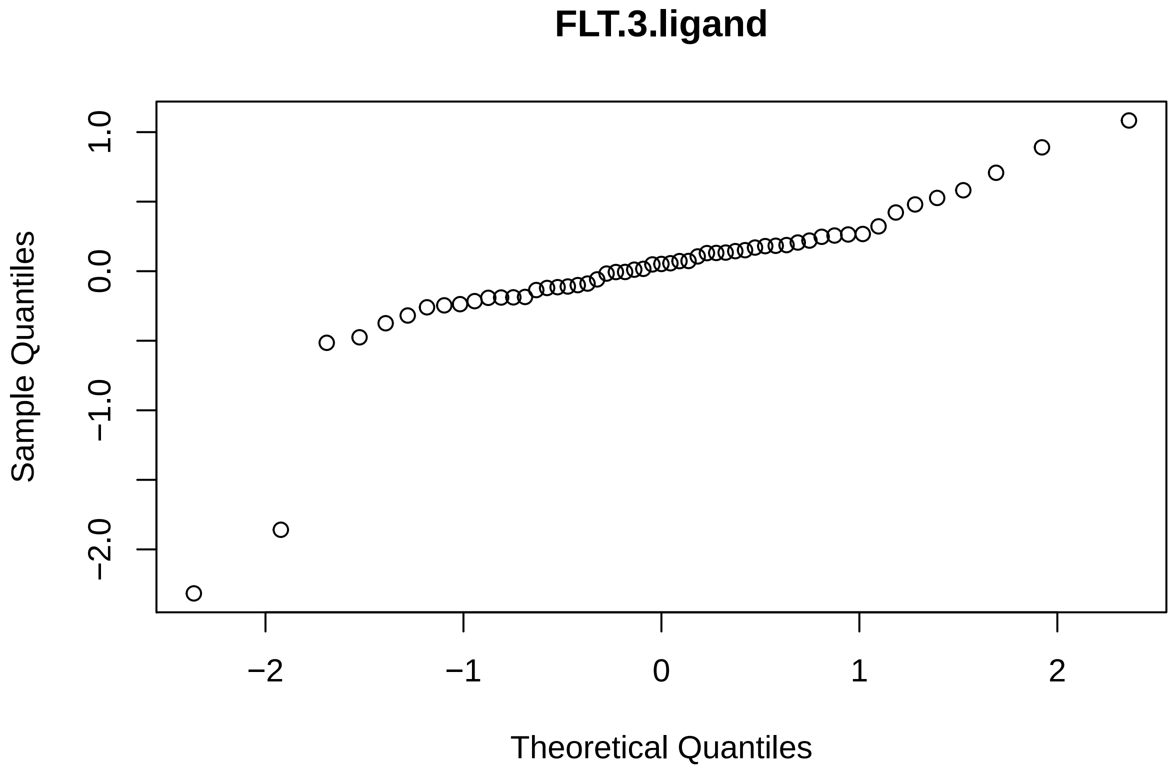

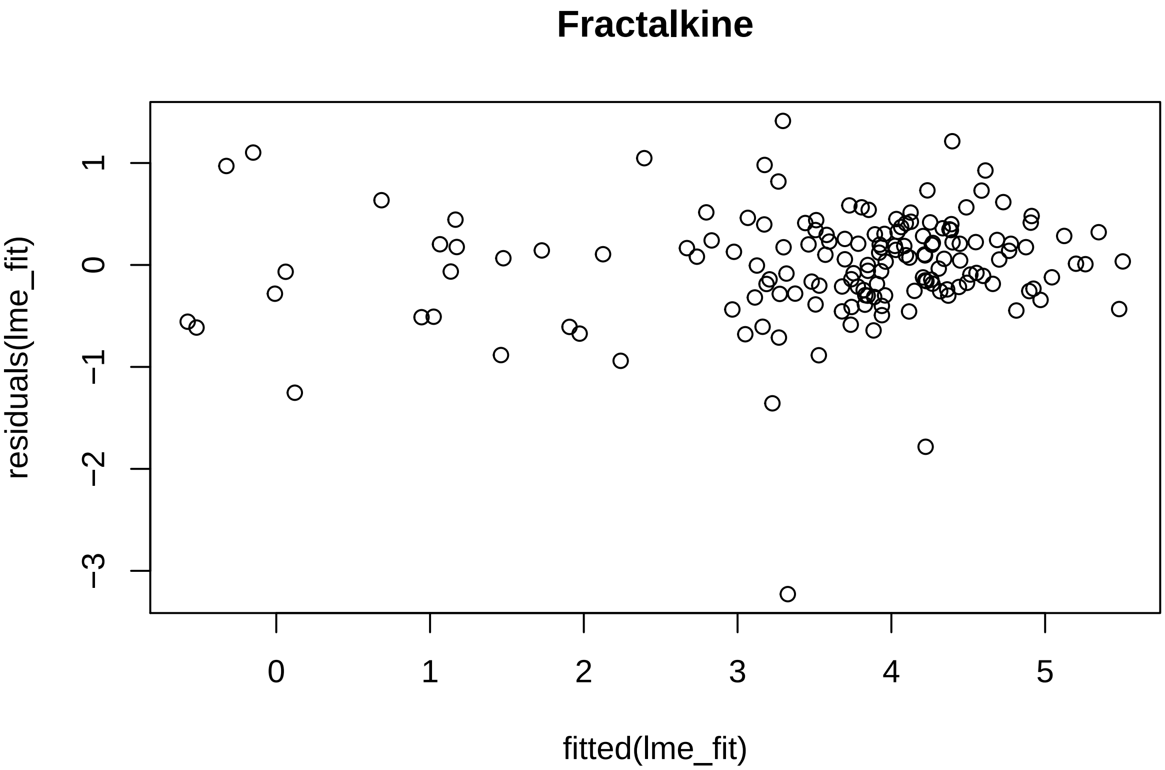

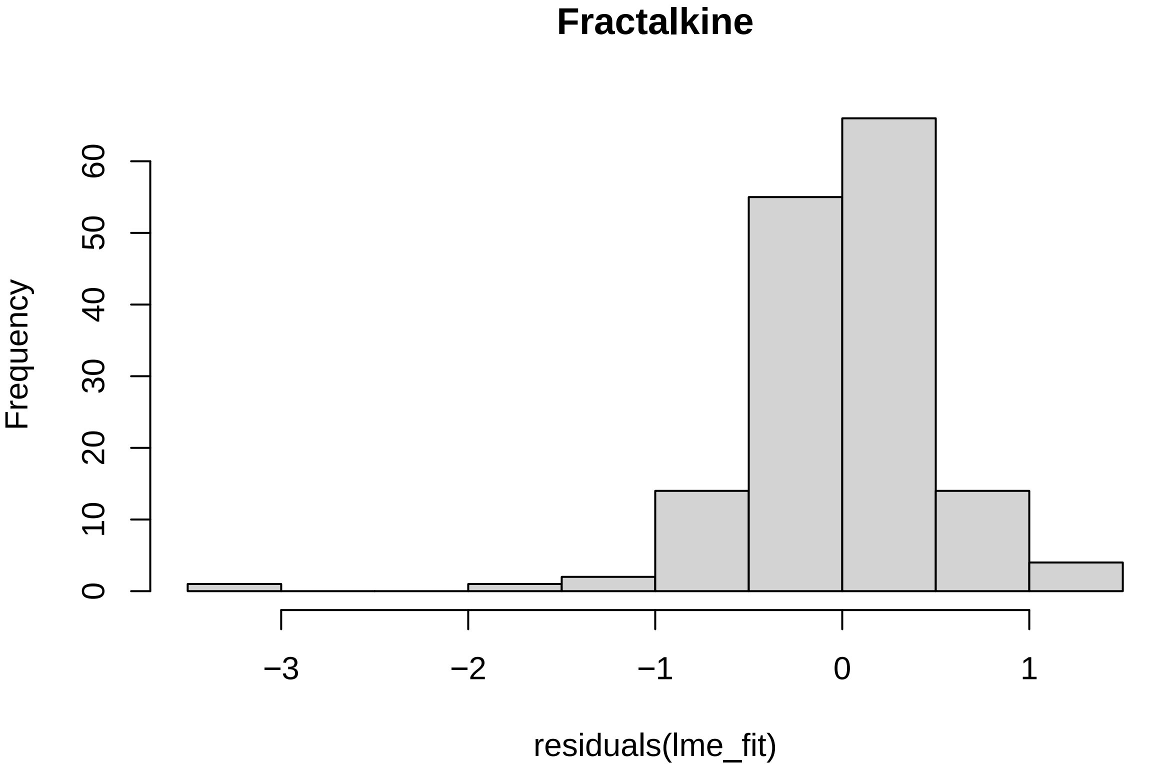


```
## Warning in if (!is.na(a)) {: the condition has length > 1 and only the first
## element will be used
```

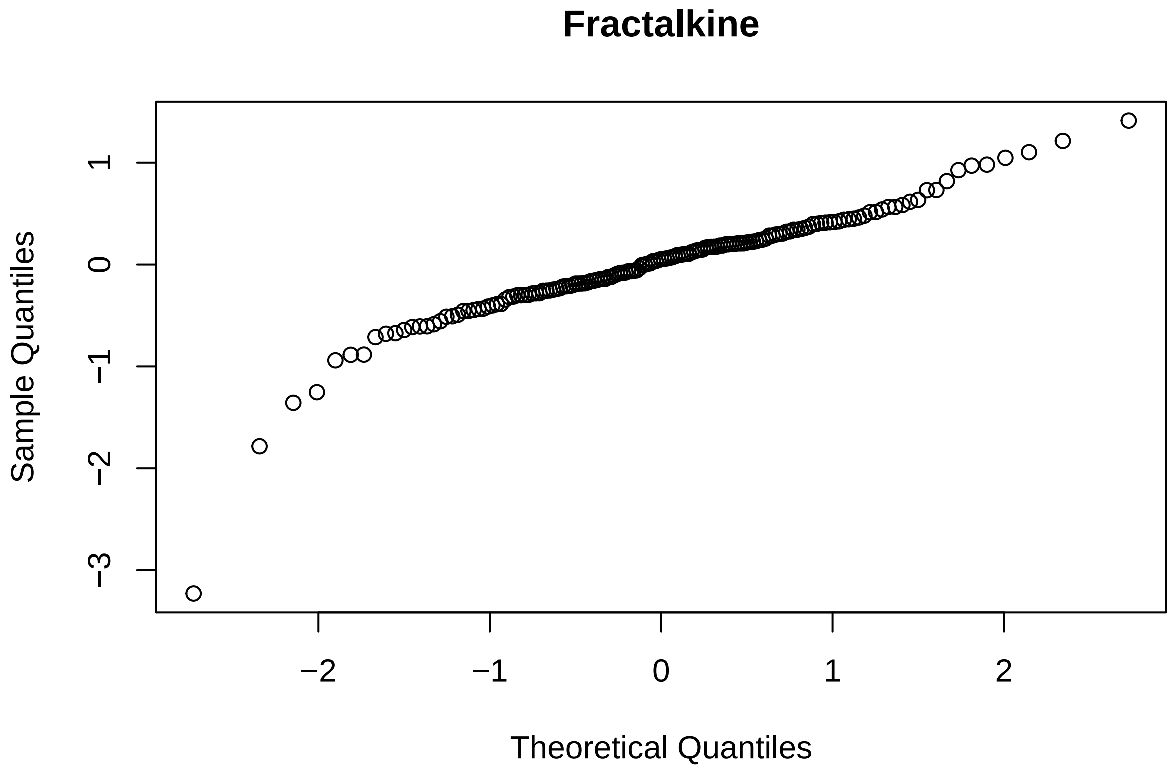
Fractalkine
Sample Quantiles
Theoretical Quantiles
−3
−2
−1
0
1
2

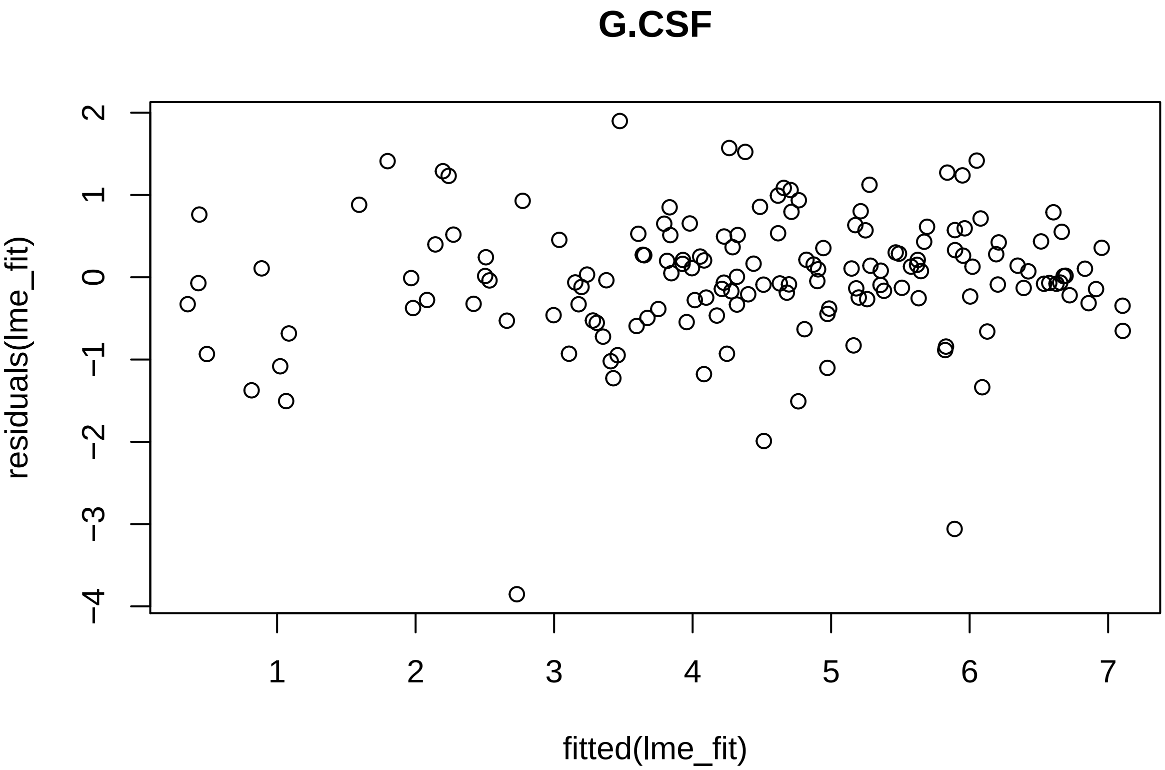
G.CSF
residuals(lme_fit)
fitted(lme_fit)
−4
−3
−2
−1
0
1
2
3
4
5
6
7

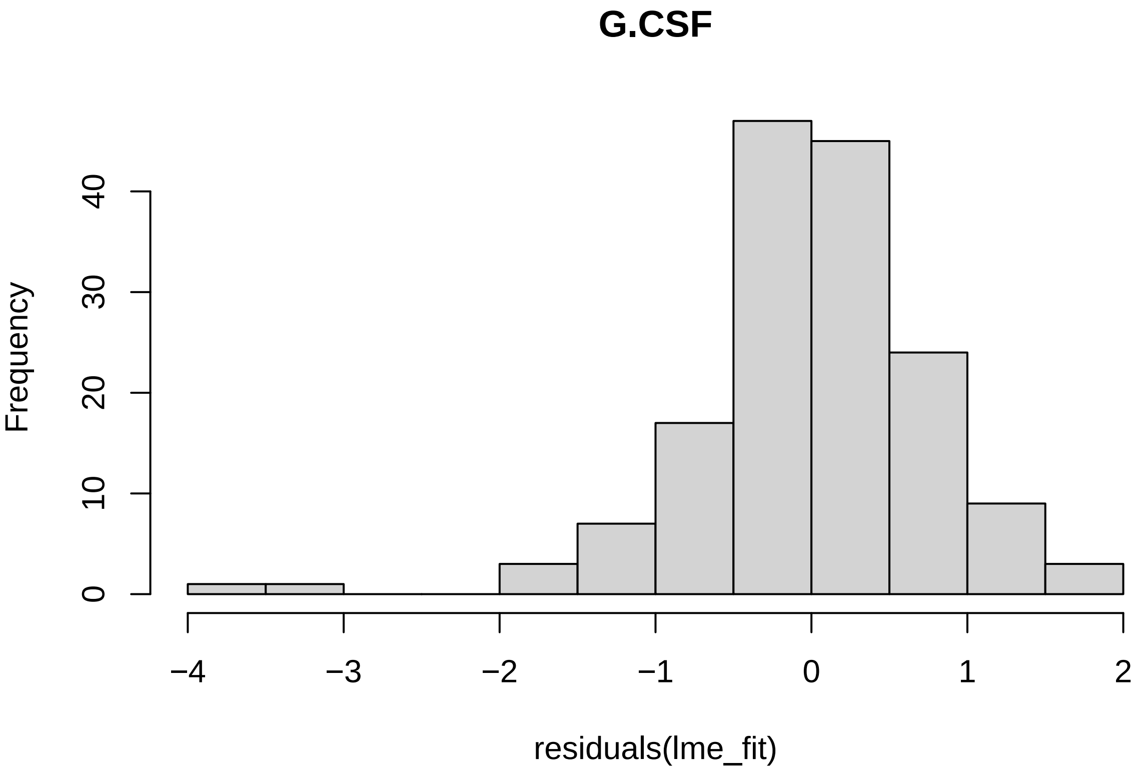


```
## Warning in if (!is.na(a)) {: the condition has length > 1 and only the first
## element will be used
```

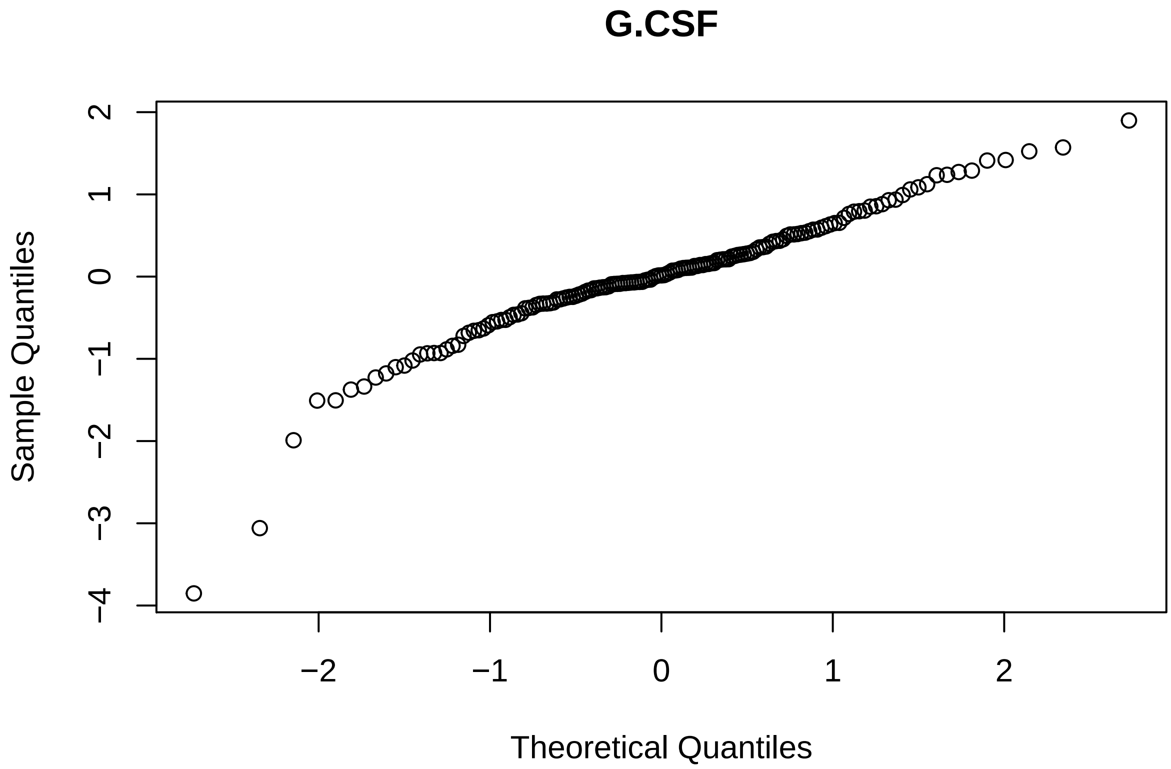

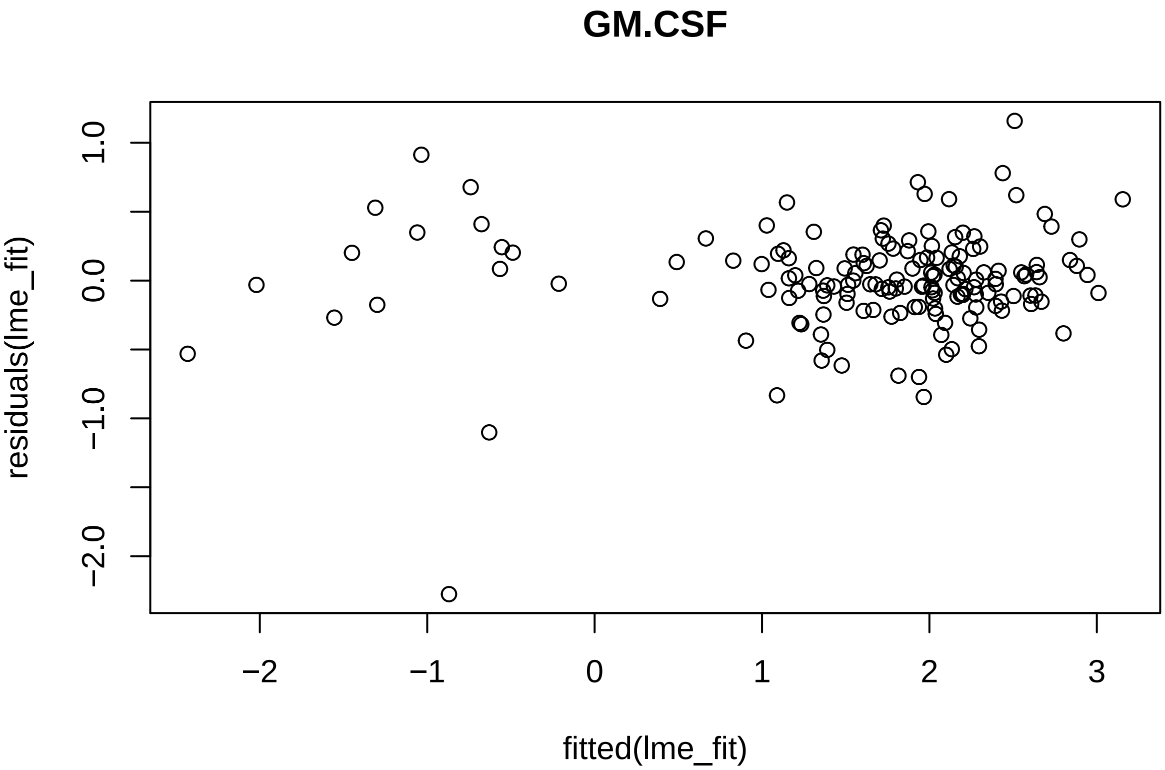

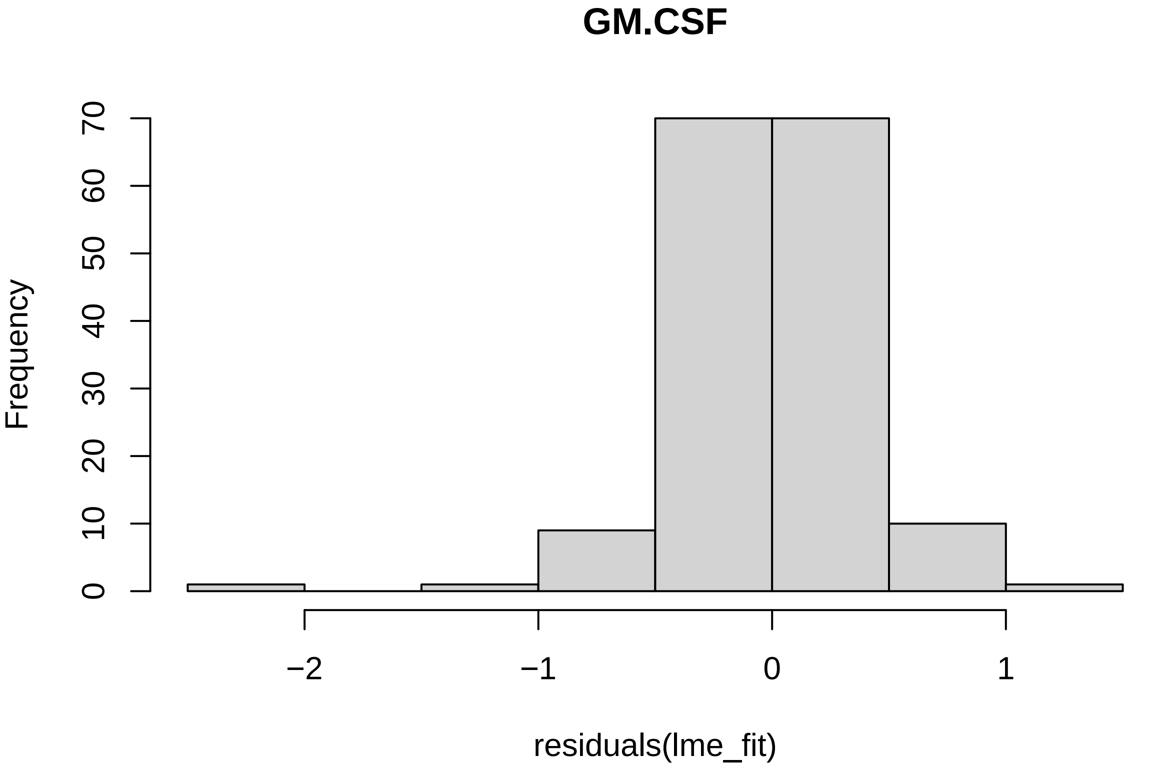


```
## Warning in if (!is.na(a)) {: the condition has length > 1 and only the first
## element will be used
```

GM.CSF

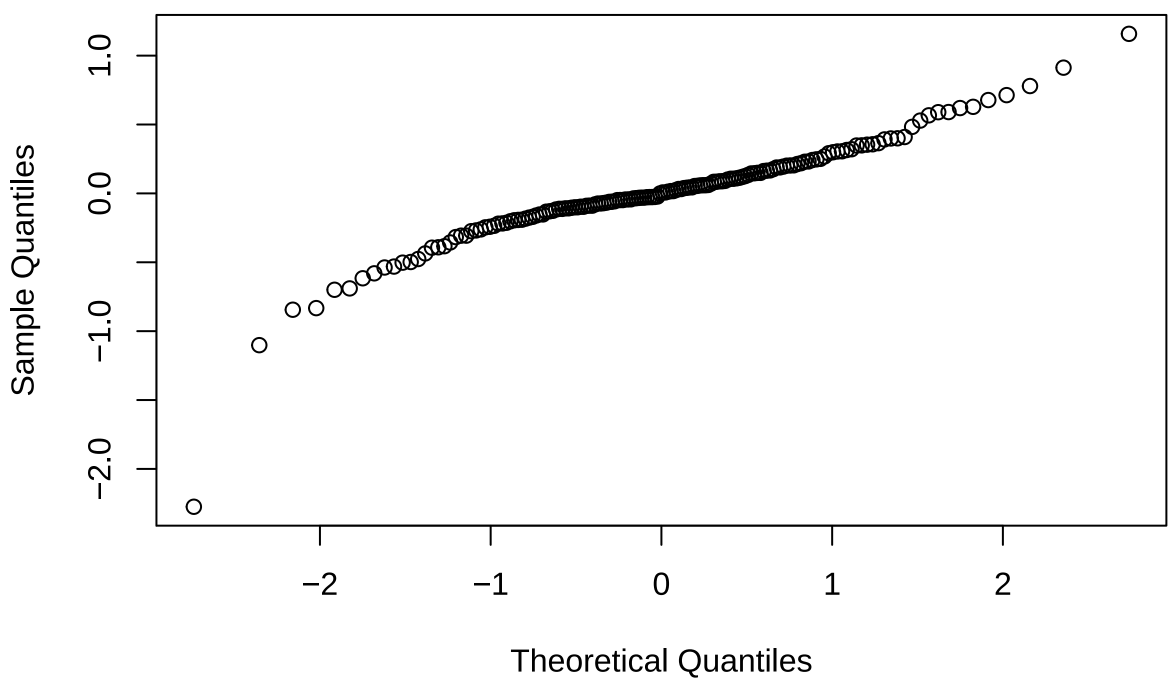

Sample Quantiles
1.0
0.0
−1.0
−2.0
−2
−1
0
1
2
Theoretical Quantiles

GRO

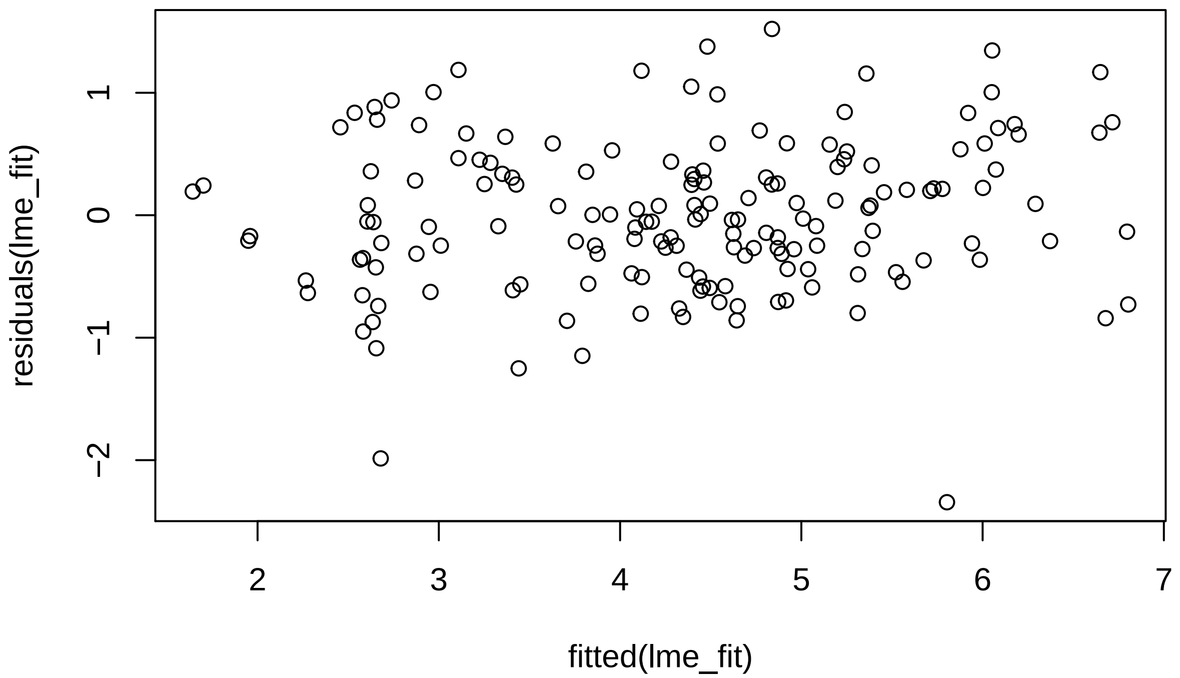

residuals(lme_fit)
1
0
−1
−2
2
3
4
5
6
7
fitted(lme_fit)

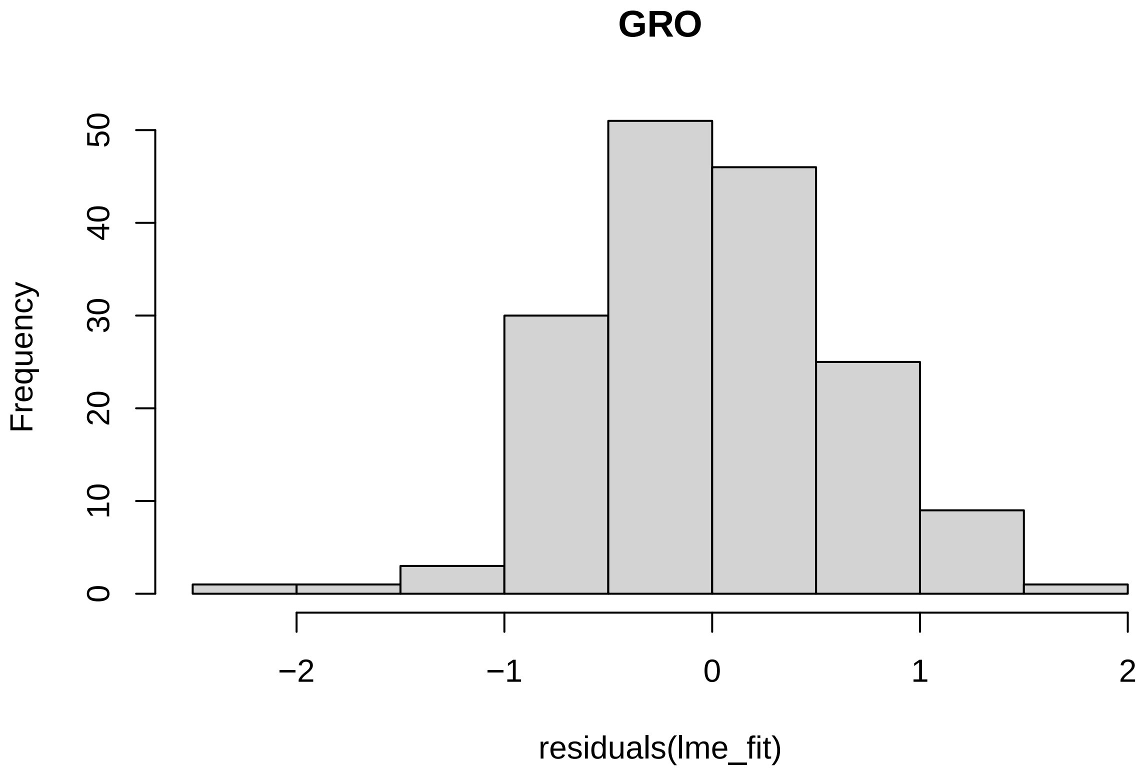


```
## Warning in if (!is.na(a)) {: the condition has length > 1 and only the first
## element will be used
```

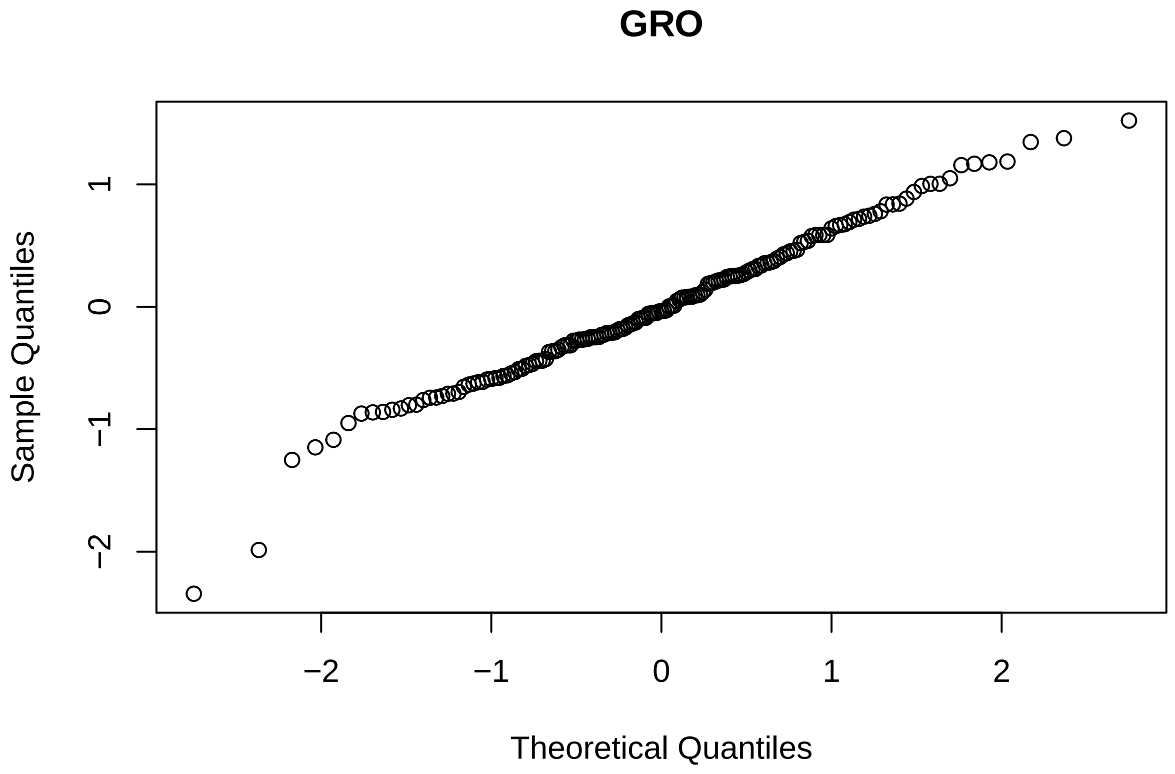

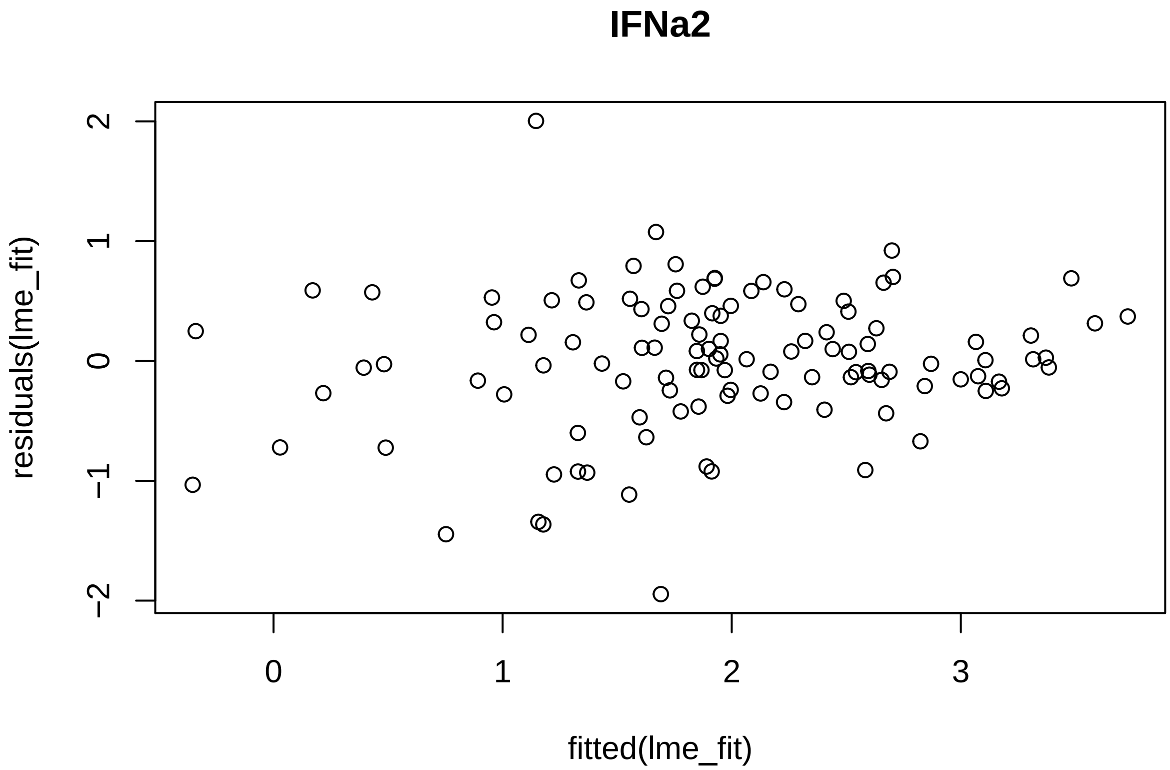

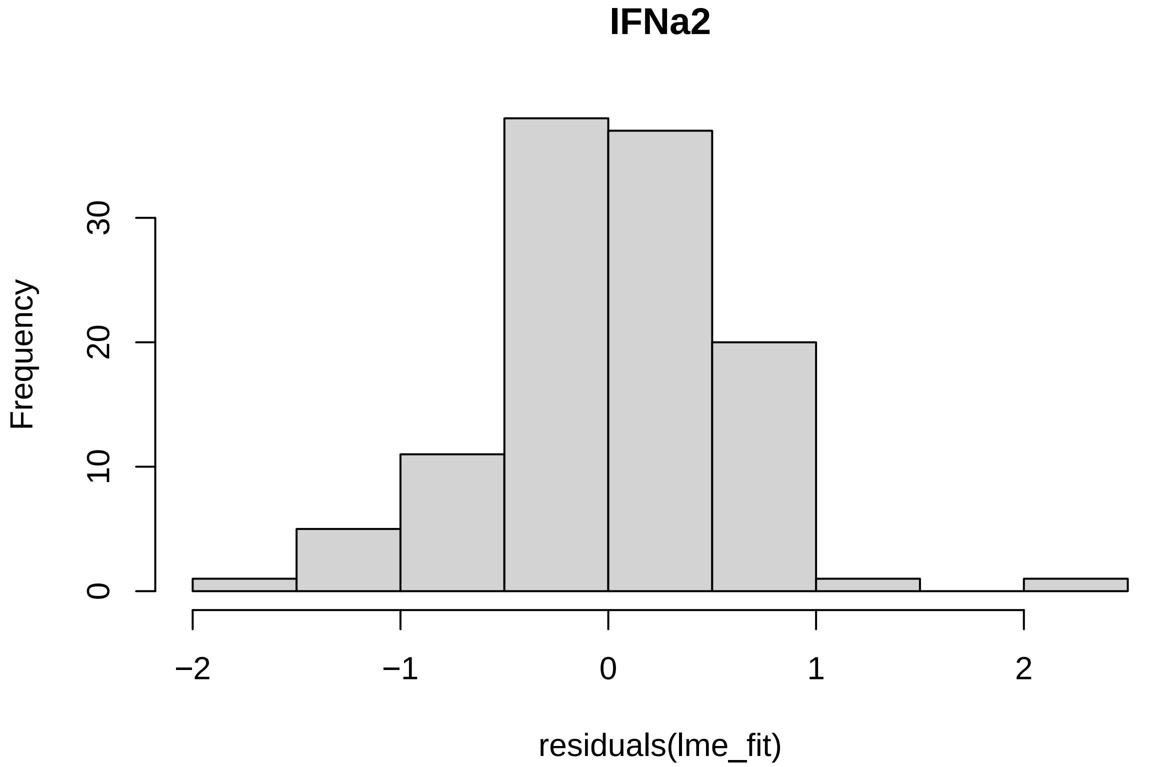


```
## Warning in if (!is.na(a)) {: the condition has length > 1 and only the first
## element will be used
```

IFNa2

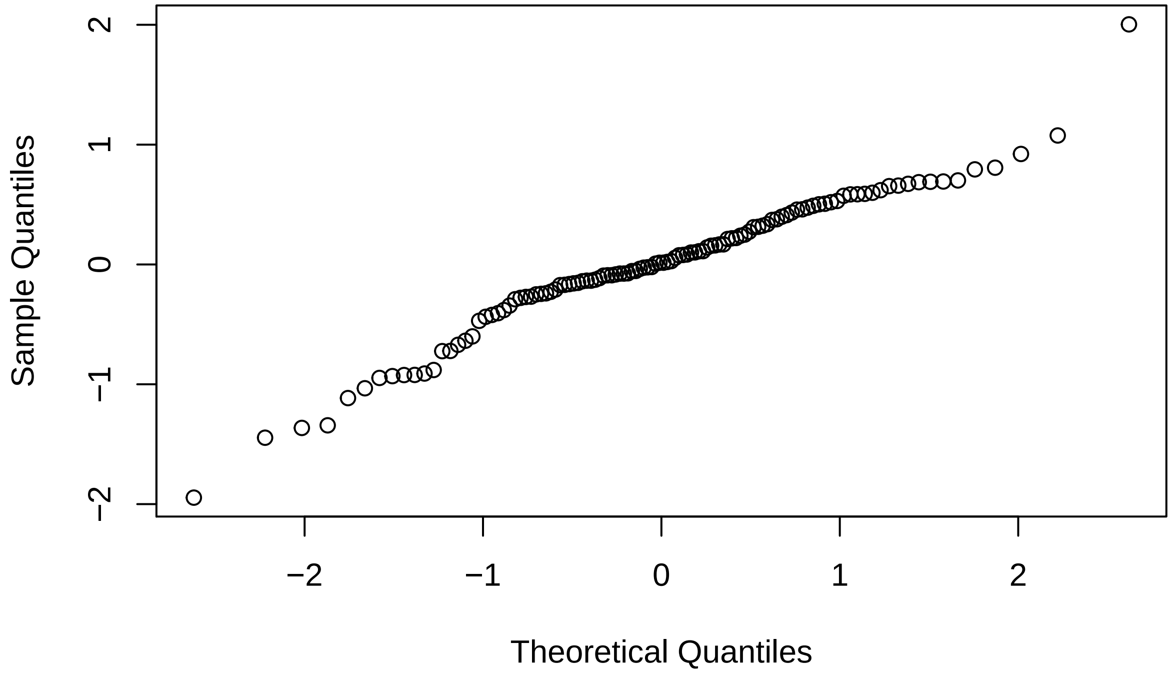
Sample Quantiles
Theoretical Quantiles
2
1
0
−1
−2
−2
−1
0
1
2

IFNg

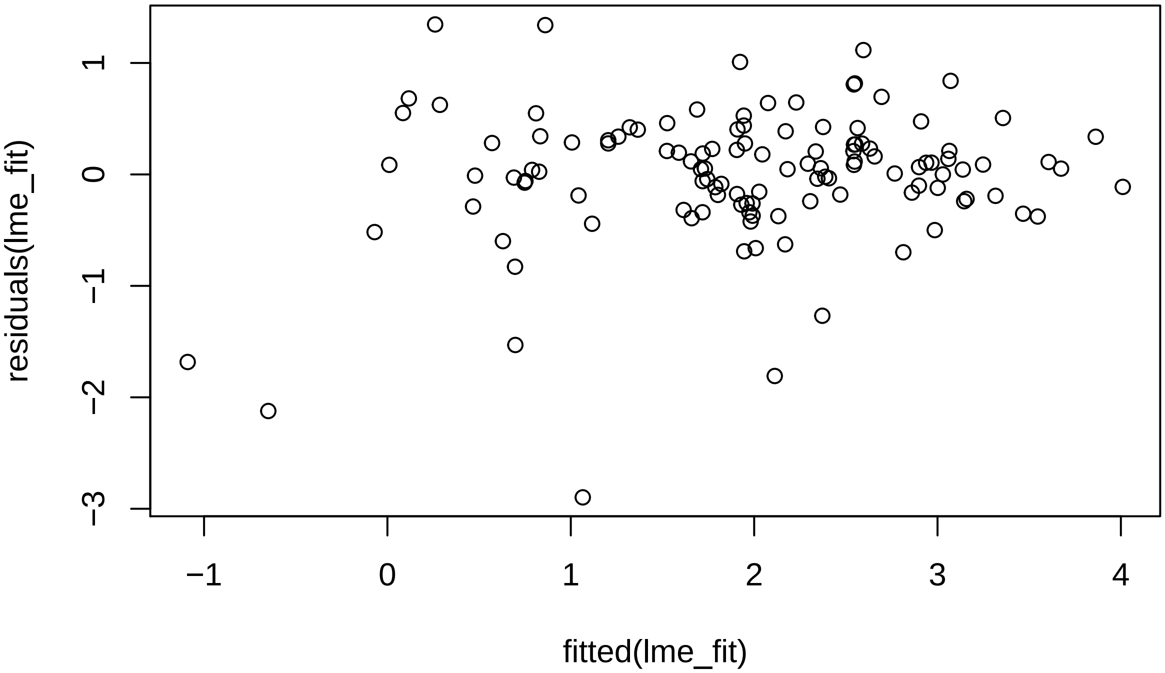
residuals(lme_fit)
fitted(lme_fit)
1
0
−1
−2
−3
−1
0
1
2
3
4

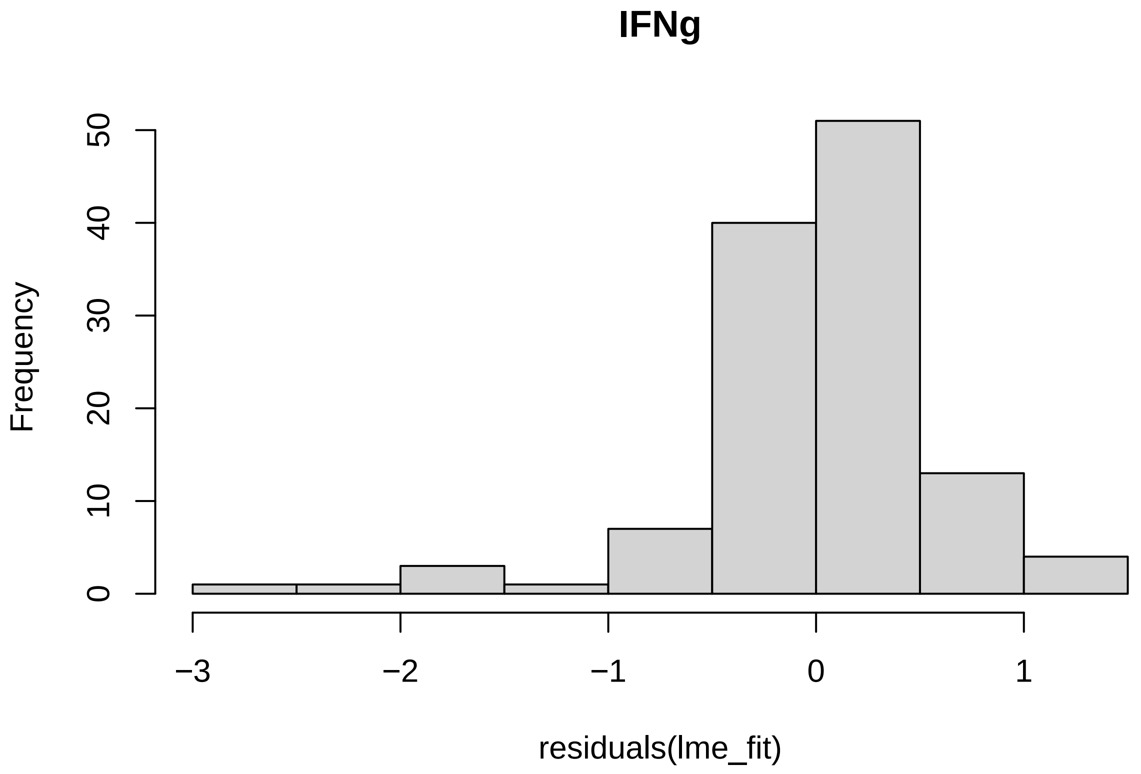


```
## Warning in if (!is.na(a)) {: the condition has length > 1 and only the first
## element will be used
```

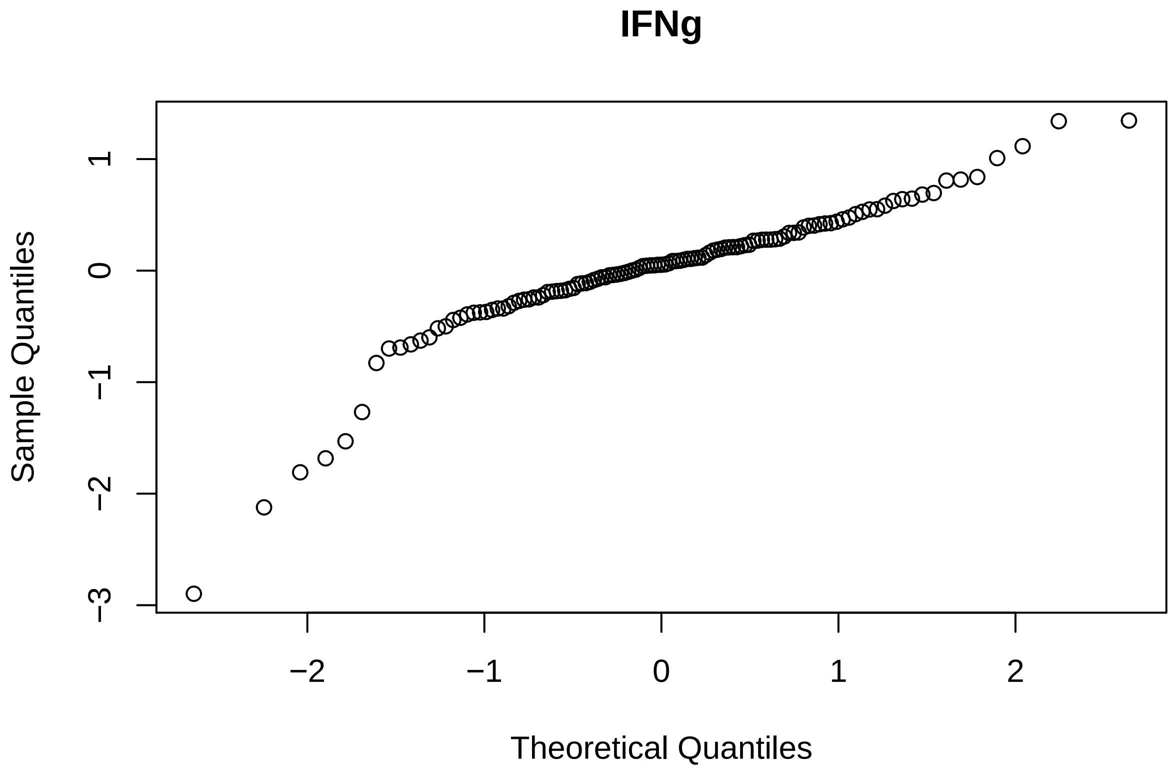

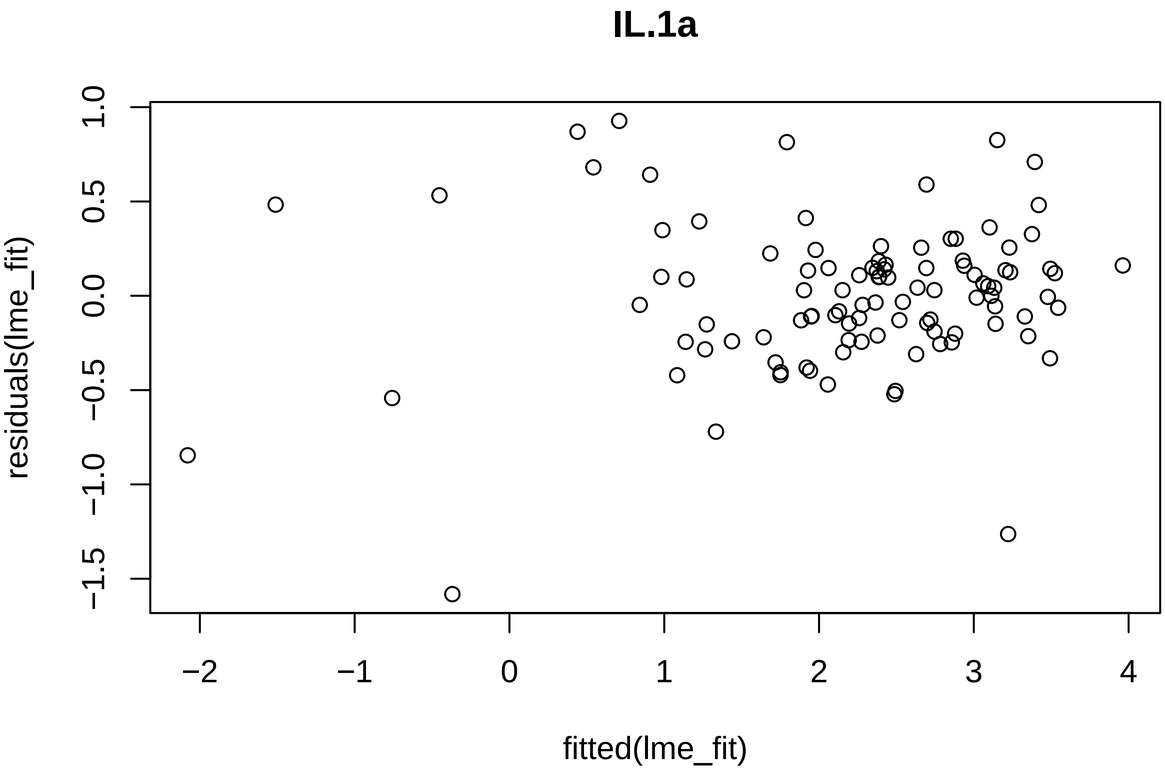
IL.1a
residuals(lme_fit)
1.0
0.5
0.0
−0.5
−1.0
−1.5
−2
−1
0
1
2
3
4
fitted(lme_fit)

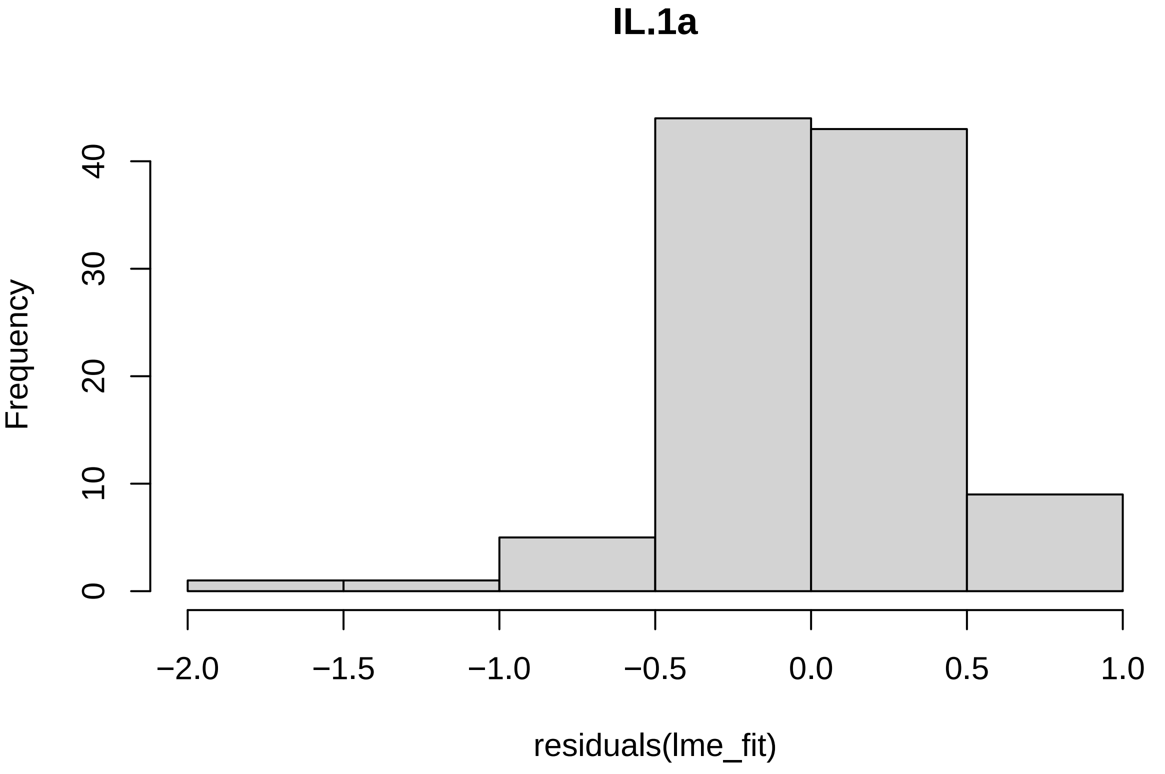
IL.1a
Frequency
0
10
20
30
40
−2.0
−1.5
−1.0
−0.5
0.0
0.5
1.0
residuals(lme_fit)

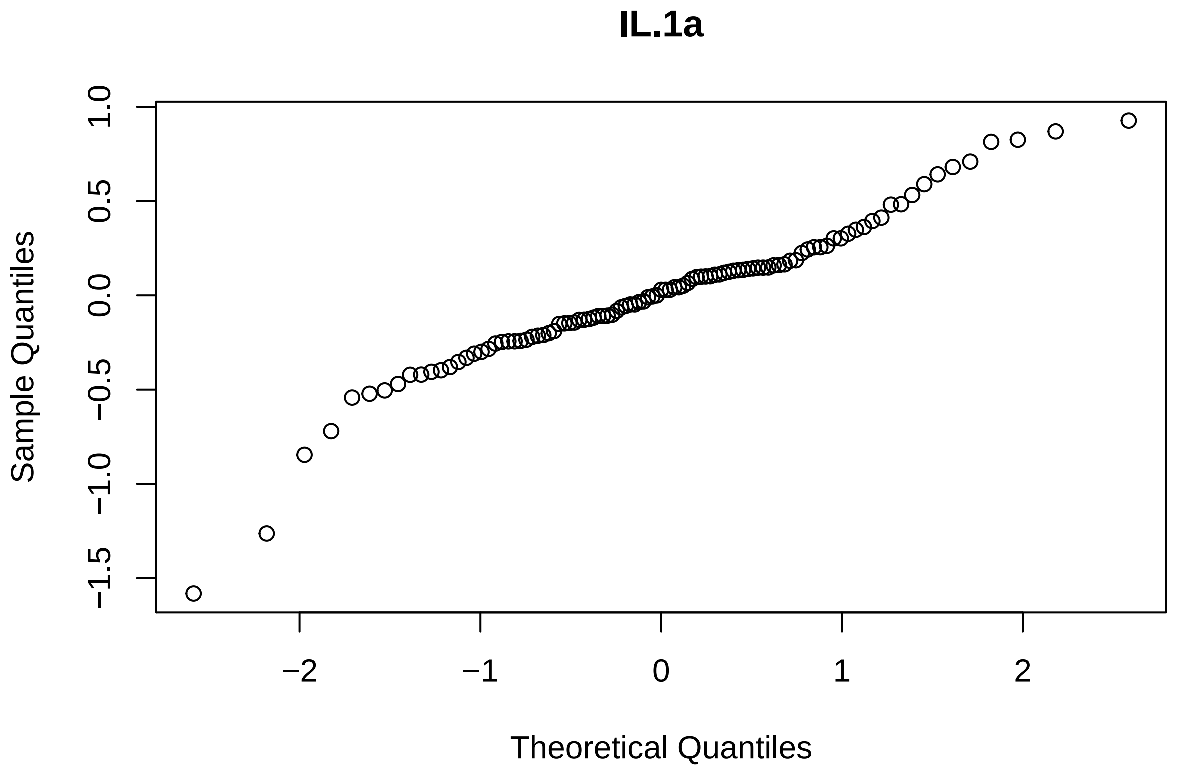
IL.1a
Sample Quantiles
Theoretical Quantiles
1.0
0.5
0.0
−0.5
−1.0
−1.5
−2
−1
0
1
2

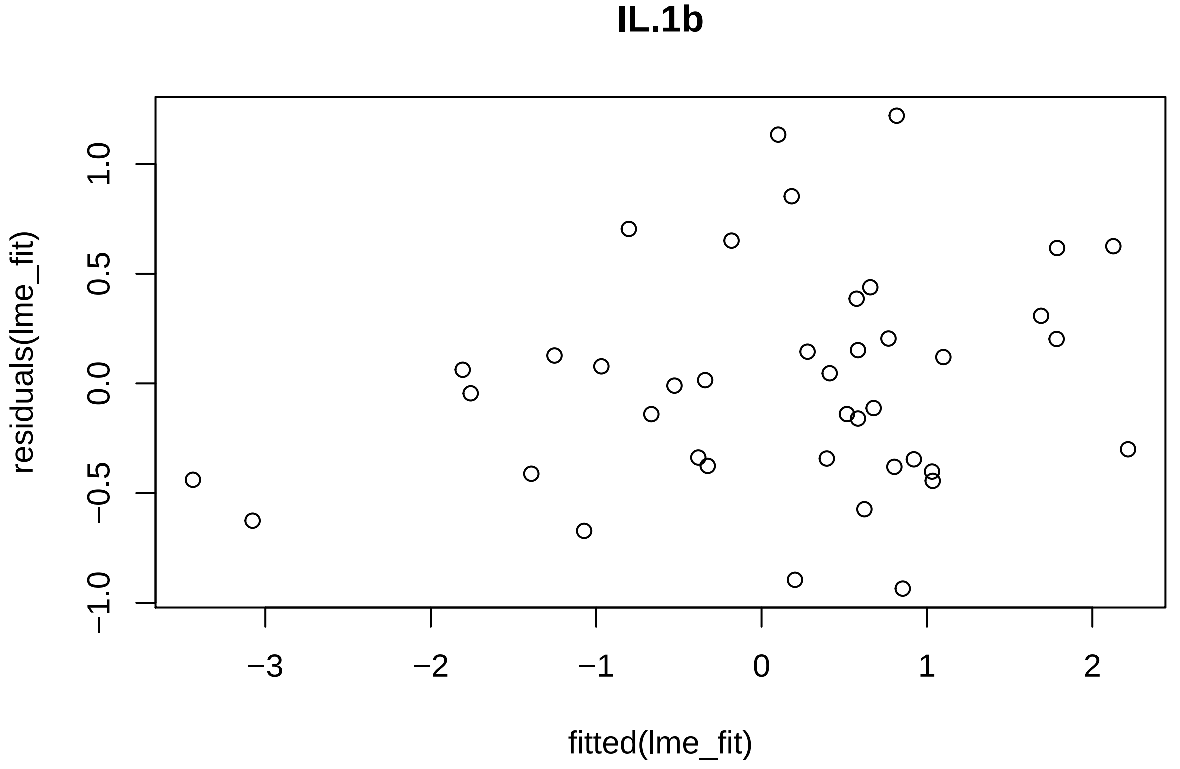
IL.1b
residuals(lme_fit)
fitted(lme_fit)
1.0
0.5
0.0
−0.5
−1.0
−3
−2
−1
0
1
2

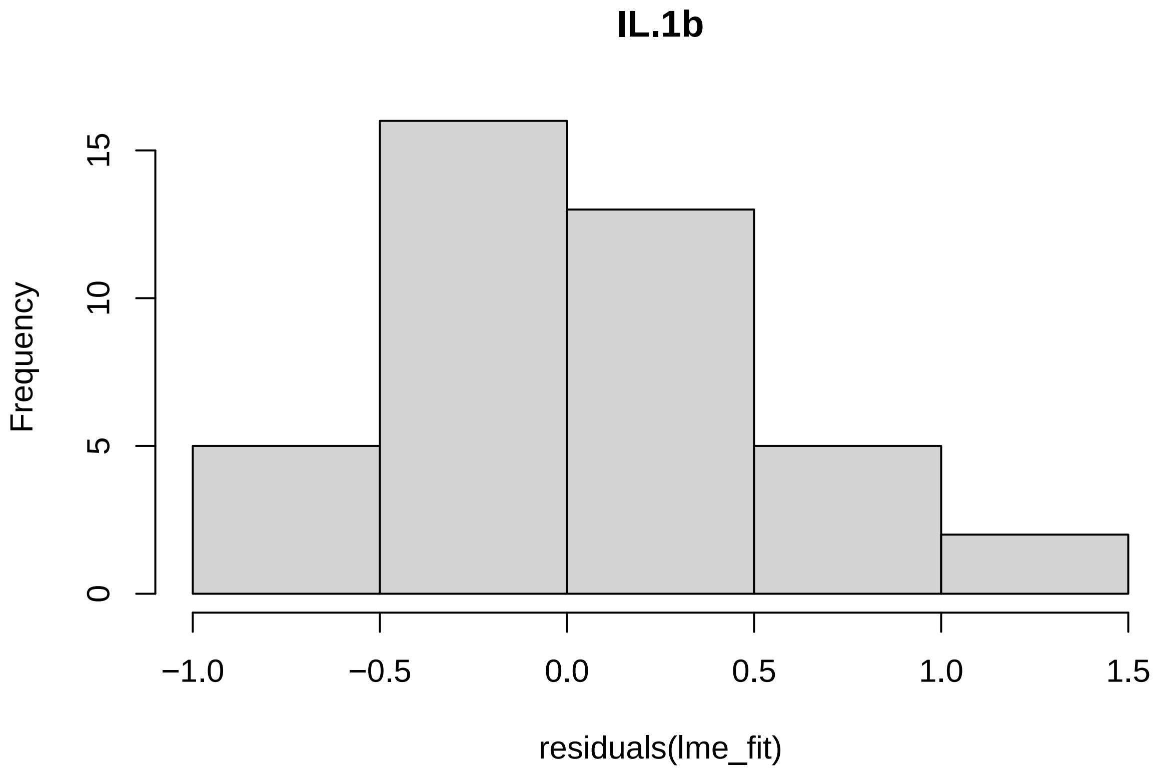


```
## Warning in if (!is.na(a)) {: the condition has length > 1 and only the first
## element will be used
```

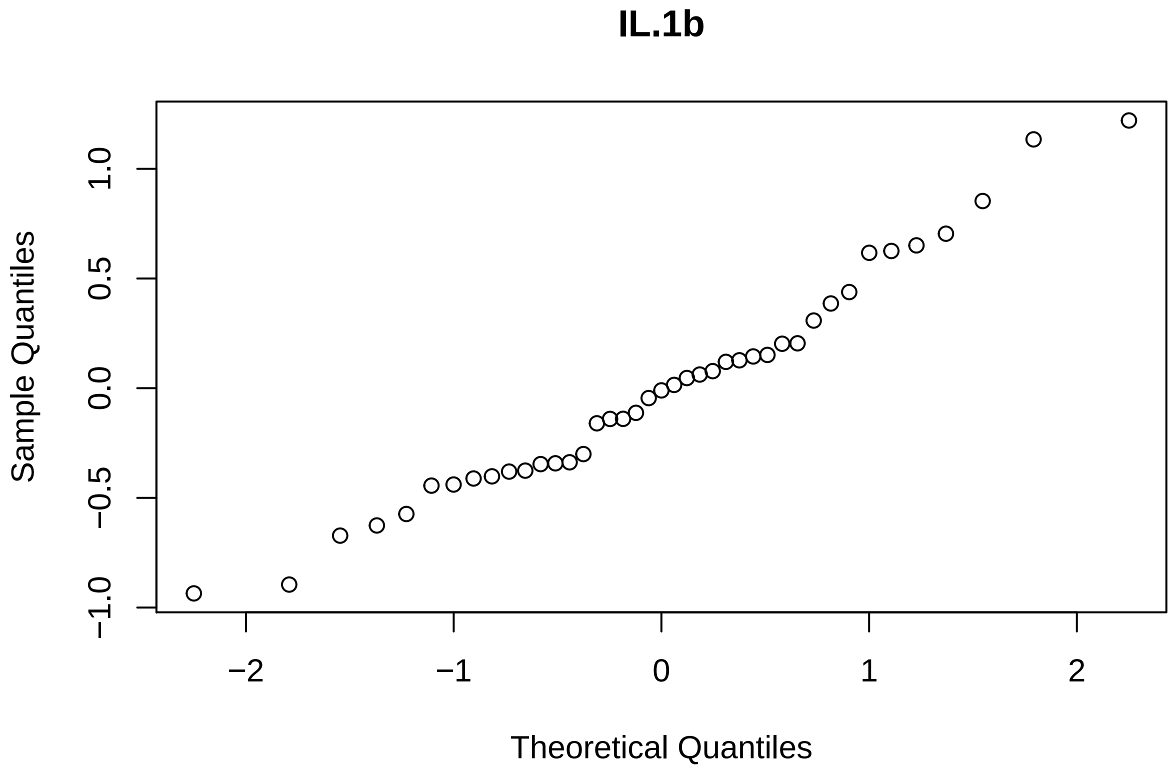

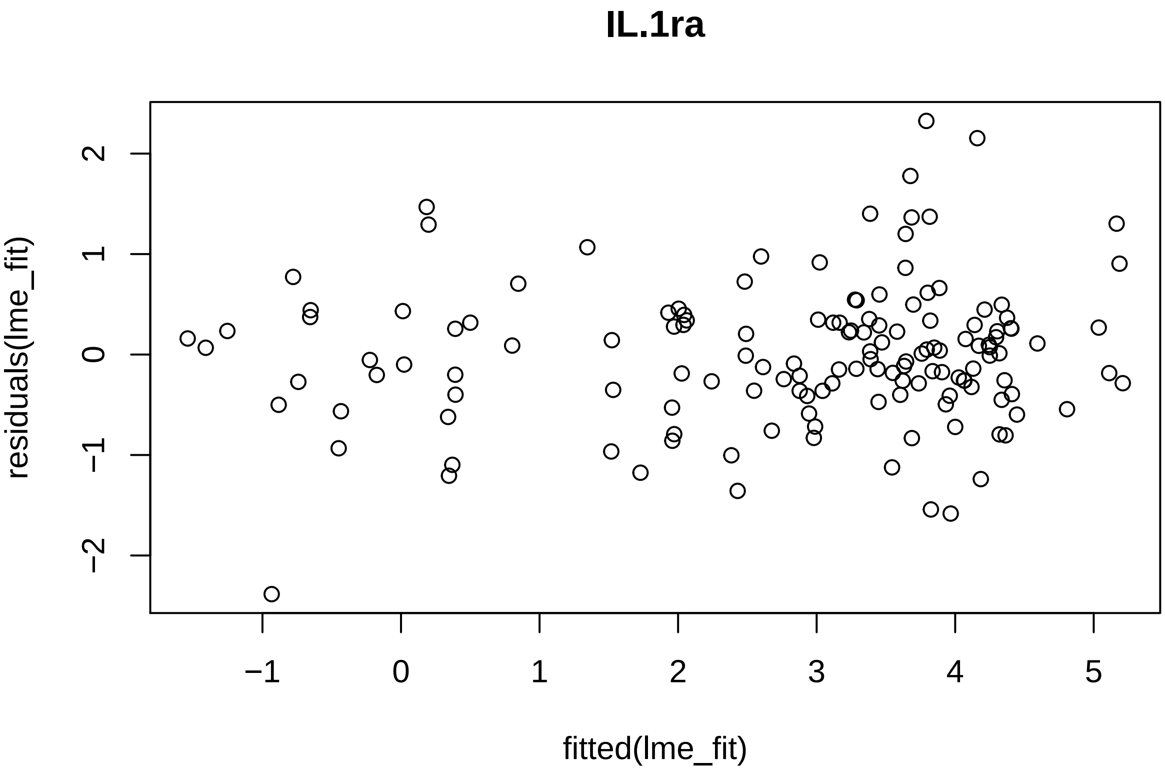

IL.1ra
residuals(lme_fit)
fitted(lme_fit)

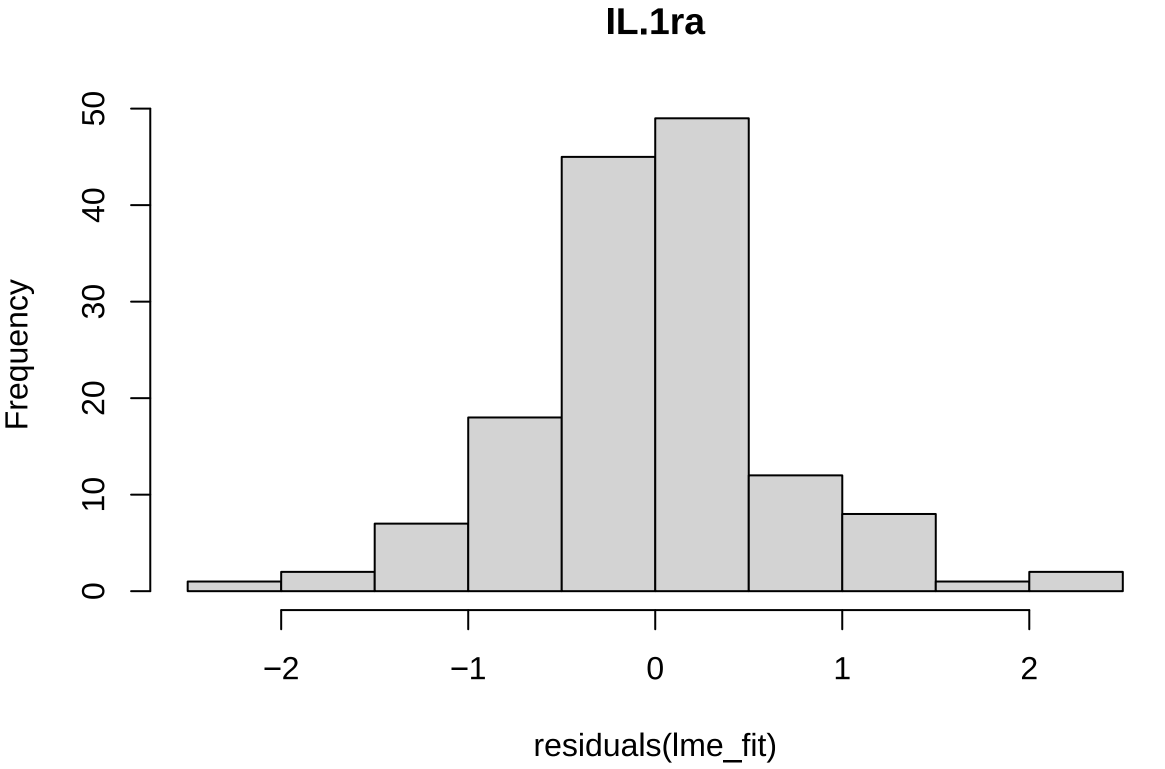

IL.1ra
Frequency
residuals(lme_fit)

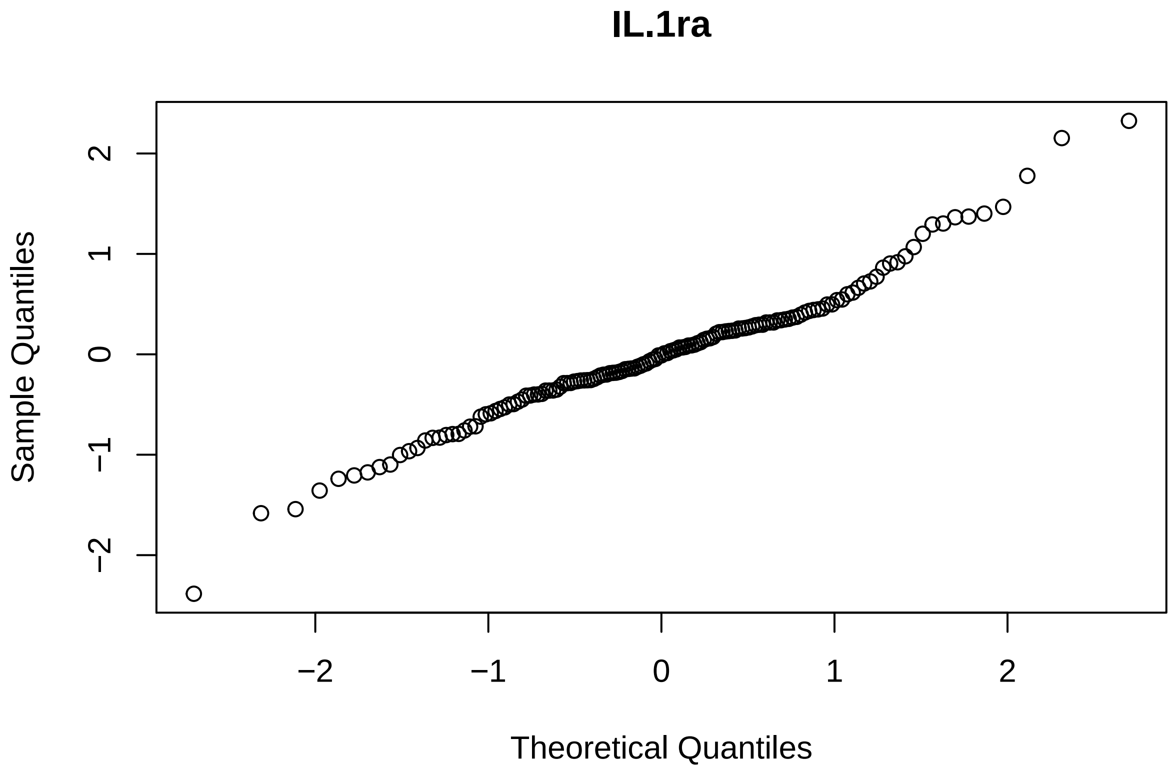


```
## [1] "Error: MD_IL.2 ~ A_IL.2 + time + WCC + CRP + Temp + Infection + rhIL1ra"
## [1] "Error: MD_IL.3 ~ A_IL.3 + time + WCC + CRP + Temp + Infection + rhIL1ra"
```

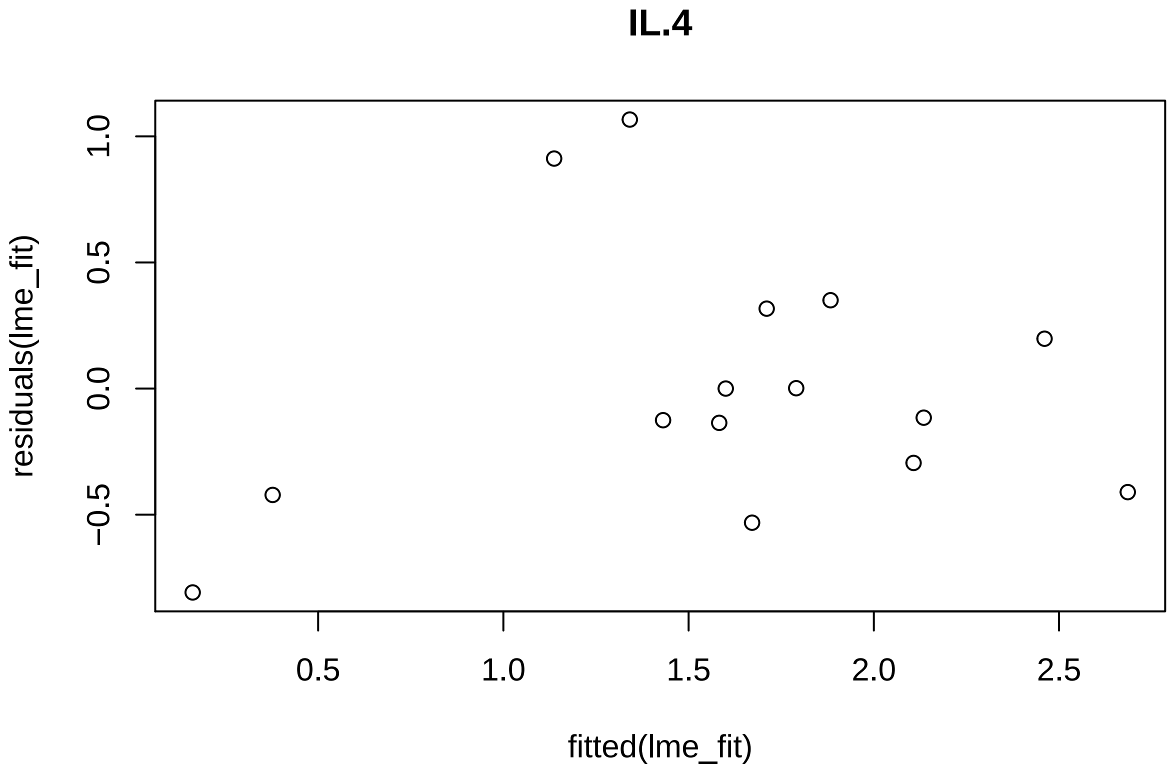

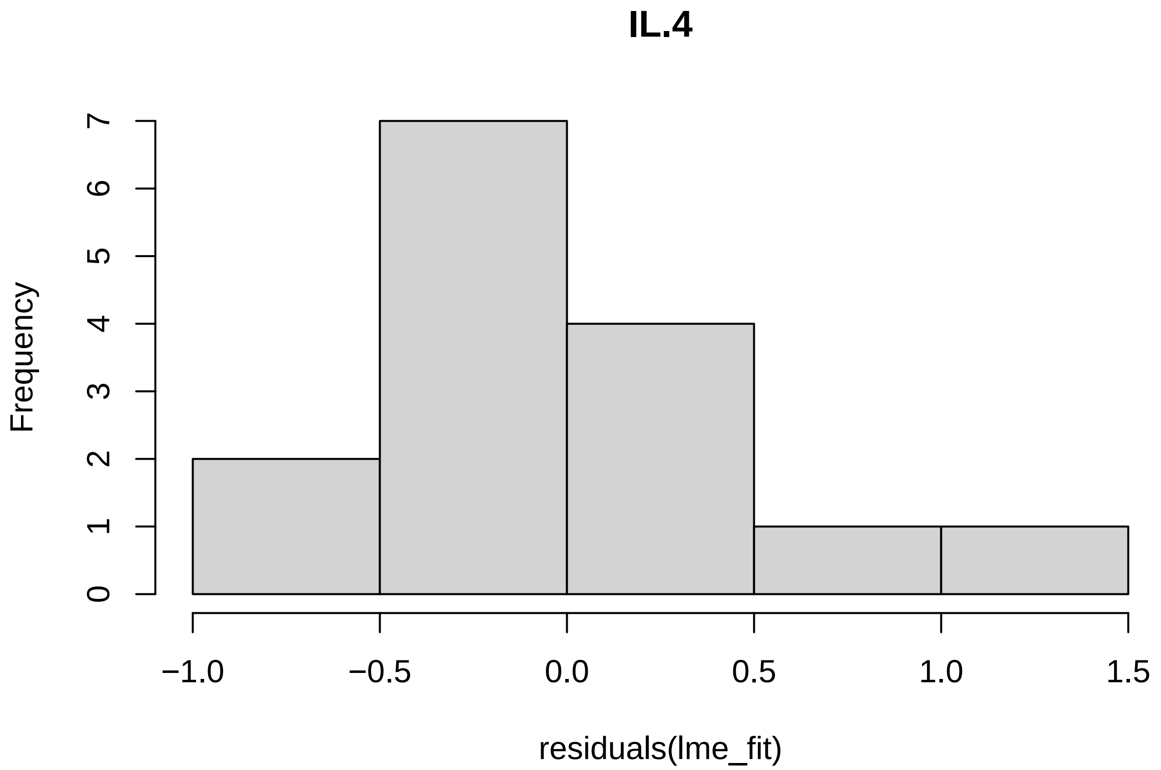


```
## [1] "Error: MD_IL.5 ~ A_IL.5 + time + WCC + CRP + Temp + Infection + rhIL1ra"

## Warning in if (!is.na(a)) {: the condition has length > 1 and only the first
## element will be used
```

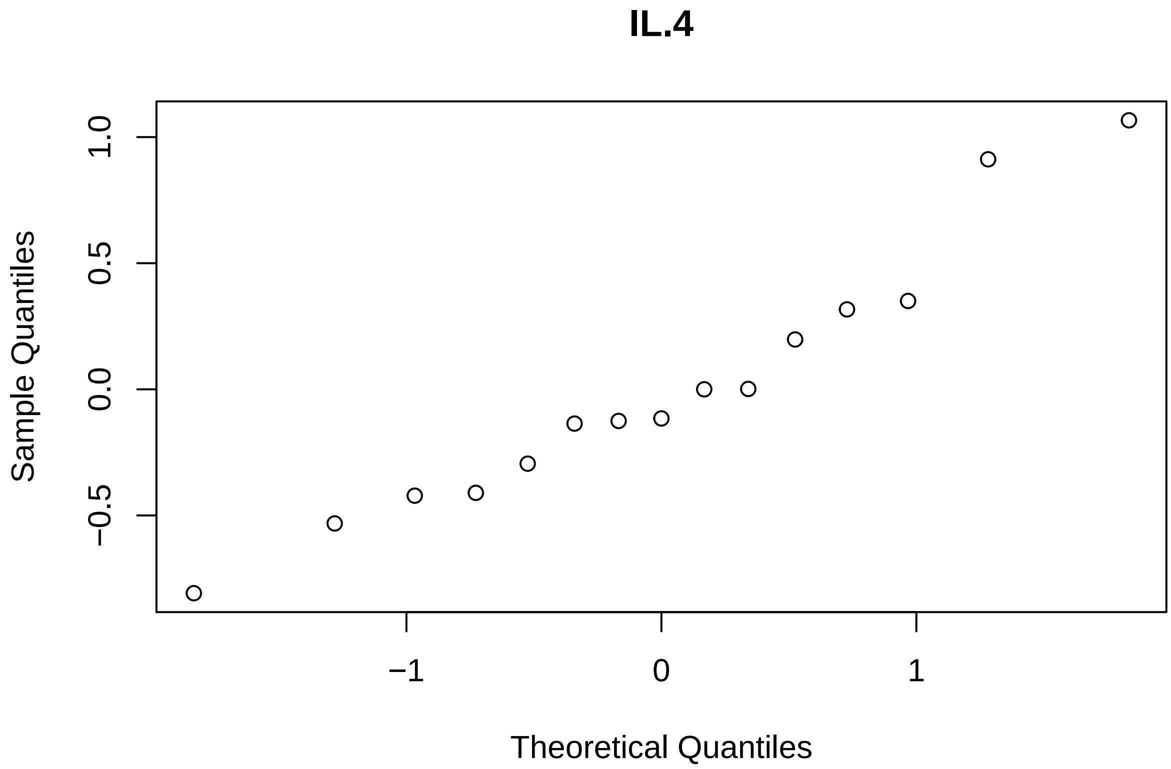

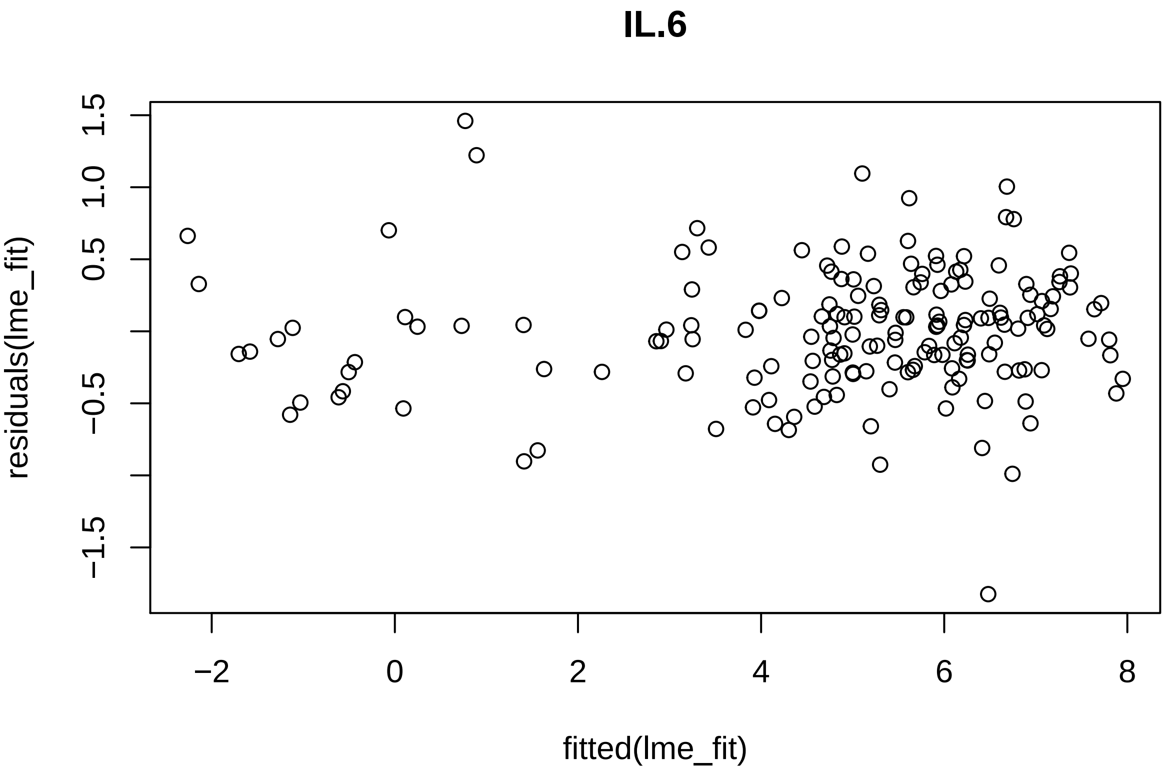

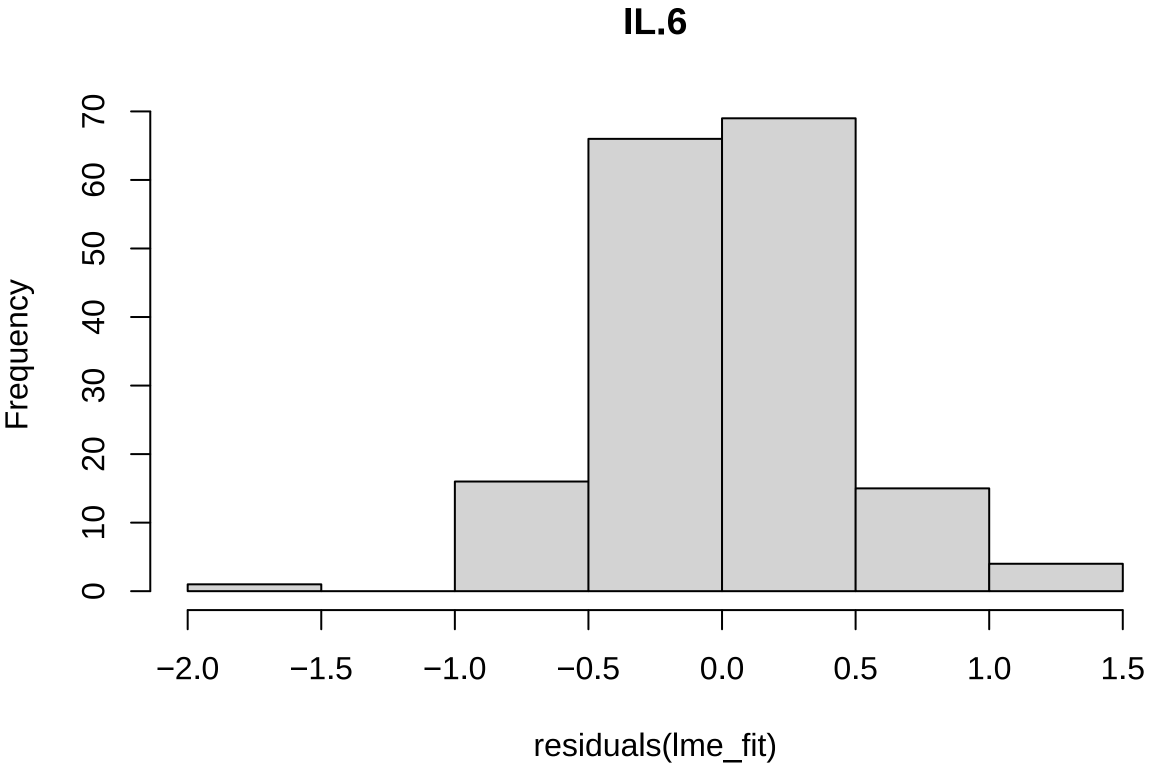


```
## Warning in if (!is.na(a)) {: the condition has length > 1 and only the first
## element will be used
```

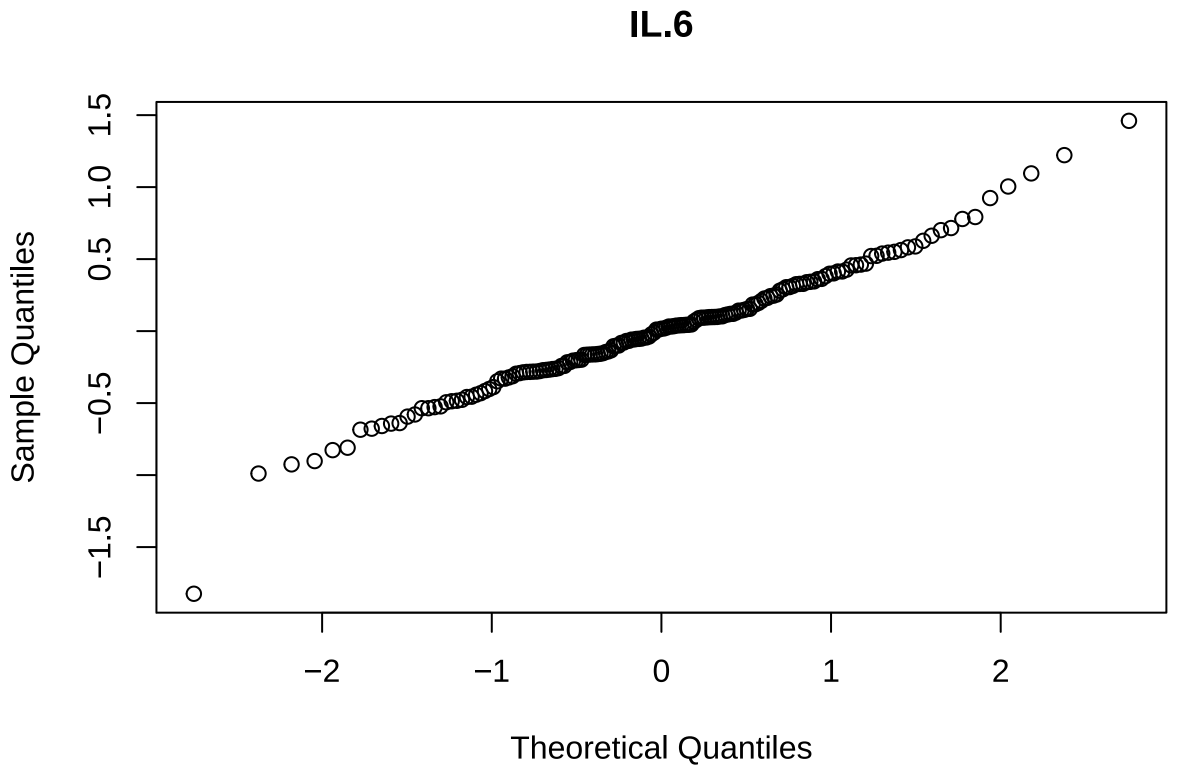
IL.6
Sample Quantiles
Theoretical Quantiles
1.5
1.0
0.5
−0.5
−1.5
−2
−1
0
1
2

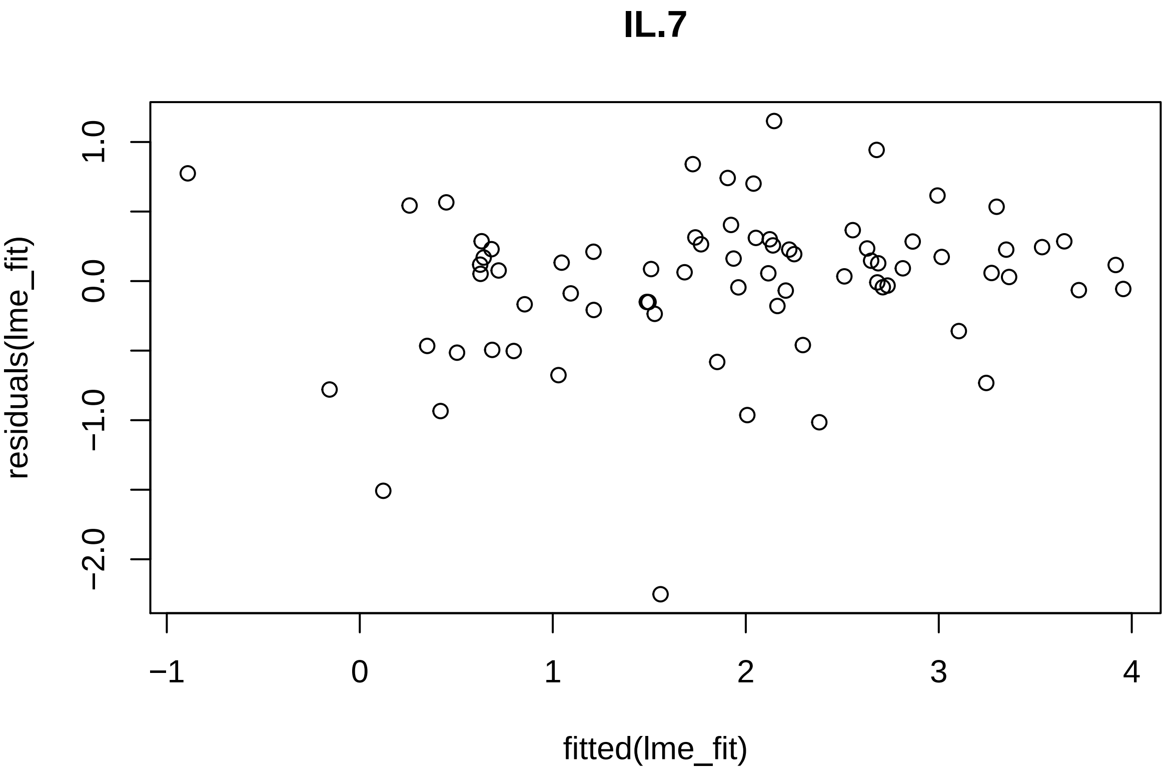
IL.7
residuals(lme_fit)
fitted(lme_fit)
1.0
0.0
−1.0
−2.0
−1
0
1
2
3
4

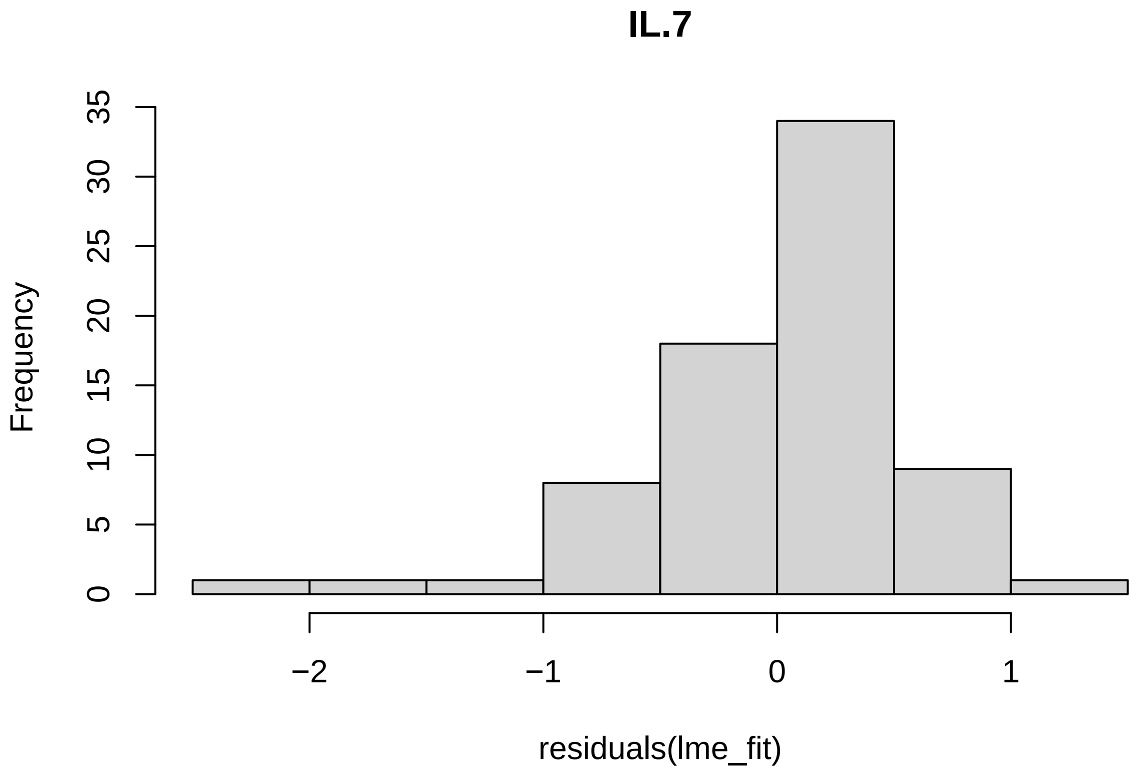


```
## Warning in if (!is.na(a)) {: the condition has length > 1 and only the first
## element will be used
```

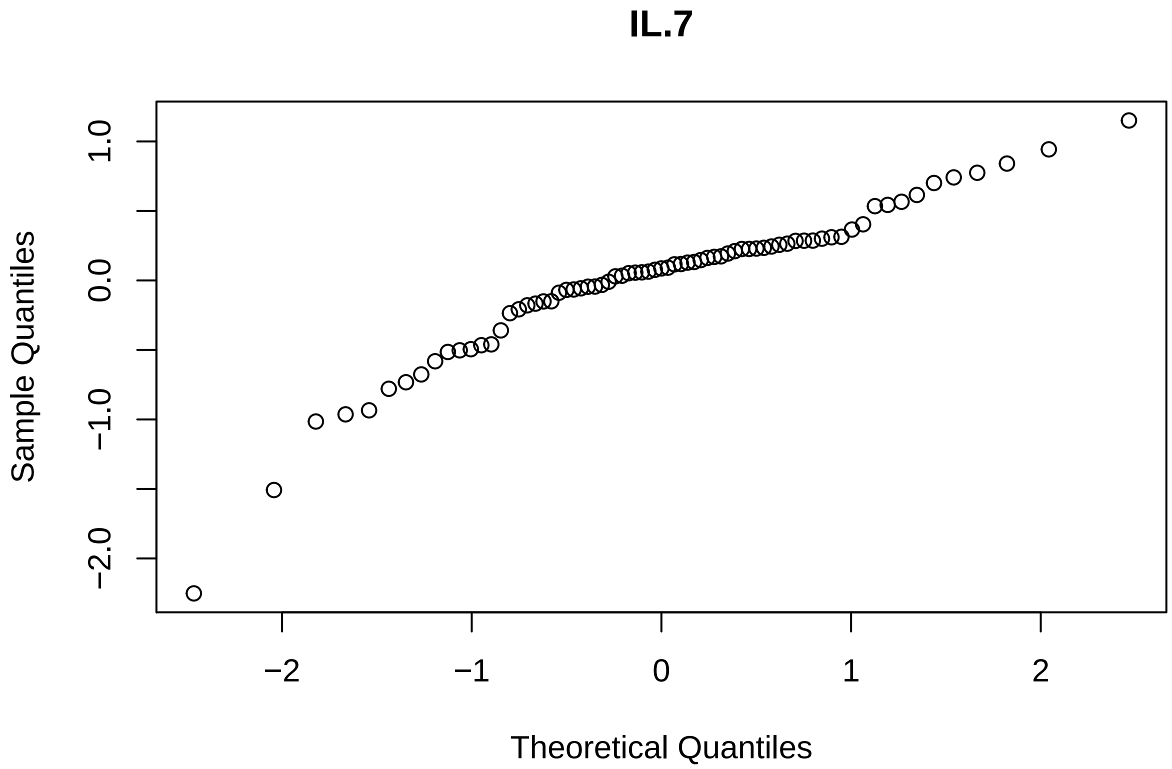

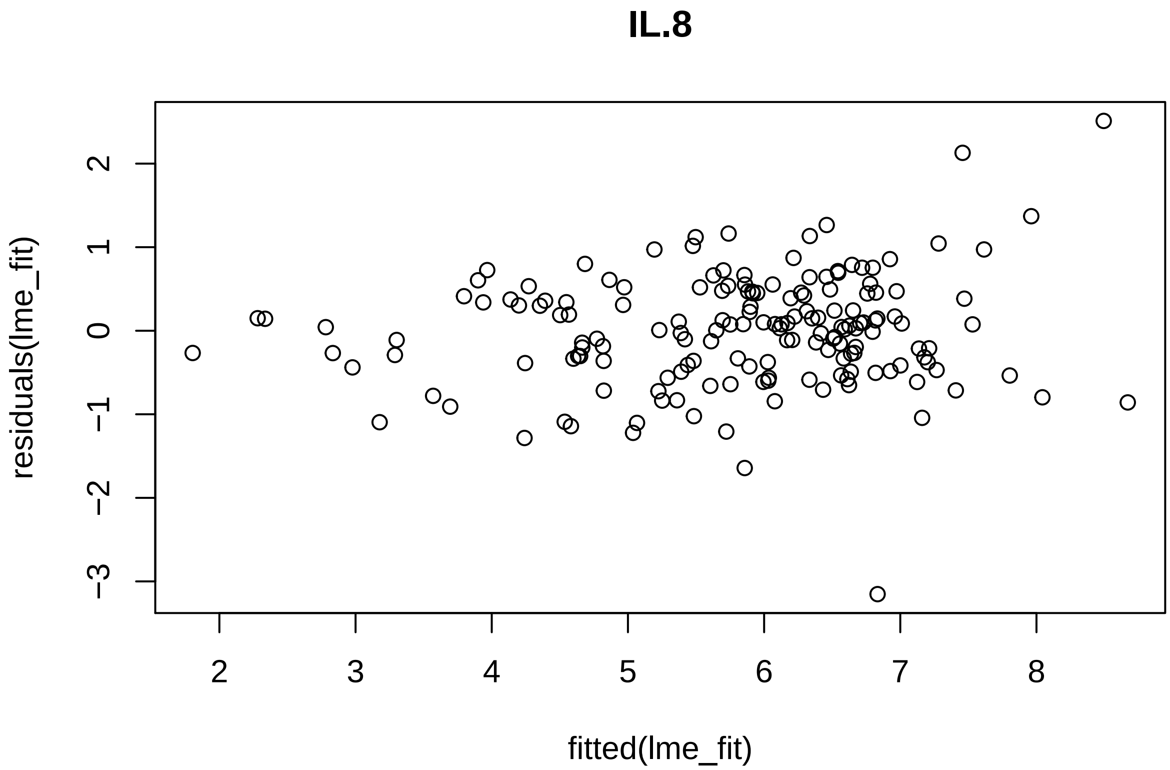
IL.8
residuals(lme_fit)
fitted(lme_fit)
2
1
0
−1
−2
−3
2
3
4
5
6
7
8

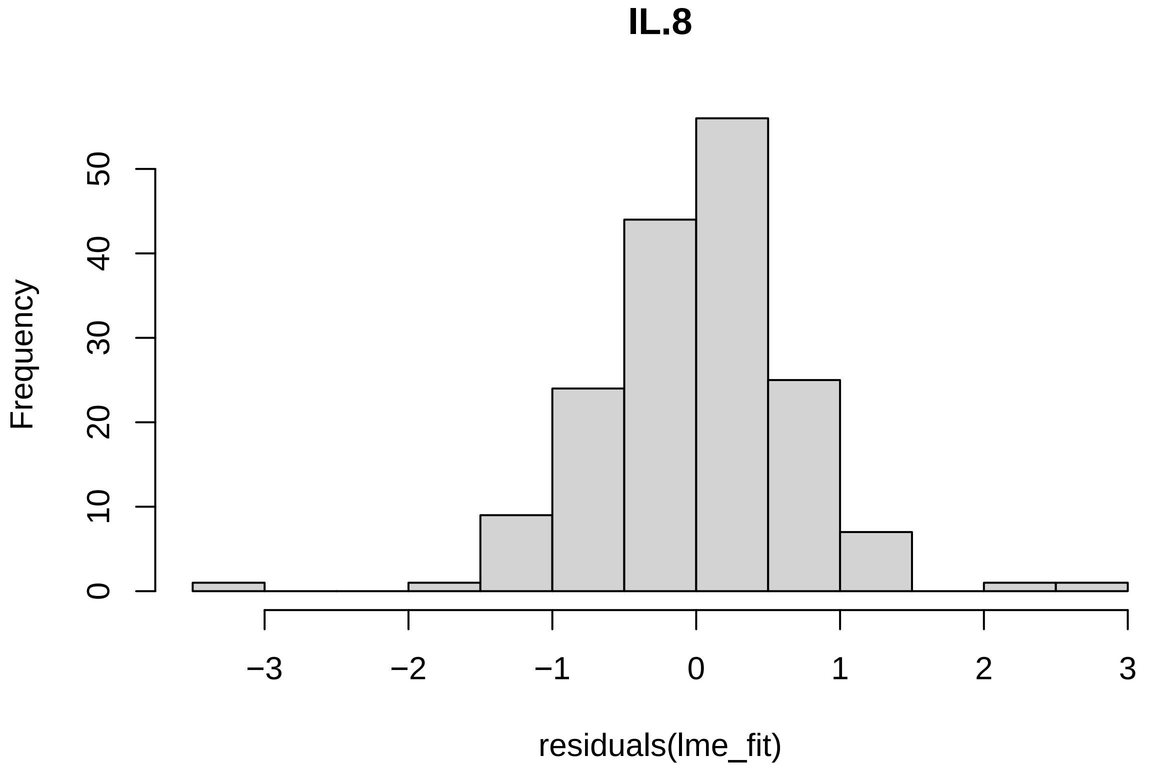
IL.8
Frequency
residuals(lme_fit)
50
40
30
20
10
0
−3
−2
−1
0
1
2
3

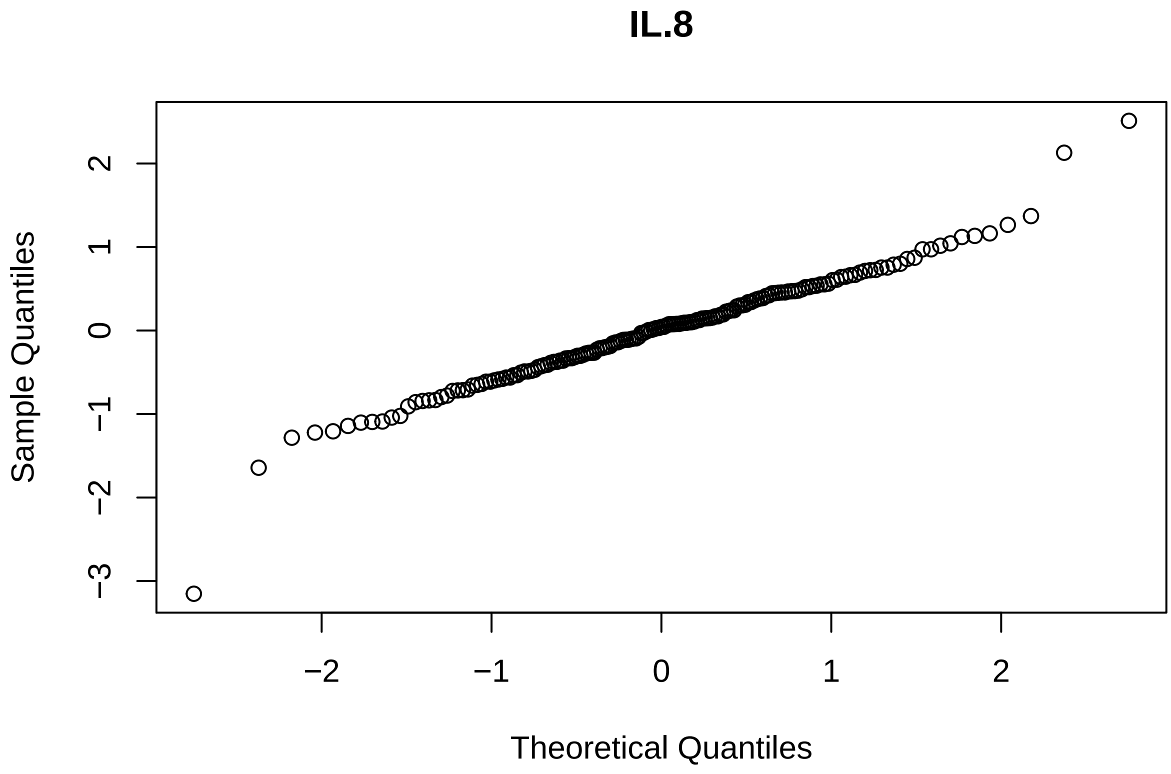
IL.8
Sample Quantiles
Theoretical Quantiles
−3
−2
−1
0
1
2

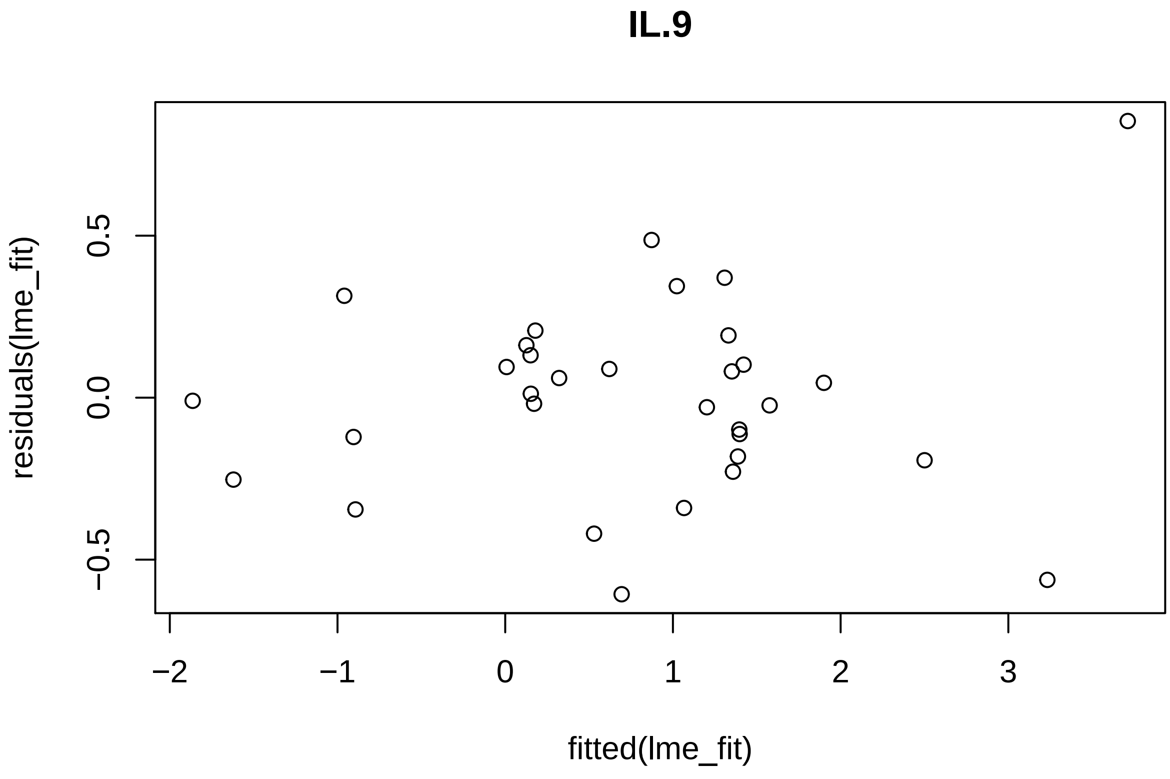
IL.9
residuals(lme_fit)
fitted(lme_fit)
−0.5
0.0
0.5
−2
−1
0
1
2
3

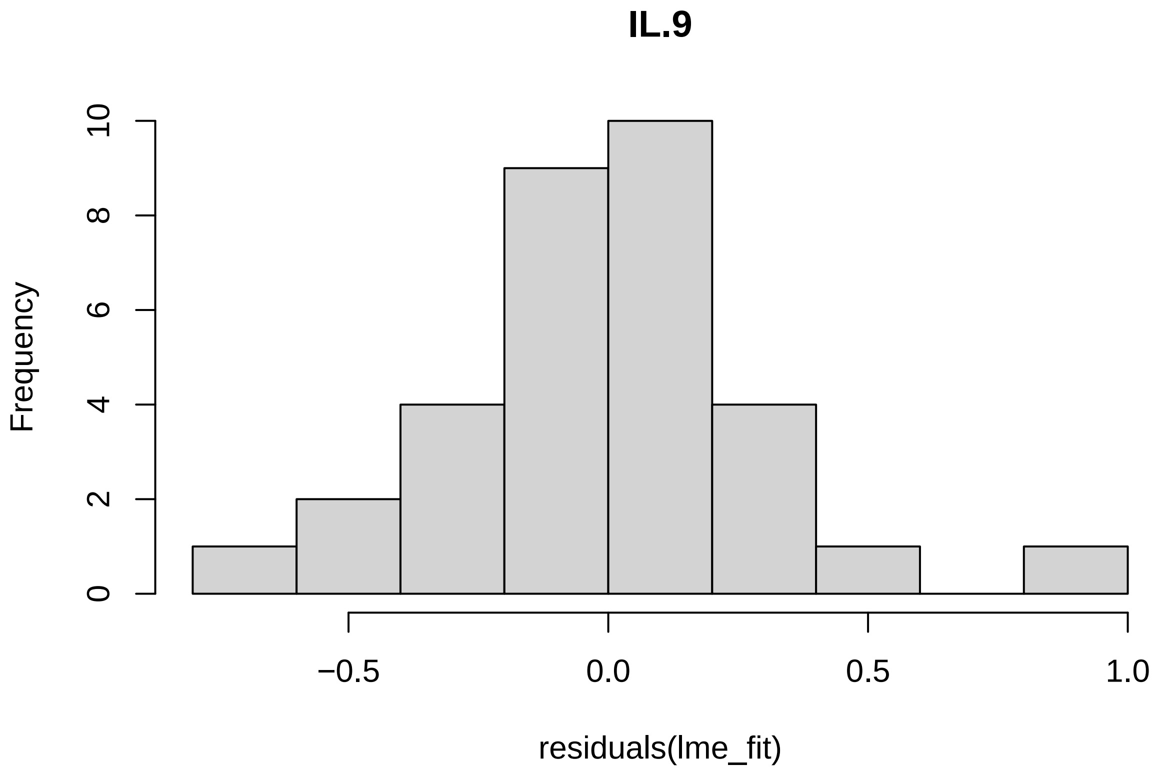


```
## Warning in if (!is.na(a)) {: the condition has length > 1 and only the first
## element will be used
```

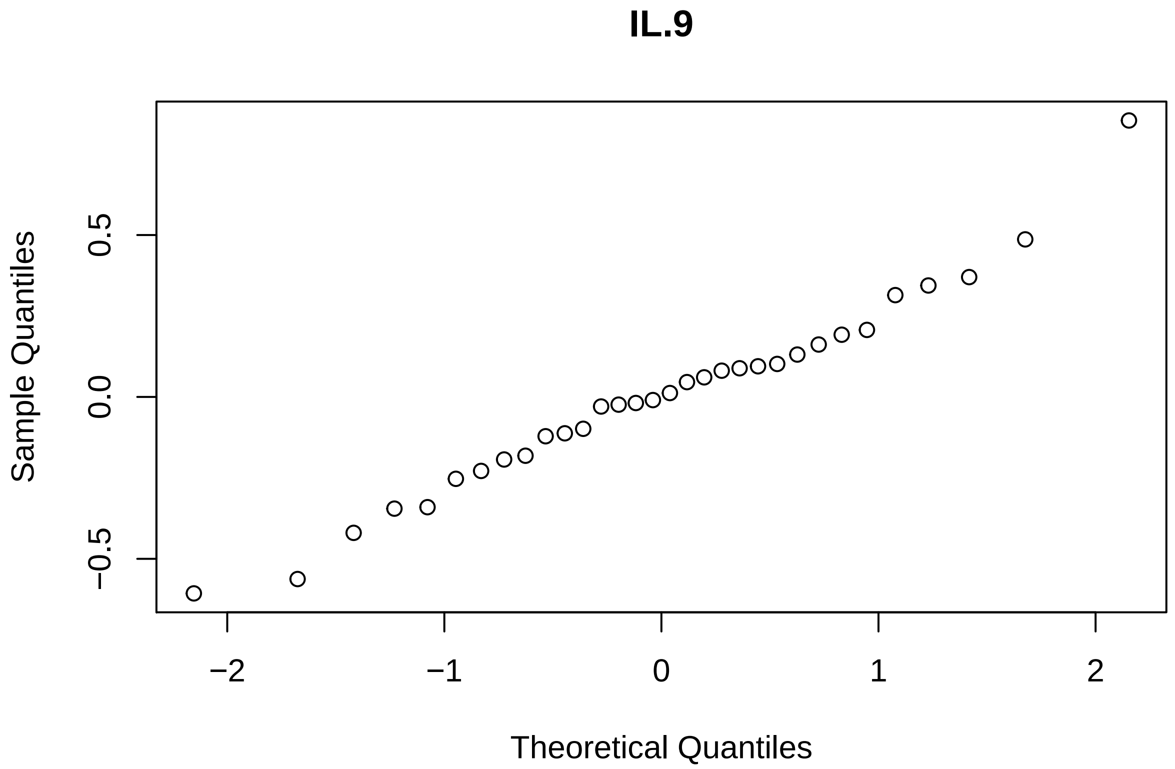

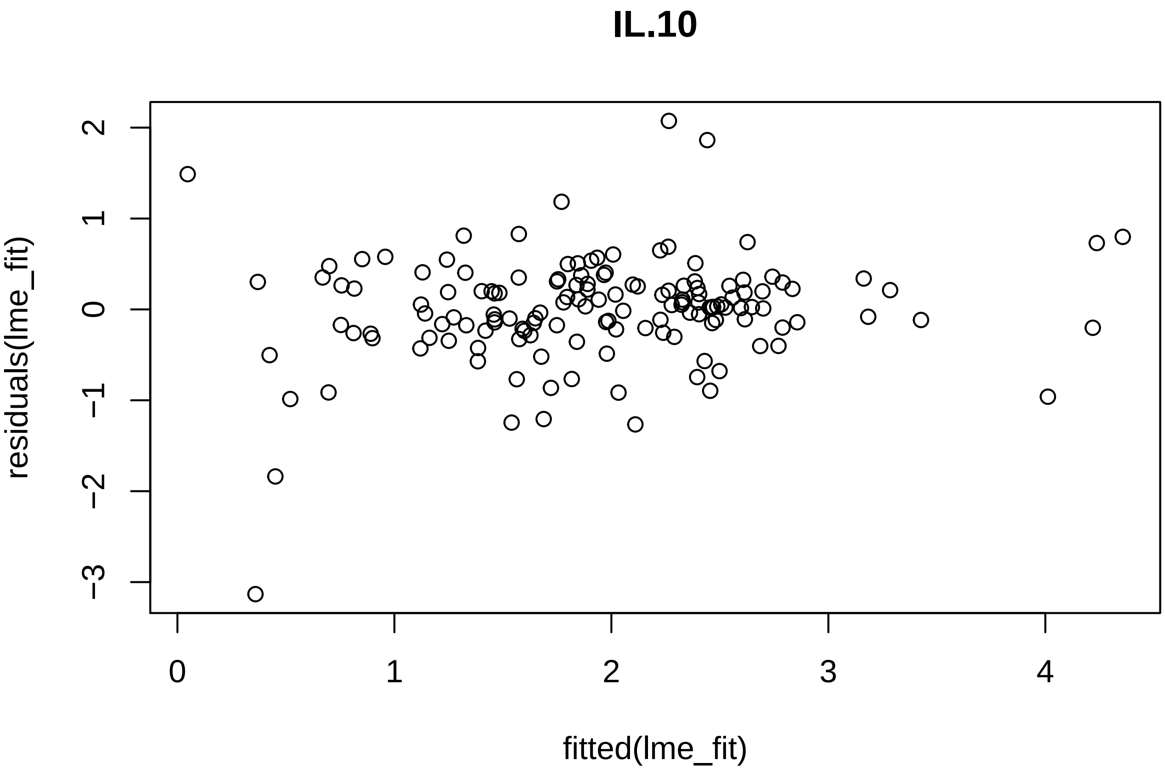

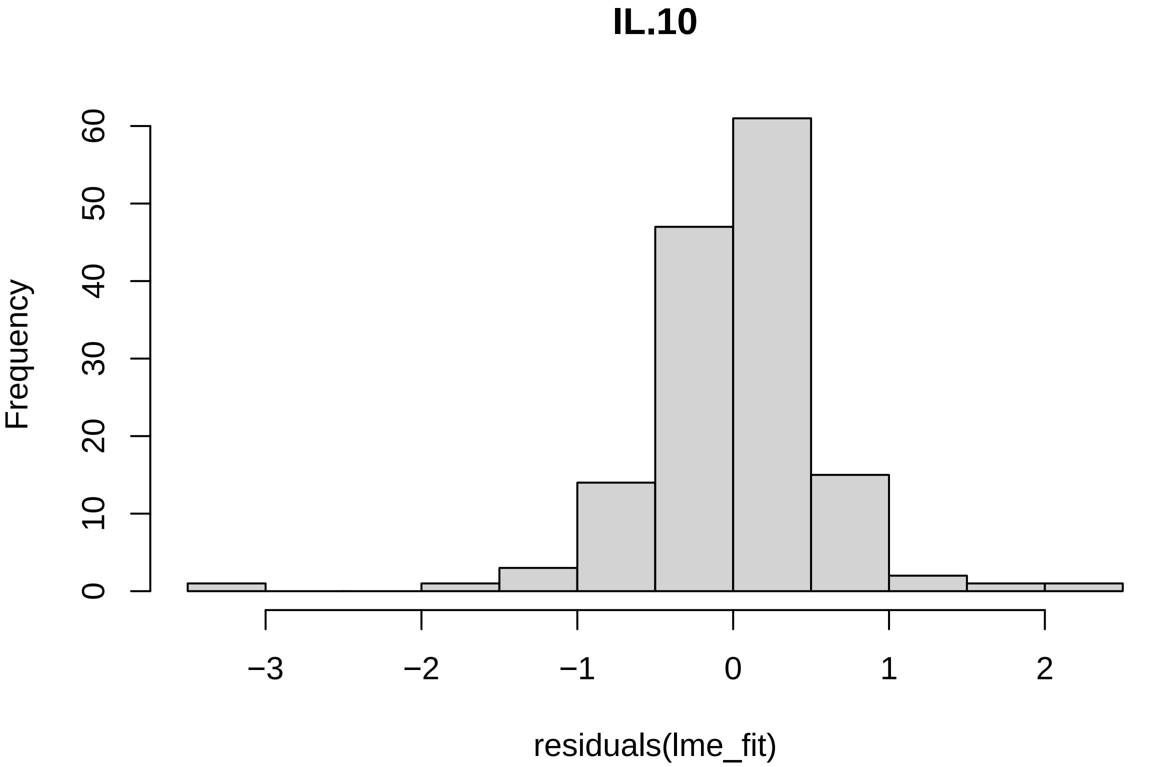


```
## Warning in if (!is.na(a)) {: the condition has length > 1 and only the first
## element will be used
```

IL.10

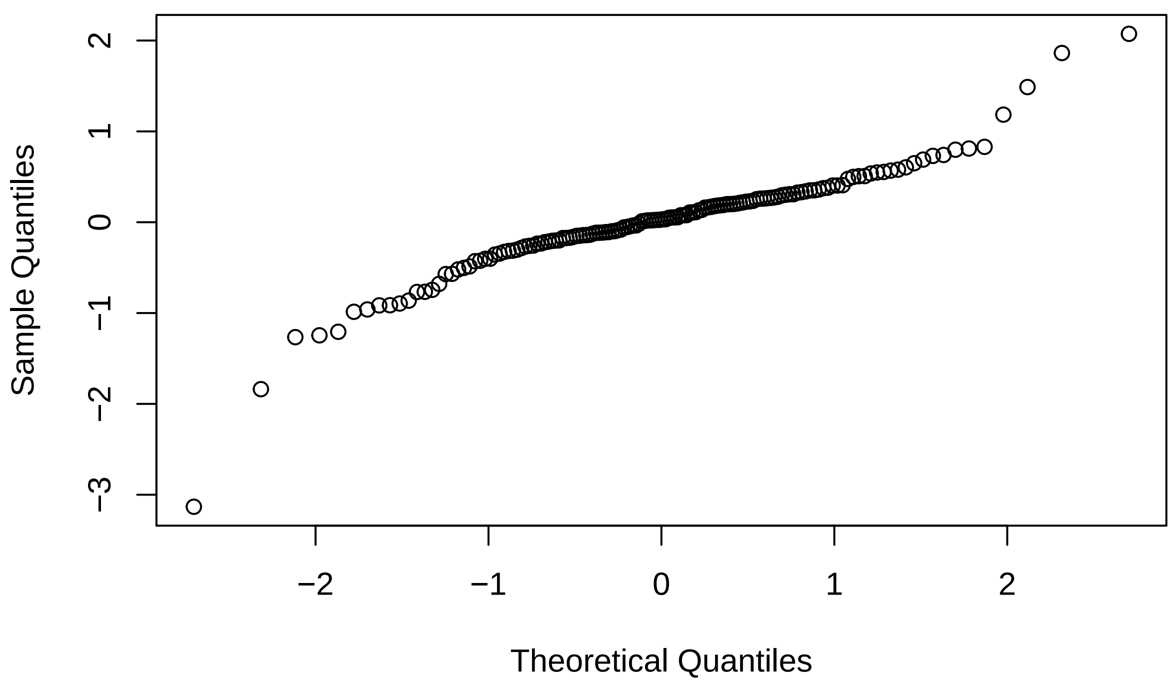

IL.12.p40

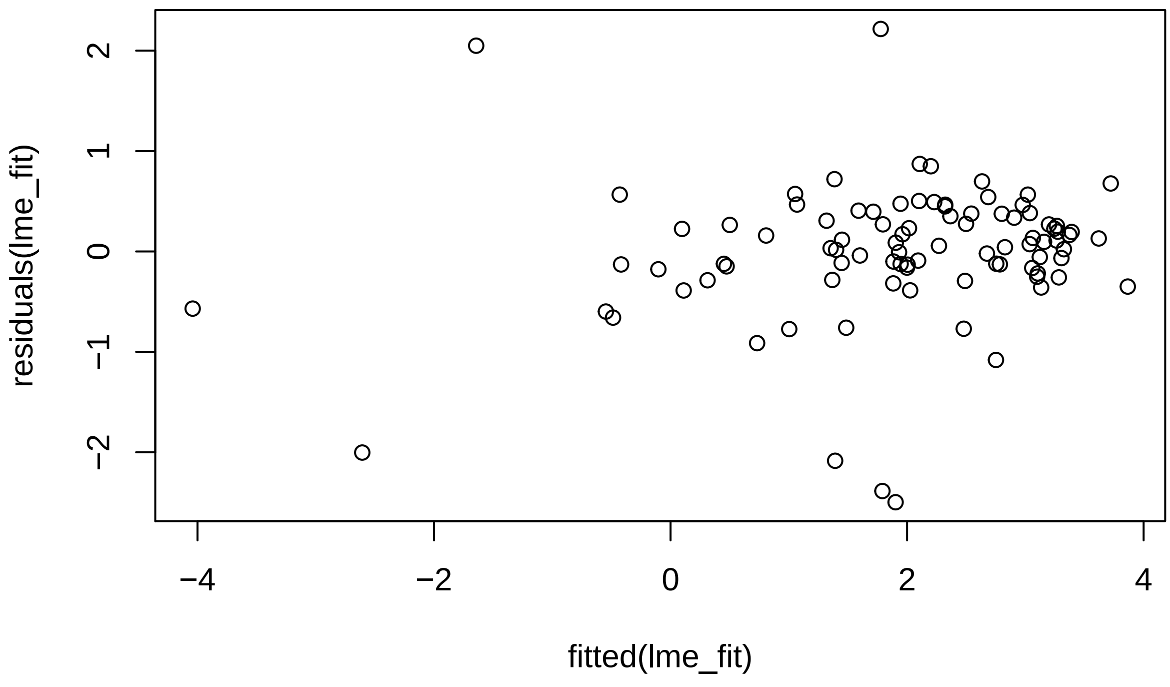

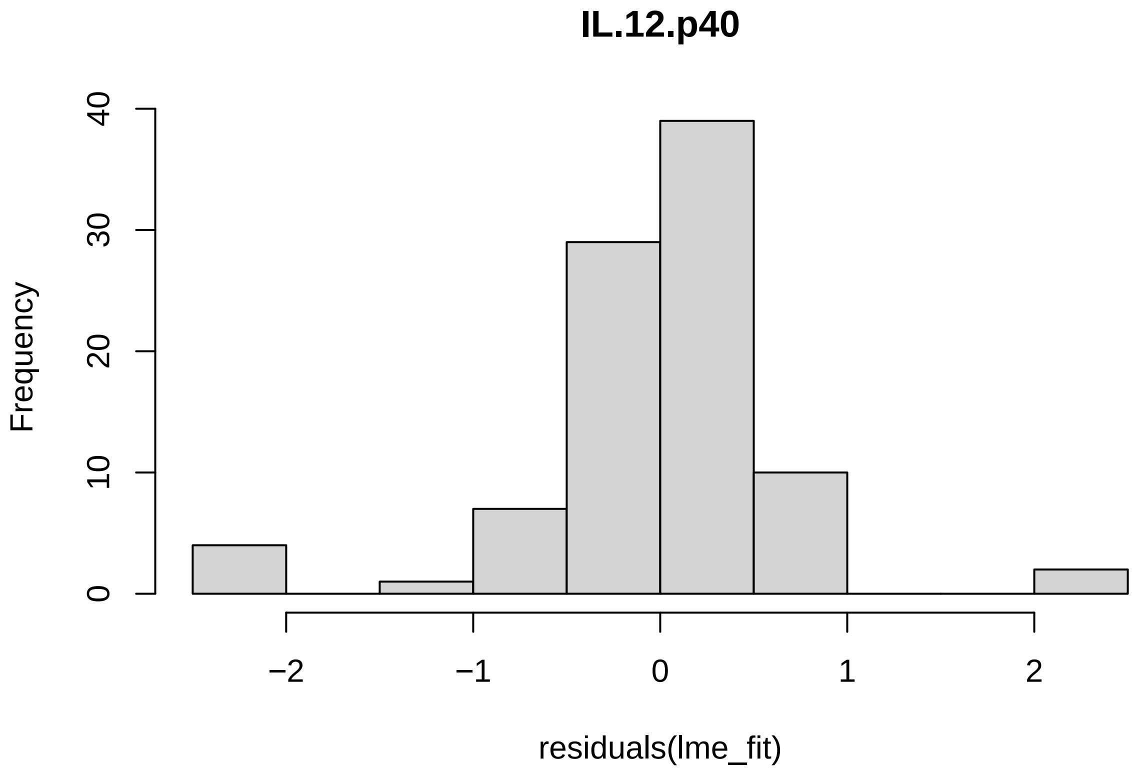


```
## Warning in if (!is.na(a)) {: the condition has length > 1 and only the first
## element will be used
```

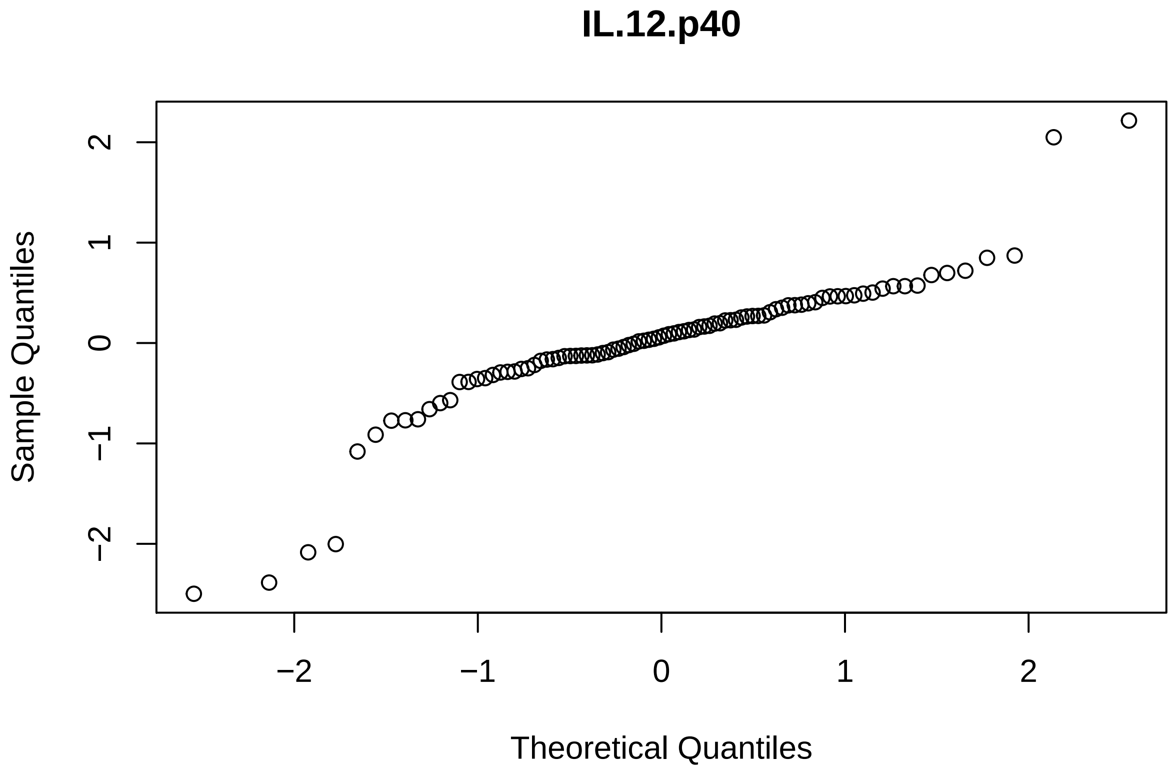

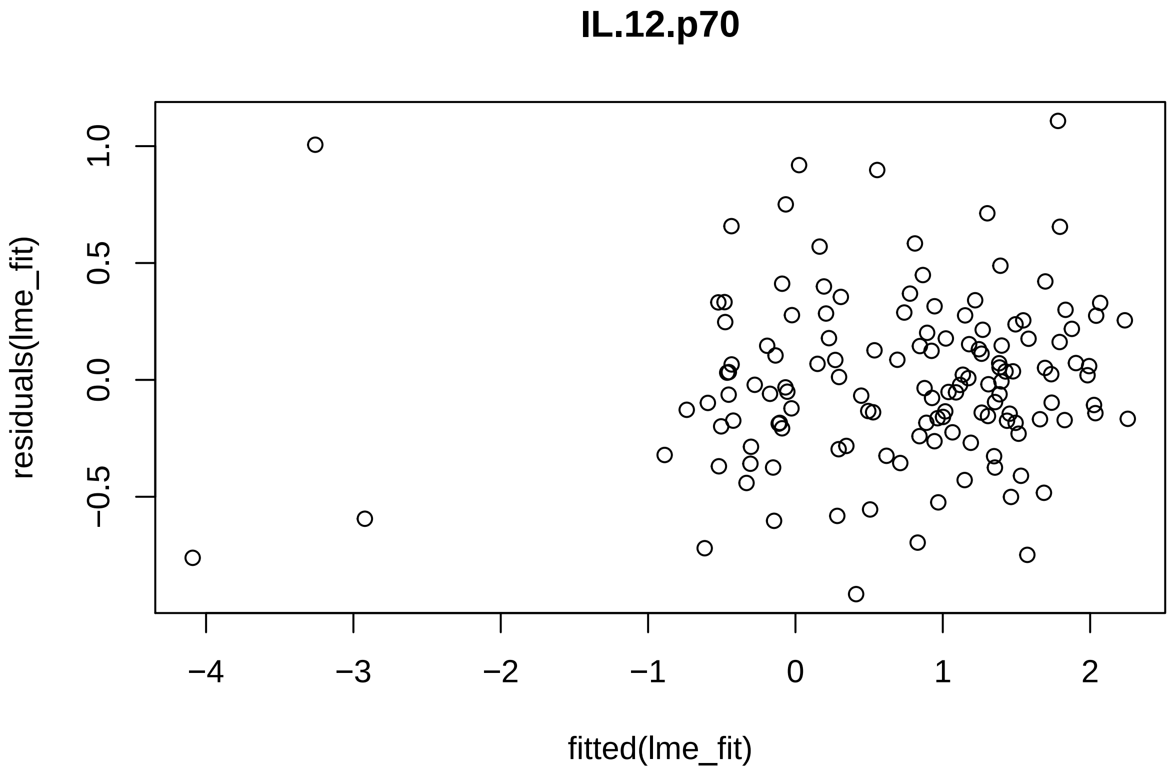
IL.12.p70
residuals(lme_fit)
fitted(lme_fit)
−4
−3
−2
−1
0
1
2
−0.5
0.0
0.5
1.0

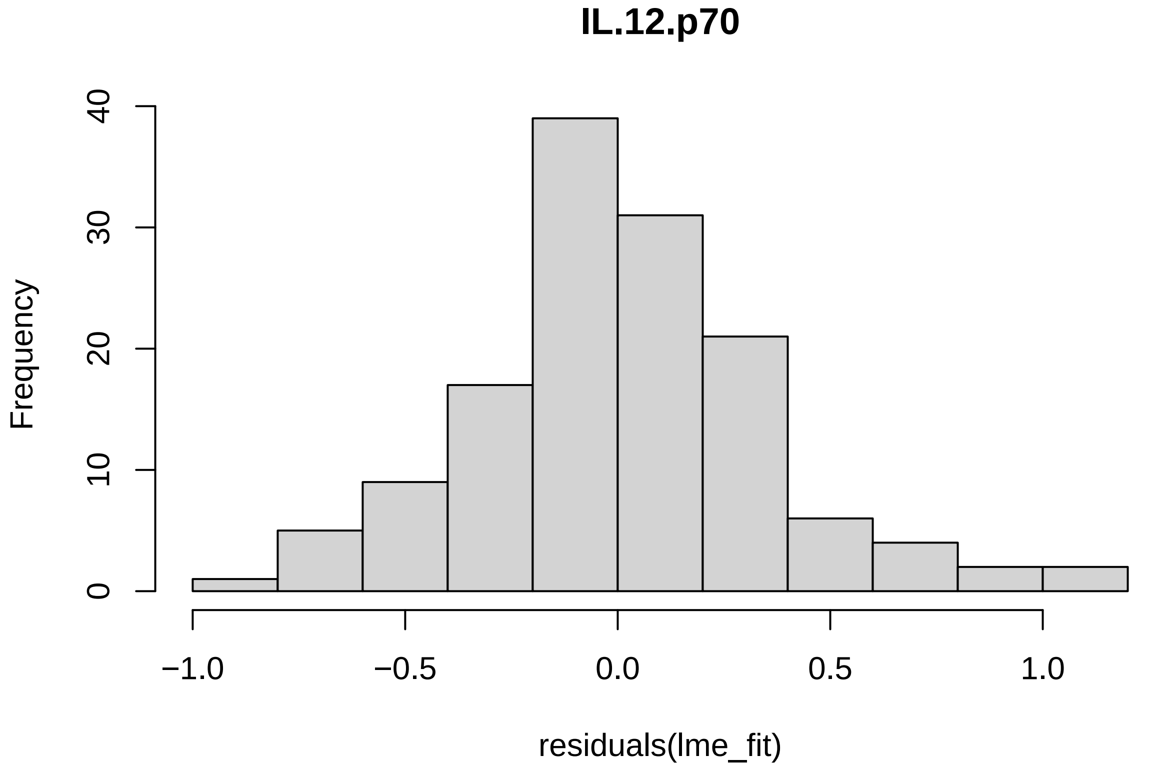
IL.12.p70
Frequency
residuals(lme_fit)
−1.0
−0.5
0.0
0.5
1.0
0
10
20
30
40

IL.12.p70

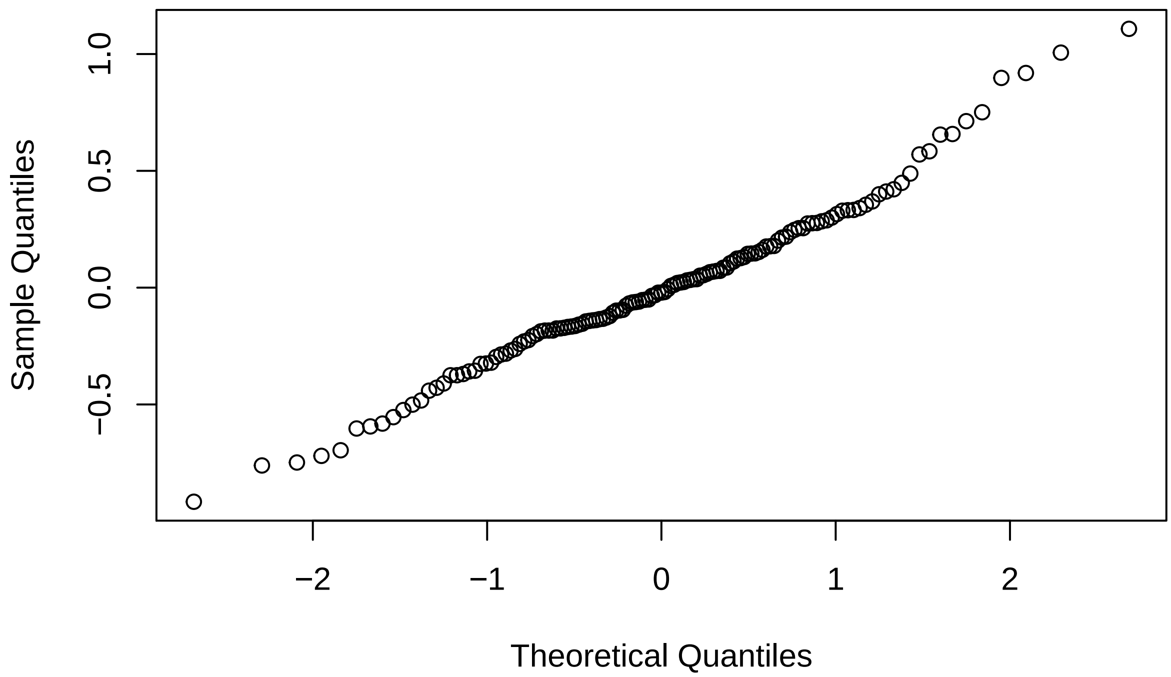

IL.13

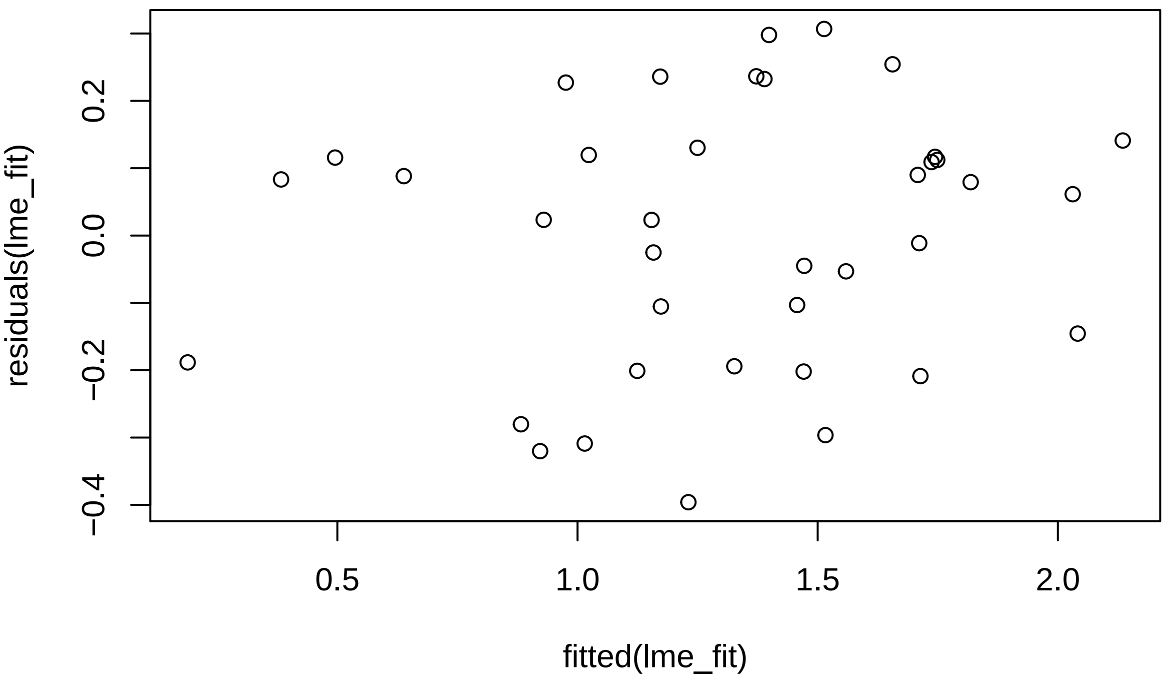

IL.13

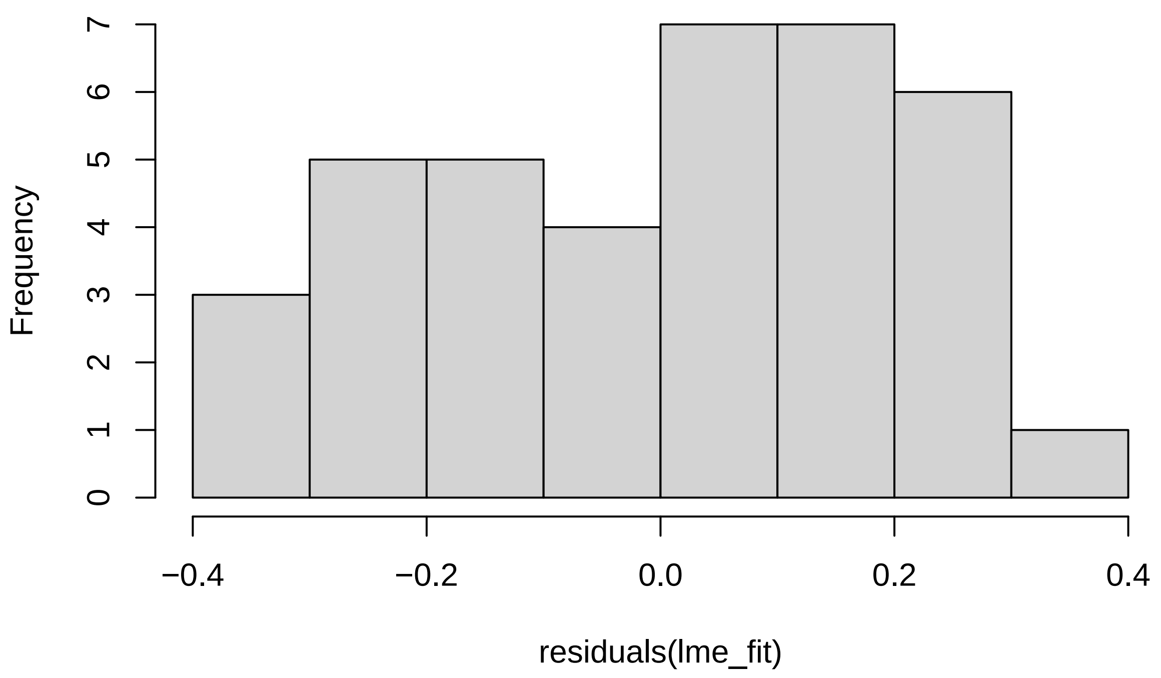


```
## Warning in if (!is.na(a)) {: the condition has length > 1 and only the first
## element will be used
```

IL.13

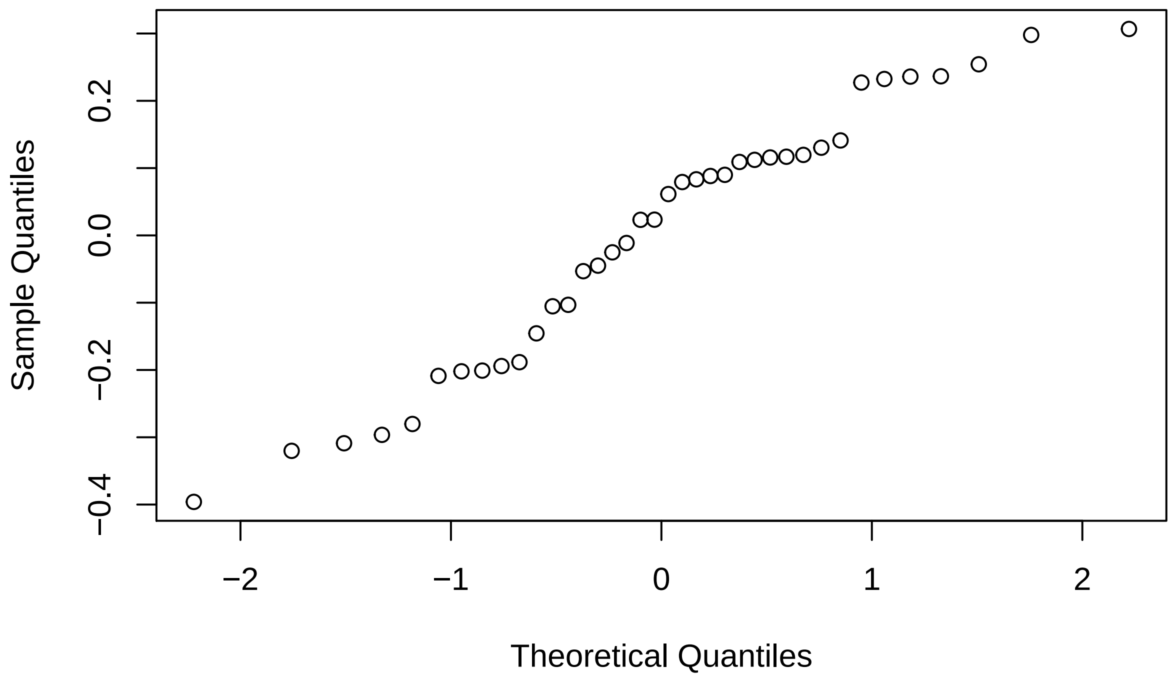

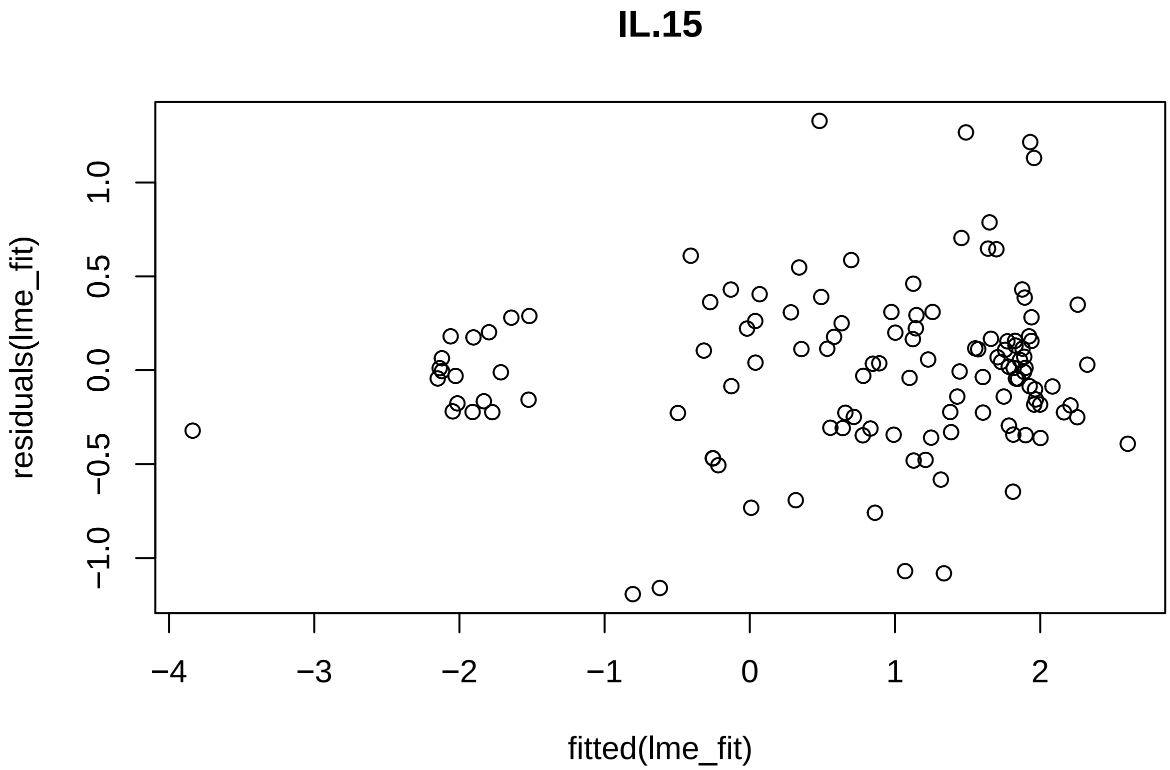

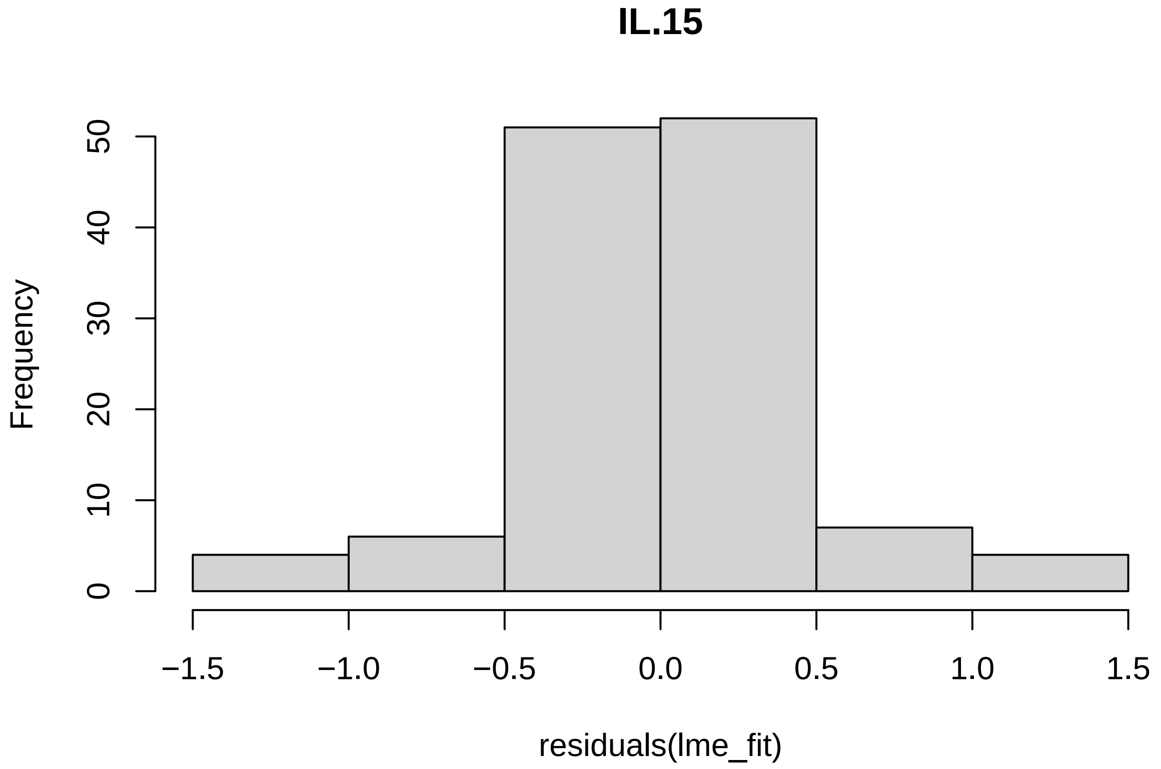


```
## Warning in if (!is.na(a)) {: the condition has length > 1 and only the first
## element will be used
```

IL.15

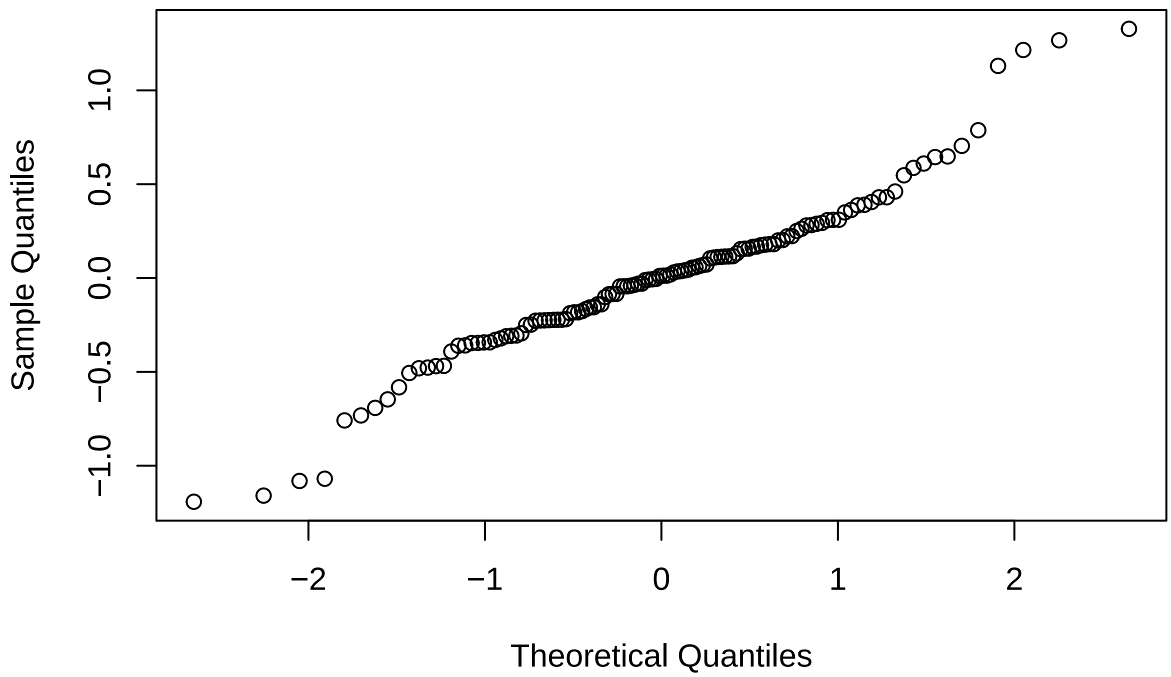

Sample Quantiles
1.0
0.5
0.0
−0.5
−1.0
−2
−1
0
1
2
Theoretical Quantiles

IL.17

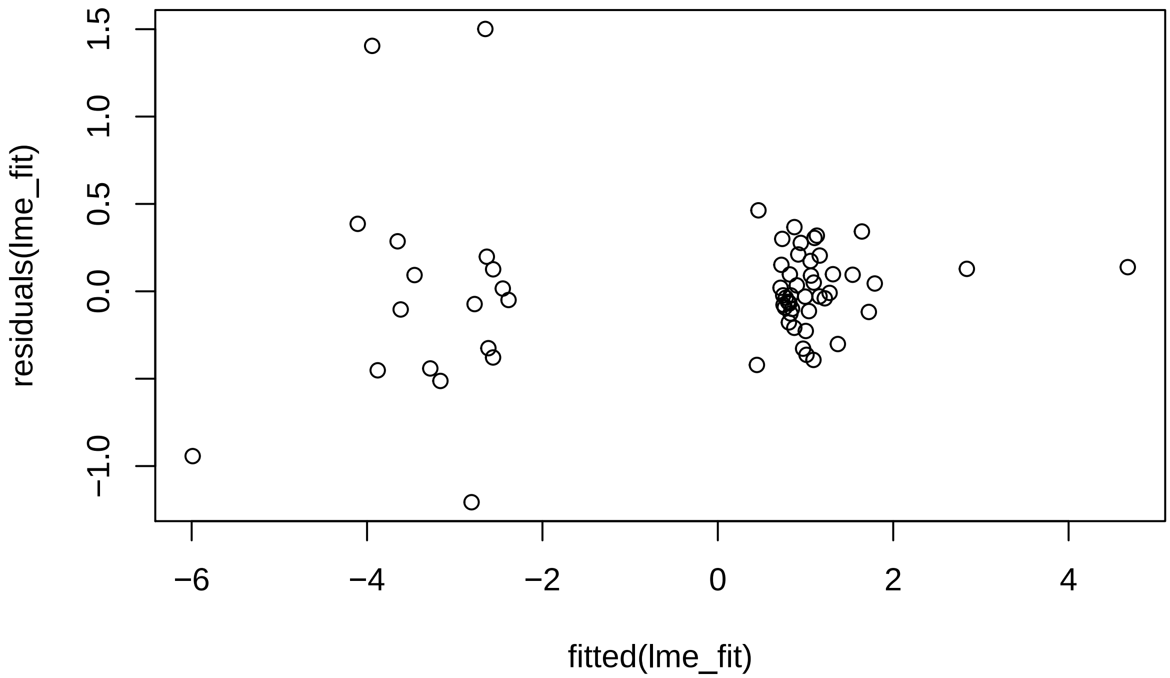

residuals(lme_fit)
1.5
1.0
0.5
0.0
−1.0
−6
−4
−2
0
2
4
fitted(lme_fit)

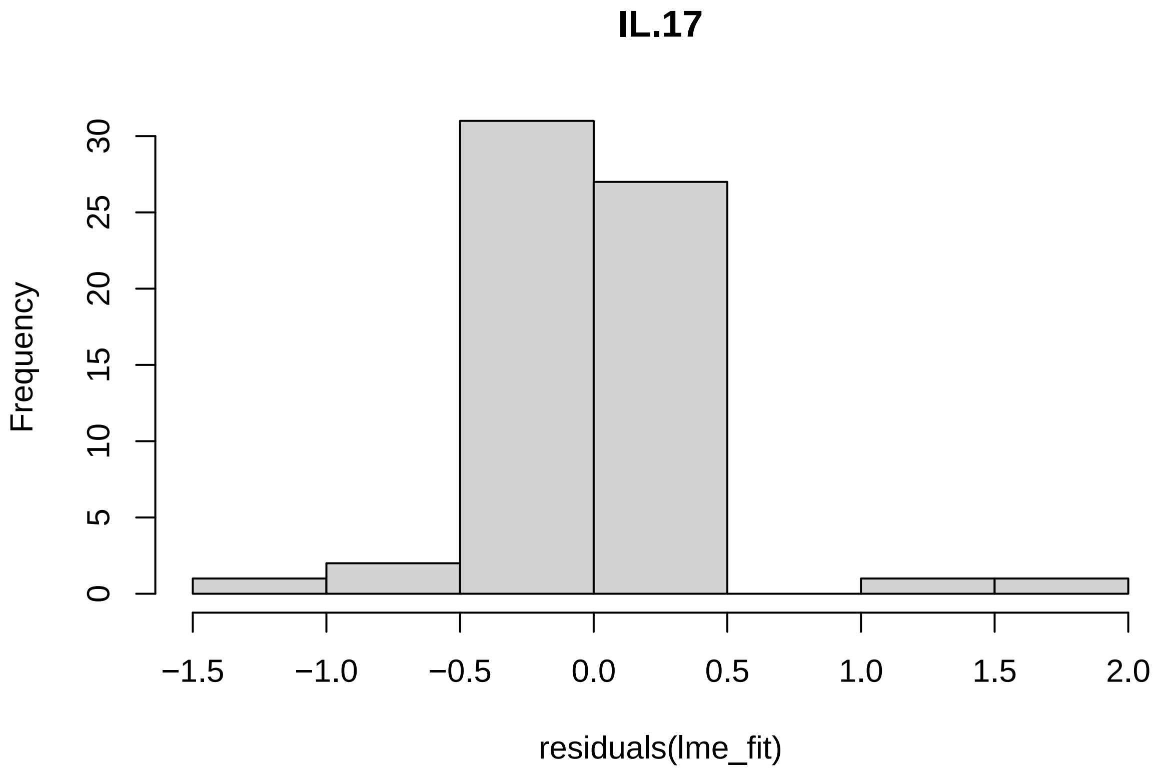


```
## Warning in if (!is.na(a)) {: the condition has length > 1 and only the first
## element will be used
```

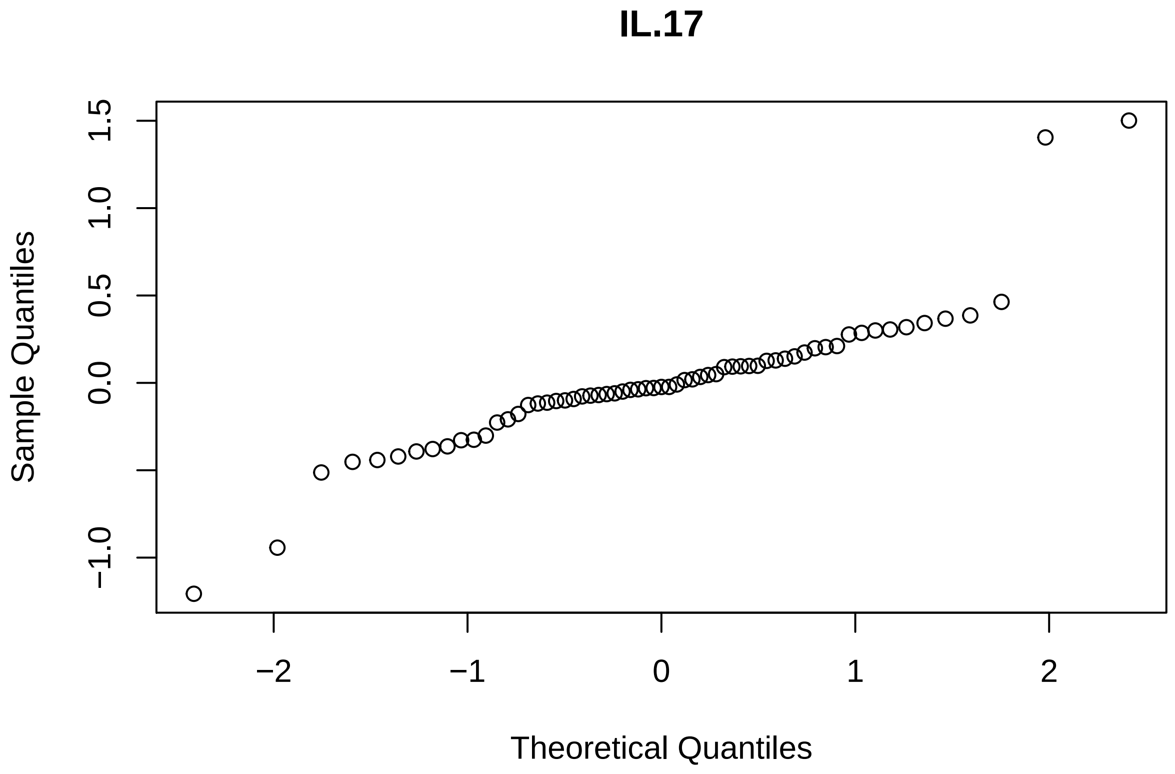

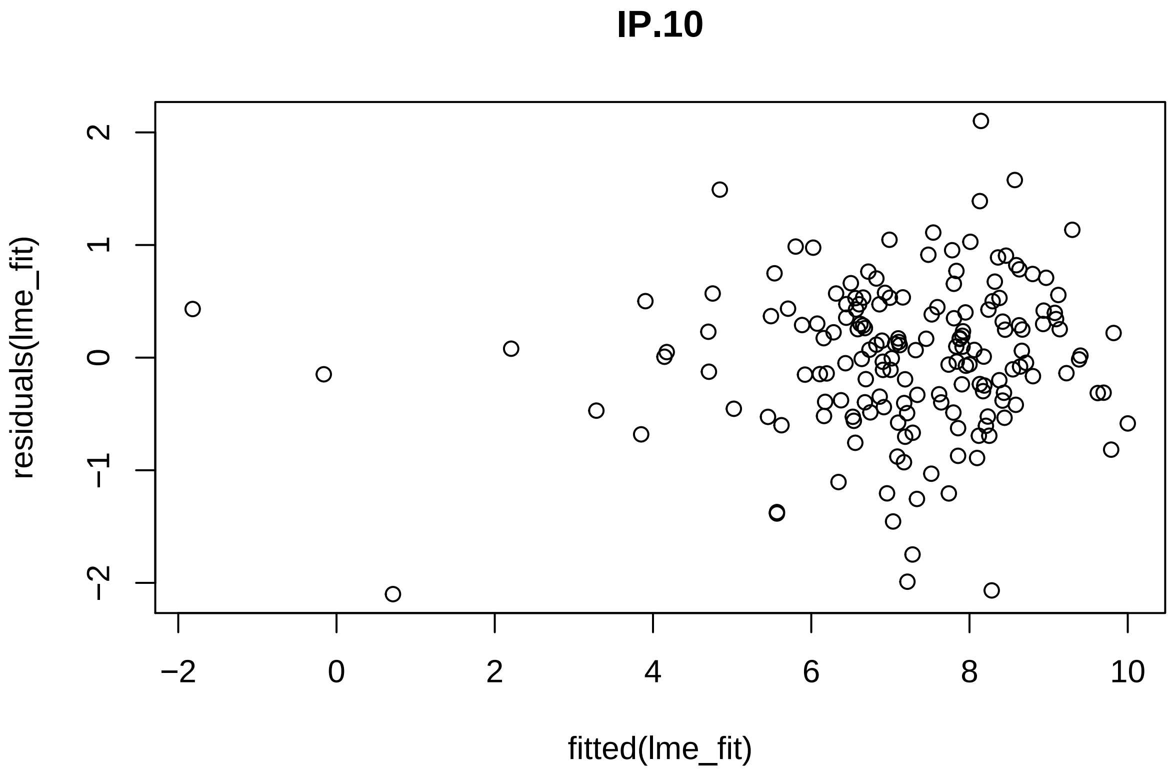

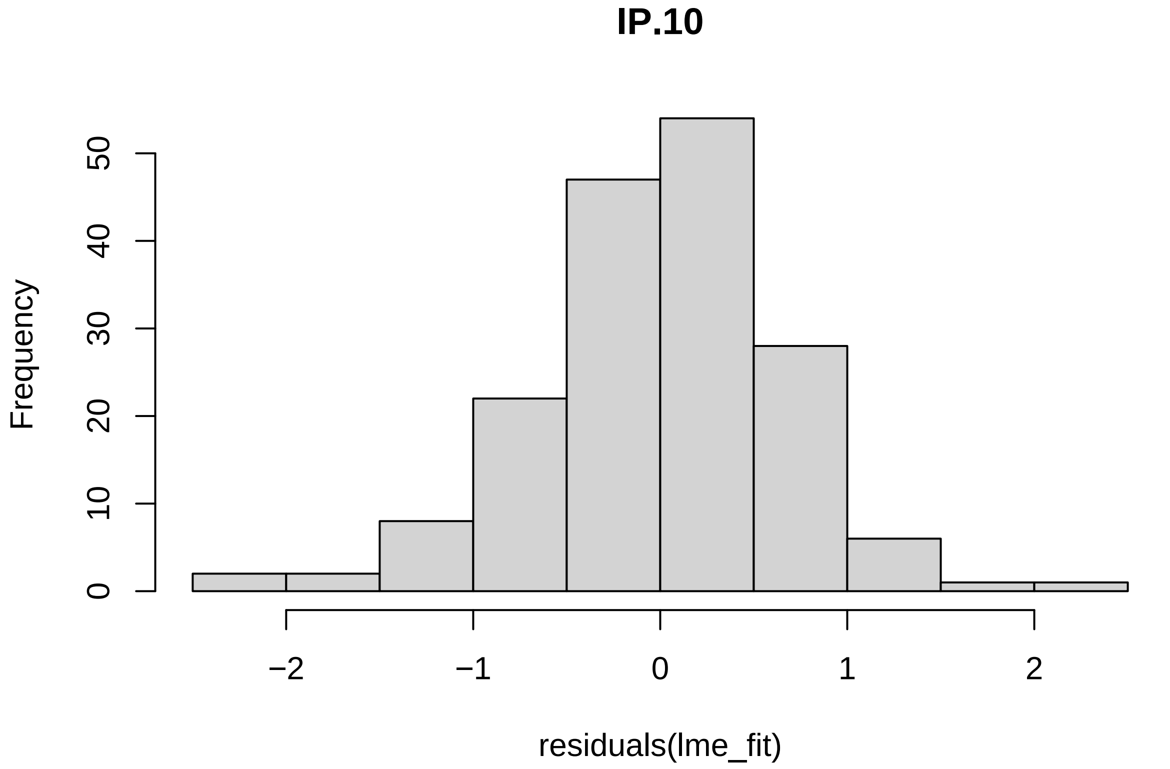


```
## Warning in if (!is.na(a)) {: the condition has length > 1 and only the first
## element will be used
```

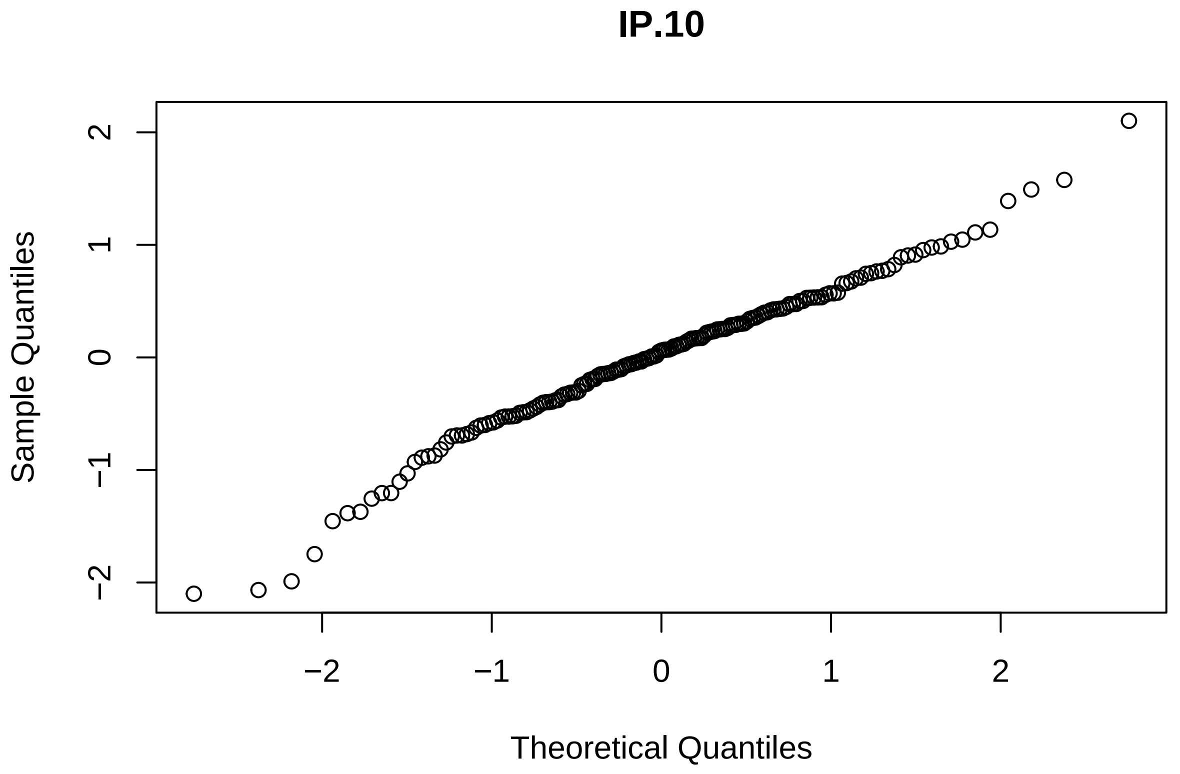
IP.10
Sample Quantiles
Theoretical Quantiles
−2
−1
0
1
2

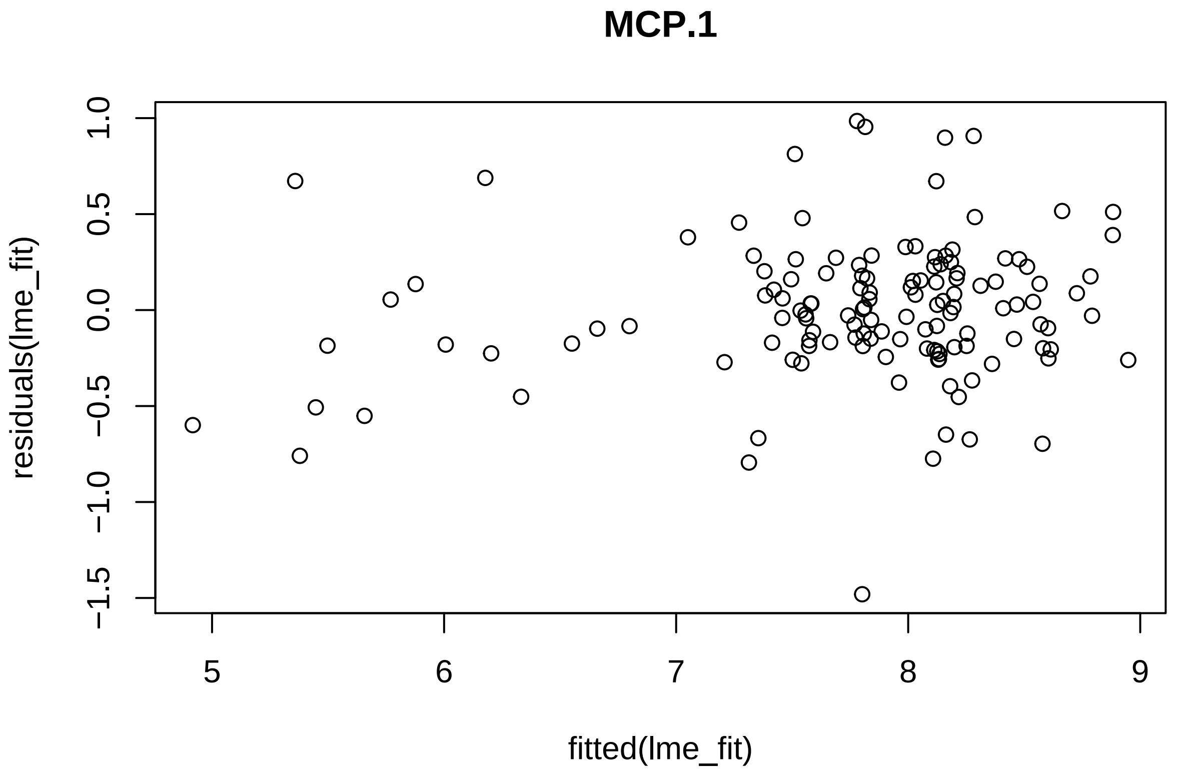
MCP.1
residuals(lme_fit)
fitted(lme_fit)
−1.5
−1.0
−0.5
0.0
0.5
1.0
5
6
7
8
9

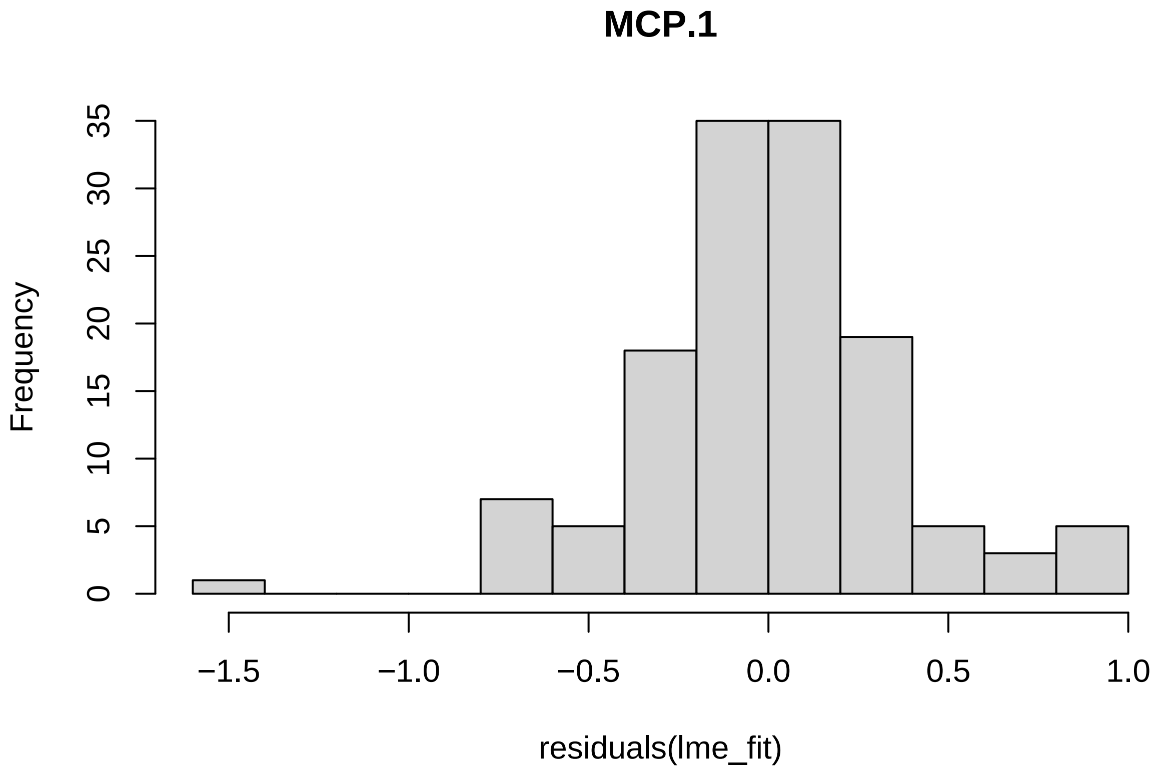


```
## Warning in if (!is.na(a)) {: the condition has length > 1 and only the first
## element will be used
```

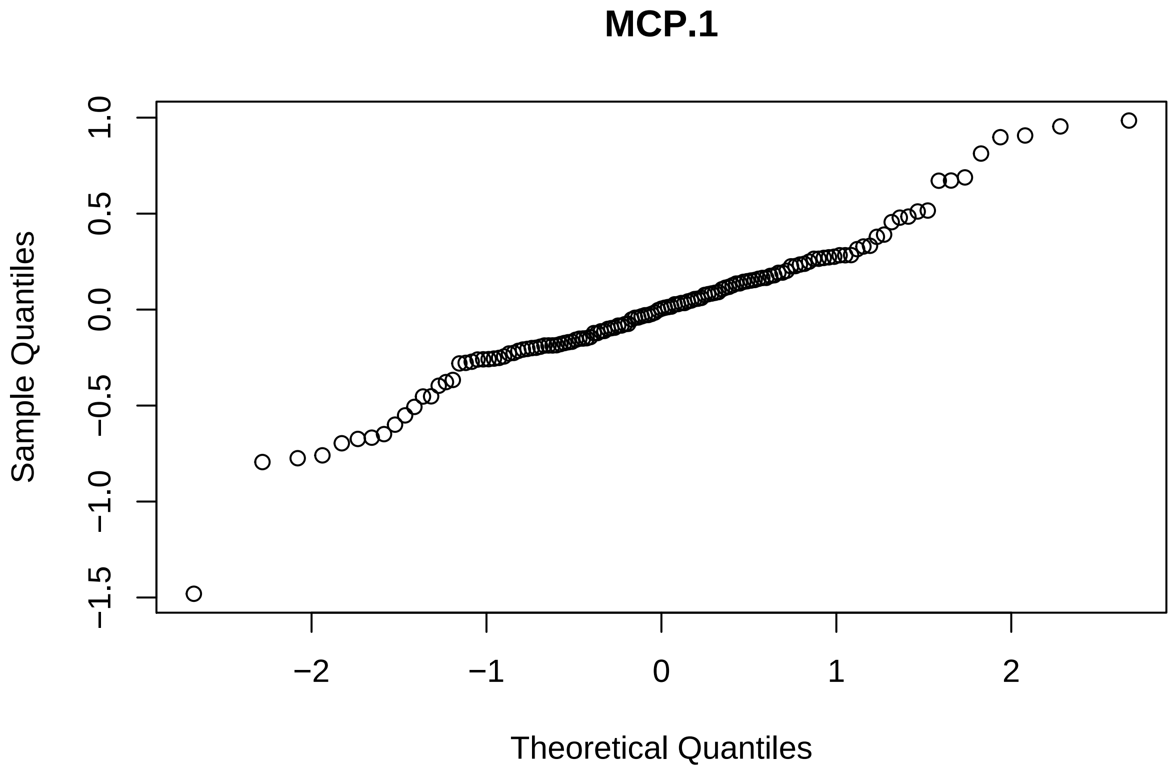

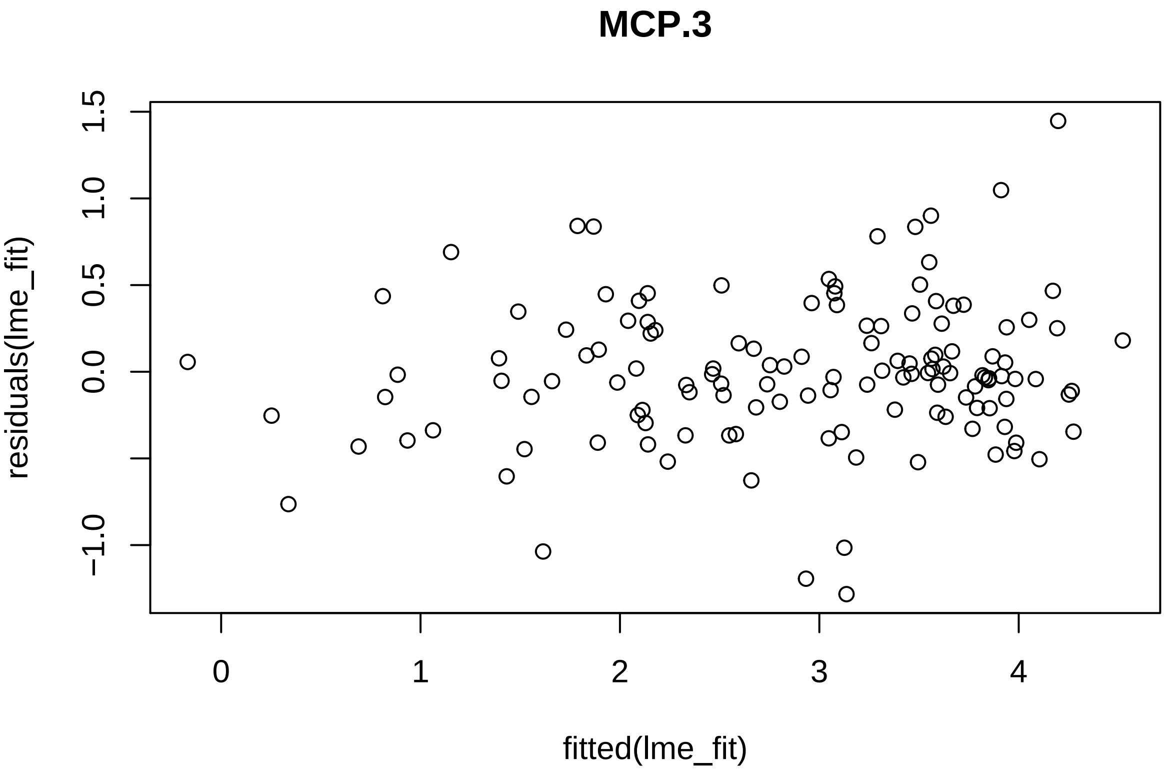

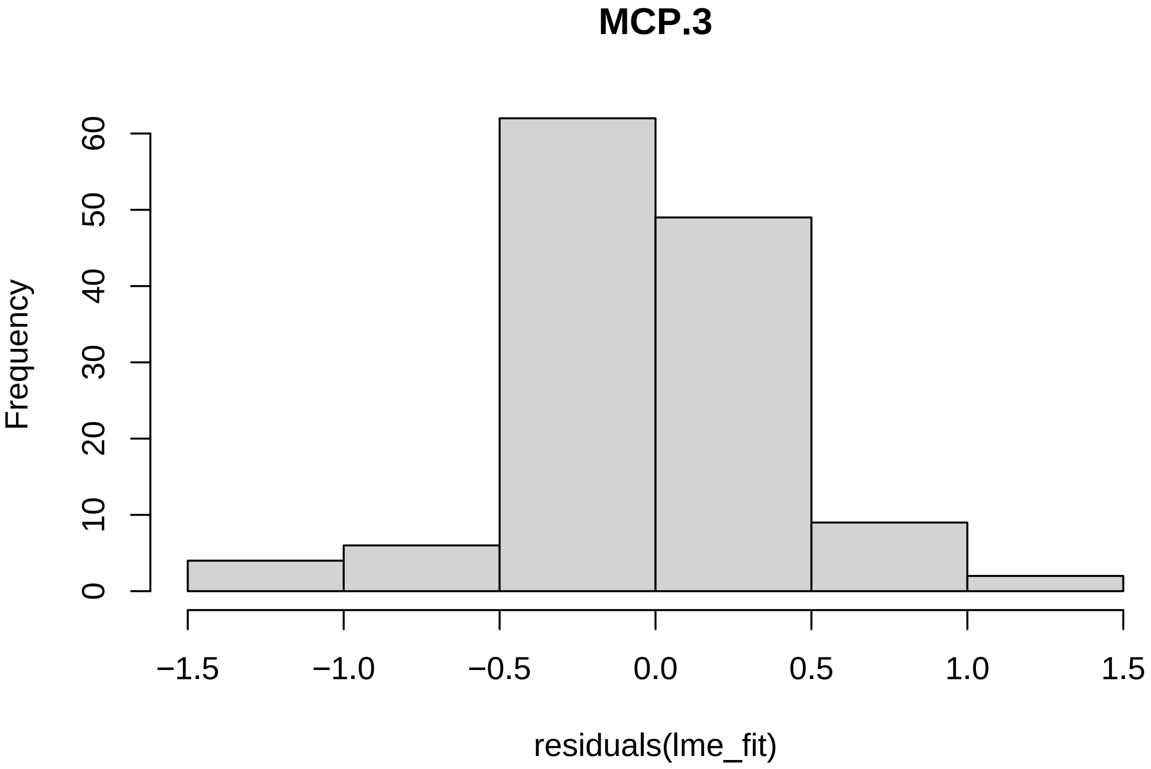


```
## Warning in if (!is.na(a)) {: the condition has length > 1 and only the first
## element will be used
```

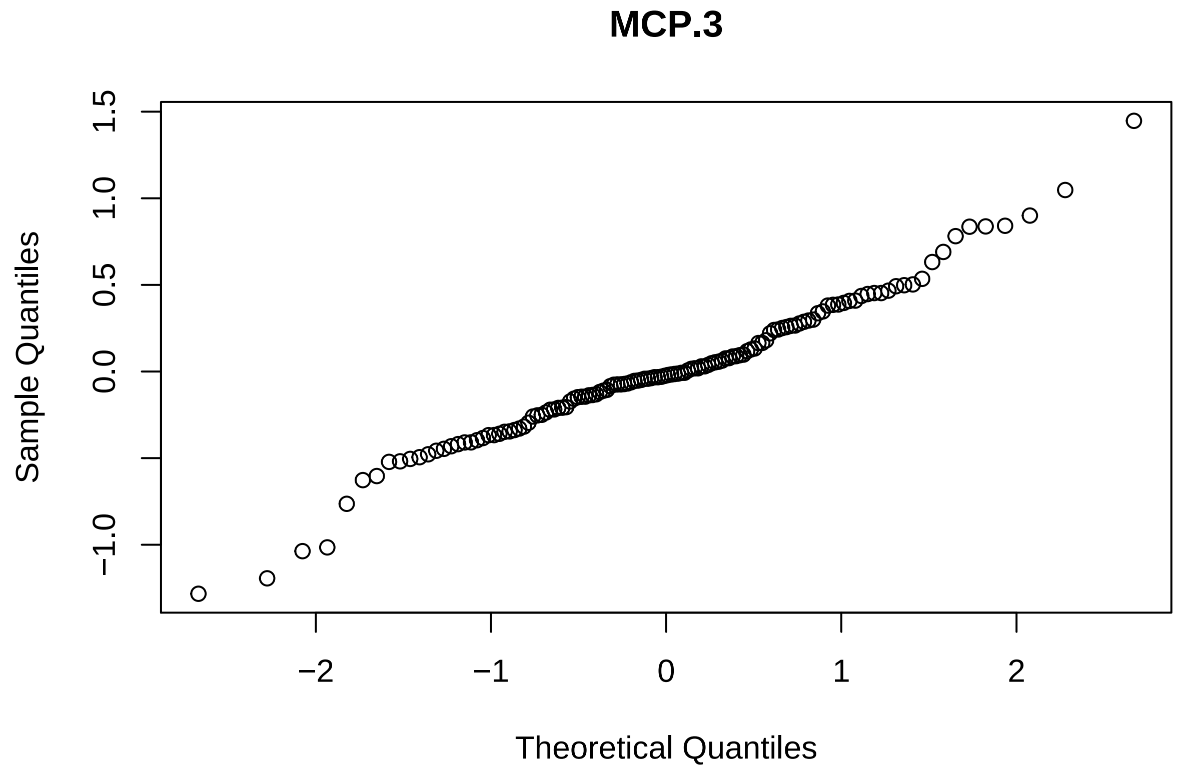
MCP.3
Sample Quantiles
Theoretical Quantiles
1.5
1.0
0.5
0.0
−1.0
−2
−1
0
1
2

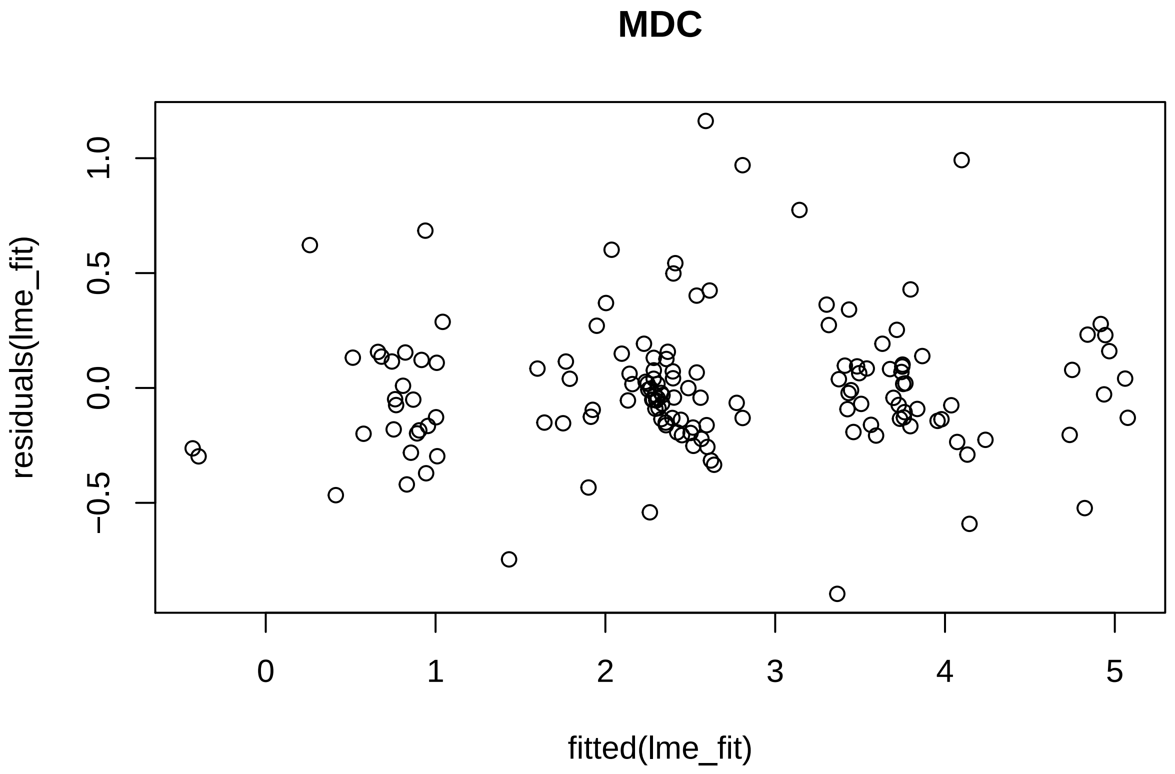
MDC
residuals(lme_fit)
fitted(lme_fit)
1.0
0.5
0.0
−0.5
0
1
2
3
4
5

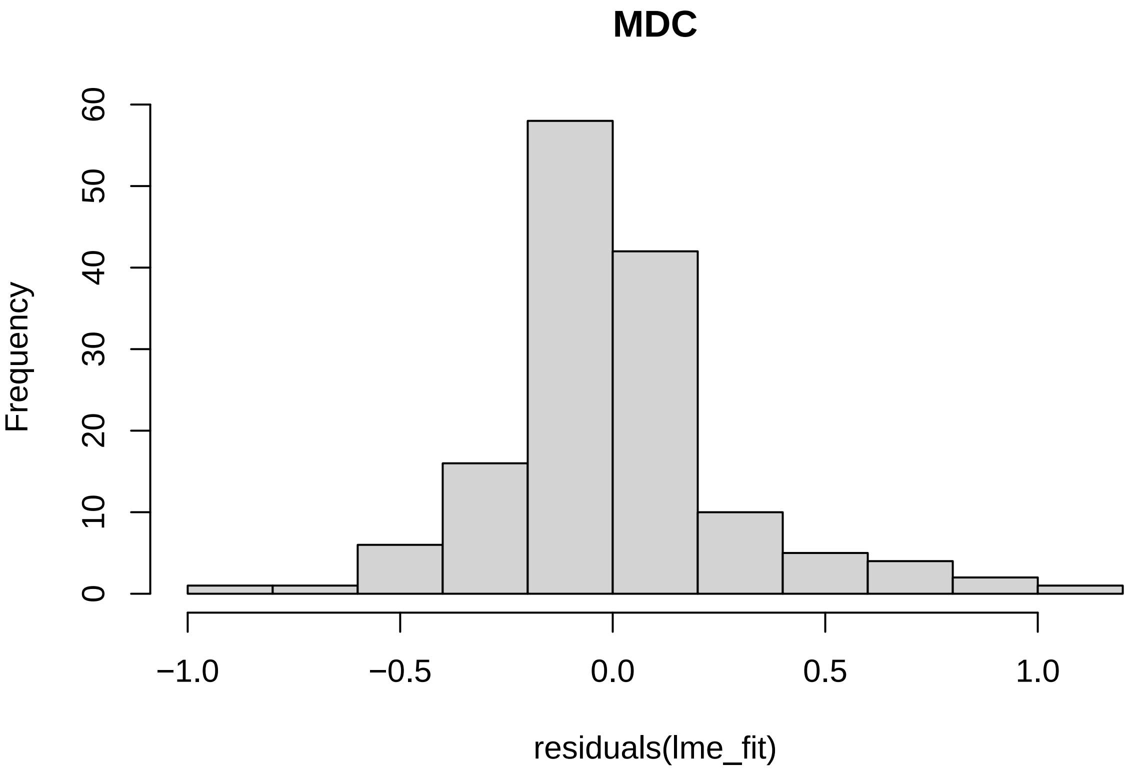


```
## Warning in if (!is.na(a)) {: the condition has length > 1 and only the first
## element will be used
```

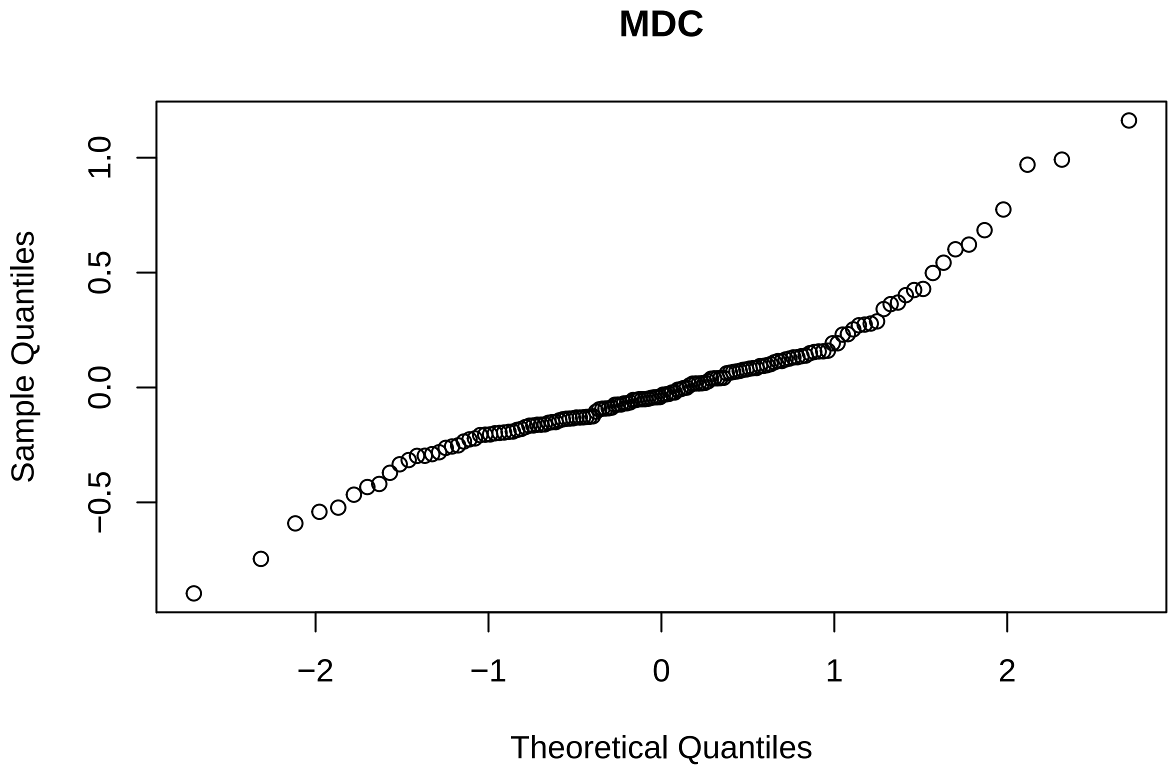

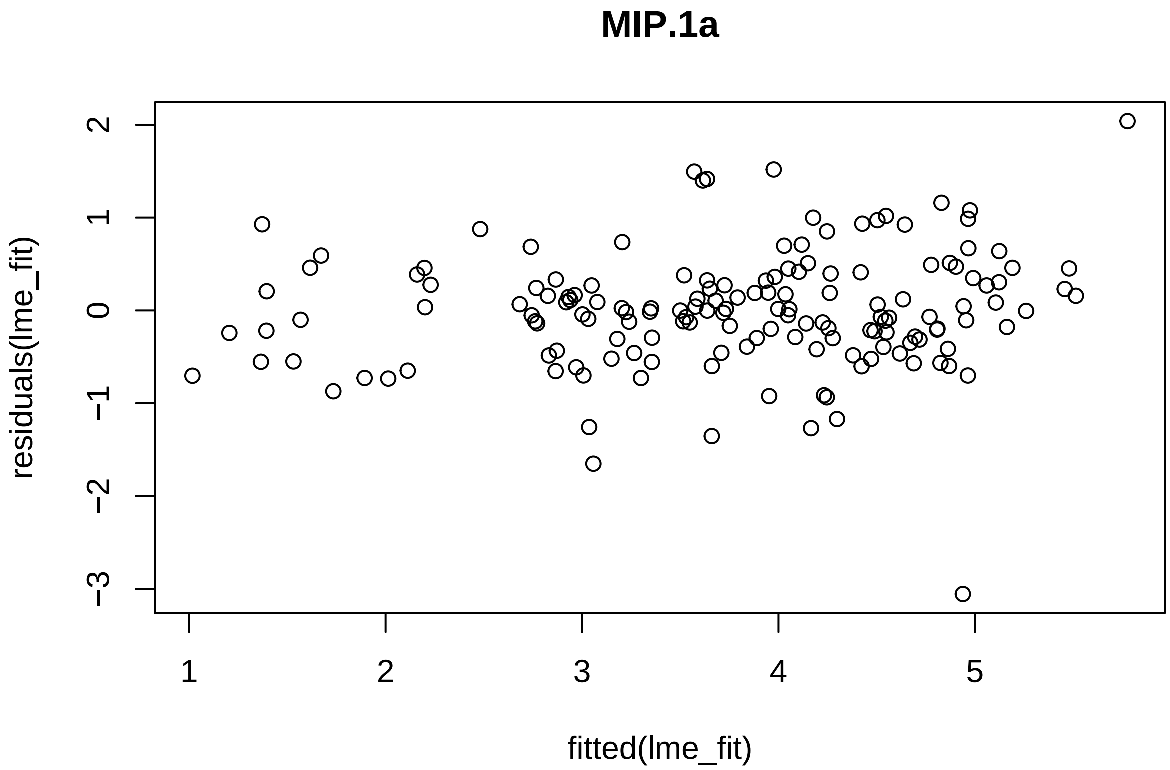

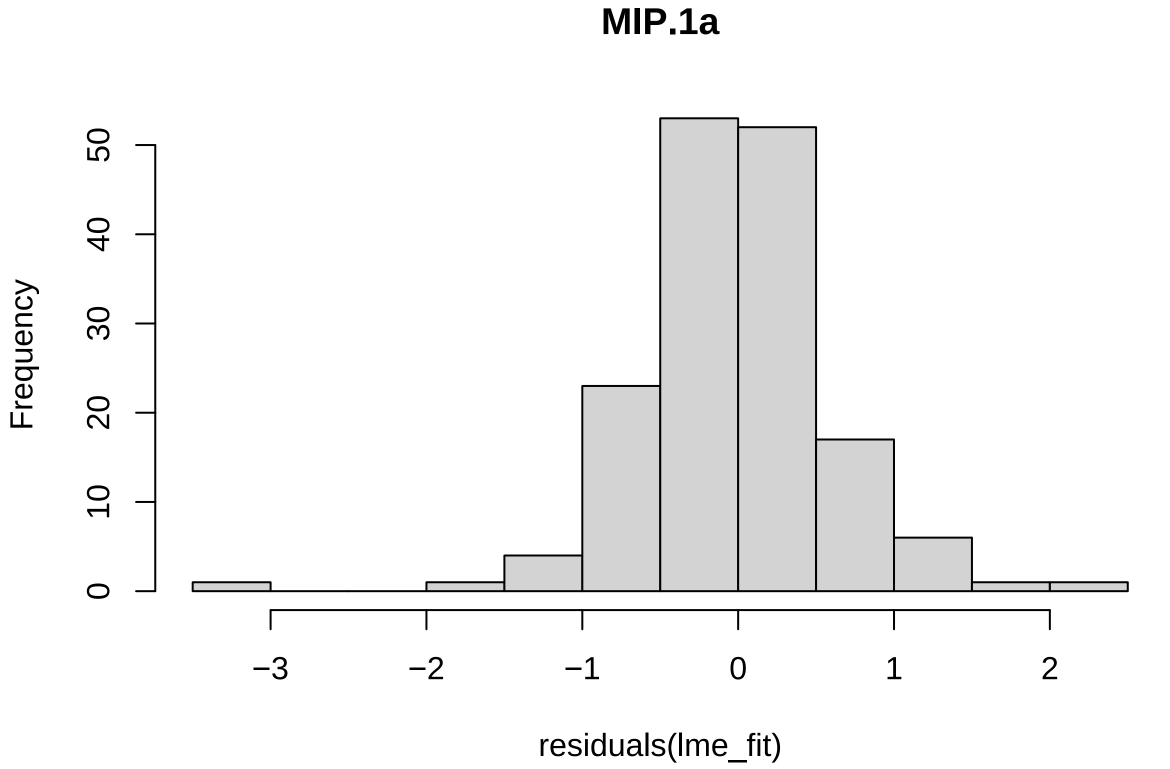


```
## Warning in if (!is.na(a)) {: the condition has length > 1 and only the first
## element will be used
```

MIP.1a

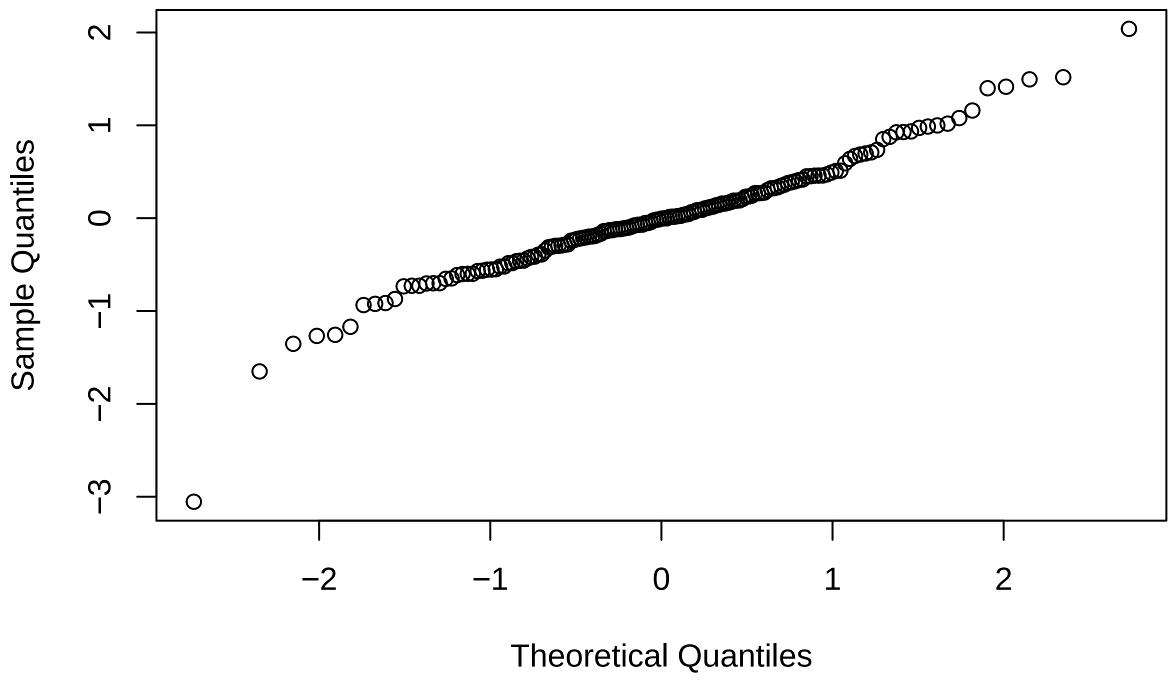
Sample Quantiles
Theoretical Quantiles
2
1
0
−1
−2
−3
−2
−1
0
1
2

MIP.1b

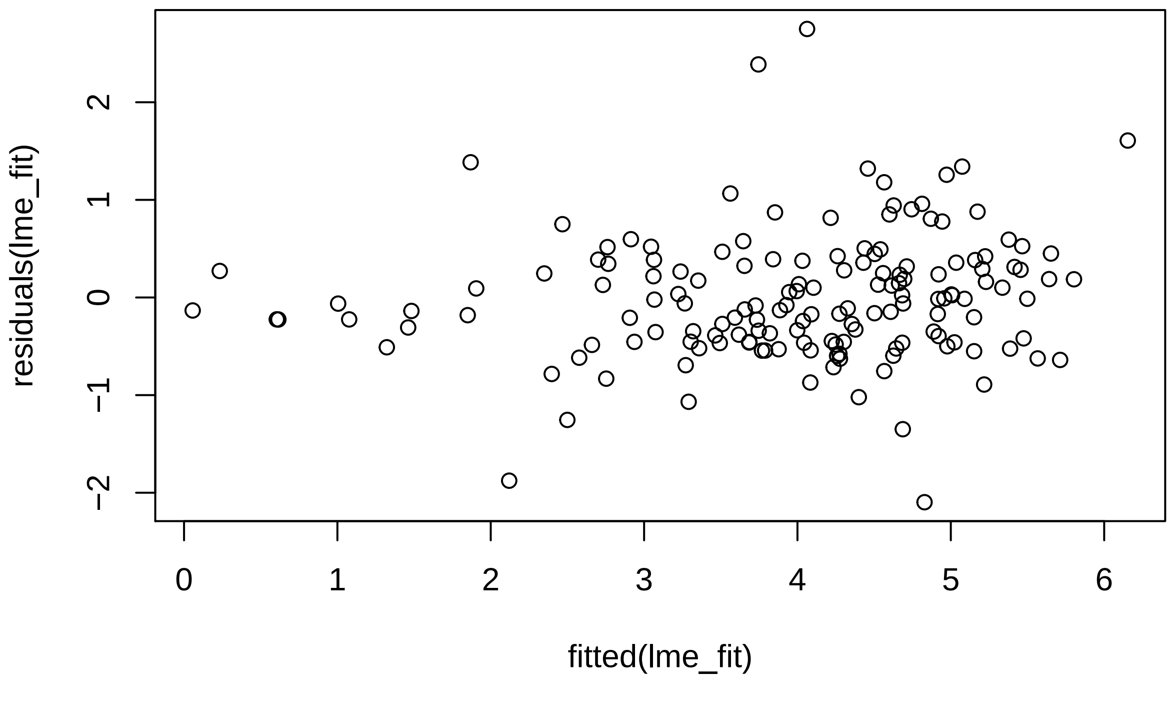
residuals(lme_fit)
fitted(lme_fit)
2
1
0
−1
−2
0
1
2
3
4
5
6

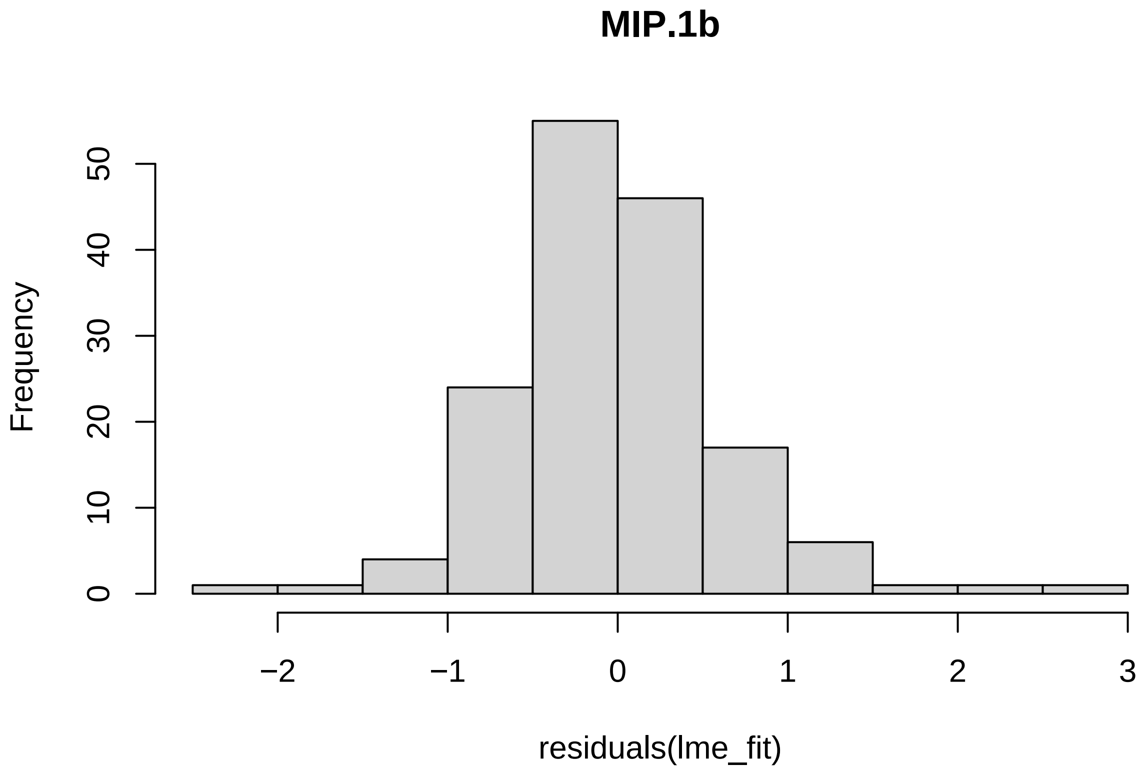


```
## Warning in if (!is.na(a)) {: the condition has length > 1 and only the first
## element will be used
```

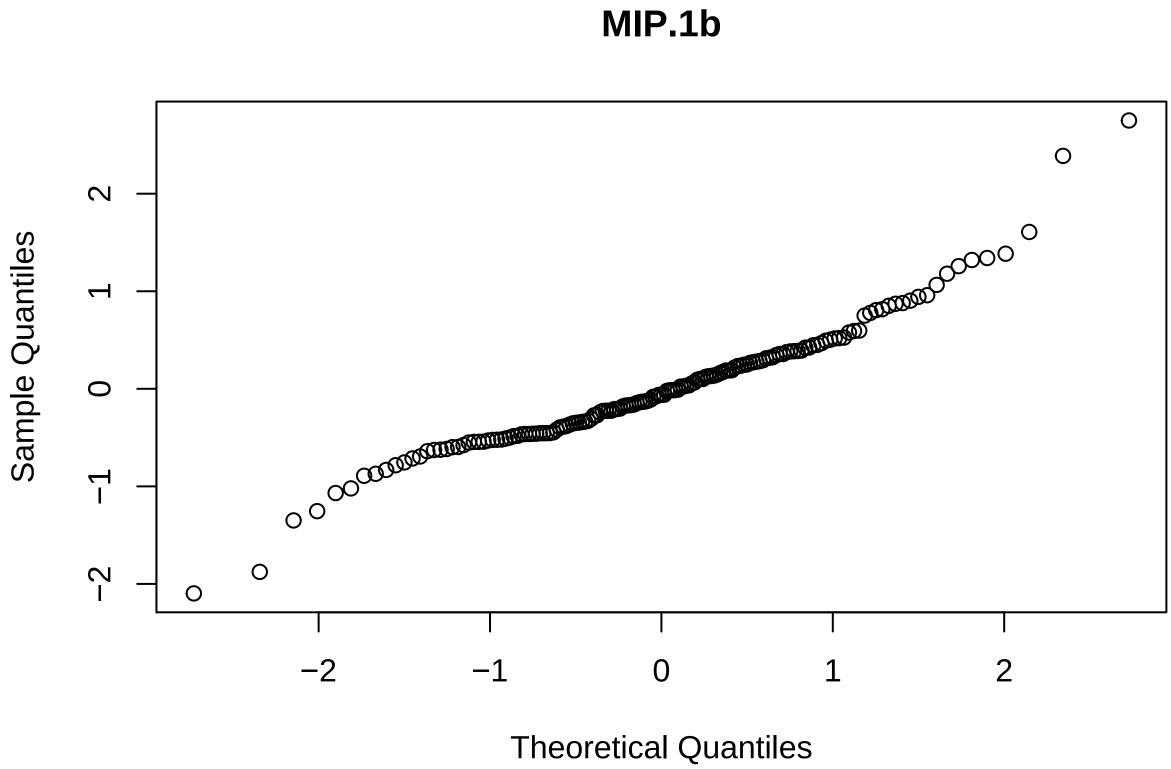

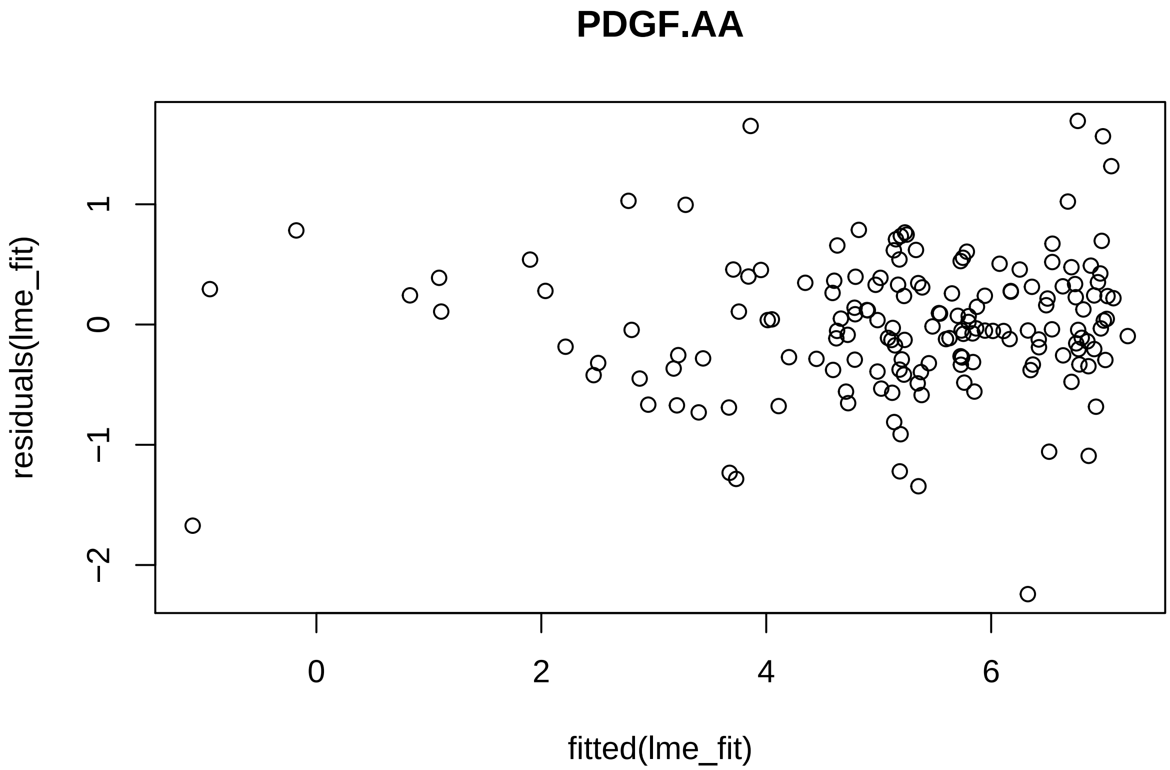

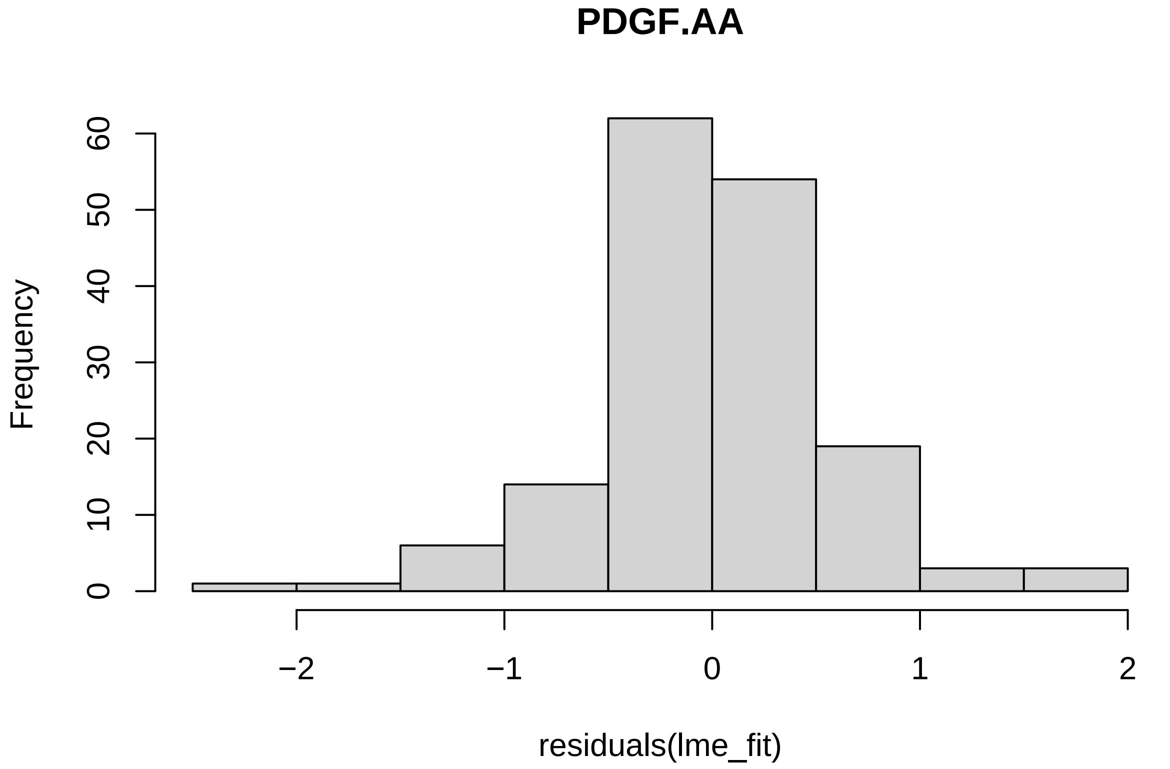


```
## Warning in if (!is.na(a)) {: the condition has length > 1 and only the first
## element will be used
```

PDGF.AA

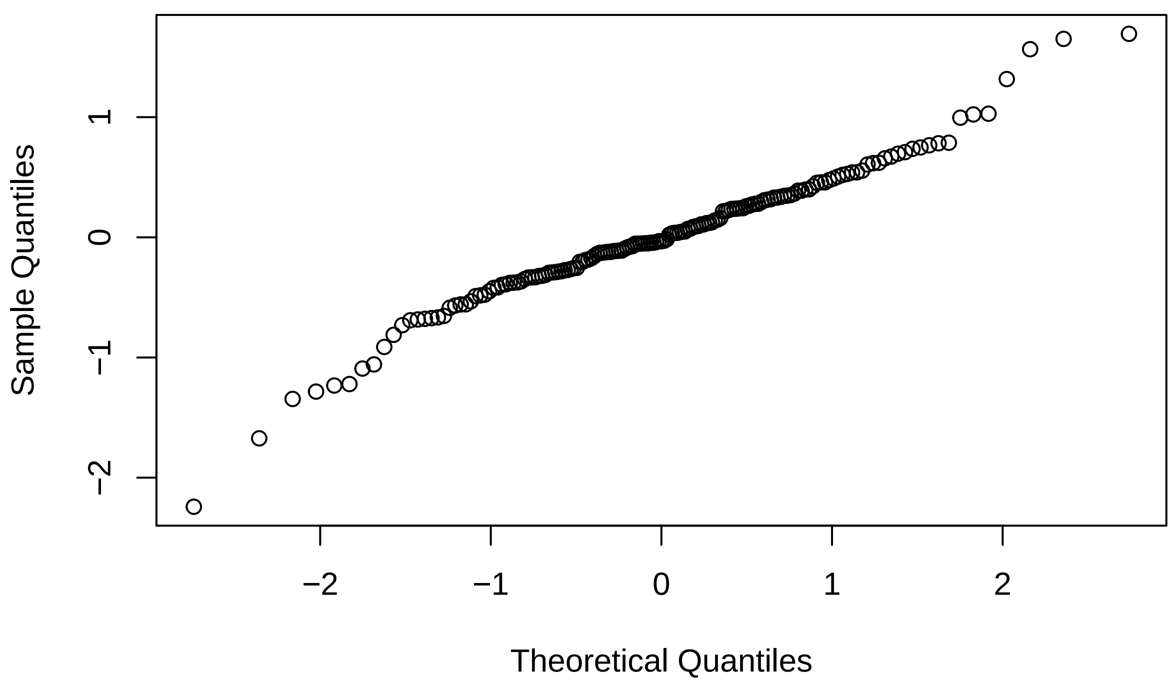

PDGF.ABBB

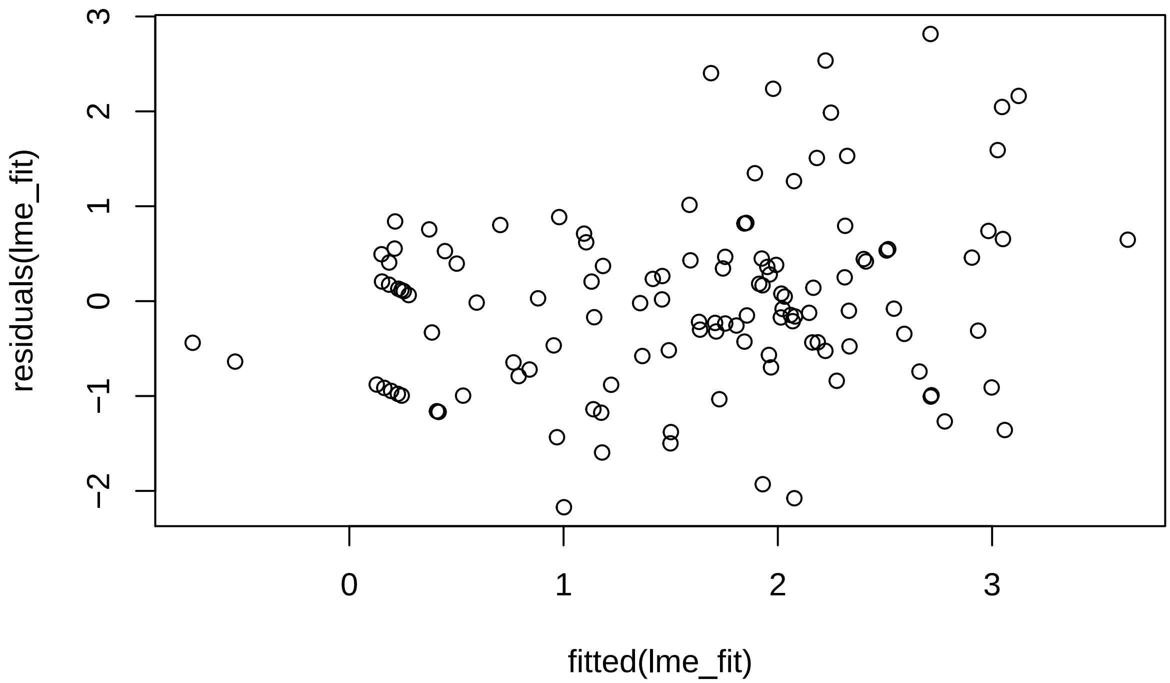

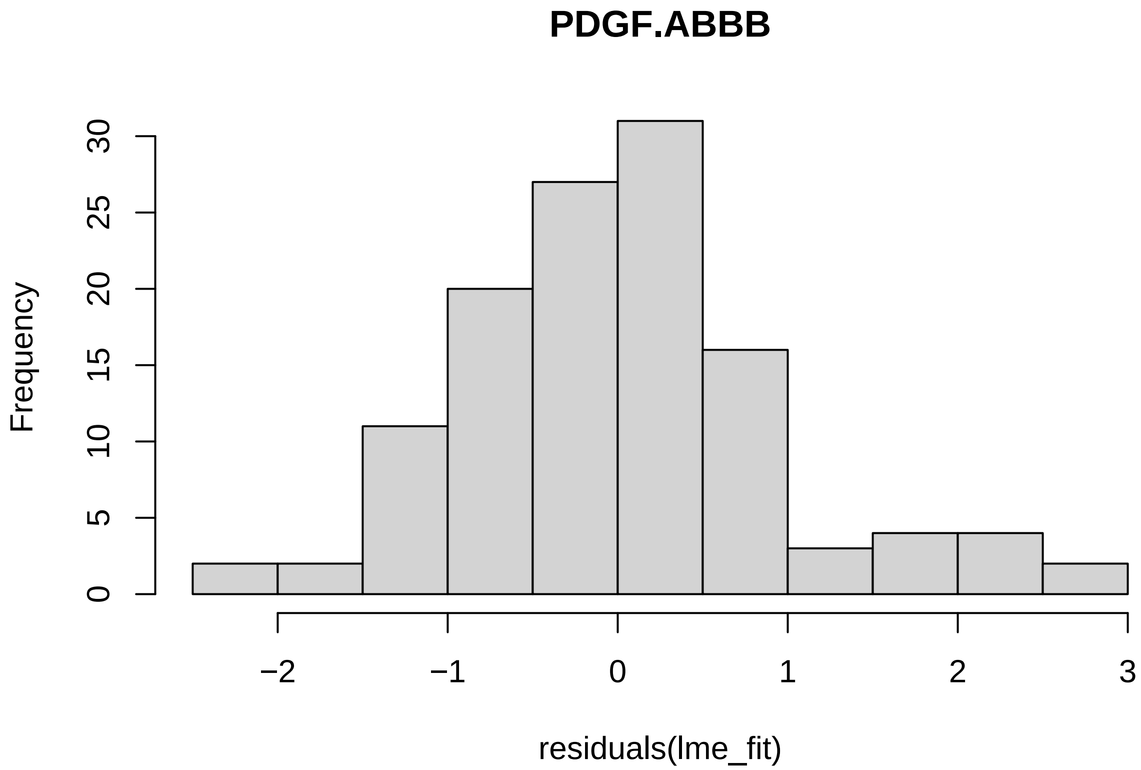


```
## Warning in if (!is.na(a)) {: the condition has length > 1 and only the first
## element will be used
```

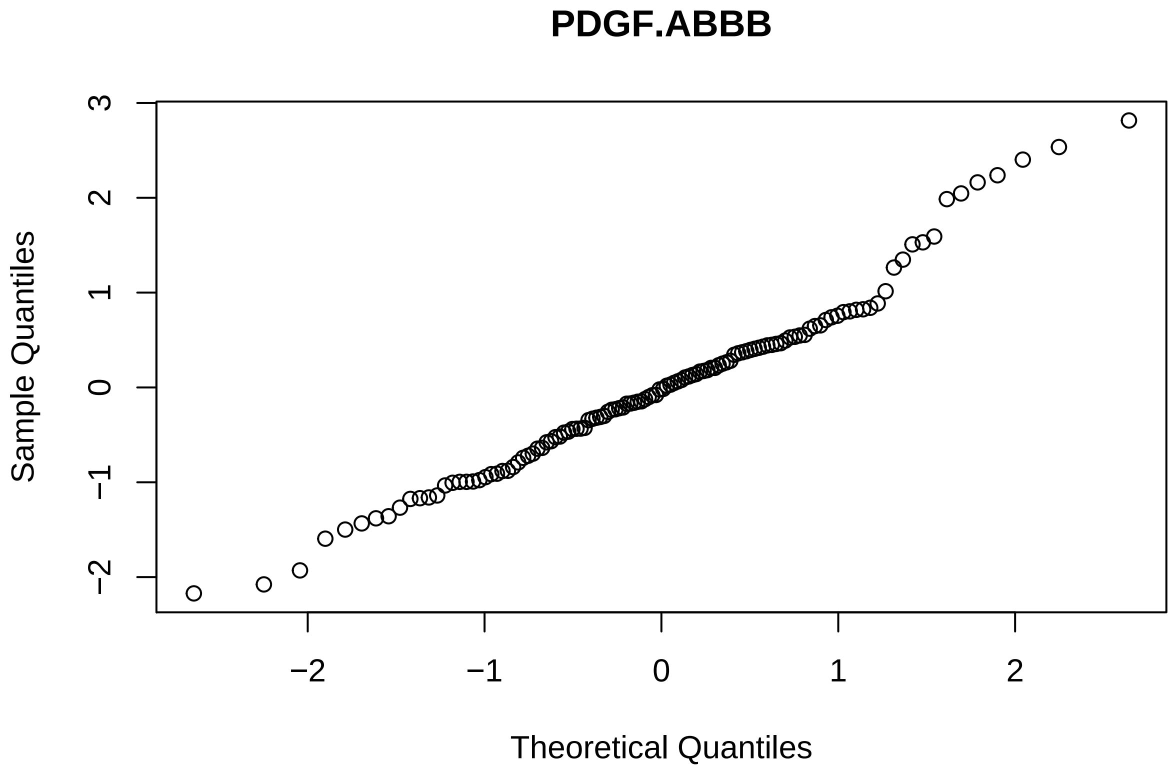

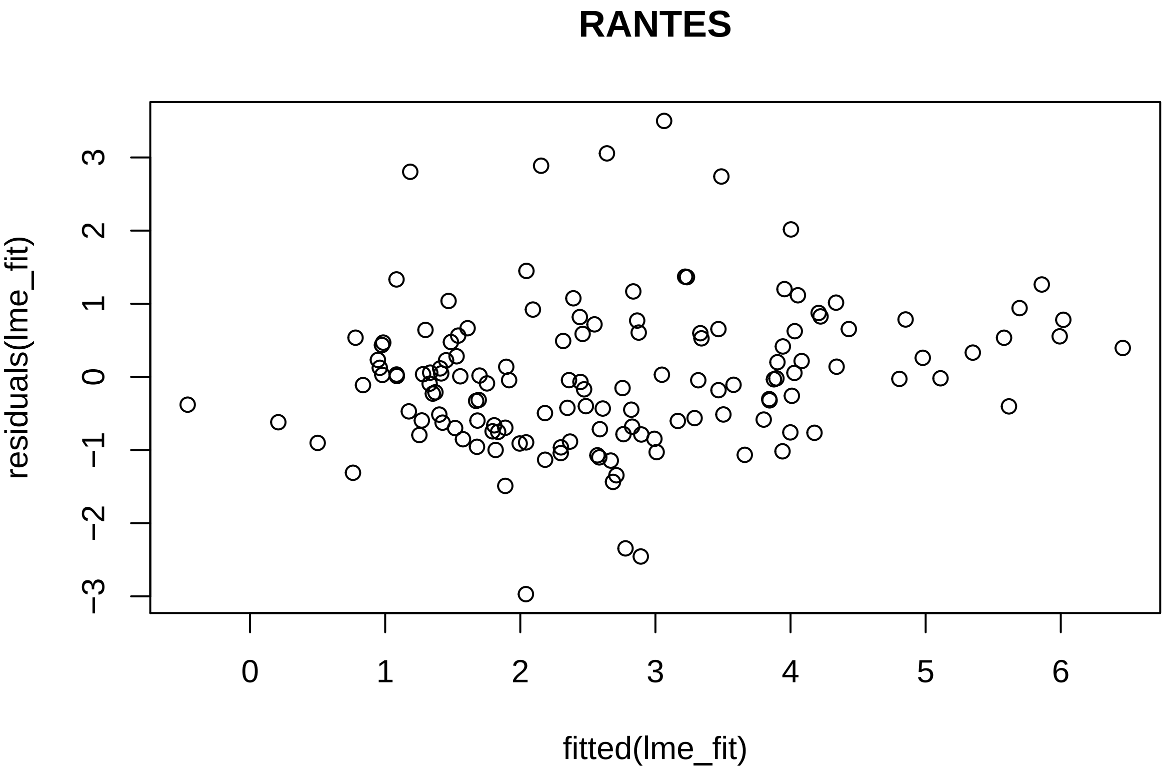

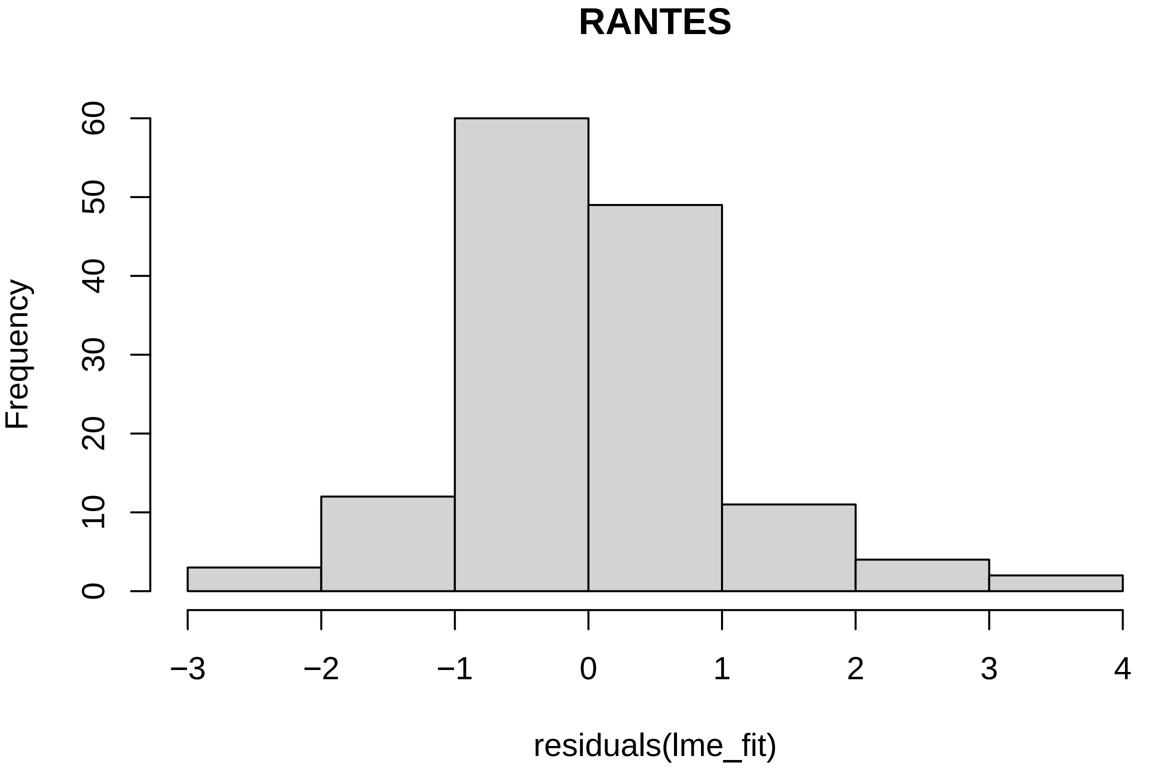


```
## Warning in if (!is.na(a)) {: the condition has length > 1 and only the first
## element will be used
```

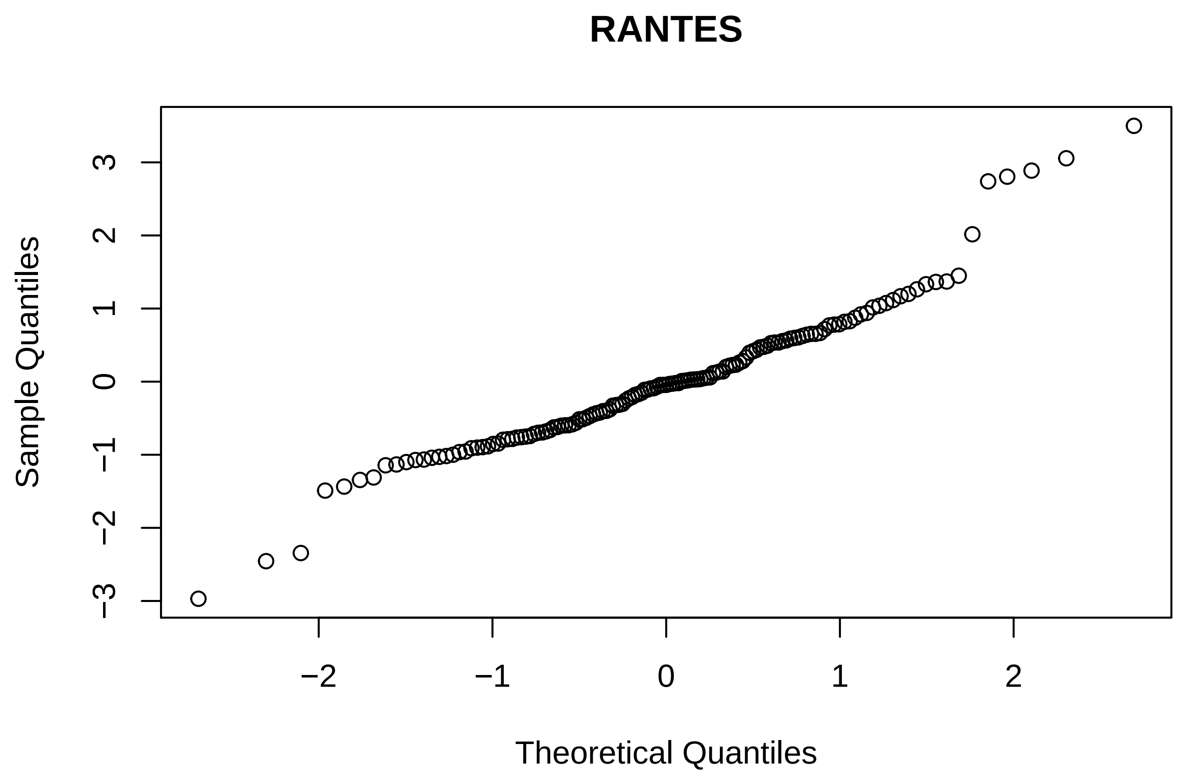
RANTES
Sample Quantiles
Theoretical Quantiles
−3
−2
−1
0
1
2
3

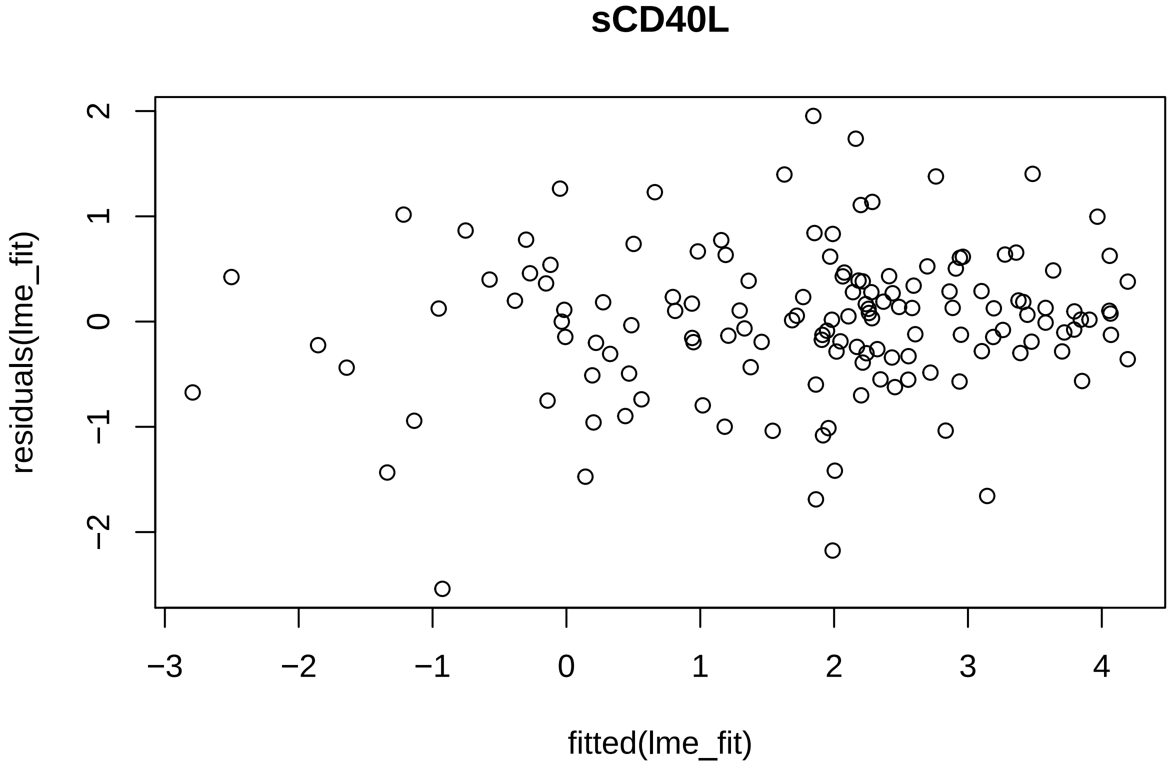
sCD40L
residuals(lme_fit)
fitted(lme_fit)
−3
−2
−1
0
1
2
3
4

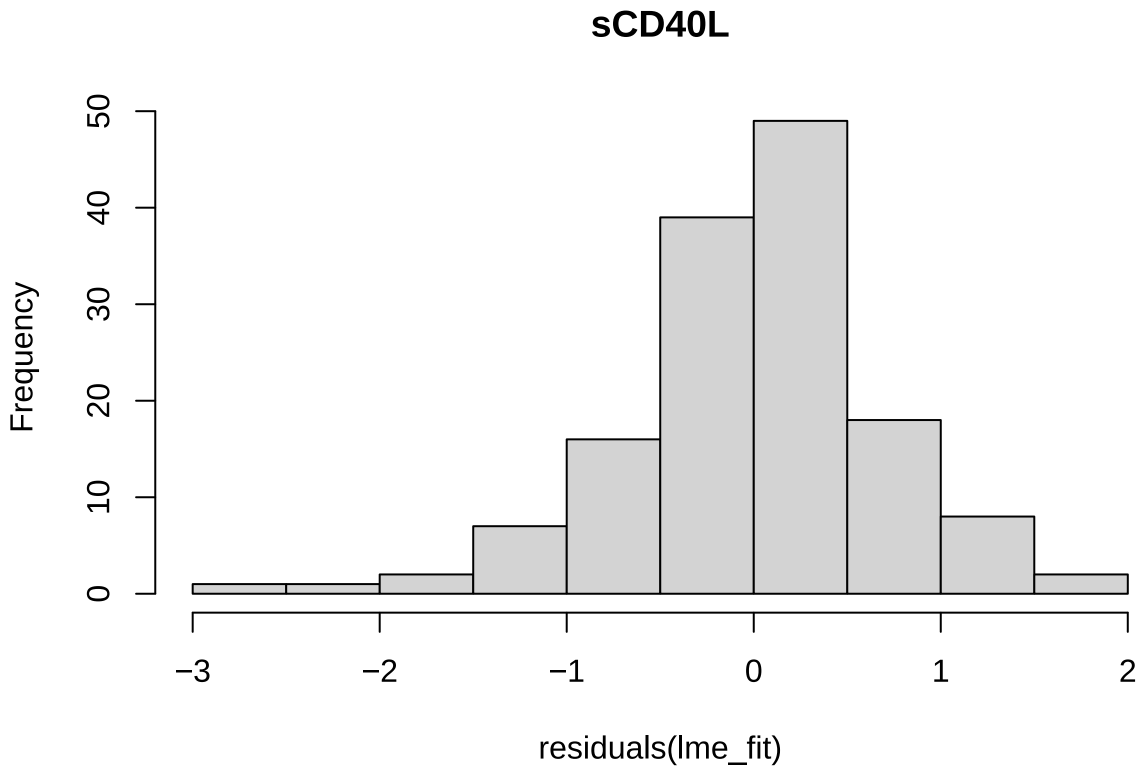


```
## [1] "Error: MD_sIL.2Ra ~ A_sIL.2Ra + time + WCC + CRP + Temp + Infection + rhIL1ra"
## [1] "Error: MD_TGFa ~ A_TGFa + time + WCC + CRP + Temp + Infection + rhIL1ra"

## Warning in if (!is.na(a)) {: the condition has length > 1 and only the first
## element will be used
```

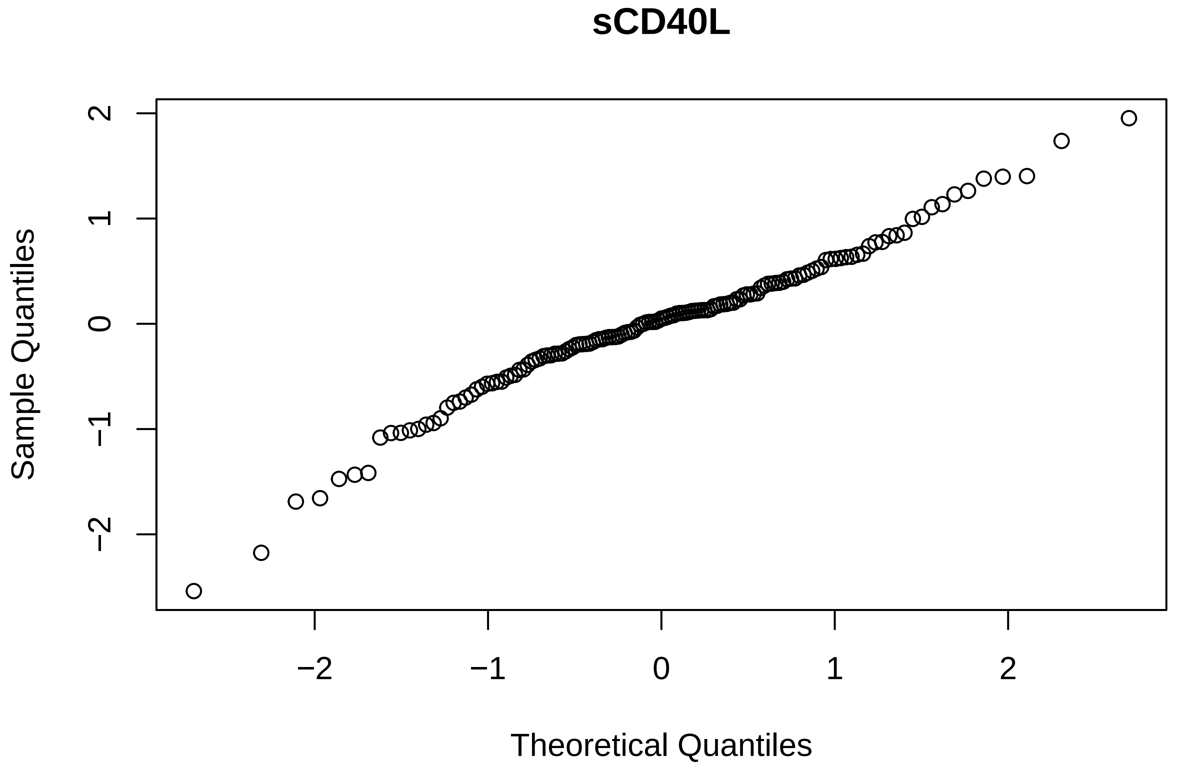

TNFa

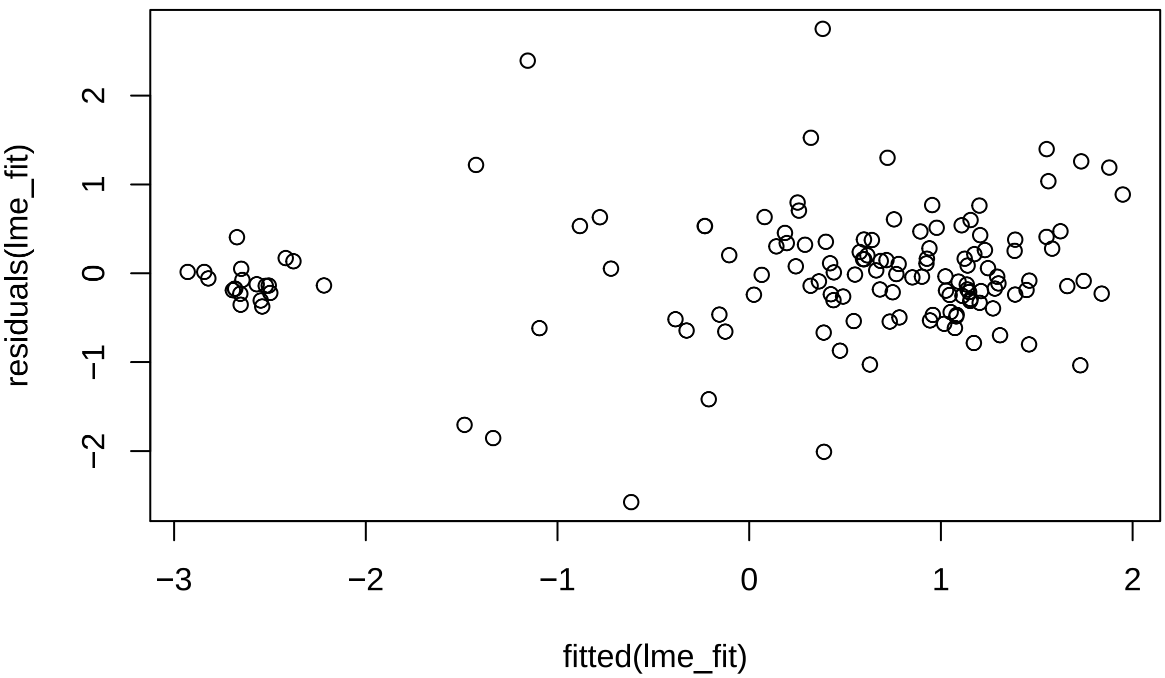

residuals(lme_fit)
fitted(lme_fit)
−3
−2
−1
0
1
2

TNFa

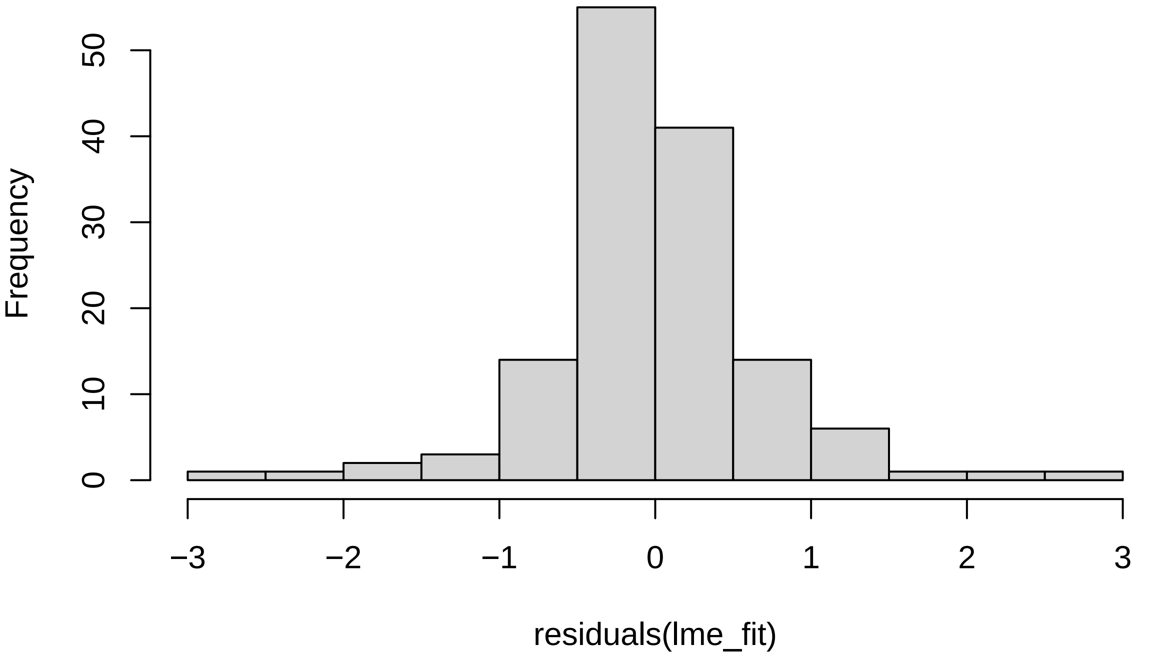

Frequency
residuals(lme_fit)
0
10
20
30
40
50
−3
−2
−1
0
1
2
3

TNFa

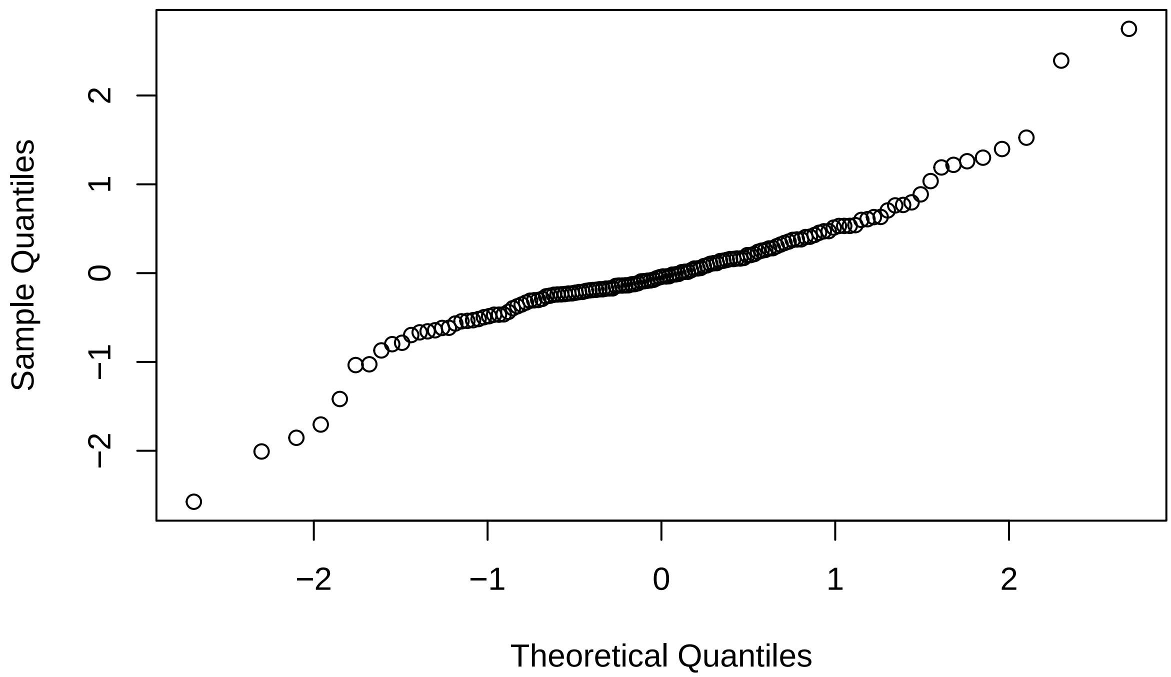

Sample Quantiles
2
1
0
−1
−2
−2
−1
0
1
2
Theoretical Quantiles

TNFb

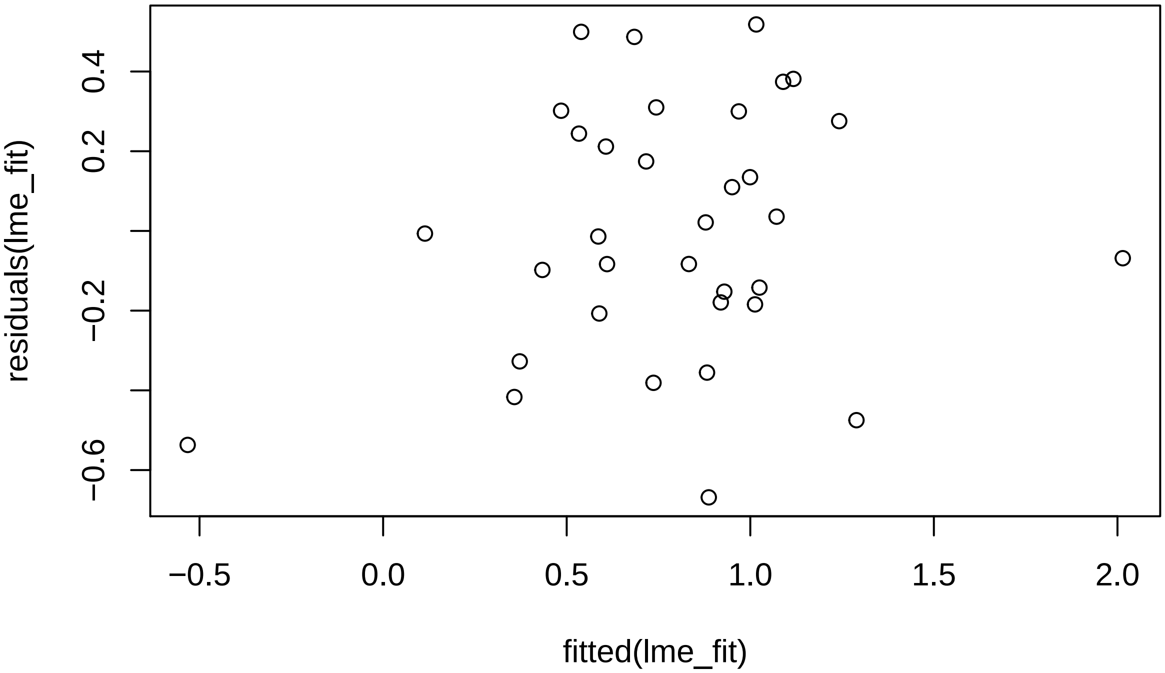

residuals(lme_fit)
0.4
0.2
−0.2
−0.6
−0.5
0.0
0.5
1.0
1.5
2.0
fitted(lme_fit)

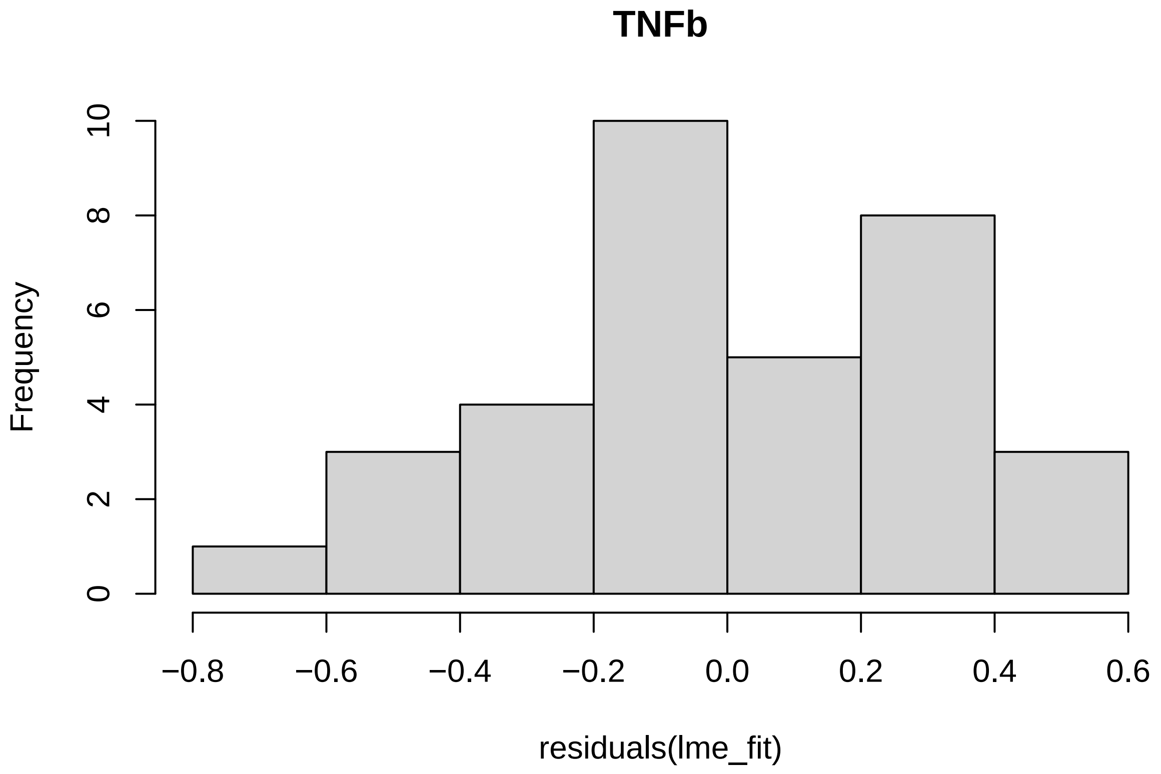


```
## Warning in if (!is.na(a)) {: the condition has length > 1 and only the first
## element will be used
```

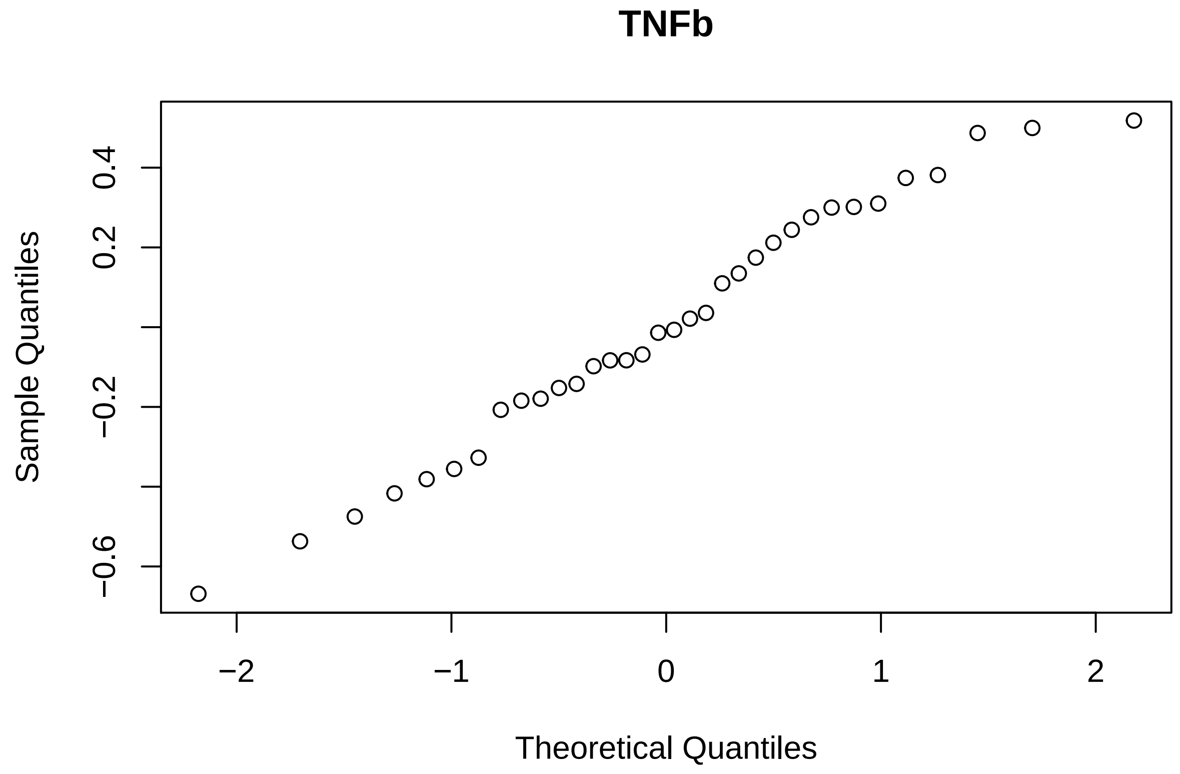

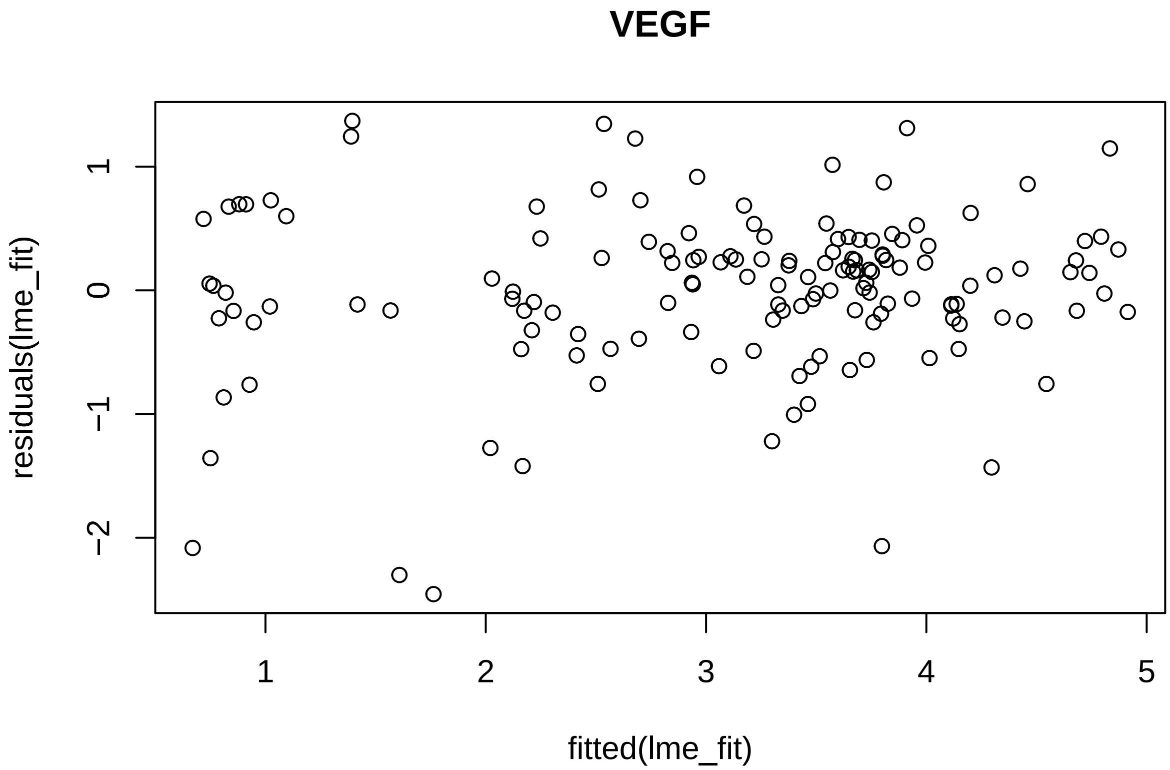
VEGF
residuals(lme_fit)
fitted(lme_fit)
1
0
−1
−2
1
2
3
4
5

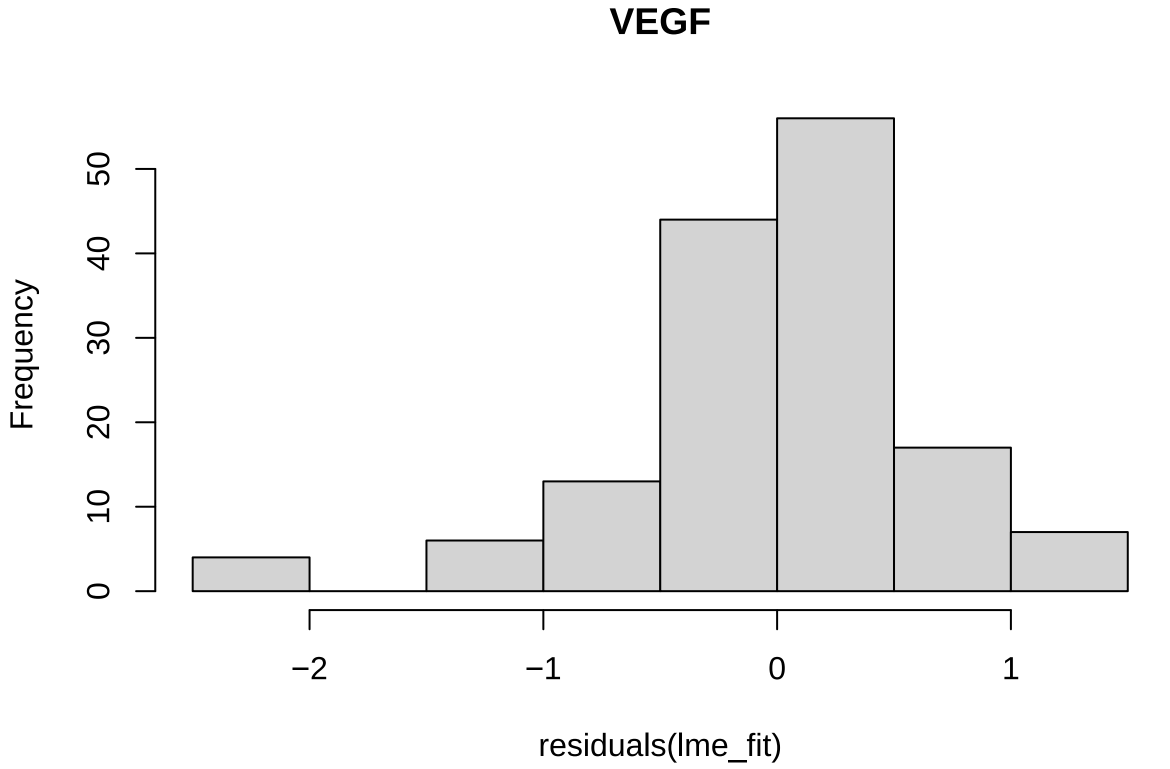
VEGF
Frequency
residuals(lme_fit)
0
10
20
30
40
50
−2
−1
0
1

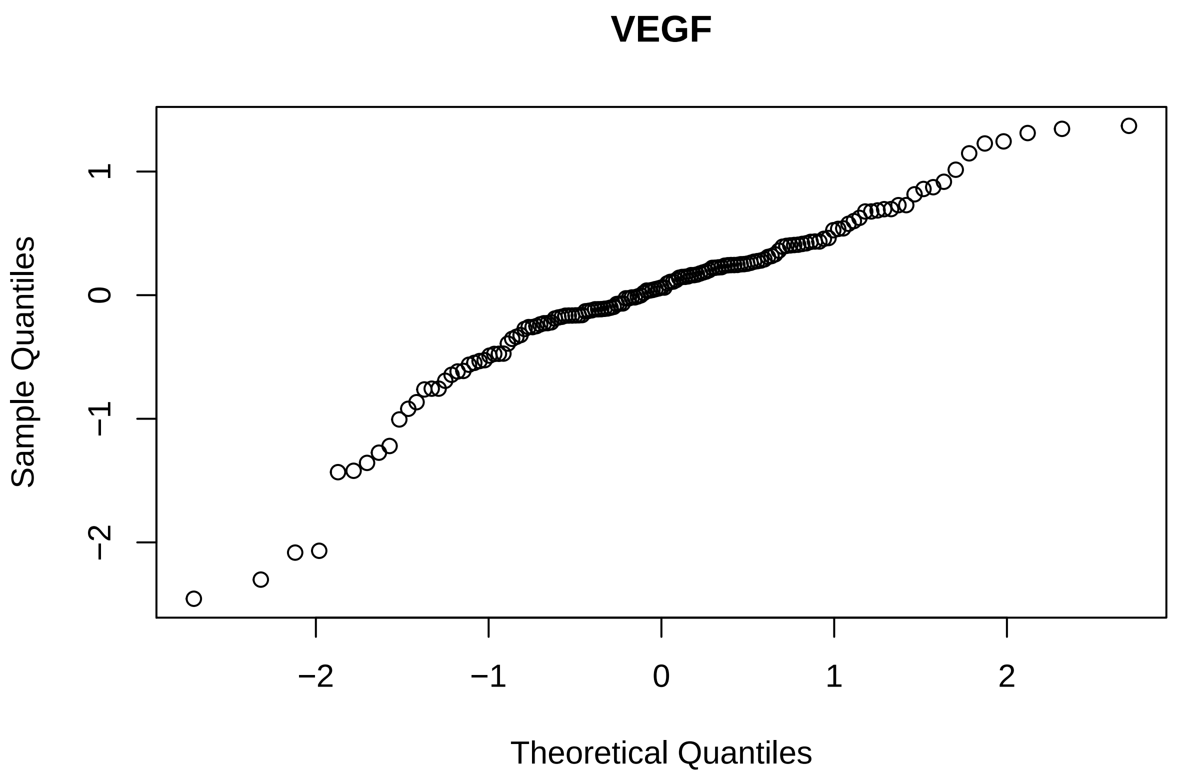


```
# print(colMeans(abl_data, na.rm = TRUE))

yield_matrix <- matrix(NA,
                       nrow = 42,
                       ncol = 7)
colnames(yield_matrix) <- colnames(coeff_matrix)
rownames(yield_matrix) <- rownames(sd_matrix)
for (cytokine in 1:42) {
  sd_md.cyt <- sd_matrix[cytokine, 1]
  sd_a.cyt <- sd_matrix[cytokine, 2]
  sd_vector[2] <- sd_a.cyt
  for (variable in 1:7) {
    yield_matrix[cytokine, variable] <-
      coeff_matrix[cytokine, variable] * sd_vector[variable] / sd_md.cyt
  }
}

# coeff_matrix.scaled <- scale(coeff_matrix)

library(massageR)
library(gplots)

pval_matrix <- cbind(rownames(pval_matrix), pval_matrix)
sign_matrix <- pval_matrix < 0.05
colnames(pval_matrix)[1] <- "Cytokine"
wb <- createWorkbook()
addWorksheet(wb, "Sheet 1")
writeData(wb = wb, sheet = "Sheet 1", x = pval_matrix)
color <- createStyle(fgFill = "yellow")
```

```r
for (cols in 1:dim(sign_matrix)[2]) {
  addStyle(wb = wb, sheet = "Sheet 1", style = color,
           rows = which(sign_matrix[ , cols]) + 1, cols = cols)
}
saveWorkbook(wb = wb, file = "pvals_fig1a_200521.xlsx", overwrite = TRUE)
labs <- matrix("", dim(na.omit(sign_matrix))[1], dim(sign_matrix)[2] - 1)
labs[na.omit(sign_matrix[ , 2:dim(sign_matrix)[2]])] <- "*"

x <- as.matrix(na.omit(yield_matrix))
colnames(x)[colnames(x) == "A_cytokine"] <- "Art. cytokine"
z <- heat.clust(x,
                scaledim="none",
                zlim=c(-0.5,0.5),
                zlim_select = c("dend","outdata"),
                reorder=c("column","row"),
                distfun  = function(x) as.dist(1-cor(t(x))),
                hclustfun= function(x) hclust(x, method="average"))

tiff("nlme_heatmap200521.revised210107.log.tiff", width = 1000, height = 1000,
     pointsize = 24)
# pdf("nlme_heatmap200521.pdf")
heatmap.2(z$data,
          Rowv = FALSE,
          Colv = FALSE,
          trace="none",
          scale="none",
          dendrogram = "none",
          symbreaks = TRUE,
          col=rev(colorRampPalette(brewer.pal(10, "RdBu"))(256)),
          cexRow = 0.8,
          cexCol = 0.8,
          cellnote = labs, notecol = "cyan", notecex = 2
          )
dev.off()
```

```
## pdf
##   2
```

```r
coeff_matrix.na <- rownames(coeff_matrix[rowSums(is.na(coeff_matrix)) > 0, ])
print(paste("No linear model was found for",
            coeff_matrix.na))
```

```
## [1] "No linear model was found for MD_IL.2"
## [2] "No linear model was found for MD_IL.3"
## [3] "No linear model was found for MD_IL.5"
## [4] "No linear model was found for MD_sIL.2Ra"
## [5] "No linear model was found for MD_TGFa"
```

```r
largest <- apply(coeff_matrix, 2, max, na.rm = TRUE)
which_largest <- apply(coeff_matrix, 2, which.max)
names_largest <- rownames(coeff_matrix)[which_largest]
equivalent_largest <- largest["Infection"]/largest
specific_equivalent_largest <-
  largest["Infection"] /
  coeff_matrix[names_largest[colnames(coeff_matrix) == "Infection"], ]
```

```r
smallest <- apply(coeff_matrix, 2, min, na.rm = TRUE)
which_smallest <- apply(coeff_matrix, 2, which.min)
names_smallest <- rownames(coeff_matrix)[which_smallest]
equivalent_smallest <- smallest["Infection"]/smallest
specific_equivalent_smallest <-
  smallest["Infection"] /
  coeff_matrix[names_smallest[colnames(coeff_matrix) == "Infection"], ]
na_row <- rep(NA, length(largest))
coeff_matrix <- rbind(coeff_matrix,
                      na_row,
                      names_largest,
                      largest,
                      equivalent_largest,
                      specific_equivalent_largest,
                      na_row,
                      names_smallest,
                      smallest,
                      equivalent_smallest,
                      specific_equivalent_smallest)
rownames(coeff_matrix)[(nrow(coeff_matrix) - 9) : nrow(coeff_matrix)] <-
  c(NA, "Largest cytokine", "Coefficient", "Maximum infection equivalent",
    paste(coeff_matrix["names_largest", "Infection"], "infection equivalent"),
    NA, "Smallest cytokine", "Coefficient", "Infection equivalent",
    paste(coeff_matrix["names_smallest", "Infection"], "infection equivalent"))
coeff_matrix <- cbind(Cytokine = rownames(coeff_matrix), coeff_matrix)

write.xlsx(coeff_matrix, "coefficients_fig1a_200521.xlsx")

vif_data <- data.frame(Cytokine = rownames(vif_matrix),
                       vif_matrix)
write.xlsx(x = vif_data,
           file = "vif_matrix4.1.xlsx")

anova_data <- data.frame(Cytokine = rownames(vif_matrix),
                         anova_matrix)

write.xlsx(x = anova_data,
           file = "anova_matrix4.1.xlsx")
```

Code for Figure 3B

Philipp

Revised: May 11, 2021

Contents

Load data **1**

Run nlme per patient and cytokine **1**

Draw PCA from coefficients **8**

Load data

```
library(openxlsx)
ip_data <- read.xlsx("../Dataset/Interpolated_data4.1.xlsx")
```

Run nlme per patient and cytokine

```
library(nlme)
library(ggplot2)
library(plyr)
library(pheatmap)
library(gplots)
```

```
##
## Attaching package: 'gplots'

## The following object is masked from 'package:stats':
##
##     lowess
```

```
library(made4)
```

```
## Loading required package: RColorBrewer

## Loading required package: scatterplot3d

## Loading required package: Biobase

## Loading required package: BiocGenerics

## Warning: package 'BiocGenerics' was built under R version 4.0.5

## Loading required package: parallel
```

```
##
## Attaching package: 'BiocGenerics'

## The following objects are masked from 'package:parallel':
##
##     clusterApply, clusterApplyLB, clusterCall, clusterEvalQ,
##     clusterExport, clusterMap, parApply, parCapply, parLapply,
##     parLapplyLB, parRapply, parSapply, parSapplyLB

## The following objects are masked from 'package:stats':
##
##     IQR, mad, sd, var, xtabs

## The following objects are masked from 'package:base':
##
##     anyDuplicated, append, as.data.frame, basename, cbind, colnames,
##     dirname, do.call, duplicated, eval, evalq, Filter, Find, get, grep,
##     grepl, intersect, is.unsorted, lapply, Map, mapply, match, mget,
##     order, paste, pmax, pmax.int, pmin, pmin.int, Position, rank,
##     rbind, Reduce, rownames, sapply, setdiff, sort, table, tapply,
##     union, unique, unsplit, which.max, which.min

## Welcome to Bioconductor
##
##     Vignettes contain introductory material; view with
##     'browseVignettes()'. To cite Bioconductor, see
##     'citation("Biobase")', and for packages 'citation("pkgname")'.

## Loading required package: SummarizedExperiment

## Loading required package: MatrixGenerics

## Loading required package: matrixStats

##
## Attaching package: 'matrixStats'

## The following objects are masked from 'package:Biobase':
##
##     anyMissing, rowMedians

## The following object is masked from 'package:plyr':
##
##     count

##
## Attaching package: 'MatrixGenerics'

## The following objects are masked from 'package:matrixStats':
##
##     colAlls, colAnyNAs, colAnys, colAvgsPerRowSet, colCollapse,
##     colCounts, colCummaxs, colCummins, colCumprods, colCumsums,
##     colDiffs, colIQRDiffs, colIQRs, colLogSumExps, colMadDiffs,
##     colMads, colMaxs, colMeans2, colMedians, colMins, colOrderStats,
##     colProds, colQuantiles, colRanges, colRanks, colSdDiffs, colSds,
##     colSums2, colTabulates, colVarDiffs, colVars, colWeightedMads,
##     colWeightedMeans, colWeightedMedians, colWeightedSds,
##     colWeightedVars, rowAlls, rowAnyNAs, rowAnys, rowAvgsPerColSet,
##     rowCollapse, rowCounts, rowCummaxs, rowCummins, rowCumprods,
##     rowCumsums, rowDiffs, rowIQRDiffs, rowIQRs, rowLogSumExps,
```

```
##     rowMadDiffs, rowMads, rowMaxs, rowMeans2, rowMedians, rowMins,
##     rowOrderStats, rowProds, rowQuantiles, rowRanges, rowRanks,
##     rowSdDiffs, rowSds, rowSums2, rowTabulates, rowVarDiffs, rowVars,
##     rowWeightedMads, rowWeightedMeans, rowWeightedMedians,
##     rowWeightedSds, rowWeightedVars

## The following object is masked from 'package:Biobase':
##
##     rowMedians

## Loading required package: GenomicRanges

## Loading required package: stats4

## Loading required package: S4Vectors

##
## Attaching package: 'S4Vectors'

## The following object is masked from 'package:gplots':
##
##     space

## The following object is masked from 'package:plyr':
##
##     rename

## The following object is masked from 'package:base':
##
##     expand.grid

## Loading required package: IRanges

##
## Attaching package: 'IRanges'

## The following object is masked from 'package:plyr':
##
##     desc

## The following object is masked from 'package:nlme':
##
##     collapse

## Loading required package: GenomeInfoDb

## Warning: package 'GenomeInfoDb' was built under R version 4.0.5
```

```r
time <- ip_data$Time
wcc <- ip_data$WCC
crp <- ip_data$CRP
temp <- ip_data$Temp
infection <- ip_data$Infection
patient <- ip_data$Patient
rhIL1ra <- ip_data$rhIL1ra

coeff_matrix <- matrix(NA,
                       nrow = 42,
                       ncol = 7)
colnames(coeff_matrix) <- c("Time",
                            "MD_cytokine",
```

```r
                            "CRP",
                            "WCC",
                            "Temp",
                            "Infection",
                            "rhIl1ra")
rownames(coeff_matrix) <- colnames(ip_data)[seq(from = 9,
                                                to = 133,
                                                by = 3)]
anova_matrix <- coeff_matrix

sd_matrix <- matrix(NA,
                    ncol = 2,
                    nrow = 42)
colnames(sd_matrix) <- c("A_cytokine",
                         "MD_cytokine")
rownames(sd_matrix) <- substr(rownames(coeff_matrix), 3, 100)
sd_time <- sd(time, na.rm = TRUE)
sd_crp <- sd(crp, na.rm = TRUE)
sd_wcc <- sd(wcc, na.rm = TRUE)
sd_temp <- sd(temp, na.rm = TRUE)
sd_infection <- sd(infection, na.rm = TRUE)
sd_rhIL1ra <- sd(rhIL1ra, na.rm = TRUE)
sd_vector <- c(sd_time,
               NA,
               sd_crp,
               sd_wcc,
               sd_temp,
               sd_infection,
               sd_rhIL1ra)

abl_data <- matrix(NA,
                   ncol = 3,
                   nrow = 42)
colnames(abl_data) <- c("AIC",
                        "BIC",
                        "loglik")

library(predictmeans)
```

```
## Loading required package: lme4

## Loading required package: Matrix

##
## Attaching package: 'Matrix'

## The following object is masked from 'package:S4Vectors':
##
##     expand

##
## Attaching package: 'lme4'

## The following object is masked from 'package:nlme':
##
##     lmList
```

```
##
## Attaching package: 'predictmeans'
## The following object is masked from 'package:gplots':
##
##     residplot
```

```
for (cytokine in 1:42) {
  a_cytokine <- log(ip_data[ , cytokine * 3 + 6])
  md_cytokine <- log(ip_data[ , cytokine * 3 + 5])

  sd_matrix[cytokine, 1] <- sd(a_cytokine, na.rm = TRUE)
  sd_matrix[cytokine, 2] <- sd(md_cytokine, na.rm = TRUE)

  lme_fit <- tryCatch({
    lme(a_cytokine ~ time + md_cytokine + crp + wcc + temp + infection + rhIL1ra,
        random = ~ time | patient,
        correlation = corAR1(form = ~ time | patient),
        na.action = na.omit)
  }, error = function(e) {
    NA
  }
  )

  if (class(lme_fit) == "lme") {
    lme_names <- names(rename(lme_fit$coefficients$fixed,
                              c("md_cytokine" =
                                  colnames(ip_data)[cytokine * 3 + 5])))

    # my_plot_hook <- function(x, options)
    #     paste("\n", knitr::hook_plot_tex(x, options), "\n")
    #     knitr::knit_hooks$set(plot = my_plot_hook)
    #
    # barplot(height = lme_fit$coefficients$fixed[-1],
    #         names.arg = lme_names[-1],
    #         main = paste0("Coefficients of ",
    #                       substring(colnames(ip_data)[cytokine * 3 + 5], 4),
    #                       " for model ",
    #                       colnames(ip_data)[cytokine * 3 + 5],
    #                       " ~ ",
    #                       colnames(ip_data)[cytokine * 3 + 6],
    #                       " + time + WCC + CRP + Temp + Infection + rhIL1ra"),
    #         ylab = "Coefficient magnitude",
    #         cex.names = 0.3,
    #         cex.main = 0.6,
    #         col = "blue")

    coeff_matrix[cytokine, ] <- lme_fit$coefficients$fixed[-1]
    anova_matrix[cytokine, ] <- anova(lme_fit)$`p-value`[2:(dim(anova_matrix)[2]+1)]

    # abl_data[cytokine, 1] <- AIC(lme_fit)
    # abl_data[cytokine, 2] <- BIC(lme_fit)
    # abl_data[cytokine, 3] <- lme_fit$logLik
    #
    # my_plot_hook <- function(x, options)
```

```
    #     paste("\n", knitr::hook_plot_tex(x, options), "\n")
    #     knitr::knit_hooks$set(plot = my_plot_hook)
    # plot(fitted(lme_fit),residuals(lme_fit),
    #      main = substr(colnames(ip_data)[cytokine * 3 + 6], 3, 100))
    # my_plot_hook <- function(x, options)
    #     paste("\n", knitr::hook_plot_tex(x, options), "\n")
    #     knitr::knit_hooks$set(plot = my_plot_hook)
    # hist(residuals(lme_fit),
    #      main = substr(colnames(ip_data)[cytokine * 3 + 6], 3, 100))
    # my_plot_hook <- function(x, options)
    #     paste("\n", knitr::hook_plot_tex(x, options), "\n")
    #     knitr::knit_hooks$set(plot = my_plot_hook)
    # qqnorm(residuals(lme_fit),
    #      main = paste(substr(colnames(ip_data)[cytokine * 3 + 6], 3, 100)))


  } else {
    print(paste0("Error: ",
                 colnames(ip_data)[cytokine * 3 + 6],
                 " ~ ",
                 colnames(ip_data)[cytokine * 3 + 5],
                 " + time + WCC + CRP + Temp + Infection + rhIL1ra"))
  }
}
```

```
## [1] "Error: A_EGF ~ MD_EGF + time + WCC + CRP + Temp + Infection + rhIL1ra"
## [1] "Error: A_IL.1b ~ MD_IL.1b + time + WCC + CRP + Temp + Infection + rhIL1ra"
## [1] "Error: A_IL.3 ~ MD_IL.3 + time + WCC + CRP + Temp + Infection + rhIL1ra"
## [1] "Error: A_IL.9 ~ MD_IL.9 + time + WCC + CRP + Temp + Infection + rhIL1ra"
## [1] "Error: A_IL.17 ~ MD_IL.17 + time + WCC + CRP + Temp + Infection + rhIL1ra"
## [1] "Error: A_RANTES ~ MD_RANTES + time + WCC + CRP + Temp + Infection + rhIL1ra"
```

```
# print(colMeans(abl_data, na.rm = TRUE))

yield_matrix <- matrix(NA,
                       nrow = 42,
                       ncol = 7)
colnames(yield_matrix) <- colnames(coeff_matrix)
rownames(yield_matrix) <- rownames(sd_matrix)
for (cytokine in 1:42) {
  sd_md.cyt <- sd_matrix[cytokine, 2]
  sd_a.cyt <- sd_matrix[cytokine, 1]
  sd_vector[2] <- sd_md.cyt
  for (variable in 1:7) {
    yield_matrix[cytokine, variable] <-
      coeff_matrix[cytokine, variable] * sd_vector[variable] / sd_a.cyt
  }
}

# coeff_matrix.scaled <- scale(coeff_matrix)

library(massageR)
library(gplots)
```

```r
anova_matrix <- cbind(rownames(anova_matrix), anova_matrix)
sign_matrix <- anova_matrix < 0.05
colnames(anova_matrix)[1] <- "Cytokine"
wb <- createWorkbook()
addWorksheet(wb, "Sheet 1")
writeData(wb = wb, sheet = "Sheet 1", x = anova_matrix)
color <- createStyle(fgFill = "yellow")
for (cols in 1:dim(sign_matrix)[2]) {
  addStyle(wb = wb, sheet = "Sheet 1", style = color,
           rows = which(sign_matrix[ , cols]) + 1, cols = cols)
}
saveWorkbook(wb = wb, file = "pvals_fig1b_200407.xlsx", overwrite = TRUE)
labs <- matrix("", dim(na.omit(sign_matrix))[1], dim(sign_matrix)[2] - 1)
labs[na.omit(sign_matrix[ , 2:dim(sign_matrix)[2]])] <- "*"

x <- as.matrix(na.omit(yield_matrix))
colnames(x)[colnames(x) == "MD_cytokine"] <- "MD cytokine"
z <- heat.clust(x,
                scaledim="none",
                zlim=c(-0.5,0.5),
                zlim_select = c("dend","outdata"),
                reorder=c("column","row"),
                distfun  = function(x) as.dist(1-cor(t(x))),
                hclustfun= function(x) hclust(x, method="average"))

tiff("nlme_heatmap200407.arterial.revised210107.tiff", width = 1000, height = 1000,
     pointsize = 24)
# pdf("nlme_heatmap200407.arterial.pdf")
heatmap.2(z$data,
          Rowv=FALSE,
          Colv=FALSE,
          trace="none",
          scale="none",
          dendrogram = "none",
          symbreaks = TRUE,
          col=rev(colorRampPalette(brewer.pal(10, "RdBu"))(256)),
          cexRow = 0.8,
          cexCol = 0.8,
          cellnote = labs, notecol = "cyan", notecex = 2
          )
dev.off()
```

```
## pdf
##   2
```

```r
coeff_matrix.na <- rownames(coeff_matrix[rowSums(is.na(coeff_matrix)) > 0, ])
print(paste("No linear model was found for",
            coeff_matrix.na))
```

```
## [1] "No linear model was found for A_EGF"
## [2] "No linear model was found for A_IL.1b"
## [3] "No linear model was found for A_IL.3"
## [4] "No linear model was found for A_IL.9"
## [5] "No linear model was found for A_IL.17"
## [6] "No linear model was found for A_RANTES"
```

```r
largest <- apply(coeff_matrix, 2, max, na.rm = TRUE)
which_largest <- apply(coeff_matrix, 2, which.max)
names_largest <- rownames(coeff_matrix)[which_largest]
equivalent_largest <- largest["Infection"]/largest
specific_equivalent_largest <-
  largest["Infection"] /
  coeff_matrix[names_largest[colnames(coeff_matrix) == "Infection"], ]
smallest <- apply(coeff_matrix, 2, min, na.rm = TRUE)
which_smallest <- apply(coeff_matrix, 2, which.min)
names_smallest <- rownames(coeff_matrix)[which_smallest]
equivalent_smallest <- smallest["Infection"]/smallest
specific_equivalent_smallest <-
  smallest["Infection"] /
  coeff_matrix[names_smallest[colnames(coeff_matrix) == "Infection"], ]
na_row <- rep(NA, length(largest))
coeff_matrix <- rbind(coeff_matrix,
                      na_row,
                      names_largest,
                      largest,
                      equivalent_largest,
                      specific_equivalent_largest,
                      na_row,
                      names_smallest,
                      smallest,
                      equivalent_smallest,
                      specific_equivalent_smallest)
rownames(coeff_matrix)[(nrow(coeff_matrix) - 9) : nrow(coeff_matrix)] <-
  c(NA, "Largest cytokine", "Coefficient", "Maximum infection equivalent",
    paste(coeff_matrix["names_largest", "Infection"], "infection equivalent"),
    NA, "Smallest cytokine", "Coefficient", "Infection equivalent",
    paste(coeff_matrix["names_smallest", "Infection"], "infection equivalent"))
coeff_matrix <- cbind(Cytokine = rownames(coeff_matrix), coeff_matrix)

write.xlsx(coeff_matrix, "coefficients_fig1b_200407.xlsx")
```

Draw PCA from coefficients

```r
# library(FactoMineR)
# library(factoextra)
# library(corrplot)
# library(dplyr)
# library(viridis)
# library(lattice)
# library(tidyverse)
# library(gridExtra)
# library(viridis)
# library(ggbiplot)
#
# yield_matrix.narm <- na.omit(yield_matrix)
# pca <- PCA(yield_matrix.narm, graph = FALSE)
# fviz_eig(pca, title = "Blood scree plot")
```

```
# # print(fviz_pca_ind(pca,
# #                    col.ind = yield_matrix.narm[ , 3],
# #                    geom = "point",
# #                    pointsize = 1,
# #                    axes = c(1,2)) +
# #         coord_fixed() +
# #         scale_color_viridis(name = "CRP") +
# #         ggtitle("graph_title") +
# #         theme(text = element_text(size = 10)))
# fviz_pca_biplot(pca, repel = TRUE, title = "Blood biblot")
```

Code for Figure 5

Philipp

Revised: May 11, 2021

Contents

Load data 1

Plot before and after infection 1

Load data

```
library(openxlsx)
ip_data <- read.xlsx("../Dataset/Interpolated_data4.3.Adel201001.xlsx")

# Fix wrong age
age <- c(44, 25, 27, 25, 58, 61, 49, 60, 30, 39,
         41, 37, 43, 22, 18, 46, 27, 47, 28, 51)
for (pat in 1:20) {
  ip_data$Age[ip_data$Patient == pat] <- age[pat]
}
```

Plot before and after infection

```
library(ggplot2)
library(tidyverse)
```

```
## -- Attaching packages --------------------------------------- tidyverse 1.3.1 --

## v tibble  3.1.3     v dplyr   1.0.7
## v tidyr   1.1.3     v stringr 1.4.0
## v readr   2.0.0     v forcats 0.5.1
## v purrr   0.3.4

## -- Conflicts ------------------------------------------ tidyverse_conflicts() --
## x dplyr::filter() masks stats::filter()
## x dplyr::lag()    masks stats::lag()
```

```
library(zoo)
```

```
##
## Attaching package: 'zoo'
```

```
## The following objects are masked from 'package:base':
##
##     as.Date, as.Date.numeric
```

```
library(cowplot)
library(ggpattern)
```

```
## Loading required namespace: memoise
```

```
inf_ind <- ip_data %>%
  group_by(Patient) %>%
  summarise(inf_yes =
              any(Infection == 1, na.rm = TRUE) &
              any(Infection == 0, na.rm = TRUE))
sign_cyt <- c(
  "G.CSF",
  "IL.1b",
  "IL.1ra",
  "IL.13",
  "MDC",
  "MIP.1b",
  "PDGF.ABBB",
  "RANTES"
) %>%
  sort()

comb_dat <- ip_data %>%
  filter(Patient %in% inf_ind$Patient[inf_ind$inf_yes]) %>%
  select(Patient, Time, Infection,
         contains(sign_cyt) & (starts_with("MD_") | starts_with("A_"))) %>%
  mutate(Infection = ifelse(Infection == 1, "Yes", "No")) %>%
  group_by(Patient) %>%
  mutate(inf_time = Time[which(Infection == "Yes")[1]], .after = Time) %>%
  ungroup() %>%
  mutate(time_from_inf = Time - inf_time, .after = inf_time) %>%
  mutate(across(!Patient:Infection, log)) %>%
  pivot_longer(cols = !Patient:Infection,
               names_to = c("Compartment", "Cytokine"),
               names_pattern = "(.+)_(.+)",
               values_to = "Concentration") %>%
  mutate(Compartment = ifelse(Compartment == "A", "Arterial blood", "Brain-ECF")) %>%
  drop_na() %>%
  group_by(Infection, time_from_inf, Cytokine, Compartment) %>%
  summarise(Conc_err = 1.96*sd(Concentration)/sqrt(length(Concentration)),
            ctr = length(Concentration),
            Concentration = mean(Concentration)) %>%
  ungroup() %>%
  drop_na() # removes all timepoints with one entry, incl. pat 12's return to non-inf
```

```
## `summarise()` has grouped output by 'Infection', 'time_from_inf', 'Cytokine'. You can override using
```

```
plot_list <- vector("list", length = length(sign_cyt))
names(plot_list) <- sign_cyt
for (cyt in sign_cyt) {
  plot_list[[cyt]] <-
    ggplot(data = comb_dat %>%
```

```
            filter(Cytokine == cyt),
          aes(x = time_from_inf, y = Concentration,
              shape = Compartment, color = Compartment, pattern = Infection)) +
    geom_ribbon_pattern(aes(ymin = Concentration - Conc_err,
                            ymax = Concentration + Conc_err,
                            pattern_fill = Compartment),
                        pattern_colour = NA,
                        pattern_angle = 45,
                        pattern_density = 0.05,
                        pattern_spacing = 0.025,
                        pattern_key_scale_factor = 1,
                        alpha = 0.1) +
    scale_pattern_manual(values = c("stripe", "none")) +
    guides(color = guide_legend(override.aes = list(pattern = "none"))) +
    geom_point() +
    geom_line() +
    geom_vline(xintercept = 0, linetype = "dashed") +
    theme_bw() +
    ggtitle(cyt) +
    ylab("log(Cytokine concentration)") +
    xlab("Time from infection (hours)") +
    xlim(min(comb_dat$time_from_inf), max(comb_dat$time_from_inf))
}

p3 <-
  plot_grid(
    plot_list[[1]],
    plot_list[[2]],
    plot_list[[3]],
    plot_list[[4]],
    plot_list[[5]],
    plot_list[[6]],
    plot_list[[7]],
    plot_list[[8]],
    ncol = 2,
    labels = "AUTO",
    align = "hv")
title <- ggdraw() +
  draw_label(
    "Cytokine levels before and after infection",
    fontface = 'bold',
    x = 0,
    hjust = 0,
    size = 28
  ) +
  theme(
    # add margin on the left of the drawing canvas,
    # so title is aligned with left edge of first plot
    plot.margin = margin(0, 0, 0, 200)
  )
p4 <- plot_grid(
  title, p3,
  ncol = 1,
```

```r
  # rel_heights values control vertical title margins
  rel_heights = c(0.1, 1)
)
ggsave(filename = paste0("before_after_infe_cyt_all_210504_210803.pdf"),
       plot = p4,
       device = "pdf",
       height = 14,
       width = 14,
       units = "in")
```

Code for Table 1 and Additional file 1

Philipp

Revised: March 18, 2021

Contents

Aim **1**

Load data **1**

Inflammations **2**

T-test **9**

Missing **9**

The table **11**

Aim

Do a nice table. Good reference: * https://cran.r-project.org/web/packages/egg/vignettes/Ecosystem.html

Load data

```
patient_data <- read.csv(file = "../Dataset/Dataset_171024.csv",
                         sep = ";",
                         dec = ".",
                         colClasses = c(rep("numeric", times = 10),
                                        "numeric",
                                        rep("numeric", times = 4),
                                        rep("POSIXct", times = 2),
                                        rep("numeric", times = 134))) #load data

patient_data[cbind(matrix(FALSE,
                          ncol = 25,
                          nrow = 195),
                   patient_data[ , 26:151] == 0)] <- NA
# patient_data$CRP[patient_data$CRP == 250] <- NA # How to handle?

# Fix wrong age
age <- c(44, 25, 27, 25, 58, 61, 49, 60, 30, 39,
         41, 37, 43, 22, 18, 46, 27, 47, 28, 51)
for (pat in 1:20) {
```

```
  patient_data$Age[patient_data$Patient == pat] <- age[pat]
}
```

Inflammations

```
patient_number <- c()
for (rows in 1:length(patient_data$Patient)) {
  if (patient_data$Patient[rows] < 10) {
    patient_number[rows] <- paste0("C0",
                                   patient_data$Patient[rows])
  } else if (patient_data$Patient[rows] == 10) {
    patient_number[rows] <- paste0("C",
                                   patient_data$Patient[rows])
  } else if (patient_data$Patient[rows] > 10 & patient_data$Patient[rows] < 20) {
    patient_number[rows] <- paste0("I0",
                                   patient_data$Patient[rows] - 10)
  } else if (patient_data$Patient[rows] == 20) {
    patient_number[rows] <- paste0("I",
                                   patient_data$Patient[rows] - 10)
  }

}
patient_data$Patient <- patient_number

time_matrix <- matrix(c(1, 2, 5, 6, 9, 10, 13, 14, 17, 18,
                        5, 7, 29, 31, 53, 55, 77, 79, 101, 103),
                      nrow = 10,
                      ncol = 2)
patient_time <- c()
for (rows in 1:length(patient_data$Patient)) {
  patient_time[rows] <-
    time_matrix[time_matrix[ , 1] == patient_data$Time.Point[rows], 2]
}
patient_data$Time.Point <- patient_time
colnames(patient_data)[colnames(patient_data) == "Time.Point"] <- "Time"
patient_data$IL1ra.treatment[patient_data$IL1ra.treatment == "0"] <- "Control"
patient_data$IL1ra.treatment[patient_data$IL1ra.treatment == "1"] <- "Intervention"
patient_data$Infection[patient_data$Infection == "0"] <- "No"
patient_data$Infection[patient_data$Infection == "1"] <- "Yes"

inf_matrix <- matrix("-",
                     ncol = 4,
                     nrow = 80)
colnames(inf_matrix) <- c("Patient", "Detail", "Time", "Categories")
inf_data <- data.frame(inf_matrix)
inf_data$Patient <- rep(levels(factor(patient_number)), each = 4)
inf_data$Time <- rep(1, 80)
inf_data$Categories <- factor(rep(c("Age",
                                    "Sex",
                                    "SAE",
                                    "Infection type"),
```

```
                                   20),
                               levels = c("Age",
                                          "Sex",
                                          "SAE",
                                          "Infection type"))
inf_data$Detail <- rep("-", 80)
inf_data$Detail[3*4] <- "VAP"
inf_data$Detail[4*4] <- "VAP"
inf_data$Detail[5*4] <- "VAP"
inf_data$Detail[10*4] <- "IAS"
inf_data$Detail[12*4] <- "CI"
inf_data$Detail[15*4] <- "VAP"
inf_data$Detail[16*4] <- "VAP"
inf_data$Detail[19*4] <- "VAP"
inf_data$Detail[2*4-1] <- "Yes"
inf_data$Detail[4*4-1] <- "Yes"
inf_data$Detail[10*4-1] <- "Yes"
inf_data$Detail[12*4-1] <- "Yes"
inf_data$Detail[14*4-1] <- "Yes"
inf_data$Detail[15*4-1] <- "Yes"
inf_data$Detail[16*4-1] <- "Yes"
sex_vec <- c("F",
             "M",
             "F",
             "F",
             "F",
             "F",
             "M",
             "M",
             "M",
             "F",
             "F",
             "M",
             "M",
             "M",
             "F",
             "F",
             "M",
             "M",
             "F",
             "M")
inf_data$Detail[seq(from = 2, to = 80, by = 4)] <- sex_vec
age_vec <- c()
for (pat in 1:20) {
  age_vec[pat] <-
    patient_data$Age[which(patient_data$Patient ==
                             levels(factor(patient_number))[pat])[1]]
}
inf_data$Detail[seq(from = 1, to = 80, by = 4)] <- age_vec

ct_matrix <- matrix(NA,
                    ncol = 4,
                    nrow = 20)
```

```r
colnames(ct_matrix) <- c("Patient", "Time", "CT", "Deterioration")
ct_data <- data.frame(ct_matrix)
ct_data$Patient <- levels(factor(patient_number))
ct_data$Time <- rep("", 20)
for (pat in 1:20) {
  ct_data$CT[pat] <-
    patient_data$Stockholm_Eric[which(patient_data$Patient ==
                                    levels(factor(patient_number))[pat])[1]]
}
patient_data$CT_deterioration[patient_data$CT_deterioration == "0"] <- "No"
patient_data$CT_deterioration[patient_data$CT_deterioration == "1"] <- "Yes"
for (pat in 1:20) {
  ct_data$Deterioration[pat] <-
    patient_data$CT_deterioration[which(patient_data$Patient ==
                                    levels(factor(patient_number))[pat])[1]]
}

iss_matrix <- matrix(NA,
                     ncol = 3,
                     nrow = 20)
colnames(iss_matrix) <- c("Patient", "Time", "ISS")
iss_data <- data.frame(iss_matrix)
iss_data$Patient <- levels(factor(patient_number))
ct_data$Time <- rep("", 20)
for (pat in 1:20) {
  iss_data$ISS[pat] <-
    patient_data$ISS[which(patient_data$Patient ==
                           levels(factor(patient_number))[pat])[1]]
}

misspat_matrix <- matrix(NA,
                         ncol = 3,
                         nrow = 80)
colnames(misspat_matrix) <- c("Patient", "Compartment", "Missing")
misspat_data <- data.frame(misspat_matrix)
misspat_data$Patient <- rep(levels(factor(patient_number)), each = 4)
misspat_data$Compartment <- factor(rep(c("Arterial cytokines",
                               "Venous cytokines",
                               "WCC, CRP & Temp",
                               "Brain cytokines"),
                             20),
                         levels = c("Arterial cytokines",
                                    "Venous cytokines",
                                    "WCC, CRP & Temp",
                                    "Brain cytokines"))
for (pat in 1:20) {
  art <- sum(is.na(patient_data[patient_data$Patient == misspat_data$Patient[pat*4],
                                seq(from = 27,
                                    to = 151,
                                    by = 3)]))
  ven <- sum(is.na(patient_data[patient_data$Patient == misspat_data$Patient[pat*4],
                                c(seq(from = 28,
                                     to = 151,
```

```
                                     by = 3))]))
  wct <- sum(is.na(patient_data[patient_data$Patient == misspat_data$Patient[pat*4],
                                c(19:21)]))
  bra <- sum(is.na(patient_data[patient_data$Patient == misspat_data$Patient[pat*4],
                                seq(from = 26,
                                    to = 151,
                                    by = 3)]))
  total <- sum(patient_data$Patient == misspat_data$Patient[pat*4])

  misspat_data$Missing[pat*4-3] <- art/(total*42)
  misspat_data$Missing[pat*4-2] <- ven/(total*42)
  misspat_data$Missing[pat*4-1] <- wct/(total*3)
  misspat_data$Missing[pat*4] <- bra/(total*42)
}

misscyt_matrix <- matrix(NA,
                         ncol = 3,
                         nrow = 42*3)
colnames(misscyt_matrix) <- c("Cytokine", "Compartment", "Missing")
misscyt_data <- data.frame(misscyt_matrix)
cyt_names <- substr(colnames(patient_data)[seq(from = 26, to = 151, by = 3)], 4, 100)
cyt_names <- gsub("\\.", "-", cyt_names)
misscyt_data$Cytokine <- rep(cyt_names, each = 3)
misscyt_data$Compartment <- factor(rep(c("Arterial cytokines",
                                         "Venous cytokines",
                                         "Brain cytokines"),
                                       42),
                                   levels = c("Arterial cytokines",
                                              "Venous cytokines",
                                              "Brain cytokines"))
total <- dim(patient_data)[1]
for (cyt in 1:42) {
  art <- sum(is.na(patient_data[ , cyt*3+24]))
  ven <- sum(is.na(patient_data[ , cyt*3+25]))
  bra <- sum(is.na(patient_data[ , cyt*3+23]))

  misscyt_data$Missing[cyt*3-2] <- art/(total)
  misscyt_data$Missing[cyt*3-1] <- ven/(total)
  misscyt_data$Missing[cyt*3] <- bra/(total)
}
misscyt_data$Cytokine <- factor(misscyt_data$Cytokine,
                                levels = cyt_names)

library(ggplot2)
library(grid)
library(gridExtra)
library(gtable)
wcc_plot <- qplot(x = Time, y = WCC, data = patient_data, na.rm = TRUE) +
  facet_grid(cols = vars(Patient)) +
  theme(axis.text.x = element_blank(), axis.ticks = element_blank(),
        plot.title = element_text(hjust = 0.5), axis.title.x = element_blank()) +
  geom_smooth(method='lm', na.rm = TRUE)
crp_plot <- qplot(x = Time, y = CRP, data = patient_data, na.rm = TRUE) +
```

```r
  facet_grid(cols = vars(Patient)) +
  theme(axis.text.x = element_blank(), axis.ticks = element_blank(),
        axis.title.x = element_blank(), strip.background = element_blank(),
        strip.text = element_blank()) +
  geom_smooth(method='lm', na.rm = TRUE)
temp_plot <- qplot(x = Time, y = Temp, data = patient_data, na.rm = TRUE) +
  facet_grid(cols = vars(Patient)) +
  theme(axis.text.x = element_blank(), axis.ticks = element_blank(),
        axis.title.x = element_blank(), strip.background = element_blank(),
        strip.text = element_blank()) +
  geom_smooth(method='lm', na.rm = TRUE)
inf_plot <- ggplot(data = patient_data, aes(x = Time, y = Infection,
                                          color = Infection)) +
  scale_color_manual(values= c("Yes" = "red",
                               "No" = "green4")) +
  facet_grid(cols = vars(Patient)) +
  theme(axis.text.x = element_blank(), axis.ticks = element_blank(),
        legend.position = "none", strip.background = element_blank(),
        strip.text = element_blank()) +
  geom_count(size = 1)
inf_text <- ggplot(data = inf_data, aes(x = Time, y = Categories)) +
  geom_text(aes(label = Detail, color = Detail), size = 3) +
  scale_color_manual(values= c("F" = "orange1",
                               "M" = "darkslateblue",
                               "Yes" = "red",
                               "-" = "green4",
                               "?" = "red",
                               "VAP" = "red",
                               "IAS" = "red",
                               "CI" = "red",
                               "18" = "black",
                               "22" = "black",
                               "24" = "black",
                               "25" = "black",
                               "27" = "black",
                               "28" = "black",
                               "30" = "black",
                               "37" = "black",
                               "39" = "black",
                               "41" = "black",
                               "43" = "black",
                               "44" = "black",
                               "46" = "black",
                               "47" = "black",
                               "49" = "black",
                               "51" = "black",
                               "52" = "black",
                               "58" = "black",
                               "60" = "black",
                               "61" = "black")) +
  facet_grid(cols = vars(Patient)) +
  theme(axis.text.x = element_blank(), axis.ticks = element_blank(),
        axis.title.x = element_blank(), legend.position = "none",
```

```r
        panel.grid = element_blank()) +
  ylab("")
ct_plot <- ggplot(data = ct_data, aes(x = Time, y = CT, fill = Deterioration)) +
  geom_col(width = 0.3) +
  scale_fill_manual(values= c("Yes" = "red",
                              "No" = "green4")) +
  facet_grid(cols = vars(Patient)) +
  theme(axis.text.x = element_blank(), axis.ticks = element_blank(),
        axis.title.x = element_blank(), legend.key.size = unit(0.2, "cm"),
        legend.position = c(0.12, 0.9), legend.text = element_text(size = 7),
        legend.title = element_text(size = 7), legend.direction = "horizontal",
        strip.background = element_blank(), strip.text = element_blank()) +
  ylab("Stockholm CT")
iss_plot <- ggplot(data = iss_data, aes(x = Time, y = ISS)) +
  geom_col(width = 0.3) +
  facet_grid(cols = vars(Patient)) +
  theme(axis.text.x = element_blank(), axis.ticks = element_blank(),
        axis.title.x = element_blank(), strip.background = element_blank(),
        strip.text = element_blank())
misspat_plot <- ggplot(data = misspat_data, aes(x = Compartment, y = Missing,
                                          fill = Compartment)) +
  geom_col() +
  scale_fill_manual(values= c("Arterial cytokines" = "red",
                              "Venous cytokines" = "darkslateblue",
                              "WCC, CRP & Temp" = "orange1",
                              "Brain cytokines" = "green4")) +
  facet_grid(cols = vars(Patient)) +
  theme(axis.text.x = element_blank(), axis.ticks = element_blank(),
        axis.title.x = element_blank(), legend.key.size = unit(0.2, "cm"),
        legend.position = c(0.5, 0.8), legend.text = element_text(size = 7),
        legend.title = element_text(size = 7), legend.direction = "horizontal",
        strip.background = element_blank(), strip.text = element_blank())
misscyt_plot <- ggplot(data = misscyt_data, aes(x = Compartment, y = Missing,
                                          fill = Compartment)) +
  geom_col() +
  scale_fill_manual(values= c("Arterial cytokines" = "red",
                              "Venous cytokines" = "darkslateblue",
                              "Brain cytokines" = "green4")) +
  facet_grid(cols = vars(Cytokine)) +
  theme(axis.text.x = element_blank(), axis.ticks = element_blank(),
        axis.title.x = element_blank(), legend.key.size = unit(0.2, "cm"),
        legend.position = c(0.75, 0.9), legend.text = element_text(size = 7),
        legend.title = element_text(size = 7), legend.direction = "horizontal",
        strip.text = element_text(size = 7, angle = 90)) +
  ylim(0, 1)
g_wcc <- ggplotGrob(wcc_plot)
```

```
## `geom_smooth()` using formula 'y ~ x'
```

```r
g_crp <- ggplotGrob(crp_plot)
```

```
## `geom_smooth()` using formula 'y ~ x'
```

```r
g_temp <- ggplotGrob(temp_plot)
```

```
## `geom_smooth()` using formula 'y ~ x'
```

```
g_inf <- ggplotGrob(inf_plot)
g_text <- ggplotGrob(inf_text)
g_ct <- ggplotGrob(ct_plot)
g_iss <- ggplotGrob(iss_plot)
g_misspat <- ggplotGrob(misspat_plot)
g_misscyt <- ggplotGrob(misscyt_plot)
g <- rbind(g_wcc, g_crp, g_temp, g_inf, g_text, g_ct, g_iss, g_misspat,
           size = "first")
g$widths <- unit.pmax(g_wcc$widths, g_crp$widths, g_temp$widths, g_inf$widths,
                      g_text$widths, g_ct$widths, g_iss$widths, g_misspat$widths)
grid.newpage()
# grid.draw(g)
g <- grid.arrange(g, g_misscyt, nrow = 2, heights = c(5,1))
```

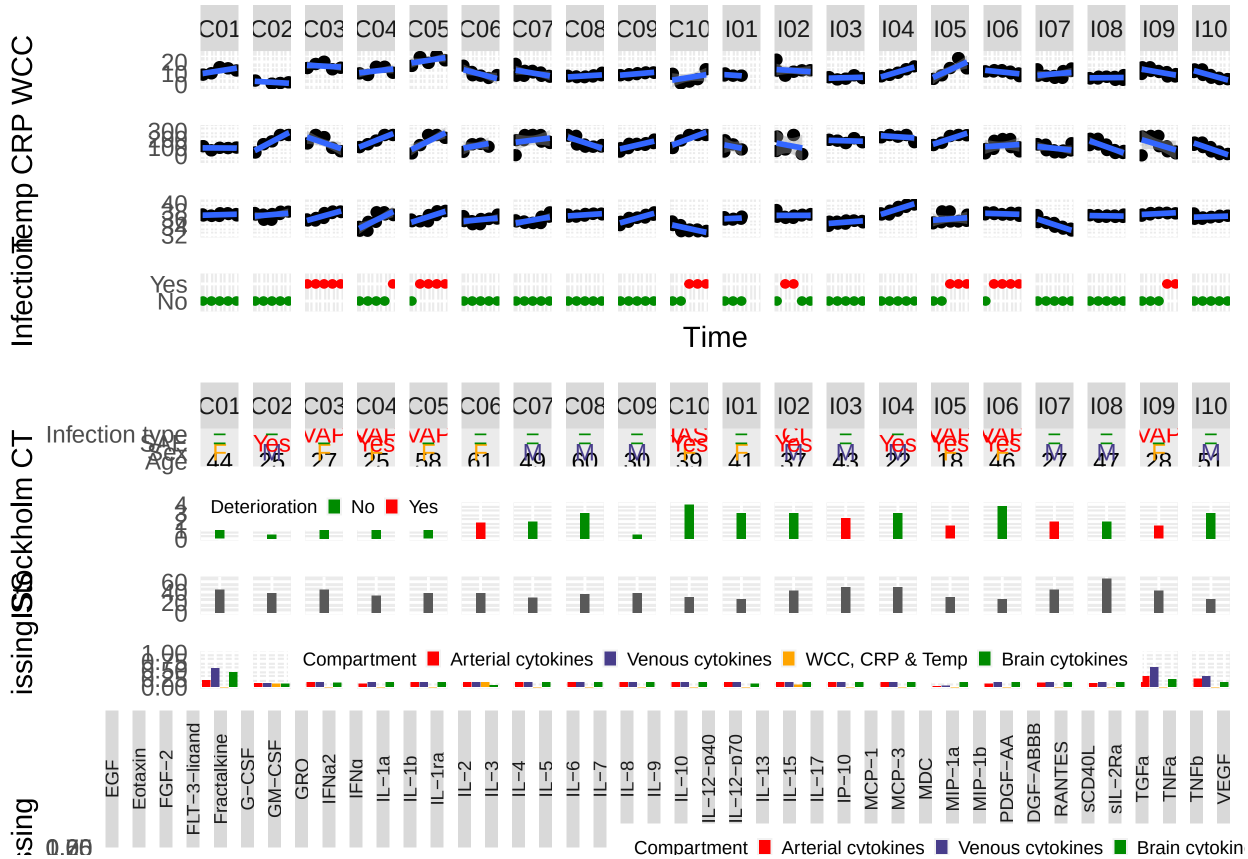


```
ggsave("table1_fig201113_update210204_update210304.pdf",
       plot = g,
       device = "pdf",
       width = 10,
       height = 12,
       units = "in")
```

T-test

```
t.test(ct_data[1:10,3], ct_data[11:20,3], var.equal=TRUE)
```

```
##
##  Two Sample t-test
##
## data:  ct_data[1:10, 3] and ct_data[11:20, 3]
## t = -1.2122, df = 18, p-value = 0.2411
## alternative hypothesis: true difference in means is not equal to 0
## 95 percent confidence interval:
##  -1.448578  0.388578
## sample estimates:
## mean of x mean of y
##      1.99      2.52
```

```
t.test(iss_data[1:10,3], iss_data[11:20,3], var.equal=TRUE)
```

```
##
##  Two Sample t-test
##
## data:  iss_data[1:10, 3] and iss_data[11:20, 3]
## t = -0.85408, df = 18, p-value = 0.4043
## alternative hypothesis: true difference in means is not equal to 0
## 95 percent confidence interval:
##  -13.147464   5.547464
## sample estimates:
## mean of x mean of y
##      37.0      40.8
```

Missing

```
total_rows <- dim(patient_data)[1]
arterial <- sum(is.na(patient_data[ , seq(from = 27,
                                          to = 151,
                                          by = 3)]))/(42*total_rows)
venous <- sum(is.na(patient_data[ , seq(from = 28,
                                        to = 151,
                                        by = 3)]))/(42*total_rows)
wct <- sum(is.na(patient_data[ , 19:21]))/(3*total_rows)
brain <- sum(is.na(patient_data[ , seq(from = 26,
                                       to = 151,
                                       by = 3)]))/(42*total_rows)
data.frame(arterial, venous, wct, brain)
```

```
##    arterial    venous         wct   brain
## 1 0.1846154 0.3087912 0.02051282 0.26337
```

```
miss_art <- misscyt_data[misscyt_data$Compartment == "Arterial cytokines", c(1,3)]
miss_art <- miss_art[order( - miss_art$Missing), ]
rownames(miss_art) <- order( - miss_art$Missing)
print(miss_art)
```

```
##        Cytokine     Missing
## 1          IL-4 0.830769231
## 2          IL-9 0.707692308
## 3   FLT-3-ligand 0.692307692
## 4         IL-13 0.610256410
## 5          IL-3 0.579487179
## 6          TNFb 0.564102564
## 7         IL-1b 0.482051282
## 8          IL-7 0.446153846
## 9          TGFa 0.389743590
## 10    IL-12-p40 0.343589744
## 11         IL-5 0.338461538
## 12        IL-1a 0.276923077
## 13          EGF 0.261538462
## 14       IL-1ra 0.164102564
## 15         IL-2 0.164102564
## 16        MCP-3 0.138461538
## 17        IFNa2 0.133333333
## 18      sIL-2Ra 0.107692308
## 19        IL-17 0.102564103
## 20       RANTES 0.071794872
## 21  Fractalkine 0.066666667
## 22        IL-15 0.041025641
## 23       MIP-1a 0.041025641
## 24       sCD40L 0.041025641
## 25       GM-CSF 0.035897436
## 26    IL-12-p70 0.035897436
## 27         IFNg 0.030769231
## 28         IL-6 0.015384615
## 29        G-CSF 0.010256410
## 30        IL-10 0.010256410
## 31         VEGF 0.010256410
## 32         IL-8 0.005128205
## 33    PDGF-ABBB 0.005128205
## 34      Eotaxin 0.000000000
## 35        FGF-2 0.000000000
## 36          GRO 0.000000000
## 37        IP-10 0.000000000
## 38        MCP-1 0.000000000
## 39          MDC 0.000000000
## 40       MIP-1b 0.000000000
## 41      PDGF-AA 0.000000000
## 42         TNFa 0.000000000
```

```
miss_brain <- misscyt_data[misscyt_data$Compartment == "Brain cytokines", c(1,3)]
miss_brain <- miss_brain[order( - miss_brain$Missing), ]
rownames(miss_brain) <- order( - miss_brain$Missing)
print(miss_brain)
```

```
##        Cytokine    Missing
## 1          IL-2 0.67692308
## 2           EGF 0.58461538
## 3          IL-3 0.55897436
## 4          TNFb 0.55384615
## 5         IL-17 0.54871795
```

```
## 6          IL-5 0.54358974
## 7         IL-1b 0.44102564
## 8          IL-4 0.42564103
## 9         IL-13 0.40512821
## 10         IL-9 0.40000000
## 11      sIL-2Ra 0.40000000
## 12         IFNg 0.33846154
## 13    PDGF-ABBB 0.32307692
## 14        IL-15 0.29230769
## 15        MCP-1 0.28205128
## 16    IL-12-p40 0.27692308
## 17        IFNa2 0.26153846
## 18         TGFa 0.26153846
## 19        IL-1a 0.25641026
## 20         IL-7 0.25641026
## 21    IL-12-p70 0.24102564
## 22         TNFa 0.24102564
## 23        IL-10 0.20512821
## 24          MDC 0.20512821
## 25         VEGF 0.18974359
## 26       sCD40L 0.17948718
## 27        FGF-2 0.16923077
## 28       RANTES 0.16923077
## 29       MIP-1b 0.14871795
## 30        MCP-3 0.14358974
## 31        G-CSF 0.13846154
## 32      PDGF-AA 0.11794872
## 33       MIP-1a 0.10256410
## 34          GRO 0.09743590
## 35      Eotaxin 0.08717949
## 36  Fractalkine 0.08717949
## 37       GM-CSF 0.08717949
## 38        IP-10 0.08717949
## 39         IL-8 0.08205128
## 40 FLT-3-ligand 0.06666667
## 41       IL-1ra 0.06666667
## 42         IL-6 0.06153846
```

The table

```
library(openxlsx)
pat_id <- levels(factor(patient_number))

wcc_med <- c()
crp_med <- c()
temp_med <- c()
inf_type <- c()
sae <- c()
sthlm_ct <- c()
iss <- c()

library(EnvStats)
```

```
##
## Attaching package: 'EnvStats'

## The following objects are masked from 'package:stats':
##
##     predict, predict.lm

## The following object is masked from 'package:base':
##
##     print.default
```

```
for (pat in 1:20) {
  pn <- pat_id[pat]
  wcc_stats <- summaryStats(patient_data$WCC[patient_data$Patient == pn],
                            na.rm = TRUE)
  wcc_med[pat] <- paste0(wcc_stats[4],
                         " (",
                         wcc_stats[5],
                         " - ",
                         wcc_stats[6],
                         ")"
                         )
  crp_stats <- summaryStats(patient_data$CRP[patient_data$Patient == pn],
                            na.rm = TRUE)
  crp_med[pat] <- paste0(crp_stats[4],
                         " (",
                         crp_stats[5],
                         " - ",
                         crp_stats[6],
                         ")"
                         )
  temp_stats <- summaryStats(patient_data$Temp[patient_data$Patient == pn],
                             na.rm = TRUE)
  temp_med[pat] <- paste0(round(temp_stats[4], digits = 2),
                          " (",
                          round(temp_stats[5], digits = 2),
                          " - ",
                          round(temp_stats[6], digits = 2),
                          ")"
                          )
  inf_type[pat] <- inf_data[pat*4, 2]
  sae[pat] <- inf_data[pat*4 - 1, 2]
  sthlm_ct[pat] <- as.character(ct_data$CT[pat])
  if (ct_data$Deterioration[pat] == "Yes") {
    sthlm_ct[pat] <- paste0(sthlm_ct[pat], "*")
  }
  iss[pat] <- iss_data$ISS[pat]
}
medians <- data.frame(pat_id, wcc_med, crp_med, temp_med,
                      inf_type, sae, sthlm_ct, iss)
colnames(medians) <- c("Patient ID", "White cell count", "C-reactive protein",
                       "Temperature", "Infection type", "Severe adverse event",
                       "Stockholm CT score", "Injury severity score")
write.xlsx(medians, "table1_median201113_update210204_update210304.xlsx")
```

Code for Table 2

Philipp

Revised: March 4, 2021

Contents

Load the data 1

Get median gradients for patient and cytokines 1

Load the data

```
library(openxlsx)
ip_data <- read.xlsx("../Dataset/Interpolated_data4.3.Adel201001.xlsx")
```

Get median gradients for patient and cytokines

```
library(reshape)
library(ggplot2)

median_gradient_matrix_AJ <- matrix(NA, nrow = 20, ncol = 42)
colnames(median_gradient_matrix_AJ) <-
  substr(colnames(ip_data)[seq(from = 8, to = 133, by = 3)], 4, 100)
rownames(median_gradient_matrix_AJ) <- rep(NA, 20)
rownames(median_gradient_matrix_AJ)[1:9] <- paste0("C0", 1:9)
rownames(median_gradient_matrix_AJ)[10] <- "C10"
rownames(median_gradient_matrix_AJ)[11:19] <- paste0("I0", 1:9)
rownames(median_gradient_matrix_AJ)[20] <- "I10"

posneg_matrix <- matrix(NA, nrow = 42, ncol = 1)
colnames(posneg_matrix) <- "AJ"
rownames(posneg_matrix) <- colnames(median_gradient_matrix_AJ)
summary_matrix <- posneg_matrix

for (cytokine in 1:42) {
  for (patient in 1:20) {
    grad_AJ <-
      ip_data[ip_data$Patient == patient, cytokine * 3 + 6] -
      ip_data[ip_data$Patient == patient, cytokine * 3 + 7]
    median_gradient_matrix_AJ[patient, cytokine] <- median(grad_AJ, na.rm = TRUE)
  }
  cytokine_median <- median(median_gradient_matrix_AJ[ , cytokine], na.rm = TRUE)
```

```
  summary_matrix[cytokine, "AJ"] <- cytokine_median
  posneg_matrix[cytokine, "AJ"] <-
    ifelse(cytokine_median > 0, "Positive", "Negative")
}

colSums(posneg_matrix == "Positive")
```

```
## AJ
## 28
```

```
colSums(posneg_matrix == "Negative")
```

```
## AJ
## 14
```

```
long_posneg_matrix <- melt(posneg_matrix)
colnames(long_posneg_matrix) <- c("Cytokine", "Direction", "Gradient")
p1 <- ggplot(long_posneg_matrix, aes(x = Direction, y = Cytokine)) +
  geom_tile(aes(fill = Gradient))
print(p1)
```

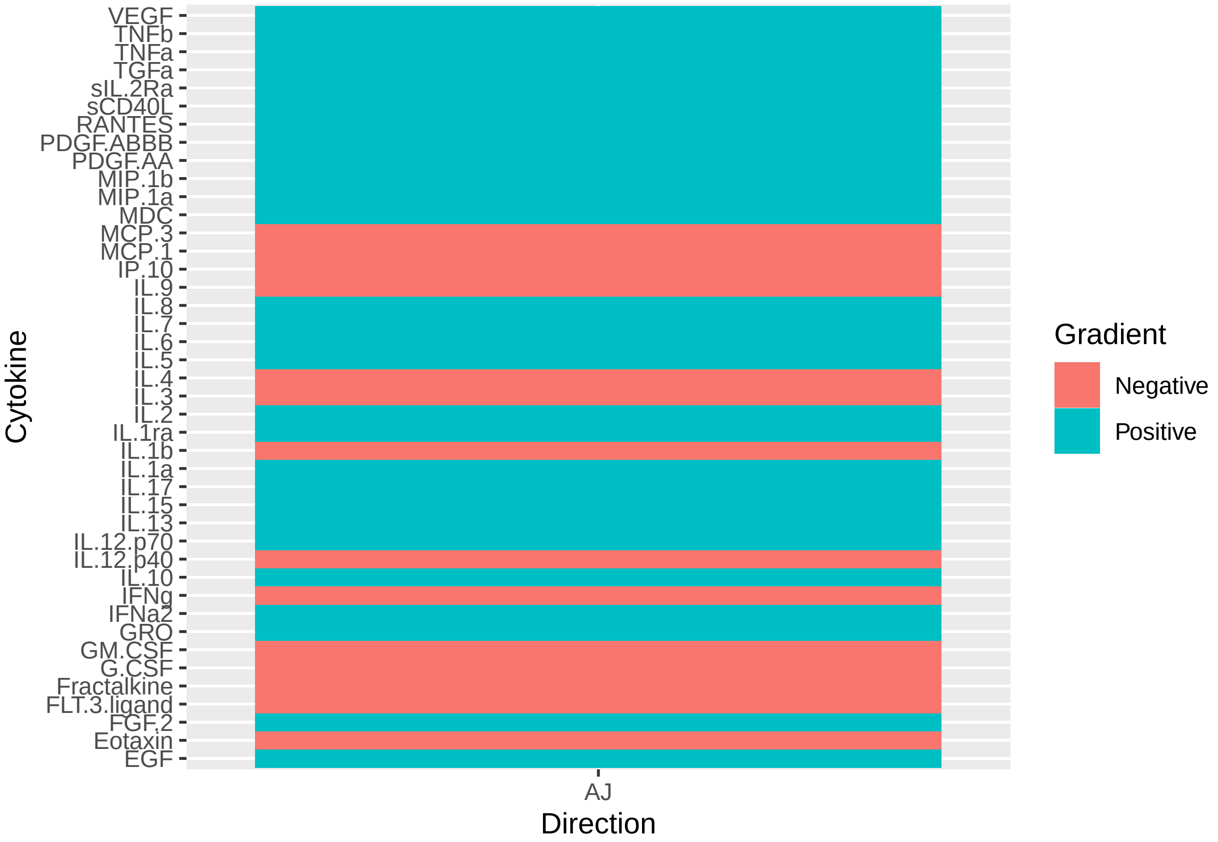


```
ggsave("mediangrad_201116.pdf",
       plot = p1,
       device = "pdf",
       width = 3,
       height = 12,
       units = "in")
```

```
summary_matrix <- data.frame(round(summary_matrix, digits = 3))
write.xlsx(cbind(Cytokine = colnames(median_gradient_matrix_AJ),
                 summary_matrix), "mediansummary_201116.xlsx")
write.xlsx(cbind(Cytokine = rownames(median_gradient_matrix_AJ),
                 median_gradient_matrix_AJ), "mediangrad_201116.xlsx")
```

Code for Additional File 6A

Philipp

Revised: May 11, 2021

Contents

Load data 1

Run nlme per patient and cytokine 1

Load data

```
library(openxlsx)
ip_data <- read.xlsx("../Dataset/Interpolated_data4.3.Adel201001.xlsx")

# Fix wrong age
age <- c(44, 25, 27, 25, 58, 61, 49, 60, 30, 39,
         41, 37, 43, 22, 18, 46, 27, 47, 28, 51)
for (pat in 1:20) {
  ip_data$Age[ip_data$Patient == pat] <- age[pat]
}
```

Run nlme per patient and cytokine

```
library(nlme)
library(ggplot2)
library(plyr)
library(pheatmap)
library(gplots)
```

```
##
## Attaching package: 'gplots'

## The following object is masked from 'package:stats':
##
##     lowess
```

```
library(made4)
```

```
## Loading required package: RColorBrewer

## Loading required package: scatterplot3d

## Loading required package: Biobase
```

```
## Loading required package: BiocGenerics
## Loading required package: parallel
##
## Attaching package: 'BiocGenerics'
## The following objects are masked from 'package:parallel':
##
##     clusterApply, clusterApplyLB, clusterCall, clusterEvalQ,
##     clusterExport, clusterMap, parApply, parCapply, parLapply,
##     parLapplyLB, parRapply, parSapply, parSapplyLB
## The following objects are masked from 'package:stats':
##
##     IQR, mad, sd, var, xtabs
## The following objects are masked from 'package:base':
##
##     anyDuplicated, append, as.data.frame, basename, cbind, colnames,
##     dirname, do.call, duplicated, eval, evalq, Filter, Find, get, grep,
##     grepl, intersect, is.unsorted, lapply, Map, mapply, match, mget,
##     order, paste, pmax, pmax.int, pmin, pmin.int, Position, rank,
##     rbind, Reduce, rownames, sapply, setdiff, sort, table, tapply,
##     union, unique, unsplit, which.max, which.min
## Welcome to Bioconductor
##
##     Vignettes contain introductory material; view with
##     'browseVignettes()'. To cite Bioconductor, see
##     'citation("Biobase")', and for packages 'citation("pkgname")'.
## Loading required package: SummarizedExperiment
## Loading required package: MatrixGenerics
## Loading required package: matrixStats
##
## Attaching package: 'matrixStats'
## The following objects are masked from 'package:Biobase':
##
##     anyMissing, rowMedians
## The following object is masked from 'package:plyr':
##
##     count
##
## Attaching package: 'MatrixGenerics'
## The following objects are masked from 'package:matrixStats':
##
##     colAlls, colAnyNAs, colAnys, colAvgsPerRowSet, colCollapse,
##     colCounts, colCummaxs, colCummins, colCumprods, colCumsums,
##     colDiffs, colIQRDiffs, colIQRs, colLogSumExps, colMadDiffs,
##     colMads, colMaxs, colMeans2, colMedians, colMins, colOrderStats,
##     colProds, colQuantiles, colRanges, colRanks, colSdDiffs, colSds,
##     colSums2, colTabulates, colVarDiffs, colVars, colWeightedMads,
##     colWeightedMeans, colWeightedMedians, colWeightedSds,
```

```
##     colWeightedVars, rowAlls, rowAnyNAs, rowAnys, rowAvgsPerColSet,
##     rowCollapse, rowCounts, rowCummaxs, rowCummins, rowCumprods,
##     rowCumsums, rowDiffs, rowIQRDiffs, rowIQRs, rowLogSumExps,
##     rowMadDiffs, rowMads, rowMaxs, rowMeans2, rowMedians, rowMins,
##     rowOrderStats, rowProds, rowQuantiles, rowRanges, rowRanks,
##     rowSdDiffs, rowSds, rowSums2, rowTabulates, rowVarDiffs, rowVars,
##     rowWeightedMads, rowWeightedMeans, rowWeightedMedians,
##     rowWeightedSds, rowWeightedVars

## The following object is masked from 'package:Biobase':
##
##     rowMedians

## Loading required package: GenomicRanges

## Loading required package: stats4

## Loading required package: S4Vectors

##
## Attaching package: 'S4Vectors'

## The following object is masked from 'package:gplots':
##
##     space

## The following object is masked from 'package:plyr':
##
##     rename

## The following objects are masked from 'package:base':
##
##     expand.grid, I, unname

## Loading required package: IRanges

##
## Attaching package: 'IRanges'

## The following object is masked from 'package:plyr':
##
##     desc

## The following object is masked from 'package:nlme':
##
##     collapse

## Loading required package: GenomeInfoDb
```

```
library(car)
```

```
## Loading required package: carData
```

```
time <- ip_data$Time
wcc <- ip_data$WCC
infection <- ip_data$Infection
rhil1ra <- ip_data$rhIL1ra
iss <- ip_data$ISS
pyruvate <- ip_data$Pyruvate
lactate <- ip_data$Lactate
glycerol <- ip_data$Glycerol
glutamate <- ip_data$Glutamate
```

```r
glucose <- ip_data$Glucose
lpr <- ip_data$LP_ratio
ct <- ip_data$Stockholm_CT
age <- ip_data$Age
sex_male <- ip_data$Sex_male
sex_female <- sex_male
sex_female[sex_male == 0] <- 1
sex_female[sex_male == 1] <- 0
gcs_m <- ip_data$GCS_m
patient <- ip_data$Patient

coeff_matrix <- matrix(NA,
                      nrow = 42,
                      ncol = 5)
colnames(coeff_matrix) <- c("Sex_female",
                           "Age",
                           "GCS_m",
                           "ISS",
                           "Stockholm_CT")
rownames(coeff_matrix) <- colnames(ip_data)[seq(from = 8,
                                               to = 133,
                                               by = 3)]
anova_matrix <- coeff_matrix

sd_matrix <- matrix(NA,
                   ncol = 1,
                   nrow = 42)
colnames(sd_matrix) <- c("MD_cytokine")
rownames(sd_matrix) <- substr(rownames(coeff_matrix), 4, 100)
sd_time <- sd(time, na.rm = TRUE)
sd_wcc <- sd(wcc, na.rm = TRUE)
sd_infection <- sd(infection, na.rm = TRUE)
sd_rhil1ra <- sd(rhil1ra, na.rm = TRUE)
sd_iss <- sd(iss, na.rm = TRUE)
sd_pyruvate <- sd(pyruvate, na.rm = TRUE)
sd_lactate <- sd(lactate, na.rm = TRUE)
sd_glycerol <- sd(glycerol, na.rm = TRUE)
sd_glutamate <- sd(glutamate, na.rm = TRUE)
sd_glucose <- sd(glucose, na.rm = TRUE)
sd_lpr <- sd(lpr, na.rm = TRUE)
sd_ct <- sd(ct, na.rm = TRUE)
sd_age <- sd(age, na.rm = TRUE)
sd_sex_female <- sd(sex_female, na.rm = TRUE)
sd_gcs_m <- sd(gcs_m, na.rm = TRUE)
sd_vector <- c(sd_sex_female,
               sd_age,
               sd_gcs_m,
               sd_iss,
               sd_ct)

abl_data <- matrix(NA,
                  ncol = 3,
                  nrow = 42)
```

```r
colnames(abl_data) <- c("AIC",
                        "BIC",
                        "loglik")

library(predictmeans)
```

```
## Loading required package: lme4

## Loading required package: Matrix

##
## Attaching package: 'Matrix'

## The following object is masked from 'package:S4Vectors':
##
##     expand

##
## Attaching package: 'lme4'

## The following object is masked from 'package:nlme':
##
##     lmList

## Loading required package: lmeInfo

##
## Attaching package: 'predictmeans'

## The following object is masked from 'package:gplots':
##
##     residplot
```

```r
for (cytokine in 1:42) {
  md_cytokine <- log(ip_data[ , cytokine * 3 + 5])

  sd_matrix[cytokine, 1] <- sd(md_cytokine, na.rm = TRUE)

  lme_fit <- tryCatch({
    lme(md_cytokine ~ sex_female + age + gcs_m + iss + ct,
        random = ~ 1 | patient,
        na.action = na.omit)
  }, error = function(e) {
    NA
  }
  )

  if (class(lme_fit) == "lme") {
    coeff_matrix[cytokine, ] <- lme_fit$coefficients$fixed[-1]
    anova_matrix[cytokine, ] <- anova(lme_fit)$`p-value`[2:(dim(anova_matrix)[2]+1)]
  } else {
    print(paste0("Error: ",
                 colnames(ip_data)[cytokine * 3 + 5],
                 " ~ ",
                 colnames(ip_data)[cytokine * 3 + 6],
                 " + sex_female + age + gcs_m + iss + ct"))
  }
}
```

```
yield_matrix <- matrix(NA,
                       nrow = 42,
                       ncol = 5)
colnames(yield_matrix) <- colnames(coeff_matrix)
rownames(yield_matrix) <- rownames(sd_matrix)
for (cytokine in 1:42) {
  sd_md.cyt <- sd_matrix[cytokine, 1]
  for (variable in 1:5) {
    yield_matrix[cytokine, variable] <-
      coeff_matrix[cytokine, variable] * sd_vector[variable] / sd_md.cyt
  }
}

# coeff_matrix.scaled <- scale(coeff_matrix)

library(massageR)
library(gplots)

anova_matrix <- cbind(rownames(anova_matrix), anova_matrix)
sign_matrix <- anova_matrix < 0.05
colnames(anova_matrix)[1] <- "Cytokine"
wb <- createWorkbook()
addWorksheet(wb, "Sheet 1")
writeData(wb = wb, sheet = "Sheet 1", x = anova_matrix)
color <- createStyle(fgFill = "yellow")
for (cols in 1:dim(sign_matrix)[2]) {
  addStyle(wb = wb, sheet = "Sheet 1", style = color,
           rows = which(sign_matrix[ , cols]) + 1, cols = cols)
}
saveWorkbook(wb = wb, file = "pvals_fig2a_201008.revised210204.xlsx", overwrite = TRUE)
labs <- matrix("", dim(na.omit(sign_matrix))[1], dim(sign_matrix)[2] - 1)
labs[na.omit(sign_matrix[ , 2:dim(sign_matrix)[2]])] <- "*"

x <- as.matrix(na.omit(yield_matrix))
colnames(x) <- c("Female sex", "Age", "GCSm", "ISS", "Sthlm CT")
z <- heat.clust(x,
                scaledim="none",
                zlim=c(-0.5,0.5),
                zlim_select = c("dend","outdata"),
                reorder=c("column","row"),
                distfun  = function(x) as.dist(1-cor(t(x))),
                hclustfun= function(x) hclust(x, method="average"))

tiff("nlme_heatmap201008admission.revised210108.revised210204.tiff",
     width = 1000, height = 1000,
     pointsize = 24)
# pdf("nlme_heatmap201008admission.pdf")
heatmap.2(z$data,
          Rowv=FALSE,
          Colv=FALSE,
          trace="none",
          scale="none",
          dendrogram = "none",
```

```
          symbreaks = TRUE,
          col=rev(colorRampPalette(brewer.pal(10, "RdBu"))(256)),
          cexRow = 0.8,
          cexCol = 0.8,
          cellnote = labs, notecol = "cyan", notecex = 2
          )
dev.off()
```

```
## pdf
##   2
```

```
coeff_matrix.na <- rownames(coeff_matrix[rowSums(is.na(coeff_matrix)) > 0, ])
print(paste("No linear model was found for",
            coeff_matrix.na))
```

```
## [1] "No linear model was found for "
```

```
largest <- apply(coeff_matrix, 2, max, na.rm = TRUE)
which_largest <- apply(coeff_matrix, 2, which.max)
names_largest <- rownames(coeff_matrix)[which_largest]
equivalent_largest <- largest["Sex_female"]/largest
specific_equivalent_largest <-
  largest["Sex_female"] /
  coeff_matrix[names_largest[colnames(coeff_matrix) == "Sex_female"], ]
smallest <- apply(coeff_matrix, 2, min, na.rm = TRUE)
which_smallest <- apply(coeff_matrix, 2, which.min)
names_smallest <- rownames(coeff_matrix)[which_smallest]
equivalent_smallest <- smallest["Sex_female"]/smallest
specific_equivalent_smallest <-
  smallest["Sex_female"] /
  coeff_matrix[names_smallest[colnames(coeff_matrix) == "Sex_female"], ]
na_row <- rep(NA, length(largest))
coeff_matrix <- rbind(coeff_matrix,
                      na_row,
                      names_largest,
                      largest,
                      equivalent_largest,
                      specific_equivalent_largest,
                      na_row,
                      names_smallest,
                      smallest,
                      equivalent_smallest,
                      specific_equivalent_smallest)
rownames(coeff_matrix)[(nrow(coeff_matrix) - 9) : nrow(coeff_matrix)] <-
  c(NA, "Largest cytokine", "Coefficient", "Female sex equivalent",
    paste(coeff_matrix["names_largest", "Sex_female"], "female sex equivalent"),
    NA, "Smallest cytokine", "Coefficient", "Female sex equivalent",
    paste(coeff_matrix["names_smallest", "Sex_female"], "female sex equivalent"))
coeff_matrix <- cbind(Cytokine = rownames(coeff_matrix), coeff_matrix)
colnames(coeff_matrix) <- c("Cytokine", "Female sex", "Age", "GCSm", "ISS",
                            "Stockholm CT Score")

write.xlsx(coeff_matrix, "coefficients_fig2a_201008.revised210204.xlsx",
           overwrite = TRUE)
```

Code for Additional File 6B

Philipp

Revised: May 11, 2021

Contents

Load data 1

Run nlme per patient and cytokine 1

Load data

```
library(openxlsx)
ip_data <- read.xlsx("../Dataset/Interpolated_data4.3.Adel201001.xlsx")

# Fix wrong age
age <- c(44, 25, 27, 25, 58, 61, 49, 60, 30, 39,
         41, 37, 43, 22, 18, 46, 27, 47, 28, 51)
for (pat in 1:20) {
  ip_data$Age[ip_data$Patient == pat] <- age[pat]
}
```

Run nlme per patient and cytokine

```
library(nlme)
library(ggplot2)
library(plyr)
library(pheatmap)
library(gplots)
```

```
##
## Attaching package: 'gplots'

## The following object is masked from 'package:stats':
##
##     lowess
```

```
library(made4)
```

```
## Loading required package: RColorBrewer

## Loading required package: scatterplot3d

## Loading required package: Biobase
```

```
## Loading required package: BiocGenerics

## Loading required package: parallel

##
## Attaching package: 'BiocGenerics'

## The following objects are masked from 'package:parallel':
##
##     clusterApply, clusterApplyLB, clusterCall, clusterEvalQ,
##     clusterExport, clusterMap, parApply, parCapply, parLapply,
##     parLapplyLB, parRapply, parSapply, parSapplyLB

## The following objects are masked from 'package:stats':
##
##     IQR, mad, sd, var, xtabs

## The following objects are masked from 'package:base':
##
##     anyDuplicated, append, as.data.frame, basename, cbind, colnames,
##     dirname, do.call, duplicated, eval, evalq, Filter, Find, get, grep,
##     grepl, intersect, is.unsorted, lapply, Map, mapply, match, mget,
##     order, paste, pmax, pmax.int, pmin, pmin.int, Position, rank,
##     rbind, Reduce, rownames, sapply, setdiff, sort, table, tapply,
##     union, unique, unsplit, which.max, which.min

## Welcome to Bioconductor
##
##     Vignettes contain introductory material; view with
##     'browseVignettes()'. To cite Bioconductor, see
##     'citation("Biobase")', and for packages 'citation("pkgname")'.

## Loading required package: SummarizedExperiment

## Loading required package: MatrixGenerics

## Loading required package: matrixStats

##
## Attaching package: 'matrixStats'

## The following objects are masked from 'package:Biobase':
##
##     anyMissing, rowMedians

## The following object is masked from 'package:plyr':
##
##     count

##
## Attaching package: 'MatrixGenerics'

## The following objects are masked from 'package:matrixStats':
##
##     colAlls, colAnyNAs, colAnys, colAvgsPerRowSet, colCollapse,
##     colCounts, colCummaxs, colCummins, colCumprods, colCumsums,
##     colDiffs, colIQRDiffs, colIQRs, colLogSumExps, colMadDiffs,
##     colMads, colMaxs, colMeans2, colMedians, colMins, colOrderStats,
##     colProds, colQuantiles, colRanges, colRanks, colSdDiffs, colSds,
##     colSums2, colTabulates, colVarDiffs, colVars, colWeightedMads,
##     colWeightedMeans, colWeightedMedians, colWeightedSds,
```

```
##     colWeightedVars, rowAlls, rowAnyNAs, rowAnys, rowAvgsPerColSet,
##     rowCollapse, rowCounts, rowCummaxs, rowCummins, rowCumprods,
##     rowCumsums, rowDiffs, rowIQRDiffs, rowIQRs, rowLogSumExps,
##     rowMadDiffs, rowMads, rowMaxs, rowMeans2, rowMedians, rowMins,
##     rowOrderStats, rowProds, rowQuantiles, rowRanges, rowRanks,
##     rowSdDiffs, rowSds, rowSums2, rowTabulates, rowVarDiffs, rowVars,
##     rowWeightedMads, rowWeightedMeans, rowWeightedMedians,
##     rowWeightedSds, rowWeightedVars

## The following object is masked from 'package:Biobase':
##
##     rowMedians

## Loading required package: GenomicRanges

## Loading required package: stats4

## Loading required package: S4Vectors

##
## Attaching package: 'S4Vectors'

## The following object is masked from 'package:gplots':
##
##     space

## The following object is masked from 'package:plyr':
##
##     rename

## The following objects are masked from 'package:base':
##
##     expand.grid, I, unname

## Loading required package: IRanges

##
## Attaching package: 'IRanges'

## The following object is masked from 'package:plyr':
##
##     desc

## The following object is masked from 'package:nlme':
##
##     collapse

## Loading required package: GenomeInfoDb
```

```
library(car)
```

```
## Loading required package: carData
```

```
time <- ip_data$Time
wcc <- ip_data$WCC
infection <- ip_data$Infection
rhil1ra <- ip_data$rhIL1ra
iss <- ip_data$ISS
pyruvate <- ip_data$Pyruvate
lactate <- ip_data$Lactate
glycerol <- ip_data$Glycerol
glutamate <- ip_data$Glutamate
```

```
glucose <- ip_data$Glucose
lpr <- ip_data$LP_ratio
ct <- ip_data$Stockholm_CT
age <- ip_data$Age
sex_male <- ip_data$Sex_male
sex_female <- sex_male
sex_female[sex_male == 0] <- 1
sex_female[sex_male == 1] <- 0
gcs_m <- ip_data$GCS_m
patient <- ip_data$Patient

coeff_matrix <- matrix(NA,
                       nrow = 42,
                       ncol = 5)
colnames(coeff_matrix) <- c("Sex_female",
                            "Age",
                            "GCS_m",
                            "ISS",
                            "Stockholm_CT")
rownames(coeff_matrix) <- colnames(ip_data)[seq(from = 9,
                                                to = 133,
                                                by = 3)]
anova_matrix <- coeff_matrix

sd_matrix <- matrix(NA,
                    ncol = 1,
                    nrow = 42)
colnames(sd_matrix) <- c("A_cytokine")
rownames(sd_matrix) <- substr(rownames(coeff_matrix), 3, 100)
sd_time <- sd(time, na.rm = TRUE)
sd_wcc <- sd(wcc, na.rm = TRUE)
sd_infection <- sd(infection, na.rm = TRUE)
sd_rhil1ra <- sd(rhil1ra, na.rm = TRUE)
sd_iss <- sd(iss, na.rm = TRUE)
sd_pyruvate <- sd(pyruvate, na.rm = TRUE)
sd_lactate <- sd(lactate, na.rm = TRUE)
sd_glycerol <- sd(glycerol, na.rm = TRUE)
sd_glutamate <- sd(glutamate, na.rm = TRUE)
sd_glucose <- sd(glucose, na.rm = TRUE)
sd_lpr <- sd(lpr, na.rm = TRUE)
sd_ct <- sd(ct, na.rm = TRUE)
sd_age <- sd(age, na.rm = TRUE)
sd_sex_female <- sd(sex_female, na.rm = TRUE)
sd_gcs_m <- sd(gcs_m, na.rm = TRUE)
sd_vector <- c(sd_sex_female,
               sd_age,
               sd_gcs_m,
               sd_iss,
               sd_ct)

abl_data <- matrix(NA,
                   ncol = 3,
                   nrow = 42)
```

```
colnames(abl_data) <- c("AIC",
                        "BIC",
                        "loglik")

library(predictmeans)
```

```
## Loading required package: lme4

## Loading required package: Matrix

##
## Attaching package: 'Matrix'

## The following object is masked from 'package:S4Vectors':
##
##     expand

##
## Attaching package: 'lme4'

## The following object is masked from 'package:nlme':
##
##     lmList

## Loading required package: lmeInfo

##
## Attaching package: 'predictmeans'

## The following object is masked from 'package:gplots':
##
##     residplot
```

```
for (cytokine in 1:42) {
  a_cytokine <- log(ip_data[ , cytokine * 3 + 6])

  sd_matrix[cytokine, 1] <- sd(a_cytokine, na.rm = TRUE)

  lme_fit <- tryCatch({
    lme(a_cytokine ~ sex_female + age + gcs_m + iss + ct,
        random = ~ 1 | patient,
        na.action = na.omit)
  }, error = function(e) {
    NA
  }
  )

  if (class(lme_fit) == "lme") {
    coeff_matrix[cytokine, ] <- lme_fit$coefficients$fixed[-1]
    anova_matrix[cytokine, ] <- anova(lme_fit)$`p-value`[2:(dim(anova_matrix)[2]+1)]
  } else {
    print(paste0("Error: ",
                 colnames(ip_data)[cytokine * 3 + 6],
                 " ~ ",
                 " + sex_female + age + gcs_m + iss + ct"))
  }
}
yield_matrix <- matrix(NA,
```

```
                        nrow = 42,
                        ncol = 5)
colnames(yield_matrix) <- colnames(coeff_matrix)
rownames(yield_matrix) <- rownames(sd_matrix)
for (cytokine in 1:42) {
  sd_a.cyt <- sd_matrix[cytokine, 1]
  for (variable in 1:5) {
    yield_matrix[cytokine, variable] <-
      coeff_matrix[cytokine, variable] * sd_vector[variable] / sd_a.cyt
  }
}

# coeff_matrix.scaled <- scale(coeff_matrix)

library(massageR)
library(gplots)

anova_matrix <- cbind(rownames(anova_matrix), anova_matrix)
sign_matrix <- anova_matrix < 0.05
colnames(anova_matrix)[1] <- "Cytokine"
wb <- createWorkbook()
addWorksheet(wb, "Sheet 1")
writeData(wb = wb, sheet = "Sheet 1", x = anova_matrix)
color <- createStyle(fgFill = "yellow")
for (cols in 1:dim(sign_matrix)[2]) {
  addStyle(wb = wb, sheet = "Sheet 1", style = color,
           rows = which(sign_matrix[ , cols]) + 1, cols = cols)
}
saveWorkbook(wb = wb, file = "pvals_fig2b_201009.revised210204.xlsx", overwrite = TRUE)
labs <- matrix("", dim(na.omit(sign_matrix))[1], dim(sign_matrix)[2] - 1)
labs[na.omit(sign_matrix[ , 2:dim(sign_matrix)[2]])] <- "*"

x <- as.matrix(na.omit(yield_matrix))
colnames(x) <- c("Female sex", "Age", "GCSm", "ISS", "Sthlm CT")
z <- heat.clust(x,
                scaledim="none",
                zlim=c(-0.5,0.5),
                zlim_select = c("dend","outdata"),
                reorder=c("column","row"),
                distfun  = function(x) as.dist(1-cor(t(x))),
                hclustfun= function(x) hclust(x, method="average"))

tiff("nlme_heatmap201009admission.arterial.revised210108.revised210204.tiff",
     width = 1000, height = 1000,
     pointsize = 24)
# pdf("nlme_heatmap201009admission.arterial.pdf")
heatmap.2(z$data,
          Rowv=FALSE,
          Colv=FALSE,
          trace="none",
          scale="none",
          dendrogram = "column",
          symbreaks = TRUE,
```

```r
          col=rev(colorRampPalette(brewer.pal(10, "RdBu"))(256)),
          cexRow = 0.8,
          cexCol = 0.8,
          cellnote = labs, notecol = "cyan", notecex = 2
          )
```

```
## Warning in heatmap.2(z$data, Rowv = FALSE, Colv = FALSE, trace = "none", :
## Discrepancy: Colv is FALSE, while dendrogram is `column'. Omitting column
## dendogram.
```

```r
dev.off()
```

```
## pdf
##   2
```

```r
coeff_matrix.na <- rownames(coeff_matrix[rowSums(is.na(coeff_matrix)) > 0, ])
print(paste("No linear model was found for",
            coeff_matrix.na))
```

```
## [1] "No linear model was found for "
```

```r
largest <- apply(coeff_matrix, 2, max, na.rm = TRUE)
which_largest <- apply(coeff_matrix, 2, which.max)
names_largest <- rownames(coeff_matrix)[which_largest]
equivalent_largest <- largest["Sex_female"]/largest
specific_equivalent_largest <-
  largest["Sex_female"] /
  coeff_matrix[names_largest[colnames(coeff_matrix) == "Sex_female"], ]
smallest <- apply(coeff_matrix, 2, min, na.rm = TRUE)
which_smallest <- apply(coeff_matrix, 2, which.min)
names_smallest <- rownames(coeff_matrix)[which_smallest]
equivalent_smallest <- smallest["Sex_female"]/smallest
specific_equivalent_smallest <-
  smallest["Sex_female"] /
  coeff_matrix[names_smallest[colnames(coeff_matrix) == "Sex_female"], ]
na_row <- rep(NA, length(largest))
coeff_matrix <- rbind(coeff_matrix,
                      na_row,
                      names_largest,
                      largest,
                      equivalent_largest,
                      specific_equivalent_largest,
                      na_row,
                      names_smallest,
                      smallest,
                      equivalent_smallest,
                      specific_equivalent_smallest)
rownames(coeff_matrix)[(nrow(coeff_matrix) - 9) : nrow(coeff_matrix)] <-
  c(NA, "Largest cytokine", "Coefficient", "Female sex equivalent",
    paste(coeff_matrix["names_largest", "Sex_female"], "female sex equivalent"),
    NA, "Smallest cytokine", "Coefficient", "Female sex equivalent",
    paste(coeff_matrix["names_smallest", "Sex_female"], "female sex equivalent"))
coeff_matrix <- cbind(Cytokine = rownames(coeff_matrix), coeff_matrix)
colnames(coeff_matrix) <- c("Cytokine", "Female sex", "Age", "GCSm", "ISS",
                            "Stockholm CT Score")
```

```
write.xlsx(coeff_matrix, "coefficients_fig2b_201009.revised210204.xlsx",
           overwrite = TRUE)
```

Code for Additional file 7

Philipp

Revised: March 18, 2021

Contents

Load the data 1

Do a PCA for the inflammatory markers 1

Load the data

```
library(openxlsx)
library(tidyr)
library(dplyr)
```

```
##
## Attaching package: 'dplyr'

## The following objects are masked from 'package:stats':
##
##     filter, lag

## The following objects are masked from 'package:base':
##
##     intersect, setdiff, setequal, union
```

```
ip_data <- read.xlsx("../../Dataset/Interpolated_data4.3.Adel201001.xlsx")
small_data <- ip_data %>%
  select(c(WCC, CRP, Temp, Infection, rhIL1ra) | starts_with("A_")) %>%
  drop_na(!starts_with("A_"))
small_data[is.na(small_data)] <- 0
small_data$Infection <- ifelse(small_data$Infection == 1, "Yes", "No")
small_data$rhIL1ra <- ifelse(small_data$rhIL1ra == 1, "Yes", "No")
```

Do a PCA for the inflammatory markers

```
library(FactoMineR)
library(factoextra)
```

```
## Loading required package: ggplot2

## Welcome! Want to learn more? See two factoextra-related books at https://goo.gl/ve3WBa
```

```r
library(ggplot2)
library(ggrepel)

pca.infl <- PCA(small_data, graph = TRUE, quanti.sup = 1:3, quali.sup = 4:5)
```

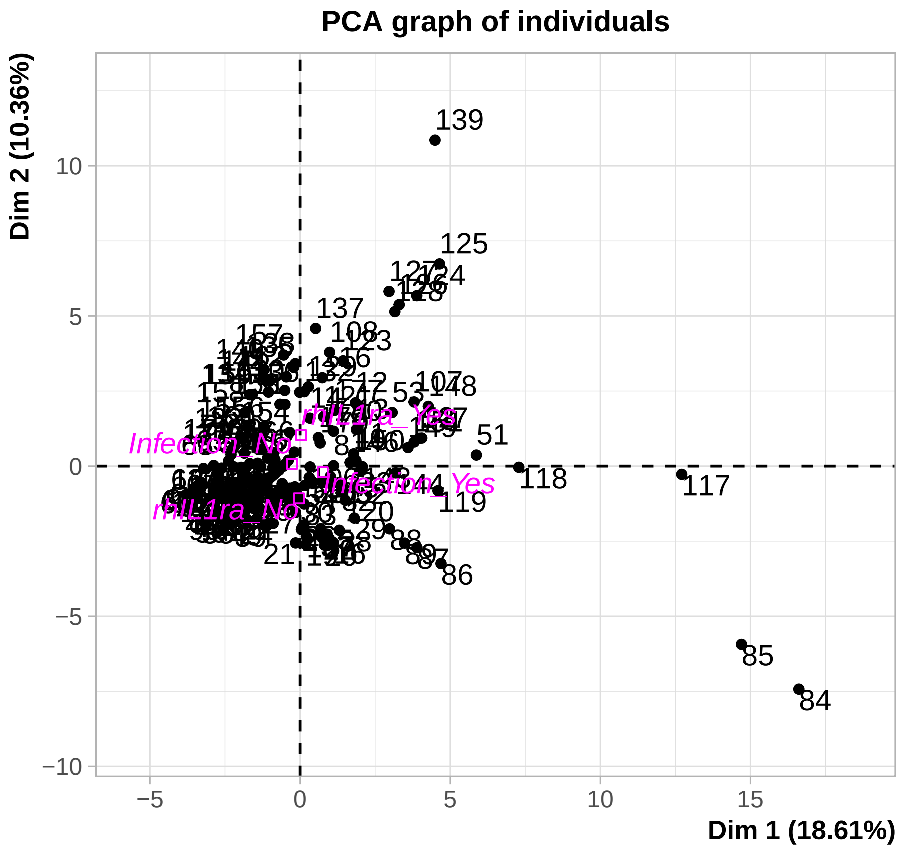


```
## Warning: ggrepel: 21 unlabeled data points (too many overlaps). Consider
## increasing max.overlaps
```

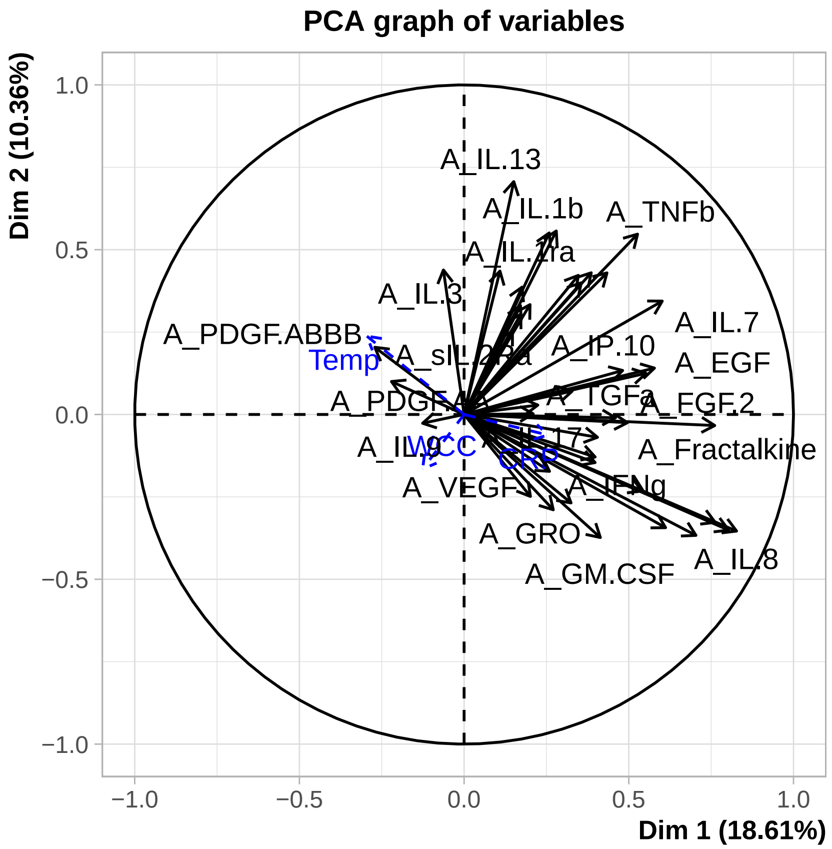


```
fviz_pca_var(pca.infl, col.var = "contrib") +
  scale_color_gradient2(low = "white", mid = "blue",
                        high = "red", midpoint = 6) +
  theme_minimal()
```

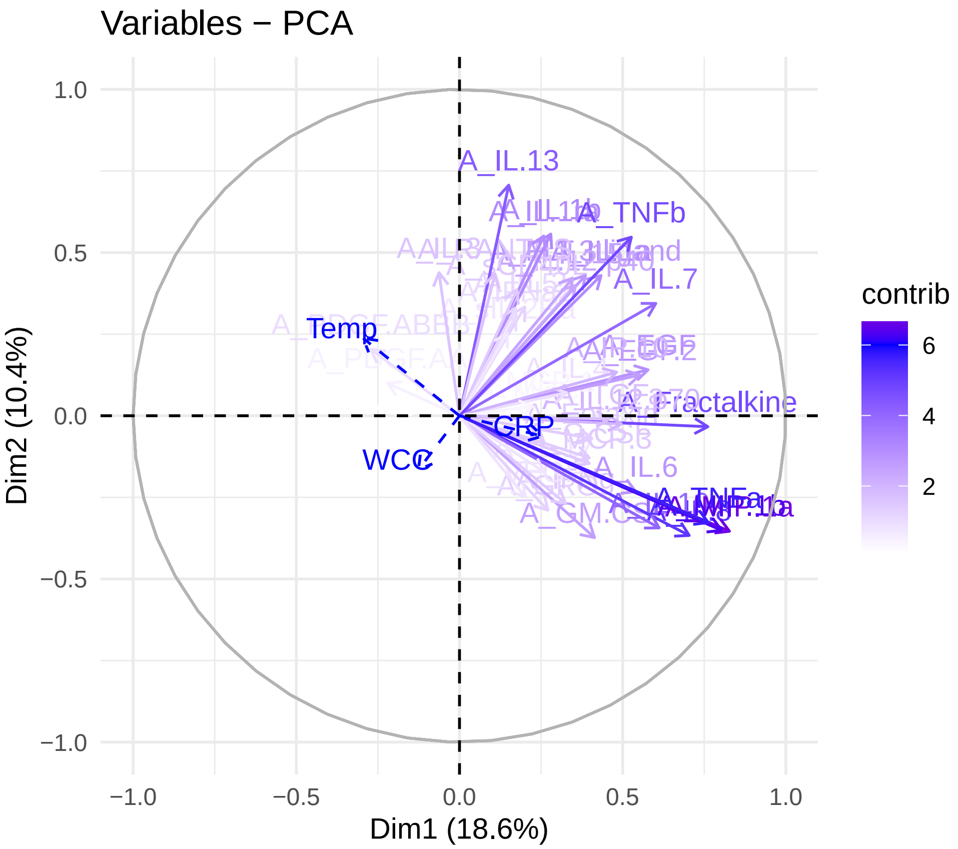


```r
p1 <- fviz_pca_biplot(pca.infl, col.var = "contrib", label = "none",
                      col.ind = "white", fill.ind = as.factor(small_data$Infection),
                      addEllipses = TRUE, geom.ind = "point") +
  scale_color_gradient2(low = "white", mid = "blue",
                        high = "red", midpoint = 6, guide = FALSE) +
  labs(fill = "Infection") +
  theme_minimal() +
  theme(plot.title = element_blank())

ggsave("collinear201228.pdf",
       plot = p1,
       device = "pdf",
       width = 5,
       height = 3,
       units = "in")
```

```
## Warning: It is deprecated to specify `guide = FALSE` to remove a guide. Please
## use `guide = "none"` instead.
```

```r
ggsave("collinear201228.tiff",
       plot = p1,
       device = "tiff",
       width = 5,
       height = 3,
       units = "in",
       dpi = "retina")
```

```
## Warning: It is deprecated to specify `guide = FALSE` to remove a guide. Please
```

```
## use `guide = "none"` instead.
```
