# Supplementary material for: Systemic inflammation alters the neuroinflammatory response: a prospective clinical trial in traumatic brain injury
Source: J Neuroinflammation. 2021 Sep 25;18:221. doi: 10.1186/s12974-021-02264-2 (PMC8464153; doi:10.1186/s12974-021-02264-2)
Supplement: Supplementary file 3 — Additional file 3. Title of data: Mixed models details. Description of data: Statistical details. [file 12974_2021_2264_MOESM3_ESM.docx]

**Additional file 3**

**An in-depth explanation of the choices made when using cross-correlations**

In order to investigate the direction of cytokine level changes between the different compartments, we performed cross-correlation analyses on all cytokines between their respective concentrations in brain-ECF and in arterial blood. We used the “ccf” package in R to find the cross-correlations and calculated the signed absolute maximum value for each cytokine and patient time series. An exclusion algorithm was employed in order to select robust cross-correlation series, as shown in **Box**.

**Box**: Exclusion algorithm with examples. A: Algorithm of exclusion. B: An example of an excluded cytokine and patient series. C: An example of an included cytokine and patient series.

**Box A**:

for every cytokine

for every patient

calculate CCs for lags in [-10, 10]

if series has only NAs

exclude series

else if there is no unique AM

exclude series

else if there are any NAs around the AM

exclude series

else if the CC plot is not strictly monotonous around the AM

exclude series

else

include series

CC = cross-correlation; NA = not available; AM = absolute maximum; around the AM = in the interval [AM - 2 lags, AM + 2 lags]

**Box B**: EGF for patient 2 was excluded because of the non-monotonous trend around the absolute maximum (red point). The points at lag 1 and lag -1 are both greater than the points at lag 2 and -2, respectively, violating the criteria of monotonicity on either side of the absolute maximum. This criterion decreases the risk of recording random variations as maxima.


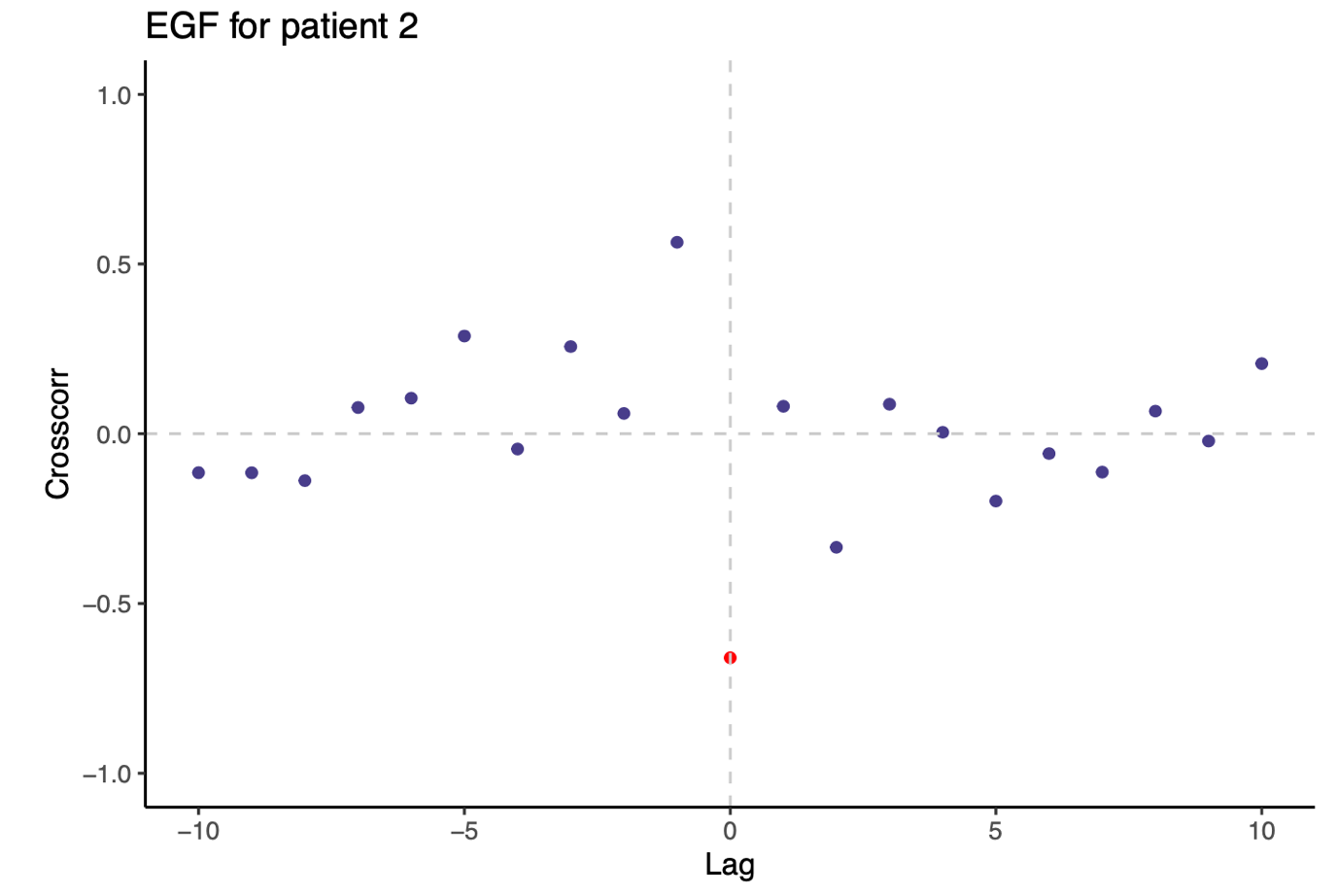


**Box C**: G-CSF for patient 13 is one of the most unambiguous cross-correlation series. Here, the maximum is unique, and cross-correlations are decreasing with time from the maximum. All series are available in the supplementary documents.


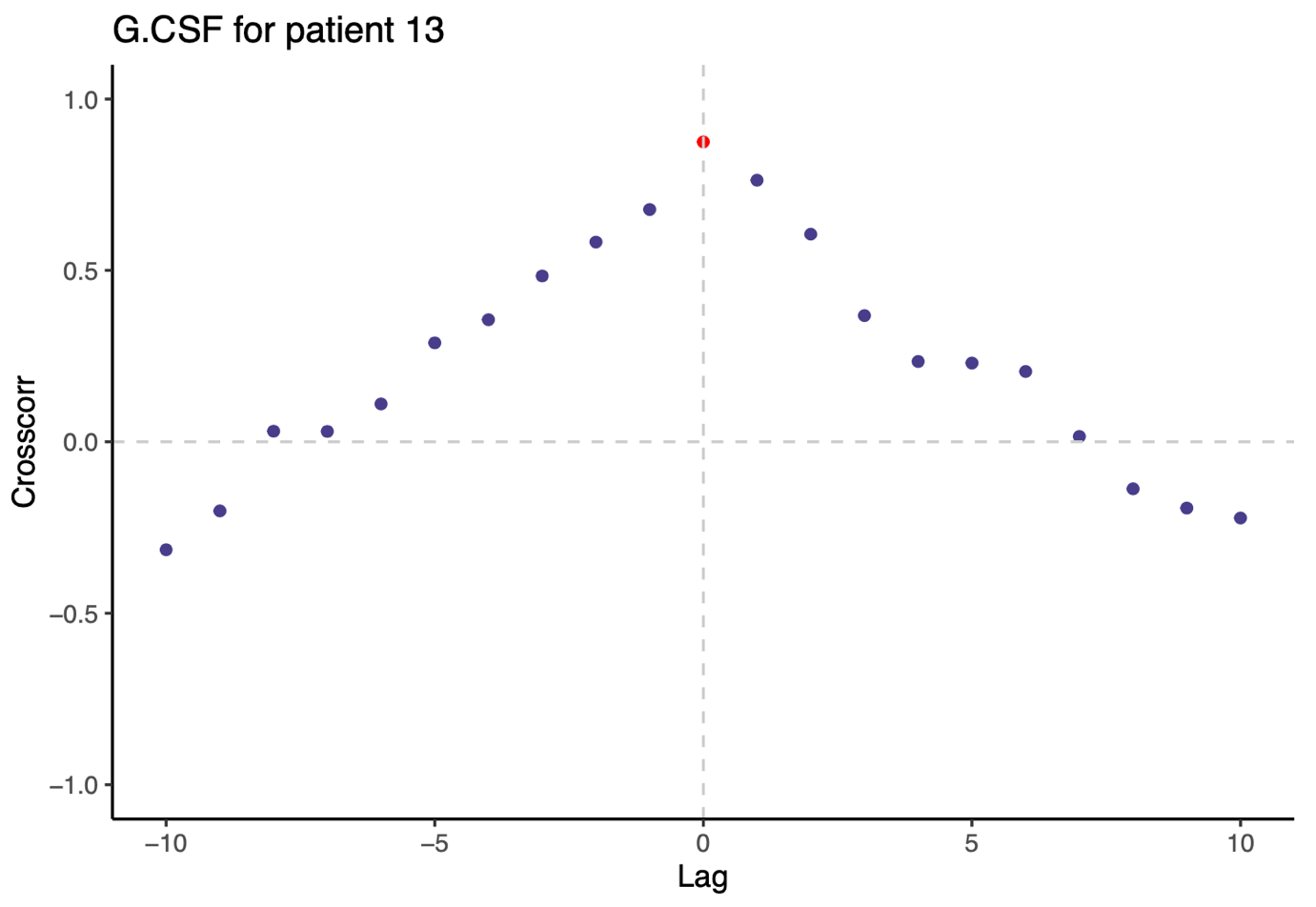


**Figure 1**: Proportion of patients included and excluded, per reason, for every cytokine.

**Figure 2**: Signed absolute maximum cross-correlations for each cytokine and patient series, grouped by (A) treatment arm, (B) occurrence of infection, (C) median CRP, (D) median WCC, (E) median temperature and (F) median ISS (injury severity score). In the figures reporting median scores, median values for each patient are compared to overall median values for the given parameter. Each lag is approximately six hours; the timings of the blood samples were matched to the appropriate time interval during which the brain-CSF samples were drawn. Lag < 0 indicates that an early activity in the brain correlates with a late activity in the blood; lag > 0 indicates the opposite. Correlation > 0 indicates that a positive concentration change in the brain correlates with a positive change in the blood; correlation < 0 indicates that a positive concentration change in the brain correlates with a negative change in the blood, and vice versa. Error bars show 95 % confidence intervals for the distribution of the respective groups.

**Figure 2, A**:

**Figure 2, B**:

**Figure 2, C**:

**Figure 2, D**:

**Figure 2, E**:

**Figure 2, F**:

Figure 2 depicts a fairly even distribution of all signed absolute maxima of cytokine and patient series in the four quadrants. The points are distributed such that there is a global symmetry around lag zero for both positively and negatively cross-correlated cytokine and patient series. This indicates that there is no observed global trend in neither directionality in movement between the blood and brain-CSF compartments nor in relative concentration changes between the two compartments. Furthermore, when grouping by treatment (Figure 2A), by inflammatory indicators (Figures 2B-2E) or by level of multi-trauma measured by the injury severity score (ISS) (Figure 2F), there is no significant difference between the groups globally. However, on average for all cytokine and patient series, activity in the brain seems to precede activity in the blood, with a small positive correlation. Analogously insignificantly, we can observe the following weak trends of averages: (1) IL1ra-treated patients have an insignificant inclination to a brain-before-blood pattern, (2) patients with infection seem to have an insignificant inclination to a brain-before-blood pattern, (3) there are no differences in lag for patients with high values of CRP or WCC, (4) patients with *lower* body temperature have an insignificant inclination to a brain-before-blood pattern, and (5) patients with higher ISS have an insignificant inclination to a brain-before-blood pattern. Speculatively, this could indicate that patients who have extracerebral inflammation – for example infection or extracranial trauma – have controlled levels of cytokines in the brain, which subsequently influence cytokine levels in the blood. Yet, since the confidence intervals are extensively overlapping, the data shows no clear general relationship between the inflammatory parameters and the temporal relationships of systemic and cerebral cytokines.

Although there might not be an average global trend for all 42 cytokines in the 20 patients included in this study, individual cytokines or patients could have distinct patterns. In an attempt to address this hypothesis, we visually examined plots for every cytokine, colored as in Figure 2, as well as plots for every patient, annotated with the same parameters **[Supplementary Materials: Supplementary Materials 1 – Code: Figure 2 code]**. Generally, we find a similar picture when looking at individual cytokines as for the overall figures, where there is a small difference between two groups that is insignificant, with confidence intervals vastly overlapping. Significant findings for cytokines are summarized in **Table**. When looking at individual patients, there are some patients leaning toward a brain-to-blood pattern and others oppositely, but there seems to be no pattern in regard to the inflammatory parameters studied.

| Cytokine | Parameter | Finding | # of data points in plot |
| --- | --- | --- | --- |
| GM-CSF | WCC | Brain-after-blood pattern for low WCC levels. | 10 |
| IL-1ra | Infection | Negative cross-correlations for patients with infection. (Both groups have a brain-after-blood pattern.) | 10 |
| IL-7 | Treatment | Positive cross-correlations for patients with IL-1ra treatment. (Both groups have mixed signs of lags.) | 4 |
| IL-7 | ISS | Brain-before-blood pattern for low ISS. | 4 |
| IL-12-p70 | Infection | Brain-before-blood pattern for patients with infection. | 6 |
| IL-12-p70 | WCC | Brain-before-blood pattern for patients with high WCC levels. | 6 |

**An in-depth explanation of the choices made when applying the linear-mixed models**

The natural logarithms were taken of the cytokine levels in brain-ECF and blood.

Variance inflation factor (VIF) values were below 2 for all independent variables and cytokines, apart for IL-4, for which VIF values were below 4.5. An interaction term was hypothesized between WCC and temperature, but since the Akaike information criterion (AIC) was lower without the interaction term and merely 2 out of 42 cytokines were significant (*p* < 0.05) in an analysis of variance (ANOVA), we decided to keep the model without interaction terms.

A first-order autoregressive model was employed, since it was best in terms of AIC when comparing several autoregressive moving average (ARMA) models; autoregressive integrated moving average (ARIMA) were not used as the Kwiatkowski–Phillips–Schmidt–Shin (KPSS) test was insignificant at *p* < 0.05. Visual inspection of the first-order autocorrelation function and the partial autocorrelation function were nonsignificant and the residuals were small for this model. Furthermore, for the linear mixed-effect model, visual inspection of residual and Q-Q plots did not reveal any obvious deviations from homoscedasticity or normality **[Supplementary Materials: Figure 3-4 code]**. In order to compare the parameters of fixed effects to each other and across different cytokines, we normalized the regression coefficients by multiplication of the fraction of standard deviations of the independent variable and the dependent variable; these may be regarded as correlations.

In short, the model that has the best information criteria and may show how inflammation parameters are associated with brain cytokine levels, is the following:

$$M_{ij}= \beta_{0j}+\beta_{1j}t_{ij}{+ \beta}_{2}A_{ij}+\beta_{3}C_{ij}+\beta_{4}L_{ij}+\beta_{5}T_{ij}+\beta_{6}I_{ij}+R_{ij}$$

where

$$\beta_{0j}= \gamma_{00}+\gamma_{01}X_{j}+S_{0j}$$

and

$$\beta_{1j}= \gamma_{10}+S_{1j}$$

*M* is the brain cytokine level for a particular cytokine, *t* is the time, *A* is the arterial cytokine level, *C* is CRP, *L* is white cell count, *T* is temperature, *I* is presence of infection, *X* is treatment randomization, *R* is a random error, with a first-order auto-regressive structure, *S* is the individual error term, and *i* is the measurement occasion for individual *j*. In summary, this is a mixed-effect model, with individual intercepts and time gradients for each patient.

In order to compare the inflammation parameters, all partial gradients *ß_k_* for each cytokine are weighted with the fraction of standard deviations of the parameter itself and the brain cytokine. That is,

$$weighted gradient=\beta_{k}\cdot\underset{\mathrm{weight}}{\underbrace{\frac{\sigma\left( P_{k} \right)}{\sigma\left( M \right)}}},$$

where *P_k_* is the inflammation parameter *k*. By rewriting the expression,

$$\beta_{k}\cdot\frac{\sigma\left( P_{k} \right)}{\sigma\left( M \right)}=\frac{\partial M}{\partial P_{k}}\cdot\frac{\sigma\left( P_{k} \right)}{\sigma\left( M \right)}=\frac{\partial\left( \frac{M}{\sigma\left( M \right)} \right)}{\partial\left( \frac{P_{k}}{\sigma\left( P_{k} \right)} \right)},$$

it is clear that the weighted gradient is a scaling of the data adjusted for its spread. This was then repeated and performed using different parameters in different analyses, changing between predicting arterial and brain-ECF cytokines.

The normalized fixed effect coefficients are plotted as a heatmap in, including a dendrogram using 1 minus Pearson correlation distance with average linkage.
