# Supplementary figures and images for: Systemic inflammation alters the neuroinflammatory response: a prospective clinical trial in traumatic brain injury
Source: J Neuroinflammation. 2021 Sep 25;18:221. doi: 10.1186/s12974-021-02264-2 (PMC8464153; doi:10.1186/s12974-021-02264-2)

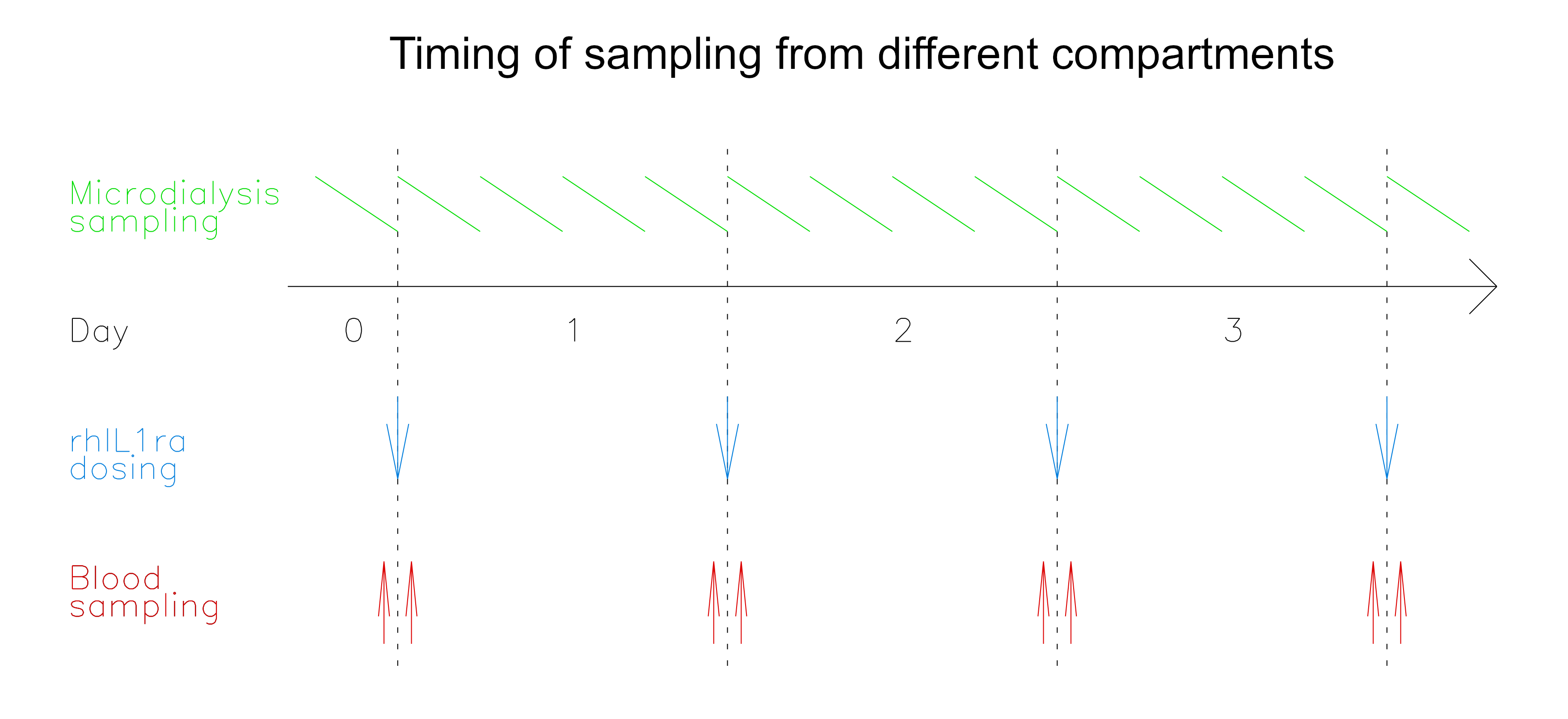

Supplement: Supplementary file 4 — Additional file 4. Title of data: Timing of blood samples and cytokine pooling. Description of data: Illustrating the timing between microdialysis samples, blood compartment samples and recombinant human Interleukin 1 receptor antagonist (rhIL1ra) treatment. Microdialysis sampling (green) were pooled during 6 h epochs throughout the study. The rhIL1ra treatment was administered once daily to patients in the treatment arm (blue). Blood samples were taken one hour before and one hour after administration of rhIL1ra (or equivalent timing, but no drug, in the control group) (red). [file 12974_2021_2264_MOESM4_ESM.tiff]

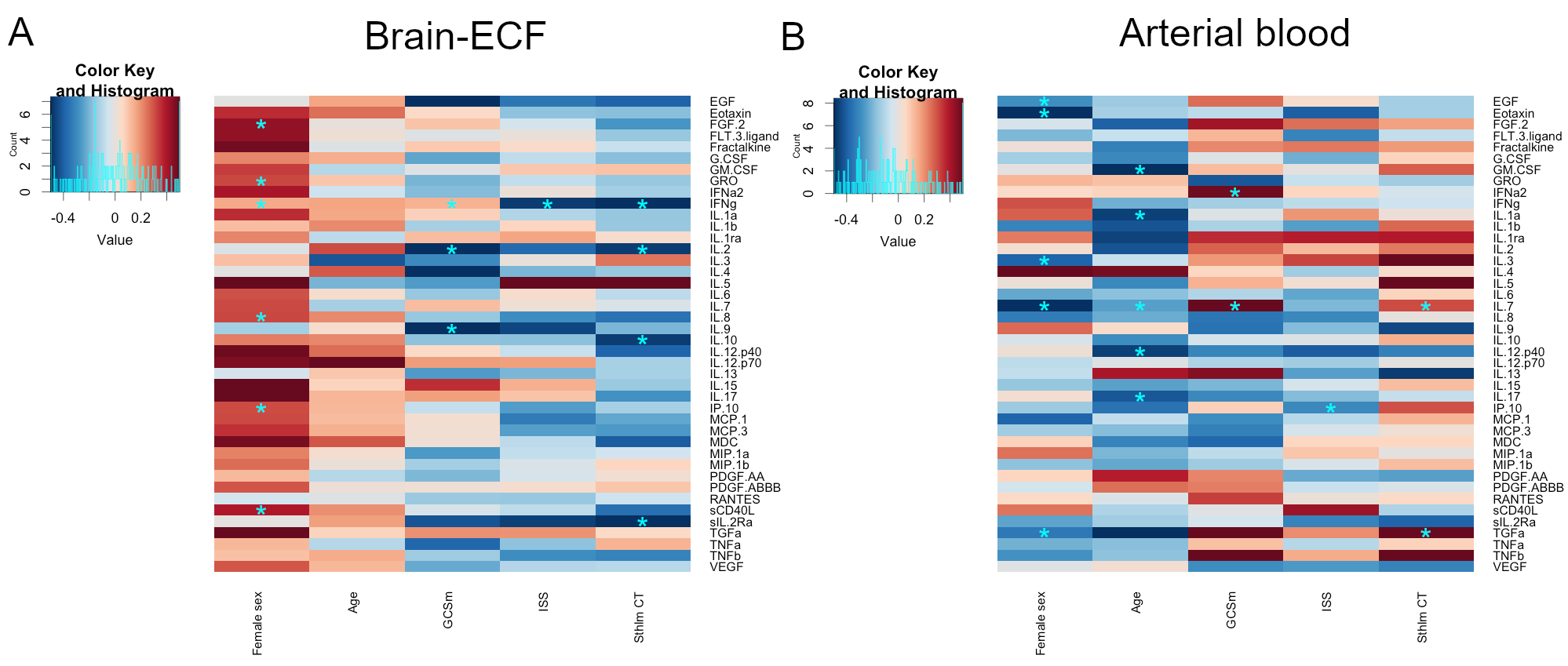

Supplement: Supplementary file 6 — Additional file 6. Description of data: Coefficients of linear mixed effect models, displayed as heatmaps. The colours of the heatmaps are graded such that red represents positive coefficients and blue represents negative coefficients. All coefficients are normalized using the quotient of their standard deviation and that of the dependent variable. Significant coefficients are highlighted with an asterisk. Independent variables are along the x-axis and the dependent variable for each model is the cytokine of the respective row on the y-axis in either (A) the brain extracellular fluid or (B) arterial blood. Differences in cytokines displayed between the two subfigures is due to insufficient data to generate all coefficients from the model. [file 12974_2021_2264_MOESM6_ESM.tiff]

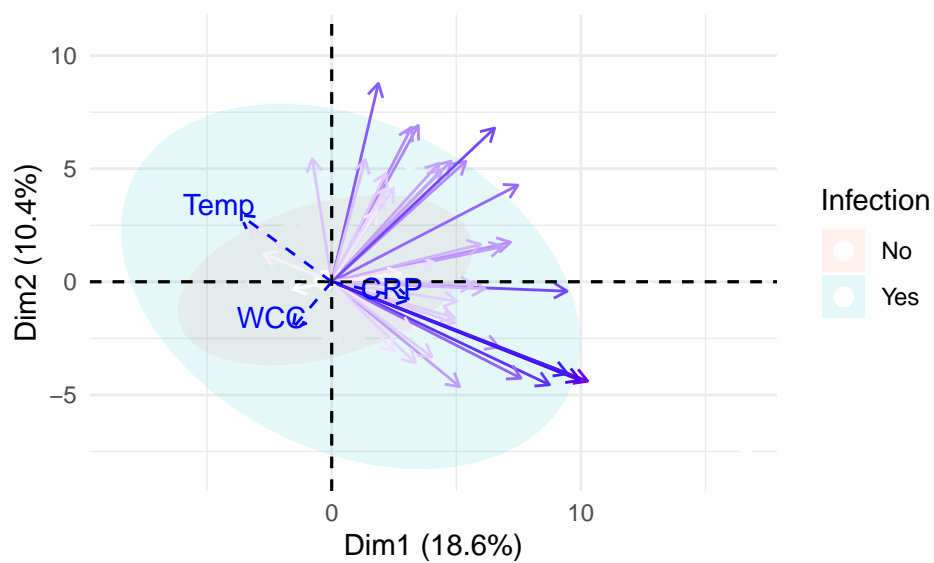

Supplement: Supplementary file 7 — Additional file 7. Title of data: Principal component analysis of all cytokines, with additional inflammatory variables. Description of data: Unlabelled arrows are loadings of cytokines. Labelled and dashed arrows are post-analysis projections of continuous inflammatory markers. Infection and non-infection shaded areas show confidence intervals of scores labelled as infection or non-infection. [file 12974_2021_2264_MOESM7_ESM.pdf]
