# Supplementary material for: Systemic inflammation alters the neuroinflammatory response: a prospective clinical trial in traumatic brain injury
Source: J Neuroinflammation. 2021 Sep 25;18:221. doi: 10.1186/s12974-021-02264-2 (PMC8464153; doi:10.1186/s12974-021-02264-2)
Supplement: Supplementary file 1 — Additional file 1. Title of data: Baseline data. Description of data: Columns for the first eight rows are for each patient (C, control; I, intervention); moreover, the first four rows have time along the horizontal axis. Columns for the final row are for each cytokine. Rows 1-3 display changes over time of white cell count (WCC), C-reactive protein (CRP) and temperature, with corresponding lines of best fit. Row 4 displays infection status of patients over time. Row 5 shows infection (VAP, ventilator-associated pneumonia; IAS intra-abdominal sepsis; CI, chest infection), severe adverse events (SAE), sex (M, male; F, female) and years of age for each patient. Row 6 shows Stockholm computed tomography (CT) score for each patient, coloured after whether a deterioration was observed during hospital stay. Row 7 shows injury severity score (ISS) for each patient. Row 8 shows fraction of missingness in each compartment and for each patient. Row 9 shows fraction of missing cytokines in each compartment. The chest infection was a result of mucous plugging resulting in a right lower lobe collapse, hence not fulfilling VAP criteria. [file 12974_2021_2264_MOESM1_ESM.pdf]

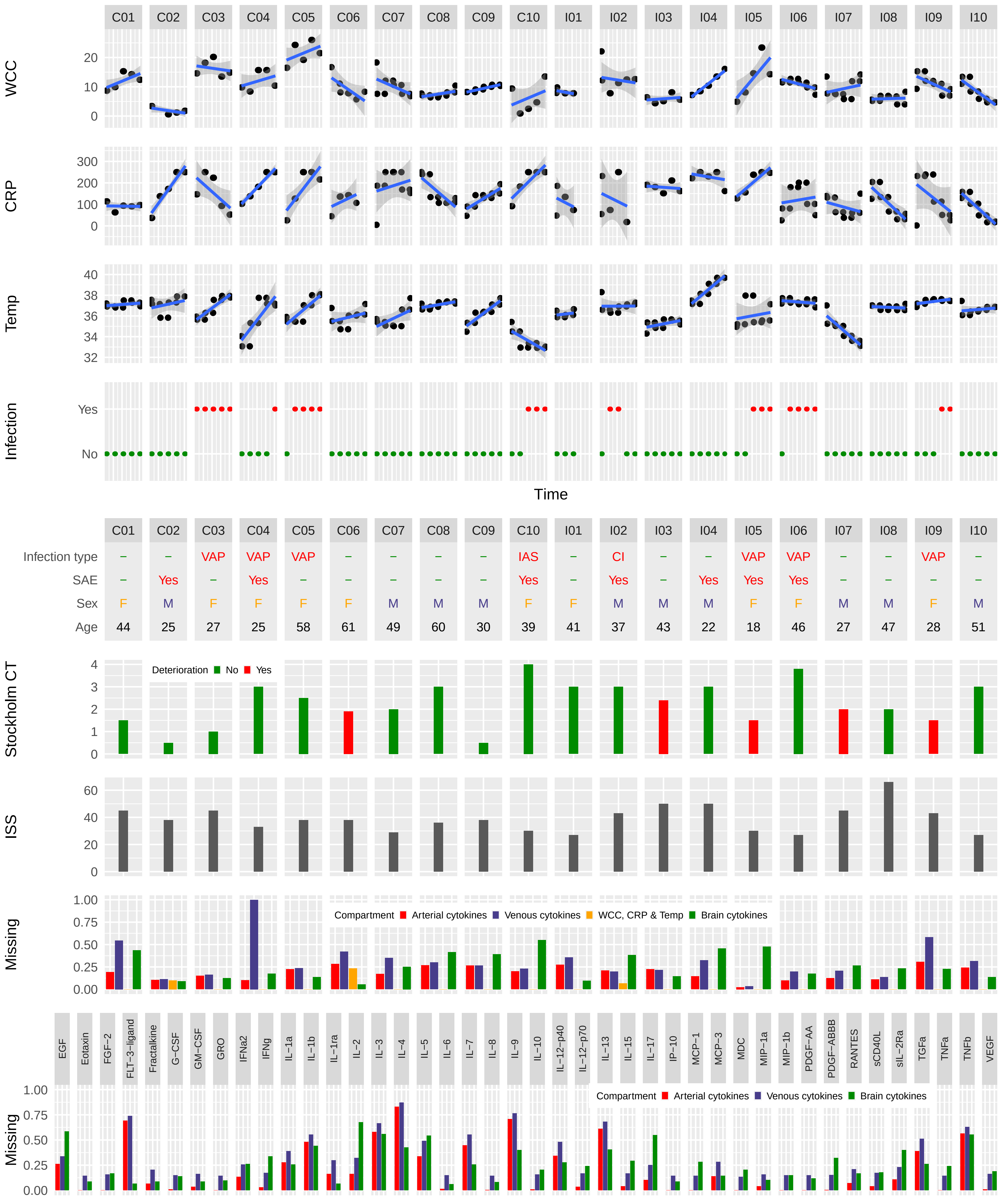

C01 C02 C03 C04 C05 C06 C07 C08 C09 C10 I01 I02 I03 I04 I05 I06 I07 I08 I09 I10
WCC
CRP
Temp
Infection
Yes
No
Time
Infection type: – – VAP VAP VAP – – – – IAS – CI – – VAP VAP – – VAP –
SAE: – Yes – Yes – – – – – Yes – Yes – Yes Yes Yes – – – –
Sex: F M F F F F M M M F F M M M F F M M F M
Age: 44 25 27 25 58 61 49 60 30 39 41 37 43 22 18 46 27 47 28 51
Stockholm CT
Deterioration No Yes
ISS
Missing
Compartment Arterial cytokines Venous cytokines WCC, CRP & Temp Brain cytokines
EGF Eotaxin FGF−2 FLT−3−ligand Fractalkine G−CSF GM−CSF GRO IFNa2 IFNg IL−1a IL−1b IL−1ra IL−2 IL−3 IL−4 IL−5 IL−6 IL−7 IL−8 IL−9 IL−10 IL−12−p40 IL−12−p70 IL−13 IL−15 IL−17 IP−10 MCP−1 MCP−3 MDC MIP−1a MIP−1b PDGF−AA PDGF−ABBB RANTES sCD40L sIL−2Ra TGFa TNFa TNFb VEGF
Missing
Compartment Arterial cytokines Venous cytokines Brain cytokines
